# DNA variation and brain region-specific expression profiles exhibit different relationships between inbred mouse strains: implications for eQTL mapping studies

**DOI:** 10.1186/gb-2007-8-2-r25

**Published:** 2007-02-26

**Authors:** Iiris Hovatta, Matthew A Zapala, Ron S Broide, Eric E Schadt, Ondrej Libiger, Nicholas J Schork, David J Lockhart, Carrolee Barlow

**Affiliations:** 1 The Salk Institute for Biological Studies, Laboratory of Genetics, 10010 North Torrey Pines Road, La Jolla, CA 92037, USA; 2 National Public Health Institute, Department of Molecular Medicine, Haartmaninkatu 8, 00290 Helsinki, Finland; 3 INSERM U513, Neurobiology and Psychiatry, Faculté de Médecine, 8 rue du Général Sarrail, Créteil 94010 cedex, France; 4 Biomedical Sciences Graduate Program, School of Medicine, University of California, San Diego, 9500 Gilman Drive, La Jolla, CA 92093, USA; 5 Polymorphism Research Laboratory, Department of Psychiatry, University of California, San Diego, 9500 Gilman Drive, La Jolla, CA 92093, USA; 6 Neurome Inc., 11149 North Torrey Pines Road, La Jolla, CA 92037, USA; 7 Rosetta Inpharmatics Inc., 401 Terry Avenue North, Seattle, WA 98109, USA; 8 Amicus Therapeutics, 6 Ceder Brook Drive, Cranbury, NJ 08512, USA; 9 BrainCells Inc., 10835 Road to the Cure, San Diego, CA 92121, USA

## Abstract

**Background:**

Expression quantitative trait locus (eQTL) mapping is used to find loci that are responsible for the transcriptional activity of a particular gene. In recent eQTL studies, expression profiles were derived from either homogenized whole brain or collections of large brain regions. However, the brain is a very heterogeneous organ, and expression profiles of different brain regions vary significantly. Because of the importance and potential power of eQTL studies in identifying regulatory networks, we analyzed gene expression patterns in different brain regions from multiple inbred mouse strains and investigated the implications for the design and analysis of eQTL studies.

**Results:**

Gene expression profiles of five brain regions in six inbred mouse strains were studied. Few genes exhibited a significant strain-specific expression pattern, whereas a large number of genes exhibited brain region-specific patterns. We constructed phylogenetic trees based on the expression relationships between the strains and compared them with a DNA-level relationship tree. The trees based on the expression of strain-specific genes were constant across brain regions and mirrored DNA-level variation. However, the trees based on region-specific genes exhibited a different set of strain relationships, depending on the brain region. An eQTL analysis showed enrichment of *cis*-acting regulators among strain-specific genes, whereas brain region-specific genes appear to be mainly regulated by *trans*-acting elements.

**Conclusion:**

Our results suggest that many regulatory networks are highly brain region specific and indicate the importance of conducting eQTL mapping studies using data from brain regions or tissues that are physiologically and phenotypically relevant to the trait of interest.

## Abstract 2

Gene expression profiles of five brain regions from six inbred mouse strains suggest that many regulatory networks are highly specific to particular brain regions.

## Background

Recent genome sequencing efforts have catalogued DNA-level variation between different species, strains, and individuals. In addition, gene expression profiling data indicate that there is considerable variation in expression patterns between strains of inbred mice and individual humans, and several recent articles have studied some of the underlying regulatory mechanisms responsible for this variation [1-5]. The expression studies are based on mapping of so-called 'expression quantitative trait loci' (eQTL), in which gene expression profiles are treated as quantitative traits, and genome-wide association and linkage mapping are performed to localize regulatory elements that affect the expression of the corresponding differentially expressed genes. The underlying logic is that if a regulatory element coincides with the known location of the differentially expressed gene, then it most likely represents a *cis*-acting regulatory element, whereas a regulatory element identified at a different location most likely represents a *trans*-acting regulatory element. However, the relationship between DNA sequence differences and gene expression levels on a genomic scale, and how these two types of variation influence the activities of genes across different tissues has not been studied in detail.

We believe that inbred mouse strains offer an excellent model to study the relationship between DNA-level variation and variation in gene expression patterns, because the genealogy and DNA-level variation across different strains are well known. We investigated whether inbred strains that are closely related have gene expression profiles that on average resemble each other more than strains that are distantly related. In addition, we were interested in localizing regulatory elements of genes with either strain- or brain region-specific expression patterns by eQTL analyses.

## Results

**Figure 1:**
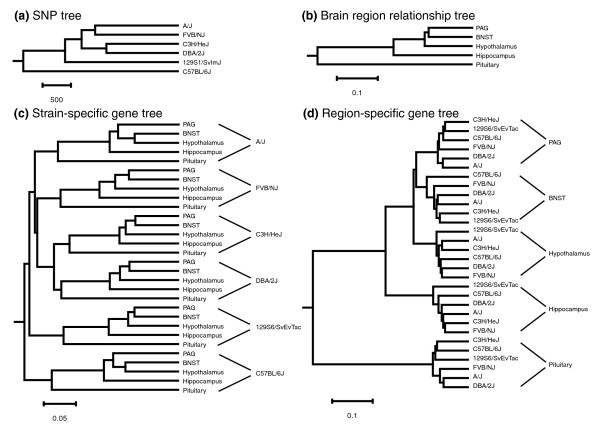
Relationships of inbred mouse strains. **(a) **A phylogenetic tree based on the fraction of allelic differences across 12,473 loci between inbred mouse strains. **(b) **A phylogenetic tree based on the gene expression differences between brain regions averaged over six inbred mouse strains used in this study. **(c) **A phylogenetic tree based on the gene expression relationship of 2,235 strain-specific genes. **(d) **A phylogenetic tree based on the gene expression relationship of 19,813 brain region-specific genes. Scale bars show the number of allelic differences (panel a) or the distance based on gene expression (panels b, c, and d). BNST, bed nucleus of the stria terminalis; PAG, periaqueductal gray; SNP, single nucleotide polymorphism.

We considered how global DNA-level variation correlates with gene expression pattern variation across five brain regions in six inbred mouse strains. The genealogy of these strains is well known [6], and single nucleotide polymorphism (SNP) data are publicly available [7,8]. We constructed a DNA-level phylogenetic tree based on genetic similarity across 12,473 SNPs [8] (Figure 1a). The derived relationships correlate well with the known genealogies of the strains and previously published DNA variation-based relationships [9,10].

### Indentification of genes with strain-specific or brain region-specific expression

We carefully dissected five different brain regions (bed nucleus of the stria terminalis [bnst], hippocampus, hypothalamus, periaqueductal gray [pag], and pituitary gland) from six commonly used inbred mouse strains (129S6/SvEvTac, A/J, C3H/HeJ, C57BL/6J, DBA/2J, and FVB/NJ). Replicate gene expression patterns were measured using the Affymetrix mouse genome 430 2.0 arrays, which contain 45,037 probe sets and cover a significant portion of the mouse transcriptome. Next, we performed a multiple regression formulation of an analysis of variance (ANOVA) using the different mouse strains and brain regions, as well as their interactions, as the independent variables, and using gene expression signal as the dependent variable to identify genes that exhibited either strain-specific or region-specific effects. We chose to use a regression model because of the fact that we had an imbalance (61 observations) in our design.

A total of 2,235 probe sets (5.0%) exhibited a significant strain-specific effect (*P *< 0.01; the strain effect was more significant than the brain region effect; false discovery rate q value < 0.004). The q values were obtained using the 'smoother method' of Storey and Tibshirani [11]. However, even using the more conservative Benjamini and Hochberg [12] method produces q values of 0.02 for *P *values < 0.01. Somewhat surprisingly, 19,813 probe sets (44.0%) exhibited a brain region-specific expression pattern (*P *< 0.01; the region-specific effect was more significant than the strain effect; q value < 0.001).

In addition to the regression formulation that accounted for an unbalanced sample design, a simple two-way ANOVA, in which the outlying unbalanced sample (least correlated) was removed, was conducted in order to determine the number of probe sets that exhibited a significant interaction between strain and brain region. This analysis yielded virtually identical results to those of the regression formulation in terms of F statistics (the F statistics and *P *values of the regression formulation and two-way ANOVA are available for all probe sets in Additional data file 1). The number of probe sets that exhibited a significant brain region and strain interaction (*P *< 0.01; q value = 0.01) in the two-way ANOVA model was 7,415. These data indicate that although there are significant differences in gene expression between different inbred strains, a large proportion of genes exhibit region-specific expression patterns and interactions between strain and brain region, suggesting that multiple region-specific regulatory mechanisms control gene expression. 

### Correlation of DNA sequence variation and gene expression level variation

In order to determine the extent to which DNA sequence variation correlates with gene expression level variation in different brain regions, we constructed phylogenetic trees of strain relatedness using either strain-specific or region-specific genes identified by the regression model (Figure 1). We averaged the (scaled) gene expression signals for the replicate samples for each gene and calculated a Pearson correlation coefficient for the signal intensities between all possible strain combinations for each brain region. We then transformed these correlation coefficients into distances to construct phylogenetic trees (Figure 1). The tree based on the expression levels of the strain-specific genes (Figure 1c) has branches that exhibit strain relationships that parallel those based on the SNPs (Figure 1a). Within each strain, brain-region relationships follow the molecular architecture of the brain [13] shown in Figure 1b. Likewise, the tree based on region-specific genes (Figure 1d) has branches that show individual brain region clustering according to the molecular architecture. However, the strain relatedness within each brain region branch varies and exhibits a different set of strain relationships depending on the brain region. Because both the strain-specific and region-specific genes cluster in brain regions according to the known molecular architecture of the brain [13], it is not likely that the observed clustering patterns are due to random noise.

To test whether these correlations between the gene expression-based trees and the SNP tree are significant, we broke down the expression trees by brain region and used Mantel's matrix correspondence test. We compared the strain-specific gene expression trees and the region-specific gene expression trees with the SNP tree for each brain region separately. By using the strain-specific genes, there was a significant correlation between the SNP tree and each of the strain-specific expression trees (bnst: R = 0.727, *P *= 0.008; hippocampus: R = 0.680, *P *= 0.002; hypothalamus: R = 0.529, *P *= 0.008; pag: R = 0.715, *P *= 0.004; pituitary: R = 0.512, *P *= 0.023). By contrast, there was no statistically significant correlation between the SNP tree and any of the region-specific expression trees (bnst: R = 0.466, *P *= 0.180; hippocampus: R = 0.476, *P *= 0.195; hypothalamus: R = 0.370, *P *= 0.169; pag: R = -0.072, *P *= 0.524; pituitary: R = 0.271, *P *= 0.135). The strain-specific gene trees were more similar to the SNP tree than the region-specific gene trees (paired *t*-test *P *= 0.006). When the strain-specific expression trees where compared with each other, all pair-wise comparisons (*n *= 10) were statistically significant (R > 0.48, *P *< 0.024). When the region-specific expression trees where compared with each other, only two comparisons out of ten were statistically significant (bnst versus pituitary: R = 0.406, *P *= 0.04; and hippocampus versus hypothalamus: R = 0.620, *P *= 0.025), which is consistent with our proposition that the strain-specific expression trees resemble the SNP tree and each other, and that the region-specific expression trees do not correlate with each other, DNA-level variation, or known genealogy. In other words, the known genetic differences (SNPs between strains) have a low and insignificant correlation to brain region-specific differences, whereas the strain-specific differences exhibit a high and significant correlation to genetic differences.

These data suggest that because the relatedness of the strains based on strain-specific genes correlate with the DNA-level variation and known genealogy, the expression of strain-specific genes (that comprise only about 5% of all genes on the array) is mostly regulated by *cis*-acting regulatory elements. DNA variations in a *cis*-regulatory element are likely to affect mainly the transcription of a single gene close to that regulatory element, and more dramatic gene expression differences between strains are associated with *cis*-acting eQTLs (Schadt, unpublished data). Therefore, a phylogenetic tree based on SNPs and a tree based on genes with *cis*-acting regulators should be similar.

### Global eQTL analysis shows an enrichment of *cis*-acting eQTLS among strain-specific genes

To assess this hypothesis we conducted an eQTL analysis on gene expression data from the six inbred strains. Indeed, 48% of the strain-specific probe sets with SNP markers within 4 megabases (Mb) had significant *cis*-acting eQTLs (*P *≤ 0.001; 1,015 out of 2,115 probe sets [a subset of the original 2,235 strain-specific probe sets that had SNP markers located within 4 Mb]), whereas only 10% of the region-specific probe sets exhibited significant *cis*-acting eQTLs (1,940 of 18,868 region-specific genes with markers within 4 Mb).

Strain-specific SNPs within a probe sequence could cause differential hybridization and affect expression results, leading to spurious associations and an artificial enrichment of strain-specific *cis*-acting eQTLs. In order to control for strain-specific SNPs that could affect hybridization, we used an algorithm developed in our laboratory that takes advantage of the fact that Affymetrix GeneChips use a series of oligonucleotides that span up to hundreds of bases of a given gene to detect potential sequence variations between the strains (Greenhall and coworkers, unpublished data; see Materials and methods, below). These oligonucleotides (called probes) yield distinct patterns of intensity for each gene. The probe pairs are sensitive enough that appropriately positioned single base differences between the probe pair and the detected RNA can significantly change the signal intensity, and thus produce different patterns between slightly different sequences [14].

We compared the underlying patterns of signal intensity between the strains to identify probe sets that may harbor sequence differences. Using a Bonferroni corrected *P *< 0.01 (calculated from a two-tailed Student's *t*-test [unpaired, equal variance]), 144 out of the 1015 strain-specific probe sets with significant *cis*-acting eQTLs were predicted to harbor sequence differences within the probe set that may affect hybridization. Of the 1940 region-specific probe sets with significant *cis*-acting eQTLs, 167 were predicted to harbor sequence differences. When we ignore all probe sets that are predicted to harbor strain-specific sequence differences that could adversely influence hybridization, 56% of the strain-specific probe sets with SNP markers within 4 Mb had significant *cis*-acting eQTLs (*P *≤ 0.001; 901 out of 1611 probe sets), whereas only 10% of region-specific probe sets exhibited significant *cis*-acting eQTLs (1,773 of 17,422 probe sets). Using a less conservative *P *value threshold for the polymorphism detection algorithm did not change the relative enrichment of *cis*-acting eQTLs among strain-specific genes (see Additional data file 2). 

A caveat of the eQTL analysis is that the limited number of strains leads to a high rate of type I errors. However, the likelihood that significant false-positive eQTLs will be located within 4 Mb of the gene of interest, rather than anywhere in the genome, is greatly reduced. Moreover, our eQTL analysis should not be thought of as a traditional eQTL mapping study because it was not focused on the effect of an individual gene or marker, but rather on overall genomic trends or the trends of large groups of genes. For a detailed discussion concerning the determination of the false positive rate, see Materials and methods (below). Our regression model analysis showed that a large proportion of genes that are expressed in the brain are brain region-specific, and the derived relationships of the strains differed depending on the brain region, suggesting mainly *trans*-acting regulators for these genes, at least in these brain regions. Although the eQTL analysis showed a larger number of potentially *trans*-acting eQTLs among the brain region-specific genes (3023, as compared with 1358 *trans*-acting eQTLs among the strain-specific genes), it is difficult to demonstrate this trend definitively with the small number of strains analyzed.

### Certain genes have complicated expression patterns in the brain

**Figure 2:**
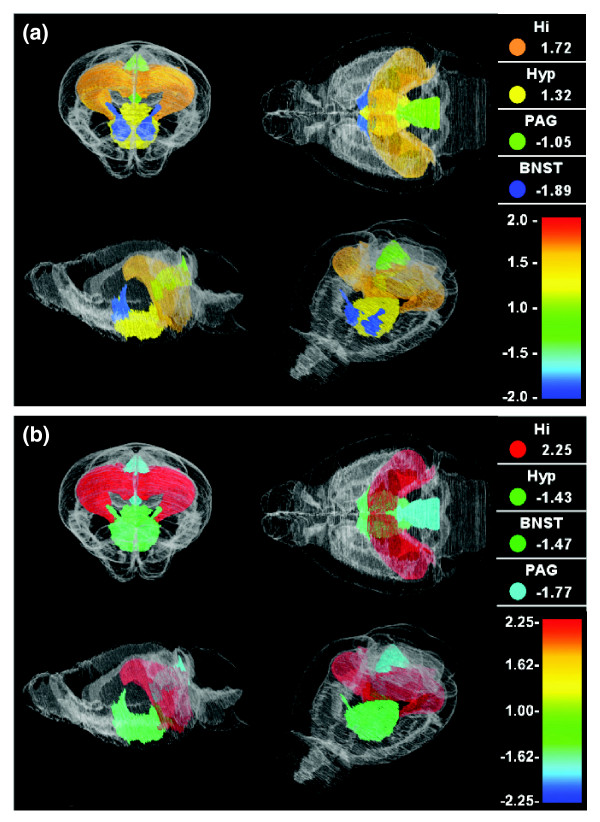
Brain gene expression levels of *Penk *(encoding preproenkephalin) and *Foxp1 *(encoding forkhead box P1). The signal intensities of two genes, *Penk *and *Foxp1*, were imported into the NeuroZoom software tool to visualize the three-dimensional gene expression patterns of these genes in the context of brain anatomy. A ratio of the signal intensities of **(a) ***Penk *and **(b) ***Foxp1 *between 129S6/SvEvTac (129) and A/J (A) strains is shown in hippocampus (Hi), hypothalamus (Hyp), periaqueductal gray (PAG), and bed nucleus of the stria terminalis (BNST). The expression fold change values are shown in the upper right corner of each panel for each brain region separately, together with color coding that matches the color of each brain region in the three-dimensional mouse brain atlas, shown from four different angles. Note that the gene expression level of *Penk *in Hi and Hyp is higher in the 129 strain than in the A strain, but in Pag and Bnst it is higher in the A strain than in the 129 strain. Similarly, the expression level of *Foxp1 *in Hi is higher in the 129 strain than in the A strain, whereas in Hyp, Bnst, and Pag the expression level is higher in the A strain than in the 129 strain.

Our findings show that there is a large number of brain region-specific genes, suggesting that many regulatory networks are highly brain region specific. Certain genes have extremely complicated expression patterns whose variation is dependent on both strain and brain region effects. For example, the relative expression levels for two genes that exhibit significant strain and brain region variation, namely *Penk *(which encodes preproenkephalin) and *Foxp1 *(which encodes forkhead box P1), are shown in Figure 2 in a virtual three-dimensional brain atlas. Both genes exhibit interesting strain and region-specific expression patterns. In the hippocampus and hypothalamus, the expression level of *Penk *is higher in the 129S6/SvEvTac strain than in the A/J strain. However, in the bnst and in the pag, the expression level of *Penk *is higher in the A/J strain than in the 129S6/SvEvTac strain. Similarly, the expression level of *Foxp1 *is higher in the 129S6/SvEvTac hippocampus than in the A/J hippocampus, but in all other regions studied *Foxp1 *expression level is higher in A/J animals than in 129S6/SvEvTac animals.

## Discussion

We have shown that the extent of global DNA sequence variation does not directly determine the extent of gene expression variation between inbred mouse strains. Furthermore, the strains that are genetically and genealogically most closely related sometimes have significantly different expression patterns. Interestingly, we observed that the expression of the strain-specific genes appear to be driven mainly by *cis*-acting regulatory elements, whereas the brain region-specific genes are mainly regulated by *trans*-acting regulators. It has been shown that *trans*-acting regulators affect expression levels of multiple genes [15], and that both *cis*-acting and *trans*-acting loci regulate variation in the expression levels of genes, although most act in *trans *[1]. The heritability estimates for gene expression regulation are relatively low (median value 0.34) [3], at least based on expression data from cell lines. Therefore, it is likely that the expression of the majority of genes is influenced by environmental or nongenetic factors, including epigenetic mechanisms, such as DNA methylation and histone acetylation.

The large differences in gene expression patterns across the strains depending on brain region indicate that it is essential to conduct eQTL mapping using data from brain regions that are physiologically and phenotypically relevant to the disease or trait being investigated. Our results show that it is important to dissect a sufficiently small, reasonably homogeneous anatomic regions for gene expression profiling studies in order to avoid 'dilution' of strain-specific and region-specific effects. If several brain regions are combined, then the observed gene expression profiles will be a weighted average of the expression profiles of the individual regions. If a gene is expressed at measurable levels in multiple regions, then there will be a decrease in sensitivity to a change in any one region. If there are opposing gene expression patterns in multiple regions, then the measurement from a combined sample could miss important changes or even yield misleading information about underlying regulatory mechanisms.

## Conclusion

By investigating DNA polymorphisms and gene expression profiles of various brain regions in six inbred mouse strains, we noticed an enrichment of *cis*-acting regulators among the strain-specific genes, whereas the brain region-specific genes seem to be mainly regulated by *trans*-acting elements. In addition, our data suggest that different inbred mouse strains have very different relative amounts of certain transcripts in some brain regions, indicating that there are complex brain region-specific regulatory networks. Our findings shed light on regulatory mechanisms of gene expression in different tissues and strains on a genomic scale, and have important implications for the design and analysis of eQTL mapping studies. In order to identify meaningful regulatory networks, it is important to obtain gene expression profiles from sufficiently small, anatomically refined tissues.

## Materials and methods

### Animals

Seven-week-old male inbred mice were received from the Jackson Laboratory (Bar Harbor, ME, USA) (A/J, C3H/HeJ, C57BL/6J, DBA/2J, and FVB/NJ) or from Taconic Farms (Germantown, NY, USA) (129S6/SvEvTac). Animals were singly housed for 1 week before dissections were conducted. All animal procedures were performed according to protocols approved by the Salk Institute for Biological Studies Institutional Animal Care and Use Committee.

### Tissue collection and RNA preparation for gene expression analysis

All brain dissections were done between 11:00 and 17:00 hours on a petri dish filled with ice using a dissection microscope. The dissected brain regions for gene expression analysis included hypothalamus, hippocampus, pituitary gland, periaqueductal gray (pag), and bed nucleus of the stria terminalis (bnst). Hippocampus samples were directly frozen on dry ice and stored at -80°C. The smaller brain structures were collected in RNA Later buffer (Ambion, Austin, TX, USA) and samples from two to five animals were pooled and stored at -80°C. At least two independent replicate samples for each strain and brain region using independent animals were dissected. If samples were pooled, at least two independent pools were collected. The extraction of total RNA from the tissues was performed using the TRIzol reagent (Invitrogen, Carlsbad, CA, USA), in accordance with the manufacturer's instructions. 

### Microarray experiments

Gene expression analysis was done using mouse genome 430 2.0 arrays (Affymetrix, Santa Clara, CA, USA), which contain about 45,000 probe sets. Labeling of samples, hybridization, and scanning were performed as described elsewhere [13]. Two replicate samples from independent animals were prepared for each strain and each tissue (analysis of bnst for C3H/HeJ was performed in triplicate).

### Data analysis

Array results were analyzed using several different methods. First, .cel files were generated using Affymetrix software, imported into the TeraGenomics expression database, and then processed within the TeraGenomics analysis system (Information Management Consultants, Reston, VA, USA) [13]. More detailed information on the statistical methods and the TeraGenomics platform can be found in Additional data file 3 and at the TeraGenomics home page [16].

Phylogenetic trees were constructed using the UPGMA option of the MEGA3 software [17]. SNP trees were constructed based on the fraction of allele differences across all loci between strains. Several different metrics were tested using this strategy resulted in a tree that correlated best with the known genealogy of inbred strains. The SNP genotypes were from the same mouse strains as the expression data, except for the 129 strain. We used genotypes from 129S1/SvImJ and gene expression data from 129S6/SvEvTac substrain. We had genotypes available from four different 129 substrains and all of them clustered into a separate clade close to each other in a phylogenetic tree [8]. We selected the 129S1/SvImJ genotype because this strain is genealogically closest to 129S6/SvEvTac. Therefore, the analysis should not have suffered from using a slightly different, but closely related 129 strain for the two types of analyses.

Two-factor regression formulations of an ANOVA were performed using an in-house software program written in standard FORTRAN for Unix using the gene expression files of each array from the absolute analysis of the TeraGenomics analysis system. The results were refined and sorted in Excel. Only genes that scored as 'Present' in one of the files were included in the analysis. In order to test the statistical significance of strain, region, and locus effects on expression levels, we used two-factor linear regression models. Note that we had independent replicate observations on five mouse brain regions across six mouse strains for a total of 61 observations on the approximately 45,000 probe sets represented on the microarray (the bnst for C3H/HeJ was performed in triplicate). Let *y*_*i*,*j*,*k *_be the expression value of the *i*th replicate (*I *= 1, 2 ...) on the *j*th strain (*j *= 1 ... 6) for the *k*th brain region (*k *= 1 ... 5). A linear model for the expression values can be written as follows:

*y*_*i*,*j*,*k *_= *b*_0 _+ *b*_*s*(1)_*x*_*i*,*j*,*k*_(*s*1) + *b*_*s*(2)_*x*_*i*,*j*,*k*_(*s*2) + *b*_*s*(3)_*x*_*i*,*j*,*k*_(*s*3) + *b*_*s*(4)_*x*_*i*,*j*,*k*_(*s*4) + *b*_*s*(5)_*x*_*i*,*j*,*k*_(*s*5) + *b*_*r*(1)_*x*_*i*,*j*,*k*_(*r*1) + *b*_*r*(2)_*x*_*i*,*j*,*k*_(*r*2) + *b*_*r*(3)_*x*_*i*,*j*,*k*_(*r*3) + *b*_*r*(4)_*x*_*i*,*j*,*k*_(*r*4) + [∑s,rbs,r(δs,r)]
 MathType@MTEF@5@5@+=feaafiart1ev1aaatCvAUfeBSjuyZL2yd9gzLbvyNv2Caerbhv2BYDwAHbqedmvETj2BSbqee0evGueE0jxyaibaiKI8=vI8tuQ8FMI8Gi=hEeeu0xXdbba9frFj0=OqFfea0dXdd9vqai=hGuQ8kuc9pgc9s8qqaq=dirpe0xb9q8qiLsFr0=vr0=vr0dc8meaabaqaciGacaGaaeqabaqadeqadaaakeaadaWadaqaamaaqafabaGaamOyamaaBaaaleaacaWGZbGaaiilaiaadkhaaeqaaOGaaiikaGGaciab=r7aKnaaBaaaleaacaWGZbGaaiilaiaadkhaaeqaaOGaaiykaaWcbaGaam4CaiaacYcacaWGYbaabeqdcqGHris5aaGccaGLBbGaayzxaaaaaa@4381@ + *e*_*i*,*j*,*k*_

where *b*_0 _is an intercept term, *b*_*s*(h) _is the regression coefficient associated with the effect of the *h*th strain, *b*_*r*(g) _is the regression coefficient associated with the effect of the *g*th brain region, and *e*_*i*,*j*,*k *_is an error term. The x_*i*,*j*,*k*_(*sh*) and x_*i*,*j*,*k*_(*rg*) are indicator variables set to 1 if the *ijk*th observation is from strain *h *and/or region *g*, respectively, and 0 otherwise. Note that we test only five strain and four region terms because of redundancy in adding the sixth strain and fifth region in the model.

Tests of significance of the strain and region effects involve the hypothesis that the relevant regression coefficient departs from 0.0. Tests of more global hypotheses of any strain and/or region effects can be constructed by fitting reduced models that do not include the strain (or region) terms and comparing these reduced models with the 'full' model described above. These global tests involved five and four degrees of freedom for the strain and region effect tests, respectively. We assessed the significance of the difference between the reduced and full models using permutation tests assuming 99 data permutations (with lowest possible *P *= 0.01). Data were permuted across brain region and strain to determine accurate *P *values for the main effects of brain region and strain. To obtain accurate *P *values for the interaction terms, the residuals must be permuted, which was not done because of increased computational time and complexity. Instead, the F statistics from the resulting regression model were used to calculate *P *values for the cumulative f distribution; these *P *values were also calculated for the strain and brain region effects and used in the false discovery rate calculations to calculate the q values.

Note that, for the interaction terms, *δ*_*s*,*r*_, the summation is over all combinations of individual brain regions and strains, such that the *δ*_*s*,*r *_simply reflect the product of relevant strain and brain region 0-1 dummy variables. This formulation of interaction terms in regression models is standard in regression contexts. With our regression model, we could have tested each individual regression coefficient in the model for its deviation from 0.0 and hence been able to draw inferences about which brain regions or strains were most likely to deviate from the others in terms of expression level. However, although we included interaction terms in the full model, we chose not to focus on them because of potential overfitting and an insufficient number of observations. In order to identify interactions properly, we utilized a two-way ANOVA calculated using the 'anovan' function in Matlab, in which the least correlated unbalanced sample was removed. To test hypotheses on individual locus effects, we replaced the strain terms in the full model with a single locus effect (regression coefficient) term, *b*_*l*_, and an indicator variable, *x*_*i*,*j*,*k*_(*l*), set to 1 if observation *i*,*j*,*k *has a particular allele at locus *l *and 0 otherwise.

Pearson correlation coefficients were calculated using Excel. The formula used to transform correlations into distances is √(2 × [1 - R]), where R is the correlation coefficient. Mantel's matrix correspondence test was performed with 999 permutations and calculated using GenAlEx 6 [18].

eQTL analysis was performed using an in-house software program written in standard FORTRAN for Unix in which an F statistic from a regression model was used at each marker loci to test for an association. A two-factor regression model was used, similar to the previous analysis. Results were sorted and analyzed in a separate in-house C++ program. A marker was considered to be *cis*-acting if it was within 4 Mb of the start or end position of the gene of interest. Windows of 5 Mb and 2 Mb windows yielded similar results. The genomic start and end positions of a gene corresponding to the probe set was determined using the Entrez Gene IDs from the Affymetrix database, NetAffx [19]. Both the probe set positions and the SNP marker positions were aligned to NCBI Build 34 (Additional data files 4 and 5).

We note that our analysis of *cis*-acting and *trans*-acting eQTLs was simply meant to complement the single degree-of-freedom similarity matrix-based Mantel tests of the hypothesis that similarity in global gene expression patterns do not necessarily correlate with strain DNA sequence similarity, and hence is not meant to unequivocally or definitively identify variations that influence gene expression. It is in this context that we consider what we would expect to observe for our eQTL analyses if no relationship exists between mouse strain and brain region gene expression and the genetic variations the strains possess throughout the genome. To test the association of each locus to each probe set, we used the regression model described above, using the *P *value associated with the hypothesis that the regression coefficient, *b*_*l*_, was equal to 0 in a one degree of freedom *t*-test (no permutation tests were pursued). We make some simplifying assumptions in our calculations given the difficulty in accounting for correlations between the expression levels of the genes and the haplotype block patterns encompassing the SNPs we examined across the genome.

We note that we tested 8,680 loci (ignoring monomorphic and missing SNP information; see attached SNP data in Additional data file 4) for 22,048 probe sets in our eQTL analysis, for a total of 191,376,640 tests of association. We set a *P *value threshold of 0.001 to delineate loci worth considering as harboring *cis*-acting or *trans*-acting variations. We would thus expect 191,376 of these tests to produce *P *< 0.001 by chance alone if the expression values were independent of each other as well as the relationships between the strains with respect to regulatory variations in their genomes. We observed 3,225,220 associations with *P *< 0.001, which is much higher than expected. For the analysis of *cis*-acting eQTLs we note that we included SNPs within 4 Mb of each gene represented by a probe set as being located near enough to the gene to count as possibly *cis*-acting, and, on average, there were 29 SNPs within 4 Mb of each gene. We would expect that 640 tests (29 SNPs × 22,048 probe sets × 0.001 [*P *value cutoff]) would be needed to produce *P *< 0.001 by chance alone. We observed 2,955 probe sets with *P *< 0.001 for SNPs within 4 Mb of the physical positions of the probe sets.

### Polymorphism prediction

Candidate genes harboring predicted polymorphisms were identified using an algorithm developed by our laboratory (Greenhall and coworkers, unpublished data). Briefly, the algorithm works as follows. First, for the selected probe sets, the individual hybridization intensity values are extracted and the difference between the perfect match and the mismatch (PM-MM) intensities is calculated for each probe pair for each sample, excluding probe sets from samples that do not meet certain pattern quality measures. The PM-MM values for each of the probe sets for each sample are globally scaled (by a factor derived from the standard deviation across the multi-probe pattern obtained in each experiment) to compensate for gene expression differences. Next, the scaled values for each sample group are averaged across the strain, and an average and a standard deviation are calculated for each probe pair in a probe set. The appropriate degrees of freedom are calculated and the two-tailed Student's *t*-test (unpaired, equal variance) is derived for each probe pair for each strain comparison. The algorithm was written in C++ and runs on standard UNIX machines. The algorithm has been previously used and validated to identify sequence variation between inbred mouse strains [20] and between human, chimpanzee, and rhesus macaque [21]. The algorithm is in principle similar to two previously reported methods [14,22].

### Three-dimensional visualization of gene expression

Data containing signal intensity values from gene expression microarray analyses were imported in the NeuroZoom software (Neurome, La Jolla, CA, USA). Visualization of the signal intensities was performed as described previously [13].

## Additional data files

The following additional data are available with the online version of this paper. Additional data file 1 contains F statistics and *P *values for brain region, strain and interaction effects from the multiple regression model and two-way ANOVA. Additional data file 2 shows the number of strain-specific and brain region-specific probe sets with genetic *cis*-associations after removing probe sets with putative polymorphisms using a detection algorithm. Additional data file 3 provides detailed information regarding the methods used in the microarray data pre-processing. Additional data file 4 contains the SNP marker positions and genotypes. Additional data file 5 contains the genomic start and end positions of genes used in the eQTL analysis.

## Supplementary Material

Additional data file 1 This file contains F statistics and *P *values for brain region, strain, and interaction effects from the multiple regression model and two-way ANOVA; only genes that scored as 'Present' in at least one file are included. Click here for file

Additional data file 2 This table shows the number of strain-specific and brain region-specific probe sets with genetic *cis*-associations after removing probe sets with putative polymorphisms using a detection algorithm. Click here for file

Additional data file 3 This file includes detailed methods used in the microarray data pre-processing. Click here for file

Additional data file 4 This file contains the SNP marker positions and genotypes. Click here for file

Additional data file 5 This file contains the genomic start and end positions of genes used in the eQTL analysis. Click here for file
