# Supplementary material for: DNA variation and brain region-specific expression profiles exhibit different relationships between inbred mouse strains: implications for eQTL mapping studies
Source: Genome Biol. 2007 Feb 26;8(2):R25. doi: 10.1186/gb-2007-8-2-r25 (PMC1852412; doi:10.1186/gb-2007-8-2-r25)
Supplement: Additional data file 2 — This table shows the number of strain-specific and brain region-specific probe sets with genetic cis-associations after removing probe sets with putative polymorphisms using a detection algorithm. [file gb-2007-8-2-r25-S2.pdf]

p-value threshold	# of probe sets with no putative polymorphisms					
	strain specific			region specific		
	# cis	# total	% cis enrichment	# cis	# total	% cis enrichment
0.01	206	338	61%	347	3621	10%
0.001	529	870	61%	1081	10912	10%
0.0001	682	1189	57%	1472	14832	10%
0.00001	758	1392	54%	1640	16296	10%
1E-6	820	1548	53%	1703	16902	10%
1E-7	853	1662	51%	1747	17299	10%
1E-8	886	1738	51%	1786	17611	10%
1E-9	918	1834	50%	1808	17839	10%

Additional data file 2. Enrichment of probe sets with genetic cis-association at various p-value thresholds after ignoring probe sets harboring putative polymorphisms. Probe set signal values were scaled to take into account varying levels of gene expression. Then the hybridization pattern at each probe pair within the probe set was tested using a t-test between the strains to identify likely sequence variants. Probe sets determined to harbor a hybridization difference at any one of their probe pairs based on various p-value thresholds were eliminated from the analysis since they were deemed to harbor potential sequence variation that could affect probe hybridization and lead to false eQTL associations. The table shows the number of strain-specific or region-specific probe sets that still show cis-association after eliminating those probe sets with putative polymorphisms under the probe sequences at different p-value thresholds.
