# Supplementary material for: DNA variation and brain region-specific expression profiles exhibit different relationships between inbred mouse strains: implications for eQTL mapping studies
Source: Genome Biol. 2007 Feb 26;8(2):R25. doi: 10.1186/gb-2007-8-2-r25 (PMC1852412; doi:10.1186/gb-2007-8-2-r25)
Supplement: Additional data file 3 — This file includes detailed methods used in the microarray data pre-processing. [file gb-2007-8-2-r25-S3.pdf]

Supporting Methods

TeraGenomics analysis algorithms and criteria. The arrays are scanned and initially processed using the Affymetrix GCOS software to create the .CEL files. These .CEL files are then uploaded to the TeraGenomics database system via the internet and analyzed as previously described [23, 13, 20, 21]. A more thorough discussion of the TeraGenomics analysis follows. The .CEL files are normalized to a target intensity of 200 using a normalization procedure similar to Affymetrix MAS 5.0. In short, a scaling factor is multiplied to the experimental output to make the experimental output's average intensity equal to an arbitrary target intensity (default = 200) [24, 25]. Scaling allows a number of experiments to become normalized to one target intensity, allowing comparison between any two experiments. In order to calculate the scaling factor, the arbitrary target intensity is divided by a modified average intensity for the experimental output. The modified average intensity for the experimental output is the average of the smoothed 65th percentile of the probe set intensity (discussed in depth below) ignoring the highest 10% and the lowest 60% of probe set intensities. Ignoring the highest 10% and the lowest 60% of intensities produced scaling factors that were consistent and well-behaved (e.g., the mean and median of the resulting distributions were approximately equal). More important, scaling factors calculated in this way consistently resulted in the smallest number of genes scoring as "differentially expressed" between replicates (unpublished).

In order to calculate an average signal across an entire probe set, TeraGenomics utilizes a smoothed version of the 65th percentile. The calculation is done as follows:

- Take the difference between the perfect match (PM) and mismatch (MM) (PM-MM) for each probe pair.
- Rank the differences, from smallest to largest, employing tied ranks where appropriate.
- Average the 65th percentile value, the two ranked signals in front of the 65th percentile value and the two ranked signals behind the 65th percentile value for probe sets with greater than 15 probe pairs. For probe sets with less than 16 probe pairs the 65th percentile value and the rank signal in front and the rank signal behind are averaged together.

The smoothed 65th percentile is a much simpler calculation than the previous calculation in the Affymetrix software of a trimmed mean. However, the 65th percentile performs similar to the trimmed mean. In addition, the 65th percentile consistently provides higher correlation between replicate samples (unpublished).

In addition to determining if a signal is "detectable" for an appropriate probe set, a measure of probability is given by calculating a variety of statistics. It is important to note that analyses in TeraGenomics account for the behavior of all probe pairs in a given probe set. Thus, the positive fraction (**Positive Fraction**) is a measure of the fraction of all probe set probe pairs in which the perfect match (**PM**) probe cells hybridize to a target at a greater level than the control mismatch (**MM**) probe cells.

The analysis algorithm applies a total of 4 common statistical tests to analyze whether the population of perfect match signals is greater than the signals obtained from the mismatch population. The Binominal Distribution (**BD p-value**) generates a statistic that measures how far the observed distribution of perfect match and mismatch probe cells deviates from a random chance distribution between both populations in a probe set. The Student's Paired Two-Tailed T-Test calculates a statistic (**t-test p-value**) that is a measure for the likelihood

that there is a significant difference between the means of the perfect match and mismatch probe populations.

TeraGenomics employs the non-parametric Wilcoxon Signed-Rank Test to generate two-tailed p-values for matched probe pairs using either the absolute difference between perfect match and mismatch probes or their difference relative to the sum of the intensities of perfect and mismatch probes as a means of ranking (**WSRA p-value**, **WSRR p-value**). Finally, the algorithm generates an absolute call (**P,M,A,RP**) for the qualitative determination of whether a probe set is detectable. Calls are generated after comparing the WSRR p-value **and** the positive fraction metric against empirically determined thresholds. A call of reverse present (**RP**) indicates that the population of mismatch signals is greater than the perfect match signal population in a statistically significant fashion.

The absolute call is designed to provide a qualitative determination of whether there is clear evidence that a specific mRNA is present in the hybridization sample. This determination is based on the observed hybridization pattern across all the probes in a probe set, and is meant to indicate whether the mRNA is clearly detectable (Present), marginally detectable (Marginal), or not detectable (Absent). The call algorithm uses the collection of (PM-MM) values across a probe set, and is based on the p-value (paired, two-tailed) calculated using the non-parametric Wilcoxon signed rank test (relative) as well as the "positive fraction" (PF, the fraction of PM-MM values that are greater than zero). Additionally a call of Reverse Present is made when the population of MM signals is greater than the PM signals. The call thresholds were set to maximize sensitivity to low abundance mRNAs while keeping the false positive rate to a minimum, and were based on an analysis of a large amount of experimental data. A call of Present (P) is made when $p \leq 0.0316$ and the positive fraction ≥ 0.6 or when $p \leq 0.1$ and the positive fraction ≥ 0.75. A call of Marginal (M) is made when $0.0316 < p \leq 0.1$ and $0.6 \leq$ positive fraction < 0.75, or not called P and positive fraction ≥ 0.75. A call of Reverse Present (RP) is made when $p \leq 0.0316$ and the positive fraction ≤ 0.4, or $p \leq 0.1$ and the positive fraction ≤ 0.25. A call of Absent (A) is made for all other probe sets that do not fall into the above categories. For further information, please visit TeraGenomics [16].
