# Supplementary material for: DNA variation and brain region-specific expression profiles exhibit different relationships between inbred mouse strains: implications for eQTL mapping studies
Source: Genome Biol. 2007 Feb 26;8(2):R25. doi: 10.1186/gb-2007-8-2-r25 (PMC1852412; doi:10.1186/gb-2007-8-2-r25)
Supplement: Additional data file 4 — This file contains the SNP marker positions and genotypes. [file gb-2007-8-2-r25-S4.pdf]

Additional data file 4. SNP positions from NCBI Build 34 and genotypes of the mouse strains used in this study.

SNP_ID	rsSNP	Chr	Pos	129S1/SvImJ	A/J	C3H/HeJ	C57BL/6J	DBA/2J	FVB/NJ
1	rs6396997	6	58100664	G	G	G	G	G	G
2	rs3164478	1	133269119	G	A	A	A	G	G
3	rs6357429	1	3007431	G	G	G	G	G	G
4	rs3683945	1	3201415	A	A	G	A	A	A
5	rs3707673	1	3410201	G	G	A	G	G	G
6	rs6269442	1	3495168	G	A	G	A	G	G
7	rs2228910	1	3555923	C	C	C	C	C	C
8	rs6336442	1	3583692	A	A	G	A	G	A
9	rs4140116	1	3584226	G	G	G	G	G	G
10	rs6243160	1	4171743	A	A	A	A	G	A
11	rs6198069	1	4731989	G	A	G	G	G	G
12	rs3659303	1	4778495	A	A	G	A	G	A
13	rs4230167	1	4782615	G	G	G	G	C	G
14	rs3674785	1	4873527	G	G	A	G	G	G
15	rs6376963	1	5030491	C	C	C	C	A	C
16	rs3677817	1	5197303	G	G	A	A	G	G
17	mCV23863067	1	8408319	C	C	G	G	G	C
18	rs8236463	1	5600331	T	T	T	T	A	T
19	rs8236467	1	5614153	G	G	G	G	G	G
20	rs8236468	1	5614198	A	A	A	A	A	A
21	rs3710263	1	6071196	G	A	G	G	A	A
22	rs6333200	1	6242057	A	G	G	A	G	G
23	rs6298633	1	6845155	A	A	G	A	G	A
24	rs3667401	1	7167592	A	G	G	G	G	G
25	mCV24839713	1	10578928	G	A	G	G	G	G
26	rs6321307	1	7691737	A	A	C	A	A	A
27	rs3707314	1	7701608	A	A	C	C	C	A
28	rs3717997	1	7879984	A	A	G	G	G	A
29	rs3684358	1	8111551	A	A	C	C	C	A
30	rs3679592	1	8542265	A	A	A	G	A	A
31	rs3716083	1	9092463	A	G	A	G	A	G
32	rs6382958	1	9228418	G	G	G	G	G	G
33	rs3665459	1	9328928	G	G	G	A	A	G
34	rs6195532	1	9696644	A	A	A	A	G	A
35	rs3667692	1	9786644	G	G	G	A	G	G
36	rs3680898	1	9871859	C	C	G	G	G	C
37	rs3703103	1	10010177	A	A	A	T	A	A
38	rs3698285	1	10098141	A	A	A	G	G	A
39	rs3678510	1	10752289	G	G	A	A	A	G
40	rs3697012	1	10980605	T	T	A	T	T	T
41	mCV24107897	1	14297381	C	G	G	G	G	G
42	rs6360236	1	11163199	C	C	A	A	C	C
43	rs6372808	1	11203312	C	C	C	C	C	C
44	rs3722996	1	11348581	A	A	C	A	C	A
45	rs3724763	1	11348812	A	A	G	A	G	A
46	rs4222149	1	11480996	A	A	G	A	G	A
47	rs3687812	1	11544080	A	A	C	A	A	A
48	rs6228313	1	11975086	A	G	G	G	A	G
49	rs3715450	1	12006929	T	T	A	A	T	T
50	rs3681537	1	12027164	C	C	A	A	C	C
51	rs2020774	1	12431293	C	C	C	C	C	C
52	rs3726952	1	12906283	G	G	G	A	A	G
53	rs3654377	1	13330054	A	A	A	G	G	A
54	rs3655978	1	13486779	G	G	G	A	G	G
55	rs3670630	1	13638736	C	C	C	A	C	C
56	rs3675633	1	13740462	C	A	A	C	A	A
57	rs3654866	1	13815557	G	G	G	A	G	G
58	rs3669485	1	14102655	A	A	A	G	A	A
59	rs3088721	1	14166456	T	T	T	T	T	T
60	rs8253369	1	14385276	C	C	C	C	C	C
61	rs8253368	1	14385715	C	C	C	C	C	C
62	rs8253364	1	14386024	A	A	A	A	A	A
63	rs3713198	1	14629358	C	C	C	G	C	C
64	rs3723784	1	14838567	G	G	G	A	A	G
65	rs3664075	1	14878509	A	A	A	G	G	A
66	rs6291839	1	14957062	A	G	G	G	A	G
67	rs3088964	1	15365996	C	C	C	C	A	C
68	rs3708560	1	15552631	A	G	G	G	G	G
69	rs3678179	1	15668758	A	A	A	T	A	A
70	rs3669513	1	15720686	A	A	A	C	A	A
71	rs6201380	1	15973423	G	A	A	A	G	A

72	rs6278832	1	16392616	G	G	G	G	G	G
73	rs6279455	1	16392813	A	A	A	A	A	A
74	rs3671256	1	16443156	T	T	T	A	T	T
75	rs3706411	1	16448089	A	A	A	T	T	A
76	rs6389205	1	16598344	A	T	T	A	A	T
77	rs6199072	1	17368383	A	A	A	A	A	A
78	rs3675398	1	17751670	G	A	A	A	A	A
79	rs6270008	1	17929623	G	A	A	A	A	A
80	rs6398620	1	18560717	A	T	T	T	T	T
81	rs3656562	1	18692924	G	A	A	A	A	A
82	mCV23695025	1	22386585	G	G	G	A	G	G
83	rs4222153	1	19445316	A	A	A	A	A	A
84	rs3658044	1	19498836	T	A	A	T	T	A
85	rs4222165	1	19540757	A	A	A	A	A	A
86	rs3718050	1	19900658	A	A	A	C	C	A
87	rs6336494	1	19965523	G	G	G	A	A	G
88	rs3720411	1	20266680	A	G	G	A	A	G
89	rs6334092	1	20368047	C	A	A	C	C	A
90	rs3687041	1	20634051	G	A	A	G	G	A
91	rs3670611	1	20841621	A	A	A	G	A	A
92	rs6404446	1	20994482	A	A	A	C	A	A
93	rs3662305	1	21144565	G	G	G	A	G	G
94	rs3088778	1	21223535	A	A	A	G	A	A
95	rs3704179	1	21253854	A	G	G	A	G	G
96	mCV24781171	1	24680601	A	G	G	A	G	G
97	rs8243843	1	21452574	A	A	A	A	A	A
98	rs8243886	1	21468418	A	A	A	A	A	A
99	rs6253968	1	21852704	G	G	G	A	G	G
100	mCV24784983	1	25471405	A	C	C	A	C	C
101	rs3711079	1	22156681	A	A	A	G	A	A
102	rs3682202	1	22168773	G	G	G	A	G	G
103	rs3089410	1	22181163	A	A	A	A	A	A
104	rs3708897	1	22578801	G	A	A	A	A	A
105	rs3672208	1	22921227	G	A	A	G	A	A
106	rs6173215	1	22977896	A	G	G	A	G	G
107	rs3691092	1	23017895	G	A	A	G	A	A
108	rs3090015	1	23088053	A	A	A	A	A	A
109	rs3659806	1	23400609	A	C	C	A	C	C
110	rs6351482	1	23424241	C	G	G	C	G	G
111	rs3695916	1	23514086	G	A	A	G	A	A
112	rs3682782	1	23897617	G	G	G	G	G	G
113	mCV22980985	1	27363304	T	A	A	T	A	A
114	rs4222215	1	24234159	A	G	G	A	G	G
115	rs4222217	1	24234275	G	C	C	G	C	C
116	rs4222220	1	24234447	A	A	A	A	A	A
117	rs6231303	1	24426016	A	A	A	A	A	A
118	mCV25266528	1	28043045	C	A	A	C	A	A
119	rs4222227	1	25005587	A	A	A	A	A	A
120	rs4222228	1	25005693	A	A	A	A	A	A
121	rs6342281	1	25022769	A	A	A	A	A	A
122	rs3711129	1	25607261	A	A	A	G	A	A
123	rs3657464	1	25686911	G	G	G	A	G	G
124	rs6166266	1	26136349	A	C	C	A	C	C
125	rs3673503	1	26267798	A	A	A	C	A	A
126	rs3686971	1	26426004	A	G	G	G	G	G
127	rs6380710	1	26459079	A	C	C	C	C	C
128	rs3709207	1	26590966	A	C	C	A	C	C
129	rs3690896	1	26999255	A	A	A	A	A	A
130	rs3660438	1	27267888	A	A	A	G	A	A
131	rs3677683	1	27510356	G	G	G	A	G	G
132	rs3692609	1	27510406	C	C	C	A	C	C
133	rs6218992	1	27526370	A	A	A	T	A	A
134	rs6192641	1	28145674	G	A	A	G	A	A
135	rs3666231	1	28664680	A	G	G	G	G	G
136	rs3711203	1	28973206	A	A	A	G	A	A
137	rs3713281	1	29201052	A	A	A	C	A	A
138	rs6384194	1	29421983	A	A	A	G	A	A
139	rs3719194	1	29770616	C	C	C	A	C	C
140	rs3660938	1	29770817	A	A	A	G	A	A
141	rs6288570	1	29898295	A	A	A	A	A	A
142	rs6168330	1	29987616	A	G	G	A	G	G
143	rs3695988	1	29990732	C	C	C	A	C	C
144	rs3653504	1	30103194	A	A	A	C	A	A
145	rs3722689	1	30237048	G	G	G	A	G	G

146	rs3722005	1	30710165	G	A	A	A	A	A
147	rs6237824	1	30982111	C	C	C	A	C	C
148	rs4137502	1	31102378	G	A	A	G	A	A
149	rs6329963	1	31193026	A	G	G	A	G	G
150	rs3676270	1	31435680	G	A	A	A	A	A
151	rs3724031	1	31440272	C	A	A	A	A	A
152	rs6209502	1	31728284	T	T	T	T	T	T
153	rs3712692	1	32012045	C	C	C	C	C	C
154	rs3678653	1	32094986	A	G	G	G	G	G
155	rs3655881	1	32740123	G	G	G	A	A	G
156	rs6321337	1	32742292	A	A	A	G	G	A
157	rs3707642	1	32799125	A	A	A	C	A	A
158	rs3088844	1	32986387	A	A	A	A	C	A
159	rs3703203	1	33280447	A	C	C	C	A	C
160	rs3664658	1	33465930	A	A	A	A	G	A
161	rs3690508	1	33532109	A	C	C	A	C	C
162	rs3725230	1	33616011	A	G	G	A	G	G
163	mCV23749183	1	37637629	A	G	G	A	G	A
164	rs6379161	1	33819378	A	G	G	G	G	G
165	rs6176536	1	34313457	G	C	C	G	G	C
166	rs3681732	1	34414162	G	G	G	A	A	G
167	rs3709457	1	34457593	A	G	G	A	A	G
168	rs6282096	1	34523471	A	T	T	T	T	T
169	rs4222252	1	34728973	G	G	G	G	G	G
170	mCV25004024	1	38715755	T	T	T	T	A	A
171	rs4222264	1	34830256	A	A	A	A	A	A
172	rs6251423	1	35047722	G	A	A	G	A	A
173	rs6279250	1	35094165	G	G	G	C	G	G
174	rs3657255	1	35323998	A	A	A	G	G	A
175	rs3687379	1	35737360	C	G	G	C	C	G
176	rs6401503	1	35862251	C	C	C	C	A	C
177	rs3022778	1	36009624	C	C	C	C	C	C
178	mCV23414826	1	40111128	A	A	A	C	C	A
179	rs3683997	1	36198091	G	A	A	G	G	A
180	rs3684440	1	36480288	A	G	G	A	G	A
181	mCV24644372	1	40449019	A	G	G	A	A	A
182	mCV24153753	1	41423174	A	C	C	C	A	A
183	rs6255606	1	37662659	A	G	G	A	G	A
184	rs4222275	1	37732620	A	C	C	A	C	A
185	rs3692113	1	37808998	A	A	A	G	A	A
186	rs6164278	1	38757090	A	A	A	A	G	A
187	rs3706514	1	38820158	A	A	A	T	A	A
188	rs3707984	1	38829792	A	A	A	G	A	A
189	rs3685569	1	38871455	A	A	A	G	A	A
190	rs4222292	1	39687384	G	G	G	G	G	G
191	rs4222297	1	39320282	A	G	G	A	G	G
192	rs6387241	1	39621383	G	A	A	G	G	G
193	rs6366151	1	39653238	C	C	C	G	G	G
194	rs3665127	1	39735558	A	G	G	A	A	A
195	mCV25154866	1	43374960	A	G	G	G	G	G
196	rs3680400	1	40179424	A	A	A	G	G	G
197	rs6385050	1	40215549	G	G	G	G	G	G
198	rs3090550	1	40639069	G	G	G	G	G	G
199	rs4222303	1	40691309	A	A	A	A	A	A
200	rs6247051	1	40764710	A	A	A	A	A	A
201	rs8237691	1	40765736	A	A	A	A	A	A
202	rs3677272	1	40907247	G	G	G	A	A	G
203	rs3708475	1	41013071	A	C	C	A	A	A
204	rs3723444	1	41013127	G	C	C	G	G	G
205	rs3696088	1	41156578	C	G	G	C	C	C
206	rs4222307	1	41184728	G	G	G	G	G	G
207	rs3706173	1	41800892	G	A	A	G	G	G
208	rs3089696	1	41804355	G	G	G	G	G	G
209	rs3671534	1	41942580	A	C	C	C	A	A
210	rs6262666	1	41976768	G	A	A	G	G	G
211	rs3667075	1	42249198	G	A	A	G	G	G
212	rs3663706	1	42337818	A	A	A	G	G	G
213	rs3661842	1	42344022	T	T	T	A	A	A
214	rs6269695	1	42402297	A	A	A	A	A	A
215	mCV23249065	1	45942199	C	C	C	A	C	C
216	rs3675140	1	42818754	A	A	A	G	G	A
217	rs3715199	1	42975729	A	C	C	C	C	C
218	rs6208403	1	43106815	G	G	G	G	G	G
219	rs4222315	1	43444336	G	G	G	G	G	G

220	mCV23614325	1	43902930	A	A	A	C	A	A
221	rs3662842	1	43777889	G	G	G	A	G	A
222	rs6187198	1	43783321	G	G	G	G	G	G
223	rs3088589	1	43820556	A	A	A	A	A	A
224	mCV23611562	1	47908310	G	A	A	A	A	A
225	rs8253394	1	44490396	G	A	A	G	A	A
226	rs6328990	1	44490756	NONE	G	G	G	G	G
227	rs6329563	1	44490870	C	A	A	A	A	A
228	rs8253429	1	44510235	A	G	G	A	G	A
229	rs4222319	1	44556886	C	C	C	C	C	C
230	mCV25321857	1	49276410	G	G	G	A	G	G
231	rs6287344	1	45585331	G	G	G	G	G	G
232	rs8259907	1	45663969	G	G	G	G	G	G
233	rs3091082	1	45705059	A	A	A	A	A	C
234	rs3655885	1	45794573	T	T	T	A	T	A
235	rs3668645	1	45803282	A	A	A	G	A	G
236	rs6370110	1	45866589	A	A	A	G	A	A
237	rs6161243	1	46253419	A	A	A	A	A	A
238	mCV23596299	1	50101434	G	A	A	A	A	A
239	rs6322563	1	47222530	G	G	G	A	G	G
240	rs3714543	1	47399793	A	G	G	A	G	G
241	rs6248985	1	47628442	G	G	G	G	G	G
242	mCV23591750	1	51617463	A	T	T	A	T	T
243	rs6181164	1	47960648	G	A	A	A	A	A
244	rs3661491	1	48378539	A	G	G	G	G	G
245	rs6350409	1	48440490	C	C	C	C	C	C
246	rs3682720	1	48533511	G	G	G	C	G	G
247	rs6311681	1	49120883	T	T	T	T	T	T
248	rs3659331	1	49569069	G	G	G	A	G	G
249	rs6260801	1	50005628	A	T	T	T	T	T
250	rs6201240	1	50325597	G	A	A	A	A	A
251	rs6281180	1	50578107	A	A	A	A	A	A
252	rs3724092	1	50613798	A	A	A	C	A	A
253	rs3724707	1	50613890	A	A	A	G	A	A
254	rs3672520	1	50947514	A	G	G	G	G	G
255	rs3699422	1	51473061	G	G	G	G	G	G
256	rs6232640	1	51733707	A	A	A	A	A	A
257	rs4222323	1	51837872	A	A	A	A	A	A
258	rs3663063	1	51913800	G	A	A	A	A	A
259	rs3725203	1	52176938	A	G	G	G	G	G
260	rs3667466	1	52222673	G	A	A	A	A	A
261	rs3668739	1	52222864	G	A	A	A	A	A
262	rs4222335	1	52273996	A	A	A	A	A	A
263	rs6217547	1	52308389	G	A	A	A	A	A
264	rs8239131	1	52524284	A	A	A	A	A	A
265	rs6252183	1	53123445	G	G	G	G	G	G
266	rs6253368	1	54162484	A	A	A	A	A	A
267	rs3666880	1	55369154	C	C	C	C	C	C
268	rs3668074	1	55369352	C	C	C	C	C	C
269	rs4222342	1	55369837	A/G	G	G	G	A/G	G
270	mCV23023127	1	59472326	A	A	A	A	A	A
271	rs6241350	1	55683457	A	A	A	A	A	A
272	rs3723035	1	55942220	G	G	G	G	G	G
273	rs3726233	1	56253497	A	A	A	A	A	A
274	rs6287068	1	56566321	T	T	T	T	T	T
275	rs6190280	1	56936475	A	A	A	A	A	A
276	rs3708772	1	57136600	G	G	G	G	G	G
277	rs3653534	1	57196154	A	A	A	A	A	A
278	rs3089908	1	57221022	G	G	G	G	G	G
279	rs3022801	1	57221053	G	G	G	G	G	G
280	rs6341554	1	57291136	A	A	A	A	A	A
281	rs4222363	1	57804780	A	A	A	A	A	A
282	rs3679641	1	58003090	G	G	G	G	G	G
283	rs8236470	1	58422123	A	A	A	A	A	A
284	rs8238898	1	58447431	G	G	G	G	G	G
285	rs8238935	1	58470193	A	A	A	A	A	A
286	rs8238949	1	58471013	G	G	G	G	G	G
287	rs3686293	1	58509052	C	C	C	C	A	C
288	rs4222372	1	58592916	G	A	A	A	G	A
289	rs3663226	1	58629442	A	G	G	G	G	G
290	rs3725715	1	58807423	C	G	G	G	C	G
291	rs4222380	1	58995519	A	A	A	A	A	A
292	rs4222383	1	59247476	G	G	G	G	G	G
293	rs4222381	1	59247796	A	A	A	A	A	A

294	rs6245700	1	59523855	A	C	C	A	A	C
295	rs3713747	1	59568162	A	G	G	G	G	G
296	rs3708287	1	59581890	G	A	A	A	A	A
297	rs3675875	1	59616435	A	G	G	G	G	G
298	rs6282881	1	60684365	G	G	G	G	G	G
299	rs3661555	1	61165706	G	G	G	G	G	G
300	rs8254826	1	62973583	G	G	G	G	G	G
301	rs6249719	1	61922858	A	A	A	A	A	A
302	rs3716105	1	61943426	A	A	A	A	A	A
303	rs6257119	1	62383801	A	A	A	A	A	A
304	mCV24306886	1	66803983	A	A	A	G	A	A
305	rs6244074	1	63000271	G	G	G	A	G	G
306	rs3688436	1	63053082	G	G	G	A	G	G
307	rs3699303	1	63057729	C	C	C	A	C	C
308	rs3690322	1	63062858	C	C	C	A	C	C
309	rs6322485	1	63649681	A	A	A	T	A	A
310	rs3662027	1	63696981	A	A	A	G	A	A
311	rs3703813	1	63972106	A	A	A	G	A	A
312	rs4222401	1	63988445	G	G	G	G	G	G
313	rs3088612	1	64741555	C	C	C	C	C	C
314	rs3699216	1	64808554	G	G	G	A	G	G
315	rs3726861	1	65086580	G	G	G	A	A	G
316	rs3654452	1	65086845	A	A	A	G	G	A
317	rs3679650	1	65091475	G	G	G	A	A	G
318	mCV24085696	1	69168683	G	G	G	A	G	G
319	rs6293581	1	65143788	G	G	G	A	A	G
320	rs6395308	1	65272783	G	G	G	G	A	G
321	rs4222404	1	65353420	A	A	A	A	A	A
322	rs4222405	1	65353510	G	G	G	G	G	G
323	rs3089386	1	65446024	A	A	A	A	A	A
324	rs3022802	1	65446114	C	C	C	A	C	C
325	rs4137302	1	65909810	G	G	G	A	G	G
326	rs6194369	1	66121673	G	G	G	G	G	G
327	rs3719486	1	66298840	A	A	A	G	A	A
328	rs6162259	1	66387841	G	G	G	G	G	G
329	rs3722989	1	67112327	G	G	G	A	G	G
330	rs4222406	1	67193344	G	G	G	A	G	G
331	rs3712639	1	67373031	A	A	A	C	A	A
332	rs3698088	1	67742707	G	G	G	A	G	G
333	rs6368250	1	67981741	A	A	A	G	A	A
334	rs3665792	1	68182670	G	G	G	A	G	G
335	rs3697638	1	68256628	G	G	G	A	G	G
336	rs3022803	1	68282629	C	C	C	A	C	C
337	rs3720839	1	68492187	A	G	G	G	A	G
338	rs6288543	1	68917890	A	A	A	T	A	A
339	rs3688421	1	68960316	G	G	G	A	G	G
340	rs3681669	1	69536221	G	G	G	A	G	G
341	rs3690668	1	69556576	C	C	C	A	C	C
342	rs3659932	1	69663163	A	A	A	G	A	A
343	mCV23057534	1	74035890	C	C	C	A	C	A
344	rs6332333	1	70916947	G	G	G	G	G	G
345	rs4222410	1	71141705	G	G	G	G	G	G
346	rs4222413	1	71494310	A	A	A	A	A	A
347	mCV22629698	1	76106271	C	A	A	C	C	C
348	rs6191076	1	72377589	A	C	C	A	A	A
349	rs3722564	1	72536254	C	A	A	C	C	C
350	rs4222426	1	72822922	G	A	A	G	G	G
351	mCV24115911	1	76984491	G	A	A	G	G	A
352	rs2020469	1	72892720	A	A	A	A	A	A
353	rs3687595	1	73120598	G	A	A	G	A	G
354	rs6163328	1	73243855	C	A	A	C	C	C
355	rs4222429	1	73294036	A	A	A	A	A	A
356	rs6214216	1	73298313	A	A	A	A	A	A
357	rs3021883	1	73308713	G	A	A	G	G	G
358	rs3665947	1	73492476	A	C	C	A	A	A
359	mCV22884877	1	78129333	A	A	A	G	G	A
360	mCV23042608	1	78210733	G	A/G	A/G	G	G	A/G
361	rs6238696	1	74205245	G	G	G	G	G	G
362	rs3678350	1	74506287	C	C	C	A	C	A
363	rs8253451	1	74619619	G	G	G	G	G	G
364	rs3713616	1	74900046	G	G	G	A	G	A
365	rs4222453	1	74988989	G	G	G	G	G	G
366	rs3089921	1	75146756	G	G	G	G	G	G
367	rs6356603	1	75362320	A	T	T	A	T	A

368	rs3702635	1	75930259	A	C	C	C	A	C
369	rs4222458	1	75955813	C	C	C	C	C	C
370	mCV23431007	1	80839073	G	G	G	G	A	G
371	rs3680779	1	76295249	A	G	G	A	A	A
372	rs6237227	1	76302351	A	C	C	A	A	A
373	rs3090333	1	76369467	A	A	A	A	A	A
374	rs3689327	1	76410223	G	A	A	G	G	G
375	rs3676306	1	76655143	G	C	C	G	G	G
376	rs3699038	1	76846395	A	C	C	C	A	A
377	rs6382116	1	76875875	A	A	A	A	A	A
378	mCV23433457	1	81493440	T	T	T	A	A	T
379	rs3090737	1	77145500	A	A	A	A	A	A
380	rs3715800	1	77348073	G	G	G	A	A	G
381	rs3670525	1	77408016	G	G	G	A	A	G
382	rs6200201	1	77599384	A	A	A	G	G	G
383	rs3722710	1	77684642	G	A	A	G	G	G
384	rs3672387	1	77953642	G	G	G	A	A	G
385	rs3703285	1	78023442	G	A	A	G	G	A
386	rs6321468	1	78484909	A	A	A	G	G	A
387	rs3091091	1	78557484	G	G	G	G	G	G
388	rs3667200	1	78622208	G	G	G	A	A	G
389	rs4222463	1	78586698	A	A	A	A	A	A
390	rs4222459	1	78586608	G	G	G	G	G	G
391	rs6182343	1	78786284	A	A	A	G	G	A
392	rs4222472	1	78895325	A	A	A	A	A	A
393	rs6401883	1	79039814	A	A	A	A	A	A
394	rs3692514	1	79247521	G	A	A	A	A	G
395	rs8253472	1	79953904	G	G	G	G	G	G
396	rs8253473	1	79954389	A	A	A	C	C	A
397	rs4222478	1	79954778	A	A	A	A	A	A
398	rs8253469	1	79954839	G	G	G	G	G	G
399	rs3692549	1	80277258	C	C	C	A	A	C
400	rs6157189	1	80279272	A	A	A	A	A	A
401	rs3675339	1	80417261	C	C	C	G	G	C
402	rs6379484	1	81076550	A	A	A	A	A	A
403	rs6306738	1	81826643	C	C	C	C	C	C
404	rs6290463	1	81891125	A	A	A	G	G	G
405	rs6207863	1	82384635	A	A	A	T	T	T
406	rs3708204	1	82599182	C	A	A	C	C	C
407	rs3682229	1	82603755	G	A	A	G	G	G
408	rs6329101	1	82888512	A	A	A	A	A	A
409	rs4222480	1	83783360	G	G	G	G	G	G
410	rs3089629	1	84621062	C	C	C	C	C	C
411	rs4222486	1	84836130	A	A	A	A	A	A
412	rs6335678	1	84842577	A	A	A	A	A	A
413	rs6154738	1	85304810	C	C	C	C	C	C
414	rs8253487	1	85545924	G	G	G	G	G	G
415	rs8253485	1	85546004	G	G	G	G	G	G
416	rs8253483	1	85546254	A	A	A	A	A	A
417	rs3678377	1	85674579	G	G	G	A	G	G
418	rs6337132	1	85793140	C	C	C	C	C	C
419	rs3723062	1	85865441	A	A	A	G	A	A
420	rs3694406	1	86305470	A	A	A	G	A	A
421	rs4222490	1	86428221	G	G	G	G	G	G
422	rs3690292	1	86488097	A	A	A	G	G	A
423	rs3669598	1	86805316	A	A	A	C	C	A
424	rs6281921	1	86846297	G	G	G	G	G	G
425	rs8252886	1	87095892	G	G	G	G	G	G
426	rs8252888	1	87095918	T	T	T	T	T	T
427	rs8252890	1	87096046	A	A	A	A	A	A
428	rs4222496	1	87103024	G	G	G	G	G	G
429	rs6389062	1	87847249	A	A	A	A	A	A
430	mCV22863351	1	90003736	A	A	A	A	G	A
431	rs3655427	1	88032840	G	G	A	A	G	G
432	rs8258190	1	88110441	T	T	T	T	T	T
433	rs8258188	1	88111476	G	G	G	G	G	G
434	rs3670389	1	88653077	G	G	A	A	G	G
435	rs3693508	1	88905194	C	C	C	C	C	C
436	rs4222508	1	89065860	G	G	A	A	A	G
437	rs4222507	1	89065986	G	G	A	A	A	G
438	rs6189552	1	89442405	A	A	A	A	A	A
439	mCV24380249	1	92063384	T	T	T	T	A	T
440	rs3684025	1	90370182	A	A	G	A	A	A
441	rs3089918	1	90567388	A	A	A	A	A	A

442	rs3022827	1	90567434	G	G	A	G	G	G
443	rs3702854	1	90733001	G	A	A	A	A	G
444	rs4135536	1	90815588	A	G	A	G	G	A
445	rs4138577	1	90920916	A	G	G	G	G	A
446	rs3687969	1	91016311	G	A	A	G	G	G
447	rs6356583	1	91202338	G	G	G	G	G	G
448	rs4222523	1	91481659	A	A	A	A	A	A
449	rs3686660	1	91493572	G	A	A	A	G	G
450	rs3664800	1	91752577	G	A	A	G	G	G
451	rs6285286	1	91880671	G	G	G	G	G	G
452	mCV24377815	1	93847721	G	G	G	A	G	G
453	rs3089830	1	92335881	A	A	A	A	A	A
454	rs6345367	1	92546322	A	A	A	A	A	A
455	rs8277054	1	92879113	A	A	A	A	A	A
456	rs4222530	1	92881578	A	A	A	A	A	A
457	rs8239165	1	92974654	C	C	C	C	C	C
458	rs3723232	1	93016179	A	A	A	G	A	G
459	mCV24817024	1	95061106	G	C	C	C	G	C
460	rs3683358	1	93162732	G	G	A	A	G	G
461	rs3692309	1	93188277	A	A	A	C	A	A
462	rs6268443	1	93319361	G	G	G	A	G	G
463	rs3088531	1	93398192	G	G	G	G	G	G
464	rs6308266	1	93565178	G	G	G	G	G	G
465	rs4221925	1	93739088	A	A	A	A	A	A
466	rs6334006	1	94492281	C	C	C	C	C	C
467	rs3675505	1	95019059	G	A	A	A	G	A
468	rs6221119	1	95225416	G	G	G	G	G	G
469	rs4136618	1	95735262	G	A	A	A	G	A
470	rs6175979	1	95799863	G	A	A	A	G	A
471	rs6169995	1	95917602	A	A	A	A	A	A
472	rs3726669	1	96875139	A	T	T	T	A	T
473	rs6276969	1	97652743	G	G	G	G	G	G
474	rs8260479	1	97734334	A	A	A	A	A	A
475	rs3088469	1	98013583	A	A	A	A	A	A
476	rs6343692	1	98338303	G	G	G	G	G	G
477	rs3695980	1	99464143	A	T	T	T	A	T
478	rs3668458	1	99468628	A	G	G	G	A	G
479	rs6169301	1	99478610	G	G	G	G	G	G
480	rs6226332	1	99755580	T	T	T	T	T	T
481	mCV23748993	1	101977176	C	A	A	A	C	A
482	rs3685663	1	100268247	A	G	G	G	A	G
483	rs6238133	1	100514988	G	G	G	G	G	G
484	rs6177448	1	101418384	G	G	G	G	G	G
485	rs3655293	1	101731906	C	A	A	A	C	A
486	rs3719046	1	101871731	NONE	A	A	A	G	A
487	rs6191585	1	102177808	T	T	T	T	T	T
488	mCV25151171	1	105302182	A	A	A	G	A	A
489	rs6365547	1	103144363	C	C	C	C	C	C
490	rs3707664	1	103345801	G	G	G	G	G	G
491	rs6370992	1	104865066	T	T	T	T	T	T
492	rs3707424	1	105241938	G	A	A	G	G	A
493	rs3696200	1	105266613	T	T	T	A	T	T
494	rs3692731	1	105952948	G	A	A	G	G	A
495	rs3090077	1	106508879	G	G	G	G	G	G
496	rs4222565	1	106854872	A	A	A	A	A	A
497	rs6170540	1	106913583	A	A	A	A	A	A
498	rs3664301	1	107059245	A	G	G	A	A	G
499	rs6398681	1	107588935	A	T	T	T	A	T
500	rs3088689	1	107737982	T	T	T	T	T	T
501	rs3689841	1	108045516	T	A	A	A	T	A
502	rs6194816	1	108229496	C	C	C	C	C	C
503	rs3663003	1	108755734	C	C	C	A	C	C
504	rs3685665	1	108829325	A	C	A	C	A	C
505	rs6405157	1	109534583	A	A	A	A	A	A
506	rs3685919	1	109634975	A	A	A	G	A	A
507	mCV22824651	1	112342512	A	A	A	A	G	G
508	rs6382744	1	109866496	C	C	C	C	A	A
509	rs6356298	1	110799560	C	C	C	C	C	C
510	rs3710646	1	110959265	C	C	C	A	C	C
511	rs3722486	1	110985352	A	A	A	G	G	G
512	rs3693142	1	111176675	G	G	G	A	A	A
513	rs6342760	1	111302057	A	A	A	A	A	A
514	mCV23695506	1	114421193	T	T	T	A	T	T
515	rs3726043	1	111780707	G	G	G	A	A	A

516	rs3657012	1	111784281	T	T	T	A	A	A
517	rs3673856	1	112097434	A	A	A	G	A	A
518	rs6242357	1	112435540	A	A	A	A	A	A
519	rs6244177	1	112767627	A	A	A	A	A/G	G
520	mCV24201027	1	115751113	G	G	A	G	A	G
521	rs3693265	1	113150455	C	C	C	G	C	G
522	rs3721888	1	113375760	A	A	A	G	G	G
523	rs3662693	1	113556351	A	A	A	C	C	A
524	rs6270144	1	113617641	G	G	G	G	G	G
525	rs3697152	1	113691025	G	G	G	A	A	A
526	rs3725808	1	114183371	G	G	A	G	A	G
527	rs3684324	1	114187805	A	A	G	A	G	A
528	rs6375239	1	114928764	A	A	A	A	A	A
529	rs3696077	1	115014679	G	G	A	G	A	G
530	mCV22804366	1	117713098	T	A	A	T	A	T
531	rs3662498	1	115218241	G	G	G	A	G	G
532	rs6346019	1	115413753	G	G	G	G	G	G
533	rs6163037	1	115836271	G	G	A	A	A	G
534	rs6323783	1	116094786	A	A	A	A	A	A
535	rs3724298	1	116291109	A	A	A	C	A	A
536	rs3725409	1	116434018	A	G	G	G	G	A
537	mCV22708763	1	119316169	A	A	A	A	A	A
538	rs3694901	1	116786547	A	T	T	A	T	A
539	rs3727162	1	116954774	A	C	C	C	C	A
540	rs6179293	1	116972222	A	G	G	G	G	A
541	rs3694226	1	117143094	A	A	A	G	A	A
542	rs6347845	1	117301398	A	G	G	G	G	A
543	rs3674655	1	117379659	C	C	C	G	C	C
544	rs6187505	1	117508758	G	G	G	G	G	G
545	rs3679459	1	118198878	G	A	A	A	A	G
546	rs6216134	1	118236415	A	A	A	G	A	A
547	rs3695581	1	118500635	G	A	A	G	A	G
548	rs6391635	1	118607078	G	G	G	G	G	G
549	rs3719973	1	118834067	A	A	A	G	A	A
550	rs3724153	1	118844172	G	G	G	A	G	G
551	rs6298343	1	118999101	G	G	G	G	G	G
552	rs6373485	1	119261214	A	A	A	G	A	G
553	rs3723088	1	119832140	G	G	G	A	G	G
554	rs3678121	1	119901621	A	A	A	G	A	A
555	rs3696498	1	120067656	G	G	G	C	G	G
556	rs6230293	1	120079867	G	G	G	A	G	G
557	rs6247820	1	120541604	A	A	A	A	A	A
558	rs3667720	1	120697988	G	G	G	C	G	C
559	rs3709675	1	120831727	G	G	G	A	G	A
560	rs3659976	1	120842643	A	A	A	A	A	A
561	mCV23664640	1	124504905	C	G	G	G	C	C
562	rs3686671	1	121712148	G	G	G	A	G	G
563	rs6163600	1	121970051	A	A	A	A	A	A
564	rs6288019	1	122600243	A	G	G	A	A	A
565	rs6161239	1	123117528	G	G	G	G	G	G
566	rs6320798	1	123126320	T	T	T	A	A	A
567	rs3691057	1	123194531	A	A	A	G	G	G
568	rs3694822	1	123642529	A	A	A	C	C	C
569	rs3088725	1	124249458	A	G	G	G	G	G
570	rs6189020	1	124433526	G	A	A	A	G	G
571	rs3698067	1	124629280	A	G	G	G	G	A
572	rs3687720	1	124828004	G	A	A	G	A	G
573	rs3697826	1	124896697	A	G	G	G	G	G
574	rs3022832	1	125100062	G	G	G	C	G	G
575	rs4137908	1	125475941	A	G	G	G	G	A
576	rs3090765	1	125695758	A	A	A	C	A	C
577	rs4222577	1	125699151	G	G	G	A	G	A
578	rs4222579	1	125699272	A	A	A	C	A	C
579	rs4222584	1	125703426	G	G	G	C	G	C
580	rs4222583	1	125703682	G	G	G	G	G	G
581	rs3716165	1	125769962	G	G	G	A	G	A
582	rs6254676	1	125856717	A	A	A	A	A	A
583	mCV23162659	1	128951433	G	G	G	G	G	G
584	rs6281562	1	126123079	G	G	G	A	G	A
585	rs6188380	1	126268669	G	G	G	A	G	A
586	rs6228473	1	126460218	A	A	A	G	A	G
587	rs3662928	1	126483684	A	A	A	G	A	G
588	rs3088540	1	126546056	A	A	A	A	A	A
589	rs3691374	1	126763355	A	A	A	G	A	A

590	rs6263067	1	126998549	G	G	G	A	G	G
591	rs3714423	1	127018944	G	G	G	A	G	G
592	rs4222589	1	127260664	A	A	A	A	A	A
593	rs6309523	1	128073171	A/G	G	G	G	G	G
594	rs6354736	1	128165659	G	A	A	A	G	G
595	rs6360388	1	128382293	A	A	A	A	A	A
596	rs8256197	1	128426093	A	A	A	G	A	A
597	rs8256196	1	128426126	A	A	A	G	A	A
598	rs8243514	1	128427104	A	A	A	A	A	A
599	rs8256174	1	128428865	A	A	A	A	A	A
600	rs3723276	1	128453766	G	G	G	A	G	G
601	mCV24753602	1	131899095	A	G	G	G	A	G
602	rs3090725	1	129216924	C	C	C	C	C	C
603	rs6379018	1	129282488	A	A	A	A	A	A
604	rs6355835	1	130487043	A	A	A	A	A	A
605	rs4222601	1	130504159	G	G	G	G	G	G
606	rs4222611	1	130613339	G	G	G	G	G	G
607	rs6257371	1	130935380	A	A	A	A	A	A
608	rs3699561	1	130962369	A	G	G	G	A	A
609	rs3724826	1	131376437	G	A	A	A	G	G
610	rs6250833	1	131532754	A	G	G	G	A	A
611	rs3718090	1	131636546	G	A	A	A	G	G
612	rs4222635	1	131854580	G	G	G	G	G	G
613	rs4222630	1	131854769	G	G	G	G	G	G
614	rs3713473	1	131948608	T	A	A	A	T	A
615	rs3678662	1	132359677	G	A	A	G	G	A
616	rs3681022	1	132451217	G	A	A	A	A	A
617	rs3713478	1	132668384	G	G	G	G	G	A
618	rs6241653	1	132978623	A	G	G	G	A	G
619	rs6331598	1	133746515	C	C	C	C	C	C
620	mCV24510697	1	137099135	T	A	A	A	T	A
621	rs4222644	1	134088014	G	G	G	G	G	G
622	rs4222650	1	134188548	G	G	G	G	G	G
623	rs4222651	1	134189626	G	G	G	G	G	G
624	rs3703729	1	134418903	G	G	G	A	G	G
625	rs6195473	1	134441421	G	G	G	G	G	G
626	rs3664806	1	134544118	C	C	C	A	C	C
627	rs6290558	1	134948045	G	G	G	A	G	G
628	rs8250053	1	135044501	T	T	T	A	T	T
629	rs8271110	1	135160893	A	A	A	G	G	G
630	rs8270851	1	135171872	A	A	A	T	A	A
631	rs4222666	1	135271276	G	G	G	A	G	G
632	rs3667307	1	135404032	G	G	G	A	G	G
633	rs8260536	1	135690070	A	C	C	C	A	C
634	rs8236489	1	135694188	A	A	A	G	A	A
635	rs8236490	1	135694634	A	A	A	A	A	A
636	rs8239512	1	135694700	G	G	G	C	G	G
637	rs3674857	1	135720939	A	G	G	G	A	G
638	rs3022842	1	135723686	G	A	A	A	G	A
639	rs6209720	1	135821029	G	G	G	G	G	G
640	rs3691222	1	136202062	G	A	A	A	G	A
641	rs6359631	1	136664129	A	A	A	A	A	A
642	mCV24712435	1	139758846	A	A	A	C	C	A
643	rs4222670	1	136809624	T	T	T	T	T	T
644	rs4222667	1	136809837	G	G	G	G	G	G
645	rs3688929	1	136944348	A	G	G	G	A	G
646	rs6250257	1	137529520	A	G	G	A	A	G
647	rs3712900	1	137775519	A	A	A	G	A	A
648	mCV24707614	1	141186686	G	C	C	C	C	C
649	rs6388989	1	138040472	A	A	A	A	A	G
650	rs6327536	1	138154871	C	A	A	C	C	C
651	rs6374344	1	139394100	T	T	T	T	T	T
652	rs3666261	1	139403008	A	G	G	A	A	G
653	rs6309356	1	140514012	G	G	G	G	G	G
654	mCV23582257	1	144412111	A	A	A	C	C	A
655	rs3726927	1	141024820	A	A	A	T	T	A
656	rs6313878	1	141395165	A	A	A	A	A	A
657	mCV23572856	1	145483327	A	G	G	A	A	G
658	rs3677844	1	142083512	A	A	A	G	G	A
659	rs6398676	1	142241603	A	A	A	A	A	A
660	rs6382880	1	142470600	T	T	T	A	A	T
661	rs3657970	1	142976384	G	A	A	G	G	A
662	rs3022841	1	143203110	G	A	A	A	A	A
663	rs6295690	1	143282073	G	G	G	G	G	G

664	rs3703202	1	143834489	A	A	A	T	T	A
665	rs3655943	1	144089161	G	A	A	G	G	A
666	rs6171515	1	144154535	T	T	T	T	T	T
667	rs6194815	1	144730521	C	C	C	A	A	C
668	rs3718645	1	144835918	A	C	C	A	A	C
669	rs3722469	1	145409234	A	G	G	G	G	G
670	rs6364156	1	146112228	G	A	A	A	G	A
671	rs6185344	1	146239381	A	C	C	C	A	C
672	rs3022838	1	146390257	G	A	A	A	G	A
673	rs3705105	1	146472985	A	G	G	G	A	G
674	rs3089732	1	146776582	G	G	G	G	G	G
675	rs3688638	1	146986258	C	A	A	C	C	A
676	rs3711440	1	147155745	A	G	G	G	A	G
677	rs6287238	1	147515993	A	A	A	A	A	A
678	rs6156126	1	148195572	C	C	C	C	C	C
679	rs3657056	1	148825962	G	G	G	A	G	G
680	rs6259479	1	148892765	A	A	A	A	A	A
681	rs6411476	1	148913234	A	G	G	G	A	A
682	rs6261978	1	149633978	C	C	C	C	C	C
683	mCV24629487	1	153740686	A	A	A	A	C	C
684	rs3654223	1	149755613	A	C	C	A	A	C
685	rs3655459	1	149755775	G	A	A	G	G	A
686	rs3680116	1	149760366	G	C	C	G	C	C
687	rs4222679	1	149802753	A	A	A	A	A	A
688	rs8238845	1	150035150	A	A	A	A	A	A
689	rs8238862	1	150036792	G	G	G	G	G	G
690	rs8238863	1	150037246	A	A	A	A	A	A
691	rs6325623	1	150619645	A	G	G	G	G	G
692	rs3676801	1	150705980	C	G	G	G	G	G
693	rs6393307	1	150967309	C	C	C	C	C	C
694	rs3688042	1	151217854	G	A	A	G	G	G
695	rs4222683	1	151325897	G	G	G	G	G	G
696	rs6263578	1	151459063	G	G	G	C	G	G
697	rs4222695	1	152338330	A	A	A	A	A	A
698	rs3688785	1	152499712	A	G	G	G	G	G
699	rs6267646	1	152516981	A	A	A	A	G	A
700	rs6207200	1	152591605	A	A	A	A	A	A
701	rs3706385	1	152595237	G	A	A	A	A	A
702	rs4222700	1	153097502	C	C	C	C	C	C
703	rs6305232	1	153201897	A	T	T	T	T	T
704	rs3674280	1	153207764	A	G	G	G	G	G
705	rs6362681	1	153446392	G	G	G	G	A	A
706	rs8242884	1	153881732	G	G	G	G	G	G
707	rs8242897	1	153883852	G	G	G	G	C	C
708	rs8242929	1	153886245	A	A	A	A	A	A
709	rs6181202	1	154592603	A	A	A	A	C	A
710	rs4222704	1	155120600	G	G	G	G	G	G
711	rs6248193	1	155445258	G	G	G	G	G	G
712	rs3709142	1	155805622	A	A	A	G	A	A
713	rs6385438	1	155837938	G	G	G	G	G	G
714	rs4222717	1	156320692	A	A	A	A	A	A
715	rs4222712	1	156320852	A	A	A	A	A	A
716	rs3677638	1	156461112	A	G	G	A	G	G
717	rs3711833	1	156645988	C	C	C	A	C	C
718	rs3721839	1	156732054	T	A	A	T	A	A
719	rs4222732	1	156807973	G	A	A	G	A	A
720	rs4222731	1	156808094	A	C	C	A	C	C
721	rs3719034	1	157158100	G	G	G	G	A	G
722	rs6384235	1	157209759	G	G	G	G	A	G
723	rs6400326	1	157366888	G	G	A	G	G	A
724	rs3726420	1	157711587	A	G	A	G	G	A
725	rs6387609	1	157898706	A	A	A	A	C	A
726	rs3696645	1	157941226	A	G	G	G	A	G
727	rs3653666	1	158553484	A	A	G	A	G	A
728	rs6318983	1	158797908	G	G	G	G	A	G
729	rs3709285	1	159043870	A	A	C	A	C	A
730	rs3701299	1	159111737	A	G	G	A	G	A
731	rs3719736	1	159200954	C	C	C	C	A	C
732	rs6297729	1	159642757	G	G	G	G	A	G
733	rs3022845	1	159766079	G	G	G	G	A	G
734	rs3701630	1	159892022	A	A	G	G	G	A
735	rs6180312	1	159901077	G	G	G	G	G	G
736	rs3674707	1	160235332	G	A	G	A	G	A
737	rs3693161	1	160294867	G	G	G	G	A	G

738	rs3662685	1	160516559	C	C	C	C	G	C
739	rs6176869	1	160845853	G	G	G	G	A	G
740	rs4135672	1	161068696	A	G	A	G	G	G
741	rs3703679	1	161182098	G	G	G	A	A	A
742	rs4135752	1	161344083	A	A	A	A	C	A
743	rs3661305	1	161529176	C	A	C	A	A	A
744	rs6225956	1	161568305	A	A	A	A	G	A
745	rs3689151	1	161850281	A	A	T	A	A	A
746	rs4222742	1	161983558	G	G	G	G	G	G
747	rs3677385	1	162154091	C	C	C	C	A	C
748	rs6297557	1	162159298	C	C	C	C	G	C
749	rs3685643	1	162261064	G	G	G	A	G	A
750	rs3686149	1	162261116	A	A	A	T	A	T
751	rs3686151	1	162261117	G	G	G	A	G	A
752	rs3669913	1	162304551	A	A	A	G	A	G
753	rs4222750	1	162614632	G	G	G	G	G	G
754	rs4222753	1	162663902	G	G	G	G	G	G
755	rs6244640	1	162908345	G	G	G	G	G	G
756	mCV27581773	1	167113019	A	A	A	A	C	A
757	rs3695661	1	163020090	G	G	G	A	G	A
758	rs3686593	1	163307274	G	G	G	A	G	A
759	rs4222763	1	163400150	G	G	G	G	A	G
760	rs4222762	1	163400188	A	A	A	A	A	A
761	mCV24468263	1	167261814	C	C	C	C	A	C
762	rs6391991	1	163872132	A	A	A	A	NONE	A
763	rs8256585	1	164081256	G	G	G	G	G	G
764	rs8256589	1	164082577	G	G	G	G	A	A
765	rs4222766	1	164305136	T	T	T	T	T	T
766	rs4222765	1	164305225	A	A	A	A	G	A
767	rs3707322	1	164408862	A	G	G	G	NONE	G
768	rs6161898	1	165255063	C	C	C	C	G	C
769	rs6159183	1	165366830	C	C	C	C	C	C
770	rs4222778	1	165501591	A	A	A	A	G	A
771	rs3090344	1	165746831	G	G	G	G	A	G
772	rs6412182	1	166301492	A	A	A	A	C	C
773	rs4222785	1	166331470	C	C	C	C	C	C
774	mCV22660057	1	171348718	A	A	A	G	A	A
775	mCV22660056	1	171349781	A	A	A	C	A	A
776	mCV22660046	1	171349782	A	A	A	T	A	A
777	mCV22660045	1	171351686	G	A	G	G	G	A
778	mCV22660044	1	171352242	A	A	A	T	A	A
779	mCV22660022	1	171352443	G	G	G	A	G	G
780	mCV22659835	1	171352984	A	A	A	G	A	A
781	rs6399765	1	167289594	G	G	G	G	A	G
782	rs8261392	1	167400785	A	A	A	A	C	A
783	rs8237094	1	167401042	G	G	G	G	G	G
784	rs8261408	1	167401511	G	G	G	G	C	G
785	rs8261478	1	167404390	A	A	A	A	G	A
786	mCV24414091	1	171225534	A	A	A	A	A	A
787	rs6224524	1	167988330	A	A	A	A	G	A
788	rs3714825	1	168244893	A	A	A	C	C	A
789	rs3676031	1	168357572	G	G	G	A	A	G
790	rs3699304	1	168438969	A	A	A	G	G	A
791	rs6404462	1	168772935	G	G	G	G	G	G
792	rs3720335	1	168923766	A	A	A	G	G	A
793	rs3657320	1	169454880	C	C	C	A	A	C
794	rs3088784	1	169857292	A	G	A	A	A	A
795	rs3712524	1	169926797	G	G	G	A	A	G
796	rs3719206	1	170111888	G	G	G	A	A	G
797	rs6347656	1	170141080	G	G	G	A	A	G
798	rs3090341	1	170490287	G	G	G	A	A	G
799	rs6353966	1	170858319	T	T	T	T	T	T
800	rs8242852	1	171128307	G	A	G	A	G	A
801	rs8237062	1	171290176	G	A	G	G	G	G
802	rs8237068	1	171291158	G	G	G	G	G	G
803	rs8258245	1	171296638	G	G	G	A	G	G
804	rs8242752	1	171308480	G	G	G	G	G	G
805	rs8238326	1	171311449	A	A	A	A	A	A
806	rs8245237	1	171317381	C	C	C	G	C	C
807	rs4222834	1	171347539	G	G	G	G	G	G
808	rs4222833	1	171347663	G	G	G	G	G	G
809	rs4222831	1	171347708	G	G	G	G	G	G
810	rs4222830	1	171347739	G	G	G	A	G	G
811	rs4222829	1	171347804	A	A	A	T	A	A

812	rs4222828	1	171347834	G	G	G	G	G	G
813	rs4222827	1	171347836	A	A	A	A	A	A
814	rs4222837	1	171413746	T	T	T	T	T	T
815	rs6286950	1	171451577	C	C	C	C	C	C
816	rs3670967	1	171607279	G	G	G	C	G	G
817	rs3089480	1	171684949	A	A	A	G	A	A
818	rs3722740	1	171820349	G	G	G	A	G	G
819	rs8259388	1	176180371	A	A	A	G	A	G
820	rs8259386	1	172394048	G	G	G	G	G	A
821	rs8259383	1	172397118	A	A	A	A	A	A
822	rs8259381	1	172397490	G	G	G	C	G	G
823	rs3707910	1	172614886	A	A	A	G	A	A
824	rs6152558	1	172697377	C	C	C	C	C	C
825	rs6154458	1	173079960	G	G	G	G	G	G
826	rs3022869	1	173087590	A	G	A	G	A	A
827	rs6412497	1	173119317	G	G	G	A	G	G
828	rs3654285	1	173119613	G	G	G	A	G	G
829	rs8242501	1	173349659	T	T	T	T	T	T
830	rs8242504	1	173349717	C	C	C	C	C	C
831	rs8245299	1	173350014	A	A	A	A	A	A
832	rs8242481	1	173351118	A	A	A	G	A	A
833	rs8245308	1	173351297	C	C	C	C	C	C
834	rs3659655	1	173541044	A	A	A	G	A	A
835	rs3682996	1	173874028	G	G	G	A	G	G
836	rs3705656	1	174280751	G	G	G	A	G	G
837	rs6220667	1	174330648	G	G	G	G	G	G
838	rs3663380	1	174576926	A	A	A	G	A	A
839	rs3669108	1	174667766	C	C	C	A	C	C
840	rs2020486	1	174729572	A	A	A	G	A	A
841	rs6375522	1	174805110	G	G	G	A	G	G
842	rs4137914	1	174920147	A	A	A	T	A	A
843	rs3658234	1	175197661	G	G	G	A	G	G
844	rs6213386	1	175461915	A	G	A	A	G	A
845	rs4136041	1	175566930	G	G	G	A	G	G
846	rs3708441	1	175788442	G	A	G	A	A	A
847	rs3700831	1	176145762	G	A	G	A	A	A
848	rs6317022	1	176246857	A	G	A	G	G	A
849	rs3723788	1	176314187	T	T	T	A	A	T
850	rs4135465	1	176357094	C	C	C	A	A	C
851	mCV23632994	1	180166490	T	T	T	A	A	A
852	rs3705103	1	176539301	C	C	C	A	A	C
853	rs3702990	1	177059830	G	G	G	A	A	G
854	rs6308631	1	177417268	G	G	G	G	G	G
855	rs8239104	1	177663886	T	T	T	T	T	T
856	mCV23640374	1	182131679	A	G	A	A	A	G
857	rs6230017	1	178206836	A	A	A	A	A	A
858	rs3090726	1	178270623	G	G	G	G	G	G
859	rs3022875	1	178270654	G	G	G	G	G	G
860	rs4222855	1	178336717	G	G	G	G	G	G
861	rs4222856	1	178336769	G	A	G	G	G	A
862	rs4222869	1	178344148	G	G	G	G	G	G
863	rs6371431	1	178949017	A	A	A	A	A	A
864	rs4222874	1	179709923	G	G	G	G	G	G
865	rs6301437	1	179841460	A	A	A	A	A	A
866	mCV23522667	6	44456212	G	A	G	G	A	G
867	mCV24589568	1	180036813	A	C	A	A	A	A
868	rs3658861	1	180232508	C	C	C	C	C	C
869	mCV23509126	1	180812801	G	A	G	G	A	A
870	rs8265812	1	181012175	G	A	G	G	G	G
871	rs8265821	1	181014279	A	G	A	A	A	A
872	rs8265824	1	181015139	A	C	A	A	A	A
873	rs4222877	1	181254659	A	A	A	A	A	A
874	rs6308816	1	181837624	G	G	G	G	G	G
875	rs6208459	1	182427090	G	A	G	G	G	G
876	rs8280291	1	182554942	A	A	A	A	A	A
877	rs8280870	1	182582402	G	A	G	G	A	A
878	rs3725956	1	183053576	T	A	T	A	T	A
879	rs6367740	1	183064619	G	A	G	A	G	A
880	mCV23022620	1	187197423	G	A	G	G	A	A
881	rs6157620	1	183197685	A	A	A	G	G	G
882	rs3692513	1	183213015	G	G	G	C	C	C
883	rs3693165	1	183213131	G	G	G	A	A	A
884	rs3708652	1	183213392	A	A	A	A	A	A
885	mCV23613178	1	187363784	A	A	A	G	G	G

886	rs6214478	1	183923032	A	A	A	A	A	G
887	rs3681873	1	184081153	A	A	A	G	A	A
888	rs4222882	1	184363490	C	C	C	C	C	C
889	rs4222878	1	184363606	C	C	C	C	C	C
890	rs6195541	1	184367705	G	G	G	G	G	G
891	mCV24555989	1	184440029	A	A	A	G	G	A
892	rs6154379	1	184815448	A	A	A	G	A	A
893	rs6312659	1	184842516	G	G	G	G	G	G
894	rs3674929	1	185025841	G	G	G	A	A	G
895	rs3678066	1	185367238	A	G	G	A	A	G
896	rs3714960	1	185576663	G	G	G	A	G	G
897	rs3714333	1	185737175	A	A	A	G	A	A
898	rs6202172	1	185986373	G	G	G	G	G	G
899	mCV24542444	1	185806972	G	A	A	A	G	A
900	mCV25202052	1	185875456	A	G	G	G	A	G
901	rs3694793	1	186568403	C	C	G	C	C	C
902	rs6249933	1	186761457	G	G	G	G	G	G
903	mCV22849619	1	186531848	G	A	G	A	G	A
904	mCV22849612	1	186532020	C	A	C	A	C	A
905	mCV23599971	1	186816908	G	A	G	A	G	A
906	mCV23599683	1	186822111	A	G	A	G	A	G
907	rs6200700	1	187354320	A	A	A	A	A	A
908	rs2228899	1	187731038	G	G	G	G	G	G
909	rs3666905	1	187691426	A	A	A	G	A	A
910	mCV23595105	1	187569693	T	A	A	A	T	A
911	rs3655423	1	188171196	G	A	A	G	A	A
912	mCV23994528	1	188019233	C	A	A	A	C	A
913	mCV23994206	1	188025144	G	A	A	A	G	A
914	mCV22540547	1	188034654	A	G	G	G	A	G
915	mCV22305130	1	188045709	A	G	G	G	A	G
916	rs3667164	1	188355284	G	G	G	C	G	G
917	mCV23993168	1	192468118	C	A	A	C	A	A
918	mCV23993161	1	192468287	G	A	A	G	A	A
919	rs4222890	1	192536231	A	A	A	A	A	A
920	rs4222892	1	188530010	G	G	G	G	G	G
921	rs3680295	1	188570862	G	A	A	A	A	A
922	rs6254961	1	188616412	C	C	C	C	C	C
923	rs3654705	1	189194896	G	G	G	G	G	C
924	rs6212571	1	189394179	A	A	A	A	A	A
925	rs3675669	1	189671727	G	G	G	A	G	G
926	rs3699009	1	189754789	A	A	A	C	A	A
927	rs6175872	1	189812827	G	G	G	G	G	G
928	mCV23587459	1	189639914	C	A	A	C	A	C
929	mCV23586427	1	189667334	G	A	A	A	A	G
930	rs3694932	1	189953741	A	C	C	A	C	C
931	mCV23205157	1	189964077	A	G	G	A	G	G
932	mCV23204820	1	189975437	C	A	A	C	A	C
933	mCV23934092	1	189990195	A	G	G	G	A	A
934	mCV23990401	1	190024384	A	A	A	G	G	A
935	mCV23990376	1	190026671	G	G	G	A	A	G
936	rs3090928	1	190426007	A	A	A	A	A	A
937	mCV24786469	1	194384980	G	A	A	A	G	A
938	rs4222905	1	190661345	G	G	G	G	G	G
939	rs4222907	1	190661509	C	C	C	C	C	C
940	rs3713460	1	190931753	G	G	G	A	G	G
941	rs6159328	1	191184223	G	G	G	G	G	G
942	rs6332864	1	191507310	G	G	G	G	G	G
943	rs4222910	1	191671729	A	A	A	A	A	A
944	rs8253327	1	191804270	A	A	A	G	A	A
945	rs3669814	1	191804564	C	C	C	A	C	C
946	mCV23575714	1	192157140	G	G	G	G	A	G
947	rs3689947	1	192397145	G	G	G	A	G	G
948	rs6186854	1	192406972	A	A	A	A	A	A
949	rs6273007	1	192791105	A	A	A	A	A	A
950	rs4222920	1	193166521	A	C	C	A	C	C
951	rs6284614	1	193859684	A	A	A	A	A	A
952	mCV23970227	1	194273895	G	A	A	G	A	G
953	rs6247319	1	194622860	A	A	A	A	A	A
954	rs8253280	1	194832307	G	G	G	G	G	G
955	rs8253291	1	194853783	G	G	G	G	G	G
956	rs6277765	1	195082858	G	G	G	A	G	G
957	mCV24145570	1	195161421	G	A	A	G	A	G
958	mCV23128319	10	1154911	A	A	G	G	G	G
959	rs3715574	10	7013075	A	A	G	G	G	G

960	rs3701657	10	6494189	A	A	G	G	G	G
961	rs6379089	10	6441727	C	C	C	C	C	C
962	rs3676474	10	5923276	A	A	G	G	G	G
963	rs6222763	10	5716417	A	A	A	A	A	A
964	rs8244291	10	5639448	G	G	G	G	G	G
965	rs6185923	10	5384467	A	A	A	G	A	A
966	mCV25374719	10	8325975	G	G	A	G	A	G
967	rs6152477	10	4664218	A	A	G	A	G	G
968	rs4228079	10	4618764	G	G	A	G	A	A
969	rs4228085	10	4555345	G	G	A	A	A	A
970	rs6274838	10	4311605	A	A	A	A	A	A
971	rs6236943	10	3853200	A	A	G	G	G	G
972	rs6264790	10	3739872	G	G	G	G	G	G
973	rs8244279	10	3560877	C	C	A	A	A	A
974	rs8244274	10	3560535	A	A	A	A	A	A
975	rs3090968	10	3497849	A	A	T	T	T	T
976	rs3721803	10	3343120	A	A	A	G	A	A
977	mCV25163613	10	6888336	A	C	C	C	A	C
978	rs3695003	10	7584259	A	A	C	A	C	C
979	rs3684570	10	7744573	A	A	A	C	A	A
980	rs4228096	10	8298850	A	A	A	A	A	A
981	rs4228098	10	8298950	A	A	G	A	G	A
982	rs6281222	10	8425147	A	A	A	A	A	A
983	rs3697929	10	8485347	G	G	A	A	A	A
984	mCV25368979	10	11529641	T	A	A	T	T	T
985	rs3680128	10	9019231	A	G	G	A	A	A
986	rs3673471	10	9079808	G	A	A	G	G	G
987	rs3664101	10	9286770	C	C	C	A	C	C
988	rs3712998	10	9902073	C	C	C	A	C	C
989	rs3088640	10	9937074	A	A	A	A	A	A
990	rs3669183	10	10149498	A	G	G	G	A	A
991	rs3685588	10	10540887	G	G	G	A	G	A
992	rs3678364	10	10792434	A	C	C	A	A	A
993	rs6296352	10	11085789	G	G	G	G	G	G
994	rs3708450	10	11175767	G	A	A	G	G	G
995	rs3656217	10	11487786	A	G	G	A	A	A
996	mCV23854739	10	14703173	A	A	A	A	C	A
997	rs6252721	10	11876375	A	A	A	A	A	A
998	rs4139678	10	12000078	G	G	G	A	G	G
999	mCV24605795	10	15115872	A	A	A	A	G	A
1000	rs6196278	10	12328078	A	G	G	A	A	A
1001	rs3697240	10	12351098	G	A	A	G	G	G
1002	rs4228112	10	12719980	A	G	G	G	A	A
1003	rs6171150	10	12765828	A	G	G	A	A	A
1004	rs3668617	10	13010915	A	A	A	G	G	A
1005	rs3693867	10	13067549	A	G	G	A	G	A
1006	rs4136728	10	13337565	A	A	A	G	A	A
1007	rs4228116	10	13358133	G	G	G	G	G	G
1008	rs4228121	10	13400769	A	A	A	A	C	A
1009	rs3653545	10	13553669	G	G	G	C	G	G
1010	rs6190586	10	13879486	G	G	G	G	A	G
1011	rs3718959	10	14114198	A	A	A	G	G	A
1012	rs4228126	10	14115727	G	G	G	A	G	G
1013	rs4228131	10	14115863	G	A	A	G	G	G
1014	rs4228132	10	14115929	A	A	A	A	A	A
1015	rs3686911	10	14116427	A	C	C	A	C	A
1016	rs3680457	10	14135322	A	G	G	A	NONE	A
1017	rs6214525	10	14447814	G	G	G	G	G	G
1018	mCV23053895	10	18114009	A	T	T	A	T	A
1019	rs6376966	10	14815556	G	G	G	G	A	G
1020	rs3673798	10	14886406	C	A	A	A	C	C
1021	rs3699409	10	15499377	A	A	A	T	A	A
1022	rs6270428	10	15682371	G	G	G	G	G	G
1023	rs6279725	10	16058353	G	G	G	G	G	G
1024	rs3666649	10	16210359	A	G	G	G	G	G
1025	rs6396621	10	16835823	G	G	G	G	G	G
1026	rs6378338	10	17168511	G	A	A	A	A	A
1027	rs4135995	10	17237149	G	G	G	A	G	A
1028	rs3677768	10	17330139	C	G	G	C	G	G
1029	rs3680292	10	17339993	A	G	G	A	G	G
1030	rs3712394	10	17639020	G	G	G	A	G	A
1031	rs3678286	10	17717136	A	A	A	G	A	G
1032	rs6163414	10	17791055	A	A	A	A	A	A
1033	rs3696055	10	17883074	G	G	G	A	G	A

1034	rs6309008	10	18193508	G	G	G	G	G	G
1035	rs4228139	10	18229624	G	A	G	A	G	G
1036	rs4228142	10	18229713	G	G	G	G	G	G
1037	rs3703211	10	18233288	A	C	C	A	C	C
1038	rs3667141	10	18639899	G	G	G	A	G	G
1039	rs6253577	10	19330321	G	G	G	G	G	G
1040	rs3688363	10	19785232	C	C	C	A	C	C
1041	rs3667453	10	19790720	G	G	G	A	G	A
1042	rs6386111	10	19824750	A	A	A	G	A	G
1043	rs3702675	10	20039819	T	T	T	A	T	A
1044	rs4228152	10	20044066	G	G	G	G	G	G
1045	rs6391056	10	20204184	G	G	G	G	G	G
1046	rs3677302	10	20816103	G	G	A	A	A	G
1047	rs6177920	10	20819664	G	G	G	G	G	G
1048	rs8256431	10	20865644	A	A	A	A	A	A
1049	rs6274648	10	21322195	A	A	T	T	T	A
1050	rs6310100	10	21519349	A	A	A	A	A	A
1051	rs3679120	10	22685808	C	A	C	A	A	C
1052	rs6163568	10	22880762	A	A	A	A	A	A
1053	rs6220110	10	23006089	G	A	G	A	A	G
1054	rs3682303	10	23040179	G	A	G	A	A	G
1055	rs3653750	10	23450805	A	G	G	A	A	G
1056	rs4136532	10	23450968	C	A	A	C	C	A
1057	rs3711723	10	24287075	G	G	G	A	A	G
1058	rs3667888	10	24292421	A	A	A	G	G	A
1059	rs6343370	10	24355784	A	A	A	C	C	A
1060	rs8254420	10	24359354	G	G	G	A	A	G
1061	rs3090587	10	24360057	G	G	G	G	G	G
1062	rs3685111	10	24397132	C	A	A	A	A	A
1063	rs6217650	10	24709794	T	T	T	A	A	T
1064	rs3663890	10	24785010	A	A	A	G	G	A
1065	rs3659676	10	25195765	G	A	A	G	G	A
1066	rs3722105	10	25287297	A	G	G	G	G	G
1067	rs3706825	10	25296563	G	A	A	G	G	A
1068	rs3672342	10	25455146	A	G	G	A	A	G
1069	rs3689005	10	25559360	C	C	C	A	A	C
1070	mCV23525682	10	28693966	T	T	A	T	A	A
1071	rs4228165	10	26052089	G	G	G	G	G	G
1072	rs3663497	10	26223114	G	G	G	A	A	G
1073	rs6221681	10	26242259	A	A	A	G	G	A
1074	rs3666315	10	26321240	G	G	G	C	C	G
1075	rs3661395	10	26771628	G	G	G	A	A	G
1076	rs3719045	10	26884697	G	G	G	A	A	G
1077	rs3705722	10	26903750	A	A	A	C	C	A
1078	rs6396283	10	26952321	G	G	G	A	A	G
1079	rs6167054	10	27866193	C	C	C	C	C	C
1080	rs6391751	10	28239358	C	C	A	C	A	A
1081	rs3023233	10	28385223	G	G	A	G	A	A
1082	rs3089169	10	28686593	G	G	G	G	G	G
1083	rs3704618	10	28826382	C	C	G	C	G	G
1084	rs6244647	10	29010991	A	A	A	A	A	A
1085	rs6226323	10	29948399	C	C	C	C	C	C
1086	rs3088921	10	29970701	G	G	G	G	G	G
1087	rs6281141	10	30186916	A	A	A	A	A	A
1088	rs8244315	10	31207228	A	A	A	A	A	A
1089	rs8244312	10	31207652	A	A	A	A	A	A
1090	rs6280091	10	31482690	G	G	G	G	G	G
1091	rs6283464	10	31903532	G	G	G	G	G	G
1092	rs6311865	10	33621010	A	A	A	A	A	A
1093	rs4228184	10	33981727	A	A	A	A	A	A
1094	rs6364780	10	34162397	C	C	C	C	C	C
1095	rs8244301	10	34225247	A	A	A	A	A	A
1096	rs3703268	10	34347375	G	G	G	G	G	G
1097	rs6227503	10	35980741	C	C	C	C	C	C
1098	rs6329545	10	36677885	A	A	A	A	A	A
1099	rs4228185	10	36951591	A	A	A	A	A	A
1100	rs6396204	10	37447663	A	A	A	A	A	A
1101	mCV25441323	10	41094472	A	G	G	G	G	G
1102	rs6208129	10	38074094	G	G	G	G	G	G
1103	rs6312880	10	38853590	G	G	G	G	G	G
1104	rs6310683	10	39526423	A	A	A	A	A	A
1105	rs6163277	10	40248528	C	A	A	A	A	A
1106	rs3709849	10	40826428	G	A	A	A	A	A
1107	rs6238898	10	40932769	T	T	T	T	T	T

1108	rs3700514	10	40943657	A	G	G	G	G	G
1109	rs6243429	10	41385841	A	A	A	A	A	A
1110	rs4228204	10	41429529	T	T	T	T	T	T
1111	rs3699726	10	41693458	A	T	T	T	T	T
1112	rs6285294	10	42070390	A	G	G	G	G	G
1113	rs3716113	10	42609040	G	A	A	A	A	A
1114	rs3722104	10	43067872	A	G	G	G	G	G
1115	rs6230282	10	43172623	A	A	A	A	A	A
1116	rs3686482	10	43402131	A	G	G	G	G	G
1117	rs8256464	10	43490934	G	G	G	G	G	G
1118	rs8244391	10	43491801	A	A	A	A	A	A
1119	rs3706577	10	44291326	T	A	A	A	A	A
1120	rs6196044	10	44566044	G	A	A	A	A	A
1121	rs3655258	10	44892712	A	G	G	G	G	G
1122	rs3661754	10	44896565	G	A	A	A	A	A
1123	rs6391464	10	44944101	G	A	A	A	A	A
1124	rs4228211	10	45243264	A	A	A	A	A	A
1125	rs3090642	10	45065470	G	G	G	G	G	G
1126	rs6339088	10	46298011	A	A	A	A	A	A
1127	rs6247222	10	46473107	A	A	A	A	A	A
1128	rs3677589	10	46828606	G	G	A	G	A	G
1129	rs3676667	10	46923056	G	G	A	G	G	G
1130	rs3679201	10	46923454	A	A	G	A	A	A
1131	rs6335794	10	47100636	G	G	G	G	G	G
1132	rs6325627	10	47829404	T	T	T	T	T	T
1133	rs3715820	10	48556301	G	G	G	G	G	G
1134	rs6382436	10	48683209	G	A	A	A	A	A
1135	rs3720313	10	49150536	C	C	C	C	C	C
1136	rs6409301	10	49150917	G	G	G	G	G	G
1137	rs6298808	10	49216525	A	A	A	A	A	A
1138	rs8244397	10	49248597	G	G	G	G	G	G
1139	rs6187868	10	49387553	C	C	C	C	C	C
1140	rs3687807	10	49629081	A	A	A	A	A	A
1141	rs6205658	10	50401888	G	G	G	G	G	G
1142	rs8244360	10	50929592	A	A	A	A	A	A
1143	rs6200562	10	50934137	A	A	A	A	A	A
1144	rs3656829	10	50960906	G	G	G	G	G	G
1145	rs8244379	10	51005835	A	A	A	A	A	A
1146	rs6272974	10	52434829	G	G	G	G	G	G
1147	rs8244413	10	52572819	G	G	G	G	G	G
1148	rs6270574	10	52933590	A	A	A	A	A	A
1149	rs6375660	10	53444830	G	G	G	G	G	G
1150	rs3696307	10	53783742	G	G	G	C	G	G
1151	rs6242097	10	54833239	C	C	C	C	C	C
1152	rs6190748	10	55832573	A	A	A	A	A	A
1153	rs8244463	10	56418021	A	A	A	A	A	A
1154	rs8260768	10	56429407	G	G	G	G	G	G
1155	rs8237361	10	56430563	A	A	A	A	A	A
1156	rs6315852	10	56613283	A	A	A	A	A	A
1157	rs3682060	10	56651845	A	A	G	A	A	A
1158	rs3676909	10	56657626	G	G	A	G	G	G
1159	rs3089794	10	56684509	A	A	G	A	A	A
1160	rs3090942	10	57844647	A	A	A	A	A	A
1161	rs6231019	10	57860320	C	C	C	C	C	C
1162	rs4228213	10	57867835	G	G	G	G	G	G
1163	rs4228214	10	57867907	A	A	A	A	A	A
1164	rs6378491	10	58875807	G	G	G	G	G	G
1165	mCV24754478	10	63349704	G	G	G	G	G	G
1166	rs8244704	10	60052653	A	A	A	A	A	A
1167	rs4228216	10	60054367	A	A	A	A	A	A
1168	rs6388774	10	60103839	G	G	G	G	G	G
1169	rs3694974	10	60398058	G	G	G	G	G	G
1170	rs6374078	10	60860895	G	G	G	G	G	G
1171	rs4228221	10	60937390	G	G	G	G	G	G
1172	rs8242303	10	61056038	A	A	A	A	A	A
1173	rs8244736	10	61061975	G	G	G	G	G	G
1174	rs8244738	10	61062324	G	G	G	G	G	G
1175	rs8244640	10	61628370	A	A	A	A	A	A
1176	rs8244562	10	61654983	A	A	A	A	A	A
1177	rs6362278	10	61686016	T	T	T	T	T	T
1178	rs8244504	10	61743733	G	G	G	G	G	G
1179	rs6355901	10	62215934	A	A	A	A	A	A
1180	mCV22766787	10	66337376	A	A	A	G	A	A
1181	rs4228228	10	62573082	G	G	G	G	G	G

1182	rs6248261	10	62727142	A	A	A	A	A	A
1183	rs6299108	10	64789686	A	A	A	A	A	A
1184	rs3089067	10	65502482	G	G	G	G	G	G
1185	rs6172306	10	65522957	G	G	G	G	G	G
1186	rs6263039	10	66214853	C	C	C	C	C	C
1187	rs3725117	10	66179612	C	C	A	C	C	C
1188	rs3723140	10	66179274	G	G	A	G	G	G
1189	rs6301985	10	66984495	G	G	G	G	G	G
1190	rs4228240	10	67314856	A	A	A	A	A	A
1191	rs8244660	10	67316100	G	G	G	G	G	G
1192	rs3712063	10	67865783	A	A	A	G	A	A
1193	rs3680724	10	67917504	G	G	G	A	G	G
1194	rs3683193	10	67966008	A	A	A	G	G	A
1195	rs6302539	10	68351666	A	A	A	A	A	A
1196	rs4228247	10	68552569	G	G	G	G	G	G
1197	mCV25067862	10	72556969	G	G	G	A	G	G
1198	rs3683124	10	68961987	A	G	G	G	G	G
1199	rs3679735	10	69153889	A	T	A	T	T	T
1200	rs3690226	10	69158406	A	G	A	G	G	G
1201	rs2228923	10	69184527	G	G	G	G	G	G
1202	rs6326263	10	69534393	A	G	A	G	G	G
1203	rs3654108	10	70274895	G	G	A	G	G	G
1204	rs3090776	10	70289786	A	A	A	A	A	A
1205	rs3089848	10	70398077	G	G	G	G	G	G
1206	rs6333189	10	70761476	C	C	C	C	C	C
1207	rs3681069	10	70884356	A	A	G	A	A	A
1208	rs6238136	10	69808403	C	C	C	C	C	C
1209	rs3682523	10	69818779	A	A	G	A	A	A
1210	rs6387158	10	71883373	G	G	G	G	G	G
1211	rs6226553	10	73413045	A	A	A	A	A	A
1212	mCV23043653	10	77742753	A	A	A	A	A	A
1213	rs6301951	10	73987366	A	A	A	A	A	A
1214	rs6409642	10	74891087	C	C	C	C	C	C
1215	mCV24228019	10	79395750	G	G	G	G	G	G
1216	rs6293552	10	75427567	G	G	G	G	G	G
1217	rs8274234	10	75475900	A	A	A	A	A	A
1218	rs8274276	10	75481250	A	A	A	A	A	A
1219	rs4228270	10	76580369	A	A/G	A	A	A	A
1220	rs8236826	10	76926620	C	C	C	C	C	C
1221	rs8244926	10	76945378	T	T	T	T	T	T
1222	rs8244889	10	76949655	A	A	A	A	A	A
1223	rs4228279	10	77153313	A	A	A	A	A	A
1224	rs6400510	10	77164416	A	A	A	A	A	A
1225	rs4228285	10	77435327	A	A	A	A	A	A
1226	rs4228297	10	77606724	A	A	A	A	A	A
1227	rs6241143	10	78243840	G	G	G	G	G	G
1228	rs6315627	10	79039142	G	G	G	G	G	G
1229	rs4228310	10	79572648	G	G	G	G	G	G
1230	rs8243626	10	79601979	G	G	G	G	G	G
1231	rs6294842	10	79604551	A	A	A	A	A	A
1232	rs8243632	10	79606378	A	A	A	A	A	A
1233	rs8236829	10	79787139	A	A	A	A	A	A
1234	rs4228319	10	79847759	A	A	A	A	A	A
1235	rs6216433	10	79857056	A	A	A	A	A	A
1236	rs8236831	10	80711622	A	A	A	A	A	A
1237	rs8236832	10	80713709	G	G	G	G	G	G
1238	rs6347110	10	81249166	A	A	A	A	A	A
1239	rs8236836	10	81261519	G	G	G	G	G	G
1240	rs8236837	10	81261763	A	A	A	A	A	A
1241	rs3654332	10	81991031	A	A	A	G	A	A
1242	rs3656551	10	81994180	A	A	A	G	A	A
1243	rs6249559	10	82021489	G	G	G	A	G	G
1244	rs3667120	10	82327650	G	G	G	A	G	G
1245	rs3721306	10	82360108	G	G	G	A	G	G
1246	rs3717445	10	82649121	G	G	G	C	G	G
1247	rs6299415	10	82686321	T	T	T	A	T	T
1248	mCV24379439	10	86322095	G	A	A	G	G	G
1249	rs4228354	10	83257735	G	G	G	G	G	G
1250	rs6221047	10	83311101	C	C	C	C	C	C
1251	rs3661402	10	84477700	A	A	A	A	A	A
1252	rs6358480	10	84508765	A	A	A	A	A	A
1253	rs6351708	10	84586416	A	T	T	A	T	T
1254	rs4228360	10	84799276	A	A	A	A	A	A
1255	rs3689549	10	84885312	G	A	A	G	A	A

1256	rs3691945	10	84885647	T	A	A	T	A	A
1257	rs3679902	10	85007769	G	A	A	G	G	A
1258	rs8258322	10	86033270	G	G	G	G	G	A
1259	rs8258352	10	86034550	G	G	G	G	G	G
1260	rs8258353	10	86034834	G	A	A	G	G	G
1261	rs6278961	10	86074333	A	G	G	A	A	G
1262	rs8236848	10	86080254	A	A	A	A	A	A
1263	rs8258304	10	89052392	G	G	G	G	G	A
1264	rs8258305	10	86454468	C	C	C	C	C	C
1265	rs8258306	10	86454516	G	G	G	G	G	G
1266	rs8258307	10	86454760	G	G	G	G	G	G
1267	mCV25373751	10	90475134	A	G	G	A	G	G
1268	rs6394370	10	87251221	A	G	G	A	A	G
1269	rs3089109	10	90111304	C	C	C	C	C	G
1270	rs6222436	10	87905079	A	G	G	A	G	G
1271	rs4228380	10	88423292	G	G	G	G	G	G
1272	rs4228381	10	88423409	A	G	G	A	G	G
1273	rs3090760	10	88866278	A	A	A	A	A	A
1274	rs3090759	10	88866283	G	A	G	G	G	A
1275	rs3701829	10	89091027	A	G	G	A	A	G
1276	rs6182528	10	89109985	G	A	A	G	G	A
1277	rs4228386	10	89213890	A	A	A	A	A	A
1278	rs6221944	10	89413413	A	A	A	A	C	A
1279	rs3089366	10	89439033	G	A	G	G	G	A
1280	rs3089313	10	89536824	A	A	A	A	G	A
1281	rs3678268	10	89809517	A	A	G	A	G	A
1282	rs3712657	10	90682863	G	A	A	G	A	A
1283	rs4228402	10	91437167	G	G	G	G	G	G
1284	rs4228403	10	91437324	A	A	A	A	A	A
1285	rs6295508	10	91665955	A	A	A	A	A	A
1286	rs6191721	10	91687354	A	A	A	G	A	A
1287	rs6367212	10	91860467	G	G	G	A	G	G
1288	rs6267580	10	91881484	G	G	G	A	G	G
1289	rs3704031	10	91965927	G	G	G	A	G	G
1290	rs3669765	10	92140506	A	A	A	T	A	A
1291	rs3661495	10	92425957	A	A	A	G	A	A
1292	rs6397304	10	92744319	A	A	A	G	A	A
1293	rs6331511	10	93028993	G	G	G	A	G	G
1294	rs3663375	10	93088669	G	G	G	C	G	G
1295	rs3090164	10	93223964	G	G	G	G	G	G
1296	rs6212903	10	93270577	G	G	G	A	G	G
1297	rs6233970	10	93629017	C	C	C	A	C	C
1298	rs3686681	10	93822765	A	A	A	T	A	A
1299	rs3664737	10	93866897	A	A	A	G	A	A
1300	rs3704460	10	93868807	A	A	A	G	A	A
1301	rs8258420	10	96525941	A	A	A	A	A	A
1302	rs8258418	10	93919462	G	G	G	G	G	G
1303	rs8258411	10	93919739	G	G	G	G	G	G
1304	rs8258435	10	93920696	G	G	G	A	G	G
1305	rs6337832	10	94513865	C	C	C	C	C	C
1306	rs6389778	10	95081795	G	G	G	G	G	G
1307	rs6388405	10	95877749	A	A	A	A	A	A
1308	rs6362637	10	96986620	T	T	T	T	T	T
1309	rs8259805	10	97390170	A	A	A	A	A	A
1310	mCV24985767	10	100942617	C	G	C	G	G	C
1311	rs6191187	10	97532365	A	A	A	A	A	A
1312	rs3089906	10	98028624	G	G	G	G	G	G
1313	rs2020560	10	98529546	T	T	T	T	T	T
1314	rs6324242	10	98738223	G	G	G	G	G	G
1315	rs6339227	10	98955216	G	G	G	G	G	G
1316	rs3704401	10	99023683	T	T	A	T	T	T
1317	rs3683912	10	99061427	G	G	A	G	G	G
1318	rs4228447	10	99231575	A	A	A	A	A	A
1319	rs6383172	10	99761430	A	A	A	A	A	A
1320	rs8258500	10	99793385	A	A	C	A	A	C
1321	rs6185093	10	100449156	G	G	A	G	G	A
1322	mCV25004810	10	104983124	G	A	G	G	A	G
1323	rs6349133	10	101854815	A	A	A	A	A	A
1324	mCV24205612	10	105707311	G	G	G	A	G	A
1325	rs6226142	10	102475171	A	A	A	C	A	A
1326	rs6382566	10	102496550	G	G	G	A	G	G
1327	rs3694232	10	102506199	A	A	A	G	A	A
1328	rs3694833	10	102506283	G	G	G	A	G	G
1329	rs3711434	10	102581113	C	C	C	A	C	A

1330	mCV24206699	10	106695924	A	A	A	T	A	A
1331	rs6246096	10	103269765	A	A	A	G	A	A
1332	rs6326791	10	103514385	G	A	G	G	A	G
1333	rs6336700	10	103818862	A	C	A	A	C	A
1334	rs3688351	10	104194765	A	G	A	G	G	A
1335	rs3716899	10	104644893	C	A	C	A	A	A
1336	rs6196597	10	104650349	G	G	G	A	G	A
1337	rs3699498	10	104945236	C	C	A	C	C	C
1338	rs4137343	10	105109550	A	T	A	A	T	A
1339	rs3716443	10	105187761	A	G	A	A	G	A
1340	rs6283474	10	105256777	C	A	C	C	A	C
1341	rs3691939	10	105878552	G	G	G	A	G	G
1342	rs6284148	10	106510239	G	G	G	G	G	A
1343	rs3089137	10	106565030	A	A	A	A	A	A
1344	mCV24654052	10	110149064	C	C	C	C	A	C
1345	rs3654717	10	106878678	A	A	A	G	A	G
1346	rs8258523	10	107167195	A	A	A	A	A	A
1347	rs8258512	10	107168257	A	A	A	A	A	A
1348	rs6333891	10	107462809	C	C	C	A	C	C
1349	rs3655255	10	107536865	G	G	G	A	G	G
1350	rs3697161	10	107582863	A	A	A	G	A	A
1351	rs6290359	10	107672808	C	C	C	A	C	C
1352	rs6221535	10	107689532	C	C	C	G	C	C
1353	rs3714942	10	107966095	A	A	A	T	A	A
1354	rs3725618	10	108203078	G	G	G	A	G	G
1355	rs3659212	10	108207109	G	G	G	A	G	G
1356	rs6176884	10	109430801	T	T	T	T	T	T
1357	rs3090205	10	110214740	A	A	A	A	A	A
1358	rs6353445	10	111476107	A	A	A	A	G	A
1359	rs6230659	10	111949585	G	G	G	G	G	G
1360	rs3088653	10	112074533	A	A	A	A	G	A
1361	rs6336042	10	113270971	G	G	G	G	G	G
1362	rs8248732	10	113524924	C	C	C	C	C	C
1363	rs8248730	10	113524992	G	G	G	G	G	G
1364	mCV24217147	10	117613736	A	T	T	A	T	A
1365	rs3680872	10	114181187	A	G	A	G	A	A
1366	rs8275446	10	114221912	G	G	G	A	G	G
1367	rs8275482	10	114275667	G	A	G	A/G	G	G
1368	rs3698227	10	114278041	G	A	G	A	G	G
1369	rs3690259	10	114332741	G	A	G	A	G	G
1370	rs8275440	10	114343165	A	A	A	A	A	A
1371	rs3702621	10	114594032	C	A	A	A	A	A
1372	rs6201222	10	114650095	A	A	T	T	T	T
1373	rs3706484	10	114888970	A	A	G	G	A	G
1374	rs4228488	10	115159632	G	G	G	G	G	G
1375	rs4228484	10	115159793	A	A	A	A	A	A
1376	mCV25264026	10	118669410	A	C	A	A	C	A
1377	mCV25429984	10	119011153	A	A	A	T	A	A
1378	rs4228490	10	115836940	G	G	G	G	G	G
1379	rs4228492	10	115837144	G	G	G	G	G	G
1380	rs6386163	10	115923164	G	G	G	G	G	G
1381	rs6293295	10	116552545	G	G	G	G	G	G
1382	rs3670118	10	117190262	G	G	G	A	G	A
1383	rs6256918	10	117348939	A	G	A	G	G	G
1384	rs6284081	10	117927784	A	C	A	A	C	A
1385	rs3707488	10	117972011	C	C	C	A	C	A
1386	rs3657221	10	118091133	G	A	G	A	A	A
1387	rs6360342	10	118727864	G	C	G	G	C	C
1388	rs3661470	10	118877769	G	A	G	G	A	A
1389	rs6317716	10	119164417	A	G	A	A	G	G
1390	rs3659070	10	119755023	A	G	A	A	G	G
1391	rs3659572	10	119755089	G	A	G	G	A	A
1392	rs4228504	10	119772340	A	A	A	A	A	A
1393	rs6197175	10	120390339	A	G	A	A	G	A
1394	mCV24888322	10	123695257	T	A	A	T	T	A
1395	rs6237927	10	121114917	G	C	G	G	C	C
1396	rs4228517	10	124561203	C	C	C	C	C	C
1397	rs6197961	10	121604090	A	G	A	A	A	A
1398	rs3673336	10	121760044	C	C	C	C	C	C
1399	rs3687863	10	121963259	A	A	G	G	G	G
1400	rs3707479	10	122097853	G	G	A	A	A	A
1401	rs6243232	10	122430808	A	A	A	A	A	A
1402	mCV22832306	10	126086653	G	A	A	G	A	G
1403	rs3719419	10	122558849	A	A	G	G	G	G

1404	rs3700457	10	123100878	A	G	A	A	A	A
1405	rs6282839	10	123395705	G	C	C	G	C	C
1406	mCV22735181	10	127834096	A	A	A	A	G	G
1407	mCV23897761	10	127985283	C	C	C	A	A	C
1408	rs3690963	10	123937385	G	G	A	G	A	A
1409	rs3658341	10	124265798	A	G	G	A	A	G
1410	rs6315152	10	124423988	G	A	A	G	G	A
1411	rs3667606	10	124582635	C	C	G	C	C	G
1412	rs4140243	10	124619881	A	G	G	A	A	G
1413	rs6227461	10	124638302	A	G	G	A	A	G
1414	rs6281696	10	125373094	A	G	G	A	G	A
1415	rs6322698	10	126355329	G	G	G	G	G	G
1416	rs6211774	10	126454201	G	G	G	G	G	G
1417	rs3697243	10	126634957	G	C	G	C	C	G
1418	rs3680343	10	126709623	A	G	A	G	G	A
1419	rs3718732	10	127276330	T	T	T	T	T	T
1420	rs8259806	10	127481352	G	A	A	G	G	A
1421	rs8243604	10	127482124	G	G	G	G	G	G
1422	rs3676616	10	127579076	G	A	A	G	G	A
1423	rs4228532	10	128255634	C	C	C	C	C	C
1424	rs3676330	10	128337313	A	A	A	T	T	A
1425	rs4228534	10	128337576	A	A	A	A	A	A
1426	rs6171719	10	128562635	A	A	A	T	T	A
1427	rs3719526	10	128579965	G	G	G	A	A	G
1428	rs8274570	10	128685267	C	C	C	C	C	C
1429	rs6317670	10	129535451	G	G	G	G	G	G
1430	rs3653715	10	129573768	G	G	C	G	G	G
1431	rs3701568	10	129611809	A	A	G	A	A	A
1432	rs6394321	10	130060895	A	A	A	A	A	A
1433	rs4228735	11	704	A	A	A	A	A	A
1434	rs4228736	11	740	A	A	A	A	A	A
1435	rs3721534	11	1792	G	G	G	A	G	A
1436	rs6295404	11	112171619	G	A	A	A	A	G
1437	rs6202743	11	3937845	A	A	A	A	A	A
1438	rs3090318	11	4110196	A	A	A	A	A	A
1439	rs6237739	11	4165808	A	G	A	A	A	A
1440	rs3659787	11	4403518	A	A	A	G	A	A
1441	rs3682937	11	4575120	G	A	G	G	G	G
1442	mCV22909481	11	7280769	T	A	T	T	T	T
1443	rs6173098	11	5328061	A	A	G	G	G	A
1444	rs6253982	11	5424672	C	A	A	A	A	A
1445	rs4223042	11	5529213	G	G	G	G	G	G
1446	rs4223045	11	5529304	A	A	A	A	A	A
1447	rs8236862	11	5764061	A	A	A	A	A	A
1448	rs4228567	11	5765046	G	G	G	G	G	G
1449	rs4228568	11	5765109	G	G	G	G	G	G
1450	rs4228569	11	5765245	A	A	A	A	A	A
1451	rs6393401	11	5765890	G	G	G	G	G	G
1452	rs3670360	11	6148459	A	A	G	A	A	A
1453	rs6159177	11	6311153	A	G	G	A	A	A
1454	rs3714263	11	6483553	G	A	A	G	G	G
1455	rs3698320	11	6856309	A	A	C	C	C	C
1456	rs3673327	11	7360830	G	A	A	A	A	A
1457	rs3691311	11	7400206	A	G	A	G	G	A
1458	rs6327459	11	7733213	G	G	G	G	G	G
1459	rs3660658	11	8158387	A	T	T	A	A	A
1460	mCV24434350	11	10733105	G	G	G	A	G	G
1461	rs3088673	11	8396987	A	A	C	A	A	A
1462	rs3089123	11	8523547	G	G	G	G	G	G
1463	rs6290313	11	8580129	A	G	A	A	A	A
1464	rs3714397	11	8731519	G	G	G	A	G	A
1465	rs6230380	11	8949828	G	G	G	A	G	G
1466	rs6351798	11	8969129	G	A	A	G	G	G
1467	rs3661631	11	9229338	G	A	A	G	G	A
1468	rs6406927	11	9315119	G	G	G	G	G	G
1469	rs6166904	11	9579688	A	C	C	A	A	A
1470	rs6335368	11	9674792	A	G	G	A	A	A
1471	rs3680429	11	9723224	T	A	A	T	T	T
1472	rs3688266	11	9897084	A	A	A	G	A	A
1473	rs3714537	11	9967121	A	A	A	G	A	A
1474	rs3695907	11	10603483	A	A	A	G	A	A
1475	mCV23851630	11	13163316	A	G	A	A	A	A
1476	rs3023249	11	11065936	C	C	C	A	C	C
1477	rs3023250	11	11065963	A	A	A	G	A	A

1478	rs3726213	11	11071710	A	A	A	G	A	A
1479	rs3685735	11	11386048	C	A	A	C	C	C
1480	mCV24455206	11	14141745	C	A	A	C	C	A
1481	rs3659698	11	11764924	A	G	G	G	A	G
1482	rs4228582	11	11825266	G	G	G	G	G	G
1483	rs6185261	11	11906527	G	A	A	G	G	A
1484	rs3674916	11	11967887	A	A	A	G	A	G
1485	mCV22963182	11	14514503	G	G	G	A	G	G
1486	rs3718522	11	12187272	A	A	G	G	A	G
1487	rs3663826	11	12188090	A	G	G	A	A	A
1488	rs6397172	11	12242841	T	T	A	A	T	A
1489	rs3656716	11	12765831	T	A	A	T	T	T
1490	rs6250760	11	12897492	A	A	G	G	A	G
1491	rs3723497	11	12912745	A	A	G	G	A	G
1492	rs3705394	11	13459831	G	A	A	G	G	A
1493	rs3673722	11	13569212	G	A	A	A	G	A
1494	mCV23871121	11	16324361	A	G	A	A	A	A
1495	rs3713053	11	13825318	G	A	A	G	G	A
1496	rs6158396	11	13985767	A	G	G	G	A	G
1497	rs3687754	11	14262520	C	C	A	C	C	C
1498	rs6314339	11	14305562	A	G	G	A	A	A
1499	rs3684585	11	14455318	T	T	T	A	T	T
1500	rs3724116	11	14607512	C	C	A	A	C	A
1501	rs3703198	11	14898098	A	A	A	G	A	G
1502	rs6213490	11	14917267	A	A	A	G	A	G
1503	rs6313050	11	15647995	A	G	A	A	A	A
1504	rs6276419	11	16145150	G	G	G	G	G	G
1505	rs3657739	11	16502281	C	A	C	C	C	C
1506	rs3667506	11	16759040	A	G	G	A	A	A
1507	rs4228583	11	16770739	A	A	A	A	A	A
1508	rs4228584	11	16770799	G	G	G	G	G	G
1509	rs3091140	11	16807675	G	G	G	G	G	G
1510	mCV25346958	11	12575016	G	A	A	G	G	A
1511	rs6279977	11	16898338	C	G	G	C	G	C
1512	rs4228590	11	17099154	G	G	G	G	G	G
1513	rs3676204	11	17173224	A	G	G	A	G	A
1514	rs3705582	11	17498921	G	G	G	A	G	G
1515	rs3700313	11	17577159	C	C	C	A	C	C
1516	rs3679497	11	17632170	G	G	G	A	G	G
1517	rs3679597	11	17632214	A	A	A	G	A	A
1518	rs6223549	11	17844448	T	T	T	A	T	T
1519	rs3716790	11	17999950	A	A	A	G	A	A
1520	rs3674910	11	18063225	G	G	G	A	G	G
1521	rs6292290	11	18478343	G	G	G	A	G	G
1522	rs3693118	11	18659854	G	A	G	A	G	G
1523	rs3720687	11	18877207	A	G	G	A	G	G
1524	rs6313528	11	18940584	G	A	G	A	G	G
1525	rs3090214	11	19069935	C	C	C	C	C	C
1526	rs3723987	11	19364495	C	C	C	A	C	A
1527	rs6199297	11	19407330	A	A	A	A	A	A
1528	rs3711924	11	19430896	G	G	G	C	G	C
1529	rs3658216	11	19458677	G	G	G	A	G	A
1530	rs3678321	11	19522724	T	A	T	T	T	T
1531	rs3707185	11	19875244	G	A	G	A	G	A
1532	rs4228593	11	20134354	G	A	G	G	G	G
1533	rs6220017	11	20203551	G	G	G	G	G	G
1534	rs8248101	11	20204192	G	A	G	G	G	G
1535	rs8248143	11	20208302	G	G	G	G	G	G
1536	rs8241442	11	20209030	A	A	A	A	A	A
1537	rs8241443	11	20209122	C	C	C	C	C	C
1538	rs8248200	11	20214159	C	C	C	C	C	C
1539	rs8241436	11	20221803	G	G	G	G	G	A
1540	rs8241679	11	20227150	G	G	G	G	G	G
1541	rs3715577	11	20485062	G	A	G	G	G	A
1542	rs6393028	11	20749377	A	A	A	A	A	A
1543	rs3720127	11	20845026	G	A	G	A	G	A
1544	rs3023251	11	20848871	G	A	G	A	G	A
1545	rs3090219	11	21324911	A	A	A	A	A	A
1546	mCV25386480	11	22094321	G	G	G	G	G	G
1547	rs3679664	11	21534637	A	A	A	A	A	A
1548	rs4135796	11	21651535	C	G	C	G	C	C
1549	rs3683150	11	21741637	G	A	A	A	A	G
1550	rs3708339	11	21752934	A	G	A	G	A	A
1551	rs6204872	11	21901288	A	A	A	A	A	A

1552	rs3661074	11	22648047	A	A	G	A	G	G
1553	rs4228612	11	22654812	G	G	G	G	G	G
1554	rs4228622	11	22732501	C	C	A	C	A	A
1555	rs4228626	11	22901192	A	A	A	A	A	A
1556	rs6376327	11	23662154	C	C	C	C	C	C
1557	rs3667046	11	24114875	A	A	A	A	A	A
1558	rs3677232	11	24568317	G	G	G	A	G	G
1559	rs6398304	11	24648645	A	G	G	A	G	A
1560	rs3726603	11	24737772	A	G	G	A	G	A
1561	rs3699245	11	24821753	A	A	A	A	A	A
1562	rs3697361	11	25077085	A	C	C	A	C	A
1563	rs3718784	11	25182715	G	A	A	G	A	G
1564	rs3727156	11	25186897	G	A	A	G	A	G
1565	mCV23508321	11	26181653	A	G	G	A	G	A
1566	rs6238406	11	25465051	G	A	A	G	A	G
1567	rs6367881	11	26054403	C	A	A	C	A	C
1568	rs4228638	11	26285280	A	A	A	A	A	A
1569	rs3719024	11	26366413	G	A	A	G	A	A
1570	rs3723990	11	26517914	G	A	A	A	A	A
1571	rs3090752	11	26724827	G	A	A	G	A	A
1572	rs3717048	11	26861268	C	G	G	C	G	C
1573	rs6330945	11	26890859	A	G	G	A	G	A
1574	rs3674800	11	27016835	G	A	A	G	A	G
1575	rs6266683	11	27402569	A	G	G	A	G	A
1576	rs3703024	11	27424886	T	A	A	T	A	T
1577	rs3706539	11	27480537	G	A	A	G	A	G
1578	rs3718803	11	28187587	A	C	C	A	C	C
1579	rs3706694	11	28310427	A	G	G	A	G	A
1580	rs6390270	11	28714278	G	A	A	G	A	A
1581	rs6177292	11	29026174	C	A	A	C	A	C
1582	rs4228640	11	29114495	G	G	G	G	G	G
1583	rs3720425	11	29125437	A	A	A	A	A	G
1584	rs3656724	11	29418060	A	T	T	A	T	A
1585	rs3659581	11	29615516	A	G	G	A	G	G
1586	rs3723433	11	29666093	A	G	G	A	G	A
1587	rs6412884	11	30001764	G	G	A	G	G	G
1588	rs3700830	11	30266839	G	A	G	G	A	A
1589	rs3656706	11	30499626	G	A	G	G	A	A
1590	rs6168508	11	30709328	A	G	A	A	G	G
1591	mCV23785265	11	32131213	A	G	A	A	G	A
1592	rs6376887	11	31757184	G	G	A	G	G	A
1593	rs3707274	11	31817191	G	G	G	G	G	G
1594	rs3656589	11	31916577	A	A	G	A	A	G
1595	rs3703534	11	32006575	A	A	G	A	A	G
1596	rs3705963	11	32036495	A	A	G	A	A	G
1597	rs3677196	11	32323270	C	C	A	C	C	C
1598	rs6205294	11	32390549	A	A	T	A	A	A
1599	rs3726288	11	32659564	G	A	G	G	A	A
1600	rs3657760	11	32856509	A	G	G	A	G	G
1601	rs6297874	11	32886695	A	A	A	A	A	G
1602	rs3723833	11	32946888	A	A	G	A	A	G
1603	rs4228663	11	33525841	A	A	A	A	A	A
1604	rs4228664	11	33525962	G	G	G	G	G	G
1605	rs3710499	11	33606699	C	C	C	C	C	C
1606	rs6201941	11	33863971	A	A	A	A	A	A
1607	rs6328092	11	34678802	A	A	A	A	A	A
1608	mCV24813109	11	27134128	G	C	C	G	C	G
1609	rs3673465	11	35174599	G	A	A	G	A	G
1610	rs3664950	11	35853924	A	G	G	A	G	G
1611	rs3674958	11	35994926	A	C	C	A	C	C
1612	rs3704943	11	36212035	C	G	G	C	G	G
1613	rs3720767	11	36226910	A	G	G	A	G	G
1614	rs6303903	11	36230987	A	G	G	A	G	G
1615	rs3724175	11	36317429	A	A/G	G	A	G	G
1616	rs3662388	11	36554065	A	A	A	G	G	A
1617	rs3690160	11	36695995	C	C	C	A	C	C
1618	mCV24159035	11	35575829	A	G	G	A	G	A
1619	rs4139111	11	37081663	A	A	A	G	A	A
1620	rs3724155	11	37138788	G	G	G	A	G	G
1621	rs6406223	11	37451001	A	A	A	A	C	A
1622	rs3677530	11	37827385	A	A	A	C	C	A
1623	rs4139734	11	38151752	A	A	A	C	A	C
1624	rs6359329	11	38177953	A	A	A	A	G	G
1625	rs3696538	11	38488380	C	C	C	A	A	A

1626	rs6325706	11	38589606	G	G	G	G	A	G
1627	rs6319607	11	38673500	A	A	A	T	T	T
1628	rs6189512	11	38875994	A	A	A	G	G	G
1629	rs3692365	11	39510070	G	G	G	A	A	A
1630	rs3709054	11	39724700	A	A	A	G	G	G
1631	rs6164170	11	39929979	G	G	G	A	A	A
1632	rs3665719	11	39947166	A	A	A	G	G	G
1633	rs6317915	11	40052999	G	G	G	A	A	A
1634	rs3701734	11	40967464	G	G	G	A	A	A
1635	rs6267987	11	40996551	A	A	A	G	G	G
1636	rs4228665	11	41552034	T	T	T	T	T	T
1637	rs3692893	11	42011558	A	A	A	G	G	G
1638	rs3693664	11	42011736	C	C	C	A	C	A
1639	rs6307831	11	42458111	G	G	G	A	G	G
1640	rs3718475	11	42619598	A	A	A	G	G	G
1641	rs3706713	11	42670361	A	A	A	C	NONE	A
1642	rs3023336	11	43060091	G	G	G	G	A	A
1643	rs3023335	11	43060186	C	C	C	C	A	A
1644	rs3023327	11	43064921	A	A	A	A	C	C
1645	rs3023325	11	43065107	G	G	G	G	A	A
1646	rs3023324	11	43065157	G	G	G	G	A	A
1647	rs3023322	11	43065253	A	A	A	A	T	T
1648	rs3023317	11	43066477	A	A	A	A	G	G
1649	rs4228679	11	43360926	C	C	C	C	C	C
1650	rs6329109	11	43372552	G	G	G	A	G	G
1651	rs3676349	11	43696438	G	G	G	A	A	A
1652	rs3676315	11	43728382	A	A	A	G	G	G
1653	rs3654344	11	43847855	C	C	C	A	A	C
1654	rs3706346	11	44030023	A	A	A	C	C	C
1655	rs3090802	11	44187083	T	T	T	T	T	T
1656	rs6291843	11	44490256	A	A	A	A	A	A
1657	rs3688183	11	44745786	A	A	A	G	A	A
1658	mCV22950866	11	43061790	G	G	G	G	A	A
1659	rs2020823	11	45533876	A	A	A	A	A	G
1660	rs6318723	11	45562564	A	A	A	G	G	A
1661	rs3716545	11	45563889	G	G	G	A	A	A
1662	rs3717281	11	45564020	G	G	G	A	A	A
1663	rs3660692	11	45855756	G	G	G	A	G	G
1664	rs6274326	11	45887629	G	G	G	G	G	G
1665	rs6184392	11	46406842	A	A	A	G	G	G
1666	rs3697000	11	46540346	A	A	A	G	G	G
1667	rs4138873	11	46828694	G	G	G	A	A	G
1668	rs6412660	11	47007845	G	G	G	G	G	G
1669	rs6276177	11	47396531	C	C	C	C	C	C
1670	rs3090966	11	48505435	T	T	T	T	T	T
1671	rs6167130	11	48796514	G	G	G	G	G	G
1672	rs6399552	11	49276937	A	A	A	A	A	A
1673	rs4228692	11	49314906	G	G	G	G	G	G
1674	rs3692257	11	49356721	A	A	A	A	A	A
1675	rs3089612	11	49669452	C	C	C	C	C	C
1676	rs6332908	11	49719764	C	C	C	C	C	C
1677	rs4228697	11	49841526	A	A	A	A	A	A
1678	mCV24271437	11	48574138	G	G	G	G	G	G
1679	rs4228698	11	50710438	C	C	C	C	C	C
1680	rs4228707	11	51296927	C	C	C	C	C	C
1681	rs8243055	11	51893791	G	G	G	G	A	G
1682	rs8241535	11	51895629	G	G	G	G	G	G
1683	rs8243077	11	51896154	T	T	T	T	A	T
1684	rs8243006	11	51921275	G	G	G	G	G	G
1685	rs8243015	11	51921839	C	C	C	C	A	C
1686	rs8243022	11	51924320	G	G	G	G	G	G
1687	rs8243026	11	51924485	A	A	A	A	A	A
1688	rs3023259	11	52218692	C	C	C	C	G	C
1689	rs6327224	11	52724565	G	G	G	G	G	G
1690	rs8264668	11	53523429	G	G	G	G	G	G
1691	rs6358010	11	53585635	A	A	A	A	G	A
1692	mCV23125083	11	51667194	A	A	A	A	T	A
1693	rs4228726	11	53630655	T	T	T	T	T	T
1694	rs8260618	11	53631556	A	A	A	A	G	A
1695	rs8264464	11	53644196	A	A	A	A	G	A
1696	rs8264368	11	53644511	A	A	A	A	A	A
1697	rs8264468	11	53644899	A	A	A	A	A	A
1698	rs6399660	11	53706293	A	A	A	A	A	A
1699	rs3023258	11	53814546	C	C	C	A	A	C

1700	rs3698063	11	53961524	G	G	G	A	A	G
1701	rs6153538	11	54001495	A	A	A	A	A	A
1702	rs4228727	11	54549573	A	A	A	A	A	A
1703	rs6394459	11	54834391	A	A	A	A	A	A
1704	rs4228731	11	55034293	G	G	G	A	G	G
1705	rs3668680	11	55712245	G	G	G	A	G	G
1706	rs3684076	11	56050650	A	A	A	G	G	A
1707	rs3088940	11	56155810	G	G	G	A	G	A
1708	rs3715495	11	56670853	G	G	G	G	G	G
1709	rs3088624	11	56684378	A	A	A	A	A	A
1710	rs6318831	11	56794312	A	A	A	A	A	A
1711	rs3685511	11	56805628	A	G	G	G	G	G
1712	rs3023260	11	56887064	G	G	G	A	G	G
1713	rs3715214	11	57136832	C	C	C	A	C	C
1714	rs3702614	11	57139882	G	G	G	A	G	G
1715	rs6152357	11	57261319	G	G	G	G	G	G
1716	rs3023265	11	57373142	A	A	A	G	A	A
1717	rs3023263	11	57441854	G	G	G	A	G	G
1718	rs6371333	11	57744962	C	C	C	A	C	C
1719	rs4228762	11	57820097	A	A	A	A	A	A
1720	rs6193359	11	57832610	G	G	G	G	G	G
1721	rs3714321	11	58031080	G	G	G	A	G	G
1722	rs3697686	11	58180077	A	A	A	G	A	A
1723	rs6313602	11	58618992	A	A	A	G	A	A
1724	rs3683066	11	58783592	A	A	A	G	A	A
1725	rs3657266	11	58841882	A	A	A	G	A	A
1726	rs3088586	11	59311929	G	G	G	G	G	G
1727	rs6268685	11	59399517	G	G	G	G	G	G
1728	rs4228772	11	59583116	C	G	G	C	C	C
1729	rs4228773	11	59583186	G	A	A	G	G	G
1730	rs4228790	11	60363569	G	A	A	G	G	G
1731	rs4228797	11	60401474	G	G	G	G	G	G
1732	rs3702556	11	60611577	A	G	G	A	A	A
1733	rs6310839	11	60682012	A	G	G	A	A	G
1734	rs6253450	11	60862703	A	A	A	A	A	A
1735	rs8239366	11	60870993	C	C	C	C	C	C
1736	rs8239354	11	60878971	T	T	T	T	T	T
1737	rs8239353	11	60879365	C	C	C	C	C	C
1738	rs3711357	11	61065104	G	A	A	A	A	G
1739	rs4228814	11	61878652	T	T	T	T	T	T
1740	rs4228813	11	61878829	G	G	G	G	G	G
1741	rs3675335	11	62091257	C	A	A	A	A	A
1742	rs6263230	11	62830121	A	G	G	A	G	A
1743	rs3673863	11	62955168	A	G	G	A	A	A
1744	rs16322	11	63624180	C	C	C	C	C	C
1745	rs6361209	11	63640650	A	A	A	A	A	A
1746	mCV23637656	11	59766042	G	A	A	G	G	G
1747	rs3713702	11	63883819	A	G	G	A	A	G
1748	rs3714311	11	63883927	A	C	C	A	A	C
1749	rs3686912	11	63939094	C	G	G	C	C	G
1750	mCV23561340	11	59949810	G	A	A	G	G	G
1751	rs3656583	11	64241956	C	G	G	C	C	C
1752	rs6283243	11	64270998	A	G	G	A	A	A
1753	rs3722712	11	64276704	G	A	A	G	G	G
1754	rs3695202	11	64314334	A	G	G	G	A	A
1755	rs3698199	11	65087082	G	A	A	A	G	G
1756	rs3088814	11	65165941	G	G	G	G	G	G
1757	rs6361961	11	65577244	G	G	G	G	A	A
1758	rs3663524	11	65862473	A	G	G	G	A	A
1759	rs6350709	11	65869373	G	A	A	A	G	G
1760	rs3714979	11	65888171	A	G	G	G	A	A
1761	mCV24123456	11	61864452	A	G	G	G	A	A
1762	rs6299418	11	66784499	G	G	G	A	G	G
1763	mCV23975681	11	62517192	A	A	A	G	A	A
1764	rs3691200	11	66889647	A	A	A	G	A	A
1765	rs3088872	11	66933016	A	A	A	A	A	A
1766	rs3715924	11	66991466	G	G	A	G	A	A
1767	rs3718573	11	67150983	G	G	A	G	A	A
1768	rs3697101	11	67375207	C	C	C	A	C	A
1769	rs6262977	11	67504964	A	A	G	G	G	A
1770	rs3090216	11	67669167	A	A	A	A	A	A
1771	rs3677986	11	67760094	G	G	C	G	C	G
1772	rs3666734	11	67763306	A	A	A	A	A	A
1773	rs3672689	11	68546955	A	G	G	A	G	A

1774	rs8239186	11	68702490	G	A	A	A	A	A
1775	rs3658906	11	68721622	G	A	G	A	G	A
1776	rs3698009	11	68830792	A	G	G	G	G	G
1777	rs3696966	11	68940454	G	G	A	A	G	G
1778	rs4228833	11	68954128	C	C	C	C	C	C
1779	rs3668244	11	69360019	G	G	G	A	G	G
1780	rs3023277	11	69701020	A	A	A	A	G	A
1781	mCV23044839	11	65492498	A	A	A	A	G	G
1782	rs6380524	11	70295829	A	A	A	A	G	A
1783	rs4228834	11	70301319	C	C	C	C	C	C
1784	rs3694522	11	70319319	G	A	G	A	G	G
1785	rs4228842	11	70385221	C	C	C	C	C	C
1786	rs3726991	11	70414751	G	A	G	A	G	A
1787	rs3705078	11	70486760	A	G	A	G	A	G
1788	rs6166031	11	70517955	G	A	G	A	G	A
1789	rs3142826	11	70707486	C	A	C	A	C	C
1790	rs3142842	11	70709284	G	A	G	A	A	G
1791	rs3148189	11	70725501	A	C	A	C	A	A
1792	rs3142817	11	70737362	A	G	A	G	G	A
1793	rs6211374	11	71300721	A	A	A	A	NONE	A
1794	rs4228847	11	71660350	A	A	A	A	A	A
1795	rs3695424	11	71949477	A	C	C	C	A	C
1796	rs4228854	11	72063466	A	A	A	A	G	A
1797	rs3724115	11	72373089	T	A	A	A	A/T	A
1798	rs4228857	11	72627243	C	C	C	C	C	C
1799	rs6335028	11	72724428	G	A	G	A	G	G
1800	rs3701609	11	72876179	G	G	G	A	A	A
1801	rs6353059	11	74159536	T	T	T	A	T	A
1802	rs3663256	11	74284851	G	A	G	A	G	A
1803	rs8239493	11	74289451	G	G	G	G	G	G
1804	rs3023303	11	74320921	A	G	A	A/G	A	G
1805	rs3672001	11	74409366	C	G	C	G	C	G
1806	mCV23528728	11	69438756	A	A	A	G	G	G
1807	rs4228871	11	74441050	G	G	G	G	G	G
1808	rs3679340	11	74834125	A	G	A	G	A	G
1809	rs4228875	11	75022533	A	A	A	A	A	A
1810	rs3091201	11	75044553	A	A	A	A	A	A
1811	rs3705751	11	75319403	G	G	G	A	G	A
1812	rs3700578	11	75818131	G	G	G	A	G	G
1813	rs3715356	11	75849191	G	G	G	A	G	G
1814	rs3700353	11	75967538	A	A	A	G	A	A
1815	rs6339901	11	75968112	G	G	G	A	G	G
1816	mCV24113391	11	71092401	G	G	G	G	A	G
1817	rs6366450	11	76089305	T	T	T	A	T	T
1818	rs8241150	11	76559188	A	A	A	A	A	A
1819	rs3662476	11	76560106	A	A	A	G	A	A
1820	rs8241101	11	76560705	A	A	A	C	A	A
1821	rs8241202	11	76581130	G	G	G	A	G	G
1822	rs8247972	11	76581781	G	G	G	G	G	G
1823	rs6371313	11	77300977	G	G	G	G	G	G
1824	rs4228923	11	78114515	G	G	G	G	G	G
1825	rs6173406	11	78184916	G	G	G	G	G	G
1826	rs3091022	11	78628505	G	G	G	G	G	G
1827	rs6401371	11	79062509	G	G	A	G	G	G
1828	rs6379880	11	79407138	A	A	A	A	A	G
1829	rs6201655	11	79456676	A	A	A	A	A	G
1830	mCV22728592	11	75211813	G	G	G	A	G	A
1831	rs3688710	11	80283227	A	A	A	A	A	A
1832	rs3689225	11	80283282	A	A	A	A	A	A
1833	rs6382522	11	80726274	G	G	G	G	G	G
1834	rs6155988	11	81422813	A	A	A	A	A	A
1835	rs3088627	11	81675502	C	C	C	C	C	C
1836	mCV23024199	11	77253543	G	A	G	G	G	G
1837	rs4228934	11	82378874	A	A	A	A	A	A
1838	rs4228935	11	82378912	C	C	C	C	C	C
1839	rs3717361	11	82476096	G	G	G	A	A	A
1840	rs3723910	11	82479992	A	A	A	G	G	G
1841	mCV23968112	11	77684333	G	G	G	G	G	G
1842	rs4228937	11	82792113	G	G	G	G	G	G
1843	rs3702415	11	82905686	G	G	G	A	A	A
1844	rs3689955	11	82927791	A	A	A	T	T	T
1845	rs4228948	11	83507289	A	A	A	A	A	A
1846	rs3658433	11	83649923	G	G	G	A	A	A
1847	rs3089860	11	83784707	G	G	G	G	G	G

1848	rs6229186	11	83995960	G	G	G	A	G	A
1849	rs3686654	11	84143774	A	A	A	T	T	T
1850	rs3661657	11	84334033	G	G	G	A	A	A
1851	mCV22974307	11	87646244	A	A	A	G	A	A
1852	rs6365608	11	85052523	G	G	G	G	C	G
1853	rs3090221	11	85185238	G	G	G	G	A	G
1854	rs3724750	11	85271382	C	C	C	G	C	G
1855	rs3712644	11	85533504	G	G	G	C	G	C
1856	rs4228962	11	85813935	C	C	C	C	C	C
1857	rs4228961	11	85814048	A	A	A	A	A	A
1858	rs3655537	11	86362842	A	A	A	C	A	C
1859	rs3657136	11	86412819	A	A	A	G	A	G
1860	rs3719581	11	86560546	A	A	A	G	A	G
1861	rs4228970	11	86820819	G	G	G	G	G	G
1862	rs3720112	11	87456245	G	G	G	A	G	G
1863	rs6192689	11	87481870	A	A	A	G	A	A
1864	mCV22974275	11	87648649	T	T	T	A	T	T
1865	rs4136072	11	87702483	G	G	G	A	G	G
1866	rs3666396	11	87835970	A	A	A	C	A	A
1867	rs3677121	11	87877969	G	G	G	A	G	G
1868	rs3659504	11	88332743	G	G	G	A	G	G
1869	rs8270514	11	88596053	C	C	C	A	C	C
1870	rs6370920	11	88787797	A	A	A	G	A	A
1871	rs3687072	11	88920437	G	G	G	A	G	G
1872	rs3676661	11	88952583	A	A	A	C	A	A
1873	rs3697441	11	89211848	A	A	A	G	A	A
1874	rs6376709	11	89498391	G	G	G	A	G	G
1875	rs3653766	11	89571691	G	G	G	A	G	G
1876	rs3714867	11	89749152	A	A	A	G	A	G
1877	rs3722590	11	89778531	G	G	G	A	G	G
1878	rs3688955	11	90207658	G	G	G	A	G	A
1879	rs6408734	11	90340391	A	A	A	A	A	A
1880	rs6277263	11	90968105	C	C	C	C	C	C
1881	rs6344690	11	91620697	C	C	C	C	C	C
1882	rs6335262	11	92185522	A	G	G	G	G	G
1883	rs3714299	11	92734680	G	A	A	A	A	G
1884	rs3682457	11	93184699	A	A	A	G	G	G
1885	rs3656982	11	93326874	T	A	A	T	T	T
1886	mCV24677428	11	86295961	A	A	A	G	A	G
1887	mCV24677429	11	86296048	G	G	G	A	G	A
1888	mCV24677430	11	86296107	G	G	G	A	G	A
1889	mCV24677440	11	86296571	G	G	G	A	G	A
1890	mCV24677441	11	86296728	G	G	G	A	G	A
1891	mCV24677442	11	86298897	G	G	G	A	G	A
1892	mCV24677452	11	86299367	G	G	G	A	G	A
1893	mCV24677453	11	86299818	A	A	A	G	A	G
1894	mCV24677454	11	86299876	G	G	G	A	G	A
1895	mCV24677464	11	86300191	T	T	T	A	T	A
1896	mCV24677465	11	86300214	NONE	A	A	C	A	C
1897	mCV24677466	11	86300575	A	A	A	G	A	G
1898	mCV24677476	11	86301027	G	G	G	A	G	A
1899	mCV24677761	11	86307311	G	G	G	A	G	A
1900	mCV24677762	11	86308545	A	A	A	G	A	G
1901	mCV24677763	11	86308681	G	G	G	A	G	A
1902	mCV24677773	11	86309336	G	G	G	A	G	A
1903	mCV24677774	11	86309469	G	G	G	A	G	A
1904	mCV24677775	11	86309540	A	A	A	G	A	G
1905	mCV24677797	11	86310909	G	G	G	A	G	A
1906	mCV24677799	11	86310994	A	A	A	C	A	C
1907	mCV24677809	11	86311335	G	G	G	A	G	A
1908	mCV24677810	11	86311420	A	A	A	G	A	G
1909	mCV24677811	11	86311593	G	G	G	A	G	A
1910	mCV24677822	11	86311878	A	A	A	C	A	C
1911	mCV24678093	11	86311991	A	A	A	T	A	T
1912	mCV24678094	11	86312021	A	A	A	G	A	G
1913	mCV24678095	11	86312118	C	C	C	A	C	A
1914	mCV24678105	11	86312160	G	G	G	A	G	A
1915	mCV24678106	11	86312258	C	C	C	A	C	A
1916	mCV24678107	11	86312642	G	G	G	A	G	A
1917	mCV24678118	11	86313165	A	A	A	T	A	T
1918	mCV24678119	11	86313357	A	A	A	C	A	C
1919	mCV24678129	11	86313752	A	A	A	G	A	G
1920	mCV24678130	11	86315199	G	G	G	A	G	A
1921	mCV24678131	11	86315412	C	C	C	A	C	A

1922	mCV24678141	11	86315613	A	A	A	T	A	T
1923	mCV24678142	11	86315821	A	A	A	G	A	G
1924	mCV24678167	11	86316133	A	A	A	T	A	T
1925	mCV24678439	11	86316486	G	G	G	A	G	A
1926	mCV24678449	11	86316599	G	G	G	A	G	A
1927	mCV24678450	11	86316628	G	G	G	A	G	A
1928	mCV24678462	11	86318120	A	A	A	T	A	T
1929	mCV24678473	11	86318188	G	G	G	A	G	A
1930	mCV24678474	11	86318221	A	A	A	G	A	G
1931	mCV24678475	11	86318505	A	A	A	G	A	G
1932	mCV24678484	11	86318545	A	A	A	C	A	C
1933	mCV24678485	11	86318677	A	A	A	T	A	T
1934	mCV24678486	11	86318800	T	T	T	A	T	A
1935	mCV24678496	11	86318996	G	G	G	A	G	A
1936	mCV24678520	11	86320086	A	A	A	G	A	G
1937	mCV24678521	11	86320418	A	A	A	G	A	G
1938	mCV24678522	11	86320554	A	A	A	G	A	G
1939	mCV24678795	11	86321117	A	A	A	C	A	C
1940	mCV24678804	11	86321474	G	G	G	A	G	A
1941	mCV24678805	11	86321770	G	G	G	A	G	A
1942	mCV24678816	11	86322314	G	G	G	G	G	G
1943	mCV24678817	11	93337739	G	G	G	A	G	A
1944	mCV24678828	11	86322889	C	C	C	A	C	A
1945	mCV24678829	11	86325017	A	A	A	G	A	G
1946	mCV24678840	11	86325161	A	A	A	G	A	G
1947	mCV24678841	11	86325203	A	A	A	G	A	G
1948	mCV24678842	11	86326062	C	C	C	G	C	G
1949	mCV24678852	11	86326113	G	G	G	A	G	A
1950	mCV24678853	11	86326269	G	G	G	A	G	A
1951	mCV24678864	11	86326460	A	A	A	G	A	G
1952	mCV24678865	11	86326688	C	C	C	A	C	A
1953	rs6316145	11	93432418	G	A	A	G	G	G
1954	mCV24678866	11	86328403	A	A	A	T	A	T
1955	mCV24679137	11	86328547	G	G	G	G	G	G
1956	mCV24679139	11	86329515	A	A	A	A	A	A
1957	mCV24679150	11	86331271	A	A	A	G	A	G
1958	mCV24679151	11	86331562	A	A	A	G	A	G
1959	mCV24679162	11	86331664	A	A	A	C	A	C
1960	mCV24679163	11	86331801	C	C	C	A	C	A
1961	mCV24679164	11	86331802	C	C	C	C	C	C
1962	rs6378431	11	93453785	G	A	A	G	G	G
1963	rs3023312	11	93969968	G	A	A	G	G	G
1964	rs3088501	11	94001578	G	A	A	A	A	G
1965	rs6209951	11	94071646	T	A	A	T	T	T
1966	rs4228987	11	94237500	G	G	G	G	G	G
1967	rs3671548	11	94237576	C	A	A	C	C	C
1968	rs4228988	11	94237615	G	G	G	G	G	G
1969	rs3716598	11	94273810	G	G	G	A	A	G
1970	rs3711235	11	94607639	T	A	A	T	T	T
1971	rs4229003	11	94696205	G	A	A	G	G	G
1972	rs4229012	11	94939964	G	A	A	G	G	G
1973	rs4229020	11	94940198	A	A	A	A	A	A
1974	rs3090050	11	95024664	G	A	A	G	G	G
1975	rs6238153	11	95025231	A	G	G	A	A	A
1976	rs3684399	11	95395305	G	A	A	G	G	G
1977	rs3684436	11	95395327	G	A	A	G	G	G
1978	rs4229023	11	95516309	C	C	C	C	G	C
1979	rs6247550	11	95719913	A	G	G	A	A	A
1980	rs3708716	11	95769909	G	A	A	G	G	G
1981	rs3091065	11	95961280	A	A	A	A	A	A
1982	rs4229033	11	95977357	G	G	G	G	G	G
1983	rs3710148	11	96196554	A	A	G	A	G	A
1984	rs6298456	11	96212076	G	C	C	G	C	G
1985	rs3708840	11	96632720	A	A	A	G	A	G
1986	rs8270318	11	96877104	G	G	G	G	G	G
1987	rs8270290	11	96883020	A	G	A	A	A	G
1988	rs8270303	11	96884884	A	G	A	A	A	G
1989	rs8270256	11	96886865	G	A	G	G	G	A
1990	rs8236884	11	96911765	G	A	G	G	G	A
1991	rs8270199	11	96912774	A	G	A	A	A	G
1992	rs8236885	11	96916153	A	C	A	A	A	C
1993	rs8270183	11	96929404	G	G	G	G	G	G
1994	mCV22876119	11	90086233	G	G	G	A	G	A
1995	rs6169425	11	97437210	A	G	A	A	A	G

1996	rs6236574	11	97780069	G	A	G	A	G	G
1997	rs3682334	11	97799411	A	G	A	G	A	A
1998	rs3686162	11	97814884	A	T	A	T	A	A
1999	rs4229050	11	98028564	C	C	C	C	C	C
2000	rs4229059	11	98477100	A	A	A	A	A	A
2001	rs4229063	11	98477252	C	C	C	C	C	C
2002	rs4229073	11	98581928	A	A	A	A	A	A
2003	rs3661058	11	98590564	G	A	G	A	G	G
2004	rs3700197	11	98670622	G	A	G	A	G	G
2005	rs4229084	11	98737306	G	G	G	G	G	G
2006	rs6255749	11	98740111	A	A	A	A	A	A
2007	rs3023315	11	99179949	G	G	G	C	G	G
2008	rs4229088	11	99251260	G	NONE	G	A	G	G
2009	rs17537	11	99401164	A	A	A	A	A	A
2010	rs3090212	11	99721952	A	G	A	A	A	A
2011	rs8243562	11	99875155	G	G	G	G	G	G
2012	rs8243536	11	99919048	G	G	G	G	G	G
2013	rs6272953	11	100060831	A	G	A	A	A	A
2014	rs4229090	11	100128925	G	G	G	G	G	G
2015	rs6212976	11	100345749	G	G	G	G	G	G
2016	rs3090971	11	100548067	A	A	A	A	A	A
2017	rs3695865	11	101350115	G	G	G	A	G	A
2018	rs3722448	11	101523337	A	G	A	G	A	G
2019	rs3091185	11	101549493	A	A	A	A	A	A
2020	rs6163209	11	101629465	A	T	A	A	A	A
2021	rs3653651	11	101817813	A	G	A	G	A	G
2022	rs4229101	11	101820035	A	G	A	G	A	G
2023	rs8265648	11	102021171	G	G	G	G	G	G
2024	rs8265649	11	102022466	A	A	A	A	A	A
2025	rs8265669	11	102024409	G	G	G	G	G	G
2026	rs6255041	11	102024867	G	G	G	G	G	G
2027	rs6269080	11	102025203	A	A	A	A	A	A
2028	rs8265673	11	102027703	G	A	G	G	G	G
2029	rs8265690	11	102031425	A	G	A	A	A	A
2030	rs8265701	11	102032051	G	A	G	G	G	G
2031	rs4229109	11	102124390	A	A	A	A	A	A
2032	rs4137882	11	102249410	G	C	G	C	G	C
2033	rs3685813	11	102465591	C	G	C	G	C	G
2034	rs3709793	11	102581561	A	A	A	G	A	G
2035	rs6180460	11	102625210	G	G	G	G	G	G
2036	rs4229111	11	102643325	A	T	A	A	A	A
2037	rs6333045	11	103179106	A	G	A	A	A	G
2038	rs6393948	11	103272243	A	G	A	A	A	G
2039	rs3690511	11	103388619	A	G	A	G	A	G
2040	rs4229114	11	103609008	G	G	G	G	G	G
2041	rs3723163	11	103650773	A	A	A	G	A	G
2042	rs3680754	11	103682192	C	C	C	A	C	C
2043	rs3708643	11	103798635	A	G	A	A	A	G
2044	rs3722158	11	104069759	A	C	A	C	A	C
2045	rs3721088	11	104175231	A	G	A	G	A	G
2046	rs4139506	11	104188708	A	G	A	G	A	A
2047	rs3658778	11	104699568	A	C	A	C	A	A
2048	rs3698446	11	104908478	G	A	G	A	G	G
2049	rs3699664	11	104908698	A	A	A	A	A	A
2050	rs4229123	11	105072820	A	A	A	A	A	A
2051	rs4229124	11	105072879	C	C	C	C	C	C
2052	rs6358392	11	105203763	A	A	A	A	A	A
2053	rs3724864	11	105496926	A	A	A	G	A	A
2054	rs3686516	11	105715539	A	A	A	C	A	A
2055	rs6192754	11	105891591	G	G	G	G	G	G
2056	rs4229126	11	106173676	A	A	A	A	A	A
2057	rs4229128	11	106173946	G	G	G	G	G	G
2058	rs4229153	11	106489617	A	A	A	A	A	A
2059	rs4229158	11	106489802	A	A	A	A	A	A
2060	mCV25445390	11	97297201	G	A	G	G	G	A
2061	rs3705163	11	106563339	A	A	A	C	A	A
2062	rs6386362	11	106649550	G	G	G	A	G	G
2063	rs3700560	11	107304711	G	A	A	A	A	A
2064	rs3089281	11	107374537	G	G	G	G	G	G
2065	rs3704454	11	107445540	A	G	G	A	G	G
2066	rs3670642	11	107589920	G	A	A	G	A	A
2067	rs3669823	11	107635521	G	G	G	A	G	G
2068	rs3720981	11	108308030	A	A	A	G	A	A
2069	rs6152151	11	108327864	G	G	G	G	G	G

2070	rs3707743	11	108560503	A	A	A	G	A	A
2071	mCV25379202	11	99112042	G	A	G	A	G	G
2072	rs3683086	11	109004052	A	A	A	G	A	A
2073	rs6370458	11	109065922	A	A	A	G	A	A
2074	rs4229173	11	109343954	G	G	G	G	G	G
2075	rs4229174	11	109344088	A	A	A	A	A	A
2076	mCV22971719	11	100922003	A	A	A	G	A	G
2077	rs3690064	11	109901626	G	G	A	A	A	G
2078	rs3661749	11	109913912	A	A	G	G	G	A
2079	rs6305694	11	109914476	G	G	A	A	A	G
2080	rs4229189	11	110699182	A	A	A	A	A	G
2081	rs3659410	11	111150576	A	A	G	G	G	G
2082	rs6335078	11	111412690	A	A	G	G	G	G
2083	rs3672597	11	111905327	G	G	G	A	A	G
2084	rs3658195	11	111966481	G	G	G	A	A	A
2085	rs3687980	11	112026795	G	A	A	A	A	G
2086	rs3682143	11	112397417	C	A	A	C	C	C
2087	rs3697369	11	112397544	G	A	A	G	G	G
2088	rs6280443	11	112660932	T	A	A	T	T	T
2089	rs6407460	11	113060943	G	G	G	A	A	G
2090	rs3699056	11	113674817	G	G	G	A	A	G
2091	rs3693846	11	113680576	T	T	T	A	A	T
2092	rs3708239	11	114313230	G	A	A	A	A	G
2093	rs4135850	11	114347919	G	A	A	G	A	G
2094	rs6354903	11	114451457	C	C	C	C	A	A
2095	rs6349487	11	114567190	A	G	G	A	A	A
2096	rs3712667	11	114830840	A	G	G	A	A	A
2097	rs3662930	11	115193172	G	A	A	A	G	A
2098	rs6364987	11	115720616	A	G	G	A	A	A
2099	rs3707957	11	115782928	A	G	G	A	A	A
2100	rs3707906	11	115805492	C	G	G	C	G	C
2101	rs3688691	11	116300204	G	G	G	A	G	G
2102	rs6230748	11	116341699	G	G	G	A	A	G
2103	rs3703110	11	116355859	G	G	G	A	A	G
2104	rs6271461	11	117263112	A	A	A	A	A	A
2105	rs3661724	11	117356846	A	A	A	G	A	G
2106	rs3678982	11	117532598	A	A	A	C	A	C
2107	rs3721943	11	118015080	A	A	A	G	A	A
2108	rs3697076	11	118049242	C	C	C	A	C	C
2109	rs6377381	11	118322709	T	T	T	T	T	T
2110	rs6331863	11	118577592	G	G	G	G	G	G
2111	rs8243512	11	119214035	A	A	A	A	A	A
2112	rs8243507	11	119215074	A	A	G	A	G	G
2113	rs8243495	11	119219809	A	A	G	A	G	G
2114	rs4229215	11	119281012	C	C	G	C	G	G
2115	rs4229217	11	119281160	A	A	A	A	A	A
2116	rs6346368	11	119706533	G	G	A	G	A	G
2117	rs6192404	11	119836113	A	A	G	A	G	G
2118	rs4229221	11	120632796	G	G	G	G	G	G
2119	rs3712881	11	120738678	G	G	G	G	G	A
2120	rs6322554	11	120835832	C	C	C	C	C	C
2121	rs3712388	11	120918323	G	A	A	A	A	A
2122	rs6192174	11	120959942	A	G	G	G	G	G
2123	rs3693796	11	121485409	G	A	A	A	A	G
2124	mCV22625013	11	114220237	G	A	A	G	G	G
2125	rs6198851	12	3686182	G	A	A	A	A	A
2126	rs6369108	12	3753145	G	G	G	G	G	G
2127	rs4229224	12	3825365	C	C	C	C	C	C
2128	rs3699421	12	3922406	G	G	A	G	G	G
2129	rs3699525	12	3922447	A	A	C	A	A	A
2130	rs6246656	12	4009306	A	A	G	A	A	A
2131	rs4229230	12	4588718	G	G	G	G	G	G
2132	rs4229232	12	4588848	A	A	A	A	A	A
2133	rs4229234	12	4935717	A	A	A	A	A	A
2134	rs3653990	12	5131626	G	G	A	G	G	G
2135	rs6308052	12	5186221	A	A	A	A	A	A
2136	rs3676815	12	5280179	A	G	G	A	A	A
2137	rs4229239	12	5303857	A	A	A	A	A	A
2138	rs6278204	12	5982290	C	C	G	C	C	C
2139	rs4138084	12	6177730	A	A	C	A	A	A
2140	rs6283105	12	6964499	G	G	G	G	G	G
2141	rs4217527	12	7360628	C	A	C	C	C	C
2142	rs3678128	12	7371530	C	A	A	C	C	C
2143	rs6370381	12	7437227	C	A	A	C	C	C

2144	rs3680632	12	8342543	A	G	A	A	A	A
2145	rs8243609	12	8807777	G	G	G	G	G	G
2146	rs8259864	12	8809713	A	A	A	A	A	A
2147	rs3089046	12	9007288	G	G	G	G	G	G
2148	mCV23300324	12	11987108	A	A	A	C	C	A
2149	rs6176675	12	9248169	A	C	A	A	A	A
2150	rs3717661	12	9585058	C	C	C	G	G	C
2151	rs6389512	12	9607391	G	G	G	A	A	G
2152	rs3708337	12	9687168	G	G	G	A	A	G
2153	rs3709002	12	9687267	T	A	T	A	A	T
2154	rs3688676	12	10301624	C	C	C	G	C	C
2155	rs3090664	12	10432887	A	A	A	G	A	A
2156	rs6169448	12	10454406	T	T	T	A	T	T
2157	rs4229244	12	10485656	A	A	A	A	A	A
2158	rs4229245	12	10485704	T	T	T	T	T	T
2159	rs4229247	12	10485803	T	T	T	T	T	T
2160	rs3665012	12	10622266	A	A	A	C	A	A
2161	rs3707458	12	10627537	G	G	G	A	G	G
2162	rs3708624	12	10627687	G	G	G	C	G	G
2163	rs6209157	12	10664050	C	C	C	A	A	C
2164	rs3717933	12	11197043	G	G	G	A	G	G
2165	rs3689412	12	11257698	A	A	G	G	A	G
2166	rs6197264	12	11354427	A	C	A	A	A	A
2167	rs3695968	12	11918334	T	A	A	T	A	A
2168	rs3718992	12	12114801	A	G	A	G	A	A
2169	rs3090132	12	12260007	A	C	A	C	A	A
2170	rs3725027	12	12296917	G	G	A	A	G	A
2171	rs3693390	12	12630509	G	G	A	G	A	A
2172	rs6271997	12	12631788	G	C	C	G	C	C
2173	rs6254898	12	12820070	A	G	A	A	A	A
2174	rs3090234	12	13120082	C	C	C	C	C	C
2175	rs3692433	12	13244341	G	A	G	G	G	G
2176	rs3715715	12	13280695	A	G	A	G	A	A
2177	rs4229249	12	13345894	G	G	G	G	G	G
2178	rs3655057	12	13507742	A	A	G	G	G	G
2179	rs3088558	12	13622262	A	A	A	A	A	A
2180	rs6186506	12	13748340	G	A	G	G	G	G
2181	rs3716261	12	13997370	A	G	A	A	A	A
2182	rs6277726	12	14270128	A	A	A	A	A	A
2183	rs3653621	12	14316714	G	G	A	A	A	A
2184	rs3662757	12	14382136	A	A	G	G	G	G
2185	rs6403731	12	15047192	A	G	A	A	A	A
2186	rs6235226	12	15177221	G	G	A	A	A	A
2187	rs3696623	12	15276918	A	A	G	G	G	G
2188	rs3712011	12	15661214	C	A	C	A	A	C
2189	rs3715137	12	15661664	G	A	G	A	A	G
2190	rs3657682	12	15855810	A	C	A	C	A	A
2191	mCV24328836	12	16297134	C	A	C	A	C	C
2192	rs3658504	12	16292652	A	A	A	G	A	A
2193	rs6376853	12	16420934	A	A	A	G	A	A
2194	rs6292712	12	16437176	A	A	A	G	A	A
2195	rs3706330	12	16713381	G	G	A	A	A	G
2196	rs3694821	12	16716565	A	A	A	G	A	A
2197	rs3701159	12	16862980	A	G	G	A	A	A
2198	rs6180964	12	16960089	G	G	G	A	G	G
2199	mCV24339635	12	17249793	C	C	C	A	NONE	C
2200	rs3665211	12	17001811	T	T	T	A	T	T
2201	rs6163686	12	17301280	C	C	C	G	C	C
2202	rs3667752	12	17608958	A	A	A	G	A	A
2203	rs6375532	12	3070988	C	C	C	G	C	C
2204	rs4229258	12	1413031	G	G	G	A	G	G
2205	rs4229250	12	1412792	A	A	A	A/C	A	A
2206	rs3667585	12	1490933	C	C	C	G	C	C
2207	rs3717860	12	19689285	A	A	A	C	A	A
2208	rs3655651	12	19733458	A	A	A	G	A	A
2209	rs3023339	12	19765125	C	C	C	A	C	C
2210	rs6183596	12	19790476	C	C	C	C	C	C
2211	rs4229275	12	20022319	G	G	G	G	G	G
2212	rs4229284	12	20058227	C	C	C	A	C	C
2213	rs3719944	12	20284893	G	G	G	G	G	G
2214	rs6221035	12	20786909	G	G	G	G	G	G
2215	mCV25284008	5	12135883	C	A	A	C	A	A
2216	rs6328018	12	21090189	A	G	A	A	A	A
2217	rs4229294	12	21263775	C	C	C	A	C	C

2218	rs3710723	12	21279851	G	G	G	A	G	G
2219	rs6401555	12	21400794	A	A	A	T	T	A
2220	rs6271290	12	21531861	T	T	T	T	T	T
2221	mCV23582150	5	12883363	G	A	A	G	A	A
2222	rs3666132	12	22018908	C	C	C	A	C	C
2223	rs3705249	12	22027283	G	G	G	A	G	G
2224	rs3694635	12	22209879	A	A	A	G	A	A
2225	rs3713853	12	22276217	G	G	G	A	G	G
2226	rs3684699	12	22491531	C	C	C	A	C	C
2227	mCV22670586	5	13894947	A	G	G	G	G	G
2228	rs6238712	12	22789619	G	G	G	A	G	G
2229	rs3708704	12	23402031	G	G	G	A	G	G
2230	rs6307981	12	23588362	C	C	C	C	C	C
2231	rs3655333	12	23704034	A	A	A	G	A	A
2232	rs3700242	12	23732399	G	A	A	A	G	A
2233	rs6292845	12	23821732	A	A	A	C	A	A
2234	rs3685299	12	23872081	G	G	G	A	G	G
2235	rs3089800	12	23976362	A	G	G	A	A	G
2236	rs3680640	12	24038099	G	G	G	A	G	G
2237	rs3715020	12	24378566	G	G	G	A	G	G
2238	rs3707174	12	24688708	A	A	A	G	A	A
2239	rs3719667	12	24761765	G	G	G	A	G	G
2240	rs6284233	12	24766153	G	G	G	G	G	G
2241	rs6328532	12	24780605	A	A	A	G	A	A
2242	rs3694816	12	24784007	C	C	C	A	C	C
2243	rs4229311	12	24908259	G	G	G	G	G	G
2244	rs3023342	12	25037508	G	G	G	A	G	G
2245	rs3663018	12	25212442	G	G	G	A	G	A
2246	rs6381718	12	25283139	G	G	G	A	G	G
2247	rs6191026	12	25560891	G	G	G	G	G	G
2248	rs3688798	12	25701041	C	C	C	A	C	C
2249	rs3724411	12	25751719	A	A	A	G	A	A
2250	rs3088883	12	25419978	A	A	A	T	A	A
2251	rs6156567	12	26353043	A	A	A	A	A	A
2252	rs3667808	12	26623728	G	G	G	A	A	A
2253	rs3695121	12	26627241	A	A	A	G	G	G
2254	rs4135956	12	26709335	A	A	A	G	G	G
2255	rs6391257	12	26832868	A	A	A	A	A	A
2256	rs3708270	12	27204179	A	A	A	C	C	C
2257	rs4229312	12	27395395	C	C	C	C	C	C
2258	rs3654802	12	27460625	G	G	G	A	A	A
2259	rs3658897	12	27568325	A	A	A	G	G	G
2260	rs6349075	12	27734606	A	A	A	A	A	A
2261	rs3702802	12	28177625	G	A	A	G	G	G
2262	rs3668221	12	28310479	A	G	G	G	G	G
2263	rs6187012	12	28579503	C	C	C	A	A	A
2264	rs3666912	12	28640887	G	A	A	A	A	A
2265	rs3713102	12	28757599	A	G	G	A	A	A
2266	rs3688343	12	29294408	C	A	A	C	C	C
2267	rs3684855	12	29356050	A	G	G	A	A	A
2268	rs6203767	12	29603206	A	A	A	A	A	A
2269	rs3655860	12	29796244	G	A	A	G	A	A
2270	rs3724198	12	29803337	A	G	G	G	A	G
2271	mCV24802848	12	26126892	A	A	A	A	A	A
2272	rs3679148	12	30186113	G	G	G	C	G	G
2273	rs6182576	12	30449399	A	G	G	A	A	G
2274	rs3711448	12	30453856	A	G	G	G	A	G
2275	rs3021923	12	30553841	A	G	G	A	A	G
2276	rs6390300	12	31065032	G	A	A	G	G	A
2277	rs4229321	12	31224458	G	G	G	G	G	G
2278	rs4229313	12	31224702	G	G	G	G	G	G
2279	rs3695382	12	31352037	A	G	G	A	A	A
2280	rs3696037	12	31352149	G	A	A	G	G	G
2281	rs6278497	12	31494698	A	G	G	A	A	A
2282	rs4229333	12	31714203	A	A	A	A	A	A
2283	rs4229328	12	31714425	A	A	A	A	A	A
2284	rs6212948	12	32138344	A	A	A	A	A	A
2285	rs3023209	12	32405486	A	A	A	A	G	G
2286	rs3088766	12	32811200	C	A	A	C	C	C
2287	mCV24442129	12	29148107	A	A	A	G	G	A
2288	rs3659601	12	32889324	G	A	A	G	G	A
2289	rs6317604	12	32915193	G	G	G	G	G	G
2290	rs3658100	12	33110635	A	G	G	A	G	G
2291	rs6218247	12	33460040	A	A	A	A	A	A

2292	rs3090124	12	33900011	G	G	G	G	G	G
2293	rs6316395	12	34049383	C	C	C	C	C	C
2294	rs6340536	12	34772179	G	G	G	G	G	G
2295	rs6408286	12	35243144	G	G	G	G	G	G
2296	rs6263867	12	35365869	A	A	A	A	A	A
2297	rs3665526	12	35440052	G	C	C	C	C	C
2298	rs6313441	12	36653630	G	G	G	G	G	G
2299	rs6177471	12	37020899	G	G	G	G	G	G
2300	rs3716646	12	37775611	G	A	A	G	A	G
2301	rs3658320	12	37775748	A	G	G	A	G	A
2302	rs3670955	12	38018739	A	C	C	A	C	C
2303	mCV23299449	12	34544453	A	A	A	A	G	A
2304	rs6387250	12	38370786	A	A	A	A	A	A
2305	rs6369313	12	38835107	A	A	A	A	A	A
2306	mCV24388900	12	35498829	A	A	A	A	A	A
2307	rs6343325	12	39324445	G	G	G	G	G	G
2308	rs3726096	12	39432172	G	A	A	G	A	G
2309	mCV24385481	12	36365147	C	A	A	A	C	A
2310	rs3686437	12	39778323	T	A	A	T	A	T
2311	rs3701242	12	40487282	A	G	G	G	A	A
2312	rs6197363	12	40497255	G	G	G	G	G	G
2313	rs6215996	12	41135438	A	A	A	A	A	A
2314	rs3667804	12	41704542	A	A	A	T	A	A
2315	rs3667177	12	41760552	A	T	T	A	A	A
2316	rs6295757	12	41944962	T	T	T	T	T	T
2317	rs3709102	12	41980455	C	C	C	A	C	C
2318	rs8255801	12	41981787	G	G	G	G	G	G
2319	rs8255800	12	41982038	G	G	G	G	G	G
2320	rs3671614	12	42085961	G	A	A	A	G	G
2321	rs3714242	12	42476185	A	G	G	G	A	A
2322	rs6317361	12	42513715	G	A	A	G	G	G
2323	rs6272759	12	43108703	A	A	A	A	A	A
2324	rs6170134	12	43739756	A	A	A	A	A	A
2325	rs6292281	12	45189635	A	A	A	A	A	A
2326	rs6198011	12	45399957	C	C	C	C	C	C
2327	rs3724479	12	45630420	A	G	G	G	G	G
2328	rs6255802	12	45968866	G	A	A	G	G	G
2329	rs6170639	12	46297719	A	G	G	G	G	G
2330	rs3676345	12	46298146	C	A	A	A	A	A
2331	rs4229358	12	46958585	G	G	G	G	G	G
2332	rs4229359	12	46958614	G	G	G	G	G	G
2333	rs6213741	12	46811633	A	T	T	T	T	T
2334	rs3673246	12	47202251	G	G	G	A	A	A
2335	rs3663333	12	47287387	G	A	A	G	G	G
2336	rs3727109	12	47512278	A	C	C	C	C	C
2337	rs3663603	12	47893550	G	G	G	A	A	A
2338	rs3677499	12	47917557	G	A	A	G	G	G
2339	rs3690123	12	47947796	G	A	A	A	A	A
2340	rs3670749	12	48181013	A	A	A	G	G	A
2341	rs4229363	12	48372377	A	G	G	G	G	G
2342	rs6359128	12	48495671	A	G	G	G	G	G
2343	rs3701314	12	48536764	G	A	A	A	A	A
2344	rs2020842	12	48630447	G	A	A	A	A	A
2345	rs4135641	12	49059109	G	G	G	A	A	G
2346	rs3700857	12	49142988	A	A	A	G	G	G
2347	rs6236032	12	49237121	A	A	A	A	A	A
2348	rs3725916	12	49242377	G	G	G	A	A	G
2349	rs6370023	12	49529840	G	G	G	G	G	G
2350	rs3689063	12	50059458	A	G	G	A	G	G
2351	rs6355857	12	50536420	A	A	A	A	A	A
2352	rs6222411	12	51604111	A	A	A	A	A	A
2353	rs3688680	12	51829630	C	A	A	C	A	C
2354	rs6159691	12	52315800	C	G	G	C	G	G
2355	rs3704934	12	52424699	G	A	A	G	A	A
2356	rs4229370	12	52437813	A	A	A	A	A	A
2357	rs3659385	12	52773393	G	A	A	G	A	A
2358	rs3719306	12	52871066	A	G	G	A	G	G
2359	rs3726434	12	52875088	A	G	G	A	G	G
2360	rs6226467	12	52986261	C	C	C	C	C	C
2361	rs3705833	12	53150854	G	G	G	A	G	G
2362	rs3717784	12	53229241	G	G	G	A	G	G
2363	rs3685560	12	53280710	C	G	G	C	G	G
2364	rs4229376	12	53465273	A	A	A	A	A	A
2365	rs4229375	12	53465379	A	A	A	A	A	A

2366	rs6347754	12	53939576	A	A	A	A	A	A
2367	rs3662939	12	54082727	A	T	T	T	T	A
2368	rs3664781	12	54576349	C	C	C	A	C	C
2369	rs3674641	12	55129109	G	G	G	A	A	G
2370	rs3690129	12	55338079	A	A	A	C	C	A
2371	rs6411363	12	55345736	G	G	G	G	G	G
2372	rs6215044	12	55927846	G	G	G	G	G	G
2373	rs3721804	12	56413174	T	A	A	T	T	A
2374	rs4139129	12	56528942	A	A	A	G	G	A
2375	rs3660822	12	56812162	A	C	C	A	A	A
2376	mCV22351241	12	54143643	A	G	G	G	G	A
2377	rs6276393	12	57076990	A	A	A	A	A	A
2378	rs6381937	12	57623071	A	A	A	A	A	A
2379	rs3673599	12	58027245	A	G	G	A	G	G
2380	rs6386766	12	58739672	T	A	A	T	A	A
2381	rs6400991	12	58779990	G	G	G	G	G	G
2382	mCV24253983	12	56253182	G	A	A	G	A	G
2383	rs3657568	12	59225844	A	A	A	C	C	C
2384	rs3707833	12	59283229	A	A	A	T	A	T
2385	rs6199243	12	59345732	A	A	A	A	A	A
2386	rs3681130	12	59481270	G	G	G	A	G	A
2387	rs3677344	12	59848542	C	C	C	A	A	A
2388	rs3724627	12	60632584	G	G	G	A	A	G
2389	rs3686891	12	60735544	A	G	G	A	A	G
2390	mCV24690992	12	58174760	G	A	A	G	A	G
2391	rs4137680	12	61533001	C	C	C	G	G	G
2392	rs3668686	12	61540175	C	C	C	A	A	A
2393	rs3669929	12	61540420	A	A	A	C	C	C
2394	rs3685346	12	61540594	A	A	A	G	G	G
2395	rs6329307	12	61910476	G	G	G	G	G	G
2396	rs3691763	12	61922672	A	A	A	G	G	G
2397	rs4229388	12	62011126	A	A	A	A	A	A
2398	rs3660226	12	62208538	A	A	A	G	G	A
2399	rs3689341	12	62652711	G	A	A	A	A	A
2400	rs6185479	12	62706123	C	C	C	C	C	C
2401	rs3716547	12	62831831	A	C	C	C	A	C
2402	rs6341977	12	63021698	A	A	A	A	A	A
2403	rs3686378	12	63477601	A	A	A	G	G	G
2404	rs3089561	12	63517344	A	A	A	A	A	A
2405	rs6357043	12	64059650	A	G	G	A	A	G
2406	rs6348266	12	64170164	G	G	G	G	G	G
2407	rs3713923	12	64368458	G	A	A	G	G	G
2408	rs6383133	12	64426353	G	A	A	G	G	A
2409	rs3660644	12	64473664	C	C	C	A	A	C
2410	rs3705675	12	65105842	G	G	G	A	G	G
2411	rs4229406	12	65290824	G	G	G	G	G	G
2412	rs4229408	12	65290926	A	A	A	A	A	A
2413	rs6195664	12	65498422	A	C	C	A	C	C
2414	rs3023346	12	65522126	G	A	A	G	A	A
2415	rs3689845	12	65526885	A	G	G	A	G	G
2416	rs6327000	12	65590325	A	A	A	A	A	A
2417	rs3088485	12	65790339	G	G	G	G	G	G
2418	rs6229531	12	66516892	G	G	G	G	G	G
2419	rs3662140	12	67180434	G	A	A	A	A	G
2420	rs3669366	12	67275491	A	A	A	G	G	A
2421	rs3674010	12	67423261	G	C	C	C	C	G
2422	rs3674090	12	67673332	A	G	G	A	A	A
2423	rs3695552	12	67683895	A	G	G	A	A	G
2424	rs6203329	12	67927371	A	G	G	G	G	G
2425	rs3714720	12	68057798	A	C	C	A	A	A
2426	rs4229413	12	68073270	G	G	G	G	G	G
2427	rs4229411	12	68073471	A	A	A	A	A	A
2428	rs4229417	12	68322473	A	A	A	A	A	A
2429	rs4229415	12	68322697	G	A	A	A	A	A
2430	rs6387942	12	68566805	G	G	G	G	G	G
2431	rs3687032	12	68778319	G	G	G	A	A	A
2432	rs3683927	12	68885189	A	G	G	G	G	G
2433	rs4137363	12	69184710	C	C	C	A	A	C
2434	rs3023349	12	69210214	T	T	T	A	A	T
2435	rs8259450	12	69221987	G	G	G	A	A	G
2436	rs3685417	12	69267758	A	A	A	G	G	G
2437	rs6325248	12	69695532	C	C	C	C	C	C
2438	rs3709008	12	69967120	G	G	G	A	A	G
2439	rs3656227	12	70010983	G	G	G	A	A	G

2440	rs3658768	12	70011385	A	A	A	G	G	A
2441	rs6346026	12	70290897	G	A	A	A	G	G
2442	rs6300343	12	71305416	C	C	C	C	C	C
2443	rs3665469	12	71367121	G	A	A	A	G	A
2444	rs3682382	12	71586706	G	A	A	A	A	A
2445	rs6344480	12	71913451	A	A	A	A	A	A
2446	rs4229420	12	72092079	A	A	A	A	A	A
2447	rs3700106	12	72162132	G	G	G	A	A	G
2448	rs6403750	12	72538872	G	G	G	G	G	G
2449	rs4229428	12	73374791	G	G	G	G	G	G
2450	rs3724069	12	73456090	G	C	C	C	C	C
2451	rs6263997	12	73504905	A	A	A	A	A	A
2452	rs6308760	12	74349342	G	G	G	G	G	G
2453	mCV23169261	12	72254494	C	C	C	A	A	C
2454	rs6199755	12	74448630	A	A	A	A	A	A
2455	rs4229440	12	74470873	T	T	T	T	T	T
2456	rs3662628	12	74558132	A	G	G	G	G	A
2457	rs6320805	12	74958005	G	A	A	A	A	G
2458	rs3696769	12	75038513	G	A	A	A	A	A
2459	rs3674330	12	75415973	A	G	G	A	G	G
2460	rs3689015	12	75460620	G	A	A	A	A	G
2461	rs4229448	12	75537438	A	A	A	A	A	G
2462	rs6379829	12	75637041	G	G	G	G	G	G
2463	rs8238659	12	76255843	A	A	A	A	A	A
2464	rs8238658	12	76262730	G	G	G	G	G	G
2465	rs3654718	12	76277248	G	G	G	A	G	G
2466	rs3693948	12	76279025	G	G	G	A	G	G
2467	rs3725854	12	76440446	G	A	A	G	A	A
2468	rs3653954	12	76440760	G	A	A	G	A	A
2469	rs4229449	12	76874876	G	G	G	G	G	G
2470	rs6170344	12	76890398	G	C	C	G	C	C
2471	rs6205632	12	76936954	A	G	G	A	G	G
2472	rs3696951	12	77991846	A	G	G	A	G	A
2473	rs6263380	12	78119135	C	A	A	C	A	C
2474	rs3088680	12	78294575	C	C	C	C	C	C
2475	rs4229463	12	78526744	A	A	A	A	A	A
2476	rs6224091	12	79325737	A	A	A	A	A	A
2477	rs3658658	12	79821009	A	A	A	A	A	A
2478	rs3088896	12	79829077	C	C	C	C	C	C
2479	rs6401803	12	80045455	A	A	A	A	A	A
2480	rs4229466	12	80239439	G	G	G	G	G	G
2481	rs4229469	12	80489865	G	G	G	G	G	G
2482	mCV22806754	12	78442240	A	A	A	A	A	A
2483	rs6298386	12	80919627	A	A	A	A	A	A
2484	rs6261325	12	80956834	G	G	G	G	G	G
2485	rs3692226	12	81341363	C	C	C	C	C	C
2486	rs4229478	12	81555593	A	A	A	A	A	A
2487	rs6387971	12	81958771	A	A	A	A	A	G
2488	rs4229483	12	82293493	G	G	G	G	G	G
2489	rs4229484	12	82293655	C	C	C	C	C	C
2490	rs3719282	12	82356288	G	G	G	A	G	G
2491	rs3660605	12	82425750	A	A	A	G	A	G
2492	rs4222057	12	82441531	A	A	A	A	A	A
2493	rs8259763	12	82491932	A	A	A	G	A	A
2494	rs8259789	12	82496042	A	A	A	A	A	A
2495	rs6353902	12	83062418	G	G	G	A	G	G
2496	rs3699539	12	83175209	G	G	G	A	G	A
2497	rs6351282	12	83633780	G	G	G	G	G	G
2498	rs3711162	12	83723888	G	A	A	A	A	G
2499	rs4229495	12	84926043	A	A	A	A	A	A
2500	rs3682466	12	85218939	A	A	A	A	A	A
2501	rs6347765	12	85263005	C	C	C	C	C	G
2502	rs6288403	12	85563437	A	A	A	T	A	A
2503	rs3670410	12	85932990	G	G	G	A	G	G
2504	rs4229506	12	86141518	G	G	G	A	G	A
2505	rs3088937	12	86151911	A	A	A	A	A	A
2506	rs3089776	12	86631131	A	A	A	A	A	A
2507	rs3720969	12	87023258	C	C	C	A	C	C
2508	rs3676063	12	87028895	A	A	A	G	A	A
2509	rs6213448	12	87072159	A	A	A	A	A	A
2510	rs6399840	12	87554618	A	A	A	A	A	A
2511	rs3676085	12	87765992	A	G	G	A	G	G
2512	rs3709687	12	88015458	C	C	C	C	C	C
2513	rs6214514	12	88127638	A	C	C	A	C	A

2514	rs6356138	12	88350411	A	G	G	A	G	A
2515	rs3090088	12	88420775	C	C	C	C	C	C
2516	rs6408857	12	89113620	A	A	A	A	A	A
2517	rs3090231	12	89644192	G	G	G	G	G	G
2518	rs6297004	12	89782983	G	G	G	G	G	G
2519	rs6217018	12	91062241	A	A	A	A	A	A
2520	rs6244774	12	91861271	G	G	G	G	G	G
2521	rs3679514	12	92576929	G	A	A	A	A	G
2522	rs6228161	12	93072334	G	G	G	G	G	G
2523	rs3089061	12	93396509	A	A	A	A	A	A
2524	rs4136039	12	93415640	G	A	A	A	A	G
2525	rs6269096	12	93648867	T	A	A	A	A	T
2526	rs3666004	12	94082142	G	C	C	C	C	G
2527	rs3716084	12	94501426	G	A	A	A	G	G
2528	rs6177548	12	94582022	A	A	A	G	A	A
2529	rs3674235	12	94587391	G	G	G	A	G	G
2530	rs6263565	12	94855105	G	G	G	G	G	G
2531	rs3698001	12	95092951	G	G	G	A	A	A
2532	rs3698544	12	95093015	G	G	G	A	A	A
2533	rs6295839	12	95201551	G	G	G	G	G	G
2534	rs6184745	12	95492537	A	G	G	G	A	A
2535	rs3695075	12	96232684	C	A	A	A	C	C
2536	rs3700012	12	96438694	A	G	G	G	G	A
2537	rs6388125	12	96515763	A	A	A	A	A	A
2538	rs6245139	12	96914467	A	A	A	A	A	A
2539	rs3693605	12	96990963	C	A	A	A	C	C
2540	rs4229526	12	97077380	G	G	G	G	G	G
2541	rs6227567	12	97634630	A	A	A	A	A	A
2542	rs3088666	12	97704550	G	G	G	G	G	G
2543	rs4229534	12	98084994	C	C	C	C	C	C
2544	rs6278016	12	98325422	A	A	A	A	A	A
2545	rs8271271	12	98863568	G	G	G	G	A	A
2546	rs3724260	12	99431602	A	A	A	A	G	G
2547	rs3663596	12	99565379	G	A	A	A	G	G
2548	rs6216501	12	99776400	A	A	A	A	A	A
2549	rs8245329	12	100310626	A	A	A	A	A	A
2550	rs8245330	12	100310731	A	A	A	A	A	A
2551	rs8245337	12	100310929	A	A	A	A	A	A
2552	rs8245333	12	100311022	A	A	A	A	A	A
2553	rs6317509	12	100757538	C	C	C	C	C	C
2554	rs3667299	12	101109359	G	G	G	G	G	G
2555	rs6376610	12	101185032	A	A	A	A	A	A
2556	rs3721148	12	101353045	C	C	C	C	C	A
2557	rs3653844	12	102255090	A	C	C	A	A	A
2558	rs6303051	12	102387656	G	A	A	G	G	G
2559	rs3663172	12	102647407	T	A	A	T	A	A
2560	rs3694191	12	102651625	A	A	C	A	A	A
2561	rs6223643	12	102961454	G	G	A	G	G	G
2562	rs6239861	12	103259405	A	A	G	A	A	A
2563	rs3707852	12	103274219	A	A	C	A	A	A
2564	rs3707436	12	103442463	G	G	A	G	G	G
2565	rs6348190	12	104342236	G	G	G	G	G	G
2566	rs4229571	12	104497467	C	C	C	C	C	C
2567	rs3672124	12	105361486	A	G	G	A	A	A
2568	rs6345717	12	105432501	G	G	G	G	G	G
2569	mCV23906390	12	102852763	A	G	A	A	G	A
2570	rs3674174	12	105774185	C	C	A	C	C	C
2571	rs6396219	12	105853934	A	A	A	A	A	A
2572	rs4229586	12	106457683	A	C	C	A	C	C
2573	rs3693074	12	106596291	A	G	G	A	G	G
2574	rs6333099	12	106604815	A	G	G	A	G	G
2575	rs3691931	12	107204115	A	G	G	A	G	G
2576	rs4229602	12	107367411	A	G	G	A	A	G
2577	rs4229600	12	107367519	A	A	A	A	A	A
2578	rs6406827	12	107766327	A	G	G	A	A	G
2579	rs6310252	12	107970036	G	G	G	G	G	G
2580	rs4229611	12	108009492	G	A	G	A	G	G
2581	rs4229612	12	108009606	A	A	A	G	G	G
2582	rs4229613	12	108009694	A	A	A	A	A	A
2583	rs4140265	12	108065683	G	G	G	A	G	G
2584	rs3021903	12	108081557	G	A	A	G	G	G
2585	rs3090605	12	108183110	G	A	G	G	G	G
2586	rs6278495	12	108970749	G	G	G	G	G	G
2587	mCV23895237	12	106512272	G	A	A	G	A	A

2588	mCV23895227	12	106515798	T	A	A	T	A	A
2589	mCV23895225	12	106515931	A	G	G	A	G	G
2590	mCV23894910	12	106516117	G	A	A	G	A	A
2591	mCV23894909	12	106516181	G	C	C	G	C	C
2592	mCV23894908	12	106519874	G	A	A	G	A	A
2593	mCV23894889	12	106522357	T	A	A	T	A	A
2594	mCV23894888	12	106522367	A	G	G	A	G	G
2595	mCV23894883	12	106529000	A	G	G	A	G	G
2596	mCV23894882	12	106529149	G	C	C	G	C	C
2597	mCV23894881	12	106529161	G	NONE	A	G	A	A
2598	mCV23893876	12	106529430	A	G	A	A	A	A
2599	mCV23893864	12	106530153	A	G	G	A	G	A
2600	mCV23893863	12	106530190	A	T	T	A	T	T
2601	mCV23893852	12	106530561	A	G	G	A	G	A
2602	mCV23893851	12	106530652	A	G	G	A	G	G
2603	mCV23893844	12	106531971	A	G	G	A	G	A
2604	mCV23893843	12	106532041	G	A	A	G	A	A
2605	mCV23893842	12	106532478	G	A	A	G	A	G
2606	mCV23893832	12	109982126	A	C	C	A	C	A
2607	mCV24934271	12	106535074	G	C	C	G	C	G
2608	mCV24934270	12	106535100	A	G	G	A	G	A
2609	mCV24934262	12	106535327	A	C	C	A	C	C
2610	mCV24934253	12	106545944	G	G	G	G	G	G
2611	mCV24934246	12	106546390	G	A	A	G	A	A
2612	mCV24934244	12	106546546	C	A	A	C	A	A
2613	mCV24934236	12	106546964	C	A	A	C	A	A
2614	mCV24934234	12	106547162	G	A	A	G	A	A
2615	mCV24934227	12	106547494	A	G	G	A	G	G
2616	mCV24934226	12	106547539	C	G	G	C	G	G
2617	mCV24934218	12	106547730	A	G	G	A	G	G
2618	rs3709829	12	109139529	A	A	C	A	A	A
2619	rs3711043	12	109139726	G	G	C	G	G	G
2620	rs4138747	12	109261892	A/T	T	T	A	T	T
2621	rs3679326	12	109442366	G	A	A	A	A	A
2622	mCV23100902	12	107255389	A	G	G	A	G	G
2623	rs6153883	12	109848662	A	A	G	A	A	A
2624	rs6376011	12	110360295	A	A	A	C	NONE	A
2625	mCV23887919	12	111901600	T	T	T	T	T	T
2626	rs6403551	12	111018750	A	T	A	T	A	A
2627	rs3688774	12	111130939	G	G	G	A	G	G
2628	rs3700528	12	111290218	G	G	G	A	G	G
2629	rs3670898	12	111707329	G	G	G	C	G	G
2630	rs6320467	12	111953626	G	G	G	G	G	G
2631	rs3654706	12	112065076	G	G	G	A	A	G
2632	rs3713779	12	112325864	C	A	C	A	A	A
2633	rs3694890	12	112374446	A	G	A	G	A	A
2634	rs3705923	12	112411308	G	G	G	A	G	A
2635	rs6292408	12	112644408	C	G	C	C	C	G
2636	rs3692361	12	112704261	G	G	G	A	G	A
2637	rs4222060	12	112741936	G	G	G	G	G	G
2638	rs4222062	12	112742112	A	A	A	G	A	G
2639	rs6284477	12	112755139	A	A	A	A	A	A
2640	rs3719921	12	112976311	A	C	A	C	A	C
2641	rs6395554	12	113904524	G	G	G	G	G	G
2642	rs4135448	12	114088565	G	G	G	A	G	A
2643	rs6327003	12	114194856	G	G	G	G	G	G
2644	rs3672335	12	114651707	C	C	C	A	C	A
2645	rs3686531	12	114773303	G	G	G	A	G	A
2646	rs3679276	12	114928237	A	G	A	G	A	G
2647	mCV24244050	13	119922	G	G	A	A	A	G
2648	rs6215262	13	3556871	A	A	G	G	G	A
2649	rs3661352	13	3641527	G	G	C	C	C	G
2650	rs3695486	13	3931389	G	G	A	A	A	G
2651	rs3721849	13	4363382	C	C	C	C	C	C
2652	rs6384115	13	4381215	A	A	A	A	A	A
2653	rs6249257	13	4596693	C	C	C	C	C	C
2654	rs3656652	13	4671653	G	G	A	A	A	G
2655	rs3686663	13	4702493	G	G	A	A	A	G
2656	rs3713268	13	4795576	G	G	A	A	A	G
2657	rs6298246	13	5123199	G	G	G	G	G	G
2658	rs6364621	13	5133192	G	G	G	G	G	G
2659	rs6318987	13	5767683	G	G	G	G	G	G
2660	rs4229621	13	6009386	A	A	A	A	A	A
2661	rs3694393	13	33544897	G	C	C	G	C	G

2662	rs6243819	13	6897674	G	G	G	G	G	G
2663	rs6301008	13	7490090	G	G	G	G	G	G
2664	mCV24980830	13	11184998	A	T	T	T	T	T
2665	rs6250327	13	8160005	A	A	A	A	A	A
2666	rs6178370	13	8898140	C	C	C	C	C	C
2667	rs3695750	13	9264112	C	G	G	G	G	G
2668	rs6329684	13	9950160	G	A	A	A	A	A
2669	rs3090033	13	10200383	A	A	A	A	A	A
2670	rs4229629	13	10420533	C	A	A	A	A	A
2671	rs4229628	13	10420670	G	G	G	G	G	G
2672	mCV23972264	13	12914818	A	G	G	G	G	G
2673	rs3717128	13	11339529	A	G	G	G	G	G
2674	rs3691043	13	11505904	A	G	G	G	G	G
2675	rs6187266	13	11603829	A	T	T	T	T	T
2676	rs6153178	13	12363118	A	G	G	G	G	G
2677	rs3663551	13	12426262	A	G	G	G	G	A
2678	rs6192173	13	12522128	T	A	A	A	A	A
2679	rs4232414	13	13010590	G	G	G	G	G	G
2680	rs6257075	13	13128340	A	C	C	C	C	A
2681	rs3691785	13	13330479	C	A	A	A	A	A
2682	rs6304179	13	13555082	A	A	A	A	A	A
2683	rs4229632	13	13960934	A	A	A	A	A	A
2684	rs3684256	13	14258620	G	A	A	A	A	A
2685	rs6352759	13	14657783	T	T	T	T	T	T
2686	rs4229656	13	15084608	A	A	A	A	A	A
2687	rs6180409	13	15468943	A	A	A	A	A	A
2688	rs3701757	13	15541555	G	G	G	A	G	G
2689	rs6335891	13	15938044	C	C	C	C	C	G
2690	rs3674247	13	16167183	G	G	G	A	G	G
2691	rs3695969	13	16217832	G	G	G	A	G	G
2692	rs3705255	13	16630127	A	G	G	G	G	A
2693	rs3678616	13	16665395	C	A	A	C	A	C
2694	rs3721858	13	16752051	G	A	A	G	A	A
2695	rs3699519	13	16786707	G	A	A	G	A	A
2696	rs6349338	13	16856178	G	G	G	G	G	G
2697	rs6320527	13	17525335	A	A	A	A	A	A
2698	rs3023379	13	17669053	G	G	G	A	G	G
2699	rs3715806	13	17712010	G	G	G	A	G	G
2700	rs3693929	13	17744115	G	G	G	A	G	G
2701	rs4229666	13	17940399	C	C	C	C	C	C
2702	rs3656183	13	18147750	G	G	G	A	G	G
2703	rs3656715	13	18147800	A	A	A	G	A	A
2704	rs6167514	13	18429845	A	A	A	A	A	A
2705	rs3701424	13	18788201	A	A	A	G	A	A
2706	rs3714148	13	19262448	G	G	G	A	G	G
2707	rs3700270	13	19520841	A	G	G	G	G	G
2708	rs3676630	13	19798486	A	G	G	A	G	G
2709	rs6367784	13	20007396	G	G	G	A	G	G
2710	rs3679784	13	20286264	G	A	A	G	A	A
2711	rs6226055	13	20359236	A	A	A	A	A	A
2712	rs3710676	13	20633724	C	C	C	A	C	C
2713	rs8266909	13	20668830	G	G	G	G	G	G
2714	rs8266906	13	20669498	C	C	C	C	C	C
2715	rs8266896	13	20671498	G	G	G	A	G	G
2716	rs3668544	13	21097503	A	G	G	G	G	G
2717	rs3091203	13	21159699	G	A	A	G	A	A
2718	rs6376461	13	22226310	A	A	A	A	A	A
2719	rs4229670	13	22230130	C	C	C	C	C	C
2720	rs4139825	13	23111032	A	A	A	A	A	A
2721	rs3681123	13	22603016	G	G	G	G	G	G
2722	rs6294004	13	22822215	A	A	A	A	A	A
2723	rs8267056	13	23080696	G	G	G	A	G	G
2724	rs8267050	13	23080918	A	A	A	C	A	A
2725	rs8267104	13	23085429	G	G	G	G	G	G
2726	rs8267012	13	23088244	G	G	G	G	G	G
2727	rs8266971	13	23601518	A	A	A	T	A	A
2728	rs3654242	13	23166741	A	A	A	T	A	A
2729	rs3719353	13	23170517	G	G	G	A	G	G
2730	rs6206744	13	24054287	A	A	A	A	A	A
2731	mCV23904194	13	27025016	A	A	A	A	A	A
2732	rs3090112	13	25254830	G	G	G	G	G	G
2733	rs6325339	13	25755040	C	C	C	C	C	C
2734	rs6391726	13	26232703	A	A	A	A	A	A
2735	rs4229678	13	26444733	A	A	A	A	A	A

2736	rs4229674	13	26444977	A	A	A	A	A	A
2737	mCV23833242	13	30504063	A	C	C	A	C	A
2738	rs6374214	13	27839476	A	A	A	A	A	A
2739	rs3089940	13	28368714	G	G	G	G	G	G
2740	rs6403272	13	29199486	G	G	G	G	G	G
2741	mCV23984302	13	32023514	C	G	G	C	G	C
2742	rs4229680	13	29762601	G	G	G	G	G	G
2743	rs3669896	13	29808059	C	A	A	A	A	A
2744	rs3682248	13	30430397	A	T	T	T	T	T
2745	rs3710348	13	30653000	C	C	C	A	C	A
2746	rs6192273	13	31114426	G	G	G	G	G	G
2747	rs6308946	13	31284166	A	G	G	A	G	A
2748	rs3711424	13	31374009	A	G	G	A	G	A
2749	rs3668303	13	31379494	A	G	G	A	G	A
2750	rs4139503	13	31418904	A	G	G	A	G	A
2751	rs3720707	13	31547838	A	G	G	A	G	A
2752	rs3687359	13	31791219	A	T	T	A	T	A
2753	rs8273595	13	32222998	G	G	G	G	G	G
2754	rs8273601	13	32226239	G	G	G	G	G	G
2755	rs4140378	13	32335386	A	G	G	A	G	A
2756	rs3710232	13	32446420	A	G	G	A	G	A
2757	mCV25144745	13	34616421	C	A	A	C	A	C
2758	rs6158160	13	33031997	G	G	G	G	G	G
2759	rs3707097	13	33614779	G	A	A	G	A	G
2760	rs3723893	13	33615142	G	A	A	G	A	G
2761	rs6355039	13	33629153	G	C	C	G	C	G
2762	rs3727136	13	33688045	G	A	A	G	A	G
2763	rs3089977	13	33867512	G	G	G	G	G	G
2764	rs3725187	13	34496200	G	A	A	G	A	G
2765	rs6238465	13	34677203	G	A	A	G	A	G
2766	rs3684485	13	34795024	A	G	G	A	G	A
2767	rs4229686	13	35310554	G	G	G	G	G	G
2768	rs6293765	13	35365452	A	G	G	A	G	A
2769	rs3726262	13	35576181	A	G	G	A	G	A
2770	rs6309675	13	35701126	T	T	T	T	T	T
2771	rs3655084	13	36719996	T	A	A	T	T	T
2772	rs3694317	13	36760555	G	A	A	G	G	G
2773	rs3090248	13	36767228	A	A	A	A	A	A
2774	rs3685130	13	36832493	G	A	A	A	A	G
2775	rs3088825	13	37071864	A	G	G	A	A	A
2776	rs6327619	13	37153901	C	C	C	G	G	C
2777	rs3660981	13	37175330	C	G	G	G	G	C
2778	rs3724709	13	37678501	A	A	A	C	C	A
2779	rs3684564	13	37868653	G	A	A	A	A	G
2780	rs4229688	13	37943851	G	G	G	G	G	G
2781	rs3716346	13	38186094	G	A	A	G	G	G
2782	rs3720620	13	38196197	T	A	A	T	T	T
2783	rs6206708	13	38372973	C	G	G	G	G	C
2784	rs6253201	13	38829631	C	C	C	C	C	C
2785	rs3701164	13	39243547	T	T	T	A	T	T
2786	rs6379320	13	39489274	A	A	A	G	A	A
2787	rs3700038	13	39820007	A	A	A	G	A	A
2788	rs3722232	13	40040441	A	G	G	A	A	G
2789	rs6213978	13	40182397	G	G	G	G	G	G
2790	rs4229691	13	40465202	G	G	G	G	G	G
2791	rs4229690	13	41056720	C	C	C	C	C	C
2792	rs3725354	13	40574587	G	G	G	A	G	G
2793	rs6350793	13	40575438	G	G	G	A	G	G
2794	rs6162128	13	40774787	A	A	A	A	A	A
2795	mCV22969866	13	42864639	A	A	A	G	G	A
2796	rs3089478	13	41391520	A	A	A	A	A	A
2797	rs6265536	13	41481623	C	C	C	C	C	C
2798	rs6271232	13	41903543	G	G	G	A	G	G
2799	rs3677720	13	41921003	A	A	A	G	A	A
2800	rs2020805	13	42333911	G	G	G	G	G	G
2801	rs6373215	13	42523480	A	A	A	G	G	A
2802	rs3712907	13	42620079	C	C	C	A	A	C
2803	rs3719648	13	42694778	A	A	A	T	T	A
2804	rs3675293	13	42716137	A	A	A	C	C	A
2805	rs3670127	13	42986342	C	C	C	G	G	C
2806	rs6162782	13	42992074	G	G	G	G	G	G
2807	rs6192767	13	43032625	G	G	G	G	G	G
2808	rs3089333	13	43304918	A	A	A	A	A	A
2809	rs3684719	13	43704533	G	G	G	A	A	G

2810	rs3685941	13	43775546	G	G	G	A	A	G
2811	rs3090922	13	43780947	G	G	G	G	G	G
2812	rs6356484	13	43840970	A	A	A	G	G	A
2813	rs3023382	13	43860643	C	C	C	A	A	C
2814	rs3691503	13	43995477	A	A	A	G	G	A
2815	rs4229697	13	44220656	G	G	G	G	G	G
2816	rs4229704	13	44314101	G	G	G	G	G	G
2817	rs6249046	13	44557548	A	A	A	G	G	A
2818	rs3089016	13	44681792	A	A	A	A	A	A
2819	rs6196305	13	44771304	G	G	G	A	G	G
2820	rs3661060	13	44811861	C	C	C	A	C	C
2821	rs3688207	13	44823434	A	G	G	A	G	G
2822	rs4229721	13	45011168	A	A	A	A	G	A
2823	rs4229720	13	45011293	G	G	G	G	A	G
2824	mCV22785372	13	47100064	G	G	G	A	G	G
2825	rs3709108	13	45123833	A	G	G	G	G	G
2826	rs3725387	13	45366723	A	A	A	G	A	A
2827	rs3657836	13	45532817	G	A	A	G	A	A
2828	rs6308694	13	45688433	G	A	A	A	A	A
2829	rs3707420	13	45977425	A	G	G	A	G	G
2830	rs6292911	13	46041913	G	G	G	G	G	G
2831	rs4138314	13	46971558	G	G	G	A	G	G
2832	rs6411274	13	46624183	A	A	A	G	A	A
2833	rs3670228	13	46843400	G	G	G	A	G	G
2834	rs3682400	13	47170242	A	A	A	G	A	A
2835	rs3684250	13	47170532	C	C	C	A	C	C
2836	rs3690108	13	47262499	C	C	C	A	A	C
2837	rs6244558	13	47310947	T	T	T	A	A	T
2838	rs3676930	13	47687184	G	G	G	G	G	G
2839	rs4229730	13	48073882	G	G	G	G	G	G
2840	mCV24538475	13	49385925	C	C	C	C	C	C
2841	rs6349701	13	48383909	G	G	G	G	G	G
2842	rs6291121	13	48886011	A	A	A	A	A	A
2843	rs3090370	13	49119443	A	A	A	A	A	A
2844	rs6385137	13	49689135	G	G	G	G	G	G
2845	mCV23262360	13	52589947	C	A	A	C	C	C
2846	rs6344027	13	51032933	G	G	G	G	G	G
2847	rs4229742	13	51536262	A	A	A	A	A	A
2848	rs6209128	13	51807160	G	A	A	G	A	A
2849	rs3704459	13	51880566	G	G	G	A	G	G
2850	rs3699522	13	52027179	C	C	C	G	C	C
2851	rs4229749	13	52371785	G	G	G	A	G	G
2852	rs3023086	13	52469453	A	A	A	G	A	A
2853	rs3709215	13	52704111	A	G	G	A	A	A
2854	rs3707721	13	52859852	G	G	G	A	A	A
2855	rs6222262	13	52913812	G	G	G	A	A	A
2856	rs3673174	13	53074279	C	C	C	A	A	A
2857	rs8243445	13	53259175	C	A	A	C	C	C
2858	rs3675767	13	53303366	A	G	G	A	A	A
2859	rs6236102	13	53365843	C	C	C	C	C	C
2860	rs4229756	13	53641500	C	C	C	C	C	C
2861	rs6326507	13	54000197	C	C	C	A	C	C
2862	rs3693942	13	54042656	C	C	C	G	C	C
2863	mCV25176885	13	55806563	A	C	C	A	A	A
2864	rs3675078	13	54123547	A	G	G	A	A	A
2865	rs3683883	13	54202535	A	G	G	G	A	A
2866	rs8267169	13	55175789	G	A	A	A	G	A
2867	rs8267171	13	54488142	G	G	G	G	G	G
2868	rs8267175	13	54488273	C	C	C	C	C	C
2869	rs8273881	13	54488875	G	G	G	A	G	A
2870	rs8273909	13	54535735	G	G	G	G	G	G
2871	rs8273911	13	54535877	A	A	A	A	A	A
2872	rs4229759	13	54603924	G	A	A	G	G	G
2873	rs3698807	13	55044434	G	G	G	A	G	A
2874	rs6305957	13	55174657	G	G	G	G	G	G
2875	rs3720782	13	55330594	A	A	A	G	A	G
2876	rs4232347	13	56066778	C	A	A	C	C	C
2877	rs4232351	13	56066893	A	A	A	A	A	A
2878	rs6272898	13	55401878	C	A	A	A	C	A
2879	rs3697320	13	55466675	G	G	G	A	G	A
2880	rs6372629	13	55557441	T	T	T	A	T	A
2881	rs3700819	13	56358945	G	G	G	A	G	A
2882	rs4229767	13	57156811	G	G	G	G	G	G
2883	mCV22624058	13	58311395	A	G	A	A	G	A

2884	rs6391937	13	56578346	A	C	C	C	A	C
2885	rs3700944	13	56939959	A	A	G	A	G	A
2886	mCV23289546	13	59003784	G	A	G	G	G	G
2887	rs6310690	13	57716148	G	G	G	G	G	G
2888	rs3689106	13	57839808	G	G	G	G	G	G
2889	rs6379978	13	57982535	A	A	A	A	A	A
2890	mCV24581409	13	59106119	G	A	G	G	G	G
2891	rs4184167	13	58491742	A	A	A	A	G	G
2892	mCV25327808	13	60445103	A	G	A	A	A	A
2893	rs3023383	13	59824860	G	G	G	G	G	A
2894	rs6246599	13	59253663	G	G	G	G	G	G
2895	rs8240205	13	59320792	A	A	A	A	A	A
2896	rs6362489	13	59719172	A	A	G	G	G	G
2897	rs6388524	13	59792763	A	A	G	G	G	A
2898	rs3700164	13	59865002	C	C	C	C	C	C
2899	rs4229799	13	60626795	G	G	G	G	G	G
2900	rs3089437	13	60683700	G	A	G	G	G	G
2901	mCV24354546	13	60961649	G	G	A	A	A	G
2902	rs6410679	13	60401563	A	G	A	A	A	A
2903	rs6279752	13	60625769	T	A	T	T	T	T
2904	rs3670894	13	61291661	A	A	G	G	G	G
2905	rs6255190	13	61332175	A	A	T	T	A	A
2906	rs3090506	13	61645356	G	G	G	G	G	G
2907	rs3708089	13	61820968	C	C	A	A	C	C
2908	rs6400030	13	62289714	T	T	T	A	T	T
2909	rs3655227	13	62306123	A	A	G	A	G	A
2910	rs6220659	13	62537414	G	G	G	A	G	G
2911	rs3678784	13	62597536	A	A	A	C	A	A
2912	rs3697016	13	62715709	G	G	G	C	G	C
2913	rs3668551	13	63044821	A	G	A	A	G	A
2914	rs3720797	13	63102465	G	G	A	G	G	G
2915	rs3657887	13	63277769	A	A	A	C	A	A
2916	rs3712144	13	63566794	G	G	G	A	G	G
2917	rs4229817	13	63888326	G	A	A	G	A	A
2918	rs4229816	13	63888358	G	G	G	G	G	G
2919	rs3693146	13	63944904	A	C	C	C	C	C
2920	rs6283060	13	63968307	G	G	G	A	G	G
2921	rs4229832	13	64004857	G	G	G	G	G	G
2922	rs4229818	13	64005079	A	A	A	G	A	A
2923	rs3670744	13	64048620	G	A	A	G	A	A
2924	rs4229851	13	64464356	G	G	G	G	G	G
2925	rs6273313	13	64487041	A	A	A	G	A	A
2926	rs3664897	13	64504305	C	A	C	A	C	A
2927	rs4137234	13	64547881	G	A	G	G	G	A
2928	rs6188260	13	65249689	G	G	G	G	G	G
2929	rs3662436	13	65251984	G	G	G	A	G	G
2930	rs3714056	13	65306908	A	A	A	G	A	A
2931	rs6283823	13	65381311	T	T	T	A	T	T
2932	rs3670719	13	65519227	G	G	G	A	G	G
2933	rs3718727	13	65760534	A	G	A	G	A	G
2934	rs6367778	13	65798352	G	G	G	A	G	G
2935	rs3683418	13	65931669	G	G	G	A	G	G
2936	rs6239735	13	66052637	G	G	G	A	G	G
2937	rs3672253	13	66166107	C	G	G	G	G	G
2938	rs3672334	13	66166143	A	A	A	G	A	A
2939	rs4229863	13	66254820	T	T	T	T	T	T
2940	rs4229866	13	66255048	C	C	C	A	C	C
2941	rs3694562	13	66407776	A	A	A	G	A	A
2942	rs3710071	13	66430679	A	A	A	G	A	A
2943	rs3657699	13	66449203	G	G	G	A	G	G
2944	rs6280864	13	66517595	A	A	A	G	A	A
2945	rs6360631	13	66606181	G	G	A	A	A	G
2946	rs4229876	13	66645425	A	A	A	A	A	A
2947	mCV23964641	13	63868572	C	C	C	C	C	C
2948	rs6303725	13	67209961	C	C	C	C	C	C
2949	rs3699272	13	67801964	A	A	A	G	A	A
2950	rs3691609	13	67815260	A	A	A	G	A	A
2951	rs3089497	13	67921466	G	G	G	G	G	G
2952	rs6179438	13	68057256	A	A	G	A	A	A
2953	rs3658685	13	68140298	G	G	A	G	G	G
2954	mCV25299689	13	66142640	G	G	G	A	G	G
2955	mCV25299687	13	66147365	G	G	G	A	G	G
2956	mCV25299416	13	66147534	A	A	A	G	A	A
2957	mCV25299415	13	66147589	A	A	A	C	A	A

2958	mCV25299414	13	66151042	G	G	G	A	G	G
2959	mCV25299404	13	66151134	G	G	G	A	G	G
2960	mCV25299402	13	66152141	C	C	C	G	C	C
2961	mCV25299392	13	66152463	A	G	G	G	G	G
2962	mCV25299391	13	66154029	G	G	G	A	G	G
2963	mCV25299390	13	66154192	A	A	A	C	A	A
2964	mCV25299380	13	66154230	G	G	G	A	G	G
2965	mCV25299379	13	66154257	A	A	A	C	A	A
2966	mCV24560966	13	66155995	G	A	A	G	A	A
2967	mCV24560976	13	66158821	A	G	G	A	G	G
2968	mCV24560977	13	66158934	A	A	A	G	A	A
2969	mCV24560978	13	66159036	G	G	G	A	G	G
2970	mCV24560988	13	66159180	A	A	A	G	A	A
2971	mCV24561260	13	66160363	A	G	G	A	G	G
2972	mCV24561261	13	66160441	C	A	A	C	A	A
2973	mCV24561262	13	66160594	A	G	G	A	G	G
2974	mCV24561272	13	66160629	C	C	C	A	C	C
2975	mCV24561285	13	66160856	G	A	A	G	A	A
2976	mCV24561286	13	66160884	G	G	G	A	G	G
2977	mCV24561295	13	66160961	A	C	C	A	C	C
2978	mCV24561297	13	66161425	A	G	G	A	G	G
2979	mCV24561308	13	66161560	G	A	A	G	A	A
2980	mCV22672456	13	66162780	A	G	G	A	G	G
2981	mCV22672466	13	66163028	A	G	G	A	G	G
2982	mCV22672467	13	66163054	A	G	G	A	G	G
2983	mCV22672468	13	66163236	G	C	C	G	C	C
2984	mCV22672485	13	66164053	G	C	C	G	C	C
2985	mCV22672486	13	69069214	C	A	A	C	A	A
2986	rs3693854	13	68170811	A	A	G	A	A	A
2987	rs3694554	13	68170968	A	A	G	A	A	A
2988	mCV22672493	13	66164564	A	G	G	A	G	G
2989	mCV22672494	13	66164662	G	G	G	A	G	G
2990	mCV22672495	13	66164665	A/G	A	A	G	A	A
2991	mCV22672502	13	66164765	G	G	G	A	G	G
2992	mCV22672517	13	66165091	G	G	G	A	G	G
2993	mCV22672518	13	66165247	G	G	G	A	G	G
2994	mCV22672519	13	66165275	G	G	G	A	G	G
2995	mCV22672528	13	66165298	C	C	C	A	C	C
2996	mCV22672715	13	66167189	G	G	G	A	G	G
2997	mCV22672716	13	66167295	G	G	G	A	G	G
2998	mCV22672727	13	66167989	A	A	A	T	A	A
2999	mCV22672728	13	66168361	G	G	G	C	G	G
3000	mCV22672738	13	66168543	A	G	G	G	G	G
3001	mCV22672739	13	66168837	A	G	G	G	G	G
3002	mCV22672750	13	66169845	C	A	A	A	A	A
3003	mCV22672752	13	66171934	A	G	A	G	A	G
3004	mCV22672762	13	66172053	A	A	G	G	G	A
3005	mCV22672763	13	66172061	A	A	A	G	A	A
3006	mCV22672764	13	66172103	A	G	G	G	G	G
3007	mCV22672774	13	66172955	G	G	G	A	G	G
3008	mCV22672775	13	66173062	A	A	A	T	A	A
3009	mCV22672973	13	66174600	G	G	A	G	A	G
3010	mCV22672974	13	66175026	G	G	G	A	G	G
3011	mCV22672975	13	66175162	A	A	A	G	A	A
3012	mCV22672985	13	66175183	G	G	G	A	G	G
3013	mCV22672997	13	66177903	A	G	G	A	G	G
3014	mCV22672998	13	66178014	A	G	G	A	G	G
3015	mCV24561309	13	66178845	G	A	A	G	A	A
3016	mCV24561331	13	66180479	A	C	C	A	C	C
3017	mCV22674574	13	66385617	G	A	A	G	A	A
3018	rs6346432	13	68550527	C	A	A	C	C	A
3019	rs3089535	13	68774221	G	G	G	G	G	G
3020	rs3722203	13	69035227	A	C	A	A	A	C
3021	rs4168094	13	69069571	A	G	G	A	A	G
3022	rs4168093	13	69069915	A	G	A	A	A	G
3023	rs3688781	13	69295263	G	A	A	G	G	A
3024	rs6395328	13	69674890	G	A	A	A	A	A
3025	rs3680359	13	69696881	G	A	A	A	A	A
3026	mCV24567210	13	68425157	A	T	T	A	A	T
3027	rs3721965	13	70436704	G	G	A	G	G	G
3028	rs4229899	13	70484661	G	G	G	G	G	G
3029	rs3090115	13	70662003	C	C	C	C	C	C
3030	rs4229913	13	70664876	G	G	G	G	G	G
3031	rs3680075	13	70797458	A	A	A	G	G	A

3032	rs6245426	13	70799676	C	C	C	C	C	C
3033	rs4229918	13	70809477	C	A	C	C	C	A
3034	rs8279849	13	71104261	G	G	G	G	G	G
3035	rs8279887	13	71114919	A	A	A	A	A	A
3036	rs8279885	13	71121946	G	G	G	G	G	G
3037	rs6291277	13	71254316	G	G	G	A	A	G
3038	rs3723159	13	71286539	G	G	G	A	A	G
3039	rs3670050	13	71297079	G	G	G	C	C	G
3040	rs8274705	13	71554604	A	A	A	G	G	A
3041	rs8267226	13	71572331	A	A	A	A	A	A
3042	rs3674407	13	72230763	A	A	A	G	A	A
3043	rs6227698	13	72227701	A	A	A	G	A	A
3044	rs3703582	13	72324250	A	A	A	G	A	A
3045	rs6237484	13	72465694	G	G	G	A	G	G
3046	rs3673953	13	72510669	G	G	G	A	G	G
3047	rs3722699	13	72735590	G	G	G	A	G	G
3048	rs3716730	13	72770555	C	C	C	G	G	C
3049	rs3708256	13	73021885	A	A	A	G	A	A
3050	rs3681284	13	74470502	A	A	A	G	A	A
3051	rs3678579	13	74494750	G	G	G	A	G	G
3052	rs3724350	13	73699795	A	A	A	G	A	A
3053	rs6372380	13	73703912	G	G	G	A	G	G
3054	rs3679222	13	73989720	A	A	A	G	A	A
3055	mCV24582540	13	72881657	T	T	T	A	T	T
3056	rs6212230	13	74589200	G	A	G	A	G	A
3057	rs3677220	13	74616016	T	A	T	A	T	A
3058	rs3706924	13	74621387	G	G	A	G	A	G
3059	mCV23400160	13	73445492	C	A	C	C	A	A
3060	rs3688040	13	74718183	A	G	A	G	A	G
3061	rs3683740	13	75110557	G	G	A	G	A	G
3062	rs6248891	13	75238350	G	A	A	A	A	A
3063	rs3089521	13	75266424	A	A	A	A	A	A
3064	rs3697007	13	75548389	C	A	C	A	C	A
3065	rs6315553	13	75668989	A	A	G	A	G	A
3066	rs3705043	13	76009925	G	G	A	G	A	G
3067	rs6220666	13	76304682	C	C	C	C	C	C
3068	rs3713411	13	76413109	G	A	A	A	A	A
3069	rs6268498	13	76611652	A	A	A	A	A	A
3070	rs6222317	13	77099591	G	G	G	G	G	G
3071	rs3693887	13	77224812	G	G	A	G	A	G
3072	rs3705446	13	77327201	A	G	A	G	A	G
3073	rs3722797	13	77535890	C	A	C	A	C	A
3074	rs6357295	13	77657697	G	G	G	G	G	G
3075	rs6158872	13	78380467	G	G	G	G	G	G
3076	rs3686754	13	78463997	G	G	G	A	G	G
3077	rs3682670	13	78716907	G	G	G	A	G	G
3078	rs3726588	13	78728885	G	G	G	G	G	G
3079	rs3684993	13	79103786	A	C	A	C	A	A
3080	rs6195556	13	79116316	A	A	A	G	A	A
3081	rs3702812	13	79529017	G	G	G	A	G	G
3082	mCV24607819	13	78579358	A	G	A	A	A	A
3083	rs6212654	13	79781803	G	G	G	A	G	G
3084	rs6304752	13	81426036	G	G	A	G	A	A
3085	rs6321551	13	81453706	G	G	G	G	G	G
3086	rs3669221	13	80367567	A	G	A	G	A	A
3087	rs3719251	13	80399420	G	G	G	A	G	G
3088	rs3710483	13	80757207	A	G	A	G	A	A
3089	rs3674364	13	80943119	G	A	G	A	A	A
3090	rs3665771	13	81416102	G	A	A	A	A	A
3091	rs3716022	13	81699041	A	G	G	A	G	G
3092	rs6304393	13	81700173	A	C	C	A	C	C
3093	rs3714314	13	81721563	A	T	T	A	T	T
3094	rs3659653	13	81826486	C	A	A	C	A	A
3095	rs6245977	13	82197498	G	A	A	G	A	G
3096	mCV24625340	13	81332738	G	A	G	G	A	A
3097	rs3693027	13	82543444	A	A	A	G	A	A
3098	rs3678699	13	82859266	T	A	T	A	T	T
3099	rs6377863	13	83050327	T	T	T	T	T	T
3100	rs6378492	13	83050468	A	A	A	A	A	A
3101	rs3686443	13	83174585	A	C	A	C	A	C
3102	rs3668894	13	83176850	G	A	G	A	G	A
3103	rs6296621	13	83614426	A	A	A	A	A	T
3104	rs6177298	13	83746245	A	A	C	A	C	C
3105	rs6250834	13	84248141	G	G	G	A	G	G

3106	mCV23244638	13	83381156	G	A	G	G	G	A
3107	rs6290670	13	84600795	G	G	G	G	G	G
3108	mCV24423497	13	83833399	C	A	C	C	C	C
3109	rs6288318	13	85598382	A	A	A	A	A	A
3110	rs3685298	13	86080336	A	A	A	G	A	A
3111	rs6223431	13	86155637	A	A	A	C	A	A
3112	rs8256640	13	86198114	G	G	G	G	G	G
3113	rs3655061	13	86241392	A	A	G	G	G	G
3114	rs6353311	13	86436799	G	G	G	G	G	G
3115	mCV24394003	13	83946056	C	A	A	C	A	A
3116	rs3657904	13	86644738	A	T	T	T	T	T
3117	rs3711987	13	86734941	A	A	A	A	A	A
3118	rs6330373	13	86895733	A	A	A	A	A	A
3119	rs3665985	13	87090246	A	T	T	A	T	T
3120	mCV22765543	13	84700245	A	A	A	T	A	A
3121	rs3723645	13	87372256	A	G	G	A	G	A
3122	rs6396708	13	88054253	A	A	A	A	A	A
3123	rs3090244	13	88457036	A	A	A	A	A	A
3124	rs4229929	13	88462114	G	G	G	G	G	G
3125	rs6316213	13	88817196	A	G	G	G	A	A
3126	rs3090591	13	88919916	G	G	A	G	G	G
3127	rs3700592	13	89049961	C	C	C	C	C	C
3128	rs6281579	13	89116059	G	G	G	G	G	G
3129	rs4229947	13	89195540	A	A	A	A	A	A
3130	rs4229951	13	89195671	C	C	C	C	C	C
3131	rs3667936	13	89228545	G	G	A	G	G	G
3132	rs3660654	13	89444417	C	C	C	A	C	C
3133	rs3701970	13	89950154	G	G	G	A	G	G
3134	rs3088471	13	90090575	G	G	G	G	G	G
3135	rs6293159	13	90158795	G	G	G	G	G	G
3136	rs3719701	13	90463735	A	A	A	C	A	A
3137	rs6364017	13	90523270	A	A	A	A	A	A
3138	rs3697202	13	90525106	G	A	G	A	G	G
3139	rs4229968	13	90669935	G	G	G	G	G	G
3140	rs6268154	13	91751636	A	A	A	A	A	A
3141	rs4229973	13	92003955	A	A	A	A	A	A
3142	rs4229972	13	92004037	A	A	A	A	A	A
3143	rs4229970	13	92004194	G	G	G	G	G	G
3144	rs3023386	13	92287042	A	A	G	G	G	G
3145	rs4229998	13	92334391	C	C	C	C	C	C
3146	rs4230010	13	92433517	G	G	A	G	A	A
3147	rs4230017	13	92433844	C	C	C	C	C	C
3148	rs3690969	13	92508085	A	A	A	G	A	A
3149	rs3688344	13	92540561	C	C	C	G	C	C
3150	rs6304527	13	92748300	NONE	A	A	A	A	A
3151	rs3696278	13	93118849	T	A	T	A	T	T
3152	rs6260303	13	93235132	G	G	G	G	G	G
3153	rs6268913	13	93884972	A	A	A	A	A	A
3154	rs3662543	13	94591366	G	A	G	A	A	G
3155	rs6244059	13	94592492	C	C	C	C	C	C
3156	rs3673640	13	94621276	G	A	G	A	A	G
3157	rs4230018	13	94662358	G	G	G	G	G	G
3158	rs2020720	13	94690410	G	G	G	G	G	G
3159	rs3667356	13	94846583	G	A	G	A	A	G
3160	rs6241433	13	95127428	A	A	A	A	A	A
3161	rs3692326	13	95285255	G	G	G	C	C	G
3162	rs4135523	13	95781992	A	G	G	G	A	G
3163	rs3089073	13	95833496	C	C	C	C	C	C
3164	rs3706705	13	95944170	G	G	G	A	A	G
3165	rs3666983	13	96125826	G	G	G	A	G	G
3166	rs3088752	13	96707224	A	G	G	A	A	G
3167	rs3088753	13	96707258	G	G	G	G	G	G
3168	rs3703332	13	96732558	A	G	G	G	G	G
3169	rs6196633	13	96803410	A	A	A	A	A	A
3170	rs4230019	13	96883287	A	A	A	A	A	A
3171	rs4230027	13	97200857	G	G	C	C	G	C
3172	rs6192828	13	97583932	T	A	T	T	T	T
3173	mCV25103990	13	95396576	A	C	C	A	C	C
3174	rs3697911	13	97793542	A	A	T	T	T	T
3175	rs3714142	13	98019774	A	A	T	A	A	A
3176	rs3657160	13	98020136	G	G	A	G	G	G
3177	mCV23729185	13	96187387	G	A	A	G	A	A
3178	rs3702451	13	98846673	A	A	A	G	A	A
3179	rs3698307	13	98868566	A	A	A	G	A	A

3180	rs3711084	13	99036251	A	G	A	G	A	A
3181	rs6361976	13	99050056	G	G	G	G	G	G
3182	rs3690822	13	99203909	C	G	C	G	C	C
3183	rs3706962	13	99204294	A	G	A	G	A	A
3184	rs3665205	13	99210029	A	G	A	G	A	A
3185	rs6244347	13	99298959	G	G	G	G	G	G
3186	rs3655295	13	99397142	A	C	A	C	A	A
3187	rs3688959	13	99855781	G	G	G	A	G	G
3188	rs3705092	13	100020595	G	A	G	A	A	G
3189	rs3703132	13	100303739	G	G	G	A	A	G
3190	rs3705003	13	100833770	G	G	A	G	G	G
3191	rs3660412	13	101204106	A	A	G	A	A	A
3192	rs6341216	13	101540040	A	A	A	A	A	A
3193	rs3089152	13	101867025	C	C	C	C	C	C
3194	rs6298013	13	102514157	A	A	A	A	A	A
3195	rs3700495	13	103165238	T	T	T	A	T	T
3196	rs6337318	13	103440365	G	G	G	G	G	G
3197	rs3719887	13	103498757	G	G	G	A	G	G
3198	rs3678856	13	105521730	C	C	A	C	C	C
3199	rs3687057	13	103792288	A	A	A	G	A	G
3200	rs3692862	13	103988456	C	A	C	A	C	A
3201	rs4230039	13	104241493	T	T	T	T	T	T
3202	rs3679406	13	104451097	T	T	T	A	T	T
3203	rs6356070	13	104476044	G	G	G	G	G	G
3204	rs3678312	13	104801223	A	G	G	A	G	G
3205	rs3676344	13	104904828	A	C	C	C	C	C
3206	rs6337278	13	105147948	A	A	A	A	A	A
3207	rs3697753	13	105190213	C	C	C	A	C	C
3208	rs6306874	13	105868690	G	G	G	G	G	G
3209	rs3701614	13	106123894	C	A	A	C	A	A
3210	rs3681990	13	106468932	A	A	C	A	C	A
3211	rs3682619	13	106469035	A	A	T	A	T	A
3212	rs6324165	13	106557906	A	A	A	A	A	A
3213	rs6406875	13	106942423	A	A	A	A	A	A
3214	rs3660764	13	107347748	A	A	A	G	A	A
3215	rs6284766	13	107766653	A	A	A	A	A	A
3216	mCV24457033	13	105409171	C	C	C	A	C	C
3217	rs4230046	13	108358972	C	C	C	C	C	C
3218	rs4230050	13	108651347	A	A	A	A	A	A
3219	rs3668922	13	108704702	A	A	G	A	G	G
3220	rs3722415	13	108786768	A	A	G	A	G	G
3221	rs4222698	13	108816291	A	G	A	A	A	A
3222	rs6224742	13	108944165	A	A	A	A	A	A
3223	rs3090595	13	109443015	G	G	G	G	G	A
3224	rs4230072	13	109678103	G	A	G	G	G	A
3225	rs4230062	13	109678284	G	G	G	G	G	G
3226	rs3711004	13	109743953	A	A	A	G	A	A
3227	mCV24886326	13	107388334	A	G	G	A	A	G
3228	rs6277674	13	110101636	A	C	A	A	A	C
3229	rs4230103	13	110162088	A	A	A	A	A	A
3230	rs4230105	13	110162156	A	C	A	A	A	C
3231	rs6184735	13	110201540	G	G	G	A	G	G
3232	rs3023392	13	110663989	A	G	A	A	A	G
3233	rs3088544	13	110878417	G	G	G	G	G	G
3234	rs6329103	13	111900029	A	A	A	A	A	A
3235	rs4230108	13	112039479	G	G	G	G	G	G
3236	rs6316705	13	112888923	A	A	A	G	A	A
3237	rs3088696	13	113136546	G	A	G	G	G	G
3238	rs3658551	13	113211634	C	G	C	G	C	C
3239	rs3656762	13	113290130	C	C	C	A	C	C
3240	rs3657450	13	113290258	G	G	G	A	G	G
3241	rs3657414	13	113498433	C	G	C	G	C	G
3242	rs3708958	13	113621613	A	G	A	G	A	G
3243	rs6308995	13	113876215	G	A	G	G	G	G
3244	rs3089097	13	114010147	A	A	A	A	A	A
3245	rs6190884	13	114285431	A	G	A	A	A	A
3246	rs3659752	13	114469156	G	G	G	G	G	A
3247	rs3685525	13	114754518	A	G	A	G	A	G
3248	rs3676640	13	114819954	C	G	C	G	C	G
3249	rs3708633	13	115714989	A	G	A	A	A	G
3250	rs3717171	13	116065408	C	A	C	C	C	A
3251	rs3700781	13	116244235	A	G	A	G	A	G
3252	rs3675054	13	116386374	G	A	G	A	G	A
3253	rs6163712	13	116415524	G	A	G	A	G	A

3254	mCV24995296	13	115386703	C	A	C	C	C	A
3255	mCV24397011	13	121083641	G	A	G	A	G	A
3256	rs3157619	14	45354253	G	G	G	A	G	A
3257	rs3155343	14	46051225	C	C	C	G	C	C
3258	rs3155490	14	46113760	A	A	A	G	A	A
3259	rs3155532	14	46147873	T	T	T	T	T	T
3260	rs3701221	14	1392385	G	A	A	A	A	A
3261	rs6335460	14	1631502	G	A	A	A	A	A
3262	mCV22823923	14	7199542	A	A	A	A	A	A
3263	rs3710916	14	3818468	C	A	C	A	C	C
3264	rs3712828	14	3834607	A	G	A	G	A	A
3265	rs4230148	14	3890360	A	A	T	A	T	T
3266	rs4230174	14	4020807	C	C	C	C	C	C
3267	rs4230179	14	4231703	T	A	A	A	A	A
3268	rs3088788	14	5163382	G	G	G	G	G	G
3269	rs6314527	14	5206071	G	A	G	A	G	G
3270	rs3689508	14	5056273	T	A	T	A	T	T
3271	rs6322899	14	5945767	C	A	C	A	C	C
3272	rs3722946	14	5972840	G	A	G	A	G	G
3273	rs4224037	14	5193065	NONE	G	G	G	G	G
3274	rs3150398	14	7401248	A	G	A	G	A	G
3275	rs6216285	14	7406798	G	A	G	A	G	A
3276	rs3672461	14	7512933	G	A	G	A	G	G
3277	rs6209858	14	7634631	A	G	A	G	A	G
3278	rs3701693	14	7742476	G	A	G	A	G	G
3279	rs6352512	14	7805601	T	A	T	A	T	T
3280	rs2020746	14	7811476	G	G	G	G	G	G
3281	rs3660068	14	8300488	G	A	G	A	G	A
3282	rs3692295	14	8551876	A	G	A	G	A	G
3283	rs6372890	14	8630291	A	G	A	G	A	G
3284	rs6260786	14	8690102	G	A	G	A	G	A
3285	mCV25318929	14	11797878	A	G	A	G	A	G
3286	rs3687346	14	9419393	G	A	G	A	G	A
3287	rs3669812	14	9511120	A	G	A	G	A	G
3288	rs4135584	14	9534998	G	A	G	A	G	A
3289	rs6238270	14	9615248	A	G	A	G	A	G
3290	rs3676100	14	9916017	C	A	C	A	C	A
3291	rs3090818	14	10437204	A	G	A	G	A	G
3292	rs6158384	14	10700688	T	A	T	A	T	A
3293	rs4230185	14	10720045	G	A	G	A	G	A
3294	rs3662587	14	10723797	A	G	A	A	A	A
3295	rs6408107	14	10733122	A	G	A	G	A	G
3296	rs3663932	14	11083772	A	G	A	G	A	G
3297	rs3687734	14	11212297	A	T	A	A	A	A
3298	rs6253513	14	11413304	A	G	A	G	A	G
3299	rs3089460	14	11462511	G	G	G	G	G	G
3300	rs3698600	14	11672488	G	A	G	A	G	A
3301	rs3709444	14	11917608	A	C	A	C	A	C
3302	rs3668355	14	12039779	G	G	G	G	G	G
3303	rs3709839	14	12058026	G	A	G	A	G	A
3304	rs6393665	14	12343760	G	G	G	A	G	A
3305	rs3667005	14	12362340	A	G	A	G	A	G
3306	rs3719629	14	12874743	A	A	A	G	A	G
3307	rs4230192	14	12889440	G	G	G	G	G	G
3308	rs6294895	14	13087232	A	G	A	G	A	G
3309	rs3672910	14	13126901	T	A	T	A	T	A
3310	rs3725901	14	13354145	G	A	G	A	G	A
3311	mCV25245448	14	11979126	A	G	A	G	A	G
3312	mCV23857686	14	12055982	G	A	G	A	G	A
3313	rs3703111	14	13431501	A	G	A	G	A	G
3314	rs3653743	14	13764493	A	T	A	T	A	T
3315	rs3696385	14	14028895	G	A	G	A	G	A
3316	rs3686093	14	14064387	A	G	A	G	A	G
3317	rs6238607	14	14070911	G	A	G	A	G	A
3318	rs3678171	14	14316750	G	A	G	A	G	A
3319	rs3691434	14	14340550	A	G	A	G	A	G
3320	rs6413339	14	14709385	G	A	G	A	G	A
3321	rs6304332	14	14953078	A	G	A	G	A	G
3322	rs6316987	14	14953203	A	T	A	T	A	T
3323	rs3669652	14	15233317	A	G	A	G	A	G
3324	rs3663068	14	15403925	C	G	C	G	C	G
3325	rs3706433	14	15772934	A	G	A	G	A	G
3326	rs3687889	14	15973497	G	A	A	A	A	G
3327	rs6206888	14	16442172	T	T	T	T	T	T

3328	rs6201943	14	17277171	G	G	G	G	G	G
3329	rs4230197	14	17320789	G	G	G	G	G	G
3330	rs3696019	14	17329455	A	G	G	G	A	A
3331	rs4230205	14	17736333	A	A	A	A	A	A
3332	rs3719797	14	17912717	C	G	G	G	C	C
3333	rs3690631	14	18039858	A	C	C	C	A	A
3334	rs6364305	14	18180003	C	C	C	C	C	C
3335	rs4230209	14	18272049	A	G	A	G	A	A
3336	rs3683585	14	18655239	A	C	A	C	A	A
3337	rs3706988	14	18705106	G	A	G	A	G	G
3338	rs6255409	14	18901784	G	G	G	G	G	G
3339	rs6395691	14	19311842	A	A	A	A	A	A
3340	mCV25031515	14	20803715	C	C	C	C	C	C
3341	rs3666933	14	19944914	G	G	G	G	G	G
3342	rs3692121	14	20092583	G	A	A	A	A	G
3343	rs6159306	14	20137823	C	A	A	A	A	C
3344	rs4230220	14	20597651	G	G	G	G	G	G
3345	rs4230217	14	20597784	A	A	A	A	A	A
3346	rs6336257	14	20887726	A	A	A	A	A	A
3347	rs6252078	14	21518064	A	A	A	A	A	A
3348	rs3682880	14	21635758	G	G	G	A	A	G
3349	rs6300900	14	22401046	A	A	A	A	A	A
3350	rs6323944	14	22939869	G	G	G	A	A	G
3351	rs4230230	14	22955528	G	G	G	A	A	G
3352	rs4230234	14	22955860	G	G	G	G	G	G
3353	rs3709400	14	23043000	A	A	A	G	G	A
3354	rs6399023	14	23142935	G	G	G	A	A	G
3355	rs3682024	14	23190762	G	G	G	A	A	G
3356	rs3697334	14	23407342	G	A	A	G	G	A
3357	rs3712886	14	23407595	A	G	G	A	A	G
3358	rs3663052	14	23438539	G	A	A	G	G	A
3359	rs3653376	14	23793812	G	A	A	G	G	A
3360	rs6396829	14	24042337	G	A	A	G	G	A
3361	rs3693175	14	24191935	G	G	G	A	A	G
3362	rs3658622	14	24451733	A	A	A	C	C	A
3363	rs3694569	14	24767060	G	G	G	A	A	G
3364	rs6244569	14	24946671	G	G	G	A	A	G
3365	rs3697466	14	25387014	C	C	C	A	A	C
3366	rs3720154	14	25388425	A	A	A	G	G	A
3367	rs3090249	14	25500482	A	A	A	A	A	A
3368	rs3662499	14	25549165	A	A	A	G	G	A
3369	rs3700898	14	25550835	C	C	C	G	G	C
3370	rs3692165	14	25658744	T	T	T	A	A	T
3371	rs6159786	14	25722967	T	T	T	A	A	T
3372	rs4230236	14	26076151	A	A	A	A	A	A
3373	rs4230239	14	26076242	A/G	G	G	G	G	G
3374	rs3709612	14	26381068	A	G	G	A	A	G
3375	rs6204667	14	26649815	A	G	G	A	A	G
3376	rs3678167	14	26920306	A	G	G	A	A	G
3377	rs3681994	14	26930317	G	A	A	G	G	A
3378	rs4230248	14	27218513	G	A	A	A	G	A
3379	rs6215014	14	27222493	G	A	A	A	G	A
3380	rs3661446	14	27519273	G	A	A	A	A	A
3381	rs4230249	14	27544435	G	G	G	G	G	G
3382	rs6313230	14	28306368	G	A	A	A	A	A
3383	rs3668823	14	28502233	A	G	G	G	G	G
3384	rs4230261	14	28880295	C	C	C	C	C	C
3385	rs3089138	14	29085318	A	A	A	A	A	A
3386	rs6248627	14	29163155	A	G	G	G	A	G
3387	rs6396413	14	29196132	G	A	A	A	G	A
3388	rs3719001	14	29373364	G	A	A	A	A	A
3389	rs3695383	14	29838823	A	G	G	G	A	G
3390	rs6209572	14	30117875	A	G	G	G	G	G
3391	rs4230265	14	30145682	G	G	G	G	G	G
3392	rs4230273	14	30145864	A/G	G	G	G	G	G
3393	rs3699386	14	30709023	A	G	G	G	G	G
3394	rs6335438	14	30990293	A	G	G	G	G	G
3395	rs4136381	14	31156721	G	A	A	A	A	A
3396	rs6303687	14	32384582	A	C	C	C	C	C
3397	rs3654283	14	32559577	A	G	G	G	G	G
3398	rs3722090	14	32565225	G	A	A	A	G	A
3399	rs4151922	14	32914232	G	G	G	G	G	G
3400	rs3702083	14	33145529	A	G	G	G	G	G
3401	rs3682128	14	33302372	A	C	C	C	C	C

3402	rs3701775	14	33391532	A	G	G	G	G	G
3403	rs3089928	14	33407037	A	A	A	A	A	A
3404	rs6256359	14	34144327	T	T	T	T	T	T
3405	mCV24456561	14	36005447	C	C	C	C	G	C
3406	rs6369028	14	34869678	A	A	A	A	A	A
3407	rs6329922	14	35903350	T	T	T	T	A	T
3408	rs6314716	14	36663352	G	G	G	G	A	G
3409	rs4230290	14	36960207	T	T	T	T	T	T
3410	rs8266325	14	37191379	G	G	G	G	A	G
3411	rs8266166	14	37238440	A	A	A	A	A	A
3412	rs8266165	14	37238559	G	G	G	G	G	G
3413	rs6397156	14	37477851	A	A	A	A	A	A
3414	rs4230294	14	38117147	C	C	C	C	C	C
3415	rs6169079	14	38214273	A	A	A	A	G	A
3416	rs3714840	14	38323598	C	C	C	A	C	C
3417	rs3689053	14	38368354	G	G	G	A	G	G
3418	rs3689402	14	38433817	G	G	G	A	A	G
3419	rs6175724	14	38738213	G	G	G	A	G	G
3420	rs3671357	14	38756818	A	A	A	G	G	A
3421	rs3685027	14	38971494	G	G	G	A	G	G
3422	rs3671603	14	39023864	A	A	A	C	A	A
3423	rs8236887	14	39045462	A	A	A	G	A	A
3424	rs8244064	14	39046026	A	A	A	G	A	A
3425	rs8236394	14	39046618	A	A	A	G	A	A
3426	rs8236888	14	39047096	C	C	C	G	C	C
3427	rs8236900	14	39053041	A	A	A	G	A	A
3428	rs3670380	14	39725953	G	G	G	A	G	G
3429	rs3659136	14	39862543	A	A	A	G	A	A
3430	rs6354338	14	39944436	A	A	A	G	A	A
3431	rs4230305	14	39961265	G	G	G	G	G	G
3432	rs6182700	14	40069959	T	T	T	A	T	T
3433	rs3699072	14	40156248	A	A	A	G	A	A
3434	rs3689993	14	40354993	C	C	C	G	C	C
3435	rs3657687	14	40504185	A	A	A	G	A	G
3436	rs6288476	14	40683873	C	C	C	A	C	C
3437	rs4230315	14	40926934	A	A	A	G	A	G
3438	rs4230319	14	40927091	C	C	C	C	C	C
3439	rs4135454	14	40989594	G	G	G	C	G	G
3440	rs8244142	14	41336853	G	G	G	A	G	G
3441	rs6373839	14	41560376	G	G	G	A	G	G
3442	rs3659150	14	42268201	G	G	G	G	G	G
3443	rs6278789	14	42466967	G	G	G	A	G	G
3444	rs3707497	14	42512837	G	G	G	A	G	A
3445	rs6374916	14	42824462	C	C	C	C	C	C
3446	rs3708709	14	43047330	G	G	G	A	G	G
3447	rs3687915	14	43183604	A	A	A	G	A	A
3448	mCV23384307	14	41762771	C	C	C	A	C	A
3449	rs4197422	14	43278805	A	A	A	G	A	G
3450	rs8244120	14	43620960	A	A	A	T	A	A
3451	rs3684504	14	43621534	A	A	A	G	A	A
3452	rs3724857	14	43793337	A	A	A	G	G	G
3453	mCV24777660	14	42906842	A	A	A	T	A	T
3454	rs3681670	14	44771183	G	G	G	A	G	NONE
3455	rs3680568	14	44876669	A	A	A	G	G	G
3456	rs3666583	14	44880408	A	A	A	G	A	A
3457	rs6258117	14	44911725	G	G	G	G	G	G
3458	rs3688559	14	45006165	A	A	A	G	A	A
3459	rs4230381	14	45052912	G	G	G	G	G	G
3460	rs3153444	14	46056409	T	T	T	A	T	T
3461	rs3725558	14	46220609	A	A	A	G	A	A
3462	rs3655501	14	46230668	G	G	G	A	G	G
3463	rs4230383	14	46341181	C	C	C	A	C	C
3464	rs4230384	14	46341187	C	C	C	C	C	C
3465	rs8244157	14	46590627	G	G	G	A	G	G
3466	rs3707560	14	46723061	G	G	G	A	G	G
3467	rs4222071	14	46879749	G	G	G	G	G	G
3468	rs3719196	14	46914470	G	G	G	A	G	G
3469	rs8251329	14	46937364	G	G	G	A	G	G
3470	rs8251323	14	46938331	G	G	G	G	G	G
3471	rs6216820	14	47120381	A	A	A	A	A	A
3472	rs6333066	14	47517607	A	A	A	A	A	A
3473	mCV23547823	14	46815038	A	A	A	C	A	A
3474	rs8244195	14	47102916	A	A	A	A	C	A
3475	rs6261451	14	48020869	A	A	A	A	G	A

3476	rs8244244	14	48021167	G	G	G	G	G	G
3477	rs8236919	14	48209352	A	A	A	A	G	A
3478	rs8251247	14	48210166	G	G	G	G	G	G
3479	rs8244273	14	48240598	C	C	C	C	A	C
3480	rs6356503	14	48485146	G	G	G	G	G	G
3481	rs3088661	14	48924826	A	A	A	A	G	A
3482	rs3023410	14	48924894	G	G	G	G	C	G
3483	rs3657883	14	49930376	T	T	T	T	T	T
3484	rs6376037	14	50266204	C	C	C	C	C	C
3485	rs3689948	14	50610267	A	A	G	A	G	A
3486	rs3695346	14	50614021	A	A	A/C	A	A/C	A
3487	rs3692863	14	50807707	A	G	G	A	G	A
3488	rs6280914	14	51226826	G	G	G	G	G	G
3489	rs6399403	14	51348575	G	G	G	G	G	G
3490	rs4230402	14	52297000	G	G	G	G	G	G
3491	rs3679870	14	52356020	G	A	G	G	G	G
3492	rs6207829	14	52357364	A	A	A	A	A	A
3493	rs3690953	14	52829746	A	G	G	A	G	A
3494	rs3708847	14	52897359	A	G	A	A	A	A
3495	rs6207322	14	53089767	G	G	G	G	G	G
3496	rs3710549	14	53185693	A	A	A	G	A	G
3497	rs3687710	14	53190824	A	A	A	C	A	C
3498	rs3658197	14	53313810	T	A	T	A	T	A
3499	rs3699179	14	53763412	A	A	A	G	A	A
3500	rs6245431	14	53807862	A	A	A	A	A	A
3501	mCV23132580	14	50594810	G	G	A	G	A	G
3502	rs4230406	14	54741084	A	A	A	A	A	A
3503	mCV23128760	14	51102719	A	A	G	A	G	A
3504	rs3666026	14	54756956	A	A	A	T	A	A
3505	rs3680253	14	54869217	G	A	G	G	G	A
3506	rs3703075	14	54913181	A	G	A	G	A	G
3507	rs6360386	14	54980518	A	G	A	G	A	G
3508	rs3090105	14	55063511	G	G	G	G	G	G
3509	rs6275800	14	55392769	C	C	C	C	C	C
3510	rs4230410	14	55960710	A	A	A	A	A	A
3511	rs3718193	14	56052977	A	G	A	G	A	G
3512	rs6392794	14	56065289	G	G	G	G	G	G
3513	rs3669959	14	56184698	G	G	G	A	G	G
3514	rs6161506	14	56250480	C	C	C	A	C	C
3515	rs3721918	14	56527076	G	G	G	A	G	G
3516	rs3700859	14	56728654	A	A	A	G	A	A
3517	rs6329390	14	57050768	A	A	A	A	A	A
3518	rs6255757	14	57183171	G	G	G	G	G	G
3519	rs4230419	14	57192697	G	G	G	G	G	G
3520	rs3697645	14	57347759	G	G	G	A	G	G
3521	rs3695574	14	57566391	A	A	A	G	A	A
3522	rs3696925	14	57566648	A	A	A	G	A	A
3523	rs4230420	14	57965520	G	G	G	G	G	G
3524	rs6330733	14	58054904	A	A	A	A	A	A
3525	rs8240011	14	58072553	G	G	G	G	G	G
3526	rs8240001	14	58093229	C	C	C	C	C	C
3527	rs8239993	14	58095389	A	A	A	A	A	A
3528	rs8239991	14	58095503	A	A	A	A	A	A
3529	rs3714353	14	58511050	A	A	A	C	A	A
3530	rs3654985	14	58517616	G	G	G	A	G	G
3531	mCV25372020	14	55550869	G	A	G	A	G	A
3532	mCV24914426	14	55783371	A	C	A	C	A	C
3533	rs6219411	14	58852403	A	A	A	A	A	A
3534	rs3712401	14	59239371	A	A	A	G	A	A
3535	rs3719672	14	59517387	A	A	A	C	A	A
3536	rs4230425	14	59731070	A	A	A	A	A	A
3537	rs4230426	14	59731209	A	A	A	A	A	A
3538	rs3090250	14	59788661	G	G	G	G	G	G
3539	rs3699699	14	59810769	A	A	A	G	A	A
3540	rs6309502	14	59826461	A	A	A	G	A	A
3541	rs3672425	14	60335045	A	C	A	A	A	C
3542	rs6360170	14	60654122	G	A	G	A	G	A
3543	rs3724660	14	60679103	G	A	G	A	G	A
3544	rs3677417	14	60925200	G	A	A	A	A	A
3545	rs3699418	14	61334173	A	G	G	G	G	G
3546	rs6242657	14	61724741	A	G	G	G	G	G
3547	rs4136556	14	62163288	A	G	G	G	G	G
3548	rs8266245	14	62298382	C	A	A	A	A	A
3549	rs8266250	14	62298737	G	G	G	G	G	G

3550	rs4230463	14	62380282	G	G	A	A	A	G
3551	rs6366173	14	62399064	G	G	A	A	A	A
3552	rs3695649	14	62965723	A	A	G	G	G	G
3553	rs3723413	14	63041573	G	G	G	A	G	G
3554	rs3697682	14	63513209	A	A	G	G	G	G
3555	rs6363872	14	63546489	C	C	G	G	G	G
3556	rs3657496	14	63656250	G	A	G	G	G	A
3557	rs6269487	14	64747403	C	C	C	A	C	C
3558	rs6377630	14	64753539	G	G	G	G	G	G
3559	rs4137600	14	64829224	NONE	A	G	A	G	A
3560	rs4230480	14	64949495	G	G	G	G	G	G
3561	rs3701623	14	64968413	G	A	G	A	G	A
3562	rs6351929	14	65324360	A	A	G	A	G	A
3563	rs3714110	14	65333234	G	A	G	A	G	A
3564	rs3718611	14	65454643	G	G	A	G	A	G
3565	rs3722734	14	66290004	G	A	A	A	A	A
3566	rs6346382	14	66397452	G	G	G	G	G	G
3567	rs4230487	14	66551180	A	A	A	A	A	A
3568	rs4230503	14	66360734	A	A	A	A	A	A
3569	rs3711616	14	66685220	A	C	A	C	A	C
3570	rs2020598	14	66840449	G	G	A	G	A	G
3571	rs6383423	14	67087043	A	A	A	A	A	A
3572	rs6300504	14	67399005	A	A	A	A	A	A
3573	rs3721365	14	68168875	C	A	A	A	A	A
3574	rs3656004	14	68313309	G	A	A	A	A	A
3575	rs3699310	14	68495170	G	A	G	G	G	G
3576	rs6192856	14	68906572	G	G	G	G	G	G
3577	rs3723391	14	69087437	A	G	A	A	A	A
3578	rs3674003	14	69394228	G	C	G	C	G	C
3579	rs6209200	14	69626933	A	T	A	A	A	A
3580	rs3089143	14	69658051	A	A	A	A	A	A
3581	rs6289135	14	69936501	G	G	G	G	G	G
3582	rs3665356	14	70335842	A	A	A	G	A	A
3583	rs3660202	14	70341585	G	G	G	A	G	G
3584	rs6273755	14	70761487	G	G	G	G	NONE	G
3585	rs3706792	14	70763611	G	G	G	A	G	G
3586	rs6173292	14	70896876	G	G	G	A	A	G
3587	rs6352085	14	71454776	A	A	A	A	C	A
3588	rs3023413	14	71540901	A	A	A	A	G	A
3589	rs3023412	14	72111198	A	A	A	A	C	A
3590	rs6396864	14	72817636	G	G	G	G	G	G
3591	rs6399916	14	72979539	A	A	A	A	A	A
3592	rs6349283	14	74301196	G	G	G	G	G	G
3593	rs6317880	14	74515545	G	G	G	G	G	G
3594	rs3665803	14	75192472	A	G	G	G	G	G
3595	rs3676946	14	75195795	A	C	C	C	C	C
3596	rs6222560	14	75681226	T	A	A	A	T	A
3597	rs3674288	14	75870785	C	A	A	A	A	A
3598	rs6340422	14	76298692	A	T	T	T	T	T
3599	rs3709178	14	76536133	G	A	A	A	A	A
3600	rs3699140	14	76696081	A	G	G	G	G	G
3601	rs6410417	14	76925644	G	A	A	A	A	A
3602	rs3718631	14	77163953	A	G	G	G	G	G
3603	rs3680118	14	78185123	G	A	A	G	A	A
3604	rs3668028	14	78279319	G	A	A	G	A	A
3605	rs3668114	14	78279363	G	A	A	G	A	A
3606	rs3663148	14	78682107	T	A	A	T	A	A
3607	rs3708535	14	78686463	C	A	A	C	A	A
3608	rs6326060	14	78777045	G	G	G	G	G	G
3609	rs3686226	14	78911167	A	G	G	A	G	G
3610	rs3712782	14	78977690	A	G	G	A	G	G
3611	rs6171492	14	79313186	G	A	A	G	A	A
3612	rs3680448	14	79368860	T	A	A	T	A	A
3613	rs6256136	14	79369256	T	A	A	T	A	A
3614	rs3676913	14	79942989	C	A	A	C	A	A
3615	rs3679493	14	80430045	A	G	G	A	G	G
3616	rs3709736	14	81396444	A	C	C	A	C	C
3617	rs3676856	14	81784749	G	G	G	G	G	G
3618	rs3670465	14	81902544	A	T	T	A	T	T
3619	rs3667933	14	81956876	A	G	G	A	G	G
3620	rs3725470	14	82281581	T	A	A	T	A	A
3621	rs6245063	14	82312893	G	G	G	G	G	G
3622	rs3717456	14	82650698	A	G	G	A	G	G
3623	rs6395984	14	82944063	C	A	A	C	C	A

3624	rs3661419	14	83327265	A	G	G	A	A	G
3625	rs3706761	14	83865561	A	G	G	A	A	G
3626	rs6176735	14	84102001	A	G	G	A	A	G
3627	rs6299927	14	84309814	G	A	A	G	G	A
3628	rs2020669	14	84407017	G	G	G	G	G	G
3629	rs2020662	14	84407256	A	C	C	A	A	C
3630	rs6309708	14	84466840	G	G	G	G	G	G
3631	rs4139735	14	84482998	G	A	A	G	G	A
3632	rs3724701	14	85334993	A	G	G	A	A	G
3633	rs6217888	14	85608884	G	G	G	G	G	G
3634	rs3718262	14	85692909	A	G	G	A	A	G
3635	rs3719266	14	85950983	A	G	G	A	G	G
3636	rs6393450	14	86058272	G	G	G	G	G	G
3637	rs3719489	14	86690000	A	C	C	A	C	C
3638	rs3701188	14	86910090	A	C	C	A	C	C
3639	rs3717398	14	86946266	A	G	G	A	G	G
3640	rs6220895	14	87134487	G	G	G	G	G	G
3641	rs3700154	14	87748115	G	A	A	G	A	A
3642	rs3685007	14	88257499	G	A	A	G	A	A
3643	rs6301349	14	88316888	A	G	G	A	G	G
3644	rs3679225	14	88401977	G	G	G	G	G	G
3645	rs3688189	14	88736539	A	G	G	A	G	G
3646	rs3704676	14	88736980	G	A	A	G	A	A
3647	rs3669193	14	88885503	C	A	A	C	A	A
3648	rs3684516	14	89120414	T	A	A	T	A	A
3649	rs4230511	14	90313713	G	G	G	G	G	G
3650	rs6257594	14	90562643	A	A	A	A	G	A
3651	rs6367718	14	91059903	C	C	C	C	C	C
3652	rs3670872	14	91831856	A	A	A	G	A	A
3653	rs3692362	14	92002849	C	C	C	G	C	C
3654	rs3680345	14	92006063	A	A	A	G	A	A
3655	rs6381585	14	92009360	C	C	C	C	C	C
3656	rs4230521	14	92097946	G	G	G	G	G	G
3657	rs3691209	14	92167911	C	A	A	C	A	A
3658	rs6211108	14	92402086	C	C	C	C	C	C
3659	rs6191117	14	92434091	A	G	G	G	G	G
3660	rs3670620	14	92501474	G	G	G	C	G	G
3661	rs3717612	14	92507339	G	G	G	A	G	G
3662	rs3708665	14	93083576	A	A	A	C	A	A
3663	rs3696439	14	93448908	A	A	A	G	A	A
3664	rs6179144	14	93467984	G	G	G	A	G	G
3665	rs6220962	14	93921872	A	A	A	A	A	A
3666	rs4135519	14	94104521	A	G	G	G	G	G
3667	rs3090823	14	94334437	A	A	A	A	A	A
3668	rs6319132	14	94738657	A	A	A	A	A	A
3669	rs4230523	14	95358761	A	A	A	A	A	A
3670	rs3681573	14	95364541	A	G	G	G	G	G
3671	rs3660029	14	95631189	G	A	A	A	A	G
3672	rs6305011	14	96039333	G	G	G	G	G	G
3673	rs4230537	14	96064601	A	A	A	A	A	A
3674	rs3703652	14	96408133	A	C	C	C	C	C
3675	rs6339304	14	96608916	G	G	G	G	G	G
3676	rs4230545	14	96725292	A	G	G	G	G	A
3677	rs3677141	14	97072936	G	A	A	A	A	A
3678	rs6258968	14	97115826	A	C	C	C	C	C
3679	rs4230548	14	97358405	A	A	A	A	A	A
3680	rs3088472	14	97653229	A	A	A	A	A	A
3681	rs6403004	14	98207644	G	A	A	A	A	A
3682	rs6292427	14	98574851	G	G	G	G	G	G
3683	rs3654132	14	98578208	A	G	G	A	G	G
3684	rs3678059	14	98713773	C	C	C	A	C	A
3685	rs3090772	14	98715241	G	G	G	G	G	G
3686	rs3702122	14	98720694	A	A	A	C	A	C
3687	rs3691899	14	98792413	C	C	C	A	A	A
3688	rs6156629	14	98810473	A	A	A	A	A	A
3689	rs3707149	14	99118700	A	G	G	G	G	G
3690	rs4230566	14	99335525	A	A	A	A	A	A
3691	rs3682169	14	99535523	A	G	G	G	A	G
3692	rs6246435	14	99855838	G	G	G	G	G	G
3693	rs3680792	14	100284919	A	G	G	G	A	G
3694	rs6211694	14	100975505	C	C	C	C	C	C
3695	rs6218311	14	101169889	G	G	G	G	G	A
3696	rs4139535	14	101872032	G	A	A	A	G	G
3697	rs6209981	14	101951056	T	A	A	A	T	T

3698	rs6169596	14	102767457	T	A	A	A	T	A
3699	rs3708779	14	103068000	G	A	A	A	G	A
3700	rs6336983	14	103924860	A	A	A	A	A	A
3701	rs3683181	14	103985865	A	A	G	G	G	A
3702	rs3726052	14	104244696	A	G	G	G	G	G
3703	rs3726642	14	104244789	C	A	A	A	A	A
3704	rs6399186	14	104466681	G	G	G	G	G	G
3705	rs3661551	14	104477641	G	G	A	A	A	G
3706	rs3683221	14	104867978	G	A	A	A	A	A
3707	rs6392366	14	104885073	G	G	G	G	G	G
3708	rs3672858	14	104887441	C	C	A	A	A	C
3709	rs6256423	14	106108556	G	G	G	G	G	A
3710	rs4230574	14	106631293	A	A	A	A	A	A
3711	rs6410408	14	106733464	A	A	A	A	A	A
3712	rs6226481	14	108194279	A	A	A	A	A	A
3713	rs3722416	14	108333902	G	G	G	A	G	G
3714	rs3658574	14	108647262	G	G	G	A	G	G
3715	rs6400296	14	109164992	C	C	C	C	C	C
3716	rs3654691	14	109194638	A	A	A	G	A	A
3717	rs3671398	14	109365819	A	A	A	G	A	A
3718	rs3090698	14	109444589	A	A	A	A	A	A
3719	rs6169105	14	109578622	G	G	G	A	G	G
3720	rs3090127	14	109998234	G	G	G	G	G	G
3721	rs6335025	14	110216466	G	A	A	A	A	A
3722	rs3721941	14	110342478	T	A	A	A	A	A
3723	rs4230594	14	110434211	G	A	A	A	A	A
3724	rs6365830	14	110830994	A	C	C	C	C	C
3725	rs3656556	14	110921454	A	G	G	A	G	G
3726	rs3665847	14	110951297	A	G	G	G	G	G
3727	rs4230596	14	111308626	A	A	A	C	A	A
3728	rs3665550	14	111354233	C	C	C	A	C	C
3729	rs3726296	14	111667485	T	A	A	A	A	A
3730	rs6237412	14	111854792	C	C	C	C	C	C
3731	rs3710901	14	111950778	A	A	A	T	A	A
3732	rs6177758	14	112148451	G	G	G	G	G	G
3733	rs3691815	14	112694348	G	C	C	C	G	C
3734	rs4230598	14	112766458	A	A	A	A	A	A
3735	rs3682013	14	113014012	G	A	A	G	G	G
3736	rs3682065	14	113014037	A	C	C	A	A	A
3737	rs6384035	14	113472061	G	G	G	G	A	G
3738	rs3690618	14	113588525	A	G	G	G	G	A
3739	rs3669586	14	113604821	A	G	G	A	A	A
3740	rs4230608	14	113621179	G	G	G	G	G	G
3741	rs4230609	14	113621259	A	G	G	A	A	A
3742	rs6166926	14	113663840	A	A	A	A	A	A
3743	rs3714594	14	113720943	C	A	A	C	C	C
3744	rs8236923	14	113799164	G	G	G	A	A	G
3745	rs8236924	14	113804663	G	G	G	G	G	G
3746	rs8255958	14	113811533	A	A	A	T	T	A
3747	rs8245390	14	113824164	G	A	A	A	A	G
3748	rs3698545	14	113996232	A	A	G	G	G	A
3749	rs6258782	14	114337175	A	A	A	A	A	A
3750	rs3654637	14	114723934	A	G	G	G	G	A
3751	rs4230617	14	114738214	G	G	G	G	G	G
3752	rs4230621	14	114916631	G	G	G	G	G	G
3753	rs3674353	14	115566681	G	G	A	A	A	G
3754	rs6229962	14	115619104	G	G	G	G	G	G
3755	rs8266870	15	99100827	G	G	G	G	G	G
3756	rs8277842	15	99254574	G	A	A	G	G	G
3757	rs3677296	15	40077809	G	A	G	A	A	G
3758	rs4231033	15	103783640	G	A	A	G	A	A
3759	rs8259436	15	75141907	A	A	A	G	G	G
3760	rs3724697	15	40072942	G	G	A	G	G	A
3761	rs6348185	15	52046695	A	A	C	A	C	C
3762	rs8266877	15	99101884	A	A	A	A	A	A
3763	rs3657139	15	86749385	G	G	A	G	G	G
3764	rs8279144	15	3145547	A	A	A	A	A	A
3765	rs8279140	15	3410749	C	C	C	C	C	C
3766	rs6361261	15	3580852	A	A	A	A	A	A
3767	rs3721852	15	3821767	A	G	G	G	A	A
3768	rs6274833	15	4403119	G	G	G	G	G	G
3769	rs8250965	15	5065079	G	G	G	G	G	G
3770	rs8243608	15	5064818	A	A	A	A	A	A
3771	rs8250970	15	5064104	G	G	G	G	G	G

3772	rs4230640	15	5058432	G	G	G	G	G	G
3773	rs4230630	15	5001955	A	A	A	A	A	A
3774	rs6208915	15	5343910	A	A	A	A	A	A
3775	rs3715491	15	5995303	A	A	G	A	A	G
3776	rs4230646	15	6263422	A	A	A	A	A	A
3777	rs4230647	15	6263531	A	A	A	A	A	A
3778	rs3707453	15	5737304	G	G	A	G	G	A
3779	rs3665826	15	5744460	G	G	A	G	G	A
3780	rs3695953	15	5804747	G	G	A	G	G	A
3781	rs6188822	15	7009617	G	G	G	G	G	G
3782	mCV25349597	15	10220320	T	A	T	T	T	A
3783	rs3693019	15	7948918	A	A	C	A	A	C
3784	rs3705269	15	7952392	G	G	C	G	G	C
3785	rs3088663	15	8348140	G	G	G	G	G	G
3786	rs6300414	15	8237742	G	G	G	G	G	G
3787	rs3656384	15	8972167	A	G	G	G	G	G
3788	rs6392458	15	9016966	T	T	T	T	T	T
3789	mCV23280545	15	12452418	C	C	A	A	A	C
3790	rs4139431	15	9409835	G	A	G	A	A	A
3791	rs3667918	15	9409417	T	A	T	A	A	A
3792	mCV24642661	15	13240133	A	G	A	G	A	G
3793	rs6190892	15	10378181	A	T	A	A	A	T
3794	rs3685370	15	10594037	G	A	A	A	A	A
3795	rs6176452	15	10599813	G	G	G	G	G	G
3796	rs3023415	15	10723878	A	A	G	G	G	A
3797	rs3658137	15	11255961	G	A	A	A	A	A
3798	rs6242228	15	12210991	A	G	G	G	G	G
3799	rs3088491	15	12340413	A	G	G	G	G	G
3800	rs3693764	15	12632698	A	G	G	G	G	G
3801	rs3681808	15	12947577	G	G	A	G	A	G
3802	rs4139555	15	12998398	G	G	A	G	A	G
3803	rs3656725	15	13227756	A	G	A	G	A	G
3804	rs3089730	15	13167109	A	A	A	A	A	A
3805	rs3711814	15	13122871	G	G	G	A	G	G
3806	rs6295031	15	13542177	G	G	G	G	G	G
3807	rs6410206	15	14889410	A	A	A	A	A	A
3808	mCV23663070	15	17469901	A	C	A	A	A	C
3809	rs6233580	15	15531975	A	A	A	A	A	A
3810	rs3676170	15	16292855	G	G	A	A	A	G
3811	rs6224924	15	15880169	G	G	G	G	G	G
3812	mCV23652351	15	18595358	NONE	A	C	C	C	A
3813	rs3665103	15	16726262	A	G	G	G	G	G
3814	rs6219745	15	16920338	A	G	G	G	G	G
3815	mCV23111231	15	19426941	A	G	A	A	G	G
3816	rs6197703	15	17718805	A	A	A	A	A	A
3817	rs4230664	15	18170092	A	G	G	G	G	G
3818	rs6168792	15	18374611	A	A	A	A	A	A
3819	rs3666368	15	18995366	G	C	C	C	C	C
3820	rs3680788	15	19114629	A	G	G	A	G	G
3821	rs3696823	15	19114916	A	A	A	G	A	A
3822	rs6409256	15	19167014	A	A	A	A	A	A
3823	rs3715857	15	19261351	G	A	A	G	A	A
3824	rs6215971	15	19854572	A	G	G	G	G	G
3825	rs6401788	15	20212813	G	C	C	C	C	C
3826	rs6267719	15	20154535	G	A	A	A	A	A
3827	rs3716253	15	20715725	A	G	G	G	G	G
3828	rs3677591	15	21180420	G	G	G	A	A	G
3829	rs6287380	15	21182442	C	C	C	C	C	C
3830	rs6208260	15	21368517	G	G	G	G	G	G
3831	rs3714169	15	21816884	G	G	A	A	G	G
3832	mCV23223429	15	24753097	G	G	G	A	G	G
3833	rs6260449	15	22251657	G	G	G	A	G	G
3834	rs3698831	15	22304391	C	G	C	G	C	G
3835	rs3671702	15	22498263	A	A	A	G	A	A
3836	rs6201636	15	22761863	G	G	G	G	G	G
3837	rs3697155	15	22792967	A	A	A	G	A	A
3838	rs6335389	15	23063828	A	A	A	A	A	A
3839	rs3674369	15	23245308	A	A	A	G	A	A
3840	rs3699876	15	23541427	C	C	A	C	C	C
3841	rs3719496	15	23653057	G	G	G	A	G	G
3842	mCV23271877	15	26486567	C	C	A	A	C	C
3843	rs6274937	15	23962499	A	C	C	A	A	C
3844	rs3661695	15	24798305	A	A	A	G	A	A
3845	rs6375813	15	25083118	G	G	G	G	G	G

3846	rs3654123	15	25474535	G	G	G	A	G	G
3847	rs6261669	15	25828932	A	A	A	A	A	A
3848	rs3662618	15	26094714	A	A	G	G	A	A
3849	rs6329292	15	26671912	C	C	C	C	C	C
3850	mCV23357315	15	30070515	G	C	G	G	G	C
3851	rs3670581	15	27108006	G	G	A	A	A	G
3852	rs3090071	15	27765234	A	A	A	A	A	A
3853	rs3711707	15	27843753	A	G	G	G	G	G
3854	rs6378494	15	28201562	G	G	G	G	G	G
3855	rs3702361	15	29090562	C	C	A	C	A	C
3856	rs6192378	15	29469122	A	A	A	A	A	A
3857	rs3088525	15	29671212	G	A	G	G	G	A
3858	rs6210607	15	30253148	C	A	A	A	A	A
3859	rs3683908	15	30394353	A	G	G	G	G	G
3860	rs6252277	15	30861774	A	T	T	T	T	T
3861	mCV23401463	15	34110939	A	A	G	G	G	A
3862	rs4230683	15	31006896	G	A	A	A	A	A
3863	rs3710212	15	31649437	A	G	G	G	G	G
3864	rs3679521	15	31740063	A	G	G	G	G	G
3865	rs3023416	15	31970597	G	G	A	A	A	A
3866	rs3693538	15	32067128	A	A	A	A	A	A
3867	rs6333696	15	32171906	G	A	G	A	G	A
3868	rs3708954	15	32205034	A	G	A	G	A	G
3869	rs3666257	15	32363127	A	G	A	G	A	G
3870	rs3666408	15	32363215	G	G	A	G	A	G
3871	rs3668136	15	32363470	G	G	A	G	A	G
3872	rs3661763	15	32368998	C	G	G	G	G	G
3873	rs3091174	15	32751064	A	G	A	G	A	G
3874	rs6325305	15	32616470	G	A	A	A	A	A
3875	rs8250895	15	33084415	G	A	A	A	A	A
3876	rs8236956	15	33084790	A	C	C	C	C	C
3877	rs8236958	15	33085113	G	G	G	G	G	G
3878	rs8238521	15	33085788	A	A	A	A	A	A
3879	rs8238522	15	33085834	A	A	A	A	A	A
3880	rs8267875	15	33134426	G	G	G	G	G	G
3881	rs8267890	15	33264485	G	A	A	A	A	A
3882	rs8267913	15	33301970	A	T	T	T	T	T
3883	rs8267917	15	33302195	G	A	G	A	G	A
3884	rs8267941	15	33518759	G	G	A	A	A	G
3885	rs8267966	15	33549693	A	A	C	A	C	A
3886	rs3687471	15	33568862	C	G	NONE	G	C	G
3887	rs6174755	15	33752351	A	T	T	T	T	T
3888	rs3704590	15	34026227	G	A	G	G	G	A
3889	rs8256260	15	34494889	A	G	G	G	G	G
3890	rs8250995	15	34495343	G	A	A	A	A	A
3891	mCV24802203	15	37430163	G	A	G	G	G	G
3892	rs6279676	15	34658250	G	A	A	A	A	A
3893	rs6214748	15	34875552	A	G	G	G	G	G
3894	rs3685951	15	35470919	G	A	A	A	A	A
3895	rs3089995	15	35670884	A	G	G	G	G	G
3896	rs6329380	15	35934138	A	G	G	G	G	G
3897	rs3705382	15	36996718	A	G	G	G	G	G
3898	rs6390021	15	36889042	A	G	G	G	G	G
3899	rs3090153	15	36956006	G	G	G	G	G	G
3900	rs4230705	15	36532608	A	A	A	A	A	A
3901	rs3705655	15	36340694	A	G	G	G	G	G
3902	rs3677366	15	37098131	C	A	A	A	A	A
3903	rs3090049	15	37117129	A	G	G	G	G	G
3904	rs3725816	15	38324623	G	G	A	A	A	G
3905	rs4230708	15	38372496	G	G	G	G	G	G
3906	rs3695416	15	38562542	C	A	C	A	A	A
3907	rs6331384	15	38576933	C	C	C	C	C	C
3908	rs6218280	15	39020401	A	A	A	A	A	A
3909	rs3660608	15	38958568	G	G	G	A	G	G
3910	rs3702837	15	39271679	A	G	G	G	G	G
3911	rs6226483	15	39511852	A	G	G	G	G	G
3912	rs3681810	15	39632711	G	A	A	A	A	A
3913	rs3692898	15	39894704	A	G	A	G	G	G
3914	rs6153804	15	40367963	G	A	A	A	A	A
3915	rs3680004	15	40735261	G	A	G	A	A	A
3916	rs3683326	15	41359817	C	C	A	C	C	C
3917	rs6316418	15	41575634	A	T	T	T	T	T
3918	rs3664170	15	41610813	G	A	A	A	A	A
3919	mCV23322919	15	44908031	A	A	A	T	A	A

3920	rs6313386	15	42481075	C	C	C	C	C	C
3921	rs3692586	15	42601022	A	C	C	A	C	C
3922	rs3716768	15	42604198	A	A	A	G	A	A
3923	rs3663316	15	42605189	A	G	G	G	G	G
3924	mCV23156669	15	45535477	A	G	G	A	G	G
3925	mCV23318468	15	45790702	A	T	A	A	A	A
3926	rs6303759	15	43433621	A	T	T	T	T	T
3927	rs4230712	15	43544902	G	G	G	G	G	G
3928	rs3682299	15	43553373	G	G	G	A	G	G
3929	rs3715565	15	44003431	A	G	G	G	A	G
3930	rs6188668	15	44059325	C	C	C	C	C	C
3931	rs3709561	15	44244702	A	A	G	A	A	G
3932	rs3674266	15	44244940	G	G	G	A	G	G
3933	rs3719612	15	44371925	A	G	G	G	A	G
3934	rs4230715	15	44397974	C	A	A	C	C	A
3935	rs6333177	15	44574079	A	A	A	A	A	A
3936	rs3719015	15	45104772	A	G	G	G	A	A
3937	rs6310537	15	45406964	A	A	A	A	A	A
3938	rs6329543	15	46139022	G	G	G	G	G	G
3939	mCV23302805	15	49367385	A	G	G	A	G	A
3940	rs4138760	15	46373890	A	G	G	G	G	G
3941	rs3660936	15	46899770	C	C	A	A	A	A
3942	rs4140120	15	46900061	A	A	C	C	C	C
3943	rs6258601	15	46956142	C	C	A	A	A	A
3944	rs6278499	15	47551528	A	A	A	A	A	A
3945	rs3659512	15	47929992	A	G	G	G	G	G
3946	rs6185746	15	48343654	G	G	G	G	G	G
3947	rs3666959	15	48823173	A	A	G	A	G	A
3948	rs6171444	15	48983802	C	C	C	C	C	C
3949	rs6362864	15	49425302	A	C	C	A	C	A
3950	rs3697897	15	49923310	NONE	G	G	C	G	C
3951	rs3723378	15	50038393	A	A	A	T	A	T
3952	rs6180405	15	50073041	A	A	A	C	A	C
3953	rs6310678	15	50243482	G	C	C	G	C	G
3954	rs6258673	15	50312644	A	G	G	A	G	A
3955	rs3706523	15	50547331	T	T	A	T	A	T
3956	rs3697419	15	51291649	G	G	G	A	G	A
3957	rs3665737	15	51348632	G	G	G	A	G	A
3958	rs3692040	15	51096014	C	C	A	C	A	C
3959	rs6187435	15	51670493	G	G	A	A	A	A
3960	rs3684635	15	51623465	G	G	G	A	G	A
3961	rs3658705	15	51403508	G	G	G	A	G	A
3962	rs6165881	15	51861047	A	A	G	A	G	G
3963	rs3090780	15	51878777	A	A	A	A	A	A
3964	rs3707900	15	52526197	A	A	G	A	G	G
3965	rs3667530	15	52656774	A	G	A	G	A	A
3966	rs6323414	15	52760158	G	G	G	G	G	G
3967	rs6162431	15	53341903	A	A	A	A	A	A
3968	rs3684672	15	54473969	G	G	A	A	G	G
3969	rs6366775	15	54701001	G	G	A	A	G	G
3970	rs3701428	15	55348476	G	G	A	A	G	G
3971	rs3701952	15	55348534	G	G	A	A	G	G
3972	rs3656215	15	55361701	G	G	A	A	G	G
3973	rs3090618	15	55430319	A	A	A	A	A	A
3974	rs6411953	15	55025287	G	G	A	A	G	G
3975	rs4222076	15	56418222	G	G	A	A	G	G
3976	rs4230727	15	55702669	G	G	G	G	G	G
3977	rs6154962	15	55621216	G	G	G	G	G	G
3978	rs6378118	15	57282732	C	C	C	C	C	C
3979	rs3702158	15	57352118	G	G	G	A	G	G
3980	rs6254309	15	58133510	C	C	C	C	C	C
3981	rs4230748	15	58354899	G	G	G	G	G	G
3982	rs3090252	15	58354953	C	C	C	C	C	C
3983	mCV24494343	15	61347770	A	G	A	G	G	G
3984	rs3695795	15	59005789	A	G	G	G	G	A
3985	rs3653368	15	58971277	A	A	G	A	A	A
3986	rs3697599	15	59372361	G	A	A	A	A	A
3987	rs4136487	15	59168111	G	A	G	A	A	A
3988	rs6354684	15	59578431	G	A	A	A	A	A
3989	rs6256501	15	60089318	A	G	A	G	G	A
3990	rs3701449	15	60283849	A	T	T	T	T	T
3991	rs3662901	15	60206134	A	A	G	A	A	G
3992	rs3654559	15	60169779	A	C	C	C	C	C
3993	rs6208973	15	61058598	C	G	G	G	G	G

3994	rs3683495	15	61406689	A	C	A	C	C	C
3995	rs3667658	15	61409229	G	A	G	A	A	A
3996	rs3668786	15	61418811	C	G	C	G	G	G
3997	rs3671795	15	61419330	A	G	A	G	G	G
3998	rs3090861	15	61445838	G	G	G	G	G	G
3999	rs3663595	15	61690932	A	A	G	A	A	A
4000	rs3668511	15	61758900	G	A	G	A	A	A
4001	rs3653568	15	61760204	A	G	A	G	G	G
4002	rs6215672	15	61779600	G	A	G	A	A	A
4003	rs3678001	15	61849908	A	A	C	A	NONE	A
4004	mCV22701834	15	64851000	A	G	A	A	A	A
4005	rs3658133	15	62073528	G	G	A	G	G	G
4006	rs4230764	15	62205151	A	A	A	A	A	A
4007	rs3654015	15	62479286	A	A	C	A	A	A
4008	rs6276604	15	62825138	G	G	G	G	G	G
4009	rs3692872	15	62864089	C	C	A	C	C	C
4010	rs4139476	15	63113390	G	G	A	G	A	G
4011	rs6295536	15	63152461	G	G	G	G	G	G
4012	rs3677062	15	63677792	G	A	A	G	A	G
4013	rs6366098	15	63699268	C	A	A	C	A	C
4014	rs3023419	15	63734238	T	T	A	T	T	T
4015	rs3699204	15	63737321	A	A	G	A	A	A
4016	mCV22937403	15	67462822	G	C	G	G	G	G
4017	rs6254661	15	64684884	A	G	G	A	A	A
4018	rs3662196	15	65137545	C	G	G	C	C	C
4019	rs3089203	15	65263601	G	G	G	G	G	G
4020	rs6306824	15	65379051	A	G	G	A	A	A
4021	rs3670563	15	65581635	G	G	A	G	G	G
4022	rs6381737	15	65856370	A	G	G	A	A	A
4023	rs3706861	15	66129820	C	C	G	C	C	C
4024	rs3685274	15	66669421	A	A	G	A	A	A
4025	rs6345802	15	67173023	A	A	A	A	A	A
4026	rs4230788	15	67200394	A	A	A	A	A	A
4027	rs4230789	15	67200496	G	G	G	G	G	G
4028	rs6197332	15	67276153	G	A	A	G	G	G
4029	mCV24506549	15	70345738	G	G	G	G	A	G
4030	rs4230790	15	67382019	A	A	A	A	A	A
4031	rs3726336	15	67392514	A	A	G	A	A	A
4032	rs3684996	15	68017710	G	G	A	G	G	G
4033	rs6213684	15	68114125	G	G	G	A	A	G
4034	rs3708614	15	69462082	C	C	A	C	A	A
4035	rs4230796	15	68487457	A	A	A	A	A	A
4036	rs6379157	15	68620907	C	C	G	C	G	C
4037	rs6363050	15	68704423	A	A	T	A	T	A
4038	rs4138576	15	68719828	G	G	G	G	G	G
4039	rs3670082	15	69347902	A	A	C	A	A	A
4040	mCV22728423	15	72312700	C	A	A	C	C	A
4041	rs6378719	15	69205581	G	G	G	G	G	G
4042	rs3089472	15	69907603	T	T	T	T	T	T
4043	rs6169611	15	70484965	G	A	A	G	A	A
4044	rs4230804	15	70496245	A	C	C	A	A	C
4045	rs6283580	15	71076029	A	A	A	A	A	A
4046	rs3721322	15	71261015	A	C	C	C	A	A
4047	rs3696311	15	71421315	A	A	A	G	A	A
4048	rs3696862	15	71421396	A	G	G	A	A	G
4049	rs3088560	15	71422176	A	A	A	C	A	A
4050	rs3699312	15	71605742	A	A	A	G	A	A
4051	rs3675203	15	71610697	A	A	A	C	A	A
4052	rs3090121	15	71846147	G	G	G	G	G	G
4053	rs6329940	15	71797146	T	T	T	A	A	A
4054	rs3088506	15	71793941	C	C	C	A	A	A
4055	rs6204870	15	71790221	G	G	G	A	A	A
4056	rs6336747	15	72189398	G	G	G	G	G	G
4057	rs3655952	15	72931171	C	A	A	C	C	C
4058	rs6320424	15	73211612	G	G	G	G	G	G
4059	rs4230806	15	73378194	A	A	A	A	A	A
4060	mCV22750087	15	77249727	A	C	NONE	A	A	C
4061	rs6283506	15	74282562	G	A	A	A	A	A
4062	rs6406086	15	74668646	C	G	G	G	G	G
4063	rs4230809	15	74880718	G	A	A	A	A	A
4064	rs3722684	15	75029080	G	A	A	A	A	A
4065	rs4230815	15	75039086	A	A	A	A	A	A
4066	rs4230816	15	75463148	A	G	G	A	A	A
4067	rs3662946	15	75264403	A	T	T	A	A	A

4068	rs6354330	15	76414792	A	A	A	A	A	A
4069	rs6213671	15	76138613	C	C	C	C	C	C
4070	rs3685365	15	77055395	C	G	G	C	C	G
4071	rs4138877	15	77383459	A	C	C	A	A	C
4072	rs3676322	15	77401658	A	G	G	A	A	G
4073	rs6236706	15	77534565	A	G	G	A	A	G
4074	rs3660192	15	78151819	A	G	G	G	G	G
4075	rs6342608	15	78487016	G	G	G	A	G	G
4076	rs3682805	15	78517557	G	G	G	A	A	G
4077	rs4140028	15	78518009	G	G	G	A	A	G
4078	rs3704608	15	78552232	A	A	A	G	G	A
4079	rs3701224	15	78678294	G	G	G	A	A	G
4080	rs4230846	15	79101619	C	C	C	C	C	C
4081	rs3667755	15	79237475	A	C	C	A	C	C
4082	rs3690902	15	79499628	A	A	A	G	A	A
4083	rs3698398	15	79717803	G	G	G	A	G	G
4084	rs3665030	15	79719777	A	G	G	A	G	G
4085	rs4230869	15	80000297	G	G	G	C	G	G
4086	rs6363099	15	80090603	A	A	A	A	A	A
4087	rs4230878	15	80243528	G	G	G	A	G	G
4088	rs3713989	15	80376203	A	A	A	G	G	A
4089	rs6410698	15	80519122	G	G	G	G	G	G
4090	rs3721372	15	80736406	A	A	A	G	A	A
4091	rs3686745	15	80754083	A	A	A	C	A	A
4092	rs6358242	15	81222239	A	A	A	A	A	A
4093	rs6276391	15	82164497	A	C	C	A	C	C
4094	rs3674050	15	82201743	A	G	G	A	G	A
4095	rs4230895	15	82334055	G	G	G	G	G	G
4096	rs8279290	15	82749090	G	G	G	G	G	G
4097	rs6211178	15	83169334	A	A	A	A	A	A
4098	rs4230909	15	83483973	C	A	A	A	C	A
4099	rs4230908	15	83484095	G	G	G	A	G	G
4100	rs6204958	15	83802520	C	C	C	C	C	C
4101	rs3672224	15	84016227	C	A	A	A	C	A
4102	rs6343605	15	84346068	A	A	A	A	A	A
4103	rs8274136	15	84442668	G	A	G	G	G	G
4104	rs8274177	15	84448213	A	A	A	A	A	A
4105	rs3725793	15	84730704	A	G	G	G	G	G
4106	rs3660367	15	85057715	A	G	G	A	A	A
4107	rs6357520	15	85493381	A	A	A	A	G	G
4108	rs6404768	15	85931986	G	G	G	G	G	G
4109	rs3683112	15	86054658	G	G	G	A	G	G
4110	rs8238200	15	86090930	A	A	A	G	A	A
4111	rs3681229	15	86126535	A	G	G	A	G	G
4112	rs4230930	15	86216095	A	G	G	A	A	G
4113	rs4230927	15	86216238	T	A	A	T	T	A
4114	rs3708719	15	86503322	G	G	G	A	A	G
4115	rs3697744	15	87041436	A	T	T	T	A	T
4116	rs6354372	15	87396669	G	G	G	G	G	G
4117	rs3682761	15	87767512	A	C	C	C	A	A
4118	rs3727135	15	63200433	A	A	C	A	C	A
4119	rs6367143	15	87893976	A	A	A	A	A	A
4120	rs3677860	15	88103720	A	G	G	G	G	G
4121	rs3710055	15	88344401	A	A	A	G	A	A
4122	rs3694472	15	88344070	A	A	A	G	G	G
4123	rs3724150	15	88603278	T	T	T	A	T	T
4124	rs3707587	15	88484731	C	C	C	A	C	C
4125	rs3091222	15	88440542	A	A	A	A	A	A
4126	rs6322712	15	88789727	G	G	G	G	G	G
4127	rs4230937	15	89214577	A	A	A	A	A	A
4128	rs6231305	15	89760644	G	G	G	G	G	G
4129	rs3720931	15	89891316	G	A	A	A	A	G
4130	rs3726626	15	90546529	A	A	A	A	A	A
4131	rs6353711	15	90568896	A	A	A	A	A	A
4132	rs4230974	15	90625600	G	G	G	G	G	G
4133	rs6374425	15	91025874	A	A	A	A	A	A
4134	rs3683067	15	91283809	G	G	G	G	G	G
4135	rs3717268	15	91324762	G	G	G	A	G	G
4136	rs6251635	15	92074429	G	G	G	G	G	G
4137	rs6173118	15	92329769	A	A	A	A	A	A
4138	rs3722990	15	92521445	A	A	A	G	A	A
4139	rs3716673	15	92579012	A	T	A	T	T	T
4140	rs3091079	15	92758941	A	A	A	A	A	A
4141	rs3658059	15	92970989	A	G	G	A	G	G

4142	rs3666934	15	93047270	A	A	A	G	A	A
4143	rs6246447	15	93048001	T	T	T	T	T	T
4144	rs3090254	15	93329367	G	G	G	G	G	G
4145	rs4230978	15	93924371	G	G	G	G	G	G
4146	rs3667785	15	93965130	G	A	A	G	A	A
4147	rs6163425	15	93998253	G	G	G	G	G	G
4148	rs4137261	15	94347872	G	G	G	A	G	G
4149	rs3685284	15	94612633	A	A	A	C	A	A
4150	rs3657431	15	94618365	A	A	A	G	A	A
4151	rs3667754	15	94638884	A	A	A	C	A	A
4152	rs6397043	15	94679858	G	G	G	G	G	G
4153	mCV23203552	15	97455253	G	A	A	G	G	G
4154	rs3719217	15	95679367	A	A	G	A	A	A
4155	rs3664692	15	95899565	A	A	A	G	A	A
4156	rs6336182	15	96345770	A	A	A	A	A	G
4157	rs3714765	15	96205484	G	G	G	A	G	G
4158	rs3712266	15	96205116	A	A	A	G	A	A
4159	rs6243011	15	96144834	A	A	A	A	A	A
4160	rs2020742	15	96489204	A	A	A	A	A	A
4161	rs3688273	15	96630244	G	G	A	A	G	A
4162	rs3660510	15	14500981	T	A	T	A	T	A
4163	rs3659240	15	14462019	G	A	G	A	G	A
4164	rs3089955	15	97198655	A	A	A	A	A	A
4165	rs3722513	15	97220735	A	A	A	G	A	G
4166	rs4137928	15	97377780	C	C	C	A	C	A
4167	mCV24679384	15	100437705	A	A	A	A	A	A
4168	rs6223057	15	97361752	A	A	A	A	A	A
4169	rs6157094	15	97502119	G	G	G	G	G	G
4170	rs3023427	15	98170960	A	A	A	G	G	G
4171	rs3710056	15	98027162	G	A	A	A	G	A
4172	rs4230983	15	99065701	A	A	A	A	A	A
4173	rs3708604	15	99587030	G	G	G	A	A	A
4174	rs3724301	15	99587247	G	G	G	A	A	A
4175	rs3657663	15	99751532	A	G	G	G	G	G
4176	rs2228905	15	99931008	A	A	A	A	A	A
4177	rs8243599	15	100049005	G	G	G	G	G	G
4178	rs3710400	15	99979161	G	G	G	G	G	G
4179	rs6284372	15	99970514	A	A	A	A	A	A
4180	rs4231011	15	99956969	T	T	T	T	T	T
4181	rs8277965	15	100181270	G	G	G	G	G	G
4182	rs8277942	15	100183726	G	G	G	G	G	G
4183	rs6313951	15	100853609	A	G	G	G	G	G
4184	rs6180802	15	100983246	T	T	T	T	T	T
4185	rs3703836	15	101045753	A	G	G	G	G	G
4186	rs3023430	15	101265090	G	G	G	A	A	A
4187	mCV24986357	15	102844147	G	G	G	G	A	A
4188	rs3690173	15	101419686	A	A	G	A	A	A
4189	rs3716382	15	101503749	G	G	A	G	G	G
4190	rs4231016	15	101758006	A	A	A	A	A	A
4191	rs8266829	15	102476882	G	G	G	G	G	G
4192	rs8278021	15	102479134	A	G	G	A	A	A
4193	rs8266850	15	102483342	G	G	G	G	G	G
4194	rs3665003	15	102312000	A	A	A	G	G	G
4195	rs4232432	15	104297014	G	A	A	G	G	G
4196	rs4231028	15	102816296	G	G	G	G	G	G
4197	rs3686133	15	102839135	G	A	A	A	G	G
4198	rs6248280	15	103154767	A	A	A	A	A	A
4199	rs8243680	15	103373057	G	G	G	G	G	G
4200	rs8243689	15	103373354	G	G	G	G	G	G
4201	rs6194670	15	103484696	A	A	A	A	A	A
4202	rs6313751	15	103963788	T	T	T	T	T	T
4203	rs8259830	15	104050107	G	G	G	G	G	G
4204	mCV25081968	16	5159604	C	G	C	C	C	C
4205	rs3090814	16	3369179	C	NONE	C	C	C	C
4206	rs4152031	16	3390342	G	A	G	G	G	G
4207	rs4152177	16	3494902	A	G	A	A	A	A
4208	rs4152477	16	3769762	G	G	G	G	A	G
4209	rs4152638	16	4009670	C	A	C	C	C	A
4210	rs4152662	16	4038800	A	G	A	A	A	A
4211	rs6366512	16	4090693	A	A	A	A	A	A
4212	rs6279796	16	4342086	A	A	A	A	A	A
4213	rs4153071	16	4796501	A	G	A	A	A	A
4214	rs4153455	16	5460178	A	G	A	G	A	A
4215	rs4153739	16	5572336	A	A	A	G	A	A

4216	rs3715939	16	5648501	A	A	A	C	A	A
4217	rs4153988	16	5659561	G	G	G	A	G	G
4218	rs4137339	16	5679591	A	G	A	G	A	A
4219	rs4231060	16	5907681	G	G	G	A	G	G
4220	rs4154990	16	6048671	G	C	G	G	G	G
4221	rs4155438	16	6232868	G	G	G	A	G	G
4222	rs3704351	16	6401450	A	A	A	G	A	A
4223	rs4155963	16	6441976	A	G	A	G	A	A
4224	rs4156029	16	6463662	A	G	A	A	A	A
4225	rs4157459	16	7042170	A	G	A	G	A	A
4226	rs6317732	16	6956400	C	C	C	C	C	C
4227	rs3722676	16	7018645	C	C	C	A	C	C
4228	rs4157579	16	7076593	C	C	C	A	C	C
4229	mCV24135489	16	9065553	G	A	A	A	A	A
4230	rs4158858	16	7624420	A	A	A	C	A	A
4231	mCV24137593	16	9382078	C	G	G	G	G	G
4232	rs4158669	16	7551144	A	A	A	G	A	A
4233	rs4159179	16	7898007	G	A	G	G	G	G
4234	rs4159502	16	8344201	G	A	G	G	G	G
4235	rs4159500	16	8342534	A	C	A	A	A	A
4236	mCV25079489	16	10392473	A	A	G	A	G	A
4237	rs6269373	16	8588636	A	A	A	A	A	A
4238	rs4160001	16	8746700	A	G	G	G	G	G
4239	rs4160175	16	9046289	G	A	A	A	A	A
4240	rs6214903	16	9327286	T	T	T	T	T	T
4241	rs4160502	16	9563944	G	A	G	G	G	G
4242	mCV24142461	16	11702908	G	G	G	G	G	G
4243	mCV24977246	16	11983431	G	A	A	A	A	A
4244	rs6400658	16	10299195	A	A	A	A	A	A
4245	rs4161204	16	10478118	G	G	A	G	A	G
4246	rs8247634	16	10480804	G	G	G	G	G	G
4247	rs8247631	16	10480879	G	G	G	G	G	G
4248	rs8247613	16	10482015	A	A	G	A	G	A
4249	rs4161987	16	11495439	A	G	G	G	G	G
4250	mCV23923315	16	11438937	G	G	A	G	A	G
4251	mCV24860404	16	11442136	G	G	A	G	A	G
4252	mCV24860403	16	11443214	A	A	C	A	C	A
4253	mCV24860402	16	11443464	A	A	G	A	G	A
4254	mCV24860380	16	13583636	G	G	A	G	A	G
4255	mCV24860047	16	13589855	G	G	A	G	A	G
4256	mCV24860024	16	11455935	A	A	G	A	G	A
4257	mCV24859728	16	13599753	T	T	A	T	A	T
4258	mCV24859714	16	13603718	G	G	G	G	G	G
4259	mCV24859704	16	13603915	T	T	A	T	A	T
4260	mCV24859691	16	11468568	A	A	C	A	C	A
4261	mCV24859373	16	13615843	A	A	T	A	T	A
4262	mCV24859372	16	13617549	G	G	A	G	A	G
4263	mCV24859361	16	11480824	T	T	A	T	A	T
4264	mCV24859360	16	13618676	G	G	A	G	A	G
4265	mCV24859314	16	13627029	G	G	C	G	C	G
4266	mCV24859313	16	13627964	G	G	A	G	A	G
4267	mCV25086629	16	13638633	G	G	A	G	A	G
4268	mCV25086630	16	13643062	A	A	G	A	G	A
4269	mCV25086642	16	13644853	A	A	G	A	G	A
4270	rs4162309	16	11810304	G	A	G	A	G	A
4271	rs6289275	16	12160073	A	A	A	A	A	A
4272	rs4162816	16	12222405	G	A	G	A	G	A
4273	mCV25090130	16	15165208	A	G	G	G	G	G
4274	rs4163391	16	13336999	A	A	G	A	G	A
4275	rs6389023	16	13434430	C	C	C	C	C	C
4276	rs4163522	16	13583499	A	A	C	A	C	A
4277	rs4163524	16	13584690	G	G	A	G	A	G
4278	rs4163525	16	13584746	G	G	A	G	A	G
4279	rs4163530	16	13585282	A	A	G	A	G	A
4280	rs4163531	16	13585948	G	G	A	G	A	G
4281	rs4163535	16	13587202	A	A	G	A	G	A
4282	rs4163536	16	13587541	G	G	A	G	A	G
4283	rs4163537	16	13588240	A	A	C	A	C	A
4284	rs4163540	16	13589681	A	A	G	A	G	A
4285	rs4163544	16	13408832	G	G	C	G	C	G
4286	rs4163549	16	13415606	A	A	G	A	G	A
4287	rs4163552	16	13599508	A	A	C	A	C	A
4288	rs4163554	16	13602647	A	A	C	A	C	A
4289	rs4163555	16	13602851	G	G	A	G	A	G

4290	rs4163557	16	13604441	A	A	T	A	T	A
4291	rs4163558	16	13604905	C	C	A	C	A	C
4292	rs4163560	16	13606893	G	G	A	G	A	G
4293	rs4163561	16	13606964	A	A	G	A	G	A
4294	rs4163563	16	13609071	A	A	G	A	G	A
4295	rs4163564	16	13609336	A	A	G	A	G	A
4296	rs4163565	16	13611038	G	G	A	G	A	G
4297	rs4163566	16	13613650	C	C	G	C	G	C
4298	rs4163570	16	13615134	A	A	G	A	G	A
4299	rs4163571	16	13615522	A	A	C	A	C	A
4300	rs6399033	16	13618752	A	A	A	A	A	A
4301	rs6400154	16	13618969	C	C	C	C	C	C
4302	rs4163580	16	13441064	A	A	G	A	G	A
4303	rs4163581	16	13623853	C	C	A	C	A	C
4304	rs4163588	16	13635521	A	A	C	A	C	A
4305	rs4163589	16	13635554	A	A	T	A	T	A
4306	rs4163590	16	13454476	C	C	A	C	A	C
4307	rs6218550	16	13642026	G	G	G	G	G	G
4308	rs4163594	16	13643729	A	A	G	A	G	A
4309	rs4163596	16	13645281	A	A	G	A	G	A
4310	rs3090019	16	13645514	A	A	C	A	C	A
4311	rs4163599	16	13646511	A	A	G	A	G	A
4312	rs4163600	16	13646717	G	G	A	G	A	G
4313	rs4163601	16	13646827	G	G	C	G	C	G
4314	rs8262762	16	14104647	C	C	C	C	C	C
4315	rs4164131	16	14121267	A	A	C	A	C	A
4316	rs3673183	16	14235488	A	A	G	A	G	A
4317	rs3714738	16	14439293	C	C	A	C	A	C
4318	rs4164872	16	15291555	A	G	G	G	G	G
4319	rs4164956	16	15549541	G	G	G	G	G	G
4320	rs4164966	16	15666087	A	G	G	G	G	G
4321	rs6392982	16	16863464	A	A	A	A	A	A
4322	rs8246557	16	17110007	A	A	A	A	A	A
4323	rs6178432	16	17256501	G	G	G	G	G	G
4324	mCV24708470	16	21170888	G	G	G	A	G	G
4325	mCV25133592	16	21911205	G	G	G	G	G	G
4326	rs4165233	16	19700960	A	G	A	A	A	A
4327	rs8264262	16	20137094	G	G	G	G	G	G
4328	rs8260417	16	20176415	G	G	G	G	G	G
4329	rs4165119	16	20271529	C	A	C	C	C	C
4330	rs4165123	16	20272798	A	NONE	A	A	A	A
4331	rs4231117	16	20277159	A	A	A	A	A	A
4332	rs4165089	16	19895572	G	G	G	G	G	G
4333	rs6268760	16	21070545	G	G	G	G	G	G
4334	mCV25141749	16	23519271	A	A	G	A	G	A
4335	mCV23928196	16	21862451	T	T	A	T	A	T
4336	mCV25142322	16	24203919	G	G	G	G	G	G
4337	rs4165301	16	22649634	A	A	G	A	G	A
4338	rs4174828	16	38664052	A	C	C	A	C	A
4339	rs6306333	16	22896033	A	A	A	A	A	A
4340	rs4174328	16	37931116	A	G	A	A	A	G
4341	rs4174278	16	23413634	G	A	G	A	G	A
4342	mCV25142331	16	24401446	G	A	G	G	G	G
4343	mCV23929196	16	26966102	G	G	G	G	G	G
4344	rs6204510	16	25318250	G	G	G	G	G	G
4345	rs4173196	16	35664946	A	A	A	C	A	A
4346	mCV23930218	16	27821402	G	A	G	A	G	A
4347	rs4172977	16	25587169	A	A	G	G	G	NONE
4348	rs4231205	16	35505002	G	G	G	G	G	G
4349	rs4172915	16	35504262	A	A	G	A	G	A
4350	rs4172779	16	35388554	G	G	G	A	G	G
4351	rs4172778	16	35388446	A	A	G	G	G	A
4352	mCV22602314	16	28698520	C	A	C	C	C	A
4353	mCV22555234	16	28878532	G	G	G	A	G	A
4354	rs4172185	16	34340336	G	A	G	A	G	A
4355	rs4165676	16	27339169	G	A	G	A	G	A
4356	rs6240629	16	27529763	C	A	C	A	C	A
4357	mCV24620624	16	27663359	A	A	G	G	G	A
4358	rs4166445	16	28119031	G	A	G	G	G	A
4359	mCV24708168	16	30382971	A	G	A	A	A	G
4360	rs6310468	16	28227854	A	A	A	A	A	A
4361	rs4167252	16	29012115	G	G	G	A	G	G
4362	rs6303559	16	29367928	A	A	A	A	A	A
4363	rs4167574	16	29521847	G	G	A	A	A	G

4364	rs4167865	16	29903421	G	G	A	A	A	G
4365	rs4167955	16	30157347	C	C	A	A	A	C
4366	rs6202375	16	30242408	G	G	G	G	G	G
4367	rs4168118	16	30288985	A	G	A	A	A	G
4368	mCV25020984	16	33349273	G	A	A	G	A	A
4369	rs4167921	16	30096753	A/C	NONE	C	C	C	A/C
4370	rs4169271	16	31481368	A	A	T	A	T	A
4371	rs3704997	16	31555093	G	G	A	G	A	G
4372	rs4167594	16	29587939	G	G	A	A	A	G
4373	rs6411352	16	31944788	G	G	A	G	A	G
4374	mCV25154621	16	34233339	G	A	G	A	G	A
4375	rs4170126	16	32098865	G	C	G	G	G	C
4376	rs3676973	16	32291976	A	G	G	A	G	G
4377	rs4167177	16	28837142	C	A	C	A	C	A
4378	rs4170507	16	32568890	A	A	G	A	G	A
4379	mCV23951787	16	34865535	A	A	G	A	G	A
4380	mCV23952101	16	32503299	A	A	A	A	A	A
4381	mCV24688032	16	34890184	A	A	T	A	T	A
4382	mCV24688031	16	34891202	G	G	A	G	A	G
4383	mCV24688027	16	32521226	G	A	G	A	G	A
4384	mCV24688026	16	32521267	A	A	G	A	G	A
4385	mCV24688020	16	34895178	T	T	A	T	A	T
4386	mCV24499316	16	34896520	A	A	C	A	C	A
4387	mCV24499996	16	34904371	A	A	A	A	NONE	A
4388	mCV24500003	16	34904482	G	G	G	G	G	G
4389	rs6269088	16	32688782	A	G	A	A	A	G
4390	mCV24500299	16	34912718	G	G	G	G	G	G
4391	mCV24500317	16	34914990	G	G	A	G	A	G
4392	mCV24500327	16	34917839	A	A	T	A	T	A
4393	mCV24500633	16	34926782	G	G	A	G	A	G
4394	mCV24500661	16	34931551	A	A	G	A	G	A
4395	mCV24500684	16	34932582	G	G	G	G	G	G
4396	mCV24500685	16	34933480	G	G	A	G	A	G
4397	mCV24500686	16	34935377	G	G	G	G	G	G
4398	rs4170974	16	32825259	A	C	C	A	C	C
4399	mCV23952166	16	35220991	A	G	A	A	A	G
4400	rs4166196	16	27709784	T	A	T	A	T	A
4401	rs3669767	16	27542767	G	C	G	C	G	C
4402	rs4165935	16	27539309	A	C	A	C	A	C
4403	rs3682563	16	27395910	A	G	A	G	A	G
4404	rs3682565	16	34084942	G	G	A	G	A	G
4405	rs4171932	16	34183107	G	A	G	A	G	A
4406	mCV25267427	16	37207323	G	C	C	G	C	G
4407	rs4172536	16	34863462	G	G	A	G	A	G
4408	rs4172537	16	34865150	G	G	A	G	A	G
4409	rs4172538	16	34865363	C	C	A	C	A	C
4410	rs4172540	16	34866225	A	A	G	A	G	A
4411	rs4172541	16	34866637	A	A	G	A	G	A
4412	rs4172542	16	34866726	A	A	G	A	G	A
4413	rs4172543	16	34866987	G	G	A	G	A	G
4414	rs4172544	16	34867146	A	A	G	A	G	A
4415	rs4172545	16	34867794	A	A	G	A	G	A
4416	rs4172549	16	34869987	C	C	C	C	C	C
4417	rs4172550	16	34872385	G	G	G	G	G	G
4418	rs4172551	16	34872662	G	G	A	G	A	G
4419	rs4172553	16	34878098	G	G	A	G	A	G
4420	rs4172554	16	34878125	A	A	T	A	T	A
4421	rs4172555	16	34888810	G	G	A	G	A	G
4422	rs4172556	16	34889709	A	A	G	A	G	A
4423	rs4172561	16	34894996	A	A	G	A	G	A
4424	rs4172566	16	34896422	A	A	G	A	G	A
4425	rs4172568	16	34896682	A	A	G	A	G	A
4426	rs4172572	16	34905982	G	G	A	G	A	G
4427	rs4172573	16	34907084	G	G	A	G	A	G
4428	rs4172574	16	34907452	A	A	C	A	C	A
4429	rs4172575	16	34907880	A	A	G	A	G	A
4430	rs4172576	16	34907881	A	A	G	A	G	A
4431	rs4172577	16	34910451	A	A	T	A	T	A
4432	rs4172578	16	34910632	G	C	G	C	G	C
4433	rs4172581	16	34912763	G	G	A	G	A	G
4434	rs4172582	16	34912876	A	A	T	A	T	A
4435	rs4172583	16	34912939	C	C	A	C	A	C
4436	rs4172584	16	34913289	G	G	A	G	A	G
4437	rs4172586	16	34916564	G	G	A	G	A	G

4438	rs4172587	16	34916823	A	A	G	A	G	A
4439	rs4172589	16	34917907	G	G	A	G	A	G
4440	rs4172591	16	34919444	C	C	A	C	A	C
4441	rs4172592	16	34920100	G	G	A	G	A	G
4442	rs4172594	16	34921604	A	A	G	A	G	A
4443	rs4172597	16	34925480	G	G	A	G	A	G
4444	rs4172600	16	34926009	G	G	A	G	A	G
4445	rs4172601	16	34926153	A	A	G	A	G	A
4446	rs4172603	16	34926884	C	G	G	G	G	G
4447	rs4172604	16	34928730	A	A	C	A	C	A
4448	rs4172607	16	34930392	G	G	C	G	C	G
4449	rs4172609	16	34932403	G	G	G	G	G	G
4450	rs4151957	16	34835446	G	G	G	G	G	G
4451	rs4172614	16	34939199	A	A	C	A	C	A
4452	rs4172615	16	34939382	A	A	G	A	G	A
4453	rs4172616	16	34939686	G	G	A	G	A	G
4454	rs4172617	16	34940283	G	G	A	G	A	G
4455	rs4172618	16	34940529	G	G	A	G	A	G
4456	rs4172619	16	34940566	G	G	A	G	A	G
4457	rs4172620	16	34945908	G	G	A	G	A	G
4458	mCV24597285	16	37718819	G	A	G	G	G	A
4459	rs3713911	16	35390375	T	T	T	A	T	T
4460	rs6182184	16	35525940	C	C	A	C	A	C
4461	rs4173052	16	35593001	A	A	G	A	G	A
4462	rs4173581	16	36085515	A	A	C	C	C	A
4463	rs4173858	16	36461158	G	G	A	G	A	G
4464	rs4165446	16	24194071	G	G	G	A	G	G
4465	rs4173889	16	37029092	G	A	A	G	A	G
4466	rs4165379	16	23562396	G	G	A	G	A	G
4467	rs4174320	16	37922093	G	G	G	C	G	G
4468	rs4174329	16	37932158	A	G	A	A	A	G
4469	rs4174445	16	38009487	G	A	G	G	G	A
4470	rs8257997	16	38048936	A	G	A	G	A	G
4471	rs8266690	16	38105131	G	G	G	G	G	G
4472	rs8266683	16	38105345	T	T	T	T	T	T
4473	rs4151928	16	38234549	C	G	C	G	C	G
4474	rs4174556	16	38282605	A	A	A	G	A	A
4475	rs4184015	16	38287860	A	G	A	G	G	A
4476	rs6393632	16	38441470	A	T	T	T	T	T
4477	rs4183784	16	47630353	C	G	C	G	G	G
4478	rs4175278	16	38874983	G	A	A	G	A	A
4479	rs4176333	16	39357023	A	G	G	A	G	G
4480	rs4176739	16	39655309	G	A	A	A	A	A
4481	mCV24996832	16	42098996	A	G	A	A	G	A
4482	rs6272186	16	40010527	T	A	A	A	A	A
4483	mCV23981852	16	42573263	A	G	A	A	G	A
4484	rs4181289	16	45495631	C	C	A	A	C	C
4485	rs4177651	16	40548715	A	G	A	G	G	A
4486	rs4177843	16	40649856	C	A	C	A	A	A
4487	rs4180773	16	45247290	A	A	A	G	A	A
4488	rs4180617	16	40877889	A	C	A	A	C	C
4489	rs4178232	16	41204697	C	C	C	G	C	C
4490	mCV25295325	16	44008435	T	A	A	A	A	A
4491	rs4179715	16	41725411	C	C	C	A	C	C
4492	mCV24627853	16	44560050	G	A	G	G	A	G
4493	rs4178975	16	41968873	A	G	A	A	G	G
4494	rs6238066	16	41986490	G	G	G	G	G	G
4495	rs6350435	16	42306986	G	G	G	G	G	G
4496	rs4179189	16	43401450	G	A	G	G	A	G
4497	rs3700978	16	43420279	A	G	A	A	G	A
4498	rs4179213	16	43469324	C	G	C	C	G	C
4499	rs4179226	16	43482236	G	A	G	G	A	G
4500	mCV22314190	16	46754869	G	A	G	A	A	G
4501	rs4179525	16	44160251	G	A	A	G	A	A
4502	rs3713966	16	44190062	A	G	G	A	G	G
4503	rs3663871	16	41553441	G	G	G	A	G	G
4504	rs6235507	16	44357850	G	G	G	G	G	G
4505	rs4179972	16	44416516	G	G	G	G	G	G
4506	mCV24002758	16	47453062	C	G	C	G	G	NONE
4507	rs4180198	16	44995920	C	C	C	A	C	C
4508	rs3683872	16	45016428	A	A	A	G	A	A
4509	rs3723465	16	45139003	A	A	A	G	A	A
4510	rs4177731	16	40572103	A	C	A	C	C	A
4511	rs4180943	16	45316519	G	G	A	A	G	G

4512	rs4181286	16	45494924	C	C	G	G	C	C
4513	rs6166655	16	45834712	A	A	G	A	A	A
4514	rs4182243	16	46008686	A	G	A	G	G	A
4515	mCV24006964	16	49194598	G	A	G	A	A	A
4516	rs4175670	16	46834401	C	G	G	C	G	G
4517	rs4175353	16	38902653	C	C	A	C	A	C
4518	rs3681038	16	38852853	A	G	G	A	G	G
4519	rs6238945	16	46845314	A	A	G	A	A	A
4520	rs4191391	16	59401514	G	G	A	A	G	G
4521	rs4183434	16	47142702	C	A	A	A	A	A
4522	mCV24009752	16	50117608	A	T	T	T	T	A
4523	rs3695839	16	47214540	G	A	G	A	A	A
4524	rs3682852	16	47217625	G	A	G	A	A	A
4525	rs4184240	16	48201421	G	A	G	A	A	A
4526	rs4189588	16	48656837	G	A	G	G	A	G
4527	mCV22565990	16	51371684	C	G	G	C	G	G
4528	rs4189244	16	49164403	G	G	A	A	G	A
4529	rs4184636	16	49286349	C	A	C	A	A	A
4530	rs4188825	16	56837969	G	A	G	G	A	A
4531	rs3655094	16	49685695	A	A	G	A	A	A
4532	rs6378040	16	49764473	G	G	A	G	G	G
4533	rs4185639	16	50209266	T	A	A	A	A	T
4534	rs6392667	16	50394916	A	A	G	A	A	A
4535	rs6317515	16	51012277	G	G	G	G	G	G
4536	rs4186353	16	51034616	C	A	A	C	A	A
4537	mCV24014260	16	54658355	G	G	G	G	G	G
4538	mCV24014967	16	55283802	A	T	T	T	T	T
4539	rs6267334	16	52277646	A	A	A	A	A	A
4540	rs6164817	16	53018300	G	G	G	G	G	G
4541	rs3688027	16	52746994	G	G	A	G	G	A
4542	rs6323896	16	53268570	A	A	G	A	A	G
4543	rs3702995	16	54057346	G	G	A	G	G	A
4544	rs4186939	16	51477443	C	G	G	C	G	G
4545	rs3687272	16	54658095	A	A	G	A	A	G
4546	rs3719157	16	51015659	G	A	A	G	A	A
4547	rs4187596	16	55549353	C	A	A	A	A	C
4548	rs4185336	16	49923287	A	G	G	G	G	A
4549	rs6271301	16	56191357	G	G	G	G	G	G
4550	rs4135604	16	49690572	C	A	C	A	A	A
4551	rs3690619	16	49339572	A	G	A	G	G	G
4552	rs4184685	16	49326695	G	A	G	A	A	A
4553	rs6372156	16	56592997	A	A	A	A	A	A
4554	rs4188545	16	56657953	G	G	A	A	G	A
4555	rs4188555	16	56662517	A	A	T	T	A	A
4556	rs3705921	16	49066582	C	A	C	A	A	A
4557	rs6230371	16	57366574	C	C	G	G	C	G
4558	rs3682166	16	57605776	G	G	G	A	G	G
4559	mCV23483721	16	60842443	G	A	G	G	A	G
4560	rs4189478	16	57732226	A	A	G	G	A	G
4561	rs4189488	16	57736912	A	A	G	G	A	G
4562	rs4192158	16	59914801	T	T	A	A	T	T
4563	rs3023435	16	58492725	A	G	A	A	G	G
4564	rs4190088	16	58492828	G	A	G	G	A	G
4565	rs4190470	16	58816553	A	G	A	A	G	G
4566	rs3023244	16	58937944	G	G	A	G	G	G
4567	rs6170261	16	59302156	G	G	A	A	G	G
4568	rs4191319	16	59356414	A	A	G	G	A	A
4569	mCV22518012	16	59879230	C	C	C	G	C	G
4570	rs6324074	16	59951151	A	T	T	A	T	T
4571	mCV24047247	16	63693630	A	A	A	G	A	G
4572	rs6292952	16	60766441	A	A	A	A	A	A
4573	rs4194624	16	63381090	A	A	A	G	A	G
4574	rs6191049	16	61522286	A	A	A	A	A	A
4575	rs4193194	16	61748509	A	A	A	T	A	T
4576	rs6246780	16	61792843	A	A	A	G	A	G
4577	mCV24666082	16	65120566	C	A	A	C	A	C
4578	rs4194213	16	62402521	A	A	A	G	A	G
4579	rs6341030	16	62114841	A	A	A	C	A	C
4580	rs6271608	16	62362660	C	C	C	C	C	C
4581	rs4194166	16	62377824	T	T	T	A	T	A
4582	rs3693435	16	62531264	G	G	G	C	G	G
4583	rs3675841	16	63307194	A	A	A	C	A	C
4584	rs4194670	16	63413889	G	G	G	A	G	A
4585	rs3687551	16	63841477	G	G	G	A	G	A

4586	rs4196573	16	65870790	C	C	C	A	C	A
4587	mCV22528126	16	67409333	A	G	G	A	G	G
4588	rs3675347	16	64035439	G	G	G	A	G	A
4589	rs6192780	16	64331247	G	G	G	A	G	A
4590	rs4197222	16	66614508	A	A	A	G	A	G
4591	rs4197265	16	66628728	G	G	G	A	G	A
4592	mCV24067077	16	68251776	G	A	A	G	A	G
4593	rs6251709	16	64864742	A	C	C	A	C	A
4594	rs4197634	16	67150805	G	G	G	A	G	A
4595	mCV24067730	16	68668611	G	C	C	G	C	C
4596	rs3693190	16	65622689	A	A	A	G	A	G
4597	rs6188665	16	65694286	A	A	A	G	A	G
4598	rs4198012	16	66481437	A	G	G	A	G	G
4599	rs4197150	16	66573423	G	G	G	C	G	C
4600	rs3673897	16	66604122	G	G	G	A	G	A
4601	rs3690033	16	66604407	G	G	G	C	G	C
4602	mCV23116957	16	70337916	A	A	A	C	A	A
4603	rs6375426	16	67097218	C	C	C	C	C	C
4604	rs6337165	16	69646244	C	C	C	C	C	C
4605	rs4199265	16	70091526	A	G	G	A	G	A
4606	rs4199313	16	70130456	A	G	G	A	G	A
4607	rs3695481	16	68172163	A	G	G	A	G	G
4608	rs6183224	16	68216382	C	A	A	C	A	A
4609	rs4198313	16	68385359	G	A	A	G	A	G
4610	rs4198354	16	68415912	A	A	A	A	A	A
4611	rs4198503	16	68513227	G	A	A	G	A	A
4612	rs4200381	16	71256187	A	A	A	T	A	A
4613	rs4198737	16	69301525	A	G	G	A	G	A
4614	rs4198912	16	69399222	A	G	G	A	G	A
4615	rs6223046	16	69549391	G	G	G	G	G	G
4616	rs4199279	16	70098199	A	G	G	A	G	A
4617	rs4201710	16	72678419	G	G	G	A	G	A
4618	rs3668777	16	70648699	T	T	T	A	T	T
4619	rs4199605	16	70682046	C	C	C	G	C	C
4620	rs4202239	16	73028404	G	G	G	A	G	G
4621	rs6280325	16	71105209	A	A	A	A	A	A
4622	rs4202837	16	73635230	C	C	A	A	A	A
4623	rs4200756	16	71507609	G	G	G	A	G	G
4624	rs6392872	16	71548069	A	A	A	A	A	A
4625	rs4203040	16	72209792	A	C	A	A	A	A
4626	rs4200913	16	71633618	G	G	G	A	G	G
4627	mCV24097201	16	72294606	A	C	A	A	C	A
4628	rs4203387	16	72441600	G	A	G	G	G	G
4629	rs4204213	16	74635142	A	A	A	G	A	A
4630	rs4201178	16	72338365	A	A	A	T	A	T
4631	rs4205362	16	75409933	A	C	A	A	C	A
4632	rs3710273	16	72956607	G	G	G	A	G	G
4633	rs3721202	16	73110160	A	A	A	T	A	A
4634	rs4205586	16	75963073	A	A	G	G	A	G
4635	mCV24705846	16	77599196	C	A	C	C	A	C
4636	rs4206363	16	76745925	T	A	T	T	A	T
4637	rs4203607	16	74269568	A	G	A	A	A	A
4638	rs6296788	16	74380193	A	A	A	A	A	A
4639	rs4204016	16	74511014	A	G	A	G	A	A
4640	rs4204017	16	74511918	A	A	A	T	A	A
4641	rs4204106	16	74547672	G	A	G	G	G	G
4642	rs3702474	16	74596958	A	C	A	C	A	A
4643	rs4135423	16	74745250	T	T	T	A	T	T
4644	rs4204682	16	75017568	G	G	G	A	G	G
4645	rs4204972	16	75165503	A	A	A	G	A	A
4646	rs3704648	16	75223291	A	A	A	G	A	A
4647	rs4205049	16	75234135	A	A	A	T	A	A
4648	rs4208073	16	76780682	A	G	A	A	G	G
4649	rs4208299	16	78728146	A	A	G	G	A	A
4650	rs4206327	16	76732911	G	G	C	C	G	C
4651	rs4206340	16	76739638	C	A	C	C	A	C
4652	rs3718160	16	77227010	A	A	C	C	A	C
4653	rs4206932	16	77372676	A	G	A	A	G	A
4654	rs4210182	16	80506732	G	G	A	A	G	G
4655	rs4210483	16	80733536	T	T	A	A	T	T
4656	rs4207939	16	78410075	G	A	A	A	A	A
4657	rs3685106	16	78701660	A	A	G	G	A	A
4658	rs3703235	16	78953738	A	A	G	G	A	A
4659	rs4208684	16	79017309	A	A	C	C	A	A

4660	rs6253229	16	79176635	G	G	A	A	G	G
4661	rs8269978	16	79319085	A	A	A	A	A	A
4662	rs4209296	16	79788451	G	A	G	G	A	A
4663	rs6317052	16	79873189	A	G	A	A	G	G
4664	rs4209716	16	80021082	G	A	G	G	A	A
4665	rs6277318	16	80131512	A	A	A	A	A	A
4666	rs4211580	16	83004243	G	G	G	A	G	G
4667	mCV22598580	16	84525962	G	G	G	A	G	G
4668	rs4210541	16	80752355	G	G	A	A	G	G
4669	rs3673156	16	80837451	G	G	A	A	G	G
4670	rs3693437	16	80854395	G	G	A	A	G	G
4671	rs4211783	16	84604484	A	A	A	C	A	A
4672	rs4211850	16	84666984	T	T	T	A	T	T
4673	rs6163781	16	82570740	A	A	A	A	A	A
4674	rs4212088	16	83612058	G	A	A	G	A	A
4675	mCV24707406	16	86831905	C	A	A	C	A	A
4676	rs6175321	16	83036185	G	G	G	A	G	G
4677	rs4212843	16	84538678	G	A	A	A	A	A
4678	rs4213285	16	86848205	T	A	A	T	A	A
4679	rs4213806	16	87453292	G	A	A	A	A	A
4680	rs4213876	16	87532305	G	G	G	A	A	G
4681	rs6193471	16	84822534	T	T	T	T	T	T
4682	mCV24131870	16	88997358	A	A	A	A	G	A
4683	rs3680665	16	84938923	C	C	C	G	C	C
4684	rs4214481	16	87974508	G	G	G	A	A	G
4685	rs4214515	16	87987764	A	G	G	G	G	G
4686	rs4212102	16	85479261	A	G	G	A	G	G
4687	rs4212186	16	85547887	A	G	G	A	G	G
4688	rs3693968	16	85680818	G	A	A	G	A	A
4689	rs4215067	16	88615411	A	G	G	G	A	G
4690	rs3724196	16	86336602	A	A	A	G	A	A
4691	rs6412407	16	86336950	C	C	C	C	C	C
4692	rs4212842	16	86413307	G	A	A	A	A	A
4693	rs4213071	16	86574244	C	C	C	A	C	C
4694	rs4217260	16	87711454	G	G	G	G	A	A
4695	rs3709512	16	86877439	A	G	G	A	G	G
4696	rs3656592	16	87019209	G	G	G	G	G	G
4697	mCV23505219	16	80910418	A	A	G	G	A	A
4698	rs3674782	16	87743655	G	A	A	G	G	A
4699	rs4214396	16	87920304	A	C	C	A	A	C
4700	rs4214683	16	88131565	A	A	A	A	G	A
4701	rs6231616	16	88285205	A	A	A	A	A	A
4702	rs4214876	16	88417690	A/G	A	A	A	A	A
4703	rs4214924	16	88462563	A	A	A	A	G	A
4704	rs4214927	16	88462890	G	G	G	G	A	G
4705	rs4151940	16	89077062	A	G	G	G	G	G
4706	rs4215932	16	89363885	G	A	A	A	G	A
4707	rs4216460	16	89791987	A	A	A	A	G	A
4708	rs4216475	16	89881415	A	C	C	C	A	C
4709	rs4216686	16	90020366	A	A	A	A	G	A
4710	rs4217297	16	90768465	A	G	G	G	A	A
4711	rs4219555	16	91815141	A	G	G	A	G	A
4712	rs6350538	16	91087963	G	A	A	A	G	A
4713	rs4218306	16	91297440	G	A	A	A	G	G
4714	mCV23083514	16	94883773	A	C	C	C	C	C
4715	rs4219040	16	91664240	C	C	C	A	C	C
4716	rs4217722	16	91044215	A	C	C	C	A	C
4717	rs4219239	16	92134520	A	G	G	A	G	A
4718	rs4217339	16	90796276	G	A	A	A	G	G
4719	mCV24560371	16	96083785	A	G	G	G	G	A
4720	rs8273839	16	98411195	C	C	C	C	C	C
4721	mCV25389795	16	96645024	G	G	G	G	G	G
4722	rs4221562	16	94186146	G	G	G	A	G	G
4723	rs6244229	16	93114728	G	G	G	G	G	G
4724	rs4219905	16	93471889	G	G	G	A	G	G
4725	rs6220581	16	93605148	A	A	A	A	A	A
4726	rs3664190	16	93793383	A	A	A	G	A	A
4727	rs3664755	16	93793468	T	T	T	A	T	T
4728	rs6233949	16	93869618	C	C	C	A	C	C
4729	mCV22746950	16	97849087	A	A	A	T	A	A
4730	rs6375622	16	94420190	G	G	G	A	G	G
4731	rs4220238	16	94472710	G	G	G	A	G	G
4732	rs6266287	16	94485971	A	A	A	A	A	A
4733	mCV23740174	16	96092689	A	A	A	G	A	A

4734	rs4220985	16	96803510	G	A	A	A	A	G
4735	rs4221263	16	97024198	C	C	C	C	C	C
4736	rs6210571	16	97032921	A	A	A	A	A	A
4737	rs3164054	16	97820338	A	A	A	G	A	A
4738	rs8274734	16	98377941	G	G	G	A	G	G
4739	rs6186492	16	98395340	A	A	A	A	A	A
4740	rs6254145	16	98481505	A	A	A	G	A	A
4741	rs6293022	8	71443115	A	G	A	G	A	A
4742	rs6167003	8	71508067	G	G	G	G	G	G
4743	rs6217180	17	6152941	G	G	G	G	G	G
4744	mCV23362339	17	5690382	A	G	G	G	G	G
4745	rs6169874	17	5863216	A	G	G	G	G	G
4746	rs3702143	17	5381593	A	G	G	G	G	A
4747	rs6171073	17	5165610	C	C	C	C	C	C
4748	rs3667161	17	5027585	G	A	A	A	A	A
4749	rs3686875	17	4366808	G	A	A	G	A	G
4750	rs3725494	17	4068731	A	A	A	G	G	A
4751	rs3672598	17	3913987	C	C	C	A	A	C
4752	rs6239530	17	3766890	G	G	G	A	A	A
4753	rs6240560	17	3766696	A	G	G	G	G	G
4754	rs6283871	17	3484414	A	A	A	G	A	A
4755	rs4136382	17	3388654	A	A	A	G	A	A
4756	rs3721166	17	3246965	G	A	A	A	G	G
4757	rs3724616	17	3000665	G	G	G	A	A	A
4758	rs3694629	17	6436726	G	G	G	A	G	G
4759	rs6328815	17	6652674	G	G	G	G	G	G
4760	rs3684897	17	6811803	G	A	A	G	A	A
4761	rs3710006	17	6823010	A	G	G	G	G	G
4762	mCV22941359	17	9243390	A	G	G	A	G	A
4763	rs3660905	17	7237712	G	C	C	G	C	C
4764	rs6191825	17	7395105	G	G	G	G	G	G
4765	rs3688250	17	7715521	A	G	G	A	G	G
4766	rs3706698	17	7974370	A	G	G	G	G	G
4767	rs6249429	17	8085354	G	G	G	G	G	G
4768	rs3722983	17	8252628	C	C	C	A	C	C
4769	rs3723109	17	8252703	A	A	A	G	A	A
4770	rs3090641	17	8468740	C	A	A	A	A	A
4771	rs4231330	17	8634636	A	A	A	A	A	A
4772	rs6372758	17	8877004	A	A	A	A	A	A
4773	rs3665137	17	8886026	G	G	G	A	G	A
4774	rs3702484	17	9103503	A	A	A	A	A	A
4775	rs3714793	17	9109795	G	A	A	A	A	A
4776	rs3662575	17	9388595	A	G	G	A	G	A
4777	rs6244408	17	9505215	A	A	A	A	A	A
4778	rs2020534	17	9618298	G	G	G	G	G	G
4779	rs3656290	17	10185388	A	G	G	A	G	A
4780	rs4231336	17	10313376	G	G	G	A	G	A
4781	rs6203320	17	10451573	G	A	A	A	A	A
4782	rs3658207	17	10759832	A	G	G	G	G	G
4783	rs3667406	17	10862445	G	A	A	G	A	G
4784	rs6231166	17	10944029	G	G	G	A	G	A
4785	rs3674166	17	11214621	A	G	G	A	G	A
4786	mCV24458659	17	11892953	G	G	G	A	G	A
4787	rs3662820	17	11723443	G	A	A	G	A	G
4788	rs6244331	17	11740615	G	G	G	G	G	G
4789	rs3089652	17	11896790	T	T	T	T	T	T
4790	rs3674900	17	11922826	G	A	A	A	A	A
4791	rs8262657	17	11992076	G	G	G	G	G	G
4792	rs8262658	17	11992249	G	G	G	G	G	G
4793	rs3693921	17	13105267	A	A	G	A	A	A
4794	rs3654147	17	12371207	A	A	G	G	A	G
4795	rs3723317	17	12406402	A	A	C	C	A	C
4796	rs6164117	17	12806000	A	A	A	A	A	A
4797	rs6183155	17	13037953	G	G	G	G	G	G
4798	rs8256979	17	13563599	A	A	A	A	A	A
4799	rs8256975	17	13563700	A	A	A	A	A	A
4800	rs3661800	17	13635486	A	A	A	G	A	G
4801	mCV24175979	17	15602752	T	T	A	A	T	A
4802	rs3689478	17	13831303	A	A	C	A	A	A
4803	rs3679431	17	13834888	C	C	C	C	C	C
4804	rs3714781	17	14620427	A	A	G	A	A	A
4805	rs6268777	17	14704985	G	G	G	G	G	G
4806	rs6362683	17	14990375	G	G	G	A	G	A
4807	rs3675740	17	15072904	A	A	C	C	A	C

4808	rs3726555	17	15215815	G	G	G	C	G	G
4809	rs6270865	17	15307109	A	A	G	A	A	G
4810	rs3667748	17	15457982	G	G	A	G	G	A
4811	mCV25045820	17	17611504	A	A	T	T	A	T
4812	rs3712728	17	16218065	A	A	G	G	A	A
4813	rs3719040	17	16221930	G	G	A	A	G	A
4814	rs3721884	17	16225206	T	T	A	T	T	T
4815	rs6284923	17	16415367	A	A	A	A	A	A
4816	rs6309226	17	16539988	A	A	C	A	A	C
4817	rs4137900	17	16816651	T	T	A	A	T	A
4818	rs6393324	17	16889400	T	T	T	T	T	A
4819	mCV23444673	17	19347416	A	A	A	T	A	A
4820	rs3672898	17	18097345	C	C	A	A	C	C
4821	rs3694284	17	18205555	C	C	A	A	C	C
4822	rs3671574	17	18221634	A	A	G	G	A	A
4823	rs3681769	17	18781372	C	C	A	A	C	A
4824	rs6341891	17	18893902	A	A	G	G	A	G
4825	rs3718510	17	19096830	G	G	A	A	G	A
4826	rs3667809	17	19252112	G	G	G	C	G	G
4827	rs8249644	17	19286471	A	A	C	C	A	C
4828	rs8239794	17	19287195	C	C	C	C	C	NONE
4829	rs3721675	17	19585518	G	G	G	A	G	G
4830	rs6267457	17	19659545	G	G	A	A	G	A
4831	rs6185197	17	19950473	A	A	G	G	A	G
4832	mCV24338392	17	20884774	A	A	G	G	A	G
4833	rs3698221	17	20199210	T	T	A	A	T	A
4834	rs3656149	17	21251079	G	G	G	G	G	G
4835	rs3696835	17	21394398	G	G	G	A	G	G
4836	rs6157758	17	21402389	A	A	A	A	A	A
4837	rs6305613	17	21862091	C	C	C	C	C	C
4838	rs3716211	17	22140138	G	G	A	A	G	A
4839	rs4231354	17	22374967	A	A	A	G	A	A
4840	rs6200761	17	22576942	A	A	A	G	A	A
4841	rs3669897	17	22797612	G	G	G	G	G	G
4842	rs3703275	17	22926002	A	A	A	G	A	A
4843	rs3667411	17	22992899	A	A	A	G	A	G
4844	rs3702941	17	23154579	G	G	G	A	G	G
4845	rs6407067	17	23177667	C	C	C	A	C	C
4846	rs8252745	17	23225384	T	T	T	A	T	T
4847	rs8252750	17	23225612	G	G	G	C	G	G
4848	rs3659741	17	23448649	C	C	C	A	C	C
4849	rs3669072	17	23883938	G	G	G	A	G	G
4850	rs8256992	17	23968313	G	A	G	G	A	G
4851	rs3719497	17	24100880	A	A	A	G	A	A
4852	rs6337584	17	24422663	A	A	A	A	A	A
4853	rs6338671	17	24422825	A/G	A	A	A/G	A	A
4854	rs4136360	17	24771683	A	G	A	G	G	G
4855	rs3715840	17	24970207	A	G	A	G	G	G
4856	rs8252767	17	25094658	G	G	G	G	G	G
4857	rs8252768	17	25094812	G	G	G	G	G	G
4858	mCV22893552	17	27304687	C	G	G	C	C	G
4859	mCV25130928	17	27460257	G	A	A	G	G	G
4860	rs3089389	17	25404864	A	A	A	A	A	A
4861	mCV23823298	17	27856524	G	A	A	G	G	A
4862	mCV23823306	17	27856591	A	T	T	A	A	T
4863	mCV23823307	17	27856628	A	G	G	A	A	G
4864	mCV23823308	17	27856770	G	A	A	G	G	G
4865	mCV23823315	17	27856980	G	A	A	G	G	A
4866	mCV23823316	17	27857043	C	A	A	C	C	C
4867	mCV23823317	17	27857262	A	T	T	T	A	T
4868	mCV23823324	17	27857264	G	A	A	A	G	A
4869	mCV23823325	17	27858894	G	A	A	G	G	A
4870	mCV23823605	17	27860857	A	G	G	G	A	G
4871	mCV23823611	17	27860877	A	G	G	G	A	G
4872	mCV23823620	17	27861253	A	G	G	A	A	G
4873	mCV23823622	17	26809701	G	G	G	G	G	G
4874	mCV23823630	17	27865078	A	G	G	G	A	G
4875	mCV23823642	17	27865597	G	A	A	G	G	A
4876	mCV23823653	17	27866179	G	G	G	A	G	G
4877	mCV23823654	17	27866210	G	G	G	A	G	G
4878	mCV23823665	17	27868357	G	A	A	G	G	G
4879	mCV23823666	17	27868421	A	G	G	G	A	G
4880	mCV23823676	17	27868645	G	A	A	G	G	A
4881	mCV23823689	17	27869072	C	G	G	C	C	G

4882	mCV23823690	17	27869203	G	G	G	C	G	G
4883	rs3656446	17	25935445	A	G	G	G	G	G
4884	rs6397584	17	26039914	A	A	A	C	A	A
4885	rs4231400	17	26168221	A	G	G	G	G	G
4886	rs3695831	17	26357216	A	G	G	G	G	G
4887	rs3723968	17	27393694	G	A	A	G	G	G
4888	rs6384940	17	27649241	G	G	G	A	G	G
4889	rs6308773	17	27783398	A	G	G	A	A	A
4890	rs3670354	17	27841317	A	G	G	G	A	G
4891	rs3693494	17	28381655	A	A	A	G	A	G
4892	rs3672136	17	28602063	C	C	C	G	C	G
4893	rs6264409	17	28659107	A	G	G	A	A	G
4894	rs3704177	17	28705435	G	A	A	G	G	A
4895	mCV27560398	17	30855613	G	A	A	G	G	G
4896	rs8274804	17	29792700	G	G	G	G	G	G
4897	rs6345886	17	29981285	A	C	C	A	A	A
4898	mCV22813496	17	31101498	C	A	A	C	C	C
4899	rs3684143	17	30349899	A	C	C	A	A	A
4900	rs6242153	17	30531880	C	C	C	A	C	A
4901	rs3685877	17	30535398	A	A	A	G	A	G
4902	rs6303400	17	30991976	A	A	A	A	A	A
4903	rs3705058	17	31635892	A	G	G	A	A	G
4904	rs3672987	17	31726297	A	A	A	T	A	A
4905	rs6249614	17	32051505	A	C	C	A	A	C
4906	rs3724223	17	32068525	A	G	G	A	G	G
4907	mCV22797750	17	32167323	A	G	G	A	G	G
4908	rs8242408	17	32690515	A	A	A	A	G	G
4909	rs3023452	17	32698941	A	G	G	A	G	G
4910	rs3665150	17	32834309	A	C	C	A	A	A
4911	mCV24833819	17	34280609	C	C	C	C	G	C
4912	rs8239672	17	34295376	A	G	A	A	G	G
4913	mCV22966449	17	33591420	C	A	A	C	A	A
4914	rs6288208	17	34041728	C	C	C	C	C	C
4915	mCV22965443	17	33636201	A	T	A	A	T	A
4916	rs3682923	17	34343989	A	G	A	G	G	G
4917	rs6169418	17	34483485	G	G	G	G	G	G
4918	rs8247942	17	34700180	G	G	A	A	A	G
4919	rs4139933	17	34723118	NONE	G	G	A	A	G
4920	rs6295184	17	34724657	C	A	A	A	A/C	A
4921	rs6277604	17	35019070	C	A	C	A	A	A
4922	mCV24432701	17	36599216	A	G	A	A	A	G
4923	rs6153283	17	35542332	A	A	A	A	A	A
4924	rs8237882	17	35571818	A	A	G	A	A	A
4925	rs8237880	17	35571992	A	A	A	A	A	A
4926	rs3682901	17	35961628	T	T	A	T	A	A
4927	rs6332461	17	36061319	G	A	G	A	G	G
4928	mCV24438675	17	35662531	A	G	A	A	G	G
4929	rs3145663	17	36354591	A	G	A	G	A	A
4930	mCV25060370	17	36131025	A	A	C	A	C	A
4931	rs6225551	17	36841633	G	G	G	G	G	G
4932	mCV25067640	17	36721752	G	A	A	A	A	G
4933	rs6390174	17	37542832	A	A	G	A	G	G
4934	rs3721814	17	37878210	C	G	C	G	C	C
4935	rs6370671	17	39267163	T	T	T	T	T	A/T
4936	rs3705623	17	38190965	G	A	G	A	G	G
4937	mCV23089208	17	39835658	C	G	C/G	G	C/G	C/G
4938	rs8254221	17	38941850	A	C	C	C	A	A
4939	mCV24450766	17	38306755	G	A	G	A	G	G
4940	rs3702604	17	39103029	G	A	G	A	A	G
4941	rs6295287	17	39267362	A	A	A	T	T	A
4942	rs3655646	17	39358743	G	G	A	G	G	G
4943	mCV25168292	17	41083128	G	A	A	A	G	G
4944	rs6399116	17	39888389	G	G	G	G	G	G
4945	rs4231483	17	40151966	G	G	G	G	A	G
4946	rs3664698	17	40369398	G	G	G	A	G	G
4947	rs3726572	17	40406153	G	G	G	A	G	G
4948	rs6236638	17	40550397	A	A	A	A	G	A
4949	rs6280628	17	40591253	G	G	G	A	G	G
4950	mCV23717143	17	39623874	A	A	G	G	A	G
4951	rs3090290	17	40804382	G	G	G	G	G	G
4952	rs6208897	17	41383159	T	A	A	T	T	T
4953	rs3709304	17	41572825	G	G	A	A	G	G
4954	rs3656008	17	41777024	A	A	A	G	A	G
4955	mCV25197172	17	41080900	G	G	G	G	A	G

4956	rs6278687	17	42194322	A	G	G	G	G	A
4957	rs3711632	17	42239130	A	G	G	A	G	A
4958	rs3677240	17	42314549	A	A	A	G	A	A
4959	rs6184522	17	43119719	A	A	G	G	G	A
4960	rs3668036	17	43844174	G	G	A	G	G	G
4961	rs4231504	17	43855174	A	A	A	A	A	A
4962	rs3658769	17	43857174	C	C	A	C	C	C
4963	rs3718925	17	43917579	A	A	A	G	A	A
4964	rs6356929	17	44139227	G	G	G	G	G	G
4965	rs3705319	17	44743703	A	G	G	G	G	G
4966	rs8261571	17	44885188	G	G	G	G	G	G
4967	rs3090988	17	44913748	A	A	A	A	A	A
4968	rs6413403	17	45006996	A	A	A	A	A	A
4969	rs3657977	17	45526654	A	G	G	G	G	G
4970	rs4231530	17	45985950	A	A	A	A	A	A
4971	rs3715180	17	46031381	G	A	A	G	A	A
4972	mCV22964525	17	45307498	A	T	T	T	T	T
4973	rs3705130	17	46933263	C	C	C	A	C	C
4974	rs4137228	17	46933322	G	G	G	A	G	G
4975	rs3706340	17	46933503	T	T	T	A	T	T
4976	rs3709891	17	47141909	G	G	G	A	G	A
4977	rs6314185	17	47439648	G	A	A	A	A	G
4978	rs3690039	17	47611360	A	A	A	C	A	C
4979	rs3678696	17	48018457	G	A	A	G	A	A
4980	mCV27587738	17	47274186	A	T	T	A	T	T
4981	rs3712953	17	48442632	G	G	G	A	G	G
4982	rs3682797	17	48503978	G	A	A	G	A	G
4983	rs6352244	17	48760688	C	G	G	C	G	G
4984	rs3716288	17	48939275	C	C	C	A	C	A
4985	rs6395919	17	49474335	C	C	C	G	C	C
4986	rs6193898	17	49947879	G	G	G	A	G	A
4987	rs6191282	17	50245177	A	A	A	G	A	G
4988	rs3678447	17	50312700	G	G	G	A	G	G
4989	rs3703891	17	50566379	A	G	G	A	G	A
4990	rs3700227	17	50793087	G	G	G	A	G	A
4991	rs6247950	17	51008551	T	T	T	T	T	T
4992	mCV23762311	17	50479183	G	A	A	G	A	G
4993	rs6272475	17	51663908	A	A	A	G	A	A
4994	rs3701810	17	51849685	A	A	A	T	A	A
4995	rs3671369	17	51861608	A	A	A	G	A	A
4996	rs8273948	17	52018568	A	A	A	A	A	A
4997	rs8273969	17	52020910	G	G	G	C	G	G
4998	rs4138162	17	52036347	G	G	G	A	G	G
4999	rs8258060	17	52248879	C	C	C	C	C	C
5000	rs8258074	17	52251943	A	A	A	A	A	A
5001	rs8258045	17	52259159	A	A	A	A	A	A
5002	rs6241282	17	52370790	A	A	A	G	A	A
5003	rs3665187	17	52576752	G	G	G	A	G	G
5004	rs3667784	17	52602580	T	A	A	T	A	T
5005	rs3726913	17	52765739	A	A	A	G	A	A
5006	rs6403494	17	53111449	A	A	A	G	A	A
5007	rs6188603	17	53391442	G	A	A	G	A	G
5008	rs3725839	17	53413175	A	C	C	A	C	A
5009	rs3667909	17	53413482	A	G	G	A	G	A
5010	rs3714226	17	53552802	C	C	C	A	C	C
5011	rs3714819	17	53634527	C	C	C	A	C	C
5012	rs3023455	17	53720340	G	G	G	A	G	G
5013	rs3705741	17	53745193	A	A	A	G	A	A
5014	rs6376589	17	53760239	G	G	G	G	G	G
5015	rs3709414	17	53973279	A	A	A	C	A	A
5016	rs3663425	17	53978315	A	A	A	G	A	A
5017	mCV23775834	17	53723343	C	C	C	G	C	C
5018	rs6304244	17	54320104	T	T	T	T	T	T
5019	rs6356535	17	54362877	G	G	G	G	G	G
5020	mCV25255424	17	53911709	A	G	G	G	G	A
5021	rs3714078	17	54652919	G	G	G	G	G	G
5022	rs6334729	17	54734199	C	C	C	C	C	C
5023	rs3090279	17	55307700	G	G	G	G	G	G
5024	rs3712800	17	55343894	A	G	G	G	G	A
5025	rs4231565	17	55565601	C	C	C	C	C	C
5026	rs4231576	17	55773312	C	C	C	C	C	C
5027	rs3023458	17	55806612	G	G	G	G	G	G
5028	rs3703241	17	55958582	G	A	A	A	A	A
5029	rs2020693	17	58224766	A	A	A	A	A	A

5030	rs6326012	17	56684844	A	A	A	A	A	A
5031	rs6397556	17	56695485	G	G	G	G	G	G
5032	rs3683834	17	56902266	A	T	T	T	T	T
5033	rs3715723	17	56999568	A	C	C	C	C	C
5034	rs4231577	17	57211029	G	A	A	A	A	A
5035	rs6245412	17	57237249	G	A	A	A	A	A
5036	mCV24362398	17	56083454	A	C	C	C	C	C
5037	rs6240557	17	58029831	A	A	A	A	A	A
5038	rs3089429	17	58508327	T	T	T	T	T	T
5039	rs6294080	17	59028096	G	G	G	G	G	G
5040	mCV23050965	17	57718414	C	C	C	C	G	C
5041	rs6251286	17	59203757	C	C	A	C	C	C
5042	rs6153567	17	59689409	A	A	A	A	A	A
5043	rs6180905	17	60268365	G	G	G	G	G	G
5044	rs6284634	17	60829031	A	A	A	A	A	A
5045	rs3688465	17	61144882	A	G	A	G	G	G
5046	rs4231581	17	61406487	G	G	G	G	G	G
5047	rs4231582	17	61406523	A	A	A	A	A	A
5048	rs3707749	17	61447544	A	G	A	G	G	G
5049	rs3725261	17	61514860	G	A	G	A	A	A
5050	rs3090162	17	61730499	G	G	G	G	G	G
5051	rs6402097	17	62066360	A	A	A	A	G	A
5052	rs6270149	17	62382711	C	C	C	C	C	C
5053	rs4231584	17	62494626	A	A	A	A	A	A
5054	rs3091109	17	63107197	G	G	G	G	G	G
5055	mCV22888090	17	61947366	A	A	A	A	G	A
5056	rs3719337	17	63706139	A	A	A	A	G	G
5057	mCV23053625	17	62696390	A	A	A	A	A	A
5058	rs4231587	17	64013697	A	A	A	A	A	A
5059	rs6367822	17	64143579	A	G	A	A	G	G
5060	rs3657113	17	64190941	A	G	A	A	G	G
5061	rs3090284	17	64250223	G	G	G	G	G	A/G
5062	rs6185805	17	64731493	C	C	C	A	C	C
5063	mCV23056184	17	63809601	G	G	G	G	A	A
5064	rs3708432	17	64967478	G	G	G	A	G	G
5065	rs3671056	17	65089724	G	G	G	A	G	G
5066	rs6249763	17	65207648	G	G	G	A	G	G
5067	rs4231594	17	65303321	G	G	G	G	G	G
5068	rs3089454	17	65333279	G	G	G	G	G	G
5069	rs3684212	17	65520346	A	A	A	G	A	A
5070	rs3685911	17	65536624	A	A	A	T	A	A
5071	rs3089550	17	65711637	A	A	A	A	A	A
5072	rs6315445	17	65716653	A	A	A	G	A	A
5073	rs6382997	17	65726542	G	G	G	A	G	G
5074	rs3727008	17	66156395	A	A	A	G	G	A
5075	rs4231612	17	66245021	A	A	A	A	A	A
5076	rs4231615	17	66265661	A	A	A	A	C	C
5077	rs3709922	17	66470109	G	A	G	A	A	A
5078	rs3666819	17	66496973	A	A	A	G	A	A
5079	rs3682341	17	66503793	A	G	G	G	G	G
5080	rs6399089	17	66558479	G	G	A	G	A	A
5081	rs3724918	17	66653148	A	C	A	A	A	A
5082	rs6239923	17	67144876	A	A	A	A	A	A
5083	rs3699986	17	67179467	A	C	C	C	C	C
5084	rs3690010	17	67406403	A	C	C	A	C	C
5085	rs3657117	17	67483047	G	G	G	A	G	G
5086	rs6192583	17	68204399	A	A	A	A	A	A
5087	rs6302685	17	68453937	A	C	C	C	C	C
5088	rs3701107	17	68677840	T	A	A	T	A	T
5089	rs3660112	17	69021924	A	A	A	C	A	C
5090	rs4231624	17	69402497	G	G	G	G	G	G
5091	rs3717152	17	69491587	A	A	A	G	A	G
5092	rs3681971	17	69533244	A	A	A	G	A	G
5093	rs6257479	17	69639007	G	G	G	A	G	G
5094	mCV24388347	17	68812821	G	A	A	G	A	G
5095	rs3675634	17	69670995	A	A	A	G	A	A
5096	rs3693863	17	69678219	G	G	G	A	G	G
5097	rs6155172	17	70085854	A	A	A	G	A	A
5098	rs3654545	17	70526219	G	A	G	G	G	G
5099	rs6322076	17	70683789	G	A	G	A	G	G
5100	rs3680857	17	70764446	A	A	A	G	A	A
5101	rs3691628	17	70833240	A	A	A	G	A	A
5102	rs6263744	17	70929484	G	G	A	A	A	A
5103	rs6284907	17	71119789	G	A	G	A	G	G

5104	rs4137129	17	71346669	G	G	G	A	G	G
5105	rs3706023	17	71493414	A	A	G	A	G	G
5106	rs4231637	17	71682600	G	G	G	G	G	G
5107	rs3719059	17	72131923	A	A	G	A	G	G
5108	rs6280326	17	72223894	G	G	A	A	A	A
5109	rs3089431	17	72225083	C	C	C	C	C	C
5110	rs3697418	17	72277773	A	A	A	G	A	A
5111	rs4136071	17	72713849	A	A	A	G	A	A
5112	rs4231653	17	72763263	A	A	A	A	A	A
5113	rs4231654	17	72763355	G	G	G	G	G	G
5114	rs6372166	17	72807022	G	G	G	A	G	G
5115	rs3676739	17	73452746	G	G	G	A	G	A
5116	rs4137733	17	73566504	A	T	T	T	T	T
5117	rs6229946	17	73658199	A	A	A	C	A	C
5118	rs3704554	17	73659610	G	G	G	A	G	A
5119	rs3710559	17	73742289	C	G	G	C	G	C
5120	rs3710803	17	74186168	A	A	A	G	A	G
5121	rs3694520	17	74380752	C	A	A	C	A	C
5122	rs3664306	17	74555767	C	C	C	C	C	C
5123	rs3656948	17	74942842	C	C	C	A	C	A
5124	rs6278585	17	75294848	C	A	A	C	A	C
5125	mCV24431619	17	74706211	A	C	C	C	C	C
5126	rs3717212	17	75421714	G	G	A	G	A	G
5127	rs3663088	17	75526825	G	G	G	A	G	A
5128	rs6267152	17	75753976	T	T	A	T	A	T
5129	rs6390351	17	76024538	G	G	A	G	A	G
5130	rs3699947	17	76352887	A	A	G	A	G	A
5131	rs6386440	17	76897964	A	G	A	G	A	A
5132	rs3685368	17	76901195	A	G	G	G	G	A
5133	rs3663966	17	77250063	G	G	A	A	A	G
5134	rs4231668	17	77405947	C	C	C	C	C	C
5135	rs8237851	17	77433287	A	A	A	A	A	A
5136	rs4231669	17	77435608	A	A	A	A	A	A
5137	rs3718983	17	77832707	G	G	G	A	G	G
5138	rs3692379	17	77906290	T	A	T	A	T	T
5139	rs3670607	17	77914489	A	A	A	G	A	A
5140	rs3674040	17	78030057	A	A	A	G	A	A
5141	rs8255877	17	78249305	G	G	G	G	G	G
5142	rs8253071	17	78253474	G	G	G	G	G	G
5143	mCV23092135	17	80479240	A	A	A	G	A	A
5144	rs4231674	17	78577995	A	A	A	A	A	A
5145	rs6371065	17	78627417	A	C	A	A	A	A
5146	rs4231679	17	78951087	G	G	G	G	G	G
5147	rs6305051	17	79153888	G	A	G	G	G	G
5148	mCV22762284	17	78731080	G	A	G	G	G	G
5149	rs6181774	17	79837307	A	A	G	A	G	A
5150	rs3686767	17	79930775	A	A	T	A	T	A
5151	rs3711010	17	80048342	A	C	C	C	C	C
5152	rs4231698	17	80070788	NONE	G	G	G	G	G
5153	mCV23534950	17	80188236	A	G	G	G	A	G
5154	rs6154895	17	81025139	A	A	A	A	A	A
5155	rs6276365	17	81421811	A	A	A	A	G	A
5156	rs3684732	17	81575999	G	G	G	A	A	G
5157	rs3685398	17	81576122	C	C	C	A	A	C
5158	rs3664004	17	81789955	G	G	G	A	G	G
5159	rs6263123	17	81971402	G	G	G	A	G	G
5160	rs3662869	17	82041104	A	A	A	G	A	A
5161	rs3706971	17	82357907	A	A	A	C	C	C
5162	rs3701338	17	82579890	G	G	G	A	G	A
5163	mCV22838407	17	82364401	G	G	G	A	G	G
5164	rs3694880	17	83063710	G	G	G	A	A	A
5165	rs6361015	17	83316881	A	A	A	G	A	A
5166	rs6370900	17	83747260	T	T	T	T	T	T
5167	mCV25276476	17	83163111	A	A	A	A	G	A
5168	rs4137718	17	84047966	T	T	T	A	A	A
5169	rs4138686	17	84053123	C	C	C	A	A	A
5170	rs3686841	17	84357971	G	A	A	A	A	A
5171	rs3090281	17	84579122	G	G	G	G	G	G
5172	rs3669120	17	84773276	A	G	G	A	A	G
5173	mCV23125224	17	84283431	A	G	G	A	A	A
5174	rs6208887	17	84977157	A	G	G	A	A	A
5175	rs6336971	17	85333402	G	G	G	A	G	G
5176	rs4231720	17	85385338	G	G	G	A	G	G
5177	rs4231719	17	85385360	T	T	T	T	T	T

5178	rs3660113	17	85503975	G	A	A	A	A	A
5179	rs4223253	17	85676456	G	A	A	G	A	A
5180	rs3689581	17	85678884	G	G	G	A	G	G
5181	mCV23317493	17	85098239	G	A	A	G	G	G
5182	rs6371637	17	85983636	G	G	G	G	G	G
5183	rs3660792	17	86096117	G	G	G	A	A	G
5184	rs3687741	17	86184391	G	A	A	G	G	A
5185	rs3675790	17	86210258	A	A	A	C	C	A
5186	rs3717375	17	86271144	A	G	G	G	G	G
5187	rs3717026	17	86332237	G	G	G	A	A	A
5188	rs3670275	17	87124699	G	G	G	A	A	A
5189	rs3723150	17	87166427	A	A	A	C	C	C
5190	rs3698948	17	87392863	G	G	G	A	A	A
5191	rs8252727	17	87407573	G	G	G	G	G	G
5192	rs3667644	17	88110557	A	C	C	A	C	C
5193	rs6244553	17	88134897	A	G	G	A	G	G
5194	rs3667051	17	88400292	A	A	A	C	A	C
5195	rs3720344	17	88667431	A	A	A	G	A	G
5196	rs6350864	17	88769457	A	A	A	A	A	A
5197	rs3705332	17	88886608	A	A	A	G	A	G
5198	rs3696168	17	89038700	A	A	A	G	A	A
5199	rs3711990	17	89063089	T	T	T	A	T	T
5200	rs6313030	17	89173825	A	A	A	C	A	A
5201	rs3662979	17	89783795	A	A	G	A	G	A
5202	rs6288047	17	90215134	G	G	G	A	G	G
5203	mCV23152636	17	89655809	A	A	C	C	C	C
5204	rs3711932	17	90340545	A	A	A	T	A	T
5205	rs3664022	17	90675374	G	G	A	A	A	A
5206	rs3709838	17	90801357	A	A	G	A	G	A
5207	rs6294119	17	90832316	C	C	C	C	C	C
5208	mCV23156879	17	90555098	G	G	A	A	A	A
5209	rs3710808	17	91771218	C	A	C	A	C	C
5210	rs6397044	17	91938734	NONE	A	G	A	NONE	G
5211	rs3716639	17	92056452	G	G	A	G	A	G
5212	rs6281710	17	92187851	G	G	A	G	A	G
5213	rs3707114	17	92757308	A	A/G	G	A/G	A/G	A
5214	rs3706382	17	92912930	A	A	T	A	T	A
5215	rs6391399	17	93099132	G	G	G	G	G	G
5216	rs6336001	17	93239347	G	G	G	G	G	G
5217	mCV23172085	17	93491059	A	A	G	A	G	A
5218	rs3696933	18	33968545	G	A	A	G	A	G
5219	rs3664772	18	6221939	A	G	A	G	G	G
5220	rs3671490	18	13787472	G	G	A	A	A	A
5221	rs4231891	18	61107753	C	C	C	C	C	C
5222	rs6339254	18	35140804	A	T	A	A	T	A
5223	rs3702013	18	26999816	A	A	G	A	A	A
5224	rs6404726	18	3460447	A	A	A	A	A	A
5225	rs6332628	18	4229952	A	A	A	A	A	A
5226	rs6394685	18	5198761	A	A	A	A	A	A
5227	rs3676326	18	5284178	G	A	A	A	A	A
5228	rs8254978	18	5975902	C	C	C	C	C	C
5229	rs8254981	18	6083214	G	G	G	G	G	G
5230	rs3660995	18	5993681	A	C	A	C	C	C
5231	rs3662231	18	5993948	A	G	A	G	G	G
5232	rs6291709	18	6319442	T	T	T	T	T	T
5233	rs3680906	18	6890748	A	G	A	G	G	G
5234	rs3689829	18	7051562	G	A	G	A	A	A
5235	rs3712830	18	7053066	G	G	C	G	G	G
5236	rs6256578	18	7351610	A	A	A	A	A	A
5237	rs3715093	18	7352159	A	G	G	G	G	G
5238	rs3680668	18	7403115	G	G	A	G	G	G
5239	rs3656221	18	7651270	G	G	A	G	G	G
5240	rs6160421	18	8089618	A	A	A	A	A	A
5241	rs3689558	18	8254573	G	G	A	G	G	G
5242	mCV24391893	18	11809427	C	G	G	C	G	G
5243	rs3671937	18	8662226	A	T	T	T	T	T
5244	rs3722941	18	9074049	G	A	A	A	A	A
5245	rs6200782	18	9086189	C	G	G	G	G	G
5246	rs3670421	18	9343756	G	G	A	G	G	G
5247	rs3660245	18	9491813	T	T	T	A	A	A
5248	rs3683349	18	9493278	A	A	A	C	C	C
5249	rs2020500	18	10066074	C	C	C	C	C	C
5250	rs3723041	18	10039176	G	G	G	A	A	A
5251	rs3668888	18	9942350	G	A	A	A	A	A

5252	rs3686464	18	9846952	A	A	A	T	T	T
5253	mCV23615070	18	13355068	G	A	A	A	A	A
5254	rs6402963	18	10256623	G	A	A	A	A	A
5255	rs3670316	18	11135762	C	A	A	A	A	A
5256	rs4231742	18	11306822	A	G	G	A	G	G
5257	rs4231739	18	11307003	G	G	G	G	G	G
5258	rs3653699	18	11450421	G	A	A	A	A	A
5259	rs6377403	18	11592854	G	G	G	A	G	G
5260	mCV23617245	18	14895705	G	G	G	A	A	G
5261	rs3663413	18	11996302	A	A	G	A	A	A
5262	rs3710602	18	12172015	G	G	G	A	A	G
5263	rs3711709	18	12221563	G	A	A	G	G	A
5264	rs3664343	18	12296552	A	A	G	A	A	A
5265	rs8255002	18	12575921	G	A	A	A	A	G
5266	rs3667418	18	12966886	A	G	G	G	G	G
5267	rs6194396	18	13581902	A	A	A	G	G	A
5268	rs6237628	18	13602724	G	G	G	A	A	G
5269	rs3682653	18	13678415	G	G	G	A	A	G
5270	rs3716257	18	13701374	A	A	A	G	G	A
5271	mCV23641317	1	42422145	A	A	A	G	A	A
5272	rs6303028	18	14055191	G	G	G	A	A	G
5273	rs6287645	18	14369611	G	G	A	A	A	A
5274	rs6317011	18	15094289	G	G	A	A	A	A
5275	rs3658805	18	15096200	G	G	G	A	A	A
5276	rs3683582	18	15124363	A	A	A	G	G	G
5277	rs6361186	18	15373312	G	G	G	A	A	A
5278	rs6361663	18	15373357	A	A	A	G	G	G
5279	rs3678446	18	15490522	A	A	A	G	G	G
5280	rs3688865	18	15493663	A	A	A	G	G	G
5281	rs3679437	18	15727143	A	A	C	C	C	C
5282	rs8256206	18	15764118	G	G	G	G	G	G
5283	rs6243077	18	16213979	A	A	A	A	A	G
5284	rs6244740	18	16214281	G	G	G	G	G	G
5285	rs3674389	18	16295547	A	A	G	G	G	A
5286	rs3724798	18	17144490	G	A	A	A	G	A
5287	rs6364856	18	17709970	G	A	A	A	G	A
5288	rs3090636	18	17772608	C	C	C	C	A	C
5289	rs3701343	18	18238507	G	A	A	A	G	G
5290	rs6403230	18	18735996	T	A	A	A	T	T
5291	rs3679802	18	19099880	A	G	G	G	A	A
5292	mCV23670826	18	21882551	A	C	A	A	C	C
5293	rs3656185	18	19941646	A	G	G	G	A	G
5294	rs6303064	18	19992554	C	A	A	A	C	C
5295	rs6305298	18	20914551	G	A	A	A	G	G
5296	mCV23675970	18	20775526	C	A	A	A	C	C
5297	rs6265202	18	21181978	A	G	G	G	G	G
5298	rs3697673	18	21185318	G	C	C	C	C	C
5299	rs6405412	18	21235290	A	G	G	G	A	A
5300	rs8255015	18	21473215	G	G	G	G	G	G
5301	rs4231757	18	21530950	G	G	G	G	A	A
5302	rs4231751	18	21531284	G	G	G	G	G	G
5303	rs4231759	18	21579938	A	A	A	A	A	A
5304	rs3695261	18	22135899	G	G	A	A	G	G
5305	rs3685375	18	22277575	A	A	G	G	A	A
5306	rs3680100	18	22328434	G	G	A	A	G	G
5307	rs3707236	18	22349475	G	G	A	A	G	G
5308	rs6323016	18	22400485	C	C	C	C	C	C
5309	rs3676826	18	22482188	A	A	T	T	A	A
5310	rs4138020	18	23001325	A	A	G	G	A	A
5311	rs6368237	18	23859205	A	G	G	G	A	A
5312	rs3662301	18	23916457	A	T	T	T	A	A
5313	rs3675490	18	23989819	G	A	A	A	G	G
5314	rs6365146	18	24192499	A	G	G	G	A	A
5315	rs4231762	18	24310967	A	A	A	A	A	A
5316	rs4231764	18	24382152	C	C	C	C	C	C
5317	rs3705122	18	24382288	C	A	A	C	C	C
5318	rs4231768	18	24997791	G	G	G	G	G	G
5319	rs3660676	18	25278262	G	A	A	G	A	G
5320	rs6167189	18	25303642	A	G	G	A	G	A
5321	rs3662641	18	25906081	C	A	A	A	A	C
5322	rs6229632	18	26750311	A	A	A	A	A	A
5323	rs3683414	18	26795949	C	A	C	A	A	A
5324	rs3675291	18	26809188	G	A	G	A	A	A
5325	rs6399698	18	27079169	A	A	A	A	A	A

5326	rs3090648	18	27469928	A	A	A	A	A	A
5327	rs2020544	18	28013006	A	A	A	A	A	A
5328	rs6161430	18	28109893	T	T	T	T	T	T
5329	rs6192656	18	28124261	G	G	G	G	G	G
5330	rs3671174	18	28539857	G	G	A	G	G	G
5331	rs3090850	18	28598270	A	A	C	A	A	A
5332	rs6402448	18	29169579	T	T	A	T	T	T
5333	rs3705063	18	29194269	A	A	C	A	A	A
5334	rs2020689	18	29303686	G	G	G	G	G	G
5335	rs3691362	18	30053090	C	C	A	C	C	A
5336	rs3090310	18	30195729	A	A	A	A	A	A
5337	rs3714233	18	30247058	A	A	G	A	A	G
5338	rs3023463	18	30249388	A	A	C	A	A	C
5339	rs6251727	18	30427721	G	A	A	A	A	G
5340	rs3723947	18	30885788	C	C	C	G	C	C
5341	rs3669742	18	30886721	A	G	A	G	G	G
5342	rs4228543	18	30943450	C	C	C	C	C	C
5343	rs3683699	18	30952210	A	G	A	G	G	G
5344	rs3693221	18	31215364	A	A	A	G	A	A
5345	rs6286913	18	31266611	G	A	G	A	A	A
5346	rs3672229	18	31390432	G	A	G	A	A	G
5347	rs3722973	18	31395543	A	G	A	G	G	A
5348	rs6340427	18	31500431	A	G	A	G	G	G
5349	rs6174809	18	31629539	G	A	A	A	A	A
5350	rs3666799	18	32344759	A	C	C	A	C	C
5351	rs3694865	18	32413054	A	A	G	A	A	G
5352	rs8269848	18	32591012	C	C	C	C	C	C
5353	rs6385006	18	32986999	A	A	G	A	A	A
5354	rs2228920	18	33007037	A	A	A	A	A	A
5355	rs3718586	18	33572164	G	A	A	G	A	G
5356	rs6239750	18	34039970	A	A	A	A	A	A
5357	rs3723870	18	34009418	A	A	A	G	A	A
5358	rs6247392	18	34001365	G	G	G	G	G	G
5359	rs3717761	18	34103539	A	A	A	G	A	A
5360	rs3719580	18	34113296	C	C	C	A	C	C
5361	rs3705413	18	34354033	G	G	A	A	G	A
5362	rs8255038	18	34699970	G	A	G	G	A	G
5363	rs8255039	18	34700733	A	A	A	A	A	A
5364	rs3725637	18	34867922	C	C	C	A	C	A
5365	rs4231783	18	34880525	G	G	G	G	G	G
5366	rs3663045	18	34995545	A	A	A	G	A	G
5367	rs3701931	18	35039632	A	C	A	C	C	C
5368	rs3675531	18	35484760	G	G	G	A	G	A
5369	rs3714096	18	36136675	G	G	A	G	G	G
5370	rs6163326	18	36252139	A	G	A	A	G	A
5371	rs6389331	18	36474881	A	A	A	A	A	A
5372	rs3654071	18	36457123	A	G	G	A	G	A
5373	rs4231787	18	37110628	G	G	G	G	G	G
5374	rs3703106	18	37131538	C	C	C	A	NONE	C
5375	rs8243297	18	37168597	A	A	A	A	A	A
5376	rs8255719	18	37169067	A	A	A	G	A	A
5377	rs6298679	18	37173233	G	G	G	A	G	G
5378	mCV24094701	18	39180646	A	G	G	A	G	A
5379	rs3664831	18	37737388	C	C	C	A	C	C
5380	rs3697964	18	38267355	G	G	A	G	G	G
5381	rs8255071	18	38382651	A	A	A	A	A	A
5382	rs8255054	18	38385001	G	G	G	G	G	G
5383	rs8255052	18	38386144	A	A	A	A	A	A
5384	rs8255051	18	38387266	G	G	G	G	G	G
5385	rs8255047	18	38388225	A	A	A	A	A	A
5386	rs3700198	18	38432022	G	G	G	A	G	G
5387	rs3679365	18	38918094	G	G	A	G	G	A
5388	rs4231815	18	39282149	C	C	C	C	C	C
5389	rs4231819	18	39282219	G	G	G	G	G	G
5390	rs3669650	18	39390540	A	A	A	G	A	G
5391	rs6261236	18	39421495	A	A	A	A	A	A
5392	rs3699816	18	39756682	G	G	G	A	G	A
5393	rs8259560	18	39858310	G	G	G	G	G	G
5394	rs8259534	18	39865499	G	G	G	G	G	G
5395	rs8259553	18	39867896	G	G	G	G	G	G
5396	rs8259552	18	39868041	G	G	G	G	G	G
5397	rs3678901	18	39928937	G	G	G	A	G	A
5398	rs8240448	18	39934591	A	A	A	A	A	A
5399	rs6163341	18	40258600	G	G	G	G	G	G

5400	mCV23302006	18	42614789	G	A	A	G	A	G
5401	rs3656236	18	41342056	G	G	A	A	NONE	A
5402	rs6179902	18	41394515	A	A	G	G	A	G
5403	rs6204949	18	41480954	A	A	A	A	A	A
5404	rs3089327	18	42223388	C	C	C	C	C	C
5405	rs6313313	18	42102260	G	G	G	A	G	G
5406	rs6363007	18	42111168	C	C	C	C	C	C
5407	rs6288658	18	49864878	A	A	A	A	A	A
5408	rs3711076	18	42857406	A	A	A	G	A	A
5409	rs6323500	18	42863443	A	G	G	G	G	A
5410	rs4221967	18	43015979	A	A	A	A	A	A
5411	rs3089840	18	43158143	A	A	A	A	A	A
5412	rs6217237	18	43234262	G	G	G	G	G	G
5413	rs3715190	18	43395940	A	A	A	C	A	A
5414	rs3690657	18	43631028	A	A	A	G	A	G
5415	rs3655318	18	43824277	T	T	T	A	T	T
5416	rs3722777	18	44404491	A	C	A	C	A	A
5417	rs4231839	18	44463284	A	A	A	A	A	A
5418	rs6182841	18	44699271	A	A	A	A	A	A
5419	rs6192044	18	47242647	C	C	C	C	C	C
5420	rs3656192	18	45282758	T	T	A	T	A	T
5421	rs3657131	18	45335172	G	A	A	G	A	G
5422	rs4138936	18	45372492	A	C	C	C	C	A
5423	rs3676483	18	45381444	A	A	A	C	A	A
5424	rs6159382	18	46202607	G	G	G	G	G	G
5425	rs6236248	18	46907224	A	A	A	A	A	A
5426	rs4231845	18	46684237	C	C	C	C	C	C
5427	rs4231842	18	46684495	G	G	A	G	A	G
5428	rs3716758	18	46720133	A	A	A	T	A	A
5429	rs3659020	18	46720342	A	A	A	G	A	A
5430	rs3088812	18	48025738	C	C	C	G	C	C
5431	mCV24106092	18	49464960	C	C	C	A	C	C
5432	rs3674256	18	47587690	A	A	G	A	G	A
5433	rs3721430	18	47609589	G	G	G	A	G	G
5434	rs6184541	18	47884501	A	A	G	A	G	A
5435	rs4139897	18	48301422	G	G	G	A	G	G
5436	rs3714312	18	48616500	G	G	A	A	A	G
5437	rs3707785	18	49147258	A	A	G	A	G	A
5438	rs3718133	18	49143735	G	G	A	G	A	G
5439	rs3684561	18	49102908	A	C	C	C	C	A
5440	rs3089047	18	49234950	A	A	A	A	A	A
5441	rs6326826	18	49330912	A	A	G	A	G	A
5442	rs3090896	18	49596118	A	A	G	G	G	G
5443	rs3703039	18	49719879	A	A	G	A	G	G
5444	rs4138356	18	50024484	A	A	G	A	G	G
5445	rs6384465	18	50062469	A	A	A	A	A	A
5446	rs3657958	18	50417128	A	A	A	G	A	A
5447	rs6353725	18	50338671	C	C	C	C	C	C
5448	rs3674314	18	51674308	A	T	T	T	T	T
5449	rs4231849	18	51784672	A	A	A	A	A	A
5450	rs4231846	18	51785032	A	A	A	A	A	A
5451	mCV22331476	18	54309209	A	C	C	A	A	A
5452	rs6262441	18	52814428	G	G	G	G	G	G
5453	rs3722312	18	52826928	G	G	G	A	G	G
5454	rs4231853	18	52996979	C	C	C	C	C	C
5455	rs8243426	18	53009205	G	G	G	G	G	G
5456	rs3090299	18	53550194	C	C	C	C	C	C
5457	rs3665626	18	53651426	A	A	A	T	A	A
5458	rs6315116	18	53653164	G	A	A	A	A	A
5459	rs3683337	18	53880488	G	G	G	A	G	G
5460	rs6152921	18	53745589	G	G	G	G	G	G
5461	rs4231857	18	54175849	A	A	A	A	A	A
5462	rs3681210	18	54538463	A	C	A	C	C	C
5463	rs6172588	18	55215646	A	A	A	A	A	A
5464	rs4138249	18	55239367	G	G	G	G	G	G
5465	rs3088626	18	55350910	G	G	G	G	G	G
5466	rs6159970	18	55432634	C	C	C	C	C	C
5467	rs3683378	18	56183873	C	C	G	C	C	C
5468	rs3684646	18	56184126	A	A	G	A	A	A
5469	rs6281219	18	56770661	A	A	A	A	A	A
5470	rs3654438	18	56796176	G	G	A	A	A	G
5471	mCV23191036	18	58974019	A	C	A	A	A	A
5472	rs3655793	18	57734357	G	G	G	A	G	G
5473	rs3724103	18	57741423	G	G	G	A	G	G

5474	mCV24036087	18	59617542	A	T	T	T	T	T
5475	rs3705107	18	57935634	G	A	G	A	G	G
5476	rs6328845	18	58310005	A	A	A	G	A	G
5477	rs3670254	18	58394117	T	T	T	A	T	T
5478	rs3090956	18	58450045	G	G	G	G	G	G
5479	rs8239211	18	58515377	A	A	A	A	A	A
5480	rs8239209	18	58515423	A	G	A	G	A	G
5481	rs8239208	18	58515466	G	G	G	G	G	G
5482	rs3669543	18	58570705	A	A	A	G	A	A
5483	rs3713935	18	58981153	A	G	A	G	A	A
5484	rs3666286	18	59090139	C	C	A	A	A	C
5485	rs6167993	18	59243139	T	T	T	T	T	T
5486	rs3688263	18	59727433	C	A	A	A	A	A
5487	rs6192612	18	59926407	G	A	A	A	A	A
5488	rs4231875	18	59927053	C	G	NONE	G	NONE	NONE
5489	rs4231874	18	59927141	G	G	G	G	G	G
5490	mCV24036663	18	62328246	G	G	A	A	A	A
5491	rs3685720	18	60565588	G	A	A	A	A	A
5492	rs6273531	18	60871806	G	A	A	A	A	A
5493	rs4231879	18	60942609	G	G	G	G	G	G
5494	rs8255172	18	61321870	A	A	A	A	A	A
5495	rs8237391	18	61320141	A	C	A	A	A	A
5496	rs8237392	18	61320118	C	A	C	C	C	C
5497	rs8243395	18	61533689	G	G	G	G	G	G
5498	rs8243392	18	61534483	A	A	A	A	A	A
5499	rs3677707	18	61897032	A	G	G	G	G	G
5500	rs6381950	18	61911608	A	A	A	A	A	A
5501	rs3694537	18	62145996	C	A	C	C	C	C
5502	rs3721651	18	62505058	C	A	A	A	A	A
5503	rs8252709	18	62699178	G	G	G	G	G	G
5504	rs8243367	18	62695880	A	A	A	A	A	A
5505	rs8237402	18	62695790	A	A	G	G	G	A
5506	rs3705875	18	62914964	A	G	A	A	A	A
5507	rs4231899	18	63022572	G	A	G	G	G	A
5508	rs3720827	18	63808511	A	A	A	G	G	G
5509	rs3023468	18	64005825	G	G	G	C	C	G
5510	rs3669248	18	64125633	T	A	A	T	T	A
5511	rs6352864	18	64179675	G	C	C	C	C	C
5512	rs4211918	18	64467851	A	A	A	G	G	G
5513	rs3656801	18	64981172	G	G	G	G	G	G
5514	rs6317313	18	65038629	A	A	A	A	A	A
5515	rs3688789	18	65035105	G	A	A	A	A	G
5516	rs3684964	18	65181320	A	C	C	C	C	A
5517	rs4231907	18	65502438	A	G	G	A	A	G
5518	mCV24008503	18	67657581	G	A	G	G	G	G
5519	rs6320743	18	65694361	G	A	A	A	A	G
5520	rs4138277	18	65975417	G	G	G	A	A	G
5521	rs3724033	18	66196307	G	G	G	A	G	A
5522	rs6367649	18	66532807	G	G	G	G	G	G
5523	rs3691542	18	67000836	G	A	G	A	G	G
5524	rs3704084	18	67083465	G	A	G	A	G	A
5525	rs3088449	18	66936471	A	A	A	A	A	A
5526	rs6334085	18	67318667	G	G	G	G	G	G
5527	rs6374678	18	67518735	G	G	G	G	G	G
5528	rs3716803	18	67523287	A	G	A	G	A	G
5529	rs3675194	18	67588372	G	G	G	A	G	G
5530	rs4231913	18	67844186	G	G	G	G	G	G
5531	rs3663770	18	68130437	C	C	C	G	C	C
5532	rs3720764	18	68500356	G	G	G	A	G	G
5533	rs3700328	18	68664885	A	A	A	G	A	A
5534	rs8256630	18	68864519	A	A	A	A	A	A
5535	rs8256626	18	68864802	A	A	A	A	A	A
5536	rs3091133	18	68865739	A	A	A	A	A	A
5537	rs6397718	18	68970603	NONE	G	G	G	G	G
5538	rs3088526	18	69076778	G	G	G	G	G	G
5539	rs3658163	18	69187029	T	A	A	T	A	T
5540	rs3722524	18	69242483	G	A	A	G	A	G
5541	rs4231929	18	69391594	A	A	A	A	A	A
5542	rs3718618	18	69741786	A	A	A	G	A	A
5543	rs3669949	18	69860436	A	A	A	G	A	A
5544	rs6238338	18	70525900	G	G	G	G	G	G
5545	rs3091227	18	70195462	A	A	A	A	A	A
5546	rs3706905	18	70249607	G	A	G	A	G	A
5547	rs3688280	18	70734872	G	G	G	G	G	G

5548	rs3684593	18	71217317	G	A	G	A	G	A
5549	rs3699293	18	71217426	C	C	A	C	A	C
5550	rs6193279	18	71351453	A	A	A	A	A	A
5551	rs3669074	18	71471134	A	A	A	G	A	A
5552	rs3663078	18	71896585	A	G	A	G	A	G
5553	rs6159533	18	71978007	G	G	G	G	G	G
5554	rs6338424	18	72318410	A	A	A	A	A	A
5555	rs3702134	18	72726203	C	C	C	A	C	C
5556	rs3661900	18	72635840	G	G	A	G	A	G
5557	rs3655324	18	72561102	G	G	G	A	G	G
5558	rs3672215	18	72899203	C	C	C	G	C	C
5559	rs6335278	18	73445383	C	C	C	C	C	C
5560	mCV23234519	18	75656118	C	A	C	A	C	A
5561	rs3675243	18	73509694	A	A	G	G	G	A
5562	rs3715548	18	73713729	A	G	A	A	A	G
5563	rs6402381	18	73796074	G	G	G	G	G	G
5564	rs2020564	18	74078292	C	C	C	C	NONE	C
5565	rs3654625	18	74425314	A	A	G	G	G	A
5566	rs3687542	18	74673091	G	G	G	A	G	G
5567	rs4231934	18	74800133	A	A	A	C	A	A
5568	rs6199678	18	75430903	G	G	A	A	A	A
5569	rs3676122	18	75971130	A	A	C	A	C	A
5570	rs3656890	18	76075851	A	G	A	G	A	G
5571	rs3682892	18	76300035	G	G	A	A	A	A
5572	rs3023475	18	76343871	A	G	A	A	A	A
5573	rs3690119	18	76365872	A	G	A	A	A	A
5574	rs6381624	18	76483171	A	G	G	A	G	A
5575	rs3655845	18	76686529	A	G	A	A	A	A
5576	rs3724679	18	76549319	T	A	A	A	A	A
5577	rs3667668	18	77102667	G	G	A	G	A	G
5578	rs4231935	18	77576478	T	T	T	T	T	T
5579	mCV27555925	18	79520197	G	G	A	A	A	G
5580	rs3706601	18	77890660	G	G	G	A	G	A
5581	rs6238171	18	78528167	C	C	C	C	C	C
5582	rs3718829	18	78706072	G	A	A	A	A	G
5583	rs3715003	18	79153211	G	A	A	A	A	G
5584	rs6175715	18	79898980	G	G	G	G	G	G
5585	rs6356607	18	79974863	G	A	G	G	G	G
5586	rs3676197	18	80005623	G	A	G	G	G	G
5587	rs3720876	18	80361633	T	T	A	A	A	T
5588	rs6209100	18	80505882	G	G	G	G	G	G
5589	rs3708419	18	81109284	G	G	A	A	A	A
5590	rs4231975	18	81161882	G	G	G	G	G	G
5591	rs3671707	18	81232693	G	G	A	A	A	A
5592	rs3682618	18	81275345	G	A	A	A	A	A
5593	rs3708298	18	82040765	A	G	G	G	G	G
5594	rs6221045	18	82074573	G	G	G	G	G	G
5595	rs3664173	18	82166534	A	G	G	G	G	G
5596	rs8243362	18	83030321	A	A	A	A	A	A
5597	rs4231991	18	83061521	G	G	G	G	G	G
5598	rs4231990	18	83061630	A	A	A	A	A	A
5599	rs6242815	18	83073941	C	C	C	C	C	C
5600	rs3090314	18	83928446	A	A	A	A	A	A
5601	rs6202456	18	83970622	G	G	G	G	G	G
5602	rs3701686	18	84291891	G	A	A	A	A	A
5603	mCV24836796	18	86034550	A	G	G	A	G	G
5604	rs6279692	18	85472076	G	G	G	G	G	G
5605	rs4232000	18	85493536	G	A	A	A	A	A
5606	rs4231999	18	85493622	A	A	A	A	A	A
5607	rs3676394	18	85889302	A	G	G	G	G	G
5608	rs6171557	18	86278873	C	A	A	A	A	A
5609	rs3671719	18	86313828	G	G	G	A	G	G
5610	rs3670801	18	86537199	A	G	G	G	G	G
5611	rs6323225	18	86922399	C	C	C	C	C	C
5612	rs3716100	18	87105494	T	A	A	T	A	A
5613	rs3680424	18	87107142	A	G	G	A	G	G
5614	rs3682795	18	87361323	T	A	A	A	A	A
5615	mCV24945827	18	88626089	G	A	A	A	A	A
5616	rs6244050	18	87872724	C	C	C	C	C	C
5617	mCV22792607	18	89053497	A	T	T	T	T	A
5618	rs6248120	18	87584216	A	A	A	A	A	A
5619	rs6408078	18	88842086	G	G	G	G	G	G
5620	rs3090303	18	88913666	C	C	C	C	C	C
5621	rs4137441	18	89120861	G	A	A	A	A	G

5622	rs6282736	18	89424880	A	G	G	G	G	A
5623	mCV22762066	18	90515196	T	A	A	A	A	T
5624	mCV23657398	18	91026994	T	A	A	A	A	A
5625	mCV24763607	18	90101432	A	G	G	G	G	A
5626	mCV24766238	18	90582606	G	A	A	A	A	G
5627	rs6193060	19	60615451	G	G	G	A	G	G
5628	rs3700209	19	10197210	G	G	A	G	A	A
5629	rs4232174	19	41102683	A	G	A	A	A	A
5630	rs3704150	19	45938792	G	G	G	A	G	G
5631	rs3653771	19	23736677	A	G	G	A	G	A
5632	rs6207491	19	46010432	A	A	A	A	A	A
5633	rs8239552	19	46010249	G	G	A	G	G	A
5634	rs3658667	19	19885530	A	G	A	G	A	G
5635	rs8239563	19	46009656	G	G	A	G	G	A
5636	rs8250444	19	46007615	A	A	G	A	A	G
5637	rs3723852	19	23742644	G	G	A	G	A	G
5638	mCV24130963	19	55192	G	G	G	G	C	G
5639	mCV25314100	19	4634437	G	G	G	G	A	G
5640	rs6213403	19	3053099	A	A	A	A	A	A
5641	rs3023688	19	3243816	A	G	G	A	G	G
5642	rs6296521	19	3781610	C	C	C	C	C	C
5643	rs8236985	19	3825121	A	A	A	A	A	A
5644	rs8257628	19	3830290	A	A	A	A	A	G
5645	rs8257622	19	3830481	A	A	A	A	A	A
5646	rs4232037	19	3953685	G	G	G	G	G	G
5647	rs3671671	19	4208173	C	A	A	C	C	C
5648	rs4232036	19	4214649	C	A	A	C	C	C
5649	rs3690148	19	4229871	G	A	A	G	G	A
5650	mCV23214561	19	7219351	A	A	A	A	G	A
5651	rs6350768	19	4528690	A	G	G	A	G	G
5652	rs3713033	19	4818261	G	A	A	G	A	G
5653	rs4232029	19	4838414	C	C	C	C	C	C
5654	rs6339583	19	4987483	A	A	A	A	A	A
5655	rs6215956	19	5271216	G	G	G	G	G	G
5656	rs4232023	19	5392129	A	G	G	A	G	G
5657	rs8279948	19	5800693	A	A	A	A	A	A
5658	rs8279898	19	5803150	A	A	A	A	A	A
5659	rs6236348	19	6000186	A	G	G	A	G	G
5660	rs3091023	19	6696105	A	A	A	A	A	A
5661	rs6302797	19	6710076	C	C	C	C	C	C
5662	rs6275876	19	7118769	G	G	G	G	G	G
5663	rs3023477	19	7133376	G	G	G	G	A	A
5664	rs4232011	19	7252030	G	G	G	G	G	A
5665	mCV24585577	19	9273612	G	A	A	G	A	G
5666	rs8264743	19	7717080	C	C	C	C	C	C
5667	rs8264747	19	7717348	C	C	C	C	C	A
5668	rs8264775	19	7721031	C	C	C	C	C	C
5669	rs6335227	19	7747331	C	C	C	C	C	C
5670	mCV23008375	19	10249422	A	G	A	A	A	A
5671	rs4232045	19	8026886	A	A	A	A	A	A
5672	rs4139262	19	8018568	G	A	A	G	G	G
5673	rs4232044	19	7839674	G	G	G	G	G	G
5674	rs4232042	19	7839384	G	G	G	G	A	G
5675	rs3673624	19	8264142	A	G	G	A	A	A
5676	rs4232051	19	9084182	A	A	A	A	A	A
5677	rs4232056	19	9551732	G	C	C	G	C	G
5678	rs8267736	19	9773019	A	A	A	A	A	A
5679	rs8267764	19	9774135	G	A	G	G	G	G
5680	rs6163293	19	9899424	G	A	G	G	G	A
5681	rs3090873	19	9957707	G	G	G	G	G	G
5682	rs3023075	6	78893070	C	C	C	C	C	C
5683	rs6285845	19	10909330	A	G	A	A	A	G
5684	rs4232058	19	11028367	G	A	G	G	G	A
5685	rs3674862	19	11194496	G	A	A	G	A	A
5686	rs3688406	19	11531705	A	G	A	A	A	A
5687	rs3671328	19	11873778	G	G	A	G	A	G
5688	rs3705736	19	11959885	C	C	A	C	A	C
5689	rs6349685	19	12013775	G	G	A	G	A	G
5690	rs6237846	19	12452484	C	C	A	C	A	A
5691	rs3694570	19	13056716	G	G	A	G	A	A
5692	rs6306968	19	13598306	A	A	G	A	G	A
5693	rs4137097	19	13599336	G	G	C	G	C	G
5694	rs4232068	19	13726607	T	T	T	T	T	T
5695	rs3659857	19	14072812	A	A	G	A	G	A

5696	rs3661175	19	14073038	G	G	A	G	A	G
5697	mCV24419235	19	16235572	A	A	G	G	A	A
5698	rs3704158	19	14244274	C	C	A	C	A	C
5699	rs4136257	19	14643563	G	A	A	G	A	G
5700	rs6268186	19	14655411	A	G	G	A	G	A
5701	rs3661215	19	14795903	A	G	G	A	G	G
5702	rs4232072	19	15182862	T	T	T	T	T	T
5703	rs4232071	19	15183141	NONE	C	C	C	C	C
5704	rs6319765	19	15759352	G	G	G	G	G	G
5705	rs3662712	19	15964901	G	G	G	G	G	G
5706	rs3669192	19	16124735	G	G	A	A	A	G
5707	rs3686467	19	16159229	G	G	A	A	G	G
5708	rs6172420	19	16716062	G	G	G	G	A	G
5709	rs8267682	19	16858147	G	A	A	A	G	G
5710	rs8267576	19	16873839	G	G	G	G	G	G
5711	rs8267392	19	16954644	A	G	G	G	G	A
5712	rs4232099	19	16972924	A	A	A	A	G	A
5713	rs6211545	19	16986855	A	A	A	A	G	A
5714	rs8267308	19	16991018	G	G	G	G	A	G
5715	rs8267310	19	16991104	G	G	G	G	G	G
5716	rs3681194	19	17584355	A	A	A	G	A	G
5717	rs6223359	19	17804343	A	A	A	G	A	G
5718	rs4140311	19	17884605	G	A	A	A	G	A
5719	rs3673310	19	17976672	G	A	A	A	G	G
5720	rs3694495	19	19168174	A	A	A	T	A	A
5721	rs6411707	19	19172402	G	G	G	A	G	G
5722	rs3720318	19	19458954	G	A	G	G	G	A
5723	rs3723753	19	19502328	T	T	T	A	T	A
5724	rs3023480	19	19624890	A	A	A	C	A	C
5725	rs6380630	19	19680108	G	G	G	G	G	G
5726	rs3090812	19	19695717	C	C	C	C	C	C
5727	rs8239254	19	19915169	T	T	T	T	T	T
5728	rs3668911	19	19915494	A	A	A	G	A	G
5729	rs8239869	19	19999544	C	C	C	C	C	C
5730	rs6223813	19	20066123	A	G	A	A	A	A
5731	rs3726449	19	20347272	A	A	A	A	A	C
5732	rs3682187	19	20738869	G	G	A	A	A	G
5733	rs6245242	19	20748732	A	A	A	G	A	A
5734	rs6392565	19	21461474	A	G	A	A	A	G
5735	rs3707812	19	21674170	G	G	G	A	G	G
5736	rs3672759	19	21707322	A	A	T	A	T	A
5737	rs3653630	19	21729294	C	C	C	A	C	C
5738	rs6342493	19	21905509	A	G	A	G	A	G
5739	rs3090137	19	22071353	G	G	G	A	G	G
5740	rs4232102	19	22438463	A	A	A	A	A	A
5741	rs6217094	19	22493246	A	A	C	A	C	A
5742	rs4232108	19	22993421	G	A	G	G	G	G
5743	rs3687901	19	23001090	G	G	A	G	A	G
5744	rs3688671	19	23001257	A	A	G	A	G	A
5745	rs3660275	19	23053220	A	A	A	A	A	G
5746	rs3697576	19	23088139	G	A	G	A	G	A
5747	rs6259521	19	23219454	T	T	A	T	A	T
5748	rs4232112	19	23245440	G	G	A	G	A	G
5749	rs4232111	19	23245416	C	C	A	C	A	C
5750	rs3670355	19	24245675	G	G	A	G	A	G
5751	rs3720897	19	24396755	A	G	A	A	A	A
5752	rs3691133	19	24661375	G	A	G	G	G	G
5753	rs4232120	19	24709314	C	C	C	C	C	C
5754	rs3701438	19	24941259	A	A	C	A	C	A
5755	rs6361137	19	24957266	A	A	G	A	G	A
5756	rs6166640	19	25059711	A	G	A	G	A	G
5757	rs3724885	19	25333658	A	G	G	A	G	G
5758	rs3669236	19	25643608	G	G	G	A	G	G
5759	rs6186902	19	25999644	A	G	A	A	A	A
5760	rs3090325	19	26007713	G	A	A	A	A	G
5761	rs6254371	19	26153729	A	G	A	A	A	A
5762	rs3089211	19	26389006	G	G	G	G	G	G
5763	rs4232127	19	26528340	G	G	G	G	G	G
5764	rs6291559	19	26849645	A	A	G	G	G	A
5765	rs3723418	19	27042361	C	C	A	A	A	C
5766	rs6238322	19	27335880	A	G	A	A	A	A
5767	rs3717280	19	27376721	A	A	G	G	G	G
5768	mCV24505422	19	29964780	G	A	G	A	G	A
5769	rs6312543	19	28172801	G	G	G	G	G	G

5770	rs6153867	19	28191034	A	A	A	A	A	A
5771	rs3714482	19	28235010	A	G	G	G	G	G
5772	rs4232130	19	28441324	A	A	A	A	A	A
5773	rs6366840	19	28448526	C	A	A	A	A	A
5774	rs3089688	19	28700499	G	G	G	G	G	G
5775	rs6172077	19	28797164	A	A	A	A	A	A
5776	rs3695543	19	29301588	A	T	T	T	T	T
5777	rs4232135	19	29395022	A	A	A	A	A	A
5778	mCV24995143	19	31560624	G	G	G	G	G	G
5779	rs3704503	19	29618618	G	A	G	A	G	A
5780	rs6224900	19	29848562	A	A	G	A	G	A
5781	rs6352879	19	30253148	G	G	G	G	G	G
5782	rs4232140	19	30651530	A	A	A	A	A	A
5783	rs3681148	19	30716634	A	G	G	G	G	A
5784	rs3693805	19	30840505	C	G	G	G	G	G
5785	rs6344448	19	30816535	T	A	A	A	A	T
5786	rs4232148	19	31293711	C	C	C	C	C	C
5787	rs3685192	19	31413334	A	G	G	A	G	G
5788	rs3686450	19	31413557	G	C	C	G	C	C
5789	rs6237466	19	31672951	A	A	A	T	A	A
5790	rs4232151	19	31982985	G	G	C	G	C	G
5791	rs4232150	19	31983142	G	G	A	G	A	G
5792	rs3089994	19	32190404	C	C	C	C	C	C
5793	rs3672117	19	32197321	A	G	G	A	G	G
5794	rs6269629	19	32208756	C	A	A	C	A	A
5795	rs6283850	19	32369068	A	A	A	A	A	A
5796	rs6200234	19	33442060	A	C	A	A	A	C
5797	rs4232159	19	33785778	G	G	G	G	G	G
5798	rs4232158	19	33783411	G	G	G	G	G	G
5799	rs3089343	19	34209019	G	G	G	G	G	G
5800	mCV27551335	19	36511227	G	G	A	A	A	G
5801	rs6372028	19	34954648	C	C	C	C	C	A
5802	rs6339069	19	35322257	G	G	G	G	G	G
5803	rs3705022	19	35600828	T	T	T	A	T	T
5804	rs3691354	19	35613101	A	A	A	G	A	A
5805	rs3710581	19	35620543	C	G	C	C	C	C
5806	mCV24526730	19	36968850	C	C	C	G	C	C
5807	rs6213166	19	36105184	A	A	A	A	A	A
5808	rs3713879	19	36118341	G	G	G	A	G	G
5809	rs3726962	19	36228470	A	A	A	G	A	A
5810	rs6371145	19	36282623	G	G	G	A	G	G
5811	rs3089834	19	36672715	A	A	A	A	A	A
5812	rs8248714	19	36922630	G	G	G	G	G	G
5813	rs8248762	19	36925420	A	A	A	A	A	A
5814	rs3653886	19	36962492	A	A	G	A	G	A
5815	rs6337024	19	37062964	G	G	G	A	G	G
5816	rs3660293	19	37288799	A	A	A	G	A	A
5817	rs3699974	19	37297277	A	A	A	G	A	A
5818	rs3710829	19	37393165	A	A	A	G	A	G
5819	mCV23121032	19	40316204	G	A	G	G	G	G
5820	rs6185741	19	38062272	G	G	G	C	G	G
5821	rs6162778	19	38066937	C	C	C	A	C	C
5822	rs3703185	19	38197686	C	C	C	A	C	C
5823	rs3718223	19	38326060	G	G	G	A	G	G
5824	rs8247843	19	38574094	C	C	C	C	C	C
5825	rs8247874	19	38595602	A	A	A	C	A	A
5826	rs8247885	19	38596872	A	A	A	A	A	A
5827	rs8247896	19	38608452	A	A	A	G	A	A
5828	rs8247901	19	38608577	C	C	C	C	C	C
5829	rs6289489	19	38811626	A	A	A	A	A	A
5830	rs3697139	19	39427196	A	A	T	A	T	A
5831	rs3672423	19	39425502	G	G	C	C	C	G
5832	rs3655407	19	39594395	C	C	A	A	A	C
5833	rs6350637	19	39650073	A	A	A	G	A	A
5834	rs3665780	19	39653074	C	C	C	A	C	C
5835	rs3677115	19	39919326	G	G	G	A	G	A
5836	rs6328964	19	40359209	G	A	G	G	G	G
5837	rs4232165	19	40547318	C	C	C	C	C	C
5838	rs3668166	19	40639328	G	A	G	A	G	G
5839	rs3679068	19	40685040	G	A	G	A	G	A
5840	rs3653396	19	40978193	G	G	G	A	G	G
5841	rs6396860	19	41231342	G	G	G	A	G	G
5842	rs3697841	19	41289555	G	G	G	A	G	G
5843	rs3696425	19	41364799	A	A	A	G	A	A

5844	rs4232180	19	41527482	A	A	A	A	A	A
5845	rs3656289	19	41749395	A	A	A	G	A	A
5846	rs6238526	19	42010157	G	G	G	G	G	G
5847	rs3703918	19	42106509	G	A	G	G	A	G
5848	rs3687275	19	42406846	A	A	C	C	A	A
5849	rs3673976	19	42665672	G	G	G	A	G	G
5850	rs4232185	19	42831942	C	C	C	C	C	C
5851	rs3695752	19	43015886	G	G	G	A	G	G
5852	mCV23390953	19	45356002	A	G	A	A	G	A
5853	rs8250750	19	43134869	G	A	G	G	A	G
5854	rs8245094	19	43135529	C	A	C	C	A	C
5855	rs8237002	19	43147635	A	A	A	G	A	A
5856	rs8242070	19	43154339	G	G	G	A	G	G
5857	rs8242053	19	43162445	A	G	G	A	G	A
5858	rs8237008	19	43172940	G	C	C	C	C	G
5859	rs6245539	19	43186537	G	G	G	A	G	G
5860	rs3654725	19	43253748	A	A	A	G	A	A
5861	rs3676927	19	43287520	A	A	A	C	A	A
5862	rs4232207	19	43409816	A	A	A	A	A	A
5863	rs3090951	19	43410999	G	G	G	G	G	G
5864	rs3694723	19	43417782	A	A	A	G	A	A
5865	rs3726430	19	43476737	C	A	A	A	A	C
5866	rs6276649	19	43763446	A	C	A	A	C	C
5867	mCV23045722	19	46489003	A	A	G	G	A	G
5868	rs3675915	19	44302199	C	C	A	C	C	C
5869	rs3656005	19	44674391	G	G	G	A	G	G
5870	rs3711994	19	44870299	A	A	A	G	A	A
5871	rs6235674	19	45256117	G	G	G	G	G	G
5872	rs4232223	19	45477448	C	C	C	C	C	C
5873	rs3722316	19	45576248	G	G	G	A	G	G
5874	rs4232232	19	45626576	G	G	G	G	G	G
5875	rs3654209	19	46136258	A	A	A	G	A	A
5876	rs6362473	19	46249049	G	G	G	A	G	G
5877	rs3705264	19	46315574	A	G	G	A	G	G
5878	rs3658160	19	46364695	C	A	A	C	A	A
5879	rs3655896	19	46906761	C	C	C	A	C	C
5880	rs8257524	19	47199155	C	C	C	C	C	C
5881	rs8257576	19	47204097	A	A	A	A	A	A
5882	rs8257588	19	47207858	A	A	G	A	A	A
5883	rs8257591	19	47208085	G	G	G	G	G	G
5884	rs8257619	19	47208475	A	A	A	T	A	A
5885	rs3023496	19	47482330	A	A	A	G	A	A
5886	rs6349824	19	47507066	G	G	G	A	G	G
5887	mCV24595223	19	49824850	C	C	A	A	C	C
5888	mCV23150139	19	49957079	A	A	G	G	A	A
5889	rs3660360	19	47718339	G	G	A	A	G	A
5890	rs3664825	19	47721918	G	G	A	A	G	A
5891	rs3089112	19	47730230	G	G	G	G	G	G
5892	rs3089113	19	47730255	C	C	C	C	C	C
5893	mCV23151545	19	50014396	A	A	G	G	A	A
5894	mCV23151923	19	50027917	G	G	A	A	G	G
5895	rs4232253	19	48149686	C	C	C	C	C	C
5896	rs4232254	19	48149798	T	T	T	T	T	T
5897	rs6153081	19	48731684	G	G	G	G	G	G
5898	mCV22978357	19	50298700	G	G	A	A	A	G
5899	rs6194426	19	49475053	A	G	G	G	G	G
5900	rs3676974	19	49640350	G	A	A	A	A	A
5901	mCV22979960	19	50370537	C	G	G	C	G	G
5902	mCV22979964	19	50370702	G	A	A	G	A	A
5903	rs3699073	19	49885821	T	T	A	A	T	T
5904	rs3692717	19	49920513	A	A	G	G	A	A
5905	rs6177228	19	49923803	G	G	A	A	G	G
5906	rs3716572	19	50071598	A	A	G	G	A	A
5907	mCV23069037	19	51646426	G	G	G	A	G	G
5908	mCV22608007	19	51798964	A	A	A	G	A	G
5909	rs3666302	19	51198027	G	A	A	A	A	A
5910	mCV23069572	19	52162185	A	A	A	G	A	G
5911	rs3721874	19	51317746	C	G	G	G	G	G
5912	rs3091059	19	51622213	A	A	A	A	A	A
5913	rs3718102	19	51621238	G	A	A	A	A	A
5914	rs8275600	19	52355953	A	A	A	G	A	G
5915	rs8236997	19	52356501	G	G	G	A	G	A
5916	rs3722335	19	52361173	A	A	A	G	A	G
5917	rs8275718	19	52362498	G	G	G	G	G	G

5918	rs3672780	19	52365729	G	G	G	A	G	A
5919	rs8276019	19	52368205	A	A	A	A	A	A
5920	mCV23072435	19	53100821	C	C	C	C	C	C
5921	rs4138008	19	52583233	G	G	G	A	G	A
5922	rs4232257	19	52606316	C	C	C	C	C	C
5923	rs6257938	19	52744319	G	G	G	C	G	C
5924	rs6304326	19	52797648	C	C	C	A	C	C
5925	rs6326311	19	53327828	G	G	G	G	G	G
5926	mCV24736382	19	54207735	G	G	G	A	G	G
5927	rs3691357	19	53609409	A	A	A	G	A	A
5928	rs6185483	19	54609252	A	G	G	G	G	G
5929	rs3655171	19	54628331	G	A	G	A	G	G
5930	rs6303591	19	54655608	A	G	A	G	A	A
5931	rs3654713	19	54392587	G	G	G	A	G	G
5932	rs3660143	19	54403033	G	G	G	A	G	G
5933	rs3709671	19	54991850	G	G	G	A	G	A
5934	rs6222014	19	54905003	G	G	G	A	G	G
5935	rs3089427	19	55767221	A	A	A	A	A	A
5936	rs3686750	19	55733126	C	A	A	A	A	A
5937	rs3023497	19	55994689	G	G	A	G	A	G
5938	rs3663566	19	56250823	G	A	A	G	A	G
5939	rs6211533	19	56380821	A	A	C	A	C	A
5940	rs4222107	19	56421779	G	G	G	G	G	G
5941	rs3719692	19	56997687	C	A	C	A	C	C
5942	rs3718687	19	56865524	A	G	G	G	G	A
5943	rs3716179	19	56832728	C	C	A	C	A	C
5944	rs6259462	19	57286731	G	G	G	A	G	G
5945	rs3710053	19	57518787	A	A	A	G	A	A
5946	rs4232264	19	57523483	A	A	A	A	A	A
5947	rs3694663	19	57539773	C	C	C	A	C	C
5948	rs3706406	19	57861401	G	G	G	C	G	G
5949	rs3711945	19	58109941	C	C	C	A	C	C
5950	rs6183889	19	58116358	A	G	G	A	G	A
5951	rs3676948	19	58190157	A	G	G	G	G	A
5952	rs4232265	19	58194641	A	A	A	A	A	A
5953	rs6191324	19	58720409	A	G	G	A	G	A
5954	rs3658400	19	58854931	A	A	A	C	A	A
5955	rs3712604	19	59328147	G	G	G	A	G	G
5956	rs4232268	19	59540540	C	C	C	A	C	C
5957	rs4232275	19	59540381	NONE	G	G	A	G	G
5958	mCV23482939	19	60432097	A	A	A	G	A	A
5959	rs3718998	19	59881040	A	A	A	G	A	A
5960	rs3694467	19	59928762	A	A	A	G	A	A
5961	rs6192258	19	60353870	G	G	G	G	G	G
5962	rs3692709	19	60343567	G	G	G	A	G	G
5963	rs6228270	19	60191556	G	G	G	C	G	G
5964	rs6364684	2	106701807	A	G	G	A	G	A
5965	rs4223152	2	52654660	A	A	A	A	A	A
5966	rs3712058	2	57039569	A	A	A	G	A	A
5967	rs4223204	2	68149027	C	C	C	C	C	C
5968	rs3656787	2	109026034	G	G	A	G	G	G
5969	rs3711532	2	106670167	G	G	A	G	G	G
5970	mCV25103560	2	5917582	C	G	C	G	C	C
5971	rs6220817	2	3041581	G	A	A	A	A	G
5972	rs3713997	2	3181746	A	G	G	G	G	G
5973	rs6411822	2	3273268	G	A	A	A	A	A
5974	rs3680350	2	3910588	G	A	A	A	A	G
5975	rs4136817	2	4258057	A	G	G	G	G	A
5976	rs4222934	2	4536999	A	A	A	A	A	A
5977	rs6308296	2	4701800	G	A	A	A	A	A
5978	rs4222938	2	4855239	G	G	G	G	G	G
5979	rs3692487	2	5403215	G	A	A	A	A	A
5980	rs3678168	2	5606042	C	C	C	C	C	C
5981	rs6331390	2	5921915	A	G	A	G	A	G
5982	rs3676722	2	6075725	G	A	G	A	G	G
5983	rs3695983	2	6179935	G	A	G	A	G	G
5984	rs6368110	2	6355104	A	A	A	A	A	A
5985	rs4222951	2	6491568	A	A	A	A	A	A
5986	rs3089292	2	6638777	G	G	G	G	G	G
5987	rs6184395	2	6970424	A	A	A	A	A	A
5988	rs6318892	2	6996747	A	A	A	A	A	A
5989	rs6332299	2	7592522	A	A	A	A	A	A
5990	rs6228421	2	8099809	G	A	G	G	G	G
5991	rs3090363	2	8858648	A	A	A	A	A	A

5992	rs6155402	2	9189394	G	G	G	G	G	G
5993	rs3090361	2	9561868	G	A	G	G	G	A
5994	rs4222956	2	9649100	G	G	G	G	G	G
5995	rs6302769	2	9735202	A	A/G	A/G	A	A/G	A
5996	rs3696091	2	10171941	A	A	G	G	G	A
5997	rs6159599	2	10396791	C	A	A	A	A	A
5998	rs3719255	2	10706792	G	A	A	A	A	G
5999	rs6240512	2	10929543	A	G	G	G	G	A
6000	rs3724417	2	11068501	A	G	G	G	G	G
6001	rs4222971	2	11684187	A	A	A	A	A	A
6002	rs3090977	2	11684460	G	G	G	G	G	G
6003	rs3090808	2	11955448	A	A	A	A	A	A
6004	rs4137557	2	12017866	A	G	G	G	G	A
6005	rs4222975	2	12057896	A	A	A	A	A	A
6006	rs6172408	2	12582823	A	A	A	A	A	A
6007	rs6297721	2	12791291	A	A	A	A	A	A
6008	rs4222977	2	12952322	C	C	C	C	C	C
6009	rs8238698	2	13527336	G	G	G	G	G	G
6010	rs8238707	2	13527746	A	A	A	A	A	A
6011	rs4222980	2	13602788	G	G	G	G	G	G
6012	rs6315134	2	14199316	A	A	A	A	A	A
6013	rs3091183	2	14282434	A	A	A	A	A	A
6014	mCV25433152	2	17967514	G	G	G	G	A	G
6015	rs6357799	2	14912730	A	A	A	A	A	A
6016	rs6207464	2	15881696	G	G	G	G	G	G
6017	rs6276392	2	16464264	A	A	A	A	A	A
6018	rs6204508	2	16985143	G	G	G	G	G	G
6019	rs6367650	2	17555817	A	A	A	A	A	A
6020	rs6218422	2	18817528	A	G	A	A	G	A
6021	rs4222990	2	18903152	G	G	G	G	G	G
6022	rs6236971	2	19843793	A	G	A	A	G	A
6023	rs6292983	2	20073059	G	A	G	G	A	G
6024	rs3688854	2	20337457	A	A	A	G	A	A
6025	rs3674936	2	20427488	G	G	G	A	G	G
6026	rs6165425	2	20814394	A	G	A	A	G	G
6027	rs4223007	2	21263714	C	C	C	C	C	C
6028	rs6215138	2	21581189	G	A	G	G	A	A
6029	rs6369767	2	22555462	A	G	A	A	G	G
6030	rs6193345	2	23303787	A	G	A	A	G	G
6031	mCV23574676	2	26837911	C	G	C	C	G	C
6032	rs8240181	2	24009528	G	G	G	G	G	G
6033	rs6301542	2	24169227	A	G	A	A	G	A
6034	rs8247993	2	24307886	A	A	A	A	A	A
6035	rs8241287	2	24309660	C	C	C	C	C	C
6036	rs6286688	2	24495175	G	A	G	G	A	G
6037	rs4223026	2	25160332	G	G	G	G	G	G
6038	rs8240079	2	25273724	A	G	A	A	G	A
6039	rs8250941	2	25427137	A	G	A	A	G	A
6040	rs8256021	2	25433522	G	A	G	G	A	G
6041	rs8243214	2	25435142	G	C	G	G	C	G
6042	rs8243211	2	25435591	G	A	G	G	A	G
6043	rs6164049	2	25499805	A	G	A	A	G	A
6044	rs3713143	2	26235083	G	A	A	A	A	A
6045	rs4223055	2	26310870	A	A	A	A	A	A
6046	rs3681655	2	26328377	A	A	C	C	A	C
6047	mCV24983191	2	30147850	A	G	G	G	G	G
6048	rs8243144	2	26874116	G	G	G	G	G	G
6049	rs8243162	2	26876951	G	G	G	G	G	G
6050	rs8255309	2	26877967	A	A	A	A	A	A
6051	rs3718405	2	27249691	C	A	A	A	A	A
6052	rs4223066	2	27403982	G	G	G	G	G	G
6053	rs4223068	2	27496085	C	C	C	C	C	C
6054	mCV23209429	2	31240017	C	A	C	C	A	A
6055	rs6181760	2	27847598	A	G	G	G	G	G
6056	rs6321858	2	28295174	A	G	G	G	G	G
6057	rs6308258	2	28506214	G	A	A	A	A	A
6058	rs4223075	2	28869030	A	A	A	A	A	A
6059	rs4223077	2	28869168	A	A	A	A	A	A
6060	rs4135557	2	29740065	G	A	A	A	A	A
6061	rs6372246	2	29750924	A	G	G	G	G	G
6062	rs3715001	2	30282017	A	G	G	G	G	G
6063	rs4223083	2	30362149	A	A	A	A	A	A
6064	rs3689602	2	30372859	A	C	A	C	C	C
6065	rs3089256	2	30547516	G	G	G	G	G	G

6066	rs6389436	2	30779061	A	A	A	A	A	A
6067	rs4223088	2	30852538	G	G	G	G	G	G
6068	rs4223089	2	31932722	C	C	C	C	C	C
6069	rs6171513	2	32362610	G	G	G	G	G	G
6070	rs6301234	2	32498207	G	G	G	G	G	G
6071	rs4223103	2	32749153	A	A	A	A	A	A
6072	rs4223102	2	32749275	G	G	G	G	G	G
6073	rs4223105	2	33690256	A	A	A	A	A	A
6074	rs6324892	2	33781261	A	A	A	A	A	A
6075	rs6356428	2	34589679	A	C	A	A	C	C
6076	mCV25370550	2	33902810	A	G	A	A	G	G
6077	rs4223114	2	35148160	G	G	G	G	G	G
6078	rs3683729	2	35246560	G	G	G	G	G	G
6079	rs6375627	2	35604362	A	A	A	A	A	A
6080	rs6398097	2	35895968	A	A	A	A	A	A
6081	rs4223123	2	35996244	G	G	G	G	G	G
6082	rs3699934	2	36061667	A	A	A	C	A	A
6083	rs3723453	2	36601726	A	A	A	C	A	A
6084	rs6230757	2	37054789	C	C	C	C	C	C
6085	rs3666019	2	37424334	A	A	A	G	A	A
6086	rs3709811	2	37617104	A	A	A	G	A	A
6087	rs3022883	2	37711241	C	C	C	A	C	C
6088	rs6247199	2	37891792	A	A	A	A	A	A
6089	rs3088950	2	38300034	A	A	A	A	A	A
6090	rs3682314	2	38523255	A	A	A	G	A	A
6091	rs3665986	2	38575067	C	C	C	G	C	C
6092	rs6169841	2	39014618	C	C	C	C	C	C
6093	rs4223132	2	39121478	G	G	G	G	G	G
6094	rs3668077	2	39468205	G	G	G	A	G	G
6095	rs6313371	2	39556000	A	A	A	G	A	A
6096	rs3710255	2	39693671	A	A	A	G	A	A
6097	rs6323034	2	40300906	G	G	G	A	G	G
6098	rs3668871	2	40302014	G	G	G	A	G	G
6099	rs6324652	2	40759530	T	T	T	A	T	T
6100	rs3672727	2	40901509	A	A	A	C	A	A
6101	rs3714900	2	40929271	G	G	G	A	G	G
6102	rs4136879	2	41400045	A	A	A	G	A	A
6103	rs6404799	2	41934264	A	A	A	T	A	T
6104	rs3672528	2	42361103	A	A	A	C	A	C
6105	rs3712270	2	42643097	G	G	G	C	G	C
6106	rs6288325	2	42846125	C	C	C	A	C	A
6107	mCV22297233	2	44548310	C	C	C	A	C	C
6108	rs6295520	2	43034728	A	A	A	T	A	T
6109	rs3695528	2	43120744	A	A	A	G	A	G
6110	rs6259718	2	44054897	A	A	A	A	A	A
6111	rs3680197	2	44168883	A	A	A	G	A	G
6112	rs3723568	2	44180718	A	A	A	G	A	G
6113	mCV25122694	2	45928263	A	A	A	G	A	A
6114	rs3669981	2	45081013	G	G	G	A	G	G
6115	rs3673491	2	45084457	G	G	G	A	G	G
6116	rs3675860	2	45084855	A	A	A	C	A	A
6117	rs6282473	2	45112531	A	A	A	G	A	A
6118	rs6209424	2	45627021	C	C	C	A	C	C
6119	rs3718404	2	45839971	A	A	A	G	A	A
6120	rs3721831	2	46035946	A	A	A	T	A	A
6121	rs3711308	2	46189006	T	T	T	A	T	T
6122	rs6168090	2	46264289	C	C	C	C	C	C
6123	rs6265423	2	47124603	C	G	G	C	G	G
6124	rs3699238	2	47356753	A	G	G	A	G	A
6125	rs3720745	2	47357957	C	C	C	A	C	A
6126	rs3677201	2	47958113	A	G	G	G	G	G
6127	rs6314935	2	47987258	C	C	C	A	C	A
6128	rs3675399	2	48355812	A	G	G	A	G	G
6129	rs6380260	2	48500634	A	C	C	C	C	A
6130	rs4223134	2	48864503	A	A	A	A	A	A
6131	rs4223140	2	49219001	G	G	G	G	G	G
6132	rs3089639	2	49358000	G	G	G	G	G	G
6133	rs6204382	2	49477777	G	A	A	A	A	A
6134	rs3679976	2	49667282	G	A	A	A	A	A
6135	rs3713055	2	50225113	C	A	A	A	A	C
6136	rs4223145	2	50240277	G	G	G	G	G	G
6137	rs6269713	2	51183061	A	A	A	T	A	A
6138	rs3678632	2	51184989	A	A	A	G	A	G
6139	rs3720957	2	51356779	C	C	C	G	C	C

6140	rs3725341	2	51425686	G	G	G	A	G	G
6141	rs3662008	2	51765899	G	G	G	A	G	G
6142	rs6395698	2	51875277	G	G	G	A	G	G
6143	rs4223147	2	52081878	A	A	A	A	A	A
6144	rs4223149	2	52082115	A	A	A	A	A	A
6145	rs3675868	2	52201209	A	A	A	T	A	A
6146	rs3677306	2	52713489	G	G	G	A	G	G
6147	rs6229455	2	53288469	A	A	A	C	A	A
6148	rs6354206	2	53333789	G	G	G	A	G	G
6149	rs3665866	2	53431535	T	T	T	A	T	T
6150	rs3666514	2	53431677	A	A	A	G	A	A
6151	rs3722454	2	53801198	A	A	A/G	G	A/G	A
6152	rs6293999	2	53801861	G	G	G	A	G	G
6153	rs3658919	2	54112475	A	A	A	G	A	A
6154	rs3654841	2	54484670	G	G	G	A	G	G
6155	rs3686229	2	54584766	G	G	G	A	G	G
6156	mCV25039989	2	56783206	A	A	A	T	A	A
6157	rs6201706	2	55367289	G	G	G	A	G	G
6158	rs4223159	2	55400499	A	A	A	A	A	A
6159	rs3696517	2	55634236	G	G	G	A	G	G
6160	rs3699909	2	55750856	G	G	G	A	G	G
6161	rs3718711	2	56489184	G	G	G	A	G	G
6162	rs3655184	2	56525578	A	A	A	G	A	A
6163	rs3662347	2	56550930	A	A	A	T	A	A
6164	rs3671741	2	56555351	T	T	T	A	T	T
6165	rs6253453	2	56869272	G	G	G	A	G	G
6166	rs8240489	2	57065349	G	G	G	G	G	G
6167	rs8240479	2	57067807	A	A	A	A	A	A
6168	rs3707138	2	57156202	T	T	T	A	T	T
6169	rs3727027	2	57157059	A	A	A	T	A	A
6170	rs3663027	2	57613222	A	A	A	G	A	A
6171	rs6268714	2	57665023	G	G	G	A	G	G
6172	rs6212192	2	57688265	G	G	G	G	G	G
6173	rs4223175	2	58086367	G	G	G	G	G	G
6174	rs3672719	2	58142938	C	C	C	G	C	C
6175	rs3704104	2	59370950	G	G	G	G	G	G
6176	rs3708975	2	59539084	A	G	G	G	G	G
6177	rs6195225	2	59776923	A	A	A	A	A	A
6178	rs3683761	2	59848326	A	G	G	G	G	G
6179	rs6261532	2	60121932	A	A	A	A	A	A
6180	rs4223185	2	60190997	G	G	G	G	G	G
6181	rs3660779	2	60586963	G	A	A	A	G	A
6182	rs6292751	2	60767085	G	A	A	A	A	A
6183	rs6387861	2	61516099	A	A	A	A	A	A
6184	rs4223188	2	61738506	A	A	A	A	A	A
6185	rs4223189	2	61738719	G	A	A	A	G	A
6186	rs3688501	2	61878785	G	A	A	A	G	A
6187	rs6318340	2	62101002	G	A	A	A	G	G
6188	rs6287471	2	62282583	A	A	A	A	A	A
6189	rs8252866	2	62351073	A	A	A	A	A	A
6190	rs3022886	2	62583970	G	A	G	G	A	A
6191	rs3089896	2	62584025	G	G	G	G	G	G
6192	rs3709716	2	62590944	C	C	C	G	C	C
6193	mCV24799807	2	64415531	C	A	A	A	A	A
6194	rs6391732	2	62941643	C	A	A	A	C	C
6195	rs6190548	2	62996486	G	A	A	A	G	G
6196	rs3664317	2	63787792	A	G	G	G	A	A
6197	rs6314726	2	64051479	C	A	A	A	C	C
6198	rs4223190	2	64545599	G	G	G	G	G	G
6199	rs3676478	2	64680614	G	A	A	A	G	G
6200	rs6302554	2	64797248	G	G	G	G	A	G
6201	rs3653963	2	64885735	A	C	C	C	C	A
6202	mCV25071967	2	66791415	A/T	A	A	A	A	A
6203	rs6274192	2	65296447	C	G	G	G	C	G
6204	rs6153871	2	65617313	A	A	A	A	A/G	A
6205	rs3715385	2	66288788	C	A	C	A	C	A
6206	rs6236226	2	66352120	G	A	G	A	G	A
6207	rs3723010	2	66439757	A	C	A	C	A	C
6208	rs3089671	2	66702321	A	A	A	A	A	A
6209	rs6172625	2	66869470	G	A	A	A	A	A
6210	mCV23778822	2	68601890	G	G	G	G	A	G
6211	rs3022885	2	67031499	G	G	G	G	A	G
6212	rs6177630	2	67159885	A	G	A	G	G	G
6213	rs3710978	2	67652238	A	G	NONE	G	A	G

6214	rs3689965	2	67824985	A	G	A	G	G	G
6215	rs6381994	2	68188374	A	A	A	A	G	A
6216	mCV25429456	2	69874398	T	A	A	T	A	A
6217	rs3726227	2	68782769	A	T	A	T	T	T
6218	rs6371268	2	68927682	G	G	A	A	A	G
6219	rs3022887	2	69021770	G	G	A	A	A	G
6220	rs3699027	2	69039268	G	G	A	A	A	G
6221	rs8236495	2	69177795	G	G	G	G	G	G
6222	rs8263587	2	69185883	A	G	A	G	G	G
6223	rs6218423	2	69197466	A	A	A	A	A	A
6224	rs6219012	2	69197574	A	A	A	A	A	A
6225	rs8263432	2	69215943	C	C	C	C	C	C
6226	rs8263100	2	69263952	G	G	G	G	G	G
6227	rs8263180	2	69268363	A	A	A	A	A	A
6228	rs8263075	2	69280800	G	G	G	G	G	G
6229	rs8263077	2	69281085	G	G	G	G	G	G
6230	rs8263080	2	69281486	G	G	A	G	A	G
6231	rs6275559	2	69663240	A	C	C	A	C	C
6232	rs4223211	2	69735480	T	A	A	T	A	A
6233	rs3657805	2	69824112	NONE	NONE	A	G	A	G
6234	rs4136610	2	70090167	G	A	A	G	A	A
6235	rs6165818	2	70178096	T	A	A	T	A	A
6236	rs6170389	2	70431018	G	A	A	G	A	A
6237	rs3091213	2	70539918	A	A	A	A	A	A
6238	rs6399973	2	70690980	A	A	A	A	A	A
6239	rs3682843	2	71050924	A	G	G	A	A	G
6240	rs3088797	2	71119734	G	G	G	G	G	G
6241	rs4223216	2	71168869	G	G	G	A	A	G
6242	rs4223213	2	71169017	T	T	T	T	T	T
6243	rs6181433	2	71188547	G	G	G	A	A	G
6244	rs3670752	2	71268820	G	G	G	A	A	G
6245	rs3664661	2	71492729	G	A	A	G	G	A
6246	rs6295014	2	71668506	A	C	A	A	A	C
6247	rs3663969	2	71701325	A	T	A	A	A	T
6248	rs3658672	2	71779572	A	G	G	G	G	G
6249	rs8240600	2	72200140	G	G	G	G	G	G
6250	rs4223219	2	73231904	A	A	A	A	A	A
6251	rs4223220	2	73457252	G	G	G	G	G	G
6252	rs6322742	2	73803980	T	T	T	T	T	T
6253	rs6367022	2	73969390	A	A	A	G	A	A
6254	rs4136564	2	74107900	A	A	G	A	G	A
6255	rs3691865	2	74130281	G	G	A	G	A	G
6256	rs3689263	2	74205289	A	A	A	G	A	A
6257	rs3710094	2	74253437	A	A	G	G	G	A
6258	rs4223228	2	74614126	A	A	A	A	A	A
6259	rs4223230	2	74614249	C	C	C	C	C	C
6260	rs3091097	2	74658694	T	T	T	T	T	T
6261	rs3090066	2	74813576	G	G	G	G	G	G
6262	rs3696052	2	74841092	A	A	G	G	G	A
6263	rs6284085	2	74904994	G	G	A	A	A	G
6264	rs3719468	2	74983813	A	A	G	A	G	A
6265	mCV25095764	2	76990271	G	A	A	A	A	G
6266	rs3683059	2	75399681	G	G	G	A	A	G
6267	rs4223231	2	75569982	C	C	C	C	C	C
6268	rs4223233	2	75570271	G	G	G	G	G	G
6269	rs3713848	2	75912840	T	T	T	A	T	A
6270	rs3713454	2	76043938	A	A	A	G	A	G
6271	rs3704430	2	76082498	A	A	A	G	A	G
6272	rs3673892	2	76116826	T	T	T	A	T	A
6273	rs6152313	2	76129216	C	C	C	A	C	A
6274	rs3726334	2	76285834	A	A	A	G	A	G
6275	rs3669217	2	76364909	C	C	C	A	C	A
6276	rs6281904	2	76931871	G	A	A	A	A	G
6277	rs3670874	2	77084354	C	A	A	A	A	C
6278	rs3672129	2	77084600	G	A	A	A	A	G
6279	rs6412631	2	77110432	A	T	T	T	T	A
6280	rs3711780	2	77245946	A	T	T	T	T	A
6281	rs6175722	2	78364009	C	C	C	C	C	C
6282	rs3088761	2	78462469	G	G	G	G	G	A
6283	rs3723096	2	78795843	G	A	A	A	A	A
6284	rs6268518	2	78904806	A	G	G	G	G	G
6285	rs3680028	2	79532512	G	G	G	A	G	A
6286	rs3682048	2	79532830	A	A	A	G	A	G
6287	rs3688970	2	79552667	G	G	G	A	G	A

6288	rs3693144	2	79556181	G	G	G	A	G	A
6289	rs4223246	2	79728676	A	A	A	A	A	A
6290	rs6410680	2	80137780	G	A	A	A	A	A
6291	rs3722345	2	80509747	A	G	G	A	G	G
6292	rs4223249	2	80525079	C	G	G	C	G	G
6293	mCV23002990	2	82444161	C	A	A	C	A	C
6294	rs6212296	2	81004132	A	G	G	A	G	A
6295	rs6217256	2	81234680	G	G	G	G	G	G
6296	mCV25110347	2	83337100	A	G	G	A	G	G
6297	rs3716390	2	81662002	G	A	A	G	A	A
6298	rs3667007	2	82000176	G	A	A	G	A	A
6299	rs3715573	2	82038041	C	A	A	C	A	A
6300	rs6362905	2	82464019	A	A	A	A	A	A
6301	mCV25302572	2	85038715	G	A	A	G	A	G
6302	rs6345370	2	83915656	A	G	G	A	G	G
6303	rs4137692	2	84012796	A	C	C	A	C	A
6304	mCV25115393	2	85842145	A	C	C	A	C	A
6305	rs4223251	2	84233339	G	G	G	G	G	G
6306	rs6280546	2	84486384	C	A	A	C	A	A
6307	rs3022888	2	84496199	A	G	G	A	G	G
6308	rs8273634	2	84665852	G	G	G	G	G	G
6309	rs8273639	2	84666393	A	C	C	A	C	C
6310	rs8273626	2	84668232	G	A	A	G	A	A
6311	rs8273627	2	84670068	G	G	G	G	G	G
6312	rs4223257	2	84940791	A	G	G	A	G	G
6313	rs4223258	2	84940830	G	G	G	G	G	G
6314	rs6199827	2	85174680	A	G	G	A	G	G
6315	rs3689658	2	85572783	G	A	A	G	A	G
6316	mCV24333820	2	87504536	G	A	A	G	A	A
6317	rs6413147	2	86686735	T	A	A	T	A	T
6318	rs3698393	2	86697871	G	G	A	G	G	G
6319	rs3675737	2	86941854	A	G	G	A	G	G
6320	rs6303646	2	86942901	A	G	G	A	G	G
6321	rs3723781	2	87728074	C	A	C	C	A	A
6322	rs6314788	2	88240358	T	A	T	T	A	A
6323	rs3686917	2	88478824	C	A	A	C	A	A
6324	rs4138433	2	88805767	A	C	A	A	C	C
6325	rs6404809	2	88899373	G	A	G	G	A	A
6326	rs6389618	2	89196916	C	A	C	C	A	A
6327	rs3719218	2	89303417	G	G	A	G	G	G
6328	rs3715323	2	89474577	A	A	G	A	A	A
6329	rs3670740	2	89479860	A	G	G	A	G	G
6330	mCV25354178	2	91792669	G	A	G	G	G	G
6331	rs3668989	2	87607024	A	A	C	A	A	A
6332	rs3670697	2	87607259	C	C	A	C	C	C
6333	rs6336994	2	91016462	A	G	A	A	G	A
6334	rs6289773	2	91201174	A/G	G	A/G	A	G	A/G
6335	rs3720488	2	91413564	G	A	G	G	A	G
6336	rs3090413	2	91826063	G	G	G	G	G	G
6337	rs6230107	2	91903675	A	A	A	A	G	G
6338	rs4223266	2	92259521	G	G	G	G	G	G
6339	rs4136599	2	92271258	G	G	A	G	G	A
6340	rs6152024	2	92271840	G	G	A	G	G	A
6341	rs6338205	2	92300276	G	C	G	G	G	G
6342	rs3090623	2	92515391	G	G	G	G	G	G
6343	rs3664726	2	92947140	C	C	A	C	C	A
6344	rs3661811	2	93173471	G	A	A	G	G	A
6345	rs4223268	2	93313855	A	A	G	A	G	G
6346	rs6312617	2	93582036	A	G	G	A	G	G
6347	rs6189322	2	93674979	T	T	A	A	A	A
6348	rs6252400	2	94143930	A	A	A	G	G	G
6349	rs6177174	2	94516254	G	G	G	G	G	G
6350	rs6383180	2	94578980	G	G	G	A	G	G
6351	rs3674264	2	94965895	C	C	A	A	C	A
6352	rs3672274	2	95660447	G	G	A	G	G	G
6353	rs3680540	2	95667170	T	A	A	A	A	A
6354	rs6315406	2	95696831	A	G	A	G	G	G
6355	rs3656502	2	96295685	C	C	A	C	C	C
6356	rs3707096	2	96568671	A	A	A	G	A	A
6357	rs3688911	2	96748431	A	A	G	G	G	G
6358	rs6317800	2	96788073	C	C	C	A	A	A
6359	rs3694656	2	97092501	A	G	G	G	A	A
6360	rs3686565	2	97270013	G	A	A	A	G	G
6361	rs6220079	2	97379729	G	A	A	A	G	G

6362	rs6408453	2	98394420	A	A	A	C	A	A
6363	rs3684355	2	98552250	G	G	A	G	A	G
6364	rs3719943	2	99320065	A	A	A	G	A	A
6365	rs3693024	2	99324671	A	A	A	T	A	A
6366	rs6378047	2	99378126	A	A	A	G	A	A
6367	rs3669077	2	99396048	A	A	T	T	T	A
6368	rs3659234	2	99502860	G	G	G	A	G	G
6369	mCV22319073	2	101546860	G	C	G	G	G	G
6370	rs6164718	2	99719299	G	G	G	A	G	G
6371	rs3674361	2	100029114	C	C	C	A	C	C
6372	rs6406705	2	100253963	A	G	A	A	A	A
6373	rs3721532	2	100883302	C	C	C	A	C	C
6374	rs3685454	2	100937630	G	G	A	G	A	G
6375	rs6390756	2	101085510	G	A	G	G	G	G
6376	rs4223283	2	101595109	A	G	A	A	A	A
6377	rs3695682	2	101614432	G	G	A	G	A	G
6378	rs3669332	2	101723131	A	T	A	T	A	T
6379	rs6155648	2	102059025	G	A	G	G	G	G
6380	rs6280107	2	102465019	A	G	A	A	A	A
6381	rs3700286	2	102525512	G	A	G	G	A	G
6382	rs3677262	2	102779585	C	C	C	A	A	C
6383	rs3143809	2	103177268	A	G	A	G	G	G
6384	rs3143810	2	103177373	A	A	A	C	A	C
6385	rs3726330	2	103313028	A	G	A	G	G	G
6386	rs6377411	2	103383653	C	C	C	A	C	A
6387	rs3658729	2	104083849	G	G	G	A	G	G
6388	rs3724460	2	104106537	A	A	A	G	A	G
6389	rs3727022	2	104244689	G	A	G	G	A	G
6390	rs6256701	2	104320120	C	C	C	A	C	C
6391	rs3674721	2	104594142	A	A	G	A	A	G
6392	rs3682153	2	104875790	A	G	G	A	G	G
6393	rs6292555	2	104923509	A	G	G	A	G	A
6394	rs3667167	2	105072934	A	A	A	G	A	G
6395	rs4223360	2	105197556	A	A	A	A	A	A
6396	rs4223363	2	105197826	A	A	A	A	A	A
6397	rs3721336	2	105246522	G	G	A	G	G	G
6398	rs6157070	2	105617504	A	G	G	A	G	A
6399	rs3714835	2	106081426	G	G	A	G	G	G
6400	rs3715478	2	106081523	G	G	G	A	G	G
6401	mCV22364916	2	108453842	C	G	G	C	G	G
6402	rs3090369	2	106384064	A	A	A	A	A	A
6403	rs6323360	2	106490915	A	T	T	A	T	T
6404	rs3673618	2	106656625	G	A	A	G	A	G
6405	rs6242654	2	107132515	A	G	G	A	G	G
6406	rs3710584	2	107136442	A	G	G	A	G	G
6407	rs3684115	2	107180998	A	A	T	A	A	A
6408	rs6350987	2	107641435	A	G	G	A	G	G
6409	rs3717598	2	107769975	G	C	C	G	C	C
6410	rs6253550	2	108577487	A	A	G	A	A	A
6411	rs3661171	2	108749099	G	C	C	G	C	C
6412	rs6208879	2	109674219	A	A	A	G	A	A
6413	rs6318808	2	109881363	G	G	G	A	G	G
6414	rs3022892	2	109891529	G	A	A	G	A	A
6415	rs3722739	2	110252060	A	A	G	G	A	A
6416	rs3710221	2	110280608	G	G	G	A	G	G
6417	rs6192679	2	110409683	A	A	A	G	A	A
6418	rs3658023	2	110705704	A	A	T	T	A	A
6419	rs6233337	2	111027992	T	T	A	T	T	T
6420	rs3714433	2	111122766	A	A	G	A	A	A
6421	rs3681694	2	112175825	G	G	G	A	G	G
6422	rs4223400	2	112363038	A	A	A	A	A	A
6423	rs3693678	2	112367495	A	A	A	G	A	A
6424	rs3709715	2	112417610	G	G	G	A	G	G
6425	rs3090376	2	112522889	G	G	G	G	G	G
6426	rs3022893	2	112522940	G	G	A	G	G	G
6427	rs6156488	2	112847810	T	T	T	T	T	T
6428	rs3663963	2	113012076	A	A	G	A	A	A
6429	rs3666945	2	113012586	A	A	G	A	A	A
6430	rs3698165	2	113054187	G	G	A	G	G	G
6431	rs4223405	2	113718276	A	A	A	A	A	A
6432	rs4223406	2	113718326	A	G	G	A	G	G
6433	rs6378299	2	114073240	A	A	A	A	A	A
6434	mCV22528760	2	116297687	A	G	A	G	G	A
6435	rs4138562	2	114161459	G	G	G	A	G	G

6436	rs3088447	2	114308210	A	A	T	A	A	T
6437	rs3701250	2	114662573	G	G	G	A	G	A
6438	rs6212586	2	114803331	A	G	A	A	G	A
6439	rs6196399	2	114889567	G	G	G	A	G	G
6440	mCV23825533	2	117535468	C	A	C	C	A	C
6441	rs3657162	2	115449990	A	A	A	C	A	C
6442	rs3683827	2	115599080	G	G	A	A	G	G
6443	rs3699172	2	115671423	G	G	A	A	G	G
6444	rs3708146	2	115787955	A	A	A	G	A	A
6445	rs6276129	2	116113235	G	G	A	A	G	A
6446	rs6402721	2	116366906	G	G	G	A	G	G
6447	rs3677413	2	116603199	A	G	A	G	G	G
6448	rs3659529	2	116932359	A	A	G	A	A	A
6449	rs6293684	2	117443289	G	A	A	A	A	A
6450	rs3663941	2	117586672	G	A	A	G	A	G
6451	rs3723406	2	117650393	A	C	C	A	C	A
6452	rs3663325	2	117848215	G	A	A	G	A	A
6453	rs3021887	2	117896802	A	A	A	C	A	A
6454	rs8251603	2	117899091	G	G	G	G	G	G
6455	rs6234992	2	117936685	G	A	A	A	A	A
6456	rs3725853	2	118040554	G	G	G	A	G	G
6457	rs3681225	2	118093938	G	G	G	A	G	G
6458	rs3149842	2	118429359	A	A	A	G	A	A
6459	rs3144393	2	118540348	A	A	A	G	A	A
6460	rs3669388	2	118693853	A	A	A	C	A	A
6461	rs4223410	2	118706142	C	C	C	A	C	C
6462	rs6409590	2	118911998	G	G	G	G	G	G
6463	rs8279354	2	118948317	C	C	C	A	C	C
6464	rs8279382	2	118949228	C	C	C	A	C	C
6465	rs8279381	2	118949380	G	G	G	G	G	G
6466	rs3664735	2	119095870	A	A	A	G	A	A
6467	rs3661254	2	119290749	A/G	G	G	A	A/G	G
6468	rs3654195	2	119323076	A	A	A	G	A	A
6469	mCV24435431	2	121982984	A	A	A	G	A	A
6470	mCV24435456	2	121983518	C	C	C	A	C	C
6471	mCV24435464	2	121984177	NONE	G	G	A	G	G
6472	mCV24435726	2	121986964	A	G	G	G	G	A
6473	mCV24435727	2	121987083	G	G	G	A	G	G
6474	mCV24435745	2	121988534	C	C	C	A	C	C
6475	mCV24435755	2	121988572	G	G	G	A	G	G
6476	mCV24435757	2	121989975	C	A	A	C	A	C
6477	mCV24435768	2	121990163	A	G	G	G	G	A
6478	mCV24435769	2	121990165	G	A	A	G	A	G
6479	mCV24435779	2	121994251	G	G	G	A	G	G
6480	mCV24435780	2	121994419	C	A	A	C	A	C
6481	mCV24435788	2	121994464	G	G	G	A	G	G
6482	mCV24435789	2	121995966	A	G	G	A	G	A
6483	mCV24435790	2	121996176	A	A	A	G	A	A
6484	mCV24435797	2	121996454	G	G	G	A	G	G
6485	mCV24435799	2	121997033	G	A	A	G	A	G
6486	mCV24435806	2	121997034	C	G	G	C	G	C
6487	mCV24435808	2	121997358	G	A	A	A	A	G
6488	mCV24436076	2	121997523	G	G	G	A	G	G
6489	mCV24436077	2	121997749	G	G	G	A	G	G
6490	mCV24436085	2	121998740	G	A	A	G	A	G
6491	mCV24436094	2	122000129	A	G	G	G	G	A
6492	mCV24436095	2	122000246	G	G	G	C	G	G
6493	mCV24436103	2	122001850	G	A	A	G	A	G
6494	mCV24436111	2	122002650	G	G	G	A	G	G
6495	mCV24436112	2	122002689	A	G	G	A	G	A
6496	mCV24436118	2	122004098	A	G	G	G	G	A
6497	mCV24436119	2	122004198	A	A	A	G	A	A
6498	mCV24436127	2	122004487	A	G	G	A	G	A
6499	mCV24436128	2	122006086	A	C	C	C	C	A
6500	mCV24436136	2	122006228	G	G	G	A	G	G
6501	mCV24436137	2	122006264	G	G	G	A	G	G
6502	mCV24436145	2	122006644	G	G	G	A	G	G
6503	mCV24436153	2	122007692	A	G	G	A	G	A
6504	mCV24436430	2	122009602	G	G	G	C	G	G
6505	mCV24436431	2	122009765	G	G	G	A	G	G
6506	mCV24436432	2	122010165	A	A	A	G	A	A
6507	mCV24436439	2	122010624	A	G	G	G	G	A
6508	mCV24436440	2	122010774	A	G	G	A	G	A
6509	mCV24436441	2	122010930	G	G	G	C	G	G

6510	mCV24436451	2	122010971	A	A	A	C	A	A
6511	mCV24436452	2	122011033	G	G	G	A	G	G
6512	mCV24436453	2	122012347	A	A	A	G	A	A
6513	mCV24436464	2	122012578	A	A	A	C	A	A
6514	mCV24436475	2	122012672	A	A	A	G	A	A
6515	mCV24436476	2	122012799	C	C	C	A	C	C
6516	mCV24436487	2	122013481	A	A	A	G	A	A
6517	rs8281185	2	120258386	C	A	C	A	A	C
6518	rs8281186	2	120258424	A	G	G	G	G	A
6519	rs8281377	2	120275930	G	A	G	G	A	G
6520	rs4223427	2	120277878	A	G	A	G	G	A
6521	rs4223428	2	120277888	G	G	G	A	G	G
6522	rs3686905	2	120297295	G	A	G	A	A	G
6523	rs6386814	2	120317874	G	A	G	A	A	G
6524	rs6382013	2	120466676	G	G	G	G	G	G
6525	rs3718386	2	120543620	C	C	C	A	C	C
6526	rs3658927	2	120726441	G	G	G	A	G	G
6527	rs4223432	2	120798314	G	G	G	A	G	G
6528	rs6340352	2	121051368	G	G	G	A	G	G
6529	rs3664905	2	121150448	G	G	G	A	G	G
6530	rs6221401	2	121259260	G	G	G	A	G	G
6531	rs3678620	2	121358519	A	A	A	G	A	A
6532	rs6317255	2	121655084	C	C/G	C	G	C/G	C
6533	rs3720647	2	122035039	A	A	A	G	A	A
6534	rs3723643	2	122067606	A	T	T	A	T	A
6535	rs3686617	2	122307094	A	A	A	G	A	A
6536	rs4223435	2	122343595	C	C	C	C	C	C
6537	rs3695790	2	122373260	A	A	A	G	A	A
6538	rs3696744	2	122953766	G	G	G	A	G	G
6539	rs3665194	2	123005585	G	G	G	C	G	G
6540	rs6228179	2	123216313	T	T	T	A	T	T
6541	rs6358300	2	123402037	T	T	T	A	T	T
6542	rs4223437	2	123542609	A	A	A	A	A	A
6543	rs6367262	2	123590756	G	C	G	C	C	G
6544	rs3660713	2	124401843	C	A	C	A	A	C
6545	rs3700391	2	124410352	G	A	G	A	A	G
6546	rs4223441	2	124414038	G	G	G	G	G	G
6547	rs4223442	2	124414075	A	A	A	A	A	A
6548	rs6401493	2	125046796	G	G	G	A	A	G
6549	rs3090980	2	125050441	A	A	A	A	A	A
6550	rs8251633	2	125050789	G	G	G	G	G	G
6551	rs8251635	2	125050910	G	G	A	A	A	G
6552	rs6374387	2	125228072	G	G	A	G	A	G
6553	rs8254510	2	125256258	A	A	G	A	G	A
6554	rs8254506	2	125256382	A	A	A	A	A	A
6555	rs3697020	2	125287975	A	A	G	G	G	A
6556	rs3725315	2	125487965	C	C	A	C	A	C
6557	rs3726517	2	125488175	G	G	A	G	A	G
6558	rs3708635	2	125627822	A	A	C	A	C	A
6559	rs3689994	2	125643040	C	C	G	C	G	C
6560	rs3667348	2	125790202	G	G	G	A	G	G
6561	rs3704471	2	125928983	G	G	G	A	G	G
6562	rs3720259	2	126394107	C	C	G	C	G	C
6563	rs6383064	2	126422907	A	A	G	A	G	A
6564	rs4223448	2	126852461	G	G	A	G	A	G
6565	rs4223452	2	126852555	C	C	C	C	C	C
6566	rs3022899	2	127157623	G	G	G	A	G	G
6567	rs4223462	2	127205583	G	G	A	G	A	G
6568	rs3677705	2	127392250	A	A	G	A	G	A
6569	rs3680660	2	127445593	G	G	A	G	A	G
6570	rs3702207	2	127475850	G	G	A	G	A	G
6571	rs6280404	2	127764256	G	G	A	G	A	G
6572	rs6411422	2	128122514	G	A	G	A	G	G
6573	rs6325084	2	128307025	G	A	G	A	G	G
6574	rs4223474	2	128359324	C	C	C	C	C	C
6575	rs3671147	2	128801145	G	A	G	A	G	G
6576	rs3022901	2	128838999	G	A	G	A	G	G
6577	rs3662407	2	128972497	G	A	G	A	G	G
6578	rs6318992	2	129013077	G	G	A	G	G	G
6579	rs3718550	2	129024115	A	G	A	G	G	A
6580	rs3022902	2	129048776	A	T	A	T	A	A
6581	mCV25034641	2	131410313	G	G	G	G	A	G
6582	rs6161193	2	129466095	C	A	C	A	A	C
6583	rs3655775	2	129583325	A	A	A	A	A	A

6584	rs4223491	2	129851779	G	G	G	G	G	G
6585	rs3661596	2	129893838	G	A	G	A	A	G
6586	rs6188551	2	129907322	C	A	C	A	A	C
6587	rs6397245	2	130273380	G	A	G	A	A	G
6588	rs4223500	2	130457316	A	A	G	A	A	G
6589	rs3665528	2	130762932	G	G	A	G	G	A
6590	rs8264979	2	130800811	C	C	C	C	C	C
6591	rs8264883	2	130805040	G	G	G	G	G	G
6592	rs8264884	2	130805156	G	G	G	G	A	G
6593	rs8264954	2	130807343	A	A	A	A	G	A
6594	rs8264931	2	130810331	G	G	G	G	A	G
6595	mCV22300686	2	132868150	A	G	A	G	G	G
6596	rs3690254	2	131275700	A	G	G	G	A	G
6597	rs6206689	2	131296031	G	G	G	G	A	G
6598	rs4223510	2	131602703	A	G	A	G	A	G
6599	rs4223511	2	131742558	A	G	A	G	A	G
6600	rs6325260	2	131778700	C	G	C	G	C	G
6601	rs3673192	2	131932284	A	G	G	G	G	G
6602	rs3701463	2	132017400	A	G	A	G	G	G
6603	rs3699051	2	132104826	G	A	G	A	A	A
6604	rs3722210	2	132511317	A	G	G	G	G	G
6605	rs6288723	2	132554606	G	A	G	A	A	A
6606	rs3676388	2	133269555	G	G	A	G	G	G
6607	rs3671635	2	133291360	C	C	A	C	C	C
6608	rs3022934	2	133312250	A	A	A	A	A	A
6609	rs3684247	2	133458369	A	G	A	G	G	G
6610	rs6397401	2	133671460	T	A	A	A	A	A
6611	rs3090353	2	133779335	A	A	A	A	A	A
6612	rs3090709	2	134033983	A	A	A	A	A	A
6613	rs6311917	2	134106307	A	C	C	C	C	A
6614	rs3088678	2	135319740	G	G	G	G	G	G
6615	rs6345820	2	135515842	A	A	A	A	G	A
6616	rs4223539	2	135763696	A	A	A	A	A	A
6617	rs4223537	2	135763908	A	A	A	A	A	A
6618	mCV22322685	2	137943310	A	C	C	C	C	C
6619	rs3682725	2	136072419	A	T	T	A	A	A
6620	rs6234260	2	136147940	T	T	T	T	T	T
6621	rs8246404	2	136627476	A	G	G	G	G	G
6622	rs8246419	2	136629268	G	A	A	A	A	A
6623	rs8237119	2	136629346	G	A	A	A	A	A
6624	rs3710582	2	136636205	G	A	A	A	A	A
6625	rs3710324	2	136973991	A	G	G	G	G	G
6626	rs3686872	2	136996378	A	G	G	G	G	G
6627	rs6329532	2	136997233	A	C	C	C	C	C
6628	rs6249968	2	137541446	G	A	A	A	A	A
6629	rs3673701	2	137550542	C	A	A	A	A	A
6630	rs3726475	2	138320966	T	T	T	A	T	T
6631	rs3090355	2	138449825	G	G	G	G	G	G
6632	rs3653457	2	137893532	C	A	A	A	A	A
6633	rs6282055	2	139481733	A	A	A	A	G	A
6634	rs4223545	2	139507157	A	A	A	A	G	A
6635	rs6251185	2	139669185	G	G	G	G	A	G
6636	rs6187777	2	140873150	A	A	A	A	C	A
6637	rs6397890	2	141701363	A	A	A	A	G	A
6638	rs4223547	2	142368315	A	A	A	A	A	A
6639	rs6331493	2	142453088	G	G	G	G	A	G
6640	rs6195594	2	143346715	A	G	A	A	G	A
6641	rs3022933	2	143565892	A	G	G	G	A	G
6642	mCV22528930	2	143912661	G	A	A	A	G	G
6643	rs3687229	2	143675138	A	G	G	G	A	G
6644	rs3022911	2	143683969	A	C	C	C	A	C
6645	rs3707946	2	143713082	C	A	A	A	C	A
6646	rs3653569	2	143753206	A	T	T	T	A	T
6647	mCV23169908	2	145638579	A	G	G	A	A	G
6648	rs3655895	2	144285357	G	A	A	A	G	G
6649	rs3661718	2	144562809	G	A	A	A	G	G
6650	rs6170159	2	144736816	A	G	G	G	A	G
6651	rs3022909	2	145033627	G	A	A	A	G	A
6652	rs6184615	2	145073372	A	G	G	G	A	G
6653	rs3679762	2	145359834	G	A	A	A	G	A
6654	rs4135727	2	145796023	A	C	C	A	A	C
6655	rs3674082	2	146043765	A	T	T	A	A	T
6656	rs3676491	2	146044193	A	C	C	A	A	C
6657	rs3713952	2	146061726	A	G	G	A	A	G

6658	rs4223554	2	146314337	T	A	A	T	T	A
6659	rs3668423	2	146391662	A	G	G	A	A	G
6660	rs6254019	2	146451300	A	G	G	A	A	G
6661	rs3724835	2	146807658	A	G	G	A	A	G
6662	rs6392985	2	146962181	A	G	G	A	A	G
6663	rs3697284	2	147080130	C	A	A	C	C	A
6664	rs3701696	2	147181290	A	G	G	A	A	G
6665	rs3696870	2	147404525	G	A	A	G	A	G
6666	rs3683143	2	147534193	T	A	A	T	T	A
6667	rs3088585	2	147590650	NONE	A	A	A	A	A
6668	rs3715085	2	147722042	A	G	G	G	A	G
6669	rs6209325	2	147807884	A	G	G	A	G	G
6670	rs4223557	2	148157081	C	C	A	C	C	C
6671	rs3090368	2	148449402	A	A	A	A	A	A
6672	rs6288803	2	148500685	G	C	C	G	C	C
6673	rs8256617	2	148624491	G	A	A	G	A	A
6674	rs8256618	2	148624497	A	A	A	A	A	A
6675	rs8256619	2	148624531	G	G	G	G	G	G
6676	rs3723883	2	148997446	A	G	G	A	G	A
6677	rs3676033	2	149197838	A	T	T	A	T	A
6678	mCV25337624	2	105399199	G	A	G	G	A	G
6679	rs6206791	2	149418436	G	A	A	G	A	A
6680	rs6174924	2	150080631	G	G	G	G	G	G
6681	rs4223560	2	150466827	G	G	G	G	G	G
6682	rs3704224	2	150964799	A	G	G	G	G	G
6683	rs4223561	2	151780235	G	G	G	G	G	G
6684	rs3662117	2	151898592	G	A	A	A	A	A
6685	rs6268584	2	151914328	G	A	A	A	A	A
6686	rs3717705	2	152160056	G	A	A	A	A	A
6687	rs3660554	2	152160378	G	A	A	A	A	A
6688	mCV25388133	18	43912167	A	A	G	G	G	G
6689	rs3690044	2	152642436	C	C	A	C	C	C
6690	rs3675388	2	152733387	G	A	G	A	G	G
6691	rs4223567	2	153072689	G	G	G	G	A	G
6692	rs4223564	2	153072983	G	G	A	G	G	A
6693	rs6401794	2	153499988	C	A	C	C	A	C
6694	rs4223571	2	153533945	A	G	A	A	G	A
6695	rs3670553	2	153825745	A	G	G	G	A	G
6696	rs3675650	2	154007610	A	G	A	G	G	G
6697	rs3683092	2	154011636	G	G	G	A	G	G
6698	rs6209403	2	154103561	G	A	A	G	A	G
6699	rs6376291	2	154204634	G	G	C	C	G	C
6700	mCV22991193	2	152402222	C	A	A	A	A	A
6701	rs6291774	2	154609718	A	G	A	A	G	A
6702	rs3712016	2	154694406	A	G	G	A	G	A
6703	rs3684230	2	154787103	G	G	A	G	G	A
6704	rs6248360	2	155015888	G	G	A	G	G	A
6705	rs6300182	2	155018550	G	A	G	A	A	G
6706	rs3687374	2	155127962	G	A	G	A	A	G
6707	rs6315439	2	155253980	G	C	G	C	C	G
6708	rs4223597	2	155765945	A	A	A	A	A	A
6709	rs3665636	2	155779239	A	G	A	G	G	A
6710	rs3089640	2	155799131	C	C	C	C	C	C
6711	rs3668944	2	156039463	G	A	G	A	A	G
6712	rs6304464	2	156147276	G	A	G	A	A	G
6713	rs3677398	2	156156640	G	A	A	A	A	A
6714	rs3703649	2	156532667	A	NONE	A	C	C	A
6715	rs3682465	2	156555563	NONE	A	C	A	A	C
6716	rs3662063	2	156681178	A	G	G	G	G	G
6717	rs4223600	2	156844496	G	G	G	G	G	G
6718	rs6265656	2	156927243	C	A	A	A	C	A
6719	rs4223605	2	157059147	C	C	C	C	G	C
6720	mCV23875187	2	154897120	A	G	A	G	G	A
6721	rs3662665	2	157205479	G	G	A	A	G	A
6722	rs4223625	2	157389223	A	A	A	A	A	A
6723	rs6193859	2	157693960	C	C	A	C	C	A
6724	rs3673974	2	157732398	A	A	A	T	A	A
6725	rs3691210	2	158058573	G	G	G	A	G	G
6726	rs3695266	2	158068522	G	A	G	A	G	G
6727	rs6325935	2	158128000	C	A	A	A	A	A
6728	rs8273810	2	158180064	A	A	A	A	A	A
6729	rs8273783	2	158189095	G	A	G	A	A	G
6730	rs8273805	2	158190672	A	A	A	A	A	A
6731	rs8273702	2	158195389	G	G	G	A	G	G

6732	rs8273730	2	158197123	A	A	A	A	A	A
6733	rs3679337	2	159136088	G	A	G	A	A	G
6734	rs3662897	2	159161905	A	G	A	G	G	A
6735	rs6389365	2	159606339	A	A	A	A	G	A
6736	rs3690797	2	159644358	A	A	A	G	A	A
6737	rs3690841	2	159644389	A	A	A	C	A	A
6738	rs3708176	2	159678385	A	A	A	G	A	A
6739	rs3022937	2	159778523	G	A	A	G	A	A
6740	rs3694708	2	160071391	G	A	A	G	A	A
6741	rs3088716	2	160275083	C	C	C	C	C	C
6742	rs6152944	2	160367044	A	A	A	G	A	A
6743	rs4223635	2	160826325	G	G	G	G	G	G
6744	rs3692409	2	160874526	G	G	G	A	G	G
6745	rs3693259	2	161030557	A	A	A	C	A	A
6746	rs3664408	2	161443571	C	C	C	A	A	C
6747	rs3726342	2	161516368	A	A	A	G	G	A
6748	rs3667162	2	161516447	A	C	C	A	A	C
6749	rs3089768	2	161542258	A	A	A	A	A	A
6750	rs3693685	2	161567042	A	G	G	G	G	A
6751	rs6204920	2	161803720	C	A	A	C	C	A
6752	rs6311474	2	162109008	A	A	A	G	A	A
6753	rs6184747	2	162247673	G	G	G	G	G	G
6754	rs3692104	2	162274799	G	G	G	A	G	G
6755	rs3705202	2	162396274	G	A	A	A	G	G
6756	rs3685730	2	162428763	G	A	A	G	G	G
6757	mCV22653812	2	160483078	G	C	C	C	C	C
6758	rs3673613	2	162915015	G	G	G	A	A	A
6759	rs4223639	2	163000078	A	A	A	A	A	A
6760	rs4223640	2	163000228	C	C	C	C	C	C
6761	rs6233748	2	163039795	C	A	A	A	A	A
6762	rs3671849	2	163215888	G	C	C	G	C	G
6763	rs3089167	2	163417076	G	G	G	G	G	G
6764	rs8260591	2	163798651	G	A	A	A	A	A
6765	rs8260429	2	163815109	A	T	T	A	T	A
6766	rs6345991	2	163870101	G	G	G	A	G	G
6767	rs3697980	2	164075841	A	A	A	G	G	G
6768	rs3696904	2	164212355	G	A	A	A	A	A
6769	rs3696248	2	164421153	A	A	G	A	G	G
6770	rs8275857	2	164437309	C	C	C	G	C	C
6771	rs8275858	2	164437725	C	G	C	C	C	C
6772	rs3675564	2	164453511	A	A	A	G	A	A
6773	rs3695555	2	164525260	A	G	A	A	A	A
6774	rs8269673	2	164895700	G	G	G	G	G	G
6775	rs4223658	2	165011710	G	G	G	G	G	G
6776	rs6185704	2	165016638	G	A	A	G	A	A
6777	rs3089347	2	165275447	A	A	A	A	A	A
6778	rs6177049	2	165845842	G	G	G	A	G	G
6779	rs6338914	2	165855046	A	G	G	A	G	G
6780	rs6218427	2	165965084	G	G	G	A	G	G
6781	rs3712766	2	166073326	G	G	G	A	G	G
6782	rs3653795	2	166693500	C	G	G	G	C	G
6783	rs6311427	2	166736001	A	G	G	G	A	G
6784	mCV24935609	2	164523944	G	G	G	A	G	G
6785	mCV24935595	2	164525262	A	G	A	A	A	A
6786	mCV24935594	2	164525874	C	A	C	A	C	C
6787	mCV24935590	2	164526607	G	A	G	G	G	G
6788	mCV24935589	2	164527691	C	C	C	C	C	C
6789	mCV24935588	2	164528155	A	C	A	A	A	A
6790	mCV24935319	2	164528642	T	A	T	A	T	T
6791	mCV24935318	2	164528750	A	G	A	G	A	A
6792	mCV24935310	2	164528773	G	G	G	C	G	G
6793	rs4223755	2	167559729	G	G	G	G	G	G
6794	rs3702038	2	167039415	A	G	G	G	G	G
6795	rs6219107	2	167539095	A	A	A	A	G	G
6796	rs4223671	2	168599446	G	G	G	G	G	G
6797	rs4223675	2	168811879	A	A	A	A	A	A
6798	rs4223674	2	168812009	C	C	C	C	C	C
6799	rs3708726	2	168991552	G	G	G	A	G	G
6800	rs6160839	2	169029804	G	A	G	A	G	A
6801	rs3714936	2	169150057	G	A	G	A	G	G
6802	rs3656991	2	169266292	A	G	A	G	A	A
6803	rs3689258	2	169551018	A	G	A	G	A	A
6804	rs6306203	2	169569355	A	G	A	G	A	A
6805	rs3663663	2	169707976	C	C	C	A	C	C

6806	rs3726974	2	169905098	A	A	A	C	A	A
6807	rs3143886	2	170023409	A	A	A	G	A	A
6808	rs3142729	2	170176233	A	A	A	C	A	A
6809	rs6175692	2	171375396	G	G	G	G	G	G
6810	rs3703438	2	171460563	G	G	G	A	G	G
6811	rs3724618	2	171587659	G	G	G	A	G	G
6812	rs3677660	2	171734162	T	T	T	A	T	T
6813	rs3665916	2	171800911	A	G	A	G	A	G
6814	rs3681347	2	171809050	G	A	G	A	G	A
6815	rs3672061	2	172285289	A	A	A	G	A	G
6816	rs4223683	2	172419557	A	A	A	A	A	A
6817	rs4223681	2	172419739	G	G	G	G	G	G
6818	rs3668691	2	172801637	G	G	G	C	G	G
6819	rs3681553	2	172847586	A	A	A	G	A	A
6820	rs4223689	2	173003083	G	G	G	G	G	G
6821	rs3673248	2	173087906	G	G	G	A	G	G
6822	rs3673347	2	173087963	A	A	A	G	A	A
6823	rs6384888	2	173614022	A	A	C	A	C	C
6824	mCV24846159	2	171087115	A	A	A	C	A	C
6825	rs3664044	2	173671254	G	G	G	A	G	G
6826	rs3668345	2	173714429	A	A	G	G	G	G
6827	rs4223695	2	173846338	A	A	A	A	A	A
6828	rs8238755	2	174485975	G	G	A	A	A	A
6829	mCV23614425	2	174313677	G	G	A	A	A	A
6830	rs6265399	2	177931595	A	A	A	A	A	A
6831	rs3674193	2	178405527	C	C	C	A	C	C
6832	rs6193482	2	178499082	A	A	G	G	G	G
6833	rs6335805	2	178967205	A	A	A	G	A	G
6834	rs4139310	2	178967735	G	G	G	A	G	A
6835	rs6187766	2	179210967	G	G	G	A	G	A
6836	rs3703298	2	179649104	C	C	C	G	C	C
6837	rs3719336	2	179669991	A	A	G	G	G	A
6838	rs3673139	2	179722432	NONE	A	G	G	G	A
6839	rs4223698	2	179787855	G	G	G	G	G	G
6840	rs3679483	2	179814463	G	G	G	A	G	G
6841	rs6310525	2	180550141	A	G	G	A	G	G
6842	rs3699297	2	180685302	A	C	C	C	C	C
6843	rs6305540	2	180779087	A	G	G	A	G	A
6844	rs4223701	2	181204511	G	C	C	G	C	G
6845	rs3655467	2	181209934	T	A	A	T	A	T
6846	rs8237215	2	181407046	G	G	G	G	G	G
6847	rs8237216	2	181407118	A	A	A	A	A	A
6848	rs8237217	2	181407250	G	G	G	G	G	G
6849	rs8238464	2	181408057	G	A	A	G	A	G
6850	rs8238465	2	181408146	G	A	A	G	A	G
6851	rs3022946	2	181408390	A	G	G	A	G	A
6852	rs6402916	2	181495642	G	G	G	A	G	A
6853	rs3708442	1	192239167	G	G	G	A	G	G
6854	rs3708508	1	192239190	A	A	A	G	A	A
6855	rs6171250	3	3022116	A	A	A	A	A	A
6856	mCV23626472	3	6472940	A	C	C	A	C	C
6857	rs6266594	3	3861987	A	A	A	A	A	A
6858	mCV25454657	3	7102640	G	G	G	G	A	A
6859	rs3694661	3	4302966	C	C	C	C	C	C
6860	rs6175498	3	4586214	C	C	C	C	C	C
6861	rs6191235	3	5002439	A	A	A	A	A	A
6862	rs3675711	3	5621317	A	G	G	A	A	A
6863	rs6316429	3	5716359	A	T	T	A	A	A
6864	mCV23610475	3	9025918	G	A	A	G	G	G
6865	rs6247681	3	5934431	A	G	G	A	A	A
6866	rs6219934	3	7067099	G	G	G	G	G	G
6867	rs6343366	3	7320522	A	A	A	A	A	A
6868	rs3689073	3	7408795	A	C	C	A	C	C
6869	rs3689138	3	7408829	G	A	A	G	A	A
6870	rs4223705	3	7575117	T	T	T	T	T	T
6871	rs4223706	3	7575156	A	G	G	A	G	G
6872	rs8246989	3	7638183	A	G	G	A	G	G
6873	rs8246984	3	7638311	C	C	C	C	A	A
6874	rs6403649	3	8016537	A	A	A	A	A	A
6875	rs3089579	3	8348971	A	A	A	A	A	A
6876	rs4223709	3	8596863	A	G	G	A	A	A
6877	rs8246926	3	8703710	A	A	A	A	A	A
6878	rs4223711	3	8840343	A	A	A	A	A	A
6879	rs6397899	3	9044696	A	A	A	A	A	A

6880	rs3701904	3	9535129	G	A	A	G	A	G
6881	rs6270460	3	9816746	A	A	A	A	A	A
6882	rs3719352	3	10752146	A	A	A	G	A	A
6883	rs6368632	3	10819828	G	G	G	A	G	G
6884	rs6322092	3	10973122	G	G	G	G	G	G
6885	rs6230946	3	11159401	A	A	A	A	A	A
6886	rs3703706	3	11360137	A	A	A	G	A	A
6887	mCV25445157	3	15128651	C	C	C	C	A	C
6888	rs3713697	3	11888990	A	A	A	G	G	A
6889	rs6408490	3	12212983	A	A/G	A	A	A/G	A/G
6890	rs3694133	3	12562659	T	T	T	A	A	A
6891	rs3716641	3	12564058	A	A	A	G	G	G
6892	rs6351478	3	12626499	C	G	G	G	C	G
6893	rs6193496	3	13096281	A	A	A	A	A	A
6894	rs3657571	3	13192503	T	A	A	A	T	A
6895	rs3674446	3	13818754	A	G	G	G	A	G
6896	rs4223739	3	13907061	C	C	C	C	C	C
6897	rs3721276	3	14434508	G	A	A	A	A	A
6898	mCV22614242	3	17286400	G	G	G	A	G	G
6899	rs3022949	3	14707885	G	G	G	G	A	G
6900	rs4223742	3	14956131	C	C	C	C	C	C
6901	rs6191305	3	15185752	A	A	A	A	A	A
6902	rs6354267	3	15298021	A	A	A	A	A	A
6903	mCV24712981	3	18230578	A	G	G	A	G	A
6904	rs3688496	3	15375240	G	A	A	G	A	A
6905	rs6235756	3	15676691	A	G	G	A	G	G
6906	mCV24211562	3	18932166	A	A	A	A	G	A
6907	rs3659988	3	16122429	A	G	G	A	G	G
6908	rs4138128	3	16536069	A	G	G	G	G	G
6909	rs6379358	3	16573559	G	A	A	A	A	A
6910	rs6345660	3	16834103	A	A	A	C	A	A
6911	rs3666486	3	16836912	G	G	G	A	G	G
6912	rs6195996	3	17430540	T	A	A	A	A	T
6913	rs4223753	3	17447037	T	T	T	T	T	T
6914	rs4223752	3	17447048	G	A	A	A	A	G
6915	rs3664315	3	17466555	G	A	A	A	A	G
6916	rs3696263	3	17524518	A	T	T	A	T	A
6917	rs3689046	3	17762562	G	G	G	C	G	G
6918	rs3669876	3	18932029	A	G	G	A	G	A
6919	mCV24730919	3	19343842	A	A	A	A	T	T
6920	rs3674030	3	19358916	G	C	C	C	G	C
6921	rs6410894	3	19505679	G	G	G	A	A	A
6922	rs6355775	3	19809047	C	C	C	C	C	C
6923	rs3090498	3	19889829	C	C	C	C	C	C
6924	rs3698422	3	20496779	A	A	A	C	A	A
6925	rs6321395	3	20543384	G	G	G	A	G	G
6926	rs3677150	3	20698890	A	A	A	G	G	G
6927	rs6235984	3	21215374	A	A	A	A	T	T
6928	rs6204524	3	21900163	G	G	G	G	G	G
6929	rs6289789	3	23207671	G	G	G	G	G	G
6930	rs6356704	3	23217363	G	G	G	G	G	G
6931	rs6167490	3	24525160	G	G	G	G	G	G
6932	rs6197072	3	24756319	G	G	G	G	G	G
6933	rs3710311	3	25335993	A	A	A	A	A	A
6934	rs6373308	3	25959361	A	A	A	G	A	A
6935	rs3698991	3	26097422	G	G	G	A	G	G
6936	rs3724098	3	26147418	A	G	G	A	G	G
6937	rs3715834	3	26405124	G	G	G	A	A	A
6938	rs3670738	3	27090632	G	A	A	G	G	G
6939	rs6289732	3	27135045	A	C	C	A	A	A
6940	rs4223769	3	27181895	A	G	G	A	A	A
6941	rs3661152	3	27508283	A	C	C	A	A	A
6942	rs3677132	3	27684663	G	G	G	A	A	A
6943	rs3700087	3	27740855	G	G	G	A	G	G
6944	rs8247032	3	27896885	G	G	G	G	G	G
6945	rs4223791	3	27898429	G	G	G	G	G	G
6946	rs4223797	3	28435640	A	A	A	A	A	A
6947	rs6260196	3	28495137	A	T	T	A	T	A
6948	rs6258396	3	28833451	A	G	G	A	G	A
6949	rs2020463	3	28883323	A	G	G	A	G	G
6950	rs4223804	3	29022447	A	A	A	A	A	A
6951	rs3653514	3	29295055	C	G	G	C	C	C
6952	rs6400008	3	29581930	G	A	A	G	G	A
6953	rs3660588	3	30094761	A	G	G	A	A	G

6954	rs6228571	3	30095717	C	A	A	C	C	A
6955	rs3686996	3	30149129	A	A	A	A	A	G
6956	rs4223832	3	30586739	A	A	A	A	A	A
6957	rs3023779	3	30888857	A	A	A	A	A	A
6958	rs6303076	3	31596188	G	G	G	G	G	G
6959	rs3661905	3	31634928	A	A	A	T	A	A
6960	rs6218317	3	31657743	A	A	A	G	A	A
6961	rs3702402	3	31969604	C	A	A	C	C	A
6962	rs6351323	3	32246505	T	A	A	T	T	T
6963	rs3663409	3	32363960	G	G	G	A	G	G
6964	rs4223865	3	32726880	A	A	A	A	A	A
6965	rs4223864	3	32726887	G	G	G	A	A	G
6966	rs3711011	3	32731896	G	A	A	G	G	G
6967	rs6332819	3	32774568	G	A	A	G	G	G
6968	rs3687193	3	32806508	A	G	G	A	A	A
6969	rs3695308	3	33494366	G	G	G	A	G	G
6970	rs4223871	3	33604793	T	T	T	T	T	T
6971	rs3664070	3	33708015	C	C	C	A	C	C
6972	rs4223883	3	33860283	G	A	A	A	G	G
6973	rs6371982	3	33929993	C	C	C	A	A	A
6974	rs6219246	3	34705660	A	G	G	A	A	G
6975	rs6305300	3	34854172	C	A	A	C	C	A
6976	rs3701286	3	35038794	C	A	A	C	C	A
6977	rs3660816	3	35371021	C	G	G	G	C	NONE
6978	rs3702496	3	35470885	C	C	C	A	NONE	C
6979	rs3720738	3	35779420	A	G	G	A	A	G
6980	rs4223924	3	36010945	A	A	A	A	A	A
6981	rs6246699	3	36110851	G	G	G	A	A	G
6982	rs3151902	3	36405733	A	A	A	G	A	G
6983	rs3146358	3	36664127	G	G	G	A	G	A
6984	rs3146315	3	36682882	G	G	G	A	G	A
6985	rs3151196	3	36739421	A	A	A	G	A	G
6986	rs3162526	3	37242071	G	G	G	A	G	A
6987	rs3721455	3	37398597	G	A	A	G	G	G
6988	rs6260262	3	37707494	G	G	G	G	G	G
6989	rs3705939	3	37949126	A	A	A	G	A	G
6990	rs3670471	3	38477365	A	A	A	C	A	C
6991	rs6274061	3	38689460	A	A	A	G	A	G
6992	mCV24811501	3	43098164	A	C	C	A	A	A
6993	rs6257041	3	38888412	A	A	A	C	C	A
6994	rs3665109	3	39623689	G	A	A	A	G	A
6995	rs3714497	3	39696714	G	G	G	A	G	G
6996	rs6324747	3	39835273	G	A	G	A	G	G
6997	rs3701719	3	41684309	G	A	A	G	G	G
6998	rs4223937	3	41769353	G	G	G	G	G	G
6999	rs6318748	3	41801897	G	A	A	G	G	A
7000	rs4223942	3	40570547	C	C	C	C	C	C
7001	rs3712832	3	40848437	G	G	G	A	G	G
7002	mCV24401139	3	46173120	A	G	A	A	G	G
7003	rs3725706	3	41575201	A	A	A	G	A	A
7004	rs3723018	3	41705800	A	G	G	G	A	G
7005	rs3661720	3	41838640	G	A	A	A	G	A
7006	rs6374301	3	41937597	A	T	T	T	A	T
7007	rs6235368	3	42029401	A	A	A	A	A	A
7008	rs3699423	3	42115367	G	C	C	G	G	C
7009	rs3670626	3	42241608	T	A	A	A	T	A
7010	rs3711327	3	42365259	A	G	G	G	A	G
7011	rs6316149	3	42484676	T	T	T	T	T	T
7012	rs3725189	3	42860293	G	A	A	G	G	G
7013	rs6188525	3	42993741	A	G	G	A	A	A
7014	rs6371266	3	43428882	T	T	T	T	T	T
7015	rs6346011	3	43511801	A	G	G	A	A	A
7016	rs4223947	3	43704216	C	A	A	C	C	C
7017	rs4223948	3	43704239	T	A	A	T	T	T
7018	rs3722463	3	44019288	A	G	G	A	A	A
7019	rs3667394	3	44381554	A	G	G	A	A	G
7020	rs6248252	3	44579002	A	T	T	A	A	T
7021	mCV24847225	3	49440897	G	A	A	G	A	A
7022	rs6327630	3	45054035	A	G	G	A	A	A
7023	rs3725268	3	45055169	T	A	A	T	T	T
7024	rs3707139	3	45848324	G	A	A	G	G	G
7025	rs6191141	3	45861226	A	G	G	A	A	A
7026	rs3684206	3	45885563	A	G	G	A	A	A
7027	rs6189053	3	46943543	A	A	A	A	A	A

7028	mCV24650374	3	48720060	T	A	A	A	A	T
7029	rs6205286	3	47789143	G	C	C	G	C	C
7030	rs3667675	3	47853066	C	A	A	C	A	A
7031	rs3687414	3	47853961	C	G	G	C	G	G
7032	mCV23376784	3	52590325	G	G	A	A	A	G
7033	rs3691363	3	48065869	C	A	A	C	A	A
7034	rs3678630	3	48189374	A	G	G	A	G	G
7035	rs3681246	3	48189759	A	C	C	A	C	C
7036	rs3670072	3	48641016	A	G	G	A	G	G
7037	rs6300615	3	48932481	A	C	C	A	C	C
7038	mCV22818381	3	53753953	G	G	G	G	A	A
7039	rs6381292	3	49706571	G	G	G	G	G	G
7040	rs3714309	3	49710839	G	A	A	G	A	A
7041	rs3707464	3	49772353	A	G	G	A	G	G
7042	rs3671459	3	50142982	G	A	G	G	A	A
7043	rs4223949	3	50384342	A	A	A	A	A	A
7044	rs3022958	3	50421328	G	A	G	G	G	A
7045	rs3022956	3	50450447	A	A	A	A	C	A
7046	rs3089298	3	50450529	G	A	G	G	A	A
7047	rs6367189	3	50453893	A	T	A	A	T	T
7048	rs4139913	3	50623988	G	A	G	G	A	A
7049	rs3724545	3	50776152	G	A	A	G	A	A
7050	rs3657939	3	50781075	A	G	G	A	G	G
7051	rs6327143	3	50872034	A	G	G	A	G	G
7052	rs3689832	3	51352183	G	A	G	A	A	G
7053	rs3719360	3	51328183	G	A	G	A	A	G
7054	rs6222857	3	51422976	G	C	G	C	C	G
7055	rs3694611	3	51636182	A	C	A	C	C	A
7056	rs8257976	3	52430836	A	A	A	A	A	A
7057	rs8257977	3	52431400	G	G	G	G	G	G
7058	rs6241331	3	52675823	G	G	A	A	A	G
7059	mCV22953681	3	57445834	T	A	T	T	A	A
7060	rs3685081	3	52790137	G	G	G	A	A	A
7061	rs3718812	3	52887282	A	A	A	G	G	G
7062	rs3674296	3	53019876	G	G	G	A	A	A
7063	rs6335414	3	53103565	G	A	G	A	A	A
7064	rs3665395	3	53339892	A	A	G	A	A	A
7065	rs3714799	3	53346250	C	C	A	C	C	C
7066	rs6272868	3	53597983	A	A	A	A	A	A
7067	rs6324464	3	54392652	T	T	T	T	T	T
7068	rs4223969	3	55143939	A	A	C	A	C	C
7069	rs4223971	3	55144141	A	A	C	A	C	C
7070	rs4223974	3	55144223	A	A	A	A	A	A
7071	rs6235901	3	55254969	T	T	T	T	T	T
7072	rs6337816	3	55273163	G	G	G	G	G	G
7073	rs6319642	3	56600936	G	G	G	G	A	A
7074	rs4223977	3	57569127	A	G	A	A	G	G
7075	rs6363066	3	57943185	G	A	G	G	A	A
7076	rs6301139	3	58612475	A	T	T	A	T	A
7077	rs6342158	3	59334595	A	G	A	A	A	G
7078	rs3680871	3	59376091	G	A	G	A	A	G
7079	mCV24528954	3	64169802	G	A	G	G	A	A
7080	rs3723181	3	59440244	A	C	A	C	C	A
7081	rs3691114	3	59986297	G	A	G	A	A	G
7082	rs3676098	3	60164656	A	A	A	G	G	A
7083	rs3667772	3	60329358	A	A	A	G	G	A
7084	rs6234223	3	60330036	A	A	A	G	G	A
7085	rs3719696	3	60434800	G	G	G	A	A	G
7086	rs3715395	3	60447224	T	T	T	A	A	T
7087	rs6239288	3	60673679	C	A	C	A	A	C
7088	rs4223979	3	60713909	G	A	G	A	A	G
7089	rs3706317	3	61254092	C	C	A	C	C	C
7090	rs6326241	3	61758413	A	A	A	A	C	A
7091	rs3656374	3	61779434	C	G	C	G	G	G
7092	rs3672242	3	61890751	A	G	A	G	G	A
7093	rs3662143	3	61942195	G	A	G	A	A	G
7094	rs3672601	3	61972258	A	G	A	G	G	A
7095	rs3700657	3	62104317	G	A	A	A	A	G
7096	rs6176848	3	62234752	A	C	C	A	C	A
7097	rs3672384	3	63027357	C	A	A	A	A	A
7098	rs3696955	3	63295533	G	A	A	G	G	G
7099	rs3710506	3	63415447	G	A	A	A	G	G
7100	rs3722255	3	63484408	A	G	A	G	A	A
7101	rs6326655	3	63500902	A	C	C	C	A	A

7102	rs3715851	3	63549909	C	C	A	C	C	C
7103	mCV24530618	3	64630281	G	G	G	G	A	G
7104	rs6212539	3	65222079	A	C	C	C	A	A
7105	rs3671859	3	65449616	G	A	A	A	A	A
7106	rs3091173	3	65615087	A	A	A	A	A	A
7107	rs3674751	3	65909192	A	G	A	A	G	G
7108	rs3718995	3	66265244	A	A	A	A	A	G
7109	rs6224355	3	66327900	G	G	G	G	G	G
7110	mCV25201949	3	70971737	C	C	C	G	C	C
7111	rs3707121	3	66710305	G	G	G	G	G	C
7112	rs6320689	3	67249780	A	A	A	A	A	A
7113	rs6226544	3	67856811	A	A	A	A	A	G
7114	rs6342742	3	68204212	G	G	G	G	G	G
7115	rs3089698	3	68260396	G	G	G	G	G	G
7116	rs6350199	3	68393024	G	G	G	G	G	G
7117	rs3674810	3	68455356	A	A	A	G	A	A
7118	rs3678419	3	68520434	G	G	G	A	G	G
7119	rs8259022	3	69032650	A	A	A	A	A	A
7120	rs8259028	3	69032970	A	A	A	A	A	A
7121	rs8259038	3	69033146	G	G	G	G	G	G
7122	rs4223988	3	69374806	G	G	G	G	G	G
7123	rs3715204	3	69444709	G	A	A	G	A	G
7124	rs6243018	3	69843132	G	G	G	G	G	G
7125	rs3654911	3	69882294	G	G	G	A	G	G
7126	rs6198234	3	70361430	G	A	G	A	A	G
7127	rs3714575	3	70391230	T	A	T	A	A	T
7128	rs6386080	3	70778538	G	G	G	G	G	G
7129	rs3659459	3	70978751	T	T	T	A	T	T
7130	rs3659688	3	71191410	G	G	G	C	G	G
7131	rs3698109	3	71209119	A	A	G	A	A	A
7132	rs3717453	3	71468066	G	G	A	G	A	G
7133	rs3680433	3	71525511	A	A	C	A	C	A
7134	rs6251545	3	71660011	G	G	G	G	G	G
7135	rs3090390	3	71706209	G	A	A	G	A	G
7136	rs3022961	3	71813330	A	G	A	A	A	A
7137	rs6264454	3	71858927	A	A	G	G	G	A
7138	rs3699288	3	72179568	A	G	G	G	G	A
7139	rs3693395	3	72445252	A	C	C	C	C	A
7140	rs3715136	3	72730362	A	A	A	C	A	A
7141	rs6210155	3	72881766	A	A	A	A	A	A
7142	rs2020552	3	73134921	A	A	A	A	A	A
7143	rs3671511	3	73232814	G	G	G	A	G	G
7144	rs6228802	3	73255926	A	A	A	A	A	A
7145	rs4137345	3	73568123	C	C	A	A	A	C
7146	rs6189620	3	74159485	G	G	A	A	A	G
7147	rs6401341	3	74657515	C	C	C	A	A	C
7148	rs6384991	3	75371911	A	A	A	A	A	A
7149	rs6368888	3	75377598	G	A	A	A	G	G
7150	rs3695004	3	75740748	A	G	G	G	G	G
7151	rs8273642	3	75957143	A	A	A	A	A	A
7152	rs6338429	3	76013182	A	A	A	A	A	A
7153	rs8273648	3	76016414	G	G	G	G	A	G
7154	rs8273664	3	76019138	G	G	G	G	G	G
7155	rs3718054	3	76447907	C	C	A	A	A	C
7156	rs6322772	3	77064260	A	A	A	A	A	A
7157	rs3653769	3	77244331	G	G	G	G	A	A
7158	rs3088562	3	77475627	A	A	A	A	A	A
7159	rs6224757	3	77507569	C	C	C	C	C	C
7160	rs3726567	3	77849745	A	C	C	C	C	A
7161	rs6385363	3	78223823	A	A	A	A	A	A
7162	rs6317775	3	78240792	A	G	G	G	A	G
7163	rs3672300	3	78305116	C	A	A	A	C	A
7164	rs3715352	3	78618892	G	G	G	A	A	G
7165	rs3702037	3	79254391	T	T	T	A	A	T
7166	rs6193292	3	79287399	G	G	G	G	G	G
7167	rs3720421	3	79288431	G	A	A	G	A	A
7168	rs3677276	3	79444441	G	G	G	A	G	G
7169	rs3683507	3	79585796	A	A	A	C	A	A
7170	rs4224010	3	79651222	A	A	A	A	A	A
7171	rs6310547	3	79677013	A	A	A	A	A	A
7172	rs3686804	3	79722098	G	G	G	A	G	G
7173	rs6395358	3	80649862	A	A	A	A	A	A
7174	rs3723964	3	80760887	A	G	G	G	G	G
7175	rs6157283	3	80891061	A	A	A	G	G	G

7176	rs3659866	3	81285486	A	A	A	G	A	G
7177	rs3689917	3	81520907	C	C	C	G	C	C
7178	rs6354512	3	81523646	A	A	A	A	A	A
7179	rs4224017	3	81610159	G	G	G	G	G	G
7180	rs6224368	3	81807591	A	A	A	A	A	A
7181	rs3685286	3	81893232	A	A	A	G	A	A
7182	rs3706413	3	82586808	A	G	G	G	G	G
7183	rs6315046	3	82771431	G	G	G	G	G	G
7184	rs3699123	3	82791773	A	G	G	A	G	A
7185	rs6227500	3	83408035	G	G	G	G	G	G
7186	rs3699532	3	83634097	C	A	A	C	C	C
7187	rs4221946	3	84169357	G	G	G	G	G	G
7188	rs6329605	3	84225548	G	G	G	G	G	G
7189	rs3708227	3	84422484	A	G	G	A	A	A
7190	mCV24886073	3	89418525	G	A	A	G	A	A
7191	rs3726270	3	84520730	G	G	G	A	G	A
7192	rs3665763	3	84897505	A	A	A	C	A	A
7193	rs4136366	3	85309719	G	A	A	A	G	A
7194	rs3667053	3	85577332	C	C	C	A	A	A
7195	rs6243021	3	85606676	A	A	A	G	A	G
7196	rs3696976	3	85620198	NONE	A	A	C	A	C
7197	mCV24882303	3	90520092	A	A	A	A	C	A
7198	rs3705396	3	85883601	A	G	G	G	G	G
7199	mCV24984125	3	91088111	G	A	A	A	G	A
7200	rs6340574	3	86385693	A	G	G	A	A	A
7201	rs3698196	3	86596670	G	G	G	A	G	A
7202	rs6327196	3	86767512	C	C	C	C	C	C
7203	rs3688780	3	86858364	G	G	G	C	G	C
7204	rs6364303	3	87272454	A	A	A	A	A	A
7205	mCV24245305	3	88894429	G	G	G	G	G	G
7206	rs3670634	3	87680439	A	A	A	T	A	A
7207	rs4224041	3	87858096	A	A	A	C	A	A
7208	rs3665990	3	87879075	A	A	A	G	A	A
7209	rs4138858	3	88133448	C	C	C	A	C	C
7210	rs3726528	3	88140043	A	A	A	G	A	A
7211	rs4224044	3	88443152	A	C	C	A	A	C
7212	rs4224047	3	88443277	A	A	A	A	A	A
7213	rs6368299	3	88599395	G	G	G	G	G	G
7214	rs6403572	3	89027785	A	A	A	A	A	A
7215	rs8259159	3	89656428	G	G	G	G	G	G
7216	rs4224072	3	89768648	A	A	A	A	A	A
7217	rs8260435	3	89772279	G	G	G	G	G	G
7218	rs6271093	3	89794721	G	G	G	G	G	G
7219	rs6277809	3	89934734	G	G	G	G	G	G
7220	rs6332465	3	90217503	A	A	A	A	A	A
7221	rs4224075	3	90315386	A	A	A	A	A	A
7222	mCV24550998	3	94106299	A	A	A	A	A	A
7223	rs6211610	3	90650953	G	G	G	G	A	G
7224	rs6224522	3	91037299	A	A	A	A	C	A
7225	rs6325730	3	91477570	C	C	C	C	C	C
7226	rs6317479	3	92218995	G	G	G	G	G	G
7227	rs3697710	3	93210369	C	C	C	A	A	A
7228	rs3708085	3	93247014	A	A	A	G	G	G
7229	rs3708699	3	93247088	G	G	G	A	A	A
7230	rs3661505	3	93251869	G	G	G	A	A	A
7231	rs4135965	3	93252158	G	G	G	A	A	A
7232	rs6227030	3	93316355	G	G	G	A	A	A
7233	rs6256990	3	93357257	A	G	G	A	A	A
7234	rs3696371	3	93462597	A/G	A	A/G	G	G	G
7235	rs6323442	3	93472568	A	G	G	A	A	A
7236	rs4224087	3	93508892	A	A	A	A	A	A
7237	rs4224086	3	93508938	A	A	A	A	A	A
7238	rs4224101	3	94250845	A	A	A	A	A	A
7239	rs3674983	3	94421659	G	G	G	C	G	G
7240	rs3654088	3	94467844	T	A	A	T	T	T
7241	rs6315947	3	94984041	A	A	A	A	A	A
7242	rs4224105	3	95213999	G	G	G	G	G	G
7243	rs8251931	3	95463301	G	G	G	G	G	G
7244	rs3717575	3	95484469	G	A	A	G	G	G
7245	rs8252196	3	95488608	G	G	G	G	A	G
7246	rs8252013	3	95490318	T	T	T	T	T	T
7247	rs8252014	3	95490372	A	A	A	A	A	A
7248	rs3720007	3	95517021	G	A	A	A	A	A
7249	rs4140010	3	95574347	A	G	G	G	G	G

7250	rs6217010	3	96026491	A	A	A	G	G	A
7251	mCV23386977	3	99501834	G	A	A	G	G	G
7252	rs4224122	3	96193283	A	A	A	G	G	A
7253	rs4224125	3	96193407	G	G	G	A	A	G
7254	rs3716753	3	96401770	A	A	A	G	G	A
7255	rs6405447	3	96740405	G	G	G	G	G	G
7256	rs3687989	3	96956421	G	G	G	A	A	G
7257	rs3680173	3	97007924	G	C	C	C	C	C
7258	rs3022964	3	97282711	G	A	A	A	A	G
7259	rs6230337	3	97338812	G	G	G	G	G	G
7260	mCV25367221	3	101099345	G	A	A	G	A	G
7261	rs6391963	3	98231198	C	A	A	A	A	C
7262	rs3675845	3	98325723	G	G	G	G	A	G
7263	rs3713442	3	98390016	G	A	A	A	A	G
7264	rs4224132	3	98430448	C	C	C	C	C	C
7265	mCV25105052	3	102194331	A	A	A	A	A	A
7266	rs8245788	3	98731105	A	A	A	A	A	A
7267	rs6222376	3	98777175	A	G	G	G	A	A
7268	rs3686473	3	98864236	A	G	G	G	A	A
7269	rs3708129	3	98882760	A	A	A	G	G	A
7270	rs3726226	3	98954988	G	G	G	A	A	G
7271	rs6279860	3	99683366	G	G	G	G	G	G
7272	rs3694780	3	99836337	G	G	G	A	A	A
7273	rs3667147	3	99891590	G	A	A	G	G	G
7274	mCV25363602	3	103929427	G	A	A	A	A	G
7275	rs6341239	3	101261614	A	A	A	A	A	A
7276	rs3669106	3	101263112	A	C	C	A	A	A
7277	rs3712802	3	101817233	T	T	T	A	A	T
7278	rs6199015	3	101842424	A	A	A	A	A	A
7279	mCV23283853	3	106418717	G	G	G	G	A	G
7280	rs3671622	3	102314702	G	A	A	G	G	G
7281	rs4138887	3	102619416	T	T	T	A	A	T
7282	rs6347476	3	102772837	A	A	A	C	C	A
7283	rs4224146	3	102988527	A	A	A	A	A	A
7284	rs6253109	3	103139941	G	G	G	G	G	G
7285	rs3712827	3	103490418	G	A	A	A	A	G
7286	rs3714420	3	103515881	G	A	A	G	G	G
7287	rs3701653	3	103564280	C	A	A	A	A	C
7288	rs4224155	3	103751962	A	A	A	A	A	A
7289	rs4224148	3	103752315	G	G	G	G	G	G
7290	rs3688731	3	104208035	A	T	T	T	T	A
7291	rs6315710	3	104585700	C	C	C	C	C	C
7292	rs6188615	3	105330240	G	G	G	A	A	G
7293	rs6364731	3	105692661	A	A	A	A	A	A
7294	rs8236502	3	106220623	G	G	G	G	G	G
7295	rs8236505	3	106224738	A	G	G	A	A	A
7296	rs8240587	3	106225504	G	G	G	G	G	G
7297	rs8236508	3	106225921	A	T	T	A	A	A
7298	rs8253116	3	106226221	G	A	A	G	G	G
7299	rs6386146	3	106774874	A	A	A	A	A	A
7300	rs6354538	3	107175698	A	A	A	A	A	A
7301	rs3702359	3	107508882	A	C	C	A	C	A
7302	rs3686353	3	107544480	G	C	C	G	C	G
7303	rs3662271	3	107680188	G	G	G	G	A	G
7304	rs4224160	3	107686675	G	G	G	G	G	G
7305	rs3684333	3	107850823	G	C	C	C	C	C
7306	rs6259798	3	107851256	A	G	G	G	A	G
7307	rs8259688	3	108320670	G	G	G	G	G	G
7308	rs8259738	3	108323936	A	A	A	A	A	A
7309	rs8241998	3	108344014	A	A	A	A	A	A
7310	rs8259678	3	108349178	G	G	G	G	G	G
7311	rs8236521	3	108397012	A	A	A	A	A	A
7312	rs8236522	3	108397043	C	C	C	C	C	C
7313	mCV25436350	3	113449989	G	A	A	G	A	A
7314	rs6210304	3	109675077	C	C	C	C	C	C
7315	rs3672938	3	109847591	G	G	G	G	G	G
7316	rs3722681	3	110165817	A	A	A	G	G	A
7317	rs3704665	3	110229807	A	A	A	G	G	A
7318	rs6366730	3	110251230	A	A	A	G	G	A
7319	rs8256683	3	110372955	G	G	G	A/G	A	G
7320	mCV24793263	3	117251175	G	A	A	A	G	G
7321	rs3663873	3	110571048	G	G	G	A	G	G
7322	rs4224162	3	110640144	G	G	G	G	G	G
7323	rs3676545	3	110649631	G	G	G	A	G	G

7324	rs3656131	3	110947327	T	T	T	A	T	T
7325	mCV23378745	9	5761010	A	G	G	G	G	G
7326	rs3720740	3	111041753	A	A	A	A	A	A
7327	rs3696843	3	111168609	A	A	A	G	G	A
7328	rs6313013	3	111286362	A	A	A	G	G	A
7329	mCV23317479	9	5197667	A	G	G	G	G	G
7330	rs3674856	3	111611114	G	G	G	A	G	G
7331	rs3090825	3	111647306	G	G	G	G	G	G
7332	rs3711702	3	111705986	A	A	A	G	A	A
7333	rs3717172	3	111934855	G	A	A	G	G	G
7334	rs6211037	3	111988299	A	T	T	T	A	A
7335	rs6281357	3	112109659	A	A	A	G	A	A
7336	rs6199580	3	112477321	A	A	A	G	G	G
7337	rs3698700	3	112624069	A	A	A	T	A	A
7338	mCV23893269	9	3980881	A	C	C	C	C	C
7339	rs3721786	3	112749976	A	A	A	G	G	G
7340	rs3687751	3	113054672	G	G	G	A	A	A
7341	rs3694754	3	113073920	A	A	A	G	A	A
7342	rs6207837	3	113129958	G	G	G	A	A	A
7343	rs3718507	3	113237977	G	G	G	A	A	A
7344	rs3657795	3	114344827	G	G	G	C	G	G
7345	rs3022965	3	114351927	G	G	G	A	G	G
7346	rs6221470	3	114360910	G	G	G	A	G	G
7347	rs3682339	3	114414812	A	A	A	G	A	A
7348	rs3688988	3	114722164	A	G	G	A	G	G
7349	rs6179750	3	114804209	A	C	C	A	C	C
7350	rs3694286	3	115487510	G	A	A	G	A	A
7351	rs6290322	3	115696298	G	A	A	G	A	A
7352	rs6169590	3	115710049	G	G	G	G	G	G
7353	rs3689240	3	115777190	A	G	G	A	G	G
7354	rs3710419	3	116000138	A	G	G	G	G	G
7355	rs4224168	3	116288485	A	A	A	G	A	A
7356	rs3659643	3	116403695	T	A	A	A	A	A
7357	rs3722079	3	116461864	G	A	A	G	A	A
7358	rs6175127	3	116741394	A	A	A	A	A	A
7359	rs3668353	3	117171934	A	C	C	C	C	C
7360	rs3709137	3	117343865	C	C	C	G	C	C
7361	rs6214597	3	117350554	A	A	A	A	G	G
7362	rs3708412	3	118002787	G	G	G	A	A	G
7363	rs2020778	3	118037331	A	A	A	A	A	A
7364	rs6249418	3	118088000	G	G	G	G	G	G
7365	rs3709732	3	118315060	A	G	G	G	G	G
7366	rs3664868	3	118318914	G	A	A	G	G	A
7367	rs3707883	3	119036509	A	A	A	A	A	A
7368	rs6369822	3	119202135	G	G	G	G	G	G
7369	rs6232971	3	120074521	G	G	G	G	G	G
7370	rs6353079	3	120694394	A	A	A	A	A	A
7371	rs3658791	3	120952771	G	A	A	G	G	A
7372	rs3718783	3	121131740	G	G	G	A	A	G
7373	rs3669081	3	121192201	A	A	A	G	G	A
7374	rs6259281	3	121262108	G	C	C	G	C	C
7375	mCV23385188	3	121513280	C	C	C	C	A	C
7376	rs4224178	3	121593100	G	G	G	G	G	G
7377	rs4224186	3	121744885	A	A	A	A	G	A
7378	rs6212614	3	121841101	A	G	G	A	G	G
7379	rs3659836	3	122002332	G	A	A	A	G	A
7380	rs4224199	3	122397955	A	A	A	A	G	A
7381	rs4224197	3	122398195	G	G	G	G	A	G
7382	rs4224196	3	122398222	G	G	G	G	G	G
7383	rs2228908	3	122585612	T	T	T	T	A	T
7384	rs3691246	3	122856224	G	A	A	A	G	A
7385	rs6188758	3	123003410	A	A	A	A	A	A
7386	rs3681787	3	123554127	A	G	G	G	G	G
7387	mCV23483645	3	123767337	G	A	A	G	A	G
7388	rs3675720	3	123787212	G	A	A	G	A	G
7389	rs3704417	3	123846645	T	A	A	T	T	T
7390	rs3706670	3	123863052	A	G	G	A	A	A
7391	rs6212658	3	124028534	G	A	A	G	G	G
7392	rs3720182	3	124100505	G	A	A	G	G	G
7393	rs3707706	3	124412848	A	C	C	A	A	A
7394	rs6182907	3	124480088	A	G	G	A	A	A
7395	rs3699776	3	124840765	G	G	G	A	A	A
7396	rs3697140	3	125179187	G	A	A	G	G	G
7397	rs6254464	3	125891846	A	A	A	A	A	A

7398	rs3691045	3	126325091	A	G	G	A	A	A
7399	rs4224203	3	126347985	G	A	A	G	G	G
7400	rs3022968	3	126349073	G	A	A	G	G	G
7401	rs6287083	3	126359312	A	G	G	A	A	A
7402	rs8255228	3	126397223	A	G	G	A	A	A
7403	rs3671119	3	126894715	G	G	G	A	G	A
7404	rs3675048	3	126969163	G	G	G	A	G	A
7405	rs6220661	3	127039047	A	A	A	G	G	G
7406	rs3722725	3	127042830	T	T	T	A	A	A
7407	rs3702789	3	127084731	C	C	C	A	A	A
7408	rs3717432	3	127536252	G	G	G	A	A	A
7409	rs3698974	3	127544969	A	A	A	C	C	C
7410	rs6285599	3	127728173	G	G	G	A	A	A
7411	rs6377831	3	127995597	G	G	G	A	A	A
7412	rs3688504	3	128039511	A	A	A	G	G	G
7413	rs6164477	3	128196218	A	A	A	A	A	A
7414	rs6194090	3	128321186	G	G	G	A	A	A
7415	rs3688827	3	128500828	G	G	G	A	A	A
7416	rs3679962	3	128582235	G	G	G	A	A	A
7417	rs3670168	3	128820535	A	A	A	C	A	C
7418	rs3697858	3	129106005	C	C	C	A	C	A
7419	rs6363768	3	129189421	A	A	A	A	A	A
7420	rs3090387	3	129514464	A	A	A	A	A	A
7421	mCV23446189	3	129786008	G	G	A	G	A	G
7422	rs8253183	3	129862998	G	G	G	G	G	G
7423	rs8253136	3	129863358	A	A	A	A	A	A
7424	rs6364587	3	130373139	A	A	A	A	A	A
7425	rs6202228	3	130724949	A	A	A	A	A	A
7426	rs3679440	3	130739420	T	T	A	T	A	T
7427	rs6220280	3	130836961	G	G	G	G	G	G
7428	rs3725129	3	131172066	G	G	A	G	A	G
7429	rs4224205	3	131217946	A	A	A	A	A	A
7430	rs3658914	3	131411343	A	G	A	G	A	A
7431	rs6157007	3	131705669	A	A	A	A	A	A
7432	rs3660884	3	131803570	A	A	G	A	G	G
7433	rs3710354	3	132124986	C	C	A	C	A	C
7434	rs4224211	3	132129608	G	G	G	G	G	G
7435	rs3088954	3	132354130	T	T	T	T	T	T
7436	rs3665032	3	132519108	G	A	A	G	A	A
7437	rs6199211	3	132635283	A	A	A	A	A	A
7438	rs3659168	3	133247740	G	A	A	A	G	A
7439	rs6162982	3	133462063	A	A	A	A	A	A
7440	rs6347693	3	133608106	A	A	A	A	A	A
7441	rs3724110	3	134101271	C	A	A	A	A	A
7442	rs6397585	3	134745176	A	A	A	A	A	A
7443	rs3685539	3	134945023	A	G	G	G	G	G
7444	mCV23230498	3	135570407	G	G	G	G	A	A
7445	rs6347082	3	135470746	C	C	C	C	C	C
7446	rs6199240	3	135971946	G	G	G	G	G	G
7447	rs6305792	3	136332325	A	G	G	G	G	G
7448	rs4224231	3	136601744	G	G	G	G	G	G
7449	rs3719076	3	136696437	A	G	G	G	G	G
7450	rs3676039	3	136766271	G	A	A	G	A	A
7451	rs3676561	3	136766340	G	A	A	G	A	A
7452	rs6247823	3	137432871	A	A	A	A	A	A
7453	rs6320746	3	137632413	A	A	A	A	A	A
7454	rs3671858	3	137955491	A	C	C	C	C	C
7455	rs3706263	3	138105859	C	C	C	A	C	C
7456	mCV23030574	3	138721705	C	C	C	A	C	C
7457	mCV23030567	3	138721983	G	G	G	A	G	G
7458	mCV23030565	3	138730714	A	A	A	C	A	A
7459	mCV23030558	3	138731092	A	A	A	G	A	A
7460	mCV23030557	3	138731194	G	G	G	A	G	G
7461	mCV23030556	3	138731622	T	A	A	A	T	T
7462	mCV23030549	3	138731689	A	A	A	G	A	A
7463	mCV23030542	3	138732587	A	A	A	G	A	A
7464	mCV23030541	3	138732650	G	G	G	A	G	G
7465	mCV23030363	3	138732863	G	G	G	A	G	G
7466	mCV23030361	3	138733091	G	G	G	A	G	G
7467	mCV23030357	3	138733119	G	G	G	C	G	G
7468	mCV23030356	3	138733305	A	A	A	G	A	A
7469	mCV23030350	3	138733598	A	A	A	G	A	A
7470	mCV23030349	3	138734786	A	A	A	G	A	A
7471	mCV23030345	3	138734848	A	A	A	G	A	A

7472	mCV23030344	3	138734872	A	A	A	C	A	A
7473	mCV23030343	3	138735067	G	G	G	A	G	G
7474	mCV23030339	3	138735315	G	G	G	A	G	G
7475	mCV23030338	3	138735800	G	G	G	A	G	G
7476	mCV23030333	3	138735892	A	A	A	G	A	A
7477	mCV23030328	3	138736347	A	A	A	G	A	A
7478	mCV23030323	3	138737135	A	A	A	A	A	A
7479	mCV23030322	3	138737206	A	A	A	G	A	A
7480	mCV23030321	3	138737346	C	C	C	G	C	C
7481	mCV23030317	3	138737433	A	A	A	G	A	A
7482	mCV23030316	3	138737559	A	A	A	G	A	A
7483	mCV23030308	3	138737874	A	A	A	G	A	A
7484	mCV23030306	3	141587645	A	A	A	G	A	A
7485	mCV23030300	3	138739554	C	C	C	A	C	C
7486	mCV23030088	3	138740295	A	A	A	G	A	A
7487	mCV23030080	3	138741550	A	A	A	C	A	A
7488	mCV23030079	3	138741604	A	A	A	C	A	A
7489	mCV23030078	3	138741709	G	G	G	A	G	G
7490	mCV23030071	3	138743093	A	A	A	C	A	A
7491	mCV23030070	3	138743263	A	T	T	T	A	T
7492	mCV23030069	3	138743276	A	A	A	G	A	A
7493	mCV23030061	3	138743362	G	G	G	A	G	G
7494	mCV23030060	3	138743526	A	A	A	C	A	A
7495	mCV23030052	3	138743644	G	G	G	A	G	G
7496	mCV23030051	3	138743684	A	A	A	G	A	A
7497	mCV23030044	3	138743743	A	A	A	C	A	A
7498	mCV23030043	3	138743871	G	G	G	A	G	G
7499	mCV23030042	3	138743920	T	T	T	A	T	T
7500	mCV23030025	3	138744236	A	A	A	G	A	A
7501	rs4224261	3	138474090	A	A	A	A	A	A
7502	rs6359968	3	138546973	G	G	G	G	G	G
7503	rs3699249	3	138663643	A	T	T	A	A	A
7504	rs3669047	3	138773928	G	G	G	A	G	G
7505	rs3091034	3	139012303	A	A	A	A	A	A
7506	rs3659645	3	139214752	A	G	G	G	A	G
7507	rs6357455	3	139452899	A	C	C	C	C	A
7508	mCV24856311	3	140262118	A	A	A	G	A	A
7509	rs3089626	3	140010329	A	A	A	A	A	A
7510	rs3720498	3	140030643	A	A	A	G	A	A
7511	rs6299094	3	140146813	A	A	A	A	A	A
7512	rs3655208	3	140341393	A	A	A	G	A	A
7513	rs3679306	3	140561089	G	G	G	A	G	G
7514	rs3664998	3	140619815	G	G	G	A	G	G
7515	rs6373496	3	140647239	G	G	G	G	G	G
7516	rs4138815	3	140998672	G	A	A	G	A	A
7517	rs3090380	3	141341520	G	A	A	A	A	A
7518	rs6263345	3	141480213	G	G	G	G	G	G
7519	rs3673719	3	141531951	G	A	A	A	A	A
7520	rs3698531	3	142091053	A	A	A	G	G	G
7521	rs3718489	3	142338111	G	G	G	C	C	C
7522	rs4224267	3	142398573	T	T	T	T	T	T
7523	rs3091193	3	142398849	C	C	C	C	C	C
7524	rs6192206	3	142604537	A	A	A	A	A	A
7525	rs4224277	3	143131322	G	G	G	G	G	G
7526	rs3091029	3	143131555	A	A	A	A	A	A
7527	rs6290401	3	143193940	G	G	G	G	A	A
7528	rs6261585	3	143343124	A	A	A	G	A	A
7529	rs6258013	3	143453374	A	A	A	C	A	A
7530	rs3694521	3	143476580	G	G	G	A	A	A
7531	rs6407142	3	143619317	A	A	A	G	G	G
7532	rs6391253	3	144306167	C	A	A	C	A	A
7533	rs3677650	3	144611820	A	G	G	G	G	G
7534	rs3682091	3	144870033	G	A	A	A	G	G
7535	rs4224282	3	145001550	G	G	G	G	G	G
7536	rs8236528	3	145571450	G	G	G	G	G	G
7537	rs8243522	3	145572052	A	A	A	A	G	A
7538	rs8255972	3	145575488	A	A	A	A	A	A
7539	rs8255985	3	145575936	G	G	G	G	G	G
7540	rs8243132	3	145583459	A	A	A	A	A	A
7541	rs3688244	3	146003911	T	T	T	A	T	A
7542	rs6284325	3	146181663	A	A	A	A	A	A
7543	rs6157876	3	146508618	C	C	C	C	C	C
7544	rs3710548	3	146841800	G	G	G	A	A	A
7545	rs3022970	3	146873102	G	C	C	G	G	G

7546	rs3682093	3	146978526	A	G	G	A	A	A
7547	rs4224290	3	147157302	C	C	C	C	C	C
7548	rs3688583	3	147213351	A	A	A	G	A	A
7549	rs3022971	3	147306439	G	G	G	A	G	G
7550	rs6250754	3	147481201	A	A	A	A	A	A
7551	rs3706762	3	148152773	G	G	G	A	A	G
7552	rs4139604	3	148249896	A	A	A	G	G	A
7553	rs3090238	3	148272182	T	T	T	T	T	T
7554	rs6401225	3	148336754	G	G	G	A	A	G
7555	rs3683215	3	148793419	C	C	C	C	C	C
7556	rs3657112	3	148942421	A	A	A	G	G	A
7557	rs6300594	3	148966765	T	T	T	A	A	T
7558	rs6155693	3	149177520	A	A	A	A	A	A
7559	rs4224297	3	149393785	A	A	A	A	A	A
7560	rs4224296	3	149393844	G	G	G	G	G	G
7561	rs3722447	3	149523236	A	G	G	G	G	G
7562	rs6263248	3	150055497	G	G	G	G	G	G
7563	rs6261685	3	150310520	A	A	A	A	A	A
7564	rs3718587	3	150314430	C	C	C	G	C	C
7565	rs3696452	3	150512059	A	A	A	G	A	A
7566	rs3681780	3	150866693	C	C	C	A	A	A
7567	rs3721602	3	151198677	A	A	A	C	A	A
7568	rs6380272	3	151430016	C	C	C	C	C	C
7569	rs3724562	3	151923922	A	A	A	G	A	A
7570	rs6199296	3	152070005	A	A	A	A	A	A
7571	rs6161409	3	152074162	G	G	G	A	G	G
7572	rs6262668	3	152119478	G	G	G	A	G	G
7573	rs3676735	3	152366628	A	A	A	T	T	T
7574	rs3653892	3	152373112	G	G	G	A	A	A
7575	rs4224301	3	152391347	C	C	C	C	C	C
7576	rs4224298	3	152391603	G	G	G	G	G	G
7577	rs3695139	3	152956281	T	T	T	A	A	A
7578	rs3686068	3	152985388	G	A	A	G	G	G
7579	mCV25265490	3	153485338	G	G	G	A	A	A
7580	rs3662069	3	153028926	C	G	G	C	C	C
7581	rs6381060	3	153190646	C	C	C	C	C	C
7582	rs3089790	3	153351487	G	G	G	G	G	G
7583	rs6257013	3	153600398	G	G	G	G	G	G
7584	rs6297017	3	154293256	G	A	A	A	A	A
7585	rs3656403	3	154507439	G	A	A	A	A	G
7586	rs8253213	3	154545658	A	A	A	A	A	A
7587	rs8253212	3	154545779	A	A	A	A	A	A
7588	mCV25188584	3	155123336	A	G	G	A	G	G
7589	rs3716135	3	154921605	T	A	A	T	A	T
7590	rs4139152	3	155287410	G	G	G	G	G	G
7591	rs4224343	3	155353684	G	G	G	G	G	G
7592	rs6322390	3	155360428	C	C	C	C	C	C
7593	rs3656494	3	155382051	A	G	G	A	G	G
7594	rs3667807	3	155575484	A	G	G	A	G	A
7595	rs6245577	3	155867596	G	G	G	G	G	G
7596	rs3695386	3	156396523	A	G	G	A	G	G
7597	mCV24198179	3	157639416	G	A	A	G	G	G
7598	rs6157773	3	156960730	C	NONE	A	A	A	C
7599	rs3090130	3	157177959	A	A	A	A	A	A
7600	rs3697892	3	157203410	G	A	A	G	G	G
7601	rs3089034	3	157538916	A	A	A	A	A	A
7602	rs6201220	3	157547664	G	G	G	G	G	G
7603	rs3716357	3	157675785	A	G	G	A	A	A
7604	rs4137032	3	157906567	G	G	G	G	G	G
7605	rs6341867	3	158655934	A	A	A	A	A	A
7606	rs3667025	3	158614152	G	A	A	A	A	A
7607	rs6258754	3	158848787	G	G	G	G	G	G
7608	rs4136181	3	158999479	C	C	C	A	C	C
7609	rs3674566	3	159010475	G	G	G	A	G	G
7610	rs6216866	3	159675942	A	A	A	A	A	A
7611	rs3689155	3	160106091	C	A	A	A	A	A
7612	rs3666345	3	160298455	G	NONE	A	A	A	A
7613	rs6322812	3	160482205	A	A	A	A	A	A
7614	rs4228320	3	160527867	G	G	G	G	G	G
7615	rs6191908	4	133607851	C	C	C	A	A	A
7616	rs3655655	4	1645422	A	G	G	A	G	G
7617	rs6197409	4	66449150	A	G	G	G	G	G
7618	rs3666852	4	726286	G	A	A	G	A	A
7619	rs3657480	4	10782096	A	G	G	G	G	A

7620	rs4225002	4	154132368	G	G	G	G	G	G
7621	rs4232438	4	3476410	A	A	A	A	A	A
7622	rs4232433	4	3476658	G	G	G	G	G	G
7623	rs3695715	4	3649824	G	G	G	A	G	A
7624	rs6400920	4	3824829	C	C	C	C	C	C
7625	rs3702283	4	4494215	A	A	G	A	G	A
7626	rs6305295	4	5564700	A	A	A	A	A	A
7627	rs3089556	4	5702872	G	G	G	G	G	G
7628	rs6349397	4	6141330	G	G	G	G	G	G
7629	rs3716318	4	6157797	A	A	C	A	A	A
7630	rs6205855	4	6724822	A	A	A	A	A	A
7631	rs3674982	4	6820703	G	G	G	A	G	G
7632	rs3660863	4	7127435	A	A	A	G	A	A
7633	rs6337200	4	7459072	A	A	A	A	A	A
7634	rs3655175	4	8139388	G	G	G	A	G	G
7635	rs4224356	4	8534606	A	A	A	A	A	A
7636	rs6404602	4	8778812	A	A	A	A	A	A
7637	rs3655556	4	9508720	G	G	A	G	A	G
7638	rs4224376	4	9555959	T	A	T	T	T	T
7639	rs4224360	4	9556341	A	A	A	A	A	A
7640	rs6352515	4	9558115	T	T	T	T	T	T
7641	rs6260000	4	10002397	G	G	G	G	G	G
7642	rs6324271	4	10242036	A	A	G	G	G	A
7643	rs3719315	4	10713139	C	A	A	C	A	C
7644	rs3657371	4	10725903	C	A	A	A	A	C
7645	rs6317835	4	10823373	T	T	T	A	T	T
7646	rs4224381	4	10915152	G	G	G	G	G	G
7647	rs6291404	4	10931195	G	A	A	A	A	G
7648	rs6341373	4	11311121	G	G	G	G	G	G
7649	rs6196764	4	11408038	T	A	A	T	A	A
7650	rs4224387	4	11641126	A	A	A	A	A	A
7651	mCV22939387	4	15321444	A	G	A	A	A	A
7652	rs3701047	4	12339561	G	G	A	G	G	G
7653	rs6237951	4	12554425	G	G	G	G	G	G
7654	mCV22933619	4	15929694	T	NONE	A	A	A	A
7655	rs6319783	4	13814600	G	G	G	G	G	G
7656	rs4136031	4	14237734	A	G	G	G	G	G
7657	rs3693267	4	14345285	A	G	G	G	G	G
7658	rs6256059	4	14860611	A	A	A	A	A	A
7659	rs3680907	4	14958026	C	A	A	A	A	A
7660	mCV23717969	4	18178970	G	A	G	G	G	G
7661	rs3090919	4	15072128	A	G	G	G	G	G
7662	rs6237990	4	15196232	A	A	A	A	A	A
7663	rs3688067	4	15593522	T	T	A	A	A	A
7664	rs3688624	4	15593575	G	G	A	A	A	A
7665	rs3716868	4	15610542	G	G	A	A	A	A
7666	rs4224398	4	15903938	G	G	G	G	G	G
7667	rs4224391	4	15904154	G	G	G	G	G	G
7668	rs6352165	4	16500286	G	G	G	G	G	G
7669	rs6199418	4	16781620	T	T	T	T	T	T
7670	rs3709725	4	17416859	A	A	G	A	G	G
7671	rs6176582	4	17581384	A	A	G	A	G	G
7672	rs3715008	4	17814624	C	A	A	A	A	A
7673	rs3712309	4	17847510	A	A	G	G	G	G
7674	rs6287606	4	18045313	G	G	A	G	A	A
7675	rs3679281	4	18609900	A	A	A	G	A	A
7676	rs6222305	4	18796202	A	A	A	A	A	A
7677	rs6323735	4	19601811	T	T	T	T	T	T
7678	rs6157822	4	19658375	A	A	A	G	A	A
7679	rs3022977	4	19965713	A	A	A	G	A	A
7680	rs6360006	4	20448854	A	A	A	A	A	A
7681	rs3724961	4	20643790	G	G	G	A	G	G
7682	mCV22919271	4	24149712	G	G	A	A	A	G
7683	rs6371922	4	21491966	G	G	G	G	G	G
7684	rs6211596	4	21583992	G	G	G	A	G	G
7685	rs3091044	4	21675640	G	G	G	G	G	G
7686	rs4224405	4	21763745	G	G	G	G	G	G
7687	rs4224402	4	21764020	T	T	T	T	T	T
7688	mCV23689174	4	22356614	C	C	A	A	A	C
7689	rs3088632	4	22598894	G	G	G	G	G	G
7690	rs6347318	4	22841696	C	C	C	A	C	C
7691	rs3708026	4	22843907	G	G	G	A	G	G
7692	rs3698286	4	22846014	G	G	G	A	G	G
7693	rs3653593	4	22884502	A	A	A	G	A	A

7694	rs3655029	4	23211273	G	G	G	C	G	G
7695	mCV22911972	4	27231572	G	G	G	G	G	G
7696	rs6287534	4	23825087	A	A	A	A	A	A
7697	rs3712649	4	23935189	A	A	G	G	G	A
7698	rs6175954	4	24029684	T	T	A	A	A	T
7699	rs3680190	4	24724946	A	A	C	C	C	A
7700	rs6246002	4	24831650	A	A	A	A	A	A
7701	rs4224426	4	25176238	A	A	G	G	G	A
7702	rs3657154	4	25277549	A	A	C	C	C	A
7703	rs6204487	4	26277706	G	G	G	G	G	G
7704	rs3681849	4	26609626	G	G	A	A	A	G
7705	rs6383319	4	27109771	G	G	A	A	A	G
7706	rs6401234	4	27434643	A	A	A	A	A	A
7707	rs3710785	4	27910670	G	G	A	A	A	G
7708	rs6354547	4	28107771	A	A	A	A	A	A
7709	rs6246362	4	29421164	G	G	G	G	G	G
7710	rs3701432	4	29487208	C	C	C	A	C	C
7711	rs6392393	4	30267227	G	G	G	G	G	G
7712	rs3688049	4	30505056	A	A	G	G	G	A
7713	rs3663744	4	31215491	A	A	A	G	A	A
7714	rs3665020	4	31215724	A	A	A	G	A	A
7715	rs6157431	4	31287346	G	G	G	G	G	G
7716	rs6177140	4	31796103	G	G	G	A	G	G
7717	rs6221374	4	31810940	G	G	G	G	G	G
7718	rs3726592	4	32219475	A	A	A	G	A	A
7719	rs3727171	4	32219527	G	G	G	A	G	G
7720	rs3705575	4	32224948	G	G	G	A	G	G
7721	rs3674908	4	32441103	A	A	A	G	A	A
7722	rs6387912	4	32668639	G	G	G	G	G	G
7723	rs3663938	4	32787618	A	A	G	G	G	G
7724	rs3671973	4	32807791	G	G	A	A	A	A
7725	rs3090673	4	33072682	G	G	G	G	G	G
7726	rs4224427	4	33253944	G	G	A	G	A	A
7727	rs6389474	4	33315844	G	G	A	G	A	A
7728	rs6255328	4	34017413	G	G	G	G	G	G
7729	rs6341443	4	35103453	G	G	G	G	G	G
7730	rs6240486	4	35513695	A	A	A	A	A	A
7731	rs3689359	4	35654927	G	A	A	A	A	A
7732	rs3719299	4	36221145	A	G	G	G	G	G
7733	rs6411840	4	36646878	G	G	G	G	G	G
7734	rs6245131	4	37141581	A	A	A	A	A	A
7735	rs3680929	4	37477262	G	A	A	A	A	A
7736	rs4140307	4	37528829	T	A	A	A	A	A
7737	rs3654708	4	37675112	A	T	T	T	T	T
7738	rs6220425	4	37861184	G	G	G	G	G	G
7739	rs3689942	4	37917800	G	A	A	A	A	A
7740	rs3684104	4	38414337	G	G	G	A	A	A
7741	rs6409228	4	38767611	A	A	A	A	A	A
7742	rs3665192	4	39174790	A	A	A	C	C	C
7743	rs3702229	4	39247915	T	T	T	A	A	A
7744	rs3714457	4	39258104	A	A	A	G	G	G
7745	rs3089966	4	39357645	A	A	A	A	A	A
7746	rs4224429	4	39542808	A	A	A	A	A	A
7747	mCV25401801	4	45892040	C	C	C	G	G	G
7748	rs6326201	4	39722993	T	T	T	T	T	T
7749	rs6222273	4	40093177	G	G	G	G	G	G
7750	rs4224439	4	40896146	C	C	C	C	C	C
7751	rs6344279	4	41211815	A	A	A	A	A	A
7752	rs4224449	4	41572015	A	A	A	A	A	A
7753	rs4138630	4	41748426	C	A	A	C	A	A
7754	rs4224450	4	41832242	C	C	C	C	C	C
7755	rs3090286	4	41837117	G	G	G	G	G	G
7756	rs3023448	4	41837153	T	T	T	T	T	T
7757	mCV23123688	4	44396289	G	C	C	C	C	C
7758	rs3141910	4	42290028	G	G	G	A	G	G
7759	rs3674325	4	42317351	G	G	G	A	G	G
7760	rs3674824	4	42317387	A	A	A	G	A	A
7761	rs4138960	4	42317437	T	T	T	A	T	T
7762	rs3090401	4	42441968	G	G	G	G	G	G
7763	rs3674068	4	42462482	A	G	G	G	G	G
7764	rs4137998	4	42468309	A	A	A	G	A	A
7765	rs6319833	4	42895715	G	G	G	G	G	G
7766	rs6336373	4	42974778	G	G	G	A	G	G
7767	rs4224453	4	42977245	G	G	G	G	G	G

7768	rs3689747	4	43167470	A	G	G	A	G	G
7769	rs3725792	4	43477894	G	A	A	A	G	G
7770	rs3089096	4	43624005	G	G	G	G	G	G
7771	rs6392573	4	43936825	A	A	A	A	A	A
7772	rs4224459	4	44561663	A	A	A	A	A	A
7773	rs6313392	4	44568868	A	NONE	C	A	A	A
7774	rs3665393	4	44721094	G	A	A	G	G	G
7775	rs3712230	4	44733653	G	G	G	G	G	G
7776	rs3668228	4	44764150	A	G	G	A	A	A
7777	rs3715030	4	44950097	A	G	G	A	G	A
7778	rs3694836	4	45217986	G	G	G	A	G	A
7779	rs6274863	4	45243885	G	G	G	G	G	G
7780	rs4224465	4	45417180	A	A	A	A	A	A
7781	rs6277768	4	45590942	C	C	C	C	C	C
7782	rs4224470	4	45639567	A	A	A	A	A	A
7783	rs3720736	4	45719580	G	C	C	C	C	C
7784	rs3707178	4	46281237	A	G	G	A	G	A
7785	rs3678593	4	46286806	A	G	G	A	G	A
7786	rs3679520	4	46397274	A	G	G	G	G	G
7787	rs6268280	4	46628482	A	A	A	A	A	A
7788	rs3663355	4	47358427	C	A	A	A	A	A
7789	rs6176024	4	47454282	G	G	G	G	G	G
7790	rs3699346	4	47809301	G	A	A	A	A	A
7791	rs3089035	4	48192717	A	A	A	A	A	A
7792	rs6248523	4	48298996	A	A	A	A	A	A
7793	rs3726023	4	48551778	G	A	A	A	A	A
7794	rs6316253	4	49074896	G	A	A	A	G	A
7795	rs3701176	4	49095375	A	G	G	A	A	A
7796	rs3698382	4	49140268	A	G	G	A	A	A
7797	rs3677601	4	49236356	A	C	C	A	A	A
7798	rs6171170	4	49667453	G	G	G	G	G	G
7799	rs6227828	4	50661597	G	G	G	G	G	G
7800	rs3676423	4	51035410	C	A	A	C	C	C
7801	rs6250250	4	51301901	T	T	T	T	T	T
7802	rs6378640	4	51598240	G	G	G	G	G	G
7803	rs3673502	4	52008507	A	C	C	A	A	A
7804	rs8271724	4	52294378	T	T	T	T	T	T
7805	rs8271462	4	52334774	G	G	G	G	G	G
7806	rs6246995	4	52338404	A	A	A	A	A	A
7807	rs8266692	4	52352531	A	A	A	A	A	A
7808	rs8266691	4	52354228	G	G	G	G	G	G
7809	rs8258667	4	52373452	A	A	A	A	A	A
7810	rs8258666	4	52389392	G	G	G	G	G	G
7811	rs4224486	4	52506809	G	G	G	G	G	G
7812	rs4139731	4	52706199	G	A	A	G	G	G
7813	rs3672925	4	52718080	T	A	A	T	T	T
7814	rs6287441	4	52957284	C	C	C	C	C	C
7815	rs8258806	4	53304800	G	G	G	G	G	G
7816	rs8258747	4	53317106	C	C	C	C	C	C
7817	rs8258726	4	53332671	A	A	A	A	A	A
7818	rs8258680	4	53361352	A	A	A	A	A	A
7819	rs3690224	4	53705442	A	A	A	G	A	A
7820	rs6206997	4	53707282	T	T	T	T	T	T
7821	rs3661339	4	53808261	A	A	A	G	A	A
7822	rs3657529	4	53909507	T	T	T	A	T	T
7823	rs3677770	4	53964528	A	A	A	C	C	A
7824	rs3715031	4	53968543	G	G	G	C	G	G
7825	rs6193501	4	54222818	T	T	T	T	T	T
7826	rs3727011	4	54341123	G	G	G	A	G	G
7827	rs3699453	4	54821629	A	A	A	T	A	A
7828	rs3670712	4	54827183	G	G	G	A	G	G
7829	rs3701061	4	54831193	G	G	G	A	G	G
7830	rs6412095	4	55310909	C	C	C	C	C	C
7831	rs6299312	4	55623580	C	C	C	C	C	C
7832	rs3661536	4	55796518	A	A	A	G	A	G
7833	rs4224501	4	56012680	A	A	A	G	A	G
7834	rs4224497	4	56012863	A	A	A	A	A	A
7835	rs3719355	4	56025397	G	G	G	A	G	A
7836	rs3726529	4	56029436	A	A	A	G	A	G
7837	rs3665812	4	56115059	A	A	A	G	A	G
7838	rs4224504	4	56135608	G	G	G	G	G	G
7839	rs3664867	4	56255644	A	A	A	G	A	G
7840	rs3090720	4	56476332	G	G	G	G	G	G
7841	rs6306804	4	56841851	G	G	G	G	G	G

7842	rs3714431	4	56977671	A	G	G	G	G	A
7843	rs4224506	4	57124385	G	G	G	G	G	G
7844	rs6307352	4	57546640	C	A	A	A	A	A
7845	rs6312158	4	57587494	G	G	G	A	G	G
7846	rs3091157	4	57645680	G	G	G	G	G	G
7847	rs6333919	4	58087050	G	A	A	G	A	A
7848	rs3699688	4	58225861	A	G	G	A	G	G
7849	rs4224523	4	58310030	C	C	C	C	C	C
7850	rs4224515	4	58310275	C	C	C	A	C	C
7851	rs3715009	4	58656701	G	G	G	A	G	G
7852	rs3671277	4	58712637	A	A	A	G	A	A
7853	rs6271356	4	58944260	G	G	G	G	G	G
7854	rs3713888	4	59015467	A	G	G	G	G	G
7855	rs3712541	4	59103430	A	A	A	G	A	A
7856	mCV24089992	4	63181926	A	A	A	A	T	T
7857	rs6406401	4	60519675	C	C	C	C	C	C
7858	rs3690581	4	61034761	A	A	A	G	A	G
7859	rs6284397	4	61125120	G	G	G	G	G	G
7860	rs3694308	4	61281669	C	C	C	A	C	C
7861	rs6206747	4	61967477	G	G	G	G	G	G
7862	mCV24726722	4	65141101	A	G	A	A	A	A
7863	rs6325763	4	62930919	A	A	A	A	A	A
7864	rs8276722	4	63508070	G	G	G	G	G	G
7865	rs8276721	4	63508212	A	A	A	A	A	A
7866	rs6406432	4	63508911	G	G	G	G	G	G
7867	rs8276731	4	63516923	G	G	G	G	G	G
7868	rs8275764	4	63559045	G	G	G	G	A	G
7869	rs6244083	4	63604779	A	A	A	A	A	A
7870	rs8275844	4	63627490	C	C	C	C	G	C
7871	rs3089893	4	64278800	A	A	A	A	A	A
7872	rs6403138	4	64068548	A	A	A	A	A	A
7873	rs3706082	4	64134751	A	A	A	A	A	A
7874	rs6299928	4	65078608	G	A	G	G	G	G
7875	mCV24456251	4	65355723	G	G	G	G	G	G
7876	rs3156208	4	65500529	G	A	A	A	A	A
7877	rs3718146	4	65556956	A	G	G	G	G	G
7878	rs6183656	4	66039237	G	G	G	G	G	G
7879	rs3670404	4	66109649	C	NONE	G	G	G	G
7880	rs3692150	4	66306406	G	A	A	A	A	A
7881	rs6410123	4	67126144	A	A	A	A	A	A
7882	rs6198889	4	67096191	A	A	A	A	A	A
7883	rs3688323	4	68566012	A	G	G	G	G	G
7884	rs3710245	4	68727690	G	A	A	A	A	A
7885	rs2020526	4	68757743	A	G	G	G	G	G
7886	rs6156020	4	69036017	C	NONE	G	G	G	G
7887	rs3654162	4	69241096	A	A	A	G	A	A
7888	rs3696361	4	69340056	A	G	G	G	G	G
7889	rs4139680	4	69344093	C	A	A	A	A	A
7890	rs3716506	4	69807290	G	A	A	A	A	A
7891	rs3684586	4	69896726	G	A	A	A	A	A
7892	mCV24116550	4	73037518	C	A	A	A	C	A
7893	rs6222684	4	70467819	G	A	A	A	G	A
7894	rs3696858	4	70489241	A	G	G	G	NONE	G
7895	rs3672178	4	70923913	G	A	A	A	A	A
7896	rs3705437	4	71343753	A	G	G	G	G	G
7897	rs6355846	4	71745507	A	A	A	A	A	A
7898	rs6177460	4	72301777	C	A	A	A	C	A
7899	rs3722264	4	72303838	C	A	A	A	C	A
7900	rs3683713	4	72358297	A	G	G	G	A	G
7901	rs3654493	4	73104348	A	G	G	G	A	G
7902	rs6232963	4	73133794	G	G	G	G	G	G
7903	rs3723666	4	73603270	C	G	G	G	C	G
7904	rs6217653	4	74017463	A	A	A	A	A	A
7905	rs3704239	4	74579476	A	A	G	G	A	G
7906	rs3706742	4	74791145	A	A	C	C	A	C
7907	rs6409644	4	74984365	A	A	A	A	A	A
7908	rs3658567	4	75113177	A	A	C	C	A	C
7909	rs3708471	4	75144974	G	G	A	A	A	A
7910	rs3672851	4	75169107	A	A	G	G	G	G
7911	mCV24387306	4	78487615	G	A	G	G	A	G
7912	rs6392653	4	75715655	NONE	NONE	A	A	A	A
7913	rs6181093	4	76145433	G	G	G	G	G	G
7914	rs3672159	4	76148373	G	G	A	A	A	A
7915	rs16262	4	76240223	A	A	G	G	G	G

7916	rs3678877	4	76475498	A	A	G	G	G	G
7917	rs3704069	4	76690583	G	G	A	A	A	A
7918	mCV24050012	4	79741856	A	G	A	A	A	A
7919	rs3678562	4	76845234	A	A	G	G	G	G
7920	rs3669136	4	76913204	C	C	A	A	A	A
7921	rs3676222	4	76917261	G	G	A	A	A	A
7922	rs6384996	4	76924650	G	G	G	G	G	G
7923	rs6280772	4	77627839	G	G	G	G	G	G
7924	rs3692459	4	77639360	G	A	G	G	A	G
7925	mCV25398332	4	81383006	A	G	A	A	G	A
7926	rs6288187	4	78907473	G	G	G	G	G	G
7927	rs6211012	4	79063020	A	A	A	A	A	A
7928	rs4224534	4	79229296	A	A	A	A	A	A
7929	rs4224533	4	79229355	T	A	T	T	T	T
7930	rs2020477	4	79371964	G	A	G	G	A	G
7931	rs3708061	4	79579536	G	G	A	A	G	A
7932	rs6270012	4	79711552	A	G	G	G	G	G
7933	rs6290974	4	80409897	A	A	A	A	A	A
7934	rs6258088	4	80891223	G	G	C	C	G	C
7935	rs3088838	4	80908277	A	T	A	A	T	A
7936	rs3676928	4	81040027	A	A	G	G	A	G
7937	rs4224556	4	81074517	A	A	A	A	A	A
7938	rs3692661	4	81081071	G	G	A	A	G	A
7939	rs3704935	4	81116669	C	A	A	A	A	A
7940	rs3699276	4	81462943	G	G	A	A	G	A
7941	rs6309721	4	81528478	G	G	G	G	G	G
7942	rs3674783	4	82076032	A	A	G	G	A	G
7943	rs3700579	4	82213793	G	G	A	A	G	G
7944	rs6335385	4	82670739	C	C	C	C	C	C
7945	rs6379875	4	82828564	A	A	A	A	A	A
7946	rs3661848	4	83167260	A	G	G	G	G	G
7947	rs4224569	4	83663402	C	C	C	C	C	C
7948	rs6247310	4	83825940	A	A	A	A	A	A
7949	rs3712258	4	84336328	A	NONE	G	G	A	G
7950	rs3717837	4	84363199	G	A	A	A	G	A
7951	rs3711477	4	84506191	G	G	G	A	G	A
7952	rs3685196	4	84507910	G	G	G	A	G	A
7953	rs3707373	4	85207633	G	G	G	A	G	A
7954	rs3655671	4	85542516	A	C	A	C	A	C
7955	rs6200787	4	85554799	A	A	A	A	A	A
7956	rs3659947	4	85683656	A	A	C	A	C	A
7957	rs3088625	4	85985875	C	C	C	C	C	C
7958	rs6388770	4	86216391	G	G	G	G	G	G
7959	rs3687556	4	86527963	C	C	A	C	A	C
7960	mCV23972594	4	90142651	G	A	A	A	A	A
7961	rs6306627	4	86968512	G	G	G	G	G	G
7962	rs3718205	4	87204885	C	NONE	G	G	G	G
7963	rs3707402	4	87429049	G	G	A	G	G	G
7964	rs4224587	4	87615408	G	A	A	A	G	A
7965	rs3721333	4	87835232	NONE	A	G	A	A	A
7966	rs3658697	4	87878838	A	C	C	C	C	C
7967	rs3682389	4	88768107	G	G	A	G	G	G
7968	rs6294308	4	88804369	A	C	C	C	C	C
7969	rs3676999	4	89060498	G	A	A	A	A	A
7970	rs3678990	4	89587013	T	A	A	A	A	A
7971	rs3712721	4	89683958	G	A	A	G	A	A
7972	rs4136370	4	89684174	A	A	A	C	A	A
7973	rs6252282	4	89853730	A	A	A	G	G	G
7974	rs4224594	4	89981350	G	G	G	G	G	G
7975	rs6266191	4	90448312	C	G	G	G	G	G
7976	rs6245643	4	90913070	A	G	G	G	G	G
7977	rs3686220	4	91044506	G	A	A	G	G	G
7978	mCV23916851	4	94855267	A	G	G	A	A	A
7979	rs6202006	4	91160209	C	C	C	C	C	C
7980	rs6271003	4	91308682	NONE	A	A	A	A	A
7981	rs3683848	4	91627051	A	C	C	C	C	C
7982	rs3657531	4	91796583	G	G	G	A	A	A
7983	rs3658161	4	91796688	T	A	A	T	T	T
7984	rs3686234	4	91831178	A	A	A	G	G	G
7985	rs3694739	4	92403280	A	A	A	T	T	T
7986	rs3684103	4	92572733	G	A	A	A	A	A
7987	rs3692152	4	92698220	A	G	G	A	A	A
7988	rs3663299	4	92762664	G	G	G	A	G	A
7989	rs3090406	4	92825355	A	A	A	A	A	A

7990	rs3682102	4	92867874	A	A	A	G	G	G
7991	rs6230422	4	92877333	A	A	A	A	A	A
7992	rs3704040	4	93884466	A	G	G	A	A	A
7993	rs3715056	4	93184069	G	G	G	A	A	A
7994	rs4224604	4	93237510	A	A	A	A	A	A
7995	rs3676884	4	93252122	A	A	A	G	A	G
7996	rs6159440	4	93309734	G	G	G	G	G	G
7997	rs3687764	4	94079664	C	A	A	A	C	A
7998	mCV23905937	4	97833477	A	G	G	A	A	A
7999	rs3711973	4	94235469	A	G	G	A	A	A
8000	rs3659791	4	94278186	T	A	A	T	T	T
8001	rs6313390	4	94402041	C	C	C	C	C	C
8002	rs4224615	4	94624868	A	A	A	A	A	A
8003	rs4224623	4	95343094	T	T	T	T	T	T
8004	rs6182361	4	95084745	A	A	A	A	A	A
8005	rs6263284	4	95333512	G	G	G	G	G	G
8006	rs6282784	4	96423965	T	T	T	T	T	T
8007	mCV24740485	4	100172150	C	A	A	C	C	C
8008	rs4224627	4	96899103	A	A	A	A	A	A
8009	rs4224626	4	96899157	A	A	A	A	A	A
8010	rs6255772	4	97203774	A	G	G	G	G	G
8011	rs3705454	4	97279502	G	G	G	A	G	G
8012	rs3670312	4	97475372	A	C	C	A	A	A
8013	rs3671527	4	97475574	A	A	A	G	G	A
8014	rs3680865	4	97482705	C	C	C	G	G	C
8015	rs6299159	4	97892739	G	G	G	G	G	G
8016	rs3682140	4	98140809	C	A	A	A	A	A
8017	mCV22602426	4	101827515	C	A	A	C	C	C
8018	rs3655355	4	98808683	A	A	A	A	A	A
8019	rs4139841	4	98832184	A	A	A	G	G	A
8020	rs3699252	4	99161083	T	T	T	A	A	A
8021	rs3654061	4	99324599	C	A	A	C	C	C
8022	rs6295110	4	99439814	A	A	A	A	A	A
8023	rs6371895	4	99795531	G	G	G	G	G	G
8024	rs3720743	4	99949965	A	G	G	A	A	A
8025	rs6272719	4	100640566	G	G	G	G	G	G
8026	rs3089192	4	100850043	G	G	G	G	G	G
8027	rs6345118	4	101514488	A	A	A	A	A	A
8028	mCV24675121	4	105107123	A	A	A	A	G	A
8029	mCV24675120	4	105107131	G	G	G	G	C	G
8030	mCV24675109	4	105107401	G	G	G	G	A	G
8031	mCV24675108	4	105111447	A	A	G	A	G	A
8032	mCV24675107	4	105113571	G	G	A	G	A	G
8033	mCV24675097	4	105113740	C	C	A	C	A	C
8034	mCV24675096	4	105113763	G	G	G	G	A	G
8035	mCV24675085	4	102130158	G	G	G	G	G	G
8036	mCV24675083	4	105115473	A	A	A	A	G	A
8037	mCV24675073	4	105115554	A	A	A	A	G	A
8038	mCV24675072	4	105115576	G	G	G	G	A	G
8039	mCV24675071	4	105117917	G	G	G	G	A	G
8040	mCV24675061	4	102134086	G	G	A	G	A	G
8041	mCV24675049	4	105118548	A	A	G	A	G	A
8042	mCV24674775	4	105119055	A	A	A	A	G	A
8043	mCV24674764	4	105119225	A	A	A	A	T	A
8044	mCV24674763	4	105119275	A	A	A	A	G	A
8045	mCV24674762	4	105119428	G	G	G	G	A	G
8046	mCV23364082	4	105121490	A	A	G	A	G	A
8047	mCV23364267	4	105123309	C	C	C	C	A	C
8048	mCV23364277	4	105123701	G	G	G	G	A	G
8049	mCV23364278	4	105124131	A	A	G	A	G	A
8050	mCV23364291	4	105124504	C	C	C	C	A	C
8051	mCV23364301	4	105124843	C	C	C	C	A	C
8052	mCV23364302	4	105125797	A	A	C	A	C	A
8053	mCV23364314	4	105126375	A	A	A	A	G	A
8054	mCV23364315	4	105126933	A	A	C	A	C	A
8055	mCV23364334	4	105126977	G	G	A	G	A	G
8056	mCV23364336	4	102143300	A	A	G	A	G	A
8057	mCV23364347	4	102143302	A	A	G	A	G	A
8058	mCV23364348	4	102143395	G	G	A	G	A	G
8059	mCV23364523	4	105128335	C	C	A	A	A	C
8060	mCV23364533	4	105128934	G	G	G	G	A	G
8061	mCV23364534	4	105128936	T	T	T	T	A	T
8062	mCV23364535	4	105129051	A	A	G	A	G	A
8063	mCV23364544	4	105129330	C	C	A	C	A	C

8064	mCV24674738	4	105132255	A	A	G	A	G	A
8065	mCV24674726	4	105132422	A	NONE	G	A	G	A
8066	mCV24674725	4	105132615	A	A	G	A	G	A
8067	mCV24674714	4	105133006	A	A	A	A	G	A
8068	mCV24674713	4	105134552	G	G	G	G	A	G
8069	mCV24674702	4	105135101	A	A	A	A	G	A
8070	mCV23882646	4	105136606	G	G	G	G	C	G
8071	mCV23882645	4	105137583	G	G	G	G	A	G
8072	mCV23881968	4	105138998	G	G	G	G	A	G
8073	mCV23881967	4	105139003	C	C	C	C	A	C
8074	mCV23881959	4	105139077	G	G	G	G	A	G
8075	mCV23881958	4	105139137	G	G	G	G	A	G
8076	mCV23881957	4	105139342	A	A	A	A	G	A
8077	mCV23881949	4	105140592	A	A	A	A	G	A
8078	mCV23881948	4	105140758	A	A	A	A	G	A
8079	mCV23881929	4	105141066	G	G	A	A	G	G
8080	mCV23881916	4	105142073	G	G	G	A	G	G
8081	mCV23881906	4	105142232	G	G	A	A	A	G
8082	mCV23881905	4	102158673	C	C	A	A	A	C
8083	mCV23881631	4	105143259	G	G	G	G	A	G
8084	mCV23881619	4	105145529	A	A	A	A	T	A
8085	mCV23881609	4	105145547	G	G	G	G	A	G
8086	mCV23881607	4	105145665	A	A	G	G	G	A
8087	rs3723703	4	101657612	A	G	G	A	A	G
8088	rs3726736	4	102512069	G	G	G	A	G	G
8089	rs6259114	4	102518072	G	G	G	A	G	G
8090	rs3695339	4	102860415	G	G	G	A	G	A
8091	rs6181706	4	103012113	A	A	A	A	A	A
8092	rs4224632	4	103150043	A	A	A	A	A	A
8093	rs3662056	4	103451398	A	A	G	A	A	A
8094	rs3715719	4	103509041	C	C	A	C	C	C
8095	rs6263289	4	103544865	A	A	G	A	A	A
8096	mCV23486224	4	107320896	G	G	G	A	A	G
8097	rs3702979	4	104232823	A	A	G	A	A	A
8098	rs3664801	4	104338661	A	A	A	G	G	A
8099	mCV24667075	4	107860528	A	A	T	T	A	A
8100	rs3088443	4	104580676	G	G	G	G	G	G
8101	rs3670223	4	104826849	G	G	G	A	A	G
8102	rs6386224	4	105007136	A	A	A	A	A	A
8103	rs3089990	4	105368437	A	A	A	A	A	A
8104	rs3706271	4	105421171	G	G	G	A	A	G
8105	rs3721642	4	105571371	A	A	G	A	G	A
8106	rs2020713	4	105763717	A	A	A	A	A	A
8107	rs2020712	4	105763801	A	A	A	A	A	A
8108	rs3707370	4	105894184	G	G	A	G	G	G
8109	rs3670398	4	106080858	C	C	G	C	C	C
8110	rs3709496	4	106176999	G	G	A	G	G	G
8111	rs4224656	4	106222434	T	T	T	T	T	T
8112	rs4224650	4	106222706	A	A	A	A	A	A
8113	rs6184116	4	106550698	C	C	C	C	C	C
8114	rs4224682	4	106746452	A	A	A	A	A	A
8115	rs3664637	4	106910140	C	C	C	A	C	C
8116	mCV22668736	4	111006185	T	T	T	A	T	T
8117	rs6226080	4	107056497	A	A	A	G	A	A
8118	rs4224693	4	107406921	A	A	A	A	A	A
8119	rs6337035	4	107411139	G	G	G	G	G	G
8120	rs3710865	4	107443942	A	A	G	A	A	A
8121	rs3695162	4	107621073	T	T	A	A	T	T
8122	rs4221960	4	107798885	A	A	G	G	A	A
8123	rs4221958	4	107799065	A	A	A	A	A	A
8124	rs6289137	4	108244509	T	T	T	T	T	T
8125	rs3710617	4	108380717	G	G	A	A	G	G
8126	rs6301922	4	108585619	G	G	G	G	G	G
8127	rs6193537	4	108912026	G	G	A	G	G	G
8128	mCV25387389	4	103636144	A	A	G	A	G	A
8129	rs3670382	4	109186770	A	A	G	A	A	A
8130	rs3686650	4	109200409	A	A	C	A	A	A
8131	rs6319794	4	109376235	A	A	A	A	A	A
8132	rs3659850	4	109639885	A	A	G	G	A	A
8133	rs3706233	4	109661745	G	G	A	G	G	G
8134	rs3022995	4	109717402	G	G	G	G	G	G
8135	rs3709486	4	109968331	G	G	A	G	G	G
8136	rs6393361	4	110127252	A	A	A	A	A	A
8137	mCV23198207	4	113411887	C	A	A	C	A	A

8138	rs3708362	4	110391532	G	G	G	A	G	G
8139	rs3725330	4	111058533	G	G	G	A	G	G
8140	rs3726406	4	111058678	A	A	G	A	A	A
8141	mCV24303778	4	114598120	A	G	A	A	G	A
8142	rs3687391	4	112710379	A	A	C	C	A	A
8143	rs6235260	4	112826940	G	G	G	G	G	G
8144	rs3722041	4	112859123	C	C	A	C	C	C
8145	mCV24645877	4	116109217	G	A	G	G	A	G
8146	rs3670129	4	113278448	A	A	G	A	A	G
8147	rs8249610	4	113832407	G	G	G	G	G	G
8148	rs3022993	4	113832609	G	G	A	G	G	A
8149	rs8239732	4	113839267	A	A	G	A	A	G
8150	rs8247950	4	113840561	G	G	A	G	G	A
8151	rs6173535	4	114201322	A	A	A	A	A	A
8152	rs8239976	4	114350886	T	T	T	T	T	T
8153	rs8236540	4	115044756	G	G	G	G	G	G
8154	rs6249803	4	115095280	G	A	A	G	A	A
8155	mCV24264185	4	118299804	A	A	A	C	A	A
8156	rs3689378	4	115293106	G	G	A	G	G	A
8157	rs4224732	4	115630977	G	G	G	G	G	G
8158	rs3696331	4	115756451	A	A	G	A	A	G
8159	rs3724818	4	115842883	G	G	G	A	G	G
8160	rs6172225	4	115918128	G	G	G	G	G	G
8161	rs3699061	4	115937492	A	G	G	A	G	G
8162	rs6379844	4	116247572	C	C	C	C	C	C
8163	rs3672234	4	116414859	G	G	G	A	G	G
8164	rs3692563	4	116737689	A	A	A	G	A	A
8165	rs3159805	4	117028968	C	C	C	C	C	C
8166	mCV22634282	4	121090958	G	A	A	A	A	G
8167	rs6195446	4	117332676	A	G	A	A	G	A
8168	rs3661670	4	117359996	G	G	A	G	G	A
8169	rs3664245	4	117621371	G	G	G	G	G	G
8170	rs3695349	4	117643049	G	G	A	G	G	A
8171	rs3688968	4	118066883	NONE	G	G	A	G	A
8172	rs3726907	4	118140601	C	C	A	C	C	C
8173	rs6180597	4	118162445	A	A	A	A	A	A
8174	rs6333351	4	118831743	G	G	G	G	G	G
8175	rs3678308	4	119089771	G	G	G	A	G	G
8176	rs4224744	4	119149097	G	G	G	A	G	A
8177	rs4224745	4	119149165	G	G	G	G	G	G
8178	rs3675136	4	119381586	G	G	A	A	A	G
8179	rs6338910	4	119399740	A	A	A	A	A	A
8180	rs3675629	4	119404209	A	A	A	C	A	A
8181	rs8276607	4	119420442	A	A	A	A	A	A
8182	rs8276586	4	119427150	A	A	A	A	A	A
8183	rs4224746	4	121227093	A	A	A	A	A	A
8184	rs6296781	4	121193166	A	A	A	A	A	A
8185	rs3702881	4	121182316	G	A	A	A	A	G
8186	rs4224757	4	122277336	G	G	G	G	G	G
8187	rs3682864	4	122316985	G	A	G	A	G	A
8188	rs6207969	4	122542570	G	G	G	G	G	G
8189	rs3714811	4	122578236	A	A	A	G	A	A
8190	rs3666419	4	122592298	G	G	G	A	G	G
8191	rs3670142	4	123224033	A	G	A	G	A	G
8192	rs6191300	4	123227617	A	A	A	A	A	G
8193	rs3671259	4	123272188	A	A	A	G	A	G
8194	rs6276600	4	123612958	A	A	A	A	A	A
8195	rs3704486	4	123958705	A	C	A	C	A	C
8196	rs4224765	4	124684143	G	G	G	G	G	G
8197	rs6333428	4	124743553	C	C	C	C	C	C
8198	rs3666537	4	124843162	A	A	T	A	T	A
8199	rs3667597	4	124843286	C	C	A	C	A	C
8200	rs3692521	4	125087597	A	G	G	A	G	G
8201	rs4224769	4	125232244	C	A	C	C	C	C
8202	rs6171443	4	125378697	A	A	A	A	A	A
8203	rs3023000	4	125681860	C	C	C	C	C	C
8204	rs4224772	4	125683315	A	A	A	A	A	A
8205	rs4224771	4	125683489	A	A	A	A	A	G
8206	mCV24231492	4	129874356	C	A	A	A	A	C
8207	rs3717809	4	126378807	A	G	G	A	G	A
8208	rs6360227	4	126486689	A	A	A	A	A	A
8209	rs3721916	4	126503138	G	A	A	A	A	G
8210	rs3711319	4	126893224	A	A	A	T	A	T
8211	rs4138996	4	127247890	A	G	G	G	G	G

8212	rs4224785	4	127281725	A	A	A	A	A	A
8213	rs3023007	4	127922145	A	A	A	C	A	A
8214	rs3023009	4	127942488	A	A	A	G	A	A
8215	rs4139270	4	127994032	G	G	G	A	G	G
8216	rs3708285	4	128000012	A	A	A	G	A	A
8217	rs6355837	4	128083691	C	C	C	A	C	C
8218	rs3661158	4	128300912	T	T	T	A	T	T
8219	rs3698956	4	128468831	A	G	G	G	G	NONE
8220	rs8272651	4	128536032	A	A	A	A	A	A
8221	rs8274322	4	128538100	G	A	A	A	A	A
8222	rs8236541	4	129086832	T	T	T	T	T	T
8223	rs8236544	4	128543858	A	A	A	A	A	A
8224	rs6370137	4	128885948	T	T	T	T	T	T
8225	rs3673061	4	129606752	G	A	A	A	A	A
8226	rs6206222	4	129915072	A	A	A	A	A	A
8227	rs3089399	4	130078205	A	A	A	A	A	A
8228	rs6218778	4	130258948	C	C	C	C	C	C
8229	rs3696551	4	130943710	G	A	A	A	A	G
8230	rs3664871	4	131307526	G	A	A	G	A	G
8231	rs6235031	4	131343591	G	A	A	G	A	G
8232	rs3721169	4	131381162	C	A	A	C	A	C
8233	rs4224808	4	131453665	G	C	C	C	G	G
8234	rs4224809	4	131453707	C	C	C	C	C	C
8235	rs6243362	4	131499407	G	G	G	G	G	G
8236	mCV24210995	4	135242958	G	A	A	G	A	A
8237	rs6297037	4	131734739	C	C	C	G	C	C
8238	rs3697583	4	131942653	G	G	G	A	G	G
8239	rs3671045	4	132166253	G	G	G	A	G	G
8240	rs3682399	4	132339612	G	G	G	A	G	G
8241	rs4224814	4	132458351	C	C	C	C	C	C
8242	rs6404906	4	132462369	C	C	C	G	C	C
8243	rs3680937	4	132707663	C	C	C	A	C	C
8244	rs6368842	4	133472901	A	A	A	A	A	A
8245	mCV22976835	4	134813801	A	G	G	G	G	G
8246	rs3663950	4	134187547	G	G	A	A	G	G
8247	rs6353992	4	134308567	C	C	G	G	G	G
8248	rs4224831	4	134902093	G	A	A	G	A	A
8249	rs6381419	4	135148164	G	G	G	G	G	G
8250	rs4224836	4	135347941	A	G	G	A	G	G
8251	rs4224854	4	135677363	A	A	A	A	A	A
8252	rs4224852	4	135677422	G	A	A	G	A	A
8253	rs4224841	4	135677675	A	A	A	A	A	A
8254	rs6378257	4	135933671	A	G	G	A	G	G
8255	rs3661463	4	136080850	A	G	G	A	G	G
8256	rs4224858	4	136115446	G	G	G	G	G	G
8257	rs3661970	4	136604410	G	A	A	G	A	A
8258	rs4224860	4	136661764	G	G	G	G	G	G
8259	rs4224864	4	136662000	G	A	A	G	A	A
8260	rs6350149	4	136763274	A	C	C	A	C	C
8261	rs3703479	4	136844302	G	A	A	G	A	A
8262	rs3090961	4	137197698	A	A	A	A	A	A
8263	rs6340721	4	137318349	A	A	G	A	G	G
8264	rs3685316	4	137349649	G	G	A	G	A	A
8265	rs4224870	4	137512969	A	A	G	A	G	G
8266	rs3686214	4	138010250	G	G	A	G	A	A
8267	rs3686283	4	138010276	G	G	A	G	A	A
8268	rs6323117	4	138056905	T	T	T	T	T	T
8269	rs6386918	4	138224144	A	A	G	A	G	G
8270	rs3718952	4	138506526	G	G	A	G	A	A
8271	mCV23620754	4	138704812	A	A	A	A	G	G
8272	mCV23723677	4	138931510	G	G	G	G	A	A
8273	rs3724911	4	138982190	T	T	T	T	A	A
8274	rs3657367	4	139122755	A	G	G	G	G	A
8275	rs4232380	4	139463518	A	A	A	A	A	A
8276	rs6382504	4	139523933	G	G	G	G	G	G
8277	rs3722135	4	139700222	G	C	C	C	G	G
8278	rs3023026	4	139818353	G	A	A	A	G	G
8279	rs3688566	4	140026440	G	A	G	A	A	A
8280	rs6215302	4	140110739	G	A	G	A	A	A
8281	rs3659226	4	140162829	A	A	G	A	A	A
8282	rs8260738	4	140282474	G	G	A	G	G	G
8283	rs6250787	4	140385565	G	A	G	A	G	G
8284	rs4224893	4	140559314	G	G	G	G	G	G
8285	rs4224894	4	140559352	G	G	C	G	G	G

8286	rs3695719	4	140800805	A	G	G	G	G	G
8287	rs3719891	4	140961583	A	A	G	A	G	G
8288	rs3720051	4	140961672	G	G	A	G	A	A
8289	rs3684126	4	141244229	A	A	T	A	T	T
8290	rs3718552	4	141332792	G	G	G	A	G	G
8291	rs3023025	4	141638718	T	A	A	A	T	T
8292	rs6316136	4	141826127	G	A	A	A	G	G
8293	mCV22757103	4	142788270	A	C	C	C	A	A
8294	mCV24105383	4	143338813	A	C	A	C	A	C
8295	rs4224919	4	143308550	A	G	A	G	A	G
8296	rs4136314	4	143380772	A	G	A	G	G	G
8297	rs6233441	4	143381201	G	G	G	G	G	G
8298	rs3694109	4	143654943	G	A	G	A	G	A
8299	rs6230717	4	145785696	G	C	G	C	G	C
8300	rs4224932	4	145795708	G	G	G	G	G	G
8301	rs4224929	4	145796031	G	G	G	G	G	G
8302	rs3720542	4	145888240	A	G	A	G	A	G
8303	rs3724210	4	145898312	G	C	G	C	G	C
8304	rs3693400	4	145932485	A	G	A	G	A	G
8305	rs3675987	4	146171058	G	G	G	A	G	A
8306	rs6306557	4	146280236	A	A	A	G	A	G
8307	rs3165478	4	146494437	G	G	G	A	A	A
8308	mCV22914328	4	144478532	G	G	G	G	G	G
8309	rs4137378	4	146556633	G	G	G	A	G	A
8310	rs3669806	4	146708911	A	A	A	G	A	G
8311	rs3715154	4	146726644	A	G	A	G	A	G
8312	rs3713685	4	147025784	G	G	G	A	G	A
8313	rs3702606	4	147272985	A	G	A	G	A	A
8314	rs3720325	4	147647868	A	G	A	A	A	G
8315	rs3697097	4	148283295	A	A	A	G	A	A
8316	rs6223564	4	148304705	C	C	C	A	C	C
8317	rs3695426	4	148339149	C	A	C	C	C	C
8318	rs4140148	4	148362291	C	C	C	G	C	C
8319	rs3725019	4	148702440	A	G	G	G	G	G
8320	rs4224962	4	148945757	A	A	A	A	A	A
8321	rs4224961	4	148946009	G	G	G	G	G	G
8322	rs3686555	4	148963391	A	G	A	G	A	A
8323	rs6358921	4	148971795	A	NONE	A	G	A	A
8324	rs3680006	4	149383989	G	A	G	A	G	G
8325	rs6268364	4	149902058	G	A	A	A	A	A
8326	rs4224986	4	150171120	G	G	G	G	G	G
8327	rs6256662	4	150187273	G	A	A	A	A	A
8328	rs3686348	4	150897581	A	G	G	G	G	G
8329	rs3667663	4	150923668	G	A	A	A	A	A
8330	rs3668413	4	150923815	G	A	A	A	A	A
8331	rs6378384	4	151446994	G	A	A	A	A	A
8332	rs6181146	4	151939989	G	G	G	A	G	G
8333	rs4139190	4	152060247	A	T	T	T	T	T
8334	rs3670828	4	152211118	A	A	A	T	A	A
8335	rs3693138	4	152591465	A	A	A	C	A	A
8336	rs6263119	4	152919587	C	C	C	A	C	C
8337	rs4224988	4	152959953	G	G	G	G	G	G
8338	rs3696703	4	153111615	A	A	A	G	A	G
8339	rs3091009	4	153709972	A	A	A	A	A	A
8340	rs3091010	4	153709974	A	A	A	A	A	A
8341	rs3720634	4	153793536	A	A	A	G	A	A
8342	rs3693087	4	154007691	G	G	G	A	G	G
8343	rs6279100	4	154069720	G	G	G	A	G	G
8344	mCV24977441	4	153708022	A	A	A	G	A	A
8345	mCV24977452	4	153708070	G	G	G	A	G	G
8346	mCV24977637	4	153710692	G	G	G	A	G	G
8347	mCV24977638	4	153710738	A	A	A	G	A	A
8348	mCV24977639	4	153710793	G	G	G	A	G	G
8349	rs3705659	5	134508741	C	C	C	A	C	C
8350	rs8259990	5	8863543	G	G	G	G	G	G
8351	rs6332539	5	81683053	G	A	G	A	G	G
8352	rs8268905	5	8864490	A	A	A	A	A	A
8353	rs4225123	5	28674211	A	A	A	A	A	A
8354	rs3688859	5	96948957	C	C	C	A	C	C
8355	rs8260004	5	8865410	A	A	A	A	A	A
8356	rs3656462	5	66391242	A	A	A	A	A	A
8357	rs4225290	5	74884361	G	G	A	A	A	G
8358	rs8280080	5	8901038	G	G	G	G	G	G
8359	rs6152903	5	6675515	A	A	A	T	A	A

8360	rs3666675	5	25543809	G	A	A	G	G	A
8361	rs3707823	5	15379563	A	G	G	G	G	G
8362	rs3719870	5	65332177	A	A	A	G	A	G
8363	rs3698071	5	9497540	A	G	G	G	G	G
8364	rs4173745	16	36388382	C	C	G	C	G	C
8365	rs3153753	5	74875328	G	G	G	A	G	G
8366	mCV23404305	5	5274596	A	A	A	G	A	A
8367	rs4225034	5	3589864	G	G	G	G	G	G
8368	rs6189262	5	3806733	G	G	G	G	G	G
8369	rs3674947	5	3941458	G	G	G	A	G	G
8370	rs3670745	5	4013495	G	G	G	C	G	G
8371	rs4225033	5	4088312	A	G	G	G	G	G
8372	rs3713790	5	4092754	C	G	G	G	G	G
8373	rs3666313	5	4812113	A	G	G	G	G	G
8374	rs6190354	5	4865632	C	A	A	A	A	A
8375	rs3709655	5	5044871	G	G	G	A	G	G
8376	rs6246086	5	5134008	T	T	T	A	T	T
8377	mCV24178939	5	8292817	A	G	G	G	G	G
8378	rs3667728	5	8374626	C	A	A	A	A	A
8379	rs6402980	5	5438798	C	C	C	G	C	C
8380	rs3714332	5	5438984	G	G	G	A	G	G
8381	rs4225021	5	5587957	A	G	G	G	G	A
8382	mCV22641745	5	6395635	A	NONE	C	C	C	C
8383	rs6354478	5	6246355	G	A	A	A	A	A
8384	rs3672328	5	7246940	A	A	A	A	A	A
8385	rs3671855	5	7246966	G	A	A	G	A	G
8386	rs6245801	5	7469880	A	G	G	G	G	A
8387	rs3666998	5	6963830	A	G	G	A	G	A
8388	rs3089100	5	6839681	G	G	G	G	G	G
8389	rs3709946	5	6799048	A	G	G	G	G	A
8390	rs3023033	5	7172256	T	A	A	T	A	T
8391	rs3718443	5	7881689	A	G	G	G	G	G
8392	mCV25347777	5	15986460	G	A	A	A	A	A
8393	rs6222248	5	7900641	G	A	A	A	A	A
8394	rs8266805	5	8813167	A	A	A	A	A	A
8395	rs8237256	5	8826911	A	A	A	A	A	A
8396	rs6353486	5	8852362	G	G	G	G	G	G
8397	rs8237291	5	8854148	G	G	G	G	G	G
8398	rs6410022	5	8917993	G	G	G	G	G	G
8399	rs8277484	5	8929252	A	A	A	A	A	A
8400	rs3692422	5	9101440	G	A	A	A	A	A
8401	rs3704546	5	10347575	G	G	G	G	G	G
8402	rs3676096	5	10677032	A	G	G	G	G	G
8403	rs6232180	5	10769121	T	T	T	T	T	T
8404	rs3659713	5	11081268	A	G	G	G	G	G
8405	rs3708118	5	11358555	A	G	G	A	A	A
8406	rs3714258	5	11417181	A	A	A	G	A	A
8407	rs6285349	5	11441518	A	A	A	A	A	A
8408	rs6321461	5	11718088	G	G	G	G	G	G
8409	mCV22832219	5	15698089	A	A	A	A	G	A
8410	rs3660027	5	12010866	A	G	G	G	G	G
8411	rs3701181	5	13194121	C	A	A	A	A	A
8412	rs6404215	5	13058225	A	A	A	C	A	A
8413	mCV23262554	5	17124962	A	T	T	T	T	T
8414	rs3687916	5	13389454	A	A	A	G	A	A
8415	rs6353097	5	13875606	G	A	A	A	G	G
8416	rs3676443	5	13905415	G	C	C	C	G	G
8417	rs3701972	5	14242321	G	A	A	A	A	A
8418	rs6356492	5	14453277	A	A	A	A	A	A
8419	rs6248947	5	15202306	T	T	T	T	T	T
8420	rs6214590	5	15540280	A	G	G	G	G	G
8421	rs6297493	5	15890773	G	G	G	G	G	G
8422	rs3684715	5	16423235	A	G	G	G	G	G
8423	rs3681166	5	16589143	A	G	G	G	G	G
8424	rs4225047	5	16627946	G	G	G	G	G	G
8425	rs4225046	5	16627972	A	A	A	A	A	A
8426	rs3709325	5	16691950	A	G	G	G	G	G
8427	rs6178561	5	16743186	C	C	C	C	C	C
8428	rs4225050	5	17948714	C	C	C	C	C	C
8429	rs4225063	5	17948328	G	G	G	G	G	G
8430	rs6207468	5	17786361	A	A	A	A	A	A
8431	rs3671075	5	18495485	A	G	G	G	G	G
8432	mCV22781030	5	22537262	A	G	G	G	A	A
8433	rs6408115	5	52449828	A	A	A	A	A	A

8434	rs3697291	5	18676327	A	G	G	G	G	G
8435	rs4225066	5	19344217	G	G	G	G	G	G
8436	rs6194519	5	19193683	A	A	A	A	A	A
8437	rs3664312	5	19185678	A	G	G	G	G	G
8438	rs4225084	5	20041999	G	G	G	G	G	G
8439	rs6244687	5	20178223	G	G	G	C	C	C
8440	rs3665481	5	20202897	G	G	G	A	A	A
8441	rs3676378	5	20385265	T	A	A	A	A	A
8442	rs3671575	5	20646278	A	A	A	G	A	G
8443	rs3690874	5	20653698	A	A	A	G	A	G
8444	rs6294047	5	20815246	C	C	C	C	C	C
8445	rs6255331	5	21311602	C	C	C	C	C	C
8446	rs3724053	5	21453217	A	G	G	G	A	A
8447	rs3720911	5	21951785	A	G	G	G	A	A
8448	rs3654251	5	22375271	A	G	G	G	A	A
8449	rs3090417	5	22630311	C	A	A	A	C	C
8450	rs3089039	5	22756981	A	A	A	A	A	A
8451	rs4225096	5	22807243	A	A	A	A	C	A
8452	rs6260804	5	22901492	T	T	T	T	A	T
8453	rs3023039	5	23287553	A	A	A	A	G	A
8454	rs3706626	5	23524916	A	G	G	A	G	A
8455	rs6347403	5	23607213	T	T	T	A	A	A
8456	rs4225108	5	23662405	G	G	G	G	G	G
8457	rs4225109	5	23662465	G	A	A	G	G	G
8458	rs3676101	5	24582694	A	G	G	G	G	G
8459	rs3713077	5	24619890	T	A	A	T	T	A
8460	rs6275634	5	24727380	A	C	C	C	C	C
8461	rs3705209	5	24811094	A	G	G	A	A	G
8462	rs6309009	5	25210041	G	C	C	C	C	C
8463	rs3666952	5	25412948	A	G	G	G	G	G
8464	rs3699500	5	25485429	A	G	G	A	A	G
8465	rs3088839	5	26109156	G	G	G	G	G	G
8466	rs4225113	5	26232631	G	G	G	G	G	G
8467	rs4225111	5	26232591	A	A	A	A	A	A
8468	rs3715679	5	26228802	A	A	A	C	C	C
8469	rs3668113	5	26583853	C	C	C	G	G	C
8470	rs6159963	5	26815473	G	G	G	A	A	G
8471	rs3671062	5	26763624	G	A	A	G	G	A
8472	rs3670423	5	26763519	A	G	G	A	A	G
8473	rs3700261	5	26758056	C	A	A	C	C	A
8474	rs3718551	5	26873766	T	A	A	T	T	A
8475	rs3680434	5	27162034	C	A	A	C	C	A
8476	rs4225121	5	28525894	G	G	G	G	G	G
8477	rs4225120	5	28525935	G	G	G	G	G	G
8478	rs8259850	5	28414545	A	A	A	A	A	A
8479	rs3706017	5	28286942	A	T	T	T	T	T
8480	rs6281932	5	28855668	G	A	A	A	A	A
8481	rs3700507	5	29226425	C	A	A	A	A	A
8482	rs3654169	5	29335055	G	G	G	A	A	G
8483	rs3697620	5	29406808	A	A	A	C	C	A
8484	rs4225138	5	29564319	C	C	C	C	C	C
8485	rs6254142	5	29669430	G	G	G	A	A	G
8486	rs4225141	5	29978018	A	A	A	A	A	A
8487	rs6325044	5	30099239	G	NONE	A	A	A	A
8488	rs3682333	5	30357756	G	G	G	A	A	G
8489	rs3718492	5	30465115	A	A	A	G	G	G
8490	rs3695034	5	30512882	A	A	A	G	G	G
8491	rs3090139	5	30537667	A	A	A	A	A	G
8492	rs6234655	5	30730773	G	G	G	A	A	G
8493	rs3679280	5	30917512	G	G	G	A	A	G
8494	rs3700706	5	31071103	C	C	C	A	A	C
8495	rs3705746	5	31726667	G	G	A	A	A	A
8496	rs6196732	5	31710308	C	C	A	A	A	A
8497	rs3658401	5	31765822	A	A	C	A	A	C
8498	rs4225164	5	32343195	G	G	G	G	G	G
8499	rs3726925	5	32525291	A	G	G	A	G	A
8500	rs6152183	5	32534751	C	A	A	C	A	C
8501	rs6237647	5	33082673	A	G	G	A	A	A
8502	rs3090192	5	33536864	A	A	A	A	A	A
8503	rs6408534	5	33877338	A	A	A	A	G	G
8504	rs3673363	5	33963604	C	G	G	C	C	C
8505	rs6172801	5	33989584	C	G	G	C	C	C
8506	mCV24314853	5	38628101	T	A	A	A	T	T
8507	rs6193862	5	34667066	A	G	G	A	G	G

8508	rs4225177	5	34917291	T	T	T	T	T	T
8509	rs4225178	5	34917333	G	A	A	G	A	A
8510	rs3698586	5	35400359	G	A	A	G	G	G
8511	rs3089816	5	35450379	G	G	G	G	G	G
8512	rs6341620	5	35567326	A	A	A	G	G	G
8513	rs6197573	5	35855759	G	A	A	G	G	G
8514	rs3723722	5	35856605	A	G	G	A	A	A
8515	rs3656280	5	36046250	A	G	G	A	A	A
8516	rs3692554	5	36142398	A	G	G	A	A	A
8517	rs3680406	5	36687347	A	G	G	A	A	A
8518	rs3667998	5	36690479	A	C	C	A	A	A
8519	rs6364639	5	36726060	G	A	A	G	G	G
8520	rs4225190	5	37234479	A	A	A	A	A	A
8521	rs3702623	5	37351343	C	A	A	C	C	C
8522	rs3685832	5	38023009	C	A	A	A	C	C
8523	rs6377392	5	37980576	A	G	G	A	A	A
8524	rs3716546	5	38054339	G	A	A	G	G	G
8525	rs3703261	5	38200794	G	G	G	A	G	G
8526	rs6367025	5	38351511	G	A	A	A	G	G
8527	rs3673482	5	38503436	A	A	A	G	A	A
8528	mCV22331571	5	43271643	A	A	A	C	A	A
8529	rs3705677	5	38986924	G	G	G	A	G	G
8530	rs6236042	5	39054617	G	A	A	A	A	A
8531	mCV25308685	5	43719553	A	A	A	A	A	A
8532	rs3682652	5	40017418	A	G	G	G	G	G
8533	rs3656789	5	40014073	G	A	A	A	A	A
8534	rs6294270	5	39879527	C	A	A	A	C	C
8535	rs3089654	5	39823740	G	G	G	G	G	G
8536	rs3662682	5	40160856	A	A	A	A	C	C
8537	rs6345266	5	40569099	A	A	A	A	A	A
8538	rs3716195	5	40684934	C	C	C	C	A	A
8539	rs3088905	5	40757938	C	C	C	C	C	C
8540	rs6361711	5	41263965	T	T	T	T	A	A
8541	rs6255687	5	42117069	T	T	T	T	A	A
8542	rs3660047	5	42530143	C	C	C	C	G	G
8543	rs6215373	5	42914749	A	A	A	G	A	A
8544	rs6401836	5	42950240	G	G	G	C	G	G
8545	rs3669254	5	43347335	A	A	A	G	A	A
8546	rs3689331	5	43368208	A	A	A	G	A	A
8547	rs3656439	5	43476863	T	T	T	A	T	T
8548	rs8272268	5	44113071	G	G	G	G	G	G
8549	rs8272540	5	44126846	C	C	C	C	C	C
8550	rs8272620	5	44129521	G	G	G	G	G	G
8551	rs6265196	5	44018930	G	G	G	G	G	G
8552	mCV24168740	5	48413252	G	A	A	G	A	A
8553	rs3663092	5	43668813	C	C	C	A	C	C
8554	rs3694939	5	44297911	G	A	A	G	A	A
8555	rs6315432	5	44855350	G	A	A	G	A	A
8556	rs6325300	5	45573305	T	A	A	T	A	A
8557	rs3722515	5	46225155	G	A	A	G	A	A
8558	rs6352069	5	46310157	A	G	G	A	G	G
8559	rs4140247	5	46509743	A	G	G	A	G	G
8560	rs3672859	5	46559630	A	G	G	A	G	G
8561	rs6192958	5	47975043	A	G	G	A	G	A
8562	rs6370750	5	48114559	G	G	G	G	G	A
8563	rs6388130	5	48154288	A	A	A	A	A	A
8564	rs4225202	5	48285534	A	A	A	A	A	A
8565	rs3681071	5	48461082	A	G	G	A	G	G
8566	rs3692841	5	48676800	A	G	G	A	G	A
8567	mCV23125912	5	53853311	G	A	A	G	A	A
8568	rs3023045	5	49189073	A	C	C	C	C	A
8569	rs3714001	5	49310150	G	C	C	C	C	G
8570	rs3685952	5	49643969	C	G	G	G	G	G
8571	rs6154859	5	49665481	A	G	G	G	G	G
8572	rs3687137	5	50119081	G	A	A	A	A	A
8573	rs3655238	5	50443296	A	G	G	G	G	G
8574	rs6237983	5	50559438	A	G	G	G	G	G
8575	rs4225204	5	50999407	G	A	A	A	A	A
8576	rs6326139	5	51003702	G	A	A	A	A	A
8577	rs3695532	5	51017093	A	A	A	A	A	A
8578	rs3660964	5	51028268	A	G	G	G	G	G
8579	rs4221979	5	51496599	G	G	G	G	G	G
8580	rs6248036	5	51438288	A	A	A	A	C	A
8581	rs4225221	5	51736500	A	A	A	A	A	A

8582	rs3089311	5	52145097	A	A	A	A	A	A
8583	rs3664008	5	52283097	A	G	G	A	G	G
8584	rs4225223	5	52283651	A	A	A	A	A	A
8585	rs6242729	5	52318577	G	A	A	G	A	A
8586	rs3704283	5	53039874	G	A	A	G	A	A
8587	rs6237838	5	53462569	G	C	C	G	C	C
8588	mCV22594101	5	58523211	A	A	A	A	G	A
8589	rs3706294	5	53362435	C	A	A	C	A	A
8590	rs3690218	5	53362108	A	C	C	A	C	C
8591	rs3688942	5	53361927	G	G	A	G	A	A
8592	rs3696973	5	53786865	A	A	A	A	A	A
8593	mCV24707394	5	59198398	G	C	C	G	C	C
8594	rs6209928	5	54178978	G	A	A	G	A	A
8595	rs4229959	5	54255046	A	A	A	A	A	A
8596	rs3713492	5	54302408	G	A	A	G	A	A
8597	mCV23375358	5	59597927	T	A	A	T	A	A
8598	rs3654245	5	55002903	A	C	C	A	C	C
8599	rs3654975	5	55003024	A	G	G	A	G	G
8600	rs6403771	5	55119859	G	G	G	G	G	G
8601	rs3678683	5	55489937	A	T	T	A	T	T
8602	rs6200992	5	55391110	A	G	G	A	G	G
8603	rs6339023	5	56825527	A	G	G	A	G	G
8604	rs3699784	5	56922657	A	C	C	A	C	C
8605	rs6154545	5	57020216	A	G	G	A	G	G
8606	mCV23386455	5	62818443	A	G	G	A	G	G
8607	rs6210120	5	57763412	A	A	A	A	A	A
8608	rs3713195	5	57883360	C	C	A	C	A	C
8609	rs6406122	5	58657704	A	G	G	A	G	G
8610	rs3090667	5	58891807	G	A	A	G	G	A
8611	rs3704920	5	59103073	C	A	A	C	A	A
8612	mCV27558149	5	64412079	C	G	G	C	G	G
8613	rs6338685	5	59847783	A	A	A	A	A	A
8614	rs3724321	5	60369206	A	C	C	A	C	C
8615	rs3679606	5	61255160	A	A	G	A	G	A
8616	rs3692826	5	61531469	G	A	A	G	A	A
8617	rs3657916	5	61800901	A	A	G	A	G	A
8618	rs3707026	5	62014298	G	G	G	G	G	G
8619	rs3088862	5	62317643	A	A	A	A	A	A
8620	rs3703900	5	62465563	A	C	C	A	C	C
8621	rs6281588	5	62586928	A	G	G	A	G	G
8622	rs3715307	5	62615736	A	C	C	A	C	C
8623	rs3711950	5	62648600	C	A	A	C	A	A
8624	rs3090421	5	62726076	A	A	A	A	A	A
8625	rs4225234	5	62979175	A	A	A	A	A	A
8626	rs6152354	5	62985378	A	G	G	A	G	G
8627	rs3718814	5	63308017	T	A	A	T	A	A
8628	rs6265085	5	63380661	A	A	G	A	G	A
8629	rs6158897	5	64230914	G	G	G	G	G	G
8630	rs6339313	5	64343993	A	G	G	A	G	A
8631	rs3656989	5	64815360	G	G	G	A	G	G
8632	rs6267669	5	65103231	G	G	G	A	G	G
8633	rs3711269	5	65501814	G	G	G	A	G	A
8634	mCV24275411	5	70936994	C	C	A	C	A	C
8635	mCV24275412	5	70939275	G	G	G	G	NONE	G
8636	mCV24275413	5	70939277	G	G	G	G	G	G
8637	mCV24275414	5	70942699	C	C	A	C	A	C
8638	mCV24275415	5	70954573	G	G	A	G	A	G
8639	mCV24275418	5	70969823	C	C	C	C	C	C
8640	mCV24275419	5	70977453	A	A	G	A	G	A
8641	mCV24275424	5	70983954	A	A	G	A	G	A
8642	mCV24275425	5	70984290	G	G	G	G	G	G
8643	mCV24275430	5	71010940	G	G	G	G	G	G
8644	mCV24275431	5	71011056	A	A	C	A	C	A
8645	mCV24275432	5	71015127	G	G	A	G	A	G
8646	mCV24275438	5	71063173	G	G	A	G	A	G
8647	mCV24275706	5	71063271	A	A	G	A	G	A
8648	mCV24275707	5	71063475	A	A	G	A	G	A
8649	mCV24275708	5	71063647	A	A	C	A	C	A
8650	mCV24275718	5	71063688	A	A	T	A	T	A
8651	mCV24275719	5	71066245	C	C	C	C	C	C
8652	mCV24275720	5	71076132	G	G	G	G	G	G
8653	mCV24275730	5	71076134	C	C	C	C	C	C
8654	mCV24275742	5	66697965	G	G	A	G	A	G
8655	rs4225260	5	65894761	C	C	C	C	C	C

8656	mCV24275743	5	71081942	A	A	C	A	C	A
8657	mCV24275744	5	71081979	G	G	A	G	A	G
8658	mCV24275755	5	71082277	C	C	A	C	A	C
8659	mCV24275767	5	71083067	A	A	G	A	G	A
8660	mCV24275768	5	71083841	C	C	A	C	A	C
8661	mCV24275778	5	71085211	A	A	C	A	C	A
8662	mCV24275779	5	71086650	G	G	A	G	A	G
8663	mCV24275780	5	71086733	T	T	A	T	A	T
8664	mCV24276050	5	71086745	A	A	T	A	T	A
8665	mCV24276051	5	71088221	A	A	G	A	G	A
8666	mCV24276062	5	71089699	G	G	A	G	A	G
8667	mCV24276063	5	71090703	G	G	G	G	G	G
8668	mCV24276064	5	71091474	A	A	C	A	C	A
8669	mCV24276098	5	71099218	T	T	A	T	A	T
8670	mCV24276099	5	71099511	C	C	A	C	A	C
8671	mCV24276111	5	71099839	A	A	G	A	G	A
8672	mCV24276112	5	71100148	C	C	A	C	A	C
8673	mCV24276122	5	71100307	A	A	G	A	G	A
8674	mCV24276123	5	71100410	A	A	G	A	G	A
8675	mCV24276124	5	71100526	A	A	C	A	C	A
8676	mCV24276133	5	71100556	G	G	A	G	A	G
8677	mCV24276134	5	66718444	C	C	A	C	A	C
8678	mCV24276135	5	71102920	A	A	T	A	T	A
8679	mCV24276418	5	71103133	G	G	A	G	A	G
8680	mCV24276419	5	71103385	C	C	A	C	A	C
8681	mCV24276439	5	71103433	G	G	A	G	A	G
8682	mCV24276440	5	71103709	A	A	G	A	G	A
8683	mCV24276450	5	71104334	G	G	A	G	A	G
8684	mCV24276462	5	71105453	A	A	G	A	G	A
8685	mCV24276464	5	71105556	A	A	G	A	G	A
8686	mCV24276479	5	71105845	A	A	G	A	G	A
8687	mCV24276480	5	71105878	G	G	A	G	A	G
8688	mCV24276481	5	71106944	C	C	G	C	G	C
8689	mCV24276748	5	71107580	G	G	A	G	A	G
8690	mCV24276749	5	71110108	A	A	G	A	G	A
8691	mCV24276756	5	71110222	A	A	G	A	G	A
8692	mCV24276757	5	71110565	C	C	A	C	A	C
8693	mCV24276763	5	71112258	A	A	G	A	G	A
8694	mCV24276764	5	71112285	G	G	A	G	A	G
8695	mCV24276768	5	71112434	G	G	A	G	A	G
8696	mCV24276769	5	71112574	G	G	A	G	A	G
8697	mCV24276770	5	71112672	A	A	T	A	T	A
8698	mCV24276775	5	71113182	A	A	G	A	G	A
8699	mCV24276776	5	71113346	G	G	A	G	A	G
8700	mCV24276780	5	71113472	A	A	G	A	G	A
8701	mCV24276781	5	71113531	G	G	A	G	A	G
8702	mCV24276782	5	71114341	G	G	A	G	A	G
8703	mCV24276789	5	71114359	A	A	G	A	G	A
8704	mCV24276790	5	71115218	A	A	G	A	G	A
8705	mCV24276791	5	71117849	A	A	G	A	G	A
8706	mCV24276798	5	71123568	T	T	A	T	A	T
8707	mCV24276799	5	71123842	A	A	G	A	G	A
8708	mCV24276800	5	71125182	G	G	A	G	A	G
8709	mCV24276810	5	71125190	G	G	A	G	A	G
8710	mCV24276811	5	66745356	C	C	A	C	A	C
8711	mCV24276812	5	71125956	G	G	A	G	A	G
8712	mCV24276821	5	71126094	G	G	A	G	A	G
8713	mCV24276822	5	71129034	A	A	G	A	G	A
8714	mCV22918434	5	71130372	G	G	A	G	A	G
8715	mCV22918435	5	71130569	A	A	C	A	C	A
8716	mCV22918441	5	71130668	A	A	G	A	G	A
8717	mCV22918442	5	71130694	G	G	A	G	A	G
8718	mCV22918443	5	71130827	A	A	G	A	G	A
8719	mCV22918450	5	71130887	A	A	G	A	G	A
8720	mCV22918451	5	71131375	G	G	C	G	C	G
8721	mCV22918452	5	71131386	G	G	A	G	A	G
8722	mCV22918459	5	71131405	A	A	G	A	G	A
8723	mCV22918460	5	71136568	C	C	A	C	A	C
8724	mCV22918468	5	71136740	G	G	A	G	A	G
8725	mCV22918469	5	71136913	G	G	A	G	A	G
8726	mCV22918470	5	71137145	A	A	G	A	G	A
8727	mCV22918651	5	71137168	C	C	G	C	G	C
8728	mCV22918652	5	71137236	A	A	C	A	C	A
8729	mCV22918660	5	71137572	A	A	G	A	G	A

8730	mCV22918661	5	71137754	A	A	G	A	G	A
8731	mCV22918671	5	71138548	G	G	A	G	A	G
8732	rs3684754	5	65964490	G	G	G	A	G	G
8733	mCV22996091	5	66852497	C	C	A	C	A	C
8734	rs6170457	5	66180535	G	G	G	G	G	G
8735	rs3659098	5	66181856	G	G	G	A	G	G
8736	rs3662462	5	66209208	A	A	A	G	A	A
8737	rs6227779	5	66701098	G	G	G	G	G	G
8738	rs3691938	5	66879354	G	G	G	G	G	G
8739	rs6411304	5	67249846	A	A	A	A	A	A
8740	rs3088773	5	67602502	C	C	C	C	C	C
8741	rs3090910	5	68012404	A	A	A	A	A	A
8742	rs6242879	5	68506442	C	A	A	A	A	A
8743	rs6168670	5	68660765	G	A	A	A	A	A
8744	rs3675400	5	68661347	G	A	A	A	A	A
8745	rs3681370	5	68768688	A	G	G	G	G	G
8746	rs6158490	5	68943118	C	C	C	C	C	C
8747	rs3695107	5	69049778	A	G	G	G	G	G
8748	rs3722869	5	68998655	A	G	G	G	G	G
8749	rs3088650	5	69511947	A	A	A	A	A	A
8750	mCV24307017	5	74853431	A	A	G	A	G	A
8751	rs3678516	5	69652238	A	A	A	A	A	A
8752	rs6397126	5	70143718	A	A	A	A	A	A
8753	rs3090696	5	70605484	A	A	A	A	A	A
8754	rs6308779	5	70267762	G	G	G	G	G	G
8755	rs3710735	5	71369230	C	C	G	C	G	C
8756	rs6259856	5	71398209	A	A	G	A	G	A
8757	rs6409508	5	71768054	G	G	G	A	G	G
8758	mCV23012415	5	77071630	A	C	A	C	A	A
8759	rs3669361	5	71592665	A	A	G	A	G	A
8760	rs3689647	5	72316977	A	A	G	G	G	A
8761	rs4138743	5	73109674	G	G	A	G	A	G
8762	rs3670420	5	73116196	T	T	A	T	A	T
8763	rs3090429	5	71835734	G	G	G	G	G	G
8764	rs3708666	5	71983570	G	A	G	G	G	A
8765	mCV22834148	5	77731523	G	A	G	A	G	G
8766	rs4225267	5	71950258	A	A	C	C	C	A
8767	rs6161105	5	73093296	G	A	G	A	G	A
8768	rs6354067	5	73116738	A	G	A	G	A	G
8769	rs3672514	5	73156460	G	A	G	A	G	A
8770	rs3700540	5	73177555	A	C	A	C	A	C
8771	rs3657238	5	73302032	NONE	G	A	A	A	G
8772	rs3684542	5	74138933	A	G	G	G	G	G
8773	rs3707537	5	74177041	C	A	A	A	A	A
8774	rs3722245	5	74543251	A	A	G	A	G	A
8775	rs3665183	5	74332420	A	C	C	C	C	C
8776	rs6221589	5	74399064	G	G	A	A	A	G
8777	rs4225284	5	74655004	G	G	G	G	G	G
8778	rs4225286	5	74655122	C	C	C	C	C	C
8779	rs4225287	5	74655225	C	C	C	C	C	C
8780	mCV22996021	5	75065363	T	A	A	T	A	A
8781	rs6257272	5	75618342	A	G	G	A	G	G
8782	rs4225300	5	75645166	A	G	G	A	G	G
8783	rs3090786	5	75677192	A	A	A	A	A	A
8784	rs3720626	5	75760098	G	A	A	G	A	A
8785	rs4225316	5	76018660	T	T	T	T	T	T
8786	rs4225315	5	76018697	A	A	A	A	A	A
8787	rs6407418	5	76207606	G	G	A	A	A	A
8788	rs6309185	5	76387346	G	G	G	G	G	G
8789	rs6252217	5	76773822	A	A	A	A	A	A
8790	rs3662055	5	77161759	A	A	T	A	T	T
8791	rs3713970	5	77194549	A	A	C	A	C	C
8792	rs3678094	5	77385778	A	C	C	C	C	C
8793	rs4138803	5	77522470	A	A	T	A	T	T
8794	rs3090419	5	77538720	A	A	A	A	A	A
8795	rs3704367	5	77550939	A	G	G	G	G	G
8796	mCV23424074	5	84156336	A	C	C	C	C	C
8797	rs3658895	5	78003333	A	G	A	G	A	A
8798	rs3714275	5	78425503	A	C	C	C	C	C
8799	rs6300728	5	78452816	T	A	T	A	T	T
8800	rs3658150	5	78563019	A	A	G	A	G	G
8801	rs2020550	5	78578727	C	C	A	C	A	A
8802	rs3722733	5	78641871	A	A	G	A	G	G
8803	mCV23427890	5	85495374	C	G	G	G	G	G

8804	rs6319876	5	79250625	A	G	G	G	G	G
8805	rs3708749	5	79591322	G	A	A	A	A	A
8806	rs3658818	5	80314941	G	A	A	A	A	A
8807	rs6409842	5	80552594	G	A	A	A	A	A
8808	rs6388786	5	81124536	G	G	G	G	G	G
8809	rs3721607	5	81282125	G	A	G	A	G	G
8810	rs3685079	5	81316994	A	C	A	C	A	C
8811	mCV22572591	5	102014263	G	A	A	G	A	A
8812	rs3667334	5	81819770	C	A	C	A	C	A
8813	rs3662368	5	81766675	A	G	A	G	A	A
8814	rs6264120	5	81778708	A	G	A	G	A	A
8815	rs3725100	5	81877043	G	A	G	A	G	G
8816	rs6400622	5	82823777	A	T	A	T	A	T
8817	rs6154126	5	82824119	A	G	A	G	A	G
8818	rs3659819	5	82846170	G	A	G	A	G	A
8819	rs3678577	5	83411956	G	A	G	A	G	A
8820	rs3699967	5	83697096	C	A	A	A	A	A
8821	rs3719592	5	84647838	A	G	A	G	A	G
8822	rs3670431	5	84772632	T	A	T	A	T	A
8823	rs3090163	5	84772845	T	T	T	T	T	T
8824	rs6301814	5	84821564	T	A	T	A	T	A
8825	rs3090530	5	84974690	A	A	A	A	A	A
8826	rs6318943	5	85121741	A	A	G	A	G	A
8827	rs3688741	5	85151024	A	A	G	A	G	A
8828	rs3657487	5	86378495	G	A	A	A	A	A
8829	rs8265626	5	86360584	A	A	A	A	A	A
8830	rs8274304	5	86351947	A	A	A	A	A	A
8831	rs8274297	5	86348742	A	A	A	A	A	A
8832	rs8259908	5	86488011	A	A	A	A	A	A
8833	rs3089910	5	86612686	A	A	A	A	A	A
8834	rs6304491	5	86912970	C	C	C	C	C	C
8835	rs3687494	5	86893433	T	A	T	A	T	A
8836	rs3669458	5	77109755	C	A	C	A	C	C
8837	rs6343240	5	87587201	A	A	A	A	A	A
8838	rs3656354	5	87803418	A	A	T	A	T	A
8839	rs3701843	5	87955097	G	A	G	A	G	A
8840	rs3685452	5	87980373	G	A	G	A	G	A
8841	rs3724733	5	88022166	C	A	C	A	C	A
8842	rs3089014	5	88236895	A	A	A	A	A	A
8843	rs6404893	5	88405441	A	A	A	A	A	A
8844	rs3673049	5	88498569	C	G	C	G	C	C
8845	mCV23876305	5	93159592	G	A	A	A	A	A
8846	mCV25016380	5	93385973	A	G	G	G	G	G
8847	rs4225366	5	89038627	G	G	G	G	G	G
8848	rs4225367	5	89038485	A	A	A	A	A	A
8849	rs4225368	5	89306578	A	A	G	A	G	G
8850	rs3689962	5	89358945	C	G	G	G	G	G
8851	rs3716445	5	89461092	A	T	A	T	A	A
8852	rs3678132	5	89876185	G	G	A	G	A	A
8853	rs3723202	5	89956609	G	A	G	A	G	G
8854	rs6306077	5	90114627	G	G	G	G	G	G
8855	rs3090680	5	90305723	C	C	C	C	C	C
8856	rs6227466	5	91117068	G	A	G	A	G	G
8857	rs3723011	5	91459891	A	G	G	G	G	G
8858	rs3680521	5	91663683	C	C	A	C	A	A
8859	rs6350541	5	92110414	G	G	G	G	G	G
8860	mCV24608431	5	92470267	C	A	C	A	C	C
8861	rs4225377	5	91790272	G	G	G	G	G	G
8862	rs6163295	5	91982403	A	A	A	A	A	A
8863	rs3695616	5	93341555	G	A	A	A	A	A
8864	rs6348455	5	93526584	A	A	A	A	A	A
8865	rs3090670	5	93866531	G	G	G	G	G	G
8866	rs6249855	5	94164595	G	G	G	A	G	G
8867	rs6216254	5	94228665	C	C	C	C	C	C
8868	rs3697719	5	94363359	G	G	G	A	G	G
8869	rs4225379	5	94386352	T	T	T	T	T	T
8870	rs4225381	5	94699228	G	G	G	G	G	G
8871	rs4225380	5	94699448	G	G	G	A	G	G
8872	rs3707832	5	94773717	G	G	G	A	G	G
8873	rs3706737	5	94827314	T	T	T	A	T	T
8874	rs3699266	5	94857761	A	C	A	C	A	A
8875	rs6344187	5	95124209	A	A	A	A	A	A
8876	rs3712722	5	95127691	G	G	G	A	G	G
8877	rs3661241	5	95154383	A	T	A	T	A	A

8878	rs3696322	5	95831320	A	A	A	T	A	A
8879	rs3705458	5	96618852	C	C	C	G	C	C
8880	rs3683611	5	97536219	G	A	A	G	A	A
8881	rs3726313	5	97883499	A	A	A	C	A	A
8882	mCV24425645	5	102803377	A	G	G	A	G	G
8883	rs6188215	5	97779115	G	G	G	G	G	G
8884	rs3088620	5	97788599	G	G	G	G	G	G
8885	rs4221981	5	98143573	G	G	G	G	G	G
8886	rs3090420	5	98397874	A	A	A	A	A	A
8887	rs3721248	5	98703608	G	G	G	A	G	G
8888	rs6384391	5	98914657	G	G	G	G	G	G
8889	rs3714102	5	99238408	A	C	C	C	C	C
8890	rs6327271	5	99696517	A	A	A	A	A	A
8891	rs3720559	5	100374400	C	G	C	G	C	G
8892	mCV24416913	5	105506767	G	A	G	G	A	G
8893	rs4225383	5	100960120	A	A	A	A	A	A
8894	rs3694887	5	101097424	G	A	G	A	G	G
8895	rs6191112	5	101315864	A	A	C	A	A	C
8896	rs3689574	5	101327935	G	G	A	G	G	A
8897	rs6412966	5	101621477	A	A	A	A	A	A
8898	rs3090525	5	103450903	A	A	A	A	G	A
8899	mCV24413438	5	106826186	A	A	G	G	A	G
8900	rs8256226	5	101806000	A	A	A	A	A	A
8901	rs8256230	5	101807634	A	A	G	A	A	G
8902	rs8256232	5	101808182	G	G	A	G	G	A
8903	rs3663200	5	101872969	G	A	A	G	A	A
8904	rs6218435	5	102359523	G	A	A	G	A	A
8905	rs3722596	5	102581717	C	C	A	C	C	A
8906	rs6191447	5	102883647	C	C	A	C	C	A
8907	rs4225386	5	103004322	T	T	A	T	T	A
8908	rs3654076	5	103489732	G	G	A	A	A	A
8909	rs6350578	5	103696203	C	C	A	A	A	A
8910	rs3680366	5	104206906	G	A	A	A	G	A
8911	rs3677428	5	104353483	A	G	G	A	G	G
8912	rs6236826	5	104562823	T	T	T	T	T	T
8913	rs6202283	5	104987392	G	G	G	G	G	G
8914	rs4225395	5	105083944	G	G	G	G	G	G
8915	rs4225398	5	105084101	A	A	A	A	G	A
8916	rs3663502	5	105124196	C	A	C	A	C	C
8917	rs3669104	5	105418682	A	G	A	G	A	A
8918	rs3726547	5	105832285	G	A	G	A	A	G
8919	rs3682429	5	106078273	G	G	A	G	G	A
8920	rs3665124	5	107073938	A	A	A	G	G	A
8921	rs3725274	5	107384723	G	G	G	A	G	G
8922	rs4225404	5	107987221	A	A	A	A	A	A
8923	rs4225405	5	107987130	A	A	A	A	A	A
8924	rs3708939	5	107911845	G	A	A	A	A	A
8925	rs3677162	5	107943458	A	A	A	C	A	A
8926	rs6314134	5	108241775	C	C	C	C	C	C
8927	rs3710018	5	108309646	G	G	A	A	G	G
8928	rs3725746	5	108437594	A	G	G	A	G	G
8929	rs3653831	5	108437901	A	G	G	A	G	G
8930	mCV23798232	5	114103101	A	A	A	A	C	A
8931	rs3663213	5	109378856	G	G	A	G	G	A
8932	rs6272142	5	109393681	A	A	A	A	A	A
8933	rs6299102	5	109512907	G	G	G	G	G	G
8934	rs3655095	5	109677144	A	G	G	G	G	G
8935	rs4225423	5	109771038	G	G	G	G	G	G
8936	rs4225425	5	109771341	A	A	A	A	A	A
8937	rs3688590	5	110056163	A	G	A	G	G	A
8938	mCV22554962	5	115013560	G	G	A	A	G	A
8939	rs3658755	5	110117726	G	G	A	G	G	G
8940	rs3698363	5	110305748	A	A	A	G	A	A
8941	rs3657810	5	110760303	G	G	G	A	G	G
8942	rs3672354	5	111189170	G	G	A	G	G	G
8943	rs4225426	5	111134034	A	A	A	A	A	A
8944	rs6176561	5	111373825	G	G	G	G	G	G
8945	rs3719351	5	111382152	G	A	G	A	A	G
8946	rs6160086	5	111402241	A	A	A	A	A	A
8947	rs4225432	5	111872548	C	C	C	C	C	C
8948	mCV23397413	5	116774498	A	A	G	G	A	G
8949	mCV25130934	5	117090128	C	C	A	A	C	A
8950	rs6343470	5	112128132	A	A	A	A	A	A
8951	rs4225446	5	112360279	A	A	A	A	A	A

8952	rs4225448	5	112360414	A	A	A	A	A	A
8953	rs3090568	5	112360539	C	C	C	C	C	C
8954	rs3719767	5	112561293	G	G	G	A	G	G
8955	rs3716512	5	112857770	A	A	G	G	A	G
8956	rs6238193	5	113059073	A	A	A	A	A	A
8957	mCV22739565	5	118393576	G	G	G	C	C	G
8958	rs3709746	5	113367366	T	T	A	A	T	A
8959	rs6223587	5	113701651	A	A	A	A	A	A
8960	rs3663141	5	114196024	G	G	A	A	G	A
8961	mCV24888028	5	119264682	A	A	G	G	G	G
8962	rs4225451	5	114538600	C	C	C	C	C	C
8963	rs4225452	5	114538695	A	A	A	A	A	A
8964	rs3023051	5	114664343	G	G	A	A	G	A
8965	rs6158283	5	114686528	A	A	A	A	A	A
8966	rs3680360	5	114730338	A	A	C	C	A	C
8967	rs3662161	5	114905705	G	G	G	A	G	G
8968	rs3693466	5	114936743	G	G	A	A	A	A
8969	rs4225453	5	115458279	G	G	G	G	G	G
8970	rs3662655	5	115709104	A	A	A	G	A	G
8971	rs6284287	5	115662246	A	A	A	A	A	A
8972	rs3656108	5	115650410	A	A	A	G	A	G
8973	rs3655469	5	115898881	G	G	A	G	G	G
8974	rs6332004	5	116351231	A	A	C	A	A	C
8975	rs3723083	5	116664942	A	A	C	A	A	A
8976	rs3707486	5	116664769	A	A	G	A	A	A
8977	rs3653889	5	116976007	G	G	G	A	G	A
8978	mCV23328629	5	122551746	G	G	G	A	G	A
8979	mCV23328630	5	122551908	C	C	C	C	C	C
8980	mCV23328631	5	122552078	A	A	G	G	G	G
8981	mCV23328638	5	122552371	G	G	G	A	G	A
8982	mCV23328639	5	122552491	A	A	C	C	C	C
8983	mCV23328640	5	122552657	A	A	G	A	G	A
8984	mCV23328908	5	122552680	A	A	A	G	A	G
8985	mCV23328909	5	122553583	C	C	G	G	G	G
8986	mCV23328910	5	122554026	A	A	A	G	A	G
8987	mCV23328919	5	122555734	A	A	A	G	A	G
8988	mCV23328938	5	122555834	G	G	G	A	G	A
8989	mCV25274159	5	122557840	A	A	A	G	A	G
8990	mCV25274160	5	122560450	A	A	A	G	A	G
8991	mCV25274170	5	122560987	G	G	G	A	G	A
8992	mCV25274178	5	122561571	G	G	G	A	G	A
8993	mCV25274180	5	122563098	T	T	A	A	A	A
8994	mCV25274187	5	122563140	A	A	G	G	G	G
8995	mCV25274189	5	122563327	A	A	G	G	G	G
8996	mCV25274196	5	122563970	C	C	C	A	C	A
8997	mCV25274197	5	122565292	A	A	G	G	G	G
8998	mCV25274198	5	122565842	G	G	G	A	G	A
8999	mCV25274205	5	122567226	A	A	A	G	A	G
9000	mCV25274214	5	122568290	G	G	A	G	A	G
9001	mCV25274215	5	122568643	A	A	A	G	A	G
9002	mCV25274222	5	122569080	A	A	A	A	A	A
9003	mCV25274223	5	122570675	A	A	C	C	C	C
9004	mCV25274224	5	122571985	T	T	A	T	A	T
9005	mCV25274491	5	122573732	G	G	A	A	A	A
9006	mCV25274492	5	122574377	A	A	A	G	A	G
9007	mCV25274493	5	122574800	G	G	G	A	G	A
9008	mCV25274500	5	122575011	A	A	G	G	G	G
9009	rs4225464	5	117686202	G	G	G	G	G	G
9010	mCV25274501	5	122575517	G	G	A	G	A	G
9011	mCV25274502	5	122578689	G	G	G	A	G	A
9012	mCV25274509	5	122579755	A	A	A	T	A	T
9013	rs6167888	5	117681273	G	G	G	G	G	G
9014	mCV25274510	5	122580476	A	A	G	G	G	G
9015	mCV25274511	5	122581079	A	A	A	G	A	G
9016	mCV25274530	5	122581084	A	A	G	G	G	G
9017	mCV22863581	5	122581878	A	A	T	T	T	T
9018	mCV22863582	5	122582498	A	A	A	G	A	G
9019	mCV22863583	5	122582518	C	C	C	A	C	A
9020	mCV22863587	5	122582971	G	G	G	A	G	A
9021	mCV22863588	5	122583748	A	A	G	G	G	G
9022	mCV22863593	5	122585221	A	A	A	G	A	G
9023	mCV22863594	5	122585452	A	A	A	G	A	G
9024	mCV22863602	5	122585720	G	G	A	G	A	G
9025	mCV22863604	5	122587549	G	G	G	A	G	A

9026	mCV22863614	5	122587587	G	G	G	C	G	C
9027	mCV22863615	5	122587687	G	G	G	A	G	A
9028	mCV22863616	5	122588050	G	G	G	A	G	A
9029	mCV22863626	5	122588565	G	G	G	A	G	A
9030	mCV22863627	5	122588596	G	G	G	A	G	A
9031	mCV22863646	5	122588640	C	C	C	G	C	G
9032	mCV22863647	5	122589816	T	T	A	T	A	T
9033	mCV22863833	5	122590750	A	A	C	C	C	C
9034	mCV22863834	5	122591332	G	G	G	A	G	A
9035	mCV24594284	5	122594513	A	A	A	T	A	T
9036	mCV24594283	5	122595458	A	A	A	C	A	C
9037	mCV24594282	5	122595614	G	G	G	A	G	A
9038	mCV24594275	5	122595690	G	G	G	A	G	A
9039	mCV24594274	5	122596103	A	A	C	C	C	C
9040	mCV24594009	5	122596628	G	G	G	A	G	A
9041	mCV24593993	5	122599193	A	A	G	G	G	G
9042	mCV24593992	5	122600338	G	G	G	G	G	G
9043	mCV24593982	5	122600507	A	A	A	G	A	G
9044	mCV24593980	5	122603912	G	G	G	A	G	A
9045	mCV24593969	5	122606202	G	G	A	A	A	A
9046	mCV24593968	5	122607193	G	G	G	A	G	A
9047	mCV24593958	5	122607515	G	G	A	A	A	A
9048	mCV24593957	5	122607543	A	A	G	G	G	G
9049	mCV24593956	5	122607565	G	G	A	A	A	A
9050	mCV24593945	5	122610201	G	G	A	G	A	G
9051	mCV24593933	5	122611250	G	G	G	A	G	A
9052	mCV24593662	5	122615306	A	A	G	A	G	A
9053	mCV24593660	5	122617432	G	G	G	A	G	A
9054	mCV24593649	5	122617625	G	G	G	A	G	A
9055	mCV24593637	5	122620224	A	A	G	G	G	G
9056	mCV24593636	5	122620554	G	G	G	A	G	A
9057	mCV24593625	5	122625304	A	A	C	C	C	C
9058	mCV25181545	5	122631433	A	A	G	A	G	A
9059	mCV25181536	5	122631621	G	G	A	G	A	G
9060	mCV25181535	5	122631985	A	A	A	G	A	G
9061	mCV25181534	5	122632014	G	G	G	A	G	G
9062	mCV25181262	5	122632748	T	T	T	A	T	A
9063	mCV24763330	5	122634899	A	A	G	A	G	A
9064	mCV23328939	5	122640469	A	A	G	G	G	G
9065	mCV23328949	5	122641368	G	G	A	G	A	G
9066	mCV23328950	5	122641517	A	A	G	G	G	G
9067	mCV22863846	5	122644608	C	C	G	C	G	C
9068	mCV22863856	5	122645121	G	G	G	A	G	A
9069	mCV22863858	5	122645489	A	A	G	A	G	A
9070	mCV22863869	5	122645743	G	G	A	G	A	G
9071	mCV22863881	5	122646174	G	G	A	G	A	G
9072	mCV22863882	5	119132739	G	G	G	G	G	G
9073	mCV22863892	5	119133911	A	A	C	A	C	A
9074	mCV22863893	5	122650234	G	G	A	G	A	G
9075	mCV22863904	5	122651178	C	C	A	C	A	C
9076	mCV22863905	5	122651482	G	G	A	G	A	G
9077	mCV22863906	5	122651666	A	A	G	A	G	A
9078	mCV22864088	5	122651870	G	G	A	G	A	G
9079	mCV22864089	5	122652105	C	C	G	C	G	C
9080	mCV22864090	5	122652879	G	G	C	G	C	G
9081	mCV22864102	5	122657207	C	C	C	A	C	A
9082	mCV22864111	5	122657412	G	G	G	A	G	A
9083	mCV22864112	5	122657618	A	A	A	G	A	G
9084	mCV22864113	5	122657799	G	G	G	A	G	A
9085	mCV22864123	5	122657886	A	A	G	G	G	G
9086	mCV22864125	5	122658745	G	G	A	G	A	G
9087	mCV22864134	5	122658840	A	A	G	G	G	G
9088	mCV22864135	5	122659352	A	A	A	G	A	G
9089	rs4225477	5	118627667	A	A	G	G	G	G
9090	rs4225478	5	118627630	C	NONE	C	C	C	C
9091	mCV22627574	5	123355119	A	A	T	T	T	T
9092	rs3710934	5	119951783	A	A	G	G	G	A
9093	rs6184919	5	118760361	A	A	A	A	A	A
9094	rs8239889	5	120442335	A	A	G	G	G	G
9095	rs8239888	5	119078055	G	G	A	A	A	A
9096	rs3655477	5	119464666	A	A	G	G	G	G
9097	rs2020514	5	119759233	C	C	C	C	C	C
9098	mCV25048733	5	124854429	A	A	T	T	T	T
9099	rs4221990	5	119838125	A	A	A	A	A	A

9100	rs6303499	5	120008358	G	G	G	G	G	G
9101	rs3710640	5	120566858	A	A	G	G	G	G
9102	rs6164615	5	120478023	G	G	G	G	G	G
9103	rs3713339	5	121438789	G	G	A	A	A	A
9104	rs6283454	5	121341264	A	A	A	A	A	A
9105	rs3662726	5	121941514	C	C	A	C	A	C
9106	rs3671202	5	122343598	G	G	G	A	G	A
9107	rs3668379	5	122437981	G	G	G	A	G	A
9108	rs3023057	5	123217417	G	G	G	A	G	A
9109	rs6204114	5	123232111	G	G	G	G	G	G
9110	rs3711485	5	123308483	A	A	G	A	G	A
9111	rs3667069	5	123312244	A	A	G	A	G	A
9112	mCV25307043	5	128482872	A	A	A	A	C	A
9113	rs3711495	5	123683739	G	G	G	A	G	G
9114	rs3692387	5	124449911	A	G	G	G	A	G
9115	rs3702446	5	124228851	A	G	A	G	A	A
9116	rs3701266	5	124228641	G	A	G	A	G	G
9117	rs3669188	5	124126610	A	G	A	G	A	A
9118	rs6395486	5	125114574	G	G	G	G	G	G
9119	rs3663155	5	125118491	A	G	A	G	A	A
9120	rs6316854	5	125413911	A	A	A	A	A	A
9121	rs3721565	5	126003798	G	A	A	G	G	G
9122	rs3659566	5	126067363	C	C	A	A	A	A
9123	rs6208181	5	126271620	G	G	G	G	G	G
9124	rs3689007	5	126361150	A	G	G	A	A	G
9125	rs6345336	5	126207424	A	G	A	G	G	A
9126	rs6287880	5	126658077	G	G	G	G	G	G
9127	mCV23024548	5	131997673	T	A	T	T	T	A
9128	rs3090425	5	126802262	T	T	T	T	T	T
9129	rs4225489	5	127064826	G	G	G	G	G	G
9130	rs3674594	5	127669017	C	A	C	C	C	C
9131	rs6295048	5	128982919	G	G	G	G	G	A
9132	rs3706272	5	128208675	T	A	A	A	A	T
9133	rs6228589	5	128437142	A	A	A	A	A	G
9134	rs3661159	5	128187011	G	C	C	C	C	G
9135	rs6377710	5	129964072	A	G	A	G	A	G
9136	rs3685067	5	130174980	A	G	A	G	A	G
9137	mCV23223919	5	135105846	A	G	G	G	G	G
9138	rs6346775	5	130758552	A	A	A	A	A	A
9139	rs4225495	5	130478824	G	G	G	G	G	G
9140	rs6374233	5	132750302	G	G	G	G	G	G
9141	mCV25017167	5	137827134	A	A	A	C	A	A
9142	rs8263003	5	133174378	G	G	G	G	G	G
9143	rs8263013	5	133176473	G	G	G	G	G	G
9144	rs8262988	5	133180237	A	A	A	A	A	A
9145	rs3023058	5	133274564	A	A	A	A	A	A
9146	rs3707215	5	133695486	G	C	C	G	C	C
9147	rs6336873	5	133959558	A	A	A	A	A	A
9148	rs3677166	5	134383323	A	A	A	G	A	A
9149	rs3711751	5	134365541	A	A	A	G	A	A
9150	rs8265855	5	135293146	G	A	A	G	A	A
9151	rs8265922	5	135313619	G	G	G	G	G	G
9152	rs8265966	5	135314940	G	G	G	G	G	G
9153	rs8265964	5	135315091	A	A	A	A	A	A
9154	rs8265976	5	135315942	G	A	A	G	A	A
9155	rs3688124	5	135046440	A	G	G	G	G	G
9156	rs6341225	5	135636416	T	T	T	T	T	T
9157	rs4225536	5	135836567	G	G	G	A	G	G
9158	rs8259893	5	136536377	A	A	A	A	A	A
9159	rs8259892	5	136536515	A	A	A	A	A	A
9160	rs3721911	5	137293980	A	A	A	T	A	A
9161	rs3701629	5	137205181	A	G	A	G	A	G
9162	rs4225541	5	137208924	A	A	A	A	A	A
9163	mCV25009162	5	142004156	A	G	G	A	G	A
9164	rs6367076	5	137484365	A	A	A	A	A	A
9165	rs3717290	5	138470267	G	A	A	A	A	A
9166	rs6292362	5	138598981	G	G	G	G	G	G
9167	rs6342577	5	138679930	C	C	C	C	C	C
9168	mCV23270057	5	140101364	G	G	G	G	G	G
9169	rs3706689	5	138826808	G	A	A	A	G	A
9170	rs3656197	5	139206611	A	A	A	G	G	A
9171	rs3700374	5	139522305	G	A	A	G	A	A
9172	rs4225550	5	139877041	A	A	A	A	A	A
9173	rs3701329	5	139807314	G	G	G	G	G	G

9174	rs6332778	5	139852979	A	A	A	A	A	A
9175	mCV22895274	5	143907789	A	G	G	A	G	G
9176	rs3684729	5	140361179	G	A	A	A	G	A
9177	rs6383868	5	140887180	A	G	G	G	G	G
9178	rs6284348	5	141098146	C	C	C	A	C	C
9179	rs3023061	5	141390786	A	G	G	G	G	G
9180	rs4137844	5	141406333	G	A	A	A	A	A
9181	rs3091206	5	141479460	A	A	A	A	A	A
9182	rs3696754	5	141905670	G	A	A	G	A	G
9183	rs3088741	5	142335567	A	A	A	A	A	A
9184	rs6341368	5	142348442	A	A	A	A	A	A
9185	rs3690014	5	142759003	A	G	G	G	G	G
9186	rs4225576	5	142653102	A	A	A	A	A	A
9187	rs6191249	5	143668186	G	A	A	G	A	G
9188	mCV22689215	5	147293460	G	A	A	G	G	A
9189	rs6302473	5	143608091	A	A	A	A	A	A
9190	rs3713156	5	143519878	G	A	A	G	A	A
9191	rs8268085	5	143206209	G	G	G	G	G	G
9192	rs8262798	5	143204005	A	A	A	A	A	A
9193	rs4225605	5	144279585	G	G	G	G	G	C
9194	rs4138683	5	144672860	G	G	G	A	A	G
9195	mCV22936617	5	149216840	G	A	A	A	A	A
9196	rs6229002	5	145306213	G	A	A	G	G	A
9197	rs3686840	5	145930999	G	G	G	A	A	A
9198	rs3687473	5	145931117	G	G	G	A	A	A
9199	rs6336221	5	145778148	G	G	G	G	G	G
9200	rs3668534	5	145832700	G	G	G	A	A	A
9201	rs3681571	5	145877872	G	G	G	A	A	A
9202	rs6239438	5	145883901	C	C	C	C	C	C
9203	rs4225626	5	145652750	G	G	G	G	G	G
9204	rs4225638	5	145653553	G	G	G	G	G	G
9205	rs3666366	5	146129346	G	C	C	G	G	C
9206	rs3710365	5	146274003	C	A	A	C	C	A
9207	rs6189812	5	146369812	A	A	A	A	A	A
9208	rs3681853	5	146801639	G	A	A	G	G	A
9209	rs3718776	5	147051702	A	A	A	G	G	A
9210	rs3090535	5	147078808	A	A	A	A	A	A
9211	rs6221768	5	147091610	G	G	G	G	G	G
9212	rs3692702	5	147670924	C	G	G	C	C	G
9213	rs6198385	5	147688128	A	C	C	A	A	C
9214	rs3722999	5	148710753	G	A	A	A	A	A
9215	rs6306257	5	148789945	A	G	G	G	G	G
9216	rs3161358	6	17792061	T	A	A	A	A	A
9217	rs6338207	6	60829506	A	A	A	A	A	A
9218	rs4225647	6	3158077	C	C	C	C	C	C
9219	rs4225651	6	3158349	C	C	C	C	C	C
9220	rs3661828	6	3167392	A	A	A	G	A	A
9221	rs6312730	6	3550315	A	A	A	A	A	A
9222	mCV25294121	6	6240972	A	A	A	C	C	A
9223	rs6180149	6	3858687	T	T	T	A	T	T
9224	rs3665949	6	4414308	A	A	A	T	A	A
9225	rs6292526	6	4420740	C	C	C	G	C	C
9226	rs6273123	6	4518414	C	C	C	G	C	C
9227	rs6315440	6	5065696	G	G	G	A	G	G
9228	rs6177560	6	5124386	G	G	G	G	G	G
9229	rs6339665	6	6015044	G	G	G	G	G	G
9230	rs3699833	6	6161470	G	G	G	A	A	G
9231	rs6168747	6	6511310	G	G	G	G	G	G
9232	rs3711696	6	6519830	A	A	A	G	G	A
9233	rs3693467	6	6643542	C	C	C	A	A	C
9234	rs3681549	6	6692081	A	A	A	G	G	A
9235	rs6355700	6	6920517	G	G	G	A	A	G
9236	mCV24751450	6	9790852	G	G	A	A	G	A
9237	rs3669958	6	7448015	C	C	A	C	C	C
9238	rs3710142	6	7795895	G	G	A	A	G	G
9239	rs4225663	6	7969036	A	A	A	A	A	A
9240	rs6380716	6	7992660	G	G	A	A	G	G
9241	rs3657898	6	8295370	G	A	A	A	A	A
9242	rs6277253	6	8687869	G	G	G	G	G	G
9243	rs6347164	6	8959287	G	G	G	G	G	G
9244	rs6257091	6	10028130	A	A	A	A	A	A
9245	rs3701161	6	10110230	A	A	G	G	A	G
9246	rs4225672	6	10747176	A	A	A	A	A	A
9247	rs6238110	6	11794382	A	A	G	G	A	A

9248	rs3668014	6	11895693	G	G	C	C	G	G
9249	rs3694099	6	12220868	G	G	A	A	G	G
9250	rs6367077	6	12729411	G	G	G	G	G	G
9251	rs6267497	6	12940301	A	A	A	A	A	A
9252	rs3655979	6	12941155	A	A	G	A	A	A
9253	rs3679568	6	13128025	G	G	A	G	G	G
9254	mCV24739681	6	15983533	T	A	A	T	A	A
9255	rs4225673	6	13381893	G	G	G	G	G	G
9256	rs3654490	6	13936631	A	A	G	A	A	A
9257	mCV23259040	6	16734530	G	G	G	A	G	G
9258	rs3678711	6	14045168	A	A	G	A	A	A
9259	rs6180825	6	14110618	G	G	A	G	G	G
9260	rs4137688	6	14289888	C	C	A	C	C	A
9261	rs6410396	6	14940956	G	G	G	G	G	G
9262	rs3090437	6	15070097	C	C	C	C	C	C
9263	rs3658616	6	15250750	G	C	C	C	C	C
9264	mCV24734110	6	18049601	A	G	G	A	G	G
9265	rs6286158	6	15526132	A	A	A	A	A	A
9266	rs3725037	6	15840490	A	G	G	A	G	G
9267	rs3705268	6	15911762	G	A	A	G	A	A
9268	rs3090436	6	15992443	A	A	A	A	A	A
9269	mCV25247470	6	18949600	A	G	G	G	G	G
9270	rs3700771	6	16385609	G	A	A	A	A	A
9271	rs3090905	6	16396375	A	A	A	A	A	A
9272	rs6375936	6	16450958	A	A	A	A	A	A
9273	rs3663703	6	16958509	G	A	A	G	A	A
9274	rs3023064	6	17466136	A	A	A	C	A	A
9275	rs6181626	6	17517562	G	A	A	A	A	A
9276	rs3655269	6	17715100	A	G	A	G	A	A
9277	mCV24714620	6	20583049	G	G	G	G	G	G
9278	rs6182329	6	18284771	G	A	A	A	A	A
9279	rs6212891	6	18285811	G	A	A	A	A	A
9280	rs6205464	6	18357860	G	A	A	A	A	A
9281	rs4225686	6	18690718	A	G	G	G	G	G
9282	rs3663804	6	19283980	G	A	A	A	A	A
9283	rs3669272	6	19446648	T	A	A	A	A	A
9284	rs6161593	6	19645161	G	C	C	C	C	C
9285	rs3666271	6	20274644	A	G	G	G	G	G
9286	rs3682861	6	20275015	G	A	A	A	A	A
9287	rs6351279	6	20296530	A	G	G	G	G	G
9288	rs6235883	6	20906640	A	A	A	A	A	A
9289	rs3089748	6	21539126	A	A	A	A	A	A
9290	rs6200683	6	21830493	A	A	A	A	A	A
9291	rs3088527	6	22701780	C	C	A	C	C	A
9292	rs6361227	6	22995693	C	C	C	C	C	C
9293	rs3710004	6	23029867	A	A	G	A	A	G
9294	rs3684277	6	23074666	A	A	G	A	A	G
9295	rs6381644	6	23271166	G	G	G	G	G	G
9296	rs3677074	6	23348833	C	G	C	C	C	C
9297	rs3090865	6	23556765	A	A	A	A	A	A
9298	rs4225716	6	23910890	A	A	A	A	A	A
9299	rs3684494	6	24248321	C	A	C	C	A	C
9300	rs6276257	6	24629292	G	G	G	G	G	G
9301	rs3091004	6	24634158	G	G	G	G	G	G
9302	rs3665582	6	24784666	A	G	A	A	G	A
9303	rs6248585	6	24976915	C	C	C	C	C	C
9304	rs3671709	6	25646449	G	G	A	G	G	A
9305	mCV25250213	6	28727285	G	G	G	G	G	G
9306	rs6264221	6	26039475	G	A	G	A	A	G
9307	rs3694385	6	26212381	A	G	A	G	G	A
9308	rs3672206	6	26299368	A	C	A	C	C	A
9309	rs3693271	6	26300455	G	A	G	A	A	G
9310	rs3696686	6	26303836	G	A	G	A	A	G
9311	rs6339670	6	26540280	A	A	A	A	A	A
9312	rs6230858	6	27327707	C	C	C	C	C	C
9313	rs4225722	6	28097157	A	A	A	A	A	G
9314	rs3686145	6	28475570	G	C	G	C	C	C
9315	rs6257799	6	28515187	A	A	A	A	A	A
9316	rs3706286	6	28633657	G	G	G	G	G	G
9317	rs6376031	6	29058140	C	C	C	C	C	C
9318	rs4225731	6	29340324	A	A	A	A	A	A
9319	rs6293344	6	29567327	G	G	G	A	G	G
9320	rs6302781	6	29730847	G	G	G	G	G	G
9321	rs3677815	6	29900346	G	G	G	A	G	G

9322	rs4225750	6	30096020	A	A	A	A	A	A
9323	rs3701429	6	30157365	G	G	G	A	G	G
9324	rs4225758	6	30538424	G	G	G	G	G	G
9325	rs6171821	6	30803395	A	A	A	A	A	A
9326	rs6314334	6	31429338	G	G	G	G	G	G
9327	rs3660699	6	31572511	G	A	A	A	A	G
9328	rs3661820	6	31572700	G	C	C	C	C	G
9329	rs3678887	6	32186519	A	A	A	G	A	A
9330	rs3670590	6	32199701	A	A	A	G	A	A
9331	rs3676393	6	32203524	A	A	A	C	A	A
9332	rs6333627	6	32315355	A	A	A	A	A	A
9333	rs4225772	6	32857341	G	G	G	G	G	G
9334	rs3710202	6	33581333	A	G	G	G	G	A
9335	rs6162891	6	33849327	C	C	C	C	C	C
9336	rs8239427	6	34209878	C	C	C	C	C	C
9337	rs4225796	6	34406305	A	A	A	A	A	A
9338	rs3674895	6	34461655	A	A	A	G	A	A
9339	rs3090069	6	34511539	A	A	A	A	A	A
9340	mCV24673275	6	37596404	C	C	A	C	A	A
9341	rs6153151	6	34766278	A	A	A	A	A	A
9342	rs4225802	6	34781650	G	G	G	G	G	G
9343	rs3685011	6	34796976	C	C	A	C	C	A
9344	rs3716901	6	35758467	G	G	A	G	G	A
9345	rs6286891	6	35925623	A	A	A	A	A	A
9346	rs3023067	6	35969149	T	T	T	A	A	T
9347	rs3704635	6	36040358	A	A	A	G	G	A
9348	rs3670489	6	36263207	C	C	C	A	A	C
9349	rs6354750	6	36276256	G	G	G	G	G	G
9350	rs3653923	6	36299590	A	A	A	G	G	A
9351	rs8252871	6	36733866	G	G	G	G	G	G
9352	rs3678202	6	36995978	G	A	A	A	A	A
9353	rs6382825	6	37856916	A	A	A	A	A	A
9354	rs6215575	6	38058604	A	A	G	A	G	G
9355	rs6401658	6	38721547	A	A	A	A	A	A
9356	rs6172651	6	39595547	T	T	T	T	T	T
9357	rs6263401	6	40027509	G	G	G	G	G	G
9358	rs6182787	6	40776801	A	A	A	A	A	A
9359	rs6304139	6	41663383	A	A	A	A	A	A
9360	mCV24657328	6	45316437	C	G	C	C	C	G
9361	rs6372754	6	43234459	G	G	G	G	G	G
9362	rs4225857	6	43242630	C	C	C	C	C	C
9363	rs4225856	6	43242677	C	C	C	C	C	C
9364	rs3662661	6	43917351	A	A	A	G	A	G
9365	rs6258667	6	44094488	A	A	A	A	A	A
9366	mCV22897612	6	47559396	G	A	A	G	A	A
9367	rs3666470	6	45021385	G	A	G	A	G	A
9368	rs3668229	6	45021639	C	A	C	A	C	A
9369	rs6319193	6	45081840	G	A	G	A	G	A
9370	mCV22323972	6	48305626	A	A	A	T	A	A
9371	rs6156701	6	45744539	G	G	G	G	G	G
9372	rs6390612	6	46436601	G	C	G	G	G	C
9373	rs6198015	6	47430301	C	C	C	C	C	C
9374	rs3686723	6	47632856	A	G	G	A	G	G
9375	rs4225865	6	47712247	C	C	C	C	C	C
9376	rs6179500	6	47812230	C	C	C	C	C	C
9377	rs3089355	6	47853523	A	A	A	A	A	A
9378	rs6290329	6	48462557	A	G	G	A	G	G
9379	rs4225870	6	48510702	A	G	A	A	A	A
9380	rs4225874	6	48510932	G	G	G	G	G	G
9381	rs3684860	6	48907491	G	G	C	G	C	C
9382	rs3669453	6	48900760	G	G	G	A	G	G
9383	rs4225890	6	49051395	A	A	G	A	G	G
9384	rs4225897	6	49267523	A	A	A	A	A	A
9385	rs3698072	6	49369832	C	A	A	C	A	A
9386	rs6155681	6	49599767	A	G	G	A	G	G
9387	rs4139698	6	49813889	G	A	A	A	A	A
9388	rs3088971	6	50071521	G	G	G	G	G	G
9389	rs3708108	6	50169071	G	G	G	A	G	G
9390	rs3664026	6	50179748	G	A	A	A	A	A
9391	rs3718512	6	50309925	G	G	G	A	G	G
9392	rs6302586	6	50314167	G	A	A	A	A	A
9393	rs6239043	6	50948126	A	A	A	G	A	A
9394	rs6320475	6	10765644	C	A	A	C	A	A
9395	rs3710220	6	51145866	G	A	A	A	A	A

9396	rs3716680	6	51742743	A	A	A	G	A	A
9397	rs3704207	6	51785906	A	A	A	G	A	A
9398	rs6228043	6	51842843	C	C	C	C	C	C
9399	rs3089234	6	51960028	A	A	A	A	A	A
9400	rs3023069	6	52190471	C	A	C	A	C	A
9401	rs4225899	6	52715551	C	C	C	C	C	C
9402	rs6207831	6	52760601	A	A	A	A	A	A
9403	rs3722786	6	52861881	A	T	A	T	A	T
9404	rs3688920	6	53559932	G	G	G	A	G	G
9405	rs3706868	6	53593969	G	G	G	A	G	G
9406	rs6219138	6	53595273	C	C	C	A	C	C
9407	rs3653600	6	53715471	G	A	G	G	G	G
9408	rs6272774	6	54131745	G	G	G	A	G	G
9409	rs6372575	6	54221556	G	G	G	A	G	G
9410	rs4225919	6	54457424	C	C	C	C	C	C
9411	rs4225914	6	54969688	G	G	G	G	G	G
9412	rs3655236	6	54501546	A	A	A	G	A	A
9413	mCV23230863	6	57474918	G	C	C	C	C	C
9414	rs6199040	6	54764534	C	C	C	A	C	C
9415	rs3714727	6	54950032	C	C	C	A	C	C
9416	rs6308025	6	55132441	G	G	G	A	G	G
9417	rs4225924	6	55298555	G	G	G	G	G	G
9418	rs3023071	6	55410751	G	G	G	A	G	G
9419	rs3687179	6	55479778	G	A	A	G	A	A
9420	rs3664475	6	55518347	T	A	A	T	A	A
9421	rs4138262	6	55980402	A	G	G	G	G	G
9422	rs6291482	6	55993216	G	A	A	A	A	A
9423	rs4225937	6	56090907	G	G	G	G	G	G
9424	rs6351606	6	56838795	A	C	C	C	C	C
9425	rs3680689	6	56849758	T	A	A	A	A	A
9426	rs6299256	6	57600793	A	C	C	C	C	C
9427	rs3657885	6	57639538	G	A	A	A	A	A
9428	rs6172269	6	58003691	A	A	A	A	A	A
9429	rs4225947	6	58696969	G	G	G	G	G	G
9430	rs4225950	6	58939188	A	A	A	A	A	A
9431	rs6251021	6	59454182	G	G	G	G	G	G
9432	rs3089524	6	60080963	A	A	A	A	A	A
9433	mCV22357717	6	61526265	G	A	A	A	A	A
9434	rs6212857	6	61065869	A	A	A	A	A	A
9435	rs3672806	6	61306235	A	G	G	G	G	G
9436	rs6393315	6	61408211	G	A	A	A	A	A
9437	rs6154559	6	62092666	G	A	A	A	A	A
9438	rs3705231	6	62157950	A	A	A	A	A	A
9439	mCV24558382	6	64479451	A	A	G	A	G	A
9440	rs6203413	6	63274630	T	A	A	A	A	A
9441	rs3721391	6	63320432	A	C	C	C	C	C
9442	rs3724916	6	63434326	G	A	A	A	A	A
9443	mCV22576656	6	65132955	G	G	A	G	A	G
9444	rs6152576	6	63684569	G	A	A	A	A	A
9445	rs3686305	6	63830085	A	G	G	G	G	G
9446	rs3089540	6	64222243	A	A	G	A	G	A
9447	rs3089541	6	64222303	G	G	G	G	G	G
9448	rs3697325	6	64279555	A	A	C	A	C	A
9449	rs4136902	6	64299739	A	A	G	A	G	A
9450	rs6160457	6	64368321	A	A	G	A	G	A
9451	rs6160989	6	65213559	G	G	G	G	G	G
9452	rs8270116	6	65221128	T	T	A	T	A	T
9453	rs8270121	6	65223957	C	C	C	C	C	C
9454	rs4225954	6	65941419	G	G	G	G	G	G
9455	rs3089195	6	66539144	C	C	C	C	C	C
9456	rs6320818	6	66619473	T	T	T	T	T	T
9457	rs3090935	6	67147970	C	C	C	C	C	C
9458	rs6157367	6	67399954	A	A	A	T	A	A
9459	rs4225970	6	67407049	A	A	A	A	A	A
9460	rs4225973	6	67407161	A	A	A	A	A	A
9461	mCV24561783	6	68077964	A	A	A	A	A	A
9462	mCV27563186	6	71079194	A	G	G	G	G	G
9463	mCV24929761	6	71823606	A	A	C	A	C	A
9464	rs6177126	6	70336034	C	C	C	C	C	C
9465	mCV23348277	6	72456837	A	A	G	A	G	A
9466	rs6323214	6	71148204	C	C	C	C	C	C
9467	rs4225976	6	71408170	G	G	G	G	G	G
9468	rs4225979	6	71580466	G	G	G	G	G	G
9469	rs3723437	6	71596090	T	T	A	T	A	T

9470	rs3665567	6	71596382	A	A	G	A	G	A
9471	rs6246241	6	71998176	A	A	G	A	G	A
9472	rs6243983	6	72069405	A	A	T	A	T	A
9473	rs4225996	6	72109892	A	A	G	A	G	A
9474	rs4225998	6	72110069	G	G	C	G	C	G
9475	rs3673305	6	72148155	C	C	A	C	A	C
9476	mCV24572596	6	75426013	G	G	G	A	G	G
9477	rs3717302	6	73401556	C	C	C	G	C	C
9478	rs6191521	6	73554469	T	T	T	A	T	T
9479	rs6382785	6	73881876	C	C	C	C	C	C
9480	rs3725176	6	73929048	A	A	G	G	G	A
9481	rs3719988	6	73958293	A	A	A	G	A	A
9482	rs3663417	6	74802824	G	G	G	A	G	G
9483	rs6309736	6	74829036	A	A	A	C	A	A
9484	rs3659285	6	74962608	G	G	G	A	G	G
9485	rs3712284	6	74995203	C	C	C	A	C	C
9486	rs3672029	6	75631345	A	A	A	C	A	A
9487	rs6411497	6	75750645	G	G	A	A	A	G
9488	rs3710429	6	75776014	G	G	A	A	A	G
9489	rs3659315	6	75777359	A	A	G	G	G	A
9490	mCV25007388	6	78038399	A	G	G	G	G	G
9491	rs3686586	6	76026695	A	A	G	G	G	A
9492	rs6234428	6	76036137	G	G	A	A	A	G
9493	rs3699863	6	76063904	G	G	A	A	A	G
9494	rs4226002	6	76217717	C	C	C	C	C	C
9495	rs3699367	6	76679118	A	C	C	A	C	C
9496	rs4136200	6	77033114	A	A	G	G	G	G
9497	rs6344545	6	77072638	A	A	G	G	G	G
9498	rs3724558	6	77098302	G	G	A	A	A	A
9499	rs3706039	6	77343585	G	G	A	G	A	A
9500	rs3663024	6	77763528	G	G	G	A	G	G
9501	rs6412632	6	77876232	C	C	C	C	C	C
9502	rs4226013	6	78704205	G	G	G	G	G	G
9503	rs6259164	6	78776040	G	G	A	A	A	A
9504	mCV23923871	6	81736483	C	A	C	C	C	A
9505	rs3717633	6	79653940	A	G	G	G	G	G
9506	rs6302422	6	79825735	A	A	A	A	A	A
9507	rs3694069	6	79826449	G	A	A	A	A	A
9508	rs6377140	6	80032286	G	G	A	A	A	G
9509	rs6331100	6	81084801	T	T	A	A	A	T
9510	rs3698364	6	81140583	A	G	G	G	G	G
9511	rs6181382	6	81674296	A	G	A	A	A	G
9512	rs4226026	6	82185657	A	A	A	A	A	A
9513	rs4226027	6	82185782	A	A	G	G	G	A
9514	rs3691925	6	82188051	A	A	C	C	C	A
9515	rs6308628	6	82758911	G	A	A	A	A	A
9516	rs3152159	6	82980113	C	C	A	A	A	C
9517	mCV24996788	6	84667724	A	A	A	A	A	A
9518	rs4226028	6	83825455	A	G	G	G	G	G
9519	rs4226036	6	84714866	A	A	A	A	A	A
9520	rs6280381	6	83902721	A	A	G	G	G	A
9521	rs4226046	6	84006793	C	C	C	C	C	C
9522	rs4226048	6	84007106	G	G	A	A	A	G
9523	rs6384008	6	84260732	A	A	A	A	A	A
9524	mCV24609370	6	87070705	G	G	G	A	G	G
9525	mCV24609371	6	87070848	G	G	G	A	G	G
9526	mCV24609372	6	87070902	G	G	G	A	G	G
9527	mCV24609382	6	87071701	G	G	G	A	G	G
9528	mCV24609384	6	87072455	A	A	A	G	A	A
9529	mCV24609655	6	87072551	G	G	G	A	G	G
9530	mCV24609657	6	87074787	A	A	A	G	A	A
9531	mCV24609668	6	87075045	G	G	G	A	G	G
9532	mCV24609678	6	87075229	C	C	C	G	C	C
9533	mCV24609690	6	87076117	G	G	G	A	G	G
9534	mCV24609692	6	87076262	G	G	G	A	G	G
9535	mCV24609702	6	87076454	A	A	A	G	A	A
9536	mCV24609704	6	87076774	G	G	G	A	G	G
9537	mCV24609711	6	87076825	G	G	G	A	G	G
9538	mCV24609712	6	87077363	A	A	A	G	A	A
9539	mCV24609713	6	87077471	C	C	C	A	C	C
9540	mCV24609730	6	87077947	A	A	A	A	A	A
9541	mCV23945442	6	87079291	T	T	T	A	T	T
9542	mCV23945452	6	87080589	G	G	G	G	G	G
9543	mCV23945453	6	87089469	A	A	A	G	A	A

9544	mCV23945465	6	87093506	A	A	A	G	A	A
9545	mCV23945466	6	87093939	G	G	G	A	G	G
9546	mCV23945476	6	87097235	T	A	T	T	T	T
9547	mCV23945478	6	87103512	A	A	A	C	A	A
9548	mCV23945488	6	87103524	G	G	G	A	G	G
9549	mCV23945762	6	87103992	G	G	G	A	G	G
9550	mCV23945772	6	87104462	G	G	G	A	G	G
9551	mCV23945773	6	87104562	G	G	G	A	G	G
9552	mCV23945783	6	87104999	A	A	A	G	A	A
9553	mCV23945785	6	87105082	A	A	A	G	A	A
9554	mCV23945795	6	87105189	G	G	G	A	G	G
9555	mCV23945796	6	87105223	A	A	A	G	A	A
9556	mCV23945808	6	87105538	A	A	A	G	A	A
9557	mCV23945819	6	87105596	G	G	G	A	G	G
9558	mCV23945820	6	87105620	G	G	G	A	G	G
9559	mCV23945830	6	87105769	A	A	A	G	A	A
9560	mCV23945832	6	87106474	C	C	C	A	C	C
9561	mCV23946102	6	87107046	G	G	G	A	G	G
9562	mCV23946108	6	87107098	A	A	A	G	A	A
9563	mCV23946117	6	87110285	A	A	A	G	A	A
9564	mCV23946126	6	87111446	A	A	A	G	A	A
9565	mCV23946127	6	87112300	T	T	T	A	T	T
9566	mCV23946128	6	87112839	C	C	C	A	C	C
9567	mCV23946135	6	87112916	C	C	C	A	C	C
9568	mCV23946136	6	87113000	A	A	A	G	A	A
9569	mCV23946137	6	87113554	G	G	G	A	G	G
9570	mCV23946144	6	87113559	G	G	G	A	G	G
9571	mCV23946145	6	87113600	C	C	C	A	C	C
9572	mCV23946153	6	87113855	C	C	C	A	C	C
9573	mCV23946155	6	87114407	A	A	A	T	A	A
9574	mCV23946169	6	87114686	A	A	A	G	A	A
9575	rs6325249	6	84816591	A	A	A	A	A	A
9576	mCV23946174	6	87114958	G	G	G	A	G	G
9577	mCV23946175	6	87115002	A	A	A	G	A	A
9578	mCV25186131	6	87116062	A	A	A	G	A	A
9579	mCV25186132	6	87116396	A	A	A	G	A	A
9580	mCV25186133	6	87116906	A	A	A	G	A	A
9581	mCV25186663	6	87117993	G	G	G	A	G	G
9582	mCV22847896	6	87119116	G	G	G	A	G	G
9583	mCV22847897	6	87119156	A	A	A	C	A	A
9584	mCV22847898	6	87120681	G	G	G	A	G	G
9585	mCV22847909	6	87121306	G	G	G	G	G	G
9586	mCV22847922	6	87122442	C	C	C	A	C	C
9587	mCV22847923	6	87122456	A	A	A	G	A	A
9588	mCV22848107	6	87123074	A	A	A	G	A	A
9589	mCV22848108	6	87123125	A	A	A	G	A	A
9590	mCV22848109	6	87125088	A	A	A	G	A	A
9591	mCV22848121	6	87125918	G	G	G	A	G	G
9592	mCV22848131	6	87126780	A	A	A	C	A	A
9593	rs3662130	6	85043367	A	C	C	C	C	C
9594	rs6317737	6	85659308	A	A	A	A	A	A
9595	rs6268328	6	86736209	C	C	C	A	C	C
9596	rs4221991	6	86745667	A	A	A	A	A	A
9597	rs4221995	6	86745902	T	T	T	A	T	T
9598	rs4226061	6	86963614	A	A	A	G	A	A
9599	rs4226063	6	86963688	A	A	A	G	A	A
9600	rs6209558	6	86988811	A	A	A	G	A	A
9601	rs4226074	6	87070336	G	G	G	G	G	G
9602	rs6268125	6	87214811	C	C	C	A	C	C
9603	rs3677567	6	87604055	G	G	G	G	G	G
9604	rs3712958	6	87831455	G	G	G	G	G	G
9605	rs3676646	6	87902736	A	G	G	G	G	G
9606	mCV24614895	6	89046445	G	A	G	G	G	G
9607	rs6314354	6	88014594	G	G	G	G	G	G
9608	rs3089307	6	88688843	C	C	C	C	C	C
9609	rs4135401	6	88714929	A	A	A	A	A	A
9610	rs3668979	6	88984171	G	A	G	A	G	G
9611	rs6195822	6	89025012	C	A	C	A	C	C
9612	rs3671004	6	89036720	G	C	G	C	G	G
9613	rs3713705	6	89055087	A	A	A	C	A	A
9614	mCV23257111	6	91707639	G	A	G	G	G	G
9615	rs4226089	6	89485781	G	A	G	A	G	G
9616	rs6190582	6	89642953	A	G	A	A	A	A
9617	rs3701169	6	89686517	A	A	A	C	A	A

9618	rs6330399	6	90413491	A	T	A	A	A	A
9619	rs3661039	6	90696160	A	A	A	C	A	A
9620	rs4226131	6	90742264	G	G	G	G	G	G
9621	rs4226133	6	90816023	A	A	A	A	A	A
9622	rs6156752	6	91115486	A	A	A	G	A	A
9623	rs3680743	6	91514685	A	G	G	G	A	G
9624	rs3683775	6	91591146	G	A	A	A	G	A
9625	rs4226134	6	92155618	G	G	G	G	G	G
9626	rs3704682	6	92377910	A	A	C	C	A	C
9627	rs3091092	6	92594013	G	G	G	G	G	G
9628	rs3691686	6	92952138	G	G	A	A	G	A
9629	rs6223362	6	92975843	A	A	A	G	A	A
9630	rs3723136	6	93022028	G	G	A	G	G	G
9631	rs3716206	6	93247281	C	A/C	C	A	C	C
9632	mCV24791247	6	95949403	A	G	A	A	G	G
9633	rs6285738	6	93892091	G	G	A	G	G	G
9634	rs6239023	6	94413050	A	A	G	G	G	A
9635	rs6193006	6	94643264	G	G	A	G	G	G
9636	rs3718968	6	94883844	G	G	A	G	G	G
9637	rs3720133	6	94884022	G	G	A	G	G	G
9638	rs3659436	6	95011700	G	G	G	A	G	G
9639	rs6226609	6	95048665	G	G	G	A	G	G
9640	rs4226142	6	95615475	C	C	C	C	C	C
9641	rs6344085	6	95870755	A	A	A	A	G	A
9642	rs3090144	6	96569906	C	C	C	C	C	C
9643	rs6308758	6	96962182	A	A	A	A	G	A
9644	rs6349084	6	97121415	G	A	G	G	A	A
9645	rs3090740	6	97360646	A	A	A	A	A	A
9646	rs3023081	6	97661458	G	A	G	G	G	A
9647	rs4226156	6	97665288	G	G	G	G	G	G
9648	rs4226157	6	97665320	C	C	C	C	C	C
9649	rs6234973	6	98059474	A	G	A	A	G	G
9650	rs3702663	6	98686528	G	A	A	G	A	A
9651	rs4138572	6	98790763	A	G	G	A	G	G
9652	rs6294376	6	98962961	G	G	G	G	G	G
9653	rs6243885	6	99754807	C	C	C	C	C	C
9654	rs4226159	6	100006710	G	G	G	G	G	G
9655	mCV23117732	6	103251269	T	A	T	T	T	T
9656	rs6326610	6	101030119	A	A	A	A	A	A
9657	rs3718735	6	101508462	G	G	G	A	G	A
9658	rs3663448	6	101542613	A	A	A	G	A	G
9659	rs6396159	6	101801742	T	T	T	A	T	T
9660	rs3694444	6	101838023	G	G	G	A	G	G
9661	rs3678079	6	101868513	G	G	G	C	G	G
9662	rs6323483	6	102626389	G	G	G	A	G	A
9663	rs3672073	6	102863317	A	A	A	G	A	G
9664	rs6303579	6	102971130	G	G	G	G	G	G
9665	rs3692519	6	103850659	C	A	A	A	A	A
9666	rs4226164	6	104111155	T	T	T	T	T	T
9667	rs4226162	6	104111424	G	G	G	G	G	G
9668	rs6221826	6	104277893	G	G	G	G	G	G
9669	rs3691330	6	104559313	A	C	C	A	A	A
9670	rs6202661	6	104733791	A	G	G	A	A	G
9671	mCV24647857	6	107658117	T	A	A	A	A	T
9672	rs6292642	6	104934072	C	C	C	A	A	C
9673	rs3677553	6	104960377	G	G	G	A	A	G
9674	rs3671969	6	105115728	A	A	A	G	G	A
9675	rs3722170	6	105265106	A	A	A	G	G	A
9676	rs3676140	6	105268876	T	A	A	T	T	T
9677	rs6208251	6	105270851	G	A	A	G	G	A
9678	rs3667187	6	105606247	A	A	A	G	G	G
9679	rs3718253	6	106356052	G	A	A	G	G	G
9680	rs6344812	6	106399804	A	A	A	G	A	A
9681	rs3688307	6	106450850	A	A	A	G	A	A
9682	rs3725108	6	106507018	T	T	T	A	T	T
9683	rs6314022	6	106779555	A	C	C	A	C	A
9684	rs6216848	6	106912146	G	G	G	A	G	G
9685	rs3703994	6	106917378	A	A	A	C	A	A
9686	rs3691050	6	106920335	A	T	T	A	T	A
9687	rs3657731	6	107019973	A	A	A	G	A	A
9688	rs4226166	6	107173366	G	G	G	G	G	G
9689	rs4226167	6	107173546	A	A	A	A	A	A
9690	rs3703026	6	108133583	A	A	A	G	G	G
9691	rs6221865	6	108176275	C	C	C	C	A	C

9692	rs3655148	6	108183645	A	A	A	G	G	A
9693	rs3702255	6	108358015	C	C	C	A	NONE	C
9694	rs3023084	6	108852931	G	G	G	G	A	G
9695	rs3089219	6	108852953	C	C	NONE	C	C	C
9696	rs6239446	6	108926981	G	G	G	A	G	G
9697	rs6233194	6	109044672	A	G	G	G	A	A
9698	rs3722224	6	109099435	A	A	A	C	C	A
9699	rs3723422	6	109099648	A	G	G	A	G	A
9700	rs4226177	6	109238569	G	G	G	G	G	G
9701	rs3688610	6	109248452	G	G	G	A	A	G
9702	mCV24107884	6	110746472	A	G	A	A	G	A
9703	rs6270048	6	109694094	A	A	G	A	G	A
9704	rs4138146	6	109912067	A	G	G	G	G	A
9705	rs6256236	6	110228065	A	A	A	G	A	A
9706	mCV24788350	6	113337615	A	A	G	A	G	G
9707	rs3668709	6	110396763	G	G	G	A	G	G
9708	rs3670475	6	110397055	A	A	C	A	C	A
9709	rs3716629	6	110710569	G	A	G	G	A	G
9710	rs6250128	6	110791152	A	A	A	A	A	A
9711	rs6386722	6	111079059	G	G	G	G	G	G
9712	rs3690473	6	111262564	G	C	G	C	C	C
9713	rs3722772	6	111802735	G	G	G	A	G	G
9714	rs3723911	6	111802901	C	C	C	A	C	C
9715	rs3698226	6	111944017	C	C	C	NONE	C	C
9716	rs6290844	6	111979111	A	A	G	G	A	G
9717	rs6331868	6	112020795	G	A	A	A	A	G
9718	mCV24115224	6	115030499	G	A	G	A	G	G
9719	rs4138136	6	112574689	A	G	A	A	G	A
9720	rs3089440	6	113258243	A	A	A	A	A	A
9721	rs2228903	6	113742528	A	A	A	A	A	A
9722	rs6155326	6	115072187	A	A	G	A	G	G
9723	rs3693392	6	113842151	G	G	A	G	G	A
9724	rs4226201	6	113909952	C	C	C	C	C	C
9725	rs4226202	6	113990801	A	A	A	A	A	A
9726	mCV23042866	6	117147180	C	A	C	A	C	A
9727	rs3680164	6	114380213	C	C	A	C	A	A
9728	rs2228931	6	115638568	G	G	G	G	G	G
9729	rs6241276	6	114406699	A	A	G	A	G	G
9730	rs8259195	6	114823368	A	A	A	A	A	A
9731	rs8259354	6	114835146	C	C	C	C	C	C
9732	rs3709206	6	114870193	A	G	G	G	A	A
9733	rs6393943	6	114932370	A	G	G	G	A	A
9734	rs3720842	6	114972506	A	G	A	G	A	A
9735	rs8243438	6	115005944	G	G	A	G	G	G
9736	rs3023088	6	115557643	A	A	G	A	A	A
9737	rs4226207	6	115669743	G	G	G	G	G	G
9738	rs8254778	6	115749258	A	A	G	A	A	A
9739	rs8254782	6	115878764	A	A	G	A	A	A
9740	rs3705972	6	116177465	A	A	C	A	A	A
9741	rs3722915	6	116251732	T	T	A	T	T	T
9742	rs6204829	6	116303467	A	G	A	G	A	A
9743	rs3668689	6	116318240	A	G	G	G	A	G
9744	rs4226214	6	116330113	G	G	A	G	G	G
9745	rs3722084	6	116376059	C	G	G	G	C	G
9746	rs6401637	6	116477552	G	A	G	A	G	G
9747	rs3717778	6	116777385	C	A	C	A	C	A
9748	rs4226224	6	116800560	G	G	G	G	G	G
9749	rs4226225	6	116800621	A	G	G	G	A	G
9750	rs8245876	6	117562025	G	G	G	G	G	C
9751	rs8245877	6	117562115	G	G	G	G	G	G
9752	rs6380803	6	117672619	A	A	A	A	A	C
9753	rs3716551	6	118860281	A	C	C	C	A	C
9754	mCV24892629	6	121702158	G	G	G	A	A	A
9755	rs6309473	6	119087694	G	A	A	A	G	G
9756	rs3707602	6	119356834	G	G	G	A	G	G
9757	rs4137862	6	119576264	C	A	A	C	C	C
9758	rs6306957	6	119802898	C	G	G	G	C	C
9759	rs4226235	6	119824419	A	G	G	G	A	A
9760	rs4226241	6	119824591	G	G	G	G	G	G
9761	rs3718884	6	120148978	A	A	A	G	A	G
9762	rs3721974	6	120197698	G	A	A	A	G	A
9763	rs6257337	6	120358766	C	C	C	C	C	C
9764	rs3695724	6	120403722	T	T	T	A	T	A
9765	rs2020691	6	120436078	A	A	A	A	A	A

9766	rs6211640	6	121490896	G	G	G	A	A	A
9767	rs3723821	6	121529823	G	G	G	A	A	A
9768	rs6342020	6	121825584	A	A	A	A	A	A
9769	rs6303910	6	122025275	A	A	A	A	A	A
9770	rs3709324	6	122048530	A	A	A	T	T	T
9771	rs3727110	6	122527688	A	A	A	T	A	A
9772	rs8258829	6	122941147	C	C	C	C	C	C
9773	rs3023093	6	122941218	C	C	C	A	C	C
9774	rs6308350	6	122944401	A	A	A	A	A	A
9775	rs3722157	6	123050511	C	C	C	G	G	G
9776	rs6365235	6	124740027	G	G	G	G	G	G
9777	rs3714944	6	125731325	G	A	A	G	A	A
9778	rs3716297	6	125731562	T	A	A	T	A	A
9779	rs6276451	6	125873555	G	G	G	G	G	G
9780	rs3684245	6	125990135	A	G	G	A	G	G
9781	rs6200835	6	126340874	G	A	A	A	A	A
9782	rs6201882	6	126530929	G	A	A	A	A	A
9783	rs6189836	6	127188740	A	G	G	G	G	G
9784	rs6389420	6	127676012	A	G	G	A	G	G
9785	rs4226273	6	127716362	A	A	A	A	A	A
9786	rs4226280	6	127716606	A	G	G	A	G	G
9787	rs3708837	6	127800257	G	A	A	G	A	A
9788	mCV24646413	6	129352951	A	G	G	A	G	G
9789	rs3673730	6	128402950	A	A	A	C	A	A
9790	rs3698224	6	128484855	G	G	G	A	G	G
9791	rs6390151	6	128541601	G	G	G	A	G	A
9792	mCV22521691	6	132934802	T	A	A	T	A	T
9793	rs3681620	6	128654310	G	G	G	A	G	G
9794	rs3654472	6	128992745	G	G	G	A	G	G
9795	rs3670851	6	129225234	G	G	G	A	G	G
9796	rs6354131	6	129234018	A	A	A	G	A	A
9797	rs3709615	6	129512920	G	G	G	A	G	A
9798	rs4226301	6	129541739	A	A	A	C	A	C
9799	rs6338676	6	129811183	G	G	G	A	G	A
9800	rs3696743	6	130182816	G	A	A	G	A	A
9801	rs4226310	6	130203071	A	A	A	A	A	A
9802	rs3662241	6	130271567	A	G	G	A	G	A
9803	rs6375880	6	130282498	G	A	A	G	A	A
9804	rs6169509	6	131152582	T	A	A	A	A	T
9805	rs6307910	6	131203637	A	A	A	G	A	A
9806	rs3654483	6	131216000	A	G	G	A	G	A
9807	mCV23007537	6	145567593	A	A	G	G	G	A
9808	rs3722480	6	131616421	G	A	A	G	A	A
9809	rs3699247	6	131621532	A	C	C	A	C	A
9810	rs6290696	6	131622994	A	G	G	A	G	A
9811	rs3677485	6	131740498	A	A	A	G	A	A
9812	mCV23509699	6	133078488	A	C	C	A	C	C
9813	rs3688358	6	131958443	A	A	A	T	A	A
9814	rs3704289	6	132322704	G	C	C	G	C	G
9815	rs3695948	6	132331850	A	A	A	C	A	C
9816	rs6312743	6	132975892	A	G	G	A	G	A
9817	rs4226321	6	133171895	G	A	A	G	A	G
9818	rs3655878	6	133684195	A	G	G	A	G	A
9819	rs3670215	6	133742547	A	C	C	A	C	A
9820	rs3664937	6	133765572	C	G	G	C	G	C
9821	rs6339546	6	134019679	A	G	G	A	G	G
9822	rs3665786	6	134069741	G	A	A	G	A	G
9823	rs3655188	6	134153594	G	G	G	A	G	G
9824	rs3721792	6	134232590	G	A	A	A	A	A
9825	rs3678980	6	134521641	G	A	A	G	A	G
9826	rs6334723	6	134753250	G	G	G	A	G	G
9827	rs3671932	6	134909143	C	C	C	G	C	C
9828	rs3700264	6	134926240	A	A	A	G	A	G
9829	rs3699650	6	135135979	C	C	C	A	C	C
9830	rs6215934	6	135786603	A	A	A	G	A	A
9831	rs3693762	6	135850098	G	G	G	A	G	G
9832	rs3711652	6	136037310	A	A	A	G	A	A
9833	rs3657332	6	136038097	A	A	A	C	A	A
9834	rs3704502	6	136317219	G	G	G	A	G	G
9835	rs4226339	6	136461837	G	G	G	A	G	G
9836	rs3673059	6	136491989	A	A	A	G	A	G
9837	rs6340756	6	136674146	A	A	A	A	A	A
9838	rs3687201	6	137063948	G	G	G	A	G	G
9839	rs4137105	6	137063994	A	A	A	A	A	A

9840	rs6317763	6	137465427	G	G	G	G	G	G
9841	rs3725987	6	137657411	G	G	G	A	G	G
9842	rs4226344	6	137848932	T	A	A	A	A	A
9843	rs8261311	6	138254620	G	G	G	G	G	A
9844	rs8261333	6	138261225	A	A	A	A	A	A
9845	rs3682699	6	138541364	G	A	A	G	A	G
9846	rs3675155	6	138637212	A	C	NONE	A	C	A
9847	rs3712962	6	138670785	G	A	A	G	A	G
9848	rs6261913	6	138754751	G	A	A	G	A	G
9849	rs3658123	6	138953974	A	G	G	A	G	G
9850	rs3690783	6	139110749	G	C	C	C	C	C
9851	rs6255954	6	139167397	A	G	G	A	G	A
9852	rs6198929	6	139269336	A	C	C	A	C	A
9853	rs3715443	6	139563765	A	G	G	G	G	G
9854	rs6288584	6	139627353	A	G	G	A	G	G
9855	rs3679077	6	139637531	G	A	A	G	A	G
9856	rs3672808	6	139965472	G	A	A	G	A	A
9857	rs3664540	6	140232726	A	A	A	G	A	G
9858	rs3676517	6	140276377	A	A	A	C	A	A
9859	rs6335021	6	140415198	A	A	A	A	A	G
9860	rs4226345	6	140776739	A	A	A	A	A	A
9861	rs6396986	6	141296758	G	G	G	G	G	G
9862	rs8268650	6	141660661	G	G	G	G	G	A
9863	rs8262225	6	141661043	G	G	G	G	G	G
9864	rs8255572	6	141675921	T	T	T	T	T	T
9865	rs8262499	6	141694583	G	G	G	G	G	A
9866	rs8262468	6	141695864	A	A	A	A	A	G
9867	rs4226351	6	142734106	A	A	A	A	A	A
9868	rs6345000	6	143108355	G	G	G	G	G	G
9869	rs4226359	6	143447035	C	C	C	C	C	A
9870	rs6152631	6	144204769	G	G	A	G	A	G
9871	rs6272420	6	144223974	A	A	C	A	C	C
9872	rs3671022	6	144245274	A	A	G	G	G	A
9873	rs3705112	6	144266213	C	C	G	G	G	C
9874	rs6386657	6	144464865	G	G	A	G	A	G
9875	rs3658783	6	144465978	G	G	G	A	G	G
9876	rs3716907	6	144568344	A	A	G	A	G	A
9877	rs3705745	6	144833783	A	A	G	A	G	A
9878	rs3677570	6	144838062	C	C	A	C	A	C
9879	rs3694396	4	112812399	A	A	G	A	A	A
9880	mCV23060344	6	146411748	C	C	G	G	G	G
9881	rs4140069	6	145027219	A	A	G	A	G	A
9882	rs6195302	6	145124564	A	A	G	G	G	A
9883	rs3671011	6	145136382	C	C	A	A	A	C
9884	rs3721559	6	145343140	A	A	G	G	G	A
9885	rs4226363	6	145375378	G	G	G	G	G	G
9886	rs3089737	6	145624262	G	G	G	A	G	G
9887	rs6169153	6	145732310	G	G	A	G	A	G
9888	rs6387265	6	145879903	G	G	G	A	G	G
9889	rs6329917	6	145944662	G	G	A	G	A	G
9890	rs3721620	6	145951335	A	A	T	A	T	A
9891	rs3654777	6	145957856	G	G	G	A	G	G
9892	rs3023100	6	146208358	C	C	A	C	A	C
9893	rs3023105	6	146597312	G	G	G	A	G	G
9894	rs3090435	6	146754186	A	A	A	G	A	A
9895	rs4226371	6	146770974	G	G	G	A	G	G
9896	rs3725661	6	146788550	G	G	G	A	G	G
9897	rs3684551	6	146803819	A	A	A	G	A	A
9898	rs3712253	6	146863715	G	G	G	A	G	G
9899	rs4226375	6	147158309	A	A	A	A	A	A
9900	rs4226380	6	147158529	G	G	G	G	G	G
9901	rs6265387	6	147380171	G	G	A	A	A	G
9902	rs3686286	6	147668759	A	A	G	G	G	A
9903	rs3655959	6	147857079	G	G	C	C	C	C
9904	rs3695746	6	147858955	A	A	G	A	G	A
9905	rs6293645	6	148187893	G	G	G	G	G	G
9906	mCV23338838	6	149618644	A	A	G	G	G	G
9907	rs6281356	6	148403426	A	A	A	A	A	A
9908	rs3711088	6	148456534	G	G	G	C	G	C
9909	rs3677539	6	148742850	A	A	G	A	G	G
9910	rs3659280	6	148890307	G	G	A	G	A	G
9911	mCV23069133	6	147328303	A	A	C	C	C	A
9912	rs6392070	6	149072242	G	G	G	G	G	G
9913	rs3688686	7	2589779	A	A	A	G	A	G

9914	rs8259626	7	114261276	C	G	C	C	C	C
9915	rs8259625	7	114261271	G	G	G	G	G	G
9916	rs8259618	7	114259599	A	G	A	A	A	A
9917	rs3724540	7	94665666	G	A	G	A	G	A
9918	rs3658777	7	76791012	A	A	A	G	A	A
9919	rs3671943	7	90328805	C	C	C	A	C	C
9920	rs8269316	7	45411815	G	A	G	A	G	A
9921	rs3685570	7	106597989	A	G	G	A	G	A
9922	rs8269265	7	45392760	G	A	G	A	G	A
9923	rs3023159	7	106579694	A	G	G	A	G	A
9924	rs3696018	7	44701332	C	C	C	A	C	C
9925	rs3662508	7	16157466	A	G	G	A	A	G
9926	mCV24206490	7	128075	G	A	G	A	G	A
9927	rs3726290	7	100623211	G	G	A	G	A	G
9928	mCV24212075	7	10471645	A	T	T	A	NONE	T
9929	rs4232318	7	3334313	A	A	T	T	T	A
9930	rs4232319	7	3334454	A	A	A	A	A	A
9931	rs6292076	7	31782869	G	G	G	A	G	G
9932	rs8252653	7	26778269	A	A	A	A	A	A
9933	rs8252588	7	26778359	A	A	A	G	A	A
9934	rs8252567	7	26780733	A	A	A	C	A	A
9935	rs4226385	7	14972886	A	A	A	A	A	A
9936	rs6376323	7	43847133	G	G	G	G	G	G
9937	rs3700068	7	38982521	G	G	G	A	G	G
9938	rs3714915	7	12206182	C	C	C	A	C	C
9939	rs3714976	7	12206215	G	G	G	C	G	G
9940	rs8269138	7	11684039	G	G	G	G	G	G
9941	rs8269404	7	11735279	A	A	A	A	A	A
9942	mCV23733213	7	5115174	A	G	A	A	A	A
9943	mCV23738426	7	4906617	A	A	G	G	G	G
9944	rs3659033	7	10565513	G	G	G	A	G	G
9945	rs6384973	7	12916620	G	G	G	C	G	G
9946	rs6377546	7	43266814	G	G	G	G	G	G
9947	rs6369575	7	20865015	G	G	G	G	G	G
9948	rs3701600	7	25329604	A	A	G	G	G	G
9949	rs3659551	7	25331884	A	A	G	G	G	G
9950	mCV23738042	7	4919303	T	T	A	A	A	A
9951	mCV23044771	7	8573197	C	C	C	C	C	C
9952	rs6316571	7	5715334	A	A	A	A	A	A
9953	rs3658362	7	37176366	C	C	C	A	C	C
9954	rs6261745	7	42789176	G	G	G	G	G	G
9955	rs4226402	7	43063853	A	A	A	A	A	A
9956	rs6262171	7	24049333	G	G	G	G	G	G
9957	mCV22975338	7	7874146	A	A	A	C	A	A
9958	rs6377837	7	7208796	C	C	C	C	C	C
9959	rs6330715	7	7580376	C	C	C	A	C	C
9960	rs3675839	7	7713719	A	A	A	G	A	G
9961	rs3724197	7	7950972	A	G	A	G	A	A
9962	rs3711159	7	7979467	A	G	A	G	A	G
9963	rs3711200	7	7979500	C	A	C	A	C	A
9964	rs6295100	7	8141383	G	C	G	C	G	C
9965	rs3724594	7	8251928	G	A	G	A	G	A
9966	rs3709711	7	8286687	C	A	C	A	C	A
9967	rs4226418	7	13709013	C	C	C	C	A	C
9968	rs6365609	7	8618040	G	A	G	A	A	A
9969	rs6361247	7	9296488	C	A	C	A	C	A
9970	rs3705671	7	9631451	A	G	A	G	G	G
9971	rs3658600	7	9747120	G	A	G	A	A	A
9972	rs6309378	7	10517932	T	A	T	A	A	A
9973	rs3689218	7	10546409	G	A	G	G	A	A
9974	rs3726684	7	10554470	G	G	G	A	G	G
9975	rs8253990	7	10610894	C	C	C	C	C	C
9976	rs8254016	7	10614066	A	A	A	A	A	A
9977	rs8254031	7	10615404	A	A	A	A	A	A
9978	rs8254032	7	10615533	A	G	A	G	G	G
9979	mCV25415374	7	13168023	A	G	A	G	G	G
9980	rs3672220	7	10731932	G	A	G	G	A	G
9981	rs6162720	7	10883183	G	G	G	C	C	C
9982	rs4226441	7	43133188	A	G	A	G	G	G
9983	rs8252878	7	41145059	G	A	G	A	A	A
9984	rs3680778	7	7778948	G	A	G	A	A	A
9985	rs6188308	7	8134358	C	C	C	C	C	C
9986	rs6201966	7	8134650	C	A	C	A	A	A
9987	rs3724657	7	42376007	G	A	G	A	A	A

9988	rs3713852	7	42378081	A	G	A	G	G	G
9989	mCV25413535	7	14294911	G	A	A	A	A	A
9990	rs3692711	7	8431933	C	C	C	A	C	A
9991	mCV22517300	7	15336482	A	C	A	A	A	A
9992	mCV23045369	7	16001150	G	A	A	A	G	A
9993	rs6364065	7	24450985	G	A	G	A	A	A
9994	rs3697089	7	5489001	A	G	A	G	G	G
9995	rs3724397	7	12934995	G	A	G	A	A	A
9996	rs4226453	7	12878775	C	A	C	A	A	A
9997	mCV25406219	7	16848232	G	A	A	A	A	A
9998	rs3708913	7	13894574	A	C	C	C	C	C
9999	rs8258834	7	19867051	G	G	G	G	G	G
10000	rs8236562	7	14623274	G	G	G	G	G	G
10001	rs8257222	7	14620068	T	A	A	A	A	A
10002	rs6209066	7	14558752	G	G	G	G	G	G
10003	rs3671222	7	14641958	G	A	A	A	NONE	A
10004	rs8236590	7	14636260	A	A	A	A	A	A
10005	rs8236380	7	14484936	G	G	G	G	G	G
10006	mCV25220583	7	17994561	A	A	G	G	G	A
10007	rs8265514	7	15674000	C	C	C	C	C	C
10008	rs8236604	7	15683935	G	G	G	G	G	G
10009	rs8265547	7	15683992	A	A	A	A	A	A
10010	rs8265554	7	15687800	G	G	G	G	G	G
10011	rs6171548	7	16642471	G	G	G	G	G	G
10012	rs6164551	7	17546872	G	G	G	G	G	G
10013	rs4226499	7	17800749	G	G	A	G	G	G
10014	rs6288041	7	17718981	G	A	A	A	A	G
10015	rs3712359	7	17855508	G	G	C	G	G	G
10016	rs4226514	7	18196287	A	A	A	A	A	A
10017	rs4226520	7	18758740	A	A	G	G	A	G
10018	rs3688693	7	18305753	G	A	A	A	A	A
10019	rs6208019	7	18884852	G	A	G	G	A	G
10020	rs8261820	7	19399656	A	G	A	A	G	A
10021	rs3718762	7	19401297	G	A	G	G	A	G
10022	rs6388890	7	19436754	G	G	A	A	G	A
10023	rs3147878	7	19483951	G	G	A	A	G	A
10024	rs3147860	7	19496283	C	A	A	A	A	A
10025	rs8251849	7	19694692	C	C	C	C	G	C
10026	rs3662961	7	19697390	A	A	G	G	A	G
10027	rs8236618	7	19698519	A	A	A	A	C	A
10028	mCV23114039	7	24694450	G	G	G	G	A	G
10029	rs8240950	7	19856201	A	A	A	A	G	A
10030	rs8240966	7	19854223	T	T	T	T	A	T
10031	rs8240976	7	19843026	G	G	G	G	A	G
10032	rs6241757	7	20033189	G	G	G	G	G	G
10033	rs3725610	7	20070708	A	G	G	G	NONE	G
10034	mCV25384765	7	19820991	A	T	T	T	T	T
10035	mCV23212922	7	26160748	T	T	T	T	A	T
10036	mCV23088150	7	23070928	A	A	C	C	C	A
10037	rs6363390	7	21890869	G	G	A	A	G	A
10038	rs6389011	7	14122794	G	G	G	G	G	G
10039	rs3719311	7	22647606	A	A	T	T	T	T
10040	rs6248554	7	22937979	G	G	G	G	G	G
10041	rs3023117	7	22966455	G	A	A	A	A	G
10042	rs3722149	7	23171241	C	A	C	C	C	C
10043	rs3694031	7	23329413	G	A	A	A	G	A
10044	rs4226535	7	23818358	G	G	G	G	G	G
10045	rs4226545	7	23818922	G	G	G	G	G	G
10046	rs3091038	7	23460710	G	G	G	G	G	G
10047	rs3699705	7	23486681	G	A	A	A	A	A
10048	rs6293181	7	23658370	G	NONE	G	G	G	G
10049	rs3663763	7	23954426	A	T	A	A	A	A
10050	rs4226547	7	24197894	G	A	G	G	G	G
10051	rs6257814	7	24457426	G	A	G	G	A	G
10052	mCV23663629	7	26432125	A	A	A	C	C	A
10053	rs4136084	7	24771509	G	A	G	G	A	A
10054	rs6217275	7	24859883	C	C	C	G	G	C
10055	rs3724638	7	24907837	G	G	G	A	A	G
10056	rs3720735	7	25217824	G	G	G	A	G	G
10057	rs6300275	7	25290406	A	A	A	G	NONE	A
10058	rs3666900	7	25588748	A	A	A	G	G	A
10059	rs3665855	7	25731783	G	G	G	A	A	G
10060	rs3716814	7	25851129	G	G	G	A	A	G
10061	rs6176786	7	25920266	G	G	G	G	G	G

10062	mCV23658052	7	27361308	C	C	C	A	A	C
10063	rs6230901	7	26715575	A	A	A	G	G	A
10064	mCV25420884	7	27578282	G	G	G	A	A	G
10065	rs3663724	7	27093819	G	G	G	A	A	G
10066	rs3658393	7	27171690	G	G	G	A	A	G
10067	rs3670807	7	27871533	A	C	A	C	NONE	A
10068	rs3675080	7	27770401	A	G	A	G	G	G
10069	rs3668040	7	27737655	G	A	G	A	G	A
10070	rs3676967	7	28061226	G	A	G	G	A	A
10071	mCV23516314	7	33348088	G	G	G	G	A	G
10072	rs6381706	7	28315909	A	G	A	A	G	G
10073	rs6355384	7	28828489	A	A	A	A	T	A
10074	rs4232449	7	28755779	A	A	A	A	G	A
10075	rs4226555	7	29081555	A	A	A	A	A	A
10076	rs6226457	7	29974905	G	G	G	G	A	G
10077	rs4226558	7	30453904	G	G	G	G	G	G
10078	rs6316536	7	30898583	A	A	A	A	G	A
10079	rs8271756	7	31209052	G	G	G	G	A	G
10080	rs8271753	7	31207812	G	G	G	G	A	G
10081	rs8271728	7	31205367	G	G	G	G	A	G
10082	rs3682321	7	31203269	G	A	G	G	G	G
10083	mCV25286555	7	31890677	A	C	A	C	C	A
10084	mCV25286826	7	31891068	A	G	A	G	G	A
10085	mCV25287219	7	31891905	G	A	G	A	G	G
10086	mCV25287231	7	31893534	G	A	G	A	G	G
10087	mCV25287232	7	31894305	A	G	A	G	A	A
10088	mCV25287243	7	31894493	G	A	G	A	G	G
10089	mCV25287244	7	31894499	A	G	A	G	G	A
10090	mCV25287517	7	31895154	A	A	A	A	G	A
10091	mCV25287527	7	31895269	G	A	G	A	G	G
10092	mCV25287528	7	31895354	A	G	A	G	G	A
10093	mCV25287529	7	31895424	A	G	A	G	G	A
10094	mCV25287539	7	31895752	G	A	G	A	G	G
10095	mCV25287540	7	31895772	A	A	A	A	G	A
10096	mCV25287541	7	31895797	G	G	G	G	A	G
10097	mCV25287551	7	31895932	C	C	C	C	G	C
10098	mCV25287552	7	31895953	A	G	A	G	G	A
10099	mCV25287553	7	31896092	G	G	G	G	A	G
10100	mCV25287563	7	31896125	A	G	A	G	G	A
10101	mCV25287564	7	31896161	G	A	G	A	G	G
10102	rs4226572	7	31698029	A	C	A	A	A	A
10103	rs6201929	7	31937382	G	A	G	A	G	G
10104	rs3719256	7	31933809	G	A	G	A	G	G
10105	rs3689879	7	31923072	A	G	A	G	NONE	A
10106	rs8260952	7	31986114	A	A	A	A	G	A
10107	rs4226575	7	32223846	A	A	A	A	A	A
10108	rs6228386	7	32285087	C	C	C	A	C	A
10109	rs6313526	7	32302462	G	G	G	A	G	A
10110	rs3689409	7	32401134	A	A	A	G	A	G
10111	rs8269037	7	32805221	A	A	A	A	A	A
10112	rs8269035	7	32805005	G	G	G	G	G	G
10113	rs8269023	7	32801804	A	A	A	A	A	A
10114	rs3721550	7	33484199	G	A	G	A	G	A
10115	rs3703247	7	33404727	G	G	G	A	G	A
10116	rs3722844	7	33353344	C	C	C	A	C	A
10117	rs8255275	7	33574760	A	A	A	G	A	G
10118	rs6296138	7	33671479	T	T	T	A	T	A
10119	rs8260975	7	33835314	C	A	C	A	C	A
10120	rs8260982	7	33836281	G	G	G	G	G	G
10121	rs3659049	7	33842276	G	A	G	A	G	A
10122	rs6200877	7	33995268	G	G	G	G	G	G
10123	rs3024224	7	34137225	A	A	A	A	A	A
10124	rs3023119	7	34159147	A	A	A	A	A	A
10125	rs8266426	7	34160446	A	A	A	A	A	A
10126	rs6324063	7	34578745	G	G	G	G	G	G
10127	mCV24991351	7	35506119	C	C	C	C	NONE	G
10128	rs6226757	7	36239627	G	G	G	G	G	G
10129	rs3023125	7	36795665	A	A	A	A	A	A
10130	rs3680765	7	36839834	C	A	C	A	C	A
10131	rs3673884	7	36912381	A	G	A	G	A	A
10132	rs6222964	7	36970470	G	A	G	A	G	G
10133	rs3679637	7	37013786	G	A	G	A	G	G
10134	rs4226605	7	37074347	G	G	G	G	G	G
10135	rs4226609	7	37074460	A	A	A	A	A	A

10136	rs3676147	7	37235801	G	A	G	A	G	A
10137	rs3692261	7	37285251	A	G	A	G	A	G
10138	rs3687031	7	37354238	G	G	G	A	G	A
10139	rs6371491	7	37474244	G	G	G	G	G	G
10140	rs6158386	7	37488740	G	G	G	G	G	G
10141	rs3090660	7	37795990	C	C	C	A	C	C
10142	rs6273712	7	38022969	A	A	A	A	A	A
10143	rs3699938	7	38476481	G	A	G	G	G	A
10144	rs6222568	7	38661925	G	G	G	A	G	G
10145	rs3725186	7	38807273	A	A	A	G	A	A
10146	rs3703728	7	38867055	A	A	A	T	A	A
10147	rs3720603	7	38867495	A	A	A	G	A	A
10148	rs6221808	7	39051518	G	G	G	G	G	G
10149	rs6233515	7	39124686	G	G	G	G	G	G
10150	rs8266533	7	39319866	A	A	A	A	A	A
10151	rs8266566	7	39383361	T	T	T	T	T	T
10152	rs8266585	7	39401066	A	A	A	A	A	A
10153	rs3089213	7	39556820	G	G	G	G	G	G
10154	rs6200784	7	39727826	A	A	A	A	A	A
10155	rs3663846	7	39891494	A	A	A	G	A	A
10156	rs3672699	7	40657260	G	A	G	G	G	A
10157	rs3718641	7	41170581	G	A	G	A	G	A
10158	rs6178483	7	41300381	C	C	C	C	C	C
10159	rs3688876	7	41376403	A	A	A	C	A	A
10160	rs3713297	7	47634249	A	C	A	C	A	C
10161	rs3688386	7	42673653	A	G	A	G	A	G
10162	rs6209038	7	42765839	G	G	G	G	G	G
10163	rs6260795	7	42835290	C	C	C	C	C	C
10164	rs3688093	7	42836704	T	A	T	A	T	A
10165	rs4137599	7	43005593	G	G	G	A	G	G
10166	rs3717293	7	43537055	A	A	A	A	G	A
10167	rs6404048	7	43504831	A	A	A	A	A	A
10168	rs3663313	7	43718739	C	C	C	G	G	C
10169	rs6326195	7	44368276	G	G	G	G	G	G
10170	rs4136662	7	44384942	G	G	G	G	G	G
10171	rs3657400	7	44453486	A	A	A	A	G	A
10172	rs8260772	7	50196588	G	G	G	G	G	G
10173	rs3668134	7	44972563	A	A	A	A	A	A
10174	rs6323139	7	44930293	G	A	G	A	A	A
10175	rs8260829	7	44920691	G	G	G	G	G	G
10176	rs8260943	7	45323239	A	A	A	A	A	A
10177	rs8260944	7	45323186	G	G	G	G	G	G
10178	rs3693038	7	45306964	A	C	A	C	A	C
10179	rs3023132	7	45240565	C	C	C	C	A	C
10180	rs8269185	7	45191436	A	A	A	A	A	A
10181	rs6312804	7	45090611	A	A	A	A	A	A
10182	rs8260906	7	45332817	G	G	G	G	G	G
10183	rs3090914	7	45962289	A	A	A	A	A	A
10184	rs6410460	7	45968201	A	A	A	A	A	A
10185	rs3705089	7	46323059	C	G	C	G	C	G
10186	rs3664823	7	46418664	A	G	A	G	G	G
10187	mCV23672419	7	45173762	A	C	A	A	A	C
10188	rs6217158	7	46552979	C	C	C	C	C	C
10189	rs3715948	7	47451904	A	A	A	A	A	A
10190	rs6361518	7	47891718	C	C	C	C	A	C
10191	rs3672223	7	47999587	A	G	A	G	A	G
10192	rs6301305	7	48038664	A	A	A	A	T	A
10193	mCV23423763	7	47949709	C	A	C	A	C	A
10194	rs3697532	7	48569362	A	A	A	A	A	A
10195	rs6204175	7	48906853	G	G	G	G	A	G
10196	rs3723625	7	49769236	A	A	A	A	G	A
10197	rs6166250	7	50295092	A	A	A	A	G	A
10198	mCV24979486	7	56117427	T	T	T	T	A	T
10199	rs6345670	7	50681480	A	A	A	A	G	A
10200	rs3679779	7	51392430	G	A	G	A	A	A
10201	rs3719301	7	51394274	C	A	C	A	C	A
10202	rs3717935	7	51547846	G	G	G	A	G	G
10203	rs3717846	7	51547793	C	C	C	A	C	C
10204	rs3714908	7	51544526	A	A	A	G	G	A
10205	rs3726993	7	51700275	G	G	G	A	G	G
10206	rs4226632	7	51787025	G	G	G	G	G	G
10207	mCV23752700	7	49919613	G	G	G	G	A	G
10208	rs6336159	7	52270784	G	G	G	G	G	G
10209	rs4226649	7	53081232	A	A	A	A	G	A

10210	rs4226655	7	53120682	A	A	A	A	G	A
10211	rs3088794	7	53227218	A	A	A	G	G	A
10212	rs3667815	7	53257383	A	A	A	G	G	A
10213	rs6160140	7	53312532	A	A	A	G	A	G
10214	rs3090723	7	53541409	C	C	C	C	C	C
10215	rs3713031	7	59065999	A	A	A	G	G	G
10216	rs4226680	7	53745747	A	A	A	A	A	A
10217	rs3706446	7	54666576	G	G	G	A	A	G
10218	rs3667441	7	54664806	G	G	G	A	A	G
10219	rs3675028	7	54549581	G	G	G	A	A	G
10220	rs6287797	7	54500474	G	G	G	A	A	G
10221	rs3698065	7	55148835	A	A	A	G	A	A
10222	rs6193753	7	55192242	G	G	G	G	G	G
10223	rs3023145	7	55565910	C	C	C	C	A	A
10224	rs3705155	7	55557090	A	A	A	C	C	C
10225	rs3667208	7	55522049	G	G	G	A	A	G
10226	rs8239069	7	55772977	A	A	A	A	A	A
10227	rs6266745	7	55890385	A	A	A	A	A	A
10228	rs3707769	7	55944997	G	G	G	A	G	G
10229	rs3706258	7	56190774	A	A	A	T	T	T
10230	rs3695904	7	56434818	G	G	G	A	G	G
10231	mCV23318496	7	55001384	T	T	T	T	T	T
10232	rs3657147	7	57152088	G	G	G	A	A	A
10233	rs3723790	7	57204266	G	G	G	A	G	G
10234	rs6167203	7	57205349	A	A	A	A	A	A
10235	rs3683475	7	57615070	A	A	A	G	A	A
10236	rs3710183	7	57749690	A	A	A	G	A	A
10237	rs3707330	7	57739844	G	G	G	A	G	G
10238	rs6224381	7	58264944	A	A	A	A	A	A
10239	rs3661581	7	58409321	G	G	G	A	A	G
10240	rs3088592	7	58628840	A	A	A	A	A	A
10241	rs3660824	7	58609412	A	A	A	G	G	G
10242	rs3691291	7	58916534	G	G	G	A	A	G
10243	rs3691975	7	58916652	G	G	G	A	A	G
10244	rs6327571	7	59006294	A	A	A	G	G	G
10245	mCV23006400	7	57525735	A	A	A	G	G	G
10246	rs6236572	7	59624234	G	G	G	G	G	G
10247	rs3676254	7	59722158	A	A	A	G	A	A
10248	rs3686406	7	59735970	T	T	T	A	T	T
10249	rs3668546	7	59744844	G	G	G	A	G	G
10250	rs4226685	7	60351780	A	A	A	A	A	G
10251	rs6405925	7	60564586	A	A	A	A	A	A
10252	rs3716002	7	61171781	A	A	A	G	G	G
10253	mCV25443682	7	59448296	G	G	G	G	A	G
10254	rs8268371	7	61353940	G	C	G	C	G	C
10255	rs8268494	7	61400766	A	T	A	T	A	A
10256	rs8268322	7	61408291	A	A	A	C	A	A
10257	rs3677657	7	61680107	A	G	A	G	A	A
10258	rs3687109	7	61862304	T	A	T	A	T	T
10259	rs6337903	7	62344833	G	G	G	G	G	G
10260	rs3718836	7	62508432	A	A	A	G	A	G
10261	mCV25303361	7	60653332	A	A	A	C	C	C
10262	rs6224142	7	62625901	A	A	A	A	A	A
10263	rs3684797	7	62813289	A	G	A	G	A	G
10264	rs3671471	7	63497493	T	T	T	A	T	T
10265	rs6371577	7	63837740	G	G	G	G	G	G
10266	rs6251120	7	64691089	A	A	A	A	A	A
10267	rs3720746	7	65283918	A	C	A	C	A	C
10268	rs6394640	7	65357595	G	G	G	G	G	G
10269	rs6297524	7	66135318	A	A	A	A	A	A
10270	rs8247788	7	66215986	G	G	G	A	G	G
10271	rs8247796	7	66215809	A	A	A	A	A	A
10272	rs8247827	7	66208872	C	C	C	C	C	C
10273	rs8247824	7	66208579	G	G	G	A	G	G
10274	rs8279749	7	66906419	A	A	A	A	A	A
10275	rs4226715	7	67346534	A	A	A	G	A	A
10276	rs6209569	7	67191920	A	A	A	A	A	A
10277	rs4226716	7	72742235	G	G	G	G	G	G
10278	rs4226722	7	72763296	A	C	A	C	A	C
10279	rs8236681	7	67465555	G	G	G	G	G	G
10280	rs8250183	7	67465864	G	G	G	G	G	G
10281	rs8248617	7	67468317	A	G	A	A	A	G
10282	rs8248576	7	67546520	C	C	C	A	C	C
10283	rs8248449	7	67583104	G	G	G	G	G	G

10284	rs8248433	7	67585590	G	A	G	G	G	A
10285	rs3700384	7	67715592	A	G	A	G	A	G
10286	rs3663343	7	68631303	G	G	G	A	G	G
10287	rs4139879	7	68687219	A	A	A	T	A	A
10288	rs3660529	7	69074604	G	G	G	A	G	G
10289	rs3718695	7	69074357	A	A	A	G	A	A
10290	rs4226744	7	69059985	G	G	G	G	G	G
10291	rs6360431	7	69166832	A	A	A	G	A	A
10292	mCV23906089	7	67307193	G	A	G	A	G	A
10293	mCV23906088	7	67307750	A	A	A	G	A	A
10294	mCV23906087	7	67309012	A	C	A	C	A	C
10295	mCV23906080	7	67309511	A	G	A	G	A	G
10296	mCV23906079	7	74630576	G	C	G	C	G	C
10297	mCV23906071	7	67310756	A	G	A	G	A	G
10298	mCV23906070	7	67311154	C	C	C	C	C	C
10299	mCV23906062	7	67311645	C	A	C	A	C	A
10300	mCV23906061	7	67311825	G	A	G	G	G	A
10301	mCV23906060	7	67312113	A	G	A	A	A	G
10302	mCV23906049	7	67312740	A	G	A	G	A	G
10303	rs3684222	7	69621504	A	A	A	G	A/G	A
10304	rs3690268	7	69625363	A	A	A	G	A	A
10305	mCV25289592	7	67863784	A	G	A	A	A	G
10306	rs3688593	7	70183689	G	G	G	C	G	G
10307	rs6285160	7	70258168	A	A	A	A	A	A
10308	rs3686742	7	70288715	A	G	A	G	A	G
10309	rs3090916	7	70433880	A	A	A	A	A	A
10310	rs3712640	7	70508689	G	A	G	G	G	A
10311	rs6317673	7	70922273	A	A	A	A	A	A
10312	rs3663050	7	71419890	A	C	A	C	A	C
10313	rs3664330	7	71420141	A	G	A	G	A	G
10314	rs4226752	7	71715908	A	A	A	A	A	A
10315	rs3656832	7	72176553	A	A	A	T	A	A
10316	rs6327115	7	72178197	C	C	C	A	C	C
10317	rs6272937	7	72466085	A	A	A	A	A	A
10318	rs3696768	7	72703334	G	A	G	A	G	A
10319	rs3672290	7	73483259	C	G	C	G	C	G
10320	rs6391093	7	73788835	A	A	A	A	A	A
10321	rs3664145	7	73737208	T	A	T	A	T	A
10322	rs6394492	7	74390340	C	C	C	A	C	C
10323	rs4226759	7	74555364	A	A	A	A	A	A
10324	rs6277853	7	75060063	G	G	G	G	G	G
10325	rs3663323	7	75481063	C	C	C	G	C	C
10326	mCV23442976	7	73239811	A	G	A	A	A	G
10327	rs6276442	7	75757289	A	A	A	A	A	A
10328	rs3671486	7	76142320	T	A	T	A	T	A
10329	rs3654681	7	76327531	C	A	C	A	C	A
10330	mCV22985833	7	74279063	A	C	A	C	A	C
10331	rs3711806	7	76616362	A	A	A	G	A	A
10332	mCV23669669	7	74768852	A	C	A	A	A	C
10333	rs6216414	7	76990382	A	A	A	A	A	A
10334	rs3676511	7	77017488	G	G	G	A	G	G
10335	rs4226768	7	77680859	A	A	A	A	A	A
10336	rs3715742	7	77450062	A	G	G	A	G	G
10337	rs3089116	7	77985485	G	A	A	A	A	A
10338	rs6212025	7	78027228	A	A	A	A	A	A
10339	rs3088819	7	78387137	A	A	A	A	A	A
10340	rs6353096	7	78659108	G	G	G	G	G	G
10341	rs3663374	7	78686554	T	T	T	A	T	T
10342	rs3670309	7	78761356	C	C	A	C	C	C
10343	rs3703127	7	79213917	G	A	A	A	A	A
10344	rs3672893	7	79408640	G	G	G	A	G	G
10345	rs3680261	7	83452910	G	G	A	G	A	G
10346	rs6297335	7	79592928	A	A	A	A	A	A
10347	rs4139557	7	79642492	A	G	G	A	G	G
10348	rs6346458	7	79836880	A	A	A	A	A	A
10349	rs4226772	7	79964718	G	G	G	G	G	G
10350	rs4226771	7	79964860	A	A	A	A	A	A
10351	rs3720958	7	80403000	G	A	A	G	A	G
10352	rs3683030	7	80319797	C	G	G	C	G	C
10353	rs3719097	7	80623111	A	C	C	C	C	C
10354	rs6321517	7	80887455	A	G	G	A	G	G
10355	rs3714287	7	81068640	C	G	G	C	G	G
10356	rs3691805	7	81500798	C	C	C	A	C	C
10357	rs6293103	7	82058809	A	A	A	G	A	A

10358	rs6182508	7	82204546	A	A	A	A	A	A
10359	rs3704458	7	83064152	A	A	A	G	A	A
10360	rs6202435	7	83149511	G	G	G	G	G	G
10361	rs3726013	7	83212590	G	A	A	A	A	A
10362	rs3717689	7	83214984	A	G	G	A	G	G
10363	rs3699086	7	83322675	C	C	A	C	A	C
10364	rs6385816	7	83795009	G	A	A	G	A	A
10365	rs3023151	7	83894345	G	G	A	G	A	G
10366	rs6394612	7	83973375	A	A	A	G	A	A
10367	rs3692018	7	84013500	A	A	A	G	A	A
10368	rs3675638	7	84056040	G	G	C	G	C	G
10369	rs3657451	7	84225112	T	T	T	A	T	T
10370	rs4226786	7	84282400	C	C	C	C	C	C
10371	rs4226793	7	84282551	A	A	A	A	A	A
10372	rs4226794	7	84282609	C	C	A	C	A	C
10373	rs4226797	7	84766938	G	G	G	G	G	G
10374	rs4226798	7	84766944	G	G	G	G	G	G
10375	rs3654376	7	85138024	T	T	A	A	A	T
10376	rs6276149	7	85669930	G	G	A	G	A	G
10377	rs8281437	7	85350939	G	G	A	G	A	G
10378	rs3660677	7	85370237	A	A	G	G	G	A
10379	mCV27585803	7	83376279	A	A	G	A	G	A
10380	rs8281442	7	85351767	A	A	C	A	C	A
10381	rs8281458	7	85355566	G	G	A	A	A	G
10382	rs4226811	7	85443076	C	C	C	C	C	C
10383	rs4226812	7	85443106	A	A	C	A	C	A
10384	rs3707067	7	85992410	A	A	C	C	C	A
10385	rs6405344	7	86185334	G	G	G	A	G	G
10386	rs6227780	7	86699867	G	G	A	G	A	G
10387	rs3679473	7	87088848	A	A	G	G	G	A
10388	rs3713440	7	87022719	G	G	A	G	A	G
10389	rs6245036	7	86994794	G	G	G	A	G	G
10390	rs3709784	7	86924505	T	T	A	T	A	T
10391	rs3708313	7	87777594	G	G	A	A	A	G
10392	rs4226829	7	87825318	C	C	A	C	A	C
10393	rs4226832	7	87825502	G	G	A	G	A	G
10394	rs3714598	7	87990927	A	A	G	A	G	A
10395	rs3656411	7	88568797	G	G	A	G	A	G
10396	rs6279641	7	88999444	A	A	G	G	G	A
10397	rs3713052	7	89115174	C	C	C	A	C	C
10398	rs3091124	7	89167841	A	A	A	A	A	A
10399	rs6213885	7	89217257	G	G	A	G	A	G
10400	rs6347862	7	94336605	G	G	G	A	G	G
10401	rs3684618	7	89758909	A	A	A	G	A	A
10402	rs3667300	7	89790549	A	A	A	G	A	A
10403	mCV24825272	7	84922277	G	G	A	G	A	G
10404	mCV24825279	7	84922330	G	G	A	G	A	G
10405	mCV24825281	7	84922660	A	A	G	G	G	A
10406	mCV24825288	7	84922975	A	A	G	G	G	A
10407	mCV24825289	7	84924084	G	G	A	G	A	G
10408	mCV24825290	7	84924311	C	C	G	C	G	C
10409	mCV24825297	7	84924474	A	A	C	A	C	A
10410	mCV24825310	7	84925602	G	G	C	G	C	G
10411	mCV24825311	7	84925623	G	G	A	G	A	G
10412	mCV24825321	7	84925751	G	G	A	G	A	G
10413	mCV24825322	7	84925793	A	A	C	A	A/C	A
10414	mCV24825323	7	84926399	G	G	A	G	A	G
10415	mCV24825333	7	84926478	A	A	G	G	G	A
10416	mCV24825334	7	84927993	A	A	A	G	A	A
10417	mCV24825335	7	84928043	A	A	NONE	A	G	A
10418	mCV24825606	7	84928048	G	G	A	G	A	G
10419	mCV24825629	7	84932111	A	A	A	G	A	A
10420	mCV24825630	7	84932171	A	A	C	C	C	A
10421	mCV24825631	7	84932211	T	T	A	T	A	T
10422	mCV24825642	7	84932432	G	G	A	A	A	G
10423	mCV24825652	7	84932564	G	G	A	G	A	G
10424	mCV24825665	7	84933676	G	G	A	G	A	G
10425	mCV24825666	7	84934197	G	G	A	G	A	G
10426	mCV24825676	7	84934640	A	A	G	A	G	A
10427	mCV24825677	7	84934673	A	A	G	G	G	A
10428	mCV24825689	7	84935032	G	G	A	G	A	G
10429	mCV24825950	7	84935306	G	G	A	G	A	G
10430	mCV24825962	7	84935416	G	G	A	G	A	G
10431	mCV24825973	7	84935644	A	A	G	G	G	A

10432	mCV24825985	7	84937818	G	G	A	G	A	G
10433	mCV24825986	7	84937960	G	G	A	G	A	G
10434	mCV24825996	7	84938091	G	G	A	G	A	G
10435	mCV24825997	7	84938175	A	A	C	A	C	A
10436	mCV24826010	7	84939101	A	A	A/G	A	A/G	A
10437	mCV24826017	7	84939447	A	A	C	A	C	A
10438	mCV24826018	7	84939574	A	A	G	A	G	A
10439	mCV24826026	7	84939800	C	C	A	C	A	C
10440	mCV24826034	7	84939973	A	A	G	G	G	A
10441	mCV24826035	7	84940077	G	G	A	A	A	G
10442	mCV24826036	7	84940787	A	A	A	T	A	A
10443	mCV24165697	7	95036671	G	A	G	A	G	A
10444	mCV22563662	7	84944200	G	G	A	G	A	G
10445	mCV22563661	7	84944247	G	G	A	G	A	G
10446	mCV22563660	7	84944292	T	T	A	T	A	T
10447	mCV22563659	7	84944294	A	A	A	G	A	A
10448	mCV22563649	7	95039100	A	A	G	A	G	A
10449	mCV22563647	7	84945510	A	A	T	A	T	A
10450	mCV22563635	7	84945692	C	C	A	C	A	C
10451	mCV22563625	7	84945725	A	A	C	A	C	A
10452	mCV22563624	7	95039608	G	G	A	G	A	G
10453	mCV23132683	7	85277658	G	G	A	A	A	G
10454	mCV23539175	7	85278118	A	A	C	C	C	A
10455	mCV23539176	7	85278156	A	A	G	G	G	A
10456	mCV23539187	7	85278517	A	A	G	G	G	A
10457	mCV23539188	7	85278547	A	A	C	C	C	A
10458	mCV23539189	7	85278600	G	G	G	A	G	G
10459	mCV23539199	7	85281373	A	A	G	G	G	A
10460	mCV23539208	7	85284658	G	G	A	G	A	G
10461	mCV23539227	7	85286994	A	A	G	G	G	A
10462	mCV23539496	7	85287160	C	C	C	A	C	C
10463	mCV23539497	7	85287214	G	G	A	G	A	G
10464	mCV23539498	7	85287321	G	G	A	G	A	G
10465	mCV23539505	7	85287780	A	A	C	C	C	A
10466	mCV23539514	7	85288260	A	A	G	G	G	A
10467	mCV23539515	7	85288409	A	A	G	A	G	A
10468	mCV23539516	7	85288504	T	T	T	A	T	T
10469	mCV23539523	7	85288611	A	A	G	A	G	A
10470	mCV23539524	7	85288759	C	C	A	C	A	C
10471	mCV23539532	7	85289261	A	A	G	G	G	A
10472	mCV23539533	7	85289511	A	A	G	G	G	A
10473	mCV23539534	7	85296104	A	A	G	G	G	A
10474	mCV23539541	7	85296232	A	A	G	G	G	A
10475	mCV23539542	7	85296293	G	G	A	A	A	G
10476	mCV23539543	7	85296365	G	G	A	A	A	G
10477	mCV23539550	7	85296411	A	A	G	G	G	A
10478	mCV23539551	7	85296597	T	T	T	A	T	T
10479	mCV23539559	7	85297131	G	G	A	A	A	G
10480	mCV23539560	7	85298005	G	G	G	A	G	G
10481	mCV23539561	7	85298257	G	G	A	G	A	G
10482	mCV23539571	7	85300125	G	G	A	A	A	G
10483	mCV23539844	7	85300312	G	G	A	A	A	G
10484	mCV23539845	7	85300614	G	G	A	A	A	G
10485	mCV23539863	7	85304106	G	G	A	A	A	G
10486	mCV23539864	7	85304238	G	G	A	G	A	G
10487	mCV23539872	7	85304542	A	A	C	A	C	A
10488	mCV23539873	7	85305872	A	A	G	G	G	A
10489	mCV23539881	7	85307021	G	G	A	G	A	G
10490	mCV23539882	7	85307552	G	G	A	G	A	G
10491	mCV23539898	7	85308219	A	A	A	G	A	A
10492	mCV23539916	7	85308893	A	A	G	G	G	A
10493	mCV23539917	7	85309085	A	A	G	G	G	A
10494	mCV23540186	7	85311738	A	A	G	G	G	A
10495	mCV23540188	7	85315477	G	G	A	A	A	G
10496	mCV23540195	7	85315603	A	A	G	G	G	A
10497	mCV23540206	7	85316087	G	G	A	A	A	G
10498	mCV23540214	7	85316411	A	A	G	G	G	A
10499	mCV23540215	7	85318856	A	A	G	G	G	A
10500	mCV23540216	7	85319010	G	G	G	A	G	G
10501	mCV23540223	7	85319189	G	G	A	A	A	G
10502	mCV23540224	7	85319223	A	A	G	G	G	A
10503	mCV24168434	7	85320087	A	A	G	G	G	A
10504	mCV24168441	7	85320112	G	G	A	G	A	G
10505	mCV24168442	7	85320113	A	A	G	A	G	A

10506	mCV24168443	7	85320568	C	C	A	C	A	C
10507	mCV24168450	7	85320645	A	A	G	A	G	A
10508	mCV24168451	7	85320758	C	C	A	C	A	C
10509	mCV24168452	7	85320916	T	T	A	T	A	T
10510	mCV24168459	7	85320984	G	G	A	G	A	G
10511	mCV24168468	7	85321131	C	C	A	C	A	C
10512	mCV24168470	7	85321396	A	A	G	A	G	A
10513	mCV24168478	7	85322344	A	A	G	A	G	A
10514	mCV24168479	7	85322662	A	A	C	A	C	A
10515	mCV24168486	7	85322705	A	A	G	G	G	A
10516	mCV24168799	7	85325167	C	C	C	C	C	C
10517	mCV24168800	7	85325204	A	A	A	G	A	A
10518	mCV24168812	7	85325578	G	G	A	G	A	G
10519	mCV24168822	7	85325602	A	A	G	A	G	A
10520	mCV24168823	7	85325675	G	G	G	A	G	G
10521	mCV24168824	7	85325848	A	A	A	C	A	A
10522	mCV24168832	7	85327381	G	G	A	A	A	G
10523	mCV24168837	7	85327738	G	G	A	G	A	G
10524	mCV24168838	7	85328672	A	A	G	A	G	A
10525	mCV24168839	7	85329200	A	A	G	G	G	A
10526	rs3691784	7	91117567	A	A	A	T	A	A
10527	rs3703889	7	91141265	G	G	G	C	G	G
10528	rs6156210	7	91148882	C	C	C	C	C	C
10529	rs3716529	7	91226327	G	G	G	A	G	G
10530	rs3717789	7	91226499	G	G	G	A	G	G
10531	rs3673390	7	91243830	G	G	G	C	G	G
10532	rs3717027	7	91403573	G	G	G	A	G	G
10533	rs3662937	7	91751530	G	G	G	A	G	G
10534	rs6340833	7	91928124	G	G	G	A	G	G
10535	rs3699690	7	91917846	A	A	A	G	A	A
10536	rs3659348	7	91909237	A	A	A	G	A	A
10537	mCV23558673	7	87087133	A	A	G	A	G	A
10538	mCV23558677	7	87087210	G	G	G	A	G	G
10539	mCV23558678	7	87087278	A	A	A	G	A	A
10540	mCV23558679	7	87087308	T	T	A	T	A	T
10541	mCV23558684	7	87087869	A	A	C	A	C	A
10542	mCV23558950	7	87087940	G	G	A	G	A	G
10543	mCV23558951	7	87088172	A	A	G	A	G	A
10544	mCV23558952	7	87088220	A	A	G	A	G	A
10545	mCV23558956	7	87088276	C	C	G	C	G	C
10546	mCV23558957	7	87088644	G	G	A	G	A	G
10547	mCV23558958	7	87088745	A	A	G	G	G	A
10548	mCV23558968	7	87089503	G	G	A	G	A	G
10549	mCV23558969	7	87089859	G	G	A	G	A	G
10550	mCV23558970	7	87090707	G	G	A	G	A	G
10551	mCV23558974	7	87090730	A	A	G	A	G	A
10552	mCV23558975	7	87090875	T	T	A	T	A	T
10553	mCV23558976	7	87090973	G	G	C	G	C	G
10554	mCV23558980	7	87091032	A	A	G	NONE	G	A
10555	mCV23559034	7	87098130	A	A	G	A	G	A
10556	mCV23559035	7	87098261	A	A	G	A	G	A
10557	mCV24183719	7	87102837	G	G	G	A	G	G
10558	mCV24183720	7	87102980	A	A	G	G	G	A
10559	mCV24183721	7	87103326	G	G	A	G	A	G
10560	mCV24183723	7	87103467	G	G	A	G	A	G
10561	mCV24183724	7	87103719	A	A	C	A	C	A
10562	mCV24183725	7	87103964	T	T	A	T	A	T
10563	mCV24184116	7	87104337	G	G	A	A	A	G
10564	mCV24184399	7	87105118	A	A	G	G	G	A
10565	rs3090793	7	92868435	A	A	A	A	A	A
10566	rs6199622	7	93011140	A	A	A	A	A	A
10567	rs3719495	7	93674602	G	G	G	A	G	A
10568	mCV23569777	7	88604052	A	A	G	A	G	A
10569	rs6229068	7	93784113	A	A	A	G	A	G
10570	rs3673653	7	94344049	G	G	G	A	G	A
10571	rs3711721	7	94492631	G	G	G	A	G	A
10572	rs3657164	7	94495926	G	G	G	A	G	A
10573	rs4137246	7	94502140	A	A	A	G	A	G
10574	rs6386601	7	94870318	A	C	A	C	A	C
10575	rs3718785	7	94910264	A	G	A	G	A	G
10576	rs6381106	7	95730393	A	A	A	A	A	A
10577	rs6318508	7	96484614	C	C	C	C	C	C
10578	rs6289536	7	97189861	G	G	G	G	G	G
10579	rs3686975	7	97250105	A	A	A	G	A	A

10580	rs6349890	7	98034092	A	C	A	A	A	A
10581	rs3089162	7	98208847	G	G	G	G	G	G
10582	mCV24704879	7	103332415	A	A	A	T	A	T
10583	rs6319753	7	98979613	A	A	A	A	A	A
10584	rs3726501	7	99098855	A	A	A	G	A	A
10585	rs3670388	7	99099446	A	A	A	C	A	A
10586	rs3670446	7	99222217	A	A	A	G	A	A
10587	rs3708100	7	99278326	A	A	A	G	A	A
10588	rs3711843	7	99278945	G	G	G	C	G	G
10589	rs6325331	7	99485551	G	G	G	G	G	G
10590	rs3679005	7	99595293	A	A	A	G	A	A
10591	mCV24718258	7	94833948	G	A	G	A	G	A
10592	mCV24718259	7	94834198	G	C	G	C	G	C
10593	mCV24718282	7	94837101	T	A	T	A	T	A
10594	mCV24718283	7	94837294	A	T	A	T	A	T
10595	mCV24718294	7	94837572	G	A	G	A	G	A
10596	mCV24718295	7	94837713	C	G	C	G	C	G
10597	mCV24718306	7	94839634	A	T	A	T	A	T
10598	mCV24718307	7	94839720	G	A	G	A	G	A
10599	mCV24718491	7	94841921	G	A	G	A	G	A
10600	mCV24718492	7	94844400	A	G	A	G	A	G
10601	mCV24718493	7	94844406	A	C	A	C	A	C
10602	mCV24718516	7	94844959	C	A	C	A	C	A
10603	mCV24718527	7	94845074	A	G	A	G	A	G
10604	mCV24718528	7	94845099	A	G	A	G	A	G
10605	mCV24718538	7	94845231	G	A	G	A	G	A
10606	mCV24718539	7	94848734	G	A	G	A	G	A
10607	mCV24718540	7	94849280	T	A	T	A	T	A
10608	mCV24718551	7	104560280	C	A	C	A	C	A
10609	mCV24718552	7	94852300	C	G	C	G	C	G
10610	mCV24718562	7	94852551	G	C	G	C	G	C
10611	mCV24718564	7	94855883	C	A	C	A	C	A
10612	mCV24718835	7	94856305	A	G	A	G	A	G
10613	mCV24718837	7	94857218	A	G	A	G	A	G
10614	mCV24718847	7	94857478	A	G	A	G	A	G
10615	mCV24718848	7	94857545	A	G	A	G	A	G
10616	mCV24718849	7	94858207	G	A	G	A	NONE	A
10617	rs3674465	7	100006800	A	A	A	G	A	G
10618	mCV23009137	7	95354456	A	A	A	A	T	A
10619	rs6280792	7	100322193	A	A	A	G	A	G
10620	rs3698711	7	100246522	A	A	G	G	G	G
10621	rs4226867	7	100841896	G	G	G	G	G	G
10622	rs4226866	7	100841770	C	C	A	C	A	C
10623	rs3691339	7	100823708	G	G	A	G	A	G
10624	rs3724711	7	101325771	A	A	G	G	G	G
10625	rs3657482	7	101345671	C	C	A	C	A	C
10626	rs3023155	7	101359397	A	A	G	A	G	A
10627	mCV27572454	7	96546544	G	A	G	G	G	G
10628	rs6194926	7	101647757	G	A	A	G	A	G
10629	rs3675993	7	102430244	A	G	A	G	A	A
10630	rs3658524	7	102429588	G	A	G	A	G	G
10631	rs6160824	7	102478563	A	G	A	G	A	G
10632	rs3705945	7	102839081	G	C	G	C	G	C
10633	rs3691303	7	102929987	A	G	A	G	A	G
10634	rs6372710	7	103158168	A	G	A	G	A	G
10635	rs3667146	7	103291992	A	G	A	G	A	G
10636	rs3669014	7	103292309	G	A	G	A	G	A
10637	rs4139261	7	103315827	G	A	G	A	G	A
10638	rs4226868	7	103406388	C	C	C	C	C	C
10639	rs3656074	7	103416924	G	A	G	A	G	A
10640	mCV24724415	7	98451431	G	G	G	G	G	G
10641	rs3089475	7	103711221	A	G	G	G	G	G
10642	rs6382230	7	103730679	G	C	C	C	C	C
10643	rs3690155	7	103972292	G	A	G	A	G	A
10644	rs3676820	7	104478024	A	G	G	G	G	G
10645	rs6200962	7	104427524	A	G	G	G	G	G
10646	rs3090098	7	104568134	C	A	A	A	A	A
10647	rs3721457	7	104981763	A	G	G	G	G	G
10648	rs3725542	7	105166893	G	A	G	A	G	A
10649	rs4226870	7	105414280	G	A	G	A	G	A
10650	mCV24766407	7	100596619	T	T	A	A	A	A
10651	rs6277857	7	105648788	G	A	G	A	G	A
10652	rs6305308	7	105951762	G	A	G	A	G	A
10653	rs4226872	7	106033606	C	C	C	C	C	C

10654	rs3693791	7	106277306	A	G	A	G	A	G
10655	rs3665698	7	106540390	G	A	G	G	G	G
10656	rs6241342	7	106479164	A	G	G	A	G	A
10657	rs6193654	7	106470742	G	A	G	A	G	A
10658	rs3722112	7	106465439	A	C	A	C	A	C
10659	rs6366212	7	106414059	A	G	A	G	A	G
10660	rs3671145	7	106768482	G	A	G	G	G	G
10661	rs2020676	7	107083294	G	G	G	G	G	G
10662	rs3709679	7	107424656	C	A	A	C	A	C
10663	mCV24779699	7	102696760	A	G	A	G	A	G
10664	rs6322316	7	107988664	G	G	G	A	G	G
10665	rs6258892	7	108969067	A	T	A	A	A	A
10666	rs3666152	7	109411567	G	A	A	A	A	A
10667	rs3666823	7	109411717	G	A	A	A	A	A
10668	rs3089537	7	109861519	T	T	T	T	T	T
10669	rs3682921	7	109867352	G	G	A	A	A	G
10670	rs6275579	7	111285662	G	A	G	G	G	A
10671	mCV24823383	7	106783744	A	G	G	A	G	A
10672	mCV24823391	7	106784111	G	G	A	G	A	G
10673	mCV24823392	7	106784312	G	G	A	G	A	G
10674	mCV24823393	7	106784440	A	A	G	A	G	A
10675	mCV24823403	7	106784539	G	G	A	G	A	G
10676	mCV24823404	7	106784642	A	A	G	A	G	A
10677	mCV24823415	7	106784744	A	A	G	A	G	A
10678	mCV24823416	7	106786834	A	G	G	A	G	A
10679	mCV24823427	7	106786947	A	A	C	A	C	A
10680	mCV24823428	7	106787047	A	A	G	A	G	A
10681	mCV24823429	7	106787204	A	A	C	A	C	A
10682	mCV24823712	7	106787546	G	G	C	G	C	G
10683	mCV24823713	7	106787547	G	G	A	G	A	G
10684	mCV24823735	7	106788936	A	G	G	A	G	A
10685	mCV24823736	7	106788956	A	A	G	A	G	A
10686	mCV24823747	7	106789457	A	A	G	A	G	A
10687	mCV24823748	7	106789501	G	G	A	G	A	G
10688	mCV24823749	7	106789757	A	A	G	A	G	A
10689	mCV24823759	7	106789827	G	G	A	G	A	G
10690	mCV24823760	7	106790034	G	G	A	G	A	G
10691	mCV24823761	7	106790084	A	A	G	A	G	A
10692	mCV24823772	7	106792624	A	C	C	A	C	A
10693	mCV24824044	7	106792920	A	G	G	A	G	A
10694	mCV24824045	7	106793340	C	C	A	C	A	C
10695	mCV24824046	7	106793487	G	G	A	G	A	G
10696	mCV24824070	7	106794019	G	G	A	G	A	G
10697	mCV24824080	7	106794027	G	G	A	G	A	G
10698	mCV24824081	7	106794441	A	A	G	A	G	A
10699	mCV24824082	7	106794674	G	G	A	G	A	G
10700	mCV24824092	7	106794720	G	G	C	G	C	G
10701	mCV24824093	7	106795098	G	G	C	G	C	G
10702	mCV24824094	7	106795174	A	G	A	A	A	A
10703	mCV24824104	7	106795176	C	C	A	C	A	C
10704	mCV24824400	7	106797003	G	G	A	G	A	G
10705	mCV24824401	7	106797593	A	A	G	A	G	A
10706	mCV24824402	7	106797929	G	G	A	G	A	G
10707	mCV24824412	7	106798637	A	A	G	A	G	A
10708	mCV24824413	7	106798841	A	A	C	A	C	A
10709	mCV24824414	7	106798942	G	G	A	G	A	G
10710	mCV24824424	7	106799248	A	A	G	A	G	A
10711	mCV24824425	7	106799373	G	G	A	G	A	G
10712	mCV24824448	7	106800225	A	G	G	A	G	A
10713	mCV24824449	7	106800333	A	A	G	A	G	A
10714	mCV24824450	7	116427685	G	G	C	G	C	G
10715	mCV24824460	7	106800594	G	G	A	G	A	G
10716	mCV24824461	7	106800870	A	G	G	A	G	A
10717	mCV24824462	7	106801071	G	A	G	G	G	G
10718	mCV24825103	7	106801200	A	A	G	A	G	A
10719	mCV24825104	7	106810050	A	A	G	A	G	A
10720	mCV24825105	7	106810099	G	G	A	G	A	G
10721	mCV24269205	7	107060853	A	C	C	A	C	A
10722	mCV24269206	7	107061372	A	G	G	A	G	A
10723	mCV23658450	7	107063710	A	G	G	A	G	A
10724	mCV23658451	7	107064246	A	C	C	A	C	A
10725	mCV23658452	7	107065603	C	A	A	C	A	C
10726	mCV23658722	7	107070757	G	A	A	G	A	G
10727	mCV23658723	7	107071013	C	A	A	C	A	C

10728	mCV23658724	7	107071123	A	T	T	A	T	A
10729	mCV23658734	7	107071288	G	A	A	G	A	G
10730	mCV23658735	7	107073039	G	A	A	G	A	G
10731	mCV23658736	7	107073138	G	A	A	G	A	G
10732	mCV23658746	7	107073915	C	A	A	C	A	C
10733	mCV23658771	7	116700730	A	G	G	A	G	A
10734	mCV23658772	7	107079833	A	G	G	A	G	A
10735	mCV23658784	7	107080719	G	A	A	G	A	G
10736	mCV23658796	7	107080809	G	A	A	G	A	G
10737	mCV23659065	7	107081189	A	G	G	A	G	A
10738	mCV23659067	7	107082369	A	G	G	A	G	A
10739	rs3674165	7	112197433	G	G	A	A	A	G
10740	mCV24269234	7	107252093	G	A	A	G	A	G
10741	rs6163967	7	112276217	G	G	G	G	G	G
10742	rs3665969	7	112378938	A	C	C	C	C	A
10743	rs8259860	7	113024642	C	C	C	C	C	C
10744	rs4226888	7	113114338	G	G	G	G	G	G
10745	rs4226889	7	113114379	G	G	G	G	G	G
10746	rs3666076	7	113129199	C	C	C	C	C	C
10747	rs6317057	7	113366854	G	G	A	A	A	G
10748	rs3691633	7	113593665	G	A	A	A	A	G
10749	rs3682038	7	113814867	A	A	G	G	G	A
10750	rs4226893	7	113920280	G	G	G	G	G	G
10751	rs8258004	7	114147403	G	A	G	G	G	G
10752	rs8258009	7	114149744	A	G	A	A	A	A
10753	rs3090575	7	113883831	A	A	A	A	A	A
10754	rs6177431	7	114442529	G	A	G	G	G	G
10755	mCV24278240	7	110114632	G	A	G	G	G	A
10756	rs3687061	7	115134183	G	G	A	G	NONE	G
10757	rs3697227	7	115138631	G	G	A	G	G	G
10758	rs4226901	7	115274716	A	A	A	A	A	A
10759	rs6278159	7	115403696	G	G	G	G	G	G
10760	rs6258497	7	115551297	A	G	G	G	G	G
10761	rs4226910	7	115677022	A	C	C	C	C	A
10762	rs6169594	7	115837321	G	G	G	G	G	G
10763	rs4226914	7	116173064	C	C	C	C	C	C
10764	rs3023161	7	116799239	A	G	G	G	G	G
10765	mCV23674762	7	111918521	C	A	C	C	C	C
10766	rs6198199	7	116945922	G	A	A	A	A	A
10767	rs3690404	7	117141022	G	A	A	A	A	A
10768	rs4226920	7	117141391	G	G	G	G	G	G
10769	rs3706605	7	117141430	C	A	A	A	A	A
10770	rs6386671	7	117314860	A	G	G	G	G	G
10771	rs3682424	7	117428333	G	A	A	A	A	A
10772	rs6178830	7	118392269	G	G	G	G	G	G
10773	rs4226927	7	118553289	G	G	G	G	G	G
10774	rs8236684	7	118599350	G	A	G	G	G	G
10775	rs8236688	7	118600522	G	A	G	G	G	G
10776	rs8265215	7	118877622	G	G	G	G	G	G
10777	rs8265232	7	118879650	G	G	G	G	G	G
10778	rs6391421	7	119396732	C	A	C	C	C	C
10779	rs4226941	7	120193334	A	A	A	A	A	A
10780	rs6245165	7	120355402	A	A	A	A	A	A
10781	rs3700735	7	120509370	G	A	G	A	G	A
10782	rs3716088	7	120621563	C	C	C	A	C	C
10783	rs6389165	7	121281233	A	A	A	A	A	A
10784	rs8236382	7	121315434	A	A	A	A	A	A
10785	rs8265338	7	121333063	A	A	A	A	A	A
10786	rs8257783	7	121337404	A	A	A	A	A	A
10787	rs8257806	7	121340014	G	G	G	G	G	G
10788	rs8257815	7	121342157	G	G	G	G	G	G
10789	mCV24295271	7	116532669	C	A	A	A	A	A
10790	rs3715149	7	121639861	A	A	A	A	A	A
10791	rs3711674	7	121752018	C	C	C	A	A	A
10792	rs6181921	7	122045918	C	C	C	A	A	C
10793	rs4228413	7	122285966	A	A	A	A	A	A
10794	rs3719258	7	122310587	A	A	A	G	G	A
10795	rs3691256	7	122316285	G	G	G	A	A	G
10796	rs6162937	7	123362744	A	A	A	A	A	A
10797	rs4226949	7	123900001	A	A	A	A	A	A
10798	rs6361727	7	124106225	C	C	C	C	G	C
10799	mCV24302212	7	119197674	A	A	G	G	G	G
10800	rs6210610	7	125244618	A	A	A	A	G	A
10801	rs6392543	7	125772315	G	G	G	G	A	G

10802	rs3680633	7	130790118	G	G	G	G	G	G
10803	rs6387891	7	126918848	G	G	G	A	G	G
10804	rs3663988	7	126965988	A	A	A	G	A	A
10805	rs6303477	7	127209009	G	G	G	A	G	G
10806	rs3660122	7	127227445	A	A	A	G	A	A
10807	rs3659292	7	127330792	A	A	A	G	A	A
10808	rs3710520	7	127435326	G	G	G	A	A	G
10809	rs3702235	7	127580455	A	A	A	G	G	A
10810	rs6299045	7	127811588	A	A	A	A	A	G
10811	rs8268006	7	128458425	A	A	A	A	A	A
10812	rs8268010	7	128462367	G	G	G	G	G	G
10813	rs8268014	7	128463197	A	A	A	A	A	A
10814	rs4226966	7	128552828	G	G	G	G	G	G
10815	rs6398876	7	128756845	A	A	A	A	A	A
10816	rs6164802	7	129071169	G	G	G	G	G	G
10817	rs8248857	7	129129838	A	A	A	A	A	A
10818	rs8240223	7	129160048	A	A	A	A	A	A
10819	rs3720654	7	129185119	A	A	A	A	A	A
10820	rs6366172	7	129251519	G	G	G	G	G	G
10821	rs8257967	7	129572235	A	A	A	A	A	A
10822	rs4226975	7	129577442	A	A	A	A	A	A
10823	mCV22291963	7	125007357	G	G	G	G	A	G
10824	rs3702894	7	130056639	A	A	A	A	A	A
10825	rs6254434	7	130220537	A	A	A	A	A	A
10826	mCV24846744	7	125526299	A	A	A	A	G	A
10827	rs8246090	7	130366433	A	A	A	A	A	A
10828	rs8246158	7	130371713	A	A	A	A	A	A
10829	rs8246189	7	130376582	G	G	G	G	G	G
10830	rs8246191	7	130376730	G	G	G	G	G	G
10831	rs3675981	7	131717874	NONE	A	A	A	A	A
10832	rs6318605	7	131542341	G	G	G	G	G	G
10833	rs4226995	7	131554187	G	G	G	G	G	G
10834	rs3710198	7	131575886	A	A	A	A	A	A
10835	rs3686436	7	132152308	A/G	A	A/G	G	A/G	G
10836	rs6212186	7	132173375	G	G	G	A	G	A
10837	rs8243991	7	132610320	A	A	A	C	A	A
10838	rs8244014	7	132610023	G	G	G	G	G	G
10839	rs8248881	7	132609406	G	G	G	G	G	G
10840	rs8248872	7	132607280	C	C	C	C	C	C
10841	rs4226997	7	132606551	G	G	G	A	G	G
10842	rs3666160	7	132546916	A	A	A	G	A	G
10843	rs3725972	7	132751185	G	G	G	A	G	G
10844	rs3709741	7	132750908	A	A	A	G	A	A
10845	rs3694146	7	132710913	G	G	G	A	G	G
10846	rs3714636	7	132657269	A	A	A	C	A	A
10847	rs6216320	7	132894685	A	A	A	G	A	A
10848	mCV25263465	7	131854482	T	A	A	A	A	A
10849	mCV23816735	17	20171689	G	G	A	A	G	A
10850	mCV23496932	17	20196932	A	A	G	G	A	G
10851	mCV23822994	17	21580353	G	G	A	A	G	A
10852	mCV25175177	8	4965824	G	A	A	A	A	A
10853	rs6297682	8	3090708	A	G	G	G	G	G
10854	rs3662707	8	3185643	C	A	A	A	A	A
10855	rs3694261	8	3222134	A	G	G	G	G	G
10856	rs6343953	8	3348981	A	G	G	G	G	G
10857	rs3683766	8	3454208	G	C	C	C	C	C
10858	rs3711570	8	4193008	C	A	A	A	A	A
10859	rs6407785	8	4258532	A	C	C	C	C	C
10860	rs3691770	8	4280007	A	G	G	G	G	G
10861	rs4227016	8	4280044	G	G	G	G	G	G
10862	rs3692453	8	4280149	A	G	G	G	G	G
10863	rs3711928	8	4670120	G	A	A	A	A	A
10864	rs6267431	8	5299732	A	G	G	G	G	G
10865	rs3695650	8	5400235	C	A	A	A	A	A
10866	rs3685615	8	5614128	G	A	A	A	A	A
10867	rs3714139	8	5780286	G	G	G	G	G	G
10868	rs3683955	8	6072916	T	A	A	A	A	A
10869	rs3687825	8	6074974	G	A	A	A	A	A
10870	rs6378648	8	6190746	A	G	G	G	G	G
10871	rs3688544	8	6763317	G	A	A	A	A	A
10872	rs6360601	8	6795642	A	C	C	C	C	C
10873	rs3681707	8	6874349	G	A	A	A	A	A
10874	rs3681890	8	6874427	A	G	G	G	G	G
10875	rs3664009	8	7286424	A	T	T	T	T	T

10876	rs6273176	8	7842210	G	A	A	A	A	G
10877	rs6153168	8	8294541	G	A	A	A	A	G
10878	rs3725303	8	8442439	A	G	G	G	G	A
10879	mCV23753467	8	11544017	G	A	A	A	A	A
10880	rs4227020	8	9202641	T	T	T	T	T	T
10881	rs6258365	8	9285636	A	A	A	A	A	A
10882	rs6323083	8	9850819	A	A	A	A	A	A
10883	rs4227021	8	9990921	A	A	A	A	A	A
10884	rs4227024	8	9992386	A	A	A	A	A	A
10885	rs6380983	8	10200537	G	G	G	G	G	G
10886	rs3090043	8	10521711	G	G	G	G	G	G
10887	rs3667295	8	11144090	G	A	A	A	A	A
10888	rs3658335	8	11342753	G	A	A	A	A	A
10889	rs6190217	8	11448141	A	A	A	A	A	A
10890	rs6256565	8	12153575	G	G	G	G	NONE	G
10891	rs6288205	8	12260500	C	C	C	C	C	C
10892	rs4227033	8	12884853	A	G	G	G	G	G
10893	rs6359406	8	13190527	G	G	G	G	G	G
10894	rs4227035	8	13274389	A	A	A	A	A	A
10895	rs4227049	8	13911274	A	A	A	A	A	A
10896	rs4227053	8	13911495	G	G	G	G	G	G
10897	rs6410533	8	14327885	G	G	G	G	G	G
10898	rs6343961	8	14514674	A	A	A	C	A	A
10899	rs3670924	8	14515925	A	A	A	G	A	A
10900	rs3694208	8	14730616	T	T	T	A	T	T
10901	rs3717752	8	14812378	A	A	A	C	A	A
10902	rs3655271	8	14819873	G	G	G	A	G	G
10903	rs6226916	8	15192313	C	A	A	A	A	A
10904	rs3660469	8	15395212	G	G	G	C	G	G
10905	rs6281247	8	15731163	A	G	G	G	G	G
10906	mCV25230888	8	16550585	A	A	A	G	A	A
10907	rs3678678	8	16274597	A	A	A	G	A	A
10908	rs6208963	8	16298914	A	C	C	C	C	C
10909	mCV25352402	8	12215023	A	G	G	G	G	G
10910	rs3657963	8	16576750	A	A	A	C	A	A
10911	rs3725437	8	16609149	A	G	G	G	G	G
10912	rs3699325	8	17919280	G	G	G	A	G	G
10913	rs3665513	8	17966616	A	A	A	G	A	A
10914	rs3705131	8	18165998	G	G	G	C	G	G
10915	rs6410820	8	18261727	A	A	A	A	A	A
10916	rs3090143	8	18394567	C	C	C	C	C	C
10917	mCV24845756	8	19147703	C	C	C	A	C	C
10918	mCV24847817	8	19206695	A	A	A	C	A	A
10919	rs3699118	8	19119911	A	A	A	G	A	A
10920	rs3695658	8	19125950	G	G	G	A	G	G
10921	rs6221216	8	19594472	G	G	G	G	G	G
10922	rs3686014	8	19828245	C	C	C	G	C	C
10923	rs4227054	8	20547790	G	G	G	G	G	G
10924	rs4227066	8	20548027	G	G	G	A	G	G
10925	rs6228462	8	21136552	A	A	A	A	A	A
10926	rs4140004	8	21285113	A	A	A	G	A	A
10927	rs3705229	8	21315339	G	G	G	A	G	G
10928	rs4227071	8	21414789	G	G	G	G	G	G
10929	rs3711691	8	21468709	A	A	G	A	G	A
10930	rs8269665	8	21536767	A	A	A	A	A	A
10931	rs6287735	8	21566544	A	A	A	A	A	A
10932	rs3655346	8	21723470	G	G	C	G	C	G
10933	rs3696265	8	21758920	G	G	G	A	G	G
10934	rs3666112	8	21796002	A	A	G	A	G	A
10935	mCV22755363	8	22618416	A	G	A	A	A	G
10936	rs3661760	8	22251497	A	A	A	C	A	A
10937	rs4227074	8	22253086	A	A	A	A	A	A
10938	rs4227075	8	22253184	G	G	G	G	G	G
10939	rs6366256	8	22550095	G	G	G	G	G	G
10940	rs2020607	8	22557353	G	G	G	G	G	G
10941	rs3721201	8	22661058	T	T	A	T	A	T
10942	rs4227086	8	23391812	G	G	G	G	G	G
10943	rs4227087	8	23391937	A	A	A	A	A	A
10944	rs3661957	8	23641399	A	G	G	A	G	G
10945	rs6391404	8	23725118	A	A	A	A	A	A
10946	rs4227089	8	24351832	A	A	A	A	A	A
10947	rs4227090	8	24351855	G	G	G	G	G	G
10948	rs6348177	8	24528778	G	G	G	G	G	G
10949	rs4227093	8	24942242	G	G	G	G	G	G

10950	rs6378439	8	24988208	C	C	C	C	C	C
10951	rs3704385	8	25083165	G	G	G	C	G	G
10952	rs3702973	8	25220557	A	A	A	G	A	A
10953	rs3684644	8	25367055	A	A	A	G	A	A
10954	rs6346909	8	25702077	A	A	A	A	A	A
10955	rs6198867	8	26599226	A	A	A	A	A	A
10956	rs3709090	8	27016024	A	A	G	A	G	A
10957	rs3679832	8	27020091	A	A	T	A	T	A
10958	rs6194373	8	27730214	A	A	A	A	A	A
10959	rs6403485	8	27994883	G	G	G	G	G	G
10960	rs6187823	8	28671621	G	G	G	A	G	G
10961	rs3724636	8	28824777	G	G	G	A	G	G
10962	rs3691295	8	28971300	A	A	A	G	A	A
10963	mCV24506226	8	29576238	A	A	A	G	A	A
10964	rs6339455	8	29428902	C	C	C	A	C	C
10965	rs3658700	8	29792667	A	A	A	T	A	A
10966	rs3672582	8	29823248	C	C	C	A	C	C
10967	rs4227097	8	30204777	G	G	G	G	G	G
10968	rs4227096	8	30204827	C	C	C	A	C	C
10969	rs6156151	8	30259771	G	G	G	A	G	G
10970	mCV24495398	8	31061779	A	G	G	A	G	G
10971	rs6391183	8	30604724	G	G	A	A	A	G
10972	rs3668545	8	30639980	A	A	A	T	A	A
10973	rs3706948	8	30816540	A	A	A	G	A	A
10974	rs3684114	8	30877798	G	G	A	G	A	G
10975	rs6166471	8	31477644	G	A	A	G	A	A
10976	rs6183691	8	31711608	G	G	G	G	G	G
10977	rs3680402	8	31779641	G	G	G	A	G	G
10978	rs3670299	8	31783103	A	G	G	G	G	G
10979	rs6276917	8	32071364	G	A	A	A	A	A
10980	rs3677050	8	32350946	A	A	A	G	A	A
10981	rs3697344	8	32482925	A	A	A	G	A	A
10982	rs3669323	8	32373639	A	G	G	A	G	G
10983	rs4227112	8	32537499	G	G	G	A	G	G
10984	rs3725269	8	32785897	G	G	G	A	G	A
10985	rs3678769	8	33055927	A	A	A	G	A	G
10986	rs3665640	8	33212528	A	A	A	G	A	G
10987	rs6182243	8	33268537	A	A	A	G	A	G
10988	rs3023184	8	33387296	G	G	G	A	G	A
10989	rs3088855	8	33387325	G	G	G	G	G	G
10990	rs3694068	8	33507144	A	T	T	A	T	A
10991	rs6361982	8	33545878	G	G	G	G	G	G
10992	rs3726395	8	34047097	C	C	C	G	C	G
10993	rs3679748	8	34085485	G	G	G	A	G	A
10994	rs3675407	8	34294637	C	C	C	G	C	G
10995	rs3726003	8	34309104	A	A	A	G	A	G
10996	rs6412537	8	34355884	C	C	C	C	C	C
10997	rs3689728	8	34422795	A	A	A	G	A	A
10998	rs3714217	8	34774983	G	G	G	A	G	G
10999	rs3665028	8	35164032	G	G	G	A	G	G
11000	rs6298769	8	35257726	G	G	G	A	G	G
11001	rs3725424	8	35299440	C	C	C	A	C	C
11002	rs3678454	8	35434074	A	A	A	G	A	A
11003	rs3657597	8	35479629	A	A	A	G	A	A
11004	rs3697917	8	35497666	A	A	A	G	A	A
11005	rs3672158	8	35519801	G	G	G	A	G	G
11006	rs3654540	8	35567409	A	A	A	C	A	A
11007	rs3709888	8	35752542	A	A	A	G	A	A
11008	rs3697360	8	36452744	G	G	G	A	G	G
11009	rs3671902	8	36561898	G	G	G	A	G	G
11010	rs6347708	8	36602239	G	G	G	A	G	G
11011	rs3672173	8	36943681	G	G	G	A	G	G
11012	rs6232107	8	37264576	C	C	C	G	C	C
11013	rs3724402	8	37307650	A	A	A	G	A	A
11014	rs3667738	8	37536463	A	A	A	T	A	A
11015	rs6305076	8	37605157	A	A	A	G	A	A
11016	rs4139770	8	37776134	G	G	G	A	G	G
11017	rs3670150	8	38146422	G	G	G	A	G	G
11018	rs3716806	8	38325760	G	G	G	A	G	G
11019	rs3657016	8	38395086	A	A	A	G	A	A
11020	rs6217381	8	38694587	A	A	A	G	A	A
11021	rs4227117	8	38690693	A	A	A	G	A	A
11022	rs4227123	8	38690516	A	A	A	A	A	A
11023	rs3089412	8	38716730	G	G	G	G	G	G

11024	rs6398885	8	38906934	A	A	A	G	A	A
11025	rs3720486	8	39226053	A	A	A	G	A	A
11026	rs3698365	8	39339806	G	G	G	C	G	G
11027	rs3674647	8	39442455	A	A	A	C	A	A
11028	rs4227127	8	39662970	G	G	G	A	G	G
11029	rs3703811	8	39770568	A	A	A	G	A	A
11030	rs4227131	8	40028723	A	A	A	A	A	A
11031	rs4227132	8	40028790	C	C	C	A	C	C
11032	rs3715224	8	40179673	A	A	A	G	A	A
11033	rs3692809	8	40290463	G	G	G	A	G	G
11034	rs3696853	8	40364763	A	A	A	G	A	A
11035	rs3685490	8	40368042	A	A	A	G	A	A
11036	rs3663650	8	40997867	G	G	A	A	A	G
11037	rs3706426	8	41123342	G	G	A	A	A	G
11038	rs3711035	8	41215138	C	C	A	A	A	C
11039	rs6270271	8	41732262	G	G	G	G	G	G
11040	rs3672546	8	41899636	G	G	A	A	A	G
11041	rs6298886	8	42076089	G	G	G	G	G	G
11042	rs3666140	8	42081791	G	G	A	A	A	G
11043	rs6393796	8	42139626	A	A	C	C	C	A
11044	mCV24436061	8	42479218	A	A	G	G	G	A
11045	rs3725540	8	42498907	T	T	T	A	T	T
11046	mCV24778752	8	42731622	G	G	G	G	G	G
11047	rs6221022	8	43227116	A	A	A	A	A	A
11048	rs6332019	8	43967468	A	A	A	A	A	A
11049	rs6164599	8	44397128	G	G	G	G	G	G
11050	rs8272033	8	44406052	A	A	A	A	A	A
11051	rs8272116	8	44420853	A	A	A	A	A	A
11052	rs8271871	8	44437204	C	C	C	C	C	C
11053	rs6403404	8	44573120	G	G	G	G	G	G
11054	rs4227148	8	45042177	A	A	A	A	A	A
11055	rs4227149	8	45042248	A	A	A	A	A	A
11056	rs6341458	8	45074802	G	A	A	A	A	A
11057	rs3691123	8	45421242	A	G	G	G	G	G
11058	rs3662336	8	45458816	G	A	A	A	A	A
11059	rs6327437	8	45931707	A	G	G	G	G	G
11060	rs3673554	8	46328232	G	A	A	A	A	A
11061	rs3725083	8	46788083	A	T	T	T	T	A
11062	rs3694071	8	47033579	A	G	G	G	G	G
11063	rs6218480	8	47853957	A	T	T	T	T	A
11064	rs3724820	8	47920080	T	A	A	A	A	T
11065	rs3720150	8	48154076	C	A	A	A	A	C
11066	rs6285908	8	48354052	G	A	A	A	A	G
11067	rs6322353	8	48356168	G	G	G	G	G	G
11068	rs3719401	8	48804736	A	G	G	G	G	A
11069	rs4136613	8	48958389	G	G	A	A	A	G
11070	rs6409915	8	49016433	G	G	A	A	A	G
11071	rs6360429	8	49945882	A	A	A	A	A	A
11072	rs3697959	8	50000714	G	G	A	A	A	G
11073	rs3088450	8	50650209	A	A	C	C	C	A
11074	rs6268002	8	50835330	G	G	G	G	G	G
11075	rs6269038	8	50835524	C	C	C	C	C	C
11076	rs6228197	8	51706476	G	G	G	G	G	G
11077	rs6231930	8	52166690	G	G	A	A	A	A
11078	rs4227159	8	52696704	A	A	A	A	A	A
11079	mCV24479810	8	53447892	A	A	G	G	G	A
11080	rs3661488	8	53330452	A	A	G	G	G	A
11081	rs3687648	8	53997362	A	A	G	G	G	G
11082	rs6282113	8	54257227	G	G	G	G	G	G
11083	rs3712458	8	54497079	A	A	G	G	G	A
11084	rs3659789	8	54813320	G	G	G	A	G	G
11085	rs6260693	8	54903858	G	G	G	G	G	G
11086	rs6168647	8	54965220	G	G	A	G	A	A
11087	rs6292169	8	55245979	G	G	C	C	C	C
11088	rs3663761	8	55465097	A	A	C	A	C	C
11089	rs3677675	8	55473057	A	A	G	A	G	G
11090	rs3659366	8	55477724	A	A	G	G	G	G
11091	mCV23056731	8	55932578	G	G	A	G	A	A
11092	mCV22562218	8	55939656	A	A	G	G	G	G
11093	rs3657344	8	56115378	A	A	A	G	A	A
11094	rs3725286	8	56141367	A	A	G	G	G	G
11095	rs6399377	8	56487610	A	A	A	G	A	A
11096	rs3685424	8	56548348	G	G	A	G	A	A
11097	rs6213025	8	56852761	A	A	A	T	A	A

11098	rs6213640	8	56852914	G	G	A	A	A	A
11099	rs3726882	8	57042211	A	A	G	A	G	G
11100	rs3679638	8	57141863	G	G	G	A	G	G
11101	rs3088776	8	57456830	G	G	G	G	G	G
11102	rs3719788	8	57542263	G	G	A	G	A	A
11103	rs3707439	8	57724335	G	G	A	G	A	A
11104	rs6351975	8	57941116	G	G	A	G	A	A
11105	rs3726906	8	58201849	G	G	A	G	A	A
11106	rs6398181	8	59013332	A	G	G	A	G	G
11107	rs3088732	8	59026354	T	T	T	T	T	T
11108	rs3704301	8	59074714	C	C	A	A	A	A
11109	rs6173355	8	59086266	A	A	G	G	G	G
11110	rs3672639	8	60120308	G	A	A	G	A	A
11111	rs6370413	8	60430582	C	A	C	C	C	C
11112	rs3717251	8	60527867	A	A	G	A	G	G
11113	rs3657679	8	60737399	G	G	C	G	C	C
11114	rs3693121	8	60745152	T	T	A	T	A	A
11115	mCV24047460	8	61454022	G	C	C	G	C	C
11116	rs6394046	8	61422061	G	A	A	G	A	A
11117	rs3680085	8	61676838	A	G	G	A	G	G
11118	mCV22586111	8	62269806	T	A	A	T	A	T
11119	rs3659852	8	62164881	A	A	T	A	T	A
11120	rs6297611	8	62194889	A	A	G	A	G	A
11121	rs3720114	8	62369674	A	A	T	A	T	A
11122	rs3667255	8	62920755	A	A	G	A	G	A
11123	rs3674032	8	63286008	G	A	A	G	A	G
11124	rs4227189	8	63518254	T	A	A	T	A	T
11125	rs4227185	8	63518415	G	G	G	G	G	G
11126	rs3700097	8	63977744	T	T	A	T	A	T
11127	mCV23853440	8	64520889	A	G	G	A	G	A
11128	rs3697988	8	64390408	A	A	G	A	G	A
11129	rs3703096	8	64394119	A	G	G	A	G	A
11130	rs6314247	8	65135750	G	A	G	G	G	G
11131	rs3712611	8	65261034	A	G	G	A	G	G
11132	rs6182473	8	65599937	T	A	T	T	T	T
11133	rs8238643	8	65662099	A	A	A	A	A	A
11134	rs8238642	8	65662571	A	A	A	A	A	A
11135	mCV24841004	8	66689813	A	G	A	A	A	A
11136	rs8236724	8	66478381	A	A	A	A	A	A
11137	rs8254797	8	66485171	A	A	A	A	A	A
11138	rs8254800	8	66485350	T	A	T	T	T	T
11139	mCV23008156	8	67317447	A	G	G	G	G	A
11140	mCV23008166	8	67321458	A	G	G	A	G	A
11141	mCV23008211	8	67323221	A	T	T	T	T	A
11142	mCV23008401	8	67323949	A	G	G	G	G	A
11143	mCV23008402	8	67324026	G	A	A	A	A	G
11144	mCV22725457	6	109145289	T	T	T	T	A	T
11145	mCV22725447	6	109145141	G	G	G	G	A	G
11146	mCV24403642	8	67331933	G	G	G	A	G	G
11147	mCV24403652	8	67332220	A	A	A	C	A	A
11148	mCV24403653	8	67332378	G	G	G	A	G	G
11149	mCV24403664	8	67332960	A	A	A	G	A	A
11150	mCV24403665	8	67333222	T	A	A	T	A	T
11151	mCV24403666	8	67333685	A	G	G	A	G	A
11152	mCV24403936	8	67333735	G	A	A	G	A	G
11153	mCV24403950	8	67334009	A	G	G	A	G	A
11154	mCV24403961	8	67334658	G	G	G	A	G	G
11155	mCV24403972	8	67335227	A	C	C	C	C	A
11156	mCV24403973	8	67335269	A	A	A	G	A	A
11157	mCV24403974	8	67335419	G	A	A	G	A	G
11158	mCV24403984	8	67856331	A	G	G	A	G	A
11159	mCV24403986	8	67337807	C	A	A	A	A	C
11160	mCV24403995	8	67338007	C	C	C	A	C	C
11161	mCV24403996	8	67338342	A	G	G	G	G	A
11162	mCV24404008	8	67338897	A	A	A	C	A	A
11163	mCV24404280	8	67339468	A	A	A	G	A	A
11164	mCV24404294	8	67863486	G	G	G	A	G	G
11165	mCV24404306	8	67347408	A	G	G	A	G	A
11166	mCV24404316	8	67347492	C	C	C	A	C	C
11167	mCV24404317	8	67347861	A	G	G	A	G	A
11168	mCV24404318	8	67348088	G	G	G	A	G	G
11169	mCV24404329	8	67348864	A	G	G	A	G	A
11170	mCV24404339	8	67351946	G	A	A	A	A	G
11171	mCV24404340	8	67351968	A	G	G	G	G	A

11172	mCV24793580	8	67354429	A	G	G	A	G	A
11173	mCV22531912	8	67358786	A	C	C	C	C	A
11174	mCV22531922	8	67358939	A	G	G	G	G	A
11175	mCV22531923	8	67359074	G	A	A	A	A	G
11176	mCV22531946	8	67359331	A	G	G	G	G	A
11177	mCV22532220	8	67878817	A	T	T	T	T	A
11178	mCV22532221	8	67878822	A	G	G	G	G	A
11179	mCV25342743	8	67361740	C	G	G	C	G	C
11180	mCV25342756	8	67361826	C	G	G	G	G	C
11181	mCV25342767	8	67362308	G	G	G	A	G	G
11182	mCV25342768	8	67363264	A	G	G	G	G	A
11183	mCV25342954	8	67883462	G	A	A	A	A	G
11184	mCV25342955	8	67364446	G	A	A	A	A	G
11185	mCV25342967	8	67365021	C	A	A	A	A	C
11186	mCV25342977	8	67365121	G	A	A	A	A	G
11187	mCV25342978	8	67365287	A	G	G	G	G	A
11188	mCV25342979	8	67365729	G	A	A	G	A	G
11189	mCV25342990	8	67366088	A	G	G	G	G	A
11190	mCV25342991	8	67366360	A	G	G	A	G	A
11191	mCV25343003	8	67366446	G	A	A	A	A	G
11192	mCV25343014	8	67366638	C	A	A	C	A	C
11193	mCV25343211	8	67368070	A	C	C	A	C	A
11194	mCV25343212	8	67368177	A	G	G	A	G	A
11195	mCV25343223	8	67887718	A	C	C	A	C	A
11196	mCV25343225	8	67369350	A	A	A	G	A	A
11197	mCV25343236	8	67372492	G	G	G	A	G	G
11198	mCV25343247	8	67372625	A	G	G	A	G	A
11199	mCV25343248	8	67372798	G	A	A	G	A	G
11200	mCV25343249	8	67373869	A	G	G	A	G	A
11201	mCV25343256	8	67373925	A	G	G	A	G	A
11202	mCV25343258	8	67374260	G	A	A	G	A	G
11203	rs6259892	8	66925554	G	G	G	G	G	G
11204	rs3702472	8	67113287	A	A	A	G	A	A
11205	rs3089890	8	67188718	G	G	G	G	G	G
11206	rs3656875	8	67222604	G	G	G	A	G	G
11207	rs3658748	8	67237674	C	C	C	G	C	C
11208	rs3720418	8	67240740	A	A	A	G	A	A
11209	rs6402928	8	67246476	T	T	T	A	T	T
11210	rs6155902	8	67321031	C	C	C	C	C	C
11211	rs6156473	8	67321145	G	G	G	G	G	G
11212	rs6156951	8	67321208	A	A	A	A	A	A
11213	rs6310665	8	67335470	G	G	G	G	G	G
11214	rs6311211	8	67335572	A	A	A	A	A	A
11215	rs6312291	8	67335784	A	A	A	G	A	A
11216	rs6228248	8	67363511	G	G	G	G	G	G
11217	rs6361420	8	67382884	G	G	G	A	G	G
11218	rs3089636	8	67756889	G	G	G	A	G	G
11219	rs8253516	8	67908410	A	A	A	G	A	A
11220	rs6268167	8	67908683	A	A	A	T	A	A
11221	rs3681089	8	67920720	G	G	G	A	G	G
11222	mCV24409492	8	68381512	A	C	A	C	A	A
11223	mCV24421661	8	68865520	G	A	G	A	G	G
11224	mCV24421662	8	68865600	A	G	A	G	A	A
11225	mCV24421673	8	68866239	G	A	G	A	G	G
11226	mCV24421685	8	68868940	A	C	A	C	A	A
11227	mCV24421686	8	68868948	A	G	A	G	A	A
11228	mCV24421698	8	68872092	G	A	G	A	G	G
11229	mCV24421699	8	68872162	G	A	G	A	G	G
11230	mCV24421735	8	68874838	A	G	A	G	A	A
11231	mCV24422006	8	69390645	A	G	A	G	A	A
11232	mCV24422007	8	68875365	C	G	C	G	C	C
11233	mCV24422017	8	68875533	A	G	A	G	A	A
11234	mCV24657217	8	68876395	G	A	G	A	G	G
11235	mCV24657226	8	68876496	A	C	A	C	A	A
11236	mCV22527651	8	68869236	A	G	A	G	A	A
11237	mCV22527661	8	68869262	G	A	G	A	G	G
11238	mCV22619744	8	68877647	A	G	A	G	A	A
11239	mCV22619745	8	68877882	C	A	C	A	C	C
11240	mCV22619752	8	68878266	G	A	G	A	G	G
11241	mCV22619754	8	68878699	A	G	A	G	A	A
11242	mCV22619781	8	68880128	A	G	A	G	A	A
11243	mCV22619789	8	68880168	G	A	G	A	G	G
11244	mCV22620057	8	68880316	A	G	A	G	A	A
11245	mCV22620059	8	68881115	C	G	C	G	C	C

11246	mCV22620066	8	68881254	C	G	C	G	C	C
11247	mCV22620067	8	68881604	G	A	G	A	G	G
11248	mCV22620068	8	68881785	G	A	G	A	G	G
11249	mCV22620075	8	68882508	G	A	G	A	G	G
11250	mCV22620077	8	68882809	A	C	A	C	A	A
11251	mCV22620084	8	68882872	A	G	A	G	A	A
11252	mCV22620086	8	68884264	A	A/C	A	C	A	A
11253	mCV22620093	8	68884711	T	A	T	A	T	T
11254	mCV22620103	8	69410452	A	G	A	G	A	A
11255	mCV22620104	8	68886159	A	G	A	G	A	A
11256	mCV22620111	8	68886420	G	A	G	A	G	G
11257	mCV22620112	8	68886589	A	G	A	G	A	A
11258	mCV22620129	8	68887137	A	G	A	G	A	A
11259	mCV22620130	8	68887192	G	A	G	A	G	G
11260	mCV22620131	8	68887219	A	G	A	G	A	A
11261	mCV22620138	8	68887315	G	A	G	A	G	G
11262	mCV22675711	8	68889566	G	A	G	A	G	G
11263	mCV22675710	8	68889567	A	G	A	G	A	A
11264	mCV22675700	8	68889669	G	A	G	A	G	G
11265	mCV22675676	8	68890206	G	A	G	A	G	G
11266	mCV22675666	8	68890603	G	A	G	A	G	G
11267	mCV22675665	8	68890873	A	G	A	G	A	A
11268	mCV22675664	8	68890970	A	G	A	G	A	A
11269	mCV22675478	8	68891100	G	A	G	A	G	G
11270	mCV22675468	8	68891268	G	A	G	A	G	G
11271	mCV22675467	8	68891301	G	A	G	A	G	G
11272	mCV22675443	8	68894298	A	G	A	G	A	A
11273	mCV22675433	8	68895325	C	G	C	G	C	C
11274	mCV22675431	8	68895614	A	G	A	G	A	A
11275	mCV22675420	8	68895780	A	G	A	G	A	A
11276	mCV22675211	8	68899558	A	G	A	G	A	A
11277	mCV22675210	8	68899690	A	T	A	T	A	A
11278	mCV22675200	8	68900079	C	A	C	A	C	C
11279	mCV22675176	8	68901194	C	G	C	G	C	C
11280	mCV22675175	8	69428179	A	C	A	C	A	A
11281	mCV22675140	8	68902907	A	C	A	C	A	A
11282	mCV22675139	8	68902924	G	G	G	G	G	G
11283	mCV22674941	8	68903379	G	A	G	A	G	G
11284	mCV22674940	8	68904093	A	G	A	G	A	A
11285	mCV22674930	8	68904288	A	G	A	G	A	A
11286	mCV22674929	8	68904587	A	G	A	G	A	A
11287	mCV22674916	8	68905002	A	G	A	G	A	A
11288	mCV22674905	8	68905545	C	G	C	G	C	C
11289	mCV22674894	8	68906132	A	G	A	G	A	A
11290	mCV22674893	8	68906223	G	A	G	A	G	G
11291	mCV22674884	8	69432014	A	G	A	G	A	A
11292	mCV22674883	8	68906703	A	G	A	G	A	A
11293	mCV27580773	8	69449677	G	A	G	A	G	G
11294	rs3699406	8	68996069	A	G	A	G	A	A
11295	rs3676851	8	69021497	A	G	A	G	A	A
11296	rs8237356	8	69175297	G	A	G	A	G	G
11297	rs4227221	8	69184037	G	C	G	C	G	G
11298	rs8254473	8	69202237	A	A	A	A	A	A
11299	rs6340562	8	69216156	G	A	G	A	G	G
11300	rs4227232	8	69217058	A	A	A	A	A	A
11301	rs3703501	8	69598472	G	A	G	A	G	G
11302	rs3711099	8	69821100	G	A	G	A	G	G
11303	mCV22907805	8	70043222	A	C	A	C	A	A
11304	rs8236355	8	70602288	G	G	G	G	G	G
11305	rs8262736	8	70618545	G	A	G	A	G	G
11306	rs8262738	8	70618891	A	C	A	C	A	A
11307	rs8262739	8	70619123	C	G	C	G	C	C
11308	rs3667475	8	70497527	A	G	A	G	A	A
11309	rs3685305	8	70778450	C	A	C	A	C	C
11310	mCV22939086	8	82437274	A	A	A	G	A	G
11311	rs3718046	8	71471329	A	T	A	T	A	A
11312	rs3722665	8	71579125	A	C	A	C	A	A
11313	rs6296189	8	71681119	C	A	C	A	C	C
11314	rs4227251	8	71872034	G	G	G	G	G	G
11315	rs3160730	8	72212224	G	A	G	A	G	G
11316	rs3683363	8	72604785	A	C	A	C	A	A
11317	rs3688799	8	73488340	G	A	G	A	G	G
11318	rs3694714	8	73492080	A	G	A	G	A	A
11319	rs3722188	8	73606592	C	G	C	G	C	C

11320	rs3691327	8	73743983	G	A	G	A	G	G
11321	rs3089702	8	73894659	A	A	A	A	A	A
11322	rs4227253	8	74193741	G	A	G	A	G	A
11323	rs4227254	8	74193772	C	C	C	C	C	C
11324	rs4227259	8	74194025	C	C	C	C	C	C
11325	rs6251877	8	74345571	A	C	C	C	A	A
11326	mCV24026034	8	73802705	C	G	C	G	C	C
11327	rs3696502	8	74806749	A	G	G	G	A	G
11328	rs6300672	8	74926925	G	G	G	G	G	G
11329	rs3722655	8	75561792	A	G	G	G	A	G
11330	rs3658934	8	75747842	A	T	T	A	A	T
11331	rs3676644	8	75949930	G	A	A	G	G	G
11332	rs6284963	8	75962184	A	T	T	A	A	A
11333	rs3690549	8	76126530	C	A	A	C	C	A
11334	rs4136401	8	76711806	A	T	T	A	A	A
11335	rs3698093	8	77144203	A	A	G	A	A	A
11336	rs6228528	8	77354995	G	G	G	G	G	G
11337	rs6266968	8	77547523	A	A	A	A	A	A
11338	rs3090466	8	77856643	G	G	G	G	G	G
11339	rs6174248	8	77879581	G	G	G	G	G	G
11340	rs4227270	8	78836461	A	A	A	A	A	A
11341	rs6356701	8	79089379	A	A	A	A	A	G
11342	rs6296891	8	79152010	C	C	G	C	C	G
11343	rs6352185	8	79350831	A	A	A	A	A	G
11344	rs3088545	8	79613809	A	A	A	A	A	A
11345	rs3719174	8	80049591	G	G	G	A	G	A
11346	rs4227272	8	80165642	C	C	C	C	C	C
11347	rs3696786	8	80182161	G	G	G	A	G	A
11348	rs6342663	8	80713272	G	G	G	A	G	A
11349	rs3662744	8	81054294	G	G	G	A	G	A
11350	mCV24159008	8	80854257	T	A	A	T	A	T
11351	rs3679284	8	81362136	A	A	A	C	A	C
11352	rs3685476	8	81508980	G	G	G	A	G	A
11353	rs8238240	8	81586346	A	A	A	A	A	A
11354	rs6282422	8	81613742	G	G	G	A	G	A
11355	rs3666835	8	81707985	A	A	A	T	A	T
11356	rs3690145	8	81773963	A	A	A	C	A	C
11357	rs6339346	8	81778963	A	A	A	T	A	T
11358	rs6185205	8	81947929	A	A	A	C	A	C
11359	rs3713940	8	82154722	G	G	G	A	G	A
11360	rs3681482	8	82548171	G	G	G	A	G	A
11361	rs3680975	8	82555372	G	G	G	A	G	A
11362	rs3667484	8	82690124	G	G	G	A	G	A
11363	rs3698182	8	82852106	A	A	A	G	A	G
11364	rs8253610	8	82933517	G	G	G	G	G	G
11365	rs8236751	8	82934737	G	G	G	G	G	G
11366	rs8236770	8	82939075	A	A	A	G	A	G
11367	rs3678433	8	83216284	C	C	C	A	C	C
11368	rs3662136	8	83412560	G	G	G	A	G	A
11369	rs3023193	8	83995333	A	A	A	G	A	A
11370	rs3667288	8	84028638	G	G	G	A	G	G
11371	rs3703660	8	84540685	G	G	G	A	G	G
11372	rs6289238	8	84599477	G	G	G	A	G	G
11373	rs3088578	8	86361081	A	A	A	A	A	A
11374	mCV24195013	8	84373885	A	G	G	A	G	G
11375	rs3700000	8	86427528	A	G	G	A	G	G
11376	rs6257357	8	84826788	G	G	G	A	G	G
11377	rs4227308	8	84916769	A	A	A	A	A	A
11378	rs3700630	8	84958180	A	A	A	G	A	A
11379	rs6364023	8	85133998	G	G	G	A	G	G
11380	rs3669548	8	85303225	A	G	G	A	G	G
11381	rs3023190	8	86018148	G	G	G	G	G	G
11382	rs6257448	8	86304226	A	A	A	G	A	A
11383	rs6258517	8	86304446	A	A	A	G	A	A
11384	rs3724725	8	86307668	G	G	G	A	G	G
11385	rs3702520	8	86395958	G	G	G	A	G	G
11386	rs3700628	8	86427657	A	G	G	A	G	G
11387	rs3682243	8	86628559	G	A	A	A	A	A
11388	rs6186316	8	86786719	A	C	C	A	A	A
11389	rs3724111	8	87188052	G	G	G	A	G	G
11390	mCV24216915	8	86879639	A	G	G	G	G	G
11391	rs6235292	8	87407229	A	G	G	A	A	A
11392	rs6210223	8	87508784	A	T	T	A	A	A
11393	rs3023196	8	87635378	G	G	G	G	A	A

11394	rs3692662	8	87665852	G	A	A	A	G	G
11395	rs3023195	8	87931962	A	G	G	G	A	A
11396	rs3023194	8	87931984	A	G	G	G	A	A
11397	rs3671185	8	88256377	T	A	A	A	T	T
11398	rs6296403	8	88390960	G	A	A	A	G	G
11399	mCV23424980	8	88630036	G	C	C	C	G	G
11400	rs3675233	8	89067207	G	A	A	A	G	G
11401	rs3662700	8	89109076	G	A	A	A	G	G
11402	rs6391152	8	89169668	C	A	A	A	C	C
11403	rs6228937	8	89335906	G	G	G	G	G	G
11404	rs3654658	8	89662188	A	G	G	G	A	A
11405	rs3672711	8	89802934	A	G	G	G	A	A
11406	rs6403541	8	89986060	G	C	C	C	G	G
11407	rs3702969	8	89987673	A	G	G	G	A	A
11408	rs4227313	8	90433691	G	A	A	A	G	G
11409	rs4227318	8	90433975	G	G	G	G	G	G
11410	rs3677073	8	90989055	A	T	T	T	A	A
11411	rs6316991	8	91265861	G	A	A	A	G	G
11412	rs3655980	8	91662852	A	G	G	G	A	A
11413	rs6334722	8	91998007	C	A	A	A	C	C
11414	rs3704645	8	92253968	C	C	C	A	C	C
11415	rs3023197	8	92291928	C	C	C	G	C	C
11416	rs8278860	8	92307242	G	G	G	G	G	G
11417	mCV24938952	8	92483578	G	G	G	A	G	G
11418	rs4227326	8	92603173	A	A	A	A	A	A
11419	rs3726239	8	93016248	G	A	A	A	A	A
11420	rs6357819	8	93144202	G	G	G	G	G	NONE
11421	rs6240783	8	93690224	G	G	G	G	G	G
11422	rs6373291	8	93750358	A	A	A	G	A	A
11423	rs4227341	8	93875013	T	T	T	T	T	T
11424	rs4137596	8	94037444	A	G	G	A	G	G
11425	rs3696582	8	94412871	C	C	C	C	C	C
11426	rs3705695	8	94705690	A	A	A	G	A	A
11427	rs6356426	8	94792921	G	G	G	A	G	G
11428	rs4227346	8	94988717	G	G	G	G	G	G
11429	rs4227350	8	94988972	G	G	A	G	A	G
11430	rs3666069	8	95051427	G	G	G	A	G	G
11431	rs6285803	8	95323284	G	G	A	A	A	G
11432	rs3685072	8	95515302	A	A	T	T	T	A
11433	rs3661882	8	95633139	A	G	A	G	A	G
11434	rs3696312	8	95820753	C	C	A	C	A	C
11435	rs3089755	8	95862495	A	A	A	A	A	C
11436	rs6290035	8	96081430	C	C	C	A	C	C
11437	rs3664869	8	96228920	A	G	G	G	G	G
11438	rs3684459	8	96365192	G	G	G	A	G	G
11439	rs3679439	8	96471408	A	C	C	C	C	A
11440	rs2020707	8	96683637	C	C	C	C	C	C
11441	rs6287320	8	96735663	G	G	G	A	G	G
11442	rs3672391	8	96746212	G	A	G	A	G	G
11443	rs3663506	8	97051415	A	G	G	G	G	A
11444	rs3088737	8	97439139	T	T	T	T	T	T
11445	rs3691353	8	97606956	G	G	G	G	G	NONE
11446	rs6371774	8	97626358	A	A	A	A	A	A
11447	mCV23909575	8	97810205	C	A	C	A	C	C
11448	rs3660197	8	97926146	G	G	A	G	A	G
11449	rs6409725	8	97995175	C	C	C	A	C	C
11450	rs3685310	8	98445448	G	G	G	A	G	G
11451	mCV25155100	8	99491355	A	A	G	G	G	G
11452	rs3687643	8	99357148	G	G	A	A	A	A
11453	rs6302053	8	99376344	G	G	A	A	A	A
11454	rs6299827	8	99742911	A	A	A	G	A	A
11455	rs3721390	8	99781130	G	A	A	A	A	A
11456	rs3683922	8	99806477	A	A	A	G	A	A
11457	rs3670293	8	100121767	G	A	A	G	A	A
11458	rs3706149	8	100766333	A	C	A	C	A	A
11459	rs3703566	8	100815188	T	A	T	A	T	T
11460	rs6184991	8	101043753	A	G	G	A	G	G
11461	rs6398478	8	101199871	G	A	A	A	A	A
11462	rs3655905	8	101210080	G	A	A	G	A	A
11463	rs3669235	8	101399186	A	G	G	A	G	G
11464	rs3088611	8	101552864	A	A	A	A	A	A
11465	rs3725510	8	101587237	G	A	A	A	A	A
11466	mCV23424042	8	101888552	T	A	T	A	T	T
11467	rs6343029	8	101690853	G	G	G	A	G	G

11468	rs6277144	8	101939750	G	G	A	G	A	A
11469	rs3675894	8	101988104	A	A	A	C	A	A
11470	rs3705275	8	102686478	G	G	G	A	G	A
11471	rs3693083	8	102726995	A	A	A	C	A	C
11472	rs6259355	8	102731137	G	G	G	A	G	A
11473	rs6266614	8	103935312	A	A	A	A	A	A
11474	rs3677807	8	106003031	T	T	T	A	T	A
11475	rs6322354	8	104555441	A	A	A	A	A	A
11476	rs8237135	8	104835786	A	A	A	A	A	A
11477	rs8278915	8	104835954	G	G	G	G	G	G
11478	rs8238610	8	104836347	G	G	G	G	G	G
11479	rs8279106	8	105247164	A	A	A	A	A	A
11480	rs8278979	8	105274413	A	A	A	A	A	A
11481	rs8237141	8	105275795	T	T	T	T	T	T
11482	rs3672761	8	106133227	G	G	G	A	G	A
11483	rs6182338	8	106291302	A	A	A	G	A	A
11484	rs3090934	8	106322625	A	A	A	A	A	A
11485	rs6200325	8	106776000	G	G	G	G	G	G
11486	rs4227362	8	106892879	A	A	A	A	A	A
11487	rs4227361	8	106893247	G	G	G	G	G	G
11488	rs3666037	8	107255801	C	C	C	A	C	C
11489	rs3715791	8	107455154	A	A	A	G	A	A
11490	rs3716360	8	107455220	A	A	A	G	A	A
11491	rs6184273	8	107477850	G	G	G	G	G	G
11492	rs3681151	8	107923763	G	G	G	G	G	G
11493	rs6200314	8	108113736	G	G	G	G	G	G
11494	rs4138940	8	108402238	A	A	A	G	A	A
11495	mCV24836362	8	108850021	A	A	A	A	A	A
11496	rs4227364	8	108496466	A	A	A	A	A	A
11497	rs3662808	8	108727362	G	G	G	A	G	G
11498	rs3691375	8	108731233	G	G	G	A	G	G
11499	rs3090468	8	109170284	G	G	G	G	G	G
11500	rs6154497	8	109244358	A	A	A	A	A	A
11501	rs6237645	8	109333466	G	G	G	A	G	G
11502	rs6341444	8	109636176	A	A	A	A	A	A
11503	rs6328195	8	109864758	G	G	G	G	G	G
11504	rs4232695	8	110066120	A	A	A	A	A	A
11505	rs6297414	8	110619806	G	G	G	G	G	G
11506	rs8238613	8	111005936	G	G	G	G	G	G
11507	rs8238614	8	111006002	A	A	A	A	A	A
11508	rs3663135	8	111029384	A	A	A	A	A	A
11509	rs3689933	8	111816074	A	A	A	A	A	A
11510	rs6350174	8	112677037	G	G	G	G	G	G
11511	rs6389434	8	112908379	G	G	G	G	G	G
11512	rs3666593	8	113096129	G	G	G	G	G	G
11513	rs3693520	8	113155823	A	G	G	G	G	G
11514	rs3700124	8	113595205	G	A	A	G	A	G
11515	rs4227398	8	113744792	A	C	C	C	C	C
11516	rs4227396	8	113744959	C	C	C	C	C	C
11517	rs6379589	8	114100638	G	G	G	G	G	G
11518	rs6282843	8	114509632	G	G	G	G	G	G
11519	rs3691294	8	114511091	A	G	G	G	G	G
11520	rs4227407	8	114583691	A	A	A	A	A	A
11521	rs4227399	8	114584003	G	G	G	G	G	G
11522	rs3671292	8	114851035	A	A	G	A	A	A
11523	rs3709966	8	115085512	A	A	G	A	G	G
11524	rs6175006	8	115169489	T	T	T	T	T	T
11525	rs3656840	8	115618395	A	A	C	A	C	C
11526	rs6347490	8	115958589	C	C	C	C	A	C
11527	rs6188329	8	116277529	T	T	T	T	T	T
11528	rs3665212	8	116281751	A	A	G	A	A	G
11529	rs4227408	8	117154433	A	A	A	A	A	A
11530	rs6210613	8	117450205	C	C	C	C	C	C
11531	rs6213878	8	118208362	G	G	G	G	G	G
11532	rs3691647	8	118756738	G	G	G	G	G	G
11533	rs6216508	8	119493663	A	A	A	A	A	A
11534	rs8238617	8	120122308	A	A	A	A	A	A
11535	rs4227416	8	120553054	G	G	G	G	G	G
11536	rs4227417	8	120553211	G	G	G	G	G	G
11537	rs6276423	8	120745248	C	C	C	C	C	C
11538	rs3657839	8	120997427	A	G	G	A	A	A
11539	rs3708073	8	121235325	T	T	T	A	A	T
11540	rs4227424	8	121271580	G	G	G	G	G	G
11541	rs6392868	8	121484953	T	T	T	A	A	T

11542	rs3708550	8	121508436	A	A	A	C	C	A
11543	rs3711782	8	121665420	G	G	G	A	A	G
11544	rs3675125	8	121724573	G	A	A	G	G	G
11545	rs6377872	8	121825605	A	A	A	T	T	A
11546	rs3667780	8	122022305	A	G	G	A	A	A
11547	rs3705615	8	122040166	G	A	A	G	G	G
11548	rs4227427	8	122405138	G	G	G	G	G	G
11549	rs3693295	8	122421153	G	G	G	A	A	A
11550	rs3723089	8	122426582	G	G	G	A	A	A
11551	mCV23469459	8	123010722	G	G	G	A	G	G
11552	rs4227429	8	122735839	A	G	A	G	G	G
11553	rs4227438	8	122874601	A	A	A	A	A	A
11554	rs4227433	8	122874883	A	A	A	A	A	A
11555	rs6153000	8	123141956	A	A	A	A	A	A
11556	rs8242265	8	123337127	A	G	A	A	A	A
11557	rs3705725	8	123350039	NONE	G	G	A	A	G
11558	rs8249856	8	123351527	A	G	G	A	A	G
11559	rs6221454	8	123380261	G	A	G	G	G	G
11560	rs3689358	8	123456483	G	G	A	G	G	A
11561	rs8238636	8	123939602	A	G	A	A	A	A
11562	rs4227456	8	124053484	G	G	G	A	A	G
11563	rs4227466	8	124219494	G	G	G	G	G	G
11564	rs4227467	8	124219529	C	A	C	C	C	C
11565	rs4227469	8	124219626	G	A	G	G	G	G
11566	mCV23966377	8	124969611	G	A	A	G	G	G
11567	rs3685264	8	124496252	A	C	C	A	A	A
11568	rs6310608	8	124710759	G	A	A	G	G	G
11569	rs6311774	8	124868636	C	A	A	C	C	C
11570	rs4227470	8	125410202	C	G	G	C	C	C
11571	rs4227473	8	125410358	A	T	T	A	A	A
11572	rs3709567	8	125589380	A	G	G	A	A	A
11573	rs6163187	8	125594720	G	A	A	G	G	G
11574	rs6400423	8	125966003	G	G	G	A	G	A
11575	rs3691086	8	126818362	G	A	A	G	G	G
11576	rs6249615	8	126966423	A	G	G	A	A	A
11577	rs3668216	8	126970686	G	G	G	A	A	A
11578	rs4227489	8	127016412	A	A	A	A	A	A
11579	rs3697596	8	127236890	G	G	G	A	G	A
11580	rs3699035	8	127485505	A	T	T	A	T	A
11581	rs3703356	8	127609301	C	C	C	A	A	A
11582	rs3703161	8	127779058	G	G	G	A	A	A
11583	rs3667341	8	127990433	G	G	G	A	A	A
11584	rs3716715	8	128006168	G	G	G	A	G	G
11585	mCV23489377	8	130602738	G	A	A	A	G	A
11586	rs6250817	8	128230076	G	G	G	G	G	G
11587	rs3695597	8	128355283	T	A	A	A	T	A
11588	rs4227297	8	84374585	G	G	G	G	G	G
11589	mCV23053483	9	6793269	A	T	T	T	T	T
11590	mCV22542369	9	7669249	G	A	A	A	A	A
11591	mCV24989119	9	7739786	C	A	A	A	A	A
11592	mCV23955099	9	8534239	C	A	A	A	A	A
11593	rs6242233	9	3355872	A	A	A	A	A	A
11594	rs6411297	9	4481459	A	A	A	A	A	A
11595	rs3678028	9	4678652	C	G	G	G	G	G
11596	rs3726620	9	5185456	A	G	G	G	G	G
11597	rs6269094	9	5305232	A	A	A	A	A	A
11598	rs3692968	9	5921068	A	G	G	G	G	G
11599	mCV24464880	9	11994842	G	G	G	G	G	G
11600	rs6379269	9	6306706	A	A	A	A	A	A
11601	rs6167568	9	6577471	G	A	A	A	A	A
11602	mCV24465575	9	12387092	G	A	A	G	A	G
11603	rs4227540	9	6874936	A	A	A	A	A	A
11604	rs6235778	9	7428167	T	T	T	T	T	T
11605	mCV24996175	9	7670190	G	A	A	G	A	G
11606	mCV24996176	9	7670488	G	A	A	G	A	G
11607	mCV23201775	9	13255595	G	A	A	G	A	G
11608	mCV23201786	9	13256439	A	C	C	A	C	A
11609	mCV23202095	9	13260761	A	G	G	A	G	A
11610	rs3661469	9	7740845	T	A	A	A	A	A
11611	mCV23202466	9	13321398	G	C	C	G	C	G
11612	mCV23202474	9	13321426	A	T	T	A	T	A
11613	mCV24962297	9	13425598	A	G	G	A	G	A
11614	rs3727132	9	8116085	A	G	G	G	G	G
11615	rs3661373	9	8120246	G	A	A	A	A	A

11616	rs6155297	9	8299999	G	A	A	A	A	A
11617	rs3701093	9	9004816	T	A	A	A	A	A
11618	rs6351660	9	9365743	G	A	A	A	A	A
11619	rs3695693	9	9632007	C	G	G	G	G	G
11620	mCV24995380	9	9657199	G	A	A	A	A	A
11621	mCV23237360	9	9666027	G	A	A	A	A	A
11622	mCV25073238	9	10587580	A	G	G	A	G	A
11623	rs6196252	9	10778066	A	A	A	A	A	A
11624	rs6324914	9	11665259	C	C	C	C	C	C
11625	rs6285155	9	13034415	A	A	A	A	A	A
11626	rs3023202	9	13263667	A	G	G	A	G	A
11627	rs3718464	9	13295532	T	A	A	T	A	T
11628	rs6180788	9	13507391	C	C	C	C	C	C
11629	rs6240706	9	14322913	A	A	A	A	A	A
11630	rs8239692	9	14586513	A	A	A	A	A	A
11631	rs6290681	9	14868879	G	G	G	G	G	G
11632	rs4227548	9	14897728	G	G	G	G	G	G
11633	rs3089697	9	15387348	A	A	A	A	A	A
11634	rs3716850	9	16191378	A	G	G	G	G	A
11635	rs6394008	9	16282893	G	G	G	G	G	G
11636	rs6275116	9	16732952	G	G	G	G	G	G
11637	rs3721992	9	17245414	G	A	A	A	A	G
11638	rs4227551	9	17355435	A	A	A	A	A	A
11639	rs4227553	9	17355553	A	A	A	A	A	A
11640	rs6277772	9	17520764	C	C	C	C	C	C
11641	rs3700279	9	17641681	G	A	A	A	A	G
11642	rs6402130	9	18122743	A	A	A	A	A	A
11643	rs8274490	9	18181566	C	C	C	C	C	C
11644	rs8274473	9	18183224	G	G	G	G	G	G
11645	rs8274442	9	18197597	A	A	A	A	A	A
11646	rs8274446	9	18197810	A	A	A	A	A	A
11647	rs3722569	9	18820769	A	C	C	C	C	A
11648	rs6331746	9	18943675	G	G	G	G	G	G
11649	rs6297821	9	19810815	A	A	A	A	A	A
11650	rs3683132	9	20140587	G	A	A	A	A	A
11651	rs3665597	9	20424801	G	A	A	A	A	G
11652	rs3656141	9	20453835	G	A	A	A	A	G
11653	rs8266443	9	20576536	A	G	G	G	G	A
11654	rs8266450	9	20584236	G	G	G	G	G	G
11655	rs3666413	9	20620188	G	A	A	A	A	G
11656	rs8243217	9	20960384	A	A	A	A	A	A
11657	rs6167265	9	21059073	G	A	A	A	A	G
11658	rs8270115	9	21900659	G	G	G	G	G	A
11659	rs8236775	9	21901138	C	C	C	C	C	A
11660	rs8236776	9	21902397	G	G	G	G	G	G
11661	rs6266909	9	22219987	C	C	C	C	C	C
11662	rs4227556	9	22273214	A	A	A	A	A	A
11663	rs3713696	9	23438112	C	A	A	A	A	C
11664	rs6382323	9	23640125	G	A	A	A	A	G
11665	rs3691360	9	24033823	A	G	G	G	G	A
11666	rs6183014	9	24567282	G	G	G	A	G	G
11667	rs4227562	9	24675552	A	A	A	A	A	G
11668	rs3088801	9	24949186	A	A	A	A	A	G
11669	rs6217232	9	25098554	T	T	T	T	T	T
11670	rs6306091	9	26029136	T	T	T	T	T	T
11671	rs3686936	9	26774587	G	A	A	A	A	G
11672	rs3662999	9	26948960	A	C	C	C	C	A
11673	rs3702035	9	26950741	A	G	G	G	G	A
11674	rs6347341	9	27254683	A	A	A	A	A	A
11675	rs4227571	9	27307904	A	A	A	A	A	A
11676	rs6227937	9	28072195	T	T	T	T	T	T
11677	rs4227572	9	28950551	A	A	A	A	A	A
11678	rs3023203	9	29090669	A	A	A	A	A	G
11679	rs6385855	9	29608871	A	A	A	A	A	G
11680	mCV25302097	9	36463035	G	G	G	A	A	G
11681	rs6371905	9	30434840	A	A	A	A	A	A
11682	rs4227577	9	30439665	A	A	A	A	A	A
11683	rs4227580	9	30439860	A	A	A	A	A	A
11684	rs3685626	9	31189234	G	G	G	G	G	G
11685	rs6243527	9	31593862	A	A	A	A	G	A
11686	rs4227587	9	32741886	A	A	A	A	G	A
11687	rs3665206	9	32825296	A	A	A	G	G	G
11688	mCV24912785	9	39336987	T	T	T	T	A	A
11689	rs3704185	9	33290307	A	A	A	C	C	A

11690	rs3713675	9	33294662	G	G	G	A	G	G
11691	rs6406454	9	33369216	G	G	G	A	G	G
11692	rs3655898	9	33657166	G	G	G	A	G	G
11693	rs3686563	9	33776333	G	G	G	A	A	G
11694	rs6284286	9	34183554	C	C	C	A	C	C
11695	rs3711756	9	34437206	G	G	G	A	G	A
11696	rs6258765	9	34555460	C	C	C	A	C	C
11697	rs4139403	9	34622296	A	A	A	C	A	A
11698	rs3686022	9	35298490	G	G	G	A	G	G
11699	rs3090475	9	35340389	G	G	G	G	G	G
11700	rs3665911	9	35973861	A	A	A	G	A	A
11701	rs6186560	9	36098858	A	A	A	A	A	A
11702	rs3687584	9	36334827	G	G	G	A	G	G
11703	mCV22570522	9	43148138	A	A	A	G	A	G
11704	rs6309058	9	36752515	A	A	A	G	G	A
11705	rs3669224	9	37029046	G	G	G	A	G	G
11706	rs6207781	9	37083126	G	G	G	A	G	A
11707	mCV22343725	9	43675889	A	G	G	A	G	A
11708	rs3719607	9	37214680	C	C	C	A	C	A
11709	rs4227601	9	37237681	G	G	G	G	G	G
11710	rs3711100	9	37679640	C	C	C	G	G	C
11711	rs6413270	9	38721575	C	C	C	C	A	A
11712	rs8254842	9	38270131	C	C	C	C	C	C
11713	rs8254841	9	38270194	T	T	T	A	T	A
11714	rs3678183	9	38346628	A	A	A	G	A	G
11715	rs3688291	9	38393208	A	A	A	G	A	G
11716	rs3694949	9	38471539	G	G	G	A	G	A
11717	rs6198455	9	39010413	G	G	G	G	G	G
11718	rs6186807	9	39501003	A	A	A	A	G	G
11719	rs3675289	9	39726307	A	A	A	C	C	C
11720	rs6240566	9	40486634	A	A	A	G	A	G
11721	rs6373035	9	40512405	G	G	G	A	G	G
11722	rs3718089	9	40533954	A	A	A	G	A	G
11723	rs6266353	9	40676863	T	T	T	A	T	T
11724	rs3714664	9	40768895	A	A	A	G	A	G
11725	rs8239740	9	40868616	G	G	G	G	G	G
11726	rs8239688	9	40868764	A	A	A	A	A	A
11727	rs3023208	9	41188295	G	G	G	A	G	G
11728	rs3671538	9	41225623	G	G	G	C	G	G
11729	rs3707491	9	41233382	G	G	G	A	G	G
11730	rs6316763	9	41336433	G	G	G	C	G	G
11731	rs3722927	9	41741220	A	A	A	G	A	A
11732	rs3668547	9	41823689	A	A	A	G	A	A
11733	rs4227602	9	42067272	A	A	A	A	A	A
11734	rs4227605	9	42067448	T	T	T	T	T	T
11735	mCV25072221	9	49588552	A	G	G	A	G	A
11736	rs3702448	9	42134462	A	A	A	G	A	A
11737	rs3674874	9	42138499	C	A	A	C	C	A
11738	rs6388711	9	42335082	G	G	G	A	G	G
11739	rs3676086	9	42913532	A	G	G	G	A	G
11740	rs4135590	9	43060131	G	G	G	A	G	A
11741	rs6399406	9	43318395	G	A	A	G	A	G
11742	rs4135836	9	43354554	G	A	A	G	A	A
11743	rs3676087	9	43594065	A	G	G	G	G	G
11744	rs3653389	9	43942674	A	G	G	G	G	A
11745	rs8236788	9	44148217	G	G	G	G	G	G
11746	rs6400838	9	44302276	A	C	C	C	C	C
11747	mCV23098764	9	51873443	G	G	G	A	G	G
11748	rs8254820	9	44438572	A	A	A	A	A	A
11749	rs3696264	9	44630289	G	G	G	A	G	A
11750	rs3700385	9	45063906	G	G	G	A	G	G
11751	rs8259427	9	46778141	G	G	G	C	NONE	G
11752	rs6338678	9	45237003	A	T	T	T	T	T
11753	rs8275141	9	45278211	G	A	A	A	A	A
11754	rs8274947	9	45286427	G	G	G	G	G	G
11755	rs8274958	9	45286612	A	G	G	G	G	G
11756	rs6329880	9	45698924	G	A	A	A	A	A
11757	rs8254904	9	46333019	G	A	A	A	A	A
11758	rs8254905	9	46333043	G	A	A	A	A	A
11759	rs8254906	9	46333141	G	G	G	G	G	G
11760	rs8254929	9	46335107	G	A	A	A	A	A
11761	rs3693115	9	46665767	A	A	A	G	A	A
11762	rs6348780	9	46880329	G	A	A	G	A	A
11763	rs3673816	9	47336242	A	G	G	A	G	G

11764	rs6395817	9	47519917	A	G	G	A	G	G
11765	rs3710199	9	47719578	G	G	G	C	G	G
11766	rs6165968	9	47739976	G	G	G	A	G	G
11767	rs3661445	9	47889231	A	A	A	G	A	A
11768	rs3665709	9	48720327	G	G	G	A	G	G
11769	rs3684930	9	48879953	G	G	G	A	G	G
11770	rs3709825	9	49384481	A	G	G	A	G	A
11771	rs6186629	9	49983607	A	A	A	A	A	A
11772	rs3659951	9	50241775	A	G	G	A	G	NONE
11773	rs3710982	9	50720207	A	G	G	G	G	G
11774	rs4227637	9	50757239	G	G	G	G	G	G
11775	rs3693234	9	50942491	A	G	G	A	G	A
11776	rs6160923	9	51490019	G	A	A	A	A	A
11777	mCV25237837	9	59540846	A	A	A	G	A	A
11778	rs4138776	9	52212448	G	G	G	A	G	A
11779	rs3656996	9	52352560	G	G	G	A	G	A
11780	rs3723670	9	52682809	C	C	C	A	A	C
11781	rs6345786	9	52692404	C	C	C	A	A	C
11782	rs3654569	9	52718460	A	C	C	C	C	A
11783	rs4227649	9	52844633	G	G	G	G	G	G
11784	rs3659196	9	53433294	G	A	A	G	G	G
11785	rs6248743	9	53601731	A	A	A	A	A	A
11786	rs3658802	9	53613660	G	A	A	A	A	A
11787	rs3689326	9	54114690	A	A	A	G	A	A
11788	rs6379062	9	54349888	A	A	A	G	G	G
11789	rs8240045	9	54359512	G	G	G	G	G	G
11790	rs8259445	9	54360101	A	A	A	A	A	A
11791	rs8259447	9	54360105	G	G	G	A	A	A
11792	rs8259443	9	54360717	A	A	A	C	C	C
11793	rs3688570	9	54868302	A	G	G	G	A	G
11794	rs6206488	9	55121701	G	A	A	A	G	A
11795	rs3660104	9	55194665	G	A	A	A	G	A
11796	rs3089391	9	55507137	A	G	G	G	A/G	G
11797	rs4227674	9	55642459	A	A	A	A	A	A
11798	rs6206353	9	56118889	A	A	A	G	A	G
11799	mCV23531990	9	64058432	A	A	A	G	G	A
11800	rs3669687	9	56139893	G	G	G	A	G	G
11801	rs3653677	9	56142404	G	G	G	C	G	G
11802	rs6334600	9	56740468	G	G	G	C	G	G
11803	rs4135891	9	56899794	C	C	C	C	C	C
11804	rs3677551	9	56909480	C	C	C	A	C	A
11805	rs4136521	9	57468932	G	G	G	A	G	G
11806	mCV24747902	9	65674795	G	G	G	A	G	G
11807	mCV24747892	9	65674956	C	C	C	G	C	C
11808	mCV24747891	9	65674981	A	A	A	G	A	A
11809	mCV24747878	9	65676763	A	A	A	C	A	C
11810	mCV23286597	9	65680678	G	G	G	A	G	G
11811	mCV23286598	9	65685669	G	G	G	A	G	A
11812	mCV23286602	9	65686179	A	A	A	A	A	A
11813	mCV23286604	9	65686368	A	A	A	C	A	C
11814	mCV23286607	9	65686399	A	A	A	G	A	NONE
11815	mCV23286608	9	65687668	C	C	C	A	C	C
11816	mCV23286609	9	65688219	A	T	T	A	T	NONE
11817	mCV23286614	9	65688458	C	C	C	G	C	C
11818	mCV23286627	9	65689102	A	A	A	G	A	A
11819	mCV25157255	9	65692431	G	A	A	G	A	G
11820	mCV25157256	9	65693054	G	G	G	A	G	G
11821	mCV25157266	9	65693084	A	A	A	C	A	A
11822	rs3708863	9	57775693	A	A	A	G	A	A
11823	mCV22595554	9	65952568	T	A	A	A	T	A
11824	rs8236802	9	57886350	G	G	G	G	G	G
11825	rs8252951	9	57887915	A	A	A	A	A	A
11826	rs3696800	9	58145060	G	A	A	A	A	A
11827	rs3089971	9	58451224	A	A	A	A	A	A
11828	rs4227694	9	58470024	A	A	A	G	A	A
11829	rs4227700	9	59087089	A	A	A	G	A	A
11830	rs3668603	9	59119964	G	G	G	A	G	G
11831	rs3676114	9	59239123	G	G	G	A	G	A
11832	rs6154433	9	59398507	G	G	G	A	G	G
11833	rs3685575	9	59598759	A	A	A	G	A	A
11834	rs3671494	9	59871675	A	G	G	G	G	G
11835	rs6224703	9	60196412	G	G	G	A	G	G
11836	rs3664495	9	60295817	C	C	C	A	C	C
11837	rs3680060	9	60460530	A	A	A	C	C	A

11838	rs6358810	9	60714546	G	G	G	A	A	G
11839	rs3699230	9	61357334	A	A	A	G	G	A
11840	rs3714012	9	61377354	A	A	A	G	G	A
11841	rs6306396	9	61733262	G	G	G	A	A	G
11842	rs6249574	9	61940796	G	A	A	G	G	A
11843	rs3696688	9	62071779	A	A	A	G	G	A
11844	rs4227704	9	62605923	G	A	A	G	G	A
11845	rs4227706	9	62605978	G	G	G	A	G	G
11846	rs6172647	9	62742880	A	G	G	A	A	G
11847	rs3703593	9	62747939	G	G	G	C	C	G
11848	rs6359506	9	62882917	A	G	G	A	A	G
11849	rs4227717	9	63400816	G	G	G	G	G	G
11850	rs4227711	9	63401085	A	A	A	A	A	A
11851	rs6276212	9	63500929	G	A	A	G	G	A
11852	rs3656848	9	63587593	G	A	A	G	G	A
11853	rs3692389	9	63902859	G	G	G	A	A	G
11854	rs3090473	9	64225262	A	A	A	A	A	A
11855	rs3685822	9	64331117	A	A	A	C	A	A
11856	rs6305469	9	64578897	A	G	G	A	G	G
11857	rs3664300	9	64692206	A	G	G	A	G	G
11858	rs6195628	9	65200985	A	A	A	A	A	C
11859	rs4227726	9	65599173	C	C	C	C	C	C
11860	rs6361767	9	65686074	G	G	G	G	G	G
11861	rs3655717	9	65704935	G	G	G	A	G	G
11862	rs3658020	9	65748136	G	G	G	A	G	A
11863	rs3672857	9	65761685	G	G	G	A	G	G
11864	rs6374858	9	65789500	G	C	C	G	G	C
11865	rs3682157	9	66030736	T	A	A	A	T	NONE
11866	rs3664533	9	66052926	G	G	G	A	G	G
11867	rs3694989	9	66399440	G	G	G	A	G	G
11868	mCV25319520	9	74796462	G	G	G	G	G	G
11869	rs3714882	9	66443388	A	A	A	T	A	A
11870	rs4139637	9	66521461	A	G	G	A	G	G
11871	rs6247360	9	66958874	T	T	T	A	T	T
11872	rs3670579	9	67147243	G	C	C	C	C	C
11873	rs4227730	9	67268286	A	A	A	A	A	A
11874	rs4227736	9	67268694	A	A	A	A	A	G
11875	rs3089092	9	67308733	G	G	G	G	G	NONE
11876	rs3697853	9	67355255	A	G	G	A	G	G
11877	rs3658496	9	67860912	A	A	A	G	G	G
11878	rs6317714	9	67956830	G	A	A	A	A	G
11879	mCV25328470	9	76379295	G	A	A	A	A	A
11880	rs3716689	9	68356831	A	G	G	G	G	G
11881	rs6174757	9	68381485	A	A	A	C	C	A
11882	rs3703662	9	68586184	G	G	G	A	G	G
11883	rs3655098	9	68887196	G	C	C	G	C	C
11884	rs6395899	9	69143535	G	C	C	G	C	C
11885	rs130238	9	69144399	A	A	A	A	A	A
11886	rs3682714	9	69301407	A	A	A	T	A	A
11887	rs6212715	9	69375366	G	G	G	A	G	G
11888	rs3663861	9	69901619	G	A	A	A	A	A
11889	rs6355445	9	70209362	A	G	G	A	G	G
11890	rs4227744	9	70272017	A	A	A	A	A	A
11891	rs4227743	9	70272179	A	A	A	A	A	A
11892	rs3089222	9	70540782	A	A	A	A	A	A
11893	mCV22794972	9	79014269	C	A	A	C	A	A
11894	rs3703045	9	70566672	C	A	A	C	A	A
11895	mCV22793119	9	79178869	G	A	A	A	A	A
11896	rs6186431	9	70803602	A	A	A	A	A	A
11897	rs3721056	9	71328971	A	A	A	G	A	A
11898	rs3023216	9	71625312	G	A	A	A	A	A
11899	rs4227754	9	71824351	A	A	A	A	A	A
11900	rs6239320	9	71878535	T	T	T	A	T	T
11901	rs3707578	9	71895758	G	G	G	A	G	G
11902	rs3679620	9	71917482	A	A	A	G	A	A
11903	rs3693209	9	71973325	G	G	G	A	G	G
11904	rs3720204	9	72095202	G	A	A	G	A	A
11905	rs6283705	9	72271784	C	A	A	C	A	A
11906	rs3698342	9	72597929	G	A	A	G	A	A
11907	rs3711813	9	72680673	G	G	G	A	G	G
11908	rs3719772	9	72719122	G	G	G	A	G	G
11909	rs3724658	9	73062303	G	A	A	G	A	A
11910	rs3703712	9	73083979	G	A	A	G	A	A
11911	rs3665549	9	73383170	A	G	G	A	G	G

11912	rs6367765	9	73421223	G	G	G	G	G	G
11913	rs6167970	9	73839572	A	C	C	A	C	C
11914	rs3676158	9	73859220	C	G	G	C	G	G
11915	rs3670437	9	74428342	A	A	A	C	A	A
11916	rs4227780	9	74604963	A	A	A	A	A	A
11917	rs6314302	9	74938295	A	G	G	G	G	G
11918	rs3724833	9	75221496	A	A	A	G	A	A
11919	rs3700733	9	75334924	A	C	C	C	C	C
11920	rs6369641	9	75500918	G	A	A	A	A	A
11921	rs8240602	9	75516091	A	A	A	A	A	A
11922	rs4227782	9	75555536	G	G	G	G	G	G
11923	rs6292345	9	75634870	A	A	A	G	A	A
11924	rs3090482	9	75689615	A	A	A	A	A	A
11925	rs6192289	9	76006820	G	G	G	A	G	G
11926	rs4227795	9	76234862	G	G	G	G	G	G
11927	rs4227791	9	76235022	G	G	G	G	G	G
11928	rs6191894	9	76344735	C	C	C	A	C	C
11929	rs3089007	9	76514916	A	A	A	A	A	A
11930	rs3685573	9	76589717	A	A	A	G	A	A
11931	mCV25075527	9	85263991	A	A	A	A	G	A
11932	rs3675604	9	76824700	C	A	A	A	A	A
11933	rs6304125	9	76933084	A	A	A	A	A	A
11934	rs3089461	9	77270643	G	G	G	G	G	G
11935	rs3725904	9	77471557	G	G	G	A	G	G
11936	rs3674482	9	77705315	G	G	G	A	G	G
11937	rs6369096	9	77780769	C	C	C	A	C	C
11938	rs3693875	9	78291825	A	T	T	T	T	T
11939	rs6217039	9	78563841	G	A	A	G	A	A
11940	rs8261605	9	78564168	A	G	G	A	G	G
11941	rs8261641	9	78570042	A	A	A	A	A	A
11942	rs8261681	9	78574601	G	A	A	G	A	A
11943	rs6172659	9	78582684	A	G	G	A	G	G
11944	rs8257510	9	78717862	A	A	A	A	A	A
11945	rs6257131	9	79059621	G	A	A	A	A	A
11946	rs6210538	9	79240002	A	G	G	G	G	G
11947	rs3712939	9	79541430	T	A	A	A	A	A
11948	rs6302459	9	79977694	G	G	G	A	G	G
11949	rs6213724	9	79999697	A	G	G	A	G	G
11950	rs4222033	9	80172471	C	C	C	C	C	C
11951	rs3686004	9	80122666	A	A	A	C	A	A
11952	rs3654196	9	80569536	A	A	A	G	A	A
11953	rs6284095	9	80606017	A	G	G	A	G	G
11954	rs3679442	9	80623654	A	A	A	G	A	A
11955	rs3658458	9	80709081	A	A	A	G	A	A
11956	rs3023217	9	80713708	G	G	G	G	G	G
11957	rs6393229	9	81058179	G	G	G	G	G	G
11958	rs3700389	9	81179058	A	A	A	T	A	A
11959	rs3697374	9	81444528	T	T	T	A	T	T
11960	rs6165929	9	81635126	G	G	G	G	G	G
11961	rs3670195	9	81690623	A	A	A	G	A	A
11962	rs6223687	9	82203633	G	G	G	G	G	G
11963	rs3673457	9	82283038	A	A	A	G	A	A
11964	mCV23571381	9	85117045	A	A	A	A	A	A
11965	rs6293292	9	83174343	C	C	C	C	C	C
11966	rs4227805	9	83275045	T	T	T	T	T	T
11967	rs3676124	9	83548399	A	A	A	G	A	A
11968	rs3677217	9	83981176	G	G	G	A	G	G
11969	rs3672897	9	84122591	A	A	A	T	A	A
11970	rs3714992	9	84686121	A	A	A	C	C	C
11971	rs6256462	9	84805949	A	A	A	G	A	A
11972	rs6251253	9	84917229	A	A	A	C	A	A
11973	rs3677629	9	85083677	A	A	A	G	G	G
11974	mCV22800726	9	90628091	A	A	A	G	G	A
11975	rs3726941	9	85101889	A	A	A	G	G	G
11976	rs3673117	9	85508520	C	C	C	A	C	A
11977	rs3695050	9	85584599	G	A	A	G	G	A
11978	rs3674339	9	85923211	A	G	G	A	G	G
11979	rs3675152	9	86169729	G	A	A	A	A	G
11980	rs6226628	9	86286594	A	A	A	G	A	A
11981	rs3700596	9	86745093	G	G	G	A	G	G
11982	rs3720276	9	86752560	G	G	G	A	G	G
11983	rs3666862	9	87310147	G	G	G	A	A	A
11984	rs3698443	9	87500014	G	G	G	A	A	G
11985	rs6280990	9	87514406	G	G	G	A	G	G

11986	rs6182207	9	87881245	A	G	G	A	A	G
11987	mCV24311071	9	93538851	G	G	G	G	G	G
11988	rs3725046	9	88164692	G	A	A	G	G	A
11989	rs3088739	9	88312295	C	C	C	C	C	C
11990	rs3669564	9	88325825	C	C	C	A	C	C
11991	rs3705632	9	88415320	C	C	C	A	A	C
11992	rs6277880	9	88646923	A	A	A	G	A	A
11993	rs3718318	9	89140953	A	A	A	G	G	G
11994	rs6308983	9	89142606	A	A	A	G	G	G
11995	rs4227830	9	89904872	G	G	G	G	G	G
11996	rs4227835	9	89905068	G	G	G	G	G	G
11997	rs3693091	9	90235325	A	A	A	G	A	A
11998	rs6352812	9	90239768	A	A	A	A	G	A
11999	rs6289295	9	90877586	G	A	A	A	G	G
12000	mCV25074968	9	96790891	A	A	A	G	A	A
12001	rs6320356	9	90957478	A	A	A	A	G	G
12002	rs6333444	9	91230897	A	G	G	A	G	G
12003	rs6309331	9	92107776	A	T	T	A	A	A
12004	rs6292067	9	92933158	A	A	A	A	C	C
12005	mCV25152676	9	98797502	A	G	G	A	A	G
12006	rs6241554	9	93604175	G	G	G	G	G	G
12007	rs4138352	9	94154288	A	A	A	G	A	A
12008	rs3712946	9	94330869	A	A	A	G	A	A
12009	rs2020612	9	94702937	A	A	A	A	A	A
12010	rs3725272	9	95000478	C	A	A	C	A	C
12011	rs3690580	9	95205462	A	G	G	A	G	A
12012	rs3023143	9	95220853	C	A	A	C	A	C
12013	rs6286096	9	95314164	G	A	A	G	A	G
12014	rs3706741	9	95665360	G	A	A	A	A	A
12015	rs3673055	9	96176970	A	G	G	A	G	G
12016	rs3689336	9	96256381	G	G	G	A	G	G
12017	rs3703831	9	96256474	A	A	A	T	A	A
12018	mCV25070728	9	102264793	A	G	G	G	G	G
12019	rs3712719	9	96380935	A	C	C	C	C	C
12020	rs6244819	9	96752986	G	G	G	A	G	G
12021	rs3705403	9	97197984	C	C	C	A	C	C
12022	rs2020556	9	97236852	A	A	A	A	A	A
12023	rs3689043	9	99904554	A	A	A	G	A	A
12024	rs6353709	9	100011170	A	A	A	A	A	A
12025	rs3699740	9	98339365	A	A	A	G	A	A
12026	rs4227862	9	98426970	G	G	G	G	G	G
12027	rs3720103	9	98576264	A	G	G	A	G	G
12028	mCV22558129	9	104516123	A	A	A	G	A	A
12029	rs6385971	9	99710289	A	G	G	A	G	A
12030	rs3708760	9	99867547	A	G	G	A	A	A
12031	rs4227879	9	100304693	A	A	A	A	A	A
12032	rs4227882	9	100304772	G	G	G	G	G	G
12033	rs3722432	9	100525842	G	A	A	G	A	A
12034	rs6235582	9	100554543	A	G	G	A	G	G
12035	rs4227886	9	100640253	G	G	G	G	G	G
12036	rs3657562	9	100677183	A	C	C	A	C	C
12037	rs6377847	9	100692014	A	G	G	A	G	A
12038	rs3688878	9	101330986	A	A	A	G	A	A
12039	rs6190068	9	101511411	C	C	C	A	C	C
12040	rs3089531	9	101778520	A	A	A	G	A	A
12041	rs3717654	9	101878542	G	G	G	A	G	G
12042	rs3672865	9	101882437	A	A	A	G	A	G
12043	rs3717183	9	101885081	T	T	T	A	T	NONE
12044	rs6286924	9	101934650	A	A	A	G	A	G
12045	rs3706728	9	102349284	A	G	G	A	G	A
12046	rs3713420	9	102425391	C	A	A	A	A	A
12047	rs3707740	9	102618317	G	G	G	A	G	G
12048	rs3023229	9	102924025	C	C	C	C	C	C
12049	rs3657346	9	103089061	A	A	A	C	C	A
12050	rs4227888	9	103282438	T	T	T	T	T	T
12051	rs3690330	9	103410092	T	T	T	T	T	T
12052	rs3088463	9	103527865	A	G	G	A	A	A
12053	rs6267252	9	103599442	C	A	A	C	C	C
12054	rs6363194	9	103623096	A	G	G	A	A	A
12055	mCV24631499	9	109787632	G	A	A	G	A	G
12056	rs3718278	9	104040283	G	G	G	A	G	G
12057	rs4227892	9	104049462	A	A	A	A	A	A
12058	rs3727071	9	104254613	A	A	A	G	A	A
12059	rs3700120	9	104421801	A	A	A	G	A	A

12060	rs6231434	9	104964701	C	C	C	A	C	C
12061	rs3653967	9	104989749	G	G	G	A	G	G
12062	rs4227908	9	105069124	G	G	G	G	G	G
12063	rs3657881	9	105158595	T	T	T	A	T	T
12064	rs3671600	9	105198590	C	C	C	A	C	C
12065	rs4138027	9	105202621	G	G	G	A	G	G
12066	rs3711089	9	105418025	C	C	C	A	C	A
12067	rs6205631	9	105565710	G	G	G	A	G	G
12068	rs3669408	9	105603036	C	C	C	A	C	C
12069	rs6299717	9	105701193	G	G	G	A	G	G
12070	mCV22843303	9	111662570	C	C	C	A	C	C
12071	rs6340679	9	106384935	A	A	A	G	A	A
12072	rs3655712	9	106689165	G	G	G	A	A	G
12073	rs3665190	9	106799213	A	A	A	G	G	A
12074	rs3691204	9	106908224	G	G	G	A	A	G
12075	rs6261192	9	107298613	A	A	A	G	G	A
12076	rs3668362	9	107307044	A	A	A	T	T	A
12077	rs4227914	9	107320673	A	A	A	A	A	A
12078	rs3681245	9	107346437	G	G	G	A	A	G
12079	rs3708144	9	107502860	G	G	G	A	A	G
12080	rs3671576	9	107527677	C	C	C	A	A	C
12081	rs8259500	9	107590813	G	G	G	A	A	G
12082	rs8259503	9	107590963	A	A	A	G	G	A
12083	rs3661532	9	107725331	G	G	G	A	A	G
12084	rs3676408	9	107853273	T	T	T	A	T	T
12085	rs6366991	9	108037887	A	G	G	G	A	G
12086	rs6389763	9	108072043	A	G	G	G	A	G
12087	rs8240622	9	108105904	C	A	A	A	A	A
12088	mCV23771355	9	110866653	C	A	A	C	C	A
12089	rs6253244	9	108807147	A	G	G	A	G	G
12090	rs3685576	9	109064188	G	G	G	A	G	A
12091	rs3702106	9	109071202	C	C	C	A	C	A
12092	mCV23375261	9	111709568	G	A	A	G	G	A
12093	rs3655948	9	109708632	C	C	C	A	C	C
12094	rs4227919	9	110468650	G	G	G	G	G	G
12095	rs4227917	9	110468739	A	A	A	A	A	A
12096	rs6285142	9	110973008	C	C	C	A	C	C
12097	rs8240355	9	111022020	G	G	G	G	G	G
12098	rs8240419	9	111032474	A	A	A	G	A	A
12099	rs3723953	9	111041625	A	A	A	G	A	A
12100	rs8254352	9	111275889	A	G	G	G	G	G
12101	rs3703121	9	111357982	A	G	G	G	G	G
12102	rs6405284	9	111404794	A	T	T	T	T	T
12103	rs6248694	9	111966801	G	G	G	A	G	G
12104	rs3720785	9	111972599	C	C	C	A	C	C
12105	rs6242686	9	112149591	A	A	A	A	A	A
12106	rs3665498	9	112450191	G	G	G	A	A	G
12107	rs3672262	9	112664623	G	A	A	A	A	A
12108	rs6380928	9	113191768	G	G	G	G	G	G
12109	rs3690992	9	113881939	G	G	G	A	G	G
12110	rs4227931	9	113916659	A	A	A	A	A	A
12111	rs4227928	9	113916734	G	G	G	G	G	G
12112	rs3721068	9	113930964	A	A	A	G	G	A
12113	rs3694903	9	114041353	G	G	G	C	G	G
12114	rs6346740	9	114336882	G	G	G	G	G	G
12115	rs6328098	9	114441364	G	G	G	G	G	G
12116	rs4227954	9	114685409	G	G	G	G	G	G
12117	rs3711381	9	115080908	G	A	A	A	A	A
12118	rs6320810	9	115090216	G	G	G	A	A	G
12119	rs4227969	9	115179605	A	A	A	G	G	G
12120	rs3678712	9	115540344	A	A	A	T	T	A
12121	rs6298993	9	115696696	G	G	G	G	G	G
12122	mCV23808189	9	120804310	A	G	G	A	G	G
12123	rs3681899	9	115918856	A	G	G	G	G	G
12124	rs4227976	9	116046157	G	G	G	G	G	G
12125	rs3720706	9	116054148	G	A	A	G	G	A
12126	rs6215614	9	116335300	A	A	A	A	A	A
12127	rs3680245	9	116463718	G	A	A	A	A	A
12128	rs3658244	9	116543959	A	G	G	A	A	G
12129	rs3693201	9	116798643	A	C	C	A	A	C
12130	rs3671909	9	116957934	A	G	G	G	G	G
12131	rs3696476	9	117081293	G	A	A	G	G	A
12132	mCV24683863	9	122369372	G	G	G	G	G	G
12133	rs6249627	9	117535031	A	A	A	A	A	A

12134	rs3678853	9	117710979	A	C	C	C	C	C
12135	rs3677250	9	117806172	A	G	G	A	A	G
12136	rs3669563	9	117891342	A	A	A	G	G	A
12137	rs6274904	9	118154725	G	G	G	G	G	G
12138	rs3680940	9	118666767	A	G	G	A	A	G
12139	rs6237606	9	118750611	A	A	A	A	A	A
12140	rs6285865	9	118792737	A	A	A	C	C	A
12141	rs6316481	9	118944837	T	T	T	A	A	T
12142	rs4227998	9	119163545	G	G	G	G	G	G
12143	rs8254361	9	119308274	A	C	C	A	C	C
12144	rs8254358	9	119308443	G	A	A	G	A	A
12145	rs8254357	9	119309441	G	G	G	G	G	G
12146	rs3700085	9	119643381	A/C	C	C	C	C	C
12147	rs4228038	9	119789950	G	G	G	G	G	G
12148	rs6180694	9	120895707	C	C	C	C	C	C
12149	rs3687724	9	120914957	A	G	G	A	A	G
12150	rs6276199	9	121648735	A	A	A	A	A	A
12151	rs6292645	9	121676387	G	A	A	G	G	A
12152	rs4228060	9	121753114	G	G	G	G	G	G
12153	rs6241431	9	122806811	C	C	C	C	C	C
12154	rs4228067	9	123028091	A	A	A	A	A	A
12155	rs3088537	9	123127141	A	A	A	A	A	A
12156	rs3687856	9	123188943	A	A	A	G	G	A
12157	rs3664397	9	123220498	A	A	A	G	G	A
12158	rs3665591	9	123220710	G	G	G	A	A	G
12159	rs3692532	9	123225657	G	G	G	A	A	G
12160	rs3089651	9	123342714	G	G	G	G	G	G
12161	rs8241505	9	123806729	G	A	A	A	A	A
12162	rs6365534	9	123812730	G	A	A	A	A	A
12163	rs8254393	9	123978813	G	A	A	A	G	A
12164	rs8254392	9	123979445	G	A	A	A	G	A
12165	rs6227097	9	124046609	T	A	A	A	T	A
12166	rs8254378	9	124140321	A/G	G	G	G	A	A/G
12167	rs3659525	X	323512	A	G	G	A	A	A
12168	rs3655710	X	3026	A	A	A	G	A	A
12169	rs6322925	X	45764783	G	C	C	G	C	G
12170	rs3725526	X	4019653	G	G	A	A	A	A
12171	rs6305735	X	4121494	A	A	A	A	A	A
12172	rs4232460	X	4602733	T	T	T	T	T	T
12173	rs6209973	X	5164816	G	G	G	G	G	G
12174	rs3698666	X	5556366	A	A	A	A	A	A
12175	rs6268289	X	5580451	G	G	G	G	G	G
12176	rs6410628	X	6287945	A	A	A	A	A	A
12177	rs3684547	X	7768932	A	G	A	G	A	G
12178	rs4232464	X	7770032	G	G	G	G	G	G
12179	rs4232466	X	7987141	A	A	A	A	A	A
12180	rs4232467	X	7987243	G	G	G	G	G	G
12181	rs6160438	X	8094879	G	G	G	G	G	G
12182	rs3722225	X	8183309	G	A	G	A	G	A
12183	rs3724552	X	8183646	G	A	G	A	G	A
12184	rs6256688	X	8927969	G	G	G	G	G	G
12185	rs4232475	X	8928150	T	T	T	T	T	T
12186	rs6303814	X	10034187	A	A	A	A	A	A
12187	rs6181710	X	10550753	G	G	G	G	G	G
12188	rs4223180	X	10984313	C	C	C	C	C	C
12189	rs6360192	X	11041276	A	A	A	A	A	A
12190	rs6244254	X	12446718	A	A	A	A	A	A
12191	rs6366883	X	12885128	A	A	A	A	A	A
12192	rs3694204	13	96162947	C	A	A	C	A	A
12193	rs3088943	X	13668644	A	A	A	A	A	A
12194	rs3088942	X	13668704	C	C	C	C	C	C
12195	rs6247454	X	14097107	A	A	A	A	A	A
12196	rs6261700	X	14749753	A	A	A	A	A	A
12197	rs6176724	X	15038264	G	G	G	G	G	G
12198	rs6278020	X	15967319	C	C	C	C	C	C
12199	rs6235400	X	17376770	G	G	G	G	G	G
12200	rs6285009	X	18149217	A	A	A	A	A	A
12201	rs6237014	X	18694367	G	G	G	G	G	G
12202	rs4232480	X	18851169	G	G	G	G	G	G
12203	rs4232479	X	18851326	A	A	A	A	A	A
12204	rs6412008	X	19669478	G	G	G	G	G	G
12205	rs4232488	X	19819966	T	T	T	T	T	T
12206	rs6249754	X	20250694	A	A	A	A	A	A
12207	rs6366200	X	20527383	G	G	G	G	G	G

12208	rs2020849	X	21163322	G	G	G	G	G	G
12209	rs6264998	X	22051500	A	A	A	A	A	A
12210	rs6259015	X	28890781	A	A	A	A	A	A
12211	rs8237641	X	29329656	A	A	A	A	A	A
12212	rs8254500	X	29340834	A	A	A	A	A	A
12213	rs8245805	X	29346067	C	C	C	C	C	C
12214	rs8245806	X	29346131	G	G	G	G	G	G
12215	rs8245822	X	29349855	G	G	G	G	G	G
12216	rs6203233	X	29711369	G	G	G	G	G	G
12217	rs6319168	X	30574121	A	A	A	A	A	A
12218	rs6238662	X	30872802	A	A	A	A	A	A
12219	rs6394815	X	31835251	G	G	G	G	G	G
12220	rs6224144	X	33417227	G	G	G	G	G	G
12221	rs6208314	X	33915030	G	G	G	G	G	G
12222	rs6202387	X	34635795	A	A	A	A	A	A
12223	rs6185184	X	35688896	A	A	A	A	A	A
12224	rs6372074	X	36481053	C	C	C	C	C	C
12225	rs6282846	X	37138460	C	C	C	C	C	C
12226	rs6210474	X	38984640	T	T	T	T	T	T
12227	rs6311930	X	39669061	A	A	A	A	A	A
12228	rs6168118	X	40446247	G	G	A	A	A	G
12229	rs3089430	X	39426983	A	A	A	A	A	A
12230	rs8276250	X	40459215	G	G	G	G	G	G
12231	rs8276255	X	40460372	A	A	A	G	A	A
12232	rs8276067	X	40461839	G	G	A	A	A	G
12233	rs8276411	X	40484117	A	A	A	A	A	A
12234	rs6304294	X	41333663	G	G	G	G	G	G
12235	rs3699591	X	42140821	G	G	G	A	G	G
12236	rs6299349	X	42234387	A	A	A	A	A	A
12237	rs6251933	X	43423297	A	A	A	A	A	A
12238	rs6373566	X	43897207	A	A	A	A	A	A
12239	rs3682875	X	44211705	G	G	G	A	G	G
12240	rs3720681	X	44249136	A	G	G	A	G	A
12241	rs6243929	X	44644574	G	G	G	G	G	G
12242	rs3691506	X	44833514	G	A	A	A	A	A
12243	rs6205800	X	45100789	G	A	A	G	A	A
12244	rs6270558	X	48137806	A	A	A	A	A	A
12245	rs6251042	X	48436858	G	G	G	G	G	G
12246	rs6368704	X	49520093	G	G	G	A	G	G
12247	rs6410633	X	50110770	A	A	A	A	A	A
12248	rs6214689	X	50896519	G	G	G	G	G	G
12249	rs6412756	X	51804986	C	C	C	C	C	C
12250	rs6349431	X	52466625	A	A	A	A	A	A
12251	rs6357136	X	53852462	G	G	G	G	G	G
12252	rs6332369	X	55095760	G	G	G	G	G	G
12253	rs6356494	X	55624743	G	G	G	G	G	G
12254	rs3695410	X	57115208	G	A	A	G	G	G
12255	rs6193526	X	57118796	A	A	A	A	A	A
12256	rs6153159	X	57758758	T	T	T	T	T	T
12257	rs6179494	X	58243988	A	A	A	A	A	A
12258	rs3689473	X	58540368	G	A	A	G	G	G
12259	rs4138351	X	59220713	G	A	A	G	G	G
12260	rs6247207	X	59416019	G	G	G	G	G	G
12261	rs6378383	X	60032081	G	G	G	G	G	G
12262	rs6243951	X	60955670	A	A	A	A	A	A
12263	rs3708247	X	61074448	A	T	T	T	A	A
12264	rs3700359	X	61078223	A	G	G	G	A	A
12265	rs6211695	X	62622256	G	G	G	G	G	G
12266	rs3157124	X	63007260	G	G	G	A	G	G
12267	rs6404038	X	63043697	C	C	C	A	C	C
12268	rs6312087	X	63487587	C	C	C	C	C	C
12269	rs6185692	X	64346530	G	G	G	G	G	G
12270	rs6369810	X	64381578	G	G	G	G	G	G
12271	rs3683005	X	64457236	A	A	A	A	A	A
12272	rs4232509	X	64471965	G	G	G	G	G	G
12273	rs8238970	X	64473794	A	A	A	A	A	A
12274	rs8251007	X	64477468	G	A	A	G	A	A
12275	rs4232517	X	65052810	A	A	A	A	A	A
12276	rs6231337	X	65954887	A	A	A	A	A	A
12277	rs6218005	X	66032641	G	G	G	G	G	G
12278	rs6249264	X	67333091	G	G	G	G	G	G
12279	rs6265042	X	67439004	C	A	A	A	A	C
12280	rs6344525	X	67564830	A	A	A	A	A	A
12281	rs4232527	X	68382081	G	G	G	G	G	G

12282	rs6400779	X	68522167	G	G	G	G	G	G
12283	rs3663719	X	69021574	A	A	A	T	A	A
12284	rs6279227	X	69613227	A	A	A	A	A	A
12285	rs6212751	X	70156558	A	A	A	A	A	A
12286	rs6399914	X	70953585	G	G	G	G	G	G
12287	rs6208405	X	71331368	G	G	G	G	G	G
12288	rs6268105	X	72219902	G	G	G	G	G	G
12289	rs6271217	X	72886989	A	A	A	A	A	A
12290	rs3676526	X	73048650	G	A	A	G	A	G
12291	rs6291695	X	74274957	G	G	G	G	G	G
12292	rs6389792	X	75325806	G	G	G	G	G	G
12293	rs6323789	X	75999142	A	A	A	A	A	A
12294	rs6311059	X	76222069	G	G	G	G	G	G
12295	rs3675552	X	76437616	A	C	C	A	C	A
12296	rs3716132	X	76872187	A	A	A	G	A	G
12297	rs3664154	X	77052880	A	A	A	G	A	G
12298	rs6199233	X	77419608	G	G	G	G	G	G
12299	rs6214040	X	77911482	G	G	G	G	G	G
12300	rs3696812	X	78059793	G	G	G	A	G	A
12301	rs6227787	X	78650357	A	A	A	A	A	A
12302	rs6340046	X	79946815	A	A	A	A	A	A
12303	rs6315877	X	81805810	A	A	A	A	A	A
12304	rs3090929	X	82281249	A	A	A	A	A	A
12305	rs6293628	X	82599410	C	C	C	C	C	C
12306	rs6403206	X	82804409	A	A	A	A	A	A
12307	rs6190600	X	84111138	A	A	A	A	A	A
12308	rs3702254	X	84621713	A	G	G	G	A	G
12309	rs8266078	X	84857768	A	A	A	A	A	A
12310	rs8266131	X	84927623	G	G	G	G	G	G
12311	rs8277807	X	85079616	A	A	A	A	A	A
12312	rs8277708	X	85125811	G	G	G	G	G	G
12313	rs8277723	X	85135895	A	G	G	A	A	G
12314	rs8277725	X	85150347	A	A	A	A	A	A
12315	rs6274287	X	85251266	A	A	A	A	A	A
12316	rs3669459	X	85285266	A	G	G	A	A	G
12317	rs3676174	X	86083993	A	G	G	A	A	G
12318	rs3712243	X	86175046	T	A	A	T	T	A
12319	rs6407378	X	86188534	A	A	A	A	A	A
12320	rs6173395	X	86598828	A	A	A	A	A	A
12321	rs6380171	X	87436208	G	G	G	G	G	G
12322	rs3687491	X	87673077	G	G	G	G	G	G
12323	rs6367841	X	88740343	A	A	A	A	A	A
12324	rs6282046	X	89056545	G	G	G	G	G	G
12325	rs6344199	X	89607388	A	A	A	A	A	A
12326	rs3672400	X	89680695	A	G	G	A	A	G
12327	rs3680829	X	90298561	A	T	T	A	A	T
12328	rs6287259	X	90765647	A	A	A	A	A	A
12329	rs8278084	X	90871590	G	G	G	G	G	G
12330	rs8278085	X	90872000	A	A	A	A	A	A
12331	rs6272170	X	91795980	T	T	T	T	T	T
12332	rs8278385	X	92090492	G	A	A	G	G	A
12333	rs8278486	X	92094330	C	A	A	C	C	A
12334	rs8278502	X	92095730	G	A	A	G	G	A
12335	rs6217048	X	92193500	G	G	G	G	G	G
12336	rs3090974	X	92668315	G	G	G	G	G	G
12337	rs4232609	X	92671509	G	G	G	G	G	G
12338	rs8238299	X	92788031	A	A	A	A	A	A
12339	rs8238300	X	92788590	G	G	G	G	G	G
12340	rs6180194	X	93306912	G	G	G	G	G	G
12341	rs3090486	X	95040886	G	G	G	G	G	G
12342	rs6213730	X	95097387	G	G	G	A	A	G
12343	rs4232613	X	95102882	A	A	A	A	A	A
12344	rs8268113	X	95693015	A	A	A	A	A	A
12345	rs8268133	X	95694824	C	C	C	C	C	C
12346	rs8260035	X	95741889	G	G	G	G	G	G
12347	rs6207541	X	97281222	G	G	G	G	G	G
12348	rs4232616	X	97426331	A	A	A	A	A	A
12349	rs8239020	X	97431378	A	A	A	A	A	A
12350	rs3700575	X	97504542	A	A	A	G	G	A
12351	rs6245485	X	97949296	A	A	A	A	A	A
12352	rs6261315	X	98329232	G	G	G	G	G	G
12353	rs6303914	X	99129228	A	A	A	A	A	A
12354	rs3654491	X	99937059	G	A	A	A	G	A
12355	rs6235879	X	100138255	G	G	G	G	G	G

12356	rs6364201	X	101040049	G	G	G	G	G	G
12357	rs6251964	X	101963612	A	A	A	A	A	A
12358	rs6227618	X	102185100	G	G	G	G	G	G
12359	rs3722609	X	103044021	C	C	C	C	C	C
12360	rs3698779	X	103921813	G	A	A	G	G	G
12361	rs3702093	X	103983957	A	G	G	A	A	A
12362	rs3719513	X	105267232	G	A	A	G	G	G
12363	rs6209136	X	105322979	G	A	A	G	G	G
12364	rs3674730	X	105690084	A	G	G	A	A	A
12365	rs6231649	X	105888859	G	A	A	G	G	G
12366	rs3704435	X	105967256	A	G	G	A	A	A
12367	rs3667940	X	106877781	G	A	A	G	G	G
12368	rs6413032	X	107019102	A	G	G	A	A	A
12369	rs3665909	X	107631092	A	G	G	A	A	A
12370	rs6166961	X	107707479	A	C	C	A	A	A
12371	rs3690372	X	108243347	A	A	A	G	A	A
12372	rs6356774	X	108350171	C	A	A	C	C	C
12373	rs3681080	X	108372419	A	C	C	A	A	A
12374	rs3686168	X	108583426	A	G	G	A	A	A
12375	rs3692970	X	108588883	A	G	G	A	A	A
12376	rs6332633	X	109372295	T	A	A	T	T	T
12377	rs3718567	X	109934187	G	A	A	G	G	G
12378	rs6217100	X	110493947	A	A	A	A	A	A
12379	rs3669031	X	111629265	A	T	T	A	A	A
12380	rs6196188	X	111727814	G	A	A	G	G	G
12381	rs6327834	X	112407503	A	G	G	A	A	A
12382	rs3658628	X	112763067	C	G	G	C	C	C
12383	rs6263351	X	113018181	A	G	G	A	A	A
12384	rs6172859	X	113940128	A	G	G	A	A	A
12385	rs3713761	X	117241451	A	C	C	A	A	A
12386	rs6314203	X	117288552	A	C	C	A	A	A
12387	rs6284340	X	117403686	G	A	A	G	G	G
12388	rs3709341	X	117667890	A	T	T	A	A	A
12389	rs3670542	X	118051351	G	A	A	G	G	G
12390	rs6210352	X	118105347	A	G	G	A	A	A
12391	rs6211820	X	119191784	A	A	A	A	A	A
12392	rs6202386	X	120299930	A	A	A	A	A	A
12393	rs6401675	X	120479693	G	G	G	A	G	G
12394	rs6339460	X	121368058	A	A	A	A	A	A
12395	rs3666174	X	122115392	A	A	A	C	C	A
12396	rs6195283	X	122179567	A	A	A	A	A	A
12397	rs6173173	X	123293483	A	A	A	A	A	A
12398	rs3661602	X	123615734	A	A	A	G	A	A
12399	rs6245373	X	123718692	A	A	A	A	A	A
12400	rs6299904	X	124020890	A	A	A	A	A	A
12401	rs8237197	X	124598445	C	C	C	C	C	C
12402	rs8237208	X	124602712	A	A	A	C	A	C
12403	rs6302291	X	124752720	G	G	G	G	G	G
12404	rs3702256	X	124956381	G	G	G	A	G	A
12405	rs3718396	X	125967038	G	G	G	G	G	G
12406	rs4232640	X	126051157	G	G	G	G	G	G
12407	rs4151956	X	126221781	G	A	G	A	G	A
12408	rs6393507	X	126654152	A	A	A	A	A	A
12409	rs8238559	X	126843634	G	G	G	G	G	G
12410	rs6268212	X	127461421	G	G	G	G	G	G
12411	rs6365530	X	128389653	C	C	C	C	C	C
12412	rs6254399	X	129576454	A	A	A	A	A	A
12413	rs6271124	X	129703240	G	G	G	G	G	G
12414	rs6253377	X	130971147	A	A	A	A	A	A
12415	rs8255331	X	131613260	G	G	G	G	G	G
12416	rs3719765	X	132305239	A	G	G	G	G	A
12417	rs6372196	X	132644145	A	A	A	A	A	A
12418	rs6392118	X	132999972	C	C	C	C	C	C
12419	rs6344174	X	134017418	G	G	G	G	G	G
12420	rs6365160	X	134443677	A	A	A	A	A	A
12421	rs3702041	X	135149039	A	G	G	G	G	G
12422	rs6379275	X	136013589	A	A	A	A	A	A
12423	rs6366047	X	136273470	C	C	C	C	C	C
12424	rs6397905	X	136818200	A	A	A	A	A	A
12425	rs2020694	X	136963817	A	A	A	A	A	A
12426	rs6313974	X	137455738	A	A	A	A	A	A
12427	rs6269954	X	140307480	C	C	C	C	C	C
12428	rs3662037	X	140716207	A	G	A	A	A	G
12429	rs4232650	X	140790711	G	G	G	G	G	G

12430	rs6160268	X	141360024	G	G	G	G	G	G
12431	rs6172731	X	141807892	A	A	A	A	A	A
12432	rs3685112	X	143539941	A	A	A	G	A	A
12433	rs6291762	X	144704684	G	G	G	G	G	G
12434	rs8255346	X	145129965	A	A	A	A	A	A
12435	rs8255343	X	145131075	G	G	G	G	G	G
12436	rs3705296	X	145632182	T	A	T	T	T	T
12437	rs6219654	X	145708456	G	G	G	G	G	G
12438	rs6339238	X	146512632	G	G	G	G	G	G
12439	rs8255374	X	147416073	G	A	G	G	G	G
12440	rs8255372	X	147416448	A	C	A	A	A	A
12441	rs8255364	X	147416946	G	G	G	G	G	G
12442	rs8255428	X	145493676	A	A	A	A	A	A
12443	rs6213362	X	147525211	A	A	A	A	A	A
12444	rs3716904	X	147688794	G	G	G	G	G	G
12445	rs6225166	X	147791915	G	G	G	G	G	G
12446	rs4135560	X	148598549	G	G	G	G	G	G
12447	rs3722862	X	148598629	A	A	A	A	A	A
12448	rs6280000	X	148820241	A	A	A	A	A	A
12449	rs6292190	X	149281267	G	G	G	G	G	G
12450	rs3667080	X	149912282	C	C	C	C	C	C
12451	rs6154628	X	150498545	G	G	G	G	G	G
12452	rs6156461	X	151286979	A	A	A	A	A	A
12453	rs3719291	X	151342239	G	G	G	G	G	G
12454	rs3719368	X	151342278	A	A	A	A	A	A
12455	rs6410946	X	151695941	C	C	C	C	C	C
12456	rs3725586	X	152064890	C	C	C	C	C	C
12457	rs6380375	X	152603815	A	A	A	A	A	A
12458	rs6159029	X	153185456	C	C	C	C	C	C
12459	rs3090488	X	153519220	G	G	G	G	G	G
12460	rs2020547	X	154096126	C	C	C	C	C	C
12461	rs6404010	X	154136916	A	A	A	A	A	A
12462	rs4136868	X	154483405	G	G	G	A	G	G
12463	rs3721473	X	154515779	A	A	A	G	A	A
12464	rs6362954	X	154541294	A	A	A	G	A	A
12465	rs6229527	X	154972568	A	A	A	A	A	A
12466	rs8255442	X	155223720	A	A	A	A	A	A
12467	rs3715986	X	155494737	C	C	C	A	C	C
12468	rs6181649	X	156091461	G	G	G	G	G	G
12469	rs6237998	X	156523757	C	C	C	C	C	C
12470	rs3693969	X	157230117	G	G	G	A	G	G
12471	rs6299123	X	157710332	G	G	G	G	G	G
12472	rs6216139	X	158229294	G	G	G	G	G	G
12473	rs6272251	X	159085629	A	A	A	A	A	A
