# Supplementary material for: DNA variation and brain region-specific expression profiles exhibit different relationships between inbred mouse strains: implications for eQTL mapping studies
Source: Genome Biol. 2007 Feb 26;8(2):R25. doi: 10.1186/gb-2007-8-2-r25 (PMC1852412; doi:10.1186/gb-2007-8-2-r25)
Supplement: Additional data file 5 — This file contains the genomic start and end positions of genes used in the eQTL analysis. [file gb-2007-8-2-r25-S5.pdf]

Additional data file 5. Genomic start and end positions of genes used in the eQTL analysis.

Gene #	ProbeSet	Category	Gene Symbol	EntrezID	Chromosome	Start	End	Orientation
1	1436066_at	Region	Kalrn	545156	16	32822253	32909981	-
2	1450996_at	Region	Fshb	14308	2	106679927	106683438	-
3	1438968_x_at	Region	Spint2	20733	7	24665189	24693522	-
4	1448647_at	Region	Man2a1	17158	17	62303281	62457082	+
5	1418301_at	Region	Irf6	54139	1	192891918	192910770	+
6	1434665_at	Region	Aga	11593	8	52476596	52488292	+
7	1421812_at	Region	None	None	17	31623289	31633036	+
8	1426972_at	Region	Sec24d	69608	3	122059142	122157282	+
9	1425974_a_at	Region	Trim25	217069	11	88820567	88841380	+
10	1440371_at	Region	None	None	X	151414698	151415426	-
11	1442113_at	Region	5330417C22Rik	229722	3	108251436	108332283	-
12	1426602_at	Region	Araf	11836	X	19087538	19099458	+
13	1452861_at	Region	2010300C02Rik	72097	1	37906151	38014285	-
14	1434745_at	Region	Ccnd2	12444	6	127802641	127824339	-
15	1448188_at	Region	Ucp2	22228	7	94600035	94608691	+
16	1424797_a_at	Region	Pitx2	18741	3	128131958	128151601	+
17	1435198_at	Region	LOC545228	545228	17	83993299	83995933	-
18	1460351_at	Region	S100a11	20195	3	93237660	93243431	+
19	1427178_at	Region	Tmc4	353499	7	352	3277	-
20	1417010_at	Region	Zfp238	30928	1	177382318	177388421	+
21	1433652_at	Region	Igsf1	209268	X	44303340	44318535	-
22	1434653_at	Region	Ptk2b	19229	14	60680682	60808472	-
23	1428116_a_at	Region	Tctex1	21648	6	136109838	136110544	-
24	1437046_x_at	Region	4930504E06Rik	75007	3	94770775	94783992	+
25	1450468_at	Region	Myoc	17926	1	162548600	162559126	+
26	1430127_a_at	Region	Ccnd2	12444	6	127802641	127824339	-
27	1460569_x_at	Region	Cldn3	12739	5	133999706	134000964	+
28	1423909_at	Region	0610011I04Rik	66058	6	48974286	48979812	+
29	1416122_at	Region	Ccnd2	12444	6	127802641	127824339	-
30	1430133_at	Region	4921505D17Rik	245638	X	133264640	133329485	+
31	1460591_at	Region	Esr1	13982	10	5503826	5892641	-
32	1416210_at	Region	Imp3	102462	9	57050989	57051887	+
33	1423948_at	Region	Bag2	213539	1	34014433	34026712	-
34	1424650_at	Region	Pdia5	72599	16	34186826	34272549	-
35	1452381_at	Region	Creb3l2	208647	6	37465296	37576712	-
36	1421088_at	Region	Gpc4	14735	X	46573716	46686048	-
37	1416687_at	Region	Plod2	26432	9	92437061	92503040	+
38	1429175_at	Region	2810417M05Rik	68027	17	78777250	78834122	+
39	1436408_at	Region	None	None	11	103470663	103471671	+
40	1427005_at	Region	Plk2	20620	13	106643742	106649524	+
41	1436796_at	Region	1110061A14Rik	68827	18	35777080	35781982	+
42	1419608_a_at	Region	Mia1	12587	7	22553265	22554674	-
43	1448698_at	Region	Ccnd1	12443	7	139319781	139352397	-
44	1449159_at	Region	Gnb3	14695	6	125491008	125497043	-
45	1416579_a_at	Region	Tacstd1	17075	17	85492970	85507589	+
46	1424089_a_at	Region	Tcf4	21413	18	69575005	69915006	+
47	1449555_a_at	Region	Fetub	59083	16	21703696	21724542	+
48	1418367_x_at	Region	Hist2h2aa1 /// Hist1h2ad /// Hist1h2an /// Hist2h2ab /// Hist2h2ac /// Hist2h2aa2	15267	3	95727548	95728125	-
49	1440418_at	Region	None	None	16	74494174	74494867	-
50	1433602_at	Region	Gabra5	110886	7	51680057	51782608	-
51	1420907_at	Region	Cd2ap	12488	17	40292779	40376218	-
52	1455819_at	Region	Rod1	230257	4	59418515	59492735	-
53	1416686_at	Region	Plod2	26432	9	92437061	92503040	+
54	1434800_at	Region	Sv2b	64176	7	69011039	69108389	-
55	1418223_at	Region	Sec11l1	56529	7	74718079	74750250	-
56	1425284_a_at	Region	Rab27a	11891	9	73177546	73230218	+
57	1448255_a_at	Region	Surf4	20932	2	26852201	26866090	-

58	1419221_a_at	Region	Rgs14	51791	13	53979045	53993961	+
59	1449123_at	Region	Itih3	16426	14	29040621	29055592	-
60	1418549_at	Region	Cga	12640	4	35032846	35046441	+
61	1417189_at	Region	Psme2	19188	14	50105458	50109026	-
62	1448372_a_at	Region	Tmem4	56530	10	128059278	128064006	+
63	1417083_at	Region	Sec61b	66212	4	47390583	47399155	+
64	1435468_at	Region	C76566	97440	8	104548267	104550781	-
65	1416968_a_at	Region	Hsd3b7	101502	7	121850479	121853610	+
66	1426440_at	Region	Dhrs7	66375	12	69493291	69509468	-
67	1433987_at	Region	Hpcal4	170638	4	122210616	122221811	+
68	1418455_at	Region	Copz2	56358	11	96670963	96682290	+
69	1415827_a_at	Region	D3Ucla1	28146	3	58166332	58170108	-
70	1436739_at	Region	Agtr1	11607	13	29816079	29862489	+
71	1429058_at	Region	1110004B13Rik	66910	11	68796467	68798950	+
72	1449468_at	Region	St6galnac5	26938	3	151822396	151984720	-
73	1417204_at	Region	Kdelr2	66913	5	142462591	142480033	+
74	1417291_at	Region	Tnfrsf1a	21937	6	126006435	126019164	+
75	1421446_at	Region	Prkcc	18752	7	3801	13486	-
76	1454673_at	Region	Wasf2	242687	4	132091532	132159229	+
77	1448972_at	Region	Gria1	14799	11	56764801	57055873	+
78	1436461_at	Region	G3bp	27041	11	55238464	55253728	+
79	1423294_at	Region	Mest	17294	6	30786136	30794399	+
80	1428184_at	Region	3110035E14Rik	76982	1	9706059	9731934	+
81	1418128_at	Region	Adcy6	11512	15	98648223	98663099	-
82	1449147_at	Region	Chst1	76969	2	92316460	92319933	+
83	1422032_a_at	Region	Za20d3	65098	7	78468968	78533265	-
84	1422439_a_at	Region	Cdk4	12567	10	126796334	126800114	+
85	1448165_at	Region	Casp2	12366	6	42408919	42426376	+
86	1453473_a_at	Region	Tctex1	21648	6	136109838	136110544	-
87	1433696_at	Region	D17Ertd441e	52009	17	22748379	22766532	-
88	1432556_a_at	Region	3100002J23Rik	75429	11	58518440	58527590	-
89	1448524_s_at	Region	Ssr4	20832	X	68447717	68451477	+
90	1456241_a_at	Region	1810073N04Rik	72055	11	119925064	119972367	-
91	1448989_a_at	Region	Myo1b	17912	1	52050933	52208258	-
92	1433795_at	Region	Tgfbr3	21814	5	106170128	106359608	-
93	1435748_at	Region	Gda	14544	19	20622929	20710514	-
94	1423422_at	Region	Asb4	65255	6	5363349	5405778	+
95	1448508_at	Region	Traf3ip2	103213	10	39682835	39725209	+
96	1435239_at	Region	Gria1	14799	11	56764801	57055873	+
97	1437687_x_at	Region	Fkbp9	27055	6	56976402	57023417	+
98	1451620_at	Region	C1ql3	227580	2	12919466	12927756	-
99	1423537_at	Region	Gap43	14432	16	41113620	41205719	-
100	1424146_at	Region	Gpr37l1	171469	1	135010513	135017687	-
101	1450061_at	Region	Enc1	13803	13	93429732	93441676	+
102	1424037_at	Region	Itpka	228550	2	119256186	119265102	+
103	1419441_at	Region	Arbp	11837	5	114672398	114676611	+
104	1439560_x_at	Region	LOC432995 /// LOC436333	432995	16	4677092	4678091	+
105	1419417_at	Region	Vegfc	22341	8	53042643	53151107	+
106	1422068_at	Region	Pou3f1	18991	4	123684765	123686282	+
107	1435008_at	Region	Slc9a6	236794	X	51364568	51416607	+
108	1416723_at	Region	Tcf4	21413	18	69575005	69915006	+
109	1416111_at	Region	Cd83	12522	13	43347736	43365665	+
110	1438478_a_at	Region	Ppp3ca	19055	3	135555655	135820664	+
111	1426353_at	Region	Stat6	20852	10	127343744	127397113	+
112	1420928_at	Region	St6gal1	20440	16	22008076	22143979	+
113	1452269_at	Region	Spnb3	20743	19	4500013	4541142	+
114	1429065_at	Region	1200009F10Rik	67454	10	91054397	91073933	+
115	1434436_at	Region	Morc4	75746	X	133366574	133416593	-
116	1435807_at	Region	Cdc42	12540	4	136200964	136217395	-
117	1452142_at	Region	Slc6a1	232333	6	114853331	114888000	+
118	1440254_at	Region	None	None	11	106322016	106322301	+
119	1436459_at	Region	Gm208Gpr	240888	1	165217876	165243810	+
120	1456175_a_at	Region	Copb2	50797	9	98463089	98487733	+
121	1418891_a_at	Region	Rab3d	19340	9	21797512	21808123	-

122	1418257_at	Region	Slc12a7	20499	13	69823556	69876719	+
123	1451701_x_at	Region	Cldn3	12739	5	133999706	134000964	+
124	1433668_at	Region	Pnrc1	108767	4	33524225	33527303	-
125	1436455_at	Region	Asph	65973	4	9376231	9595958	-
126	1435687_at	Region	Sv2b	64176	7	69011039	69108389	-
127	1428808_at	Region	Prickle2	243548	6	92828179	92934188	-
128	1434589_x_at	Region	Surf4	20932	2	26852201	26866090	-
129	1455314_at	Region	Lpp	210126	16	23179019	23766559	+
130	1448952_at	Region	A030009H04Rik	80515	11	69066475	69068299	+
131	1417081_a_at	Region	Syngr2	20973	11	117630759	117635374	+
132	1450857_a_at	Region	Col1a2	12843	6	4458646	4493615	+
133	1417988_at	Region	Resp18	19711	1	75562843	75569393	-
134	1426555_at	Region	Scpep1	74617	11	88745107	88776519	-
135	1415996_at	Region	Txnip	56338	3	96045794	96049702	+
136	1431036_a_at	Region	1110032D12Rik	56334	5	123712134	123721846	+
137	1435577_at	Region	Dab1	13131	4	103326495	103639279	+
138	1421928_at	Region	Epha4	13838	1	77656420	77802175	-
139	1451935_a_at	Region	Spint2	20733	7	24665189	24693522	-
140	1419420_at	Region	St6galnac5	26938	3	151822396	151984720	-
141	1447854_s_at	Region	Hist2h2be	319190	3	95756526	95756936	+
142	1454672_at	Region	None	None	14	62616664	62617535	+
143	1428645_at	Region	Gnai3	14679	3	107903041	107941856	-
144	1442226_at	Region	6430702L12	330043	5	13709045	13709820	+
145	1453572_a_at	Region	Plp2	18824	X	5906886	5910272	-
146	1454821_at	Region	B3gat1	76898	9	26645461	26655237	+
147	1428473_at	Region	Ppp3cb	19056	14	18861352	18907856	-
148	1419493_a_at	Region	Tpd52	21985	3	78893581	78895590	-
149	1423025_a_at	Region	Schip1	30953	3	68244719	68298773	+
150	1435524_at	Region	2010109N14Rik	69895	3	122299262	122300033	-
151	1452969_at	Region	Atp2b1	67972	10	98895589	99006572	+
152	1420647_a_at	Region	Krt2-8 /// LOC434261	16691	15	102056071	102063643	-
153	1436383_at	Region	Cplx2	12890	13	52985707	52986681	+
154	1452031_at	Region	Slc1a3	20512	15	8424368	8500974	-
155	1449278_at	Region	Eif2ak3	13666	6	71164646	71236239	+
156	1417797_a_at	Region	1810019J16Rik	69073	4	132488828	132491689	+
157	1448154_at	Region	Ndrg2	29811	14	46999704	47007774	-
158	1441165_s_at	Region	Clstn2	64085	9	97343753	97483061	-
159	1439662_at	Region	Homer1	26556	13	89489518	89588558	+
160	1424594_at	Region	None	None	14	42185149	42186364	+
161	1418447_at	Region	Golga5	27277	12	97914676	97942915	+
162	1434877_at	Region	Nptx1	18164	11	119360465	119368918	-
163	1435717_at	Region	4833428C12Rik	399589	3	114709474	114710958	-
164	1418317_at	Region	Lhx2	16870	2	38283470	38301890	+
165	1455111_at	Region	A430107J06Rik	77929	X	93539141	93548434	+
166	1439489_at	Region	Gpr120	107221	19	37440882	37458066	+
167	1418171_at	Region	Tceal8	66684	X	129715020	129717144	-
168	1452754_at	Region	5730592L21Rik	76737	15	88871575	88878610	+
169	1417799_at	Region	Atp6v1g2	66237	17	32939454	32942611	+
170	1428715_at	Region	2810423A18Rik	72178	6	87523695	87526542	+
171	1452841_at	Region	Pgm2l1	70974	7	94334296	94382030	+
172	1418497_at	Region	Fgf13	14168	X	53816864	53889150	-
173	1418357_at	Region	Foxg1	15228	12	46129076	46132757	+
174	1434638_at	Region	None	None	16	9241510	9242025	-
175	1435227_at	Region	None	None	12	103383814	103385237	-
176	1424176_a_at	Region	Anxa4	11746	6	87171158	87227870	-
177	1428130_at	Region	Lman1	70361	18	66213875	66235740	-
178	1434672_at	Region	Gpr22	73010	12	28281559	28288571	-
179	1435429_x_at	Region	Rps27l	67941	9	67073432	67076878	+
180	1453317_a_at	Region	Khdrbs3	13992	15	68952956	69118055	+
181	1449930_a_at	Region	Ssr2	66256	3	88323535	88332277	+
182	1416695_at	Region	Bzrp	12257	15	83612210	83636054	+
183	1416772_at	Region	Cpt2	12896	4	106862887	106882439	-
184	1428335_a_at	Region	Scfd1	76983	12	48128993	48201414	+
185	1452357_at	Region	Gp1bb /// Sept5	14724	16	17391369	17393453	-

186	1425176_at	Region	C1ql3	227580	2	12919466	12927756	-
187	1418315_at	Region	Nr5a1	26423	2	38624789	38646665	-
188	1458268_s_at	Region	Igfbp3	16009	11	7103173	7108623	-
189	1451814_a_at	Region	Htatip2	53415	7	43873263	43888105	+
190	1417348_at	Region	2310039H08Rik	67101	17	44283562	44284322	+
191	1427611_at	Region	Ghrhr	14602	6	55520687	55532644	+
192	1437807_x_at	Region	Ctnna1	12385	18	35342570	35478433	+
193	1428269_a_at	Region	Glt8d1	76485	14	29133655	29143945	+
194	1448577_x_at	Region	Syngr2	20973	11	117630759	117635374	+
195	1423489_at	Region	Mmd	67468	11	90070563	90099660	+
196	1448240_at	Region	Mbtps1	56453	8	118850260	118901165	-
197	1434990_at	Region	Ppm1e	320472	11	86954898	87086986	-
198	1452766_at	Region	2900041A09Rik	72948	13	70069808	70096083	+
199	1416852_a_at	Region	Ncdn	26562	4	125770868	125780472	-
200	1433883_at	Region	Tpm4	326618	8	71293472	71311309	+
201	1440442_at	Region	Map2k7	26400	8	3593945	3601130	+
202	1451558_at	Region	Fbxw7	50754	3	84695316	84720739	+
203	1458622_at	Region	Ntrk2	18212	13	57448390	57572946	+
204	1436204_at	Region	1110059G02Rik	68786	15	55640894	55642196	-
205	1425558_at	Region	Klc3	232943	7	16263684	16269100	-
206	1450435_at	Region	L1cam	16728	X	68515690	68530459	-
207	1423280_at	Region	Stmn2	20257	3	8496587	8548309	+
208	1437640_at	Region	6430704M03Rik	230235	4	56903585	56933806	-
209	1455897_x_at	Region	Hmgn1	15312	16	95558992	95565130	-
210	1423173_at	Region	Napb	17957	2	148151593	148189356	-
211	1435196_at	Region	Ntrk2	18212	13	57448390	57572946	+
212	1452648_at	Region	Tbrg1	21376	9	37590683	37598845	-
213	1448792_a_at	Region	Cyp2f2	13107	7	22492659	22506466	+
214	1435260_at	Region	Sdccag8	76816	1	176747373	176953729	+
215	1436050_x_at	Region	Hes6	55927	1	91234525	91236264	-
216	1457440_at	Region	Sstr4	20608	2	147852278	147857879	+
217	1456535_at	Region	Vps13c	320528	9	67968941	68123713	+
218	1435614_s_at	Region	Rasgrf1	19417	9	89795814	89912965	+
219	1438310_at	Region	Dgkh	380921	14	72922487	72981706	-
220	1415885_at	Region	Chgb	12653	2	132295525	132308929	+
221	1455027_at	Region	D5Bwg0860e	52822	5	87850046	87908952	+
222	1455925_at	Region	Prdm8	77630	5	97200607	97206966	+
223	1434590_at	Region	B230209C24Rik	320705	1	34121165	34176734	-
224	1433855_at	Region	Abat	268860	16	8186782	8294861	+
225	1418054_at	Region	Neurod4	11923	10	130003660	130015733	-
226	1449921_s_at	Region	Cpne6	12891	14	50028892	50034962	+
227	1440015_at	Region	None	None	18	69919011	69919744	+
228	1460248_at	Region	Cpxm2	55987	7	126406479	126517151	-
229	1419515_at	Region	Fgd2	26382	17	27174554	27193416	+
230	1438589_at	Region	Igsf1	209268	X	44303340	44318535	-
231	1452717_at	Region	Slc25a24	229731	3	108918891	108977474	+
232	1419028_at	Region	MGI:107562	74100	9	112222231	112229149	-
233	1438953_at	Region	None	None	X	158000068	158000375	+
234	1418090_at	Region	Plvap	84094	8	70647571	70660944	-
235	1437405_a_at	Region	Igfbp4	16010	11	98862347	98873733	+
236	1457273_at	Region	Odz2	23964	11	35801678	36738275	-
237	1424507_at	Region	Rin1	225870	19	4839598	4845861	+
238	1445691_at	Region	Chn1	108699	2	73308494	73358115	-
239	1460460_a_at	Region	Gorasp2	70231	2	70359319	70389486	+
240	1416656_at	Region	Clic1	114584	17	32754023	32762499	+
241	1449470_at	Region	Dlx1	13390	2	71227188	71231723	+
242	1417738_at	Region	Rab25	53868	3	88285893	88292143	-
243	1418314_a_at	Region	MGI:1926224	268859	16	6740061	7081334	+
244	1417568_at	Region	Ncald	52589	15	37371112	37797344	-
245	1428267_at	Region	Dhx40	67487	11	86497746	86535629	-
246	1423147_at	Region	Mat1a	11720	14	39252260	39271291	+
247	1415700_a_at	Region	Ssr3	67437	3	65053089	65065985	-
248	1453159_at	Region	Efhc1	71877	1	21165300	21215777	+
249	1435749_at	Region	Gda	14544	19	20622929	20710514	-
250	1428384_at	Region	D4Bwg0951e	52829	4	79909879	79953494	+

251	1449956_at	Region	Prkce	18754	17	83996500	84488821	+
252	1441785_at	Region	None	None	11	65938319	65938627	-
253	1427295_at	Region	1810073N04Rik	72055	11	119925064	119972367	-
254	1450863_a_at	Region	Dcamkl1	13175	3	54876964	55171014	+
255	1435453_at	Region	A930011O12Rik	268755	14	59116823	59121198	+
256	1423269_a_at	Region	Nedd4l	83814	18	65256461	65446324	+
257	1422583_at	Region	Rab3b	69908	4	107837975	107902229	+
258	1435179_at	Region	C130071C03Rik	320203	13	79834708	79837961	+
259	1433857_at	Region	Fath	14107	8	43895563	43999658	+
260	1422440_at	Region	Cdk4	12567	10	126796334	126800114	+
261	1449875_s_at	Region	H2-T10 /// H2-T17 /// H2-T22 /// H2-T9	15024	17	33860368	33864725	-
262	1418072_at	Region	Hist1h2bc	68024	13	23164082	23172357	+
263	1415836_at	Region	Aldh18a1	56454	19	40094578	40132690	-
264	1460038_at	Region	Pou3f1	18991	4	123684765	123686282	+
265	1434893_at	Region	Atp1a2	98660	1	172203449	172226896	-
266	1451091_at	Region	Txndc5	105245	13	38041006	38069191	-
267	1417068_a_at	Region	Ptpn1	19246	2	167396315	167485652	+
268	1441069_at	Region	None	None	16	42848367	42848933	-
269	1425833_a_at	Region	Hpca	15444	4	128138688	128145871	-
270	1418488_s_at	Region	Ripk4	72388	16	97226242	97248055	-
271	1423940_at	Region	Yif1	68090	19	4877343	4881646	+
272	1452966_at	Region	9130430L19Rik	77672	12	103381656	103382878	-
273	1421863_at	Region	Vamp1	22317	6	125872261	125878983	+
274	1424068_at	Region	Tcta	102791	9	108372719	108375712	-
275	1418047_at	Region	Neurod6	11922	6	55822148	55825394	-
276	1415741_at	Region	Tparl	21982	5	75429189	75454483	+
277	1455572_x_at	Region	Rps18	20084	17	31655742	31659385	-
278	1437277_x_at	Region	Tgm2	21817	2	157573374	157603361	-
279	1426276_at	Region	Ifih1	71586	2	62452850	62501560	-
280	1415904_at	Region	Lpl	16956	8	68029951	68056194	+
281	1422497_at	Region	Slc30a5	69048	13	96973291	96984964	-
282	1431980_a_at	Region	As3mt	57344	19	46254334	46291500	+
283	1435487_at	Region	Grid2	14804	6	64146694	64904112	+
284	1417870_x_at	Region	Ctsz	64138	2	173852401	173863899	-
285	1424891_a_at	Region	Zw10	26951	9	49086054	49109318	+
286	1419811_at	Region	D16Wsu65e	28001	16	3956182	3956737	-
287	1422491_a_at	Region	Bnip2	12175	9	70120357	70139177	+
288	1434148_at	Region	Tcf4	21413	18	69575005	69915006	+
289	1416856_at	Region	3230401D17Rik	66680	2	162862791	162876439	-
290	1423277_at	Region	Ptprk	19272	10	28105024	28627423	+
291	1426163_x_at	Region	Rpl7	19989	1	16284751	16287861	-
292	1426815_s_at	Region	AU024582	227648	2	26341598	26360508	-
293	1420127_s_at	Region	Ccpg1	72278	9	73118338	73148795	+
294	1443865_at	Region	Gabra2	14395	5	69741965	69877243	-
295	1416803_at	Region	Fkbp7	14231	2	76360793	76370819	-
296	1458662_at	Region	Daam1	208846	12	68668036	68829803	+
297	1451784_x_at	Region	H2-D1 /// H2-K1 /// H2-L /// LOC56628	14964	17	186801	249200	-
298	1416942_at	Region	MGI:1933403	80898	13	70696832	70749086	+
299	1418890_a_at	Region	Rab3d	19340	9	21797512	21808123	-
300	1435842_at	Region	1110038O08Rik	68762	5	32493703	32494179	+
301	1428129_at	Region	Lman1	70361	18	66213875	66235740	-
302	1460573_at	Region	AI848100	226551	1	161723956	161783029	-
303	1420011_s_at	Region	Xbp1	22433	11	5415754	5420659	+
304	1452752_at	Region	None	None	4	118191815	118207883	+
305	1437845_x_at	Region	Pofut2	80294	10	77370658	77380047	+
306	1419517_at	Region	Cnih3	72978	1	181306443	181413724	+
307	1423183_at	Region	Lgi1	56839	19	37608585	37652742	+
308	1426852_x_at	Region	Nov	18133	15	54747888	54755722	+
309	1433711_s_at	Region	Sesn1	140742	10	42001833	42014167	+
310	1429625_at	Region	2900054C01Rik	73049	X	154400797	154403368	-
311	1437325_x_at	Region	Aldh18a1	56454	19	40094578	40132690	-
312	1436323_at	Region	2810001A02Rik	72146	15	86264501	86265270	-

313	1451129_at	Region	Calb2	12308	8	109440214	109465416	-
314	1423720_a_at	Region	Sara1	20224	10	61646625	61659601	+
315	1433509_s_at	Region	D6Ertd253e	52250	6	72039837	72142679	+
316	1450769_s_at	Region	Stard5	170460	7	77488447	77498758	+
317	1418883_a_at	Region	Pabpc1	18458	15	36597339	36610638	-
318	1427086_at	Region	None	None	11	35494444	35506061	+
319	1420955_at	Region	Vsnl1	26950	12	10678693	10740412	-
320	1428460_at	Region	Syn2	20965	6	115570293	115710479	+
321	1434914_at	Region	Rab6b	270192	9	103083792	103160522	+
322	1433443_a_at	Region	Hmgcs1	208715	13	114526	140291	-
323	1423358_at	Region	1810009K13Rik	66275	16	19383742	19391148	+
324	1449109_at	Region	Socs2	216233	10	95388906	95391737	-
325	1418576_at	Region	Yipf5	67180	18	40429229	40443156	-
326	1418749_at	Region	BC003498	80295	8	66796365	66798946	-
327	1428650_at	Region	Tns1	21961	1	74217097	74291359	-
328	1419371_s_at	Region	Gosr2	56494	11	103497948	103518783	-
329	1433583_at	Region	Zfp365	216049	10	67941416	67966217	-
330	1448468_a_at	Region	Kcnab1	16497	3	64782663	65051651	+
331	1419360_a_at	Region	Ss18	268996	18	14813890	14871175	-
332	1447167_at	Region	None	None	6	28576425	28576934	+
333	1418538_at	Region	Kdelr3	105785	15	79569021	79580297	+
334	1416328_a_at	Region	Atp6v0e	11974	17	24471230	24494471	+
335	1436364_x_at	Region	Nfix	18032	8	83974112	84039256	-
336	1452938_at	Region	C030032C09Rik	77531	10	90548071	90943317	+
337	1441072_at	Region	Cugbp2	14007	2	6459141	6638885	-
338	1435032_at	Region	Golgb1	224139	16	35692672	35750683	+
339	1420113_s_at	Region	2410022L05Rik	66423	8	13263410	13268885	-
340	1454832_at	Region	Phactr1	218194	13	42242828	42700966	+
341	1429225_at	Region	6330417K15Rik	70740	4	85969316	85971036	-
342	1449104_at	Region	Upk3a	22270	15	85065819	85071133	+
343	1418152_at	Region	Nsbp1	50887	X	103605900	103614724	-
344	1434670_at	Region	Kif5a	16572	10	126965001	126999707	-
345	1426855_at	Region	D10Ertd610e	52666	10	126915321	126923060	-
346	1454388_at	Region	2900046F13Rik	73028	2	6962824	6963892	-
347	1438443_at	Region	Zbtb20	56490	16	42442156	42491541	+
348	1436631_at	Region	2010010M04Rik	66546	18	35406149	35406816	+
349	1452878_at	Region	Prkce	18754	17	83996500	84488821	+
350	1427308_at	Region	Dab1	13131	4	103326495	103639279	+
351	1422085_at	Region	Tbx19	83993	1	165060565	165083707	-
352	1427509_at	Region	0610007P22Rik /// Gm937	545192	17	23049489	23062900	-
353	1420871_at	Region	Gucy1b3	54195	3	81761066	81803745	-
354	1457979_at	Region	None	None	8	14231566	14231903	+
355	1427247_at	Region	D3Bwg0562e	229791	3	116093000	116134735	-
356	1417005_at	Region	Kns2	16593	12	107239749	107276237	+
357	1454899_at	Region	Lpp	210126	16	23179019	23766559	+
358	1457412_at	Region	None	None	15	101102806	101103468	+
359	1421399_at	Region	Insm1	53626	2	145679042	145681349	+
360	1455238_at	Region	Mum1l1	245631	X	132779924	132783271	+
361	1427746_x_at	Region	H2-K1	14972	17	32967489	33050036	+
362	1420925_at	Region	Tub	22141	7	102863841	102883914	+
363	1417858_at	Region	Rasal1	19415	5	119800629	119831400	+
364	1423898_a_at	Region	Trip12	14897	1	85055153	85095994	-
365	1435210_s_at	Region	Snph	241727	2	151047486	151089469	-
366	1425656_a_at	Region	Baiap2	108100	11	119764194	119822580	+
367	1416589_at	Region	Sparc	20692	11	55147342	55172707	-
368	1425567_a_at	Region	Anxa5	11747	3	35912528	35939301	-
369	1436452_x_at	Region	1110032D12Rik	56334	5	123712134	123721846	+
370	1450895_a_at	Region	1810020G14Rik	66272	12	78331960	78346366	-
371	1434881_s_at	Region	Kctd12	239217	14	97521013	97526010	-
372	1425545_x_at	Region	H2-D1 /// H2-L	14964	17	186801	249200	-
373	1450888_at	Region	Napb	17957	2	148151593	148189356	-
374	1437865_at	Region	Spata13	219140	14	55169182	55299693	+
375	1426944_at	Region	Fbxw8	231672	5	117193645	117284031	-
376	1435494_s_at	Region	Dsp	109620	13	37690386	37736832	+

377	1435940_at	Region	AI836758	99719	3	55172671	55173405	+
378	1436076_at	Region	Dlgap1	224997	17	68195196	68591524	+
379	1450723_at	Region	Isl1	16392	13	112748913	112760636	-
380	1441107_at	Region	Dmrta2	242620	4	108936958	108942589	+
381	1416713_at	Region	2700055K07Rik	67971	8	104763120	104766994	-
382	1416149_at	Region	Olig1	50914	16	90426805	90428975	+
383	1424595_at	Region	F11r	16456	1	171366400	171393432	+
384	1433465_a_at	Region	AI467606	101602	7	121141167	121143780	+
385	1426729_at	Region	2900046G09Rik	78408	16	19467198	19475177	+
386	1452026_a_at	Region	Pla2g12a	66350	3	128811504	128828682	+
387	1423287_at	Region	Cbln1	12404	8	86752336	86755765	-
388	1424701_at	Region	Pcdh20	219257	14	19456	24795	+
389	1453118_s_at	Region	Rpl22	19934	4	150818238	150826433	+
390	1428963_at	Region	Rwdd2	69519	9	86914382	86917225	+
391	1428434_at	Region	Zcchc12	72693	X	30816542	30819704	+
392	1441870_s_at	Region	Pkd2	18764	5	103495425	103541936	+
393	1452833_at	Region	Rapgef2	76089	3	78790668	79018371	-
394	1417205_at	Region	Kdelr2	66913	5	142462591	142480033	+
395	1435531_at	Region	Usp3	235441	9	66641895	66716994	-
396	1416562_at	Region	Gad1	14415	10	99423927	99427119	+
397	1451322_at	Region	2310016A09Rik	69574	15	31569511	31590725	+
398	1419308_at	Region	Invs	16348	4	48195745	48347875	+
399	1418094_s_at	Region	Car4	12351	11	84685835	84694036	+
400	1454916_s_at	Region	Arfip1	99889	3	84239544	84326073	-
401	1427964_at	Region	Cklfsf8	70031	9	114763068	114817993	-
402	1423362_at	Region	Sort1	20661	3	108079874	108157253	+
403	1426401_at	Region	Ppp3ca	19055	3	135555655	135820664	+
404	1416576_at	Region	Socs3	12702	11	117787172	117790255	-
405	1437502_x_at	Region	Cd24a	12484	10	43687333	43692412	+
406	1449254_at	Region	Spp1	20750	5	103472077	103476914	+
407	1418493_a_at	Region	Snca	20617	6	60971674	61069793	-
408	1435018_at	Region	5930434B04Rik	381356	2	26942112	26953251	+
409	1435780_at	Region	Psd	73728	19	45859429	45874498	-
410	1435991_at	Region	None	None	8	76451919	76452991	+
411	1423266_at	Region	2810405K02Rik	66469	4	153388792	153391405	-
412	1425022_at	Region	Usp3	235441	9	66641895	66716994	-
413	1434082_at	Region	None	None	10	93207825	93213923	+
414	1417875_at	Region	Ddx50	94213	10	62582408	62617583	-
415	1427232_at	Region	Sdccag33	110796	18	84180139	84254394	-
416	1418049_at	Region	Ltbp3	16998	19	5530917	5547315	+
417	1432466_a_at	Region	Apoe	11816	7	16565516	16568276	-
418	1454780_at	Region	Galntl4	233733	7	105324798	105633114	-
419	1424534_at	Region	Mmd2	75104	5	141570887	141587725	-
420	1437406_x_at	Region	Igfbp4	16010	11	98862347	98873733	+
421	1419459_a_at	Region	2610529C04Rik	67075	X	100571582	100613141	-
422	1416470_a_at	Region	Rpn1	103963	6	88518156	88540499	+
423	1460666_a_at	Region	Ebf3	13593	7	131562834	131681580	-
424	1416529_at	Region	Emp1	13730	6	136123687	136143929	+
425	1436611_at	Region	Slc39a12	277468	2	14314676	14421249	+
426	1426083_a_at	Region	Btg1	12226	10	96594653	96597253	+
427	1455463_at	Region	Phyhip	105653	14	64784073	64784814	+
428	1416259_at	Region	Pex12	103737	11	83022634	83026966	-
429	1426562_a_at	Region	Olfm1	56177	2	28137838	28162624	+
430	1455512_at	Region	None	None	11	65937377	65937722	-
431	1448388_a_at	Region	1110002B05Rik	104725	12	51401417	51412527	-
432	1421312_a_at	Region	Kifc2	16581	15	76710387	76719267	+
433	1448697_s_at	Region	Rpl36al	66483	12	66014530	66015829	-
434	1423281_at	Region	Stmn2	20257	3	8496587	8548309	+
435	1455469_at	Region	Slc6a7	240332	18	61220706	61239525	-
436	1420924_at	Region	Timp2	21858	11	118122157	118176401	-
437	1434028_at	Region	Arnt2	11864	7	78100192	78263873	-
438	1423222_at	Region	Cap2	67252	13	46100466	46248597	+
439	1434531_at	Region	GnT-IX	268510	11	116740490	116806863	+
440	1435113_x_at	Region	Stmn3	20262	2	181023416	181031457	-
441	1457132_at	Region	None	None	19	9651868	9652362	-

442	1454643_at	Region	Ubap2l	74383	3	89810481	89862883	-
443	1415698_at	Region	Golph2	105348	13	58294585	58335344	-
444	1433714_at	Region	Sult4a1	29859	15	84124696	84154353	-
445	1438780_at	Region	None	None	7	121853710	121854459	-
446	1418922_at	Region	Igsf4b	94332	1	173269605	173303046	-
447	1433667_at	Region	Lgi3	213469	14	64846859	64854311	+
448	1416352_s_at	Region	Gpsn2	106529	8	82829225	82852112	-
449	1425023_at	Region	Usp3	235441	9	66641895	66716994	-
450	1424241_at	Region	Slc30a6	210148	17	72201922	72231341	+
451	1426858_at	Region	Inhbb	16324	1	119166897	119190174	-
452	1422605_at	Region	Ppp1r1a	58200	15	103593360	103601042	-
453	1436356_at	Region	Samd4	74480	14	41964485	42183012	+
454	1416313_at	Region	Mllt11	56772	3	94706375	94716012	-
455	1417978_at	Region	Eif4e3	66892	6	100093332	100134966	-
456	1416189_a_at	Region	Sec61a1	53421	6	88938985	88954178	-
457	1421923_at	Region	Sh3bp5	24056	14	29506822	29567836	-
458	1448752_at	Region	Car2	12349	3	14902543	14916587	+
459	1421152_a_at	Region	Gnao1	14681	8	93095093	93253130	+
460	1435411_at	Region	None	None	11	98146502	98146979	-
461	1453328_at	Region	2700008G24Rik	72545	14	58194965	58196768	+
462	1450339_a_at	Region	Bcl11b	58208	12	103386487	103474485	-
463	1418954_at	Region	Camkk1	55984	11	72744711	72767731	+
464	1448598_at	Region	Mmp17	23948	5	128760081	128784078	+
465	1415696_at	Region	Sara1	20224	10	61646625	61659601	+
466	1417082_at	Region	Anp32b	67628	4	46367037	46388445	+
467	1418586_at	Region	Adcy9	11515	16	3958841	4090883	-
468	1438208_at	Region	Taok2	381921	7	120915409	120932690	-
469	1455258_at	Region	AW047325	268345	10	112134223	112135323	+
470	1435526_at	Region	Tor1aip2	240832	1	155948197	155965475	+
471	1450378_at	Region	Tapbp	21356	17	31623640	31631894	+
472	1455559_at	Region	E330039K12Rik	270109	8	125039040	125127379	-
473	1455500_at	Region	D11Ertd759e	268513	11	119298143	119308517	+
474	1422711_a_at	Region	Pnck	93843	X	68316649	68320483	-
475	1452783_at	Region	Fndc3b	72007	3	26833958	27060578	-
476	1418210_at	Region	Pfn2	18645	3	57483992	57489636	-
477	1435549_at	Region	Trpm4	68667	7	39379174	39410284	-
478	1455168_a_at	Region	Gnb2l1	14694	11	48553197	48559078	+
479	1416209_at	Region	Glud1	14661	14	32447805	32482226	+
480	1448856_a_at	Region	Msra	110265	14	58652085	58984181	-
481	1451201_s_at	Region	Rnh1	107702	7	135562313	135570695	-
482	1455324_at	Region	LOC433022	433022	16	44845024	44847514	-
483	1423985_at	Region	Gng5	14707	3	145476441	145482098	+
484	1435744_at	Region	6720401G13Rik	103012	X	45083921	45155862	-
485	1424996_at	Region	Cflar	12633	1	59018187	59060035	+
486	1425719_a_at	Region	Nmi	64685	2	51880667	51905252	-
487	1426793_a_at	Region	Rpl14	67115	9	120583908	120587044	+
488	1418539_a_at	Region	Ptpre	19267	7	129905416	130053886	+
489	1417689_a_at	Region	Pdzk1ip1	67182	4	114047628	114052798	+
490	1431037_a_at	Region	Elavl1	15568	8	3643294	3667872	-
491	1456149_at	Region	D11Bwg0517e	52897	11	118311212	118312223	-
492	1449353_at	Region	Wig1	22401	3	31742974	31773798	-
493	1434049_at	Region	Entpd3	215446	9	120552235	120580709	+
494	1456954_at	Region	Kcna6	16494	6	127379948	127411808	-
495	1422853_at	Region	Shc1	20416	3	89175332	89186744	+
496	1434456_at	Region	Gm440	242819	5	8496982	8629695	-
497	1430029_a_at	Region	Sas	67125	10	126800009	126802980	-
498	1456037_x_at	Region	Preb	50907	5	29413992	29419173	-
499	1448308_at	Region	Ap3m1	55946	14	19399475	19416850	-
500	1416783_at	Region	Tac1	21333	6	7527828	7535730	+
501	1444318_at	Region	Chchd7	66433	4	3866060	3870669	+
502	1416268_at	Region	Ets2	23872	16	95110358	95128440	+
503	1450713_at	Region	Cspg5	29873	9	110285868	110302152	+
504	1426204_a_at	Region	Oprl1	18389	2	181432000	181437928	+
505	1451217_a_at	Region	1500034J20Rik	66541	2	105609439	105671940	+
506	1450042_at	Region	Arx	11878	X	87947304	87959015	+

507	1433717_at	Region	D19Wsu162e	226178	19	46146382	46202440	+
508	1419756_at	Region	Dgkg	110197	16	21240316	21402362	-
509	1436624_at	Region	Dnm3	103967	1	161896669	162387092	-
510	1445381_at	Region	None	None	15	102554226	102554874	+
511	1418778_at	Region	9030408N13Rik	66815	3	128847850	128903077	-
512	1449151_at	Region	Pctk3	18557	1	131965579	131993404	-
513	1434219_at	Region	Stim2	116873	5	52808660	52930070	+
514	1418288_at	Region	Lpin1	14245	12	15903061	15956128	-
515	1419127_at	Region	Npy	109648	6	49963533	49970311	+
516	1453413_at	Region	Gnas	14683	2	173709228	173771651	+
517	1427464_s_at	Region	Hspa5	14828	2	34704257	34708619	+
518	1417502_at	Region	Tspan7	21912	X	8763685	8835542	+
519	1435897_at	Region	Rpl32	19951	6	116243049	116246278	-
520	1420668_a_at	Region	Yipf2	74766	9	21476083	21480147	-
521	1450089_a_at	Region	Srprb	20818	9	103163286	103177389	-
522	1429443_at	Region	Cpne4	74020	9	104547441	105015435	+
523	1425036_a_at	Region	Tnrc6a	233833	7	117180427	117250357	+
524	1425518_at	Region	Rapgef4	56508	2	71679030	71954119	+
525	1423865_at	Region	Cdw92	100434	4	53356536	53538357	+
526	1429314_at	Region	Syt11	229521	3	88489566	88516404	-
527	1433914_at	Region	LOC381236	381236	19	32884802	32917750	-
528	1448507_at	Region	Efhd1	98363	1	87080523	87126949	+
529	1454860_x_at	Region	Dad1	13135	14	48755589	48773800	-
530	1430904_at	Region	Arfgap3	66251	15	83348357	83398846	-
531	1460654_at	Region	Slc30a3	22784	5	29546887	29552436	-
532	1423286_at	Region	Cbln1	12404	8	86752336	86755765	-
533	1447669_s_at	Region	Gng4	14706	13	13229694	13273161	+
534	1450958_at	Region	Tm4sf1	17112	3	56919386	56927502	-
535	1419254_at	Region	Mthfd2	17768	6	83649797	83661835	-
536	1455288_at	Region	1110036O03Rik	66180	11	100229835	100235906	-
537	1423052_at	Region	Arf4	11843	14	24734402	24753463	+
538	1434441_at	Region	None	None	13	61674664	61675740	-
539	1416903_at	Region	Nucb1	18220	7	39574800	39592751	-
540	1452346_at	Region	B3gnt6	108902	19	4827643	4829920	+
541	1443749_x_at	Region	Slc1a3	20512	15	8424368	8500974	-
542	1418746_at	Region	MGI:1930773	56695	1	74587397	74662104	+
543	1416497_at	Region	Pdia4	12304	6	47920672	47937538	-
544	1455304_at	Region	Unc13c	208898	9	73612739	73778132	-
545	1455839_at	Region	Ugcgl1	320011	1	36434885	36535646	-
546	1448023_at	Region	Kalrn	224126	16	32783178	32784183	-
547	1436451_a_at	Region	1110032D12Rik	56334	5	123712134	123721846	+
548	1437079_at	Region	Slc18a2	214084	19	58860486	58895236	+
549	1452985_at	Region	Uaca	72565	9	60912092	60997458	+
550	1436732_s_at	Region	Fbxw8	231672	5	117193645	117284031	-
551	1425292_at	Region	Dtna	13527	18	23643946	23881842	+
552	1423085_at	Region	Efnb3	13643	11	69279783	69285836	-
553	1446174_at	Region	LOC245440	245440	X	55959993	55960712	+
554	1416213_x_at	Region	Surf4	20932	2	26852201	26866090	-
555	1433725_at	Region	Acvr1b	11479	15	101231452	101269660	+
556	1449584_at	Region	Dgkg	110197	16	21240316	21402362	-
557	1423427_at	Region	Adcyap1	11516	17	91181801	91187870	+
558	1421425_a_at	Region	Dscr1l1	53901	17	41306763	41542936	+
559	1433452_at	Region	B630019K06Rik	102941	X	7131322	7133903	+
560	1433916_at	Region	Vamp3	22319	4	149539667	149550315	-
561	1422064_a_at	Region	Zbtb20	56490	16	42442156	42491541	+
562	1417318_at	Region	Dbccr1	56710	4	67853202	67996147	-
563	1435513_at	Region	Htr2c	15560	X	140402560	140637323	+
564	1436054_at	Region	9130227C08Rik	219094	14	50402985	50414797	+
565	1460349_at	Region	BC006909	233895	7	121521345	121526489	+
566	1418046_at	Region	Nap1l2	17954	X	97785541	97788005	-
567	1422073_a_at	Region	Celsr2	53883	3	108188186	108211236	-
568	1417764_at	Region	Ssr1	107513	13	37516882	37533029	-
569	1442370_at	Region	None	None	6	136474502	136475357	-
570	1416114_at	Region	Sparcl1	13602	5	103113902	103148495	-
571	1415979_x_at	Region	Rpl7	19989	1	16284751	16287861	-

572	1455021_at	Region	Gabbr1	54393	17	34805575	34833793	+
573	1428385_at	Region	38419	71779	6	116775658	116847075	+
574	1419273_at	Region	C80913	19777	7	33124913	33183548	-
575	1452913_at	Region	Pcp4l1	66425	1	171102105	171125874	-
576	1458697_at	Region	None	None	5	47938645	47939393	-
577	1449240_at	Region	MGI:1333876	19051	6	56161840	56177013	+
578	1450757_at	Region	Cdh11	12552	8	101922774	102074855	-
579	1419122_at	Region	Mettl1	17299	10	126774651	126778180	+
580	1429113_at	Region	2900092E17Rik /// 1500031I19Rik	67278	7	121064782	121067083	-
581	1429191_at	Region	Dhx33	216877	11	70709749	70730090	-
582	1454688_x_at	Region	1110014C03Rik	68581	12	82210494	82242351	-
583	1421175_at	Region	Myt1l	17933	12	26094752	26491102	+
584	1420764_at	Region	Scrg1	20284	8	56495341	56516819	+
585	1435222_at	Region	Foxp1	108655	6	99395348	99630926	-
586	1417763_at	Region	Ssr1	107513	13	37516882	37533029	-
587	1416611_at	Region	Scamp2	24044	9	57674914	57702719	+
588	1418624_at	Region	Nsep1	22608	4	118237139	118253295	-
589	1428329_a_at	Region	Wdr56	68259	3	68563024	68674987	-
590	1435435_at	Region	Cttnbp2	30785	6	18415877	18564265	-
591	1417416_at	Region	Kcna1	16485	6	127312007	127316405	-
592	1448129_at	Region	Arpc5	67771	1	152651135	152660059	+
593	1416949_s_at	Region	Slc39a7	14977	17	31732423	31735708	-
594	1434447_at	Region	None	None	6	17555511	17557425	+
595	1440838_at	Region	AI852064	99104	2	166552704	166553624	-
596	1439500_at	Region	None	None	6	54650215	54651231	-
597	1448729_a_at	Region	38599	18952	11	87309123	87318531	+
598	1417819_at	Region	Tor1b	30934	2	30885164	30890432	+
599	1453104_at	Region	Mapk1	26413	16	15754059	15816626	+
600	1448397_at	Region	Gjb6	14623	14	51654587	51664861	-
601	1433627_at	Region	Sec23ip	207352	7	122836737	122858402	+
602	1450734_at	Region	Lztr2	89867	1	157412378	157473993	+
603	1427468_at	Region	Ppp3cb	19056	14	18861352	18907856	-
604	1449272_at	Region	Igsf4b	94332	1	173269605	173303046	-
605	1435207_at	Region	Dixdc1	330938	9	50735078	50800301	-
606	1422158_at	Region	Clstn2	64085	9	97343753	97483061	-
607	1433751_at	Region	Slc39a10	227059	1	47099555	47145520	-
608	1448272_at	Region	Btg2	12227	1	133925590	133929382	-
609	1450080_at	Region	Cxx1c	72865	X	48192667	48193756	+
610	1437414_at	Region	Zfp217	228913	2	169617376	169619047	-
611	1427413_a_at	Region	Cugbp1	13046	2	90645207	90721804	+
612	1417414_at	Region	38598	24050	15	82325610	82342778	+
613	1426225_at	Region	Rbp4	19662	19	37460433	37468611	-
614	1426666_a_at	Region	Unc84a	77053	5	138213212	138247853	+
615	1434156_at	Region	Rab11fip4	268451	11	79316871	79419671	+
616	1419225_at	Region	Cacna2d3	12294	14	27041818	27858330	-
617	1433477_at	Region	Abr	109934	11	76142392	76303385	-
618	1455792_x_at	Region	Ndn	17984	7	56122085	56123665	+
619	1453155_at	Region	Tmem50a	71817	4	133858923	133875889	-
620	1426010_a_at	Region	Epb4.1l3	13823	17	66934864	67065569	+
621	1435566_s_at	Region	Araf	11836	X	19087538	19099458	+
622	1417297_at	Region	Itpr3	16440	17	24906500	24918141	+
623	1450627_at	Region	Ank	11732	15	27456007	27584356	+
624	1435890_at	Region	5730596K20Rik	109168	19	7208422	7249028	+
625	1422943_a_at	Region	Hspb1	15507	5	134901551	134902929	+
626	1453197_at	Region	Med25	75613	7	38954595	38967682	-
627	1435336_at	Region	Celsr2	53883	3	108188186	108211236	-
628	1434290_at	Region	Gtdc1	227835	2	44496584	44793784	-
629	1448103_s_at	Region	Nono	53610	X	96031077	96049950	+
630	1417343_at	Region	Fxyd6	59095	9	45383154	45408890	+
631	1453287_at	Region	5730557B15Rik	67434	15	31296331	31368171	-
632	1436919_at	Region	Trp53i11	277414	2	92905058	92906441	+
633	1416663_at	Region	Ndufa9	66108	6	127495996	127523163	-
634	1455012_s_at	Region	Trim37	68729	11	86855069	86948674	+
635	1430583_at	Region	C130076O07Rik	319504	12	40985533	41346376	+

636	1421063_s_at	Region	Snrpn /// Snurf	20646	7	53759592	53781697	-
637	1434640_at	Region	Gm123	228777	2	151825694	151833502	-
638	1450649_at	Region	Gng10	14700	4	58978680	58985344	+
639	1460361_at	Region	5033414D02Rik	67759	19	28601276	28614470	-
640	1415823_at	Region	Scd2	20250	19	43838494	43851266	+
641	1433556_at	Region	Centa1	231821	5	138269934	138303831	-
642	1415964_at	Region	Scd1	20249	19	43938861	43951959	-
643	1450143_at	Region	Rasgrp1	19419	2	116793841	116856725	-
644	1434758_at	Region	1810049K24Rik	78892	8	119359392	119408065	+
645	1435220_s_at	Region	Cdc42se2	72729	11	54470343	54540477	-
646	1436566_at	Region	Rab40b	217371	11	121177896	121209232	-
647	1435605_at	Region	Actr3b	242894	5	24230231	24320547	+
648	1427673_a_at	Region	Sema3e	20349	5	13478445	13707444	+
649	1451526_at	Region	Arhgap12	75415	18	6067924	6178605	-
650	1460576_at	Region	Sec15l1	107371	19	36893738	37026525	+
651	1434270_at	Region	Nptxr	73340	15	79841252	79857012	-
652	1419186_a_at	Region	St8sia4	20452	1	95359334	95439253	-
653	1423519_at	Region	2210412D01Rik	70178	7	77963273	78005807	-
654	1425546_a_at	Region	Trf	22041	9	103184317	103205727	-
655	1439086_at	Region	A930009L07Rik	402733	15	72534597	72536256	-
656	1440856_at	Region	Mapk8	26419	14	31516453	31580724	-
657	1451825_a_at	Region	Copz1	56447	15	103352029	103362737	+
658	1429008_at	Region	1810074P20Rik	67490	4	25385663	25417278	-
659	1426282_at	Region	MGI:2446259	235106	9	28892331	29088826	-
660	1449030_at	Region	Syn2	20965	6	115570293	115710479	+
661	1448288_at	Region	Nfib	18028	4	81281923	81491315	-
662	1416561_at	Region	Gad1	14415	10	99423927	99427119	+
663	1422520_at	Region	Nef3	18040	14	62648482	62653258	-
664	1424757_at	Region	BC018242	235044	9	21828276	21838888	+
665	1423627_at	Region	Nqo1	18104	8	106685918	106700959	-
666	1427465_at	Region	Atp1a2	98660	1	172203449	172226896	-
667	1436334_at	Region	Synj1	104015	16	90104242	90168538	-
668	1436889_at	Region	Gabra1	14394	11	41884317	41935767	-
669	1433592_at	Region	Calm1	12313	12	95642228	95652493	+
670	1449037_at	Region	Crem	12916	18	3271060	3332223	-
671	1438676_at	Region	Mpa2l	100702	5	0	5299	-
672	1434092_at	Region	Nos3as	213948	5	22848078	22856040	-
673	1429071_at	Region	Me3	109264	7	83611590	83844667	+
674	1451454_at	Region	Pcdh20	219257	14	19456	24795	+
675	1460288_a_at	Region	Ppp4c	56420	7	120835599	120842202	-
676	1434055_at	Region	Galnt9	231605	5	109666450	109672349	+
677	1455460_at	Region	6430547I21Rik	320521	3	17128462	17130564	+
678	1434946_at	Region	None	None	12	3260343	3261335	+
679	1455604_at	Region	LOC545325	545325	1	65024018	65027347	-
680	1452779_at	Region	3110006E14Rik	76980	13	65736190	65772458	-
681	1418926_at	Region	Zfhx1a	21417	18	5709786	5817483	+
682	1430038_at	Region	Gphn	268566	12	75199951	75541337	+
683	1422072_a_at	Region	Gstm6	14867	3	107734969	107739375	-
684	1421950_at	Region	Pfdn2	18637	1	171274538	171287009	+
685	1433737_at	Region	Uhmk1	16589	1	170133433	170149570	-
686	1434785_at	Region	Cacng5	140723	11	107695694	107732451	-
687	1417883_at	Region	Gstt2	14872	10	75933625	75936026	-
688	1417504_at	Region	Calb1	12307	4	15808229	15833613	+
689	1452308_a_at	Region	Atp1a2	98660	1	172203449	172226896	-
690	1416138_at	Region	Anxa7	11750	14	18816589	18841128	-
691	1424767_at	Region	Cdh22	104010	2	164568242	164691452	-
692	1452169_a_at	Region	Dgkz	104418	2	91637575	91668348	-
693	1451175_at	Region	Spcs3	76687	8	53487715	53497276	-
694	1448541_at	Region	Kns2	16593	12	107239749	107276237	+
695	1443823_s_at	Region	None	None	1	172201421	172201688	-
696	1458842_at	Region	None	None	X	37048684	37049466	-
697	1450241_a_at	Region	Evi2a	14017	11	79252219	79256225	-
698	1450250_at	Region	AJ430384	170711	7	57389063	57532804	+
699	1429790_at	Region	Brunol6	76183	9	59695096	59724051	+
700	1455766_at	Region	Gabra1	14394	11	41884317	41935767	-

701	1444384_at	Region	None	None	6	53188844	53189622	-
702	1455987_at	Region	Sec61a1	53421	6	88938985	88954178	-
703	1418392_a_at	Region	Gbp4	55932	3	141529119	141542183	+
704	1417059_at	Region	Krtcap2	66059	3	89002910	89006194	+
705	1434058_at	Region	Pip3ap	268783	15	12016928	12083863	+
706	1423221_at	Region	Tubb4	22153	17	54770117	54777481	-
707	1435488_at	Region	C630010D07Rik	503860	5	90246954	90249118	+
708	1419252_at	Region	None	None	4	108239180	108346720	+
709	1425296_a_at	Region	Rgs3	50780	4	61651038	61793526	+
710	1419406_a_at	Region	Bcl11a	14025	11	23972907	24067984	+
711	1429556_at	Region	2610024B07Rik	269987	7	106757788	106760502	+
712	1451683_x_at	Region	H2-D1	14964	17	186801	249200	-
713	1419237_at	Region	Usp29	57775	7	5934028	6173502	+
714	1439278_at	Region	Zbtb20	56490	16	42442156	42491541	+
715	1436143_at	Region	4933425L03Rik	71169	12	12682551	12943170	+
716	1416985_at	Region	None	None	2	129107058	129146082	+
717	1423974_at	Region	Numa1	101706	7	96075802	96120917	+
718	1437400_at	Region	Nedd4l	83814	18	65256461	65446324	+
719	1435841_s_at	Region	Suclg2	20917	6	95936120	96180762	-
720	1428937_at	Region	Atp2b1	67972	10	98895589	99006572	+
721	1457325_at	Region	Kcns2	16539	15	34837430	34842137	+
722	1419403_at	Region	BC017612	170748	9	15342359	15382526	+
723	1425963_at	Region	Cabp7	192650	11	4633608	4641565	-
724	1429063_s_at	Region	Kif16b	16558	2	142170469	142397720	-
725	1448489_at	Region	Pafah2	100163	4	133358372	133388311	+
726	1423745_at	Region	1110031B06Rik	67826	11	59132779	59175585	-
727	1445359_at	Region	Adcy1	432530	11	7068440	7069089	+
728	1455109_at	Region	Tbl1xr1	81004	3	21419728	21554694	+
729	1438507_x_at	Region	Rpl14	67115	9	120583908	120587044	+
730	1417529_at	Region	Rab33a	19337	X	43040266	43051028	+
731	1452284_at	Region	Ptprz1	19283	6	22927206	23056382	+
732	1449280_at	Region	Esm1	71690	13	109607521	109615966	+
733	1434489_at	Region	Elmo3	234683	8	104601237	104606388	+
734	1441952_x_at	Region	Lynx1	23936	15	74779518	74784641	-
735	1420563_at	Region	Gria3	53623	X	35922307	36197357	+
736	1438626_x_at	Region	Rpl14	67115	9	120583908	120587044	+
737	1429085_at	Region	Vezf1	22344	11	87796271	87812715	+
738	1452792_at	Region	Dzip1	66573	14	113431542	113479731	-
739	1448668_a_at	Region	Irak1	16179	X	68675627	68684576	-
740	1437076_at	Region	A930017M01Rik	239410	15	44697904	44830364	+
741	1455267_at	Region	Esrrg	26381	1	187506691	187723715	+
742	1438532_at	Region	None	None	1	150399271	150400014	-
743	1451505_at	Region	Chchd5	66170	2	128643675	128647960	+
744	1416385_a_at	Region	M6pr	17113	6	122969976	122978644	+
745	1450833_at	Region	Chrm1	12669	19	7860376	7878114	+
746	1417956_at	Region	Cidea	12683	18	67573460	67597721	+
747	1452514_a_at	Region	Kit	16590	5	74409260	74490904	+
748	1454960_at	Region	Smad3	17127	9	63767269	63878762	-
749	1423282_at	Region	Pitpna	18738	11	75313766	75354436	+
750	1418578_at	Region	Dgka	13139	10	128457224	128481146	-
751	1422661_at	Region	Lgals8	56048	13	12479155	12500727	-
752	1423939_a_at	Region	Yif1	68090	19	4877343	4881646	+
753	1428910_at	Region	2310022B05Rik	69551	8	123922828	123950441	-
754	1438198_at	Region	Bri3bp	76809	5	124608455	124621964	+
755	1416125_at	Region	Fkbp5	14229	17	26200322	26287578	-
756	1421735_a_at	Region	St8sia5	225742	18	77307467	77377231	+
757	1443855_at	Region	None	None	7	40523176	40524053	+
758	1438710_at	Region	Htr1a	15550	13	101659844	101664197	+
759	1459921_at	Region	Nxph1	18231	6	8916972	9216425	+
760	1417434_at	Region	Gpd2	14571	2	57163836	57293151	+
761	1430527_a_at	Region	Rnf167	70510	11	70373247	70377079	+
762	1434177_at	Region	Ece1	230857	4	136743558	136846490	+
763	1440840_at	Region	D630004K10Rik	103210	10	124912854	124914030	-
764	1438415_s_at	Region	Yipf2	74766	9	21476083	21480147	-
765	1460378_a_at	Region	Tes	21753	6	17048640	17089269	+

766	1456483_at	Region	Zfp9	22750	6	118894561	118911845	-
767	1429764_at	Region	1500005K14Rik	76566	11	75746247	75753441	-
768	1422695_at	Region	Ttyh1 /// Taf1	270627	X	96134183	96201680	+
769	1451332_at	Region	Zfp521	225207	18	13876625	14161173	-
770	1416997_a_at	Region	Hap1	15114	11	100168414	100177174	-
771	1458007_at	Region	Myo1b	17912	1	52050933	52208258	-
772	1455364_a_at	Region	Rps7	20115	12	25196338	25201165	-
773	1420649_at	Region	Atbf1	11906	8	108012331	108254925	+
774	1434169_at	Region	9030409G11Rik	71529	4	140983652	141035828	-
775	1452935_at	Region	LOC546143	546143	9	73112484	73118119	-
776	1417222_a_at	Region	2310075C12Rik	71929	9	49	3543	+
777	1452892_at	Region	Stk33	117229	7	103132170	103291947	-
778	1438233_at	Region	6030443O07Rik	226151	19	44475557	44527665	+
779	1455516_at	Region	None	None	2	65886247	65886947	+
780	1434582_at	Region	D14Ertd171e	238988	14	25761526	26615037	+
781	1417703_at	Region	Pvrl2	19294	7	16585905	16618713	-
782	1426346_at	Region	Prepl	213760	17	82893334	82920056	-
783	1437234_x_at	Region	Hrmt1l1	15468	10	76308969	76339515	-
784	1436100_at	Region	BC036961	230863	4	137131739	137142223	+
785	1424015_at	Region	Rab6ip1	19347	7	103746651	103813293	-
786	1430600_at	Region	Cklfsf5	67272	14	49452973	49454996	+
787	1424400_a_at	Region	Fthfd	107747	6	90990080	91038403	+
788	1416605_at	Region	Nola2	52530	11	51372614	51376555	+
789	1436483_at	Region	Myt1l	17933	12	26094752	26491102	+
790	1421922_at	Region	Sh3bp5	24056	14	29506822	29567836	-
791	1430780_a_at	Region	Pmm1	29858	15	82001829	82011581	-
792	1422501_s_at	Region	Idh3a	67834	9	54704020	54722171	+
793	1455440_at	Region	AI850629	100227	4	146496622	146498323	-
794	1437226_x_at	Region	Mlp	17357	4	128540757	128543088	+
795	1437700_at	Region	Schip1	30953	3	68244719	68298773	+
796	1454687_at	Region	Lrfn5	238205	12	58254745	58585291	+
797	1448623_at	Region	2310075C12Rik	71929	9	49	3543	+
798	1425303_at	Region	Gck	103988	11	5795610	5844386	-
799	1448628_at	Region	Scg3	20255	9	75780953	75821921	-
800	1416212_at	Region	Magoh	17149	4	106838721	106846303	+
801	1434982_at	Region	None	None	13	43232521	43233386	+
802	1426708_at	Region	Antxr2	71914	5	96902784	97050591	-
803	1418712_at	Region	Cdc42ep5	58804	7	3372578	3387115	-
804	1452059_at	Region	Slc35f5	74150	1	125395950	125430729	+
805	1416469_at	Region	Luzp1	269593	4	135402649	135430572	+
806	1436381_at	Region	Dlgap3	242667	4	126196384	126264135	+
807	1423058_at	Region	Capza2	12343	6	17620542	17649978	+
808	1434051_s_at	Region	None	None	19	58395304	58396677	-
809	1422824_s_at	Region	Eps8	13860	6	138270172	138331399	-
810	1420981_a_at	Region	Lmo4	16911	3	143160075	143176516	-
811	1417996_at	Region	Ngb	64242	12	83977157	83982165	-
812	1426510_at	Region	Sccpdh	109232	1	179622477	179641430	+
813	1425964_x_at	Region	Hspb1	15507	5	134901551	134902929	+
814	1443485_at	Region	Epha7	13841	4	28997442	29149372	+
815	1419489_at	Region	AW049604	106014	15	87677520	87811654	+
816	1452182_at	Region	Galnt2	108148	8	123518466	123632794	+
817	1428544_at	Region	0610007L01Rik	71667	5	129392160	129415495	+
818	1435722_at	Region	Gria4	14802	9	4328268	4706535	-
819	1454953_at	Region	Rnf157	217340	11	116157470	116234559	-
820	1434203_at	Region	BC055107	268709	14	5963634	5976774	-
821	1452336_at	Region	BC027382	407796	14	59885676	59926070	+
822	1439854_at	Region	AI838259	100738	5	117317204	117318125	+
823	1425181_at	Region	Sgip1	73094	4	101719268	101930082	+
824	1435215_at	Region	Ap1gbp1	217030	11	83692463	83770081	+
825	1448147_at	Region	Tnfrsf19	29820	14	55499179	55581612	-
826	1428852_at	Region	Dock3	208869	9	106961161	107297640	-
827	1448503_at	Region	Mcl1	17210	3	95146619	95151011	+
828	1456833_at	Region	Gpr17	574402	18	32183140	32183816	-
829	1428523_at	Region	Lphn3	319387	5	80275281	81049432	+
830	1437342_x_at	Region	Pttg1ip	108705	10	77693207	77710168	+

831	1422475_a_at	Region	Rps3a	20091	3	85882334	85886981	-
832	1428853_at	Region	Ptch1	19206	13	60894523	60948510	-
833	1435999_at	Region	C630041L24Rik	78709	9	109837816	109847794	+
834	1448649_at	Region	Enpep	13809	3	128200173	128262962	-
835	1454623_at	Region	MGC107514	232680	6	30588546	30611397	+
836	1442011_at	Region	A230065H16Rik	380787	12	106880200	106885468	+
837	1445639_at	Region	None	None	10	10208589	10212761	-
838	1428926_at	Region	1110003O08Rik	76291	8	120931218	120933363	-
839	1417530_a_at	Region	Srp9	27058	1	182083607	182091245	+
840	1415943_at	Region	Sdc1	20969	12	8087711	8110003	+
841	1438272_at	Region	None	None	15	47576240	47577169	-
842	1437559_at	Region	D13Bwg1146e	52882	13	101166216	101269810	-
843	1452020_a_at	Region	MGI:1353606	30954	12	108124289	108128532	+
844	1417590_at	Region	Cyp27a1	104086	1	75015769	75040224	+
845	1428114_at	Region	Slc14a1	108052	18	78222739	78245970	-
846	1416700_at	Region	Rnd3	74194	2	51062601	51081273	-
847	1419717_at	Region	Sema3e	20349	5	13478445	13707444	+
848	1421281_at	Region	Gabra1	14394	11	41884317	41935767	-
849	1448299_at	Region	Slc1a1	20510	19	28087763	28166557	+
850	1456480_at	Region	9330186A19Rik	320365	5	149134722	149437633	+
851	1449380_at	Region	Pacsin1	23969	17	25451600	25507024	+
852	1434651_a_at	Region	Cldn3	12739	5	133999706	134000964	+
853	1448892_at	Region	Dock7	67299	4	97911748	97959905	-
854	1433888_at	Region	Atp2b2	11941	6	114316878	114489190	-
855	1428836_at	Region	2300009A05Rik	69478	9	63517826	63519651	-
856	1455130_at	Region	5830435K17Rik	101685	7	41076681	41094192	-
857	1457054_a_at	Region	D130020L05Rik	319760	12	96529227	96533638	+
858	1429922_at	Region	None	None	2	66296280	66339959	+
859	1458351_s_at	Region	Klhl2	77113	8	63808844	63849038	-
860	1433775_at	Region	C77080	97130	4	128246691	128249875	-
861	1437201_at	Region	6430556C10Rik	241568	2	97172340	97336347	+
862	1436275_at	Region	Kcnip2	80906	19	45341106	45363239	-
863	1454713_s_at	Region	Hdc	15186	2	126107521	126132518	-
864	1424526_a_at	Region	Tgds	76355	14	112667955	112688210	-
865	1455753_at	Region	None	None	2	27042541	27043248	-
866	1438320_s_at	Region	Mcm7	17220	5	137137397	137144671	-
867	1460211_a_at	Region	Kdelr1	68137	7	39958363	39969249	+
868	1460365_a_at	Region	Dnm1	13429	2	32240634	32285443	-
869	1429991_at	Region	3110069A13Rik	73191	6	23296752	23300066	-
870	1437279_x_at	Region	Sdc1	20969	12	8087711	8110003	+
871	1448125_at	Region	Rit2	19762	18	31217063	31558917	-
872	1418973_at	Region	Blzf1	66352	1	164208297	164225981	-
873	1432543_a_at	Region	Klf13	50794	7	57665144	57712634	-
874	1457825_x_at	Region	Tcn2	21452	11	3811977	3826370	-
875	1428794_at	Region	Specc1	432572	11	61946845	61948648	+
876	1428349_s_at	Region	Ebf3	13593	7	131562834	131681580	-
877	1438245_at	Region	None	None	4	81480904	81481899	-
878	1417672_at	Region	Slc4a10	94229	2	62013606	62182126	+
879	1421698_a_at	Region	Col19a1	12823	1	24508650	24832568	-
880	1450208_a_at	Region	Elmo1	140580	13	19666927	20088452	+
881	1451895_a_at	Region	Dhcr24	74754	4	105520055	105545691	+
882	1455622_at	Region	Podxl2	319655	6	89277620	89309099	-
883	1455270_at	Region	None	None	11	102600686	102601355	+
884	1433796_at	Region	2310067E08Rik	71946	9	14189509	14216547	-
885	1422487_at	Region	Smad4	17128	18	73873242	73937970	-
886	1435461_at	Region	Magi3	99470	3	103442526	103647825	-
887	1454043_a_at	Region	Kcnab1	16497	3	64782663	65051651	+
888	1423490_at	Region	Fbxo3	57443	2	103732553	103767991	+
889	1441816_at	Region	2900056M20Rik	72997	X	145849613	145849894	-
890	1433451_at	Region	Cdk5r1	12569	11	80202705	80206840	+
891	1460617_s_at	Region	Rab6b	270192	9	103083792	103160522	+
892	1434728_at	Region	Gria3	53623	X	35922307	36197357	+
893	1422638_s_at	Region	Rassf5	54354	1	131025882	131094650	-
894	1453059_at	Region	2310046A06Rik	69642	9	77240964	77487092	-
895	1448110_at	Region	Sema4a	20351	3	88179823	88199543	-

896	1418105_at	Region	Stmn4	56471	14	60871795	60889100	+
897	1416098_at	Region	Syngr3	20974	17	22489447	22494793	-
898	1434526_at	Region	Abhd7	384214	5	106474000	106500340	+
899	1437398_a_at	Region	Aldh9a1	56752	1	167283401	167301474	+
900	1443694_at	Region	Rgs20	58175	1	4870637	4980278	-
901	1441298_at	Region	Spnb3	20743	19	4500013	4541142	+
902	1429958_x_at	Region	Haghl	68977	17	23588825	23591333	-
903	1424351_at	Region	Wfdc2	67701	2	164019449	164025239	+
904	1426814_at	Region	AU024582	227648	2	26341598	26360508	-
905	1450994_at	Region	Rock1	19877	18	10112811	10226661	-
906	1434373_at	Region	B930006L02Rik	319604	7	94813378	94948322	+
907	1454640_at	Region	Chchd7	66433	4	3866060	3870669	+
908	1433761_at	Region	9430063L05Rik	229622	3	97225124	97254244	-
909	1437087_at	Region	---	433926	5	104549819	104552148	+
910	1439709_at	Region	None	None	3	67891194	67891850	+
911	1427347_s_at	Region	Tubb2 /// 2410129E14Rik /// LOC544939	22151	13	33608787	33612497	-
912	1428493_at	Region	2610511M17Rik	74206	7	24731997	24751850	-
913	1428340_s_at	Region	Atp13a2	74772	4	139868189	139888591	+
914	1424948_x_at	Region	H2-D1 /// H2-K1 /// H2-L /// LOC56628 /// LOC547343	14964	17	186801	249200	-
915	1436518_at	Region	Usp46	69727	5	72834085	72900782	-
916	1435649_at	Region	Nexn	68810	3	151218385	151247664	-
917	1434314_s_at	Region	Rab11fip5	52055	6	85684683	85724625	-
918	1437125_at	Region	Camk2a	12322	18	61150958	61213470	+
919	1427754_a_at	Region	Dnm1	13429	2	32240634	32285443	-
920	1455669_at	Region	Wdr19	213081	5	64001542	64062244	+
921	1423759_a_at	Region	Tmco1	68944	1	167241924	167267166	+
922	1452661_at	Region	Tfrc	22042	16	31419658	31443429	+
923	1433642_at	Region	Arfrp2	218639	13	110208602	110571472	+
924	1434382_at	Region	Tde2l	230779	4	129280610	129302666	-
925	1425228_a_at	Region	Dguok	27369	6	83824448	83851125	-
926	1429798_s_at	Region	Cklfsf5	67272	14	49452973	49454996	+
927	1417994_a_at	Region	Accn1	11418	11	80605839	80878562	-
928	1438986_x_at	Region	Rps17	20068	7	75150291	75152774	-
929	1434594_at	Region	B230373P09Rik	218878	14	29392221	29395109	-
930	1436413_at	Region	None	None	10	34673008	34673747	+
931	1448877_at	Region	Dlx2	13392	2	71241152	71244484	-
932	1426766_at	Region	6330403K07Rik	103712	11	70757608	70759113	-
933	1428254_at	Region	Purb	19291	11	6369560	6370704	-
934	1449697_s_at	Region	Mfn1	67414	3	31937664	31985854	+
935	1456410_at	Region	AU045128	102704	9	98856379	98857903	+
936	1418206_at	Region	Sdf2l1	64136	16	15901089	15903316	-
937	1426915_at	Region	Dapk1	69635	13	59260847	59420940	+
938	1418713_at	Region	Pcbd1	13180	10	61051323	61056283	+
939	1434424_at	Region	9630055N22Rik	226844	1	190742113	190762233	-
940	1450243_a_at	Region	Dscr1l1	53901	17	41306763	41542936	+
941	1451237_s_at	Region	Rbm7	67010	9	48506080	48512677	-
942	1416190_a_at	Region	Sec61a1	53421	6	88938985	88954178	-
943	1444361_at	Region	None	None	X	157529000	157529867	+
944	1445194_at	Region	Cnksr2	245684	X	151420978	151642515	-
945	1447903_x_at	Region	Ap1s2	108012	X	157508565	157528951	+
946	1429906_at	Region	A930035E12Rik	77966	12	85017383	85018388	+
947	1421527_at	Region	Epha6	13840	16	58635452	59588354	-
948	1434174_at	Region	Lysmd3	80289	13	77731587	77745673	+
949	1452958_at	Region	2900006N09Rik	72898	5	111455434	111462202	-
950	1418196_at	Region	Tep1	21745	14	45918453	45965182	-
951	1433600_at	Region	None	None	19	53626546	53627860	+
952	1419392_at	Region	Pclo	26875	5	13968048	14249123	+
953	1435293_at	Region	2900022I03Rik	72849	5	8080763	8082002	-
954	1434783_at	Region	D15Ertd405e	380967	15	98040510	98045837	+
955	1455729_at	Region	Gnaq	14682	19	15357188	15609698	+
956	1428552_at	Region	2610001J05Rik	66520	6	13856851	13858416	-

957	1435482_at	Region	Fibcd1	98970	2	31745456	31778128	-
958	1423383_a_at	Region	Osbpl9	100273	4	108020555	108061497	-
959	1442051_at	Region	Hist2h3c1	15077	3	95735695	95736337	+
960	1427331_at	Region	Adora1	11539	1	134049485	134085697	-
961	1436216_s_at	Region	2610204M08Rik	70435	12	108084044	108094944	+
962	1436389_at	Region	5730596K20Rik	109168	19	7208422	7249028	+
963	1433722_at	Region	Akap13	233400	7	69488734	69518761	+
964	1428958_at	Region	Paqr8	74229	1	21115565	21163699	+
965	1425467_a_at	Region	Plp1	18823	X	130367715	130382986	+
966	1434661_at	Region	Syngr1	20972	15	80144003	80172200	+
967	1455106_a_at	Region	Ckb	12709	12	107147477	107150388	-
968	1433450_at	Region	Cdk5r1	12569	11	80202705	80206840	+
969	1452751_at	Region	Ebf3	13593	7	131562834	131681580	-
970	1428630_x_at	Region	Haghl	68977	17	23588825	23591333	-
971	1433887_at	Region	Dnajc3	19107	14	113495773	113540797	+
972	1417486_at	Region	Shd	20420	17	53608827	53614960	+
973	1439962_at	Region	2310010J17Rik	78329	7	84115929	84121052	-
974	1448513_a_at	Region	Npc2	67963	12	81625079	81641589	-
975	1430379_at	Region	5830411K21Rik	78822	2	45007600	45010908	-
976	1424025_at	Region	BC013529	215751	10	7633427	7646395	-
977	1435739_at	Region	AW208599	103436	10	7579963	7582386	+
978	1419639_at	Region	Efnb2	13642	8	7983291	8026630	-
979	1449381_a_at	Region	Pacsin1	23969	17	25451600	25507024	+
980	1454961_at	Region	Synj1	104015	16	90104242	90168538	-
981	1448860_at	Region	Rem2	140743	14	48993511	48997845	+
982	1420131_s_at	Region	Pttg1ip	108705	10	77693207	77710168	+
983	1449255_a_at	Region	Rpl15	66480	14	16792351	16794376	-
984	1424308_at	Region	Slc24a3	94249	2	144905442	145098889	+
985	1422612_at	Region	Hk2	15277	6	83068853	83118265	-
986	1448246_at	Region	Hdac1	433759	4	128543217	128569759	-
987	1436237_at	Region	Ttc9	69480	12	78486357	78523920	+
988	1420996_at	Region	Plxna3	18846	X	68989754	69004418	+
989	1418101_a_at	Region	Rtn3	20168	19	7139746	7197134	-
990	1442903_at	Region	4732423E21Rik	106967	18	69800574	69801114	+
991	1434581_at	Region	None	None	6	54845740	54848176	+
992	1448568_a_at	Region	Slc20a1	20515	2	128712653	128725462	+
993	1436168_at	Region	C730029A08Rik	320926	9	95475763	95477703	+
994	1433785_at	Region	Mobp	17433	9	120159695	120186044	+
995	1426712_at	Region	Slc6a15	103098	10	103282390	103333555	+
996	1455664_at	Region	Rtn4rl1	237847	11	74919459	74992558	+
997	1417217_at	Region	Magel2	27385	7	56153201	56155377	+
998	1415803_at	Region	Cx3cl1	20312	8	94056398	94066599	+
999	1449980_a_at	Region	Gabrd	14403	4	153877342	153890424	-
1000	1431920_a_at	Region	2010004A03Rik	76527	8	110039562	110103594	-
1001	1435124_at	Region	LOC328644	328644	16	19374916	19377518	+
1002	1424076_at	Region	Gdpd1	66569	11	86761880	86802040	-
1003	1448300_at	Region	Mgst3	66447	1	167305572	167326985	-
1004	1426428_at	Region	Thap7	69009	16	16298950	16302020	-
1005	1434384_at	Region	Nrip1	268903	16	75376752	75458939	-
1006	1455239_at	Region	6330512M04Rik	320802	7	136739134	136785283	-
1007	1440681_at	Region	None	None	7	56869299	56869935	-
1008	1436974_at	Region	A230069A22Rik	320587	4	154273946	154274605	-
1009	1416519_at	Region	Rpl36	54217	17	54301885	54302698	+
1010	1435495_at	Region	Adora1	11539	1	134049485	134085697	-
1011	1428525_at	Region	4930488B01Rik	75036	7	13914134	13915520	+
1012	1436246_at	Region	None	None	13	14999971	15000364	+
1013	1416666_at	Region	Serpine2	20720	1	80125571	80191029	-
1014	1420418_at	Region	Syt2	20980	1	134496944	134599680	+
1015	1416027_at	Region	Pdcd6	18570	13	70365333	70379452	-
1016	1419757_at	Region	Pitpnm2	19679	5	123290016	123387759	-
1017	1421690_s_at	Region	Agrp	11604	8	104862326	104863926	-
1018	1440441_at	Region	Fbxl7	448987	15	26776878	26777516	-
1019	1448422_at	Region	Tmed4	103694	11	6165501	6169624	-
1020	1422860_at	Region	Nts	67405	10	102395458	102404120	-
1021	1417298_at	Region	Ebpl	68177	14	55874266	55893955	-

1022	1417492_at	Region	Ctsb	13030	14	57653056	57676512	+
1023	1429052_at	Region	Ptprd	19266	4	75002457	75004430	-
1024	1415834_at	Region	Dusp6	67603	10	99243320	99247573	+
1025	1452908_at	Region	MGI:2385920	64451	10	76364477	76452959	-
1026	1426436_at	Region	8430420C20Rik	233806	7	113977993	113996553	+
1027	1416604_at	Region	Cyc1	66445	15	76392379	76394790	+
1028	1451033_a_at	Region	Trpc4	22066	3	53790324	53952687	+
1029	1436609_a_at	Region	Lrpap1	16976	5	33580650	33593377	-
1030	1442701_at	Region	None	None	6	80448295	80448876	+
1031	1420980_at	Region	Pak1	18479	7	91889708	91958070	+
1032	1422293_a_at	Region	Kctd1	106931	18	15156946	15339485	-
1033	1436121_a_at	Region	Nsmce1	67711	7	119517375	119541266	-
1034	1456307_s_at	Region	Adcy7	11513	8	87557754	87614390	+
1035	1454079_at	Region	4933400F03Rik	71013	10	116162615	116164435	+
1036	1439232_at	Region	1500016L03Rik	78365	11	84253668	84263823	+
1037	1417823_at	Region	Gcat	26912	15	79082206	79089640	+
1038	1436166_at	Region	Stox2	71069	8	46138301	46167033	-
1039	1443876_at	Region	Camk2a	12322	18	61150958	61213470	+
1040	1418400_at	Region	Larp6	67557	9	60830665	60856345	+
1041	1419283_s_at	Region	Tns1	21961	1	74217097	74291359	-
1042	1417364_at	Region	Eef1g	67160	19	8163745	8174725	+
1043	1429918_at	Region	Arhgap20	244867	9	51837391	51925006	+
1044	1435208_at	Region	Dtx3l	209200	16	34707924	34709399	-
1045	1416658_at	Region	Frzb	20378	2	80109719	80145145	-
1046	1448842_at	Region	Cdo1	12583	18	46934961	46950098	-
1047	1416868_at	Region	Cdkn2c	12580	4	108619781	108624277	-
1048	1419415_a_at	Region	Rarg	19411	15	102294300	102316638	-
1049	1460192_at	Region	Osbpl1a	64291	18	12943381	13129769	-
1050	1423263_at	Region	H3f3a	15078	1	180756388	180767423	-
1051	1450135_at	Region	Fzd3	14365	14	59729279	59780310	-
1052	1428138_s_at	Region	Arl10c	67166	6	109286299	109326908	+
1053	1428327_at	Region	2310001H13Rik	67095	9	121314022	121472320	+
1054	1433546_at	Region	Gns	75612	10	121021344	121053499	+
1055	1424140_at	Region	Gale	74246	4	134846422	134849435	+
1056	1452858_at	Region	Elavl1	15568	8	3643294	3667872	-
1057	1420817_at	Region	Ywhag	22628	5	134921901	134948016	-
1058	1415900_a_at	Region	Kit	16590	5	74409260	74490904	+
1059	1429183_at	Region	Pkp2	67451	16	14981080	15040447	+
1060	1434149_at	Region	Tcf4	21413	18	69575005	69915006	+
1061	1433630_at	Region	LOC208158	208158	16	19006863	19012935	-
1062	1455822_x_at	Region	Surf4	20932	2	26852201	26866090	-
1063	1449031_at	Region	Cited1	12705	X	96848797	96853130	-
1064	1451342_at	Region	Spon1	233744	7	107618725	107894210	+
1065	1449296_a_at	Region	Cnp1	12799	11	100396062	100402812	+
1066	1437632_at	Region	None	None	X	10914261	10915967	-
1067	1454786_at	Region	5031439G07Rik	223739	15	84997544	85036545	-
1068	1419073_at	Region	Tmeff2	56363	1	51228467	51488214	+
1069	1435725_x_at	Region	Rpl12 /// LOC432518 /// LOC436225	269261	2	32893888	32896203	+
1070	1426758_s_at	Region	Gtl2	17263	12	105023823	105039917	+
1071	1448443_at	Region	Serpini1	20713	3	75287431	75372212	+
1072	1434146_at	Region	None	None	3	80415024	80415636	-
1073	1418963_at	Region	2310047O13Rik	66960	2	12263273	12335447	-
1074	1455695_at	Region	9330109E03Rik	320852	6	143668580	143669759	-
1075	1439128_at	Region	7330412A13Rik	78143	16	42512337	42513156	+
1076	1439452_x_at	Region	Dnpep	13437	1	75598827	75608568	-
1077	1416099_at	Region	Rpl27	19942	11	101263502	101266620	+
1078	1439109_at	Region	MGC54896	381175	18	70155687	70199612	+
1079	1439399_a_at	Region	Rnu22	83673	19	7922713	7922989	+
1080	1437510_x_at	Region	Rps17	20068	7	75150291	75152774	-
1081	1425710_a_at	Region	Homer1	26556	13	89489518	89588558	+
1082	1454966_at	Region	Itga8	241226	2	12022669	12217929	-
1083	1459859_x_at	Region	Chrac1	93696	15	73117409	73121072	+
1084	1418937_at	Region	Dio2	13371	12	86128063	86141948	-

1085	1456878_at	Region	AI646023	192734	10	75651869	75662070	-
1086	1436333_a_at	Region	Synj1	104015	16	90104242	90168538	-
1087	1425481_at	Region	Cnot6l	231464	5	95087917	95179605	-
1088	1434264_at	Region	Ank2	109676	3	125805551	125881703	-
1089	1460219_at	Region	Mag	17136	7	26310852	26327101	-
1090	1423798_a_at	Region	Sui1-rs1	20918	11	100141081	100143183	+
1091	1437231_at	Region	Slitrk6	239250	14	105286271	105292757	-
1092	1427162_a_at	Region	Elk4	13714	1	131859694	131875481	+
1093	1425213_at	Region	6430514L14Rik	76886	9	70220291	70275848	-
1094	1437670_x_at	Region	Cd151	12476	7	135869384	135873464	+
1095	1437598_at	Region	7330412A13Rik	78143	16	42514338	42515067	+
1096	1418610_at	Region	Slc17a6	140919	7	45791145	45840421	+
1097	1424137_at	Region	Gprin1	26913	13	53347815	53351668	-
1098	1437319_at	Region	Unc13c	208898	9	73612739	73778132	-
1099	1435493_at	Region	Dsp	109620	13	37690386	37736832	+
1100	1438244_at	Region	Nfib	18028	4	81281923	81491315	-
1101	1450227_at	Region	Ankrd6	140577	4	33082828	33202298	-
1102	1452890_at	Region	D630041K24Rik	320244	12	82694680	82923050	+
1103	1460335_at	Region	Lysmd3	80289	13	77731587	77745673	+
1104	1422649_at	Region	Cntn6	53870	6	104975254	105345616	+
1105	1453326_at	Region	3300001A09Rik	75697	9	67887614	67889106	+
1106	1426215_at	Region	Ddc	13195	11	11709234	11775446	-
1107	1450935_at	Region	Ercc5	22592	1	44443666	44477079	+
1108	1450923_at	Region	Tgfb2	21808	1	186121756	186210661	-
1109	1420338_at	Region	Alox15	11687	11	70069811	70077673	-
1110	1455392_at	Region	9630019K15Rik	320111	17	215	3323	+
1111	1434202_a_at	Region	BC055107	268709	14	5963634	5976774	-
1112	1452051_at	Region	Actr3	74117	1	125220868	125263588	-
1113	1448762_at	Region	Rad17	19356	13	96771115	96804715	-
1114	1435680_a_at	Region	Dpp7	83768	2	25284452	25288494	-
1115	1428377_at	Region	Btbd11	74007	10	85531912	85553015	+
1116	1434170_at	Region	Wdr40b	245404	X	39307366	39310947	-
1117	1455477_s_at	Region	Pdzk1ip1	67182	4	114047628	114052798	+
1118	1433990_at	Region	Lhfpl3	269629	5	21183622	21739491	+
1119	1427108_at	Region	9530068E07Rik	213673	11	52149269	52161564	+
1120	1448366_at	Region	Stx1a	20907	5	134037064	134064591	+
1121	1429060_at	Region	9430072K23Rik	77292	19	5586926	5589348	-
1122	1453412_a_at	Region	Sec14l1	74136	11	116936342	116980314	+
1123	1436021_at	Region	A930031D07Rik	213006	1	131888602	131919751	-
1124	1428074_at	Region	2310037P21Rik	72309	9	123277458	123278318	-
1125	1435489_at	Region	None	None	11	49877916	49878561	+
1126	1434397_at	Region	Zdhhc17	320150	10	110565885	110632856	-
1127	1415993_at	Region	Sqle	20775	15	59325629	59341730	+
1128	1457671_at	Region	9330120H11Rik	319890	7	75409078	75412512	-
1129	1416897_at	Region	Parp9	80285	16	34719879	34754014	+
1130	1435976_at	Region	None	None	4	147439851	147444383	-
1131	1429013_at	Region	2900002G04Rik	78283	X	153013977	153098157	+
1132	1416165_at	Region	Rab31	106572	17	63361172	63482123	-
1133	1426713_s_at	Region	Eprs	107508	1	184861496	184926808	+
1134	1451961_a_at	Region	Mbp	17196	18	82642914	82753380	+
1135	1449379_at	Region	Kdr	16542	5	74767886	74784843	-
1136	1432094_a_at	Region	1700034M03Rik	73288	6	3450958	3555837	+
1137	1438957_x_at	Region	Cds2	110911	2	131777151	131821546	+
1138	1422835_at	Region	Kcnd2	16508	6	21263145	21776841	+
1139	1455256_at	Region	Tnik	69014	3	27680596	28087969	+
1140	1416935_at	Region	Trpv2	22368	11	62300188	62325946	+
1141	1456755_at	Region	2310001H13Rik	67095	9	121314022	121472320	+
1142	1417170_at	Region	Lztfl1	93730	9	123721311	123741507	-
1143	1453342_at	Region	Cdc40	71713	10	40910293	40992658	-
1144	1455725_a_at	Region	H3f3b	15081	11	115843386	115845593	-
1145	1460546_at	Region	Lgi3	213469	14	64846859	64854311	+
1146	1442650_at	Region	AI841723	101166	3	157091448	157092001	-
1147	1447788_s_at	Region	AW212607	241732	2	152679307	152682319	-
1148	1433555_at	Region	Eaf1	74427	14	29627014	29641729	+
1149	1450108_at	Region	Kif1a	16560	1	92845769	92929313	-

1150	1450429_at	Region	Capn6	12338	X	137242282	137286776	-
1151	1433707_at	Region	Gabra4	14397	5	70350950	70437875	-
1152	1435703_at	Region	2810457I06Rik	72828	9	40966047	41111032	-
1153	1429965_at	Region	2900060P06Rik	381338	1	39090578	39092045	-
1154	1436033_at	Region	BC031353	235493	9	75115155	75171307	+
1155	1455235_x_at	Region	Ldh2	16832	6	143333518	143351212	-
1156	1449202_at	Region	Sema4g	26456	19	44533750	44547801	+
1157	1425532_a_at	Region	Bin1	30948	18	32617401	32675875	+
1158	1435343_at	Region	Dock10	210293	1	80833085	80997044	-
1159	1424474_a_at	Region	Camkk2	207565	5	121886097	121917203	-
1160	1423551_at	Region	Cdh13	12554	8	118009574	118667271	+
1161	1437912_at	Region	Iqsec3	243621	6	121804773	121905505	-
1162	1438120_x_at	Region	None	None	X	68675461	68675625	-
1163	1417930_at	Region	Nab2	17937	10	127397629	127403388	-
1164	1450754_at	Region	Cacna2d2	56808	9	107465393	107594839	+
1165	1417287_at	Region	H13	14950	2	152126435	152163760	+
1166	1421727_at	Region	Eya1	14048	1	14348042	14481971	-
1167	1438217_at	Region	MGI:1926224	268859	16	6740061	7081334	+
1168	1434348_at	Region	Fez2	225020	17	76193990	76234701	-
1169	1433744_at	Region	AI841794	211187	6	119747399	119762999	-
1170	1434499_a_at	Region	Ldh2	16832	6	143333518	143351212	-
1171	1422587_at	Region	Tmem45a	56277	16	55709150	55790956	-
1172	1436037_at	Region	Itga4	16401	2	78953394	79028523	+
1173	1418084_at	Region	Nrp1	18186	8	127666747	127814326	+
1174	1449967_at	Region	Sim1	20464	10	51089397	51182898	+
1175	1434008_at	Region	Scn4b	399548	9	45142396	45142988	+
1176	1427568_a_at	Region	Wdr56	68259	3	68563024	68674987	-
1177	1436302_at	Region	2410193C02Rik	76775	8	77722695	77944758	+
1178	1451276_at	Region	E030041M21Rik	75089	10	89716350	89789724	+
1179	1456310_a_at	Region	2610002J02Rik	67513	4	153742328	153749046	+
1180	1426661_at	Region	Rpl27a	26451	7	103372068	103375226	+
1181	1433900_at	Region	0610007L01Rik	71667	5	129392160	129415495	+
1182	1423942_a_at	Region	Camk2g	12325	14	19096195	19155342	-
1183	1419536_a_at	Region	Rela	19697	19	5426521	5436920	+
1184	1431046_at	Region	2410127E16Rik	76787	7	39415815	39438364	-
1185	1441930_x_at	Region	None	None	NONE	NONE	NONE	NONE
1186	1424413_at	Region	Ogfrl1	70155	1	23503663	23655192	-
1187	1431003_a_at	Region	2610002J02Rik	67513	4	153742328	153749046	+
1188	1416983_s_at	Region	Foxo1	56458	3	51900114	51981292	+
1189	1425981_a_at	Region	Rbl2	19651	8	90356622	90410210	+
1190	1455216_at	Region	Paqr6	68957	3	88108453	88174614	+
1191	1451928_a_at	Region	Rad18	58186	6	113184164	113259954	-
1192	1449410_a_at	Region	Gas5	14455	1	160939881	160942671	+
1193	1418157_at	Region	Nr2f1	13865	13	74253386	74262704	-
1194	1423298_at	Region	Add3	27360	19	52767533	52814957	+
1195	1458643_at	Region	Dtx1	14357	5	119832010	119847210	-
1196	1423958_a_at	Region	2900001O04Rik	67515	15	4971126	5003902	+
1197	1429787_x_at	Region	Zwint	52696	10	72709781	72729831	+
1198	1451431_a_at	Region	D2Bwg0891e	52840	2	163943048	163947496	+
1199	1429685_at	Region	C030002O17Rik	78533	11	42384313	42385400	+
1200	1422637_at	Region	Rassf5	54354	1	131025882	131094650	-
1201	1459160_at	Region	None	None	11	66092027	66092665	-
1202	1436001_at	Region	2310028O11Rik	433771	4	137983084	137983777	-
1203	1434520_at	Region	Sc5d	235293	9	42213010	42223066	-
1204	1420556_at	Region	Oxt	18429	2	130090026	130090841	+
1205	1422758_at	Region	Chst2	54371	9	95313309	95315530	-
1206	1426449_a_at	Region	Pja1	18744	X	94067507	94072041	-
1207	1428221_at	Region	4931406O17Rik	78267	9	108517281	108531223	-
1208	1454572_at	Region	2810414N06Rik	71815	1	151607110	151607649	-
1209	1417147_at	Region	B230317C12Rik	56279	2	26560628	26568657	+
1210	1434354_at	Region	Maob	109731	X	14948222	15056304	-
1211	1454651_x_at	Region	Mbp	17196	18	82642914	82753380	+
1212	1436911_at	Region	Ss18l1	269397	2	179759467	179787159	+
1213	1449259_at	Region	Rab3d	19340	9	21797512	21808123	-
1214	1452095_a_at	Region	H47	109815	7	59963883	59973627	+

1215	1416383_a_at	Region	Pcx	18563	19	4383136	4410488	+
1216	1433536_at	Region	Lrp11	237253	10	7454951	7490015	+
1217	1418980_a_at	Region	Cnp1	12799	11	100396062	100402812	+
1218	1423309_at	Region	Tgoln1 /// Tgoln2	22134	6	72943992	72950517	-
1219	1428338_at	Region	2610039E05Rik	78779	8	122607141	122611092	-
1220	1419200_at	Region	Fxyd7	57780	7	26450839	26459780	-
1221	1415889_a_at	Region	Tra1	22027	10	86665503	86680106	-
1222	1440918_at	Region	6330417G04Rik	108780	13	88019522	88020769	-
1223	1424581_at	Region	Stac2	217154	11	97857711	97874549	-
1224	1423247_at	Region	Txndc4	76299	4	48110289	48195480	-
1225	1429049_at	Region	Nuak2	74137	1	132172971	132190342	+
1226	1434899_s_at	Region	Tnrc6a	233833	7	117180427	117250357	+
1227	1423569_at	Region	Gatm	67092	2	122108325	122125112	-
1228	1423156_at	Region	Gnpnat1	54342	14	40454704	40467079	-
1229	1426965_at	Region	Rap2a	76108	14	115049793	115078176	+
1230	1416250_at	Region	Btg2	12227	1	133925590	133929382	-
1231	1423757_x_at	Region	Igfbp4	16010	11	98862347	98873733	+
1232	1416701_at	Region	Rnd3	74194	2	51062601	51081273	-
1233	1439616_at	Region	None	None	9	68124158	68124613	+
1234	1446585_at	Region	Dlgh2	23859	7	85860491	86494465	+
1235	1438042_at	Region	Shox2	20429	3	66645577	66654082	-
1236	1419103_a_at	Region	Abhd6	66082	14	5671130	5724783	+
1237	1443177_at	Region	None	None	12	112043125	112043482	-
1238	1420868_s_at	Region	1110032D12Rik	56334	5	123712134	123721846	+
1239	1438723_a_at	Region	Rps10 /// LOC545151	545151	16	19520273	19593779	-
1240	1427965_at	Region	Ssbp1	381760	6	40612256	40622665	+
1241	1417727_at	Region	Sfrs9	108014	5	114437561	114443646	+
1242	1442865_at	Region	MGI:3580254	331374	X	5112421	5185734	+
1243	1460603_at	Region	Samd9l	209086	6	3324779	3352093	-
1244	1421170_a_at	Region	Plcb1	18795	2	134734571	134986275	+
1245	1442180_at	Region	Dleu7	239133	14	56808751	56826240	-
1246	1421738_at	Region	Gabra2	14395	5	69741965	69877243	-
1247	1422847_a_at	Region	Prkcd	18753	14	28730904	28746227	-
1248	1452789_at	Region	Snn	20621	16	10567613	10569058	-
1249	1421048_a_at	Region	Ypel1	106369	16	15841589	15857966	+
1250	1457733_at	Region	None	None	1	116614014	116615020	+
1251	1426649_at	Region	Tmeff1	230157	4	48501115	48579053	+
1252	1433449_at	Region	B930037P14Rik	225861	19	5283700	5285818	-
1253	1419450_at	Region	Ormdl3	66612	11	98402381	98408332	-
1254	1460685_at	Region	Timm17b	21855	X	6138338	6146592	+
1255	1456533_at	Region	Dpy19l1	244745	9	24306726	24318597	-
1256	1455870_at	Region	Akap2	11641	4	57797608	57837140	+
1257	1456444_at	Region	Fbxo41	330369	6	85820443	85852910	-
1258	1424633_at	Region	Camk1g	215303	1	193085118	193108993	-
1259	1423101_at	Region	Paqr4	76498	17	21538366	21542510	-
1260	1418647_at	Region	Gna-rs1	14670	17	33699699	33707887	+
1261	1434864_at	Region	Nipa1	233280	7	50247961	50289050	-
1262	1426960_a_at	Region	Fa2h	338521	8	110642730	110691404	-
1263	1453514_at	Region	Gpm6b	14758	X	159838377	159988393	+
1264	1450987_a_at	Region	2310004I24Rik	66358	11	66763555	66778244	-
1265	1416514_a_at	Region	Fscn1	14086	5	142024679	142037166	+
1266	1454078_a_at	Region	Gal3st1	53897	11	3878500	3894102	+
1267	1422031_a_at	Region	Za20d3	65098	7	78468968	78533265	-
1268	1455603_at	Region	None	None	16	80735863	80736294	+
1269	1428415_at	Region	Rnf113a	66381	12	81283572	81286924	+
1270	1436297_a_at	Region	Grina	66168	15	76295479	76298576	+
1271	1431724_a_at	Region	P2ry12	70839	3	58866261	58912811	-
1272	1436221_at	Region	D1Ertd471e	27877	1	166236488	166238034	+
1273	1433444_at	Region	Hmgcs1	208715	13	114526	140291	-
1274	1453180_at	Region	6530404N21Rik	67795	19	32466224	32720836	-
1275	1419732_at	Region	Rtn4r	65079	16	16898663	16923450	+
1276	1434099_at	Region	None	None	5	50264886	50265410	-
1277	1439348_at	Region	S100a10	20194	3	93272307	93281786	+
1278	1449866_at	Region	Syt2	20980	1	134496944	134599680	+

1279	1428146_s_at	Region	Acaa2	52538	18	75014405	75041400	+
1280	1458779_at	Region	8030445P17Rik	319569	1	25971912	25972522	-
1281	1445679_at	Region	None	None	X	68233480	68233849	+
1282	1448557_at	Region	1200015N20Rik	71721	10	70495074	70612698	+
1283	1428812_at	Region	1700040L02Rik	73287	10	68486259	68597178	-
1284	1416882_at	Region	Rgs10	67865	7	122423435	122467551	-
1285	1455262_at	Region	0610010D24Rik	68339	12	96358158	96474441	-
1286	1433445_x_at	Region	Hmgcs1	208715	13	114526	140291	-
1287	1428603_at	Region	Glcci1	170772	6	8234651	8573400	+
1288	1421010_at	Region	Mobp	17433	9	120159695	120186044	+
1289	1429648_at	Region	Slc35a3	229782	3	115439734	115481216	-
1290	1419291_x_at	Region	Gas5	14455	1	160939881	160942671	+
1291	1436560_at	Region	AW124694	104598	11	53356297	53357090	+
1292	1429089_s_at	Region	2900026A02Rik	243219	5	112159902	112240390	-
1293	1423177_a_at	Region	Abi1	11308	2	22882346	22926763	-
1294	1422720_at	Region	Isl1	16392	13	112748913	112760636	-
1295	1451894_a_at	Region	Scn8a	20273	15	100993863	101098226	+
1296	1437386_at	Region	Lrrn6a	235402	9	56731964	56798742	-
1297	1428774_at	Region	Gpc6	23888	14	111487529	112531234	+
1298	1448850_a_at	Region	Dnajc5	13002	2	181237462	181269206	+
1299	1426179_a_at	Region	Twsg1	65960	17	63632504	63660568	-
1300	1451520_at	Region	None	None	3	54746476	54771659	+
1301	1455978_a_at	Region	Matn2	17181	15	34306355	34435949	+
1302	1450976_at	Region	Ndrg1	17988	15	66953527	66993844	-
1303	1428739_at	Region	2310040A07Rik	69638	4	41776994	41779094	-
1304	1439267_x_at	Region	Cox5a	12858	9	57635235	57646353	+
1305	1451080_at	Region	Usp1	230484	4	97898883	97910606	+
1306	1460214_at	Region	Pcp4	18546	16	95949609	96007739	+
1307	1460337_at	Region	Sh3kbp1	58194	X	153308006	153573572	+
1308	1419165_at	Region	Zfp260	26466	7	25509675	25522233	+
1309	1415780_a_at	Region	Armcx2	67416	X	128349086	128353933	-
1310	1423819_s_at	Region	Arl6ip1	54208	7	111994757	112005420	-
1311	1427288_at	Region	Apba2	11784	7	58385748	58637908	+
1312	1425340_a_at	Region	Ptpra	19262	2	130017323	130067876	+
1313	1449404_at	Region	Pip5k2a	18718	2	18884270	19040126	-
1314	1438933_x_at	Region	Rasgrp2 /// LOC381240	19395	19	6189186	6203901	+
1315	1455498_at	Region	None	None	X	66337262	66337748	+
1316	1416067_at	Region	Ifrd1	15982	12	36801602	36821637	-
1317	1448344_at	Region	Rps12 /// LOC432865 /// LOC546370 /// LOC546371 /// LOC546372 /// LOC546373	20042	10	58921849	58922343	+
1318	1451728_at	Region	Wdr13	73447	X	6362274	6371378	-
1319	1422476_at	Region	Ifi30	65972	8	69918030	69921905	-
1320	1423640_at	Region	Synpr	72003	14	11742767	12075275	+
1321	1417800_at	Region	Parp2	11546	14	45902586	45915940	+
1322	1448573_a_at	Region	Ceacam10	26366	7	19954879	19962377	+
1323	1426970_a_at	Region	Ube1l /// D330022A01Rik	320169	9	108051045	108062994	+
1324	1448845_at	Region	Rpp25	102614	9	57617748	57619093	+
1325	1430222_at	Region	9130007G19Rik	74550	2	118748953	118751113	-
1326	1442175_at	Region	C030027H14Rik	77465	10	121437394	121438056	-
1327	1441499_at	Region	Grid1	14803	14	32951534	33710256	+
1328	1436169_at	Region	C730029A08Rik	320926	9	95475763	95477703	+
1329	1426030_a_at	Region	Apeh	235606	9	108151317	108160355	-
1330	1436225_at	Region	Trpm2	28240	10	78017488	78076254	-
1331	1451446_at	Region	Antxr1	69538	6	87568334	87769510	-
1332	1427239_at	Region	Wdr10	81896	6	116291063	116364225	+
1333	1434007_at	Region	Gyltl1b	228366	2	92069735	92075694	-
1334	1419442_at	Region	Matn2	17181	15	34306355	34435949	+
1335	1415711_at	Region	Arfgef1	211673	1	10242569	10337781	-
1336	1455883_a_at	Region	Lrrtm1	74342	6	77592843	77607877	+

1337	1419063_at	Region	Ugt8a	22239	3	124748763	124839904	-
1338	1447090_s_at	Region	Arl1	104303	10	88703486	88716220	+
1339	1424221_at	Region	Susd4	96935	1	182726092	182856756	+
1340	1434743_x_at	Region	Rusc1	72296	3	88827845	88837154	-
1341	1436405_at	Region	Dock4	238130	12	37444962	37445597	+
1342	1417428_at	Region	Gng3	14704	19	8033478	8035741	-
1343	1429582_at	Region	Btbd14a	67991	2	25991155	26054942	-
1344	1425415_a_at	Region	Slc1a1	20510	19	28087763	28166557	+
1345	1426956_a_at	Region	Trp53bp1	27223	2	120712100	120784969	-
1346	1433691_at	Region	Ppp1r3c	53412	19	36074403	36079323	-
1347	1435533_s_at	Region	4933426K21Rik	108653	6	123118089	123137380	-
1348	1459813_at	Region	1700012D01Rik	72243	10	127405114	127405393	+
1349	1444506_at	Region	Tram1	72265	1	13697903	13722901	-
1350	1417773_at	Region	Nans	94181	4	46405251	46419360	+
1351	1452984_at	Region	5730405I09Rik	67974	18	9356614	9490220	-
1352	1437224_at	Region	Rtn4	68585	11	29587737	29637706	+
1353	1423038_at	Region	Stx6	58244	1	155049479	155094254	+
1354	1452527_a_at	Region	P2rx4	18438	5	121860532	121881953	+
1355	1436573_at	Region	Scrn3	74616	2	73017517	73018501	+
1356	1448404_at	Region	Scamp2	24044	9	57674914	57702719	+
1357	1436479_a_at	Region	Dpp7	83768	2	25284452	25288494	-
1358	1438751_at	Region	E130106K10Rik	226781	1	184949416	184967214	+
1359	1444998_at	Region	None	None	11	57539631	57540506	+
1360	1416503_at	Region	Lxn	17035	3	67130310	67136218	-
1361	1448258_a_at	Region	Spcs1	69019	14	29131779	29133314	-
1362	1457397_at	Region	5330411L03Rik	101210	6	30996054	31082950	-
1363	1429211_at	Region	2900078E11Rik	72986	16	65666556	65667775	-
1364	1448606_at	Region	Edg2	14745	4	58378433	58496968	-
1365	1427225_at	Region	Epn2	13855	11	61242891	61305293	-
1366	1423055_at	Region	Nsg1	18196	5	36643418	36665681	-
1367	1427410_at	Region	Dleu2	328425	14	56136336	56215549	-
1368	1421205_at	Region	Atm	11920	9	53504628	53601833	-
1369	1456481_at	Region	D9Ertd280e	272636	9	99209814	99258346	-
1370	1426553_at	Region	Dnajc14	74330	10	128542766	128556536	+
1371	1455247_at	Region	Amotl1	75723	9	14380306	14523126	-
1372	1437434_a_at	Region	5031439A09Rik	68151	3	4597	6180	+
1373	1424812_at	Region	BC017158	233913	7	122321189	122347913	-
1374	1438655_a_at	Region	1100001I22Rik /// MGC103270	434046	3	40199929	40200096	+
1375	1428163_at	Region	Sara2	66397	11	51516543	51544764	+
1376	1418364_a_at	Region	Ftl1	14325	7	6038071	6038985	-
1377	1438531_at	Region	A730054J21Rik	320862	1	178893543	178894191	+
1378	1434132_at	Region	E430025E21Rik	223593	15	59342534	59384689	-
1379	1435500_at	Region	Rab26	328778	17	22332650	22337474	-
1380	1428450_at	Region	2610034B18Rik	70420	7	73722375	73733425	-
1381	1417914_at	Region	Rap2b	74012	3	61043051	61046895	+
1382	1451564_at	Region	Parp14	432411	16	34614287	34652791	-
1383	1448238_at	Region	2700060E02Rik	68045	14	18174611	18186532	-
1384	1434332_at	Region	Zzz3	108946	3	151378383	151441249	+
1385	1416252_at	Region	Stk38	106504	17	26773019	26811387	-
1386	1438195_at	Region	Gpd1l	333433	9	114876141	114907668	-
1387	1436621_at	Region	MGI:1933179	56013	11	97332756	97396213	-
1388	1416996_at	Region	Tbc1d8	54610	1	39668412	39699827	-
1389	1417638_at	Region	Lefty1	13590	1	180888879	180892220	+
1390	1418077_at	Region	Trim21	20821	7	96666869	96674091	-
1391	1455426_at	Region	Epha3	13837	16	62549020	62869320	-
1392	1456596_at	Region	6430550H21Rik	245386	X	32718102	32773193	-
1393	1455422_x_at	Region	38599	18952	11	87309123	87318531	+
1394	1452480_at	Region	BC024760	228726	2	145502262	145671971	+
1395	1417176_at	Region	Csnk1e	27373	15	79469142	79493293	-
1396	1449093_at	Region	Ctf1	13019	7	121762467	121767916	+
1397	1435307_at	Region	6430502M16Rik	218440	13	88593913	88608244	+
1398	1426445_at	Region	Mgea6	217615	12	55862841	55923223	+
1399	1421271_at	Region	Sh3md2	59009	8	60271818	60461252	+
1400	1426776_at	Region	Wasl	73178	6	24663290	24714456	-

1401	1451536_at	Region	1300002C08Rik	67472	3	18525595	18558156	+
1402	1419253_at	Region	Mthfd2	17768	6	83649797	83661835	-
1403	1421849_at	Region	Stag2	20843	X	36722649	36796970	+
1404	1442445_at	Region	2610027H17Rik	71811	14	4628907	4800179	+
1405	1438866_at	Region	Grin3a	242443	4	49578647	49579732	-
1406	1434454_at	Region	D16Wsu65e	28001	16	3956181	3957805	-
1407	1448978_at	Region	Ngef	53972	1	87293222	87326734	-
1408	1423393_at	Region	Clic4	29876	4	134095226	134154017	-
1409	1434394_at	Region	MGI:2684414	333789	5	64628739	64632073	+
1410	1451776_s_at	Region	MGI:1916782	74318	5	76331480	76359676	-
1411	1446417_at	Region	Serpinb6a	20719	13	33449537	33467606	-
1412	1429042_at	Region	2010200O16Rik	67495	3	108352167	108358121	-
1413	1428204_at	Region	C030002O17Rik	78533	11	42384313	42385400	+
1414	1420644_a_at	Region	Sec61a2	57743	2	5788259	5812625	-
1415	1434083_a_at	Region	4831417L10	270162	9	54022688	54079170	-
1416	1433971_at	Region	Camta1	100072	4	149553741	150114842	-
1417	1416000_a_at	Region	Prdx1	18477	4	115644504	115658905	+
1418	1434120_a_at	Region	Metap2	56307	10	93834268	93863130	-
1419	1454783_at	Region	Il13ra1	16164	X	30732693	30791816	+
1420	1434415_at	Region	None	None	7	13837832	13838488	+
1421	1426992_at	Region	Xpr1	19775	1	155171522	155308066	-
1422	1426980_s_at	Region	E130012A19Rik	103551	11	97448476	97450789	-
1423	1454934_at	Region	Ppm1f	68606	16	15674119	15698163	+
1424	1451027_at	Region	Baiap2	108100	11	119764194	119822580	+
1425	1436528_at	Region	Kazald1	107250	19	44620545	44623688	+
1426	1416178_a_at	Region	Plekhb1	27276	7	94750574	94769041	-
1427	1440902_at	Region	A330104H05Rik	77767	2	57970654	57978291	-
1428	1416696_at	Region	D17Wsu104e	28106	17	53813582	53820961	-
1429	1418080_at	Region	B4galt2	53418	4	116828165	116842060	-
1430	1424478_at	Region	Bbs2	67378	8	93352126	93382983	-
1431	1452024_a_at	Region	Ldb1	16825	19	45579761	45586788	-
1432	1448198_a_at	Region	Ndufb8	67264	19	44094660	44099821	-
1433	1426024_a_at	Region	Dbn1	56320	13	54082915	54100289	-
1434	1422130_at	Region	Nptx1	18164	11	119360465	119368918	-
1435	1431226_a_at	Region	Fndc4	64339	5	29751186	29753072	-
1436	1419737_a_at	Region	Ldh1	16828	7	40932461	40940831	+
1437	1428051_a_at	Region	Cacna1d	12289	14	28175955	28625750	-
1438	1435601_at	Region	AI481772	102105	8	109241794	109242298	+
1439	1430384_at	Region	Tle4	21888	19	13671934	13821845	-
1440	1451461_a_at	Region	Aldoc	11676	11	78048858	78052417	+
1441	1418463_at	Region	Pik3r2	18709	8	69923437	69932215	-
1442	1434535_at	Region	6330509G02Rik	268481	11	99054185	99065154	-
1443	1428029_a_at	Region	H2afv	77605	11	6323158	6339126	-
1444	1418051_at	Region	Ephb6	13848	6	41737668	41752602	+
1445	1420675_at	Region	Zfp113	56314	5	137114185	137128496	-
1446	1427014_at	Region	BC026996	229541	3	90077879	90090400	+
1447	1451089_a_at	Region	Arcn1	213827	9	44730423	44756652	-
1448	1433430_s_at	Region	Cdc23	52563	18	34854705	34874773	-
1449	1434856_at	Region	E130014H08Rik	329154	1	54951699	55033924	-
1450	1449376_at	Region	Nicn1	66257	9	108360217	108366256	+
1451	1416520_x_at	Region	Rpl36	54217	17	54301885	54302698	+
1452	1439041_at	Region	Slc39a10	227059	1	47099555	47145520	-
1453	1417389_at	Region	Gpc1	14733	1	92659177	92687687	+
1454	1440813_s_at	Region	LOC546318 /// LOC547400	546318	X	68432882	68433164	+
1455	1436030_at	Region	B430218L07Rik	320508	4	99735584	99962654	+
1456	1434746_at	Region	Mga	29808	2	119416522	119478814	+
1457	1435394_s_at	Region	Rhoc	11853	3	104215833	104221240	+
1458	1428432_at	Region	2310047A01Rik	71918	14	24086842	24133887	-
1459	1442724_at	Region	AW060763	104993	12	104941007	104941544	+
1460	1435796_at	Region	4933413A10Rik	243220	5	112678787	112702304	+
1461	1435505_at	Region	Dmwd	13401	7	15944961	15951537	+
1462	1421101_a_at	Region	Ldb2	16826	5	43229785	43558377	-
1463	1433835_at	Region	Ppp3cb	19056	14	18861352	18907856	-
1464	1421317_x_at	Region	Myb	17863	10	21054488	21090667	-

1465	1445574_at	Region	None	None	18	61516793	61517322	-
1466	1456196_x_at	Region	Fkbp1a	14225	2	150999557	151018627	+
1467	1454735_at	Region	Odf2	18286	2	29822262	29854701	+
1468	1417025_at	Region	H2-Eb1	14969	17	32006680	32016938	+
1469	1455972_x_at	Region	Hadhsc	15107	3	130163431	130202003	-
1470	1435095_at	Region	C030009O12Rik	77328	5	70928809	70930245	+
1471	1423343_at	Region	Slco1c1	58807	6	142330181	142375353	+
1472	1455146_at	Region	C230082I21Rik	99105	2	37502030	37502862	-
1473	1416483_at	Region	Ttc3	22129	16	93778128	93866771	+
1474	1433924_at	Region	Peg3	18616	7	5909405	5933864	-
1475	1449859_at	Region	Golt1b	66964	6	143230512	143247127	+
1476	1423248_at	Region	Nktr	18087	9	121733384	121771319	+
1477	1460613_x_at	Region	Gh	14599	11	106121357	106122899	-
1478	1434497_at	Region	None	None	NONE	NONE	NONE	NONE
1479	1415882_at	Region	Ghitm	66092	14	35253316	35267764	-
1480	1453204_at	Region	4933431K14Rik	71295	9	86079689	86081337	+
1481	1448124_at	Region	Gusb	110006	5	129158844	129172652	-
1482	1422474_at	Region	Pde4b	18578	4	101213908	101564744	+
1483	1452251_at	Region	Nbea	26422	3	55257927	55816432	-
1484	1454917_at	Region	AU045404	380959	15	90277186	90329435	+
1485	1419688_at	Region	Gpc6	23888	14	111487529	112531234	+
1486	1428599_at	Region	2410012C07Rik	76484	7	134335528	134339092	+
1487	1449298_a_at	Region	Pde1a	18573	2	79532205	79826720	-
1488	1422502_at	Region	Parp1	11545	1	180523221	180555500	+
1489	1437626_at	Region	Zfp36l2	12193	17	82018935	82021427	-
1490	1419093_at	Region	Tdo2	56720	3	81687472	81704788	-
1491	1435972_at	Region	Cast	12380	13	70751497	70865131	-
1492	1435515_at	Region	None	None	14	19118082	19118783	-
1493	1434620_s_at	Region	2610024E20Rik	225358	18	34665373	34729845	-
1494	1448252_a_at	Region	Eef1b2	55949	1	63470149	63472674	+
1495	1424040_at	Region	Parcc1	245877	4	125259280	125283395	-
1496	1452866_at	Region	Nars	70223	18	64732225	64749104	-
1497	1449196_a_at	Region	Rps27a	78294	11	29440636	29442640	-
1498	1440376_at	Region	None	None	6	85819494	85820216	-
1499	1452692_a_at	Region	Ndufv2	72900	17	63788360	63810997	-
1500	1437723_s_at	Region	Derl1	67819	15	57878521	57901434	-
1501	1452139_at	Region	Slc35c1	228368	2	92157447	92165201	-
1502	1426901_s_at	Region	Camta2	216874	11	70395124	70413592	-
1503	1439606_at	Region	Katnal1	231912	5	147773013	147825570	-
1504	1417305_at	Region	Apeg1	11790	1	75666020	75723037	+
1505	1435396_at	Region	None	None	12	41598176	41600796	-
1506	1436800_at	Region	Fstl5	213262	3	75804137	76439608	+
1507	1436946_s_at	Region	Gng5	14707	3	145476441	145482098	+
1508	1421899_a_at	Region	Mr1	15064	1	155018615	155037520	-
1509	1426522_at	Region	Hadhb	231086	5	28621184	28642098	+
1510	1455537_at	Region	6430547I21Rik	320521	3	17126312	17128949	+
1511	1436470_at	Region	Rims2	116838	15	39098033	39582362	+
1512	1454788_at	Region	Arl7	320982	1	88517718	88521248	-
1513	1434678_at	Region	AI661274	102927	X	45634297	45635916	-
1514	1424143_a_at	Region	Ris2	67177	8	121949602	121954717	+
1515	1419672_at	Region	Spock1	20745	13	56048889	56210903	-
1516	1438196_at	Region	Gpd1l	333433	9	114876141	114907668	-
1517	1455144_s_at	Region	AU040829	216831	11	64727689	64888611	-
1518	1460643_at	Region	Ell	13716	8	69695398	69749177	+
1519	1423445_at	Region	Rock1	19877	18	10112811	10226661	-
1520	1441139_at	Region	B330003H21	328186	13	12222687	12223343	-
1521	1416600_a_at	Region	Dscr1	54720	16	91551471	91559574	-
1522	1433564_at	Region	Dgkd	227333	1	87696734	87764305	+
1523	1422731_at	Region	Limd1	29806	9	123502410	123545221	+
1524	1416028_a_at	Region	Hn1	15374	11	115318446	115335463	-
1525	1424796_at	Region	1700054N08Rik	73420	8	123127903	123147281	-
1526	1416384_a_at	Region	Cope	59042	8	69455886	69466091	+
1527	1434380_at	Region	None	None	3	141517257	141519121	+
1528	1453087_at	Region	6330403L08Rik	70744	5	137997632	137998638	+
1529	1427430_at	Region	AI848100	226551	1	161723956	161783029	-

1530	1422485_at	Region	Smad4	17128	18	73873242	73937970	-
1531	1422635_at	Region	Ache	11423	5	136240796	136248427	+
1532	1440639_at	Region	Dlgap1	224997	17	68195196	68591524	+
1533	1437660_at	Region	Nktr	18087	9	121733384	121771319	+
1534	1423237_at	Region	Galnt1	14423	18	24433937	24514141	+
1535	1436731_at	Region	Zfp533	241494	2	77108849	77514565	-
1536	1417746_at	Region	Cplx1	12889	5	107594772	107626145	-
1537	1452919_a_at	Region	1700012G19Rik	67078	17	22274058	22275187	+
1538	1425610_s_at	Region	Galnt2	108148	8	123518466	123632794	+
1539	1450955_s_at	Region	Sort1	20661	3	108079874	108157253	+
1540	1434559_at	Region	Stx3	20908	19	10973053	11015388	-
1541	1423941_at	Region	Camk2g	12325	14	19096195	19155342	-
1542	1419383_at	Region	None	None	10	76355599	76362200	+
1543	1424588_at	Region	Srgap3	259302	6	113281331	113392980	-
1544	1418186_at	Region	Gstt1	14871	10	75885555	75900324	-
1545	1435384_at	Region	Ube2n	93765	10	95491346	95521553	+
1546	1417236_at	Region	Ehd3	57440	17	71586595	71613824	+
1547	1452759_s_at	Region	Ppfibp1	67533	6	147815628	147958589	+
1548	1456147_at	Region	st8sia6	241230	2	13570943	13719792	-
1549	1416986_a_at	Region	Ptpns1	19261	2	129107171	129144785	+
1550	1433740_at	Region	2610301K12Rik	72477	2	128366491	128368115	+
1551	1434655_at	Region	Foxk1	17425	5	141407513	141522715	+
1552	1429514_at	Region	Ppap2b	67916	4	104116265	104190062	+
1553	1423330_at	Region	Ensa /// LOC383218	383218	17	20655458	20656273	+
1554	1415911_at	Region	Impact	16210	18	13160301	13180997	+
1555	1434698_at	Region	9130229H14Rik	236520	1	6211	14138	-
1556	1423668_at	Region	Zdhhc14	224454	17	5400177	5659646	+
1557	1424189_at	Region	Pigc	67292	1	161875729	161878832	+
1558	1434325_x_at	Region	Prkar1b	19085	5	138015364	138127749	-
1559	1442046_at	Region	Apxl	110380	X	146144399	146157734	-
1560	1442659_at	Region	Pcdh9	211712	14	88351670	88356571	-
1561	1451718_at	Region	Plp1	18823	X	130367715	130382986	+
1562	1450436_s_at	Region	Dnajb5	56323	4	42869016	42874654	+
1563	1435165_at	Region	Cntn2	21367	1	132366699	132400216	-
1564	1434233_at	Region	None	None	X	66483448	66485362	+
1565	1422507_at	Region	Cstb	13014	10	78536450	78538362	+
1566	1434601_at	Region	Amigo2	105827	15	97320999	97323588	-
1567	1455400_at	Region	Ddah1	69219	3	144733606	144866062	+
1568	1450026_a_at	Region	B3gnt1	53625	11	22729537	22754478	-
1569	1428143_a_at	Region	Pnpla2	66853	7	135857199	135862086	+
1570	1426543_x_at	Region	2310067E08Rik	71946	9	14189509	14216547	-
1571	1430776_s_at	Region	Ankrd24	70615	10	81777736	81784518	+
1572	1444690_at	Region	Epha5	13839	5	83317214	83675897	-
1573	1416186_at	Region	Pnrc2	52830	4	134752186	134755084	-
1574	1434251_at	Region	Cnot1	234594	8	95001488	95088729	-
1575	1455444_at	Region	Gabra2	14395	5	69741965	69877243	-
1576	1440285_at	Region	Ppp1r9a	243725	6	4856205	5118546	+
1577	1434273_at	Region	A830073O21Rik	320262	7	67630614	67633709	-
1578	1439104_at	Region	C230081A13Rik	244895	9	56336855	56527185	-
1579	1424497_at	Region	1110032O16Rik	68742	7	120935951	120972691	-
1580	1439135_at	Region	None	None	16	64567130	64567835	+
1581	1456346_at	Region	Dnm1	13429	2	32240634	32285443	-
1582	1434155_a_at	Region	2310061I04Rik	69662	17	33611840	33616255	-
1583	1422620_s_at	Region	Ppap2a	19012	13	109194243	109262166	+
1584	1417596_at	Region	Eppb9	27078	11	61230814	61238569	+
1585	1449556_at	Region	H2-T23	15040	17	33748998	33751722	-
1586	1454853_s_at	Region	AI987944	233168	7	35490664	35510152	-
1587	1439024_at	Region	None	None	8	24489602	24491336	-
1588	1437197_at	Region	9430041O17Rik	234214	8	44653046	44768456	+
1589	1455161_at	Region	AI504432	229694	3	106835333	106846991	+
1590	1435907_at	Region	Nrxn2	18190	19	6319521	6322577	+
1591	1446022_at	Region	None	None	11	88495938	88496462	-
1592	1457743_at	Region	D13Bwg1146e	52882	13	101166216	101269810	-
1593	1424314_at	Region	Prpf3	70767	3	95318454	95343585	-

1594	1448955_s_at	Region	Cadps	27062	14	10830082	11279704	-
1595	1455554_at	Region	A830039N20Rik	268723	14	21656157	21657504	-
1596	1449194_at	Region	Mrps25	64658	6	92621555	92631448	-
1597	1457553_at	Region	Cugbp2	14007	2	6459141	6638885	-
1598	1452741_s_at	Region	Gpd2	14571	2	57163836	57293151	+
1599	1434418_at	Region	Lass6	241447	2	68716957	68966690	+
1600	1417664_a_at	Region	Ndrg3	29812	2	156384314	156427107	-
1601	1417110_at	Region	Man1a	17155	10	54152456	54322172	-
1602	1434920_a_at	Region	Evl	14026	12	104025433	104159229	+
1603	1455528_at	Region	None	None	12	66576641	66577101	-
1604	1452633_s_at	Region	Aak1	269774	6	87430559	87437290	+
1605	1437663_at	Region	Pigv	230801	4	132621286	132633519	-
1606	1418927_a_at	Region	Habp4	56541	13	61546922	61571141	+
1607	1423686_a_at	Region	1110020C13Rik	66151	15	102519651	102523203	+
1608	1444845_at	Region	Scfd1	76983	12	48128993	48201414	+
1609	1416683_at	Region	Plxnb2	140570	15	89207873	89226271	-
1610	1445688_at	Region	Ece1	230857	4	136743558	136846490	+
1611	1452975_at	Region	Agxt2l1	71760	3	129547666	129565839	+
1612	1434197_at	Region	Atrn	11990	2	130420349	130541594	+
1613	1428181_at	Region	Etfb	110826	7	37518908	37532547	+
1614	1418950_at	Region	Drd2	13489	9	49378932	49446482	+
1615	1457157_at	Region	Plcl3	269437	3	63364163	63519393	-
1616	1436920_at	Region	Gm78	219228	14	78892177	78893367	+
1617	1458518_at	Region	Cpeb2	231207	5	41990144	42042375	+
1618	1442873_at	Region	None	None	2	63826905	63827834	-
1619	1445694_at	Region	None	None	16	16302150	16302483	+
1620	1434900_at	Region	Mkl1	223701	15	81065904	81097980	-
1621	1431402_at	Region	Kirrel3	67703	9	34402087	34403416	+
1622	1434101_at	Region	Nfib	18028	4	81281923	81491315	-
1623	1437442_at	Region	None	None	5	56936046	56936745	+
1624	1455737_at	Region	C030002B11Rik	78540	10	122635292	122636363	+
1625	1449947_s_at	Region	Atbf1	11906	8	108012331	108254925	+
1626	1420885_a_at	Region	Sez6	20370	11	77656602	77704707	+
1627	1449187_at	Region	Pdgfa	18590	5	137975029	137993011	-
1628	1434374_at	Region	B930006L02Rik	319604	7	94813378	94948322	+
1629	1418475_at	Region	Scnn1b	20277	7	115777086	115796374	+
1630	1417262_at	Region	Ptgs2	19225	1	149936845	149944738	+
1631	1434342_at	Region	None	None	10	76362390	76363021	+
1632	1426216_at	Region	Cog6	67542	3	52613983	52649013	-
1633	1425848_a_at	Region	Dusp26	66959	8	29872480	29879865	+
1634	1434993_at	Region	B830045N13Rik	215378	1	146348134	146735852	+
1635	1442121_at	Region	MGI:107562	74100	9	112222231	112229149	-
1636	1456043_at	Region	Usp22	216825	11	60878669	60900563	-
1637	1456901_at	Region	Adamts20	223838	15	94338866	94472961	-
1638	1436982_at	Region	Tnrc6b	213988	15	80975683	80982651	+
1639	1427164_at	Region	Il13ra1	16164	X	30732693	30791816	+
1640	1421100_a_at	Region	Dab1	13131	4	103326495	103639279	+
1641	1427052_at	Region	Acacb	100705	5	113275892	113360303	+
1642	1434194_at	Region	Mtap2	17756	1	66731470	66734648	+
1643	1429723_at	Region	6330409N04Rik	66674	14	56069017	56091598	-
1644	1438282_at	Region	Syt1	20979	10	108385089	108577499	-
1645	1450859_s_at	Region	Ube2d3	66105	3	134321860	134350243	+
1646	1416102_at	Region	Ywhaz	22631	15	36773662	36797651	-
1647	1435401_at	Region	9430097H08Rik	264134	6	38517733	38563856	+
1648	1444723_at	Region	6530418L21Rik	109050	3	105500506	105515550	+
1649	1460204_at	Region	Tec	21682	5	71537285	71651490	-
1650	1440973_at	Region	LOC552874	552874	3	151819591	151820548	-
1651	1440167_s_at	Region	Lpp	210126	16	23179019	23766559	+
1652	1448223_at	Region	MGI:1347093	26383	8	90688173	90954867	+
1653	1452199_at	Region	2700094F01Rik	72649	6	30528276	30556749	-
1654	1422592_at	Region	Ctnnd2	18163	15	30163743	31021141	+
1655	1417126_a_at	Region	Rpl22l1	68028	3	28222895	28224799	+
1656	1438024_at	Region	6230416A05Rik	76137	13	43101452	43122631	-
1657	1455249_at	Region	---	434372	9	15580852	15583518	+
1658	1438932_at	Region	LOC381240	381240	19	6191577	6192212	+

1659	1416232_at	Region	Olig2	50913	16	90382586	90385713	+
1660	1435166_at	Region	Cntn2	21367	1	132366699	132400216	-
1661	1456395_at	Region	Ppargc1a	19017	5	50268292	50364500	-
1662	1450731_s_at	Region	Tnfrsf21	94185	17	40516102	40588746	+
1663	1418671_at	Region	Capn5	12337	7	92168303	92224931	-
1664	1438756_at	Region	None	None	18	12439959	12440624	-
1665	1418588_at	Region	Vmp	22360	13	24731917	24749826	-
1666	1433561_at	Region	Centb2	78618	16	29901288	29913810	-
1667	1458544_at	Region	BC059841	407813	14	54175550	54176122	-
1668	1448193_at	Region	5730403B10Rik	66626	16	4438038	4462482	-
1669	1428077_at	Region	2610024A01Rik	72160	1	127326244	127520409	-
1670	1435389_at	Region	None	None	X	156011354	156012086	-
1671	1435431_at	Region	2310047M15Rik	69666	13	33697486	33712682	+
1672	1438975_x_at	Region	Zdhhc14	224454	17	5400177	5659646	+
1673	1435518_at	Region	Rap1b	215449	10	117505589	117536966	-
1674	1430030_at	Region	5330426P16Rik	68190	16	49579380	49599175	-
1675	1448944_at	Region	Nrp1	18186	8	127666747	127814326	+
1676	1426412_at	Region	Neurod1	18012	2	79150390	79154385	-
1677	1421880_at	Region	Mtmr1	53332	X	66033251	66087687	+
1678	1460061_at	Region	None	None	14	61267286	61267926	+
1679	1417997_at	Region	Ngb	64242	12	83977157	83982165	-
1680	1423965_at	Region	Cd99l2	171486	X	66088561	66161136	-
1681	1433983_at	Region	Cnksr3	215748	10	3289639	3383481	+
1682	1453286_at	Region	Plxna2	18845	1	194358488	194552987	+
1683	1454926_at	Region	4930544G21Rik	77629	1	83594365	83738121	-
1684	1435817_x_at	Region	Rps6 /// LOC214738 /// LOC216036 /// LOC434404 /// LOC545640	20104	4	85840854	85843549	-
1685	1429745_at	Region	LOC434795	434795	X	67958433	67968419	+
1686	1452864_at	Region	Med12l	329650	3	58966724	58968423	+
1687	1418177_at	Region	Gabrg2	14406	11	41664694	41753515	-
1688	1455741_a_at	Region	Ece1	230857	4	136743558	136846490	+
1689	1458693_at	Region	Scn8a	20273	15	100993863	101098226	+
1690	1416509_at	Region	MGI:1914262	107358	19	40758164	40808343	-
1691	1450467_at	Region	Bsn	12217	9	108169094	108256167	-
1692	1445518_at	Region	Zfhx1b	24136	2	44919283	45045178	-
1693	1418342_at	Region	Recc1	19687	5	64063687	64137434	-
1694	1415818_at	Region	Anxa6	11749	11	54731952	54786255	-
1695	1423557_at	Region	Ifngr2	15980	16	90704258	90721171	+
1696	1437064_at	Region	AW320017	103030	X	92920903	92922641	+
1697	1416782_s_at	Region	Praf2	54637	X	5967511	5970003	+
1698	1436051_at	Region	9630007J19Rik	102493	9	75359415	75361268	+
1699	1418299_at	Region	Dpysl4	26757	7	133487649	133503345	+
1700	1455643_s_at	Region	AW550801	104662	11	74623765	74634998	+
1701	1435272_at	Region	Itpkb	320404	1	180285325	180377369	+
1702	1426516_a_at	Region	Lpin1	14245	12	15903061	15956128	-
1703	1431054_at	Region	Lsm6	78651	8	21699568	21713205	-
1704	1434151_at	Region	3300001H21Rik	70152	15	100362355	100371886	+
1705	1450106_a_at	Region	Evl	14026	12	104025433	104159229	+
1706	1438251_x_at	Region	Prss11	56213	7	125247906	125297330	+
1707	1434842_s_at	Region	Upf3b	68134	X	31712224	31730817	-
1708	1443037_at	Region	Sdfr1	20320	9	58698947	58769508	+
1709	1417339_a_at	Region	Dnclc1	56455	5	114408049	114410560	-
1710	1417263_at	Region	Ptgs2	19225	1	149936845	149944738	+
1711	1449241_at	Region	Klhl1	93688	14	90574765	90989240	-
1712	1427885_at	Region	Pold4	69745	19	4020727	4022421	+
1713	1445504_at	Region	LOC331392	331392	X	19599805	19603562	+
1714	1424299_at	Region	Oma1	67013	4	102272751	102325333	+
1715	1421992_a_at	Region	Igfbp4	16010	11	98862347	98873733	+
1716	1423039_a_at	Region	Bzw1	66882	1	58698537	58711303	+
1717	1439627_at	Region	Zic1	22771	9	91251432	91256620	-
1718	1420877_at	Region	38601	56526	X	31532799	31610011	-
1719	1456730_x_at	Region	Actl6a	56456	3	32116905	32135138	+

1720	1455377_at	Region	4921517B04Rik	70892	3	145920695	145959435	+
1721	1427404_x_at	Region	LOC433182	433182	2	80535137	80536512	+
1722	1427883_a_at	Region	Col3a1	12825	1	45615394	45652804	+
1723	1424029_at	Region	D10Bwg0791e	72480	10	34358352	34362182	+
1724	1442434_at	Region	D8Ertd82e	244418	8	34897933	34950616	+
1725	1432418_a_at	Region	Ckmt1	12716	2	120872490	120877586	+
1726	1435047_at	Region	None	None	13	106302744	106303438	-
1727	1422936_at	Region	Mas1	17171	17	11664256	11691322	+
1728	1457052_at	Region	AW536275	99379	2	167769088	167769585	-
1729	1451017_at	Region	Sdbcag84	66366	2	155465054	155475216	+
1730	1418796_at	Region	Clec11a	20256	7	38378589	38381229	-
1731	1451184_at	Region	Hnrpa3 /// LOC547321	229279	2	75357026	75367156	+
1732	1435294_at	Region	None	None	3	95658616	95659376	-
1733	1449936_at	Region	8430419L09Rik	74525	6	135958808	135995778	+
1734	1448927_at	Region	Kcnn2	140492	18	45780779	45906508	+
1735	1456377_x_at	Region	0610025L06Rik	67803	11	105977345	105981231	-
1736	1433488_x_at	Region	Gns	75612	10	121021344	121053499	+
1737	1455771_at	Region	Bzrap1	207777	11	87505085	87513910	+
1738	1430781_at	Region	Ak7	78801	12	101152976	101217778	+
1739	1417962_s_at	Region	Ghr	14600	15	3098508	3365766	-
1740	1454676_s_at	Region	Ticam1	106759	17	53906901	53909199	-
1741	1460716_a_at	Region	Cbfb	12400	8	104466302	104513614	+
1742	1452092_at	Region	4631426J05Rik	77590	7	126599419	126683081	-
1743	1458663_at	Region	Large	16795	8	71965827	72364115	-
1744	1448689_at	Region	Rras2	66922	7	107899626	107913462	-
1745	1428205_x_at	Region	C030002O17Rik	78533	11	42384313	42385400	+
1746	1434302_at	Region	Raph1	77300	1	60786474	60811912	-
1747	1426493_a_at	Region	Kifc2	16581	15	76710387	76719267	+
1748	1421180_at	Region	Lix1	66643	17	15266766	15321520	+
1749	1435957_at	Region	B830032F12	328545	15	66010277	66011218	-
1750	1424773_at	Region	1110012M11Rik	73711	8	70692174	70697270	+
1751	1429427_s_at	Region	Tcf7l2	21416	19	55323190	55515901	+
1752	1432029_a_at	Region	Smap1	98366	1	24090771	24166945	-
1753	1454645_at	Region	Mgrn1	17237	16	4555925	4608181	+
1754	1422504_at	Region	Glrb	14658	3	80572600	80642620	-
1755	1429316_at	Region	Rasgef1a	70727	6	118498951	118523137	+
1756	1452249_at	Region	Prickle1	106042	15	93565936	93585713	-
1757	1428096_at	Region	Ipo11	76582	13	103003195	103145609	-
1758	1429237_at	Region	Stx16	228960	2	173501228	173523314	+
1759	1427358_a_at	Region	Dapk1	69635	13	59260847	59420940	+
1760	1449107_at	Region	Nudt4	71207	10	95523184	95539880	-
1761	1455140_at	Region	A330068P14Rik	327958	11	71773188	71773870	-
1762	1438857_x_at	Region	None	None	X	68675460	68675830	-
1763	1449044_at	Region	Eef1e1	66143	13	38186438	38199754	-
1764	1448493_at	Region	Paip2	67869	18	35822332	35840850	+
1765	1419033_at	Region	2610018G03Rik	70415	X	45362232	45412652	+
1766	1416488_at	Region	Ccng2	12452	5	92603007	92611974	+
1767	1425337_at	Region	Slc12a5	57138	2	164425052	164456464	+
1768	1420474_at	Region	Mtpn	14489	6	35603109	35634015	-
1769	1417056_at	Region	Psme1	19186	14	50096453	50099493	+
1770	1419898_s_at	Region	Zc3h7	106205	16	10466302	10505988	+
1771	1448945_at	Region	Tm4sf11	67801	8	93959067	93980414	-
1772	1446001_at	Region	None	None	10	18005297	18006064	+
1773	1434326_x_at	Region	Coro2b	235431	9	62539533	62656961	-
1774	1423915_at	Region	Olfml2b	320078	1	170579570	170617529	+
1775	1434699_at	Region	6030408C04Rik	217558	12	48125521	48128402	+
1776	1425382_a_at	Region	Aqp4	11829	18	15581082	15591914	-
1777	1434997_at	Region	Cdc2l6	78334	10	40425316	40559068	+
1778	1418487_at	Region	Ripk4	72388	16	97226242	97248055	-
1779	1425130_a_at	Region	Ptpn5	19259	7	41163785	41219667	-
1780	1447640_s_at	Region	Pbx3	18516	2	34104071	34304198	-
1781	1448812_at	Region	Hpcal1	53602	12	3143	3964	+
1782	1430869_a_at	Region	Habp4	56541	13	61546922	61571141	+
1783	1455647_at	Region	AW320017	103030	X	92923049	92924581	+

1784	1454887_at	Region	Pak2	224105	16	30827234	30888644	-
1785	1436618_at	Region	Sfxn5	94282	6	85562775	85683140	-
1786	1417798_at	Region	1810019J16Rik	69073	4	132488828	132491689	+
1787	1436114_at	Region	G630064H08Rik	320680	18	77576401	77581161	-
1788	1421436_at	Region	Grid2	14804	6	64146694	64904112	+
1789	1425452_s_at	Region	Ptprj /// AW125753	105005	12	13504950	13509390	-
1790	1416339_a_at	Region	Prkcsh	19089	9	21892809	21904011	+
1791	1457523_at	Region	9530056K15Rik	320650	5	142455934	142458249	-
1792	1418655_at	Region	Galgt1	14421	10	126897960	126905117	+
1793	1450866_a_at	Region	Mrpl17	27397	7	99924236	99925719	-
1794	1436440_at	Region	B230107K20Rik	99450	2	70969010	70969524	-
1795	1416840_at	Region	Mid1ip1	68041	X	8956438	8958633	+
1796	1435311_s_at	Region	A230052G17Rik	320881	10	86009692	86010327	-
1797	1445565_at	Region	Hist1h2be	319179	13	23063618	23101001	-
1798	1455188_at	Region	Ephb1	270190	9	101870245	102304441	-
1799	1452624_at	Region	Lrrtm1	74342	6	77592843	77607877	+
1800	1416455_a_at	Region	Cryab	12955	9	50825084	50828959	+
1801	1418984_at	Region	Inadl	12695	4	97370899	97694676	+
1802	1436811_at	Region	Kctd3	226823	1	188502528	188539374	-
1803	1456228_x_at	Region	Mbp	17196	18	82642914	82753380	+
1804	1416060_at	Region	Tbc1d15	66687	10	114883404	114937196	-
1805	1445631_at	Region	LOC545446	545446	2	110281641	110292027	-
1806	1441610_at	Region	N28178	230085	4	42833173	42857529	+
1807	1416137_at	Region	Anxa7	11750	14	18816589	18841128	-
1808	1424946_a_at	Region	1300002C08Rik	67472	3	18525595	18558156	+
1809	1431775_at	Region	3100002H09Rik	70249	4	123637323	123637916	-
1810	1416785_at	Region	Kcnip1	70357	11	33524574	33738321	-
1811	1424130_a_at	Region	Ptrf	19285	11	100779597	100791659	-
1812	1452524_a_at	Region	Elavl3	15571	9	21907565	21942715	-
1813	1434098_at	Region	Glra2	237213	X	158728422	158926386	-
1814	1422821_s_at	Region	Stard5	170460	7	77488447	77498758	+
1815	1448430_a_at	Region	Naca /// LOC432684	17938	10	127772283	127785343	+
1816	1428446_at	Region	MGI:1913996	213575	17	82455944	82485015	+
1817	1434968_a_at	Region	Actr3	74117	1	125220868	125263588	-
1818	1418467_at	Region	Smarcd3	66993	5	23056519	23086151	-
1819	1448277_at	Region	Pold2	18972	11	5766975	5773004	-
1820	1418178_at	Region	Ina	226180	19	46566002	46575960	+
1821	1440204_at	Region	3110039M20Rik	67293	12	46136560	46153259	+
1822	1415995_at	Region	Casp6	12368	3	128834335	128846822	+
1823	1438578_a_at	Region	1110056N09Rik	68815	7	107169488	107223188	-
1824	1438647_x_at	Region	Cetn2	26370	X	67574220	67578999	-
1825	1440097_at	Region	Mirg	373070	12	105220796	105226448	+
1826	1455374_at	Region	None	None	2	55523046	55523687	+
1827	1433695_at	Region	1500041B16Rik	380686	11	16973262	16974167	+
1828	1449131_s_at	Region	Cd1d1	12479	3	86740227	86743729	-
1829	1425051_at	Region	Isoc1	66307	18	58884044	58904170	+
1830	1434857_at	Region	A230106D06Rik	232785	6	48067302	48076242	+
1831	1460336_at	Region	Ppargc1a	19017	5	50268292	50364500	-
1832	1427618_at	Region	Cdh9	12565	15	16566814	16694222	+
1833	1439892_at	Region	Brsk1	381979	7	3935848	3960888	+
1834	1452342_at	Region	Apbb2	11787	5	65067237	65384014	-
1835	1419640_at	Region	Purb	19291	11	6369560	6370704	-
1836	1424805_a_at	Region	1110039B18Rik	68796	5	29328591	29336411	+
1837	1448768_at	Region	Mog	17441	17	34771008	34782703	-
1838	1436316_at	Region	9430029L20Rik	109249	6	91905323	91906057	-
1839	1455071_at	Region	Thpok	22724	3	89135879	89138250	-
1840	1434409_at	Region	MGC27863	278097	X	128293394	128296351	-
1841	1423882_at	Region	Rfwd3	234736	8	110568533	110597811	-
1842	1417836_at	Region	Gpx7	67305	4	107359316	107365619	-
1843	1423424_at	Region	Zic3	22773	X	52785357	52790617	+
1844	1437049_at	Region	None	None	3	68985432	68986006	-
1845	1452982_at	Region	A330103N21Rik	77773	7	62113386	62117987	+
1846	1456015_x_at	Region	Ndufv1	17995	19	3796295	3801515	-

1847	1438928_x_at	Region	Ninj1	18081	13	48787060	48795764	+
1848	1426236_a_at	Region	Glul	14645	1	153783869	153793637	+
1849	1427274_at	Region	D130054N24Rik	235315	9	45876943	45920129	-
1850	1456610_at	Region	Jmjd3	216850	11	69125347	69130154	-
1851	1452332_at	Region	E030025D05Rik	216613	11	28283605	28479086	-
1852	1423057_at	Region	Capza2	12343	6	17620542	17649978	+
1853	1417101_at	Region	Hspa2	15512	12	73260264	73263026	+
1854	1437351_at	Region	Cxxc4	319478	3	133121396	133143674	+
1855	1438948_x_at	Region	Bzrp	12257	15	83612210	83636054	+
1856	1444089_at	Region	Spnb2	20742	11	29995224	30114564	-
1857	1436019_a_at	Region	Trappc1	245828	11	69049638	69051452	+
1858	1421990_at	Region	Syt1	20979	10	108385089	108577499	-
1859	1436875_at	Region	Dnm3	103967	1	161896669	162387092	-
1860	1448656_at	Region	Cacnb3	12297	15	98692259	98701666	+
1861	1428883_at	Region	Tmem57	66146	4	133763662	133814033	-
1862	1438206_a_at	Region	2610042O14Rik	66460	2	163917729	163922245	+
1863	1454752_at	Region	AI606861	105200	13	46028781	46029393	+
1864	1423806_at	Region	2610034N24Rik	101861	7	91527289	91587665	+
1865	1434957_at	Region	Cdon	57810	9	35364530	35416801	+
1866	1422248_at	Region	Irs4	16370	X	135151041	135165260	-
1867	1450964_a_at	Region	Osbpl9	100273	4	108020555	108061497	-
1868	1444468_at	Region	Paqr8	74229	1	21115565	21163699	+
1869	1437139_at	Region	Glra1	14654	11	55267654	55360631	-
1870	1416405_at	Region	Bgn	12111	X	68144299	68156586	+
1871	1416719_a_at	Region	Rps10 /// LOC545151	545151	16	19520273	19593779	-
1872	1434136_at	Region	6332401O19Rik	319832	6	25005181	25007613	-
1873	1426885_a_at	Region	Cdk2ap1	13445	5	123516782	123525971	-
1874	1446364_at	Region	Wwp1	107568	4	19537563	19635959	-
1875	1422774_at	Region	1700088E04Rik	27660	15	79186045	79192517	-
1876	1450869_at	Region	Fgf1	14164	18	39062902	39142421	-
1877	1425249_a_at	Region	Tyro3	22174	2	119313419	119331330	+
1878	1451412_a_at	Region	0610009H04Rik	55978	11	78262095	78267132	+
1879	1439887_at	Region	None	None	1	105167738	105169546	-
1880	1418582_at	Region	Cbfa2t3h	12398	8	122011527	122080570	-
1881	1434917_at	Region	Cobl	12808	11	12131470	12359592	-
1882	1425157_x_at	Region	1300010A20Rik	232670	6	29741353	29765655	+
1883	1416133_at	Region	C920006C10Rik	76740	15	65810937	65897708	+
1884	1439933_at	Region	B430316J06Rik	402749	6	94371992	94372407	-
1885	1455898_x_at	Region	Slc2a3	20527	6	123394798	123409491	-
1886	1442099_at	Region	Usp31	76179	7	115517588	115530585	-
1887	1452084_at	Region	2810055E05Rik	230780	3	129343014	129365699	-
1888	1415764_at	Region	Zc3h11a	70579	1	133482838	133516350	-
1889	1436127_at	Region	Crhbp	12919	13	91617276	91630855	-
1890	1419224_at	Region	Cecr6	94047	6	120920913	120925781	-
1891	1436868_at	Region	Rtn4rl1	237847	11	74919459	74992558	+
1892	1453324_at	Region	6330509M23Rik	76155	8	106357470	106358614	+
1893	1417762_a_at	Region	Rpl8	26961	15	76955027	76957242	+
1894	1455401_at	Region	Camkk2	207565	5	121886097	121917203	-
1895	1448305_at	Region	Rab6	19346	7	94714439	94748311	+
1896	1426982_at	Region	Flywch1	224613	17	21557603	21565956	-
1897	1425090_s_at	Region	Kcnc4	99738	3	107234132	107254727	-
1898	1427191_at	Region	Npr2	230103	4	43547857	43567166	+
1899	1438412_at	Region	Phf17	269424	3	41010564	41046427	+
1900	1417963_at	Region	Pltp	18830	2	164296251	164314349	-
1901	1456331_at	Region	None	None	7	122284255	122285468	-
1902	1460558_at	Region	Gm631	269336	2	118532010	118543206	-
1903	1429088_at	Region	MGI:1925139	77889	17	70694580	70718332	+
1904	1424482_at	Region	Arhgef7	54126	8	11130773	11207926	+
1905	1420852_a_at	Region	B3gnt1	53625	11	22729537	22754478	-
1906	1418517_at	Region	Irx3	16373	8	91085097	91087946	-
1907	1448584_at	Region	Rsrc1	66880	3	66658030	67030708	+
1908	1433936_at	Region	0610010E21Rik	68332	7	25734011	25734977	-
1909	1424475_at	Region	Camkk2	207565	5	121886097	121917203	-
1910	1437392_at	Region	LOC433485	433485	2	149287733	149461328	+

1911	1459678_at	Region	LOC497255	497255	15	73452060	73452490	+
1912	1454715_at	Region	0710005M24Rik	76897	3	13473156	14177658	+
1913	1422822_at	Region	Stard5	170460	7	77488447	77498758	+
1914	1452223_s_at	Region	Gcap14	72972	14	35007246	35063441	-
1915	1427501_at	Region	BC007180	209601	3	153757130	153774794	+
1916	1429249_at	Region	4833424O15Rik	75769	3	116349459	116463339	+
1917	1445837_at	Region	Kcnq5	226922	1	22053602	22054434	-
1918	1452363_a_at	Region	Atp2a2	11938	5	121605864	121654245	-
1919	1425293_a_at	Region	1500031H04Rik	66995	X	130538117	130541317	+
1920	1418825_at	Region	Ifi1	15944	11	48618086	48624183	-
1921	1430191_at	Region	9130004J05Rik	71603	15	64302574	64303751	-
1922	1443736_at	Region	4833412E22Rik	330010	4	154536681	154551706	-
1923	1439036_a_at	Region	Atp1b1	11931	1	164355219	164376238	-
1924	1417073_a_at	Region	Qk	19317	17	8850890	8959849	-
1925	1448660_at	Region	Arhgdig	14570	17	23992353	23994520	-
1926	1425701_a_at	Region	Rgs3	50780	4	61651038	61793526	+
1927	1428777_at	Region	Spred1	114715	2	116635298	116693185	+
1928	1418232_s_at	Region	Lims1	110829	10	58281059	58380568	+
1929	1428322_a_at	Region	Ndufb10	68342	17	22527196	22529519	-
1930	1424970_at	Region	Purg	75029	8	32191926	32192989	+
1931	1423946_at	Region	Pdlim2	213019	14	64480207	64493661	-
1932	1431362_a_at	Region	Smoc2	64074	17	12330114	12456677	+
1933	1417920_at	Region	Amn	93835	12	106744502	106749817	+
1934	1426016_a_at	Region	Tro	56191	X	144179698	144191794	-
1935	1457587_at	Region	None	None	1	21625110	21625666	-
1936	1449273_at	Region	Cyfip2	76884	11	45948917	46065130	-
1937	1435769_at	Region	Akap9	100986	5	3934295	4086208	+
1938	1423646_at	Region	Zdhhc3	69035	9	123091926	123130868	-
1939	1437158_at	Region	Nipbl	71175	15	8078809	8232047	-
1940	1419634_a_at	Region	Ghrh	14601	2	156786470	156822010	-
1941	1416735_at	Region	Asah1	11886	8	40278299	40312261	-
1942	1431745_a_at	Region	2700069A02Rik	75553	12	94197945	94213620	+
1943	1443863_at	Region	Fndc3a	319448	14	66891442	67062343	-
1944	1434786_at	Region	None	None	1	134604922	134606571	-
1945	1436957_at	Region	None	None	X	67093331	67094027	-
1946	1422185_a_at	Region	Dia1	109754	15	83204176	83222883	-
1947	1452879_at	Region	Synpo2	118449	3	121904047	121951604	-
1948	1434154_at	Region	Kctd13	233877	7	120978610	120995340	+
1949	1426408_at	Region	1600010O03Rik	109120	2	90722036	90724247	+
1950	1455662_x_at	Region	Rps17	20068	7	75150291	75152774	-
1951	1424679_at	Region	Mab21l1	17116	3	55415255	55417607	+
1952	1438945_x_at	Region	Gja1	14609	10	56627159	56640230	+
1953	1437853_x_at	Region	Ndn	17984	7	56122085	56123665	+
1954	1427483_at	Region	Slc25a24	229731	3	108918891	108977474	+
1955	1415839_a_at	Region	Npm1	18148	11	33047305	33057850	-
1956	1460583_at	Region	Golt1b	66964	6	143230512	143247127	+
1957	1453096_x_at	Region	Rpl27	19942	11	101263502	101266620	+
1958	1435463_s_at	Region	Myo1d	338367	11	80309486	80505684	-
1959	1451190_a_at	Region	Sbk1	104175	7	120322350	120344730	+
1960	1441185_at	Region	Msi2h	76626	11	88067452	88208048	-
1961	1460167_at	Region	Aldh7a1	110695	18	56744086	56781108	-
1962	1450240_a_at	Region	Sytl1	269589	4	132214066	132223965	-
1963	1416003_at	Region	Cldn11	18417	3	30561416	30575743	+
1964	1434875_a_at	Region	Hmgn3	94353	9	83439717	83476376	-
1965	1417576_a_at	Region	Otub2	68149	12	98834218	98851823	+
1966	1430252_at	Region	3110027N22Rik	73168	2	44924163	44925451	-
1967	1425796_a_at	Region	Fgfr3	14184	5	32210377	32225943	+
1968	1418711_at	Region	Pdgfa	18590	5	137975029	137993011	-
1969	1429861_at	Region	Pcdh9	211712	14	88351670	88356571	-
1970	1437287_at	Region	1110020G09Rik	68646	15	8873106	8912017	+
1971	1444019_at	Region	Lrrc19	100061	4	93611732	93625216	-
1972	1448908_at	Region	Ppap2b	67916	4	104116265	104190062	+
1973	1420682_at	Region	Chrnb1	11443	11	69509694	69521601	-
1974	1426511_at	Region	Susd2	71733	10	75738363	75745729	-
1975	1437148_at	Region	Arpc2	76709	1	74538971	74570677	+

1976	1448700_at	Region	G0s2	14373	1	193010932	193011864	-
1977	1449551_at	Region	Myo1c	17913	11	75377236	75400301	+
1978	1455402_at	Region	None	None	11	97218942	97219630	+
1979	1448269_a_at	Region	Klhl13	67455	X	21458211	21603993	-
1980	1416845_at	Region	Hspa5bp1	98170	19	10054330	10066228	-
1981	1429738_at	Region	Myt1l	17933	12	26094752	26491102	+
1982	1435615_at	Region	Zfp365	216049	10	67941416	67966217	-
1983	1422740_at	Region	Tnfrsf21	94185	17	40516102	40588746	+
1984	1435967_s_at	Region	Hibadh	58875	6	52690560	52784625	-
1985	1452585_at	Region	Mrps28	66230	3	8789085	8910754	-
1986	1452981_at	Region	Cntn1	12805	15	92196046	92407969	+
1987	1418000_a_at	Region	Itm2b	16432	14	67713666	67736706	-
1988	1432057_a_at	Region	Prdm5	70779	6	66036866	66194195	+
1989	1423364_a_at	Region	Fts	14339	8	90409865	90421862	-
1990	1450761_s_at	Region	Rims2	116838	15	39098033	39582362	+
1991	1442301_at	Region	AW060742	101823	7	13938474	13939064	+
1992	1416532_at	Region	Trrap	100683	5	143879388	143901968	+
1993	1449365_at	Region	Edg8	94226	9	21129182	21132474	-
1994	1437008_x_at	Region	1110006I15Rik	68539	19	10067157	10078240	-
1995	1439432_x_at	Region	Morf4l2 /// LOC433261	433261	19	584	2363	-
1996	1415967_at	Region	Ndufv1	17995	19	3796295	3801515	-
1997	1452968_at	Region	Cthrc1	68588	15	38976511	38986747	+
1998	1423911_at	Region	Ppp2r5a	226849	1	191088246	191133306	-
1999	1435621_at	Region	Mlstd1	330450	6	149072613	149116582	+
2000	1436458_at	Region	Sema6a	20358	18	47470645	47528251	-
2001	1435532_at	Region	4933426K21Rik	108653	6	123118089	123137380	-
2002	1450905_at	Region	Plxnc1	54712	10	94767461	94918749	-
2003	1447946_at	Region	AW046396	329173	1	63888364	63889295	+
2004	1440801_s_at	Region	Adrbk2	320129	5	111987619	112085665	-
2005	1418838_at	Region	Abcd1	11666	X	68377252	68398942	+
2006	1415918_a_at	Region	Tpi1	21991	6	125467483	125471180	-
2007	1427975_at	Region	2210403B10Rik	75668	11	4952915	4955163	+
2008	1437371_at	Region	9930021J17Rik	229488	3	85403171	85465611	-
2009	1429898_at	Region	2310008M10Rik	66357	3	129626476	129640041	-
2010	1435137_s_at	Region	A130040M12Rik /// E430024C06Rik	319269	NONE	NONE	NONE	NONE
2011	1447624_s_at	Region	Stox2	71069	8	46138301	46167033	-
2012	1454919_at	Region	AU044698	99235	2	3244770	3246144	+
2013	1436463_at	Region	None	None	18	43745458	43746296	-
2014	1424085_at	Region	Ndufa4	17992	6	11884041	11891115	-
2015	1435235_at	Region	Txnl1	53382	18	63894960	63924518	-
2016	1456197_x_at	Region	None	None	NONE	NONE	NONE	NONE
2017	1416372_at	Region	Ptdss1	19210	13	63535264	63600822	+
2018	1441363_at	Region	None	None	X	133975739	133980407	+
2019	1440407_at	Region	None	None	5	107443079	107443898	-
2020	1450928_at	Region	Id4	15904	13	47859485	47862094	+
2021	1441416_at	Region	Inpp4b	234515	8	81357380	81358130	+
2022	1433506_at	Region	Lrrc5	231549	5	104762811	104877194	+
2023	1428373_at	Region	1500005N04Rik	76500	9	108842504	108852864	+
2024	1428854_at	Region	Tmed8	382620	12	84048716	84080007	-
2025	1439506_at	Region	Gm98 /// Gm1804	225908	19	9406882	9439836	-
2026	1416404_s_at	Region	Rps16	20055	7	23752643	23754589	+
2027	1418050_at	Region	Gpld1	14756	13	24423092	24470632	+
2028	1418744_s_at	Region	MGI:1930803	57816	5	117156470	117190516	+
2029	1433666_s_at	Region	Vps41	218035	13	18192301	18341778	+
2030	1428580_at	Region	Blvra	109778	2	126584520	126610937	+
2031	1423225_at	Region	MGI:1931466	80795	14	28102242	28108810	+
2032	1416857_at	Region	Sdf2	20316	11	77971405	77981145	+
2033	1429473_at	Region	1200003I07Rik	66869	8	68854689	68867301	-
2034	1428553_at	Region	2900070E19Rik	73046	12	100476825	100485046	+
2035	1425972_a_at	Region	Zfx	22764	X	88735290	88783558	-
2036	1435730_at	Region	None	None	6	119019827	119020304	-
2037	1426887_at	Region	Nudt11	58242	X	4357660	4364650	+
2038	1424220_a_at	Region	Porcn	53627	X	6432790	6445447	-

2039	1428804_at	Region	Mfap3l	71306	8	59692537	59709342	+
2040	1435978_at	Region	Gmppa	69080	1	75726707	75733896	+
2041	1449335_at	Region	Timp3	21859	10	86261055	86310024	+
2042	1416007_at	Region	Satb1	20230	17	49274277	49367498	-
2043	1437993_x_at	Region	Qdpr	110391	5	44192821	44208924	-
2044	1436031_at	Region	B430218L07Rik	320508	4	99735584	99962654	+
2045	1434369_a_at	Region	Cryab	12955	9	50825084	50828959	+
2046	1418683_at	Region	Lin7b	22342	7	39443916	39446815	-
2047	1437218_at	Region	Fn1	14268	1	71881640	71949288	-
2048	1421929_at	Region	Epha4	13838	1	77656420	77802175	-
2049	1436821_at	Region	Plcxd3	239318	15	4158026	4302949	+
2050	1423176_at	Region	Tob1	22057	11	94034727	94035815	+
2051	1434500_at	Region	Ttyh2	117160	11	114496618	114540296	+
2052	1421140_a_at	Region	Foxp1	108655	6	99395348	99630926	-
2053	1452595_at	Region	Adamts4	240913	1	171179261	171188761	+
2054	1448354_at	Region	G6pdx	14381	X	69070141	69089816	-
2055	1419250_a_at	Region	Pftk1	18647	5	4809738	5386208	-
2056	1419444_at	Region	Sap18	20220	14	52333126	52339849	+
2057	1423995_at	Region	Kif1b	16561	4	147668683	147799980	-
2058	1417927_at	Region	Ddx19a	13680	8	110272730	110295521	-
2059	1434898_at	Region	Tnrc6a	233833	7	117180427	117250357	+
2060	1424827_a_at	Region	Csnk1a1	93687	18	61784394	61817111	+
2061	1455584_at	Region	Sdf4	20318	4	154485276	154505972	+
2062	1457139_at	Region	Auts2	319974	5	130627794	130728707	-
2063	1441081_a_at	Region	1110038B12Rik	68763	17	32654022	32656311	-
2064	1435762_at	Region	Pacs1	107975	19	4922475	5061909	-
2065	1419287_at	Region	MGI:1913570	66320	8	104622006	104624683	+
2066	1424471_at	Region	Rapgef3	223864	15	97820598	97843317	-
2067	1449377_at	Region	Nicn1	66257	9	108360217	108366256	+
2068	1415973_at	Region	Marcks	17118	10	37207003	37211096	-
2069	1438044_at	Region	1700047M11Rik	67330	1	182260714	182261447	+
2070	1426667_a_at	Region	Unc84a	77053	5	138213212	138247853	+
2071	1457404_at	Region	Nfkbiz	80859	16	54703494	54731495	-
2072	1431209_s_at	Region	Tmem42	66079	9	123039099	123041256	+
2073	1450065_at	Region	Adcy7	11513	8	87557754	87614390	+
2074	1426432_a_at	Region	Slc4a4	54403	5	88232301	88532904	+
2075	1428252_at	Region	Chmp2b	68942	16	64543222	64566897	-
2076	1415799_at	Region	Wbp11	60321	6	137585452	137599924	-
2077	1428687_at	Region	Zfp687	78266	3	94494533	94503069	-
2078	1452663_at	Region	1110030H18Rik	68666	5	113137557	113202215	-
2079	1433690_at	Region	2210016L21Rik	72357	5	114051828	114058166	+
2080	1437295_at	Region	Pkn2	109333	3	141762433	141850964	-
2081	1431367_at	Region	38412	72925	8	65495563	65579550	+
2082	1448462_at	Region	Tdg	21665	10	82517850	82538768	+
2083	1460595_at	Region	None	None	1	192629997	192630801	-
2084	1428664_at	Region	Vip	22353	10	4863942	4872338	-
2085	1434787_at	Region	AI854770	105930	15	98793835	98794862	-
2086	1428506_at	Region	Atic	108147	1	71853291	71875521	+
2087	1429254_at	Region	Aqp11	66333	7	91772650	91784518	-
2088	1438961_s_at	Region	Blmh	104184	11	76671417	76713035	+
2089	1440484_at	Region	Unc5d	210801	8	27430583	27997671	-
2090	1426934_at	Region	Nhsl1	215819	10	18451363	18458365	+
2091	1420403_at	Region	Atp2b2	11941	6	114316878	114489190	-
2092	1459327_at	Region	4931406I20Rik	66743	4	116230369	116455820	-
2093	1455245_x_at	Region	Rpl13	270106	8	122483697	122486521	+
2094	1435551_at	Region	FHOS2	225288	18	24937941	25361997	+
2095	1426541_a_at	Region	2310067E08Rik	71946	9	14189509	14216547	-
2096	1460287_at	Region	Timp2	21858	11	118122157	118176401	-
2097	1426338_a_at	Region	Ntng1	80883	3	109582822	109951623	-
2098	1452579_at	Region	Nifun	66383	5	112883532	112889002	+
2099	1427914_a_at	Region	Tceb1	67923	1	16827250	16842139	-
2100	1436789_at	Region	Gm877	380694	11	43281564	43346257	+
2101	1435444_at	Region	Atf6	226641	1	170641954	170802508	-
2102	1422103_a_at	Region	Stat5b	20851	11	100601818	100643615	-
2103	1442823_at	Region	None	None	7	138836969	138837906	+

2104	1422619_at	Region	Ppap2a	19012	13	109194243	109262166	+
2105	1416472_at	Region	Syap1	67043	X	156456257	156487836	-
2106	1455592_at	Region	Ltap	93840	1	171933290	171956187	-
2107	1421127_at	Region	Tmem42	66079	9	123039099	123041256	+
2108	1421471_at	Region	Npy1r	18166	8	65812971	65815231	+
2109	1452666_a_at	Region	Tmcc2	68875	1	132213622	132248587	-
2110	1430288_x_at	Region	Rps21	66481	2	179974367	179975394	+
2111	1415906_at	Region	Tmsb4x	19241	X	160806500	160808554	-
2112	1446537_at	Region	Kcnq5	226922	1	22071665	22072317	-
2113	1416054_at	Region	Rps5	20103	7	10916008	10931517	+
2114	1428372_at	Region	St5	76954	7	103376782	103470018	-
2115	1416768_at	Region	1110003E01Rik	68552	5	64251633	64294659	-
2116	1428866_at	Region	2810037O22Rik	72711	14	54920971	54923068	+
2117	1430124_x_at	Region	Akr1a4	58810	4	115595415	115610556	-
2118	1423690_s_at	Region	Gpsm1	67839	2	26247702	26280397	+
2119	1415878_at	Region	Rrm1	20133	7	96546225	96573284	+
2120	1458364_s_at	Region	MGI:1929890	64164	1	155937531	155950350	+
2121	1455779_at	Region	B430315C20Rik	327655	2	120824412	120864866	-
2122	1435448_at	Region	Bcl2l11	12125	2	127639891	127676025	+
2123	1439374_x_at	Region	None	None	6	13448387	13448604	-
2124	1430068_at	Region	C030011L09Rik	77316	2	106949448	106950583	+
2125	1424313_a_at	Region	Ndufs7	75406	10	80374361	80381701	+
2126	1423512_at	Region	AW209491	105351	13	14074830	14083649	+
2127	1416240_at	Region	Psmb7	19177	2	38520208	38576068	-
2128	1428228_at	Region	Pgm3	109785	9	86894706	86914156	-
2129	1428605_at	Region	1810023B24Rik	234388	8	70023940	70029203	-
2130	1455294_at	Region	1110029L17Rik	68722	15	1072670	1073472	+
2131	1430348_at	Region	2900019E01Rik	72918	15	43477349	43478773	+
2132	1434943_at	Region	BC023055	226123	19	41619314	41623505	-
2133	1453651_a_at	Region	Brp44l	55951	17	16810	30658	+
2134	1455158_at	Region	Itga3	16400	11	94865569	94897801	-
2135	1415753_at	Region	D10Bwg1364e	216169	10	80715282	80721253	-
2136	1418791_at	Region	Sh3gl2	20404	4	84191648	84375572	+
2137	1424434_at	Region	BC024814	239706	16	8144106	8163490	+
2138	1421654_a_at	Region	Lmna	16905	3	88225013	88247171	-
2139	1415850_at	Region	Rasa3	19414	8	12945853	13056187	-
2140	1424070_at	Region	Napg	108123	18	63183763	63228262	+
2141	1415909_at	Region	Stip1	20867	19	6734555	6753790	-
2142	1435987_x_at	Region	1110059G02Rik	68786	15	55640894	55641284	-
2143	1429599_a_at	Region	1110019K23Rik	68621	5	90247014	90341747	+
2144	1434287_at	Region	Agpat5	52123	8	18791504	18829381	+
2145	1425601_a_at	Region	Rtkn	20166	6	83480778	83496265	+
2146	1416528_at	Region	Sh3bgrl3	73723	4	133088305	133089652	-
2147	1439148_a_at	Region	Pfkl	18641	10	78096466	78119117	-
2148	1445309_at	Region	None	None	15	72536992	72537669	-
2149	1450969_at	Region	Pccb	66904	9	100883585	100936422	-
2150	1435436_at	Region	None	None	17	84666021	84667534	+
2151	1417830_at	Region	Smc1l1	24061	X	145550823	145596362	+
2152	1438889_at	Region	None	None	X	115258137	115258803	+
2153	1451734_a_at	Region	Dbn1	56320	13	54082915	54100289	-
2154	1437631_at	Region	Kcnip4	80334	5	47153325	47273714	-
2155	1453007_at	Region	3110082I17Rik	73212	5	138357252	138458005	-
2156	1421027_a_at	Region	Mef2c	17260	13	79604158	79763912	+
2157	1434829_at	Region	Cbl	12402	9	44140278	44222839	-
2158	1419168_at	Region	Mapk6	50772	9	75524483	75535986	-
2159	1416198_at	Region	Th1l	57314	2	173840711	173852404	+
2160	1434224_at	Region	Tbl2	27368	5	134163203	134176154	+
2161	1433743_at	Region	Dach1	13134	14	92301812	92643713	-
2162	1455210_at	Region	Zhx2	387609	15	57703360	57848440	+
2163	1457396_at	Region	None	None	19	58764042	58764713	-
2164	1428683_at	Region	A930016P21Rik	68073	15	31602722	31618141	+
2165	1425913_a_at	Region	2810022L02Rik	67198	1	58079733	58253199	+
2166	1448249_at	Region	Gpd1	14555	15	99774809	99782224	+
2167	1458469_at	Region	Cblb	208650	16	50949437	51125945	+
2168	1424167_a_at	Region	Pmm1	29858	15	82001829	82011581	-

2169	1434762_at	Region	A730041O15Rik	269717	5	135160956	135184148	-
2170	1416430_at	Region	Cat	12359	2	103158757	103189874	-
2171	1415879_a_at	Region	Rplp2	67186	12	72487280	72488111	-
2172	1446282_at	Region	None	None	3	73317668	73347307	+
2173	1436332_at	Region	Hspb6	243912	7	25965994	25968131	+
2174	1423579_a_at	Region	Trpc4	22066	3	53790324	53952687	+
2175	1427307_a_at	Region	Dab1	13131	4	103326495	103639279	+
2176	1423969_at	Region	Nup37	69736	10	88119487	88150839	+
2177	1420963_at	Region	Hapln2	73940	3	87765928	87771304	-
2178	1420171_s_at	Region	Myh9	17886	15	77812545	77894062	-
2179	1453819_x_at	Region	Stx18	71116	5	36545458	36643042	+
2180	1424954_a_at	Region	Pip5k1c	18717	10	81429153	81451529	+
2181	1437397_at	Region	AI987712	105935	15	10144345	10149957	+
2182	1451134_a_at	Region	Tm2d2	69742	8	23740385	23746364	+
2183	1427011_a_at	Region	Lancl1	14768	1	67292813	67331161	-
2184	1433950_at	Region	BC055811	230868	4	138908113	139128045	-
2185	1443344_at	Region	4932409I22Rik	237397	10	79727857	79735002	-
2186	1424765_at	Region	Eps15-rs	13859	8	71489790	71569758	-
2187	1434451_at	Region	2900016G23Rik	78659	13	45124518	45126925	-
2188	1427121_at	Region	Fbxo4	106052	15	3747996	3762108	-
2189	1418784_at	Region	Grik5	14809	7	20188743	20247333	-
2190	1455278_at	Region	Wdr37	207615	13	8762169	8828183	-
2191	1434141_at	Region	Gucy1a3	60596	3	81821497	81874847	-
2192	1417086_at	Region	Pafah1b1	18472	11	74399613	74450328	-
2193	1426794_at	Region	Ptprs	19280	17	54049473	54095417	-
2194	1429382_at	Region	Nr1i3	12355	1	171142875	171147683	+
2195	1449712_s_at	Region	Atp6v1e1	11973	6	121227218	121254659	-
2196	1446157_at	Region	A330019N05Rik	215890	10	33567437	33679703	-
2197	1418518_at	Region	Furin	18550	7	74191895	74205461	-
2198	1437628_s_at	Region	Rhoa	11848	9	108375966	108407597	+
2199	1424886_at	Region	Ptprd	19266	4	75005727	75195151	-
2200	1419544_at	Region	Atp6v1c1	66335	15	38667202	38697131	+
2201	1418706_at	Region	Slc38a3	76257	9	107716936	107733151	-
2202	1436465_at	Region	Klhl1	93688	14	90574765	90989240	-
2203	1455522_at	Region	Arhgef15	442801	11	68673890	68677832	-
2204	1435743_at	Region	Klhl23	277396	2	69677873	69692048	+
2205	1454996_at	Region	Hsdl1	72552	8	118904427	118917664	-
2206	1423548_s_at	Region	Sdbcag84	66366	2	155465054	155475216	+
2207	1427386_at	Region	Arhgef16	230972	4	152770891	152794038	-
2208	1419372_at	Region	Gosr2	56494	11	103497948	103518783	-
2209	1415855_at	Region	Kitl	17311	10	99997658	100082002	+
2210	1433954_at	Region	4632419I22Rik	74038	NONE	NONE	NONE	NONE
2211	1431659_at	Region	C330014O21Rik	68255	NONE	NONE	NONE	NONE
2212	1457935_at	Region	Sdccag33l	228911	2	169281479	169281898	+
2213	1425229_a_at	Region	Tcf7l2	21416	19	55323190	55515901	+
2214	1455429_at	Region	AI450540	226252	19	56946405	56975311	+
2215	1435537_at	Region	Ptprd	19266	4	75004276	75073340	-
2216	1456838_at	Region	LOC237403	237403	10	80964861	80976092	-
2217	1438943_x_at	Region	Rpn1	103963	6	88518156	88540499	+
2218	1417354_at	Region	Sil1	81500	18	35490054	35722531	-
2219	1434018_at	Region	BC043098	214469	1	35082568	35112316	-
2220	1448769_at	Region	Slc35b1	110172	11	95206009	95212739	+
2221	1459903_at	Region	Sema7a	20361	9	58054059	58076850	+
2222	1434555_at	Region	Anp32a	11737	9	62461464	62498838	+
2223	1454758_a_at	Region	Tgfb1i4	21807	14	70758316	70850256	+
2224	1416087_at	Region	Ap1s1	11769	5	136048116	136059182	-
2225	1443170_at	Region	None	None	19	43040899	43041517	+
2226	1419286_s_at	Region	Cdv1	12589	5	121702852	121766929	-
2227	1421917_at	Region	Pdgfra	18595	5	73986573	74032425	+
2228	1428529_at	Region	2810026P18Rik	72655	9	88882596	88884174	-
2229	1457899_at	Region	Kalrn	224126	16	32818840	32825621	-
2230	1457198_at	Region	Nrp1	18186	8	127666747	127814326	+
2231	1422549_at	Region	Arl2	56327	19	5923179	5929927	-
2232	1452742_at	Region	2310001H13Rik	67095	9	121314022	121472320	+
2233	1450053_at	Region	Kif2a	16563	13	103169548	103230223	-

2234	1416843_at	Region	Pde6d	18582	1	86348646	86388343	-
2235	1416374_at	Region	Ap3m1	55946	14	19399475	19416850	-
2236	1448289_at	Region	Crmp1	12933	5	35752304	35798645	+
2237	1435873_a_at	Region	Rpl13a /// Zfp526	210172	7	20401813	20408380	+
2238	1454742_at	Region	Rasgef1b	320292	5	98235569	98270997	-
2239	1420477_at	Region	Nap1l1	53605	10	111105541	111122931	+
2240	1420938_at	Region	Hs6st2	50786	X	45909852	46201195	-
2241	1433501_at	Region	Ctso	229445	3	81661676	81685785	+
2242	1448219_a_at	Region	Ywhaz	22631	15	36773662	36797651	-
2243	1437968_at	Region	Grin1	14810	2	25224409	25251141	-
2244	1437866_at	Region	Dusp18	75219	11	3790068	3796083	+
2245	1437724_x_at	Region	Pitpnm1	18739	19	3889412	3902751	+
2246	1436862_at	Region	Doc2a	13446	7	120897284	120902436	+
2247	1415788_at	Region	BC002236	79560	11	44207410	44223282	-
2248	1451507_at	Region	Mef2c	17260	13	79604158	79763912	+
2249	1418375_at	Region	D10Wsu93e	110962	10	127018595	127020280	-
2250	1452264_at	Region	Tenc1	209039	15	102161590	102175759	+
2251	1415793_at	Region	Pnpo	103711	11	96758914	96765030	-
2252	1456856_at	Region	Ppfia2	327814	10	106357920	106820120	+
2253	1423853_at	Region	6330527O06Rik	76161	2	135572081	135583763	+
2254	1439622_at	Region	Rassf4	213391	6	117070312	117109861	-
2255	1457281_at	Region	4930461P20Rik	78244	15	10260892	10284727	-
2256	1418743_a_at	Region	MGI:1930803	57816	5	117156470	117190516	+
2257	1435404_at	Region	Disp2	214240	2	118293767	118309024	+
2258	1417946_at	Region	Abhd3	106861	18	10689755	10752040	-
2259	1434532_at	Region	BC035295	207785	15	100537108	100552777	-
2260	1428095_a_at	Region	Tmem24	71764	9	44299070	44309001	-
2261	1415824_at	Region	Scd2	20250	19	43838494	43851266	+
2262	1433884_at	Region	Syt1	20979	10	108385089	108577499	-
2263	1426759_at	Region	Map4k3	225028	17	78411242	78558951	-
2264	1436326_at	Region	Rora	19883	9	69474948	69510983	+
2265	1436263_at	Region	Mobp	17433	9	120159695	120186044	+
2266	1446286_at	Region	Kirrel3	67703	9	34554549	34555109	+
2267	1448312_at	Region	Pcsk2	18549	2	143003115	143273220	+
2268	1456497_x_at	Region	Rps10	67097	17	25426347	25431160	-
2269	1434779_at	Region	Cbln2	12405	18	86883230	86888465	+
2270	1435437_at	Region	MGI:1920501	73251	3	51152457	51192611	-
2271	1455659_at	Region	Tmcc1	330401	6	116456153	116631023	-
2272	1422052_at	Region	Cdh8	12564	8	98317068	98691929	-
2273	1426756_at	Region	Galnt2	108148	8	123518466	123632794	+
2274	1421571_a_at	Region	Ly6c	17067	15	75077718	75081535	-
2275	1455409_at	Region	Spire1	68166	18	67718995	67783507	-
2276	1440551_at	Region	Dnajc1	13418	2	18259145	18434517	-
2277	1435781_at	Region	Cand1	71902	10	118778384	118938138	-
2278	1417986_at	Region	Nrarp	67122	2	25113185	25115487	+
2279	1449929_at	Region	Tcte1l	67117	X	7893210	7901923	-
2280	1428578_s_at	Region	Ppfia4	68507	1	134149042	134182786	-
2281	1416608_a_at	Region	BC004004	80748	17	27090378	27115403	+
2282	1418982_at	Region	Cebpa	12606	7	30276207	30277487	+
2283	1419364_a_at	Region	Rps7	20115	12	25196338	25201165	-
2284	1438027_at	Region	None	None	NONE	NONE	NONE	NONE
2285	1432417_a_at	Region	Tspan2	70747	3	102162658	102198922	+
2286	1448883_at	Region	Lgmn	19141	12	97839582	97885181	-
2287	1440210_at	Region	Cacng2	12300	15	78046913	78171273	-
2288	1423283_at	Region	Pitpna	18738	11	75313766	75354436	+
2289	1458134_at	Region	None	None	NONE	NONE	NONE	NONE
2290	1456663_x_at	Region	Tm2d2	69742	8	23740385	23746364	+
2291	1429262_at	Region	Rassf6	73246	5	89900673	89938050	-
2292	1425781_a_at	Region	Plcb1	18795	2	134734571	134986275	+
2293	1451224_at	Region	Scamp5	56807	9	57554973	57581670	-
2294	1439968_x_at	Region	None	None	2	163948096	163950053	+
2295	1455376_at	Region	1300010F03Rik	219189	14	73122033	73556355	+
2296	1426790_at	Region	Ssrp1	20833	2	84735021	84744858	+
2297	1423610_at	Region	Metap2	56307	10	93834268	93863130	-
2298	1451008_at	Region	St8sia3	20451	18	64496670	64507426	+

2299	1454768_at	Region	Kcnf1	382571	12	16538786	16543211	-
2300	1452765_at	Region	Slc39a9	328133	12	77505138	77544592	+
2301	1417053_at	Region	Phb	18673	11	95488102	95501852	+
2302	1417043_at	Region	Lcat	16816	8	105235399	105239229	-
2303	1423275_at	Region	Ddx26	18130	14	57209161	57294009	-
2304	1435745_at	Region	5031439G07Rik	223739	15	84997544	85036545	-
2305	1423151_at	Region	Dnajb11	67838	16	21641386	21655847	+
2306	1416407_at	Region	Pea15	18611	1	172081687	172129498	-
2307	1431065_at	Region	Eaf1	74427	14	29627014	29641729	+
2308	1450648_s_at	Region	H2-Ab1 /// Rmcs1	114887	17	31964476	31970586	+
2309	1460043_at	Region	None	None	15	89829889	89830574	-
2310	1431055_a_at	Region	Snx10	71982	6	51687522	51734165	+
2311	1416606_s_at	Region	Nola2	52530	11	51372614	51376555	+
2312	1448201_at	Region	Sfrp2	20319	3	83502189	83510183	+
2313	1460695_a_at	Region	2010111I01Rik	72061	13	60400470	60681842	+
2314	1417677_at	Region	Opn3	13603	1	175595136	175625299	-
2315	1452196_a_at	Region	Nckap1	50884	2	80199044	80278714	-
2316	1455023_at	Region	N28178	230085	4	42833173	42857529	+
2317	1460632_at	Region	Rdh10	98711	1	16289336	16316005	+
2318	1436697_at	Region	Dscr3	13185	16	93894798	93923338	-
2319	1416155_at	Region	Hmgb3	15354	X	66224484	66229145	+
2320	1434846_at	Region	1700065A05Rik	329877	4	85760447	85836008	+
2321	1418690_at	Region	Ptprz1	19283	6	22927206	23056382	+
2322	1424718_at	Region	Mapt	17762	11	104052587	104149347	+
2323	1454619_at	Region	AI451006	105847	15	89403881	89408357	-
2324	1417885_at	Region	Mapt	17762	11	104052587	104149347	+
2325	1418237_s_at	Region	Col18a1	12822	10	77153138	77270740	-
2326	1456375_x_at	Region	Trim27	19720	13	20660436	20672921	+
2327	1423290_at	Region	Hyou1	12282	9	44368440	44381225	+
2328	1452206_at	Region	Sucla2	20916	14	67904221	67947577	+
2329	1456090_at	Region	Pdhx	27402	2	102725831	102778265	-
2330	1428570_at	Region	Ccnc	51813	4	21797101	21819917	+
2331	1460451_at	Region	Tmem52	69671	4	153961476	153963219	+
2332	1426548_a_at	Region	5730421E18Rik	66632	2	114030258	114168768	-
2333	1439068_at	Region	MGI:1933403	80898	13	70696832	70749086	+
2334	1452130_at	Region	2310042M24Rik	66958	2	84369066	84375796	-
2335	1432416_a_at	Region	Npm1	18148	11	33047305	33057850	-
2336	1437545_at	Region	Rcor1	217864	12	106514818	106586780	+
2337	1420631_a_at	Region	Blcap	53619	2	157013332	157023330	-
2338	1460689_at	Region	D15Wsu75e	28075	15	82043240	82066857	-
2339	1460385_a_at	Region	Zfp179	22671	11	61174084	61179528	-
2340	1420901_a_at	Region	Hk1	15275	10	62236465	62346218	-
2341	1428142_at	Region	Etv5	104156	16	21153380	21211575	-
2342	1459913_at	Region	Tnfsf10	22035	3	26734898	26753894	+
2343	1427411_s_at	Region	Dleu2	328425	14	56136336	56215549	-
2344	1451289_at	Region	Dcamkl1	13175	3	54876964	55171014	+
2345	1451216_at	Region	Zfp330	30932	8	82004797	82015303	-
2346	1425025_at	Region	0610008L10Rik	217203	11	101403405	101412000	+
2347	1420337_at	Region	Gbx2	14472	1	89746512	89749726	-
2348	1422567_at	Region	Niban	63913	1	151492935	151576687	+
2349	1426823_s_at	Region	Psme4	103554	11	30666852	30774524	+
2350	1445539_at	Region	Pde7b	29863	10	20324950	20654021	-
2351	1425760_a_at	Region	Pitpnm1	18739	19	3889412	3902751	+
2352	1433510_x_at	Region	Rpl36	54217	17	54301885	54302698	+
2353	1446921_at	Region	None	None	11	11896001	11896574	-
2354	1424842_a_at	Region	Arhgap24	231532	5	101508673	101932043	+
2355	1429269_at	Region	LOC73072	73072	8	3564501	3572270	-
2356	1426212_s_at	Region	BC021367	234371	8	69325384	69336638	+
2357	1433793_s_at	Region	Nrip2	60345	6	129089953	129098438	+
2358	1446612_at	Region	9330118A15Rik	319756	4	25146480	25147122	+
2359	1421166_at	Region	Atrn	11990	2	130420349	130541594	+
2360	1429592_at	Region	Lhfpl3	269629	5	21183622	21739491	+
2361	1434932_at	Region	Adarb1	110532	10	77405789	77470808	-
2362	1423033_at	Region	Itm1	16430	9	36668263	36704803	-
2363	1433648_at	Region	Spag9	70834	11	93817190	93944505	+

2364	1448751_at	Region	Ap3m2	64933	8	21541934	21560210	-
2365	1424091_at	Region	AI449175	234362	8	68761621	68776282	-
2366	1437193_s_at	Region	Snrpb	20638	2	129685499	129693214	-
2367	1424160_at	Region	Alg5	66248	3	54369759	54384015	+
2368	1435848_at	Region	None	None	2	103847827	103848529	-
2369	1417367_at	Region	Ppp2ca	19052	11	51851665	51875590	+
2370	1421814_at	Region	None	None	X	90697403	90769918	+
2371	1420150_at	Region	4930422J18Rik	74646	4	148388645	148447368	-
2372	1418500_at	Region	Nap1l3	54561	X	116744828	116747653	-
2373	1420427_a_at	Region	Dhx32	101437	7	128088696	128144455	-
2374	1460386_a_at	Region	Slc1a1	20510	19	28087763	28166557	+
2375	1424274_at	Region	MGI:1929095	56041	5	91473766	91538623	+
2376	1455793_at	Region	BC035537	212326	8	44287329	44330422	-
2377	1434773_a_at	Region	Slc2a1	20525	4	118067673	118096232	+
2378	1419470_at	Region	Gnb4	14696	3	31991704	32024713	-
2379	1423022_at	Region	Adra2a	11551	19	53625137	53626489	+
2380	1451506_at	Region	Mef2c	17260	13	79604158	79763912	+
2381	1434892_x_at	Region	Rbbp4	19646	4	128334213	128362483	-
2382	1441845_at	Region	Caps2	353025	10	111832648	111885573	+
2383	1428469_a_at	Region	Dzip1	66573	14	113431542	113479731	-
2384	1458104_a_at	Region	BC038167	270150	9	44229553	44236161	+
2385	1448530_at	Region	Gmpr	66355	13	45082625	45121536	+
2386	1418263_at	Region	Ddx25	30959	9	35454284	35470923	-
2387	1454604_s_at	Region	Tspan12	269831	6	21818432	21899551	-
2388	1448556_at	Region	Prlr	19116	15	9991399	10143512	+
2389	1440425_at	Region	Odz2	23964	11	35801678	36738275	-
2390	1448533_at	Region	Ckap1	66411	7	25637313	25645090	-
2391	1440545_at	Region	None	None	10	10560914	10561650	-
2392	1436328_at	Region	Rnmt	67897	18	68531175	68554593	+
2393	1451047_at	Region	Itm2a	16431	X	101998560	102004725	-
2394	1456258_at	Region	Emx2	13797	19	59057931	59064916	+
2395	1430286_s_at	Region	Ppp1r14c	76142	10	7041618	7140096	-
2396	1455717_s_at	Region	Daam2	76441	17	46989103	47096816	-
2397	1450745_at	Region	C1galt1	94192	6	7821663	7848379	+
2398	1418038_s_at	Region	Dusp19	68082	2	80314963	80329410	+
2399	1451041_at	Region	Rock2	19878	12	16262121	16345038	+
2400	1435926_at	Region	E030003F13Rik	98357	1	175614946	175616280	-
2401	1437568_at	Region	Mmp16	17389	4	17780448	18043598	+
2402	1416239_at	Region	Ass1	11898	2	31402432	31452832	+
2403	1416153_at	Region	Srp54	24067	12	51906616	51907500	+
2404	1417286_at	Region	Ndufa5	68202	6	24568151	24577172	-
2405	1458129_at	Region	Rora	19883	9	69474948	69510983	+
2406	1419267_at	Region	Nfyb	18045	10	82637842	82652084	-
2407	1420798_s_at	Region	Pcdha4 /// Pcdha6 /// Pcdha7 /// Pcdha5 /// Pcdha11 /// Pcdha10 /// Pcdha1 /// Pcdha9 /// Pcdha3 /// Pcdha12 /// Pcdha2 /// Pcdha8 /// Pcdhac1 /// Pcdhac2	116731	18	37154024	37411380	+
2408	1424823_s_at	Region	9630044O09Rik	105439	14	98195231	98249790	+
2409	1437569_at	Region	Gm644	270893	11	82128559	82185986	+
2410	1454630_at	Region	BC034054	217125	11	94830966	94847171	+
2411	1427432_a_at	Region	Sfrs10	20462	16	21019108	21039299	-
2412	1426769_s_at	Region	Maml1	103806	11	50008479	50019709	-
2413	1427364_a_at	Region	Odc1 /// LOC545783 /// LOC546355	18263	12	16906701	16912688	+
2414	1459890_s_at	Region	1110008P14Rik	73737	2	32311270	32314046	-
2415	1429857_at	Region	2900092D14Rik	73100	1	42995052	42996125	+
2416	1426465_at	Region	Dlgap4	228836	2	156157648	156219872	+

2417	1426447_at	Region	Nup35	69482	2	80336563	80356651	+
2418	1423444_at	Region	Rock1	19877	18	10112811	10226661	-
2419	1453070_at	Region	Gm78	219228	14	78894017	78896229	+
2420	1423753_at	Region	Bambi	68010	18	3512543	3520990	+
2421	1440346_at	Region	Jmjd3	216850	11	69124177	69124997	+
2422	1425718_a_at	Region	Ivns1abp	117198	1	151200258	151220009	+
2423	1423712_a_at	Region	Qars	97541	9	108556974	108564815	+
2424	1430752_at	Region	C330006D17Rik	77616	9	7841818	7843031	-
2425	1422465_a_at	Region	Nxn	18230	11	75983616	76124799	-
2426	1423202_a_at	Region	Ncor1	20185	11	62043183	62164152	-
2427	1454621_s_at	Region	5730472N09Rik	108958	2	30296497	30317681	+
2428	1421622_a_at	Region	Rapgef4	56508	2	71679030	71954119	+
2429	1455149_at	Region	Sh3md2	59009	8	60271818	60461252	+
2430	1427522_at	Region	Arhgap20	244867	9	51837391	51925006	+
2431	1421201_a_at	Region	Tro	56191	X	144179698	144191794	-
2432	1441751_at	Region	AW493563	101365	6	67546604	67547052	+
2433	1434565_at	Region	Cgrrf1	68755	14	41912127	41934073	+
2434	1453384_at	Region	4632404N19Rik	77060	1	138939816	138983492	+
2435	1424119_at	Region	Prkab1	19079	5	115135107	115145945	-
2436	1448361_at	Region	Ttc3	22129	16	93778128	93866771	+
2437	1445008_at	Region	1600019O04Rik	72007	3	26833958	27060578	-
2438	1418478_at	Region	Lmo1	109594	7	102991520	103023462	-
2439	1429033_at	Region	Gcc1	74375	6	28464824	28475387	-
2440	1439274_at	Region	A230083H22Rik	353211	19	16360850	16417892	+
2441	1435704_at	Region	C920006O11Rik	320295	9	78344674	78347716	+
2442	1422184_a_at	Region	Ak1	11636	2	32561666	32567214	+
2443	1452211_at	Region	Psme4	103554	11	30666852	30774524	+
2444	1456871_a_at	Region	Phf20l1	239510	15	66601624	66648123	+
2445	1416214_at	Region	Mcm4	17217	16	14393171	14406424	-
2446	1434121_at	Region	Lgi4	243914	7	26468265	26479106	+
2447	1460465_at	Region	A930038C07Rik	68169	6	65929438	65964757	+
2448	1447725_at	Region	C030034E14Rik	77469	X	97617303	97617574	+
2449	1446059_at	Region	2900086E13Rik	73057	3	151969763	151970485	-
2450	1452291_at	Region	Centd1	212285	5	61409120	61555599	-
2451	1415933_a_at	Region	Cox5a	12858	9	57635235	57646353	+
2452	1449243_a_at	Region	Rps19	20085	7	20062265	20067353	+
2453	1449630_s_at	Region	Mark1	226778	1	184396055	184497931	-
2454	1457142_at	Region	Efcbp1	69352	4	14879213	15076079	-
2455	1428334_at	Region	Ostm1	14628	10	42785277	42808815	+
2456	1431502_a_at	Region	Exosc3	66362	4	45232551	45236525	-
2457	1422168_a_at	Region	Bdnf	12064	2	109298487	109350793	+
2458	1434100_x_at	Region	None	None	5	50264886	50265410	-
2459	1428971_at	Region	5730405I09Rik	67974	18	9356614	9490220	-
2460	1419367_at	Region	Decr1	67460	4	15844205	15872472	-
2461	1419272_at	Region	Myd88	17874	9	119330249	119334301	-
2462	1417695_a_at	Region	Soat1	20652	1	156336595	156379733	-
2463	1423967_at	Region	Palm	18483	10	79929264	79943520	+
2464	1424311_at	Region	0710008K08Rik	69225	8	10870028	10885710	+
2465	1448461_a_at	Region	1500006O09Rik	66231	14	12411167	12423386	-
2466	1442311_at	Region	None	None	15	11935334	11935727	+
2467	1446621_at	Region	Chd7	320790	4	8617703	8793952	+
2468	1426068_at	Region	Slc7a4	224022	16	16343928	16347174	-
2469	1438417_at	Region	Pwwp2	101631	7	133644194	133662714	+
2470	1422935_x_at	Region	Cbx1	12412	11	96610246	96629727	+
2471	1441782_at	Region	Aak1	269774	6	87421662	87422107	+
2472	1438685_at	Region	Zfp258	100177	4	126104496	126151407	+
2473	1438288_x_at	Region	1110059G02Rik	68786	15	55640896	55641301	-
2474	1424002_at	Region	Pdcl3	68833	1	39284731	39294153	+
2475	1424876_s_at	Region	Spg20	229285	3	54746476	54770293	+
2476	1416338_at	Region	Sh3gl1	20405	17	53654884	53674711	-
2477	1435527_at	Region	1500041O16Rik	69000	10	81533107	81535984	-
2478	1448583_at	Region	AA960436	101985	8	94616511	94631989	+
2479	1417423_at	Region	Grina	66168	15	76295479	76298576	+
2480	1436424_at	Region	1600020E01Rik	72012	6	86961038	86999299	+
2481	1457334_at	Region	C130057M05Rik	399581	2	59038211	59038889	+

2482	1448869_a_at	Region	Mrps16	66242	14	18752543	18754867	-
2483	1418580_at	Region	5830458K16Rik	67775	16	22393472	22397762	+
2484	1421871_at	Region	Sh3bgrl	56726	X	103696772	103763832	+
2485	1443526_at	Region	Bhc80	192285	2	91918732	92065593	+
2486	1423739_x_at	Region	Aplp2	11804	9	31068390	31130767	-
2487	1456900_at	Region	None	None	NONE	NONE	NONE	NONE
2488	1435286_at	Region	AW125296	100928	5	83313855	83315294	-
2489	1450191_a_at	Region	Sox13	20668	1	133243646	133254561	-
2490	1435952_at	Region	None	None	14	70783174	70784116	+
2491	1433871_at	Region	R3hdm	226412	1	127946018	128080726	+
2492	1418754_at	Region	Adcy8	11514	15	64715509	64946754	-
2493	1436303_at	Region	Mllt4	17356	17	11961032	11968166	+
2494	1450268_at	Region	Fign	60344	2	63833231	63953388	-
2495	1435078_at	Region	3526402J09Rik	237960	11	105747891	105750394	+
2496	1451101_a_at	Region	Rps28	54127	17	31521434	31522877	-
2497	1425525_a_at	Region	P2rx4	18438	5	121860532	121881953	+
2498	1416958_at	Region	Nr1d2	353187	14	16722078	16757007	-
2499	1455070_at	Region	AL118268	404593	18	44642566	44644468	+
2500	1449091_at	Region	Cldn8	54420	16	87717961	87720270	-
2501	1420824_at	Region	Sema4d	20354	13	50254932	50347328	-
2502	1423368_at	Region	Laptm4a	17775	12	8237726	8255043	+
2503	1434115_at	Region	None	None	8	118667300	118668747	+
2504	1455147_at	Region	None	None	17	66619665	66620418	+
2505	1418819_at	Region	Arl10c	67166	6	109286299	109326908	+
2506	1433169_at	Region	5830456J23Rik	76105	8	80748226	80749437	+
2507	1452350_at	Region	Brd8	78656	18	34822848	34847583	-
2508	1417704_a_at	Region	Arhgap6	11856	X	162395378	162903843	+
2509	1435452_at	Region	Tmem20	240660	19	37739783	37749410	+
2510	1417594_at	Region	Gkap1	56278	13	56862150	56893001	-
2511	1442561_at	Region	Mamdc1	320772	12	63250963	63650479	-
2512	1452371_at	Region	Sfrs11	69207	3	157020040	157032607	-
2513	1419659_s_at	Region	Chic2	74277	5	73840608	73878810	-
2514	1454764_s_at	Region	None	None	15	96638716	96639445	-
2515	1436278_at	Region	None	None	X	15794540	15795257	-
2516	1427940_s_at	Region	Mycbp	56309	4	122932163	122939256	+
2517	1437974_a_at	Region	Hk1	15275	10	62236465	62346218	-
2518	1417592_at	Region	Frap1	56717	4	146940969	147050045	+
2519	1438181_x_at	Region	Tm2d2	69742	8	23740385	23746364	+
2520	1434602_at	Region	Thrap2	76199	5	117691302	117893539	+
2521	1436836_x_at	Region	Cnn3	71994	3	120205773	120237098	+
2522	1437885_at	Region	D030029J20Rik	99324	2	84285069	84285864	-
2523	1436555_at	Region	AI158848	101999	8	39856551	39857966	+
2524	1417023_a_at	Region	Fabp4	11770	3	10205894	10210101	-
2525	1423332_at	Region	Sdcbp	53378	4	6304221	6322796	+
2526	1439050_at	Region	AI649393	99692	3	121046233	121048320	+
2527	1437497_a_at	Region	Hspca /// LOC544800 /// LOC545508	15519	12	106166269	106171628	-
2528	1443870_at	Region	Abcc4	239273	14	113037659	113045535	-
2529	1453257_at	Region	Agpat5	52123	8	18791504	18829381	+
2530	1460323_at	Region	Tars	110960	15	11193522	11211606	-
2531	1420859_at	Region	Pkia	18767	3	7341242	7419938	+
2532	1439633_at	Region	B230112P13Rik	109325	19	9648338	9649678	+
2533	1451867_x_at	Region	Arhgap6	11856	X	162395378	162903843	+
2534	1423161_s_at	Region	Spred1	114715	2	116635298	116693185	+
2535	1450712_at	Region	Kcnj9	16524	1	172251006	172258201	-
2536	1423665_a_at	Region	Rpl5	19983	5	106971039	106978499	+
2537	1451197_s_at	Region	Gatad2a	234366	8	69059749	69147743	-
2538	1434094_at	Region	6330530A05Rik	230678	4	117499847	117502633	-
2539	1434064_at	Region	BC061259	269999	7	121819655	121824814	+
2540	1455205_a_at	Region	Usp19	71472	9	108541037	108550266	+
2541	1416952_at	Region	Atp6v1d	73834	12	75699581	75718058	-
2542	1450726_at	Region	Asah2	54447	19	31310118	31428612	-
2543	1428966_at	Region	2610204K14Rik	67148	7	75571092	75577629	+
2544	1460220_a_at	Region	Csf1	12977	3	107536887	107556052	-

2545	1417981_at	Region	Insig2	72999	1	121067561	121095788	-
2546	1431230_a_at	Region	Btbd9	224671	17	28030141	28340405	-
2547	1449275_at	Region	2310038H17Rik	67099	1	64900564	64911252	-
2548	1426811_at	Region	Ppp2r5b	225849	19	6016555	6024421	-
2549	1416107_at	Region	Nsg2	18197	11	31895253	31953996	+
2550	1421959_s_at	Region	Adcy3	104111	12	3286276	3366296	+
2551	1448211_at	Region	Atp6v0e2	76252	6	48670494	48674677	+
2552	1425987_a_at	Region	Kcnma1	16531	14	21672901	22164598	-
2553	1431058_at	Region	Htatsf1	72459	X	51808329	51821897	+
2554	1435896_at	Region	Sfxn2	94279	19	46120441	46142562	+
2555	1419359_at	Region	Hexim1	192231	11	102937414	102940814	+
2556	1424494_s_at	Region	2810417J12Rik	76917	17	21579098	21588259	-
2557	1423596_at	Region	Nek6	59126	2	38443862	38520203	+
2558	1427552_a_at	Region	Gstz1	14874	12	84027344	84044349	+
2559	1452832_s_at	Region	Cds2	110911	2	131777151	131821546	+
2560	1451663_a_at	Region	Trim3	55992	7	99724514	99748443	-
2561	1431081_a_at	Region	Plscr3	70310	11	69572334	69577716	+
2562	1455720_at	Region	Adamts2	216725	11	50354926	50556890	+
2563	1431239_at	Region	Nono	53610	X	96031077	96049950	+
2564	1427489_at	Region	Itga8	241226	2	12022669	12217929	-
2565	1425553_s_at	Region	Hip1r	29816	5	123144623	123174525	+
2566	1436648_at	Region	Nanos1	332397	19	60357055	60358437	+
2567	1428938_at	Region	Gnaq	14682	19	15357188	15609698	+
2568	1429881_at	Region	Arhgap15	76117	2	43681013	44318825	+
2569	1451040_at	Region	Hars2	66044	2	144056919	144225657	+
2570	1424391_at	Region	Nrd1	230598	4	107959710	108020676	+
2571	1434139_at	Region	Parp11	101187	6	128134841	128183937	+
2572	1437133_x_at	Region	Akr1b3 /// LOC432549	11677	6	34398202	34411739	-
2573	1421252_a_at	Region	Mef2a	17258	7	61122705	61262817	-
2574	1452083_a_at	Region	Pja1	18744	X	94067507	94072041	-
2575	1426738_at	Region	Dgkz	104418	2	91637575	91668348	-
2576	1425089_at	Region	Kcnc4	99738	3	107234132	107254727	-
2577	1426342_at	Region	1300006C19Rik	68292	9	115225956	115293786	-
2578	1450560_a_at	Region	Ppp2r5d	21770	17	44193595	44215645	-
2579	1435363_at	Region	Plekhg1	213783	10	6541247	6634764	-
2580	1437069_at	Region	Osbpl8	237542	10	110787653	110920098	+
2581	1455599_at	Region	AI850995	328232	13	42757680	42758797	-
2582	1428876_at	Region	Srp72	66661	5	76219172	76244365	+
2583	1428213_at	Region	2410003A14Rik	67872	7	124655799	124696376	-
2584	1445498_at	Region	Ryr2	20191	13	11561136	11985749	-
2585	1435625_at	Region	Entpd7	93685	19	43234237	43276021	+
2586	1450368_a_at	Region	Ppp3r1 /// AI553587	103784	11	17106711	17128589	+
2587	1435265_at	Region	None	None	15	58184883	58186400	-
2588	1428484_at	Region	Osbpl3	71720	6	50441339	50527369	-
2589	1417041_at	Region	Rpo1-1	20016	17	43753849	43757884	-
2590	1426662_at	Region	Cmas	12764	6	143600335	143622749	+
2591	1434266_at	Region	AI847670	330050	5	19877356	19886950	+
2592	1424271_at	Region	Dcamkl1	13175	3	54876964	55171014	+
2593	1446280_at	Region	Foxp1	108655	6	99395348	99630926	-
2594	1458306_at	Region	F630022B06Rik	239827	16	30743325	30755417	+
2595	1442050_at	Region	Zfp608	269023	18	55107188	55211729	-
2596	1422621_at	Region	Ranbp2	19386	10	58404785	58452252	+
2597	1434406_at	Region	Srgap2	14270	1	131135970	131377678	-
2598	1428537_at	Region	Csnk1a1	93687	18	61784394	61817111	+
2599	1416598_at	Region	Glis2	83396	16	4266217	4287457	+
2600	1448131_at	Region	Mfn2	170731	4	146365961	146397066	-
2601	1424764_at	Region	Sez6l	56747	5	111489140	111647147	-
2602	1449011_at	Region	Slc12a7	20499	13	69823556	69876719	+
2603	1451348_at	Region	Depdc6	97998	15	55114270	55254734	+
2604	1436642_x_at	Region	AI849053	329697	3	87942302	87947130	-
2605	1418116_at	Region	MGI:1929890	64164	1	155937531	155950350	+
2606	1423931_s_at	Region	Anapc4	52206	5	51644274	51676873	+
2607	1434854_a_at	Region	Rps10	67097	17	25426347	25431160	-

2608	1416647_at	Region	Bckdha	12039	7	20794453	20823194	-
2609	1452862_at	Region	B930013M22Rik	68750	13	37486050	37490651	+
2610	1419749_at	Region	Dnmt2	13434	2	13426173	13460673	-
2611	1455950_x_at	Region	Rpl35	66489	2	38933743	38937293	-
2612	1456071_a_at	Region	Cycs	13063	6	50708965	50711007	-
2613	1448549_a_at	Region	Dpagt1	13478	9	44315700	44322455	+
2614	1422779_at	Region	Smpd3	58994	8	105548395	105633835	-
2615	1422895_at	Region	Vamp4	53330	1	162480609	162508875	+
2616	1419874_x_at	Region	Zbtb16	235320	9	48684510	48863820	-
2617	1442556_at	Region	None	None	14	73072014	73072676	-
2618	1435380_at	Region	None	None	11	63688277	63689440	-
2619	1458295_at	Region	BC038331	408056	3	88078606	88078982	-
2620	1418553_at	Region	Arhgef18	102098	8	4103	13701	-
2621	1428987_at	Region	Dncl2b	75465	8	115826983	115837883	+
2622	1448707_at	Region	Taf13	99730	3	108367441	108377810	+
2623	1434398_at	Region	9430034D17Rik	77286	X	31508160	31510142	-
2624	1437576_at	Region	2810427A07Rik	72767	17	33639368	33640114	+
2625	1416634_at	Region	5730536A07Rik	68250	9	66249938	66262282	+
2626	1416655_at	Region	C1galt1c1	59048	X	33151587	33155905	-
2627	1437199_at	Region	Gm337	240672	19	53094474	53112857	+
2628	1420839_at	Region	Plekha3	83435	2	76373044	76394322	+
2629	1415821_at	Region	Sdfr1	20320	9	58698947	58769508	+
2630	1454301_at	Region	2900073C17Rik	73020	8	47806282	47807725	-
2631	1458386_at	Region	C030009J22Rik	320436	12	97716964	97717978	+
2632	1434001_at	Region	2210019E14Rik	70387	19	8007843	8015835	-
2633	1434595_at	Region	Trim9	94090	12	67079894	67179488	-
2634	1425348_a_at	Region	Srprb	20818	9	103163286	103177389	-
2635	1438115_a_at	Region	Slc9a3r1	26941	11	114984496	115002271	+
2636	1440841_at	Region	Ywhae	22627	11	75458597	75491496	+
2637	1429765_at	Region	1500005K14Rik	76566	11	75746247	75753441	-
2638	1452163_at	Region	Ets1	23871	9	32617946	32676163	+
2639	1437556_at	Region	Zfhx4	80892	3	5216397	5389236	+
2640	1443139_at	Region	None	None	2	112100460	112100964	-
2641	1426830_a_at	Region	Ahcyl1	229709	3	107460335	107492339	-
2642	1452296_at	Region	Slit3	20564	11	34912664	35502537	+
2643	1416008_at	Region	Satb1	20230	17	49274277	49367498	-
2644	1425323_a_at	Region	BC008155	214917	17	23596506	23598387	-
2645	1454745_at	Region	Arhgap29	214137	3	120732219	120795050	+
2646	1418252_at	Region	Padi2	18600	4	139787688	139833847	+
2647	1449038_at	Region	Hsd11b1	15483	1	192960453	193002809	-
2648	1456941_at	Region	Tert	21752	13	69686209	69708153	+
2649	1434834_at	Region	Socs7	192157	11	97183638	97214721	+
2650	1449029_at	Region	Mknk2	17347	10	80797130	80803778	-
2651	1429155_at	Region	4933411K20Rik	66756	8	45112900	45139459	+
2652	1451353_at	Region	Tm6sf1	107769	7	75667263	75692207	+
2653	1436270_at	Region	---	327951	11	69119270	69121291	-
2654	1416940_at	Region	Ppif	105675	14	24069368	24075666	+
2655	1437711_x_at	Region	Odc1 /// LOC545783 /// LOC546355	18263	12	16906701	16912688	+
2656	1454627_a_at	Region	Rpl29 /// LOC433350 /// LOC433383 /// LOC433941 /// LOC545658	19944	9	106423093	106425121	+
2657	1446181_at	Region	C85699	97104	3	72946026	72946451	+
2658	1451275_at	Region	E030041M21Rik	75089	10	89716350	89789724	+
2659	1436934_s_at	Region	Aco2	11429	15	81923222	81965850	+
2660	1436546_at	Region	6330549D23Rik	229613	3	96094498	96117655	-
2661	1415705_at	Region	9130011J15Rik	66818	8	71716686	71721857	-
2662	1435240_at	Region	5830435C13Rik	407823	2	59754763	59767240	-
2663	1433587_at	Region	Rgmb	68799	17	13809790	13830123	-
2664	1455716_at	Region	Epb4.1l1	13821	2	155877989	155997768	+
2665	1430123_a_at	Region	Akr1a4	58810	4	115595415	115610556	-

2666	1447160_at	Region	Nono /// LOC434808	434808	X	75193538	75198479	-
2667	1416286_at	Region	Rgs4	19736	1	169675852	169681797	-
2668	1453000_at	Region	Camsap1l1	67886	1	136115147	136192998	-
2669	1455416_at	Region	C130021I20	329385	2	33573493	33578511	+
2670	1436665_a_at	Region	Ltbp4	108075	7	22679262	22713186	-
2671	1436028_at	Region	Tmem33	67878	5	66026818	66052665	+
2672	1424215_at	Region	Fundc1	72018	X	15795509	15811237	-
2673	1447980_s_at	Region	Rutbc2	52850	5	112319917	112387408	-
2674	1435177_a_at	Region	Anapc5	59008	5	121940636	121974194	-
2675	1439757_s_at	Region	Epha4	13838	1	77656420	77802175	-
2676	1420499_at	Region	Gch1	14528	14	42234216	42269658	-
2677	1436602_x_at	Region	Cacna1b	12287	2	24538537	24695314	-
2678	1434052_at	Region	AI593442	330941	9	52753199	52758226	-
2679	1443369_at	Region	BC052055	328643	16	19362287	19370589	+
2680	1440998_at	Region	2310021P13Rik	268721	14	19076126	19076808	+
2681	1432195_s_at	Region	Ccnl2	56036	4	154304851	154316905	+
2682	1416389_a_at	Region	Chc1l	105670	14	67493623	67535123	+
2683	1434951_at	Region	Armc8	74125	9	99381642	99469875	-
2684	1418581_a_at	Region	Limk2	16886	11	3239044	3303961	-
2685	1423361_at	Region	Yme1l1	27377	2	23088708	23130798	+
2686	1452166_a_at	Region	Krt1-10	16661	11	99206514	99210440	-
2687	1454680_at	Region	D5Ertd579e	320661	5	35088907	35109670	-
2688	1430293_a_at	Region	1300006N24Rik	71766	9	20950505	20975490	-
2689	1448690_at	Region	Kcnk1	16525	8	125282699	125318214	+
2690	1436029_at	Region	4930533K18Rik	75189	10	70976804	70977955	-
2691	1448297_a_at	Region	Tnk2	51789	16	31477206	31491474	+
2692	1420711_a_at	Region	Pxmp3	19302	3	5535229	5550853	-
2693	1443924_at	Region	Prkwnk3	279561	X	144764584	144852704	+
2694	1448610_a_at	Region	Sod2	20656	17	11516794	11524420	-
2695	1423758_at	Region	E430034L04Rik	23881	5	91390328	91419486	-
2696	1449046_a_at	Region	1110007C05Rik	66124	7	38544994	38549086	+
2697	1426642_at	Region	Fn1	14268	1	71881640	71949288	-
2698	1449711_at	Region	Atp6v1e1	11973	6	121227218	121254659	-
2699	1428813_a_at	Region	Drd1ip	68566	7	134468884	134481545	-
2700	1437418_at	Region	None	None	10	92039536	92040577	-
2701	1426987_at	Region	5430417L22Rik	108676	2	118260842	118262660	+
2702	1449522_at	Region	Unc5c	22253	3	140432729	140804799	+
2703	1419380_at	Region	Zfp423	94187	8	86945300	87244946	-
2704	1426463_at	Region	Gphn	268566	12	75199951	75541337	+
2705	1424226_at	Region	9030617O03Rik	217830	12	96224888	96318068	+
2706	1423649_at	Region	2010300G19Rik	72098	4	3476187	3501914	-
2707	1423669_at	Region	Col1a1	12842	11	94757357	94772954	+
2708	1457284_at	Region	None	None	17	54298361	54299272	+
2709	1442270_at	Region	Pbx3	18516	2	34104071	34304198	-
2710	1426407_at	Region	1600010O03Rik	109120	2	90722036	90724247	+
2711	1439881_at	Region	C030013E06Rik	77421	11	102140887	102141344	-
2712	1457182_at	Region	Map2k7	26400	8	3593945	3601130	+
2713	1417536_at	Region	Zmat2	66492	18	37017688	37023425	+
2714	1452128_a_at	Region	MGI:2389572	210766	X	70077355	70114652	+
2715	1423305_at	Region	Extl1	56219	4	133317276	133332682	-
2716	1448189_a_at	Region	Fliih	14248	11	60439791	60452863	-
2717	1418172_at	Region	Hebp1	15199	6	135895149	135929067	-
2718	1433861_at	Region	Cc2d1b	319965	4	107578861	107593027	+
2719	1426248_at	Region	Stk24	223255	14	115857532	115949796	-
2720	1440990_at	Region	4832420M10	269152	1	178819927	178887843	+
2721	1439906_at	Region	None	None	12	35473160	35474153	+
2722	1458112_at	Region	Adarb2	94191	13	8160097	8716266	+
2723	1448968_at	Region	D7Wsu128e	28018	7	115945280	115956990	+
2724	1455328_at	Region	Accn2	11419	15	99727934	99758344	+
2725	1419251_at	Region	Eps15	13858	4	108239208	108344599	+
2726	1436539_at	Region	Clmn	94040	12	100215893	100309212	-
2727	1430052_at	Region	C030048H21Rik	77481	2	26177730	26178870	-
2728	1417901_a_at	Region	Ica1	15893	6	8605506	8727274	-
2729	1421428_at	Region	Slc5a7	63993	17	51909352	51932924	-

2730	1455179_at	Region	Mpp7	75739	18	7422318	7651672	-
2731	1443396_at	Region	Smndc1 /// LOC545792	545792	5	108093108	108094027	-
2732	1429188_at	Region	Cox11	69802	11	90459260	90466023	+
2733	1420533_at	Region	Gucy1a3	60596	3	81821497	81874847	-
2734	1437349_at	Region	Ckap5	75786	2	91296435	91325425	+
2735	1437000_at	Region	Dgkq	110524	5	107723259	107736984	-
2736	1456339_at	Region	2810410D24Rik	69254	2	181580663	181581151	-
2737	1436840_x_at	Region	Rpl35	66489	2	38933743	38937293	-
2738	1423947_at	Region	1110008P14Rik	73737	2	32311270	32314046	-
2739	1418161_at	Region	Jph3	57340	8	121112150	121172408	+
2740	1419951_at	Region	Lman1	70361	18	66213875	66235740	-
2741	1451676_at	Region	Drap1	66556	19	5211601	5213706	-
2742	1448732_at	Region	Ctsb	13030	14	57653056	57676512	+
2743	1421055_at	Region	Lats2 /// Xpo4	50523	14	52225857	52269062	-
2744	1426462_at	Region	Gphn	268566	12	75199951	75541337	+
2745	1437585_x_at	Region	Zfp161	22666	17	67159059	67165625	+
2746	1419976_s_at	Region	Nfatc3	18021	8	105355731	105424292	+
2747	1423726_at	Region	Vat1	26949	11	101279835	101287286	-
2748	1456064_at	Region	AI504432	229694	3	106835333	106846991	+
2749	1452401_at	Region	Wtap	60532	17	11539853	11559714	+
2750	1435019_at	Region	Atxn7l3	217218	11	102112742	102118843	-
2751	1448971_at	Region	2410022L05Rik	66423	8	13263410	13268885	-
2752	1420970_at	Region	Adcy7	11513	8	87557754	87614390	+
2753	1417969_at	Region	Fbxo31	76454	8	120933796	120960646	-
2754	1434718_at	Region	Cul3	26554	1	80599109	80655022	-
2755	1440268_at	Region	Trim41	211007	11	48559241	48570190	-
2756	1427789_s_at	Region	Gnas	14683	2	173709228	173771651	+
2757	1439965_at	Region	None	None	11	75302898	75303115	+
2758	1435850_at	Region	None	None	1	3160079	3160728	-
2759	1417760_at	Region	Nr0b1	11614	X	80852445	80856737	+
2760	1437085_at	Region	D630039A03Rik	242484	4	57851984	57859716	-
2761	1428113_at	Region	4930403J22Rik	70551	14	117484003	117547608	-
2762	1454763_at	Region	Ankrd17	81702	5	89524594	89663613	-
2763	1418144_a_at	Region	Pip5k1b	18720	3	94547426	94594689	-
2764	1436079_s_at	Region	Vapb	56491	2	173198044	173239833	+
2765	1445805_x_at	Region	Kcnh3	16512	15	99282782	99300623	+
2766	1421951_at	Region	Lhx1	16869	11	84247371	84253526	-
2767	1454969_at	Region	E130115E03Rik	320343	2	50060532	50125726	+
2768	1421477_at	Region	Cplx2	12890	13	52985707	52986681	+
2769	1451131_at	Region	Arl6ip1	54208	7	111994757	112005420	-
2770	1427038_at	Region	Penk1	18619	4	4060682	4065591	-
2771	1423855_x_at	Region	Rpl17 /// LOC432963 /// LOC545631	319195	18	75235707	75238565	+
2772	1443120_at	Region	Pdzk4	245469	X	68458430	68459109	-
2773	1427763_a_at	Region	Camk2d	108058	3	125480582	125726370	+
2774	1422519_at	Region	Cask	12361	X	11759987	12085496	-
2775	1417182_at	Region	Dnaja2	56445	8	84810414	84828045	-
2776	1434095_at	Region	Unc5a	107448	13	53558754	53615348	+
2777	1435151_a_at	Region	Rps3	27050	7	93560951	93572082	-
2778	1447830_s_at	Region	Rgs2	19735	1	143832060	143836212	-
2779	1435554_at	Region	Tmcc3	319880	10	94549417	94562436	+
2780	1440721_at	Region	5930433N17Rik	399623	13	113470318	113470862	-
2781	1434199_at	Region	Gm323	210573	17	43052487	43058009	-
2782	1435645_at	Region	Mmd	67468	11	90070563	90099660	+
2783	1420800_a_at	Region	Kcnq2	16536	2	180792537	180852183	-
2784	1423191_at	Region	Fnbp4	55935	2	90450119	90485770	+
2785	1460509_at	Region	B3galt1	26877	2	67973343	67974323	+
2786	1456039_at	Region	Snx14	244962	9	88737448	88799515	-
2787	1427988_s_at	Region	Safb2	224902	17	54251255	54263875	-
2788	1436122_at	Region	A830025F02Rik	213010	7	5498316	5499450	+
2789	1454704_at	Region	Scarb2	12492	5	91779618	91841353	-
2790	1450864_at	Region	Calm3	12315	7	13866351	13874927	-
2791	1429330_at	Region	Gabra4	14397	5	70350950	70437875	-

2792	1453078_at	Region	8430408J07Rik	74520	X	102384111	102386461	-
2793	1424791_a_at	Region	Lu	57278	7	16625383	16639719	-
2794	1435501_at	Region	C030004M05Rik	109357	12	101061010	101061772	-
2795	1456743_x_at	Region	Morf4l2 /// LOC433261	433261	19	584	2363	-
2796	1429401_at	Region	Stox2	71069	8	46138301	46167033	-
2797	1426301_at	Region	Alcam	11658	16	51168811	51372049	-
2798	1424704_at	Region	Runx2	12393	17	42112502	42318792	-
2799	1416243_a_at	Region	Rpl35	66489	2	38933743	38937293	-
2800	1439172_at	Region	Atp13a5	268878	16	28020880	28167802	-
2801	1416304_at	Region	Litaf	56722	16	7206983	7214387	-
2802	1419414_at	Region	Gng13	64337	17	23523224	23525085	+
2803	1435424_x_at	Region	None	None	16	11009158	11009499	+
2804	1434277_a_at	Region	6430570G24	327989	11	86664419	86665560	-
2805	1422889_at	Region	Pcdh18	73173	3	49331829	49346004	-
2806	1425830_a_at	Region	2810452K22Rik	67236	12	106348398	106364332	-
2807	1423767_at	Region	2810410M20Rik	66310	17	72104982	72121482	-
2808	1423045_at	Region	Ncbp2	68092	16	30758010	30767552	+
2809	1453061_at	Region	2610018O07Rik	69201	18	73969713	73971071	-
2810	1446967_at	Region	None	None	15	26707996	26708550	-
2811	1424163_at	Region	0610039K22Rik	66089	11	51376514	51388737	-
2812	1447904_s_at	Region	Fnta	14272	8	24723471	24740350	-
2813	1421112_at	Region	Nkx2-2	18088	2	146640088	146643295	-
2814	1458276_x_at	Region	Cit	12704	5	114963119	115127860	+
2815	1438765_at	Region	Dhx33	216877	11	70709749	70730090	-
2816	1449256_a_at	Region	Rab11a	53869	9	64838434	64859957	-
2817	1434556_at	Region	None	None	13	41199263	41201470	+
2818	1426834_s_at	Region	D930015E06Rik	229473	3	83635417	83777077	-
2819	1455912_x_at	Region	AW538196	101869	7	74125404	74140375	-
2820	1422407_s_at	Region	Hras1	15461	7	135593335	135594927	-
2821	1434260_at	Region	Fchsd2	207278	7	95215371	95390959	+
2822	1452703_at	Region	4631427C17Rik	74340	6	29815630	29959406	+
2823	1448473_at	Region	Bub3	12237	7	125876724	125888172	+
2824	1439064_at	Region	BC030046	277154	14	50372196	50391628	+
2825	1441905_x_at	Region	None	None	7	53758980	53759255	-
2826	1457984_at	Region	Crh	12918	3	19034688	19036603	-
2827	1416699_at	Region	1110008F13Rik	67388	2	156320097	156330528	+
2828	1424871_s_at	Region	1500031H01Rik	207740	16	4545094	4549646	-
2829	1434792_at	Region	2010320M18Rik	72093	8	69932415	69933332	+
2830	1445597_s_at	Region	Hrasls3	225845	19	7271359	7299646	+
2831	1419428_a_at	Region	Gaa	14387	11	119089137	119106545	+
2832	1424674_at	Region	Slc39a6	106957	18	24808377	24830068	-
2833	1419247_at	Region	Rgs2	19735	1	143832060	143836212	-
2834	1443095_at	Region	9630019E01Rik	319967	2	145754156	145754838	-
2835	1442336_at	Region	None	None	15	77878885	77879146	-
2836	1434111_at	Region	Lphn2	99633	3	147794533	147874904	-
2837	1434399_at	Region	Galnt6	207839	15	100749352	100786914	-
2838	1421594_a_at	Region	Sytl2	83671	7	84398260	84460718	+
2839	1416825_at	Region	Snta1	20648	2	153833265	153865018	-
2840	1435564_at	Region	C230078M08Rik	319749	17	24238307	24241147	+
2841	1457840_at	Region	Plxna4	243743	6	32229575	32668174	-
2842	1415956_a_at	Region	Pctk1	18555	X	18927433	18938817	+
2843	1426989_at	Region	Clstn3	232370	6	125043444	125077134	-
2844	1428600_at	Region	3110068G20Rik	73198	12	66843596	66845631	-
2845	1420617_at	Region	Cpeb4	67579	11	31767001	31826367	+
2846	1460252_s_at	Region	Zfp105	22646	9	122940872	122948796	+
2847	1455095_at	Region	Hist2h2be	319190	3	95756526	95756936	+
2848	1431285_at	Region	Mgrn1	17237	16	4555925	4608181	+
2849	1448140_at	Region	Ciapin1	109006	8	94106719	94122472	-
2850	1448554_s_at	Region	Myh6	17888	14	49458413	49483066	-
2851	1451775_s_at	Region	Il13ra1	16164	X	30732693	30791816	+
2852	1439056_at	Region	None	None	12	23694008	23694667	-
2853	1450918_s_at	Region	None	None	2	156914044	156928808	+
2854	1441102_at	Region	AI987712	105935	15	10150000	10151138	+
2855	1434256_s_at	Region	Cds2	110911	2	131777151	131821546	+

2856	1418277_at	Region	Rp9h	55934	9	22338723	22358719	-
2857	1460201_a_at	Region	Rpl24	68193	16	54862320	54867361	+
2858	1433915_s_at	Region	Epn2	13855	11	61242891	61305293	-
2859	1415701_x_at	Region	Rpl23	65019	11	97598630	97603460	-
2860	1433825_at	Region	Ntrk3	18213	7	71981888	72367304	-
2861	1421118_a_at	Region	Gpr56	14766	8	94268775	94298363	+
2862	1443365_at	Region	None	None	18	62695990	62696617	+
2863	1449945_at	Region	Ppargc1b	170826	18	61523462	61625757	-
2864	1421834_at	Region	Pip5k1a	18719	19	23533932	23683715	-
2865	1445475_at	Region	Pak6	214230	2	118177426	118211869	+
2866	1449862_a_at	Region	Pi4k2b	67073	5	51551747	51579476	+
2867	1417929_at	Region	Slc7a8	50934	14	49239052	49298723	-
2868	1454974_at	Region	Ntn1	18208	11	67938664	68111778	-
2869	1421821_at	Region	Ldlr	16835	9	21613867	21637395	+
2870	1444372_at	Region	None	None	NONE	NONE	NONE	NONE
2871	1453135_at	Region	Fndc5	384061	4	128164173	128171706	+
2872	1428775_at	Region	1110008L16Rik	66132	12	52024382	52103104	+
2873	1459238_at	Region	None	None	10	53587679	53588234	-
2874	1439926_at	Region	4632417D23	245843	16	32492548	32530995	+
2875	1415998_at	Region	Vdac1	22333	11	52127140	52142237	+
2876	1416442_at	Region	Ier2	15936	8	83924268	83927565	-
2877	1441020_at	Region	None	None	NONE	NONE	NONE	NONE
2878	1424682_at	Region	D5Ertd708e	68080	5	121524856	121535121	+
2879	1417106_at	Region	Tpd52l2	66314	2	181214210	181233714	+
2880	1455509_at	Region	LOC192950	192950	11	6492611	6500842	-
2881	1444663_at	Region	Vezf1	22344	11	87796271	87812715	+
2882	1438075_at	Region	Fem1c	240263	18	46726362	46747727	-
2883	1452812_at	Region	Lphn1	330814	8	83175087	83200065	+
2884	1423584_at	Region	Igfbp7	29817	5	76593491	76652290	-
2885	1430143_at	Region	None	None	18	21872751	21877294	+
2886	1426751_s_at	Region	Nup107	103468	10	117441635	117483697	-
2887	1460679_at	Region	Exosc4	109075	15	76375587	76378860	+
2888	1415877_at	Region	Dpysl3	22240	18	43544269	43613318	-
2889	1448717_at	Region	Gcdh	270076	8	84153759	84160870	-
2890	1415923_at	Region	Ndn	17984	7	56122085	56123665	+
2891	1423860_at	Region	Ptgds	19215	2	25398874	25401893	-
2892	1433716_x_at	Region	None	None	14	65294915	65295355	+
2893	1427314_at	Region	Tmed7	66676	18	46809246	46819270	-
2894	1423570_at	Region	Abcg1	11307	17	28870974	28929775	+
2895	1417795_at	Region	Chl1	12661	6	103991578	104209457	+
2896	1455022_at	Region	Strn	268980	17	76477464	76558724	-
2897	1436910_at	Region	Rasal2	226525	1	157043874	157318163	-
2898	1417772_at	Region	Grhpr	76238	4	44897333	44906618	+
2899	1438355_at	Region	None	None	12	81546738	81547166	-
2900	1452921_at	Region	B130050I23Rik	213027	8	3521545	3563004	+
2901	1443609_s_at	Region	Syvn1	74126	19	5835452	5842499	+
2902	1434338_at	Region	1110029E03Rik	242406	4	43494851	43503409	+
2903	1449094_at	Region	Gja7	14615	11	102620668	102625730	-
2904	1415948_at	Region	Creg1	433375	1	165685557	165697081	+
2905	1423820_at	Region	Elof1	66126	9	22004130	22005310	-
2906	1439652_at	Region	9930033H14Rik	217351	11	117475908	117583657	+
2907	1448979_at	Region	Muted	17828	13	38143450	38175853	-
2908	1436416_x_at	Region	Fxc1	14356	7	99755452	99756758	+
2909	1441556_at	Region	None	None	2	59977478	59978140	-
2910	1455218_at	Region	6330503K22Rik	101565	7	112588468	112612876	+
2911	1438958_x_at	Region	Fkbp1a	14225	2	150999557	151018627	+
2912	1434091_at	Region	Faah	14073	4	114955573	114976807	-
2913	1423107_at	Region	Ube2b	22210	11	51738403	51753228	-
2914	1434735_at	Region	Hlf	217082	11	90159652	90211982	-
2915	1433965_at	Region	Atp8a1	11980	5	66388740	66614923	-
2916	1423844_s_at	Region	Cbs	12411	17	29420528	29445025	-
2917	1448682_at	Region	Dnclc1	56455	5	114408049	114410560	-
2918	1423349_at	Region	Socs5	56468	17	84964483	84994389	+
2919	1456872_a_at	Region	D230010M03Rik	241547	2	91415666	91420179	+
2920	1450311_at	Region	Slc8a3	110893	12	78060702	78194436	-

2921	1431931_a_at	Region	Rabl2a	68708	15	89635410	89644738	-
2922	1439021_at	Region	Centb5	140500	4	154384237	154399613	+
2923	1416166_a_at	Region	Prdx4	53381	X	148950069	148964603	-
2924	1431326_a_at	Region	Tmod2	50876	9	75710299	75748747	-
2925	1436250_at	Region	5430405G05Rik	108832	2	151159247	151169095	+
2926	1435742_at	Region	1110034C04Rik	68734	12	96484976	96528858	-
2927	1451148_at	Region	Pink1	68943	4	137194673	137207532	-
2928	1423608_at	Region	Itm2a	16431	X	101998560	102004725	-
2929	1456756_at	Region	E430016P22Rik	320578	6	38423603	38424937	-
2930	1436614_at	Region	AI843639	106945	18	56995517	56995932	-
2931	1448743_at	Region	Ssx2ip	99167	3	145381238	145416690	+
2932	1434617_x_at	Region	1810073N04Rik	72055	11	119925064	119972367	-
2933	1447773_x_at	Region	6330409D20Rik	70730	2	32664800	32665040	-
2934	1423082_at	Region	Derl1	67819	15	57878521	57901434	-
2935	1435875_at	Region	Prkab2	108097	3	97153614	97159355	+
2936	1449343_s_at	Region	Sin3a	20466	9	57189858	57241642	+
2937	1417632_at	Region	Atp6v0a1	11975	11	100830569	100884801	+
2938	1417710_at	Region	0610012D09Rik	59052	7	114910012	114952402	+
2939	1434211_at	Region	Sh3bgrl2	212531	9	83878025	83930061	+
2940	1435862_at	Region	Son	20658	16	90805044	90836353	+
2941	1460732_a_at	Region	Ppl	19041	16	4756515	4802113	-
2942	1455001_x_at	Region	Rpl13a	22121	7	39202318	39205277	-
2943	1445543_at	Region	9830124H08Rik	219105	14	51315574	51334091	-
2944	1418459_at	Region	1810060J02Rik	67015	6	148405067	148561800	+
2945	1418168_at	Region	Zcchc14	142682	8	120981449	121033085	-
2946	1437675_at	Region	Slc8a1	20541	17	79218202	79483338	-
2947	1451573_a_at	Region	Stx4a	20909	7	121891618	121898789	+
2948	1423945_a_at	Region	Pkig	18769	2	163150999	163183121	+
2949	1435060_at	Region	Tmod2	50876	9	75710299	75748747	-
2950	1416219_at	Region	Rpl19	19921	11	97848073	97851564	+
2951	1428622_at	Region	Depdc6	97998	15	55114270	55254734	+
2952	1449979_a_at	Region	Spock3	72902	8	62017573	62420359	+
2953	1434788_at	Region	D930050A07Rik	320969	14	59719321	59720106	-
2954	1443528_at	Region	C330008L01Rik	106763	17	43951697	43953616	-
2955	1435325_at	Region	Usp46	69727	5	72834085	72900782	-
2956	1450730_at	Region	Hs2st1	23908	3	143402785	143541813	-
2957	1417635_at	Region	Spa17	20686	9	37544795	37555111	-
2958	1428162_at	Region	4933421E11Rik	321000	3	106517510	106530210	+
2959	1419159_at	Region	Golga3	269682	5	109227485	109273939	+
2960	1428275_at	Region	1110065L07Rik	68904	8	9345221	9357725	+
2961	1429194_at	Region	Tigd2	68140	6	59454555	59457413	+
2962	1421955_a_at	Region	Nedd4	17999	9	72794997	72882331	+
2963	1452453_a_at	Region	Camk2a	12322	18	61150958	61213470	+
2964	1455300_at	Region	E130014J05Rik	214133	3	132356252	132362213	-
2965	1455515_at	Region	1810041L15Rik	72301	15	84428627	84495696	-
2966	1443410_at	Region	Grm5	108071	7	81779037	82108756	+
2967	1440969_at	Region	BC030308	407797	2	78625033	78626542	+
2968	1425143_a_at	Region	Ndufs1	227197	1	63436616	63469727	-
2969	1419054_a_at	Region	Ptpn21	24000	12	94103584	94161513	-
2970	1454900_s_at	Region	Phr1	105689	14	97658865	97891435	-
2971	1420484_a_at	Region	Vtn	22370	11	78224945	78227983	+
2972	1439753_x_at	Region	Six4	20474	12	69946438	69959424	-
2973	1416109_at	Region	Fuca1	71665	4	134802014	134821570	+
2974	1460310_a_at	Region	Gh	14599	11	106121357	106122899	-
2975	1436718_at	Region	Nxph1	18231	6	8916972	9216425	+
2976	1430388_a_at	Region	Sulf2	72043	2	165530889	165612339	-
2977	1423508_at	Region	Myst4	54169	14	19864536	20036801	+
2978	1449192_at	Region	Atf7ip	54343	6	137280522	137369083	+
2979	1423270_at	Region	Nedd4l	83814	18	65256461	65446324	+
2980	1456125_a_at	Region	Dnclc1	56455	5	114408049	114410560	-
2981	1428408_a_at	Region	D12Ertd551e	52635	12	112044347	112136958	+
2982	1416046_a_at	Region	Fuca2	66848	10	13382818	13399065	+
2983	1455485_x_at	Region	Rpl13a	22121	7	39202318	39205277	-
2984	1435392_at	Region	Wdr17	244484	8	53596923	53685958	-
2985	1442062_at	Region	7120426M23Rik	319898	18	76543653	76545947	+

2986	1445479_at	Region	None	None	17	43963417	43964026	-
2987	1426998_at	Region	Tex27	21769	17	27815127	28020060	+
2988	1440358_at	Region	Arhgef15	442801	11	68673890	68677832	-
2989	1459563_x_at	Region	None	None	NONE	NONE	NONE	NONE
2990	1418450_at	Region	Islr	26968	9	58270295	58273168	-
2991	1433933_s_at	Region	Slco2b1	101488	7	93746839	93800194	-
2992	1425405_a_at	Region	Adar	56417	3	89536967	89557589	+
2993	1419651_at	Region	2610200G18Rik	67149	NONE	NONE	NONE	NONE
2994	1450992_a_at	Region	Meis1	17268	11	18775221	18913473	-
2995	1451913_a_at	Region	Hyou1	12282	9	44368440	44381225	+
2996	1429526_at	Region	Brd8	78656	18	34822848	34847583	-
2997	1426111_x_at	Region	Irf3	54131	7	39074202	39079390	+
2998	1437461_s_at	Region	Rnpc3	67225	3	14547	39217	+
2999	1437147_at	Region	Gabrg2	14406	11	41664694	41753515	-
3000	1419155_a_at	Region	Sox4	20677	13	28430338	28433304	-
3001	1436137_at	Region	Slc6a17	229706	3	107263377	107313847	-
3002	1435337_at	Region	Zfp537	243931	7	31861436	31936775	+
3003	1458161_at	Region	Kcnq1ot1	63830	7	137694289	137694834	-
3004	1427107_at	Region	Slc16a11	216867	11	69939738	69942071	+
3005	1423603_at	Region	Zfpm1	22761	8	121663726	121718834	+
3006	1429265_a_at	Region	Rnf130	59044	11	49778172	49857576	+
3007	1416902_a_at	Region	Cox5b	12859	1	36986129	36987976	+
3008	1424646_at	Region	Uckl1	68556	2	181286109	181298930	-
3009	1424122_s_at	Region	Commd1	17846	11	22794554	22873786	-
3010	1460511_at	Region	Pkp2	67451	16	14981080	15040447	+
3011	1436034_at	Region	Rab1	19324	11	20096395	20121649	+
3012	1416086_at	Region	Tpst2	22022	5	111346715	111385345	+
3013	1439413_x_at	Region	Morf4l2	56397	X	130277881	130286548	-
3014	1423831_at	Region	Prkag2	108099	5	23326651	23569979	-
3015	1425104_at	Region	Kctd1	106931	18	15156946	15339485	-
3016	1450269_a_at	Region	Pfkl	18641	10	78096466	78119117	-
3017	1451073_at	Region	Sppl3	74585	5	114120890	114208417	+
3018	1426318_at	Region	Serpinb1b	282663	13	32563792	32573934	+
3019	1438006_at	Region	4933439F18Rik	66771	11	60142945	60170919	+
3020	1436348_at	Region	None	None	8	124192769	124193828	+
3021	1434794_at	Region	Rhof	23912	5	122256437	122285538	-
3022	1439805_at	Region	Nfat5	54446	8	106591549	106677210	+
3023	1448942_at	Region	Gng11	66066	6	3956635	3961093	+
3024	1450264_a_at	Region	Chka	12660	19	3640704	3683157	+
3025	1450798_at	Region	Tnxb	81877	17	32373858	32423088	+
3026	1424544_at	Region	Nrbp2	223649	15	76134266	76138685	-
3027	1433528_at	Region	Gtf2a2	235459	9	70143470	70153708	+
3028	1435089_at	Region	2010111I01Rik	72061	13	60400470	60681842	+
3029	1423074_at	Region	Lman2	66890	13	53952542	53972056	-
3030	1427981_a_at	Region	Csad	246277	15	102236358	102248403	-
3031	1424470_a_at	Region	Rapgef3	223864	15	97820598	97843317	-
3032	1455472_at	Region	A630071D13Rik	103300	NONE	NONE	NONE	NONE
3033	1421202_at	Region	Chrna4	11438	2	180739269	180756135	-
3034	1453422_a_at	Region	1110020G09Rik	68646	15	8873106	8912017	+
3035	1416462_at	Region	Gpiap1	53872	2	103469773	103502351	-
3036	1448162_at	Region	Vcam1	22329	3	114880536	114900204	-
3037	1449127_at	Region	Selpl	20345	5	112929691	112941077	-
3038	1448112_at	Region	Cox7c	12867	13	82151364	82153299	-
3039	1455273_at	Region	Zfp482	241322	2	37359545	37375333	-
3040	1422613_a_at	Region	Rpl7a	27176	9	26842969	26845473	+
3041	1428516_a_at	Region	Spata11	66400	17	54687275	54689244	+
3042	1427020_at	Region	Scara3	219151	14	60446815	60481164	-
3043	1426966_at	Region	Axin1	12005	17	23931904	23988981	+
3044	1445517_at	Region	Jmy	57748	13	89619364	89683698	-
3045	1440429_at	Region	MGI:107562	74100	9	112222231	112229149	-
3046	1435281_at	Region	Cpt1c	78070	7	39035915	39051316	-
3047	1429461_at	Region	2810417D08Rik	70422	11	85940080	85985336	-
3048	1426663_s_at	Region	Pcanap6	212980	1	131822427	131834782	+
3049	1450840_a_at	Region	Rpl39	67248	X	31703074	31705738	-
3050	1458263_at	Region	Cugbp2	14007	2	6459141	6638885	-

3051	1435880_at	Region	E430012K20Rik	99696	3	37921264	37950389	-
3052	1452193_a_at	Region	Wasl	73178	6	24663290	24714456	-
3053	1440120_at	Region	Gnb2-rs1	14694	11	48553197	48559078	+
3054	1458524_at	Region	Fndc3a	319448	14	66891442	67062343	-
3055	1455699_at	Region	None	None	6	145840270	145841280	-
3056	1436395_at	Region	Card6	239319	15	4883018	4894105	-
3057	1451120_at	Region	Rpo1-3	20018	5	145969234	146003052	+
3058	1455363_at	Region	Bai1	107831	15	74548346	74620885	+
3059	1439940_at	Region	2900019G14Rik	72932	2	102488249	102488895	+
3060	1417807_at	Region	2700038N03Rik	70240	5	136245535	136246530	+
3061	1449020_at	Region	Plscr3	70310	11	69572334	69577716	+
3062	1451983_at	Region	Irx1	16371	13	68009760	68014632	-
3063	1429198_at	Region	1810030O07Rik	69155	X	10893824	10912486	-
3064	1454716_x_at	Region	Cox5b	12859	1	36986129	36987976	+
3065	1441403_at	Region	Ttll4	67534	1	74978679	74979358	+
3066	1435881_at	Region	Pcbp2	18521	15	102531206	102559300	+
3067	1429515_at	Region	Ubr2	224826	17	44441356	44523334	-
3068	1438047_at	Region	C130073D16Rik	269800	6	125665749	125688791	+
3069	1426524_at	Region	Gnpda2	67980	5	68341941	68359622	-
3070	1426558_x_at	Region	3100002L24Rik	66376	2	175129264	175144107	-
3071	1428387_at	Region	Acsl3	74205	1	78992139	79040786	+
3072	1450150_a_at	Region	Rpl13	270106	8	122483697	122486521	+
3073	1457477_at	Region	Mbnl2	105559	14	114846670	115003029	+
3074	1460735_at	Region	Svil	225115	18	4925311	5124034	+
3075	1443086_at	Region	Alcam	11658	16	51168811	51372049	-
3076	1428935_at	Region	Canx	12330	11	50047308	50078432	-
3077	1437679_a_at	Region	Glrx2	69367	1	143571711	143578676	+
3078	1422169_a_at	Region	Bdnf	12064	2	109298487	109350793	+
3079	1448206_at	Region	Psma2	19166	13	14057818	14070140	+
3080	1448318_at	Region	Adfp	11520	4	85642758	85656186	-
3081	1448678_at	Region	3110048E14Rik	73225	15	85085724	85111429	+
3082	1421435_at	Region	Grid2	14804	6	64146694	64904112	+
3083	1429900_at	Region	5330406M23Rik	76671	6	31148801	31150468	-
3084	1423197_a_at	Region	AW011752	104570	11	29067786	29113926	+
3085	1428344_at	Region	4932443D16Rik	74411	19	28216517	28219402	+
3086	1433435_at	Region	AW551984	244810	9	39530212	39546940	-
3087	1425898_x_at	Region	Olfm3	229759	3	113850919	113895474	+
3088	1419445_s_at	Region	Sap18	20220	14	52333126	52339849	+
3089	1436140_at	Region	1700027J05Rik	69440	15	89237134	89248812	-
3090	1449010_at	Region	Hspa4l	18415	3	40165310	40210062	+
3091	1455313_at	Region	Ablim2	231148	5	34245478	34371978	+
3092	1455122_at	Region	Ptchd2 /// LOC545719	242748	4	146732885	146735266	-
3093	1431059_x_at	Region	Htatsf1	72459	X	51808329	51821897	+
3094	1416481_s_at	Region	MGI:1930666	56295	9	121862188	121871251	-
3095	1451902_at	Region	BC021442	224598	17	20282140	20297899	+
3096	1442177_at	Region	Slc6a5	104245	7	44024879	44077972	+
3097	1422616_s_at	Region	Wdr54	75659	6	83496437	83500056	-
3098	1422852_at	Region	Cib2	56506	9	54657936	54673669	-
3099	1437787_at	Region	None	None	18	35432995	35434650	-
3100	1455701_at	Region	Snx26	233071	7	25934918	25947701	-
3101	1431057_a_at	Region	Prss23	76453	7	83485290	83495057	-
3102	1438566_at	Region	None	None	2	13567269	13568387	-
3103	1418773_at	Region	Fads3	60527	19	9238187	9256168	+
3104	1452414_s_at	Region	D19Ertd678e	108673	19	10139228	10145763	-
3105	1439732_at	Region	None	None	15	4363268	4363829	+
3106	1446618_at	Region	LOC432971	432971	15	85728839	85729479	+
3107	1421847_at	Region	Wsb2	59043	5	116486085	116507368	+
3108	1452619_a_at	Region	Agbl3	76223	6	34875137	34899893	+
3109	1419753_at	Region	Nfx1	74164	4	41109832	41164029	+
3110	1425149_a_at	Region	Pdcl	67466	2	37282236	37291394	-
3111	1448029_at	Region	Tbx3	21386	5	118821012	118834673	+
3112	1434075_at	Region	BC030336	233812	7	114553247	114606821	+
3113	1415910_s_at	Region	Ciapin1	109006	8	94106719	94122472	-
3114	1427192_a_at	Region	Brd8	78656	18	34822848	34847583	-

3115	1428585_at	Region	Actn1	109711	12	77028440	77032905	-
3116	1453222_at	Region	Gse1	382034	8	119846048	119938723	+
3117	1455646_at	Region	2010004M13Rik	76511	7	30902316	30902970	+
3118	1452021_a_at	Region	Hes6	55927	1	91234525	91236264	-
3119	1442138_at	Region	Gpr62	436090	9	106528967	106532763	-
3120	1434468_at	Region	D8Ertd69e	73945	8	78854651	78892469	+
3121	1424634_at	Region	Tceal1	237052	X	130253004	130254807	+
3122	1450985_a_at	Region	Tjp2	21873	19	23332679	23411932	-
3123	1456220_at	Region	Fbxl7	448987	15	26529709	26530364	-
3124	1427243_at	Region	AA536743	100532	5	62707529	62766865	-
3125	1443989_at	Region	Trim9	94090	12	67079894	67179488	-
3126	1424930_s_at	Region	MGC27770	213956	15	80724488	80753043	+
3127	1441531_at	Region	Plcb4	18798	2	135319401	135526921	+
3128	1452245_at	Region	G630039L02	268543	12	43459997	43567383	-
3129	1424467_at	Region	Phldb1	102693	9	44675163	44724049	-
3130	1436569_at	Region	C030045D06Rik	109294	1	11407410	11409217	+
3131	1452338_s_at	Region	Itsn1	16443	16	90939437	91069253	+
3132	1443128_at	Region	Ddx26	18130	14	57209161	57294009	-
3133	1440619_at	Region	Mapk8ip1	19099	2	92088363	92105946	-
3134	1431137_at	Region	Rusc1	72296	3	88827845	88837154	-
3135	1432850_at	Region	5430434G16Rik	71385	15	36252623	36253976	-
3136	1433970_at	Region	Bola3	78653	6	83693715	83702623	+
3137	1426604_at	Region	Rnasel	24014	1	153637567	153646977	+
3138	1438322_x_at	Region	Fdft1	14137	14	57675741	57708384	-
3139	1420048_at	Region	C78859	97455	8	93611773	93612171	+
3140	1435175_at	Region	BC034090	207792	1	155103210	155123477	-
3141	1416435_at	Region	Ltbr	17000	6	125963256	125970534	-
3142	1426399_at	Region	Vwa1	246228	4	154260857	154266876	-
3143	1419829_a_at	Region	None	None	7	91353590	91354371	+
3144	1451804_a_at	Region	Lrrc16	68732	13	23492536	23760672	-
3145	1434967_at	Region	Zswim6	67263	13	103950578	104158812	-
3146	1453478_at	Region	Pou3f2	18992	4	22556165	22557502	-
3147	1424828_a_at	Region	Fh1	14194	1	175534087	175558287	-
3148	1415716_a_at	Region	Rps27	57294	3	90023087	90024065	-
3149	1440206_at	Region	A930024E05Rik	109202	5	122142263	122151248	+
3150	1445459_at	Region	None	None	17	23295726	23296342	-
3151	1433875_at	Region	4732418C07Rik	230648	4	114696978	114736232	+
3152	1420475_at	Region	Mtpn	14489	6	35603109	35634015	-
3153	1454734_at	Region	Lef1	16842	3	130040399	130153310	+
3154	1456523_at	Region	None	None	7	78431257	78432189	+
3155	1453479_at	Region	1700086P04Rik	67349	4	128077201	128085088	-
3156	1419452_at	Region	Uchl5	56207	1	143609398	143638878	+
3157	1452583_s_at	Region	Galm	319625	17	77944865	78002426	+
3158	1427186_a_at	Region	Mef2a	17258	7	61121784	61125517	-
3159	1418132_a_at	Region	D7Wsu128e	28018	7	115945280	115956990	+
3160	1426997_at	Region	Thra	21833	11	98562960	98586198	+
3161	1460196_at	Region	Cbr1	12408	16	92774230	92776713	+
3162	1428351_at	Region	Ppm1m	67905	9	106188548	106192870	-
3163	1435443_at	Region	Eya3	14050	4	131599945	131685662	+
3164	1419045_at	Region	Slc25a23	66972	17	54733419	54749804	-
3165	1459665_s_at	Region	Mrvi1	17540	7	104721209	104835598	-
3166	1421449_at	Region	Csmd1	94109	8	15273983	16089202	-
3167	1454874_at	Region	Btbd7	238386	12	98231027	98324543	-
3168	1416082_at	Region	Rab1	19324	11	20096395	20121649	+
3169	1423884_at	Region	Tex292	21771	8	106196204	106220781	+
3170	1445642_at	Region	Lemd1	213409	1	132045455	132110831	+
3171	1453299_a_at	Region	Pnp /// LOC545044	18950	14	46039165	46048052	+
3172	1439163_at	Region	None	None	9	48682062	48682773	-
3173	1431131_s_at	Region	A630007B06Rik	213993	19	56376334	56399260	-
3174	1443620_at	Region	Gpc4	14735	X	46573716	46686048	-
3175	1439316_at	Region	Rnpc2	170791	2	155604575	155637077	-
3176	1416521_at	Region	Sepw1	20364	7	4226	7136	-
3177	1450442_at	Region	Add2	11519	6	86507734	86549220	+
3178	1422457_s_at	Region	Sumo3	20610	10	77717706	77728812	+

3179	1452140_at	Region	Tbc1d20	67231	2	151750808	151769526	+
3180	1459749_s_at	Region	Fat4	329628	3	38357245	38481982	+
3181	1455624_at	Region	None	None	3	87972647	87973824	+
3182	1456078_x_at	Region	4930542G03Rik /// LOC433839	227613	2	25154324	25156803	-
3183	1424191_a_at	Region	Tmem41a	66664	16	20705838	20717737	-
3184	1429425_at	Region	Rnf139	75841	15	58900098	58911654	+
3185	1449189_at	Region	BC003266	80284	4	126270938	126274922	+
3186	1433023_at	Region	2310068G24Rik	70185	12	78104714	78106328	-
3187	1421126_at	Region	Ryr2	20191	13	11561136	11985749	-
3188	1428923_at	Region	Ppp1r3g	76487	13	35482984	35505121	+
3189	1416415_a_at	Region	H2afz	51788	3	136753528	136755851	+
3190	1455073_at	Region	Cdadc1	71891	14	54094451	54133002	-
3191	1435778_at	Region	Ankrd11	77087	8	122264618	122423554	-
3192	1421961_a_at	Region	Dnajb5	56323	4	42869016	42874654	+
3193	1424561_at	Region	Ece2	107522	16	19402051	19418060	+
3194	1440570_at	Region	6030443J06Rik	320719	5	21269408	21270120	+
3195	1428182_at	Region	Prpsap1	67763	11	116291939	116320020	-
3196	1426378_at	Region	Eif4b	75705	15	102141346	102156533	+
3197	1449576_at	Region	Eif1ay	66235	X	152971635	152987226	+
3198	1447783_x_at	Region	D11Ertd333e	68066	11	102224234	102228559	-
3199	1434379_at	Region	Mxd4	17122	5	32664918	32676048	-
3200	1442707_at	Region	Camk2a	12322	18	61150958	61213470	+
3201	1454637_at	Region	Klhl8	246293	5	102896841	102946020	-
3202	1428101_at	Region	Rnf38	73469	4	44044581	44074976	-
3203	1452216_at	Region	BC025519	223722	15	83595396	83604296	-
3204	1427057_at	Region	C630002B14Rik	103466	10	86809415	86813050	+
3205	1440439_at	Region	None	None	6	53156170	53156818	-
3206	1431114_at	Region	Dock4	238130	12	37387382	37389875	+
3207	1460538_at	Region	Cdh10	320873	15	18747204	18942493	+
3208	1425491_at	Region	Bmpr1a	12166	14	32547251	32638735	-
3209	1441371_at	Region	Plxna4	243743	6	32229575	32668174	-
3210	1440148_at	Region	Gpr6	140741	10	41178103	41179194	-
3211	1444151_at	Region	2600009P04Rik	66572	9	21938933	21939642	-
3212	1456705_at	Region	Zfp532	328977	18	65813456	65922159	+
3213	1437192_x_at	Region	Vdac1	22333	11	52127140	52142237	+
3214	1416393_at	Region	Grcc2f	14791	6	125359657	125367507	-
3215	1453569_s_at	Region	Mark4	232944	7	16295319	16327738	-
3216	1420838_at	Region	Ntrk2	18212	13	57448390	57572946	+
3217	1426819_at	Region	Hipk3	15259	2	104134518	104198998	-
3218	1429850_x_at	Region	2010004B12Rik	72041	5	135149640	135155104	+
3219	1435941_at	Region	Rhbdl4	246104	11	80026735	80081614	+
3220	1450828_at	Region	Synpo2	118449	3	121904047	121951604	-
3221	1439018_at	Region	6330505N24Rik	229474	3	84187486	84222923	-
3222	1421936_at	Region	Dapp1	26377	3	136820534	136870453	-
3223	1451571_s_at	Region	9130019O22Rik /// E430018J23Rik /// 8030466O12Rik /// 6430604K15Rik	101604	7	121433485	121443350	-
3224	1441729_at	Region	None	None	2	33493127	33493634	-
3225	1458125_at	Region	C80913	19777	7	33124913	33183548	-
3226	1437062_s_at	Region	Phyhipl	70911	10	70611653	70653244	-
3227	1416810_at	Region	Mea1	17256	17	44191851	44193708	+
3228	1425134_a_at	Region	Pigx	72084	16	30893795	30909102	-
3229	1439990_at	Region	None	None	4	18051182	18052354	+
3230	1435465_at	Region	4930465M17Rik	74901	8	14398719	14411815	+
3231	1439872_at	Region	None	None	3	106904004	106904655	+
3232	1434686_at	Region	None	None	3	54351100	54362983	+
3233	1436124_at	Region	Pcyt1b	236899	X	88315521	88410606	+
3234	1448476_at	Region	Nap1l4	17955	7	137926598	137962109	-
3235	1456396_at	Region	None	None	14	52112615	52114074	-
3236	1438906_at	Region	Tlk1	228012	2	70410150	70523223	-
3237	1442887_at	Region	AI853363	98471	1	125702831	125703636	-
3238	1443522_s_at	Region	Phip	83946	9	83198955	83305205	-
3239	1456044_at	Region	Nedd4l	83814	18	65256461	65446324	+

3240	1453120_at	Region	D2Bwg1356e	52837	2	134108354	134157974	-
3241	1427670_a_at	Region	Tcf12	21406	9	71980605	72246476	-
3242	1454979_at	Region	Diap1	13367	18	38068549	38159133	-
3243	1452979_at	Region	2610110G12Rik	73242	17	33619005	33629198	-
3244	1418401_a_at	Region	Dusp16	70686	6	135472936	135549943	-
3245	1454743_at	Region	Nup205	70699	6	35271467	35341643	+
3246	1415732_at	Region	Bat5	193742	17	32793071	32806767	+
3247	1433523_at	Region	D930005D10Rik	231858	5	141492144	141552261	-
3248	1422650_a_at	Region	Riok3	66878	18	12316360	12344298	+
3249	1435248_a_at	Region	Btaf1	107182	19	36299109	36355422	+
3250	1426993_at	Region	Xpr1	19775	1	155171522	155308066	-
3251	1451666_at	Region	Acly	104112	11	100297443	100348988	-
3252	1429987_at	Region	9930013L23Rik	80982	7	77789128	77940416	-
3253	1448414_at	Region	Rad1	19355	15	10300285	10307801	+
3254	1438799_at	Region	MGI:2443217	320038	6	6792946	6834425	-
3255	1423895_a_at	Region	Cugbp2	14007	2	6459141	6638885	-
3256	1454987_a_at	Region	H2-Ke6	14979	17	31730089	31732207	-
3257	1450186_s_at	Region	Gnas	14683	2	173709228	173771651	+
3258	1442072_at	Region	C230081A13Rik	244895	9	56336855	56527185	-
3259	1440374_at	Region	Pde1c	18575	6	56226106	56506844	-
3260	1427943_at	Region	Acyp2	75572	11	30400782	30544186	-
3261	1443843_x_at	Region	Rpl9	20005	5	64190194	64192821	-
3262	1415678_at	Region	Ppm1a	19042	12	69630561	69641441	+
3263	1423418_at	Region	Fdps	110196	3	88837452	88845809	-
3264	1439298_at	Region	None	None	X	36604948	36605564	+
3265	1441558_at	Region	D230044B12Rik	399635	15	80885229	80885833	+
3266	1422327_s_at	Region	G6pd2 /// G6pdx	14380	5	60615551	60617092	+
3267	1459830_at	Region	None	None	7	23703122	23703299	-
3268	1431145_a_at	Region	Cuedc2	67116	19	45877886	45885966	-
3269	1418901_at	Region	Cebpb	12608	2	167197648	167199151	+
3270	1415845_at	Region	Syt4	20983	18	31677574	31687445	-
3271	1429266_at	Region	Tmhs	328789	17	26377207	26385076	+
3272	1435221_at	Region	Foxp1	108655	6	99395348	99630926	-
3273	1454074_a_at	Region	1500011J06Rik	208606	5	122899619	122920601	-
3274	1417999_at	Region	Itm2b	16432	14	67713666	67736706	-
3275	1434021_at	Region	C230096C10Rik	230866	4	138233859	138259973	+
3276	1418687_at	Region	Arc	11838	15	74700745	74704214	-
3277	1451893_s_at	Region	Magi1	14924	6	94131999	94737301	-
3278	1423763_x_at	Region	Rps28	54127	17	31521434	31522877	-
3279	1436200_at	Region	Rnf127	74365	X	30986683	30987411	+
3280	1436705_at	Region	Tmem32	236792	X	51340226	51352633	-
3281	1423976_at	Region	4930453N24Rik	67609	16	63789732	63795010	-
3282	1444250_at	Region	None	None	3	30240843	30241987	+
3283	1423843_at	Region	BC027309	243371	6	48687831	48703601	+
3284	1424912_at	Region	Slc25a17	20524	15	81369721	81411565	-
3285	1441166_at	Region	A330050F15Rik	320722	17	67214915	67262703	+
3286	1450889_at	Region	Smarca3	20585	3	19399088	19457883	+
3287	1429328_at	Region	Nsfl1c	386649	2	150951244	150968241	+
3288	1438049_at	Region	A430108E01Rik	384382		70218046	70219091	+
3289	1440263_at	Region	Alkbh	211064	12	84553546	84554547	+
3290	1426796_at	Region	Txln	109658	4	128653196	128667753	-
3291	1433472_x_at	Region	Rpl38	67671	11	114489617	114493424	+
3292	1451695_a_at	Region	Gpx4	14779	10	46036806	46037580	+
3293	1416786_at	Region	Acvr1	11477	2	58371977	58441698	-
3294	1435893_at	Region	Vldlr	22359	19	26457498	26489686	+
3295	1455860_at	Region	Pigh	110417	12	75938752	75946053	-
3296	1449334_at	Region	Timp3	21859	10	86261055	86310024	+
3297	1438456_at	Region	H13	14950	2	152126435	152163760	+
3298	1448884_at	Region	Gtf2e2	68153	8	32537643	32582765	+
3299	1423870_at	Region	2610208M17Rik	108909	8	3442132	3444816	+
3300	1450646_at	Region	None	None	5	4086152	4110752	-
3301	1418382_at	Region	Apcdd1	494504	18	63040206	63182348	+
3302	1451345_at	Region	Mtap	66902	4	88123618	88167275	+
3303	1455018_at	Region	Lmtk2	231876	5	143211341	143229817	+
3304	1426565_at	Region	Igf1r	16001	7	61836803	62111098	+

3305	1435023_at	Region	Sh3d1B	20403	12	3775688	3876446	+
3306	1449283_a_at	Region	Mapk12	29857	15	89182510	89192715	-
3307	1436507_at	Region	Irak2	108960	6	114206414	114263340	+
3308	1416491_at	Region	Numbl	18223	7	22631965	22655228	+
3309	1415813_at	Region	Api5 /// LOC329706	11800	2	94116415	94142756	-
3310	1425956_a_at	Region	Cdadc1	71891	14	54094451	54133002	-
3311	1431094_at	Region	1110006E14Rik	76286	12	105072403	105074202	+
3312	1452905_at	Region	Gtl2 /// Lphn1	17263	12	105023823	105039917	+
3313	1452911_at	Region	Spred1	114715	2	116635298	116693185	+
3314	1427771_x_at	Region	Itgb1	16412	8	3634	31325	+
3315	1422631_at	Region	Ahr	11622	12	32111362	32147826	-
3316	1426265_x_at	Region	Dlat	235339	9	50706961	50732106	-
3317	1419248_at	Region	Rgs2	19735	1	143832060	143836212	-
3318	1444199_at	Region	None	None	1	131875684	131876289	-
3319	1434023_at	Region	AU016693	225523	18	53927584	53963545	-
3320	1453069_at	Region	Pik3cb	74769	9	98937760	99039445	-
3321	1416894_at	Region	1500019G21Rik	66245	7	3905141	3929660	-
3322	1448858_at	Region	Ulk2	29869	11	61501302	61580259	-
3323	1435049_s_at	Region	AI854703	243373	6	48760814	48766567	+
3324	1448635_at	Region	Smc2l1	14211	4	52356135	52402534	+
3325	1460300_a_at	Region	Ltk	17005	2	119265175	119274280	-
3326	1424826_s_at	Region	Mtss1	211401	15	58954404	59092928	-
3327	1457913_at	Region	5730601F06Rik	77519	9	20365696	20393979	-
3328	1424012_at	Region	4930506L13Rik	78802	2	75675996	75679518	-
3329	1418850_at	Region	Epc1	13831	18	6478658	6558794	-
3330	1425542_a_at	Region	Ppp2r5c	26931	12	105961123	106055786	+
3331	1450416_at	Region	Cbx5	12419	15	103258024	103277308	-
3332	1426349_s_at	Region	Tmpo	21917	10	91133084	91142861	-
3333	1451015_at	Region	Tkt	21881	14	28683743	28709316	+
3334	1448101_s_at	Region	Trim27	19720	13	20660436	20672921	+
3335	1423450_a_at	Region	Hs3st1	15476	5	38365401	38506941	-
3336	1426427_at	Region	Ttll1	319953	15	83532371	83559476	-
3337	1434554_at	Region	Trim37	68729	11	86855069	86948674	+
3338	1442542_at	Region	B130023L16Rik	319558	10	23081959	23082634	-
3339	1452034_at	Region	Prepl	213760	17	82893334	82920056	-
3340	1435419_at	Region	Rgag4	331474	X	96667912	96672557	-
3341	1456664_x_at	Region	Hnrpf /// LOC544823	544823	11	120926636	120928218	-
3342	1425822_a_at	Region	Dtx1	14357	5	119832010	119847210	-
3343	1448251_at	Region	9030425E11Rik	71566	9	40636751	40734839	+
3344	1423642_at	Region	4930542G03Rik	227613	2	25154324	25156803	-
3345	1422771_at	Region	Smad6	17130	9	64074118	64142302	-
3346	1417567_at	Region	Ctnnbip1	67087	4	148010605	148059087	+
3347	1434649_at	Region	Ccm2	216527	11	6441744	6491532	+
3348	1450058_at	Region	Asph	65973	4	9376231	9595958	-
3349	1452125_at	Region	Thrap3	230753	4	125199956	125213701	-
3350	1417474_at	Region	1500035H01Rik	76568	9	44761864	44781569	+
3351	1450001_a_at	Region	Ush1c	72088	7	40280705	40323838	-
3352	1454824_s_at	Region	Mtus1	102103	8	39926813	40021115	-
3353	1434776_at	Region	Sema5a	20356	15	32419980	32698954	+
3354	1428748_at	Region	5830428H23Rik	78823	NONE	NONE	NONE	NONE
3355	1435285_at	Region	2700082O15Rik	77015	2	106432845	106487143	+
3356	1427101_at	Region	Metrn	70083	17	23600571	23603045	-
3357	1421375_a_at	Region	S100a6	20200	3	90421654	90422884	+
3358	1427341_at	Region	E130103I17Rik	319845	9	22366102	22779125	+
3359	1433519_at	Region	Nucks1	98415	1	131762351	131784790	+
3360	1454678_s_at	Region	A130022J15Rik	101351	6	97577445	97615221	-
3361	1428465_at	Region	5033425B17Rik	69804	7	26136939	26138720	-
3362	1426750_at	Region	Flnb	286940	14	5564611	5619816	+
3363	1440412_at	Region	Igsf4d	239857	16	65672212	65964483	-
3364	1450877_at	Region	BC005537	79555	13	24281534	24292776	+
3365	1428890_at	Region	Fem1c	240263	18	46726362	46747727	-
3366	1437402_x_at	Region	Tmem41b	233724	7	103825350	103839101	-
3367	1448624_at	Region	Cd2bp2	70233	7	121241391	121245731	-

3368	1434298_at	Region	Zfhx1b	24136	2	44919283	45045178	-
3369	1449687_at	Region	D10Ertd610e	52666	10	126915321	126923060	-
3370	1428541_at	Region	3321401G04Rik	77574	6	42816018	42836530	-
3371	1451486_at	Region	1200006F02Rik	71706	5	146769947	146786310	-
3372	1455388_at	Region	A030012M09Rik	319263	1	7090301	7142844	+
3373	1452679_at	Region	2410129E14Rik	73710	13	33661517	33664863	-
3374	1449004_at	Region	Mrpl46	67308	7	72567449	72575197	-
3375	1437563_at	Region	Phf20l1	239510	15	66601624	66648123	+
3376	1424754_at	Region	Ms4a7	109225	19	10517536	10531192	-
3377	1454958_at	Region	Gsk3b	56637	16	36906890	37063968	+
3378	1438454_at	Region	B430203M17Rik	320193	19	32132692	32135237	+
3379	1456499_at	Region	LOC552912	552912	11	49872645	49874145	+
3380	1421160_a_at	Region	Rfng	19719	11	120601838	120605297	-
3381	1421962_at	Region	Dnajb5	56323	4	42869016	42874654	+
3382	1450674_at	Region	Cdk5	12568	5	22882139	22887427	-
3383	1458355_x_at	Region	AI553587	103784	11	17106711	17128589	+
3384	1448480_at	Region	1110017C15Rik	66164	8	106354564	106356932	+
3385	1415670_at	Region	Copg	54161	6	88324602	88350257	+
3386	1425826_a_at	Region	Sorbs1	20411	19	39839082	40058071	-
3387	1460390_at	Region	Sorl1	20660	9	41927330	42082982	-
3388	1451078_at	Region	2510039O18Rik	77034	4	146433257	146439676	+
3389	1438988_x_at	Region	Hn1	15374	11	115318446	115335463	-
3390	1418176_at	Region	Vdr	22337	15	97930914	97984749	-
3391	1420977_at	Region	Man1a2	17156	3	99992328	100111138	-
3392	1441623_at	Region	None	None	1	98385859	98387340	+
3393	1423890_x_at	Region	Atp1b1	11931	1	164355219	164376238	-
3394	1434261_at	Region	Sipa1l2	244668	8	124705397	124857145	-
3395	1432713_at	Region	6430709C05Rik	76240	10	108819033	108820567	-
3396	1434683_at	Region	Cutl1	13047	5	135261627	135499343	-
3397	1423816_at	Region	Cxx1b	553127	X	48227121	48228397	-
3398	1450749_a_at	Region	Nr4a2	18227	2	57033695	57040794	-
3399	1455970_at	Region	None	None	3	121649091	121650893	+
3400	1426937_at	Region	6330406I15Rik	70717	5	148350053	148369950	+
3401	1449961_at	Region	Rph3a	19894	5	120092306	120161325	-
3402	1459322_at	Region	Syne2	319565	12	72696171	72966517	+
3403	1434272_at	Region	Cpeb2	231207	5	41990144	42042375	+
3404	1434717_at	Region	Cul3	26554	1	80599109	80655022	-
3405	1455192_at	Region	A230078I05Rik	319998	1	75770303	75776428	+
3406	1419396_at	Region	Arid3a	13496	10	80053267	80077345	+
3407	1435723_at	Region	None	None	13	90540800	90541233	-
3408	1436600_at	Region	Tnrc9	244579	8	89533527	89634739	-
3409	1433460_at	Region	Ttc7b	104718	12	95744448	95966178	-
3410	1419812_s_at	Region	D11Ertd99e	52469	11	101099057	101100035	-
3411	1429076_a_at	Region	Gdpd2	71584	X	95331301	95340254	+
3412	1452896_at	Region	Gtl3	14894	8	94698722	94713270	-
3413	1446094_at	Region	Atbf1	11906	8	108012331	108254925	+
3414	1435629_at	Region	None	None	3	87764423	87764854	-
3415	1448653_at	Region	Eed	13626	7	83945801	83972934	-
3416	1456174_x_at	Region	Ndrg1	17988	15	66953527	66993844	-
3417	1454892_at	Region	Pitpnb	56305	5	110380456	110438052	+
3418	1437037_x_at	Region	AL033314	56463	6	28527393	28935815	+
3419	1428521_at	Region	Thap3	69876	4	150475001	150481359	-
3420	1454717_at	Region	Ankrd27	245886	7	30750473	30804195	+
3421	1454863_at	Region	Ankrd11	77087	8	122264618	122423554	-
3422	1420720_at	Region	Nptx2	53324	5	143587516	143599211	+
3423	1429047_at	Region	Gtl7	76246	2	119188947	119248563	+
3424	1455028_at	Region	Mapt	17762	11	104052587	104149347	+
3425	1450505_a_at	Region	1810015C04Rik	66270	15	25929616	25961245	+
3426	1427002_s_at	Region	6330406P08Rik	74008	11	109310969	109394419	+
3427	1417873_at	Region	Pwp1	103136	10	85832809	85850040	+
3428	1417613_at	Region	Ier5	15939	1	154988211	154990321	-
3429	1436217_at	Region	Zfp148	22661	16	32188918	32308456	+
3430	1435803_a_at	Region	Eif4e2	26987	1	87030076	87054566	+
3431	1432073_at	Region	1700113I22Rik	73635	11	101239901	101246420	-
3432	1457161_at	Region	9530029O12Rik	399629	11	45615754	45616493	+

3433	1456700_x_at	Region	Marcks	17118	10	37207003	37211096	-
3434	1427770_a_at	Region	Slc2a3	20527	6	123394798	123409491	-
3435	1416781_at	Region	Praf2	54637	X	5967511	5970003	+
3436	1426912_at	Region	Rfwd2	26374	1	159136169	159251421	+
3437	1435130_at	Region	LOC383103	383103	16	10101468	10121829	-
3438	1451044_at	Region	Sip1	66603	12	55746625	55761702	+
3439	1416164_at	Region	Fbln5	23876	12	97192023	97264513	-
3440	1425577_at	Region	9830124H08Rik	219105	14	51315574	51334091	-
3441	1454977_at	Region	AU020772	277939	7	94478918	94576823	+
3442	1422165_at	Region	Pou3f4	18994	X	105415830	105416915	+
3443	1418942_at	Region	Ccdc2	67694	4	93629901	93668301	+
3444	1423465_at	Region	Sdfr2	20321	3	115599594	115672860	+
3445	1453721_a_at	Region	Slc31a2	20530	4	61377636	61389602	+
3446	1437908_a_at	Region	1200007D18Rik	67458	17	24356346	24451768	+
3447	1430549_at	Region	Bet1l	54399	7	135255293	135258149	-
3448	1428140_at	Region	Oxct1	67041	15	3809284	3936636	+
3449	1433544_at	Region	Als2cr2	227154	1	59278531	59300058	+
3450	1421508_at	Region	Odz1	23963	X	37053187	37688613	-
3451	1415745_a_at	Region	Dscr3	13185	16	93894798	93923338	-
3452	1440516_at	Region	None	None	X	58933477	58934253	-
3453	1441791_at	Region	Oprm1	18390	10	3465202	3714699	-
3454	1435921_at	Region	D8Ertd587e	52335	8	109024127	109027469	-
3455	1425574_at	Region	Epha3	13837	16	62549020	62869320	-
3456	1438046_at	Region	AU019823	270156	9	50677566	50689572	-
3457	1439537_at	Region	None	None	6	31168762	31169604	-
3458	1440901_at	Region	Dgkb	217480	12	34483693	34776186	+
3459	1457112_at	Region	A230106N23	329274	1	155972580	155984674	-
3460	1437151_at	Region	Usp22	216825	11	60878669	60900563	-
3461	1449442_at	Region	Pex11a	18631	7	73535535	73541429	-
3462	1420614_at	Region	Tcte1l	67117	X	7893210	7901923	-
3463	1426950_at	Region	Parp16	214424	9	65336986	65361483	+
3464	1425339_at	Region	Plcb4	18798	2	135319401	135526921	+
3465	1434231_x_at	Region	Rpl35	66489	2	38933743	38937293	-
3466	1424916_x_at	Region	8030466O12Rik	233893	7	121453399	121456553	-
3467	1444447_at	Region	Nek7	59125	1	138334093	138469075	-
3468	1420967_at	Region	Slc25a15	18408	8	21128178	21150724	-
3469	1416118_at	Region	None	None	3	68705851	68714366	-
3470	1432931_at	Region	LOC544914	544914	12	1837	24141	-
3471	1435751_at	Region	Abcc9	20928	6	143432402	143545542	-
3472	1448467_a_at	Region	LOC114601	114601	19	5496839	5515060	-
3473	1417409_at	Region	Jun	16476	4	94024127	94027259	-
3474	1429796_at	Region	Kalrn	224126	16	32822303	32837233	-
3475	1429273_at	Region	Bmper	73230	9	23116165	23378425	+
3476	1435776_at	Region	LOC545365	545365	1	138696345	138701165	-
3477	1435676_at	Region	BC027092	214552	9	45780203	45790044	-
3478	1447849_s_at	Region	None	None	8	114996442	114996559	-
3479	1436964_at	Region	D7Ertd715e	52480	7	53714049	53752237	-
3480	1440831_at	Region	6230421P05Rik	109033	16	86887727	86889563	+
3481	1450299_at	Region	Chrna7	11441	7	56872429	56986255	-
3482	1457707_at	Region	Gm489	244049	7	65956375	66185373	-
3483	1437061_at	Region	Mbd1	17190	18	74502520	74516914	+
3484	1423274_at	Region	Ddx26	18130	14	57209161	57294009	-
3485	1452811_at	Region	Atic	108147	1	71853291	71875521	+
3486	1436757_a_at	Region	Cox6b1	110323	7	26029666	26038843	-
3487	1418451_at	Region	Gng2	14702	14	18235904	18323407	-
3488	1434047_x_at	Region	Hnrpa2b1	53379	6	51608402	51617123	-
3489	1423228_at	Region	B4galt6	56386	18	20900931	20961332	-
3490	1460346_at	Region	Arsa	11883	15	89525354	89529236	-
3491	1427705_a_at	Region	Nfkb1	18033	3	134467828	134574475	-
3492	1429423_at	Region	4930518I15Rik	74704	2	156313792	156315008	-
3493	1450062_a_at	Region	Maged1	94275	X	89196132	89202732	-
3494	1444215_at	Region	None	None	14	88328796	88329302	-
3495	1418792_at	Region	Sh3gl2	20404	4	84191648	84375572	+
3496	1423589_at	Region	Arpc4	68089	6	113945942	113957478	+
3497	1426398_at	Region	6130401J04Rik	66799	1	16725948	16804454	-

3498	1444100_at	Region	1110003E01Rik	68552	5	64251633	64294659	-
3499	1426696_at	Region	Lrpap1	16976	5	33580650	33593377	-
3500	1436938_at	Region	Rbms3	207181	9	116564518	117239065	-
3501	1423846_x_at	Region	Tuba2	22143	15	98989227	98992223	-
3502	1416859_at	Region	Fkbp3	30795	12	61840122	61851631	-
3503	1424577_at	Region	BC008103	229524	3	88653506	88657764	-
3504	1435844_at	Region	A330009N23Rik	380977	15	101279404	101280305	+
3505	1451367_at	Region	Cops6	26893	5	137133910	137136792	+
3506	1451474_a_at	Region	Parp8	52552	13	113306754	113477704	-
3507	1449370_at	Region	Sox4	20677	13	28430338	28433304	-
3508	1415929_at	Region	Map1lc3b	67443	8	120972448	120980028	+
3509	1453021_at	Region	Stxbp5	78808	10	9633720	9718785	-
3510	1456778_at	Region	Nrp2	18187	1	62996720	63108906	+
3511	1455526_at	Region	Diras1	208666	10	81153435	81159222	-
3512	1424049_at	Region	Lrrc42	77809	4	106192419	106212342	-
3513	1431030_a_at	Region	Rnf14	56736	18	38520411	38541525	+
3514	1426049_a_at	Region	Terf2ip	57321	8	111309109	111318168	+
3515	1436663_at	Region	1700105P06Rik	68229	19	7070309	7096879	-
3516	1432181_s_at	Region	Ecgf1	72962	15	89424804	89429909	-
3517	1425702_a_at	Region	Enpp5	83965	17	41582289	41590057	+
3518	1425050_at	Region	Isoc1	66307	18	58884044	58904170	+
3519	1454729_at	Region	B130017P16Rik	81907	9	103460526	103476281	-
3520	1426757_at	Region	Ampd2	109674	3	107869804	107882369	-
3521	1423162_s_at	Region	Spred1	114715	2	116635298	116693185	+
3522	1439257_x_at	Region	Rpn1	103963	6	88518156	88540499	+
3523	1435432_at	Region	Centg2	347722	1	89273393	89708522	+
3524	1451980_at	Region	MGI:2384865	213819	6	4553951	4596266	+
3525	1433726_at	Region	2310035C23Rik	227446	1	105555177	105695178	+
3526	1444424_at	Region	Uble1a	56459	7	13308414	13337163	-
3527	1460166_at	Region	Rit2	19762	18	31217063	31558917	-
3528	1421818_at	Region	Bcl6	12053	16	22748871	22772551	-
3529	1436411_at	Region	Atp13a5	268878	16	28020880	28167802	-
3530	1423961_at	Region	Wdr26	226757	1	181127684	181165201	-
3531	1419641_at	Region	Purb	19291	11	6369560	6370704	-
3532	1426108_s_at	Region	Cacnb1	12295	11	97823988	97904149	-
3533	1425221_at	Region	E030025L21Rik	403205	12	32532013	32555904	+
3534	1450483_at	Region	Gja12	118454	11	58901195	58908842	-
3535	1448894_at	Region	Akr1b8	14187	6	34448432	34462722	+
3536	1451430_at	Region	Slit2	20563	5	46748038	47069334	+
3537	1424412_at	Region	Ogfrl1	70155	1	23503663	23655192	-
3538	1425400_a_at	Region	Cited4	56222	4	119689045	119690264	+
3539	1456320_at	Region	BC049806	213056	1	58832103	58870824	-
3540	1426791_at	Region	Rusc2	100213	4	43322447	43343004	+
3541	1424683_at	Region	1810015C04Rik	66270	15	25929616	25961245	+
3542	1425362_at	Region	Hrbl	231801	5	136601442	136635293	-
3543	1423189_at	Region	6720456B07Rik	101314	6	114172603	114184782	+
3544	1440655_at	Region	3110007P09Rik	73094	4	101719268	101930082	+
3545	1425893_a_at	Region	Fhit	14198	14	7225961	7249456	-
3546	1455329_at	Region	None	None	19	28271216	28272833	+
3547	1452964_at	Region	4932702F08Rik	74410	2	35683403	35911458	-
3548	1448651_at	Region	Nudt5	53893	2	5762306	5786008	+
3549	1436266_x_at	Region	Cbx1	12412	11	96610246	96629727	+
3550	1416618_at	Region	Ppox	19044	1	171205831	171209999	-
3551	1417786_a_at	Region	Rgs19	56470	2	181405379	181410880	-
3552	1454606_at	Region	4933426M11Rik	217684	12	77651814	77742088	+
3553	1428503_a_at	Region	Nkiras1	69721	14	16794546	16805426	+
3554	1441927_at	Region	Syt7	54525	19	9585980	9644941	+
3555	1425684_at	Region	2310005E10Rik	67861	6	34478555	34491217	+
3556	1451205_at	Region	Psmb4	19172	3	94372151	94374759	-
3557	1438113_at	Region	Zmat4	320158	8	22424307	22817717	+
3558	1435009_at	Region	Slc9a6	236794	X	51364568	51416607	+
3559	1424411_at	Region	AI840826	407243	2	167099958	167170277	-
3560	1456990_at	Region	None	None	9	77685607	77686270	+
3561	1455724_at	Region	None	None	X	73110270	73111259	-
3562	1455123_at	Region	St18	240690	1	6695648	6826537	+

3563	1455462_at	Region	Adcy2	210044	13	64641047	65020780	-
3564	1431780_at	Region	1700021K14Rik	69398	9	108063750	108067667	+
3565	1433606_at	Region	Mitc1	75901	14	28614206	28661650	+
3566	1424466_at	Region	Ipo9	226432	1	135234946	135287679	-
3567	1426503_a_at	Region	Rnf121	75212	7	96126119	96178400	-
3568	1422858_at	Region	Trip4	56404	9	65956276	66032003	-
3569	1422241_a_at	Region	Ndufa1	54405	X	31808155	31811717	-
3570	1425196_a_at	Region	Hint2	68917	4	43570149	43572439	-
3571	1435820_x_at	Region	Ddr1	12305	17	33397888	33421229	-
3572	1446212_at	Region	Kcnq5	226922	1	22083632	22084318	-
3573	1419162_s_at	Region	Dnajc3	19107	14	113495773	113540797	+
3574	1419425_at	Region	Cnr1	12801	4	34203466	34224064	+
3575	1426668_at	Region	Slc30a9	109108	5	66073098	66122283	+
3576	1459722_at	Region	Zswim6	67263	13	103950578	104158812	-
3577	1456144_at	Region	Nav3	260315	10	109569106	109718861	-
3578	1437395_at	Region	Zcchc11	230594	4	107418943	107518318	+
3579	1450733_at	Region	Bicd2	76895	13	48941109	48984662	+
3580	1416792_at	Region	Ppm1g	14208	5	29661606	29679483	-
3581	1434147_at	Region	4631403P03Rik	225392	18	38179349	38182885	+
3582	1453731_a_at	Region	2610318G18Rik	67171	3	106343709	106369981	+
3583	1425186_at	Region	Lmbrd1	68421	1	24831774	25014116	+
3584	1435555_at	Region	None	None	1	165786931	165788757	-
3585	1436970_a_at	Region	Pdgfrb	18596	18	61270767	61310385	+
3586	1440076_at	Region	Sqstm1	18412	11	49952993	49963631	-
3587	1428386_at	Region	Acsl3	74205	1	78992139	79040786	+
3588	1422730_at	Region	Limd1	29806	9	123502410	123545221	+
3589	1428251_at	Region	Smchd1	74355	17	69117606	69200198	-
3590	1449773_s_at	Region	Gadd45b	17873	10	81061547	81063660	+
3591	1431085_a_at	Region	Pcmt1	18537	10	7494777	7527640	-
3592	1437832_x_at	Region	Wars	22375	12	104331027	104364190	-
3593	1442982_at	Region	E230015L20Rik	320234	14	25620649	25647415	-
3594	1428279_a_at	Region	Atxn7l4	72174	12	29753981	29857347	+
3595	1457248_x_at	Region	Hsd17b7	15490	1	169883718	169903346	-
3596	1456155_x_at	Region	Fuca1	71665	4	134802014	134821570	+
3597	1424714_at	Region	Aldoc	11676	11	78048858	78052417	+
3598	1442809_at	Region	Scn9a	20274	2	66338406	66490352	-
3599	1458205_at	Region	None	None	10	54310358	54310817	-
3600	1450099_a_at	Region	Gba	14466	3	88959413	88965154	+
3601	1434958_at	Region	Sacs	50720	14	55749728	55750681	+
3602	1417624_at	Region	Nab1	17936	1	52758832	52802132	-
3603	1452649_at	Region	Rtn4	68585	11	29587737	29637706	+
3604	1416429_a_at	Region	Cat	12359	2	103158757	103189874	-
3605	1459219_at	Region	Rapgef2	76089	3	78790668	79018371	-
3606	1425676_a_at	Region	Elovl1	54325	4	117387050	117391522	+
3607	1447100_s_at	Region	5730508B09Rik	70617	3	126755462	126781211	-
3608	1428261_at	Region	2310042L06Rik	76457	15	82178713	82192917	+
3609	1429907_at	Region	1700094D03Rik	73545	3	89873214	89876294	+
3610	1444488_at	Region	Cadps	27062	14	10830082	11279704	-
3611	1436584_at	Region	Spry2	24064	14	100435326	100439630	-
3612	1428240_at	Region	Nrxn1	18189	17	87900413	88568779	-
3613	1441559_at	Region	B930007P11Rik	98080	17	40019159	40019823	+
3614	1426448_at	Region	Pja1	18744	X	94067507	94072041	-
3615	1456547_at	Region	Ski	20481	4	153649703	153714882	-
3616	1423549_at	Region	Slc1a4	55963	11	20196998	20227464	-
3617	1456207_at	Region	None	None	X	70073103	70074645	-
3618	1451002_at	Region	Aco2	11429	15	81923222	81965850	+
3619	1427004_at	Region	Fbxo2	230904	4	146653030	146658779	+
3620	1416006_at	Region	Mdk	17242	2	91634570	91637031	-
3621	1458492_x_at	Region	MGI:2446259	235106	9	28892331	29088826	-
3622	1424088_at	Region	MGI:1929864	60315	15	102392178	102398329	+
3623	1450632_at	Region	LOC433749	433749	4	116687028	116688500	-
3624	1440522_at	Region	B930075F07	331484	X	97996786	97997457	+
3625	1417701_at	Region	Ppp1r14c	76142	10	7041618	7140096	-
3626	1448688_at	Region	Podxl	27205	6	31596475	31641044	-
3627	1437422_at	Region	Sema5a	20356	15	32419980	32698954	+

3628	1423125_at	Region	Dcamkl1	13175	3	54876964	55171014	+
3629	1454603_a_at	Region	Cnot2	72068	10	116174961	116270953	-
3630	1450219_at	Region	Htr1a	15550	13	101659844	101664197	+
3631	1455767_x_at	Region	Rpl21	19933	5	145724838	145728723	+
3632	1420847_a_at	Region	Fgfr2	14183	7	124341981	124389452	-
3633	1431422_a_at	Region	Dusp14	56405	11	83776037	83796349	-
3634	1427652_x_at	Region	Synj2	20975	17	5881998	5950653	+
3635	1437624_x_at	Region	Nudt16l1	66911	16	4609000	4610909	+
3636	1452464_a_at	Region	Metapl1	66559	2	71151081	71222934	+
3637	1421042_at	Region	Arhgef2	16800	3	88365002	88390778	+
3638	1437615_s_at	Region	5730409F24Rik	107305	19	9885312	9910916	+
3639	1439850_at	Region	BC066028	407812	7	135211557	135223941	-
3640	1425455_a_at	Region	Churc1	211151	12	73588088	73639767	+
3641	1437460_x_at	Region	Rin1	225870	19	4839598	4845861	+
3642	1416829_at	Region	Atp5b	11947	10	127820017	127827098	+
3643	1456659_at	Region	LOC552902	552902	17	5075931	5076497	+
3644	1421842_a_at	Region	Scamp4	56214	10	80733774	80746677	+
3645	1448917_at	Region	Thrap6	69790	15	52712125	52730111	+
3646	1433604_x_at	Region	Aldoa	11674	7	120844967	120848914	-
3647	1427640_a_at	Region	Cbfa2t1h	12395	4	13711747	13818082	+
3648	1449682_s_at	Region	2410129E14Rik	73710	13	33661517	33664863	-
3649	1427084_a_at	Region	Map4k5	399510	12	66635229	66724562	-
3650	1425508_s_at	Region	Arfrp1	76688	2	181074790	181082318	-
3651	1434359_at	Region	6330500D04Rik	193385	13	24184806	24185879	+
3652	1424283_at	Region	Jtb	23922	3	90042068	90046256	+
3653	1438702_at	Region	None	None	12	91193373	91194426	+
3654	1440815_x_at	Region	LOC545039	545039	14	40208840	40216744	+
3655	1420953_at	Region	Add1	11518	5	33061487	33119289	+
3656	1418019_at	Region	Cpd	12874	11	76507648	76572655	-
3657	1458833_at	Region	C130076O07Rik	319504	12	40985533	41346376	+
3658	1416345_at	Region	Timm8a	30058	X	128082197	128086568	-
3659	1431228_s_at	Region	4930526I15Rik	75135		33381768	33382476	+
3660	1450224_at	Region	Col4a3	12828	1	82916082	83050374	+
3661	1433947_at	Region	Rab37	58222	11	114975278	114983321	+
3662	1454587_at	Region	9330199C07Rik	77245	14	37072410	37073349	-
3663	1417896_at	Region	Tjp3	27375	10	81409313	81427318	-
3664	1444015_at	Region	Papola	18789	12	101247264	101284473	+
3665	1426926_at	Region	Plcg2	234779	8	116832841	116969047	+
3666	1422525_at	Region	Atp5k	11958	5	107509508	107510602	-
3667	1439964_at	Region	D8Bwg1414e	66817	8	111162487	111174264	-
3668	1442328_at	Region	None	None	7	39916613	39916968	-
3669	1456150_at	Region	A630082K20Rik	338523	6	39276016	39343119	-
3670	1452806_at	Region	1500016O10Rik	68952	7	120873047	120879946	+
3671	1439292_at	Region	None	None	6	31276718	31277134	+
3672	1455722_at	Region	None	None	NONE	NONE	NONE	NONE
3673	1428375_at	Region	4932415G12Rik	67723	10	94647892	94662897	-
3674	1452972_at	Region	1700013G20Rik	75516	12	8346312	8352709	+
3675	1436315_at	Region	Myst3	244349	8	21614114	21695221	+
3676	1426047_a_at	Region	Ptprr	19279	10	115705801	115963929	+
3677	1453063_at	Region	Cltb	74325	13	53203300	53220857	-
3678	1424258_at	Region	Polr2d	69241	18	32029303	32036845	+
3679	1451092_a_at	Region	Rangap1	19387	15	81754967	81780618	-
3680	1428620_at	Region	Ensa	56205	3	95112951	95119278	+
3681	1438876_at	Region	A130014A01Rik	402737	NONE	NONE	NONE	NONE
3682	1438231_at	Region	Foxp2	114142	6	14888986	15429612	+
3683	1428355_at	Region	Osbp2	74309	11	3598519	3616991	-
3684	1426482_at	Region	Prkrir	72981	7	92794813	92809512	+
3685	1419110_at	Region	Riok1	71340	13	37576092	37600226	+
3686	1422231_a_at	Region	Tnfrsf25	85030	4	150608296	150612481	+
3687	1421879_at	Region	Mtmr1	53332	X	66033251	66087687	+
3688	1436279_at	Region	Slc26a7	208890	4	14432162	14548722	-
3689	1455390_at	Region	AI839550	233065	7	25721615	25726918	+
3690	1435715_x_at	Region	Tdg /// LOC434200 /// LOC545124	21665	10	82517850	82538768	+
3691	1427401_at	Region	Chrna5	110835	9	55117369	55125764	+

3692	1422704_at	Region	Gyk	14933	X	80362593	80437339	-
3693	1437311_at	Region	A930034L06Rik	319317	2	157832651	157838559	+
3694	1416875_at	Region	Parvg	64099	15	84373319	84391538	+
3695	1449168_a_at	Region	Akap2	11641	4	57797608	57837140	+
3696	1453149_at	Region	MGI:1917156	69906	15	38993909	39012345	-
3697	1455491_at	Region	LOC432467	432467	10	62980904	62990550	-
3698	1456495_s_at	Region	Osbpl6	99031	2	76246089	76293794	+
3699	1436142_at	Region	3526401B18Rik	70774	12	73189718	73190242	+
3700	1439994_at	Region	None	None	5	52092712	52093649	+
3701	1449294_at	Region	Mrps15	66407	4	125074041	125082649	+
3702	1426527_at	Region	Toe1	68276	4	115762908	115766464	-
3703	1448331_at	Region	Ndufb7	66916	8	82824283	82829148	+
3704	1421534_at	Region	None	None	6	50344693	50347100	-
3705	1458342_at	Region	None	None	12	81545948	81546219	-
3706	1445710_x_at	Region	1110051B16Rik	278672	14	24358464	24365306	+
3707	1452962_at	Region	Tmem25	71687	9	44782634	44788071	-
3708	1452895_at	Region	Fbxo45	268882	16	31039483	31056396	-
3709	1458702_at	Region	2610316D01Rik	72511	3	44739307	44837135	-
3710	1452046_a_at	Region	Ppp1cc	19047	5	121310629	121327619	+
3711	1457275_at	Region	Dmn	233335	7	61614971	61644496	-
3712	1439959_at	Region	Fgf11	14166	11	69523860	69527283	-
3713	1427284_a_at	Region	Ttpa	50500	4	20109542	20132072	+
3714	1427275_at	Region	Smc4l1	70099	3	68675494	68705145	+
3715	1452850_s_at	Region	Brms1l	52592	12	52556120	52589380	+
3716	1434127_a_at	Region	H3f3a	15078	1	180756388	180767423	-
3717	1456510_x_at	Region	MGI:3026615	393082	15	100410740	100418501	+
3718	1420950_at	Region	Znrf1	170737	8	110834431	110921367	+
3719	1450961_a_at	Region	Tceal3 /// LOC331528 /// Tceal5 /// LOC594844	331528	X	130135507	130213313	+
3720	1453102_at	Region	Flrt3	71436	2	140172056	140185326	-
3721	1443890_at	Region	None	None	2	34782772	34783257	-
3722	1449108_at	Region	Fdx1	14148	9	52015254	52035480	-
3723	1445723_at	Region	Akr1b3	11677	6	34398202	34411739	-
3724	1434223_at	Region	1810007P19Rik	230751	4	125085714	125116447	+
3725	1435616_at	Region	Cyp20a1	77951	1	60674554	60691350	+
3726	1449054_a_at	Region	Pcbp4	59092	9	106517872	106528323	+
3727	1436482_a_at	Region	Sdc3	20970	4	129589463	129623202	+
3728	1433756_at	Region	4930429A08Rik	74648	4	128175113	128176098	-
3729	1457999_at	Region	2410005O16Rik	66302	4	19502173	19533898	+
3730	1456616_a_at	Region	Bsg	12215	10	79827124	79834519	+
3731	1431229_at	Region	C030032C09Rik	77531	10	90548071	90943317	+
3732	1439038_at	Region	9130227C08Rik	219094	14	50402985	50414797	+
3733	1417089_a_at	Region	Ckmt1	12716	2	120872490	120877586	+
3734	1423102_a_at	Region	Rnf10	50849	5	114351397	114382520	-
3735	1443882_at	Region	None	None	18	61419706	61421388	-
3736	1431101_a_at	Region	Srd5a1	78925	13	65625480	65642329	-
3737	1451248_at	Region	BC006705	214572	8	105506901	105547541	+
3738	1429238_a_at	Region	5730405M13Rik	66627	5	123283663	123286801	+
3739	1450685_at	Region	Arpp19	59046	9	75190732	75198588	+
3740	1453271_at	Region	Phf14	75725	6	11909551	11991719	+
3741	1450383_at	Region	Ldlr	16835	9	21613867	21637395	+
3742	1446159_at	Region	Pak7	241656	2	135595752	135901791	-
3743	1417329_at	Region	Slc23a2	54338	2	131566349	131658961	-
3744	1438635_x_at	Region	B930041F14Rik	230991	4	154186704	154188843	+
3745	1418048_at	Region	1110059G10Rik	66202	9	122962862	122968745	-
3746	1424372_at	Region	Mrpl32	75398	13	14054854	14057590	-
3747	1450804_at	Region	Kif5c	16574	2	49551476	49706940	+
3748	1422486_a_at	Region	Smad4	17128	18	73873242	73937970	-
3749	1415930_a_at	Region	Map1lc3b /// LOC434375	434375	9	1	1533	+
3750	1451452_a_at	Region	Rgs16	19734	1	153624984	153630102	+
3751	1430999_a_at	Region	Scoc	56367	8	82691600	82699587	-
3752	1449614_s_at	Region	AI314976	106821	17	45940087	45947164	+

3753	1459750_s_at	Region	Gpr123	52389	7	134230082	134274053	+
3754	1427070_at	Region	5730407K14Rik	101113	2	164242750	164244365	+
3755	1447917_x_at	Region	Ntan1	18203	16	12555143	12571313	+
3756	1453365_at	Region	Rabgap1l	29809	1	160161852	160697585	-
3757	1421662_a_at	Region	Tusc3	80286	8	37881026	38001726	+
3758	1417916_a_at	Region	Fxc1 /// 8030491N06Rik	14356	7	99755452	99756758	+
3759	1448213_at	Region	Anxa1	16952	19	19618706	19635993	-
3760	1438640_x_at	Region	Pgk1 /// LOC381164 /// LOC432633 /// LOC433594 /// LOC545273	18655	X	100788594	100805024	+
3761	1448657_a_at	Region	Dnajb10	56812	1	75524048	75533251	+
3762	1435977_at	Region	2700022B06Rik	72589	7	75689389	75689956	-
3763	1436125_at	Region	D16Ertd472e	67102	16	77629000	77662117	-
3764	1460307_at	Region	Akt3	23797	1	176955410	177064396	-
3765	1434948_at	Region	Rab11fip2	74998	19	59505244	59508287	-
3766	1449937_at	Region	Pp11r	19011	15	97786664	97796795	-
3767	1459537_at	Region	None	None	6	29893596	29894061	+
3768	1423560_at	Region	Nell2	54003	15	95287908	95595549	-
3769	1426280_at	Region	AV249152	216560	11	21467074	21793479	+
3770	1428535_at	Region	9430020K01Rik	240185	18	4653875	4685495	+
3771	1419078_at	Region	Nin	18080	12	66850915	66944121	-
3772	1441642_at	Region	None	None	NONE	NONE	NONE	NONE
3773	1422609_at	Region	Arpp19	59046	9	75190732	75198588	+
3774	1416312_at	Region	Rars	104458	11	35602413	35628536	-
3775	1426390_a_at	Region	Arf1	11840	11	58937042	58939152	-
3776	1434759_at	Region	Lrrtm3	216028	10	63866884	64028642	-
3777	1436680_s_at	Region	Ddb2	107986	2	90916605	90941731	-
3778	1421772_a_at	Region	Cox7a2l	20463	17	81335584	81347955	-
3779	1426783_at	Region	Gcn5l2	14534	11	100526325	100533491	-
3780	1442786_s_at	Region	D5Bwg0860e	52822	5	87850046	87908952	+
3781	1455309_at	Region	Tmem16f	105722	15	95858140	96042070	+
3782	1417404_at	Region	Elovl6	170439	3	128465310	128571362	+
3783	1428159_s_at	Region	Ndufab1	70316	7	115965902	115979706	-
3784	1452820_at	Region	Brwd2	207425	7	123667627	123711434	+
3785	1426517_at	Region	Gnaz	14687	10	75068431	75117077	+
3786	1417030_at	Region	2310028N02Rik	66950	1	191062230	191089190	+
3787	1416201_at	Region	Crk	12928	11	75404968	75431752	+
3788	1430498_at	Region	9130009I01Rik	71594	7	76564101	76565340	+
3789	1418699_s_at	Region	Fech	14151	18	64689244	64721633	-
3790	1435086_s_at	Region	Klhdc2	69554	12	66128557	66142253	+
3791	1456069_at	Region	Dtna	13527	18	23643946	23881842	+
3792	1452768_at	Region	Tex261	21766	6	84114647	84119990	-
3793	1416311_s_at	Region	Tuba3 /// Tuba7	22144	6	125934969	125942711	-
3794	1433184_at	Region	6720477C19Rik	77731	NONE	NONE	NONE	NONE
3795	1434000_at	Region	Kras	16653	6	146066254	146096662	-
3796	1438727_at	Region	Wdr32	242418	4	45258063	45290836	+
3797	1443805_at	Region	2700080J24Rik	67969	NONE	NONE	NONE	NONE
3798	1459922_at	Region	AI646383	106627	17	34030421	34030829	+
3799	1431602_a_at	Region	1810064L21Rik	66435	14	113542917	113545445	-
3800	1424745_at	Region	2900006B13Rik	72947	11	51337644	51355878	+
3801	1447314_at	Region	Tgfbr3	21814	5	106170128	106359608	-
3802	1435147_x_at	Region	Igsf4d	239857	16	65672212	65964483	-
3803	1447774_x_at	Region	5730469M10Rik	70564	14	39139992	39160295	-
3804	1444681_at	Region	D14Ertd171e	238988	14	25761526	26615037	+
3805	1459961_a_at	Region	None	None	2	167726091	167726379	-
3806	1436571_at	Region	None	None	4	137772324	137773542	-
3807	1436235_x_at	Region	4732471D19Rik	319719	13	53135721	53161052	+
3808	1459311_at	Region	Pde4d	238871	13	105985350	106200700	+
3809	1439767_at	Region	Dlgap2	244310	8	14073597	14226162	+
3810	1443822_s_at	Region	None	None	10	71384458	71384664	-
3811	1441956_s_at	Region	Cutl1	13047	5	135261627	135499343	-
3812	1424007_at	Region	Gdf10	14560	14	32056733	32068431	+

3813	1437093_at	Region	Dnaic1	68922	4	41708645	41777001	+
3814	1424436_at	Region	Gart	14450	16	90778573	90804117	-
3815	1456217_at	Region	D7Wsu128e	28018	7	115945280	115956990	+
3816	1460363_at	Region	Tnrc6c	217351	11	117475908	117583657	+
3817	1459774_at	Region	Tm4sf9	56224	3	137631430	137793659	+
3818	1426728_x_at	Region	Ptdss2	27388	7	135533273	135558141	+
3819	1453414_at	Region	Ypel2	77864	11	86668572	86700001	-
3820	1451077_at	Region	Rpl5 /// LOC545265 /// LOC545304 /// LOC545478 /// LOC545858	19983	5	106971039	106978499	+
3821	1460434_at	Region	Fundc2	67391	X	70043077	70056771	+
3822	1420866_at	Region	Zfp161	22666	17	67159059	67165625	+
3823	1431818_at	Region	1700012B15Rik	74173	12	273	3622	+
3824	1415802_at	Region	Slc16a1	20501	3	104066454	104086248	+
3825	1417108_at	Region	Knsl8	74764	17	44141181	44155796	-
3826	1457048_at	Region	Gpr103	229214	3	35641294	35684794	-
3827	1419674_a_at	Region	Dpep1	13479	8	122561123	122576107	+
3828	1418146_a_at	Region	Rbl2	19651	8	90356622	90410210	+
3829	1424374_at	Region	Gimap4	107526	6	48816795	48824784	+
3830	1450986_at	Region	Nol5	55989	1	59988637	60015046	+
3831	1436173_at	Region	Dlc1	50768	8	35376686	35421146	-
3832	1422477_at	Region	Cables1	63955	18	12026875	12133165	+
3833	1441603_at	Region	None	None	15	78588299	78588940	-
3834	1427157_at	Region	E030025D05Rik	216613	11	28283605	28479086	-
3835	1436248_at	Region	A330066M24Rik	320357	1	157040754	157041935	-
3836	1459976_s_at	Region	Sod1	20655	16	89378174	89383756	+
3837	1449531_at	Region	Leprel2	14789	6	125497863	125514455	-
3838	1417619_at	Region	Gadd45gip1	102060	8	84099337	84102537	+
3839	1437421_at	Region	6330509M05Rik	102913	X	18340396	18341844	-
3840	1460690_at	Region	BC003940	192173	11	120363931	120370960	-
3841	1417666_at	Region	Dnttip1	76233	2	164202748	164224953	+
3842	1439136_at	Region	Ssbp3	72475	4	105870375	106008599	+
3843	1427212_at	Region	Mapkap1	227743	2	34339345	34556075	+
3844	1417169_at	Region	Usp2	53376	9	44056022	44083332	+
3845	1438221_at	Region	C130065N10Rik	319340	1	58827446	58828542	-
3846	1422042_at	Region	Gje1	118446	5	136904409	136913559	-
3847	1455379_at	Region	Dnali1	75563	4	124082452	124092770	-
3848	1460673_at	Region	Fpgs	14287	2	32614771	32626294	-
3849	1433100_at	Region	AI451006	105847	15	89403881	89408357	-
3850	1443337_at	Region	B130020M22Rik	320217	NONE	NONE	NONE	NONE
3851	1460398_at	Region	Phf8	320595	X	145055576	145160228	+
3852	1439175_at	Region	A230006I23Rik	319919	4	75198759	75199393	-
3853	1456943_a_at	Region	None	None	2	163948096	163950053	+
3854	1452659_at	Region	Dek	110052	13	46682934	46704259	-
3855	1439266_a_at	Region	Polr3k	67005	2	181581407	181587121	+
3856	1421012_at	Region	Srprb	20818	9	103163286	103177389	-
3857	1456057_x_at	Region	1110006I15Rik	68539	19	10067157	10078240	-
3858	1421900_at	Region	Eif2ak1	15467	5	142939806	142944198	+
3859	1425509_at	Region	Mark1	226778	1	184396055	184497931	-
3860	1422562_at	Region	Rrad	56437	8	103921586	103924050	-
3861	1456726_x_at	Region	Qars	97541	9	108556974	108564815	+
3862	1448882_at	Region	0610009E20Rik	66048	11	72901191	72902695	-
3863	1452821_at	Region	Tial1	21843	7	122491640	122510627	-
3864	1416535_at	Region	Mcrs1 /// LOC433780	433780	4	143502382	143504216	+
3865	1434109_at	Region	Sh3bgrl2	212531	9	83878025	83930061	+
3866	1430636_at	Region	C030010B13Rik	77310	2	173046327	173120005	-
3867	1436338_at	Region	None	None	9	50961974	50962715	-
3868	1419064_a_at	Region	Ugt8a	22239	3	124748763	124839904	-
3869	1444728_at	Region	None	None	6	33695094	33695970	+
3870	1424248_at	Region	MGI:107562	74100	9	112222231	112229149	-
3871	1452283_at	Region	5133400D11Rik	71323	6	146741343	146750544	+
3872	1435232_x_at	Region	None	None	1	4736767	4737197	-

3873	1444640_at	Region	MGI:3576484	433931	5	107388818	107425565	+
3874	1433842_at	Region	Lrrfip1	16978	1	90875248	90952241	+
3875	1444538_at	Region	AL033314	56463	6	28527393	28935815	+
3876	1460579_at	Region	Dnpep	13437	1	75598827	75608568	-
3877	1424992_at	Region	1810034M08Rik	69786	6	86345388	86360039	+
3878	1439542_at	Region	Zfp651	270210	9	121778127	121782382	+
3879	1448529_at	Region	Thbd	21824	2	147861407	147865039	-
3880	1422551_at	Region	Zfp306	72739	13	20867145	20884810	-
3881	1455876_at	Region	Slc4a7	218756	14	13169299	13264198	+
3882	1428596_at	Region	2700008N14Rik	76795	11	49884214	49925626	+
3883	1449485_at	Region	Ripk1	19766	13	33537383	33569679	+
3884	1416538_at	Region	Ysg2	22619	9	37555344	37589819	+
3885	1435695_a_at	Region	A030007L17Rik	68252	6	55129420	55137192	-
3886	1433870_at	Region	BC022765	217138	11	96750411	96756735	+
3887	1458399_at	Region	Lrrc3	237387	10	78008170	78010833	-
3888	1422045_a_at	Region	Ptpn12	19248	5	19439215	19508367	-
3889	1455675_a_at	Region	Tial1	21843	7	122491640	122510627	-
3890	1455825_s_at	Region	Lnx1	16924	5	73427153	73512831	-
3891	1433655_at	Region	None	None	7	104666413	104668232	-
3892	1420841_at	Region	Ptprf	19268	4	117168706	117236351	-
3893	1438470_at	Region	Socs2	216233	10	95388906	95391737	-
3894	1437784_at	Region	None	None	4	13817906	13819589	+
3895	1435434_at	Region	Braf	109880	6	39749542	39860924	-
3896	1417607_at	Region	Cox6a2	12862	7	122255424	122256162	-
3897	1449362_a_at	Region	Mink1	50932	11	70288548	70340136	+
3898	1437318_at	Region	Pak3	18481	X	137105791	137231857	+
3899	1437320_s_at	Region	Xpa	22590	4	46091161	46112233	-
3900	1450950_at	Region	Cspg6	13006	19	53166173	53210193	+
3901	1430316_at	Region	4930579E17Rik	75847	12	32988680	33110743	+
3902	1448936_at	Region	Stx12	100226	4	131814963	131845357	-
3903	1438800_at	Region	Nagk	56174	6	84139275	84146789	+
3904	1440356_at	Region	None	None	8	94104071	94104851	-
3905	1417042_at	Region	Slc37a4	14385	9	44387031	44391821	+
3906	1417167_at	Region	Exosc5	27998	7	20823620	20832720	+
3907	1437205_at	Region	None	None	8	122775522	122778505	+
3908	1457842_at	Region	None	None	4	34966524	34967050	-
3909	1425650_at	Region	Tle4	21888	19	13671934	13821845	-
3910	1436595_at	Region	Rbm34	52202	8	126234922	126258829	-
3911	1448609_at	Region	Tst	22117	15	78450841	78457139	-
3912	1455197_at	Region	Rnd1	223881	15	98726495	98734751	-
3913	1434633_at	Region	AW558298	106441	16	3752907	3754307	-
3914	1436447_at	Region	A630026N12Rik	320961	2	126183781	126184348	-
3915	1457649_x_at	Region	AI848149	102944	X	149195975	149196263	-
3916	1436978_at	Region	None	None	11	59058470	59059184	+
3917	1416318_at	Region	Serpinb1a	66222	13	32321748	32330807	-

			Pcdhgb1 /// Pcdhgb2 /// Pcdhgb4 /// Pcdhgb5 /// Pcdhgb6 /// Pcdhgb7 /// Pcdhgb8 /// Pcdhgc3 /// Pcdhgc4 /// Pcdhgc5 /// Pcdhga1 /// Pcdhga2 /// Pcdhga3 /// Pcdhga4 /// Pcdhga5 /// Pcdhga6 /// Pcdhga7 /// Pcdhga8 /// Pcdhga9 /// Pcdhga10 ///					
3918	1423628_s_at	Region	Pcdhga11 /// P	93699	18	38043397	38065590	+
3919	1457747_at	Region	9330109K16Rik	77549	8	55185601	55186176	+
3920	1428714_at	Region	Pgrmc2	70804	3	40493634	40529585	-
3921	1424209_at	Region	Rarsl	109093	4	34754045	34799231	+
3922	1451519_at	Region	Rnf2	19821	1	151325156	151356573	-
3923	1460712_s_at	Region	Ap1g1	11765	8	109076386	109159034	+
3924	1428345_at	Region	4932443D16Rik	74411	19	28216517	28219402	+
3925	1440091_at	Region	Mrg1	17536	2	115376917	115578960	-
3926	1419657_a_at	Region	Slc25a36	192287	9	96976369	97010399	-
3927	1433485_x_at	Region	Gpr56	14766	8	94268775	94298363	+
3928	1438570_at	Region	None	None	17	49370498	49371323	+
3929	1416386_a_at	Region	M6pr	17113	6	122969976	122978644	+
3930	1430368_s_at	Region	1700019D03Rik	67080	1	53227975	53255688	-
3931	1439874_at	Region	9330102E08Rik	330427	6	128859876	128873978	-
3932	1434790_a_at	Region	Lta4h	16993	10	93425960	93457364	+
3933	1441732_at	Region	Sdccag33	110796	18	84180139	84254394	-
3934	1443983_at	Region	Sorbs1	20411	19	39839082	40058071	-
3935	1417508_at	Region	Rnf19	30945	15	36241202	36267284	-
3936	1436094_at	Region	---	381677	5	136011085	136048860	+
3937	1419112_at	Region	Nlk	18099	11	78292827	78423032	-
3938	1437194_x_at	Region	1200011O22Rik	71735	5	135136559	135149493	-
3939	1423572_at	Region	Bcl2l2	12050	14	49400262	49405071	+
3940	1423910_at	Region	Centg3	213990	5	22916074	22965944	+
3941	1439922_at	Region	1190002C06Rik	73137	18	57571393	57606175	+
3942	1444020_at	Region	Cspg3	13004	8	69246056	69273784	-
3943	1456908_at	Region	BC023202	407816	12	21514538	21515893	+
3944	1416106_at	Region	1110001A12Rik	100087	4	107806762	107808317	+
3945	1425477_x_at	Region	H2-Ab1 /// Rmcs1	114887	17	31964496	31970584	+
3946	1459906_at	Region	Dgkh	380921	14	72922487	72981706	-
3947	1415920_at	Region	Cstf2t	83410	19	30334913	30338502	+
3948	1417781_at	Region	Lass4	67260	8	3848044	3880287	+
3949	1425755_at	Region	Mtcp1 /// LOC547409	17763	X	70065501	70077198	-
3950	1435084_at	Region	C730049O14Rik	320117	18	21200498	21202284	+
3951	1447342_at	Region	None	None	NONE	NONE	NONE	NONE
3952	1440928_at	Region	D630037F22Rik	319841	10	56264598	56265531	-
3953	1454011_a_at	Region	Rpa2	19891	4	131729260	131739382	+
3954	1436575_at	Region	Grin3a	242443	4	49577485	49578618	-
3955	1434839_s_at	Region	Tbl1xr1	81004	3	21419728	21554694	+
3956	1449101_at	Region	Ebf2	13592	14	61760384	61959861	+
3957	1420610_at	Region	Prkacb	18749	3	145706130	145789479	-
3958	1448781_at	Region	Nab1	17936	1	52758832	52802132	-
3959	1425611_a_at	Region	Cutl1	13047	5	135261627	135499343	-
3960	1416593_at	Region	Glrx1	93692	13	71896546	71906783	+
3961	1419824_a_at	Region	A230062G08Rik	231326	5	76120378	76149959	-

3962	1427484_at	Region	Eml5	319670	12	94214380	94328523	-
3963	1418390_at	Region	Phf21a	192285	2	91918732	92065593	+
3964	1453481_at	Region	Zdhhc2	70546	8	39367458	39428485	+
3965	1418692_at	Region	Rab8a	17274	8	71319426	71338872	+
3966	1441187_at	Region	E330037M01Rik	319775	5	24438034	24473535	+
3967	1445277_at	Region	None	None	6	99803878	99804567	-
3968	1430101_at	Region	6330406O05Rik	70716	7	50985942	50987241	-
3969	1452882_at	Region	Pgrmc2	70804	3	40493634	40529585	-
3970	1454230_a_at	Region	Slc25a27	74011	17	41143342	41168531	-
3971	1430353_at	Region	Glis3	226075	19	27503039	27780224	-
3972	1455160_at	Region	2610203C20Rik	70455	9	41545294	41546567	+
3973	1435371_x_at	Region	Ces3	104158	8	92449775	92481484	-
3974	1439518_at	Region	Mmrn2	105450	14	32512110	32539866	+
3975	1448262_at	Region	Psmb2	26445	4	125704779	125736819	+
3976	1416498_at	Region	Ppic	19038	18	53624470	53636136	-
3977	1455556_at	Region	Notch2	18129	3	97499747	97633658	+
3978	1429250_at	Region	Dnchc2	110350	9	6893083	6980932	-
3979	1434682_at	Region	6430601A21Rik	228491	2	113707303	113715272	-
3980	1425082_s_at	Region	9030416H16Rik	71521	5	64424699	64449836	-
3981	1456233_at	Region	None	None	12	17802777	17803445	+
3982	1458866_at	Region	A930001M12Rik	320500	4	40611031	40614504	+
3983	1423727_at	Region	Cnih	12793	14	41855551	41868219	-
3984	1418442_at	Region	Xpo1	103573	11	23150860	23192321	+
3985	1436295_at	Region	Hcrtr1	230777	4	129157330	129166275	-
3986	1457497_at	Region	Syt1	20979	10	108385089	108577499	-
3987	1425116_a_at	Region	Spnb4	80297	7	22732993	22822146	-
3988	1421195_at	Region	Cckar	12425	5	52508626	52518241	-
3989	1427382_a_at	Region	Suv39h1	20937	X	6300242	6313526	-
3990	1451465_at	Region	Ubl7	69459	9	58024910	58043894	+
3991	1419742_at	Region	1700037H04Rik	67326	2	130660178	130673873	-
3992	1460510_a_at	Region	1500041J02Rik	67876	1	55354394	55373108	+
3993	1441547_at	Region	Nfia	18027	4	96752972	97087465	+
3994	1439259_x_at	Region	Abhd4	105501	14	48779225	48789095	+
3995	1429390_at	Region	Acpl2	235534	9	96722701	96788780	-
3996	1435002_at	Region	zfp507	243924	7	30935188	31085431	-
3997	1447791_s_at	Region	Gna14	14675	19	15659862	15834920	+
3998	1438142_s_at	Region	1700021K14Rik	69398	9	108063750	108067667	+
3999	1417058_a_at	Region	Krtcap2	66059	3	89002910	89006194	+
4000	1417184_s_at	Region	Hbb /// Hbb-b1 /// Hbb-b2	15127	7	38286	50332	+
4001	1427504_s_at	Region	Sfrs2	20382	11	116671000	116674175	-
4002	1438402_at	Region	9630050M13Rik	269233	2	3035684	3145036	+
4003	1430978_at	Region	Rps25	75617	9	44396569	44399261	+
4004	1425066_a_at	Region	1110061O04Rik	97287	6	113800971	113844666	+
4005	1438625_s_at	Region	Ndn /// Pctk1	17984	7	56122085	56123665	+
4006	1435045_s_at	Region	None	None	15	89516283	89517342	+
4007	1426797_at	Region	2700094F01Rik	72649	6	30528276	30556749	-
4008	1455473_at	Region	Usp13	72607	3	32345581	32346253	+
4009	1457111_at	Region	AA415038	103570	11	104240459	104241135	-
4010	1460168_at	Region	Slbp	20492	5	32128914	32141162	-
4011	1448565_at	Region	Ppp1r11	76497	17	34708791	34711487	-
4012	1426724_at	Region	Cnn3	71994	3	120205773	120237098	+
4013	1451512_s_at	Region	Hibch	227095	1	53147894	53223708	+
4014	1422665_a_at	Region	Pcmt1	18537	10	7494777	7527640	-
4015	1436043_at	Region	Scn7a	20272	2	66530712	66640318	-
4016	1453052_at	Region	3110005G23Rik	73067	8	64061460	64083308	+
4017	1437889_x_at	Region	Bgn	12111	X	68144299	68156586	+
4018	1426831_at	Region	Ahcyl1	229709	3	107460335	107492339	-
4019	1450755_at	Region	Pafah1b2	18475	9	45981441	45998071	-
4020	1449472_at	Region	Gpr12	14738	5	145473843	145476932	-
4021	1446071_at	Region	Steap2	74051	5	5676943	5699766	-
4022	1423492_at	Region	Mrpl45	67036	11	97136943	97151007	+
4023	1416260_a_at	Region	Snx1	56440	9	66211465	66248213	-
4024	1448208_at	Region	Smad1	17125	8	78552485	78613558	-
4025	1449709_s_at	Region	Ate1	11907	7	124514765	124642811	-

4026	1428577_at	Region	Ppfia4	68507	1	134149042	134182786	-
4027	1417316_at	Region	Them2	66834	13	24297832	24311366	-
4028	1434696_at	Region	BC037708	245866	2	162474441	162503104	+
4029	1419700_a_at	Region	Prom1	19126	5	42750678	42858751	-
4030	1426314_at	Region	Ednrb	13618	14	98359614	98388688	-
4031	1457583_at	Region	A530083M17Rik	320505	3	152407159	152407607	-
4032	1447720_x_at	Region	Prkaca	18747	8	83233159	83256577	+
4033	1448618_at	Region	Mvp	78388	7	121036597	121064325	-
4034	1439899_at	Region	Galnt13	271786	2	54361926	55038861	+
4035	1422593_at	Region	Ap3s1	11777	18	46963683	47012375	+
4036	1417252_at	Region	Nt5c	50773	11	115311518	115312907	-
4037	1456438_x_at	Region	Rpn1	103963	6	88518156	88540499	+
4038	1422703_at	Region	Gyk	14933	X	80362593	80437339	-
4039	1419552_at	Region	Echdc1	52665	10	29344397	29378616	+
4040	1437943_s_at	Region	Mea1	17256	17	44191851	44193708	+
4041	1415962_at	Region	Eif3s3	68135	15	51783792	51862690	-
4042	1422751_at	Region	Tle1	21885	4	71208335	71292050	-
4043	1453846_at	Region	2810013C04Rik	69959	5	23056650	23059765	+
4044	1458967_at	Region	None	None	NONE	NONE	NONE	NONE
4045	1455372_at	Region	Cpeb3	208922	19	36366605	36550135	-
4046	1438416_at	Region	Thrap5	216154	10	80017343	80031526	-
4047	1451421_a_at	Region	MGI:1913299	66049	16	4678527	4683301	-
4048	1423440_at	Region	1110001A07Rik	66140	11	86837253	86850966	+
4049	1424358_at	Region	Ube2e2	218793	14	17092347	17412397	-
4050	1432282_a_at	Region	2010305C02Rik	380712	11	75193737	75196303	+
4051	1435738_x_at	Region	Serf2	378702	2	120963266	120964906	+
4052	1455684_at	Region	Map3k9	338372	12	78588739	78641336	-
4053	1420381_a_at	Region	Rpl31	114641	1	39664836	39668824	+
4054	1433903_at	Region	AU021838	328099	12	53972281	53973651	+
4055	1451448_a_at	Region	1110005A03Rik	74319	11	116664693	116670827	+
4056	1421263_at	Region	Gabra3	14396	X	67094472	67316841	-
4057	1434702_at	Region	2600009E05Rik	77006	2	130167812	130178512	-
4058	1427456_at	Region	Wdfy3	72145	5	100857137	101091457	-
4059	1418183_a_at	Region	Pscd1	19157	11	117985268	118069653	-
4060	1437016_x_at	Region	Rap2c	72065	X	45525090	45538760	-
4061	1416861_at	Region	Stam	20844	2	14000384	14074610	+
4062	1428617_at	Region	1700129L13Rik	67933	10	82611323	82629097	+
4063	1436161_at	Region	Aprin	100710	5	149615145	149749995	+
4064	1451535_at	Region	Il31ra	218624	13	108915224	108959127	-
4065	1449815_a_at	Region	Ssbp2	66970	13	87661341	87840293	+
4066	1447852_x_at	Region	2900002H16Rik	75695	5	123664423	123702734	-
4067	1447807_s_at	Region	Plekhh1	211945	12	75867965	75938050	+
4068	1439204_at	Region	Scn3a	20269	2	65315942	65422882	-
4069	1438321_x_at	Region	4930504E06Rik	75007	3	94770775	94783992	+
4070	1441793_at	Region	Ppp1r11	76497	17	34708791	34711487	-
4071	1458375_at	Region	LOC212390	212390	4	24748644	24986601	-
4072	1428257_s_at	Region	Dncl2a	67068	2	154693509	154707210	+
4073	1423308_at	Region	Tgoln1	22134	6	72943992	72950517	-
4074	1440847_at	Region	Mtss1	211401	15	58954404	59092928	-
4075	1447723_at	Region	None	None	NONE	NONE	NONE	NONE
4076	1448985_at	Region	Dusp22	105352	13	30139718	30190847	+
4077	1424270_at	Region	Dcamkl1	13175	3	54876964	55171014	+
4078	1440393_at	Region	Odz2	23964	11	35801678	36738275	-
4079	1452307_at	Region	Cables2	252966	2	179976719	179990423	-
4080	1452497_a_at	Region	Nfatc3	18021	8	105355731	105424292	+
4081	1440092_at	Region	Ext1	14042	15	53068219	53345629	-
4082	1433603_at	Region	Ndufs6	407785	13	69375417	69384002	-
4083	1448928_at	Region	Hdac6	15185	X	6169066	6186184	-
4084	1448537_at	Region	Ttc1	66827	11	43482843	43500810	-
4085	1417340_at	Region	Txnl2	30926	7	131806311	131837120	+
4086	1450146_at	Region	Freq	14299	2	31178085	31227634	+
4087	1448722_s_at	Region	6330579B17Rik	106564	4	118377438	118381296	-
4088	1452271_at	Region	Xpr1	19775	1	155171522	155308066	-
4089	1424227_at	Region	Polr3h	78929	15	81965747	81976930	-
4090	1427562_a_at	Region	Prkca	18750	11	107759337	108165017	-

4091	1448825_at	Region	Pdk2	18604	11	94847345	94862402	-
4092	1435319_at	Region	1500005N04Rik	76500	9	108842504	108852864	+
4093	1427091_at	Region	AI481105	98999	2	166492526	166519674	-
4094	1435701_at	Region	None	None	16	84554007	84554735	-
4095	1428837_at	Region	Klhl14	225266	18	21773688	21873554	-
4096	1449373_at	Region	Dnajc3	19107	14	113495773	113540797	+
4097	1448475_at	Region	Olfml3	99543	3	103162790	103165339	-
4098	1450654_a_at	Region	Dhdds	67422	4	132929956	132961763	-
4099	1433909_at	Region	None	None	7	112256570	112257034	-
4100	1427567_a_at	Region	Tpm3	59069	3	89883112	89911022	+
4101	1441372_at	Region	5930405F01Rik	320550	18	68179047	68179854	+
4102	1455297_at	Region	Spin2	278240	X	147366689	147368636	+
4103	1419443_at	Region	Sap18	20220	14	52333126	52339849	+
4104	1452180_at	Region	Phf17	269424	3	41010564	41046427	+
4105	1425027_s_at	Region	SFT2 domain containing 2	108735	1	165097432	165117255	-
4106	1459388_at	Region	C030036D22Rik	77607	18	72458844	72459491	-
4107	1422479_at	Region	Acas2	60525	2	154974980	155019680	+
4108	1428830_at	Region	Atm	11920	9	53504628	53601833	-
4109	1417722_at	Region	Pgls	66171	8	70741855	70745938	+
4110	1425977_a_at	Region	Slk	20874	19	47131319	47193755	+
4111	1453240_a_at	Region	Gcap14	72972	14	35007246	35063441	-
4112	1452857_at	Region	MGI:2675296	233490	7	84493551	84494754	+
4113	1448686_at	Region	Il16	16170	7	77499493	77591920	-
4114	1434435_s_at	Region	Cox17	12856	16	37164821	37170611	+
4115	1437211_x_at	Region	Elovl5	68801	9	78084592	78151807	+
4116	1425543_s_at	Region	Plekha5	109135	6	141327040	141388972	+
4117	1436403_at	Region	BC025575	217219	11	102258068	102268730	-
4118	1438871_at	Region	Son	20658	16	90805044	90836353	+
4119	1457003_at	Region	AW490526	101362	6	136491096	136491549	-
4120	1447404_at	Region	None	None	18	77684040	77684459	-
4121	1438624_x_at	Region	Hs3st2	195646	7	115360837	115377332	+
4122	1428361_x_at	Region	Hba-a1	15122	11	32178468	32179287	+
4123	1420632_a_at	Region	Bscl2	14705	19	8036050	8045214	+
4124	1421580_at	Region	Cntnap1	53321	11	100997204	101010934	+
4125	1450998_at	Region	Zfp110	65020	7	10828742	10844258	+
4126	1460741_x_at	Region	D17Wsu92e	224647	17	25549195	25616460	-
4127	1419400_at	Region	Mttp	17777	3	136979815	137020253	-
4128	1423925_at	Region	Dhx16	69192	17	33598972	33611823	+
4129	1425812_a_at	Region	Cacna1b	12287	2	24538537	24695314	-
4130	1416229_at	Region	Rfk	54391	19	16618183	16625435	+
4131	1460638_at	Region	Yars	107271	4	128217064	128246720	+
4132	1451828_a_at	Region	Acsl4	50790	X	135758036	135830547	-
4133	1423430_at	Region	Mybbp1a	18432	11	72167036	72177208	+
4134	1416993_at	Region	Cog4	102339	8	110144769	110179947	+
4135	1452826_s_at	Region	Fbxl20	72194	11	97909793	97970681	-
4136	1452413_at	Region	C230081A13Rik	244895	9	56336855	56527185	-
4137	1451307_at	Region	Mrpl14	68463	17	43197098	43209297	+
4138	1426257_a_at	Region	Sars1	20226	3	108222354	108240911	-
4139	1416950_at	Region	Tnfaip8	106869	18	50268616	50306875	+
4140	1456138_at	Region	E130115E03Rik	320343	2	50060532	50125726	+
4141	1454620_x_at	Region	Rps6 /// LOC434404 /// LOC545221 /// LOC545640	20104	4	85840854	85843549	-
4142	1454793_x_at	Region	Ddx5	13207	11	106602635	106609565	-
4143	1455623_at	Region	A930031D07Rik	213006	1	131888602	131919751	-
4144	1448637_at	Region	Med25	75613	7	38954595	38967682	-
4145	1423832_at	Region	Prkag2	108099	5	23326651	23569979	-
4146	1448663_s_at	Region	Mvd	192156	8	121815191	121824977	-
4147	1425114_at	Region	Rbbp6	19647	7	116848797	116880431	+
4148	1418700_at	Region	Lias	79464	5	64193326	64211036	+
4149	1422180_a_at	Region	Mcpt6	17229	17	23172190	23174949	+
4150	1448542_at	Region	Bccip	66165	7	128076989	128088796	+
4151	1426354_at	Region	Bap1	104416	14	29383425	29391768	+

4152	1449553_at	Region	2610200G18Rik	67149	NONE	NONE	NONE	NONE
4153	1452599_s_at	Region	AI413582	106672	17	25359687	25361618	-
4154	1423736_a_at	Region	Dym	69190	18	75253957	75522151	+
4155	1451119_a_at	Region	Fbln1	14114	15	85254610	85334255	+
4156	1423207_at	Region	0610041E09Rik	66074	13	86207541	86225129	+
4157	1423801_a_at	Region	Aprt	11821	8	121956224	121958447	-
4158	1426007_a_at	Region	Ubxd3	212190	4	137599799	137618428	-
4159	1426761_at	Region	Aof2	99982	4	135431814	135483967	-
4160	1453791_at	Region	2810449C10Rik	72758	13	79828320	79831546	+
4161	1416858_a_at	Region	Fkbp3	30795	12	61840122	61851631	-
4162	1423188_a_at	Region	6720456B07Rik	101314	6	114172603	114184782	+
4163	1443205_at	Region	D5Buc30e	27785	5	70922666	70923316	+
4164	1416207_at	Region	Taz	66826	X	68943417	68950803	+
4165	1418814_s_at	Region	Ndufa12	66414	10	94172393	94194766	+
4166	1425255_s_at	Region	Hnrpll	72692	17	77846881	77879599	-
4167	1424449_at	Region	Frs3	107971	17	45213667	45222746	+
4168	1417195_at	Region	D8Ertd594e	52357	8	46797192	46960652	-
4169	1428512_at	Region	Bhlhb9	70237	X	129433841	129436014	+
4170	1432096_at	Region	Snrpn	20646	7	53759592	53781697	-
4171	1447559_at	Region	AI834762	98429	1	136105808	136106129	-
4172	1460262_a_at	Region	1700037H04Rik	67326	2	130660178	130673873	-
4173	1450694_at	Region	Fkbp2	14227	19	6691587	6694278	-
4174	1426645_at	Region	Hspca	15519	12	106166269	106171628	-
4175	1422809_at	Region	Rims2	116838	15	39098033	39582362	+
4176	1429766_at	Region	2810013P06Rik	67206	8	122423784	122425711	+
4177	1422768_at	Region	Syncrip	56403	9	88814754	88842894	-
4178	1426660_x_at	Region	Rpl23a	268449	11	77906594	77909202	-
4179	1451665_a_at	Region	Ap4s1	11782	12	48442857	48490868	+
4180	1436436_at	Region	Cnih4	98417	1	181121921	181122681	+
4181	1434025_at	Region	None	None	14	93838296	93840097	+
4182	1427172_at	Region	Ofd1	237222	X	159990341	160039960	-
4183	1455228_at	Region	Whsc1	107823	5	32363965	32365554	+
4184	1431322_at	Region	Igsf3	78908	3	100804224	100886780	+
4185	1423234_at	Region	Psmd5	66998	2	34784262	34803092	-
4186	1431309_at	Region	4833412E22Rik	330010	4	154536681	154551706	-
4187	1423304_a_at	Region	Rnf111	93836	9	70563535	70644248	-
4188	1452060_a_at	Region	Limk2	16886	11	3239044	3303961	-
4189	1425510_at	Region	Mark1	226778	1	184396055	184497931	-
4190	1432269_a_at	Region	Sh3kbp1	58194	X	153308006	153573572	+
4191	1453419_at	Region	2900078C09Rik	73053	9	99285879	99287285	-
4192	1425517_s_at	Region	Ogt	108155	X	96241425	96285710	+
4193	1416908_s_at	Region	Tsn	22099	1	118048034	118060649	-
4194	1421926_at	Region	Mapk11	19094	15	89194712	89201863	-
4195	1441467_at	Region	Tm4sf9	56224	3	137631430	137793659	+
4196	1450971_at	Region	Gadd45b	17873	10	81061547	81063660	+
4197	1424732_s_at	Region	3110005G23Rik	73067	8	64061460	64083308	+
4198	1436195_at	Region	BC046404	192976	11	62330534	62374173	-
4199	1416294_at	Region	Scamp3	24045	3	88934051	88939238	+
4200	1454799_at	Region	4933408F15	231510	5	99866783	99918835	+
4201	1453492_at	Region	5330408M12Rik	329795	4	11966610	12014922	-
4202	1440819_s_at	Region	BC059842	230676	4	117321648	117333952	-
4203	1451453_at	Region	Dapk2	13143	9	66281592	66395569	+
4204	1434883_at	Region	Mtdh	67154	15	34082454	34141052	+
4205	1453267_at	Region	Atbf1	11906	8	108012331	108254925	+
4206	1423706_a_at	Region	Pgd	110208	4	147642356	147659030	-
4207	1426380_at	Region	Eif4b	75705	15	102141346	102156533	+
4208	1437035_x_at	Region	Rnf14	56736	18	38520411	38541525	+
4209	1426285_at	Region	Lama2	16773	10	26981182	27483486	-
4210	1453171_s_at	Region	Ppm1a	19042	12	69630561	69641441	+
4211	1434781_at	Region	None	None	4	140641436	140642017	-
4212	1418929_at	Region	Esrrbl1	73916	16	48540632	48606670	+
4213	1436926_at	Region	Esrrb	26380	12	83231310	83394599	+
4214	1444785_at	Region	Ski	20481	4	153649703	153714882	-
4215	1420833_at	Region	Vamp2	22318	11	68814186	68818039	+
4216	1450409_a_at	Region	4930570C03Rik	67739	15	97860010	97868333	+

4217	1420655_at	Region	Adpn	116939	15	84216436	84238113	+
4218	1439244_a_at	Region	Tnrc6a	233833	7	117180427	117250357	+
4219	1460033_at	Region	C030002C11Rik	320977	1	194777203	194778275	+
4220	1417744_a_at	Region	Ralb	64143	1	119221743	119256062	-
4221	1451956_a_at	Region	Oprs1	18391	4	41877189	41880006	-
4222	1433892_at	Region	Spag5	54141	11	78027295	78048113	+
4223	1445241_at	Region	Rab11fip4	268451	11	79316871	79419671	+
4224	1433635_at	Region	Wdr18	216156	10	80082774	80090742	+
4225	1433338_at	Region	6720460K10Rik	77736	2	59539131	59540150	+
4226	1451250_at	Region	Gprin1	26913	13	53347815	53351668	-
4227	1422466_at	Region	Nxn	18230	11	75983616	76124799	-
4228	1449536_at	Region	Kcnn1	84036	8	69998871	70011298	-
4229	1420964_at	Region	Enc1	13803	13	93429732	93441676	+
4230	1416517_at	Region	MGI:1354723	50767	8	407	23399	-
4231	1451265_at	Region	2310061I09Rik	69668	1	34706179	34709175	-
4232	1451813_at	Region	Oprk1	18387	1	5551006	5565381	+
4233	1434036_at	Region	Mtss1	211401	15	58954404	59092928	-
4234	1444051_at	Region	1700019D03Rik	67080	1	53227975	53255688	-
4235	1427573_at	Region	Chic1	12212	X	97957921	97991407	+
4236	1451650_at	Region	5330420D20Rik	70503	10	40708813	40729005	+
4237	1425106_a_at	Region	Wars	22375	12	104331027	104364190	-
4238	1446473_at	Region	4732418C07Rik	230648	4	114696978	114736232	+
4239	1457702_at	Region	Gpr12	14738	5	145473843	145476932	-
4240	1451096_at	Region	Ndufs2	226646	1	171163699	171175951	-
4241	1458919_at	Region	Mkln1	27418	6	31475825	31586464	+
4242	1448391_at	Region	Rab9	56382	X	160056664	160079101	-
4243	1427213_at	Region	Pfkfb1	18639	X	144168918	144178273	+
4244	1434862_at	Region	Fut2	14344	7	39731738	39749568	-
4245	1441987_at	Region	Mbd5	109241	2	49178095	49249231	+
4246	1429955_at	Region	5031434O11Rik	75980	3	51194435	51199082	+
4247	1435536_at	Region	Rsc1a1	69994	4	140564825	140604658	-
4248	1434466_at	Region	Atcay	16467	10	81340453	81366832	-
4249	1440715_s_at	Region	D11Ertd497e	52626	11	51720504	51730177	+
4250	1435627_x_at	Region	Mlp	17357	4	128540757	128543088	+
4251	1436772_at	Region	Gria4	14802	9	4328268	4706535	-
4252	1448439_at	Region	D17Wsu104e	28106	17	53813582	53820961	-
4253	1452504_s_at	Region	Ctbs	74245	3	145427044	145442322	+
4254	1454829_at	Region	Rundc1	217201	11	101246172	101256547	+
4255	1448317_at	Region	2810021O14Rik	66309	5	36766600	36775843	+
4256	1452445_at	Region	Slc41a2	338365	10	83119063	83225741	-
4257	1453733_a_at	Region	4933434E20Rik	99650	3	89863255	89872865	+
4258	1452992_at	Region	Cdc26	66440	4	61485774	61495742	-
4259	1433782_at	Region	Cldn12	64945	5	5511114	5520878	-
4260	1419754_at	Region	Myo5a	17918	9	75209875	75357058	+
4261	1426128_a_at	Region	Kcnq2	16536	2	180792537	180852183	-
4262	1451974_at	Region	Osbpl2	228983	2	179836331	179879418	+
4263	1428412_at	Region	MGI:1914262	107358	19	40758164	40808343	-
4264	1431080_at	Region	1700021K10Rik	66331	8	56815380	56835824	-
4265	1427379_at	Region	Nte	50767	8	407	23399	-
4266	1416184_s_at	Region	Hmga1	15361	17	25352817	25359581	+
4267	1456505_at	Region	Braf	109880	6	39749542	39860924	-
4268	1434934_at	Region	Atpaf1	230649	4	114743890	114771219	+
4269	1436105_at	Region	2310015A05Rik	72315	16	16417438	16421706	+
4270	1433615_at	Region	B930062P21Rik	320709	15	94697888	95164061	+
4271	1434467_at	Region	Atcay	16467	10	81340453	81366832	-
4272	1455446_x_at	Region	Acadsb	66885	7	125726405	125763064	+
4273	1439077_at	Region	None	None	NONE	NONE	NONE	NONE
4274	1426812_a_at	Region	9130404D14Rik	227737	2	32808389	32857411	+
4275	1452061_s_at	Region	Strbp	20744	2	37415390	37579381	-
4276	1438218_at	Region	Zbtb24	268294	10	41552220	41567398	+
4277	1443045_at	Region	5930412E23Rik	77065	1	191312045	191358940	+
4278	1455355_at	Region	6030408C04Rik	217558	12	48120507	48125429	+
4279	1435020_at	Region	Sdccag1	66244	12	66143332	66188963	-
4280	1448591_at	Region	Ctss	13040	3	95014675	95044230	+
4281	1452977_at	Region	Zhx3	320799	2	160227416	160329959	-

4282	1454925_x_at	Region	Mdh1	17449	11	21451580	21466622	-
4283	1441807_s_at	Region	None	None	12	3902172	3903495	-
4284	1428262_s_at	Region	Hnrpa3 /// LOC547321	229279	2	75357026	75367156	+
4285	1434153_at	Region	Shb	230126	4	45339198	45448023	-
4286	1439571_at	Region	E230008J23Rik	319683	19	15836295	15837390	-
4287	1453111_a_at	Region	D11Ertd333e	68066	11	102224234	102228559	-
4288	1437276_at	Region	AA589481	102423	9	44284528	44294482	-
4289	1436662_at	Region	Sorcs1	58178	19	49701709	50229374	-
4290	1441511_at	Region	Kcnt1	227632	2	25849756	25850436	+
4291	1448326_a_at	Region	Crabp1	12903	9	54882658	54891162	+
4292	1448104_at	Region	Aldh6a1	104776	12	81299064	81319297	-
4293	1423621_a_at	Region	Slc33a1	11416	3	63610264	63632629	-
4294	1427039_at	Region	Epn1	13854	7	4330112	4348215	+
4295	1455105_at	Region	Ptpn12	19248	5	19439215	19508367	-
4296	1453487_at	Region	Dhdh	71755	7	39554426	39570488	-
4297	1424640_at	Region	Arl10b	68724	1	134997097	135006531	+
4298	1440031_at	Region	None	None	7	13826736	13836535	+
4299	1427912_at	Region	Cbr3	109857	16	92848492	92856369	+
4300	1417388_at	Region	Bex2	12069	X	129611505	129613049	-
4301	1437167_at	Region	Tmod2	50876	9	75710299	75748747	-
4302	1420471_at	Region	Hcrt	15171	11	100582780	100584018	-
4303	1428750_at	Region	Cdc42ep2	104252	19	5706351	5707785	-
4304	1445874_at	Region	None	None	19	18319561	18320316	-
4305	1439648_at	Region	Anln	68743	9	22222408	22279584	-
4306	1447064_at	Region	D130059P03Rik	320538	6	38570159	38638809	+
4307	1455135_at	Region	2610014I16Rik	66300	7	21060158	21060854	-
4308	1423258_at	Region	Syt9	60510	7	101319931	101498655	+
4309	1437639_at	Region	3321401G04Rik	77574	6	42816018	42836530	-
4310	1428920_at	Region	Hmg20a	66867	9	56527993	56606058	+
4311	1458430_at	Region	C87436	232196	6	86827909	86902412	+
4312	1421833_at	Region	Pip5k1a	18719	19	23533932	23683715	-
4313	1435602_at	Region	Sephs2	20768	7	121321610	121323787	-
4314	1435146_s_at	Region	Igsf4d	239857	16	65672212	65964483	-
4315	1448270_at	Region	Ddx21	56200	10	62546632	62565575	-
4316	1424050_s_at	Region	Fgfr1	14182	8	24257087	24299020	+
4317	1456686_at	Region	Zfhx1a	21417	18	5709786	5817483	+
4318	1456038_at	Region	Fbxl4	269514	4	22426936	22503417	+
4319	1457549_at	Region	None	None	17	45063158	45063794	+
4320	1422208_a_at	Region	Gnb5	14697	9	75448974	75483250	+
4321	1416085_s_at	Region	Za20d2	22682	19	20510205	20520216	+
4322	1452150_at	Region	AU040320	100317	4	125780915	125881449	+
4323	1424338_at	Region	Slc6a13	14412	6	121732494	121769696	+
4324	1440657_at	Region	None	None	6	103030931	103031586	-
4325	1424920_at	Region	Slc37a3	72144	6	39472104	39502599	-
4326	1453023_at	Region	Ankhd1	108857	18	36868943	36889684	+
4327	1451652_a_at	Region	5033428A16Rik	382030	8	87404151	87419831	+
4328	1424463_at	Region	2210010L05Rik	98682	1	52961158	53031019	-
4329	1418228_at	Region	Hirip5	56748	6	87443967	87462532	+
4330	1428445_at	Region	Yif1b	77254	7	24649958	24659140	+
4331	1428761_a_at	Region	Snapc3	77634	4	82403941	82453789	+
4332	1437454_a_at	Region	2310042M24Rik /// LOC433144	433144	17	84930790	84937034	+
4333	1445579_at	Region	Ppp1r1a	58200	15	103593360	103601042	-
4334	1424696_at	Region	Gpr89	67549	3	96357516	96391167	-
4335	1421029_a_at	Region	Hbs1l	56422	10	21249085	21311233	+
4336	1446338_at	Region	5730522E02Rik	70626	11	25778962	25779638	-
4337	1455271_at	Region	1810020C19Rik	69113	2	93685235	93715406	-
4338	1460624_at	Region	6330564D18Rik	109045	1	130572402	130573796	+
4339	1435794_at	Region	BC050254	269630	5	21896163	21897229	-
4340	1450983_at	Region	Akap8	56399	17	30115536	30133013	-
4341	1437785_at	Region	Adamts9	101401	6	93379900	93410347	-
4342	1439651_at	Region	Lmo4	16911	3	143160075	143176516	-
4343	1416960_at	Region	B3gat3	72727	19	8116940	8123783	+
4344	1457398_at	Region	Gdi3	14569	13	3422528	3450201	+

4345	1442998_at	Region	Ctps2	55936	X	156501010	156631724	+
4346	1424923_at	Region	Serpina3g	20715	12	99658720	99686078	+
4347	1448113_at	Region	Stmn1	16765	4	133429314	133434742	+
4348	1456247_x_at	Region	Plp2	18824	X	5906886	5910272	-
4349	1434991_at	Region	Fbxw17	109082	13	25133	27788	+
4350	1423252_at	Region	Hdgfrp3	29877	7	75691975	75742185	-
4351	1431815_a_at	Region	Mms19l	72199	19	41488079	41525508	-
4352	1437713_x_at	Region	1500010J02Rik	68964	11	68761853	68762129	+
4353	1452560_a_at	Region	Nfya	18044	17	45916830	45939727	-
4354	1449414_at	Region	Zfp53	24132	17	19358540	19379787	+
4355	1447510_at	Region	C530014P21Rik	399604	17	60555783	60556126	-
4356	1435458_at	Region	None	None	6	29344996	29346037	+
4357	1450167_at	Region	Rab37	58222	11	114975278	114983321	+
4358	1428897_at	Region	2610029I01Rik	77032	4	21826755	21843550	-
4359	1423367_at	Region	Wnt7a	22421	6	91807033	91860492	-
4360	1455268_at	Region	Zcsl2	105638	14	30212767	30217780	-
4361	1439496_at	Region	4921524J06Rik	77057	17	86507865	86509862	+
4362	1441047_at	Region	9330175B01Rik	106068	15	73605805	73674109	-
4363	1434736_at	Region	Hlf	217082	11	90159652	90211982	-
4364	1459034_at	Region	Wdr19	213081	5	64001542	64062244	+
4365	1460320_at	Region	Becn1	56208	11	101109354	101123354	-
4366	1437324_x_at	Region	Fmod	14264	1	133888996	133898985	+
4367	1433944_at	Region	Hectd2	226098	19	35897450	35963798	+
4368	1437610_x_at	Region	Rps8	20116	4	116112741	116115037	-
4369	1424888_at	Region	38413	224703	17	31384823	31417809	-
4370	1437841_x_at	Region	AI481750	105859	15	81987653	82001658	+
4371	1449274_at	Region	2310038H17Rik	67099	1	64900564	64911252	-
4372	1441438_at	Region	Gpc6	23888	14	111487529	112531234	+
4373	1423372_at	Region	Pole4	66979	6	82990518	82996671	-
4374	1448767_s_at	Region	Gjb1	14618	X	95982922	95986986	+
4375	1431539_at	Region	Samsn1	67742	16	74944941	74995114	-
4376	1424750_at	Region	Zbtb1	268564	12	73226354	73244538	+
4377	1439439_x_at	Region	Eef1d	66656	15	75944869	75959409	-
4378	1416309_at	Region	Nusap1	108907	2	119132566	119163626	+
4379	1455399_at	Region	Cnksr1	194231	4	133188940	133199298	-
4380	1458670_at	Region	None	None	13	8717802	8719106	+
4381	1427910_at	Region	Cst6	73720	19	5133495	5138364	-
4382	1429429_s_at	Region	A030012M09Rik	319263	1	7090301	7142844	+
4383	1446475_at	Region	Mbnl2	105559	14	114846670	115003029	+
4384	1437380_x_at	Region	Pgd	110208	4	147642356	147659030	-
4385	1426545_at	Region	Tnrc6b	213988	15	80975683	80982651	+
4386	1417454_at	Region	Cul4b	72584	X	33054070	33096992	-
4387	1435430_at	Region	Tmem1	216131	10	78296209	78354672	-
4388	1460406_at	Region	AI427122	102502	9	95656189	95718950	-
4389	1450253_a_at	Region	Map3k4	26407	17	10867071	10957848	-
4390	1450040_at	Region	Timp2	21858	11	118122157	118176401	-
4391	1459325_at	Region	None	None	2	83368554	83369145	+
4392	1441693_at	Region	6330442E02Rik	330119	3	72510189	72510739	-
4393	1427159_at	Region	2500001H09Rik	74737	7	86684260	86709889	-
4394	1448114_a_at	Region	Htf9c	15547	16	17019721	17024544	+
4395	1423918_at	Region	4930418P06Rik	76867	1	82675433	82774473	+
4396	1455977_x_at	Region	Klk6 /// Klk5	16612	7	38299769	38303949	+
4397	1425328_at	Region	BC008163	230789	4	131861517	131883410	-
4398	1429201_at	Region	Cyld	74256	8	87990637	88033868	+
4399	1420739_at	Region	Cntn3	18488	6	102641686	102940436	-
4400	1419049_at	Region	Pcnx	54604	12	78720867	78861705	+
4401	1460656_a_at	Region	5630401J11Rik	106489	17	4193	20488	+
4402	1452510_at	Region	Pax7	18509	4	138619342	138714229	-
4403	1415860_at	Region	Kpna2	16647	11	106801935	106820537	-
4404	1422972_s_at	Region	Gcn5l2	14534	11	100526325	100533491	-
4405	1418039_at	Region	Rabl3	67657	16	36359367	36391849	+
4406	1422945_a_at	Region	Kif5c	16574	2	49551476	49706940	+
4407	1434518_at	Region	Phka2	110094	X	154102095	154191127	+
4408	1448963_at	Region	Nfyc	18046	4	119779882	119848111	-
4409	1438025_at	Region	Mtrf1l	108853	10	4687031	4697881	+

4410	1448257_at	Region	Slc29a2	13340	19	4814896	4820754	+
4411	1451269_at	Region	Pdzk11	72621	X	95224267	95227268	-
4412	1454988_s_at	Region	Rab22a	19334	2	173120334	173165145	+
4413	1436995_a_at	Region	Rpl26	19941	11	68627241	68630192	+
4414	1443862_at	Region	None	None	11	93715331	93717156	+
4415	1422559_at	Region	Ube2n	93765	10	95491346	95521553	+
4416	1429180_at	Region	Gmpr2	105446	14	50190251	50197215	+
4417	1434263_at	Region	Fzr1	432479	10	81500707	81501703	+
4418	1452250_a_at	Region	Col6a2	12834	10	76697487	76725338	-
4419	1458508_at	Region	Matr3	17184	18	35795510	35815074	+
4420	1418881_at	Region	Efcbp2	117148	8	118804707	118814783	+
4421	1423723_s_at	Region	Tardbp	230908	4	147104744	147119358	-
4422	1428574_a_at	Region	Chn2	69993	6	54417146	54446135	+
4423	1419405_at	Region	Nmb	68039	7	74704936	74707736	-
4424	1438790_x_at	Region	Tmem41b	233724	7	103825350	103839101	-
4425	1416480_a_at	Region	MGI:1930666	56295	9	121862188	121871251	-
4426	1433644_at	Region	Pomt2	217734	12	83988455	84027132	-
4427	1445181_at	Region	Eml5	319670	12	94214380	94328523	-
4428	1438831_at	Region	Crk7	69131	11	98024401	98067791	+
4429	1450664_at	Region	Gabpa	14390	16	83943193	83971913	+
4430	1436893_a_at	Region	38418	57438	2	60065336	60103785	+
4431	1457983_s_at	Region	BC016198	192174	8	46500251	46519367	+
4432	1459107_at	Region	Kcnh3	16512	15	99282782	99300623	+
4433	1450018_s_at	Region	Slc25a30	67554	14	70104494	70129532	-
4434	1443536_at	Region	9930009M05Rik	319502	3	49953312	49953852	-
4435	1420472_at	Region	Mtpn	14489	6	35603109	35634015	-
4436	1445503_at	Region	None	None	X	55217217	55217714	+
4437	1443129_at	Region	None	None	7	113016410	113016985	-
4438	1438258_at	Region	Vldlr	22359	19	26457498	26489686	+
4439	1447657_s_at	Region	Synpo2l	68760	14	19020730	19029618	-
4440	1456968_at	Region	Actn2	11472	13	12307753	12379584	-
4441	1422157_a_at	Region	Itgb1bp1	16413	12	2114	18547	+
4442	1452280_at	Region	Farp1	223254	14	115842411	115854479	+
4443	1429251_at	Region	Prdm2	110593	4	142024189	142128244	-
4444	1435985_at	Region	Stk25	59041	1	93449393	93463142	-
4445	1438927_x_at	Region	LOC383587	383587	1	160796026	160825619	+
4446	1455065_x_at	Region	LOC231914	231914	5	148052836	148073109	-
4447	1427260_a_at	Region	Tpm3	59069	3	89883112	89911022	+
4448	1434766_at	Region	Prkaa2	108079	4	103993895	104068774	-
4449	1455373_at	Region	None	None	5	21595010	21595623	-
4450	1426946_at	Region	Ranbp5	70572	14	115481885	115518633	+
4451	1418311_at	Region	Fn3k	63828	11	121256061	121271577	+
4452	1417902_at	Region	Slc19a2	116914	1	164168307	164184621	+
4453	1433552_a_at	Region	Polr2b	231329	5	76554743	76593569	+
4454	1439159_at	Region	Rere	68703	4	148898702	149114159	+
4455	1455850_at	Region	2310003H01Rik	71885	11	120190655	120195298	-
4456	1419467_at	Region	Clec14a	66864	12	54996197	55000365	-
4457	1424445_at	Region	Tm4sf5	75604	11	70230935	70236830	+
4458	1451716_at	Region	Mafb	16658	2	159820730	159823983	-
4459	1424196_at	Region	Yipf1	230584	4	106273336	106318727	+
4460	1456041_at	Region	Snx16	74718	3	10419694	10441634	-
4461	1436148_at	Region	None	None	1	159835170	159835571	+
4462	1436064_x_at	Region	Rps24	20088	14	22859652	22865117	+
4463	1424879_at	Region	Lrch4	231798	5	136579783	136591102	+
4464	1424875_at	Region	Spg20	229285	3	54746476	54770293	+
4465	1457832_at	Region	None	None	2	83363245	83364571	+
4466	1442185_at	Region	Stx5a	56389	19	7938367	7952189	+
4467	1447776_x_at	Region	Rab6	19346	7	94714439	94748311	+
4468	1446543_at	Region	Gcn1l1	231659	5	114676923	114736096	+
4469	1429028_at	Region	Dock11	75974	X	30509386	30697611	+
4470	1437152_at	Region	Rkhd3	108797	7	76722819	76728094	+
4471	1454671_at	Region	Insig1	231070	5	26521457	26528860	+
4472	1423907_a_at	Region	Ndufs8	225887	19	3697661	3701550	-
4473	1435327_at	Region	AW112037	98667	1	191515786	191519464	+
4474	1419954_s_at	Region	Tex27	21769	17	27815127	28020060	+

4475	1426494_at	Region	Rg9mtd3	69934	4	45213090	45232053	+
4476	1421525_a_at	Region	Birc1e	17951	13	96407496	96443102	-
4477	1460554_s_at	Region	Glg1	20340	8	110455266	110556791	-
4478	1438853_x_at	Region	Ddx54	71990	5	119764937	119780399	+
4479	1426900_at	Region	Jmjd1c	108829	10	67223447	67293285	+
4480	1458708_at	Region	Brd4	57261	17	30005231	30094101	-
4481	1436907_at	Region	Nav1	215690	1	135289346	135436181	-
4482	1430395_at	Region	Ankrd45	73844	1	161046813	161071963	+
4483	1450010_at	Region	Hsd17b12	56348	2	93737380	93862592	-
4484	1441634_at	Region	Ntng1	80883	3	109582822	109951623	-
4485	1448604_at	Region	Uck2	80914	1	167159294	167218315	-
4486	1426754_x_at	Region	Ckap4	216197	10	84420966	84428436	-
4487	1421143_at	Region	Diap1	13367	18	38068549	38159133	-
4488	1428582_at	Region	2010208K18Rik	72096	7	127193844	127219029	-
4489	1447186_at	Region	Gm1084	381884	7	39344692	39349907	+
4490	1435981_at	Region	None	None	7	43723560	43724201	+
4491	1451608_a_at	Region	1300010A20Rik	232670	6	29741353	29765655	+
4492	1437023_at	Region	C530015C18	231866	5	142294090	142306832	+
4493	1417723_at	Region	Ube2j1	56228	4	33310330	33329073	+
4494	1428593_at	Region	1700029F09Rik	75623	1	44382435	44398181	-
4495	1425777_at	Region	Cacnb1	12295	11	97823988	97904149	-
4496	1449714_at	Region	5730472N09Rik	108958	2	30296497	30317681	+
4497	1448747_at	Region	Fbxo32	67731	15	58189095	58223887	-
4498	1419123_a_at	Region	Pdgfc	54635	3	80765412	80943027	+
4499	1435855_x_at	Region	Aldh18a1	56454	19	40094578	40132690	-
4500	1458972_at	Region	9330112F22Rik	319237	17	26080291	26080815	+
4501	1435112_a_at	Region	Atp5h	71679	11	115236790	115241012	-
4502	1427909_at	Region	2410015M20Rik	224904	17	54295903	54298222	-
4503	1449251_at	Region	Ndph	17986	X	15124464	15150551	-
4504	1415707_at	Region	Anapc2	99152	2	25204661	25218077	+
4505	1416218_x_at	Region	Rpl37a	19981	1	73012971	73015366	+
4506	1417407_at	Region	Fbxl14	101358	6	119912045	119914888	+
4507	1440001_at	Region	C130089L09Rik	403349	12	105110808	105111442	+
4508	1419301_at	Region	Fzd4	14366	7	83381899	83387590	+
4509	1449066_a_at	Region	Arhgef7	54126	8	11130773	11207926	+
4510	1440965_at	Region	Pigl	327942	11	62184102	62239548	+
4511	1449323_a_at	Region	Rpl3	27367	15	80130401	80135941	-
4512	1435590_at	Region	D430047L21Rik	319896	9	7212223	7213957	+
4513	1451820_at	Region	Diras1	208666	10	81153435	81159222	-
4514	1449816_at	Region	Sult5a1	57429	8	122524128	122539554	-
4515	1437029_at	Region	Tacr3	21338	3	133712014	133817494	+
4516	1433858_at	Region	Lrrc28	67867	7	61397957	61529777	-
4517	1439862_at	Region	None	None	19	18177569	18178337	-
4518	1436309_at	Region	Neto2	74513	8	84912206	84973698	-
4519	1434907_at	Region	Tbpl1	237336	10	22693541	22721040	-
4520	1424095_at	Region	Rtcd1	66368	3	115257493	115276637	-
4521	1457494_at	Region	1700027H10Rik	72274	3	44899129	44899832	+
4522	1445061_at	Region	None	None	14	88329672	88330445	-
4523	1434244_x_at	Region	Htf9c	15547	16	17019721	17024544	+
4524	1428772_at	Region	Xpot	73192	10	121243639	121282407	-
4525	1455439_a_at	Region	Lgals1	16852	15	78978011	78981481	+
4526	1430306_a_at	Region	Atp6v1c2	68775	12	16646095	16687377	-
4527	1415817_s_at	Region	Cct7	12468	6	85801989	85818388	+
4528	1451137_a_at	Region	Brd8	78656	18	34822848	34847583	-
4529	1445674_at	Region	Gm574	213948	5	22848078	22856040	-
4530	1446366_at	Region	Trpc4	22066	3	53790324	53952687	+
4531	1457076_at	Region	Gm693	277743	4	140249514	140265434	+
4532	1448447_at	Region	Vps28	66914	15	76672970	76676909	-
4533	1438877_at	Region	Trpm6	225997	19	17996935	18139467	+
4534	1417271_a_at	Region	Eng	13805	2	32578757	32614280	+
4535	1437685_x_at	Region	Fmod	14264	1	133888996	133898985	+
4536	1434837_at	Region	Mdc1	240087	17	33558766	33575604	+
4537	1457423_at	Region	A930012M17	268777	14	117843742	117864796	-
4538	1422965_at	Region	Agtrap	11610	4	146569423	146580360	-
4539	1435103_x_at	Region	Farslb	23874	1	78713481	78777515	-

4540	1437168_at	Region	MGI:2661424	272009	4	33487784	33512133	+
4541	1415676_a_at	Region	Psmb5	19173	14	49130463	49134465	-
4542	1416554_at	Region	Pdlim1	54132	19	39766556	39815854	-
4543	1419702_at	Region	Taf1a	21339	1	749	2328	+
4544	1415840_at	Region	Elovl5	68801	9	78084592	78151807	+
4545	1416733_at	Region	Mkln1	27418	6	31475825	31586464	+
4546	1428423_at	Region	Pcgf3	69587	5	107537545	107579312	+
4547	1423420_at	Region	Adrb1	11554	19	56308069	56309469	+
4548	1449425_at	Region	Wnt2	22413	6	17972382	18013798	-
4549	1442710_at	Region	Pdlim5	56376	3	141211634	141364594	-
4550	1415724_a_at	Region	Cdc42	12540	4	136200964	136217395	-
4551	1455804_x_at	Region	Oxct1	67041	15	3809284	3936636	+
4552	1428699_at	Region	Sms	20603	X	151043359	151091439	-
4553	1421616_at	Region	Grin2a	14811	16	9251322	9662816	-
4554	1422712_a_at	Region	Ube2i	22196	17	23066362	23080142	-
4555	1451113_a_at	Region	Ik	24010	18	36968481	36981400	+
4556	1419592_at	Region	Unc5c	22253	3	140432729	140804799	+
4557	1426457_at	Region	Slmap	83997	14	24509380	24629945	-
4558	1416148_at	Region	Laptm4b	114128	15	34237699	34283968	+
4559	1439666_at	Region	2010001J22Rik	70113	15	89430321	89431863	-
4560	1435142_at	Region	SFT2 domain containing 2	108735	1	165097432	165117255	-
4561	1436119_at	Region	Aldh1l2	216188	10	83375374	83421986	-
4562	1450629_at	Region	D15Ertd366e	65970	15	99835724	99877288	-
4563	1454803_a_at	Region	Hdac11	232232	6	91599688	91617556	+
4564	1456880_at	Region	None	None	5	128732859	128734156	+
4565	1453126_at	Region	Lrfn2	70530	17	46458498	46623707	+
4566	1424126_at	Region	Alas1	11655	9	106227522	106241451	-
4567	1416775_at	Region	2310004L02Rik	66349	7	20784000	20790057	+
4568	1448967_at	Region	Nipsnap3a	66536	4	52927803	52937938	+
4569	1419269_at	Region	Dut	110074	2	124761335	124772461	+
4570	1435415_x_at	Region	Mlp	17357	4	128540757	128543088	+
4571	1420357_s_at	Region	Xlr3a /// Xlr3b /// MGC76689	22445	X	69356679	69365564	+
4572	1419588_at	Region	Spag1	26942	15	36180636	36236878	+
4573	1427980_at	Region	4933407C03Rik	74440	8	116684557	116791688	+
4574	1417408_at	Region	F3	14066	3	120502430	120513936	+
4575	1454741_s_at	Region	AW547186	209497	X	136121461	136278281	+
4576	1453729_a_at	Region	Rpl37	67281	15	4902211	4904706	+
4577	1457218_at	Region	6430510M02Rik	319517	2	37509620	37512003	-
4578	1433654_at	Region	Mgea5	76055	19	45297695	45330727	-
4579	1429167_at	Region	8430438M01Rik	240261	18	46504298	46533757	-
4580	1419170_at	Region	2310044D20Rik	67698	1	95085396	95106717	+
4581	1423954_at	Region	C3	12266	17	54893340	54917418	-
4582	1426146_a_at	Region	Chpt1	212862	10	88445367	88476119	-
4583	1416332_at	Region	Cirbp	12696	10	80292754	80296566	+
4584	1460653_at	Region	Sca2	20239	5	120898076	120967058	+
4585	1423311_s_at	Region	Tpbg	21983	9	86173432	86176811	+
4586	1428328_at	Region	Nup50	18141	15	84972027	84991562	+
4587	1451968_at	Region	Xrcc5	22596	1	72605227	72696612	+
4588	1449579_at	Region	Sh3yl1	24057	12	27498476	27536048	+
4589	1449246_at	Region	Rap2ip	51799	11	102214763	102223640	+
4590	1450688_at	Region	Rgl2	19732	17	31633637	31641430	+
4591	1460032_at	Region	None	None	8	116661675	116662076	+
4592	1453285_at	Region	2600017H02Rik	67020	11	69122177	69123892	-
4593	1426857_a_at	Region	Hsdl2	72479	4	59525139	59561516	+
4594	1428806_at	Region	Csnk1g1	214897	9	66032337	66163764	+
4595	1448108_at	Region	Tde2	56442	10	57778279	57794950	-
4596	1448337_at	Region	2410003P15Rik	56046	2	155303838	155386985	-
4597	1424320_a_at	Region	Traf7	224619	17	22312435	22331415	-
4598	1431946_a_at	Region	Apba2bp	56846	2	154001342	154012591	-
4599	1456696_x_at	Region	4932432N11Rik	74386	13	57034424	57043384	+
4600	1423724_at	Region	Zwint	52696	10	72709781	72729831	+
4601	1418058_at	Region	Eltd1	170757	3	150417575	150524774	+
4602	1419573_a_at	Region	Lgals1	16852	15	78978011	78981481	+

4603	1430829_s_at	Region	MGI:1347093	26383	8	90688173	90954867	+
4604	1452341_at	Region	Echs1	93747	7	134504720	134515420	-
4605	1416953_at	Region	Ctgf	14219	10	24570014	24573187	+
4606	1439524_at	Region	Dnajb4	67035	3	151165498	151191485	-
4607	1453140_at	Region	9030612M13Rik	208292	17	30596599	30610894	-
4608	1426460_a_at	Region	Ugp2	216558	11	21215937	21265268	-
4609	1440030_at	Region	E330038N15Rik	240776	1	140124450	140418710	+
4610	1456005_a_at	Region	Bcl2l11	12125	2	127639891	127676025	+
4611	1450835_a_at	Region	Gfra4	14588	2	130554029	130556535	-
4612	1418967_a_at	Region	St7	64213	6	17732676	17926408	+
4613	1432653_at	Region	1700008H02Rik	66325	5	81372927	81374233	+
4614	1421393_at	Region	Grin2d	14814	7	39917070	39951776	-
4615	1450779_at	Region	Fabp7	12140	10	58050984	58054546	+
4616	1445831_at	Region	None	None	14	56192477	56193280	-
4617	1429655_at	Region	Nudcd1	67429	15	44320855	44373512	-
4618	1453424_at	Region	Fyco1	17281	9	123815289	123877843	-
4619	1457781_at	Region	Kcnq1ot1	63830	7	137690089	137690789	-
4620	1429709_at	Region	Pmpcb	73078	5	20153825	20209408	+
4621	1416031_s_at	Region	Mcm7	17220	5	137137397	137144671	-
4622	1424353_at	Region	Lrpprc	72416	17	82535095	82620363	-
4623	1416863_at	Region	Abhd8	64296	8	70606574	70613515	-
4624	1436523_s_at	Region	1810022K09Rik	69126	3	105830493	105830917	+
4625	1418483_a_at	Region	Ggta1	14594	2	35332376	35393490	-
4626	1416266_at	Region	Pdyn	18610	2	129200419	129213686	-
4627	1429615_at	Region	Zfp91	109910	19	11961648	11965116	-
4628	1421829_at	Region	Ak3l1	11639	4	100377949	100425900	+
4629	1452915_at	Region	Prkar2a	19087	9	108741435	108795663	+
4630	1418395_at	Region	1300013J15Rik	67473	11	61069042	61103706	-
4631	1422975_at	Region	Mme	17380	3	62963798	63050157	+
4632	1454915_at	Region	1110059F07Rik	98732	1	184746296	184782637	+
4633	1420385_at	Region	Gna14	14675	19	15659862	15834920	+
4634	1423881_at	Region	D19Ertd703e	52036	19	3243622	3364414	-
4635	1424229_at	Region	Dyrk3	226419	1	130977913	130987706	-
4636	1426760_at	Region	Ipo8	320727	6	149706566	149767322	-
4637	1431411_a_at	Region	Rai12	54351	11	69693882	69706168	-
4638	1425301_at	Region	Ncam2	17968	16	80540343	80705441	+
4639	1456048_at	Region	None	None	19	36365274	36366345	-
4640	1447027_s_at	Region	Lias	79464	5	64193326	64211036	+
4641	1417509_at	Region	Rnf19	30945	15	36241202	36267284	-
4642	1426701_at	Region	4632419K20Rik	74349	7	99490967	99512368	-
4643	1433699_at	Region	Tnfaip3	21929	10	18924810	18939261	-
4644	1431045_at	Region	D12Ertd553e	76820	12	11632753	11732017	+
4645	1455617_at	Region	Lmbrd1	68421	1	24831774	25014116	+
4646	1450136_at	Region	Cd38	12494	5	42625902	42669430	+
4647	1448450_at	Region	Ak2	11637	4	128020427	128038642	+
4648	1433676_at	Region	Prkwnk1	232341	6	120356763	120470204	-
4649	1453455_at	Region	1810059M14Rik	75679	4	150285226	150286487	-
4650	1453006_at	Region	2610306H15Rik	72514	19	36260220	36262269	-
4651	1430019_a_at	Region	Hnrpa1	15382	15	103302595	103308263	+
4652	1418925_at	Region	Celsr1	12614	15	85947572	86082913	-
4653	1419043_a_at	Region	Iigp1	60440	18	60615121	60617960	+
4654	1455419_at	Region	D630045J12Rik	330286	6	38261271	38390218	-
4655	1429181_at	Region	1700009P17Rik	75472	1	171050500	171055790	+
4656	1433613_at	Region	Pank3	211347	11	35563514	35580544	+
4657	1437894_at	Region	Prox1	19130	1	189836615	189885751	-
4658	1454840_at	Region	Mccc2	78038	13	96141411	96169595	-
4659	1459268_at	Region	5330439C02Rik	217143	11	97170518	97173164	-
4660	1429519_at	Region	Fpgt	75540	3	154091927	154100383	-
4661	1438493_at	Region	4933433K01Rik	194908	11	59509527	59513274	-
4662	1436198_at	Region	Prep	19072	10	45184189	45275958	+
4663	1432229_a_at	Region	Cdyl2	75796	8	115897616	115952915	-
4664	1425786_a_at	Region	Hsf4	26386	8	104565502	104571473	+
4665	1450213_at	Region	Pde7b	29863	10	20324950	20654021	-
4666	1460687_at	Region	2410195B05Rik	67956	5	123611273	123633650	+
4667	1444846_at	Region	D15Ertd529e	52510	15	22867208	22867908	+

4668	1453475_at	Region	F730014I05Rik	228866	2	164336101	164348170	+
4669	1438256_at	Region	Eif5a2	208691	3	28198695	28216230	+
4670	1426288_at	Region	Lrp4	228357	2	91162450	91218528	+
4671	1442369_at	Region	4832406H04Rik	320971	10	29239506	29240396	+
4672	1452415_at	Region	Actn1	109711	12	77029036	77121075	-
4673	1460165_at	Region	Ppp1ca	19045	19	3980964	3984209	+
4674	1422107_at	Region	2410066E13Rik	68235	6	54826097	54844731	+
4675	1422324_a_at	Region	Pthlh	19227	6	148177711	148191017	-
4676	1433659_at	Region	None	None	2	120701846	120713051	+
4677	1437791_s_at	Region	Eml5	319670	12	94214380	94328523	-
4678	1449324_at	Region	Ero1l /// LOC434220	434220	7	99364931	99367214	+
4679	1442950_at	Region	A830055N07Rik	320290	14	37350411	37351222	-
4680	1431485_at	Region	4833447I15Rik	74844	17	22269223	22273777	-
4681	1423906_at	Region	Hsbp1	68196	8	118687933	118692324	+
4682	1433808_at	Region	D330001F17Rik	223658	15	76430179	76503272	+
4683	1446224_at	Region	Hectd2	226098	19	35897450	35963798	+
4684	1427033_at	Region	Dnmbp	71972	19	43392373	43456795	-
4685	1459008_at	Region	None	None	NONE	NONE	NONE	NONE
4686	1452189_at	Region	9430077D24Rik	77305	9	106164521	106184716	+
4687	1436387_at	Region	C330006P03Rik	320588	13	89544418	89545776	+
4688	1440861_a_at	Region	None	None	7	38670996	38677042	+
4689	1449558_at	Region	F8	14069	X	69833385	70040696	-
4690	1435594_at	Region	Arl6ip2	56298	17	77665786	77673524	-
4691	1438127_at	Region	LOC552906	552906	1	162638387	162639003	-
4692	1438292_x_at	Region	Adk	11534	14	19417295	19813215	+
4693	1436545_at	Region	Dtx4	207521	19	11662421	11697539	-
4694	1455854_a_at	Region	Ssh1	231637	5	113052863	113104401	-
4695	1423031_at	Region	None	None	5	31824425	31862147	+
4696	1450397_at	Region	Mtap1b	17755	13	95613359	95705305	-
4697	1426683_at	Region	Cnot6	104625	11	49427539	49465551	-
4698	1454703_x_at	Region	Rnu22	83673	19	7920714	7922991	+
4699	1427588_a_at	Region	Dtna	13527	18	23643946	23881842	+
4700	1441700_at	Region	C230026C11	328608	15	100880057	100880317	+
4701	1448287_at	Region	Rpo1-3	20018	5	145969234	146003052	+
4702	1434383_at	Region	Pja2	224938	17	61986425	62035635	-
4703	1446632_at	Region	Cacnb2	12296	2	14530578	14913205	+
4704	1435774_at	Region	AV024533	106740	17	13000341	13001826	-
4705	1434724_at	Region	Usp31	76179	7	115517588	115530585	-
4706	1435344_at	Region	1110029I05Rik	319491	9	96220964	96222611	+
4707	1451074_at	Region	Rnf13	24017	3	57378718	57475204	+
4708	1427947_at	Region	BC028440	232987	7	20845746	20851276	+
4709	1449533_at	Region	1810057C19Rik	67888	11	89851435	89857592	+
4710	1417122_at	Region	Vav3	57257	3	109150078	109494759	+
4711	1427154_at	Region	Krt2-17	16681	15	101869161	101876597	-
4712	1424564_at	Region	2410001C21Rik	66404	2	171949311	171978632	+
4713	1424443_at	Region	Tm6sf1	107769	7	75667263	75692207	+
4714	1417220_at	Region	Fah	14085	7	78439084	78459666	-
4715	1424476_at	Region	Camkk2	207565	5	121886097	121917203	-
4716	1447522_s_at	Region	Tnks2	74493	19	36161291	36235550	+
4717	1450530_at	Region	B3galt1	26877	2	67973343	67974323	+
4718	1459469_at	Region	C78516	97147	NONE	NONE	NONE	NONE
4719	1451707_s_at	Region	Slc41a3	71699	6	91044196	91085644	+
4720	1456661_at	Region	Jarid2	16468	13	44305547	44495137	+
4721	1428873_a_at	Region	4121402D02Rik	74026	11	98616603	98627514	+
4722	1434072_at	Region	Smcr7	237781	11	60454040	60458593	+
4723	1426435_at	Region	2810439K08Rik	72759	7	83117226	83316101	-
4724	1441036_at	Region	None	None	3	53142021	53142715	+
4725	1437026_at	Region	BC057893	272027	4	46030668	46054332	-
4726	1446253_at	Region	Slc18a2	214084	19	58860486	58895236	+
4727	1442381_at	Region	Mkln1	27418	6	31475825	31586464	+
4728	1448284_a_at	Region	Ndufc1	66377	3	51037443	51040919	-
4729	1452186_at	Region	Rbm5	83486	9	107808484	107838963	-
4730	1433940_at	Region	Spag7	216873	11	70389448	70394951	-
4731	1417162_at	Region	2310061B02Rik	69660	1	74590721	74606627	-

4732	1442083_at	Region	1500011J06Rik	208606	5	122899619	122920601	-
4733	1450064_at	Region	Fmn2	54418	1	174435022	174755223	+
4734	1433738_at	Region	5730445M16Rik	214627	8	87484565	87545069	+
4735	1458245_at	Region	None	None	4	59272841	59276418	-
4736	1453139_at	Region	Nudt12	67993	17	56685627	56697548	-
4737	1459871_x_at	Region	38413	224703	17	31384823	31417809	-
4738	1428160_at	Region	9330101J02Rik	320625	7	134056422	134079630	-
4739	1427236_a_at	Region	Mll5	69188	5	21898353	21957234	+
4740	1454200_at	Region	Zfhx1b	24136	2	44919283	45045178	-
4741	1454012_a_at	Region	Parp6	67287	9	59734045	59767165	+
4742	1439097_at	Region	D10Wsu52e	28088	10	85899581	85918735	-
4743	1422150_at	Region	Hmx3	15373	7	125858776	125860738	+
4744	1417840_at	Region	1500031L02Rik	66994	16	30909173	30917425	+
4745	1416354_at	Region	Rbmx	19655	X	52141072	52147740	-
4746	1432787_at	Region	6720420G18Rik	77072	5	13209250	13210354	-
4747	1424433_at	Region	Msrb2	76467	2	19413646	19436980	+
4748	1455048_at	Region	Igsf3	78908	3	100804224	100886780	+
4749	1442739_at	Region	BC031441	240120	17	53576717	53583603	-
4750	1434639_at	Region	Kbtbd9	208439	12	4250774	4550482	-
4751	1435901_at	Region	Usp40	227334	1	87765405	87827652	-
4752	1435251_at	Region	Snx13	217463	12	31761156	31762501	+
4753	1450122_at	Region	Ptprg	19270	14	10014966	10698138	+
4754	1426556_at	Region	Suhw4	235469	9	72427922	72473749	+
4755	1417866_at	Region	Tnfaip1	21927	11	78248507	78261853	-
4756	1455649_at	Region	Ttc9	69480	12	78486357	78523920	+
4757	1452221_a_at	Region	Cxxc1	74322	18	74450441	74455720	+
4758	1419583_at	Region	Cbx4	12418	11	118901248	118907234	-
4759	1427984_at	Region	Senp6	215351	9	80272603	80350480	+
4760	1442363_at	Region	1110012J17Rik	68617	17	64055405	64103991	-
4761	1430197_a_at	Region	Pitpnm2	19679	5	123290016	123387759	-
4762	1424614_at	Region	MGI:2385286	233575	7	96329032	96343721	+
4763	1452423_at	Region	Pclo	26875	5	13968048	14249123	+
4764	1420513_at	Region	Efcab2	68226	1	178360562	178437930	+
4765	1429281_at	Region	2610008E11Rik	72128	10	79180416	79213667	-
4766	1423792_a_at	Region	Cklfsf6	67213	9	114704736	114722975	+
4767	1435374_at	Region	None	None	8	115891850	115893446	-
4768	1452744_at	Region	2210016F16Rik	70153	13	57013547	57017878	-
4769	1418337_at	Region	Rpia	19895	6	71085426	71111880	-
4770	1418329_at	Region	Pgpep1	66522	8	69805459	69816029	-
4771	1419551_s_at	Region	Stk39	53416	2	68065847	68327381	-
4772	1448646_at	Region	Wdr12	57750	1	60380183	60401193	-
4773	1416010_a_at	Region	Ehd1	13660	19	6065686	6088886	+
4774	1433553_at	Region	Garnl3	99326	2	32918528	33019366	-
4775	1459235_at	Region	None	None	3	133027580	133028278	-
4776	1428621_a_at	Region	D11Wsu68e	28084	11	101074830	101080633	+
4777	1439271_x_at	Region	Ik	24010	18	36968481	36981400	+
4778	1430089_at	Region	5830469G19Rik	76093	4	128847860	128849167	+
4779	1428565_at	Region	1300004G08Rik /// LOC545242	545242	18	8736528	8739684	+
4780	1442421_at	Region	None	None	5	134880019	134880659	+
4781	1458290_at	Region	R3hdm	226412	1	127946018	128080726	+
4782	1417335_at	Region	Sult2b1	54200	7	39813262	39844164	-
4783	1437759_at	Region	Pfkp	56421	13	6476970	6563786	-
4784	1423701_at	Region	Coasy	71743	11	100903693	100907704	+
4785	1450787_at	Region	Clcn5	12728	X	5398413	5427626	-
4786	1425926_a_at	Region	Otx2	18424	14	43747109	43751810	-
4787	1434831_a_at	Region	Foxo3a	56484	10	42292466	42383422	-
4788	1454869_at	Region	Wdr40b	245404	X	39307366	39310947	-
4789	1460703_at	Region	Ascc1	69090	10	59958483	60060150	+
4790	1449040_a_at	Region	Sephs2	20768	7	121321610	121323787	-
4791	1449336_a_at	Region	Slk	20874	19	47131319	47193755	+
4792	1434159_at	Region	Stk4	58231	2	163531147	163612490	+
4793	1426739_at	Region	Donson	60364	16	90836429	90845892	-
4794	1425351_at	Region	Srxn1	76650	2	151562666	151568289	+
4795	1459737_s_at	Region	Ttr	22139	18	20880341	20889247	+

4796	1419633_at	Region	Uncx4.1	22255	5	138541401	138545671	+
4797	1451466_at	Region	D16Ertd472e	67102	16	77629000	77662117	-
4798	1418078_at	Region	Psme3	19192	11	101137338	101144618	+
4799	1429650_at	Region	Stk40	74178	4	125131111	125167995	+
4800	1435764_a_at	Region	Gemin7	69731	7	16434193	16442587	-
4801	1456034_at	Region	Ttc18	76670	14	18755509	18813534	-
4802	1454794_at	Region	Spg4	50850	17	72144720	72194776	+
4803	1456509_at	Region	1110032F04Rik	68725	3	68516408	68540974	+
4804	1449749_s_at	Region	Tfb1m	224481	17	3480942	3519462	-
4805	1416362_a_at	Region	Fkbp4	14228	6	129120294	129128818	-
4806	1451395_at	Region	D5Bwg0834e	215707	5	123994698	124021886	-
4807	1425371_at	Region	Polb	18970	8	21382844	21407993	-
4808	1437926_at	Region	E430012M05Rik	321003	15	81450988	81505688	+
4809	1446990_at	Region	Nfia	18027	4	96752972	97087465	+
4810	1433623_at	Region	Zfp367	238673	13	61517909	61538052	-
4811	1428983_at	Region	Scx	20289	15	76507726	76509749	+
4812	1437283_at	Region	Tnpo2	212999	8	84307954	84326395	+
4813	1452655_at	Region	Zdhhc2	70546	8	39367458	39428485	+
4814	1425414_at	Region	Ppp1r16b	228852	2	158122367	158219842	+
4815	1423413_at	Region	Ndrg1	17988	15	66953527	66993844	-
4816	1455236_x_at	Region	Serf2	378702	2	120963266	120964906	+
4817	1457731_at	Region	Snapc3	77634	4	82403941	82453789	+
4818	1441312_at	Region	Cnnm1	83674	19	42985617	43040169	+
4819	1424113_at	Region	Lamb1-1	16777	12	27840552	27905646	+
4820	1426343_at	Region	1300006C19Rik	68292	9	115225956	115293786	-
4821	1451225_at	Region	Ptpn11	19247	5	120282348	120343136	-
4822	1448621_a_at	Region	Smpd1	20597	7	99666853	99670820	+
4823	1451241_at	Region	Lamb1-1	16777	12	27840552	27905646	+
4824	1426313_at	Region	Bre	107976	5	30156988	30543677	+
4825	1426923_at	Region	Hrb	15463	1	83168589	83225381	+
4826	1441989_at	Region	Bnip2	12175	9	70120357	70139177	+
4827	1453732_at	Region	6230416A05Rik	76137	13	43101452	43122631	-
4828	1422341_s_at	Region	Lypla3	192654	8	105446246	105460562	+
4829	1421997_s_at	Region	Itga3	16400	11	94865569	94897801	-
4830	1423113_a_at	Region	Ube2d3	66105	3	134321860	134350243	+
4831	1420377_at	Region	St8sia2	20450	7	67831801	67906341	-
4832	1441347_at	Region	Hrbl	231801	5	136601442	136635293	-
4833	1456120_at	Region	3110001I20Rik	70354	2	125250846	125296723	-
4834	1451868_at	Region	Kcnj6	16522	16	94170119	94405052	-
4835	1416565_at	Region	Cox6b1	110323	7	26029666	26038843	-
4836	1419011_at	Region	Cryba2	12958	1	75194153	75197344	-
4837	1427930_at	Region	Pdxk	216134	10	78549713	78575725	-
4838	1438224_at	Region	Zswim5	74464	4	115947387	115948169	+
4839	1456013_x_at	Region	Slc35a4	67843	18	36903357	36907622	+
4840	1424224_at	Region	Asb8	78541	15	98210202	98221206	-
4841	1440179_x_at	Region	LOC268291	268291	10	31542086	31649384	-
4842	1460439_at	Region	BC033915	70661	9	46026130	46236394	+
4843	1451068_s_at	Region	Rps25	75617	9	44396569	44399261	+
4844	1450901_a_at	Region	AW011752	104570	11	29067786	29113926	+
4845	1459793_s_at	Region	None	None	6	146063689	146063978	+
4846	1454805_at	Region	Wtap	60532	17	11539853	11559714	+
4847	1427512_a_at	Region	Lama3	16774	18	12521625	12770367	+
4848	1439824_at	Region	Chm	12662	X	107440914	107585000	-
4849	1459987_s_at	Region	Cct3	12462	3	88040999	88065630	+
4850	1418290_a_at	Region	Ezh1	14055	11	101012203	101049035	-
4851	1431200_a_at	Region	Zcsl2	105638	14	30212767	30217780	-
4852	1438140_a_at	Region	Zfp64	22722	2	168434104	168463992	-
4853	1454973_at	Region	Atf7ip	54343	6	137280522	137369083	+
4854	1456423_at	Region	Mbd5	109241	2	49178095	49249231	+
4855	1433489_s_at	Region	Fgfr2	14183	7	124341981	124389452	-
4856	1415891_at	Region	Suclg1	56451	6	73586370	73614579	+
4857	1424635_at	Region	Eef1a1	13627	9	78678990	78682232	-
4858	1452256_at	Region	1110002N22Rik	68550	11	79862341	79867783	-
4859	1438011_at	Region	Pcyt1a	13026	16	31261076	31281386	+
4860	1430274_a_at	Region	Stard3nl	76205	13	18839040	18877114	-

4861	1424112_at	Region	Igf2r	16004	17	11321447	11408701	-
4862	1455244_at	Region	Daam1	208846	12	68668036	68829803	+
4863	1450394_at	Region	Golph3	66629	15	12132755	12162746	+
4864	1451019_at	Region	Ctsf	56464	19	4643919	4649702	+
4865	1450750_a_at	Region	Nr4a2	18227	2	57033695	57040794	-
4866	1451479_a_at	Region	Tmem53	68777	4	116210894	116227485	+
4867	1429778_at	Region	Optn	71648	2	4937914	4981210	-
4868	1438427_at	Region	4932442K08Rik	67544	17	13399931	13435680	+
4869	1441777_at	Region	Emx1	13796	6	85553737	85554185	+
4870	1423426_at	Region	1300012G16Rik	71772	5	119635089	119655425	-
4871	1457468_at	Region	5430432P15Rik	74493	19	36161291	36235550	+
4872	1457141_at	Region	None	None	7	91771016	91771272	-
4873	1449354_at	Region	U2af1-rs1	22183	11	22866827	22869698	+
4874	1457050_at	Region	1700129O19Rik	78643	13	48940434	48940769	-
4875	1424238_at	Region	Sirt7	209011	11	120439464	120446060	-
4876	1451972_at	Region	Glcci1	170772	6	8234651	8573400	+
4877	1432555_at	Region	Whrn	73750	4	62506101	62587142	-
4878	1425287_at	Region	Zfp189	230162	4	49437099	49447415	+
4879	1441790_at	Region	Lrrn6a	235402	9	56731964	56798742	-
4880	1451346_at	Region	Mtap	66902	4	88123618	88167275	+
4881	1457955_at	Region	Gga2	74105	7	115864580	115899025	-
4882	1454798_at	Region	B930007L02Rik	321006	9	106946699	106948923	-
4883	1429571_a_at	Region	Spaca1	67652	4	34307107	34328860	-
4884	1427676_a_at	Region	Grik1	14805	16	87069420	87213311	-
4885	1417796_at	Region	Gps2	56310	11	69639850	69642249	+
4886	1436949_a_at	Region	Tceb2	67673	17	21626920	21631289	-
4887	1428436_at	Region	2700023B17Rik	67070	7	15609988	15654960	-
4888	1425492_at	Region	Bmpr1a	12166	14	32547251	32638735	-
4889	1460399_at	Region	BC018601	104479	11	5423674	5436942	-
4890	1429784_at	Region	C130032J12Rik	218975	14	42400908	42402444	+
4891	1456429_at	Region	Malt1	240354	18	65664223	65712018	+
4892	1451023_at	Region	Hcn3	15168	3	88903248	88916630	-
4893	1426329_s_at	Region	Baalc	118452	15	38833124	38850257	+
4894	1447805_s_at	Region	D11Ertd730e	193116	11	43186568	43200818	+
4895	1419532_at	Region	Il1r2	16178	1	40379307	40419909	+
4896	1456405_at	Region	6720461J16Rik	269399	2	180374922	180375704	-
4897	1427025_at	Region	Mtmr7	54384	8	39494737	39579044	-
4898	1455680_at	Region	9630025H16Rik	319878	3	68737668	68739211	-
4899	1460326_at	Region	Pik3ca	18706	3	31844384	31871009	+
4900	1452268_at	Region	2810485I05Rik	72826	9	13664776	13683571	+
4901	1451422_at	Region	Myo18a	360013	11	77502924	77591639	+
4902	1451382_at	Region	1810008K03Rik	69065	2	118865105	118868230	+
4903	1416400_at	Region	Pycrl	66194	15	75966546	75971629	-
4904	1436292_a_at	Region	Oaz1	18245	10	80958712	80961345	+
4905	1447623_s_at	Region	None	None	12	47079899	47080196	-
4906	1436867_at	Region	Srl	106393	16	4151806	4194573	-
4907	1421786_at	Region	Ppp3r1	19058	11	17087637	17089902	+
4908	1420609_at	Region	38418	57438	2	60065336	60103785	+
4909	1449088_at	Region	Fbp2	14120	13	534	13173	+
4910	1424210_at	Region	Spfh1	226144	19	43579353	43613932	-
4911	1416849_at	Region	D10Ertd214e	52637	10	71384461	71398816	-
4912	1423902_s_at	Region	Arhgef12	69632	9	42922812	43064327	-
4913	1455424_at	Region	Wnk2	75607	13	48638624	48748243	-
4914	1435620_at	Region	1190002J23Rik	68874	1	171288622	171289723	-
4915	1435211_at	Region	Ttc12	235330	9	49475000	49524266	-
4916	1419350_at	Region	Hook2	170833	8	84258797	84273699	+
4917	1438073_at	Region	None	None		165746	166396	-
4918	1434335_at	Region	AI317237	239667	15	100211934	100273204	+
4919	1456272_at	Region	2900045N06Rik	72895	6	113640716	113716498	+
4920	1416265_at	Region	Capn10	23830	1	92762343	92775432	+
4921	1452604_at	Region	Stard13 /// LOC547385	243362	5	149978354	150130919	-
4922	1428406_s_at	Region	Hcfc1r1	353502	17	21475788	21477704	+
4923	1427104_at	Region	Zfp612	234725	8	109377438	109387872	+
4924	1424018_at	Region	Hint1	15254	11	54619279	54623337	+

4925	1437467_at	Region	Alcam	11658	16	51168811	51372049	-
4926	1460586_at	Region	Egfl4	269878	7	20497229	20546922	+
4927	1453689_at	Region	Fance	72775	17	26105507	26123342	+
4928	1420286_at	Region	None	None	8	83678088	83678241	-
4929	1452263_at	Region	Slc35f4	75288	14	44388213	44617528	-
4930	1453032_at	Region	2610109B12Rik	70452	1	55454959	55456058	+
4931	1453677_a_at	Region	Derl3	70377	10	75995138	75997681	+
4932	1417390_at	Region	Xab1	74254	5	29953699	29970565	+
4933	1439305_at	Region	None	None	X	98178992	98179701	-
4934	1448115_at	Region	Htf9c	15547	16	17019721	17024544	+
4935	1417471_s_at	Region	D1Ertd622e	52392	1	97421426	97439499	-
4936	1429534_a_at	Region	Immt	76614	6	72203510	72209365	+
4937	1416773_at	Region	Wee1	22390	7	103974933	103996157	+
4938	1449571_at	Region	Trhr	22045	15	44141857	44181536	+
4939	1417947_at	Region	Pcna	18538	2	131763139	131767033	-
4940	1442030_at	Region	None	None	11	120408231	120409293	-
4941	1453314_x_at	Region	None	None	16	89876348	89884408	-
4942	1417060_at	Region	Ppp1r11	76497	17	34708791	34711487	-
4943	1457358_at	Region	Mrg1	17536	2	115376917	115578960	-
4944	1418870_at	Region	4930579J09Rik	67752	19	9670754	9679394	-
4945	1439969_at	Region	4930500O05Rik	242409	4	43584814	43606284	+
4946	1442001_at	Region	Prkab2	108097	3	97153614	97159355	+
4947	1436664_a_at	Region	Slc35a2	22232	X	6123184	6133345	+
4948	1423744_x_at	Region	Eif2s3x	26905	X	88849367	88873309	-
4949	1425677_a_at	Region	Ank1	11733	8	21812854	21905075	+
4950	1449325_at	Region	Fads2	56473	19	9260661	9298000	-
4951	1440308_at	Region	None	None	4	113918241	113918897	-
4952	1428548_at	Region	Pak4	70584	7	23964692	24004019	-
4953	1448490_at	Region	Adck4	76889	7	22607050	22630916	+
4954	1455590_at	Region	Nqo2	18105	13	33496423	33520103	+
4955	1421164_a_at	Region	Arhgef1	16801	7	20020972	20104618	+
4956	1434498_at	Region	Gm1673	381633	5	32471754	32473272	+
4957	1458644_at	Region	AI661708	100717	5	123139455	123139928	+
4958	1429469_at	Region	A930013F10Rik	68074	8	21388966	21390156	-
4959	1434161_at	Region	5830457H20Rik	217708	12	81319855	81399880	+
4960	1434995_s_at	Region	Dedd	21945	1	171267091	171270454	+
4961	1455946_x_at	Region	Tmsb10	19240	6	20039912	20040386	+
4962	1424361_at	Region	BC019943	234138	8	29932839	29943872	+
4963	1418242_at	Region	Faf1	14084	4	108635532	108922865	+
4964	1460400_at	Region	Spg4	50850	17	72144720	72194776	+
4965	1460483_at	Region	2610034E01Rik	69236	10	72713044	72713826	+
4966	1428747_at	Region	1110007F05Rik	71787	4	131272654	131290416	-
4967	1428132_at	Region	Cdc42se1	57912	3	94716611	94724240	+
4968	1452418_at	Region	1200016E24Rik	319202	4	82526160	82526678	-
4969	1435783_at	Region	B230112C05Rik	320557	13	93256692	93318815	+
4970	1436804_s_at	Region	Scyl1	78891	19	5547217	5560191	-
4971	1436307_at	Region	Myo9a	270163	9	59868073	60046149	+
4972	1428283_at	Region	Cyp2s1	74134	7	20967259	20981313	-
4973	1440359_at	Region	1700012H17Rik	242297	4	5571325	5727090	+
4974	1425262_at	Region	Cebpg	12611	7	30201783	30211794	-
4975	1438007_at	Region	AI851790	268354	10	122955023	123432354	+
4976	1419650_at	Region	Zfr	22763	15	11948204	11996199	+
4977	1433943_at	Region	BC063749	414801	19	47445900	47470581	-
4978	1447447_s_at	Region	Srrm1	51796	4	134203290	134234393	-
4979	1429841_at	Region	3000002B06Rik	70417	18	57349956	57514042	+
4980	1436425_at	Region	Ankrd38	242553	4	97729971	97792610	-
4981	1435779_at	Region	Cep1	26920	2	35064394	35110984	+
4982	1431232_a_at	Region	Mga	29808	2	119416522	119478814	+
4983	1438630_x_at	Region	Mat2a	232087	6	72764773	72771532	-
4984	1450876_at	Region	Cfh	12628	1	139935794	140062362	-
4985	1448517_at	Region	Timm22	56322	11	76132848	76141950	+
4986	1443941_at	Region	LOC433886	433886	5	51185451	51186441	-
4987	1423095_s_at	Region	Crbn	58799	6	107274263	107296083	-
4988	1417424_at	Region	Ier3ip1	66191	18	77051756	77063320	+
4989	1439717_at	Region	B230362M20Rik	320835	7	51449539	51659259	-

4990	1421823_a_at	Region	Ppp2cb	19053	8	32405226	32425404	+
4991	1433735_a_at	Region	9630015D15Rik	100201	4	15192785	15212331	+
4992	1423063_at	Region	Dnmt3a	13435	12	3001834	3066656	+
4993	1456694_x_at	Region	Ptpn6	15170	6	125376099	125388499	-
4994	1424133_at	Region	6530411B15Rik	103743	11	80536074	80547692	+
4995	1437389_x_at	Region	None	None	4	128741972	128742327	-
4996	1429555_at	Region	1110019C08Rik	224250	16	57715966	57722159	+
4997	1445406_at	Region	C77494	97789	12	82097027	82097664	+
4998	1421391_at	Region	Vipr2	22355	12	111840931	111908508	+
4999	1416590_a_at	Region	Rab34	19376	11	77914493	77917847	+
5000	1439741_x_at	Region	AI481316	98383	1	167156103	167156616	-
5001	1431752_a_at	Region	2900073H19Rik	68205	2	29759551	29777158	+
5002	1455319_x_at	Region	Rps8	20116	4	116112741	116115037	-
5003	1438143_s_at	Region	None	None	5	120963827	120967071	+
5004	1426403_at	Region	Actr1b	226977	1	36993793	37004516	-
5005	1424559_at	Region	AW060207	231571	5	106668184	106732285	+
5006	1423407_a_at	Region	Fbln2	14115	6	91655737	91715413	+
5007	1436756_x_at	Region	Hadhsc	15107	3	130163431	130202003	-
5008	1451420_at	Region	2610204L23Rik	67163	11	106021555	106037392	-
5009	1419575_s_at	Region	Zfp292	30046	4	35020884	35026191	-
5010	1421060_at	Region	Mllt1	64144	17	54582533	54625310	-
5011	1452237_at	Region	Hrb	15463	1	83168589	83225381	+
5012	1426569_a_at	Region	Frk	14302	10	34545676	34672847	+
5013	1425291_at	Region	Foxj1	15223	11	116151797	116156440	-
5014	1450691_at	Region	Caskin2	140721	11	115620445	115634685	-
5015	1428441_at	Region	1500009M05Rik	67006	3	134289421	134306505	-
5016	1435004_at	Region	Pank4	269614	4	153456495	153473235	+
5017	1434572_at	Region	Hdac9	79221	12	30981181	30981672	-
5018	1419935_s_at	Region	Csnk2a2	13000	8	94726673	94766378	-
5019	1424515_at	Region	B230354K17Rik	320063	17	42944253	42952945	+
5020	1438176_x_at	Region	1110031B06Rik	67826	11	59132779	59175585	-
5021	1421217_a_at	Region	Lgals9	16859	11	78689054	78710490	-
5022	1456084_x_at	Region	Fmod	14264	1	133888996	133898985	+
5023	1418025_at	Region	Bhlhb2	20893	6	109163628	109169924	+
5024	1455194_at	Region	None	None	15	89516283	89517342	+
5025	1452153_at	Region	Fbxo18	50755	2	11658587	11693536	-
5026	1426863_at	Region	Rbmx	19655	X	52141072	52147740	-
5027	1460691_at	Region	Zfp598	213753	17	22474097	22486361	+
5028	1454815_at	Region	Sertad2	58172	11	20438127	20543916	+
5029	1449448_at	Region	Med9	192191	11	59673892	59686765	+
5030	1435638_at	Region	None	None	7	20409114	20418683	-
5031	1427958_at	Region	Abhd10	213012	16	44615021	44627461	-
5032	1426948_at	Region	Tpr	108989	1	150229615	150286691	+
5033	1450906_at	Region	Plxnc1	54712	10	94767461	94918749	-
5034	1419299_at	Region	2010012O05Rik	66439	19	46236473	46250119	+
5035	1418296_at	Region	Fxyd5	18301	7	26440973	26450456	-
5036	1439649_at	Region	Adc	242669	4	127959259	127989530	-
5037	1457687_at	Region	Bcl2	12043	1	106479000	106655083	-
5038	1424771_at	Region	E130307C13	232440	6	137580120	137581870	+
5039	1449493_at	Region	Insl5	23919	4	101976777	101985633	-
5040	1436071_at	Region	Ankrd26	232339	6	118934772	118994843	-
5041	1446448_at	Region	Pias1	56469	9	63002575	63103377	-
5042	1444978_at	Region	None	None	6	99388128	99389025	-
5043	1423264_at	Region	Bop1	12181	15	76503230	76527558	-
5044	1429678_at	Region	5730508B09Rik	70617	3	126755462	126781211	-
5045	1439830_at	Region	Map3k5	26408	10	19860944	20067353	+
5046	1427195_at	Region	None	None	18	14383968	14385252	+
5047	1424023_at	Region	BC005752	233189	7	37747538	37754019	+
5048	1416907_at	Region	Tsn	22099	1	118048034	118060649	-
5049	1452971_at	Region	Upf3a	67031	8	13164227	13177336	+
5050	1429196_at	Region	Rabgap1l	29809	1	160161852	160697585	-
5051	1439364_a_at	Region	Mmp2	17390	8	92114446	92140538	+
5052	1420998_at	Region	Etv5	104156	16	21153380	21211575	-
5053	1438051_at	Region	D3Ertd789e	51938	3	33256108	33288047	-
5054	1423518_at	Region	Csk	12988	9	57740570	57759104	-

5055	1452158_at	Region	Eprs	107508	1	184861496	184926808	+
5056	1419158_a_at	Region	Harsl	70791	18	37007045	37016327	+
5057	1426414_a_at	Region	Rnf7	19823	9	96369942	96420174	-
5058	1420501_at	Region	Dnajc1	13418	2	18259145	18434517	-
5059	1417675_a_at	Region	Mdn1	100019	4	32935912	32975347	+
5060	1448659_at	Region	Casp7	12369	19	55981748	56027579	+
5061	1448775_at	Region	LOC547362	547362	1	143299	165189	+
5062	1454952_s_at	Region	B130055D15Rik	78658	9	26924144	26989070	+
5063	1422579_at	Region	Hspe1	15528	1	55389476	55392465	+
5064	1453567_s_at	Region	2810441K11Rik	68642	19	9746963	9752737	-
5065	1418988_at	Region	Pex7	18634	10	19786688	19833926	-
5066	1438568_at	Region	Mrgpre	244238	7	138192261	138198214	-
5067	1423127_at	Region	Impa1	55980	3	10315087	10332919	-
5068	1445567_at	Region	Gap43	14432	16	41113620	41205719	-
5069	1440586_at	Region	B430203I24Rik	97874	13	110512917	110513609	+
5070	1428524_at	Region	2310051N18Rik	71941	8	10886405	10928442	-
5071	1416330_at	Region	Cd81	12520	7	137465814	137480943	+
5072	1435813_at	Region	Mypn	68802	10	63080660	63168856	-
5073	1433893_s_at	Region	Spag5	54141	11	78027295	78048113	+
5074	1439572_at	Region	R3hdm	226412	1	127946018	128080726	+
5075	1439640_at	Region	None	None	4	135239931	135240704	+
5076	1424925_at	Region	Sec63	140740	10	42868086	42935647	+
5077	1436318_at	Region	Tardbp	230908	4	147104744	147119358	-
5078	1441052_at	Region	2310035C23Rik	227446	1	105555177	105695178	+
5079	1455019_x_at	Region	Ckap4	216197	10	84420965	84421362	-
5080	1423937_at	Region	Kctd5	69259	17	21851732	21877431	-
5081	1459009_at	Region	Utrn	22288	10	12263109	12646998	-
5082	1426430_at	Region	Jag2	16450	12	108384687	108405592	-
5083	1436185_at	Region	AI314180	230249	4	58743479	58856174	-
5084	1432184_a_at	Region	2610204M08Rik	70435	12	108084044	108094944	+
5085	1418912_at	Region	Plxdc2	67448	2	16282594	16678387	+
5086	1442312_at	Region	Tbl1xr1	81004	3	21419728	21554694	+
5087	1426586_at	Region	Slc25a11	67863	11	70369854	70372651	-
5088	1460031_at	Region	Gpr45	93690	1	43247681	43330167	+
5089	1435395_s_at	Region	Atp5j2	57423	5	144222142	144230028	-
5090	1455518_at	Region	None	None	4	132566198	132566885	-
5091	1459980_x_at	Region	Rab3a	19339	8	69909984	69913935	+
5092	1415708_at	Region	AI316828	544752	11	3534987	3539316	-
5093	1425257_at	Region	BC016076	217698	12	80933641	80940335	+
5094	1449290_at	Region	Dpysl5	65254	5	29170843	29258315	+
5095	1451086_s_at	Region	Rac1	19353	5	142563708	142586223	-
5096	1455113_at	Region	Armc8	74125	9	99381642	99469875	-
5097	1442676_at	Region	Maoa	17161	X	14858768	14924684	+
5098	1455303_at	Region	None	None	3	54437335	54438377	-
5099	1444270_at	Region	Rshl3	212892	10	33960259	33970986	+
5100	1429356_s_at	Region	Ggps1	14593	13	13494995	13505951	-
5101	1431196_at	Region	Atp2c1	235574	9	105393471	105477208	-
5102	1424969_s_at	Region	None	None	2	58680481	58716779	+
5103	1422885_at	Region	Snrpd3	67332	10	75619782	75637180	+
5104	1454820_at	Region	BC037034	231807	5	137231897	137235424	-
5105	1458389_at	Region	Klf7	93691	1	64328681	64414407	-
5106	1436056_at	Region	Kif13b	16554	14	59180809	59337895	+
5107	1418788_at	Region	Tek	21687	4	93714372	93850011	+
5108	1417507_at	Region	Cyb561	13056	11	105754793	105765236	-
5109	1418719_at	Region	2410004L22Rik	76478	8	70400736	70421775	-
5110	1434751_at	Region	Ids	15931	X	65014764	65033475	-
5111	1425662_at	Region	Cdadc1	71891	14	54094451	54133002	-
5112	1453318_at	Region	4632403M07Rik	380705	11	69528692	69531280	-
5113	1420776_a_at	Region	Auh	11992	13	51398632	51493178	-
5114	1419833_s_at	Region	Centd3	106952	18	38196350	38220837	-
5115	1455350_at	Region	B830009D23Rik	96957	2	120490911	120521692	+
5116	1450655_at	Region	Herc1	235439	9	66474369	66632783	+
5117	1433781_a_at	Region	Cldn12	64945	5	5511114	5520878	-
5118	1424052_at	Region	Thap4	67026	1	93532881	93578284	-
5119	1450431_a_at	Region	Nedd4	17999	9	72794997	72882331	+

5120	1451872_a_at	Region	Neurl	18011	19	46730295	46810735	+
5121	1422034_a_at	Region	Palm	18483	10	79929264	79943520	+
5122	1450943_at	Region	2010012C16Rik	66441	6	132063125	132071980	-
5123	1443544_at	Region	D18Ertd653e	52662	18	68295003	68486335	+
5124	1457445_at	Region	Trps1	83925	15	50659276	50888038	-
5125	1440103_at	Region	None	None	8	68329781	68330268	-
5126	1457191_at	Region	None	None	3	55726059	55726719	-
5127	1437464_at	Region	Spata7	104871	12	94055026	94096657	+
5128	1417477_at	Region	Gtlf3b	24083	11	60627888	60639432	-
5129	1455896_a_at	Region	Kcnk1	16525	8	125282699	125318214	+
5130	1448523_at	Region	Nphp1	53885	2	127254585	127302707	-
5131	1425462_at	Region	Fbxw11	103583	11	32537669	32641610	+
5132	1457189_at	Region	Itpr1	16438	6	108714663	109053117	+
5133	1441590_at	Region	None	None	9	32238162	32238812	-
5134	1425605_a_at	Region	Lmbr1	56873	5	27683542	27828674	-
5135	1426379_at	Region	Eif4b	75705	15	102141346	102156533	+
5136	1451236_at	Region	Rerg	232441	6	137826729	137942405	-
5137	1455094_s_at	Region	Ube2g1	67128	11	72333039	72409965	+
5138	1435138_at	Region	Tmem28	270028	8	8571891	9138527	-
5139	1435567_at	Region	Phka1	18679	X	97117039	97245563	-
5140	1437210_a_at	Region	Brd2	14312	17	31817639	31824919	-
5141	1422527_at	Region	H2-DMa	14998	17	31841425	31844898	+
5142	1457283_at	Region	None	None	18	36241977	36420340	-
5143	1435576_at	Region	AW413774	106046	15	5004951	5005878	+
5144	1434771_at	Region	0610011F06Rik	68347	17	23682250	23683893	+
5145	1439840_at	Region	A430088C08Rik	320892	8	21399438	21400852	-
5146	1432494_a_at	Region	1700019E19Rik	76411	12	82951854	83032603	+
5147	1416916_at	Region	Elf3	13710	1	135104072	135108931	-
5148	1419230_at	Region	Krt1-12	268482	11	99236753	99243343	-
5149	1429421_at	Region	2610203E10Rik	329470	2	93540136	93554459	-
5150	1450008_a_at	Region	Ctnnb1	12387	9	120945869	120972901	+
5151	1441615_at	Region	Cbfa2t2h	12396	2	153893455	153996293	+
5152	1435528_at	Region	D2Bwg1335e	52838	2	26280280	26284262	-
5153	1424388_at	Region	Cluap1	76779	16	3580573	3612711	+
5154	1428513_at	Region	Calcoco1	67488	15	102767198	102782488	-
5155	1437216_at	Region	A430106J12Rik	108686	11	29375382	29404231	+
5156	1415852_at	Region	Impdh2	23918	9	108609499	108614571	+
5157	1421938_at	Region	Serhl	68607	15	83150886	83173420	+
5158	1441218_at	Region	None	None	9	119978782	119979393	+
5159	1423647_a_at	Region	Zdhhc3	69035	9	123091926	123130868	-
5160	1427646_a_at	Region	Arhgef2	16800	3	88365002	88390778	+
5161	1459026_at	Region	Skiip	66354	12	84536947	84547086	+
5162	1450732_a_at	Region	Bicd2	76895	13	48941109	48984662	+
5163	1427144_at	Region	Hnrpll	72692	17	77846881	77879599	-
5164	1439998_at	Region	Jmjd1c	108829	10	67223447	67293285	+
5165	1448670_at	Region	Ube2e3	22193	2	78566796	78618332	+
5166	1442014_at	Region	Ifrd1	15982	12	36801602	36821637	-
5167	1425071_s_at	Region	Ntrk3	18213	7	71981888	72367304	-
5168	1418285_at	Region	Efnb1	13641	X	93737491	93750352	+
5169	1426491_at	Region	Herc2	15204	7	50319730	50501273	+
5170	1451576_at	Region	Prkdc	19090	16	14406957	14610487	+
5171	1451380_at	Region	Zfyve19	72008	2	118722806	118730898	+
5172	1419012_at	Region	Zfpm2	22762	15	40545548	40999330	+
5173	1433779_at	Region	Casc4	319996	2	121380983	121449712	+
5174	1435819_at	Region	None	None	NONE	NONE	NONE	NONE
5175	1431009_at	Region	B230219D22Rik	78521	13	54309682	54320058	+
5176	1420478_at	Region	Nap1l1	53605	10	111105541	111122931	+
5177	1421150_at	Region	Hivep3	16656	4	119003584	119157855	+
5178	1436118_at	Region	Ltap	93840	1	171933290	171956187	-
5179	1454991_at	Region	Slc7a1	11987	5	147223681	147291421	-
5180	1418704_at	Region	S100a13	20196	3	90325216	90334995	+
5181	1456051_at	Region	Drd1a	13488	13	52615891	52619894	-
5182	1419266_at	Region	Nfyb	18045	10	82637842	82652084	-
5183	1418042_a_at	Region	Abcc5	27416	16	19102921	19197967	-
5184	1452736_at	Region	1700020M16Rik	71843	14	64013147	64031500	-

5185	1443630_at	Region	Ppp3ca	19055	3	135555655	135820664	+
5186	1428171_at	Region	Prpf39	328110	12	61831742	61841076	+
5187	1424343_a_at	Region	Eif1a	13664	18	46819460	46831964	+
5188	1440011_at	Region	Ext1	14042	15	53068219	53345629	-
5189	1435828_at	Region	None	None	8	114996442	114997174	-
5190	1433920_at	Region	Sema4c	20353	1	36843233	36852240	-
5191	1424473_at	Region	Polr2h	245841	16	19490003	19494442	+
5192	1436471_at	Region	Rab36	76877	10	75138289	75155300	+
5193	1418599_at	Region	Col11a1	12814	3	26642	73590	-
5194	1451069_at	Region	Pim3	223775	15	88914123	88917655	+
5195	1440854_at	Region	2810403A07Rik	74200	3	88429682	88456786	+
5196	1454776_at	Region	Ehmt1	77683	2	24722957	24819233	-
5197	1416516_at	Region	Fscn1	14086	5	142024679	142037166	+
5198	1436172_at	Region	9530028C05	330256	6	3285716	3298593	-
5199	1444956_at	Region	None	None	5	22626399	22627260	+
5200	1424645_at	Region	Tnrc6c	217351	11	117475908	117583657	+
5201	1432978_at	Region	9030607L02Rik	75754	19	20839216	20840808	-
5202	1426207_at	Region	Ikbkb	16150	8	21414342	21461142	-
5203	1440462_at	Region	B930068K11Rik	320622	2	34271421	34271904	-
5204	1442819_at	Region	LOC545239	545239	18	4015000	4015904	+
5205	1440255_at	Region	AA589481	102423	9	44284528	44294482	-
5206	1446345_at	Region	Tbl1x	21372	X	72294199	72320920	+
5207	1451292_at	Region	Zfp212	232784	6	48044784	48056724	+
5208	1449660_s_at	Region	Coro1c	23790	5	112953083	113019350	-
5209	1428131_a_at	Region	Cdc42se1	57912	3	94716611	94724240	+
5210	1421295_at	Region	Chrdl1	83453	X	136738025	136834230	-
5211	1425330_a_at	Region	Ppm1b /// LOC433336	19043	17	82787863	82846969	+
5212	1455714_at	Region	Gm691	277432	2	157371734	157401688	+
5213	1424340_at	Region	Lrrc48	74665	11	60079022	60119975	+
5214	1438000_x_at	Region	Zfp622	52521	15	25973256	25987372	+
5215	1455800_x_at	Region	1110030L07Rik	68653	15	84240839	84264676	+
5216	1450011_at	Region	Hsd17b12	56348	2	93737380	93862592	-
5217	1418414_at	Region	Kcnh1	16510	1	191929938	192245812	+
5218	1428356_at	Region	Osbp2	74309	11	3598519	3616991	-
5219	1427060_at	Region	Mapk3	26417	7	120809363	120815547	+
5220	1416071_at	Region	Ddx18	66942	1	121317046	121331136	-
5221	1436997_x_at	Region	Sh3bgrl	56726	X	103696772	103763832	+
5222	1433647_s_at	Region	Rhobtb3	73296	13	71926694	72000935	-
5223	1427153_at	Region	Bckdhb	12040	9	84278990	84455206	+
5224	1460030_at	Region	Hecw1	94253	13	13672338	13968180	-
5225	1454693_at	Region	LOC545348	545348	1	91756276	91757801	-
5226	1415680_at	Region	Anapc1	17222	2	128126557	128201233	-
5227	1415829_at	Region	Lbr	98386	1	181773496	181800784	-
5228	1433474_at	Region	Edil3	13612	13	84938267	85437638	+
5229	1441587_at	Region	A930027K05Rik	269615	4	153475739	153503344	-
5230	1426727_s_at	Region	Ppp1r10 /// LOC545202	52040	17	33636151	33651436	+
5231	1436548_at	Region	1810012P15Rik	66274	11	78552254	78563925	+
5232	1418640_at	Region	Sirt1	93759	10	63283769	63303800	-
5233	1429725_at	Region	Atbf1	11906	8	108012331	108254925	+
5234	1460169_a_at	Region	Pctk1	18555	X	18927433	18938817	+
5235	1416211_a_at	Region	Ptn	19242	6	36850201	36945899	-
5236	1428172_at	Region	Prpf39	328110	12	61831742	61841076	+
5237	1427546_at	Region	Abca8b	27404	11	109755361	109816905	-
5238	1451912_a_at	Region	Fgfrl1	116701	5	107770717	107783113	+
5239	1419032_at	Region	2610018G03Rik	70415	X	45362232	45412652	+
5240	1445351_at	Region	None	None	4	150234402	150235063	-
5241	1420033_s_at	Region	Ppp2r2d	52432	7	133232628	133269299	+
5242	1426117_a_at	Region	Slc19a2	116914	1	164168307	164184621	+
5243	1448763_at	Region	Atad1	67979	19	31998709	32038444	-
5244	1423391_at	Region	Git2	26431	5	113837037	113883117	-
5245	1428494_a_at	Region	Polr2i	69920	7	25645252	25646692	+
5246	1456475_s_at	Region	Prkar2b	19088	12	28534800	28637055	-
5247	1427917_s_at	Region	Ssbp3	72475	4	105870375	106008599	+

5248	1428903_at	Region	3110037I16Rik	73172	4	119943646	119947449	-
5249	1438399_at	Region	Pex2	58869	3	32358790	32491207	-
5250	1420954_a_at	Region	Add1	11518	5	33061487	33119289	+
5251	1452243_at	Region	Kcnj14	211480	7	39901348	39909502	-
5252	1428014_at	Region	Hist1h4h	69386	13	23011114	23011583	+
5253	1448641_at	Region	Mbtd1	103537	11	93733496	93766406	+
5254	1421276_a_at	Region	Dst	13518	1	34280938	34577763	+
5255	1455859_at	Region	A330021E22Rik	207686	5	5586987	5670237	-
5256	1430307_a_at	Region	Mod1	17436	9	86923622	87038833	-
5257	1420013_s_at	Region	Lss	16987	10	76632617	76654924	+
5258	1418758_a_at	Region	Pscd3	19159	5	142683118	142770834	+
5259	1417631_at	Region	Mknk1	17346	4	114798151	114838153	+
5260	1425388_a_at	Region	Tpk1	29807	6	43507611	43830739	-
5261	1418131_at	Region	Samhd1	56045	2	156554498	156592191	-
5262	1415785_a_at	Region	Cct8	12469	16	86632779	86645309	-
5263	1445322_x_at	Region	E430025E21Rik	223593	15	59342534	59384689	-
5264	1460293_at	Region	Freq	14299	2	31178085	31227634	+
5265	1418861_at	Region	Pias4	59004	10	81288763	81303101	-
5266	1425693_at	Region	Braf	109880	6	39749542	39860924	-
5267	1448166_a_at	Region	Psmb1	19170	17	13479611	13501932	-
5268	1453406_a_at	Region	Rab28	100972	5	40380642	40464106	-
5269	1427139_at	Region	Adamts10	224697	17	31241547	31249680	+
5270	1457367_at	Region	None	None	3	32123901	32124479	+
5271	1445095_at	Region	None	None	13	108914407	108915065	-
5272	1443871_at	Region	Gm1649	381459	3	100111350	100112984	-
5273	1439614_at	Region	None	None	18	62731288	62731768	+
5274	1423468_at	Region	Steap3	68428	1	119985615	120002678	-
5275	1423153_x_at	Region	Cfh	12628	1	139935794	140062362	-
5276	1435441_at	Region	Ablim2	231148	5	34245478	34371978	+
5277	1444487_at	Region	Lrat	79235	3	82624549	82633119	-
5278	1453256_at	Region	Polr3c	74414	3	96199326	96215464	-
5279	1415791_at	Region	Rnf34	80751	5	122003097	122021854	+
5280	1446849_at	Region	None	None	NONE	NONE	NONE	NONE
5281	1439975_at	Region	BC062109	231503	5	99096511	99178447	-
5282	1418670_s_at	Region	None	None	4	136350082	136451892	+
5283	1423208_at	Region	0610041E09Rik	66074	13	86207541	86225129	+
5284	1442155_at	Region	4632427E13Rik	68186	7	86774252	86775226	-
5285	1446541_at	Region	4930434E21Rik	381693	5	148466926	148550142	+
5286	1456146_at	Region	2210411A11Rik	72358	9	115215989	115216963	+
5287	1441577_at	Region	C530014P21Rik	399604	17	60553133	60553742	-
5288	1433040_at	Region	2900018E21Rik	72943	10	90568957	90570486	+
5289	1425391_a_at	Region	Osbpl5	79196	7	138101781	138155802	-
5290	1429961_at	Region	1700021C14Rik	76927	3	88026624	88040863	-
5291	1433969_at	Region	AU067824	106734	17	78354275	78354760	-
5292	1431371_at	Region	9030411M13Rik	71535	10	124915295	124917383	-
5293	1417177_at	Region	Galk1	14635	11	115829619	115833771	-
5294	1450914_at	Region	Ppp1r14b	18938	19	6688834	6691170	+
5295	1454736_at	Region	4921515A04Rik	268301	10	59180836	59183260	+
5296	1436067_at	Region	Zbtb10	229055	3	9111529	9270577	+
5297	1452753_at	Region	Foxk2	68837	11	121080635	121128991	+
5298	1430452_at	Region	Cyp20a1	77951	1	60646611	60675997	+
5299	1448919_at	Region	Cd302	66205	2	60107393	60139818	-
5300	1423416_at	Region	Smarcc1	20588	9	110159869	110278595	+
5301	1456779_a_at	Region	4930414L22Rik	78108	6	72772008	72772590	+
5302	1426472_at	Region	Zfp52	22710	17	19404937	19434146	+
5303	1431199_at	Region	0610031G08Rik	75393	17	24813037	24817405	+
5304	1444557_at	Region	None	None	1	59945208	59945661	-
5305	1446720_at	Region	Alcam	11658	16	51168811	51372049	-
5306	1427893_a_at	Region	Pmvk	68603	3	89216043	89225732	+
5307	1453310_at	Region	Ppil6	73075	10	41592302	41616124	+
5308	1442787_at	Region	None	None	1	60784904	60785232	+
5309	1452093_at	Region	2500001K11Rik	226351	1	119277601	119279660	+
5310	1418134_at	Region	1200007B05Rik	67453	18	31819928	31849663	-
5311	1448427_at	Region	Ndufa6	67130	15	82400880	82404959	-
5312	1421448_at	Region	Garnl1	56784	12	52323288	52540884	-

5313	1419912_s_at	Region	Strap	20901	6	138527082	138543054	+
5314	1434370_s_at	Region	Faf1	14084	4	108635532	108922865	+
5315	1457024_x_at	Region	Slc35a2	22232	X	6123184	6133345	+
5316	1437289_at	Region	1110001C20Rik	242291	4	4691488	4720452	-
5317	1437354_at	Region	None	None	7	53585497	53587990	+
5318	1452289_a_at	Region	Rnf135	71956	11	79909531	79925412	+
5319	1436629_at	Region	A830041P22Rik	320208	7	20833839	20839866	-
5320	1417358_s_at	Region	Sorbs1	20411	19	39839082	40058071	-
5321	1451309_at	Region	Arhgap1	228359	2	91357957	91377067	+
5322	1445846_at	Region	None	None	7	50987935	50988845	-
5323	1456824_at	Region	None	None	8	109389844	109390417	+
5324	1444295_at	Region	Neo1	18007	9	58995038	59154898	-
5325	1439702_at	Region	Myt1l	17933	12	26094752	26491102	+
5326	1426065_a_at	Region	Trib3	228775	2	151794358	151800968	-
5327	1451572_a_at	Region	5230400G24Rik	75734	1	83054024	83081496	+
5328	1433703_s_at	Region	Bahd1	228536	2	118415306	118438363	+
5329	1420412_at	Region	Tnfsf10	22035	3	26734898	26753894	+
5330	1423586_at	Region	Axl	26362	7	20922056	20953324	-
5331	1429284_at	Region	8430436F23Rik	71559	4	35088141	35089705	-
5332	1455126_x_at	Region	2310028O11Rik	433771	4	137985045	137985274	-
5333	1449055_x_at	Region	Pcbp4	59092	9	106517872	106528323	+
5334	1446508_at	Region	None	None	15	68367427	68367967	-
5335	1415737_at	Region	Rfk	54391	19	16618183	16625435	+
5336	1436089_at	Region	Ddx26	18130	14	57209161	57294009	-
5337	1434777_at	Region	Lmyc1	16918	4	122023211	122029597	+
5338	1455772_at	Region	Pgr	18667	9	8911412	8976391	+
5339	1435200_at	Region	6330419J24Rik	320237	X	51129329	51132510	+
5340	1423922_s_at	Region	C77668	229543	3	90201806	90244054	-
5341	1429590_at	Region	Tacc1	320165	8	23877518	23925420	-
5342	1418258_s_at	Region	6720463E02Rik	68097	11	87707525	87715497	-
5343	1428156_at	Region	Gng2	14702	14	18235904	18323407	-
5344	1424810_at	Region	4930485D02Rik	75812	2	139347439	139580613	-
5345	1435324_x_at	Region	Hmgb1 /// LOC213079 /// LOC433238 /// LOC433305 /// LOC434174	15289	5	147944154	147949960	-
5346	1420429_at	Region	Pcdhb3	93874	18	37524705	37527701	+
5347	1446386_at	Region	Tcf4	21413	18	69575005	69915006	+
5348	1420704_at	Region	Csf2ra	12982	11	15	635	+
5349	1448244_at	Region	Lypla1	18777	1	4768285	4807127	+
5350	1429321_at	Region	Rnf149	67702	1	39848213	39874285	-
5351	1452041_at	Region	C630013N10Rik	234378	8	69605293	69631982	-
5352	1437313_x_at	Region	Hmgb2 /// LOC433785 /// LOC433788 /// LOC433799 /// LOC545710	433785	4	144644465	144645615	-
5353	1453266_at	Region	Zbtb4	75580	11	69492223	69505059	+
5354	1417741_at	Region	Pygl	110095	12	67022972	67059778	-
5355	1454615_x_at	Region	Srp14	20813	2	117989699	117993510	-
5356	1434538_x_at	Region	Eif2b2	217715	12	82088696	82095811	+
5357	1451555_at	Region	Nln	75805	13	100241399	100327761	-
5358	1419821_s_at	Region	Idh1	15926	1	65452617	65473274	-
5359	1440496_at	Region	None	None	15	84265156	84265770	+
5360	1444615_x_at	Region	Cbfa2t1h	12395	4	13711747	13818082	+
5361	1435062_at	Region	1500001A10Rik	68955	5	115563065	115714339	-
5362	1451808_at	Region	Kcnj4	16520	15	79536173	79557665	-
5363	1420990_at	Region	Chd1	12648	17	13708827	13773901	+
5364	1426184_a_at	Region	Pdcd6ip	18571	9	113708127	113760882	-
5365	1416601_a_at	Region	Dscr1	54720	16	91551471	91559574	-
5366	1417852_x_at	Region	Clca1	12722	3	143701991	143732414	-
5367	1459666_at	Region	AI413908	102495	9	121478609	121479049	+
5368	1424182_at	Region	Acat1	110446	9	53644099	53673628	-
5369	1436351_at	Region	Coq3	230027	4	21949046	21981497	+

5370	1434560_at	Region	Wdtc1	230796	4	132253365	132300004	-
5371	1460000_at	Region	D830007B15Rik	330096	5	66374549	66380118	+
5372	1456695_x_at	Region	Anapc5	59008	5	121940636	121974194	-
5373	1439482_at	Region	F830021D11Rik	224648	17	25651716	25693135	+
5374	1455981_at	Region	Rps6 /// LOC214738 /// LOC216036 /// LOC236932 /// LOC434404 /// LOC545640	20104	4	85840854	85843549	-
5375	1449025_at	Region	Ifit3	15959	19	33932821	33937973	+
5376	1456096_at	Region	6430573F11Rik	319582	8	35293919	35317741	+
5377	1440230_at	Region	9530051K01Rik	244152	7	92397408	92398404	-
5378	1421374_a_at	Region	Fxyd1	56188	7	26460006	26463982	-
5379	1431074_a_at	Region	Pitpnc1	71795	11	107033195	107291788	-
5380	1418584_at	Region	Ccnh	66671	13	81281211	81305457	+
5381	1440883_at	Region	None	None	2	6362314	6363663	+
5382	1416205_at	Region	Glb1	12091	9	114364315	114438701	+
5383	1453722_s_at	Region	Sfrs1	110809	11	87775771	87779641	+
5384	1441453_at	Region	B930096L08Rik	209773	6	39597808	39693264	-
5385	1428672_at	Region	Snrpf	69878	4	148807469	148807918	-
5386	1453852_at	Region	8430408E17Rik	71464	10	62589317	62590270	-
5387	1428165_at	Region	Vps24	66700	6	71875831	71913551	+
5388	1422572_at	Region	Rhog	56212	7	96345084	96356054	-
5389	1442174_at	Region	Tspan18	241556	2	92906443	93039133	-
5390	1454404_at	Region	2610011E03Rik	72460	5	131185791	131187998	-
5391	1449075_at	Region	4930539P14Rik	97827	12	77337250	77359377	+
5392	1457464_at	Region	C130015C19	403342	13	53896405	53897224	+
5393	1452897_at	Region	Cdc2l5	69562	13	17181756	17269467	-
5394	1456581_x_at	Region	Gdi3	14569	13	3422528	3450201	+
5395	1443660_at	Region	None	None	NONE	NONE	NONE	NONE
5396	1420136_a_at	Region	None	None	1	23534126	23534479	+
5397	1426124_a_at	Region	Clk1	12747	1	58716755	58728765	-
5398	1415735_at	Region	Ddb1	13194	19	9802122	9826319	+
5399	1438389_x_at	Region	Acly	104112	11	100297443	100348988	-
5400	1449085_at	Region	Phf10	72057	17	12997725	13044853	-
5401	1428438_s_at	Region	2700023B17Rik	67070	7	29498435	29543405	-
5402	1442269_at	Region	None	None	18	68004512	68005173	-
5403	1431116_at	Region	C030003D03Rik	77220	10	25982477	26078288	-
5404	1458147_at	Region	Mamdc1	320772	12	63250963	63650479	-
5405	1417770_s_at	Region	None	None	14	11426901	11490818	-
5406	1444827_at	Region	Btbd9	224671	17	28030141	28340405	-
5407	1451675_a_at	Region	Alas2	11656	X	144081840	144105014	+
5408	1436360_at	Region	Hkr2	232878	7	10891496	10901920	+
5409	1420476_a_at	Region	Nap1l1	53605	10	111105541	111122931	+
5410	1445546_at	Region	AI844685	106267	3	156619633	156620192	+
5411	1448261_at	Region	Cdh1	12550	8	105899005	105965883	+
5412	1420707_a_at	Region	Traip	22036	9	108018950	108040255	+
5413	1440864_at	Region	AI836737	103288	10	32939882	32940562	+
5414	1423663_at	Region	Flcn	216805	11	59517379	59535402	-
5415	1415921_a_at	Region	Tnfrsf19	29820	14	55499179	55581612	-
5416	1416850_s_at	Region	D10Ertd214e	52637	10	71384461	71398816	-
5417	1443184_at	Region	Cdc14a	229776	3	115042345	115193064	-
5418	1445984_at	Region	Pftk1	18647	5	4809738	5386208	-
5419	1455686_at	Region	None	None	5	44455827	44456994	-
5420	1450056_at	Region	Apc	11789	18	34484045	34541630	+
5421	1433620_at	Region	None	None	13	91181251	91207437	+
5422	1428969_at	Region	D030074E01Rik	75964	18	21032145	21044024	-
5423	1440850_at	Region	Rnf12	19820	X	98558532	98582649	-
5424	1431861_a_at	Region	D3Ertd250e	52184	3	144090760	144127134	+
5425	1421830_at	Region	Ak3l1	11639	4	100377949	100425900	+
5426	1456580_s_at	Region	Atp5d	66043	10	80265814	80269260	+
5427	1443917_at	Region	0610007H07Rik	68212	10	119786523	119802594	+
5428	1431429_a_at	Region	Arl4	11861	12	36632028	36636075	-
5429	1418458_at	Region	Anapc7	56317	5	121574831	121596929	+

5430	1455961_at	Region	Mme	17380	3	62963798	63050157	+
5431	1455668_at	Region	6720429000	330725	8	24443658	24444733	+
5432	1424851_at	Region	Chchd5	66170	2	128643675	128647960	+
5433	1439108_at	Region	Mll5	69188	5	21950220	21960092	+
5434	1417730_at	Region	Ext1	14042	15	53068219	53345629	-
5435	1433513_x_at	Region	Ndufa12	66414	10	94172393	94194766	+
5436	1453028_at	Region	4631424J17Rik	70802	11	43434843	43460028	+
5437	1418533_s_at	Region	Fzd2	57265	11	102425622	102427671	+
5438	1450074_at	Region	Kif3b	16569	2	152748365	152789150	+
5439	1435787_at	Region	Ppm1l	242083	3	68987383	69225607	+
5440	1426266_s_at	Region	Zbtb8os	67106	4	128363139	128374142	+
5441	1460708_s_at	Region	Cdc42	12540	4	136200964	136217395	-
5442	1423229_at	Region	Inpp5e	64436	2	26328411	26341350	-
5443	1429117_at	Region	Tradd	71609	8	104553932	104560238	-
5444	1453229_s_at	Region	Uqcrh	66576	4	115025870	115033953	-
5445	1448755_at	Region	Col15a1	12819	4	47124115	47229087	+
5446	1441505_at	Region	None	None	3	90106118	90106624	+
5447	1439120_at	Region	BC010304	218236	13	48508119	48513447	-
5448	1419644_at	Region	Cstf2	108062	X	127604288	127631760	+
5449	1438543_at	Region	Spata13	219140	14	55169182	55299693	+
5450	1421097_at	Region	Endog	13804	2	30103686	30106231	+
5451	1438628_x_at	Region	Cntn3	18488	6	102641686	102940436	-
5452	1460630_at	Region	Camk2d	108058	3	125480582	125726370	+
5453	1433832_at	Region	B230369L08Rik	223697	15	79776391	79794850	-
5454	1449019_at	Region	Akap1	11640	11	88652757	88672590	-
5455	1439890_at	Region	Fbxl7	448987	15	26532728	26884891	-
5456	1431375_s_at	Region	Parva	57342	7	106281338	106445320	+
5457	1460308_a_at	Region	Ict1	68572	11	115224859	115232006	+
5458	1423333_at	Region	1200007D18Rik	67458	17	24356346	24451768	+
5459	1418389_at	Region	2810453I06Rik	67238	5	142607596	142623478	+
5460	1416037_a_at	Region	Cct2	12461	10	116740451	116753217	-
5461	1426361_at	Region	Zc3h11a	70579	1	133482838	133516350	-
5462	1448790_at	Region	Sema6b	20359	17	53760130	53769910	-
5463	1432205_a_at	Region	C130038G02Rik /// mKIAA0774	545821	5	147156887	147205334	+
5464	1457799_at	Region	Kcns3	238076	12	10443638	10503809	-
5465	1436752_at	Region	5730478M09Rik	70573	16	21596550	21641135	-
5466	1420799_at	Region	Ntsr1	18216	2	180216934	180261937	+
5467	1417418_s_at	Region	Cox6a1	12861	5	114456220	114459517	-
5468	1454972_at	Region	Atcay	16467	10	81340453	81366832	-
5469	1439664_at	Region	Wdr68	71833	11	105857961	105876453	+
5470	1437345_a_at	Region	Bscl2	14705	19	8036050	8045214	+
5471	1426821_at	Region	Cog8	97484	8	106338850	106354346	-
5472	1417625_s_at	Region	Cmkor1	12778	1	90022553	90034838	+
5473	1441248_at	Region	None	None	8	59975938	59976351	-
5474	1422258_at	Region	Chrm3	12671	13	9860822	10353284	-
5475	1460224_at	Region	Snx2	67804	18	53394494	53438989	+
5476	1434135_at	Region	B3galnt2	97884	13	13397005	13441487	+
5477	1437550_at	Region	Dhx36	72162	3	62137118	62174023	-
5478	1416263_at	Region	Abcb9	56325	5	123233182	123267116	-
5479	1422594_at	Region	5730470L24Rik	66641	3	102423707	102431881	+
5480	1435400_at	Region	1500004F05Rik	69765	8	39328856	39329952	+
5481	1418727_at	Region	Nup155	170762	15	7896928	7946387	+
5482	1423840_at	Region	D11Ertd99e	52469	11	101099057	101100035	-
5483	1450097_s_at	Region	Gna12	14673	5	139758776	139829256	-
5484	1434865_a_at	Region	Exoc7	53413	11	116110276	116127766	-
5485	1450478_a_at	Region	Ptpn12	19248	5	19439215	19508367	-
5486	1423310_at	Region	Tpbg	21983	9	86173432	86176811	+
5487	1421354_at	Region	Prkg2	19092	5	97950831	98055222	-
5488	1416215_at	Region	Gosr1	53334	11	76452260	76489213	-
5489	1452066_a_at	Region	Ndfip2	76273	14	99801590	99852059	+
5490	1432156_a_at	Region	Rnf32	56874	5	27646557	27676001	+
5491	1418403_at	Region	Adam19	11492	11	45808889	45900180	+
5492	1425148_a_at	Region	Snx6	72183	12	0	675	-
5493	1446329_at	Region	Gkap1	56278	13	56862150	56893001	-

5494	1448736_a_at	Region	Hprt1	15452	X	47508933	47542455	+
5495	1454891_at	Region	Cds2	110911	2	131777151	131821546	+
5496	1422797_at	Region	MGI:1932697	83409	3	88293683	88296791	-
5497	1416011_x_at	Region	Ehd1	13660	19	6065686	6088886	+
5498	1447211_at	Region	Nrip1	268903	16	75376752	75458939	-
5499	1456383_at	Region	Rsl1d1	66409	16	10439299	10449554	+
5500	1431110_at	Region	5430431D22Rik	74506	2	16679533	16682213	+
5501	1434442_at	Region	D5Ertd593e	52331	5	91938800	91942328	+
5502	1423230_at	Region	Inpp5e	64436	2	26328411	26341350	-
5503	1421026_at	Region	Gna12	14673	5	139758776	139829256	-
5504	1440273_at	Region	LOC545753	545753	5	63599806	63601660	-
5505	1460740_at	Region	Cltb	74325	13	53203300	53220857	-
5506	1415744_at	Region	H2-Ke2	14976	17	31642666	31644048	-
5507	1434750_at	Region	Sec6l1	211446	13	70234117	70270772	-
5508	1460303_at	Region	Nr3c1	14815	18	39638075	39711047	-
5509	1426487_a_at	Region	Rbbp6	19647	7	116848797	116880431	+
5510	1451744_a_at	Region	Zadh1	77219	12	81153770	81182471	+
5511	1436244_a_at	Region	Tle2	21886	10	81711856	81727530	+
5512	1426891_at	Region	Rpap1	68925	2	119277813	119301347	-
5513	1437111_at	Region	C230027N18Rik	330940	9	52181537	52183610	-
5514	1456135_s_at	Region	Pxn	19303	5	114657142	114667534	+
5515	1455752_a_at	Region	2410001H17Rik	66990	19	3914777	3921075	+
5516	1422256_at	Region	Sstr2	20606	11	113440569	113446988	+
5517	1419000_at	Region	Cpxm2	55987	7	126406479	126517151	-
5518	1437479_x_at	Region	Tbx3	21386	5	118821012	118834673	+
5519	1441645_s_at	Region	Ipo9	226432	1	135234946	135287679	-
5520	1435122_x_at	Region	Dnmt1	13433	9	20784243	20829937	-
5521	1435719_at	Region	AI448984	100124	4	34718484	34719775	+
5522	1429737_a_at	Region	0610009I22Rik	66586	2	132361587	132380004	+
5523	1448893_at	Region	Ncor2	20602	5	124178459	124286279	-
5524	1429329_at	Region	Cox10	70383	11	63689518	63805064	-
5525	1438538_at	Region	D630021H01Rik	319914	X	68741703	68742351	-
5526	1451294_s_at	Region	Snrpe	20643	1	133465225	133471606	-
5527	1460082_at	Region	Ing3	71777	6	21996682	22023073	+
5528	1434059_at	Region	B230312A22Rik	230088	4	42948432	42962125	-
5529	1447205_x_at	Region	Rps12 /// LOC432865 /// LOC434839 /// LOC434843 /// LOC546370 /// LOC546371 /// LOC546372 /// LOC546373	20042	10	58921849	58922343	+
5530	1455703_at	Region	Akt2	11652	7	22980718	23014488	+
5531	1423583_at	Region	Fem1a	14154	17	53893863	53898116	+
5532	1455534_s_at	Region	Osbpl11	106326	16	31993123	32051348	+
5533	1437967_at	Region	Ttn	22138	2	76707652	76708282	-
5534	1424488_a_at	Region	1110013G13Rik	74776	3	132200512	132268465	+
5535	1434294_at	Region	None	None	1	122641244	122641972	+
5536	1428454_at	Region	Bcas3	192197	11	85081206	85554043	+
5537	1415742_at	Region	Aup1	11993	6	13227949	13231164	+
5538	1433935_at	Region	AU020206	101757	NONE	NONE	NONE	NONE
5539	1428231_at	Region	Cpsf6	432508	10	117034119	117066391	-
5540	1460221_at	Region	Ptges3	56351	10	127795692	127813964	+
5541	1424915_s_at	Region	None	None	12	83821014	83839147	+
5542	1416542_at	Region	Phf1	21652	17	24728395	24732952	+
5543	1438368_a_at	Region	Matr3	17184	18	35795510	35815074	+
5544	1456045_at	Region	1700106N22Rik	73582	17	82920574	83288888	+
5545	1433503_at	Region	Zadh1	77219	12	81153770	81182471	+
5546	1424980_s_at	Region	Aph1a	226548	3	95381828	95386135	+
5547	1453017_at	Region	D5Ertd585e	71782	5	109281825	109307432	+
5548	1448007_at	Region	5830435C13Rik	407823	2	59787511	59817573	-
5549	1458441_at	Region	Slc6a17	229706	3	107263377	107313847	-
5550	1440385_at	Region	9530051E23Rik	319699	3	69429902	69430806	+
5551	1427273_at	Region	D130054N24Rik	235315	9	45876943	45920129	-

5552	1452756_at	Region	0610009J22Rik	71673	11	4029989	4035959	+
5553	1447685_x_at	Region	Ets2	23872	16	95110358	95128440	+
5554	1418577_at	Region	Trim8	93679	19	46050008	46064111	+
5555	1439583_x_at	Region	D130029J02Rik	234695	8	104986097	104993919	+
5556	1429103_at	Region	Tomm22	223696	15	79723188	79725182	+
5557	1416380_at	Region	Mov10	17454	3	104221617	104245068	-
5558	1439598_at	Region	Foxo3	56484	10	42292466	42383422	-
5559	1449316_at	Region	Cyp4f15	106648	17	30509817	30527507	+
5560	1455214_at	Region	Mitf	17342	6	98457025	98483990	+
5561	1429396_at	Region	2410118P20Rik	73683	7	95396169	95417540	-
5562	1446497_at	Region	Nav1	215690	1	135289346	135436181	-
5563	1455159_at	Region	2900057D21Rik	72993	14	25057193	25108756	-
5564	1454739_at	Region	Cdc27	217232	11	104326687	104371504	-
5565	1444980_at	Region	C730009D12	328974	18	64623966	64624844	+
5566	1429529_at	Region	Tsen54	76265	11	115635803	115644182	+
5567	1456745_x_at	Region	Sdbcag84	66366	2	155465054	155475216	+
5568	1438698_at	Region	4632425D07Rik	208213	5	126411362	126733552	+
5569	1441097_at	Region	None	None	3	101582184	101583119	-
5570	1420916_at	Region	Fnbp3	56194	2	53070637	53123349	-
5571	1441682_s_at	Region	Xpot	73192	10	121243639	121282407	-
5572	1438982_s_at	Region	2810417J12Rik	76917	17	21579098	21588259	-
5573	1424539_at	Region	Ubl4	27643	X	69028056	69029182	-
5574	1453022_at	Region	MGI:1915703	68453	15	75646572	75648127	+
5575	1440443_at	Region	E030016H06Rik	402722	2	132072859	132073496	-
5576	1433681_x_at	Region	Capn3	12335	2	119977722	120018348	+
5577	1451489_at	Region	Slc25a35	71998	11	68693789	68698173	+
5578	1434439_at	Region	Gsk3b	56637	16	36906890	37063968	+
5579	1437552_at	Region	2410127L17Rik	67383	19	17917778	17951174	+
5580	1445703_at	Region	AI848149	102944	X	149196633	149196936	-
5581	1451039_at	Region	2610027L16Rik	67842	14	50263729	50272080	+
5582	1446091_at	Region	Anp32b	67628	4	46367037	46388445	+
5583	1420912_at	Region	Aff4	93736	11	53103673	53174681	+
5584	1452773_at	Region	5730494N06Rik	70612	2	131753347	131761635	-
5585	1458737_at	Region	C77097	98105	18	21392040	21392910	-
5586	1452303_at	Region	Arhgef10	234094	8	14307382	14378920	+
5587	1435349_at	Region	Nrp2	18187	1	62996720	63108906	+
5588	1438506_s_at	Region	Tprt	56075	2	22857432	22877326	+
5589	1421858_at	Region	Adam17	11491	12	18771955	18822033	+
5590	1457043_at	Region	Gm1057	381694	5	148692189	148700421	+
5591	1415833_x_at	Region	Rps29	20090	12	65989514	65990859	-
5592	1422540_at	Region	Fbln1	14114	15	85254610	85334255	+
5593	1441687_at	Region	Wnt4	22417	4	136158896	136178040	+
5594	1444780_at	Region	5330421F07Rik	78286	7	43479111	43568752	+
5595	1436736_x_at	Region	D0H4S114	27528	18	33660051	33686464	-
5596	1423930_at	Region	Anapc4	52206	5	51644274	51676873	+
5597	1434822_at	Region	Pphln1	223828	15	93464569	93557721	+
5598	1436242_a_at	Region	Cklf	75458	8	103545239	103557779	+
5599	1447484_x_at	Region	2610002F03Rik	72091	2	26571927	26572166	-
5600	1436733_at	Region	E130309F12Rik	272031	4	48975384	49256135	+
5601	1429422_at	Region	4933412E12Rik	71086	10	116640022	116652332	+
5602	1454766_at	Region	C730024G19Rik	232566	6	150093461	150124565	-
5603	1428055_at	Region	Rian	75745	12	105129924	105131988	+
5604	1440325_at	Region	None	None	7	86884930	86885521	+
5605	1455751_at	Region	Cand1	71902	10	118778384	118938138	-
5606	1443880_at	Region	Zbtb39	320080	10	127476248	127484059	+
5607	1436616_at	Region	R74740	96938	1	37704144	37705174	+
5608	1452105_a_at	Region	Tsc2	22084	17	22400105	22436183	-
5609	1451406_a_at	Region	Pcsk5	18552	19	16656932	17062422	-
5610	1444485_at	Region	None	None	6	101134893	101135531	-
5611	1415892_at	Region	Sgpl1	20397	10	61060660	61110932	-
5612	1450071_at	Region	Ash1l	192195	3	88709675	88822741	+
5613	1458344_at	Region	Nagpa	27426	16	4864956	4873675	-
5614	1426489_s_at	Region	Bfar	67118	16	12408146	12438648	+
5615	1418843_at	Region	Slc30a4	22785	2	122195092	122216516	-
5616	1451450_at	Region	2010011I20Rik	67017	2	171854342	171864482	+

5617	1448733_at	Region	Bmi1	12151	2	18719028	18728639	+
5618	1425459_at	Region	Mtmr2	77116	9	13585449	13642846	+
5619	1420008_s_at	Region	BC037006	211652	11	35632434	35633256	-
5620	1422623_x_at	Region	Rpl41	67945	10	128285207	128286206	-
5621	1418938_at	Region	Dio2	13371	12	86128063	86141948	-
5622	1436502_at	Region	Mtus1	102103	8	39926813	40021115	-
5623	1434994_at	Region	Dedd	21945	1	171267091	171270454	+
5624	1453527_a_at	Region	Neurl	18011	19	46730295	46810735	+
5625	1441155_at	Region	2810446P07Rik	72745	13	80317260	80390898	+
5626	1416336_s_at	Region	Snrpd1	20641	18	10663140	10673574	+
5627	1426984_at	Region	2310067B10Rik	71947	11	115586611	115620126	+
5628	1422553_at	Region	Pten	19211	19	32083643	32146174	+
5629	1428700_at	Region	P2ry13	74191	3	58857895	58860871	-
5630	1438246_at	Region	Csnk1g1	214897	9	66032337	66163764	+
5631	1419353_at	Region	Dpm1	13480	2	167717793	167739104	-
5632	1427254_at	Region	Zfp445	235682	9	122869105	122879937	-
5633	1451569_at	Region	Nr2c2	22026	6	92539374	92616647	+
5634	1450387_s_at	Region	Ak3l1	11639	4	100377949	100425900	+
5635	1422573_at	Region	Ampd3	11717	7	104630686	104662745	+
5636	1422555_s_at	Region	Gna13	14674	11	109184029	109219306	+
5637	1449733_s_at	Region	Siah1a	20437	8	86007609	86029536	-
5638	1451971_at	Region	Cul4a	99375	8	12484378	12526411	+
5639	1435916_at	Region	2210410P13Rik	75684	7	25195304	25196136	+
5640	1418585_at	Region	Ccnh	66671	13	81281211	81305457	+
5641	1450479_x_at	Region	Ptpn12	19248	5	19439215	19508367	-
5642	1437394_at	Region	Centg2	347722	1	89273393	89708522	+
5643	1425493_at	Region	Bmpr1a	12166	14	32547251	32638735	-
5644	1425615_a_at	Region	Pck2	74551	14	50057801	50067552	+
5645	1431364_a_at	Region	2810047C21Rik	72716	7	6837010	7126349	+
5646	1428399_a_at	Region	4930438O05Rik	78795	1	85961103	86084595	+
5647	1441192_at	Region	Scly	50880	1	91121380	91144116	+
5648	1456501_at	Region	2610528E23Rik	66497	16	56210874	56232420	-
5649	1452052_s_at	Region	Eif3s1	78655	2	121561316	121567484	+
5650	1452043_at	Region	2310011J03Rik	66374	10	80443263	80445524	-
5651	1460562_at	Region	Eftud1	101592	7	76505164	76634375	+
5652	1448282_at	Region	Plrg1	53317	3	82784505	82801258	+
5653	1416834_x_at	Region	Ndufb2	68198	6	39728013	39734901	+
5654	1447139_at	Region	Bcl7c	12055	7	121754709	121758497	-
5655	1446304_at	Region	None	None	5	104869313	104869985	+
5656	1442587_at	Region	C130076O07Rik	319504	12	40985533	41346376	+
5657	1446235_at	Region	None	None	3	126705757	126706414	-
5658	1428607_at	Region	None	None	X	19087499	19091832	+
5659	1458330_x_at	Region	Cnnm3	94218	1	36807398	36820275	+
5660	1449236_at	Region	Dll3	13389	7	23691404	23699388	-
5661	1444779_s_at	Region	Zfp59	22717	7	23185040	23248345	+
5662	1455629_at	Region	Drd1a	13488	13	52615891	52619894	-
5663	1427111_s_at	Region	1300006N24Rik	71766	9	20950505	20975490	-
5664	1416315_at	Region	Abhd4	105501	14	48779225	48789095	+
5665	1449530_at	Region	Trps1	83925	15	50659276	50888038	-
5666	1428550_at	Region	1810015A11Rik	69101	16	15917957	15919874	+
5667	1437833_at	Region	Ltbp3	16998	19	5530917	5547315	+
5668	1416095_x_at	Region	1110005A23Rik	66118	10	128559009	128614707	+
5669	1460548_a_at	Region	Eral1	57837	11	77799035	77806004	-
5670	1442899_at	Region	None	None	5	135927893	135928527	+
5671	1460342_s_at	Region	AA536749	26936	11	59388161	59501851	+
5672	1439181_at	Region	BC043301	210104	7	37639789	37650611	+
5673	1453715_at	Region	Sv2c	75209	13	92166381	92323903	-
5674	1455613_at	Region	E130308A19Rik	230259	4	59569847	59664423	+
5675	1452581_at	Region	2610304G08Rik	70470	2	157485763	157534289	+
5676	1426698_a_at	Region	Hnrpm	76936	17	31345420	31384514	-
5677	1451745_a_at	Region	Znhit1	70103	5	135995323	136001004	-
5678	1429066_at	Region	4930565B19Rik	75325	5	121391846	121392547	-
5679	1427505_a_at	Region	Cradd	12905	10	95150586	95300324	-
5680	1441481_at	Region	Mfap3l	71306	8	59692537	59709342	+
5681	1421054_at	Region	Xpo4	57258	14	52117369	52199998	-

5682	1448632_at	Region	Psmb10	19171	8	105231611	105233952	-
5683	1423864_at	Region	Nrbp	192292	5	29699856	29710501	+
5684	1437419_at	Region	Bmp2k	140780	5	96015785	96109144	+
5685	1431133_at	Region	Arhgap18	73910	10	26772308	26918428	+
5686	1426862_at	Region	9130023F12Rik	216549	11	20579877	20636277	-
5687	1423794_at	Region	D2Ertd391e	51897	2	91379374	91415325	-
5688	1428078_at	Region	0610013E23Rik	76892	11	86212649	86226999	+
5689	1416653_at	Region	Stxbp3	20912	3	108588922	108636244	-
5690	1424185_a_at	Region	Morf4l1	21761	9	89979040	90001962	-
5691	1428015_at	Region	D12Wsu118e	28115	NONE	NONE	NONE	NONE
5692	1428783_at	Region	Prkar2a	19087	9	108741435	108795663	+
5693	1424735_at	Region	Slc25a25	227731	2	32346649	32383584	-
5694	1453141_at	Region	0610009L18Rik	66838	11	120169817	120172300	+
5695	1444185_at	Region	Stim2	116873	5	52808660	52930070	+
5696	1426763_at	Region	Oaz2	18247	9	65798855	65812562	+
5697	1415709_s_at	Region	Gbf1	107338	19	45699725	45833852	+
5698	1418030_at	Region	Slco3a1	108116	7	68181298	68451736	-
5699	1458141_at	Region	---	382344	10	20292074	20327184	-
5700	1419397_at	Region	Pola1	18968	X	87965425	88292813	-
5701	1453562_a_at	Region	1110025F24Rik	67824	16	4382757	4390413	-
5702	1427809_at	Region	Lphn3	319387	5	80275281	81049432	+
5703	1425538_x_at	Region	Ceacam1	26365	7	20682164	20703996	-
5704	1441970_at	Region	E430010N07Rik	399572	X	129310000	129311316	+
5705	1417385_at	Region	Npepps	19155	11	97028020	97101650	-
5706	1416588_at	Region	Ptprn	19275	1	75534602	75554785	-
5707	1453122_at	Region	4921533L14Rik	68187	1	24256337	24331230	-
5708	1417001_a_at	Region	D4Wsu53e	27981	4	133884543	133888276	+
5709	1436233_at	Region	AI117581	103629	11	48918423	48918694	-
5710	1451327_a_at	Region	Tmed1	17083	9	21396609	21399241	-
5711	1453079_at	Region	5730494M16Rik	66648	18	25367107	25397117	-
5712	1440976_at	Region	1110035M17Rik	432396	11	95521193	95533826	-
5713	1427891_at	Region	Gimap6	231931	6	48834349	48841010	-
5714	1426295_at	Region	Hapln1	12950	13	85655519	85726506	+
5715	1446861_at	Region	Gns	75612	10	121021344	121053499	+
5716	1430857_s_at	Region	Pex11c	69129	8	4142	7868	+
5717	1426879_at	Region	1190005F20Rik	98685	1	151291502	151313933	+
5718	1434246_at	Region	L3mbtl3	237339	10	26275100	26374931	-
5719	1439501_at	Region	Cbara1	216001	10	59664687	59819557	+
5720	1424222_s_at	Region	Rad23b	19359	4	55265936	55307091	+
5721	1457252_x_at	Region	Pld2	18806	11	70265822	70283768	+
5722	1434552_at	Region	Wdr77	70465	3	105755327	105765589	+
5723	1423936_at	Region	Kctd5	69259	17	21851732	21877431	-
5724	1434303_at	Region	Raph1	77300	1	60786474	60811912	-
5725	1456555_at	Region	4933401K09Rik	234964	9	15398505	15452293	-
5726	1454976_at	Region	None	None	17	11514068	11515174	-
5727	1460325_at	Region	Pum1	80912	4	129460274	129578157	+
5728	1424883_s_at	Region	Sfrs7	225027	17	78017474	78024683	-
5729	1417321_at	Region	Zcchc7	28027	4	44840075	44848124	+
5730	1438516_at	Region	Rif1	51869	2	52004994	52054222	+
5731	1440478_at	Region	D10Ertd438e	52014	10	52670740	52691207	+
5732	1435816_at	Region	Rps6 /// LOC214738 /// LOC216036 /// LOC434404 /// LOC545640	20104	4	85840854	85843549	-
5733	1430020_x_at	Region	Hnrpa1 /// LOC225307	15382	15	103302595	103308263	+
5734	1443896_at	Region	Tbc1d5	72238	17	48265628	48712039	-
5735	1417436_at	Region	Large	16795	8	71965827	72364115	-
5736	1426189_at	Region	Dusp15	252864	2	152401869	152408341	-
5737	1437030_at	Region	Plcd4	18802	1	74846994	74868444	+
5738	1454670_at	Region	Rere	68703	4	148898702	149114159	+
5739	1449702_at	Region	AA407930	100494	5	138468708	138481909	-
5740	1438078_at	Region	None	None	11	88856266	88857359	-
5741	1436808_x_at	Region	Mcm5	17218	8	74273686	74291750	+

5742	1453011_at	Region	Dhrs6	69772	3	134164214	134187348	+
5743	1424801_at	Region	Enah	13800	1	181864370	181978518	-
5744	1438091_a_at	Region	H2afz	51788	3	136753528	136755851	+
5745	1423924_s_at	Region	Tspan14	52588	14	39051271	39079112	-
5746	1428374_at	Region	MGI:2136405	93683	9	62180047	62242646	-
5747	1440671_at	Region	A130012E19Rik	320235	10	115082377	115082896	+
5748	1425934_a_at	Region	B4galt4	56375	16	37563886	37590567	+
5749	1451979_at	Region	Kras	16653	6	146066254	146096662	-
5750	1435815_at	Region	Ldoc1	434784	X	56378815	56379550	+
5751	1420948_s_at	Region	4833408C14Rik	67403	X	100400840	100530738	-
5752	1423201_at	Region	Ncor1	20185	11	62043183	62164152	-
5753	1427937_at	Region	None	None	X	66477436	66483670	+
5754	1452387_a_at	Region	Amotl2	56332	9	102673186	102684065	+
5755	1459693_x_at	Region	Ywhag	22628	5	134921901	134948016	-
5756	1455317_at	Region	Epc2	227867	2	49383678	49483345	+
5757	1415816_at	Region	Cct7	12468	6	85801989	85818388	+
5758	1447005_at	Region	None	None	NONE	NONE	NONE	NONE
5759	1427606_at	Region	Usp27x	54651	X	5610216	5614766	-
5760	1452082_at	Region	6430548M08Rik	234797	8	119500738	119518927	+
5761	1452450_at	Region	Atp8b2	54667	3	89745559	89769413	-
5762	1427951_s_at	Region	1700009P13Rik	215814	10	18141400	18159451	-
5763	1417815_a_at	Region	Tde1	26943	2	163080242	163102100	-
5764	1434129_s_at	Region	Lhfpl2	218454	13	90241860	90379474	+
5765	1415715_at	Region	None	None	5	32142070	32144343	-
5766	1434425_at	Region	AI597080	99681	3	93165763	93166219	+
5767	1435242_at	Region	Aprin	100710	5	149615145	149749995	+
5768	1415704_a_at	Region	Cdv3	321022	9	103328549	103340979	-
5769	1434979_at	Region	4933403F05Rik	108654	18	68497464	68531006	-
5770	1415982_at	Region	5031400M07Rik	80517	9	25003408	25046786	-
5771	1434509_at	Region	Rapgef6	192786	11	54450466	54452127	+
5772	1450084_s_at	Region	Ivns1abp	117198	1	151200258	151220009	+
5773	1450385_at	Region	Kpna3	16648	14	55898702	55974523	-
5774	1417728_at	Region	Mbd3	17192	10	80523342	80530300	-
5775	1431782_s_at	Region	Ypel1	106369	16	15841589	15857966	+
5776	1452680_at	Region	Snrpd2	107686	7	16018599	16021487	+
5777	1435470_at	Region	None	None	8	19654535	19655405	+
5778	1437083_at	Region	A830020B06Rik	271711	1	72582077	72600701	+
5779	1423540_at	Region	Rbms2	56516	10	127870047	127913260	-
5780	1460319_at	Region	Fut8	53618	12	74095608	74332578	+
5781	1428247_at	Region	2310075A12Rik	69091	9	26901688	26924026	-
5782	1421850_at	Region	Mtap1b	17755	13	95613359	95705305	-
5783	1424697_at	Region	Dtwd1	69185	2	125666028	125679129	+
5784	1433927_at	Region	E430026A01Rik	231915	5	148087253	148114739	+
5785	1432447_a_at	Region	2310005N01Rik	70088	4	124112260	124137002	+
5786	1437593_x_at	Region	Api5	11800	2	94116415	94142756	-
5787	1431275_at	Region	Chaf1b	110749	16	93055266	93077291	+
5788	1415838_at	Region	Tde2	56442	10	57778279	57794950	-
5789	1423721_at	Region	Tpm1	22003	9	67151552	67178023	-
5790	1425342_a_at	Region	Kcnk3	16527	5	29047028	29082424	+
5791	1446299_at	Region	6720456B07Rik	101314	6	114172603	114184782	+
5792	1442276_at	Region	Lphn3	319387	5	80275281	81049432	+
5793	1450844_at	Region	Stx6	58244	1	155049479	155094254	+
5794	1450395_at	Region	Slc22a5	20520	11	53617383	53644501	-
5795	1422992_s_at	Region	Pik3cd	18707	4	148143002	148155646	-
5796	1424214_at	Region	9130213B05Rik	231440	5	90839183	90945494	+
5797	1441106_at	Region	Za20d2	22682	19	20510205	20520216	+
5798	1434299_x_at	Region	Rabl4	67042	15	78211456	78226101	-
5799	1428960_at	Region	4933434I06Rik	71233	2	21222741	21247375	-
5800	1444613_at	Region	None	None	8	80738819	80739041	+
5801	1456467_s_at	Region	Nlk	18099	11	78292827	78423032	-
5802	1417442_a_at	Region	Pex3	56535	10	13405906	13434917	-
5803	1450041_a_at	Region	Tub	22141	7	102863841	102883914	+
5804	1441663_at	Region	None	None	15	93635671	93636268	-
5805	1416693_at	Region	Foxc2	14234	8	120472104	120474416	+
5806	1425212_a_at	Region	Tnfrsf19	29820	14	55499179	55581612	-

5807	1417467_a_at	Region	Tada3l	101206	6	113934484	113945349	-
5808	1451220_at	Region	2310040A13Rik	69641	12	106213222	106270262	+
5809	1460635_at	Region	Fastk	66587	5	22904937	22908267	-
5810	1460574_at	Region	Fat4	329628	3	38357245	38481982	+
5811	1440908_at	Region	D030063E12	328530	15	38990884	38992880	-
5812	1433812_at	Region	Lix1l	280411	3	96089184	96112327	+
5813	1456109_a_at	Region	Mrps15	66407	4	125074041	125082649	+
5814	1443951_at	Region	C230076A16Rik	320496	3	137031723	137032591	-
5815	1417196_s_at	Region	D8Ertd594e	52357	8	46797192	46960652	-
5816	1431388_at	Region	Mphosph10	67973	7	58155241	58166183	-
5817	1434938_at	Region	Rbm9	93686	15	77134294	77358885	-
5818	1416740_at	Region	Col5a1	12831	2	27818847	27969686	+
5819	1428759_s_at	Region	1300013D05Rik	67480	11	97566702	97587643	-
5820	1456130_at	Region	LOC553901	553091	6	12295318	12296311	-
5821	1426616_at	Region	0610007A15Rik	68385	11	77904425	77906431	+
5822	1426958_at	Region	Rps9 /// LOC546015	546015	7	135574033	135580491	-
5823	1419104_at	Region	Abhd6	66082	14	5671130	5724783	+
5824	1425790_a_at	Region	Grik2	14806	10	49284376	49966826	-
5825	1424289_at	Region	BC010311	209212	4	15924086	15940842	-
5826	1441183_at	Region	Jmjd2b	193796	17	53963133	54039904	+
5827	1422639_at	Region	Calcb	116903	7	108577837	108582423	+
5828	1436390_a_at	Region	MGI:2385186	229725	3	108449989	108473754	+
5829	1426499_at	Region	Sh3glb2	227700	2	30276971	30291393	-
5830	1418496_at	Region	Foxa1	15375	12	54272111	54277439	-
5831	1434258_s_at	Region	Phactr4	100169	4	131116497	131182926	-
5832	1437508_at	Region	Sp4	20688	12	114051387	114115389	-
5833	1435093_at	Region	Zfyve20	78287	6	92634150	92662609	-
5834	1451970_at	Region	E330036I19Rik	231871	5	142522593	142562419	+
5835	1455543_at	Region	Klhl18	270201	9	110467462	110517991	-
5836	1459348_at	Region	Ebf1	13591	11	44370971	44758012	+
5837	1417566_at	Region	Abhd5	67469	9	122371891	122401684	+
5838	1423573_at	Region	Srd5a2l	57357	5	75385516	75400710	+
5839	1435106_at	Region	None	None	5	65822281	65823300	+
5840	1452951_at	Region	2410089E03Rik	73692	15	7956639	8058734	+
5841	1459858_x_at	Region	Crtac1	72832	19	41827441	41976053	-
5842	1429267_at	Region	Thea	329910	4	105703485	105758671	-
5843	1441549_at	Region	4931408L03Rik	78266	3	94494533	94503069	-
5844	1448531_at	Region	Lmnb2	16907	10	81033305	81049800	-
5845	1440564_at	Region	Gpr73l1	246313	2	131885018	131899253	-
5846	1437880_at	Region	Lbxcor1	207667	9	63259400	63268242	-
5847	1450803_at	Region	Ntf3	18205	6	126767210	126830735	-
5848	1460584_at	Region	B430315C20Rik	327655	2	120824412	120864866	-
5849	1443338_at	Region	None	None	2	49057871	49058162	+
5850	1434041_at	Region	Appbp2	66884	11	84919300	84963112	-
5851	1448413_at	Region	2410016O06Rik	71952	12	80815051	80817327	+
5852	1449655_a_at	Region	1300018I05Rik	74157	17	27470422	27513276	+
5853	1421810_at	Region	Dgcr2	13356	16	16612273	16662559	-
5854	1452939_a_at	Region	Pitpnc1	71795	11	107033195	107291788	-
5855	1442320_at	Region	LOC553096	553096	19	15467392	15467899	+
5856	1430043_at	Region	Ttc19	72795	11	62007122	62040890	+
5857	1416131_s_at	Region	C920006C10Rik	76740	15	65810937	65897708	+
5858	1436220_at	Region	Zfp287	170740	11	62438937	62454743	-
5859	1428691_at	Region	5630401D06Rik	71437	7	67319558	67321791	-
5860	1422511_a_at	Region	Ogfr	72075	2	180306419	180312789	+
5861	1456615_a_at	Region	Falz	207165	11	106856593	106978743	-
5862	1433946_at	Region	Zik1	22775	7	8786037	8794152	-
5863	1417737_at	Region	Mrps31	57312	8	21163591	21181648	+
5864	1427592_at	Region	Pcdh7	54216	5	56521609	56532964	+
5865	1419205_x_at	Region	Gpatc4	66614	3	87786988	87799852	+
5866	1441409_at	Region	AI449705	103220	10	86680474	86751505	+
5867	1445264_at	Region	Catsper2	212670	2	120908208	120927384	-
5868	1455935_at	Region	2410131K14Rik	76792	5	117373963	117392047	+
5869	1443225_at	Region	Acvr1c	269275	2	58197361	58241564	-
5870	1435809_at	Region	LOC545554	545554	3	96086860	96087615	+

5871	1416704_at	Region	Mapk14	26416	17	26492551	26549631	+
5872	1427345_a_at	Region	Sult1a1	20887	7	120722601	120726088	-
5873	1428972_at	Region	0610012D17Rik	66061	16	31229115	31238469	+
5874	1433557_at	Region	Cbx7	52609	15	79965309	79984931	-
5875	1416308_at	Region	Ugdh	22235	5	64215050	64237671	-
5876	1450656_at	Region	Gna13	14674	11	109184029	109219306	+
5877	1421629_at	Region	Gabre	14404	X	66918087	66935376	-
5878	1424501_at	Region	4732497O03Rik	216987	11	79659615	79688046	-
5879	1444909_at	Region	Gm574	213948	5	22848078	22856040	-
5880	1443988_at	Region	B330012G18Rik	320419	2	155610458	155611210	-
5881	1435479_at	Region	None	None	2	172328482	172329344	-
5882	1438268_at	Region	Mnab	319817	2	37306963	37355065	-
5883	1416417_a_at	Region	Ndufb7	66916	8	82824283	82829148	+
5884	1428468_at	Region	3110043O21Rik	73205	4	35330354	35365190	-
5885	1439884_at	Region	Nudt16	75686	9	105111048	105113181	-
5886	1428403_at	Region	2410025L10Rik	381668	5	109413571	109437880	-
5887	1430533_a_at	Region	Ctnnb1	12387	9	120945869	120972901	+
5888	1439498_at	Region	Punc	19289	9	65263454	65308137	+
5889	1415961_at	Region	Itm2c	64294	1	85700921	85715048	+
5890	1437966_at	Region	B230206N24Rik	210673	6	114064130	114069723	-
5891	1459497_at	Region	Fdft1	14137	14	57675741	57708384	-
5892	1448498_at	Region	Rps6ka4	56613	19	6542932	6554449	-
5893	1454657_s_at	Region	Mak10	78689	13	58245009	58294396	+
5894	1437936_at	Region	LOC434778	434778	X	47311398	47319850	+
5895	1433150_at	Region	4933415B22Rik	71178	5	111437201	111438462	-
5896	1439105_at	Region	Cdadc1	71891	14	54094451	54133002	-
5897	1445465_at	Region	N28178	230085	4	42833173	42857529	+
5898	1448471_a_at	Region	Ctla2a	13024	13	59552384	59592485	-
5899	1435333_at	Region	1110007M04Rik	68493	4	25033642	25037728	+
5900	1456567_x_at	Region	Grn	14824	11	102251594	102257892	+
5901	1433880_at	Region	Dnajc11	230935	4	150426093	150469046	+
5902	1421022_x_at	Region	Acyp1	66204	12	82141604	82149527	-
5903	1439448_x_at	Region	Tmed9	67511	13	54209701	54214246	+
5904	1444856_at	Region	Crebbp	12914	16	3785202	3786206	-
5905	1451768_a_at	Region	Slc20a2	20516	8	21228953	21324191	+
5906	1451042_a_at	Region	Mina	67014	16	58457856	58478079	+
5907	1421199_at	Region	Dlgh2	23859	7	85860491	86494465	+
5908	1439074_a_at	Region	Son	20658	16	90805044	90836353	+
5909	1450382_at	Region	Nf2	18016	11	4662781	4744337	-
5910	1426624_a_at	Region	Ypel3	66090	7	120826706	120830240	+
5911	1450427_at	Region	Chrna6	11440	8	26172766	26183611	-
5912	1436344_at	Region	5830404H04Rik	207781	16	97343062	97406898	-
5913	1431921_a_at	Region	Stag1	20842	9	100545170	100860091	+
5914	1436106_x_at	Region	2310015A05Rik	72315	16	16417438	16421706	+
5915	1435059_at	Region	Ddef1	13196	15	64104369	64365898	-
5916	1436878_at	Region	Baalc	118452	15	38833124	38850257	+
5917	1456904_at	Region	None	None	11	97009053	97010427	+
5918	1453036_at	Region	5430439M09Rik	71348	17	92845573	92847457	+
5919	1447381_at	Region	None	None	NONE	NONE	NONE	NONE
5920	1416089_at	Region	Rps15	20054	10	80417366	80419021	+
5921	1460004_x_at	Region	Stx6	58244	1	155049479	155094254	+
5922	1451381_at	Region	1810020D17Rik	66273	7	91596611	91625686	-
5923	1429260_at	Region	1810014B01Rik	544717	10	82507736	82511798	-
5924	1453737_at	Region	5730509C05Rik	68524	11	98684896	98724607	+
5925	1446101_at	Region	Rere	68703	4	148898702	149114159	+
5926	1438714_at	Region	Zfp207	22680	11	80109005	80121796	+
5927	1444376_at	Region	Sesn1	140742	10	42008166	42008843	+
5928	1447816_x_at	Region	BC019806	218885	14	30218148	30235400	+
5929	1454723_at	Region	1110033M05Rik	68675	13	73773385	74230386	+
5930	1445502_at	Region	None	None	8	93608483	93609066	+
5931	1426682_at	Region	Cnot6	104625	11	49427539	49465551	-
5932	1433938_at	Region	Trp53bp2	209456	1	182387965	182421393	+
5933	1426386_at	Region	Rpl7l1	66229	17	44284905	44294348	-
5934	1447150_at	Region	Phr1	105689	14	97658865	97891435	-
5935	1433325_at	Region	9330154F10Rik	77554	4	67891745	67892824	-

5936	1419313_at	Region	Ccnt1	12455	15	98601104	98626096	-
5937	1415751_at	Region	Hp1bp3	15441	4	137097923	137124421	+
5938	1455967_at	Region	Sorbs1	20411	19	39839082	40058071	-
5939	1455583_at	Region	Gne	50798	4	43952748	44000088	-
5940	1415736_at	Region	Pfdn5	56612	15	102386650	102391967	+
5941	1448496_a_at	Region	Ing1	26356	8	10928492	10935676	+
5942	1418589_a_at	Region	Mlf1	17349	3	67046502	67072273	+
5943	1445857_at	Region	None	None	1	157364166	157398213	+
5944	1450384_at	Region	Bace1	23821	9	45851835	45875693	+
5945	1427944_at	Region	C1qdc1	232560	6	149778375	149832094	-
5946	1428707_at	Region	2610009E16Rik	69202	6	125570449	125574707	-
5947	1425805_a_at	Region	Usp12	22217	5	145626505	145686649	-
5948	1416017_at	Region	Copg	54161	6	88324602	88350257	+
5949	1429591_at	Region	Tacc1	320165	8	23877518	23925420	-
5950	1434276_x_at	Region	BC004004	80748	17	27090378	27115403	+
5951	1440449_at	Region	2610040E16Rik	67144	3	157046099	157077874	+
5952	1451740_at	Region	Paip1	218693	13	232	27441	+
5953	1447084_at	Region	Nfatc1	18018	18	80729557	80836410	-
5954	1427688_a_at	Region	Ptprs	19280	17	54049473	54095417	-
5955	1454950_at	Region	B930006L02Rik	319604	7	94813378	94948322	+
5956	1433881_at	Region	Dnajc11	230935	4	150426093	150469046	+
5957	1418720_at	Region	Cops7b	26895	1	86392941	86412324	+
5958	1431997_at	Region	3000002C10Rik	378954	9	109851351	109853882	-
5959	1433588_at	Region	D6Wsu116e	28006	6	116645592	116700200	+
5960	1452203_at	Region	5830411E10Rik	109019	1	51769634	51779343	-
5961	1430064_at	Region	4933424A20Rik	76860	2	35909771	35911703	-
5962	1433957_at	Region	C030048B08Rik	269623	5	3589987	3602413	-
5963	1433624_at	Region	5830434P21Rik	227723	2	32083319	32166687	+
5964	1426975_at	Region	4632413K17Rik	216440	10	126828369	126853844	-
5965	1427173_a_at	Region	Mrps33	14548	6	39939988	39949724	-
5966	1447991_at	Region	Pcsk2	18549	2	143003115	143273220	+
5967	1444644_x_at	Region	Tatdn1	69694	15	58900953	58944887	-
5968	1443029_at	Region	Ppfibp1	67533	6	147815628	147958589	+
5969	1421968_a_at	Region	Nipa2	93790	7	50201064	50231927	-
5970	1454214_a_at	Region	2410019A14Rik	229937	3	144551999	144581025	+
5971	1426684_at	Region	Cnot6	104625	11	49427539	49465551	-
5972	1443307_at	Region	Psmc2	19181	5	20237647	20256148	+
5973	1449149_at	Region	Traf3	22031	12	106693201	106740539	+
5974	1457625_s_at	Region	Cdkl2	53886	5	91341903	91378846	-
5975	1429222_at	Region	None	None	9	35471054	35479299	+
5976	1454797_at	Region	BC039161	219024	14	46021855	46025473	-
5977	1449069_at	Region	Zfp148	22661	16	32188918	32308456	+
5978	1431694_a_at	Region	Ctnnbip1	67087	4	148010605	148059087	+
5979	1460631_at	Region	Ogt	108155	X	96241425	96285710	+
5980	1419678_at	Region	Lats2	50523	14	52225857	52269062	-
5981	1416994_at	Region	Ttc1	66827	11	43482843	43500810	-
5982	1429603_at	Region	2610110G12Rik	73242	17	33619005	33629198	-
5983	1436865_at	Region	Slc26a11	268512	11	119183742	119202142	+
5984	1449480_at	Region	Sap18	20220	14	52333126	52339849	+
5985	1426478_at	Region	Rasa1	218397	13	81307760	81348198	-
5986	1435878_at	Region	Stk38l	232533	6	147645808	147699736	+
5987	1442157_at	Region	A930041H05Rik	77834	13	113302587	113303328	-
5988	1453364_x_at	Region	9130019O22Rik	78921	7	121431991	121436897	-
5989	1455327_at	Region	Senp2	75826	16	20782192	20820660	+
5990	1426959_at	Region	Bdh	71911	16	30231569	30266772	+
5991	1429100_at	Region	2010109K11Rik	72123	12	28982023	28982836	+
5992	1454044_a_at	Region	Pex3	56535	10	13405906	13434917	-
5993	1416420_a_at	Region	Rpl9	20005	5	64190194	64192821	-
5994	1441656_at	Region	B930068K11Rik	320622	2	34270671	34271275	-
5995	1457466_at	Region	AA409368	98783	2	3041906	3042622	+
5996	1440312_at	Region	Elovl7	74559	13	104441513	104512532	+
5997	1460389_at	Region	Cdk8	264064	5	145118521	145189661	+
5998	1451209_at	Region	Lass5	71949	15	99792808	99829731	-
5999	1426106_a_at	Region	Syt6	54524	3	103002769	103058318	+
6000	1434283_at	Region	Arid5b	71371	10	68150685	68334013	-

6001	1438277_at	Region	E130308A19Rik	230259	4	59569847	59664423	+
6002	1452107_s_at	Region	Npnt	114249	3	131774826	131840439	-
6003	1424326_at	Region	Lemd2	224640	17	24985675	24999666	-
6004	1421359_at	Region	Ret	19713	6	118586419	118629700	-
6005	1422464_at	Region	Mrpl3	94062	9	105034159	105058367	+
6006	1423500_a_at	Region	Sox5	20678	6	144679075	145055646	-
6007	1422647_at	Region	Ring1	19763	17	31725138	31728697	-
6008	1450048_a_at	Region	Idh2	269951	7	73893703	73914172	-
6009	1418075_at	Region	St6galnac4	20448	2	32519652	32532644	+
6010	1416130_at	Region	Prnp	19122	2	131423810	131452280	+
6011	1438296_at	Region	C430010C01	329584	2	181102960	181104209	+
6012	1453411_at	Region	2010110K16Rik	67899	9	118060948	118143996	-
6013	1433597_at	Region	9430010O03Rik	234023	8	8032433	8056378	-
6014	1433906_at	Region	4933402J24Rik	74438	4	9196508	9378837	+
6015	1418466_at	Region	Rnf141	67150	7	104668395	104697253	-
6016	1419385_a_at	Region	Ubqln1	56085	13	56804764	56844390	-
6017	1423688_at	Region	Man2c1	73744	9	57244266	57255699	+
6018	1444651_at	Region	Map2k4	26398	11	65413894	65513947	-
6019	1455687_at	Region	Ick	56542	9	78282435	78336986	+
6020	1439799_at	Region	None	None	17	61196370	61197292	+
6021	1423104_at	Region	Irs1	16367	1	82571724	82626530	-
6022	1426271_at	Region	Smc5l1	226026	19	22446187	22511419	-
6023	1454161_s_at	Region	0610007P14Rik	58520	12	82685029	82694208	-
6024	1443735_at	Region	None	None	13	60121869	60241129	+
6025	1450641_at	Region	Vim	22352	2	13489961	13498816	+
6026	1443585_at	Region	Zfp526	210172	7	20401813	20408380	+
6027	1447326_s_at	Region	Zfp261	56364	X	96005746	96022210	-
6028	1422499_at	Region	D15Ertd366e	65970	15	99835724	99877288	-
6029	1430554_at	Region	Lrig3	320398	10	125665572	125714849	+
6030	1433911_at	Region	AI317223	103677	11	74651530	74890106	+
6031	1421322_a_at	Region	Isgf3g	16391	14	50122590	50128041	+
6032	1418007_at	Region	1810007M14Rik	67367	16	90171076	90201546	-
6033	1423144_at	Region	Pik3ca	18706	3	31844384	31871009	+
6034	1437017_at	Region	AI480653	268880	16	29764506	29890256	-
6035	1453666_at	Region	Rnf32	56874	5	27646557	27676001	+
6036	1423306_at	Region	2010002N04Rik	106878	18	60699517	60726851	-
6037	1457438_at	Region	None	None	4	11050923	11053963	+
6038	1447209_at	Region	Foxp1	108655	6	99395348	99630926	-
6039	1424371_at	Region	Psmf1	228769	2	151172998	151198229	-
6040	1442151_at	Region	None	None	13	13510425	13511430	+
6041	1435403_at	Region	---	385627	16	32181350	32188822	-
6042	1442586_at	Region	D130043N08Rik	103250	10	95384895	95385568	-
6043	1453293_a_at	Region	2810408A11Rik	70419	11	69623016	69626644	-
6044	1421083_x_at	Region	Banf1	23825	19	5153430	5155435	-
6045	1416896_at	Region	Rps6ka1	20111	4	132808193	132848683	-
6046	1449648_s_at	Region	Rpo1-1	20016	17	43753849	43757884	-
6047	1451368_at	Region	Alg1 /// LOC547231	208211	16	4903299	4914559	+
6048	1450700_at	Region	Cdc42ep3	260409	17	77155449	77176516	-
6049	1416459_at	Region	Arf2	11841	11	103787960	103806425	+
6050	1426469_a_at	Region	Tbp	21374	17	13506727	13520435	+
6051	1434213_x_at	Region	Ndufs8	225887	19	3697661	3701550	-
6052	1416747_at	Region	D15Mgi27	106073	15	102339753	102342043	+
6053	1453932_at	Region	4931433A01Rik	78933	4	109356566	109621875	+
6054	1437776_at	Region	3632431M01Rik	330401	6	116456153	116631023	-
6055	1440243_at	Region	Cpne7	102278	8	122498738	122516463	+
6056	1447409_at	Region	Chd7	320790	4	8617703	8793952	+
6057	1425674_a_at	Region	Ssu72	68991	4	154197486	154226144	+
6058	1424175_at	Region	Tef	21685	15	81853534	81877580	+
6059	1416173_at	Region	Pes1	64934	11	3858762	3874791	+
6060	1449041_a_at	Region	Trip6	22051	5	136260765	136265107	-
6061	1437650_at	Region	C730026J16	331006	9	96582585	96584688	-
6062	1454664_a_at	Region	Eif5	217869	12	107012763	107017765	+
6063	1448396_at	Region	D1Bwg0491e	56030	1	37086785	37233778	-
6064	1421867_at	Region	Nr3c1	14815	18	39638075	39711047	-

6065	1459670_at	Region	Hnrpll	72692	17	77846881	77879599	-
6066	1417458_s_at	Region	Cks2	66197	13	50199009	50204338	+
6067	1438400_at	Region	4632411B12Rik	226976	1	36630324	36663775	-
6068	1430597_at	Region	MGI:1923321	76071	5	35557428	35631807	+
6069	1442732_at	Region	Hadhb	231086	5	28621184	28642098	+
6070	1417054_a_at	Region	0610009D07Rik	66055	12	3985835	3999611	+
6071	1435806_at	Region	D530033C11Rik	78581	15	51874725	51881202	+
6072	1428258_at	Region	2010107E04Rik	70257	12	107440675	107446276	-
6073	1429438_at	Region	Bcor	71458	X	10276230	10318808	-
6074	1436486_x_at	Region	Whrn	73750	4	62506101	62587142	-
6075	1431827_a_at	Region	Tlk2	24086	11	104998272	105103265	+
6076	1417221_at	Region	Ppm1a	19042	12	69630561	69641441	+
6077	1452100_at	Region	Dullard	67181	11	69706826	69716259	+
6078	1447260_at	Region	H2-Bl /// LOC547349	14963	17	115	1182	-
6079	1457272_at	Region	None	None	18	73869019	73870225	-
6080	1416115_at	Region	Orc3l	50793	4	34708370	34754011	-
6081	1422671_s_at	Region	Naalad2	72560	9	18164010	18226920	-
6082	1442740_at	Region	Prdm5	70779	6	66036866	66194195	+
6083	1417771_a_at	Region	Psmc6	67089	14	862	12455	-
6084	1436113_a_at	Region	St13	70356	15	81415844	81450494	-
6085	1434680_at	Region	Plekhg3	263406	12	73390267	73424857	+
6086	1423084_at	Region	B3galt2	26878	1	143472806	143481893	+
6087	1443583_at	Region	C030010B13Rik	77310	2	173046327	173120005	-
6088	1425671_at	Region	Homer1	26556	13	89489518	89588558	+
6089	1428061_at	Region	Hat1	107435	2	71087000	71139365	+
6090	1426715_s_at	Region	D11Ertd18e	52466	11	78191377	78197589	+
6091	1455020_at	Region	Snx25	102141	8	44973816	44987988	-
6092	1431828_a_at	Region	Synj2	20975	17	5881998	5950653	+
6093	1432083_a_at	Region	Lrpb7	16977	6	125426629	125436487	-
6094	1457802_at	Region	B930012P20Rik	320140	3	78960491	78961026	-
6095	1419508_at	Region	Ripk1	19766	13	33537383	33569679	+
6096	1440932_at	Region	Trim17 /// Hist3h2a	319162	11	58680314	58682303	+
6097	1425661_at	Region	Cdadc1	71891	14	54094451	54133002	-
6098	1442918_at	Region	Nav3	260315	10	109569106	109718861	-
6099	1451846_at	Region	Nebl	74103	2	17388744	17773079	-
6100	1420616_at	Region	Ash2l	23808	8	24541624	24565518	-
6101	1428918_at	Region	Scyl3	240880	1	163847656	163872719	+
6102	1452037_at	Region	Mgat2	217664	12	66016002	66018036	+
6103	1437382_at	Region	Acvr2a	11480	2	48746319	48834325	+
6104	1426383_at	Region	Cry2	12953	2	92108329	92138711	-
6105	1419027_s_at	Region	MGI:1929253	56356	5	113779116	113800561	-
6106	1436991_x_at	Region	Gsn	227753	2	35214602	35240054	+
6107	1434656_at	Region	B230339M05Rik	228850	2	157887219	157956217	+
6108	1437204_a_at	Region	None	None	8	122775522	122778505	+
6109	1427122_at	Region	Copg2as2	54158	6	30846849	30849105	+
6110	1453457_at	Region	2900070H08Rik	73025	5	8069682	8070888	+
6111	1423674_at	Region	Usp1	230484	4	97898883	97910606	+
6112	1451003_at	Region	Map3k7ip2	68652	10	7771623	7790347	-
6113	1421910_at	Region	Tcf20	21411	15	82860358	82962283	-
6114	1417112_at	Region	Arl2bp	107566	8	93950927	93958594	+
6115	1434591_at	Region	4732460K03Rik	58894	6	48666616	48667711	+
6116	1434160_at	Region	Zfp592	233410	7	74796384	74845397	+
6117	1418032_at	Region	2700050P07Rik	101142	6	129099631	129115097	-
6118	1425202_a_at	Region	Ank3	11735	10	69589190	70079102	+
6119	1451051_a_at	Region	Scyl1	78891	19	5547217	5560191	-
6120	1418469_at	Region	Nrip1	268903	16	75376752	75458939	-
6121	1454801_at	Region	Ankrd28	105522	14	29830803	29831990	-
6122	1442016_at	Region	D630004N19Rik	402747	7	94652424	94654296	-
6123	1435652_a_at	Region	Gnai2	14678	9	107680276	107701110	-
6124	1456526_at	Region	C130034I24Rik	320794	2	91458928	91459645	+
6125	1441592_at	Region	None	None	10	67169992	67170493	+
6126	1436537_at	Region	Zfp629	320683	7	121656766	121664164	-
6127	1444071_at	Region	9630013A20Rik	319903	14	78818360	78835088	-

6128	1422514_at	Region	Aebp1	11568	11	5756734	5766837	+
6129	1420913_at	Region	Slco2a1	24059	9	102980160	103058431	+
6130	1447056_at	Region	Slit2	20563	5	46748038	47069334	+
6131	1422048_at	Region	Trpc5	22067	X	137821717	138128226	-
6132	1451026_at	Region	Ftsj3	56095	11	106070233	106076891	-
6133	1424575_at	Region	Rabl5	67286	5	135921272	135926366	+
6134	1428210_s_at	Region	Chuk	12675	19	43617742	43651851	-
6135	1429593_at	Region	Slc38a2	67760	15	96755014	96767320	-
6136	1436454_x_at	Region	Fen1	14156	19	9395636	9400440	-
6137	1425383_a_at	Region	Pbx1	18514	1	168054563	168366387	-
6138	1415970_at	Region	Cox6c	12864	15	35933506	35939687	-
6139	1440357_at	Region	None	None	15	85755490	85756167	+
6140	1439895_at	Region	AU021025	105285	13	110400275	110401249	+
6141	1443138_at	Region	Sult5a1	57429	8	122524128	122539554	-
6142	1442692_at	Region	Mapkap1	227743	2	34339345	34556075	+
6143	1455036_s_at	Region	Ndufc2	68197	7	91446361	91454068	+
6144	1417209_at	Region	Sertad2	58172	11	20438127	20543916	+
6145	1441538_at	Region	Dpp6	13483	5	25258284	26167391	+
6146	1453105_at	Region	Zfp263	74120	16	3415695	3422348	+
6147	1423634_at	Region	Gsdm1	57911	11	98485438	98498795	+
6148	1437638_at	Region	Srrm2	75956	17	21605406	21626916	+
6149	1436396_at	Region	D430033N04Rik	217935	12	112002973	112026119	-
6150	1430841_at	Region	3300002P13Rik	70230		65522618	65523551	+
6151	1424106_at	Region	1200003C05Rik	104771	12	68925221	68939959	+
6152	1417253_at	Region	Frg1	14300	8	40335099	40354883	-
6153	1435046_at	Region	Myo1d	338367	11	80309486	80505684	-
6154	1433476_at	Region	C78339	97863	13	46253320	46273975	+
6155	1417812_a_at	Region	Lamb3	16780	1	193040798	193082646	+
6156	1441356_at	Region	Shc2	216148	10	79740620	79759030	-
6157	1417733_at	Region	Rnf146	68031	10	29378124	29394428	-
6158	1429031_at	Region	4833408D11Rik	73789	7	23213413	23214291	+
6159	1435261_at	Region	4732416N19Rik	320737	6	149166254	149169251	-
6160	1436305_at	Region	LOC268291	268291	10	31542086	31649384	-
6161	1421750_a_at	Region	Vbp1	22327	X	70175004	70195547	+
6162	1418884_x_at	Region	Tuba1	22142	15	99007626	99011282	-
6163	1417384_at	Region	Entpd5	12499	12	81245023	81273816	-
6164	1450673_at	Region	Col9a2	12840	4	120061776	120077745	+
6165	1449445_x_at	Region	Mfap1	67532	2	121007192	121020508	-
6166	1451455_at	Region	BC051244	232078	6	71460328	71476268	-
6167	1437144_x_at	Region	Psma6	26443	12	52119691	52139243	+
6168	1423558_at	Region	Ifngr2	15980	16	90704258	90721171	+
6169	1440966_at	Region	38418	57438	2	60065336	60103785	+
6170	1418292_at	Region	Asna1	56495	8	84288403	84295917	-
6171	1443885_at	Region	None	None	7	20265238	20266112	-
6172	1427478_at	Region	Usp12	22217	5	145626505	145686649	-
6173	1450897_at	Region	Arhgap5	11855	12	49267090	49318371	+
6174	1437987_at	Region	None	None	12	49322710	49323013	+
6175	1432144_a_at	Region	Rchy1	68098	5	91284732	91298896	-
6176	1451550_at	Region	Ephb3	13845	16	19975855	19994364	+
6177	1420561_at	Region	Trpc7	26946	13	55393457	55515937	-
6178	1453097_a_at	Region	Ubtf	21429	11	102127078	102137561	-
6179	1425142_a_at	Region	Hnrpd	11991	5	98979646	98997188	-
6180	1428763_at	Region	Smug1	71726	15	103215352	103225303	-
6181	1426850_a_at	Region	Map2k6	26399	11	110220211	110334726	+
6182	1448947_at	Region	0610039J04Rik	66523	8	124126680	124150314	-
6183	1422597_at	Region	Mmp15	17388	8	94636817	94655679	+
6184	1448204_at	Region	Sav1	64010	12	66797137	66819163	-
6185	1446548_at	Region	Gltscr1	276711	7	21882524	21883153	-
6186	1448435_at	Region	Pcqap	94112	16	16422190	16493791	-
6187	1430889_a_at	Region	Tpmt	22017	13	46623326	46641352	-
6188	1443161_at	Region	Trps1	83925	15	50659276	50888038	-
6189	1451253_at	Region	Pxk	218699	14	5766471	5833846	+
6190	1418343_at	Region	Gtf2ird2	114674	5	133475414	133509522	+
6191	1452704_at	Region	1200015F23Rik	67809	2	118650852	118681688	-
6192	1419066_at	Region	Ier5l	72500	2	30403700	30406381	-

6193	1417871_at	Region	Hsd17b7	15490	1	169883718	169903346	-
6194	1449339_at	Region	D10Ertd641e	52717	10	59943553	59958756	-
6195	1451081_a_at	Region	D8Ertd325e	66855	8	122748919	122778703	+
6196	1433822_x_at	Region	Anapc5	59008	5	121940636	121974194	-
6197	1428031_at	Region	Gpr24	207911	15	81286574	81288912	+
6198	1448376_at	Region	Wrnip1	78903	13	32281660	32302231	+
6199	1443244_at	Region	Zadh1	77219	12	81153770	81182471	+
6200	1435560_at	Region	None	None	7	121383509	121384867	+
6201	1455483_at	Region	Zfp148	22661	16	32188918	32308456	+
6202	1416274_at	Region	Ctns	83429	11	72908791	72924677	-
6203	1438236_at	Region	Nfia	18027	4	96752972	97087465	+
6204	1424698_s_at	Region	Gca	227960	2	62519727	62549509	+
6205	1437871_at	Region	Pgm5	226041	19	24056158	24101717	-
6206	1443947_at	Region	Lims1	110829	10	58281059	58380568	+
6207	1428429_at	Region	Rgmb	68799	17	13809790	13830123	-
6208	1452297_at	Region	Ccnt2	72949	1	127616552	127647225	+
6209	1433830_at	Region	Hnrpa2b1	53379	6	51608402	51617123	-
6210	1423491_at	Region	Fbxo3	57443	2	103732553	103767991	+
6211	1451123_at	Region	C330016O10Rik	212706	11	51395930	51403914	-
6212	1456061_at	Region	Gimap8	243374	6	48780240	48793754	+
6213	1458984_at	Region	None	None	4	59672321	59672899	+
6214	1428401_at	Region	Zcchc3	67917	2	151869891	151871963	-
6215	1450506_a_at	Region	Isg20l1	68048	7	72688034	72700940	+
6216	1417293_at	Region	Hs6st1	50785	1	36359851	36397843	+
6217	1459667_at	Region	Arhgap17	70497	7	117335650	117426263	-
6218	1429039_s_at	Region	1500034J01Rik	66498	8	70619056	70625966	+
6219	1425021_a_at	Region	Pex16	18633	2	92080181	92085900	+
6220	1443017_at	Region	Cpeb2	231207	5	41990144	42042375	+
6221	1418204_s_at	Region	Aif1	11629	17	32874698	32879734	-
6222	1426504_a_at	Region	Rnf121	75212	7	96126119	96178400	-
6223	1417157_at	Region	Actr10	56444	12	67772900	67797290	+
6224	1424834_s_at	Region	Itpr2	16439	6	147342110	147422837	-
6225	1455403_at	Region	Manea	242362	4	26460019	26482165	-
6226	1431118_at	Region	6720427H10Rik	77884	13	110389021	110390636	+
6227	1448152_at	Region	Igf2	16002	7	137063787	137074879	-
6228	1430251_at	Region	D330022H12Rik	77095	16	4264424	4265735	-
6229	1431236_at	Region	Stk32b	64293	5	35953339	36223576	-
6230	1426464_at	Region	Nr1d1	217166	11	98589019	98595909	-
6231	1435944_s_at	Region	Cenpb	12616	2	130691142	130693865	-
6232	1448607_at	Region	Pbef1	59027	12	29426677	29457809	+
6233	1442265_at	Region	Fancc	14088	13	60694745	60814715	-
6234	1417478_a_at	Region	4930511A21Rik	59032	12	51935442	52023946	-
6235	1426781_at	Region	Rsafd1	100929	5	129432846	129455440	+
6236	1448138_at	Region	Ppp2r4	110854	2	30348212	30379966	+
6237	1422767_at	Region	Bysl	53414	17	45118540	45129944	-
6238	1418109_at	Region	Gspt2	14853	X	89296727	89299220	+
6239	1453983_a_at	Region	2810013M15Rik	67493	11	74496521	74544350	+
6240	1456010_x_at	Region	Hes5	15208	4	153453285	153454733	+
6241	1450426_at	Region	Chrna6	11440	8	26172766	26183611	-
6242	1422437_at	Region	Col5a2	12832	1	45679868	45806698	-
6243	1449135_at	Region	Sox18	20672	2	181386799	181388565	-
6244	1435905_at	Region	D3Ertd300e	56790	3	54327325	54351057	+
6245	1433266_at	Region	2810416A17Rik	70241	14	94465299	94466451	-
6246	1421896_at	Region	Elk1	13712	X	19173867	19189533	-
6247	1419300_at	Region	Flt1	14254	5	146453691	146617483	-
6248	1422890_at	Region	Pcdh18	73173	3	49331829	49346004	-
6249	1424780_a_at	Region	D10Ucla1	28193	10	67048137	67134030	-
6250	1451180_a_at	Region	C330027I04Rik	68106	11	100243408	100262161	-
6251	1436304_at	Region	C030003D03Rik	77220	10	25982477	26078288	-
6252	1433520_at	Region	Scap	235623	9	110373444	110426587	+
6253	1460549_a_at	Region	Cdc23	52563	18	34854705	34874773	-
6254	1433846_s_at	Region	C430003P19Rik	109359	7	127237961	127251442	+
6255	1460547_a_at	Region	Hnrpk	15387	13	57024107	57034672	-
6256	1457651_x_at	Region	Rem2	140743	14	48993511	48997845	+
6257	1426688_at	Region	Sdha	66945	13	70384460	70412084	-

6258	1437136_at	Region	5830436I19Rik	319946	17	45048588	45049456	+
6259	1417403_at	Region	Elovl6	170439	3	128465310	128571362	+
6260	1448679_at	Region	Hyal2	15587	9	107634944	107638559	+
6261	1452507_at	Region	Dlx6	13396	6	6836503	6840727	+
6262	1424263_at	Region	2810003C17Rik	108897	2	31882465	31905604	+
6263	1426895_at	Region	Zfp191	59057	18	24240763	24249267	-
6264	1435464_at	Region	1110003E01Rik	68552	5	64251633	64294659	-
6265	1425030_at	Region	Zfp622	52521	15	25973256	25987372	+
6266	1460209_at	Region	Usp39	28035	6	72650650	72677149	-
6267	1426529_a_at	Region	Tagln2	21346	1	172429183	172436299	+
6268	1416316_at	Region	Slc27a2	26458	2	126067008	126101789	+
6269	1420038_at	Region	Atp6v1e1	11973	6	121227218	121254659	-
6270	1424648_at	Region	Rabl4	67042	15	78211456	78226101	-
6271	1439173_at	Region	Hook1	77963	4	94942452	94999350	+
6272	1424251_a_at	Region	Hnrpdl	50926	5	99053360	99057516	-
6273	1428314_at	Region	Pcnp	328694	16	54911436	54920579	-
6274	1448820_a_at	Region	Eif2s2	67204	2	154328353	154349719	-
6275	1442625_at	Region	None	None	14	40604571	40605076	-
6276	1437200_at	Region	Fcho2	218503	13	94911844	95003580	-
6277	1440519_at	Region	Sp8	320145	12	114659034	114664054	+
6278	1429124_s_at	Region	Tceb3bp1	66932	10	80672809	80692760	-
6279	1440481_at	Region	None	None	1	52422237	52423203	+
6280	1440566_at	Region	D330005C11Rik	319398	5	141117468	141118174	+
6281	1451459_at	Region	Ahctf1	226747	1	179700337	179757911	-
6282	1450072_at	Region	Ash1l	192195	3	88709675	88822741	+
6283	1425141_at	Region	None	None	1	13758085	13793589	-
6284	1452917_at	Region	Rfc5	72151	4	116507926	116517824	-
6285	1418117_at	Region	Ndufs4	17993	13	110702711	110802452	-
6286	1452999_at	Region	Smndc1	76479	19	52947147	52958506	-
6287	1420828_s_at	Region	Ywhaq	22630	12	18728420	18754333	+
6288	1433730_at	Region	Elmod2	244548	8	82560189	82579982	-
6289	1429110_a_at	Region	2810405F18Rik	72181	4	114991747	115012781	-
6290	1418201_at	Region	Plekhg2	101497	7	23762000	23774164	-
6291	1426360_at	Region	Zc3h11a	70579	1	133482838	133516350	-
6292	1448831_at	Region	Angpt2	11601	8	18636337	18686551	-
6293	1436202_at	Region	9430072K23Rik	77292	19	5584481	5586545	-
6294	1460678_at	Region	Klhdc2	69554	12	66128557	66142253	+
6295	1437003_at	Region	None	None	8	106316713	106317403	+
6296	1451191_at	Region	Crabp2	12904	3	87692354	87697104	+
6297	1454390_at	Region	Lrch4	231798	5	136579783	136591102	+
6298	1439051_a_at	Region	Mark4	232944	7	16295319	16327738	-
6299	1458182_at	Region	Rbm18	67889	2	36048243	36068773	-
6300	1442525_at	Region	2610204L23Rik	67163	11	106021555	106037392	-
6301	1422539_at	Region	Extl2	58193	3	114777993	114799388	+
6302	1460577_at	Region	AA591059	105098	13	89614530	89615176	-
6303	1444065_at	Region	9330151E16Rik	192986	11	72520694	72521700	-
6304	1422793_at	Region	Pafah1b2	18475	9	45981441	45998071	-
6305	1434003_a_at	Region	Dhps	330817	8	84342639	84345999	+
6306	1440095_at	Region	None	None	7	124625642	124626112	-
6307	1426716_at	Region	Tdrd7	100121	4	45903384	45950683	+
6308	1445787_at	Region	5033413D22Rik	75973	10	41640673	41689577	-
6309	1454784_at	Region	Hs3st2	195646	7	115360837	115377332	+
6310	1425623_a_at	Region	Cbs	12411	17	29420528	29445025	-
6311	1416835_s_at	Region	Amd1 /// Amd2 /// LOC432454	11702	10	40363437	40378151	-
6312	1424316_at	Region	Slc25a19	67283	11	115436076	115449232	-
6313	1428090_at	Region	2810422B04Rik	69956	6	72212611	72240739	-
6314	1456128_at	Region	Atp5g2	67942	15	102723287	102731466	-
6315	1458140_at	Region	Slit2	20563	5	46748038	47069334	+
6316	1429554_at	Region	Sdccag3	68112	2	26315523	26321468	-
6317	1457432_at	Region	Prox1	19130	1	189836615	189885751	-
6318	1417937_at	Region	Dact1	59036	12	68146768	68155678	+
6319	1458379_at	Region	9330159F19Rik	212448	10	29242835	29259565	+
6320	1416731_at	Region	Top2b	21974	14	14861822	14927407	+
6321	1438164_x_at	Region	Flot2	14252	11	77765963	77786091	+

6322	1449184_at	Region	Pglyrp1	21946	7	15754519	15760353	+
6323	1444386_at	Region	Pde1c	18575	6	56226106	56506844	-
6324	1424658_at	Region	Taok1	216965	11	77255718	77311108	-
6325	1460358_s_at	Region	Nudt22	68323	19	6706867	6709885	-
6326	1419030_at	Region	Ero1l	50527	14	950	11169	+
6327	1417953_at	Region	D6Wsu176e	27999	6	22353558	22403117	-
6328	1439877_at	Region	Ext1	14042	15	53068219	53345629	-
6329	1447062_at	Region	Vps24	66700	6	71875831	71913551	+
6330	1424752_x_at	Region	Zfp71-rs1 /// 6720487G11Rik	235907	13	1294	6997	-
6331	1421754_at	Region	AY036118	170798		14927945	14929071	+
6332	1449940_a_at	Region	Eif2b4	13667	5	29646506	29652031	-
6333	1442924_at	Region	Clstn2	64085	9	97343753	97483061	-
6334	1434918_at	Region	Sox6	20679	7	109336962	109662307	-
6335	1437113_s_at	Region	Pld1	18805	3	27441540	27550616	+
6336	1416536_at	Region	Mum1	68114	10	80353235	80368810	+
6337	1436053_at	Region	Tbc1d22b	381085	17	27359185	27416188	+
6338	1438103_at	Region	Pcdhgc4	93707	18	38041516	38042278	+
6339	1455310_at	Region	Rbm16	106583	17	3065810	3160145	+
6340	1449443_at	Region	Decr1	67460	4	15844205	15872472	-
6341	1438955_x_at	Region	Ppif	105675	14	24069368	24075666	+
6342	1421975_a_at	Region	Add2	11519	6	86507734	86549220	+
6343	1441979_at	Region	BC060267	212516	6	116248431	116275947	-
6344	1436715_s_at	Region	Cdipt	52858	7	121026080	121030232	+
6345	1437730_at	Region	Ppp2r2a	71978	14	61544871	61704372	-
6346	1451288_s_at	Region	1810043G02Rik	67884	10	78087790	78093995	+
6347	1428996_at	Region	Gm1705	382051	8	103888941	103889832	+
6348	1416505_at	Region	Nr4a1	15370	15	101325011	101332941	+
6349	1457543_at	Region	4921525L17Rik	70918	5	65025474	65063365	+
6350	1455803_at	Region	Slco4a1	108115	2	180177936	180191810	+
6351	1439543_at	Region	1110064A23Rik	68893	2	31880307	31880831	+
6352	1434469_at	Region	D8Ertd69e	73945	8	78854651	78892469	+
6353	1437225_x_at	Region	Gnai3	14679	3	107903041	107941856	-
6354	1455697_at	Region	E130113E03Rik	319433	14	57089999	57211210	+
6355	1435170_at	Region	2310007F12Rik	69499	X	144621704	144630913	-
6356	1426630_at	Region	Exosc2	227715	2	31602898	31613482	+
6357	1417382_at	Region	Entpd5	12499	12	81245023	81273816	-
6358	1432504_at	Region	1700106N22Rik	73582	17	82920574	83288888	+
6359	1435352_at	Region	Pigk	329777	3	151707256	151782191	+
6360	1435292_at	Region	Tbc1d4	210789	14	95967184	95986190	-
6361	1428229_at	Region	Prkcn	75292	17	76772784	76835125	-
6362	1416691_at	Region	Gtpbp2	56055	17	43670992	43679289	+
6363	1456919_at	Region	A930013B10Rik	414094	11	88884711	88890185	+
6364	1418562_at	Region	Sf3b1	81898	1	55286605	55333156	-
6365	1429392_at	Region	Wdr40a	68970	4	41430155	41453667	-
6366	1453362_x_at	Region	Rps24	20088	14	22859652	22865117	+
6367	1417322_at	Region	Hlrc1	102115	10	81519803	81523644	+
6368	1429491_s_at	Region	Rif1	51869	2	52004994	52054222	+
6369	1416300_a_at	Region	Slc25a3	18674	10	91087904	91095289	-
6370	1446949_at	Region	4930431P19Rik	73886	7	103607453	103608424	+
6371	1433680_x_at	Region	MGI:1353606 /// LOC433642	30954	12	108124289	108128532	+
6372	1439749_at	Region	Zap70	22637	1	37056452	37077407	+
6373	1427093_at	Region	1500031N24Rik	69020	15	76010507	76025834	+
6374	1444336_at	Region	None	None	2	55360356	55361377	-
6375	1451927_a_at	Region	Mapk14	26416	17	26492551	26549631	+
6376	1446382_at	Region	None	None	10	102330633	102331300	+
6377	1459011_at	Region	Btbd9	224671	17	28030141	28340405	-
6378	1417511_at	Region	Lyar	17089	5	36726775	36740522	+
6379	1440360_at	Region	Arl6ip5	65106	6	97677079	97699198	+
6380	1428532_at	Region	5930412E23Rik	77065	1	191312045	191358940	+
6381	1441391_at	Region	Gnb1l	13972	16	17269870	17335574	+
6382	1422673_at	Region	Prkcm	18760	12	47080386	47388266	-
6383	1417801_a_at	Region	Ppfibp2	19024	7	101545266	101694885	+
6384	1454859_a_at	Region	Rpl23	65019	11	97598630	97603460	-

6385	1443713_at	Region	Chl1	12661	6	103991578	104209457	+
6386	1430360_at	Region	4833412C15Rik	74613	15	77165687	77167758	-
6387	1437533_at	Region	Birc4	11798	X	36614524	36626297	+
6388	1416367_at	Region	1110001J03Rik	66117	6	38671070	38675658	+
6389	1444041_at	Region	AU041133	103369	10	82031015	82031654	+
6390	1434285_at	Region	Frmd4a	209630	2	4348468	4531213	+
6391	1451700_a_at	Region	1110007L15Rik	67604	5	138250433	138268108	+
6392	1441313_x_at	Region	Lhx9	16876	1	138672251	138686188	-
6393	1423089_at	Region	Tmod3	50875	9	75635361	75697149	-
6394	1418905_at	Region	Nubp1	26425	16	10086475	10098031	+
6395	1417307_at	Region	Dmd	13405	X	78006916	79865697	+
6396	1433486_at	Region	Clcn3	12725	8	59960629	60030782	-
6397	1449023_a_at	Region	Ezh1	14055	11	101012203	101049035	-
6398	1437185_s_at	Region	Tmsb10	19240	6	20039912	20040386	+
6399	1418603_at	Region	Avpr1a	54140	10	122099734	122104688	+
6400	1424047_at	Region	Dera	232449	6	138546535	138629830	+
6401	1416534_at	Region	Dpf2	19708	19	5686167	5701613	-
6402	1423373_at	Region	Rpp30	54364	19	35426401	35447443	+
6403	1434069_at	Region	BC067047	277360	2	166023078	166170565	-
6404	1450981_at	Region	Cnn2	12798	10	80111268	80118024	+
6405	1452627_at	Region	Senp6	215351	9	80272603	80350480	+
6406	1430073_at	Region	2900016B01Rik	67262	8	14389507	14405568	+
6407	1428527_at	Region	Snx7	76561	3	116555087	116642555	-
6408	1455336_at	Region	9030625G08Rik	66816	10	115056945	115071473	-
6409	1436205_at	Region	Nfasc	269116	1	132427503	132603217	-
6410	1454211_a_at	Region	Shrm	27428	5	92019185	92300704	+
6411	1447301_at	Region	Gm258	238276	12	73183905	73187107	+
6412	1421148_a_at	Region	Tial1	21843	7	122491640	122510627	-
6413	1426628_at	Region	Tmem34	234463	8	76808045	76823552	-
6414	1442382_at	Region	Mast4	328329	13	98930827	99546711	-
6415	1426936_at	Region	LOC277193 /// LOC432650 /// LOC432898 /// LOC433593 /// LOC434667	277193	10	25882199	25886543	+
6416	1416989_at	Region	3100002B05Rik	68299	11	75771884	75905288	-
6417	1455874_at	Region	1810059G22Rik	67706	19	7969076	7970989	-
6418	1427088_at	Region	Ccnt2	72949	1	127616552	127647225	+
6419	1440310_at	Region	Cbfa2t1h	12395	4	13711747	13818082	+
6420	1424492_at	Region	Trpc2	22064	7	96181838	96202292	+
6421	1416496_at	Region	Mrfap1	67568	5	35301379	35303216	-
6422	1436703_x_at	Region	Snapc2	102209	8	3608060	3611178	+
6423	1428658_at	Region	Pin4	69713	X	96720808	96733533	+
6424	1447811_s_at	Region	Amigo1	229715	3	107982077	107987519	+
6425	1456512_at	Region	Pdzrn4	239618	15	92665020	92838995	+
6426	1416614_at	Region	Cri1	58521	2	125186948	125188636	+
6427	1429231_at	Region	None	None	7	73507486	73509559	+
6428	1458432_at	Region	Nck2	17974	1	43740468	43865231	+
6429	1455810_a_at	Region	E130112L23Rik	268739	14	47085678	47106098	+
6430	1439707_at	Region	C230034O21Rik	319342	3	38348095	38348495	-
6431	1433686_at	Region	Cabin1	104248	10	75747852	75760539	-
6432	1434963_at	Region	Supt3h	109115	17	42281336	42624366	+
6433	1434961_at	Region	Asb1	65247	1	91386183	91386800	+
6434	1436377_at	Region	AI428855	107173	19	6651918	6655327	-
6435	1453002_at	Region	Sox11	20666	12	23886753	23889164	-
6436	1448486_at	Region	Mut	17850	17	38451138	38475743	+
6437	1453928_a_at	Region	Ssb	20823	2	69717028	69727204	+
6438	1423737_at	Region	Ndufs3	68349	2	90599385	90609470	-
6439	1436767_at	Region	Luc7l2	192196	6	38687728	38745622	+
6440	1423390_at	Region	Siah1a	20437	8	86007609	86029536	-
6441	1440104_at	Region	Ranbp2	19386	10	58404785	58452252	+
6442	1448241_at	Region	Gm2a	14667	11	54850958	54863739	+
6443	1445461_at	Region	Lztr1	66863	16	16279939	16297298	+
6444	1424606_at	Region	Cplx3	235415	9	57713916	57720156	-
6445	1427255_s_at	Region	Zfp445	235682	9	122869105	122879937	-

6446	1440491_at	Region	Slc1a3	20512	15	8424368	8500974	-
6447	1424045_at	Region	5730437N04Rik	70544	17	5318249	5347622	-
6448	1448311_at	Region	Usp5	22225	6	125471787	125486215	-
6449	1423791_at	Region	Ik	24010	18	36968481	36981400	+
6450	1426949_s_at	Region	Tpr	108989	1	150229615	150286691	+
6451	1447237_at	Region	Pde4b	18578	4	101213908	101564744	+
6452	1417346_at	Region	Pycard	66824	7	122041913	122043557	-
6453	1453734_at	Region	Atrx	22589	X	100401518	100530707	-
6454	1439603_at	Region	---	435662	2	35423370	35490493	-
6455	1419292_at	Region	Htra3	78558	5	34139553	34167077	-
6456	1424149_at	Region	1110014D18Rik	68501	15	59384784	59612226	+
6457	1428616_at	Region	Zfp131	72465	13	10118	57012	+
6458	1448880_at	Region	Ube2l3	22195	16	15923031	15972515	-
6459	1430816_at	Region	3110003A22Rik	68053	4	6118254	6143705	+
6460	1432680_at	Region	9130009M17Rik	71588	19	40018920	40020861	-
6461	1421405_at	Region	Zim1	22776	7	5878764	5899877	-
6462	1421215_a_at	Region	Slmap	83997	14	24509380	24629945	-
6463	1427256_at	Region	Cspg2	13003	13	85771194	85857423	-
6464	1426247_at	Region	Stk24	223255	14	115857532	115949796	-
6465	1441528_at	Region	Ttc15	217449	12	25256255	25315622	-
6466	1438539_at	Region	Ascc1	69090	10	59958483	60060150	+
6467	1437484_at	Region	Zbtb5	230119	4	44907164	44928192	-
6468	1425037_at	Region	Fgd4	224014	16	15192773	15330937	-
6469	1455260_at	Region	A830039H10Rik	209707	5	44458346	44613918	-
6470	1455731_at	Region	Slc29a3	71279	10	60670868	60713357	-
6471	1451984_at	Region	Hnrpul1	232989	7	20886817	20919507	-
6472	1419429_at	Region	Cntfr	12804	4	41796349	41835894	-
6473	1426422_at	Region	BC016188	212139	8	83395895	83410820	-
6474	1416439_at	Region	2410015N17Rik	66422	7	121306690	121310398	-
6475	1420365_a_at	Region	Hnrpa2b1	53379	6	51608402	51617123	-
6476	1459835_s_at	Region	Dnaja1	15502	4	40861384	40873815	+
6477	1444452_at	Region	D930002L09Rik	320876	3	44860222	44860878	+
6478	1457125_at	Region	None	None	3	152201308	152201929	-
6479	1441083_at	Region	None	None	7	16500824	16501250	-
6480	1450724_at	Region	MGI:2149839	84652	5	22424927	22494571	-
6481	1432439_at	Region	2610041B18Rik	69930	7	37370962	37388012	-
6482	1448395_at	Region	Sfrp1	20377	8	22166110	22202536	+
6483	1435433_at	Region	Centg2	347722	1	89273393	89708522	+
6484	1438378_at	Region	1700013G23Rik	69378	11	59672552	59673570	-
6485	1442667_at	Region	Cdc40	71713	10	40910293	40992658	-
6486	1421743_a_at	Region	Pcbp2	18521	15	102531206	102559300	+
6487	1455039_a_at	Region	Sin3b	20467	8	71873343	71907207	+
6488	1454724_x_at	Region	5730446C15Rik	226016	19	20916280	20924330	+
6489	1428383_a_at	Region	2310021P13Rik	268721	14	19082480	19084932	+
6490	1435455_at	Region	C79267	212632	4	138478741	138498358	+
6491	1428508_at	Region	1810061M12Rik	67016	9	90089335	90158057	-
6492	1426827_at	Region	A730098D12Rik	231386	5	86084480	86098514	+
6493	1448828_at	Region	Smc6l1	67241	12	10625170	10671328	+
6494	1428941_at	Region	Zfp198	76007	14	51415856	51488606	+
6495	1455014_at	Region	AV009015	103388	10	30645939	30647381	-
6496	1431123_s_at	Region	Dnajc8	68598	4	131496455	131514634	+
6497	1450472_s_at	Region	Smad3	17127	9	63767269	63878762	-
6498	1429816_at	Region	Armc3	70882	2	19241312	19352242	+
6499	1417972_s_at	Region	Pop5	117109	5	114345472	114350598	+
6500	1443939_at	Region	LOC230628	230628	4	113368101	113569645	+
6501	1454790_at	Region	Wdr35	74682	12	8290207	8345162	+
6502	1455277_at	Region	Hhip	15245	8	79185726	79272113	-
6503	1426679_at	Region	3110006P09Rik	68036	15	36999940	37010315	-
6504	1418828_at	Region	Thex1	67276	8	34268325	34298553	-
6505	1432385_a_at	Region	Agtpbp1	67269	13	58109493	58216884	-
6506	1435966_x_at	Region	Mrpl13	68537	15	55539170	55562387	-
6507	1418295_s_at	Region	Dgat1	13350	15	76552397	76562200	-
6508	1448865_at	Region	Hsd17b7	15490	1	169883718	169903346	-
6509	1444157_a_at	Region	Jarid1c	20591	X	145767667	145808191	+
6510	1436985_at	Region	Zfp644	52397	5	105678595	105758844	-

6511	1441998_at	Region	None	None	9	66718053	66718735	+
6512	1439775_at	Region	Brwd3	382236	X	103336563	103435714	-
6513	1429762_a_at	Region	Bbs5	72569	2	69502655	69522969	+
6514	1417851_at	Region	Cxcl13	55985	5	94970111	94974240	+
6515	1424332_at	Region	Rab40c	224624	17	23689151	23711170	-
6516	1430066_at	Region	4930589M24Rik	75906	10	53878840	53940180	-
6517	1444126_at	Region	None	None	17	62026280	62027138	-
6518	1434775_at	Region	Pard3	93742	8	126354044	126919239	+
6519	1438315_x_at	Region	Akr7a5	110198	4	138192005	138199686	+
6520	1452840_at	Region	1500009L16Rik	69784	10	83610822	83650687	+
6521	1435472_at	Region	Kremen1	84035	11	5086339	5156397	-
6522	1433753_x_at	Region	Eral1	57837	11	77799035	77806004	-
6523	1440020_at	Region	Rlf	109263	4	120167817	120237523	-
6524	1454698_at	Region	AW742319	57874	9	65109184	65143902	-
6525	1456879_at	Region	C130022K22Rik	232236	6	92326024	92347807	+
6526	1452461_a_at	Region	MGC79224	432486	10	88350966	88419158	+
6527	1447483_s_at	Region	2610002F03Rik	72091	2	26571927	26572166	-
6528	1435834_at	Region	Gas2	14453	7	46048672	46163985	+
6529	1453709_at	Region	3426406O18Rik	70677	9	32185933	32186952	+
6530	1460737_at	Region	Igbp1	18518	X	95095706	95117362	+
6531	1436630_at	Region	None	None	7	91770544	91770961	-
6532	1428409_at	Region	Mak3	72117	16	43023139	43046694	+
6533	1433877_at	Region	4732473B16Rik	100342	4	132441036	132448835	+
6534	1438699_at	Region	Srd5a1	78925	13	65625480	65642329	-
6535	1423661_s_at	Region	Ctdsp2	52468	10	126728331	126732688	+
6536	1449632_s_at	Region	Fkbp10	14230	11	100236784	100245912	+
6537	1434184_s_at	Region	9430080K19Rik	68120	1	40319317	40320478	+
6538	1433801_at	Region	9930012K11Rik	268759	14	64470394	64475460	-
6539	1418265_s_at	Region	Irf2	16363	8	45686560	45794326	+
6540	1423026_at	Region	Rad51c	114714	11	87107378	87132413	-
6541	1425861_x_at	Region	Cacna2d1	12293	5	14583526	14791513	+
6542	1426461_at	Region	Ugp2	216558	11	21215937	21265268	-
6543	1429926_at	Region	6720473G16Rik	77078	X	106710866	106711990	+
6544	1450954_at	Region	Yme1l1	27377	2	23088708	23130798	+
6545	1430074_x_at	Region	8430426H19Rik	71508	13	7134	10068	+
6546	1455553_at	Region	5330401P04Rik	227094	1	52934448	52954337	+
6547	1436681_x_at	Region	0610009D07Rik	66055	12	3985835	3999611	+
6548	1450953_at	Region	Wdr39	26371	2	126754791	126761595	-
6549	1425507_at	Region	Arfrp1	76688	2	181074790	181082318	-
6550	1452198_at	Region	Fbxl10	30841	5	122023577	122142001	-
6551	1433866_x_at	Region	Prdx1	18477	4	115644504	115658905	+
6552	1452526_a_at	Region	Pax6	18508	2	105381199	105401823	+
6553	1418167_at	Region	Tcfap4	83383	16	4216161	4231223	-
6554	1444897_at	Region	AU017263	104936	12	94771317	94772245	+
6555	1442302_at	Region	None	None	11	32125455	32126040	+
6556	1425646_at	Region	BC016495	225994	19	17878968	17899143	+
6557	1419734_at	Region	Actb	11461	5	141967352	141970960	-
6558	1435899_at	Region	9430079B08Rik	320481	1	106661191	106663294	-
6559	1428643_at	Region	Mgat5	107895	1	127142098	127323193	+
6560	1460698_a_at	Region	Sec11l3	66286	18	66033804	66050883	+
6561	1423710_at	Region	Dlst	78920	12	81979590	82002780	+
6562	1460254_at	Region	1810049H13Rik	66431	11	120277697	120279156	-
6563	1427285_s_at	Region	Malat1	72289	19	5588755	5591120	-
6564	1456349_x_at	Region	Sumo1	22218	1	59942975	59974347	-
6565	1448735_at	Region	Cp	12870	3	19298453	19333864	+
6566	1445191_at	Region	4932702D22Rik	241624	2	119033253	119061476	-
6567	1421733_a_at	Region	Tpst1	22021	5	129243151	129305554	+
6568	1457694_at	Region	6430537H07Rik	226265	19	58542983	58570971	+
6569	1439597_at	Region	4932417H02Rik	74370	11	119424903	119718575	+
6570	1443279_at	Region	Nlk	18099	11	78292827	78423032	-
6571	1441635_at	Region	Nr6a1	14536	2	38655532	38859850	-
6572	1424204_at	Region	Mrpl13	68537	15	55539170	55562387	-
6573	1418244_at	Region	Nat5	67877	2	145360205	145373252	+
6574	1436758_at	Region	Hdac4	208727	1	91760230	91975898	-
6575	1428285_at	Region	8430427H17Rik	329540	2	152864402	152940719	-

6576	1448958_at	Region	MGI:1929890	64164	1	155937531	155950350	+
6577	1422533_at	Region	Cyp51	13121	5	4088954	4110454	-
6578	1435257_at	Region	Klhl17	231003	NONE	NONE	NONE	NONE
6579	1436102_at	Region	Sec22l3	215474	9	121695485	121719087	-
6580	1436996_x_at	Region	Lzp-s	17110	10	116977248	116982319	-
6581	1418339_at	Region	Recql	19691	6	143205415	143230300	-
6582	1418964_at	Region	Pigm	67556	1	172305475	172309452	+
6583	1458969_at	Region	AU019559	102225	8	11158707	11159185	+
6584	1450405_at	Region	Mrpl19	56284	6	82245436	82301881	-
6585	1458439_a_at	Region	2310047C04Rik	224170	16	47767664	47835780	-
6586	1424644_at	Region	Tbcc	72726	17	44403093	44404256	+
6587	1437709_x_at	Region	Ttc13	234875	8	123958404	124009066	-
6588	1458679_a_at	Region	Tatdn1	69694	15	58900953	58944887	-
6589	1435866_s_at	Region	Hist3h2a	319162	11	58680314	58682303	+
6590	1452042_a_at	Region	5730537D05Rik	70652	3	79543274	79572782	-
6591	1456153_at	Region	Ssh2	237860	11	77053112	77181207	+
6592	1419521_at	Region	Zfp94	22756	7	19475849	19490824	-
6593	1427922_at	Region	2310061C15Rik	66531	8	116217815	116249733	-
6594	1433803_at	Region	Jak1	16451	4	100111937	100224138	-
6595	1449222_at	Region	Ebi3	50498	17	53590984	53595366	+
6596	1426242_at	Region	Polr2a	20020	11	69460068	69483881	-
6597	1424322_at	Region	Apex2	77622	X	144105902	144122544	-
6598	1452916_at	Region	2610014H22Rik	75410	7	25981548	25983534	-
6599	1435556_at	Region	Zfp597	71063	16	3533092	3535419	-
6600	1421323_a_at	Region	E430034L04Rik	23881	5	91390328	91419486	-
6601	1453750_x_at	Region	Pitpnc1	71795	11	107033195	107291788	-
6602	1442484_at	Region	D9Ertd306e	52203	9	88820023	88820728	-
6603	1434622_at	Region	Iqce	74239	5	139662337	139701210	-
6604	1433534_a_at	Region	Cct2	12461	10	116740451	116753217	-
6605	1423345_at	Region	Degs1	13244	1	182232966	182239955	-
6606	1450113_at	Region	None	None	12	75605546	75695819	+
6607	1416223_at	Region	BC003251	79566	11	58056379	58073354	+
6608	1433447_x_at	Region	Cct4	12464	11	22885383	22898126	+
6609	1427881_at	Region	4930588M11Rik	99480	3	121052751	121063608	+
6610	1424625_a_at	Region	6030446I19Rik	227801	2	37731153	38219509	-
6611	1419617_at	Region	Kcnn1	84036	8	69998871	70011298	-
6612	1440650_at	Region	Slit2	20563	5	46748038	47069334	+
6613	1455138_x_at	Region	Cfl1	12631	19	5279315	5283991	+
6614	1423235_at	Region	Ap3b1	11774	13	90543097	90750381	+
6615	1442583_a_at	Region	AI854703	243373	6	48760814	48766567	+
6616	1448079_at	Region	Rnf166	68718	8	121847734	121858060	-
6617	1420795_at	Region	Fgf9	14180	14	52607790	52644523	+
6618	1453263_at	Region	Mak10	78689	13	58245009	58294396	+
6619	1440026_at	Region	Trps1	83925	15	50659276	50888038	-
6620	1429471_at	Region	1110017D15Rik	73721	4	41643865	41656183	-
6621	1453013_at	Region	1110034O07Rik	68744	15	102267622	102274961	+
6622	1420945_at	Region	Atrx	22589	X	100401518	100530707	-
6623	1426292_at	Region	6330581L23Rik	233056	7	25230836	25245657	+
6624	1427446_s_at	Region	Ttn	22138	2	76401836	76406317	-
6625	1458489_at	Region	Prkag1	19082	15	98869730	98888520	-
6626	1434056_a_at	Region	Gm137	230075	4	40409442	40418222	-
6627	1460553_at	Region	1700025K23Rik	66337	10	39188204	39203291	-
6628	1431360_s_at	Region	4921520P21Rik	70887	X	97504587	97510052	-
6629	1451471_at	Region	3230401I01Rik	67417	7	115916679	115944921	-
6630	1422766_at	Region	Stau1	20853	2	166405006	166453032	-
6631	1423118_at	Region	1200014J11Rik	66874	11	72773538	72805874	+
6632	1421729_a_at	Region	Fert2	14158	17	61637960	61842673	+
6633	1432182_at	Region	4922502B01Rik	70954	8	108548150	108549390	-
6634	1441349_at	Region	LOC552904	552904	3	128945934	128946968	-
6635	1440440_at	Region	None	None	9	13318586	13319228	+
6636	1426842_at	Region	Ythdf3	229096	3	15522868	15556473	+
6637	1455311_at	Region	Dgcr8	94223	16	17025006	17060210	-
6638	1429186_a_at	Region	Cdadc1	71891	14	54094451	54133002	-
6639	1457175_at	Region	Numb	18222	12	80659465	80706369	-
6640	1441931_x_at	Region	Gss	14854	2	155020120	155044513	-

6641	1435036_at	Region	A530050D06Rik	104816	12	107586202	107606905	+
6642	1455437_at	Region	BC033915	70661	9	46026130	46236394	+
6643	1454726_s_at	Region	AW456874	218232	13	48175930	48223631	-
6644	1448727_at	Region	Tle6	114606	10	81727590	81737585	-
6645	1424821_at	Region	Ndfip1	65113	18	38640514	38688909	+
6646	1436379_at	Region	2610312F20Rik	70461	7	74390859	74491412	-
6647	1456498_at	Region	Itga4	16401	2	78953394	79028523	+
6648	1430522_a_at	Region	Vamp5	53620	6	72701002	72702306	-
6649	1458641_at	Region	Braf	109880	6	39749542	39860924	-
6650	1443829_x_at	Region	Coasy	71743	11	100903693	100907704	+
6651	1420618_at	Region	Cpeb4	67579	11	31767001	31826367	+
6652	1430623_s_at	Region	5830411E10Rik	109019	1	51769634	51779343	-
6653	1435055_a_at	Region	Tom1 /// LOC545878	21968	8	74197771	74234173	+
6654	1425007_at	Region	Zfp566	72556	7	25492062	25505056	-
6655	1434254_at	Region	Gna11	14672	10	81666416	81680827	-
6656	1454648_s_at	Region	D10Wsu102e	28109	10	83248177	83256759	+
6657	1424259_at	Region	2400010G15Rik	76483	17	23384865	23468723	+
6658	1422110_at	Region	Hr	15460	14	64870045	64889532	+
6659	1424947_at	Region	Dnclic1	235661	9	114662314	114697320	+
6660	1417046_at	Region	Tusc4	56032	9	107608007	107611476	+
6661	1437057_at	Region	Egfl3	230971	4	152663105	152768075	+
6662	1452159_at	Region	2310001A20Rik	71881	2	150040017	150065459	-
6663	1435965_at	Region	Cnot3	232791	7	95942392	95943786	-
6664	1430555_s_at	Region	Lrig3	320398	10	125665572	125714849	+
6665	1424458_at	Region	Jmjd2c	76804	4	73313167	73467080	+
6666	1420887_a_at	Region	Bcl2l1	12048	2	152237604	152288618	-
6667	1459457_at	Region	Camk2d	108058	3	125480582	125726370	+
6668	1417072_at	Region	Slc22a6	18399	19	7814586	7823070	+
6669	1450657_at	Region	Ppie	56031	4	122154237	122167053	-
6670	1460094_at	Region	AI790205	277463	2	31084496	31148729	+
6671	1442617_at	Region	Cbfb	12400	8	104466302	104513614	+
6672	1416763_at	Region	G431001I09Rik	98828	2	5711566	5762232	-
6673	1459839_x_at	Region	Btbd11	74007	10	85552377	85552631	+
6674	1439416_x_at	Region	4930542G03Rik /// LOC433839	227613	2	25154324	25156803	-
6675	1433387_at	Region	2900022M07Rik	72951	NONE	NONE	NONE	NONE
6676	1452925_a_at	Region	38416	69104	19	99847336	99848552	-
6677	1453127_at	Region	Ppm1j	71887	3	104207837	104212729	+
6678	1453360_a_at	Region	Tex9	21778	9	72593752	72624808	-
6679	1426446_at	Region	6430548M08Rik	234797	8	119500738	119518927	+
6680	1436620_at	Region	4732496G21Rik	320162	11	106610364	106639918	+
6681	1438517_at	Region	Wwox	80707	8	113753199	114666211	+
6682	1443799_at	Region	Ppp1r14c	76142	10	7041618	7140096	-
6683	1427067_at	Region	4933439F18Rik	66771	11	60142945	60170919	+
6684	1424902_at	Region	Plxdc1	72324	11	97744325	97807467	-
6685	1424341_s_at	Region	Pcdha4 /// Pcdha6 /// Pcdha7 /// Pcdha5 /// Pcdha11 /// Pcdha10 /// Pcdha1 /// Pcdha9 /// Pcdha3 /// Pcdha12 /// Pcdha2 /// Pcdha8 /// Pcdhac1 /// Pcdhac2	116731	18	37367691	37411386	+
6686	1459317_at	Region	Ank2	109676	3	125805551	125881703	-
6687	1426614_at	Region	Prkcbp1	228880	2	165242034	165353683	-
6688	1443182_at	Region	A930041C12Rik	78443	5	106693490	106694118	+
6689	1423993_at	Region	Atp6v1f	66144	6	29514880	29517606	+
6690	1439728_at	Region	D330027H18Rik	101359	6	29216329	29226681	-
6691	1460693_a_at	Region	Col9a3	12841	2	180315202	180339143	+
6692	1455929_x_at	Region	Ppp2r1a	51792	17	18817271	18837722	+
6693	1452817_at	Region	Smyd3	69726	1	178910022	179472249	-

6694	1457548_at	Region	Adamts6	108154	13	100587418	100712006	+
6695	1416880_at	Region	Mcl1	17210	3	95146619	95151011	+
6696	1451901_at	Region	Smarca3	20585	3	19399088	19457883	+
6697	1434019_at	Region	Pdap1	231887	5	144168088	144178675	-
6698	1416616_s_at	Region	Clpp	53895	17	54680231	54686109	+
6699	1430195_at	Region	2810043O03Rik	72697	9	72118680	72120526	-
6700	1455112_at	Region	Amid	71361	10	61681617	61705566	+
6701	1424179_at	Region	Plekhj1	78670	10	80928041	80930223	-
6702	1454774_at	Region	Zfp445	235682	9	122869105	122879937	-
6703	1454971_x_at	Region	Tgfb1i4	21807	14	70758316	70850256	+
6704	1429367_at	Region	2510001I10Rik	74781	5	141637713	141677424	+
6705	1458807_at	Region	Epb4.1	269587	4	130721061	130868982	-
6706	1429431_at	Region	Zfpn1a5	67143	7	125704215	125726182	-
6707	1438977_x_at	Region	Ran	19384	5	128195181	128199286	+
6708	1419670_at	Region	Ftcd	14317	10	76676702	76691057	+
6709	1441567_at	Region	4732465J09Rik	270163	9	59868073	60046149	+
6710	1425742_a_at	Region	Tgfb1i4	21807	14	70758316	70850256	+
6711	1426841_at	Region	Ythdf3	229096	3	15522868	15556473	+
6712	1419173_at	Region	Acy1	109652	9	106426550	106431807	-
6713	1423415_at	Region	Gpr83	14608	9	14693694	14702939	+
6714	1434308_at	Region	Slc43a2	215113	11	75257363	75300699	+
6715	1417713_at	Region	Eif2s2	67204	2	154328353	154349719	-
6716	1416376_at	Region	1810014L12Rik	69071	11	78267476	78276394	-
6717	1459677_at	Region	None	None	3	95357721	95357967	-
6718	1419460_at	Region	Rpp14	67053	14	5748595	5760064	+
6719	1437642_at	Region	Hrbl	231801	5	136601442	136635293	-
6720	1416425_at	Region	Pex19	19298	1	172055691	172064699	+
6721	1437159_at	Region	Glb1	12091	9	114364315	114438701	+
6722	1436208_at	Region	Asb1	65247	1	91385187	91386171	+
6723	1425341_at	Region	Kcnk3	16527	5	29047028	29082424	+
6724	1420502_at	Region	Sat1	20229	X	148839287	148842558	-
6725	1447270_at	Region	Rybp	56353	6	100696164	100754244	-
6726	1454649_at	Region	Srd5a1	78925	13	65625480	65642329	-
6727	1415990_at	Region	Vdac2	22334	14	20196229	20210547	+
6728	1455881_at	Region	Ier5l	72500	2	30403700	30406381	-
6729	1423394_at	Region	Pcyox1	66881	6	86820763	86870063	-
6730	1418910_at	Region	Bmp7	12162	2	172330153	172400760	-
6731	1447655_x_at	Region	Sox6	20679	7	109336962	109662307	-
6732	1426437_s_at	Region	Hdac3	15183	18	38160701	38178657	-
6733	1418626_a_at	Region	Clu	12759	14	60496045	60508968	+
6734	1421052_a_at	Region	Sms	20603	X	151043359	151091439	-
6735	1455337_at	Region	9030023J02Rik	320417	16	15187902	15188353	-
6736	1428736_at	Region	9130427A09Rik	107022	18	56619358	56721861	+
6737	1434801_x_at	Region	Slc25a5	11740	X	31416206	31419360	+
6738	1449861_at	Region	Nek4	23955	14	29083439	29119352	+
6739	1416906_at	Region	Anapc5	59008	5	121940636	121974194	-
6740	1433752_s_at	Region	D030016E14Rik	320714	8	46456668	46491560	-
6741	1423612_at	Region	AI462438	98985	2	84420871	84425017	-
6742	1417495_x_at	Region	Cp	12870	3	19298453	19333864	+
6743	1436930_x_at	Region	Hmbs	15288	9	44325203	44333042	-
6744	1439088_at	Region	Pdzk8	107368	19	58893653	58894229	-
6745	1421987_at	Region	Papss2	23972	19	31944210	31991770	+
6746	1446409_at	Region	Gtdc1	227835	2	44496584	44793784	-
6747	1427182_s_at	Region	D18Ertd653e	52662	18	68295003	68486335	+
6748	1456926_at	Region	Rims4	241770	2	163320850	163375652	-
6749	1437014_x_at	Region	LOC545161 /// LOC547275	545161	16	47056509	47057435	+
6750	1435039_a_at	Region	Pip5k1b	18720	3	94547426	94594689	-
6751	1441545_at	Region	9230115F04Rik	320841	4	107501254	107502139	+
6752	1428924_at	Region	Mocs3	69372	2	167739156	167741028	+
6753	1418979_at	Region	9030611N15Rik	105387	13	3943971	3974802	+
6754	1459981_s_at	Region	Rsbn1	229675	3	103355742	103393938	+
6755	1454789_x_at	Region	2610031L17Rik	68879	2	181318349	181372617	+
6756	1423575_a_at	Region	Wbscr22	66138	5	134066451	134078451	-
6757	1438892_at	Region	Qk	19317	17	8850890	8959849	-

6758	1436792_at	Region	BB081391	434204	7	75379417	75404632	+
6759	1423116_at	Region	Dom3z	112403	17	32539831	32542044	+
6760	1417926_at	Region	Luzp5	76044	12	112179035	112226477	+
6761	1438377_x_at	Region	Slc13a3	114644	2	164862028	164929930	-
6762	1458573_at	Region	None	None	NONE	NONE	NONE	NONE
6763	1457118_at	Region	6230417E10Rik	271849	2	125143458	125237240	-
6764	1418694_at	Region	Kcmf1	74287	6	73177405	73198187	-
6765	1460655_a_at	Region	Ubp1	22221	9	113984479	114029010	+
6766	1435586_at	Region	A730042J05Rik	234069	8	12455825	12484040	-
6767	1439932_at	Region	None	None	4	6148142	6148835	+
6768	1417218_at	Region	2810048G17Rik	72691	19	46683539	46689594	-
6769	1424782_at	Region	2610318G18Rik	67171	3	106343709	106369981	+
6770	1455351_at	Region	AU023006	102683	9	95365380	95368889	-
6771	1450199_a_at	Region	Stab1	192187	14	29270851	29300410	-
6772	1445259_at	Region	None	None	2	65537683	65539267	+
6773	1443362_at	Region	Zfp277	246196	12	36913519	37044268	-
6774	1455524_at	Region	2810046L04Rik	212127	3	53098037	53115975	+
6775	1439255_s_at	Region	Tm7sf1	83924	13	12777506	12812482	-
6776	1443524_x_at	Region	Bcl10	12042	3	144898767	144908672	+
6777	1454865_at	Region	Slc9a8	77031	2	166878454	166933731	+
6778	1457015_at	Region	None	None	14	62659605	62659816	+
6779	1448907_at	Region	Thop1	50492	10	81203817	81216225	+
6780	1448336_at	Region	Drg1	13494	11	3144709	3161173	-
6781	1438167_x_at	Region	Flcn	216805	11	59517379	59535402	-
6782	1426126_a_at	Region	MGC30332	101883	7	25978772	25980651	+
6783	1420425_at	Region	Prdm1	12142	10	44551626	44573197	-
6784	1441223_at	Region	38415	381270	1	72728777	72838594	-
6785	1418036_at	Region	Prim2	19076	1	33722760	33938707	-
6786	1416932_at	Region	Cog2	76332	8	123807899	123839080	+
6787	1417739_at	Region	1110030J09Rik	66172	11	70177589	70179385	+
6788	1455391_at	Region	Rad23a	19358	8	84101707	84107702	-
6789	1440926_at	Region	Flt1	14254	5	146453691	146617483	-
6790	1422742_at	Region	Hivep1	110521	13	41684912	41746312	+
6791	1445653_at	Region	Gsk3b	56637	16	36906890	37063968	+
6792	1445134_at	Region	Mkl2	239719	16	12094900	12148700	+
6793	1440038_at	Region	B430007K19Rik	320708	9	43058119	43058802	-
6794	1450874_at	Region	Matr3	17184	18	35795510	35815074	+
6795	1447299_at	Region	None	None	NONE	NONE	NONE	NONE
6796	1433995_s_at	Region	D16Bwg1543e	52910	16	26233287	26235466	+
6797	1456983_at	Region	Trps1	83925	15	50659276	50888038	-
6798	1443258_at	Region	Foxp1	108655	6	99395348	99630926	-
6799	1420089_at	Region	Nfkbia	18035	12	52210017	52212762	-
6800	1423672_at	Region	2510042P03Rik	72421	2	75633612	75636188	-
6801	1430045_at	Region	Tsnax	53424	8	124300320	124321515	+
6802	1454612_at	Region	Rkhd2	240396	18	73807650	73826798	+
6803	1451071_a_at	Region	Atp1a1	11928	3	101002221	101030658	-
6804	1442360_at	Region	None	None	5	130887413	130888910	-
6805	1457009_at	Region	Rhobtb3	73296	13	71926694	72000935	-
6806	1431089_at	Region	Cpsf2	51786	12	97421457	97450986	+
6807	1444569_at	Region	None	None	4	8574109	8574599	-
6808	1424291_at	Region	Nup93	71805	8	93498773	93599238	+
6809	1431173_at	Region	A930008G19Rik	77938	7	127078161	127179580	-
6810	1450423_s_at	Region	Bxdc1	67239	10	40293133	40323010	-
6811	1427901_at	Region	Mrps18c	68735	5	99819412	99825120	+
6812	1437077_at	Region	AW495713	102323	8	12634596	12666770	-
6813	1418856_a_at	Region	Fanca	14087	8	122643200	122693477	-
6814	1420116_s_at	Region	Golph3	66629	15	12132755	12162746	+
6815	1438826_x_at	Region	Calm3	12315	7	13866351	13874927	-
6816	1444549_at	Region	None	None	7	50989210	50989561	-
6817	1433498_at	Region	2010005J08Rik	72046	11	5608972	5635937	-
6818	1422556_at	Region	Gna13	14674	11	109184029	109219306	+
6819	1448818_at	Region	Wnt5a	22418	14	26642805	26662537	+
6820	1422985_at	Region	Fzd1	14362	5	4759844	4763976	-
6821	1455916_at	Region	AI931714	102182	8	76707830	76793401	+
6822	1440126_at	Region	BC037704	269087	19	43221014	43221577	+

6823	1424651_at	Region	BC021611	257633	8	122162467	122199551	+
6824	1450115_at	Region	Gnaq	14682	19	15357188	15609698	+
6825	1449851_at	Region	Per1	18626	11	68824607	68835618	+
6826	1429012_at	Region	Arhgef6	73341	X	51986201	52093443	-
6827	1426246_at	Region	Pros1	19128	16	61841913	61916636	+
6828	1446769_at	Region	2810439F02Rik	72747	18	12831155	12924403	+
6829	1447478_at	Region	Lrp1b	94217	2	40528935	42585760	-
6830	1428433_at	Region	Hipk2	15258	6	38834084	39012133	-
6831	1419730_at	Region	4631427C17Rik	74340	6	29815630	29959406	+
6832	1456528_x_at	Region	None	None	1	86156796	86157492	-
6833	1433962_at	Region	6720458F09Rik	328162	12	107156263	107162678	+
6834	1455688_at	Region	LOC98434	98434	1	169906320	169907120	-
6835	1449988_at	Region	Gimap1	16205	6	48871811	48876559	+
6836	1455755_at	Region	Gm88	224813	17	43763607	43767237	+
6837	1437310_at	Region	Bbs1	52028	19	4675672	4695428	-
6838	1452669_at	Region	2810012G03Rik	66306	18	34982563	34997418	+
6839	1431254_at	Region	4930465M17Rik	74901	8	14398719	14411815	+
6840	1429500_at	Region	Ppm1a	19042	12	69630561	69641441	+
6841	1421985_a_at	Region	Eif4e2	26987	1	87030076	87054566	+
6842	1443356_at	Region	None	None	9	112232392	112232979	-
6843	1422734_a_at	Region	Myb	17863	10	21054488	21090667	-
6844	1424349_a_at	Region	Lpgat1	226856	1	191453775	191513698	+
6845	1423046_s_at	Region	Ncbp2	68092	16	30758010	30767552	+
6846	1447218_at	Region	1110003F02Rik	68466	5	119755631	119756056	+
6847	1418321_at	Region	Dci	13177	17	22230268	22242899	+
6848	1425593_at	Region	Tnpo2	212999	8	84307954	84326395	+
6849	1416624_a_at	Region	Uba52	22186	8	69663643	69665714	-
6850	1433843_at	Region	Hs1bp3	58240	12	7558840	7588145	+
6851	1444004_at	Region	6330441O12Rik	331401	X	36316177	36444623	-
6852	1415673_at	Region	Psph	100678	5	128941432	128963612	-
6853	1441038_at	Region	Utrn	22288	10	12263109	12646998	-
6854	1458450_at	Region	Zfr	22763	15	11948204	11996199	+
6855	1455008_at	Region	6530401C20Rik	231842	5	139722959	139752142	+
6856	1418793_at	Region	Idua	15932	5	107745580	107760769	+
6857	1419140_at	Region	Acvr2b	11481	9	119396762	119427776	+
6858	1453847_at	Region	A930037O16Rik	77971	6	25782552	25783712	-
6859	1427884_at	Region	Col3a1	12825	1	45615394	45652804	+
6860	1451164_a_at	Region	Mrps18b	66973	17	33629538	33635522	-
6861	1436799_at	Region	D230005D02Rik	239188	14	71499296	72063839	+
6862	1423030_at	Region	Vcp	269523	4	42895886	42916356	-
6863	1456200_at	Region	Ipmk	69718	10	71401760	71439983	+
6864	1438900_at	Region	Sacm1l	83493	9	123553586	123616302	+
6865	1419996_s_at	Region	Arnt	11863	3	94936201	94983401	+
6866	1428410_at	Region	Mak3	72117	16	43023139	43046694	+
6867	1449102_at	Region	Ebf2	13592	14	61760384	61959861	+
6868	1417850_at	Region	Rb1	19645	14	67546936	67677175	-
6869	1457713_at	Region	Ercc5	22592	1	44443666	44477079	+
6870	1451159_at	Region	Arhgef12	69632	9	42922812	43064327	-
6871	1424054_at	Region	Btbd2	208198	10	80773518	80779624	-
6872	1424237_at	Region	Zfp639	67778	3	31918770	31928914	+
6873	1424510_at	Region	Nudt6	229228	3	36868582	36883188	-
6874	1439930_at	Region	Smarca2	67155	19	25845226	26018387	+
6875	1419687_at	Region	D930010J01Rik	107227	19	6770655	6911902	+
6876	1426286_at	Region	AF233884	57753	19	38132935	38163004	-
6877	1426009_a_at	Region	Pip5k1b	18720	3	94547426	94594689	-
6878	1440128_s_at	Region	Sez6l	56747	5	111489140	111647147	-
6879	1458414_at	Region	D2Ertd93e	51842	2	167710860	167711582	-
6880	1423932_at	Region	Cugbp1	13046	2	90645207	90721804	+
6881	1432478_a_at	Region	Ibrdc3	75234	4	128085580	128112996	+
6882	1455790_at	Region	E2f2	242705	4	135053651	135075971	+
6883	1446598_at	Region	None	None	11	108004702	108005342	-
6884	1430131_at	Region	Crry	12946	1	194844160	194871942	-
6885	1416355_at	Region	Rbmx	19655	X	52141072	52147740	-
6886	1421528_a_at	Region	Surf5	20933	2	26837429	26842804	-
6887	1443144_at	Region	Prkcb1	18751	7	116167155	116506625	+

6888	1428884_at	Region	Tmem57	66146	4	133763662	133814033	-
6889	1452261_at	Region	Shprh	268281	10	11027612	11087542	+
6890	1453649_at	Region	C030039L03Rik	112415	7	23049710	23081243	+
6891	1425425_a_at	Region	Wif1	24117	10	120617678	120684090	+
6892	1426195_a_at	Region	Cst3	13010	2	148328668	148332404	-
6893	1430529_at	Region	Csnk1a1	93687	18	61784394	61817111	+
6894	1419264_at	Region	1200014M14Rik	67463	13	92579692	92606895	+
6895	1442277_at	Region	Chka	12660	19	3640704	3683157	+
6896	1425024_at	Region	E430018J23Rik	101604	7	121433485	121443350	-
6897	1449205_at	Region	Zfp339	107586	2	143762111	143789016	-
6898	1445484_at	Region	None	None	6	50707092	50707690	-
6899	1452376_at	Region	6230401O10Rik	72667	7	5377063	5384550	+
6900	1451748_a_at	Region	D12Ertd771e	52690	12	103577429	103650252	-
6901	1421826_at	Region	Dll4	54485	2	118839678	118849505	+
6902	1437837_x_at	Region	Poldip3	73826	15	83176653	83200011	-
6903	1418760_at	Region	Rdh11	17252	12	76030739	76048695	-
6904	1416075_at	Region	Sav1	64010	12	66797137	66819163	-
6905	1418129_at	Region	Dhcr24	74754	4	105520055	105545691	+
6906	1424301_at	Region	Zfp219	69890	14	47106560	47120849	-
6907	1449269_at	Region	F5	14067	1	164069551	164140290	+
6908	1427448_at	Region	Rabep1	54189	11	70570583	70666598	+
6909	1452672_at	Region	Fmip	107829	11	4790130	4823652	+
6910	1430077_at	Region	Sfrs11	69207	3	157020040	157032607	-
6911	1446374_at	Region	Arhgef10	234094	8	14307382	14378920	+
6912	1440006_at	Region	BC026600	414070	17	22260152	22260590	+
6913	1452067_at	Region	Asahl	67111	5	91594004	91613926	-
6914	1431033_x_at	Region	Agl	77559	3	115512007	115576987	-
6915	1443355_at	Region	1700081L11Rik	76719	11	104154591	104263380	-
6916	1436481_at	Region	None	None	8	20744957	20745413	-
6917	1442890_at	Region	Socs5	56468	17	84964483	84994389	+
6918	1450984_at	Region	Tjp2	21873	19	23332679	23411932	-
6919	1438066_at	Region	Gtf2b	229906	3	141734221	141752580	+
6920	1459644_at	Region	AU020772	277939	7	94478918	94576823	+
6921	1416020_a_at	Region	Atp5g1	11951	11	95894073	95896691	-
6922	1437688_x_at	Region	Atp6ap2	70495	X	10826811	10855905	+
6923	1416144_a_at	Region	Dhx15	13204	5	50961014	51001194	-
6924	1426705_s_at	Region	Iars	105148	13	49279007	49331120	+
6925	1439265_at	Region	None	None	14	58351896	58352168	+
6926	1457896_at	Region	Esrrg	26381	1	187506691	187723715	+
6927	1437750_at	Region	2310037P21Rik	72309	9	123277458	123278318	-
6928	1445128_at	Region	None	None	9	96967541	96968499	-
6929	1436960_at	Region	Brd3	67382	2	27379952	27419365	-
6930	1453482_at	Region	2310024N18Rik	69570	1	59021861	59022834	+
6931	1418789_at	Region	Sntg2	268534	12	26745849	26946110	-
6932	1453893_at	Region	4933412O06Rik	71071	13	15230943	15251910	-
6933	1444599_at	Region	Herc4	67345	10	63208216	63282585	+
6934	1418087_at	Region	Ufd1l	22230	16	17583376	17606305	+
6935	1419381_at	Region	Terf2ip	57321	8	111309109	111318168	+
6936	1450023_at	Region	Gtpbp1	14904	15	79743329	79772459	+
6937	1459619_at	Region	Epb4.1l2	13822	10	25420347	25491840	+
6938	1454740_at	Region	Mib1	225164	18	10770969	10857561	+
6939	1453166_at	Region	D130073L02Rik	215999	10	59411719	59579123	-
6940	1416264_at	Region	Abcb9	56325	5	123233182	123267116	-
6941	1424642_at	Region	Thoc1	225160	18	10003524	10040828	+
6942	1421117_at	Region	Dst	13518	1	34280938	34577763	+
6943	1429050_at	Region	Chic2	74277	5	73840608	73878810	-
6944	1440621_at	Region	Kitl	17311	10	99997658	100082002	+
6945	1437740_at	Region	Plekhm2	69582	4	140506995	140546160	-
6946	1440636_at	Region	None	None	9	105060109	105060774	+
6947	1451192_a_at	Region	Ttc4	72354	4	105621161	105637787	-
6948	1452903_at	Region	6230427J02Rik	68176	9	108052213	108053866	-
6949	1451057_x_at	Region	Dnm2	13430	9	21313566	21396180	+
6950	1423340_at	Region	Tcfap2b	21419	1	19426696	19452210	+
6951	1429760_at	Region	Rps6ka6	67071	X	105788074	105862607	-
6952	1430302_at	Region	1500041B16Rik	380686	11	16946649	16947898	+

6953	1426707_at	Region	Tubgcp3	259279	8	11992912	12050735	-
6954	1441487_at	Region	Trim2	80890	3	83898180	83960638	-
6955	1453814_at	Region	Bcl11a	14025	11	23972907	24067984	+
6956	1415854_at	Region	Kitl	17311	10	99997658	100082002	+
6957	1438672_at	Region	None	None	15	84362823	84364291	+
6958	1456917_at	Region	Arfgef1	211673	1	10242569	10337781	-
6959	1438658_a_at	Region	Edg3	13610	13	2912	7069	-
6960	1454629_at	Region	A930037G23Rik	320678	6	125801418	125818067	+
6961	1434600_at	Region	Tjp2	21873	19	23332679	23411932	-
6962	1438388_at	Region	None	None	6	9216488	9216775	+
6963	1435466_at	Region	A830091I15Rik	237868	11	78198950	78223362	-
6964	1417088_at	Region	Zfp346	26919	13	53716348	53745853	+
6965	1439215_at	Region	Atp2c1	235574	9	105393471	105477208	-
6966	1452580_a_at	Region	Mrpl21	353242	19	3071741	3081531	+
6967	1428559_at	Region	Fvt1	70750	1	106664678	106700512	-
6968	1455456_a_at	Region	Timm50	66525	7	23703430	23709650	-
6969	1425602_a_at	Region	Rabep2	70314	7	120479171	120495632	+
6970	1424800_at	Region	Enah	13800	1	181864370	181978518	-
6971	1418419_at	Region	4632423N09Rik	103861	11	97903641	97905254	-
6972	1449186_at	Region	Bag4	67384	8	24492266	24510265	-
6973	1450821_at	Region	Pcaf	18519	17	51101486	51203822	+
6974	1435635_at	Region	A030012M09Rik	319263	1	7090301	7142844	+
6975	1428244_at	Region	Larp1	73158	11	57734518	57787663	+
6976	1452266_at	Region	Las1l	76130	X	90536683	90558302	-
6977	1443790_x_at	Region	4930414L22Rik	78108	6	72772296	72772585	+
6978	1438114_x_at	Region	Efs	13644	14	49433145	49442417	-
6979	1448638_at	Region	Mtbp	105837	15	55562483	55630929	+
6980	1427870_x_at	Region	Igh-4	16017	12	108801158	108801309	-
6981	1452944_at	Region	Syngr2	20973	11	117630759	117635374	+
6982	1442309_at	Region	None	None	NONE	NONE	NONE	NONE
6983	1426986_at	Region	2810485I05Rik	72826	9	13664776	13683571	+
6984	1433580_at	Region	Nup54	269113	5	91751291	91770868	-
6985	1427190_at	Region	2900057D21Rik	72993	14	25057193	25108756	-
6986	1426922_s_at	Region	Hrb	15463	1	83168589	83225381	+
6987	1440736_at	Region	AI131651	98858	2	59976509	59977009	-
6988	1451104_a_at	Region	Snrp70	20637	7	39452705	39457003	-
6989	1452701_x_at	Region	Uba52	22186	8	69663643	69665714	-
6990	1433463_at	Region	0610010K06Rik	71678	1	10115	28628	-
6991	1451385_at	Region	2310056P07Rik	70186	16	34825251	34857490	-
6992	1428099_a_at	Region	Sfrs1	110809	11	87775771	87779641	+
6993	1416802_a_at	Region	Cdca5	67849	19	5873887	5880563	+
6994	1420170_at	Region	Myh9	17886	15	77812545	77894062	-
6995	1427548_a_at	Region	Clns1a	12729	7	91742962	91765150	+
6996	1450858_a_at	Region	Ube2d3	66105	3	134321860	134350243	+
6997	1416813_at	Region	Tia1	21841	6	86837265	86854216	+
6998	1448292_at	Region	Uqcr	66594	10	80533820	80537651	-
6999	1435224_at	Region	Crebbp	12914	16	3754292	3755973	-
7000	1435909_at	Region	C030034I22Rik	77533	17	67192735	67218840	-
7001	1427051_at	Region	Tnks1bp1	228140	2	84745922	84770797	+
7002	1423553_at	Region	Dnajb3	15504	1	88023840	88024853	-
7003	1448072_at	Region	None	None	6	125378004	125378711	+
7004	1443084_at	Region	Ctso	229445	3	81661676	81685785	+
7005	1451605_at	Region	Dyx1c1	67685	9	73091351	73105809	+
7006	1423356_at	Region	Snap29	67474	16	16177019	16203795	+
7007	1458586_at	Region	AI849286	269966	7	96277205	96277799	-
7008	1440238_at	Region	None	None	7	21778388	21778923	-
7009	1443963_at	Region	Fmr2	14266	X	64028927	64536633	+
7010	1446793_at	Region	None	None	12	7534887	7535438	+
7011	1443640_at	Region	None	None	NONE	NONE	NONE	NONE
7012	1418810_at	Region	Crhr1	12921	11	103954131	103996611	+
7013	1420819_at	Region	Sla	20491	15	66806690	66825714	-
7014	1418649_at	Region	Egln3	112407	12	50934400	50959266	-
7015	1436605_at	Region	Tkt	21881	14	28683743	28709316	+
7016	1445880_at	Region	C030036D22Rik	77607	NONE	NONE	NONE	NONE
7017	1428290_at	Region	Mipep	70478	14	55319724	55440621	+

7018	1454163_at	Region	5730420D15Rik	70523	10	49278	60632	-
7019	1438508_at	Region	None	None	18	35003585	35003888	+
7020	1416874_a_at	Region	5730511K23Rik	54624	7	23798598	23804984	+
7021	1417643_at	Region	Tsga2	22092	17	29064786	29085171	-
7022	1441971_at	Region	None	None	2	104531780	104532569	-
7023	1434832_at	Region	Foxo3a	56484	10	42292466	42383422	-
7024	1424309_a_at	Region	Mocs2	17434	13	111232567	111243010	+
7025	1455789_x_at	Region	Hspa8 /// LOC432883 /// LOC545316	15481	9	40751958	40756793	+
7026	1435303_at	Region	Taf4b	72504	18	14971506	15086389	+
7027	1453573_at	Region	Hist1h3d	319149	13	23055870	23056280	+
7028	1434768_at	Region	Cln2	12751	7	99859499	99866859	-
7029	1442593_at	Region	None	None	12	37719408	37720082	+
7030	1447300_at	Region	AW551984	244810	9	39530212	39546940	-
7031	1458345_s_at	Region	Colec11	71693	12	25159743	25188570	-
7032	1416738_at	Region	Brap	72399	5	120815393	120837810	+
7033	1424908_at	Region	Mtfmt	69606	9	65558047	65574667	+
7034	1426691_at	Region	Tjp4	74094	17	43767797	43792935	-
7035	1423384_s_at	Region	Tex261	21766	6	84114647	84119990	-
7036	1416188_at	Region	Gm2a	14667	11	54850958	54863739	+
7037	1444058_at	Region	2310047C04Rik	224170	16	47767664	47835780	-
7038	1451641_at	Region	Dbr1	83703	9	99476600	99485145	+
7039	1451754_a_at	Region	Wdr45	54636	X	5961189	5967141	+
7040	1421178_at	Region	9130411I17Rik	67569	10	102288141	102304017	+
7041	1433918_at	Region	Atg4dl	235040	9	21151564	21160633	+
7042	1424290_at	Region	BC010311	209212	4	15924086	15940842	-
7043	1447234_s_at	Region	Snx6	72183	12	0	675	-
7044	1454213_at	Region	4930438O05Rik	78795	1	85961103	86084595	+
7045	1438459_x_at	Region	Sfpq	71514	4	126048448	126057909	+
7046	1424839_a_at	Region	2810405F18Rik	72181	4	114991747	115012781	-
7047	1448020_at	Region	Rap1a	109905	3	105524111	105597165	-
7048	1420307_a_at	Region	Pitpnb	56305	5	110380456	110438052	+
7049	1435282_at	Region	Gm967	381217	19	23211527	23268980	-
7050	1436120_at	Region	None	None	14	53937157	53944677	-
7051	1434611_at	Region	Rnf123	84585	9	108119827	108143707	-
7052	1458167_at	Region	Kcnd2	16508	6	21263145	21776841	+
7053	1440387_at	Region	---	434626	NONE	NONE	NONE	NONE
7054	1444717_at	Region	Zwint	52696	10	72709781	72729831	+
7055	1450959_at	Region	ICRFP703B1614Q5 .6	57373	7	103556608	103565052	+
7056	1458185_at	Region	Thap4	67026	1	93532881	93578284	-
7057	1417647_at	Region	Snx5	69178	2	143707062	143727467	-
7058	1460550_at	Region	Mtmr11	194126	3	95649869	95659546	+
7059	1419132_at	Region	Tlr2	24088	3	83572215	83577710	-
7060	1455780_at	Region	4932418K24Rik	237930	11	95953110	95986538	+
7061	1456584_x_at	Region	LOC224870 /// Phgdh /// LOC269242 /// LOC383450 /// LOC434173 /// LOC546010	224870	17	50730422	50732759	+
7062	1431836_x_at	Region	5430432N15Rik /// AW743872	101899	7	39723120	39728912	+
7063	1429394_at	Region	A130010J15Rik	319266	1	192912406	192915067	+
7064	1430287_s_at	Region	Hemk1	69536	9	107393488	107404076	-
7065	1424183_at	Region	Acat1	110446	9	53644099	53673628	-
7066	1424921_at	Region	Bst2	69550	8	70683434	70686579	-
7067	1417303_at	Region	Mvd	192156	8	121815191	121824977	-
7068	1431428_a_at	Region	Nosip	66394	7	39139035	39153958	+
7069	1416317_a_at	Region	Stambp	70527	6	83887437	83916666	-
7070	1424192_at	Region	1500011H22Rik	68948	5	121516936	121524705	-
7071	1426740_s_at	Region	Armc5	233912	7	122287167	122294910	+
7072	1441229_at	Region	D230019N24Rik	399607	10	63259865	63260423	+
7073	1423409_a_at	Region	2500003M10Rik	66511	3	90309771	90320256	-

7074	1420526_at	Region	Dcbld2	73379	16	57413972	57455222	+
7075	1457034_at	Region	D14Abb1e	218850	14	25576551	25619645	+
7076	1424359_at	Region	Oplah	75475	15	76344528	76355172	-
7077	1441742_at	Region	6430546I09	238271	12	71750845	72033694	-
7078	1417107_at	Region	Tpd52l2	66314	2	181214210	181233714	+
7079	1434599_a_at	Region	Tjp2	21873	19	23332679	23411932	-
7080	1418127_a_at	Region	Pdcd8	26926	X	42995740	43034222	-
7081	1436209_at	Region	Dnajc16	214063	4	140643259	140671905	-
7082	1455671_at	Region	Commd8	27784	5	70938206	70949389	-
7083	1453016_at	Region	2900042B11Rik	72938	4	106212803	106238837	+
7084	1434913_at	Region	Hmgcll1	208982	9	76153103	76274476	+
7085	1434579_x_at	Region	Ndufs8	225887	19	3697661	3701550	-
7086	1441049_at	Region	Kcna6	16494	6	127379948	127411808	-
7087	1459433_at	Region	C130051F05Rik	320348	13	74177838	74178470	+
7088	1456927_at	Region	Mast2	17776	4	115265667	115422753	-
7089	1455434_a_at	Region	Ktn1	16709	14	42751248	42824049	+
7090	1430764_at	Region	1700023F06Rik	69441	11	103019929	103021175	-
7091	1426395_s_at	Region	Eif3s1	78655	2	121561316	121567484	+
7092	1436016_x_at	Region	Gdi3	14569	13	3422528	3450201	+
7093	1421356_at	Region	Tex9	21778	9	72593752	72624808	-
7094	1456514_at	Region	Rbm16	106583	17	3065810	3160145	+
7095	1416793_at	Region	Arl6ip2	56298	17	77665786	77673524	-
7096	1431201_at	Region	Slc25a27	74011	17	41143342	41168531	-
7097	1447908_x_at	Region	Ttc3	22129	16	93778128	93866771	+
7098	1443902_at	Region	6430573F11Rik	319582	8	35293919	35317741	+
7099	1455644_at	Region	3100002B05Rik	68299	11	75771884	75905288	-
7100	1435140_at	Region	Ide	15925	19	36613620	36677821	-
7101	1437378_x_at	Region	Scarb1	20778	5	124438866	124503358	-
7102	1457027_at	Region	Dhtkd1	209692	2	5813387	5860053	-
7103	1435233_at	Region	Ncoa2	17978	1	13272503	13507008	-
7104	1434604_at	Region	Eif5b	226982	1	38328500	38347645	+
7105	1458882_at	Region	Serpinb8	20725	1	107578943	107597781	+
7106	1443934_at	Region	9230110C19Rik	234912	9	8027636	8048787	-
7107	1419374_at	Region	Wbp4	22380	14	73815794	73835161	-
7108	1452889_at	Region	2310007H09Rik	76429	7	126976517	127072441	+
7109	1425746_at	Region	D730039F16Rik	77996	2	34806598	34824295	+
7110	1423959_at	Region	Ropn1l	252967	15	31441819	31454253	-
7111	1440972_at	Region	Nsd1	18193	13	53842763	53849719	+
7112	1460085_at	Region	None	None	1	136481330	136481881	+
7113	1428453_at	Region	4930487N19Rik	70646	14	44261607	44280316	+
7114	1443430_at	Region	Cradd	12905	10	95150586	95300324	-
7115	1458624_at	Region	LOC380843	380843	13	46016804	46028743	+
7116	1452740_at	Region	Myh10	77579	11	68417625	68542200	+
7117	1424269_a_at	Region	Myl6 /// LOC432856 /// LOC433297 /// LOC546126	17904	10	128227888	128230852	-
7118	1446288_at	Region	C78692	97148	4	119825806	119826139	-
7119	1424205_at	Region	Smarca5	93762	8	79911951	79951467	-
7120	1428225_s_at	Region	Hnrpdl	50926	5	99053360	99057516	-
7121	1418876_at	Region	Foxd1	15229	13	94542754	94545191	+
7122	1442066_at	Region	C530028O21Rik	319352	6	125652724	125659098	+
7123	1456998_at	Region	Dbpht2	386753	12	71155249	71156206	+
7124	1456811_at	Region	Cxxc4	319478	3	133121396	133143674	+
7125	1457028_at	Region	A430033K04	243308	5	137594368	137620231	+
7126	1416580_a_at	Region	Stub1	56424	17	23636797	23639522	-
7127	1452748_at	Region	Xrcc3	74335	12	107284533	107295382	-
7128	1433478_at	Region	Psarl /// LOC383654	381038	16	19051435	19073977	-
7129	1428871_at	Region	4121402D02Rik	74026	11	98616603	98627514	+
7130	1423742_at	Region	Rbm10	236732	X	18858936	18889829	+
7131	1452152_at	Region	AI642036	216705	11	45594714	45663462	+
7132	1454938_at	Region	Snx13	217463	12	31759593	31761148	+
7133	1452926_at	Region	Ttc14	67120	3	33243957	33251783	+
7134	1417893_at	Region	Sfxn3	94280	19	44592080	44600788	+

7135	1460728_s_at	Region	Ing4	28019	6	125695852	125705268	+
7136	1435481_at	Region	Zfp653	319601	9	21946165	21962759	-
7137	1458941_at	Region	D130016B08Rik	319524	2	44940594	44941248	-
7138	1449350_at	Region	Osr1	23967	12	8890186	8896599	+
7139	1452451_at	Region	MGI:1929597	65115	8	103464769	103513458	+
7140	1429487_at	Region	Ppp1r12a	17931	10	108049328	108167253	+
7141	1434361_at	Region	E130307J07Rik	235406	9	57030694	57040389	-
7142	1451094_at	Region	Ggtl3	207182	2	154947322	154971764	-
7143	1431175_at	Region	1810019D21Rik	69771	8	105432613	105434567	+
7144	1425531_at	Region	Znhit1	70103	5	135995323	136001004	-
7145	1428296_at	Region	Polr2l	66491	7	135874755	135877129	-
7146	1418573_a_at	Region	Raly	19383	2	154248094	154324181	+
7147	1443707_at	Region	2900046B09Rik	74327	9	36785709	36785967	+
7148	1436861_at	Region	Il7	16196	3	7546649	7587999	-
7149	1421874_a_at	Region	Mrps23	64656	11	87932410	87939499	+
7150	1424465_at	Region	AI413631	381045	16	34857660	34877238	+
7151	1415720_s_at	Region	Mad2l1bp	66591	17	43657310	43663457	-
7152	1441608_at	Region	None	None		67051729	67052352	-
7153	1443836_x_at	Region	Wdr48	67561	9	119904848	119936532	+
7154	1436882_at	Region	Ubl5	66177	9	20514222	20517693	+
7155	1429976_at	Region	Clasp2	76499	9	113865413	113971281	+
7156	1431830_at	Region	4632409L22Rik	67230	7	10799782	10812533	-
7157	1423799_at	Region	Sui1-rs1	20918	11	100141081	100143183	+
7158	1451271_a_at	Region	Acat1	110446	9	53644099	53673628	-
7159	1425108_a_at	Region	BC004728	207818	15	100678898	100694279	-
7160	1429668_at	Region	E130119J07Rik	78006	14	99005077	99007471	-
7161	1444273_at	Region	AW555355	99413	2	90272998	90274123	-
7162	1436057_at	Region	Gtl2	17263	12	105023823	105039917	+
7163	1435931_at	Region	None	None	4	59668259	59669843	+
7164	1452281_at	Region	Sos2	20663	12	66415427	66507784	-
7165	1424121_at	Region	Commd1	17846	11	22794554	22873786	-
7166	1444312_at	Region	4930477M19	242126	3	101284848	101322324	-
7167	1459154_at	Region	Eya3	14050	4	131599945	131685662	+
7168	1447822_x_at	Region	2700038N03Rik	70240	5	136245535	136246530	+
7169	1434307_at	Region	9630015D15Rik	100201	4	15192785	15212331	+
7170	1443981_at	Region	LOC544892	544892	12	105954467	105960875	-
7171	1444349_at	Region	None	None	15	96372348	96373090	+
7172	1452101_at	Region	Blmh	104184	11	76671417	76713035	+
7173	1448875_at	Region	Zhx1	22770	15	58056024	58078371	-
7174	1423891_at	Region	Gstt3	103140	10	75875862	75883154	-
7175	1426804_at	Region	Smarca4	20586	9	21522528	21593154	+
7176	1426366_at	Region	Eif2c2	239528	15	73133858	73173566	-
7177	1456999_at	Region	2510001I10Rik	74781	5	141637713	141677424	+
7178	1450151_at	Region	Zfp316	54201	5	142310603	142328821	-
7179	1434896_at	Region	MGI:3028594	77652	17	30988427	31006091	+
7180	1418436_at	Region	Stx7	53331	10	24123924	24163523	+
7181	1447820_x_at	Region	Cpt2	12896	4	106862887	106882439	-
7182	1428421_a_at	Region	2700085E05Rik	67201	11	75947168	75969314	-
7183	1438681_at	Region	None	None	12	84570510	84571205	+
7184	1419067_a_at	Region	Rabgef1	56715	5	129357005	129384162	+
7185	1452899_at	Region	Rian	75745	X	59165199	59166522	+
7186	1424703_at	Region	Hemk1	69536	9	107393488	107404076	-
7187	1416628_at	Region	0610006I08Rik	66836	19	7967543	7969022	+
7188	1439695_a_at	Region	Mphosph1	240641	19	34265710	34319122	+
7189	1429068_at	Region	2810488G03Rik	72842	6	8482382	8484485	-
7190	1427439_s_at	Region	Skb1	27374	14	49025095	49035051	-
7191	1451678_at	Region	4430402O11Rik	67608	11	121058346	121076948	+
7192	1458060_at	Region	None	None	4	133702458	133703156	-
7193	1423239_at	Region	Impdh1	23917	6	29247537	29259368	-
7194	1438843_x_at	Region	Mtch2	56428	2	90551904	90571383	+
7195	1451496_at	Region	Mtss1	211401	15	58954404	59092928	-
7196	1451272_a_at	Region	2510010F15Rik	67921	1	91073608	91108966	+
7197	1449878_a_at	Region	Slc12a6	107723	2	111890057	111986394	+
7198	1417008_at	Region	Crat	12908	2	30334148	30347622	-
7199	1439349_at	Region	Stno	216161	10	80180244	80225913	-

7200	1442411_at	Region	Glcci1	170772	6	8234651	8573400	+
7201	1418593_at	Region	Taf6	21343	5	137151434	137157311	-
7202	1425523_at	Region	2600011C06Rik	67039	12	80506670	80547102	+
7203	1440701_at	Region	None	None	5	75947446	75948048	+
7204	1434104_at	Region	6030458H05	270066	8	71632032	71642844	-
7205	1424872_at	Region	2310001H12Rik	69504	5	109003198	109009422	+
7206	1453134_at	Region	Pik3ca	18706	3	31844384	31871009	+
7207	1438546_x_at	Region	Slc25a5 /// LOC383528 /// LOC433326 /// LOC433923 /// LOC545025	11740	X	31416206	31419360	+
7208	1418873_at	Region	Sfxn4	94281	19	60439150	60461769	-
7209	1438922_x_at	Region	Slc25a5 /// LOC433326	11740	X	31416206	31419360	+
7210	1437458_x_at	Region	Clu	12759	14	60496045	60508968	+
7211	1417187_at	Region	Hip2	53323	5	64339100	64400757	+
7212	1447947_at	Region	Zfyve16	218441	13	88655606	88698984	-
7213	1426429_at	Region	Thap7	69009	16	16298950	16302020	-
7214	1456668_at	Region	Zcchc11	230594	4	107418943	107518318	+
7215	1450112_a_at	Region	Gas2	14453	7	46048672	46163985	+
7216	1445933_at	Region	None	None	14	16847716	16848416	-
7217	1456761_at	Region	D630030B22Rik	53963	NONE	NONE	NONE	NONE
7218	1439958_at	Region	LOC546257	546257	12	84514431	84514898	-
7219	1455049_at	Region	Igsf3	78908	3	100804224	100886780	+
7220	1427124_at	Region	Stk35	67333	2	129314605	129341840	+
7221	1451834_at	Region	Cacnb1	12295	11	97823988	97904149	-
7222	1426532_at	Region	Zmynd11	66505	13	9669934	9749340	-
7223	1448083_at	Region	A930012M17	268777	14	117843742	117864796	-
7224	1444085_at	Region	5430420P03Rik	71365	10	43327846	43546962	+
7225	1443746_x_at	Region	Dmp1	13406	5	103238499	103249944	+
7226	1424460_s_at	Region	BC005662	210992	13	69524486	69573696	+
7227	1438005_at	Region	B230315F11Rik	319653	5	8429484	8466624	+
7228	1455673_at	Region	AU016977	231642	5	113234577	113238864	-
7229	1455542_at	Region	C630043F03Rik	68285	4	71292437	71294029	+
7230	1455035_s_at	Region	Nol5a	67134	2	129788312	129793166	+
7231	1455032_at	Region	9630037P07Rik	227210	1	64989291	65019720	+
7232	1437862_at	Region	2600011C06Rik	67039	12	80506670	80547102	+
7233	1428464_at	Region	Ndufa3	66091	7	1492	4280	+
7234	1448263_a_at	Region	Cndp2	66054	18	84835201	84853374	-
7235	1426330_at	Region	Cacng5	140723	11	107695694	107732451	-
7236	1420857_at	Region	Lancl2	71835	6	57865048	57900894	+
7237	1440446_at	Region	Nkd1	93960	8	87806676	87877632	+
7238	1441510_at	Region	Rhof	23912	5	122256437	122285538	-
7239	1425009_at	Region	Tcte2	21646	17	11774717	11823520	-
7240	1428389_s_at	Region	Wdr43	72515	17	69392451	69435728	+
7241	1428110_x_at	Region	Vps11	71732	9	44336960	44350525	-
7242	1432435_s_at	Region	C030004A17Rik	109229	9	35131009	35181307	-
7243	1428905_at	Region	Rraga	68441	4	85561878	85563477	+
7244	1423811_at	Region	Sf3a3	75062	4	123741889	123759573	+
7245	1441327_a_at	Region	Ssr1	107513	13	37516882	37533029	-
7246	1438991_x_at	Region	Ppp2r1a	51792	17	18817271	18837722	+
7247	1419125_at	Region	Ptpn18	19253	1	34729261	34743287	+
7248	1459920_at	Region	None	None	12	29986273	29987153	+
7249	1435480_at	Region	Braf	109880	6	39749542	39860924	-
7250	1452869_at	Region	Prpf38b	66921	3	108698549	108707446	-
7251	1416635_at	Region	Smpdl3a	57319	10	58060696	58077885	+
7252	1438580_at	Region	4930572I07Rik	319885	4	44672569	44825209	+
7253	1436464_at	Region	None	None	1	189885431	189932179	+
7254	1426925_at	Region	Mnab	319817	2	37306963	37355065	-
7255	1416323_at	Region	2410004N11Rik	66989	17	26754605	26771684	+
7256	1456712_at	Region	A830039H10Rik	209707	5	44458346	44613918	-
7257	1417368_s_at	Region	Ndufa2	17991	18	36966131	36968313	-
7258	1421005_at	Region	Cep1	26920	2	35064394	35110984	+
7259	1424116_x_at	Region	Ppp5c	19060	7	13955563	13978781	-

7260	1441960_x_at	Region	5730494M16Rik	66648	18	25367107	25397117	-
7261	1426828_at	Region	1300018I17Rik	72325	8	122617239	122629245	-
7262	1416623_at	Region	Thbs3	21827	3	88971680	88983291	+
7263	1443349_at	Region	Dock10	210293	1	80833085	80997044	-
7264	1418229_s_at	Region	Hirip5	56748	6	87443967	87462532	+
7265	1417457_at	Region	Cks2	66197	13	50199009	50204338	+
7266	1446880_at	Region	None	None	10	8813595	8814186	-
7267	1426272_at	Region	Lmbr1	56873	5	27683542	27828674	-
7268	1436761_s_at	Region	1200015N20Rik	71721	10	70495074	70612698	+
7269	1418591_at	Region	Dnaja4	58233	9	54817109	54833863	+
7270	1451177_at	Region	Dnajb4	67035	3	151165498	151191485	-
7271	1457261_at	Region	A930025H08Rik	77807	13	27889490	27890232	-
7272	1434708_at	Region	Vhlh	22346	6	114191852	114199465	+
7273	1433515_s_at	Region	Etnk1	75320	6	144013618	144053017	+
7274	1427321_s_at	Region	Cxadr	13052	16	77387226	77445217	+
7275	1445395_at	Region	AI875142	103885	11	108092142	108092743	-
7276	1448031_at	Region	AW050213	102284	8	93243919	93244864	+
7277	1452933_at	Region	Ankrd39	109346	1	36832764	36841792	-
7278	1439689_at	Region	9030224M15Rik	327747	10	40761991	40889750	+
7279	1442934_at	Region	C81608	97314	6	91238881	91239799	-
7280	1417535_at	Region	Fbxo25	66822	8	13286421	13319136	+
7281	1418526_at	Region	Fusip1	14105	4	134737416	134751165	+
7282	1443705_at	Region	D14Ertd171e	238988	14	25761526	26615037	+
7283	1425466_at	Region	Senp2	75826	16	20782192	20820660	+
7284	1417941_at	Region	Hdhd4	67311	2	150486633	150496291	-
7285	1424608_a_at	Region	LOC432458 /// LOC432468 /// LOC432469 /// LOC432527 /// LOC432783 /// LOC432784 /// LOC432804 /// LOC432823 /// LOC432857 /// LOC432890 /// LOC432932 /// LOC433038 /// LOC433041 /// LOC433042 /// LOC433154 /// LOC433156 /// LOC433244 /// LOC433285 /// LOC	432458	10	47488094	47490071	-
7286	1442399_at	Region	Xrn1	24127	9	95858413	95956167	+
7287	1422254_a_at	Region	Dyrk1b	13549	7	23575246	23582675	+
7288	1440218_at	Region	BC040758	268663	13	53328471	53347805	+
7289	1439092_at	Region	Nxf1	53319	19	7953650	7967457	+
7290	1452146_a_at	Region	Cox15	226139	19	43279531	43297387	-
7291	1424195_a_at	Region	Inpp5d	16331	1	87436767	87537073	+
7292	1445766_at	Region	Ppfia2	327814	10	106357920	106820120	+
7293	1438623_x_at	Region	Rbx1	56438	15	81517173	81527164	+
7294	1438191_a_at	Region	Rnf40	233900	7	121638515	121653317	+
7295	1451518_at	Region	BC021921	236193	8	2346	7667	+
7296	1422119_at	Region	Rab5b	19344	10	128414967	128433354	-
7297	1427240_at	Region	Dock6	67539	9	21693020	21737604	-
7298	1455395_at	Region	4933425A18Rik	71147	14	14735221	14746504	-
7299	1451076_s_at	Region	Ga17	98221	2	104704410	104721781	-
7300	1455943_at	Region	Zfp451	98403	1	34030702	34083136	-
7301	1448345_at	Region	Tomm34	67145	2	163510510	163528071	-
7302	1422736_at	Region	Ranbp9	56705	13	42965819	43043413	-
7303	1421965_s_at	Region	Notch3	18131	17	29928825	29975691	-
7304	1416911_a_at	Region	6330407G11Rik	68050	4	122762469	122777457	-
7305	1454051_at	Region	2600011E07Rik	72125	14	54913220	54915871	+
7306	1446272_at	Region	Pctk2	237459	10	93180223	93239410	+

7307	1428955_x_at	Region	Slc9a3r2	65962	17	22442873	22453949	-
7308	1424333_at	Region	Rg9mtd1	52575	16	54929659	54933715	-
7309	1421611_at	Region	AB041806	58213	4	137276459	137278961	+
7310	1417749_a_at	Region	Tjp1	21872	7	59180206	59255462	-
7311	1438602_s_at	Region	Masp1	17174	16	22233196	22304246	-
7312	1454464_at	Region	2700078F05Rik	72626	1	181910788	181912233	+
7313	1453334_at	Region	B230216N24Rik	78603	1	97811078	97827715	-
7314	1450704_at	Region	Ihh	16147	1	75249527	75255860	-
7315	1433747_at	Region	2010309L07Rik	66485	17	15385299	15387122	-
7316	1436086_at	Region	4921526F01Rik	385668	16	95596843	95628432	-
7317	1431450_at	Region	4930444P10Rik	75799	1	16244204	16276731	-
7318	1434142_at	Region	Ppm1d	53892	11	85039246	85075058	+
7319	1442745_x_at	Region	C79248	96982	2	155626396	155626710	-
7320	1417792_at	Region	Zfml	18139	6	84261646	84334988	+
7321	1446297_at	Region	Hibadh	58875	6	52690560	52784625	-
7322	1423380_s_at	Region	Nfatc4	73181	14	50343024	50351959	+
7323	1442258_at	Region	None	None	8	85949367	85950034	+
7324	1427496_at	Region	AI851464	99100	2	125076943	125138966	-
7325	1440471_x_at	Region	Waspip	215280	2	73127366	73227159	-
7326	1441988_at	Region	Ppm1k	243382	6	57667241	57693162	-
7327	1433742_at	Region	Ankrd15	107351	19	24649338	24674526	+
7328	1459847_x_at	Region	None	None	14	65295090	65295338	+
7329	1442821_at	Region	Smbp	107358	19	40758164	40808343	-
7330	1436722_a_at	Region	Actb	11461	5	141967352	141970960	-
7331	1438768_at	Region	None	None	1	83284292	83285120	-
7332	1416170_at	Region	Trap1	68015	16	3711543	3749386	-
7333	1433104_at	Region	5730412F04Rik	70483	9	35471945	35473316	+
7334	1437379_x_at	Region	Trap1	68015	16	3711543	3749386	-
7335	1434420_x_at	Region	Tomm22	223696	15	79723188	79725182	+
7336	1444178_at	Region	LOC433237	433237	19	28317234	28322100	+
7337	1427397_at	Region	2810046L04Rik	212127	3	53098037	53115975	+
7338	1418159_at	Region	Tcfcp2	21422	15	100560334	100609485	-
7339	1419815_at	Region	D14Ertd209e	52535	14	46985534	46986284	+
7340	1447869_x_at	Region	Rhobtb3	73296	13	71926694	72000935	-
7341	1423071_x_at	Region	6720475J19Rik	68157	NONE	NONE	NONE	NONE
7342	1448351_at	Region	Coro1b	23789	19	3937450	3942825	+
7343	1448581_at	Region	Zfp346	26919	13	53716348	53745853	+
7344	1453243_at	Region	0610030E20Rik	68364	6	72679317	72685122	+
7345	1452804_at	Region	1700010A17Rik	75495	2	35981635	36011871	+
7346	1428661_at	Region	Nfkbil2	72749	15	76677119	76690793	-
7347	1435504_at	Region	Rsnl2	78785	17	69558453	69633262	+
7348	1418648_at	Region	Egln3	112407	12	50934400	50959266	-
7349	1423253_at	Region	Mpz	17528	1	171079553	171089962	+
7350	1452399_at	Region	Rgs6	50779	12	79477666	80021017	+
7351	1424751_at	Region	Abt1	30946	13	22898425	22903930	-
7352	1436032_at	Region	None	None	1	181195800	181196168	+
7353	1416195_at	Region	MGI:1194899	19062	11	75356678	75374523	+
7354	1440531_at	Region	Rbm11	224344	16	74674504	74683522	+
7355	1440935_at	Region	Grb10	14783	11	11828255	11865408	-
7356	1438205_at	Region	1110014N23Rik	68505	19	5858871	5865993	-
7357	1416461_at	Region	Gpiap1	53872	2	103469773	103502351	-
7358	1443899_at	Region	BC028799	213473	9	87536273	87546897	-
7359	1416934_at	Region	Mtm1	17772	X	65883573	65983756	+
7360	1453119_at	Region	Otud1	71198	2	19697585	19889760	+
7361	1456865_x_at	Region	Rrs1	59014	1	9650211	9652221	+
7362	1441918_x_at	Region	LOC435350	435350	13	33364069	33372760	-
7363	1424145_at	Region	Prr3	75210	17	33691562	33699257	-
7364	1426703_at	Region	Gak	231580	5	107645627	107705954	-
7365	1435097_at	Region	Mmab	77697	5	113541603	113554596	-
7366	1418381_at	Region	Zfp148	22661	16	32188918	32308456	+
7367	1426384_a_at	Region	Ywhae	22627	11	75458597	75491496	+
7368	1448770_a_at	Region	Atpif1	11983	4	131491458	131494544	-
7369	1429446_at	Region	Sdccag1	66244	12	66143332	66188963	-
7370	1423087_a_at	Region	1110002E23Rik	66119	17	45205200	45206846	-
7371	1417670_at	Region	Timm44	21856	8	3614696	3630853	-

7372	1450212_at	Region	Fmnl1	57778	11	102992486	103019315	+
7373	1454955_at	Region	Ipo7	233726	7	103871296	103907985	+
7374	1423807_a_at	Region	Calm2	12314	17	85290221	85303654	-
7375	1420652_at	Region	Ate1	11907	7	124514765	124642811	-
7376	1437146_x_at	Region	Coro7	78885	16	4297661	4351229	-
7377	1449451_at	Region	Serpinb11	66957	1	107351186	107369347	+
7378	1445283_at	Region	None	None	2	96275041	96275719	+
7379	1454909_at	Region	Tacc1	320165	8	23877518	23925420	-
7380	1418361_at	Region	Gas8	104346	8	122891361	122910698	+
7381	1445323_at	Region	MGI:2149712	268527	12	16037980	16123596	-
7382	1418724_at	Region	Cfi	12630	3	128769608	128808203	+
7383	1444177_at	Region	None	None	1	153298267	153298859	-
7384	1434126_at	Region	4930402H24Rik	228602	2	130221684	130353963	-
7385	1435747_at	Region	Fgf14	14169	14	118506813	118721824	-
7386	1418344_at	Region	Tmem8	60455	17	23906487	23916424	+
7387	1425552_at	Region	Hip1r	29816	5	123144623	123174525	+
7388	1441813_at	Region	None	None	5	96014299	96014531	-
7389	1452940_x_at	Region	Pitpnc1	71795	11	107033195	107291788	-
7390	1444328_at	Region	Clta	12757	4	43928606	43948768	+
7391	1459302_at	Region	A030007N12Rik	109237	15	102138906	102139535	+
7392	1447114_x_at	Region	4930414L22Rik	78108	6	72772131	72772585	+
7393	1419081_at	Region	Atg10l	66795	13	87068126	87173332	-
7394	1424609_a_at	Region	BC003993 /// LOC432468 /// LOC432512 /// LOC432823 /// LOC432857 /// LOC432890 /// LOC432924 /// LOC432932 /// LOC433043 /// LOC433244 /// LOC433340 /// LOC433373 /// LOC433596 /// LOC433657 /// LOC433893 /// LOC433915 /// LOC434014 /// LOC434071 /// LOC4	432468	10	66312437	66314332	+
7395	1424913_at	Region	2310044G17Rik	217732	12	83821045	83837587	+
7396	1449013_at	Region	Eef2k	13631	7	114718450	114779568	+
7397	1431806_at	Region	4931408D14Rik	77059	19	36601644	36607149	-
7398	1435194_at	Region	Hspa4	15525	11	53014415	53053230	-
7399	1458711_at	Region	None	None	15	101291534	101292103	+
7400	1456828_at	Region	C030023E24Rik	320247	X	55862069	55862762	+
7401	1436582_at	Region	None	None	X	99138334	99139054	-
7402	1434044_at	Region	Repin1	58887	6	48726819	48731963	+
7403	1454697_at	Region	Tloc1	69276	3	30204876	30231459	+
7404	1426254_at	Region	Tm2d1	94043	4	97330443	97358338	-
7405	1446507_at	Region	None	None	4	41591437	41591913	-
7406	1416522_a_at	Region	Grcc10	14790	6	125394709	125396685	-
7407	1421107_at	Region	Stk4	58231	2	163531147	163612490	+
7408	1450172_at	Region	Pknox1	18771	17	29391575	29415568	+
7409	1446294_at	Region	Tcf12	21406	9	71980605	72246476	-
7410	1439516_at	Region	2610201A13Rik	70434	11	33045296	33045960	-
7411	1420744_at	Region	Chrnb2	11444	3	89559631	89570768	-
7412	1452901_at	Region	3526402H21Rik	66683	1	64893251	64897055	+
7413	1429248_at	Region	2810002N01Rik	68020	12	107191903	107212849	+
7414	1423766_at	Region	Pak1ip1	68083	13	40561850	40573601	+
7415	1424211_at	Region	5730438N18Rik	70556	4	148236837	148266549	-
7416	1420705_at	Region	Foxb1	64290	9	69888791	69891882	-
7417	1443770_x_at	Region	None	None	5	130720592	130720893	-
7418	1425424_at	Region	None	None	6	141243512	141245799	+

7419	1441738_at	Region	None	None	NONE	NONE	NONE	NONE
7420	1438753_at	Region	Porcn	53627	X	6432790	6445447	-
7421	1445089_at	Region	D16Ertd778e	52714	NONE	NONE	NONE	NONE
7422	1424003_at	Region	4930444A02Rik	74653	8	24705353	24718870	-
7423	1441768_at	Region	9430051O21Rik	77339	4	79917608	79917958	+
7424	1447228_at	Region	Zfp289	77038	2	90970055	90981680	+
7425	1422518_at	Region	Cask	12361	X	11759987	12085496	-
7426	1435801_at	Region	Fcmd	246179	4	53635886	53679150	+
7427	1427639_a_at	Region	Nek4	23955	14	29083439	29119352	+
7428	1446850_at	Region	Ppap2b	67916	4	104116265	104190062	+
7429	1436232_a_at	Region	Gabpb1	14391	2	126142766	126189340	-
7430	1429156_at	Region	2610036L11Rik	66311	10	30232476	30408425	-
7431	1454233_at	Region	2310006M14Rik	76422	1	136028546	136054934	-
7432	1428019_at	Region	4932425I24Rik	320214	16	37114992	37159933	-
7433	1416177_at	Region	Rbmxrt	19656	14	40378978	40380600	+
7434	1450189_at	Region	Xkh	22439	X	7511854	7550113	+
7435	1422575_at	Region	Mxd4	17122	5	32664918	32676048	-
7436	1426913_at	Region	Lss	16987	10	76632617	76654924	+
7437	1430791_at	Region	6330415B21Rik	70753	6	77730464	77732574	-
7438	1426576_at	Region	Tmem23	208449	19	31448202	31573509	-
7439	1448929_at	Region	F13a1	74145	13	36407592	36590165	-
7440	1423315_at	Region	Bbc3	170770	7	1582	5761	+
7441	1447962_at	Region	None	None	15	36798410	36799070	+
7442	1439901_at	Region	2610208M17Rik	108909	8	3442132	3444816	+
7443	1453980_at	Region	5330434G04Rik	78269	X	99990916	99993138	-
7444	1456021_at	Region	Atf6	226641	1	170641954	170802508	-
7445	1454625_at	Region	Phf6	70998	X	47433062	47477739	+
7446	1431222_at	Region	Tiam2	24001	17	3358883	3481083	+
7447	1443302_at	Region	6720403M19Rik	320619	6	146699973	146700622	+
7448	1421646_a_at	Region	Pias3	229615	3	96184305	96193798	+
7449	1438972_x_at	Region	2810410L24Rik	76377	11	120006436	120007769	+
7450	1418534_at	Region	Fzd2	57265	11	102425622	102427671	+
7451	1430151_at	Region	Nisch	64652	14	29302768	29323388	-
7452	1434901_at	Region	Gm1103 /// LOC544682	381990	10	125154233	125161026	+
7453	1428542_at	Region	2010003J03Rik	69860	19	5155603	5160301	+
7454	1446080_at	Region	Cutl2	13048	5	121012566	121200175	-
7455	1442237_at	Region	Sqstm1	18412	11	49952993	49963631	-
7456	1436478_at	Region	1810013L24Rik	69053	16	8502119	8528217	+
7457	1436241_s_at	Region	Hira	15260	16	17647800	17741359	+
7458	1424366_at	Region	Tmem15	227697	2	30216424	30218241	-
7459	1430526_a_at	Region	Smarca2	67155	19	25845226	26018387	+
7460	1438056_x_at	Region	Abcc5	27416	16	19102921	19197967	-
7461	1416163_at	Region	Cops4	26891	5	99537083	99566415	+
7462	1450413_at	Region	Pdgfb	18591	15	80048194	80067128	-
7463	1438789_s_at	Region	Dpysl3	22240	18	43544269	43613318	-
7464	1423653_at	Region	Atp1a1	11928	3	101002221	101030658	-
7465	1448705_at	Region	Zfp297	81630	17	31620251	31623277	+
7466	1458738_at	Region	Gna14	14675	19	15659862	15834920	+
7467	1455103_at	Region	Ddx46	212880	13	54296789	54297817	+
7468	1429521_at	Region	4930562C03Rik	67667	9	3286376	3289860	+
7469	1421855_at	Region	Fgl2	14190	5	19824807	19830476	+
7470	1418013_at	Region	Cml1	66116	6	86339786	86345291	-
7471	1424928_at	Region	LOC545983	545983	7	92685157	92692326	-
7472	1443157_at	Region	None	None	19	27872963	27873268	-
7473	1438583_at	Region	Ern1	78943	11	106218709	106308885	-
7474	1433754_at	Region	Mbnl2	105559	14	114846670	115003029	+
7475	1447995_x_at	Region	1700026B20Rik	69434	12	100474801	100476412	-
7476	1452321_at	Region	Brwd1	93871	16	95429851	95519830	-
7477	1425497_a_at	Region	Prpf4b	19134	13	34407672	34435208	+
7478	1456825_at	Region	Wdr33	74320	18	32067429	32147139	+
7479	1460129_at	Region	Slc6a2	20538	8	92248264	92288701	+
7480	1449176_a_at	Region	Dck	13178	5	88030861	88049111	+
7481	1420823_at	Region	Sema4d	20354	13	50254932	50347328	-
7482	1453209_at	Region	Brf1	72308	12	108436225	108439583	-

7483	1449785_at	Region	AA414993	103569	11	59561235	59561500	-
7484	1445492_at	Region	Mapre2	212307	18	24032480	24122349	+
7485	1459360_at	Region	Sp3	20687	2	72634943	72710690	-
7486	1450612_a_at	Region	Pemt	18618	11	59696259	59762481	-
7487	1438242_at	Region	Usp40	227334	1	87765405	87827652	-
7488	1453282_at	Region	Cxadr	13052	16	77387226	77445217	+
7489	1434311_at	Region	Cnot6l	231464	5	95087917	95179605	-
7490	1454028_at	Region	4931402H11Rik	70963	3	87971300	87976201	-
7491	1436746_at	Region	Prkwnk1	232341	6	120356763	120470204	-
7492	1431712_a_at	Region	2310022A10Rik	66367	7	22928910	22956812	+
7493	1420110_s_at	Region	Zfp334	228876	2	164834169	164844992	-
7494	1455685_at	Region	9530064J02	330631	7	106205812	106208818	+
7495	1416574_at	Region	5730589K01Rik	268741	14	47379749	47394617	+
7496	1452517_at	Region	Plekhh1	211945	12	75867965	75938050	+
7497	1428483_a_at	Region	None	None	16	89876353	89884308	-
7498	1454746_at	Region	Plekhm1	353047	11	103186181	103233753	-
7499	1429086_at	Region	Tcfcp2l3	252973	15	37236160	37368500	+
7500	1437838_x_at	Region	Grsf1	231413	5	87925715	87942166	-
7501	1431774_a_at	Region	4930404J24Rik	73919	7	113771859	113792317	+
7502	1436085_at	Region	Zbtb34	241311	2	33338273	33363486	-
7503	1424503_at	Region	Rab22a	19334	2	173120334	173165145	+
7504	1433807_at	Region	6720463M24Rik	77744	14	93570533	93598212	+
7505	1455886_at	Region	Cbl	12402	9	44140278	44222839	-
7506	1427008_at	Region	4732452J19Rik	207742	11	87427889	87463530	+
7507	1460436_at	Region	Ndst1	15531	18	60911289	60938715	-
7508	1443624_at	Region	BC013481	245945	5	2381	11024	+
7509	1434843_at	Region	A430041B07Rik	328108	12	61743753	61801012	+
7510	1439539_at	Region	Tram2	170829	1	21226321	21304168	-
7511	1423458_at	Region	Slc35a5	74102	16	44022615	44041681	-
7512	1426089_a_at	Region	BC003331	226499	1	150199242	150227115	-
7513	1428927_at	Region	Shq1	72171	6	101047039	101145097	-
7514	1424027_at	Region	Pxn	19303	5	114657142	114667534	+
7515	1446383_at	Region	Efna5	13640	17	60297708	60574961	-
7516	1438383_x_at	Region	Ppp2r1a	51792	17	18817271	18837722	+
7517	1434300_at	Region	2610101N10Rik	67958	9	95369771	95420790	-
7518	1443745_s_at	Region	Dmp1	13406	5	103238499	103249944	+
7519	1418660_at	Region	Clock	12753	5	75457416	75549787	-
7520	1450966_at	Region	Crot	74114	5	8972791	9003889	-
7521	1447568_at	Region	None	None	5	114446249	114446563	+
7522	1424110_a_at	Region	Nme1	18102	11	93780012	93789608	-
7523	1434128_a_at	Region	Zfp574	232976	7	20255177	20260374	+
7524	1428136_at	Region	Sfrp1	20377	8	22166110	22202536	+
7525	1420635_a_at	Region	Tcirg1	27060	19	3684843	3694020	-
7526	1449403_at	Region	Pde9a	18585	17	29194101	29286091	+
7527	1458398_at	Region	1700027F06Rik	72268	6	124975959	124976196	-
7528	1458459_a_at	Region	AI452102	330594	7	84020274	84040181	+
7529	1442603_at	Region	Pb1	76748	14	29191861	29192436	+
7530	1442670_at	Region	6430517E21Rik	240843	1	158150731	158260041	-
7531	1440168_x_at	Region	Kctd7	212919	5	129314713	129325633	+
7532	1453147_at	Region	Polr3e	26939	7	114793384	114821816	+
7533	1428067_at	Region	Rasl12	70784	9	65520753	65534971	+
7534	1429197_s_at	Region	Rabgap1l	29809	1	160161852	160697585	-
7535	1448930_at	Region	3010026O09Rik	68067	11	49927692	49952950	+
7536	1420813_at	Region	Hdac7a	56233	15	97868326	97887406	-
7537	1435864_a_at	Region	1810063B05Rik	67892	8	125710414	125713320	+
7538	1454086_a_at	Region	Lmo2	16909	2	103674977	103686628	+
7539	1450013_at	Region	2900073G15Rik	67268	17	68765368	68773934	-
7540	1453350_at	Region	2010005J08Rik	72046	11	5608972	5635937	-
7541	1418428_at	Region	Kif5b	16573	18	6245790	6284051	-
7542	1422558_at	Region	Gamt	14431	10	80383108	80385921	-
7543	1419212_at	Region	Icosl	50723	10	78178359	78188516	+
7544	1443518_at	Region	Sgce	20392	6	4627235	4699984	-
7545	1445101_at	Region	None	None	10	67203600	67204298	+
7546	1432007_s_at	Region	Ap2a2	11772	7	135965073	136035847	+
7547	1455086_at	Region	Uchl5	56207	1	143609398	143638878	+

7548	1453796_a_at	Region	1200009B18Rik	67456	6	149113867	149145859	-
7549	1445866_at	Region	Mast4	328329	13	98930827	99546711	-
7550	1451082_at	Region	1300018I05Rik /// LOC445272	445272	17	27470394	27513279	+
7551	1427113_s_at	Region	2700049H19Rik	109008	2	128607662	128610132	+
7552	1434715_at	Region	1600014C10Rik	72244	7	33351336	33365081	+
7553	1447471_at	Region	None	None	NONE	NONE	NONE	NONE
7554	1449413_at	Region	Mpv17l	93734	16	12676558	12685220	+
7555	1429585_s_at	Region	Mynn	80732	3	30012186	30028739	+
7556	1436734_at	Region	E130309F12Rik	272031	4	48975384	49256135	+
7557	1423797_at	Region	Aacs	78894	5	124642786	124684316	+
7558	1460464_at	Region	2700089E24Rik	381820	6	133875346	133882217	+
7559	1437797_at	Region	D5Wsu150e	28023	5	121649873	121650200	-
7560	1437115_at	Region	AA673488	101471	7	135633236	135664738	+
7561	1424055_at	Region	Ncoa5	228869	2	164457090	164491512	-
7562	1423505_at	Region	Tagln	21345	9	45942880	45949150	-
7563	1438783_at	Region	Tmepai	65112	2	172688256	172737000	-
7564	1421894_a_at	Region	Tpp2	22019	1	44229826	44298790	+
7565	1439251_at	Region	Idua	15932	5	107745580	107760769	+
7566	1449886_a_at	Region	Timm10	30056	12	67958182	67971658	-
7567	1438438_at	Region	2900002H16Rik	75695	5	123664423	123702734	-
7568	1433209_at	Region	2210017G18Rik	70386	6	4872511	4873706	+
7569	1429351_at	Region	4930429H24Rik	75785	16	18869169	18899359	+
7570	1437644_at	Region	B3galt2	26878	1	143472806	143481893	+
7571	1457306_at	Region	Pole3	59001	4	61614989	61616205	-
7572	1451007_at	Region	Cnnm2	94219	19	46312725	46429008	+
7573	1432410_a_at	Region	Bmp7	12162	2	172330153	172400760	-
7574	1459409_at	Region	D130073L02Rik	215999	10	59411719	59579123	-
7575	1425814_a_at	Region	Calcrl	54598	2	84028375	84074736	-
7576	1434823_x_at	Region	1110002M09Rik	66915	1	92464648	92469431	-
7577	1441652_at	Region	Bmpr2	12168	1	60068175	60174399	+
7578	1430557_at	Region	4930434J08Rik	73979	11	20636557	20638179	+
7579	1422701_at	Region	Zap70	22637	1	37056452	37077407	+
7580	1426764_at	Region	Oaz2	18247	9	65798855	65812562	+
7581	1425439_a_at	Region	Slc41a3	71699	6	91044196	91085644	+
7582	1434561_at	Region	Asxl1	228790	2	152803270	152859009	+
7583	1446141_at	Region	Tgfb2	21808	1	186121756	186210661	-
7584	1424663_at	Region	BC017647	216971	11	77841285	77881745	+
7585	1424661_at	Region	Pmpca	66865	2	26321510	26329283	+
7586	1425304_s_at	Region	Prima1	170952	12	98643479	98689656	-
7587	1441724_at	Region	None	None	9	100833426	100833940	+
7588	1419710_at	Region	Nxph3	104079	11	95330939	95335358	-
7589	1460660_x_at	Region	Rer1	67830	4	153566474	153578659	-
7590	1456599_at	Region	Nxt2	237082	X	135668250	135679730	+
7591	1429411_a_at	Region	Eny2	223527	15	44373735	44383309	+
7592	1457930_at	Region	Jarid1c	20591	X	145767667	145808191	+
7593	1422865_at	Region	Runx1	12394	16	91765168	91858413	-
7594	1450090_at	Region	Zfp101	22643	17	31079009	31092917	-
7595	1458308_at	Region	Stno	216161	10	80180244	80225913	-
7596	1432217_a_at	Region	1700019F09Rik	71860	11	67650462	67691292	-
7597	1441938_x_at	Region	Cables1	63955	18	12026875	12133165	+
7598	1423209_at	Region	0610041E09Rik	66074	13	86207541	86225129	+
7599	1424638_at	Region	Cdkn1a	12575	17	26902192	26909139	+
7600	1429612_at	Region	Eml4	78798	17	81184646	81313423	+
7601	1432644_at	Region	6430711C07Rik	76225	6	27952566	27954091	+
7602	1419486_at	Region	Foxc1	17300	13	31286268	31290257	+
7603	1427417_at	Region	Scml4	268297	10	42967077	43067140	+
7604	1434262_at	Region	None	None	11	113482450	113484044	+
7605	1447945_at	Region	None	None	8	115019852	115020530	-
7606	1460474_at	Region	2610028L16Rik	76368	12	17816096	17818049	+
7607	1427550_at	Region	Peg10	170676	6	4700265	4713361	+
7608	1431436_a_at	Region	3110023G01Rik	71206	18	77114915	77169011	-
7609	1438581_at	Region	4932439K10Rik	74392	10	75332436	75414133	+
7610	1436310_at	Region	Gemin5	216766	11	57846919	57894102	-
7611	1436976_a_at	Region	9930028C20Rik	226418	1	130566762	130571455	+

7612	1436625_at	Region	Fcgr1	14129	3	95770745	95781805	-
7613	1416346_at	Region	Timm8a	30058	X	128082197	128086568	-
7614	1432000_a_at	Region	Dedd	21945	1	171267091	171270454	+
7615	1425550_a_at	Region	Prkar1a	19084	11	109472038	109490737	+
7616	1428832_at	Region	1600002H07Rik	72016	17	22018639	22024354	-
7617	1433679_at	Region	Fubp3	320267	2	31527019	31549061	+
7618	1427168_a_at	Region	Col14a1	12818	15	55311277	55525876	+
7619	1418543_s_at	Region	D11Ertd707e	52715	11	102505777	102518814	-
7620	1423555_a_at	Region	Ifi44	99899	3	150711335	150730313	-
7621	1449750_at	Region	AA407331	106839	NONE	NONE	NONE	NONE
7622	1455834_x_at	Region	Tacc3	21335	5	32147518	32160849	+
7623	1439871_at	Region	Lmtk2	231876	5	143211341	143229817	+
7624	1438664_at	Region	Prkar2b	19088	12	28534800	28637055	-
7625	1424591_at	Region	5830433M19Rik	67770	4	93475153	93531856	-
7626	1418592_at	Region	Dnaja4	58233	9	54817109	54833863	+
7627	1457303_at	Region	2700067D09Rik	72615	16	4611327	4634162	-
7628	1446036_at	Region	AU018728	106981	18	42978398	42979134	-
7629	1433941_at	Region	Pib5pa	170835	11	3389170	3399608	-
7630	1453237_at	Region	Qars	97541	9	108556974	108564815	+
7631	1451005_at	Region	Sumo1	22218	1	59942975	59974347	-
7632	1426375_s_at	Region	BC019806	218885	14	30218148	30235400	+
7633	1424487_x_at	Region	Txnrd1	50493	10	82746224	82784757	+
7634	1455638_at	Region	None	None	8	94607184	94607824	-
7635	1446626_at	Region	D16H22S680E	27883	16	17071867	17095487	-
7636	1453746_at	Region	Fnbp1	14269	2	30985397	31074101	-
7637	1457751_at	Region	4832420A03Rik	320612	7	91735524	91736416	+
7638	1420368_at	Region	Denr	68184	5	123078362	123100021	+
7639	1429806_at	Region	Eral1	57837	11	77799035	77806004	-
7640	1435098_at	Region	None	None	10	39959929	39960674	-
7641	1436608_at	Region	MGC47262	239647	15	97323750	97462319	+
7642	1449092_at	Region	Ppm1d	53892	11	85039246	85075058	+
7643	1423929_at	Region	Phgdhl1	68889	14	116446863	116589587	+
7644	1436793_at	Region	St18	240690	1	6695648	6826537	+
7645	1423218_a_at	Region	Mrpl49	18120	19	5842420	5846541	-
7646	1448962_at	Region	Myh11	17880	16	12930823	13027159	-
7647	1455700_at	Region	Mterfd3	74238	10	85014096	85022661	-
7648	1459869_x_at	Region	4930402E16Rik	319518	8	110392463	110432862	+
7649	1439656_at	Region	Pafah1b1	18472	11	74399613	74450328	-
7650	1437583_x_at	Region	Acas2l	68738	2	150075047	150125165	-
7651	1434182_at	Region	Ddx31	227674	2	28819047	28837734	+
7652	1431253_s_at	Region	4933431N12Rik	71310	8	82412909	82519979	+
7653	1430955_at	Region	2810403A07Rik	74200	3	88429682	88456786	+
7654	1460317_s_at	Region	Gna13	14674	11	109184029	109219306	+
7655	1436972_at	Region	Gpr61	229714	3	107944063	107950624	-
7656	1430952_at	Region	3110039C02Rik	73127	3	25065670	25066876	-
7657	1440548_at	Region	Btbd9	224671	17	28030141	28340405	-
7658	1441554_at	Region	4833446K15Rik	78923	18	59406652	59407180	+
7659	1460345_at	Region	2610208M17Rik	108909	8	3442132	3444816	+
7660	1423957_at	Region	Isg20l1	68048	7	72688034	72700940	+
7661	1434647_at	Region	AU040377	268780	15	6993687	7185872	-
7662	1435076_at	Region	2310047D13Rik	116972	11	75927673	75935074	+
7663	1434274_at	Region	Phr1	105689	14	97658865	97891435	-
7664	1433673_at	Region	LOC432582	432582	11	74366252	74367175	+
7665	1440223_at	Region	Rbm6	19654	9	107841550	107940685	-
7666	1452929_at	Region	Rsn	56430	5	122749321	122826732	-
7667	1447643_x_at	Region	Snai2	20583	16	13416441	13419967	+
7668	1446230_at	Region	Gm944	381126	18	21343840	21521608	-
7669	1421901_at	Region	Eif2ak1	15467	5	142939806	142944198	+
7670	1450670_at	Region	Dbh	13166	2	27097395	27115081	+
7671	1421692_at	Region	Cacna1e	12290	1	154285134	154615539	-
7672	1457140_s_at	Region	4632411J06Rik	78748	7	106807656	106811140	+
7673	1424104_at	Region	Cbpin	68592	4	133891883	133898440	+
7674	1450480_a_at	Region	Gprk6	26385	13	54054856	54068930	+
7675	1418073_at	Region	MGI:1928939	56360	X	148888659	148923797	+
7676	1450818_a_at	Region	Ndufa7	66416	17	31523009	31536811	+

7677	1439263_at	Region	Fin15	14210	6	50344694	50347651	+
7678	1441140_at	Region	Rere	68703	4	148898702	149114159	+
7679	1455895_x_at	Region	1110012M11Rik	73711	8	70692174	70697270	+
7680	1430352_at	Region	8430417A20Rik	74516	6	93270147	93297136	+
7681	1442040_at	Region	None	None	4	117499012	117499705	-
7682	1445480_at	Region	Gpr19	14760	6	135626628	135645189	-
7683	1443242_at	Region	D5Ertd121e	52230	5	23871885	23872226	-
7684	1458446_at	Region	3222401L13Rik	320513	18	37881705	37882651	+
7685	1450278_at	Region	Tacr3	21338	3	133712014	133817494	+
7686	1442342_at	Region	Sp6	83395	11	96834656	96845821	+
7687	1446627_at	Region	Stk3	56274	15	34876656	35155734	-
7688	1453851_a_at	Region	Gadd45g	23882	13	50400377	50402159	+
7689	1448708_at	Region	Pparbp	19014	11	97973241	98014347	-
7690	1449342_at	Region	Ptplb	70757	16	33805502	33889652	+
7691	1458849_at	Region	Asah2	54447	19	31310118	31428612	-
7692	1441405_at	Region	None	None	18	38946621	38947439	+
7693	1439580_at	Region	B930053N05Rik	105425	14	69131879	69132728	-
7694	1425135_a_at	Region	Dnm2	13430	9	21313566	21396180	+
7695	1435372_a_at	Region	Pa2g4	18813	10	128295733	128303038	-
7696	1434465_x_at	Region	Vldlr	22359	19	26457498	26489686	+
7697	1438226_at	Region	AU022252	230696	4	118185114	118191611	-
7698	1435160_at	Region	1110064P04Rik	268390	11	23383337	23392736	-
7699	1457869_at	Region	None	None	12	73305786	73306333	+
7700	1444466_at	Region	Ncald	52589	15	37371112	37797344	-
7701	1425699_a_at	Region	Abhd14a	68644	9	106433630	106441207	-
7702	1440079_at	Region	3632454L22Rik	320606	X	128567667	128568316	-
7703	1457145_at	Region	Plekhg4	102075	8	104670532	104678487	+
7704	1428119_a_at	Region	Rap2c	72065	X	45525090	45538760	-
7705	1427908_at	Region	Bnip1	224630	17	24576290	24587338	+
7706	1420822_s_at	Region	Sgpp1	81535	12	72570627	72592022	-
7707	1445456_at	Region	Kcmf1	74287	6	73177405	73198187	-
7708	1424788_at	Region	BC021438	215194	9	21159727	21174226	-
7709	1433598_at	Region	9430010O03Rik	234023	8	8032433	8056378	-
7710	1427683_at	Region	Egr2	13654	10	67591672	67595982	+
7711	1426060_at	Region	Cul4a	99375	8	12484378	12526411	+
7712	1415723_at	Region	Eif5	217869	12	107012763	107017765	+
7713	1459222_x_at	Region	None	None	NONE	NONE	NONE	NONE
7714	1455868_a_at	Region	Tubgcp2	74237	7	134394993	134435263	-
7715	1441667_s_at	Region	Smyd1	12180	6	71547104	71594179	-
7716	1438967_x_at	Region	Amhr2	110542	15	102505839	102515049	+
7717	1420387_at	Region	Mpv17	17527	5	29599886	29613169	-
7718	1457203_at	Region	None	None	3	55462048	55462695	-
7719	1424118_a_at	Region	Spbc25	66442	2	69049296	69061557	-
7720	1453799_at	Region	9430038I01Rik	77252	7	131739470	131779393	-
7721	1434195_at	Region	Prss35	244954	9	87085453	87100637	+
7722	1447091_at	Region	Jmjd2c	76804	4	73313167	73467080	+
7723	1457676_at	Region	Tirap	117149	9	35100808	35113918	-
7724	1456961_at	Region	Eny2	223527	15	44373735	44383309	+
7725	1415952_at	Region	Mark2	13728	19	6990598	7055161	-
7726	1451531_at	Region	Cbwd1	226043	19	24160457	24201588	-
7727	1456356_at	Region	B230217O12Rik	320879	19	56908922	56909654	-
7728	1435438_at	Region	Sox8	20681	17	23371562	23376362	-
7729	1460018_at	Region	D11Ertd759e	268513	11	119297145	119297814	-
7730	1433398_at	Region	5830461L01Rik	76091	13	48876129	48877130	-
7731	1434521_at	Region	9930116O05Rik	319758	9	72752141	72755606	+
7732	1443566_at	Region	A330103N21Rik	77773	7	61958738	61959255	+
7733	1460122_at	Region	Tmem41b	233724	7	103825350	103839101	-
7734	1455832_a_at	Region	Umps	22247	16	32763201	32775192	-
7735	1458630_at	Region	Vav3	57257	3	109150078	109494759	+
7736	1429419_at	Region	2310007A19Rik	66353	3	93952806	93961414	-
7737	1438469_at	Region	Bcorl1	320376	X	42889570	42927501	+
7738	1451909_a_at	Region	Prpf4b	19134	13	34407672	34435208	+
7739	1445550_at	Region	None	None	4	24748076	24748629	-
7740	1455461_at	Region	A530017D24Rik	103880	11	61591297	61594459	-
7741	1426090_a_at	Region	Fert2	14158	17	61637960	61842673	+

7742	1444231_at	Region	Zdhhc6	66980	19	54859814	54896233	-
7743	1424846_at	Region	BC027174	216543	11	20123299	20144210	-
7744	1439071_at	Region	5430416N02Rik	71426	5	99439474	99448174	-
7745	1420363_at	Region	Biklk	12124	15	83575461	83593234	+
7746	1448264_a_at	Region	Eif3s2	54709	4	128619087	128627726	-
7747	1453833_a_at	Region	Rnaseh1	19819	12	25214880	25224831	+
7748	1452471_at	Region	Il17rd	171463	14	25177287	25240714	+
7749	1452698_at	Region	Tsfm	66399	10	126755051	126763533	-
7750	1424424_at	Region	Slc39a1	30791	3	90058648	90064030	+
7751	1456587_x_at	Region	2010005J08Rik	72046	11	5608972	5635937	-
7752	1426147_s_at	Region	Cldn10	58187	14	113343391	113430546	+
7753	1453200_at	Region	Rai1	19377	11	59865724	59917667	+
7754	1419245_at	Region	Rab14	68365	2	35113675	35132954	-
7755	1419354_at	Region	Klf7	93691	1	64328681	64414407	-
7756	1415703_at	Region	Huwe1	59026	X	145451533	145469813	+
7757	1451464_at	Region	Mfap3	216760	11	57244336	57256528	+
7758	1437227_at	Region	2900073H19Rik	68205	2	29759551	29777158	+
7759	1430428_at	Region	2310066F23Rik	70409	19	55135717	55137191	+
7760	1426018_a_at	Region	Sox6	20679	7	109336962	109662307	-
7761	1429508_at	Region	2310057M21Rik	68277	7	125654758	125677792	-
7762	1459041_at	Region	None	None	X	95764991	95765448	-
7763	1455250_at	Region	Sh3bp4	98402	1	88890095	88973785	+
7764	1432452_at	Region	1700054O19Rik	74272	14	24081604	24082471	-
7765	1430778_a_at	Region	Nubp1	26425	16	10086475	10098031	+
7766	1429352_at	Region	Mocos	68591	18	24881983	24930071	+
7767	1449572_at	Region	Trhr	22045	15	44141857	44181536	+
7768	1443916_at	Region	2900026A02Rik	243219	5	112159902	112240390	-
7769	1451370_at	Region	None	None	4	43409286	43411844	+
7770	1458128_at	Region	C430010P07Rik	227227	1	67011628	67093590	-
7771	1418737_at	Region	Nudt2	66401	4	41616365	41619763	+
7772	1439963_x_at	Region	1700129I04Rik	330577	7	76489483	76505046	-
7773	1448195_at	Region	Taf5l	102162	8	123283382	123308381	-
7774	1457791_at	Region	B830008H07Rik	101646	7	39551853	39552521	-
7775	1417134_at	Region	Srpk2	20817	5	21967253	22080468	-
7776	1448957_at	Region	Rbpsuh	19664	5	52400484	52466172	+
7777	1454475_at	Region	4921524M04Rik	70890	7	119541286	119542790	+
7778	1442326_at	Region	Pcdh15	11994	10	74319368	74727834	+
7779	1447415_at	Region	G630013P12Rik	218513	13	96291899	96292159	-
7780	1416596_at	Region	2210409B01Rik	70129	17	32618260	32634136	+
7781	1427030_at	Region	D16Ertd480e	212514	16	43230787	43271716	+
7782	1424452_at	Region	5730555F13Rik	66660	9	70720790	70732757	+
7783	1449933_a_at	Region	5730449L18Rik	66637	1	152255223	152271166	-
7784	1456335_at	Region	Gm106	226866	1	16033017	16070386	-
7785	1458637_x_at	Region	Ube3c	100763	5	28019724	28126560	+
7786	1448332_at	Region	Pex19	19298	1	172055691	172064699	+
7787	1422839_at	Region	Neurog2	11924	3	126516140	126518626	+
7788	1416831_at	Region	Neu1	18010	17	32635159	32639101	+
7789	1444359_at	Region	2810407L07Rik	69228	6	48173858	48174736	+
7790	1415870_at	Region	Calu	12321	6	29395242	29423765	+
7791	1429694_at	Region	4930402H24Rik	228602	2	130221684	130353963	-
7792	1415864_at	Region	Bpgm	12183	6	34570624	34599878	+
7793	1441702_at	Region	Rfx3	19726	19	27008207	27251206	-
7794	1457216_at	Region	N28103	96936	1	160834701	160835015	+
7795	1426044_a_at	Region	Prkcq	18761	2	11088122	11217235	+
7796	1432591_at	Region	Pappa	18491	4	64215365	64448700	+
7797	1456674_at	Region	E130016E03Rik	320890	4	11485906	11542772	+
7798	1433422_at	Region	2210008N01Rik	76579	2	57048875	57049460	+
7799	1435107_at	Region	AW456874	218232	13	48175930	48223631	-
7800	1448710_at	Region	Cxcr4	12767	1	128431063	128435143	-
7801	1433032_at	Region	2900064K03Rik	72976	5	48281355	48282908	-
7802	1427373_at	Region	Amigo1	229715	3	107982077	107987519	+
7803	1418081_at	Region	Wbscr18	66114	5	134077698	134078857	+
7804	1423615_at	Region	Zfp364	67845	3	96215500	96278811	+
7805	1440985_at	Region	D130023J23Rik	319769	NONE	NONE	NONE	NONE
7806	1421487_a_at	Region	Nck1	17973	9	100396550	100447600	-

7807	1417721_s_at	Region	Laptm5	16792	4	129708150	129733024	+
7808	1439122_at	Region	None	None	9	44626449	44627219	+
7809	1449483_at	Region	Polk	27015	13	92672015	92733811	-
7810	1425900_at	Region	Hkdc1	216019	10	62349472	62388792	-
7811	1438498_at	Region	LOC574428	574428	11	70190479	70191873	+
7812	1434627_at	Region	None	None	6	30163315	30187986	+
7813	1421034_a_at	Region	Il4ra	16190	7	119602018	119627698	+
7814	1460098_at	Region	None	None	12	104260749	104261840	-
7815	1439820_at	Region	Ebf1	13591	11	44370971	44758012	+
7816	1430268_at	Region	9630005C17Rik	78743	19	21791848	21793126	+
7817	1444459_at	Region	Lmnb1	16906	18	56949007	56973792	+
7818	1428341_at	Region	Zfp655	72611	5	144270195	144285742	+
7819	1423989_at	Region	2210010N04Rik	70381	5	143236935	143265219	-
7820	1449623_at	Region	Txnrd3	232223	6	90079638	90111154	+
7821	1416870_at	Region	Dnalc4	54152	15	79813776	79826755	-
7822	1427654_a_at	Region	Htr4	15562	18	62640984	62693462	+
7823	1457696_at	Region	Rilp	280408	11	75236579	75238823	+
7824	1417234_at	Region	Mmp11	17385	10	76024964	76034203	-
7825	1427564_at	Region	Diap2	54004	X	123208926	123787987	+
7826	1451029_at	Region	Bcl2l2	12050	14	49400262	49405071	+
7827	1442358_at	Region	AA409587	101447	7	94053651	94054550	-
7828	1430969_at	Region	Mtch2	56428	2	90551904	90571383	+
7829	1416073_a_at	Region	Pcnt1	445007	11	115385575	115405078	+
7830	1442003_at	Region	None	None	X	123923942	123924962	+
7831	1459020_at	Region	Amigo1	229715	3	107982077	107987519	+
7832	1451337_at	Region	Psmf1	228769	2	151172998	151198229	-
7833	1422973_a_at	Region	Thrsp	21835	7	91459228	91463781	-
7834	1439034_at	Region	Spn	20737	7	121184353	121187067	-
7835	1457712_at	Region	Chd8	67772	14	47298256	47322726	-
7836	1444895_at	Region	Jam3	83964	9	26991141	27048370	-
7837	1429882_at	Region	2610005L07Rik	381598	15	1398	8167	+
7838	1442678_at	Region	1810010N17Rik	66264	4	128646387	128651013	-
7839	1429407_at	Region	Pex11c	69129	8	4142	7868	+
7840	1457467_at	Region	None	None	NONE	NONE	NONE	NONE
7841	1444716_at	Region	Mettl2	52686	11	104947576	104961490	+
7842	1447007_at	Region	1700008I05Rik	71841	X	129214254	129214725	+
7843	1436914_at	Region	None	None	2	121528276	121529176	+
7844	1450855_at	Region	Arvcf	11877	16	17119316	17178126	+
7845	1455009_at	Region	Cpd	12874	11	76507648	76572655	-
7846	1439972_at	Region	Etnk1	75320	6	144013618	144053017	+
7847	1443930_at	Region	BC052046	240063	17	30574901	30633632	-
7848	1418944_at	Region	Cysltr1	58861	X	101177882	101204827	-
7849	1416018_at	Region	Dr1	13486	5	107345107	107356731	+
7850	1459565_at	Region	Uty	22290	Y	41346	184254	+
7851	1437449_at	Region	BC056485	237926	11	94360886	94361344	-
7852	1439484_at	Region	4930431H11Rik	73785	3	18560494	18561480	-
7853	1445684_s_at	Region	Hdac2	15182	10	37046787	37075975	+
7854	1436844_at	Region	6330403M23Rik	109169	18	36517844	36520598	+
7855	1449718_s_at	Region	4930453N24Rik	67609	16	63789732	63795010	-
7856	1439062_at	Region	Kbtbd3	69149	9	4217421	4239297	+
7857	1452506_a_at	Region	Ilkap	67444	1	91198873	91221825	-
7858	1438701_at	Region	Bicd1	12121	6	4102	11525	+
7859	1453231_at	Region	Cdkl1	71091	12	66578318	66622177	-
7860	1421035_a_at	Region	Magi3	99470	3	103442526	103647825	-
7861	1447357_at	Region	None	None	11	87739983	87775999	-
7862	1440354_at	Region	Elovl7	74559	13	104441513	104512532	+
7863	1425739_at	Region	Pld1	18805	3	27441540	27550616	+
7864	1456089_at	Region	Trim23	81003	13	100397284	100420234	+
7865	1427135_at	Region	Sfrs12	218543	13	99959715	99982622	-
7866	1438661_a_at	Region	Arf2	11841	11	103787960	103806425	+
7867	1446833_at	Region	Dock9	105445	14	116113166	116206323	-
7868	1445870_at	Region	Cdc42ep2	104252	19	5706351	5707785	-
7869	1431832_x_at	Region	Exosc10	50912	4	147050820	147074759	+
7870	1432272_a_at	Region	Ercc4	50505	16	11850826	11883488	+
7871	1424728_at	Region	BC011248	224823	17	44178175	44184988	-

7872	1455306_at	Region	BC037112	231128	5	32898910	32974787	+
7873	1420968_at	Region	Btbd14b	66830	8	83935746	83953228	-
7874	1416291_at	Region	Psmc4	23996	7	23437587	23445795	-
7875	1444703_at	Region	2810403D21Rik	69964	X	103435843	103488262	+
7876	1435174_at	Region	Rsbn1	229675	3	103355742	103393938	+
7877	1424585_at	Region	Ranbp10	74334	8	105064168	105123194	-
7878	1416104_at	Region	Mpdu1	24070	11	69382362	69388300	-
7879	1417097_at	Region	Nrbf1	26922	4	130640356	130664652	+
7880	1422954_at	Region	Zfp60	22718	7	23111116	23124185	+
7881	1421911_at	Region	Stat2	20847	10	128007395	128029668	+
7882	1434610_at	Region	Plec1	18810	15	76219648	76280050	-
7883	1419568_at	Region	Mapk1	26413	16	15754059	15816626	+
7884	1452796_at	Region	Def6	23853	17	26003748	26024517	+
7885	1443683_at	Region	Ptprj	19271	2	90140801	90285334	-
7886	1427879_at	Region	1810031K17Rik	69171	1	75423424	75430579	-
7887	1423106_at	Region	Ube2b	22210	11	51738403	51753228	-
7888	1443213_at	Region	Gtdc1	227835	2	44496584	44793784	-
7889	1446760_at	Region	None	None	13	69971333	69971842	-
7890	1436103_at	Region	Rab3ip	216363	10	116595236	116639832	-
7891	1429743_at	Region	6720468P15Rik	78073	19	57094313	57098510	+
7892	1442154_at	Region	U2af1-rs2	22184	X	157534848	157558003	-
7893	1437933_at	Region	Hhip	15245	8	79185726	79272113	-
7894	1439760_x_at	Region	Upk1b	22268	16	37594913	37621984	-
7895	1437835_a_at	Region	0610011L14Rik	68295	2	156004536	156025909	+
7896	1419309_at	Region	Gp38	14726	4	142182695	142214661	-
7897	1454268_a_at	Region	Cyba	13057	8	121806363	121814514	-
7898	1426005_at	Region	Dmp1	13406	5	103238499	103249944	+
7899	1439180_at	Region	A430093A21Rik	329170	1	63346790	63347271	-
7900	1428354_at	Region	Foxk2	68837	11	121080635	121128991	+
7901	1448984_at	Region	Ercc4	50505	16	11850826	11883488	+
7902	1426100_a_at	Region	Tk2	57813	8	103521036	103542872	-
7903	1449322_at	Region	Ptp4a1 /// LOC385423 /// LOC433406 /// LOC434095	19243	1	31213857	31222010	-
7904	1423921_at	Region	C77668	229543	3	90201806	90244054	-
7905	1451890_at	Region	Kif13a	16553	13	46347970	46527871	-
7906	1436441_at	Region	None	None	15	5014873	5015567	+
7907	1456940_at	Region	Slc43a2	215113	11	75257363	75300699	+
7908	1457062_at	Region	1700081L11Rik	76719	11	104154591	104263380	-
7909	1454538_at	Region	D730048A03Rik	77541	12	75465837	75466836	+
7910	1431071_at	Region	Mtx3	382793	13	89039781	89041514	+
7911	1458230_at	Region	Gria4	14802	9	4328268	4706535	-
7912	1456813_at	Region	None	None	NONE	NONE	NONE	NONE
7913	1424019_at	Region	Nol1	110109	6	125787913	125800927	+
7914	1453880_s_at	Region	1700041C02Rik	73332	4	118281798	118374408	-
7915	1443554_at	Region	Ssbp3	72475	4	105870375	106008599	+
7916	1459054_at	Region	BC035954	194162	4	147443534	147445675	+
7917	1435283_s_at	Region	Gm967	381217	19	23211527	23268980	-
7918	1455775_at	Region	None	None	NONE	NONE	NONE	NONE
7919	1427305_at	Region	Piga	18700	X	158019221	158033321	+
7920	1457883_at	Region	None	None	4	33449202	33449956	-
7921	1434162_at	Region	2700078E11Rik	78832	19	60127914	60181028	-
7922	1449119_at	Region	Arih2	23807	9	108651717	108698223	-
7923	1446712_at	Region	Ntrk2	18212	13	57448390	57572946	+
7924	1445364_at	Region	None	None	17	24766842	24767498	+
7925	1426312_at	Region	Bre	107976	5	30156988	30543677	+
7926	1458951_at	Region	Vrk1	22367	12	101457929	101523008	+
7927	1455547_at	Region	Scrg3	20286	15	81795789	81846239	+
7928	1436845_at	Region	Axin2	12006	11	108741455	108771872	+
7929	1435753_a_at	Region	Nucks1	98415	1	131762351	131784790	+
7930	1441550_at	Region	9330184L24Rik	402729	X	114284287	114284952	+
7931	1421145_at	Region	Slc26a2	13521	18	10304	24932	+
7932	1430694_at	Region	None	None	8	24537265	24539255	-
7933	1439207_at	Region	mKIAA1934	385377	X	67695873	67697729	-

7934	1433047_at	Region	5330430B06Rik	78280	17	7249452	7250645	+
7935	1428076_s_at	Region	Ndufb4	68194	16	36467069	36473835	-
7936	1427571_at	Region	Shh	20423	5	26906864	26917112	-
7937	1443833_at	Region	None	None	6	127490507	127490708	-
7938	1455666_at	Region	A230042K10Rik	218314	13	63913184	63932103	-
7939	1418356_at	Region	Mpst	246221	15	78461324	78465292	+
7940	1433608_at	Region	Scfd2	212986	5	73291008	73366380	-
7941	1430827_a_at	Region	Ptk2	14083	15	73233486	73423105	-
7942	1454369_a_at	Region	Nfatc4	73181	14	50343024	50351959	+
7943	1434217_at	Region	C330019G07Rik	215476	5	31277729	31316129	-
7944	1433617_s_at	Region	B4galt5	56336	2	166757794	166805916	-
7945	1424342_at	Region	Fyttd1	69823	16	31685892	31716927	+
7946	1439233_at	Region	Tloc1	69276	3	30204876	30231459	+
7947	1444700_at	Region	E530011G23Rik	319856	12	85108664	85109688	-
7948	1455404_at	Region	Jph2	59091	2	162794742	162854919	-
7949	1430046_at	Region	3110030K17Rik	73162	4	137776644	137803746	-
7950	1457588_at	Region	C76213	97124	4	42908649	42908946	-
7951	1442085_at	Region	Whsc1l1	234135	8	24322608	24439465	+
7952	1429080_at	Region	Mphosph10	67973	7	58155241	58166183	-
7953	1437198_at	Region	None	None	11	82542022	82543999	+
7954	1433029_at	Region	Kcnj9	16524	1	172251006	172258201	-
7955	1450913_at	Region	B4galt6	56386	18	20900931	20961332	-
7956	1455414_at	Region	Ttc16	338348	2	32694340	32707501	-
7957	1428220_at	Region	5730419I09Rik	74741	6	143857967	143947128	-
7958	1444744_at	Region	None	None	2	180382893	180383696	-
7959	1454683_at	Region	Sfrs8	231769	5	128676882	128691989	+
7960	1423752_at	Region	Ddx47	67755	6	135769073	135781224	+
7961	1430370_at	Region	Eprs	107508	1	184906893	184908652	+
7962	1450643_s_at	Region	Acsl1	14081	8	45417959	45481923	+
7963	1435037_at	Region	Perld1	320655	11	98209764	98224134	-
7964	1423779_at	Region	Chchd6	66098	6	89818799	90031255	-
7965	1424398_at	Region	Dhx36	72162	3	62137118	62174023	-
7966	1426869_at	Region	Boc	117606	16	43368375	43441912	-
7967	1421082_s_at	Region	Banf1	23825	19	5153430	5155435	-
7968	1450810_at	Region	Fshr	14309	17	86847074	87063567	-
7969	1416878_at	Region	Mrpl51	66493	6	125848878	125851070	+
7970	1424576_s_at	Region	Cyp2c44	226143	19	43549428	43573605	-
7971	1439563_at	Region	AI854635	193043	11	70490105	70498572	+
7972	1422054_a_at	Region	Skil	20482	3	30508258	30530416	+
7973	1420139_s_at	Region	Hrb2	52705	10	111675789	111690089	+
7974	1416280_at	Region	Uble1b	50995	7	29294488	29322320	-
7975	1446565_at	Region	Man1b	17156	3	99992328	100111138	-
7976	1433874_at	Region	Ssh1	231637	5	113052863	113104401	-
7977	1446031_at	Region	None	None	NONE	NONE	NONE	NONE
7978	1447117_at	Region	Lrrtm1	74342	6	77592843	77607877	+
7979	1441507_at	Region	Spnb2	20742	11	29995224	30114564	-
7980	1447411_at	Region	Ugdh	22235	5	64215050	64237671	-
7981	1441412_s_at	Region	Trim45	229644	3	100348526	100359922	+
7982	1446143_at	Region	Myst3	244349	8	21614114	21695221	+
7983	1451428_x_at	Region	Egfl7	353156	2	26513245	26524844	+
7984	1447443_at	Region	None	None	10	60067731	60068134	+
7985	1417742_a_at	Region	Dmap1	66233	4	116633591	116641130	-
7986	1443426_at	Region	5730555F13Rik	66660	9	70720790	70732757	+
7987	1441891_x_at	Region	Elovl7	74559	13	104441513	104512532	+
7988	1434236_at	Region	Zdhhc20	75965	14	52372502	52425052	-
7989	1447897_x_at	Region	Anapc11	66156	11	120419572	120429291	+
7990	1443257_at	Region	9630050E16Rik	320168	14	40566715	40567128	-
7991	1458397_at	Region	---	433696	4	40858910	40859528	-
7992	1435702_s_at	Region	Ywhae	22627	11	75458597	75491496	+
7993	1426240_at	Region	Chmp4b	75608	2	154114001	154151720	+
7994	1448173_a_at	Region	Top3b	21976	16	15641732	15663698	+
7995	1446240_at	Region	Map2k5	23938	9	63284916	63498276	-
7996	1457264_at	Region	Phf20l1	239510	15	66601624	66648123	+
7997	1444194_at	Region	Mllt10	17354	2	18097281	18254400	+
7998	1449150_at	Region	A930040G15Rik	77827	6	48528544	48549779	+

7999	1433650_at	Region	Haghl	68977	17	23588825	23591333	-
8000	1454932_at	Region	Rcor1	217864	12	106514818	106586780	+
8001	1433810_x_at	Region	None	None	11	106601570	106603057	-
8002	1442245_at	Region	Carm1	59035	9	21456863	21476863	+
8003	1448394_at	Region	Myl2	17906	5	121253338	121259202	+
8004	1450680_at	Region	Rag1	19373	2	101342965	101354184	-
8005	1417300_at	Region	Smpdl3b	100340	4	131693865	131718070	-
8006	1421636_at	Region	9030605E16Rik	75757	X	133248091	133250428	+
8007	1431511_at	Region	9230110F15Rik	77080	9	35750788	35756609	-
8008	1416185_a_at	Region	Adh5	11532	3	137332399	137344734	+
8009	1429560_at	Region	MGI:3028594	77652	17	30988427	31006091	+
8010	1424048_a_at	Region	Nqo3a2	72017	1	134256253	134262001	+
8011	1430672_at	Region	5033418A18Rik	75961	X	42962347	42963908	-
8012	1433622_at	Region	Gemin4	276919	11	75936229	75943170	-
8013	1415867_at	Region	Cct4	12464	11	22885383	22898126	+
8014	1422804_at	Region	Serpinb6b	20708	13	32445135	32458659	+
8015	1428010_at	Region	Timm10	30056	12	67958182	67971658	-
8016	1434413_at	Region	Igf1	16000	10	87833042	87904980	+
8017	1433821_at	Region	1110012D08Rik	73827	10	128537126	128541460	-
8018	1452018_at	Region	Nkx2-6	18092	14	63705608	63709129	+
8019	1434189_at	Region	Stag1	20842	9	100545170	100860091	+
8020	1451391_at	Region	2700050L05Rik	214764	7	128005358	128040623	+
8021	1445553_at	Region	E130307J04Rik	320360	7	102899682	102936254	-
8022	1425654_a_at	Region	D15Ertd366e	65970	15	99835724	99877288	-
8023	1437896_at	Region	2700088M22Rik	67197	15	93452324	93464501	-
8024	1432673_at	Region	2300010F08Rik	71875	4	29110389	29112459	+
8025	1416651_at	Region	Znhit2	29805	19	5849997	5851258	+
8026	1418192_at	Region	Mnt	17428	11	74556582	74571368	+
8027	1444156_at	Region	9230112E08Rik	320800	2	180701190	180702549	+
8028	1448897_at	Region	Mkrn2	67027	6	116039545	116056254	+
8029	1460275_at	Region	Gpr3	14748	4	132170265	132172756	-
8030	1446358_at	Region	Bai3	210933	1	25316949	26075533	-
8031	1459488_at	Region	None	None	14	69633569	69634167	+
8032	1429557_at	Region	Mcm8	66634	2	132330188	132358050	+
8033	1426405_at	Region	Rnf11	29864	4	108411762	108435410	-
8034	1434546_at	Region	BC024683	229512	3	88079530	88106201	+
8035	1426897_at	Region	Rcc2	108911	4	139582803	139602736	+
8036	1425747_at	Region	Dock5	277133	14	62284170	62343871	-
8037	1435418_at	Region	Slc22a8	19879	19	7787836	7808381	+
8038	1446758_at	Region	Bre	107976	5	30156988	30543677	+
8039	1425806_a_at	Region	Surb7	108098	6	147563269	147571290	+
8040	1447365_at	Region	None	None	19	37655577	37656014	+
8041	1440653_at	Region	Phip	83946	9	83198955	83305205	-
8042	1427569_a_at	Region	Utrn	22288	10	12263109	12646998	-
8043	1428121_at	Region	2610528K11Rik	72543	2	33662118	33820108	-
8044	1457541_at	Region	LOC434756	434756	X	31771254	31789396	-
8045	1417326_a_at	Region	Anapc11	66156	11	120419572	120429291	+
8046	1436285_at	Region	4933409I22	244608	8	94812404	94837431	+
8047	1443151_at	Region	None	None	NONE	NONE	NONE	NONE
8048	1431153_at	Region	E530011G23Rik	319856	12	84989756	84991612	+
8049	1454060_a_at	Region	Nras	18176	3	102486135	102496241	+
8050	1442114_at	Region	Xab1	74254	5	29953699	29970565	+
8051	1453601_at	Region	5430400N05Rik	209225	7	73831628	73891602	+
8052	1420653_at	Region	Tgfb1	21803	7	20851917	20869779	+
8053	1456324_at	Region	2610014M12Rik	212276	13	64146690	64154874	-
8054	1453609_s_at	Region	Ccdc9	243846	7	740	2049	-
8055	1422709_a_at	Region	Wdr46	57315	17	31644466	31653438	+
8056	1451570_a_at	Region	6720467C03Rik	68099	4	12080689	12098962	-
8057	1460153_at	Region	Abi2	329165	1	60713087	60784447	+
8058	1449554_at	Region	Tle3	21887	9	61493517	61536829	+
8059	1433762_at	Region	C630043F03Rik	68285	4	71293960	71295103	+
8060	1447934_at	Region	9630033F20Rik	319801	6	127760801	127782742	-
8061	1453451_at	Region	6820402I19Rik	74509	14	17218594	17220328	-
8062	1429474_at	Region	Zadh1	77219	12	81153770	81182471	+
8063	1421146_at	Region	Rapgef1	107746	2	29551925	29670492	+

8064	1437939_s_at	Region	Ctsc	13032	7	82254582	82287335	+
8065	1442651_at	Region	Wwox	80707	8	113753199	114666211	+
8066	1456600_a_at	Region	Rnf7	19823	9	96369942	96420174	-
8067	1446732_at	Region	None	None	4	145974668	145975287	+
8068	1433886_at	Region	Eif2b5	224045	16	19270969	19281464	+
8069	1458040_at	Region	None	None	7	103971736	103972474	-
8070	1427533_at	Region	Als2cl	235633	9	110921471	110941821	+
8071	1433571_at	Region	A130038L21Rik	218442	13	88778759	88896020	+
8072	1437039_at	Region	Cops2	12848	2	125345452	125372846	-
8073	1452835_a_at	Region	Polrmt	216151	10	79858754	79869197	-
8074	1436036_at	Region	Whsc1	107823	5	32384228	32386230	+
8075	1457355_at	Region	None	None	12	40969014	40969986	+
8076	1424355_a_at	Region	Sin3b	20467	8	71873343	71907207	+
8077	1432924_at	Region	2810404I24Rik	76949	19	42110442	42112369	+
8078	1448313_at	Region	Cln2	12751	7	99859499	99866859	-
8079	1452622_a_at	Region	Tradd	71609	8	104554167	104556311	-
8080	1445566_at	Region	Centb2	78618	16	29953373	29953905	-
8081	1450401_at	Region	Ncoa6ip	116940	4	3502044	3543770	+
8082	1456002_at	Region	Xpa	22590	4	46091161	46112233	-
8083	1441976_at	Region	Rnf19	30945	15	36241202	36267284	-
8084	1437206_at	Region	2900045N06Rik	72895	6	113640716	113716498	+
8085	1459900_at	Region	C79468	97294	6	55041125	55042294	+
8086	1444632_at	Region	None	None	6	129694506	129695564	+
8087	1417526_at	Region	Pcbp3	59093	10	76863181	76993973	-
8088	1421518_at	Region	Kcns1	16538	2	163621274	163628058	-
8089	1420546_at	Region	Th	21823	7	137305795	137312985	-
8090	1426746_at	Region	1810026J23Rik	69773	9	21480123	21486981	+
8091	1454944_at	Region	Hic2	58180	16	16025980	16032597	+
8092	1422744_at	Region	Phka1	18679	X	97117039	97245563	-
8093	1421981_at	Region	Kcnc3	16504	7	38665933	38671377	+
8094	1437829_s_at	Region	Eef2k	13631	7	114718450	114779568	+
8095	1445689_at	Region	Rbms1	56878	2	60608122	60818320	-
8096	1441200_at	Region	Klf3	16599	5	63601098	63627704	+
8097	1436269_s_at	Region	Prss25	64704	6	49801	53090	-
8098	1428575_at	Region	Fcho1	74015	8	70858707	70875819	-
8099	1428634_at	Region	Twistnb	28071	12	30039599	30048310	+
8100	1445697_at	Region	---	270387		1619156	1619474	-
8101	1416805_at	Region	1110032E23Rik	68659	3	79616111	79673508	+
8102	1419404_s_at	Region	Siah1a /// Siah1b	20437	8	86007609	86029536	-
8103	1416324_s_at	Region	2410004N11Rik	66989	17	26754605	26771684	+
8104	1423887_a_at	Region	1200003M09Rik	71718	17	22905428	22921626	-
8105	1456624_at	Region	D11Ertd498e	52639	11	109394610	109432478	-
8106	1453636_at	Region	Pcgf5	76073	19	35721737	35798874	+
8107	1430590_at	Region	2810009O15Rik	73852	3	41247547	41261082	+
8108	1428091_at	Region	Klhl7	52323	5	22564507	22624682	+
8109	1448016_at	Region	None	None	3	115398610	115399916	+
8110	1440302_at	Region	Slc25a3	18674	10	91087904	91095289	-
8111	1433977_at	Region	Hs3st3b1	54710	11	63614682	63647884	-
8112	1442622_at	Region	Strbp	20744	2	37415390	37579381	-
8113	1423565_at	Region	Paics	67054	5	76195870	76211964	+
8114	1451789_a_at	Region	Ryk	20187	9	102804509	102877376	+
8115	1451390_s_at	Region	1110060O18Rik	68818	1	75456524	75459403	+
8116	1450882_s_at	Region	Tm7sf1	83924	13	12777506	12812482	-
8117	1434411_at	Region	None	None	9	79804293	79805243	-
8118	1430279_at	Region	3110038A09Rik	73126	18	31873754	31874286	-
8119	1427920_at	Region	Phf19	74016	2	34825917	34846138	-
8120	1423012_at	Region	Syt7	54525	19	9585980	9644941	+
8121	1448128_at	Region	Ppgb	19025	2	164290539	164297765	+
8122	1451168_a_at	Region	Arhgdia	192662	11	120399205	120402340	-
8123	1453920_a_at	Region	Mospd2	76763	X	158535574	158579740	-
8124	1425309_at	Region	Catsper2	212670	2	120908208	120927384	-
8125	1422883_at	Region	Usp8	84092	2	126221216	126273150	+
8126	1428191_s_at	Region	D14Ertd209e	52535	14	46979197	46986291	+
8127	1424578_at	Region	Arrdc1	215705	2	24857514	24867325	-
8128	1448385_at	Region	Slc15a4	100561	5	126762650	126784376	-

8129	1449904_at	Region	2610510B01Rik /// LOC547290	547290	16	1165278	1193622	-
8130	1430025_at	Region	Ppp3cc	19057	14	64533887	64605438	-
8131	1424665_at	Region	5430405G24Rik	237943	11	102298304	102329534	-
8132	1421395_at	Region	Zik1	22775	7	8786037	8794152	-
8133	1422516_a_at	Region	Fibp	58249	19	5249484	5253784	+
8134	1458493_a_at	Region	None	None	8	6403706	6404162	-
8135	1445037_at	Region	6430510B20Rik	320025	5	113863874	113864707	-
8136	1457954_at	Region	None	None	4	8794230	8794757	-
8137	1427638_at	Region	Zbtb16	235320	9	48684510	48863820	-
8138	1460171_at	Region	Cops5	26754	1	10129944	10143020	-
8139	1424078_s_at	Region	Pex6	224824	17	44222425	44236648	+
8140	1451819_at	Region	Zswim6	67263	13	103950578	104158812	-
8141	1422888_at	Region	Rnf5	54197	17	32303550	32306102	-
8142	1457760_at	Region	A930004J17Rik	319635	4	130676253	130677249	+
8143	1430966_at	Region	Cml3	93674	6	86243280	86244659	-
8144	1438530_at	Region	Tfpi	21788	2	84130609	84171923	-
8145	1435234_at	Region	Ncoa2	17978	1	13272503	13507008	-
8146	1459707_at	Region	Pacs1	107975	19	4922475	5061909	-
8147	1439888_at	Region	None	None	1	192593007	192595716	+
8148	1416477_at	Region	Ube2d2	56550	18	35995272	36030828	+
8149	1425074_at	Region	Wrn	22427	8	32039864	32191103	-
8150	1448849_at	Region	Mrpl40	18100	16	17643267	17647687	-
8151	1423143_at	Region	Gtpbp4	69237	13	8956055	8979603	-
8152	1445949_at	Region	Tnfrsf19	29820	14	55499179	55581612	-
8153	1432997_at	Region	5830462P14Rik	76098	14	60791949	60793605	-
8154	1425824_a_at	Region	Pcsk4	18551	10	80446311	80454484	-
8155	1458811_at	Region	9430047L24Rik	320850	3	140465086	140465921	+
8156	1447176_at	Region	A930008G19Rik	77938	7	127078161	127179580	-
8157	1435182_at	Region	C530028I08Rik	232933	7	15760799	15780242	-
8158	1453308_at	Region	8430429K09Rik	71523	11	3347259	3353126	+
8159	1417228_at	Region	Capn1	12333	19	5777335	5803941	-
8160	1441367_a_at	Region	None	None	NONE	NONE	NONE	NONE
8161	1455002_at	Region	Ptp4a1	19243	1	31213857	31222010	-
8162	1443240_at	Region	Gpc3	14734	X	46793222	47134717	-
8163	1432236_a_at	Region	Suv39h1	20937	X	6300242	6313526	-
8164	1415936_at	Region	Bcar3	29815	3	121200933	121310740	+
8165	1429464_at	Region	Prkaa2	108079	4	103993895	104068774	-
8166	1440253_at	Region	None	None	11	80198613	80198903	+
8167	1436580_at	Region	D8Ertd354e	52120	8	24669208	24671231	-
8168	1452272_a_at	Region	Gfer	11692	17	22498277	22501235	-
8169	1451108_at	Region	Rnf185	193670	11	3310770	3347112	-
8170	1451102_at	Region	Cnot8	69125	11	57829782	57844223	+
8171	1433923_at	Region	Krt2-39	406220	15	101918318	101928080	-
8172	1441912_x_at	Region	C2	12263	17	32565421	32584866	-
8173	1439072_at	Region	Slc1a3	20512	15	8424368	8500974	-
8174	1418928_a_at	Region	2310038H17Rik	67099	1	64900564	64911252	-
8175	1423862_at	Region	Plekhf2	71801	4	10915627	10934525	-
8176	1418432_at	Region	Cab39	12283	1	85601719	85656721	+
8177	1419047_at	Region	Pcnx	54604	12	78720867	78861705	+
8178	1441428_at	Region	None	None	1	51808112	51808751	-
8179	1420660_at	Region	Lrrc6	54562	15	66403753	66524736	-
8180	1447349_s_at	Region	Ep400	75560	5	109714361	109820853	-
8181	1426592_a_at	Region	Fbxo22	71999	9	55320406	55335959	+
8182	1416726_s_at	Region	Ube2s	77891	7	4053868	4058194	-
8183	1419913_at	Region	Strap	20901	6	138527082	138543054	+
8184	1417449_at	Region	Pte1	170789	2	164249501	164261605	-
8185	1427043_s_at	Region	Cova1	209224	X	43531318	43809038	-
8186	1453274_at	Region	1700039D13Rik	73293	11	102702333	102706304	+
8187	1453433_at	Region	2600001A11Rik	72338	12	72487759	72525892	-
8188	1424098_at	Region	Elovl7	74559	13	104441513	104512532	+
8189	1436498_at	Region	Arih1	23806	9	59509355	59603044	-
8190	1441210_at	Region	Myo1b	17912	1	52050933	52208258	-
8191	1416622_at	Region	Wbscr16	94254	5	133439452	133468143	-
8192	1434695_at	Region	None	None	1	191273729	191274488	-

8193	1460123_at	Region	Gpr1	241070	1	63475709	63507227	-
8194	1433814_at	Region	None	None	6	119909753	119910437	-
8195	1429485_a_at	Region	Utp11l	67205	4	123705877	123720667	-
8196	1422787_at	Region	Fkbpl	56299	17	32348153	32349597	+
8197	1423680_at	Region	Fads1	76267	19	9379470	9393371	+
8198	1458538_at	Region	Sirt1	93759	10	63283769	63303800	-
8199	1445514_at	Region	Vhlh	22346	6	114191852	114199465	+
8200	1435139_at	Region	Narg1	74838	3	51048093	51105995	+
8201	1454616_at	Region	5730410I19Rik	66622	12	98203846	98224080	+
8202	1424096_at	Region	Krt2-5	110308	15	101765532	101771353	-
8203	1416860_s_at	Region	Ing1	26356	8	10928492	10935676	+
8204	1420865_at	Region	Zfp161	22666	17	67159059	67165625	+
8205	1455117_at	Region	BC062185	215112	10	53781786	53794040	-
8206	1443490_at	Region	D3Ertd34e	52061	3	89892447	89893008	+
8207	1430928_at	Region	2900002J02Rik	70412	19	19823384	19824928	+
8208	1438999_a_at	Region	Nfat5	54446	8	106591549	106677210	+
8209	1449162_at	Region	Pop7	74097	5	136452305	136453297	-
8210	1420384_at	Region	Col4a3bp	68018	13	92734071	92828876	+
8211	1455253_at	Region	4930535B03Rik	75137	3	95249480	95306695	-
8212	1426512_at	Region	Olfm3	229759	3	113850919	113895474	+
8213	1434346_at	Region	None	None	10	62970027	62970706	+
8214	1453298_at	Region	Ptpn21	24000	12	94103584	94161513	-
8215	1460619_at	Region	4931419K03Rik	211798	1	41069152	41088661	-
8216	1442645_at	Region	Atp2b3	320707	X	68163741	68231660	+
8217	1440301_at	Region	None	None	11	53291814	53292501	+
8218	1441880_x_at	Region	MGC30332	101883	7	25978772	25980651	+
8219	1440332_at	Region	Cdv3	321022	9	103328549	103340979	-
8220	1420853_at	Region	Sdc3	20970	4	129589463	129623202	+
8221	1440482_at	Region	Vps13a	271564	19	15859308	15865745	-
8222	1441439_at	Region	Ucn3	83428	13	3825536	3826030	-
8223	1429366_at	Region	Lrrc34	71827	3	30034945	30055642	-
8224	1427524_a_at	Region	4930548G07Rik	75339	14	51190396	51219682	+
8225	1428557_a_at	Region	Osgepl1	72085	1	53621177	53631578	+
8226	1416254_a_at	Region	Vps16	80743	2	129938242	129957587	+
8227	1446518_at	Region	Rock1	19877	18	10112811	10226661	-
8228	1441991_at	Region	BC039632	330657	7	121935704	121939069	-
8229	1439301_at	Region	Rad51l1	19363	12	76156299	76675619	+
8230	1439704_at	Region	Hdac2	15182	10	37046787	37075975	+
8231	1422122_at	Region	Fcer2a	14128	8	3029641	3042078	-
8232	1440958_at	Region	None	None	15	81736920	81745417	-
8233	1429664_at	Region	Cdkl1	71091	12	66578318	66622177	-
8234	1420749_a_at	Region	Pou6f1	19009	15	100632856	100643903	-
8235	1452873_at	Region	5830415F09Rik	74753	4	46295973	46308520	-
8236	1430608_at	Region	4930535I16Rik	75220	4	122943098	122945136	-
8237	1451516_at	Region	Rhebl1	69159	15	98935597	98939379	-
8238	1433384_at	Region	9330199C07Rik	77245	14	37072410	37073349	+
8239	1452778_x_at	Region	Nap1l1	53605	10	111105541	111122931	+
8240	1447311_at	Region	Slc6a2	20538	8	92248264	92288701	+
8241	1441024_at	Region	None	None	5	52930353	52931195	+
8242	1419075_s_at	Region	Saa1	20208	7	40825847	40828328	-
8243	1459195_at	Region	None	None	6	40198225	40198593	+
8244	1427149_at	Region	Plekha6	240753	1	133120188	133162992	+
8245	1447987_at	Region	D030041N04Rik	270035	8	24316834	24322553	-
8246	1432950_at	Region	5330421F21Rik	78288	4	86010044	86011838	-
8247	1438083_at	Region	Hhip	15245	8	79185726	79272113	-
8248	1449045_at	Region	Afg3l1	114896	8	122852829	122876449	+
8249	1447935_at	Region	C730036B14Rik	104806	12	61853297	61909946	+
8250	1420661_a_at	Region	4933439F18Rik	66771	11	60142945	60170919	+
8251	1456891_at	Region	A930010I20Rik	329727	3	102555072	102597254	+
8252	1448878_at	Region	Mxd3	17121	13	53934348	53938588	-
8253	1455286_at	Region	Btbd1	83962	7	75600213	75626398	-
8254	1421066_at	Region	Jak2	16452	19	28505054	28564581	+
8255	1451841_a_at	Region	Ncor2	20602	5	124178459	124286279	-
8256	1443203_at	Region	Zfhx1a	21417	18	5709786	5817483	+
8257	1440459_at	Region	None	None	NONE	NONE	NONE	NONE

8258	1449567_at	Region	Tktl1	83553	X	68837939	68869146	+
8259	1438076_at	Region	Rpl30	19946	15	34440221	34442933	-
8260	1435898_x_at	Region	Gdi3	14569	13	3422528	3450201	+
8261	1431169_at	Region	D230012E17Rik	241062	1	54784800	54786464	-
8262	1439053_at	Region	Hnrph3	107938	10	62988787	62989452	+
8263	1458190_at	Region	Arhgap4	171207	X	68555013	68571884	-
8264	1453381_at	Region	C030033M12Rik	77334	7	91737559	91739054	+
8265	1451805_at	Region	Phip	83946	9	83198955	83305205	-
8266	1458958_at	Region	Nope	56741	9	65223736	65260207	+
8267	1421378_s_at	Region	Abcc1	17250	16	13097054	13210369	+
8268	1420253_at	Region	D2Ertd63e	51845	2	167719665	167719911	-
8269	1418759_at	Region	Ptpn20	19256	14	31735571	31773221	+
8270	1430357_at	Region	H3f3b	15081	11	115843386	115845593	-
8271	1420015_s_at	Region	Ppt1	19063	4	121863367	121885494	+
8272	1418416_x_at	Region	Psg23	56868	7	781	5199	+
8273	1439005_x_at	Region	Ywhaz	22631	15	36773662	36797651	-
8274	1459369_at	Region	Epha6	13840	16	58635452	59588354	-
8275	1417302_at	Region	Rcor2	104383	19	6982928	6989070	+
8276	1433634_at	Region	Irf2bp2	270110	8	125876185	125881321	-
8277	1435064_a_at	Region	Tmem27	57394	X	157689592	157718264	+
8278	1428238_at	Region	2700059D21Rik	433693	4	34689700	34705975	+
8279	1419488_at	Region	Tnip2	231130	5	32984434	33002317	-
8280	1424587_at	Region	BC023488	237221	X	159769900	159789915	+
8281	1428651_at	Region	4930429H24Rik	75785	16	18869169	18899359	+
8282	1460359_at	Region	LOC434247	434247	7	122087459	122098021	-
8283	1425282_at	Region	Ibrdc2	218215	13	46720793	46844544	+
8284	1452954_at	Region	Ube2c	68612	2	164226662	164229635	+
8285	1430366_at	Region	5430405H02Rik	74487	2	156319009	156320028	-
8286	1437945_x_at	Region	Nap1l1	53605	10	111105541	111122931	+
8287	1422920_at	Region	Cldn13	57255	5	133927742	133929018	-
8288	1457442_at	Region	AW125324	104600	11	58080996	58081571	+
8289	1428598_at	Region	Tbc1d7	67046	13	42714184	42733799	-
8290	1418173_at	Region	4631426H08Rik	70810	11	99136931	99144028	-
8291	1440355_at	Region	Kctd12b	207474	X	147219550	147230676	-
8292	1427925_at	Region	Stx17	67727	4	48040852	48099039	+
8293	1448096_at	Region	None	None	8	93324976	93325226	+
8294	1445576_at	Region	4930526H21Rik	75136	5	142978696	143027241	+
8295	1439590_at	Region	4931440N07Rik	381560	4	131685803	131693445	-
8296	1416943_at	Region	Ube2e1	22194	14	16806204	16855591	-
8297	1423852_at	Region	Tmem46	219134	14	54160371	54166718	+
8298	1416904_at	Region	Mbnl1	56758	3	60179725	60308221	+
8299	1423480_at	Region	1500002M01Rik	68979	11	106987749	107010349	-
8300	1459202_at	Region	Cecr2	330409	6	121098395	121197332	+
8301	1438728_at	Region	Nif3l1	65102	1	58752501	58767146	+
8302	1423184_at	Region	Sh3d1B	20403	12	3775688	3876446	+
8303	1420221_at	Region	4930461P20Rik	78244	15	10260892	10284727	-
8304	1424772_at	Region	E130307C13	232440	6	137580120	137581870	+
8305	1428784_at	Region	Gmip	78816	8	68958946	68972501	+
8306	1441788_s_at	Region	BC068171	245474	X	69756509	69770234	+
8307	1460542_s_at	Region	Mdm4	17248	1	132851305	132886808	-
8308	1443832_s_at	Region	Sdpr	20324	1	51590070	51603904	+
8309	1423564_a_at	Region	Paics	67054	5	76195870	76211964	+
8310	1416545_at	Region	Zdhhc7	102193	8	119437315	119456866	-
8311	1441607_at	Region	AU015603	103336	10	29372014	29372610	+
8312	1450356_at	Region	Trhr2	170732	8	121738554	121742333	-
8313	1429454_at	Region	Gapvd1	66691	2	34609129	34687394	-
8314	1438560_x_at	Region	Cct4	12464	11	22885383	22898126	+
8315	1452402_at	Region	Uchl3	50933	14	96180426	96270208	+
8316	1455749_x_at	Region	Ndufa7	66416	17	31523009	31536811	+
8317	1441997_at	Region	Zfp184	193452	13	21425158	21440549	+
8318	1440904_at	Region	Senp5	320213	16	30771924	30812542	-
8319	1426022_a_at	Region	Vill	22351	9	119047063	119065587	+
8320	1444437_at	Region	Usp34	17847	11	23201764	23383978	+
8321	1452358_at	Region	Rai2	24004	X	155317032	155378898	+
8322	1445253_at	Region	None	None	19	11014740	11027754	+

8323	1441508_at	Region	Sirt1	93759	10	63283769	63303800	-
8324	1443922_at	Region	None	None	1	191873321	191874037	-
8325	1447748_x_at	Region	Slc29a2	13340	19	4814896	4820754	+
8326	1434390_at	Region	Hnrpu	51810	1	178283700	178291584	-
8327	1430558_at	Region	Zfp318	57908	17	43894944	43929210	+
8328	1416375_at	Region	Ap3m1 /// LOC544716	544716	10	82473353	82475506	+
8329	1433823_at	Region	AW456874	218232	13	48175930	48223631	-
8330	1425129_a_at	Region	Taldo1	21351	7	135794197	135804954	+
8331	1458286_at	Region	0610007P14Rik	58520	12	82685029	82694208	-
8332	1460443_at	Region	Brms1l	52592	12	52556120	52589380	+
8333	1436567_a_at	Region	Ndufa7	66416	17	31523009	31536811	+
8334	1442853_at	Region	None	None	17	22695374	22696427	-
8335	1442969_at	Region	None	None	NONE	NONE	NONE	NONE
8336	1439499_at	Region	AA415398	433752	4	118489213	118497674	-
8337	1416679_at	Region	Abcd3	19299	3	120537803	120594108	-
8338	1437129_at	Region	E330018D03Rik	320319	19	45309292	45312279	-
8339	1418900_at	Region	Ufm1	67890	3	53489432	53497992	-
8340	1426347_at	Region	2010321M09Rik	69882	9	65083138	65108909	+
8341	1454984_at	Region	AW061234	106020	15	6984377	6985054	+
8342	1450051_at	Region	Atrx	22589	X	100401518	100530707	-
8343	1457232_at	Region	Fbxl21	213311	13	55147303	55147813	+
8344	1426888_at	Region	Ehmt2	110147	17	32601358	32617827	+
8345	1452365_at	Region	4732435N03Rik	234356	8	67505844	67884445	-
8346	1416447_at	Region	Tmem30a	69981	9	79974649	79999130	-
8347	1454848_at	Region	Ppp1r12c	232807	7	3712775	3715587	-
8348	1455795_at	Region	Sart2	212898	10	34212320	34268478	-
8349	1449432_a_at	Region	Mell1	27390	4	153361998	153387892	+
8350	1434305_at	Region	U2af1l4	233073	7	25976032	25978056	+
8351	1436337_at	Region	E030031F02Rik	319689	5	9107480	9125785	+
8352	1432367_a_at	Region	Ufd1l	22230	16	17583376	17606305	+
8353	1437565_a_at	Region	Gnl2	230737	4	124057151	124082485	+
8354	1425670_at	Region	Rfxank	19727	8	69285462	69292088	-
8355	1422041_at	Region	Pilrb	170741	5	136802747	136808598	-
8356	1455634_at	Region	Son	20658	16	90805044	90836353	+
8357	1424961_at	Region	Tapbpl	213233	6	125880885	125888540	-
8358	1455951_at	Region	Mars	216443	10	127032863	127048276	-
8359	1417433_at	Region	Lypla2	26394	4	134849482	134853851	-
8360	1447818_x_at	Region	Rhebl1	69159	15	98935597	98939379	-
8361	1438662_at	Region	LOC433810	433810	4	151865583	151878912	-
8362	1416333_at	Region	Dok2	13449	14	65090610	65094723	+
8363	1455611_at	Region	Pias1	56469	9	63002575	63103377	-
8364	1448014_s_at	Region	Usp24	329908	4	105320122	105400231	+
8365	1426860_at	Region	Ep400	75560	5	109714361	109820853	-
8366	1437722_x_at	Region	Pcbp3	59093	10	76863181	76993973	-
8367	1457699_at	Region	E330009J07Rik	243780	6	40549078	40577713	-
8368	1454091_at	Region	Psg21	72242	7	917	1431	-
8369	1441878_s_at	Region	1810049H13Rik	66431	11	120277697	120279156	-
8370	1456107_x_at	Region	MGI:1336880	20624	11	102659566	102702006	-
8371	1439567_at	Region	Tbx3	21386	5	118821012	118834673	+
8372	1455116_at	Region	Tnfrsf19l /// LOC434148	320100	7	94952524	94970071	-
8373	1415677_at	Region	Dhrs1	52585	14	50257036	50263603	-
8374	1427617_at	Region	Fut10	171167	8	29970150	30020391	+
8375	1420487_at	Region	Nol7	70078	13	42960833	42965301	+
8376	1442552_at	Region	Gm1118	382079	9	60306143	60628579	-
8377	1460016_at	Region	AW547186	209497	X	136121461	136278281	+
8378	1447229_x_at	Region	Zfp289	77038	2	90970055	90981680	+
8379	1446657_at	Region	None	None	NONE	NONE	NONE	NONE
8380	1418637_at	Region	Etv3	27049	3	87270684	87284681	+
8381	1426784_at	Region	Trim47	217333	11	115926852	115931301	-
8382	1419355_at	Region	Klf7	93691	1	64328681	64414407	-
8383	1416048_at	Region	Phc2	54383	4	127732109	127779987	+
8384	1459088_at	Region	C79557	97074	3	108318151	108319206	+
8385	1450347_at	Region	Syt10	54526	15	89835266	89894642	-

8386	1451829_a_at	Region	Nf2	18016	11	4662781	4744337	-
8387	1423815_at	Region	Ddx56	52513	11	6152332	6162516	-
8388	1416619_at	Region	4632428N05Rik	74048	10	60305586	60331366	+
8389	1442296_at	Region	Wdr75	73674	1	46099251	46127299	+
8390	1451860_a_at	Region	Trim30	20128	7	98521385	98577468	-
8391	1442239_at	Region	None	None	18	50224256	50224814	+
8392	1445490_at	Region	C77805	97592	NONE	NONE	NONE	NONE
8393	1437586_at	Region	Cnot4	53621	6	35139222	35227761	-
8394	1454827_at	Region	Pogz	229584	3	94341777	94368636	+
8395	1444208_at	Region	E030034C22Rik	218880	14	29452210	29452780	+
8396	1446305_at	Region	LOC432657	432657	12	49495911	49497511	+
8397	1437372_at	Region	Cpsf6	432508	10	117044269	117045158	-
8398	1429733_at	Region	2900016D05Rik	72886	17	53597518	53606625	+
8399	1454137_s_at	Region	Hfe2	69585	3	96013008	96017047	+
8400	1447485_at	Region	Gpr150	238725	13	72111960	72114105	-
8401	1425514_at	Region	Pik3r1	18708	13	97857040	97865195	-
8402	1455129_at	Region	Mtdh	67154	15	34082454	34141052	+
8403	1440047_at	Region	None	None	16	9885498	9886159	+
8404	1449439_at	Region	Klf7	93691	1	64328681	64414407	-
8405	1446150_at	Region	Tm4sf10	192216	X	75731366	75757118	+
8406	1423145_a_at	Region	Tcap	21393	11	98204898	98206299	+
8407	1456715_at	Region	Phr1	105689	14	97658865	97891435	-
8408	1444087_at	Region	Prpf38a	230596	4	107523775	107538232	-
8409	1452845_at	Region	Hif1an	319594	19	44107275	44174420	+
8410	1446955_at	Region	Nr2c2	22026	NONE	NONE	NONE	NONE
8411	1437272_at	Region	4932443J21Rik	320752	9	24452062	24453053	-
8412	1427054_s_at	Region	None	None	16	55472636	55593745	+
8413	1437181_at	Region	Peli2	93834	14	43206712	43342591	+
8414	1423494_at	Region	2310042E22Rik	66561	16	19923731	19925006	-
8415	1431822_a_at	Region	Azi2	27215	9	118036598	118059690	+
8416	1434514_at	Region	Rbm15	229700	3	107121774	107129502	-
8417	1454819_at	Region	None	None	6	54789416	54790165	+
8418	1431187_s_at	Region	Dlg5	71228	14	22497841	22611777	-
8419	1436276_at	Region	AU041707	102247	8	21927525	21962925	-
8420	1418308_at	Region	Hus1	15574	11	8887934	8905929	-
8421	1429820_at	Region	Rutbc2	52850	5	112319917	112387408	-
8422	1450162_at	Region	Dpf3	70127	12	80134474	80234627	-
8423	1436090_at	Region	Enpp6	320981	8	45933793	46041689	+
8424	1452950_at	Region	Mett5d1	76894	2	108716087	108902077	-
8425	1427832_at	Region	Tuba-rs1	26947	17	27988280	27988880	+
8426	1434430_s_at	Region	Adora2b	11541	11	61974626	61992094	+
8427	1442426_at	Region	BC037674	218914	14	32808636	32880366	+
8428	1418503_at	Region	Hspa9a	15526	18	35161089	35177981	-
8429	1429742_at	Region	Chc1l	105670	14	67493623	67535123	+
8430	1460126_at	Region	None	None	11	23241221	23241873	+
8431	1458536_at	Region	Ccni	12453	5	92517687	92541778	-
8432	1420971_at	Region	Ubr1	22222	2	120375495	120484564	-
8433	1430587_at	Region	4933425L03Rik	71169	12	12682551	12943170	+
8434	1451767_at	Region	Ncf1	17969	5	133511650	133520970	-
8435	1457839_at	Region	Dhx40	67487	11	86497746	86535629	-
8436	1422818_at	Region	Nedd9	18003	13	40868522	41047402	-
8437	1446481_at	Region	Apbb2	11787	5	65067237	65384014	-
8438	1449871_at	Region	Tbx18	76365	9	88049059	88076120	-
8439	1426520_at	Region	Btg4	56057	9	51188327	51192026	+
8440	1440003_at	Region	None	None	6	114294027	114294741	+
8441	1457222_at	Region	Creb5	231991	6	53717699	53840157	+
8442	1453199_at	Region	Acbd6	72482	1	155453464	155582573	+
8443	1441387_at	Region	BC030343	269723	5	142330755	142331083	+
8444	1438542_at	Region	None	None	11	69308887	69309357	+
8445	1417319_at	Region	Pvrl3	58998	16	45281022	45382981	-
8446	1440741_at	Region	Htr1d	15552	4	135304781	135325655	+
8447	1444246_at	Region	Chd2	244059	7	67322642	67415792	-
8448	1447315_at	Region	None	None	NONE	NONE	NONE	NONE
8449	1427290_at	Region	Krt2-19	64818	15	101516799	101549911	-
8450	1437822_at	Region	Yme1l1	27377	2	23088708	23130798	+

8451	1439188_at	Region	Cpsf6	432508	10	117042913	117044350	-
8452	1442271_at	Region	4930432B04Rik	75786	2	91305906	91306575	+
8453	1424282_at	Region	Pet112l	229487	3	85317234	85397575	+
8454	1434545_x_at	Region	1110025L05Rik	66162	7	120745731	120746424	+
8455	1426590_at	Region	Gfm2	320806	13	93327341	93365553	+
8456	1421907_at	Region	Pparbp	19014	11	97973241	98014347	-
8457	1423005_a_at	Region	Espn	56226	4	150613239	150644569	-
8458	1418041_at	Region	4432406C05Rik	66690	16	8307024	8310994	-
8459	1429911_at	Region	Mcph1	244329	8	18540173	18748189	+
8460	1432908_at	Region	2810410P21Rik	72731	NONE	NONE	NONE	NONE
8461	1419940_at	Region	C030018P15Rik	109260	12	67720697	67721300	+
8462	1447255_at	Region	2310015A10Rik	69548	NONE	NONE	NONE	NONE
8463	1430198_at	Region	3110009F21Rik	67280	12	105627247	105633498	-
8464	1458926_at	Region	5830415L20Rik	68152	5	3549849	3575816	+
8465	1442254_at	Region	Dgkd	227333	1	87696734	87764305	+
8466	1456450_at	Region	Ctns	83429	11	72908791	72924677	-
8467	1435884_at	Region	Itsn1	16443	16	90939437	91069253	+
8468	1438744_at	Region	Asb7	117589	7	60532010	60573698	-
8469	1428166_at	Region	Cdan1	68968	2	120230371	120245350	-
8470	1456650_at	Region	None	None	1	137776613	137777283	+
8471	1445004_a_at	Region	A330068P14Rik	327958	11	71776765	71784195	-
8472	1416585_at	Region	Ruvbl1	56505	6	88900844	88932944	+
8473	1440851_at	Region	None	None	11	30824574	30825170	-
8474	1459997_s_at	Region	Tmem17	103765	11	22407076	22414024	+
8475	1447028_at	Region	4930563E22Rik	75304	11	71940830	71944102	+
8476	1425658_at	Region	Cd109	235505	9	78815469	78916251	+
8477	1456094_at	Region	Usp36	72344	11	118078029	118111324	-
8478	1431566_at	Region	None	None	2	147401406	147433122	-
8479	1444514_at	Region	Stx5a	56389	19	7938367	7952189	+
8480	1416448_at	Region	Itpa	16434	2	130181694	130195467	+
8481	1427534_at	Region	4930535I16Rik	75220	4	122931341	122944382	-
8482	1439838_a_at	Region	Tmie	20776	9	110907343	110921380	-
8483	1417588_at	Region	Galnt3	14425	2	65938833	65982415	-
8484	1438413_at	Region	Senp7	66315	16	54970744	55086065	+
8485	1434085_at	Region	Zfp523	224656	17	25973336	26001802	+
8486	1428366_at	Region	1600027N09Rik	73247	2	180298180	180303262	+
8487	1430311_at	Region	Marcks	17118	10	37207003	37211096	-
8488	1453712_a_at	Region	Map2k5	23938	9	63284916	63498276	-
8489	1436267_a_at	Region	Frap1	56717	4	146940969	147050045	+
8490	1441906_x_at	Region	Syap1	67043	X	156456257	156487836	-
8491	1439942_at	Region	Prep	19072	10	45184189	45275958	+
8492	1416047_at	Region	Fuca2	66848	10	13382818	13399065	+
8493	1447921_at	Region	None	None	5	142346289	142346458	-
8494	1448225_at	Region	Gpaa1	14731	15	76379484	76383089	+
8495	1420114_s_at	Region	2410022L05Rik	66423	8	13263410	13268885	-
8496	1452032_at	Region	Prkar1a	19084	11	109472038	109490737	+
8497	1459946_at	Region	None	None	1	43046937	43047603	+
8498	1435361_at	Region	AK129018	218877	14	29349698	29362188	+
8499	1456347_at	Region	2010309L07Rik	66485	17	15395468	15395912	-
8500	1439328_at	Region	Nfat5	54446	8	106591549	106677210	+
8501	1455417_at	Region	Kcnj11	16514	7	40182510	40185585	-
8502	1426806_at	Region	5830411E10Rik	109019	1	51769634	51779343	-
8503	1422320_x_at	Region	Phxr5	18690	7	103697887	103699905	-
8504	1455841_s_at	Region	Grwd1	101612	7	39909985	39915506	-
8505	1459866_x_at	Region	Nipa2	93790	7	50201064	50231927	-
8506	1438160_x_at	Region	Slco4a1	108115	2	180177936	180191810	+
8507	1443812_x_at	Region	Cstf2t	83410	19	30334913	30338502	+
8508	1450625_at	Region	Col5a2	12832	1	45679868	45806698	-
8509	1456727_a_at	Region	Csnk1d	104318	11	120784515	120812412	-
8510	1443918_at	Region	2700050L05Rik	214764	7	128005358	128040623	+
8511	1425914_a_at	Region	Armcx1	78248	X	128262928	128266856	+
8512	1453037_at	Region	None	None	17	45204318	45205222	+
8513	1424695_at	Region	2010011I20Rik	67017	2	171854342	171864482	+
8514	1425637_at	Region	6430548M08Rik	234797	8	119500738	119518927	+
8515	1426962_at	Region	6820402O20Rik	228829	2	155653612	155764373	+

8516	1448800_at	Region	Rtn4ip1	170728	10	44011554	44057609	+
8517	1426012_a_at	Region	2610301G19Rik	219158	14	64454157	64470333	-
8518	1458915_at	Region	C77949	97227	5	141422126	141422690	+
8519	1424203_at	Region	Ncln	103425	10	81621714	81631821	-
8520	1421757_at	Region	Htr6	15565	4	137942670	137956050	-
8521	1419437_at	Region	Sim2 /// LOC547289 /// LOC547335	20465	16	93258955	93522296	+
8522	1453713_s_at	Region	4930546H06Rik	75202	17	15694963	15706791	+
8523	1424858_at	Region	L2hgdh	217666	12	66521922	66556288	-
8524	1459848_x_at	Region	None	None	2	27115114	27115362	+
8525	1421515_at	Region	Nr6a1	14536	2	38655532	38859850	-
8526	1424636_at	Region	2610204L23Rik	67163	11	106021555	106037392	-
8527	1459373_at	Region	Epb4.1	269587	4	130721061	130868982	-
8528	1422696_at	Region	Ttyh1	57776	7	3340443	3356202	+
8529	1460327_at	Region	Gpr88	64378	3	115019299	115023427	-
8530	1454558_at	Region	5430416B10Rik	71430	2	113195292	113196381	+
8531	1419743_s_at	Region	Carm1	59035	9	21456863	21476863	+
8532	1436900_x_at	Region	Leprot	230514	4	100606688	100618263	+
8533	1438287_x_at	Region	Ddx39	68278	8	82975115	82983264	+
8534	1417475_at	Region	Atp13a1	170759	8	68941427	68958004	+
8535	1455761_at	Region	2310009B15Rik /// LOC545365	545365	1	138696345	138701165	-
8536	1437864_at	Region	Adipor2	68465	6	119785416	119849749	-
8537	1444917_at	Region	6130401J04Rik	66799	1	16725948	16804454	-
8538	1448901_at	Region	Cpxm1	56264	2	129904628	129911427	-
8539	1428665_at	Region	None	None	12	3935489	3945007	+
8540	1459475_at	Region	D10Ertd761e	52706	10	82101232	82101667	-
8541	1456975_at	Region	Taok1	216965	11	77255718	77311108	-
8542	1453593_at	Region	1700110N18Rik	73569	16	64821348	64869062	+
8543	1444739_at	Region	None	None	6	82966764	82967375	-
8544	1417970_at	Region	Atp5s	68055	12	66556432	66576128	+
8545	1457809_at	Region	Pcaf /// LOC330129	18519	17	51101486	51203822	+
8546	1444886_at	Region	Appbp2	66884	11	84919300	84963112	-
8547	1434711_at	Region	BC030867	217216	11	102069972	102086265	+
8548	1458765_at	Region	Rbm10	236732	X	18858936	18889829	+
8549	1450462_at	Region	Crhr2	12922	6	55234412	55277297	-
8550	1442634_at	Region	None	None	3	68162303	68162933	-
8551	1458328_x_at	Region	3110007P09Rik	73094	4	101719268	101930082	+
8552	1457249_at	Region	Ubap2l	74383	3	89810481	89862883	-
8553	1437269_at	Region	AW124722	239570	15	85880947	85905439	+
8554	1440749_at	Region	1300002E11Rik	71751	16	20663480	20702856	+
8555	1417681_at	Region	Cpsf5	68219	8	93303578	93321193	-
8556	1447499_s_at	Region	Omt2b	382088	9	78512287	78513851	-
8557	1417118_a_at	Region	Ard1	56292	X	68577530	68582568	-
8558	1444611_at	Region	None	None	X	157575331	157575652	+
8559	1443838_x_at	Region	Fads2	56473	19	9260661	9298000	-
8560	1442222_at	Region	Ifnar1	15975	16	90642257	90664445	+
8561	1446447_at	Region	4921537D05Rik	77048	10	94662974	94764320	+
8562	1452294_at	Region	Pcdh1	75599	18	38420416	38433484	-
8563	1435339_at	Region	Kctd15	233107	7	29792806	29806632	-
8564	1435127_a_at	Region	Osgepl1	72085	1	53621177	53631578	+
8565	1458850_at	Region	Myh7	140781	14	49487183	49511559	-
8566	1447511_at	Region	Cacna1b	12287	2	24538537	24695314	-
8567	1457952_at	Region	Cd80	12519	16	37277581	37317238	+
8568	1454873_at	Region	C130032F08Rik	243372	6	48746059	48756106	+
8569	1450432_s_at	Region	Mus81	71711	19	5271641	5277131	-
8570	1429038_at	Region	1500034J01Rik	66498	8	70619056	70625966	+
8571	1436052_at	Region	1700020O03Rik	70373	12	83112070	83161560	+
8572	1456625_at	Region	None	None	9	4202406	4203204	-
8573	1459248_at	Region	Ptpn9	56294	9	57108988	57176293	+
8574	1455118_at	Region	D9Ertd402e	382117	9	122853031	122854106	+
8575	1420570_x_at	Region	Tcl1b3	27378	12	100635179	100639748	+
8576	1446137_at	Region	Gbas	14467	5	128900966	128934118	+

8577	1458820_at	Region	None	None	9	20218990	20219727	-
8578	1421944_a_at	Region	Asgr1	11889	11	69780073	69783544	+
8579	1445624_at	Region	D8Ertd587e	52335	8	109034895	109035353	+
8580	1422782_s_at	Region	Tlr3	142980	8	44343914	44358907	-
8581	1423344_at	Region	Epor	13857	9	21848885	21854434	-
8582	1441240_at	Region	Rpa1	68275	11	75026006	75073962	-
8583	1435508_x_at	Region	0610009D07Rik	66055	12	3985835	3999611	+
8584	1424139_at	Region	Rap1a	109905	3	105524111	105597165	-
8585	1437788_at	Region	Sp6	83395	11	96834656	96845821	+
8586	1456132_x_at	Region	Tgfb1i4	21807	14	70758316	70850256	+
8587	1460176_at	Region	Crk	12928	11	75404968	75431752	+
8588	1444769_at	Region	Tex9	21778	9	72593752	72624808	-
8589	1428833_at	Region	4930406D14Rik	73805	11	86678121	86679892	-
8590	1446653_at	Region	None	None	NONE	NONE	NONE	NONE
8591	1455639_at	Region	Rap2ip	51799	11	102214763	102223640	+
8592	1423699_at	Region	D15Ertd785e	52683	15	89408645	89425699	+
8593	1432383_a_at	Region	4930438O05Rik	78795	1	85961103	86084595	+
8594	1418417_at	Region	Msc	17681	1	14930745	14933365	-
8595	1450568_at	Region	Galr1	14427	18	82561154	82574552	-
8596	1427334_s_at	Region	2810474O19Rik	67246	6	24756910	24759603	+
8597	1430657_at	Region	1810034E14Rik	66503	13	61632956	61652402	+
8598	1429522_at	Region	Ankrd42	73845	7	86648875	86677040	-
8599	1435188_at	Region	LOC433627	433627	3	95365378	95371717	-
8600	1426454_at	Region	Arhgdib	11857	6	137695505	137713513	-
8601	1442278_at	Region	Jarid1b	75605	1	134410458	134483140	+
8602	1425188_s_at	Region	Sel1h	20338	12	87225863	87265702	-
8603	1434048_at	Region	4930471M23Rik	74919	5	29106904	29118671	+
8604	1445606_a_at	Region	2900009J06Rik	72887	1	127596004	127616472	-
8605	1454107_a_at	Region	Kif2a	16563	13	103169548	103230223	-
8606	1455942_at	Region	Fbxl11	225876	19	4106596	4187056	-
8607	1439176_at	Region	None	None	NONE	NONE	NONE	NONE
8608	1419997_at	Region	None	None	3	31985298	31985849	-
8609	1451315_at	Region	2610511E22Rik	76547	11	101973636	101977448	-
8610	1419208_at	Region	Map3k8	26410	18	4336071	4357697	-
8611	1460002_at	Region	Tlk1	228012	2	70410150	70523223	-
8612	1432183_at	Region	C030022K24Rik	77462	5	120615004	120647228	+
8613	1437781_at	Region	None	None	12	52321795	52322476	+
8614	1435823_x_at	Region	Egfl7	353156	2	26513245	26524844	+
8615	1422095_a_at	Region	Tyki	22169	12	23014305	23024800	+
8616	1427525_at	Region	Cd151	12476	7	135869384	135873464	+
8617	1459887_at	Region	Ube4a	140630	9	44921926	44954380	-
8618	1418449_at	Region	Lad1	16763	1	135665574	135680235	+
8619	1458628_at	Region	Limk2	16886	11	3239044	3303961	-
8620	1430539_at	Region	1810057P16Rik	67622	11	116624497	116649139	-
8621	1431234_at	Region	1700041B20Rik	73338	2	126654382	126656482	-
8622	1435408_at	Region	2700092H06Rik	78325	5	149577052	149577849	-
8623	1428586_at	Region	Tmem41b	233724	7	103825350	103839101	-
8624	1431651_at	Region	C330001K17Rik	77609	9	21879781	21892408	-
8625	1444941_at	Region	None	None	5	59644779	59645539	-
8626	1421377_at	Region	Traf6	22034	2	101383123	101405659	+
8627	1443358_at	Region	None	None	14	5732841	5733441	+
8628	1437695_at	Region	Gpr73l1	246313	2	131885018	131899253	-
8629	1433221_at	Region	2610311E24Rik	70469	NONE	NONE	NONE	NONE
8630	1443551_at	Region	Atp2a2	11938	5	121605864	121654245	-
8631	1454311_at	Region	None	None	2	130525264	130526320	+
8632	1436929_x_at	Region	Adcy3	104111	12	3286276	3366296	+
8633	1426513_at	Region	Rbm28	68272	6	29172114	29205297	-
8634	1443966_at	Region	None	None	19	28858795	28859429	+
8635	1459098_at	Region	Dst	13518	1	34280938	34577763	+
8636	1417521_at	Region	Efna2	13637	10	80304628	80314613	+
8637	1437337_x_at	Region	Fuk	234730	8	110180185	110200181	-
8638	1440235_at	Region	Itga10 /// LOC547448	213119	3	96133436	96152349	+
8639	1456273_x_at	Region	Tpmt	22017	13	46623326	46641352	-
8640	1443628_at	Region	Wwp1	107568	4	19537563	19635959	-

8641	1455384_x_at	Region	D030056L22	225995	19	17960188	17965380	+
8642	1440497_at	Region	1300018I05Rik	74157	17	27470422	27513276	+
8643	1448502_at	Region	Slc16a7	20503	10	124921069	125022119	-
8644	1459728_at	Region	5830446M03Rik	57905	6	88255108	88275421	-
8645	1458910_at	Region	Tbrg3	21378	15	82917881	82918219	-
8646	1441835_x_at	Region	Mtmr11	194126	3	95649869	95659546	+
8647	1460447_at	Region	3000003F02Rik	78895	15	94591343	94612193	-
8648	1430237_at	Region	Cldn22	75677	8	46794079	46795074	+
8649	1433127_at	Region	4930513L20Rik	71656	7	101153912	101154816	+
8650	1447011_at	Region	1200009B18Rik	67456	6	149113867	149145859	-
8651	1436448_a_at	Region	Ptgs1	19224	2	36162689	36184424	+
8652	1446811_at	Region	9430023B20Rik	210719	18	6977707	7047463	-
8653	1454195_at	Region	4933433G19Rik	71226	13	63599240	63634827	-
8654	1427698_at	Region	Brca1	12189	11	101309851	101370207	-
8655	1440714_at	Region	---	433569	3	33875269	33882992	-
8656	1442780_at	Region	2610002J02Rik	67513	4	153742328	153749046	+
8657	1444129_at	Region	None	None	13	112753926	112754767	-
8658	1427385_s_at	Region	Actn1	109711	12	77029036	77121075	-
8659	1444114_at	Region	Mkln1	27418	6	31475825	31586464	+
8660	1452495_at	Region	Adam28	13522	14	63136656	63185619	-
8661	1422544_at	Region	Myo10	17909	15	25611655	25800954	+
8662	1439706_at	Region	A330106F07Rik	402724	2	23425090	23425518	-
8663	1457801_at	Region	9930024M15Rik	399602	10	41934632	41935129	+
8664	1451857_a_at	Region	5730593N15Rik	77583	11	120480250	120482908	+
8665	1421254_a_at	Region	Sgcg	24053	14	55756587	55793567	-
8666	1455127_at	Region	5430438H03Rik	319581	8	18877730	18895959	-
8667	1429135_at	Region	1110059M19Rik	68800	X	39611700	39613587	+
8668	1436635_at	Region	D11Moh35	268470	11	95868517	95886448	-
8669	1450564_x_at	Region	Ifna1	15962	4	87836279	87836848	+
8670	1456168_at	Region	Gm877	380694	11	43281564	43346257	+
8671	1437673_at	Region	None	None	14	26663643	26664551	+
8672	1425270_at	Region	Kif1b	16561	4	147668683	147799980	-
8673	1439947_at	Region	Cyp11a1	13070	9	58129644	58141559	+
8674	1433560_at	Region	9330175B01Rik	106068	15	73605805	73674109	-
8675	1443112_at	Region	Api5	11800	2	94116415	94142756	-
8676	1445506_at	Region	None	None	11	77244275	77264082	+
8677	1430588_at	Region	Mro	71263	18	74093614	74112959	+
8678	1431174_at	Region	A930036K24Rik	77829	9	107745633	107747060	+
8679	1447422_at	Region	Garnl1	56784	12	52323288	52540884	-
8680	1423504_at	Region	Jam3	83964	9	26991141	27048370	-
8681	1436191_at	Region	Arid4a	238247	12	67850998	67933600	+
8682	1419820_at	Region	Pkhd1	241035	1	20275361	20840829	-
8683	1457221_at	Region	None	None	NONE	NONE	NONE	NONE
8684	1459393_at	Region	Tcfap2b	21419	1	19426696	19452210	+
8685	1453611_at	Region	Rbbp6	19647	7	116848797	116880431	+
8686	1459588_at	Region	Kit	16590	5	74409260	74490904	+
8687	1421228_at	Region	Ccl7	20306	11	81785373	81787184	+
8688	1433739_at	Region	Gm67	217431	12	16711160	16792762	+
8689	1443361_at	Region	9430079M16Rik	109246	6	128652920	128772541	-
8690	1425075_at	Region	Gatad2b	229542	3	90152072	90168538	+
8691	1452058_a_at	Region	Rnf11	29864	4	108411762	108435410	-
8692	1419918_at	Region	Tmed7	66676	18	46809246	46819270	-
8693	1428199_at	Region	4930578F03Rik	75894	2	120654277	120670529	+
8694	1450351_a_at	Region	Rsn	56430	5	122749321	122826732	-
8695	1444934_at	Region	Zfp276	57247	8	122630839	122644636	+
8696	1428208_at	Region	Bcl7a	77045	5	122516563	122546119	+
8697	1440558_at	Region	Atp9b	50771	18	80858429	81057410	-
8698	1439509_at	Region	2900008C10Rik	72937	X	10373621	10399286	-
8699	1458180_at	Region	None	None	NONE	NONE	NONE	NONE
8700	1438102_at	Region	Senp8	71599	9	59852773	59867496	-
8701	1440433_at	Region	Nmt2	18108	2	3201559	3243640	+
8702	1422087_at	Region	Lmyc1	16918	4	122023211	122029597	+
8703	1421531_at	Region	Akap3	11642	6	127527211	127548304	+
8704	1419915_at	Region	D10Ertd438e	52014	10	52670740	52691207	+
8705	1459417_at	Region	None	None	9	65837174	65837651	-

8706	1458740_at	Region	None	None	1	115798565	115799483	+
8707	1447607_at	Region	None	None	NONE	NONE	NONE	NONE
8708	1454087_at	Region	2810031P15Rik	72701	4	62056769	62225062	+
8709	1441761_at	Region	Tgn	21819	15	66694846	66874812	+
8710	1456667_at	Region	Hdh	15194	5	33249415	33397070	+
8711	1459810_at	Region	1110008F13Rik	67388	2	156320097	156330528	+
8712	1442181_at	Region	None	None	7	13472708	13473664	-
8713	1460069_at	Region	Smc6l1	67241	12	10625170	10671328	+
8714	1449876_at	Region	Prkg1	19091	19	29820457	30038883	-
8715	1427644_at	Region	D0Kist4	27491	NONE	NONE	NONE	NONE
8716	1443911_at	Region	Xpo1	103573	11	23150860	23192321	+
8717	1424031_at	Region	Snx11	74479	11	96588642	96598618	-
8718	1442574_at	Region	None	None	16	90872236	90872802	+
8719	1437366_at	Region	AI608492	103269	10	126787728	126788276	-
8720	1433107_at	Region	Zc3h11a	70579	1	133482838	133516350	-
8721	1459340_at	Region	None	None	NONE	NONE	NONE	NONE
8722	1418249_at	Region	Crcp	12909	5	129199131	129230612	+
8723	1455742_x_at	Region	Morf4l1	21761	9	89979040	90001962	-
8724	1428044_at	Region	Ap3s2	11778	7	73672386	73717617	-
8725	1458458_at	Region	Slfn5	327978	11	82691761	82702600	+
8726	1419680_a_at	Region	Elac2	68626	11	64704747	64727719	+
8727	1446131_at	Region	None	None	9	26996076	26996777	+
8728	1416078_s_at	Region	Raf1	110157	6	116056173	116114081	-
8729	1429703_at	Region	2900072G11Rik	73005	2	14629340	14630990	+
8730	1430413_at	Region	6330540D07Rik	76170	X	143932168	143975533	-
8731	1449198_a_at	Region	St3gal5	20454	6	72429820	72486545	+
8732	1424830_at	Region	Ccnk	12454	12	103650832	103674346	+
8733	1447609_at	Region	5730410E15Rik	319613	15	44706659	44733620	-
8734	1446095_at	Region	Igf2r	16004	17	11321447	11408701	-
8735	1429702_at	Region	2900072G11Rik	73005	2	14629340	14630990	+
8736	1440876_at	Region	Rp9h	55934	9	22338723	22358719	-
8737	1416876_at	Region	Parvg	64099	15	84373319	84391538	+
8738	1442616_at	Region	Grasp	56149	15	101282359	101290906	+
8739	1425085_at	Region	6330416L07Rik	319615	17	20260455	20282030	-
8740	1439391_at	Region	2810002I04Rik	72350	12	82156575	82167280	+
8741	1422237_at	Region	Mc3r	17201	2	171757225	171759847	+
8742	1422534_at	Region	Cyp51	13121	5	4088954	4110454	-
8743	1458078_at	Region	Chd9	109151	8	90276602	90277120	+
8744	1454765_at	Region	LOC227109	227109	1	54704189	54745716	-
8745	1445229_at	Region	Dgat1	13350	15	76552397	76562200	-
8746	1453652_at	Region	4933400F21Rik	74403	1	89495997	89502443	-
8747	1419964_s_at	Region	Hdgf	15191	3	87650025	87659786	+
8748	1436983_at	Region	Crebbp	12914	16	3763367	3779836	-
8749	1456591_x_at	Region	Akap3	11642	6	127527211	127548304	+
8750	1438573_at	Region	4932432N11Rik	74386	13	57034424	57043384	+
8751	1447941_x_at	Region	Braf	109880	6	39749542	39860924	-
8752	1441302_at	Region	LOC433471	433471	2	124638104	124650569	-
8753	1418839_at	Region	Glmn	170823	5	106619473	106668024	-
8754	1419827_s_at	Region	Kif17	16559	4	137143512	137182753	+
8755	1450315_at	Region	V1rd14	81011	7	17631886	17634324	-
8756	1439382_x_at	Region	Ddr1	12305	17	33397888	33421229	-
8757	1439421_x_at	Region	None	None	3	32306908	32307152	+
8758	1445813_at	Region	Wdr27	71682	17	12872815	12987048	-
8759	1456023_at	Region	2210010B09Rik	244721	9	20454212	20467883	+
8760	1457688_at	Region	Zfp398	272347	6	47960121	47992931	+
8761	1431693_a_at	Region	Il17b	56069	18	61916747	61921349	+
8762	1445426_at	Region	Map4k5	399510	12	66635229	66724562	-
8763	1440025_at	Region	Kptn	70394	7	16668	24276	+
8764	1422375_a_at	Region	Art1	11870	7	96207701	96219891	+
8765	1431737_at	Region	None	None	12	36821231	36823697	+
8766	1444645_at	Region	Sertad2	58172	11	20438127	20543916	+
8767	1438733_at	Region	Zfp689	71131	7	121491869	121498852	-
8768	1429833_at	Region	Ly6g6e	70274	17	32780722	32782584	+
8769	1424315_at	Region	1110004E09Rik	68001	16	90082869	90091849	-
8770	1460050_x_at	Region	Klk6	16612	7	38299769	38303949	+

8771	1431762_at	Region	Htra3	78558	5	34139553	34167077	-
8772	1415953_s_at	Region	Mark2	13728	19	6990598	7055161	-
8773	1457638_x_at	Region	Rfc2	19718	5	93150	108790	-
8774	1416652_at	Region	Aspn	66695	13	49144054	49167081	+
8775	1453465_x_at	Region	4930402H24Rik	228602	2	130221684	130353963	-
8776	1446219_at	Region	D930015E06Rik	229473	3	83635417	83777077	-
8777	1433129_at	Region	2900040J22Rik	72956	18	75704661	75705969	-
8778	1456439_x_at	Region	Mical1	171580	10	41578201	41588854	+
8779	1434886_at	Region	D630048P19Rik	403187	7	16097225	16115805	+
8780	1454818_at	Region	Gmeb2	229004	2	180968408	181004923	-
8781	1452886_at	Region	Btbd5	66689	12	61720548	61743644	-
8782	1436633_at	Region	Gm1567	380741	11	120056327	120057401	-
8783	1439080_at	Region	Erbb2ip	59079	13	100037161	100140118	-
8784	1421459_a_at	Region	Lrp8	16975	4	106761166	106833072	+
8785	1428035_at	Region	Fsip1	71313	2	117718736	117770802	-
8786	1438905_x_at	Region	C030046I01Rik	109284	10	80033312	80039553	-
8787	1452262_at	Region	Grpel2	17714	18	61939158	61955132	-
8788	1421196_at	Region	Ptpn11	19247	5	120282348	120343136	-
8789	1445451_at	Region	None	None	15	83623693	83624310	-
8790	1449611_at	Region	Kai1	12521	2	93123794	93167094	-
8791	1417128_at	Region	2810052M02Rik	67220	3	95476660	95483671	-
8792	1427503_at	Region	AI324046	380795	12	108833057	108906536	-
8793	1453389_a_at	Region	MGI:1345171	23921	5	135231641	135258395	-
8794	1426192_at	Region	Smarcd2	83796	11	106084275	106088711	-
8795	1437870_at	Region	Slco4c1	227394	1	96592770	96647174	-
8796	1449818_at	Region	Abcb4	18670	5	8900460	8965969	+
8797	1417057_a_at	Region	Ppid /// Lamp3	239739	16	18424724	18477654	-
8798	1451407_at	Region	Jam4	72058	16	95842156	95884225	+
8799	1433599_at	Region	Baz1a	217578	12	51598034	51690813	-
8800	1420479_a_at	Region	Nap1l1	53605	10	111105541	111122931	+
8801	1443181_at	Region	None	None	NONE	NONE	NONE	NONE
8802	1421458_at	Region	Zfp112	57745	7	19285997	19301733	+
8803	1438951_x_at	Region	Nup54	269113	5	91751291	91770868	-
8804	1431991_at	Region	2410004P03Rik	73667	12	16371877	16420917	-
8805	1453405_at	Region	2610311B01Rik	78390	2	119780144	119782753	-
8806	1454061_at	Region	Thumpd3	14911	6	113609303	113631351	+
8807	1446464_at	Region	Psme4	103554	11	30666852	30774524	+
8808	1435483_x_at	Region	MGI:1917156	69906	15	38993909	39012345	-
8809	1439632_at	Region	Gnb4	14696	3	31991704	32024713	-
8810	1432939_at	Region	None	None	14	112457825	112459678	-
8811	1459794_at	Region	Bcl2l11	12125	2	127639891	127676025	+
8812	1447397_at	Region	1700129I04Rik	330577	7	76489483	76505046	-
8813	1455415_at	Region	A730056A06Rik	319783	7	67200765	67201391	-
8814	1456989_at	Region	None	None	14	114592687	114593086	-
8815	1425565_at	Region	Rest	19712	5	76512186	76527229	+
8816	1422004_at	Region	D730048I06Rik	68171	9	35700509	35702571	-
8817	1452538_at	Region	Igh-4	16017	12	108802515	109842781	-
8818	1417844_at	Region	Vdrip	67381	14	67861484	67869980	+
8819	1441542_at	Region	Drp2	13497	X	127949776	127998338	+
8820	1460597_at	Region	Asxl2	75302	12	1933	29919	+
8821	1426702_at	Region	4632419K20Rik	74349	7	99490967	99512368	-
8822	1440766_at	Region	C77032	30877	14	29144393	29151021	-
8823	1444152_at	Region	Cugbp2	14007	2	6459141	6638885	-
8824	1420485_at	Region	Nol7	70078	13	42960833	42965301	+
8825	1448953_at	Region	Blm	12144	7	74257792	74337688	-
8826	1440728_at	Region	Kcnma1	16531	14	21672901	22164598	-
8827	1432577_at	Region	None	None	13	109055057	109056235	-
8828	1425776_a_at	Region	C87436	232196	6	86827909	86902412	+
8829	1431083_a_at	Region	1810014B01Rik	544717	10	82507736	82511798	-
8830	1432842_s_at	Region	Ywhaq	22630	12	18728420	18754333	+
8831	1444835_at	Region	BC030499	216976	11	109439	110664	+
8832	1456687_at	Region	Arih1	23806	9	59509355	59603044	-
8833	1460074_x_at	Region	Epdr2	105298	13	19073208	19101305	-
8834	1449677_s_at	Region	Tmem38b	52076	4	53741941	53776470	+
8835	1437645_at	Region	Atf7	223922	15	102597062	102685840	-

8836	1420846_at	Region	Mrps2	118451	2	28400228	28403339	+
8837	1439456_x_at	Region	Atp6ap2	70495	X	10826811	10855905	+
8838	1447493_at	Region	A530088H08Rik	193003	11	66637641	66654350	+
8839	1418622_at	Region	Rab2	59021	4	8462790	8534848	+
8840	1419265_at	Region	1200014M14Rik	67463	13	92579692	92606895	+
8841	1438138_a_at	Region	Pex6	224824	17	44222425	44236648	+
8842	1446182_at	Region	Zmynd11	66505	13	9669934	9749340	-
8843	1449223_at	Region	Dnajb8	56691	6	88657640	88658670	+
8844	1430076_at	Region	4930432J16Rik	76872	16	15910017	15918238	-
8845	1456507_at	Region	Zfp454	237758	11	50625563	50640284	-
8846	1426008_a_at	Region	Slc7a2	11988	8	39830084	39853621	+
8847	1457258_at	Region	None	None	5	76712866	76713102	+
8848	1434108_at	Region	Fbxo11	225055	17	85850006	85924399	-
8849	1444217_at	Region	Mrpl38	60441	11	115952914	115959871	-
8850	1437488_at	Region	Slc9a9	331004	9	94575744	95138101	+
8851	1420004_s_at	Region	Pom121	107939	5	134389633	134408002	-
8852	1425419_a_at	Region	Raf1	110157	6	116056173	116114081	-
8853	1417158_at	Region	Zxdc	80292	6	90805539	90839567	+
8854	1438515_at	Region	None	None	11	80126619	80127482	+
8855	1455003_at	Region	Thap1	73754	8	24882962	24887689	+
8856	1459648_at	Region	Rutbc3	105835	15	81030424	81064690	+
8857	1459004_at	Region	Sorl1	20660	9	41927330	42082982	-
8858	1417841_at	Region	Pxmp2	19301	5	109325344	109336789	-
8859	1432293_at	Region	4930579G18Rik	75913	14	49167228	49171458	+
8860	1457537_at	Region	Rab1	19324	11	20096395	20121649	+
8861	1429154_at	Region	Slc35f2	72022	9	53875397	53921607	+
8862	1434534_at	Region	None	None	18	20178509	20180097	-
8863	1443360_x_at	Region	Arhgap4	171207	X	68555013	68571884	-
8864	1453348_at	Region	1700085D22Rik	73512	8	71699213	71700495	+
8865	1441120_at	Region	None	None	7	67546	73851	+
8866	1422886_a_at	Region	Clk4	12750	11	51016012	51034605	+
8867	1447659_x_at	Region	Atp6v1h	108664	1	5048448	5126164	+
8868	1437112_at	Region	Pld1	18805	3	27441540	27550616	+
8869	1439949_at	Region	Gsk3b	56637	16	36906890	37063968	+
8870	1423546_at	Region	Zfp207	22680	11	80109005	80121796	+
8871	1457611_x_at	Region	2010309E21Rik	66488	6	4013569	4015176	+
8872	1445188_at	Region	Gphn	268566	12	75199951	75541337	+
8873	1444469_at	Region	1110059H15Rik	68795	2	69802617	69879409	+
8874	1455264_at	Region	1110035L05Rik	67389	4	154454680	154458991	+
8875	1440748_at	Region	Scarf1	380713	11	75239199	75252230	+
8876	1450776_at	Region	AU041707	102247	8	21927525	21962925	-
8877	1442846_at	Region	Pbx1	18514	1	168054563	168366387	-
8878	1425524_at	Region	2600011C06Rik	67039	12	80506670	80547102	+
8879	1419778_at	Region	None	None	4	103706472	103706861	+
8880	1420939_at	Region	Hs6st2	50786	X	45909852	46201195	-
8881	1446924_at	Region	None	None	19	53513363	53513941	+
8882	1455745_at	Region	None	None	8	14279141	14279990	+
8883	1416602_a_at	Region	Rad52	19365	6	120335139	120355247	+
8884	1430638_at	Region	1700041C02Rik	73332	4	118281798	118374408	-
8885	1429252_at	Region	0610010K14Rik	104457	11	69960866	69963572	-
8886	1448048_at	Region	BC016495	225994	19	17878968	17899143	+
8887	1452015_at	Region	6330416G13Rik	230279	4	62651547	62677546	+
8888	1452010_at	Region	Chrna3	110834	9	55131846	55145722	-
8889	1422781_at	Region	Tlr3	142980	8	44343914	44358907	-
8890	1437338_x_at	Region	Elp3	74195	14	60057876	60120430	-
8891	1418923_at	Region	Slc17a3	105355	13	23319506	23339620	+
8892	1420312_s_at	Region	None	None	NONE	NONE	NONE	NONE
8893	1443025_at	Region	None	None	1	81658113	81658758	+
8894	1447781_s_at	Region	Rg9mtd2	108943	3	137032375	137047205	+
8895	1418541_at	Region	2810429O05Rik	52504	12	3365090	3394126	-
8896	1427463_at	Region	MGI:2384865	213819	6	4553951	4596266	+
8897	1445269_at	Region	Mrps18c	68735	5	99819412	99825120	+
8898	1418959_at	Region	Tmprss5	80893	9	49132999	49147914	+
8899	1456679_at	Region	Obfc1	108689	19	47052348	47088320	-
8900	1451359_at	Region	BC005662	210992	13	69524486	69573696	+

8901	1440032_at	Region	E130008O17Rik	320518	1	128016906	128017591	+
8902	1453025_at	Region	1110033L15Rik	73752	2	139909324	139934342	+
8903	1442538_at	Region	Rcor3	214742	1	191839449	191876966	-
8904	1430138_at	Region	Cd3eap	70333	7	16226093	16228663	-
8905	1443227_at	Region	Bzw2	66912	12	32698936	32763848	-
8906	1458331_x_at	Region	1700122C07Rik	67935	8	92783046	92819879	-
8907	1453998_at	Region	Smap1	98366	1	24090771	24166945	-
8908	1450838_x_at	Region	Rpl37 /// LOC383438	383438	19	15249551	15249820	+
8909	1457435_x_at	Region	Myom2	17930	8	14436135	14512023	+
8910	1421648_at	Region	Nlgn1	192167	3	24847948	25748398	-
8911	1437884_at	Region	Arl8	75869	2	14981634	15005463	+
8912	1422242_at	Region	Defcr-rs10	13219	NONE	NONE	NONE	NONE
8913	1442997_at	Region	Odz3	23965	8	47191599	47638956	-
8914	1459178_at	Region	D8Ertd503e	52366	8	117235428	117236176	+
8915	1434123_at	Region	Fut11	73068	14	19056280	19061509	+
8916	1451487_at	Region	Rabepk	227746	2	34710828	34731953	-
8917	1426672_at	Region	AI604832	102566	9	122196041	122314536	-
8918	1422794_at	Region	Cul3	26554	1	80599109	80655022	-
8919	1457434_s_at	Region	Ptpla	30963	2	13953103	13982307	-
8920	1450993_at	Region	Pask	269224	1	93136928	93165065	-
8921	1424327_at	Region	3200002M19Rik	75430	7	96002510	96005638	+
8922	1441942_x_at	Region	Rnut1	66069	9	57064413	57096688	+
8923	1431409_at	Region	C030015A19Rik	77418	6	90823015	90824268	+
8924	1438735_at	Region	Hbxap	233532	7	91626175	91732691	+
8925	1435818_at	Region	None	None	X	88937780	88938354	+
8926	1419928_at	Region	Lrig1	16206	6	95066417	95161798	-
8927	1430210_at	Region	C330024D12Rik	78444	7	62120928	62133948	-
8928	1444206_at	Region	None	None	NONE	NONE	NONE	NONE
8929	1452208_at	Region	Prdm4	72843	10	85852908	85878083	-
8930	1422819_at	Region	Mrpl36	94066	13	69386508	69387677	+
8931	1459564_at	Region	C87580	97569	NONE	NONE	NONE	NONE
8932	1449517_at	Region	Qpctl	67369	7	16008978	16017957	-
8933	1416886_at	Region	MGI:1927354	57316	11	17152411	17163969	+
8934	1433798_a_at	Region	E330034G19Rik	105418	14	22648374	22677449	+
8935	1429990_at	Region	Hyal4	77042	6	24797831	24817147	+
8936	1449247_at	Region	MGI:1920603	73353	4	153158794	153160095	-
8937	1426638_at	Region	Six3	20473	17	83450633	83453317	+
8938	1459493_at	Region	Setbp1	240427	18	78876677	79209594	-
8939	1418482_at	Region	Cyb561d2	56368	9	107604792	107607646	-
8940	1443288_at	Region	Traf6	22034	2	101383123	101405659	+
8941	1419124_at	Region	2210010L05Rik	98682	1	52961158	53031019	-
8942	1450658_at	Region	Adamts5	23794	16	84962621	85000488	-
8943	1419025_at	Region	Sag	20215	1	87620465	87661728	+
8944	1443953_at	Region	Tex2	21763	11	106323228	106434019	-
8945	1426291_at	Region	1500031M22Rik	66254	13	103155563	103168048	+
8946	1460142_at	Region	D13Ertd324e	52604	13	92173696	92174713	-
8947	1456296_at	Region	5830418K08Rik	319675	9	15154056	15170640	-
8948	1454719_at	Region	5730411O18Rik	279766	11	4994225	5000878	+
8949	1424042_at	Region	Tmem5	216395	10	121740118	121755674	-
8950	1441344_at	Region	Spfh1	226144	19	43579353	43613932	-
8951	1419046_at	Region	Brp16	59053	15	76417980	76420765	+
8952	1456559_at	Region	Emx2os	329078	NONE	NONE	NONE	NONE
8953	1419934_at	Region	Pdrg1	68559	2	152465826	152472319	-
8954	1420638_at	Region	Prps2	110639	X	160945738	160982104	-
8955	1438223_at	Region	None	None	NONE	NONE	NONE	NONE
8956	1430341_at	Region	Nudt5	53893	2	5762306	5786008	+
8957	1422083_at	Region	Tlr9	81897	9	106216193	106220471	+
8958	1453114_at	Region	4632412I24Rik	74035	4	150531696	150553856	+
8959	1445006_at	Region	None	None	13	41565045	41565899	+
8960	1438468_at	Region	Pramel4 /// Pramel5 /// LOC545696	347709	4	142980947	142983963	+
8961	1454117_at	Region	5330439B14Rik	321015	6	143457864	143469731	+
8962	1455163_at	Region	AA407526	231279	5	68325793	68340721	+

8963	1423293_at	Region	Rpa1	68275	11	75026006	75073962	-
8964	1436018_at	Region	None	None	3	88282768	88285258	+
8965	1422729_at	Region	Pcdhb10	93881	18	37635595	37637988	+
8966	1453551_at	Region	Polq	77782	16	35831253	35914864	+
8967	1428292_at	Region	Ndor1	78797	2	25176981	25187540	-
8968	1422363_at	Region	Olfr64	18365	7	98015814	98019893	+
8969	1446205_at	Region	Nfyc	18046	4	119779882	119848111	-
8970	1418554_at	Region	Admr	11536	10	127486332	127488403	-
8971	1451311_a_at	Region	Adipor1	72674	1	134265743	134282600	+
8972	1447416_at	Region	LOC544740	544740	10	116466001	116485583	-
8973	1428098_a_at	Region	Tm7sf3	67623	6	147522941	147555233	-
8974	1442727_at	Region	None	None	NONE	NONE	NONE	NONE
8975	1428337_at	Region	1810034K20Rik	67881	14	50175921	50178429	-
8976	1450498_at	Region	Mthfr	17769	4	146533584	146551911	+
8977	1420449_at	Region	A230048G03Rik	320473	17	76575104	76657599	-
8978	1458214_at	Region	A730012O14Rik	330301	6	47943627	47954860	-
8979	1426080_a_at	Region	Kcnq2	16536	2	180792537	180852183	-
8980	1423719_at	Region	U46068	228801	2	153647755	153677280	+
8981	1446846_at	Region	None	None	11	3215202	3215778	+
8982	1420041_at	Region	None	None	12	51612712	51613144	-
8983	1445024_at	Region	Stard7	99138	2	126784092	126812786	+
8984	1421410_a_at	Region	Pstpip2	19201	18	77916484	78001401	+
8985	1445126_at	Region	Ldb2	16826	5	43229785	43558377	-
8986	1433579_at	Region	Tmem30b	238257	12	70390928	70394209	-
8987	1435939_s_at	Region	AI987662	101202	6	3409611	3447020	-
8988	1427063_at	Region	5330417C22Rik	229722	3	108251436	108332283	-
8989	1452114_s_at	Region	Igfbp5	16011	1	73159719	73176568	-
8990	1460190_at	Region	Ap1m2	11768	9	21181723	21198572	-
8991	1451112_s_at	Region	Dap	223453	15	31222609	31273841	+
8992	1418294_at	Region	Epb4.1l4b	54357	4	57005188	57087097	-
8993	1444409_at	Region	Rph3al	380714	11	75557816	75636684	-
8994	1420545_a_at	Region	Chn1	108699	2	73308494	73358115	-
8995	1454681_at	Region	2210008M09Rik	207920	4	11258898	11311338	-
8996	1457877_at	Region	Esr1	13982	10	5503826	5892641	-
8997	1419408_at	Region	Six6	20476	12	69787131	69792085	+
8998	1419879_s_at	Region	Trim25	217069	11	88820567	88841380	+
8999	1423732_at	Region	Tram1	72265	1	13697903	13722901	-
9000	1416441_at	Region	MGI:1889205	54381	15	33211945	33593530	+
9001	1423790_at	Region	Dap	223453	15	31222609	31273841	+
9002	1428141_at	Region	Gga2	74105	7	115864580	115899025	-
9003	1423670_a_at	Region	Srpr	67398	9	35125247	35131047	+
9004	1453568_at	Region	2310032F03Rik	76747	2	59340053	59360334	+
9005	1449939_s_at	Region	Dlk1	13386	12	104931713	104938825	+
9006	1439427_at	Region	Cldn9	56863	17	21485176	21485829	-
9007	1452770_at	Region	Vkorc1	27973	7	121942873	121945427	-
9008	1439822_at	Region	None	None	2	174259045	174259767	-
9009	1451978_at	Region	Loxl1	16949	9	58402901	58427658	-
9010	1439031_at	Region	Jph4	319984	14	49623897	49633349	-
9011	1445897_s_at	Region	Ifi35	70110	11	101269568	101279549	+
9012	1425041_at	Region	Lhx3	16871	2	26133109	26140411	-
9013	1450482_a_at	Region	Pitx2	18741	3	128131958	128151601	+
9014	1447385_at	Region	None	None	16	9243076	9243485	-
9015	1425603_at	Region	0610011I04Rik	66058	6	48974286	48979812	+
9016	1456552_at	Region	None	None	9	98848567	98849123	-
9017	1417272_at	Region	9130005N14Rik	68303	5	63771941	63843703	+
9018	1441811_x_at	Region	0610011I04Rik	66058	6	48974286	48979812	+
9019	1454760_at	Region	Htatsf1	72459	X	51808329	51821897	+
9020	1427447_a_at	Region	Triobp	110253	15	78999010	79031429	+
9021	1436268_at	Region	Ddn	13199	15	98852014	98866922	-
9022	1415856_at	Region	Emb	13723	13	113672954	113726420	+
9023	1455824_x_at	Region	Itm1	16430	9	36668263	36704803	-
9024	1452953_at	Region	1810036I24Rik	67510	11	62605140	62620834	+
9025	1415857_at	Region	Emb	13723	13	113672954	113726420	+
9026	1424382_at	Region	Rcn3	52377	7	39159455	39168705	-
9027	1452056_s_at	Region	Ppp3ca	19055	3	135555655	135820664	+

9028	1452671_s_at	Region	Lman1	70361	18	66213875	66235740	-
9029	1417460_at	Region	Ifitm2	80876	7	135356826	135357948	-
9030	1450795_at	Region	Lhb	16866	7	39498047	39498872	+
9031	1457077_at	Region	None	None	X	151416131	151417519	-
9032	1429105_at	Region	Dlgap1	224997	17	68195196	68591524	+
9033	1450784_at	Region	Reck	53614	4	43791522	43860442	+
9034	1460419_a_at	Region	Prkcb1	18751	7	116167155	116506625	+
9035	1433701_at	Region	D15Bwg0669e	223726	15	83828622	83907073	+
9036	1422498_at	Region	Mageh1	75625	X	146570562	146571748	-
9037	1419569_a_at	Region	Isg20	57444	7	72705925	72712471	+
9038	1423478_at	Region	Prkcb1	18751	7	116167155	116506625	+
9039	1455936_a_at	Region	Rbpms	19663	8	32589665	32735420	-
9040	1421396_at	Region	Pcsk1	18548	13	71146157	71188465	+
9041	1448848_at	Region	None	None	2	30885134	30891176	+
9042	1455225_at	Region	Syne1	64009	10	4960414	5487942	+
9043	1415828_a_at	Region	D3Ucla1	28146	3	58166332	58170108	-
9044	1434969_at	Region	None	None	10	81592298	81592936	-
9045	1417379_at	Region	Iqgap1	29875	7	74515481	74605994	-
9046	1450841_at	Region	Itm1	16430	9	36668263	36704803	-
9047	1448149_at	Region	Ctnna1	12385	18	35342570	35478433	+
9048	1428624_at	Region	2810482I07Rik	67243	X	100569431	100571394	-
9049	1452178_at	Region	Plec1	18810	15	76219648	76280050	-
9050	1449139_at	Region	2310008M10Rik	66357	3	129626476	129640041	-
9051	1433674_a_at	Region	Rnu22	83673	19	7920687	7922991	+
9052	1418545_at	Region	Wasf1	83767	10	41029716	41047932	+
9053	1423316_at	Region	Tmem39a	67846	16	37385037	37413429	+
9054	1420624_a_at	Region	Vamp8	22320	6	72717197	72722555	-
9055	1433833_at	Region	Fndc3b	72007	3	26833958	27060578	-
9056	1450910_at	Region	Cap2	67252	13	46100466	46248597	+
9057	1416167_at	Region	Prdx4	53381	X	148950069	148964603	-
9058	1416108_a_at	Region	Tmed3	66111	9	89586270	89592056	-
9059	1419978_s_at	Region	D10Ertd610e	52666	10	126915321	126923060	-
9060	1426918_at	Region	Itgb1	16412	8	3634	31325	+
9061	1455458_x_at	Region	Gmppa	69080	1	75726707	75733896	+
9062	1418004_a_at	Region	1810009M01Rik	65963	6	48966591	48973744	-
9063	1449740_s_at	Region	Dsg2	13511	18	20773051	20817758	+
9064	1453014_a_at	Region	Sec31l1	69162	5	99380288	99448544	-
9065	1437086_at	Region	Ascl1	17172	10	87463685	87466044	-
9066	1420867_at	Region	1110032D12Rik	56334	5	123712134	123721846	+
9067	1449063_at	Region	Sec22l1	20333	3	97387503	97408594	+
9068	1416627_at	Region	Spint1	20732	2	118751326	118763044	+
9069	1440980_at	Region	Kcnh8	211468	17	50487209	50488364	-
9070	1417336_a_at	Region	Sytl4	27359	X	127481324	127526722	-
9071	1418355_at	Region	Nucb2	53322	7	110370258	110406443	+
9072	1416326_at	Region	Crip1	12925	12	108628197	108630021	+
9073	1429024_at	Region	1110018J23Rik	73713	19	53411329	53445957	+
9074	1451132_at	Region	Pbxip1	229534	3	89193476	89205693	+
9075	1455085_at	Region	1700086L19Rik	74284	12	71140731	71152721	+
9076	1426393_a_at	Region	Sdf4	20318	4	154485276	154505972	+
9077	1450123_at	Region	Ryr2	20191	13	11561136	11985749	-
9078	1421223_a_at	Region	Anxa4	11746	6	87171158	87227870	-
9079	1459740_s_at	Region	Ucp2	22228	7	94600035	94608691	+
9080	1419091_a_at	Region	Anxa2	12306	9	69585297	69623409	+
9081	1437465_a_at	Region	P4hb	18453	11	120381397	120394029	-
9082	1455074_at	Region	None	None	16	13701828	13703156	+
9083	1428986_at	Region	Slc17a7	72961	7	39246100	39252681	+
9084	1448669_at	Region	Dkk3	50781	7	105969156	106012196	-
9085	1437396_at	Region	Creb3l2	208647	6	37465296	37576712	-
9086	1416440_at	Region	Cd164	53599	10	41621324	41632866	+
9087	1450708_at	Region	Scg2	20254	1	79772940	79774793	-
9088	1423802_at	Region	Camkv	235604	9	108003907	108017678	+
9089	1438194_at	Region	2900019G14Rik	72932	2	102494336	102495534	+
9090	1437119_at	Region	Ern1	78943	11	106218709	106308885	-
9091	1426015_s_at	Region	Asph	65973	4	9376231	9595958	-
9092	1439755_at	Region	Sipa1l1	217692	12	79172130	79311045	+

9093	1416241_at	Region	Sec13l1	110379	6	114296639	114309268	-
9094	1426832_at	Region	6330505F04Rik	236790	X	51209591	51262553	+
9095	1455968_x_at	Region	1110032D12Rik	56334	5	123712134	123721846	+
9096	1428112_at	Region	Armet	74840	9	106954542	106957696	-
9097	1448229_s_at	Region	Ccnd2	12444	6	127802641	127824339	-
9098	1449007_at	Region	Btg3	12228	16	77445330	77462255	-
9099	1439611_at	Region	Chrm1	12669	19	7860376	7878114	+
9100	1423754_at	Region	Ifitm3	66141	7	135411577	135412731	-
9101	1420886_a_at	Region	Xbp1	22433	11	5415754	5420659	+
9102	1429059_s_at	Region	1110004B13Rik	66910	11	68796467	68798950	+
9103	1429069_at	Region	Stub1	56424	17	23636797	23639522	-
9104	1424621_at	Region	AA792894	57896	6	71603943	71617243	+
9105	1418790_at	Region	Zfp312	54713	14	10799639	10803365	-
9106	1451483_s_at	Region	1700054N08Rik	73420	8	123127903	123147281	-
9107	1434507_at	Region	Npepl1	228961	2	173535272	173547609	+
9108	1452428_a_at	Region	B2m	12010	2	121661540	121666935	+
9109	1423396_at	Region	Agt	11606	8	123843659	123851653	-
9110	1423231_at	Region	Nrgn	64011	9	37486032	37494348	-
9111	1439476_at	Region	Dsg2	13511	18	20773051	20817758	+
9112	1422748_at	Region	Zfhx1b	24136	2	44919283	45045178	-
9113	1451449_at	Region	4933407N01Rik	66753	11	30826209	30848913	-
9114	1424094_at	Region	Nek9	217718	12	82167435	82204517	-
9115	1427528_a_at	Region	Epha7	13841	4	28997442	29149372	+
9116	1437783_x_at	Region	Tmem4	56530	10	128059278	128064006	+
9117	1418331_at	Region	1110031I02Rik	66179	11	120998686	121025741	-
9118	1422455_s_at	Region	Nsf	18195	11	103642871	103775145	-
9119	1449679_s_at	Region	Stx5a	56389	19	7938367	7952189	+
9120	1423975_s_at	Region	Numa1	101706	7	96075802	96120917	+
9121	1452197_at	Region	Smc4l1	70099	3	68675494	68705145	+
9122	1453782_at	Region	3021401C12Rik	70678	15	31290975	31293548	-
9123	1431167_at	Region	Dgkg	110197	16	21240316	21402362	-
9124	1456487_at	Region	Adcy1	432530	11	7072012	7073299	+
9125	1447896_s_at	Region	2010109N14Rik	69895	3	122299267	122299409	-
9126	1437760_at	Region	Galnt12	230145	4	47007875	47038964	+
9127	1452411_at	Region	Lrrc1	214345	9	77571439	77639884	-
9128	1455436_at	Region	LOC544932	544932	13	51063134	51091231	-
9129	1426473_at	Region	Dnajc9	108671	14	18745950	18750192	-
9130	1455133_s_at	Region	AI848100	226551	1	161723956	161783029	-
9131	1416556_at	Region	Sas	67125	10	126800009	126802980	-
9132	1419473_a_at	Region	Cck	12424	9	121506265	121512102	-
9133	1455301_at	Region	BQ952480	330319	6	54605656	54648089	+
9134	1424617_at	Region	Ifi35	70110	11	101269568	101279549	+
9135	1423215_at	Region	Spcs2	66624	7	93927764	93947742	-
9136	1416901_at	Region	Npc2	67963	12	81625079	81641589	-
9137	1455620_at	Region	None	None	7	118447725	118448773	+
9138	1452734_at	Region	Rnaset2	68195	17	6479455	6498737	+
9139	1417977_at	Region	Eif4e3	66892	6	100093332	100134966	-
9140	1418271_at	Region	Bhlhb5	59058	3	17384970	17388074	+
9141	1436714_at	Region	Lpp	210126	16	23179019	23766559	+
9142	1435578_s_at	Region	Dab1	13131	4	103326495	103639279	+
9143	1419649_s_at	Region	Myo1c	17913	11	75377236	75400301	+
9144	1436853_a_at	Region	Snca	20617	6	60971674	61069793	-
9145	1421862_a_at	Region	Vamp1	22317	6	125872261	125878983	+
9146	1415766_at	Region	4930564D15Rik	75365	3	97408693	97409564	+
9147	1428393_at	Region	Nrn1	68404	13	36265477	36274425	-
9148	1437393_at	Region	AI875142	103885	11	107754465	107758035	-
9149	1454674_at	Region	Fez1	235180	9	36770087	36817745	+
9150	1449488_at	Region	Pitx1	18740	13	54441612	54447983	-
9151	1436509_at	Region	2410014A08Rik	109154	5	114254717	114267772	-
9152	1457296_at	Region	Cilp	214425	9	65387445	65402870	+
9153	1416830_at	Region	0610031J06Rik	56700	3	88068887	88072495	+
9154	1428380_at	Region	0610007C21Rik	381629	5	29507597	29513561	+
9155	1424708_at	Region	1110014C03Rik	68581	12	82210494	82242351	-
9156	1427450_x_at	Region	Myo1b	17912	1	52050933	52208258	-
9157	1436998_at	Region	Ankrd43	237761	11	53229420	53232920	-

9158	1457446_at	Region	Opcml	330908	9	28570800	28821443	+
9159	1418366_at	Region	Hist2h2aa1 /// Hist1h2ad /// Hist1h2an /// Hist2h2ab /// Hist2h2ac /// Hist2h2aa2	15267	3	95727548	95728125	-
9160	1448569_at	Region	Cd8b1	12526	6	71654715	71669354	+
9161	1416246_a_at	Region	Coro1a	12721	7	120749505	120754485	-
9162	1417579_x_at	Region	Gmppa	69080	1	75726707	75733896	+
9163	1416724_x_at	Region	Tcf4	21413	18	69575005	69915006	+
9164	1449372_at	Region	Dnajc3	19107	14	113495773	113540797	+
9165	1434096_at	Region	Slc4a4	54403	5	88232301	88532904	+
9166	1435895_at	Region	LOC547272	547272	16	5774751	5788961	-
9167	1419514_at	Region	Pitx1	18740	13	54441612	54447983	-
9168	1418300_a_at	Region	Mknk2	17347	10	80797130	80803778	-
9169	1424026_s_at	Region	BC013529	215751	10	7633427	7646395	-
9170	1436444_at	Region	6030405A18	329641	3	54531407	54550223	-
9171	1457311_at	Region	Camk2a	12322	18	61150958	61213470	+
9172	1425458_a_at	Region	Grb10	14783	11	11828255	11865408	-
9173	1438940_x_at	Region	Hmgn1	15312	16	95558992	95565130	-
9174	1452545_a_at	Region	Itgb1	16412	8	3634	31325	+
9175	1434440_at	Region	Gnai1	14677	5	16685204	16687252	-
9176	1449289_a_at	Region	B2m	12010	2	121661540	121666935	+
9177	1416926_at	Region	Trp53inp1	60599	4	11083406	11101342	+
9178	1436493_at	Region	BB181834	381418	2	124661366	124661696	+
9179	1429264_at	Region	C030044B11Rik	68128	13	48566948	48569375	+
9180	1417312_at	Region	Dkk3	50781	7	105969156	106012196	-
9181	1426981_at	Region	Pcsk6	18553	7	59746360	59934610	+
9182	1424329_a_at	Region	Prrg2	65116	7	39130146	39138194	-
9183	1459679_s_at	Region	Myo1b	17912	1	52050933	52208258	-
9184	1426208_x_at	Region	Plagl1	22634	10	12972582	13011561	+
9185	1446953_at	Region	None	None	18	69855808	69856379	+
9186	1456012_x_at	Region	Rnaset2	68195	17	6479455	6498737	+
9187	1421970_a_at	Region	Gria2	14800	3	80417443	80531582	-
9188	1441320_a_at	Region	AI413194	100620	5	136118764	136120238	-
9189	1452050_at	Region	Camk1d	227541	2	5214190	5631787	-
9190	1439066_at	Region	Angpt1	11600	15	42335548	42587605	-
9191	1434935_at	Region	Aak1	269774	6	87436351	87437290	+
9192	1457302_at	Region	Slc20a2	20516	8	21228953	21324191	+
9193	1423854_a_at	Region	Rasl11b	68939	5	73024750	73034009	+
9194	1435678_at	Region	2610017I09Rik	66297	1	42942917	42989541	-
9195	1417921_at	Region	2610029G23Rik	67683	X	99681140	99686551	+
9196	1445202_at	Region	None	None	5	130022101	130022810	+
9197	1429027_at	Region	0610007N19Rik	66835	15	32244217	32279632	-
9198	1441894_s_at	Region	Grasp	56149	15	101282359	101290906	+
9199	1416101_a_at	Region	Hist1h1c	50708	13	23218684	23220243	+
9200	1449233_at	Region	Bhlhb8	17341	5	143231899	143236054	+
9201	1429123_at	Region	Rab27a	11891	9	73177546	73230218	+
9202	1423172_at	Region	Napb	17957	2	148151593	148189356	-
9203	1435663_at	Region	Esr1	13982	10	5503826	5892641	-
9204	1425652_s_at	Region	Rbpms	19663	8	32589665	32735420	-
9205	1459741_x_at	Region	Ucp2	22228	7	94600035	94608691	+
9206	1454942_at	Region	Niban	63913	1	151492935	151576687	+
9207	1432474_a_at	Region	Krtcap3	69815	5	29710654	29712135	+
9208	1449544_a_at	Region	Kcnh2	16511	5	22783486	22815501	-
9209	1449616_s_at	Region	Golga3	269682	5	109227485	109273939	+
9210	1453848_s_at	Region	Zbed3	72114	13	91509451	91522055	+
9211	1423493_a_at	Region	Nfix	18032	8	83974112	84039256	-
9212	1460230_at	Region	Syn2	20965	6	115570293	115710479	+
9213	1418149_at	Region	Chga	12652	12	97999844	98009898	+
9214	1435469_at	Region	Qscn6l1	227638	2	26141292	26169563	-
9215	1453027_at	Region	Dlgap1	224997	17	68195196	68591524	+

9216	1452540_a_at	Region	Hist1h2bc /// Hist1h2be /// Hist1h2bl /// Hist1h2bm /// Hist1h2bp	319179	13	23063618	23101001	-
9217	1453191_at	Region	Col27a1	373864	4	62307017	62424499	+
9218	1440849_at	Region	6330417G04Rik	108780	13	88017031	88018036	-
9219	1426974_at	Region	4632413K17Rik	216440	10	126828369	126853844	-
9220	1450930_at	Region	Hpca	15444	4	128138688	128145871	-
9221	1423746_at	Region	Txndc5	105245	13	38041006	38069191	-
9222	1460594_a_at	Region	Gmppa	69080	1	75726707	75733896	+
9223	1440739_at	Region	Vegfc	22341	8	53042643	53151107	+
9224	1450486_a_at	Region	Oprl1	18389	2	181432000	181437928	+
9225	1450372_a_at	Region	Rpl18	19899	6	129003525	129004136	+
9226	1439143_at	Region	9.33E+12	328399	14	45989622	45990176	-
9227	1441779_at	Region	9530006C21Rik	77393	8	32733763	32734210	-
9228	1438954_x_at	Region	None	None	X	158000068	158000375	+
9229	1427176_s_at	Region	AI428936	233066	7	25727444	25731659	+
9230	1455893_at	Region	2610028F08Rik	239405	15	42929665	43116666	-
9231	1450634_at	Region	Atp6v1a1	11964	16	42970115	43022327	-
9232	1416762_at	Region	S100a10	20194	3	93272307	93281786	+
9233	1439701_at	Region	9330121K16Rik	320227	8	54058787	54059453	+
9234	1436514_at	Region	Gpc4	14735	X	46573716	46686048	-
9235	1443119_at	Region	Grm7	108073	6	112124756	112125436	+
9236	1418619_at	Region	Icam5	15898	9	20914650	20921595	+
9237	1460186_at	Region	MGI:1914262	107358	19	40758164	40808343	-
9238	1422441_x_at	Region	Cdk4	12567	10	126796334	126800114	+
9239	1433428_x_at	Region	Tgm2	21817	2	157573374	157603361	-
9240	1431829_a_at	Region	Rgl3	71746	9	21861231	21879362	-
9241	1455762_at	Region	C330002I19Rik	77480	12	21526422	21612277	+
9242	1435033_at	Region	9330140K16Rik	226970	1	35071064	35082096	+
9243	1455137_at	Region	Rapgef5	217944	12	113453236	113521583	+
9244	1454782_at	Region	Bai3	210933	1	25316949	26075533	-
9245	1416603_at	Region	Rpl22	19934	4	150818238	150826433	+
9246	1416816_at	Region	Nek7	59125	1	138334093	138469075	-
9247	1450138_a_at	Region	Serpinb6a	20719	13	33449537	33467606	-
9248	1429219_at	Region	1200009F10Rik	67454	10	91054397	91073933	+
9249	1433919_at	Region	Asb4	65255	6	5363349	5405778	+
9250	1426162_a_at	Region	Rpl7	19989	1	16284751	16287861	-
9251	1420965_a_at	Region	Enc1	13803	13	93429732	93441676	+
9252	1418579_at	Region	Cetn2	26370	X	67574220	67578999	-
9253	1452217_at	Region	Ahnak	66395	19	8185831	8273459	+
9254	1435043_at	Region	Plcb1	18795	2	134734571	134986275	+
9255	1437161_x_at	Region	Rbpms	19663	8	32589665	32735420	-
9256	1423091_a_at	Region	Gpm6b	14758	X	159838377	159988393	+
9257	1429287_a_at	Region	Prl	19109	13	26537198	26544804	+
9258	1418507_s_at	Region	D130043N08Rik	103250	10	95388033	95392041	-
9259	1456283_at	Region	Neto1	246317	18	86565134	86671455	+
9260	1415987_at	Region	Hdlbp	110611	1	93233431	93306299	-
9261	1434904_at	Region	Hivep2	15273	10	14029087	14051962	+
9262	1425175_at	Region	C1ql3	227580	2	12919466	12927756	-
9263	1418195_at	Region	Galnt10	171212	11	57371071	57513130	+
9264	1437363_at	Region	Homer1	26556	13	89489518	89588558	+
9265	1422495_a_at	Region	Hmgn1	15312	16	95558992	95565130	-
9266	1416836_at	Region	Lrp10	65107	14	48981556	48987703	+
9267	1454757_s_at	Region	D12Ertd647e	52668	12	98879691	98885687	+
9268	1439030_at	Region	Gmppb	331026	9	108117296	108119688	+
9269	1416918_at	Region	Dlgh3	53310	X	95369108	95419769	+
9270	1429359_s_at	Region	Rbpms	19663	8	32589665	32735420	-
9271	1418093_a_at	Region	Egf	13645	3	128610449	128688187	-
9272	1434295_at	Region	Rasgrp1	19419	2	116793841	116856725	-
9273	1431012_a_at	Region	Peci	23986	13	34509925	34526292	-
9274	1426552_a_at	Region	Bcl11a	14025	11	23972907	24067984	+
9275	1456885_at	Region	---	435258	11	53301378	53302447	+
9276	1439750_at	Region	None	None	6	47504699	47505151	+

9277	1435971_at	Region	Rims3	242662	4	119900313	119914004	+
9278	1436141_at	Region	2610510L01Rik	67178	11	4599465	4632453	+
9279	1450533_a_at	Region	Plagl1	22634	10	12972582	13011561	+
9280	1447057_at	Region	None	None	13	89596861	89597390	+
9281	1437060_at	Region	Olfm4	380924	14	74375875	74377189	+
9282	1456478_at	Region	Pgm2l1	70974	7	94334296	94382030	+
9283	1417680_at	Region	Kcna5	16493	6	127204164	127207023	-
9284	1451931_x_at	Region	H2-D1	14964	17	186801	249200	-
9285	1439777_at	Region	B230218O03	329333	2	6456973	6458747	-
9286	1416688_at	Region	Snap91	20616	9	87108242	87224260	-
9287	1418782_at	Region	Rxrg	20183	1	167533144	167574406	+
9288	1424477_at	Region	BC019731	231832	5	138801292	138810582	-
9289	1433992_at	Region	Apxl	110380	X	146144399	146157734	-
9290	1440534_at	Region	6330403A02Rik	381310	1	180390485	180437713	-
9291	1422079_at	Region	Prkch	18755	12	70432855	70625999	+
9292	1417820_at	Region	Tor1b	30934	2	30885164	30890432	+
9293	1435851_at	Region	Lgi1	56839	19	37608585	37652742	+
9294	1416059_at	Region	Sec23b	27054	2	144013213	144047679	+
9295	1456389_at	Region	9130203F04Rik	319891	2	44915677	44918442	-
9296	1442595_at	Region	None	None	7	78434484	78435148	+
9297	1417524_at	Region	Cnih2	12794	19	4881661	4887208	-
9298	1455956_x_at	Region	Ccnd2	12444	6	127802641	127824339	-
9299	1453152_at	Region	Mamdc2	71738	19	22540521	22686490	-
9300	1439333_at	Region	Kcnv1	67498	15	45052998	45061646	-
9301	1455858_x_at	Region	Pomc1	18976	12	3107167	3112834	+
9302	1428118_at	Region	Lrrn6a	235402	9	56731964	56798742	-
9303	1423110_at	Region	Col1a2	12843	6	4458646	4493615	+
9304	1433972_at	Region	Camta1	100072	4	149553741	150114842	-
9305	1427294_a_at	Region	1810073N04Rik	72055	11	119925064	119972367	-
9306	1433986_at	Region	BC024659	108934	13	40807525	40834510	+
9307	1430301_at	Region	Stxbp5	78808	10	9633720	9718785	-
9308	1450683_at	Region	Tagln3	56370	16	44595436	44609155	-
9309	1435083_at	Region	Ctxn	330695	8	3612618	3614168	-
9310	1427470_s_at	Region	Napb	17957	2	148151593	148189356	-
9311	1421739_a_at	Region	Matk	17179	10	81393675	81399111	+
9312	1427436_at	Region	Six2	20472	17	83512185	83516164	-
9313	1418854_at	Region	Birc2	11797	9	1250	8691	+
9314	1416956_at	Region	Kcnab2	16498	4	150883106	150969841	-
9315	1426774_at	Region	Parp12	243771	6	39222967	39254680	-
9316	1415997_at	Region	Txnip	56338	3	96045794	96049702	+
9317	1442927_at	Region	Ptk2b	19229	14	60680682	60808472	-
9318	1450850_at	Region	Vil2	22350	17	6239016	6283286	-
9319	1426255_at	Region	Nefl	18039	14	62612329	62616190	+
9320	1417420_at	Region	Ccnd1	12443	7	139319781	139352397	-
9321	1456684_at	Region	B230382K22Rik	239408	15	43812307	43815641	-
9322	1418995_at	Region	Neurod2	18013	11	98147677	98150732	-
9323	1427045_at	Region	Synpo	104027	18	60821152	60821932	-
9324	1454660_at	Region	1100001E04Rik	75404	X	43991246	44021037	+
9325	1416927_at	Region	Trp53inp1	60599	4	11083406	11101342	+
9326	1449106_at	Region	Gpx3	14778	11	54655791	54663218	+
9327	1427982_s_at	Region	Syne2	319565	12	72696171	72966517	+
9328	1427280_at	Region	Scn2a1	110876	2	65619938	65621824	+
9329	1424235_at	Region	Ormdl2	66844	10	128554992	128558684	-
9330	1430396_at	Region	5730403I07Rik	70487	9	77505709	77539876	-
9331	1416776_at	Region	Crym	12971	7	114061951	114077555	-
9332	1420820_at	Region	2900073G15Rik	67268	17	68765368	68773934	-
9333	1448946_at	Region	Kif3c	16570	12	148	2122	+
9334	1454959_s_at	Region	Gnai1	14677	5	16685204	16687252	-
9335	1421358_at	Region	H2-M3	14991	17	35031325	35034815	+
9336	1433897_at	Region	AI597468	103266	10	85010116	85012295	+
9337	1426938_at	Region	Nova1	18134	12	43448264	43449196	-
9338	1435519_at	Region	Rap1b	215449	10	117505589	117536966	-
9339	1438059_at	Region	None	None	18	57694011	57694710	+
9340	1416543_at	Region	Nfe2l2	18024	2	75373265	75402331	-
9341	1418397_at	Region	Zfp275	27081	X	68003290	68019619	+

9342	1423756_s_at	Region	Igfbp4	16010	11	98862347	98873733	+
9343	1441815_at	Region	AI851453	106953	18	86675269	86675638	+
9344	1439568_at	Region	MGI:2149712	268527	12	16037980	16123596	-
9345	1422692_at	Region	Rpo2tc1	20024	15	11792969	11807128	-
9346	1452304_a_at	Region	Arhgef5	54324	6	43427204	43455561	+
9347	1449799_s_at	Region	Pkp2	67451	16	14981080	15040447	+
9348	1449024_a_at	Region	Hexa	15211	9	59656332	59681866	+
9349	1429518_at	Region	Faim2	72393	15	99556986	99584882	-
9350	1416493_at	Region	Ddost	13200	4	137185999	137193872	+
9351	1449167_at	Region	Epb4.1l4a	13824	18	34019983	34229156	-
9352	1418896_a_at	Region	Rpn2	20014	2	156736067	156783287	+
9353	1423090_x_at	Region	Sec61g	20335	11	16396431	16402946	-
9354	1427095_at	Region	Cdcp1	109332	9	123198536	123233772	-
9355	1423689_a_at	Region	Gpsm1	67839	2	26247702	26280397	+
9356	1422600_at	Region	Rasgrf1	19417	9	89795814	89912965	+
9357	1428379_at	Region	Slc17a6	140919	7	45791145	45840421	+
9358	1442116_at	Region	Gm1012	381413	2	117790958	117887160	-
9359	1428209_at	Region	Bex4	406217	X	129683982	129685380	+
9360	1423035_s_at	Region	Txnl5	52700	11	71933212	71936145	+
9361	1425942_a_at	Region	Gpm6b	14758	X	159838377	159988393	+
9362	1450925_a_at	Region	Rps27l	67941	9	67073432	67076878	+
9363	1455272_at	Region	Grm5	108071	7	81779037	82108756	+
9364	1436087_at	Region	Dpp10	269109	1	123096743	123812573	-
9365	1434725_at	Region	4921521N14Rik	207798	16	42863685	42899689	-
9366	1450388_s_at	Region	Twsg1 /// 1810013J15Rik	65960	17	63632504	63660568	-
9367	1436363_a_at	Region	Nfix	18032	8	83974112	84039256	-
9368	1444767_at	Region	Gnas	14683	2	173709228	173771651	+
9369	1433567_at	Region	Gmps	229363	3	63642160	63687698	+
9370	1437921_x_at	Region	Zfp516	329003	18	83123821	83170925	+
9371	1460456_at	Region	2010316F05Rik	67939	11	29407845	29409823	-
9372	1433800_a_at	Region	Pomc1	18976	12	3107167	3112834	+
9373	1428331_at	Region	2210016F16Rik	70153	13	57013547	57017878	-
9374	1434986_a_at	Region	Sec61a1	53421	6	88938985	88954178	-
9375	1434998_at	Region	Iqgap1	29875	7	74515481	74605994	-
9376	1451310_a_at	Region	Ctsl	13039	13	61752983	61760075	-
9377	1440962_at	Region	Slc8a3	110893	12	78060702	78194436	-
9378	1457092_at	Region	C630007B19Rik	320265	6	96574976	97120216	+
9379	1435038_s_at	Region	Aak1	269774	6	87425235	87425928	+
9380	1449842_at	Region	1810059G22Rik	67706	19	7969076	7970989	-
9381	1436357_at	Region	None	None	11	84598872	84599730	+
9382	1440587_at	Region	None	None	16	42198886	42199577	+
9383	1423186_at	Region	Tiam2	24001	17	3358883	3481083	+
9384	1450520_at	Region	Cacng3	54376	7	116549752	116646824	+
9385	1433985_at	Region	Abi2	329165	1	60713087	60784447	+
9386	1435767_at	Region	Scn3b	235281	9	40219687	40241396	+
9387	1434709_at	Region	C130076O07Rik	319504	12	40985533	41346376	+
9388	1419938_s_at	Region	Arhgef17	207212	7	94978006	95038415	-
9389	1455899_x_at	Region	Socs3	12702	11	117787172	117790255	-
9390	1421881_a_at	Region	Elavl2	15569	4	90225838	90375056	-
9391	1426534_a_at	Region	Arfgap3	66251	15	83348357	83398846	-
9392	1418615_at	Region	Astn1	11899	1	158266071	158595619	+
9393	1423488_at	Region	Mmd	67468	11	90070563	90099660	+
9394	1453915_a_at	Region	Slc37a3	72144	6	39472104	39502599	-
9395	1449099_at	Region	Lrba	80877	3	85969078	86527074	+
9396	1433434_at	Region	AW551984	244810	9	39530212	39546940	-
9397	1437698_at	Region	Myo5b	17919	18	74676774	75005998	+
9398	1436299_at	Region	Gls	14660	1	52466745	52534174	-
9399	1447053_x_at	Region	Ssr3	67437	3	65053089	65065985	-
9400	1441491_at	Region	A330068G13Rik	414087	7	71965300	71965985	-
9401	1435385_at	Region	MGI:2153084	228911	2	169394568	169397236	+
9402	1424354_at	Region	1110007F12Rik	68487	6	34957408	34969208	+
9403	1438305_at	Region	Rims1	116837	1	22518105	22662526	-
9404	1456741_s_at	Region	Gpm6a	234267	8	53936872	54043110	+
9405	1448704_s_at	Region	H47	109815	7	59963883	59973627	+

9406	1454800_at	Region	MGI:2674071	378462	17	78107606	78114868	+
9407	1417267_s_at	Region	Fkbp11	66120	15	98781810	98785637	-
9408	1433788_at	Region	None	None	12	85736951	85737688	+
9409	1439971_at	Region	6330439K17Rik	99097	2	143927477	143928540	-
9410	1449620_s_at	Region	D16Wsu65e	28001	16	3956182	3956737	-
9411	1439833_at	Region	38598	24050	15	82325610	82342778	+
9412	1437643_at	Region	Cenpj	219103	14	51048548	51064812	-
9413	1455340_at	Region	D030011O10Rik	320560	6	149949871	150037421	-
9414	1435894_at	Region	C030014L02	329906	4	101935438	101936351	+
9415	1416344_at	Region	Lamp2	16784	X	32940306	32977217	-
9416	1452285_a_at	Region	Eif3s5	66085	7	102786972	102794245	+
9417	1419146_a_at	Region	Gck	103988	11	5795610	5844386	-
9418	1417538_at	Region	Slc35a1	24060	4	34802324	34826420	-
9419	1441706_at	Region	Dscaml1	114873	9	45443056	45766529	+
9420	1451394_at	Region	Dpp6	13483	5	25258284	26167391	+
9421	1418688_at	Region	Calcr	12311	6	3638256	3717139	-
9422	1451577_at	Region	Zbtb20	56490	16	42442156	42491541	+
9423	1436484_at	Region	C030019I05Rik	320116	11	45988394	45992708	+
9424	1418472_at	Region	Aspa	11484	11	73030646	73050292	-
9425	1435667_at	Region	Rims1	116837	1	22518105	22662526	-
9426	1428270_at	Region	Glt8d1	76485	14	29133655	29143945	+
9427	1426767_at	Region	3230401M21Rik	106618	17	23650932	23667660	-
9428	1448676_at	Region	Camk2b	12323	11	5864459	5960535	-
9429	1423301_at	Region	Copb1	70349	7	108071537	108111448	-
9430	1434403_at	Region	Spred2	114716	11	19819235	19917390	+
9431	1433689_s_at	Region	Rps9	76846	7	14674548	14677359	+
9432	1417868_a_at	Region	Ctsz	64138	2	173852401	173863899	-
9433	1419455_at	Region	Il10rb	16155	16	90564064	90583487	+
9434	1423053_at	Region	Arf4	11843	14	24734402	24753463	+
9435	1423132_a_at	Region	5730427N09Rik /// LOC433230	433230	19	21678941	21680624	+
9436	1423471_at	Region	Ptbp2	56195	3	118493626	118557890	-
9437	1423108_at	Region	Slc25a20	57279	9	108710938	108733881	+
9438	1428094_at	Region	Lamp2	16784	X	32940306	32977217	-
9439	1437485_at	Region	Nos1ap	70729	1	170252649	170555484	-
9440	1422099_a_at	Region	Oprl1	18389	2	181432000	181437928	+
9441	1422948_s_at	Region	Hist1h4h /// Hist1h4c /// Hist1h4i /// Hist1h4j /// Hist1h4k /// Hist1h4m /// Hist1h4a /// Hist1h4b	319155	13	23178015	23178326	-
9442	1433885_at	Region	LOC544963	544963	13	91817361	91882649	-
9443	1429786_a_at	Region	Zwint	52696	10	72709781	72729831	+
9444	1427974_s_at	Region	Cacna1d	12289	14	28175955	28625750	-
9445	1424058_at	Region	1190002C06Rik	73137	18	57571393	57606175	+
9446	1417951_at	Region	Eno3	13808	11	70383454	70388171	+
9447	1426585_s_at	Region	Mapk1	26413	16	15754059	15816626	+
9448	1436694_s_at	Region	Neurod4	11923	10	130003660	130015733	-
9449	1460366_at	Region	Eml3	225898	19	8126241	8138129	+
9450	1416387_at	Region	Pip5k2c	117150	10	126932905	126947384	-
9451	1455785_at	Region	Kcna1	16485	6	127312007	127316405	-
9452	1439048_at	Region	2900078E11Rik	72986	16	65667797	65668756	-
9453	1425285_a_at	Region	Rab27a	11891	9	73177546	73230218	+
9454	1450477_at	Region	Htr2c	15560	X	140402560	140637323	+
9455	1422747_at	Region	Chek2	50883	5	109890347	109924463	+
9456	1455636_at	Region	Lsamp	268890	16	40710888	41011281	+
9457	1423994_at	Region	Kif1b	16561	4	147668683	147799980	-
9458	1428411_at	Region	1700020I14Rik	66602	2	119108145	119117748	+

9459	1416142_at	Region	Rps6 /// LOC214738 /// LOC216036 /// LOC236932 /// LOC434404 /// LOC545640	20104	4	85840854	85843549	-
9460	1456245_x_at	Region	Vamp3	22319	4	149539667	149550315	-
9461	1459717_at	Region	None	None	7	5627536	5627867	+
9462	1419163_s_at	Region	Dnajc3	19107	14	113495773	113540797	+
9463	1454647_at	Region	5730439E10Rik	102632	9	104041180	104105123	+
9464	1452132_at	Region	0610007A15Rik	68385	11	77904425	77906431	+
9465	1460570_at	Region	Pgbd5	209966	8	123657103	123671680	-
9466	1436088_at	Region	0910001A06Rik	223601	15	63945267	64076491	-
9467	1447174_at	Region	Dach1	13134	14	92301812	92643713	-
9468	1427233_at	Region	Sdccag33	110796	18	84180139	84254394	-
9469	1449770_x_at	Region	D16Bwg1494e	224019	16	16047277	16049629	+
9470	1416853_at	Region	Ncdn	26562	4	125770868	125780472	-
9471	1436038_a_at	Region	Dscr5	56176	16	93758663	93765231	-
9472	1428572_at	Region	Basp1	70350	15	25353395	25354075	-
9473	1460458_at	Region	Crispld2	78892	8	119359392	119408065	+
9474	1452202_at	Region	Pde2a	207728	7	95527997	95619103	+
9475	1451411_at	Region	Gprc5b	64297	7	112847904	112871012	-
9476	1423648_at	Region	Pdia6	71853	12	16627969	16646144	+
9477	1425846_a_at	Region	Caln1	140904	5	129622542	130015605	+
9478	1438312_s_at	Region	Ltbp3	16998	19	5530917	5547315	+
9479	1456857_at	Region	1500011B03Rik	66236	NONE	NONE	NONE	NONE
9480	1426416_a_at	Region	Yipf4	67864	17	72297116	72307880	+
9481	1420895_at	Region	Tgfbr1	21812	4	47269227	47330846	+
9482	1437448_s_at	Region	Ctnnd1	12388	2	84298531	84298992	-
9483	1452682_at	Region	4632404H22Rik	78755	X	47764230	47789827	-
9484	1454198_a_at	Region	5430404L10Rik	66793	16	13626255	13644131	+
9485	1459838_s_at	Region	Btbd11	74007	10	85552377	85552631	+
9486	1433575_at	Region	Sox4	20677	13	28430338	28433304	-
9487	1455080_at	Region	Ppp1r16b	228852	2	158122367	158219842	+
9488	1433675_at	Region	Rnu22	83673	19	7920687	7922991	+
9489	1456642_x_at	Region	S100a10	20194	3	93272307	93281786	+
9490	1439904_at	Region	Fstl5	213262	3	75804137	76439608	+
9491	1426442_at	Region	Gpm6a	234267	8	53936872	54043110	+
9492	1433791_at	Region	Rab9b	319642	X	130403090	130413479	-
9493	1448398_s_at	Region	Rpl22	19934	4	150818238	150826433	+
9494	1421093_at	Region	Slc7a10	53896	7	30347870	30362566	+
9495	1451230_a_at	Region	Wbp5	22381	X	129790064	129792067	+
9496	1451674_at	Region	Slc12a5	57138	2	164425052	164456464	+
9497	1425784_a_at	Region	Olfm1	56177	2	28137838	28162624	+
9498	1419157_at	Region	Sox4	20677	13	28430338	28433304	-
9499	1423254_x_at	Region	Rps27l	67941	9	67073432	67076878	+
9500	1435218_at	Region	Rasgef1a	70727	6	118498951	118523137	+
9501	1422662_at	Region	Lgals8	56048	13	12479155	12500727	-
9502	1436579_s_at	Region	AI839402	106263	16	91076823	91077826	+
9503	1448441_at	Region	Cks1b	54124	3	89172201	89175020	-
9504	1437560_at	Region	Ntrk2	18212	13	57448390	57572946	+
9505	1429966_at	Region	3110057O12Rik	269423	3	40321711	40365570	+
9506	1448400_a_at	Region	Smarcd2	83796	11	106084275	106088711	-
9507	1428154_s_at	Region	Ppapdc1	71910	8	24440828	24449305	+
9508	1452872_at	Region	2900054D09Rik	73013	10	70079259	70081585	+
9509	1453421_at	Region	Srr	27364	11	74632454	74651361	-
9510	1435666_at	Region	Mast3	234385	8	69933651	69934783	-
9511	1448551_a_at	Region	Trim2	80890	3	83898180	83960638	-
9512	1451899_a_at	Region	Gtf2ird1	57080	5	87703	186724	-
9513	1438794_x_at	Region	Rps13	68052	7	110187198	110189820	-
9514	1448151_at	Region	Elavl1	15568	8	3643294	3667872	-
9515	1424156_at	Region	Rbl1	19650	2	156602867	156661488	-
9516	1443960_at	Region	Asrgl1	66514	19	8308266	8332113	-
9517	1435558_at	Region	Bai2	230775	4	129019181	129049461	+
9518	1419494_a_at	Region	Tpd52	21985	3	78893581	78895590	-

9519	1455410_at	Region	Faim2	72393	15	99556986	99584882	-
9520	1435056_x_at	Region	Pofut2	80294	10	77370658	77380047	+
9521	1416038_at	Region	AL033314	56463	6	28527393	28935815	+
9522	1450843_a_at	Region	Serpinh1	12406	7	93433937	93441711	-
9523	1433945_at	Region	5730507A09Rik	70638	7	58642774	58657690	-
9524	1451529_at	Region	Sgtb	218544	13	100327976	100359638	+
9525	1425582_a_at	Region	MGI:1891716	59308	3	136226028	136317669	+
9526	1417869_s_at	Region	Ctsz	64138	2	173852401	173863899	-
9527	1416573_at	Region	Pofut2	80294	10	77370658	77380047	+
9528	1417151_a_at	Region	Ntsr2	18217	12	16019829	16026582	+
9529	1449368_at	Region	Dcn	13179	10	97459372	97497709	+
9530	1417111_at	Region	Man1a	17155	10	54152456	54322172	-
9531	1436134_at	Region	Scn2b	72821	9	45118242	45118928	+
9532	1453993_a_at	Region	Bnip2	12175	9	70120357	70139177	+
9533	1454939_at	Region	Phf20l1	239510	15	66601624	66648123	+
9534	1427890_a_at	Region	5730427C23Rik	70552	7	135597131	135612042	+
9535	1428255_at	Region	Luc7l	66978	17	24046121	24075138	+
9536	1452767_at	Region	Rrbp1	81910	2	143442799	143468120	-
9537	1436340_at	Region	9630041G16Rik	100223	4	56900622	56901684	-
9538	1417109_at	Region	Tinagl	94242	4	129192832	129202235	-
9539	1448903_at	Region	MGI:1927947	93684	3	143542154	143569352	+
9540	1439843_at	Region	Camk4	12326	18	33179625	33409958	+
9541	1418153_at	Region	Lama1	16772	17	65465173	65590535	+
9542	1425132_at	Region	Neto1	246317	18	86565134	86671455	+
9543	1423423_at	Region	Pdia3	14827	2	120927809	120951829	+
9544	1417245_at	Region	E130016I23Rik	58245	14	112692638	112719594	+
9545	1437005_a_at	Region	Rpl18	19899	6	129003525	129004136	+
9546	1434947_at	Region	Kif3c	16570	12	148	2122	+
9547	1446357_at	Region	BC020402	407824	10	7544334	7544995	-
9548	1453836_a_at	Region	Mgll	23945	6	89159883	89263412	+
9549	1416127_a_at	Region	Dnpep	13437	1	75598827	75608568	-
9550	1435604_at	Region	Trim37	68729	11	86855069	86948674	+
9551	1455628_at	Region	6430543G08Rik	319794	NONE	NONE	NONE	NONE
9552	1455840_at	Region	Rapgef5	217944	12	113453236	113521583	+
9553	1435204_at	Region	Hrmt1l4	381813	6	128377963	128457962	-
9554	1419184_a_at	Region	Fhl2	14200	1	43417794	43458567	-
9555	1449389_at	Region	Tal1	21349	4	114018432	114030660	+
9556	1422756_at	Region	Slc32a1	22348	2	158068170	158072631	+
9557	1426983_at	Region	Fnbp1	14269	2	30985397	31074101	-
9558	1424806_s_at	Region	1110039B18Rik	68796	5	29328591	29336411	+
9559	1417323_at	Region	5430413I02Rik	56742	3	108179585	108183911	+
9560	1434449_at	Region	Aqp4	11829	18	15581082	15591914	-
9561	1423067_at	Region	Cdk5rap3	80280	11	96728873	96737568	-
9562	1437855_at	Region	Mtap4	17758	9	109957034	110109905	+
9563	1437143_a_at	Region	Txndc1	72736	12	67285315	67299785	+
9564	1439766_x_at	Region	Vegfc	22341	8	53042643	53151107	+
9565	1460408_at	Region	Gabrg1	14405	5	69531953	69623523	-
9566	1436322_a_at	Region	2810001A02Rik	72146	15	86264501	86265270	-
9567	1426914_at	Region	Marveld2	218518	13	96748325	96753403	-
9568	1444286_at	Region	None	None	13	91068262	91069247	+
9569	1426332_a_at	Region	Cldn3	12739	5	133999706	134000964	+
9570	1425619_s_at	Region	Dsg2	13511	18	20773051	20817758	+
9571	1419239_at	Region	Zfp54	22712	17	19292554	19304447	+
9572	1438428_at	Region	Jph1	57339	1	17181923	17282257	-
9573	1417954_at	Region	Sst	20604	16	22673099	22674362	-
9574	1417890_at	Region	Pdxp	57028	15	78965238	78970803	+
9575	1456632_at	Region	Bcl11a	14025	11	23972907	24067984	+
9576	1441963_at	Region	MGI:2656976	241638	2	130146625	130156697	-
9577	1423599_a_at	Region	Pdcl	67466	2	37282236	37291394	-
9578	1450932_s_at	Region	Dock9	105445	14	116113166	116206323	-
9579	1428260_at	Region	Spg3a	73991	12	66724668	66796156	+
9580	1422670_at	Region	Rnd2	11858	11	101289425	101292393	+
9581	1417435_at	Region	Large	16795	8	71965827	72364115	-
9582	1441778_at	Region	Adcyap1	11516	17	91181801	91187870	+
9583	1433932_x_at	Region	C030046I01Rik	109284	10	80033312	80039553	-

9584	1451583_a_at	Region	BC025076	216829	11	62374331	62392004	+
9585	1416034_at	Region	Cd24a	12484	10	43687333	43692412	+
9586	1424852_at	Region	Mef2c	17260	13	79604158	79763912	+
9587	1447837_x_at	Region	None	None	17	43681275	43681498	-
9588	1452952_at	Region	9030418K01Rik	71532	2	178139528	178141387	+
9589	1426973_at	Region	Gpr153	100129	4	150766724	150777699	+
9590	1449002_at	Region	Phlda3	27280	1	135612814	135615815	+
9591	1450055_at	Region	Vsnl1	26950	12	10678693	10740412	-
9592	1426345_at	Region	Prepl	213760	17	82893334	82920056	-
9593	1455358_at	Region	A2bp1	268859	16	6740061	7081334	+
9594	1459019_at	Region	Sprn	212518	7	134549625	134553656	-
9595	1440438_at	Region	Wasf1	83767	10	41029716	41047932	+
9596	1450437_a_at	Region	Ncam1	17967	9	49555319	49607941	-
9597	1457061_at	Region	Glcci1	170772	6	8234651	8573400	+
9598	1423978_at	Region	Sbk1	104175	7	120322350	120344730	+
9599	1448184_at	Region	Fkbp1a	14225	2	150999557	151018627	+
9600	1436443_a_at	Region	Kdelc1	72050	1	44402365	44414592	-
9601	1424069_at	Region	Napg	108123	18	63183763	63228262	+
9602	1456119_at	Region	Grm5	108071	7	81779037	82108756	+
9603	1416698_a_at	Region	Cks1b	54124	3	89172201	89175020	-
9604	1446670_at	Region	Cugbp2	14007	2	6459141	6638885	-
9605	1423126_at	Region	Atp1b3	11933	9	96231661	96263287	-
9606	1428623_at	Region	Plxna1	18844	6	89754497	89798238	-
9607	1423892_at	Region	Apbb1	11785	7	99670922	99693881	-
9608	1441305_at	Region	Nedd4l	83814	18	65256461	65446324	+
9609	1433931_at	Region	C030046I01Rik	109284	10	80033312	80039553	-
9610	1452347_at	Region	Mef2a	17258	7	61121784	61125517	-
9611	1417279_at	Region	Itpr1	16438	6	108714663	109053117	+
9612	1448231_at	Region	Fkbp5	14229	17	26200322	26287578	-
9613	1419118_at	Region	2900093B09Rik	58188	7	35016170	35046860	+
9614	1434763_at	Region	A730041O15Rik	269717	5	135160956	135184148	-
9615	1433934_at	Region	Sec24a	77371	11	51447661	51509426	-
9616	1439462_x_at	Region	1110014C03Rik	68581	12	82210494	82242351	-
9617	1448253_at	Region	Glud1	14661	14	32447805	32482226	+
9618	1434673_at	Region	Gpr22	73010	12	28281559	28288571	-
9619	1454861_at	Region	MGI:105968	109658	4	128653196	128667753	-
9620	1425634_a_at	Region	Tnk1	83813	11	69576757	69584375	-
9621	1455269_a_at	Region	Coro1a	12721	7	120749505	120754485	-
9622	1417788_at	Region	Sncg	20618	14	32506856	32511231	-
9623	1438934_x_at	Region	Sema4a	20351	3	88179823	88199543	-
9624	1435989_x_at	Region	Krt2-8 /// LOC434261	16691	15	102056071	102063643	-
9625	1437341_x_at	Region	Cnp1	12799	11	100396062	100402812	+
9626	1421074_at	Region	Cyp7b1	13123	3	17402749	17573964	-
9627	1448454_at	Region	Sfrs6	67996	2	162388514	162394090	+
9628	1442614_at	Region	Il1rap	16180	16	25368195	25502925	+
9629	1448948_at	Region	Rga	19729	3	89024719	89027033	-
9630	1416617_at	Region	Acas2l	68738	2	150075047	150125165	-
9631	1455758_at	Region	Prkcc	18752	7	3801	13486	-
9632	1436948_a_at	Region	6430550H21Rik	245386	X	32718102	32773193	-
9633	1423405_at	Region	Timp4	110595	6	115681210	115687239	-
9634	1445124_at	Region	A930009L07Rik	402733	15	72533983	72534757	-
9635	1422086_at	Region	Tbx19	83993	1	165060565	165083707	-
9636	1437237_x_at	Region	Zfp110	65020	7	10828742	10844258	+
9637	1424885_at	Region	A630065K24Rik	213417	1	132155437	132164168	+
9638	1424379_at	Region	Car11	12348	7	39783109	39787816	+
9639	1436329_at	Region	Egr3	13655	14	64393373	64396085	+
9640	1454806_at	Region	D12Ertd553e	76820	12	11632753	11732017	+
9641	1436203_a_at	Region	1110059G02Rik	68786	15	55640894	55642196	-
9642	1420191_s_at	Region	D16Bwg1494e	224019	16	16047277	16049629	+
9643	1417012_at	Region	Sdc2	15529	15	32919852	33033850	+
9644	1450070_s_at	Region	Pak1	18479	7	91889708	91958070	+
9645	1432329_a_at	Region	Matk	17179	10	81393675	81399111	+
9646	1437385_at	Region	None	None	18	66289526	66290257	-
9647	1434576_at	Region	Tsga14	83922	6	30701454	30741755	-

9648	1418583_at	Region	Hint3	66847	10	30650948	30661107	-
9649	1439552_at	Region	Trio	223435	15	27735485	27736133	-
9650	1460331_at	Region	Tm9sf2	68059	14	116675627	116727419	+
9651	1424169_at	Region	Tax1bp3	76281	11	72902741	72907704	+
9652	1435128_at	Region	Baiap2	108100	11	119764194	119822580	+
9653	1428064_at	Region	Centd2	69710	7	95491815	95518874	+
9654	1429588_at	Region	2810474O19Rik	67246	6	24764793	24765816	+
9655	1448185_at	Region	Herpud1	64209	8	93670672	93679530	+
9656	1425299_s_at	Region	0610038D11Rik	67674	19	6623999	6624872	+
9657	1427183_at	Region	Efemp1	216616	11	28762563	28821527	+
9658	1438654_x_at	Region	Mmd2	75104	5	141570887	141587725	-
9659	1428050_a_at	Region	0610007H07Rik	68212	10	119786523	119802594	+
9660	1449264_at	Region	Syt11	229521	3	88489566	88516404	-
9661	1438619_x_at	Region	Zdhhc14	224454	17	5400177	5659646	+
9662	1416981_at	Region	Foxo1	56458	3	51900114	51981292	+
9663	1424719_a_at	Region	Mapt	17762	11	104052587	104149347	+
9664	1421176_at	Region	Rasgrp1	19419	2	116793841	116856725	-
9665	1431569_a_at	Region	Lypdc1	72585	1	125707252	125747423	-
9666	1415935_at	Region	Smoc2	64074	17	12330114	12456677	+
9667	1433759_at	Region	Dpy19l1	244745	9	24306726	24318597	-
9668	1419725_at	Region	Slc26a4	23985	12	28095023	28135173	-
9669	1425585_at	Region	Tnrc11	59024	X	95876255	95898632	+
9670	1418886_s_at	Region	Idh3b	170718	2	129793165	129798290	-
9671	1436238_at	Region	Lgi3	213469	14	64846859	64854311	+
9672	1424670_s_at	Region	Zfyve21	68520	12	107295781	107308852	+
9673	1421891_at	Region	St3gal2	20444	8	110217644	110269279	+
9674	1436818_a_at	Region	Msi2h	76626	11	88067452	88208048	-
9675	1438916_x_at	Region	6720401G13Rik	103012	X	45083921	45155862	-
9676	1434062_at	Region	Rabgap1l	29809	1	160161852	160697585	-
9677	1436426_at	Region	5730509K17Rik	231214	5	42419495	42498023	+
9678	1416933_at	Region	Por	18984	5	134702706	134748817	+
9679	1440286_at	Region	Fbxl16	214931	17	23622414	23627423	+
9680	1418756_at	Region	Trh	22044	6	92691219	92693046	-
9681	1439486_at	Region	Kcnt1	227632	2	25843419	25848759	+
9682	1460203_at	Region	Itpr1	16438	6	108714663	109053117	+
9683	1448357_at	Region	Snrpg	68011	6	86805446	86812808	+
9684	1440859_at	Region	Akap6	238161	12	49603505	49902998	+
9685	1448353_x_at	Region	Rpn1	103963	6	88518156	88540499	+
9686	1444139_at	Region	Ddit4l	73284	3	136513537	136518197	+
9687	1426209_at	Region	Strn4	97387	7	13766849	13791927	+
9688	1428660_s_at	Region	Tor3a	30935	1	156560153	156579875	-
9689	1435123_at	Region	AI852640	380744	12	3114770	3191118	-
9690	1453248_at	Region	2810039B14Rik	72665	15	75692198	75697504	+
9691	1420688_a_at	Region	Sgce	20392	6	4627235	4699984	-
9692	1452714_at	Region	1200003E16Rik	66860	2	59467444	59701549	+
9693	1437223_s_at	Region	Xbp1	22433	11	5415754	5420659	+
9694	1448703_at	Region	Lsm8	76522	6	18898032	18903464	+
9695	1438761_a_at	Region	Odc1 /// LOC545783 /// LOC546355	18263	12	16906701	16912688	+
9696	1434248_at	Region	Prkch	18755	12	70432855	70625999	+
9697	1428218_a_at	Region	1600012H06Rik	67912	17	12995900	12998652	+
9698	1424223_at	Region	1700020C11Rik	67900	11	3986267	3990192	-
9699	1429185_at	Region	8430436L14Rik	71560	3	62125408	62129257	+
9700	1438656_x_at	Region	Timm17b	21855	X	6138338	6146592	+
9701	1429162_at	Region	1500015A07Rik	68982	18	61955202	61956437	+
9702	1448474_at	Region	Nek7	59125	1	138334093	138469075	-
9703	1439573_at	Region	Rtn4rl2	269295	2	84569695	84584441	-
9704	1426221_at	Region	Loh11cr2a	67776	9	38660904	38685943	+
9705	1425870_a_at	Region	Kcnip2	80906	19	45341106	45363239	-
9706	1429022_at	Region	Adcyap1r1	11517	6	55596313	55643230	+
9707	1460700_at	Region	Stat3	20848	11	100709309	100760491	-
9708	1428875_at	Region	Golph4	73124	3	75606017	75686516	-
9709	1418498_at	Region	Fgf13	14168	X	53816864	53889150	-
9710	1418902_at	Region	3110023E09Rik	68038	7	135895916	135942583	-

9711	1449267_at	Region	3110023E09Rik	68038	7	135895916	135942583	-
9712	1417709_at	Region	Cyp46a1	13116	12	103805370	103833223	+
9713	1427277_at	Region	Six1	20471	12	69888891	69893525	-
9714	1435197_at	Region	Pou3f3	18993	1	42991862	42993355	+
9715	1455145_at	Region	None	None	X	127127811	127129566	-
9716	1452380_at	Region	Epha7	13841	4	28997442	29149372	+
9717	1416937_at	Region	Gabarap	56486	11	69717028	69720604	+
9718	1438665_at	Region	Smpd3	58994	8	105548395	105633835	-
9719	1417505_s_at	Region	Il11ra1 /// Il11ra2	16157	4	41901051	41908166	+
9720	1417747_at	Region	Cplx1	12889	5	107594772	107626145	-
9721	1424437_s_at	Region	Abcg4	192663	9	44262045	44277099	-
9722	1454886_x_at	Region	None	None	12	67076694	67077367	-
9723	1426339_at	Region	Ak5	229949	3	151445720	151481554	-
9724	1417829_a_at	Region	Rab15	104886	12	73654549	73681495	-
9725	1429064_at	Region	2900024P20Rik	208440	13	9260573	9652519	+
9726	1435593_x_at	Region	Rps7	20115	12	25196338	25201165	-
9727	1437360_at	Region	Pcdh19	279653	X	127132549	127233781	-
9728	1429021_at	Region	Epha4	13838	1	77656420	77802175	-
9729	1451257_at	Region	Acsl6	216739	11	54057075	54114371	+
9730	1422563_at	Region	Crry	12946	1	194844160	194871942	-
9731	1435626_a_at	Region	Herpud1	64209	8	93670672	93679530	+
9732	1455315_at	Region	2410019G02Rik	216964	11	77233764	77238942	-
9733	1418360_at	Region	Zfp179	22671	11	61174084	61179528	-
9734	1460181_at	Region	Stmn3	20262	2	181023416	181031457	-
9735	1419421_at	Region	Ank1	11733	8	21812854	21905075	+
9736	1438134_at	Region	Pcdh10	18526	3	44837486	44841910	+
9737	1446273_at	Region	Csmd1	94109	8	15273983	16089202	-
9738	1426903_at	Region	Fndc3a	319448	14	66891442	67062343	-
9739	1429261_at	Region	2210411K11Rik	75685	7	4030762	4035755	-
9740	1451566_at	Region	BC005471	235050	9	22167374	22198034	-
9741	1433577_at	Region	A730017C20Rik	225583	18	59287736	59302184	+
9742	1436959_x_at	Region	Nelf	56876	2	24986575	24995042	+
9743	1435835_at	Region	LOC380718	380718	11	87556734	87591791	+
9744	1436073_at	Region	A630007B06Rik	213993	19	56376334	56399260	-
9745	1448459_at	Region	Kcnip1	70357	11	33524574	33738321	-
9746	1460409_at	Region	Cpt1a	12894	19	3137883	3174426	+
9747	1416124_at	Region	Ccnd2	12444	6	127802641	127824339	-
9748	1418100_at	Region	A030009H04Rik	80515	11	69066475	69068299	+
9749	1436659_at	Region	Dcamkl1	13175	3	54876964	55171014	+
9750	1428323_at	Region	Gpd2	14571	2	57163836	57293151	+
9751	1417050_at	Region	C1qtnf4	67445	2	90590607	90595268	+
9752	1451755_a_at	Region	Apobec1	11810	6	123242773	123267425	-
9753	1421883_at	Region	Elavl2	15569	4	90225838	90375056	-
9754	1454808_at	Region	Efha1	68514	14	52451152	52534134	-
9755	1420827_a_at	Region	Ccng1	12450	11	40489722	40494170	-
9756	1448236_at	Region	Rdx	19684	9	52120601	52161012	+
9757	1426219_at	Region	Scp2	20280	4	107002744	107077406	-
9758	1428964_at	Region	Slc25a18	71803	6	121205578	121226141	+
9759	1426283_at	Region	MGI:2446259	235106	9	28892331	29088826	-
9760	1436744_x_at	Region	Fbxw14	50757	9	109317856	109334407	-
9761	1419642_at	Region	Purb	19291	11	6369560	6370704	-
9762	1455871_s_at	Region	Tax1bp3 /// Rpl13	270106	8	122483697	122486521	+
9763	1433469_at	Region	Lrrn2	16980	1	132741815	132801419	+
9764	1435543_at	Region	Apc	11789	18	34484045	34541630	+
9765	1434851_s_at	Region	Crb3	224912	17	54752222	54755863	+
9766	1429104_at	Region	0610025L06Rik	67803	11	105977345	105981231	-
9767	1423213_at	Region	Plxnc1	54712	10	94767461	94918749	-
9768	1417706_at	Region	Naglu	27419	11	100891212	100898739	+
9769	1433757_a_at	Region	Nisch	64652	14	29302768	29323388	-
9770	1450945_at	Region	Prkca	18750	11	107759337	108165017	-
9771	1448720_at	Region	Lrrc40	67144	3	157046099	157077874	+
9772	1426341_at	Region	Slc1a3	20512	15	8424368	8500974	-
9773	1434027_at	Region	Dscr1l2	53902	4	134293565	134306678	-
9774	1444077_at	Region	None	None	NONE	NONE	NONE	NONE
9775	1417419_at	Region	Ccnd1	12443	7	139319781	139352397	-

9776	1437071_at	Region	Eif1ay	66235	X	152971635	152987226	+
9777	1419137_at	Region	Shank3	58234	15	89554156	89613132	+
9778	1457030_at	Region	Mirg	373070	12	105220796	105226448	+
9779	1448242_at	Region	Sec61a1	53421	6	88938985	88954178	-
9780	1449505_at	Region	Kpna1	16646	16	34764772	34817569	+
9781	1431951_a_at	Region	Usp16	74112	16	86604508	86632967	+
9782	1426486_at	Region	Ubxd2	67812	1	128087153	128120583	+
9783	1451259_at	Region	Smfn	104444	9	48485885	48497982	-
9784	1454837_at	Region	Cln6	76524	9	62961291	62974504	+
9785	1420500_at	Region	Dnajc1	13418	2	18259145	18434517	-
9786	1433507_a_at	Region	Hmgn2	15331	4	132925640	132928805	-
9787	1437744_at	Region	Slitrk4	245446	X	58938040	58945592	-
9788	1434685_at	Region	D3Bwg0562e	229791	3	116093000	116134735	-
9789	1417663_a_at	Region	Ndrg3	29812	2	156384314	156427107	-
9790	1453196_a_at	Region	Oasl2	23962	5	114006560	114021853	+
9791	1456397_at	Region	Cdh4	12561	2	179497210	179613363	+
9792	1416220_at	Region	Spcs1	69019	14	29131779	29133314	-
9793	1425533_a_at	Region	Stau2	29819	1	16527085	16558282	-
9794	1454677_at	Region	Timp2	21858	11	118122157	118176401	-
9795	1423119_at	Region	Rshl2	66832	17	6405302	6448903	-
9796	1427921_s_at	Region	2310061C15Rik	66531	8	116217815	116249733	-
9797	1451783_a_at	Region	Kifap3	16579	1	163697221	163834642	+
9798	1428637_at	Region	Dyrk2	69181	10	118436994	118438441	-
9799	1425480_at	Region	Cnot6l	231464	5	95087917	95179605	-
9800	1428253_at	Region	Chmp2b	68942	16	64543222	64566897	-
9801	1418933_at	Region	Slc1a6	20513	10	78895476	78930109	+
9802	1436526_at	Region	Gnptg	214505	17	23040168	23045967	-
9803	1429053_at	Region	1110012J17Rik	68617	17	64055405	64103991	-
9804	1437052_s_at	Region	Slc2a3	20527	6	123394798	123409491	-
9805	1449435_at	Region	B4galt3	57370	1	171199227	171205734	+
9806	1435522_a_at	Region	2310016E02Rik	67695	5	29365462	29366669	-
9807	1416721_s_at	Region	Sfrs6	67996	2	162388514	162394090	+
9808	1416749_at	Region	Prss11	56213	7	125247906	125297330	+
9809	1450907_at	Region	Spcs2	66624	7	93927764	93947742	-
9810	1431056_a_at	Region	Lpl	16956	8	68029951	68056194	+
9811	1450193_at	Region	Hcn1	15165	13	114055153	114429899	+
9812	1416091_at	Region	Mtap4	17758	9	109957034	110109905	+
9813	1452956_a_at	Region	D12Ertd647e	52668	12	98879691	98885687	+
9814	1448552_s_at	Region	2310028N02Rik	66950	1	191062230	191089190	+
9815	1459695_at	Region	None	None	16	41902852	41903163	+
9816	1452071_at	Region	Slc4a4	54403	5	88232301	88532904	+
9817	1427296_at	Region	BC010304	218236	13	48477714	48566441	-
9818	1436226_at	Region	Tceb1	67923	1	16827250	16842139	-
9819	1433662_s_at	Region	Timp2	21858	11	118122157	118176401	-
9820	1460257_a_at	Region	Mthfs	107885	9	89099733	89128768	+
9821	1436513_at	Region	3526402J09Rik	237960	11	105744223	105745391	+
9822	1460626_at	Region	38606	52398	5	92429207	92510708	+
9823	1460592_at	Region	Epb4.1l1	13821	2	155877989	155997768	+
9824	1436180_at	Region	Dnajc5	13002	2	181237462	181269206	+
9825	1423973_a_at	Region	Arf3	11842	15	98795066	98820699	-
9826	1435826_at	Region	Rad18	58186	6	113184164	113259954	-
9827	1427416_x_at	Region	Dusp7	235584	9	106362584	106369022	+
9828	1424986_s_at	Region	Fbxw7	50754	3	84695316	84720739	+
9829	1419062_at	Region	Epb4.1l3	13823	17	66934864	67065569	+
9830	1422490_at	Region	Bnip2	12175	9	70120357	70139177	+
9831	1452711_at	Region	Spg3a	73991	12	66724668	66796156	+
9832	1450391_a_at	Region	Mgll	23945	6	89159883	89263412	+
9833	1421889_a_at	Region	Aplp2	11804	9	31068390	31130767	-
9834	1452657_at	Region	Ap1s2	108012	X	157508565	157528951	+
9835	1452248_at	Region	Plekhg5	269608	4	150589081	150607762	+
9836	1458324_x_at	Region	None	None	6	93697593	93697780	+
9837	1427286_at	Region	D11Bwg0517e	52897	11	118315243	118582135	-
9838	1434446_at	Region	D630014A15Rik	319666	8	13158142	13211994	+
9839	1423233_at	Region	Cebpd	12609	16	14655589	14657307	+
9840	1455875_x_at	Region	Tm9sf2	68059	14	116675627	116727419	+

9841	1417391_a_at	Region	Il16	16170	7	77499493	77591920	-
9842	1415942_at	Region	Rpl10 /// LOC234703	110954	X	68931512	68933781	+
9843	1427878_at	Region	0610010O12Rik	66060	18	36566148	36591920	+
9844	1445328_at	Region	E130010M05Rik	319847	1	82777551	82781741	-
9845	1433868_at	Region	None	None	2	137800261	137801278	+
9846	1436827_at	Region	Gm944	381126	18	21343840	21521608	-
9847	1451679_at	Region	6530401D17Rik	76219	X	129538762	129579883	+
9848	1441382_at	Region	None	None	X	52251384	52252096	-
9849	1429759_at	Region	Rps6ka6	67071	X	105788074	105862607	-
9850	1450069_a_at	Region	Cugbp2	14007	2	6459141	6638885	-
9851	1425336_x_at	Region	H2-K1	14972	17	32967247	32971632	+
9852	1456085_x_at	Region	Cd151	12476	7	135869384	135873464	+
9853	1435511_at	Region	Syn2	20965	6	115570293	115710479	+
9854	1455754_at	Region	Lmo3	109593	6	139156439	139373926	-
9855	1417116_at	Region	Slc6a8	102857	X	68333805	68343157	+
9856	1435190_at	Region	Chl1	12661	6	103991578	104209457	+
9857	1439398_x_at	Region	Nelf	56876	2	24986575	24995042	+
9858	1424367_a_at	Region	Homer2	26557	7	75417854	75529582	-
9859	1423428_at	Region	Ror2	26564	13	51672830	51844724	-
9860	1428507_at	Region	Hdhd2	76987	18	77065841	77093117	+
9861	1435326_at	Region	AW112037	98667	1	191515786	191519464	+
9862	1437861_s_at	Region	None	None	17	84488716	84490164	+
9863	1459338_at	Region	Auts2	319974	5	130627794	130728707	-
9864	1428203_at	Region	C030002O17Rik	78533	11	42384313	42385400	+
9865	1436495_s_at	Region	Zfp260	26466	7	25509675	25522233	+
9866	1454852_at	Region	Sp1	20683	15	102466747	102493098	+
9867	1439957_at	Region	AI843190	106944	18	67455638	67456124	+
9868	1448954_at	Region	Nrip3	78593	7	103610927	103634416	-
9869	1420641_a_at	Region	Sqrdl	59010	2	122295852	122323406	+
9870	1449307_at	Region	2810427I04Rik	72185	8	122879960	122889622	-
9871	1453187_at	Region	Ociad2	433904	5	72107568	72123547	-
9872	1439381_x_at	Region	Marveld1	277010	19	41691767	41696030	+
9873	1416181_at	Region	Mesdc2	67943	7	77748975	77758250	+
9874	1449474_a_at	Region	Nelf	56876	2	24986575	24995042	+
9875	1446148_x_at	Region	C79248	96982	2	155626395	155627019	-
9876	1448846_a_at	Region	Rpl29 /// LOC240367 /// LOC433350 /// LOC433383 /// LOC433706 /// LOC433941	19944	9	106423093	106425121	+
9877	1455479_a_at	Region	Ube2d3	66105	3	134321860	134350243	+
9878	1456373_x_at	Region	Rps20	67427	4	3761619	3762746	-
9879	1437472_at	Region	Unc13a	382018	8	70776389	70778131	-
9880	1427560_at	Region	Six5	20475	7	15963564	15967107	+
9881	1427165_at	Region	Il13ra1	16164	X	30732693	30791816	+
9882	1428371_at	Region	2610507N02Rik	74210	2	120246668	120250019	-
9883	1417296_at	Region	Atf1	11908	15	100285451	100318782	+
9884	1422907_at	Region	Gnat2	14686	3	107888810	107897028	+
9885	1428268_at	Region	Psd2	74002	18	36188770	36238339	+
9886	1418899_at	Region	Ufm1	67890	3	53489432	53497992	-
9887	1428867_at	Region	4933417E01Rik	74463	7	16358300	16366004	+
9888	1460696_at	Region	BC026585	226527	1	157359757	157394630	+
9889	1434265_s_at	Region	Ank2	109676	3	125805551	125881703	-
9890	1429002_at	Region	Skiip	66354	12	84536947	84547086	+
9891	1422644_at	Region	Sh3bgr	50795	16	95636972	95655833	+
9892	1451782_a_at	Region	Slc29a1	63959	17	43096564	43103735	-
9893	1429062_at	Region	Kif16b	16558	2	142170469	142397720	-
9894	1450626_at	Region	Manba	110173	3	134368802	134454538	+
9895	1448182_a_at	Region	Cd24a	12484	10	43687333	43692412	+
9896	1428186_at	Region	Kctd6	71393	14	5880987	5890506	+
9897	1431076_at	Region	2900072M03Rik	72970	6	86552074	86553985	+
9898	1421840_at	Region	Abca1	11303	4	52946667	53075771	-
9899	1434172_at	Region	Cnr1	12801	4	34203466	34224064	+

9900	1451255_at	Region	MGI:1927471	54135	7	26365183	26380612	-
9901	1425534_at	Region	Stau2	29819	1	16527085	16558282	-
9902	1421832_at	Region	Twsg1	65960	17	63632504	63660568	-
9903	1457032_at	Region	None	None	3	151444587	151445242	-
9904	1416361_a_at	Region	Dncic1	13426	6	5698564	6000796	+
9905	1451066_at	Region	Leng4	77582	7	1165	3428	-
9906	1460551_at	Region	Ran	19384	5	128195181	128199286	+
9907	1436578_at	Region	A330104H05Rik	77767	2	57970654	57978291	-
9908	1426785_s_at	Region	Mgll	23945	6	89159883	89263412	+
9909	1457990_at	Region	C030032C09Rik	77531	10	90548071	90943317	+
9910	1432016_a_at	Region	Idh3a	67834	9	54704020	54722171	+
9911	1452298_a_at	Region	Myo5b	17919	18	74676774	75005998	+
9912	1455242_at	Region	Foxp1	108655	6	99395348	99630926	-
9913	1457898_at	Region	Kcnq5	226922	1	22045734	22046447	-
9914	1418209_a_at	Region	Pfn2	18645	3	57483992	57489636	-
9915	1448200_at	Region	Tcn2	21452	11	3811977	3826370	-
9916	1454613_at	Region	Dpysl3	22240	18	43544269	43613318	-
9917	1438769_a_at	Region	MGI:1925112	77862	9	26893639	26901251	+
9918	1417288_at	Region	Plekha2	83436	8	23764086	23824498	-
9919	1416718_at	Region	Bcan	12032	3	87731261	87744094	-
9920	1449314_at	Region	Zfpm2	22762	15	40545548	40999330	+
9921	1417283_at	Region	Lynx1	23936	15	74779518	74784641	-
9922	1438193_at	Region	Nrxn3	18191	12	84906378	85735809	+
9923	1424857_a_at	Region	Trim34 /// LOC434218	434218	7	98356814	98374591	+
9924	1429651_at	Region	Phactr3	74189	2	177858975	178053449	+
9925	1432143_a_at	Region	Hbp1	73389	12	28501152	28525243	-
9926	1417132_at	Region	Cdc25a	12530	9	109902125	109918909	+
9927	1424525_at	Region	Grp	225642	18	66106724	66119806	+
9928	1434575_at	Region	Epb4.1l1	13821	2	155877989	155997768	+
9929	1424100_s_at	Region	1500001H12Rik	57754	7	135828437	135831408	-
9930	1459881_at	Region	MGC62475	237730	11	35591414	35592604	-
9931	1424395_at	Region	Asrgl1	66514	19	8308266	8332113	-
9932	1418212_at	Region	Omg	18377	11	79227011	79229678	-
9933	1429274_at	Region	2310010M24Rik	71897	2	49719848	49881008	+
9934	1439433_a_at	Region	Slc35a2	22232	X	6123184	6133345	+
9935	1450333_a_at	Region	Gata2	14461	6	88634163	88642525	+
9936	1434528_at	Region	Aard	239435	15	52040354	52045967	+
9937	1455940_x_at	Region	Wdr6	83669	9	108621311	108627764	-
9938	1440940_at	Region	Cacnb1	12295	11	97823988	97904149	-
9939	1427510_at	Region	Gnai1	14677	5	16687230	16780697	-
9940	1436179_a_at	Region	Dnajc5	13002	2	181237462	181269206	+
9941	1420611_at	Region	Prkacb	18749	3	145706130	145789479	-
9942	1437708_x_at	Region	Vamp3	22319	4	149539667	149550315	-
9943	1423889_at	Region	MGC5739	434402	9	48512636	48513345	+
9944	1450773_at	Region	Kcnd2	16508	6	21263145	21776841	+
9945	1417275_at	Region	Mal	17153	2	127147083	127170136	-
9946	1448438_at	Region	Derl2	116891	11	70733103	70744921	-
9947	1415912_a_at	Region	Rps13	68052	7	110187198	110189820	-
9948	1429361_at	Region	Pmch	110312	10	88063262	88064562	+
9949	1457128_at	Region	AL024213	105253	13	51834168	51834693	-
9950	1430700_a_at	Region	Pla2g7	27226	17	41070175	41113348	+
9951	1416531_at	Region	Gsto1	14873	19	47406289	47416090	+
9952	1424420_at	Region	Ccpg1	72278	9	73118338	73148795	+
9953	1451601_a_at	Region	BC011467	216892	11	72177296	72218168	-
9954	1434073_at	Region	Gprasp2	245607	X	129383995	129389665	+
9955	1418107_at	Region	Tcea2	21400	2	181397266	181405007	+
9956	1424882_a_at	Region	2510015F01Rik	70021	14	29263072	29270876	+
9957	1454759_at	Region	Git1	216963	11	77219071	77233433	+
9958	1422532_at	Region	Xpc	22591	6	91938966	91964918	-
9959	1429400_at	Region	Clcn5	12728	X	5398413	5427626	-
9960	1436689_a_at	Region	Aldh9a1	56752	1	167283401	167301474	+
9961	1437066_at	Region	7330412A13Rik	78143	16	42509171	42511979	+
9962	1435021_at	Region	Gabrb3	14402	7	51863803	52099137	+
9963	1426626_at	Region	Gtf2f2	68705	14	70239863	70353356	-

9964	1416351_at	Region	Map2k1	26395	9	64306481	64375194	-
9965	1425892_a_at	Region	Pnoc	18155	14	59927812	59952527	-
9966	1434503_s_at	Region	Lamp2	16784	X	32940306	32977217	-
9967	1417011_at	Region	Sdc2	15529	15	32919852	33033850	+
9968	1440258_at	Region	None	None	2	180792969	180795082	-
9969	1438915_at	Region	6720401G13Rik	103012	X	45083921	45155862	-
9970	1447707_s_at	Region	Pde2a	207728	7	95527997	95619103	+
9971	1424612_at	Region	9330161F08Rik	223473	15	34572658	34678755	-
9972	1456854_at	Region	Neurl	18011	19	46730295	46810735	+
9973	1421280_at	Region	Gabra1	14394	11	41884317	41935767	-
9974	1417366_s_at	Region	Calm1	12313	12	95642228	95652493	+
9975	1434571_at	Region	Vps13b	328525	15	35918559	35932752	+
9976	1424624_at	Region	2900011O08Rik	67254	16	12722524	12837150	+
9977	1417179_at	Region	Tspan5	56224	3	137631430	137793659	+
9978	1458403_at	Region	Tnik	69014	3	27680596	28087969	+
9979	1450794_at	Region	Avp	11998	2	130094530	130096394	-
9980	1437089_at	Region	4833409A17Rik	74580	19	42270264	42297181	-
9981	1455136_at	Region	Atp1a2	98660	1	172203449	172226896	-
9982	1425575_at	Region	Epha3	13837	16	62549020	62869320	-
9983	1435642_at	Region	9630019K15Rik	320111	17	215	3323	+
9984	1456464_x_at	Region	None	None	NONE	NONE	NONE	NONE
9985	1428936_at	Region	Atp2b1	67972	10	98895589	99006572	+
9986	1424854_at	Region	Hist1h4i	319158	13	21521076	21521387	-
9987	1431777_a_at	Region	Hmgn3	94353	9	83439717	83476376	-
9988	1451544_at	Region	Tapbpl	213233	6	125880885	125888540	-
9989	1437217_at	Region	Ankrd6	140577	4	33082828	33202298	-
9990	1416900_s_at	Region	Gdf1 /// Lass1	14559	8	69476431	69485074	+
9991	1451840_at	Region	Kcnip4	80334	5	47153325	47273714	-
9992	1455361_at	Region	Dgkb	217480	12	34483693	34776186	+
9993	1429589_at	Region	6330404F12Rik	70758	2	22653645	22655680	+
9994	1455605_at	Region	D5Bwg0860e	52822	5	87850046	87908952	+
9995	1455447_at	Region	D430019H16Rik	268595	12	100938471	100940569	+
9996	1422186_s_at	Region	Dia1	109754	15	83204176	83222883	-
9997	1428663_at	Region	5133401H06Rik	71305	3	130248843	130250098	-
9998	1417038_at	Region	38604	53860	11	117152813	117183400	+
9999	1440742_at	Region	Epc1	13831	18	6478658	6558794	-
10000	1447825_x_at	Region	Pcdh8	18530	14	74120840	74125280	-
10001	1460239_at	Region	Tspan13	66109	12	32620734	32648498	-
10002	1450121_at	Region	Scn1a	20265	2	66128285	66206577	-
10003	1431339_a_at	Region	Efhd2	27984	4	140739403	140756181	-
10004	1429549_at	Region	Col27a1	373864	4	62307017	62424499	+
10005	1416069_at	Region	Pfkp	56421	13	6476970	6563786	-
10006	1417774_at	Region	Nans	94181	4	46405251	46419360	+
10007	1423062_at	Region	Igfbp3	16009	11	7103173	7108623	-
10008	1422789_at	Region	Aldh1a2	19378	9	71359672	71440085	+
10009	1421606_a_at	Region	Sult4a1	29859	15	84124696	84154353	-
10010	1435148_at	Region	Atp1b2	11932	11	69325411	69331487	-
10011	1426570_a_at	Region	Frk	14302	10	34545676	34672847	+
10012	1417481_at	Region	Ramp1	51801	1	91003083	91046721	+
10013	1428347_at	Region	Cyfip2	76884	11	45948917	46065130	-
10014	1426039_a_at	Region	Alox12e	11685	11	70041271	70048176	-
10015	1441662_at	Region	None	None	4	114065269	114065575	-
10016	1460432_a_at	Region	Eif3s6	16341	15	43195901	43228545	-
10017	1434902_at	Region	Rnf157	217340	11	116157470	116234559	-
10018	1417374_at	Region	Tuba4	22145	1	75502339	75506618	-
10019	1460006_at	Region	Atbf1	11906	8	108012331	108254925	+
10020	1436260_at	Region	None	None	1	154279911	154280514	-
10021	1456538_at	Region	Sdccag8	76816	1	176747373	176953729	+
10022	1426829_at	Region	Rxrip110	20184	13	53637738	53711331	-
10023	1447800_x_at	Region	Tcn2	21452	11	3811977	3826370	-
10024	1448797_at	Region	Elk3	13713	10	93221365	93283561	-
10025	1456018_at	Region	AL022779	105246	13	69997814	70020895	+
10026	1434603_at	Region	Thrap2	76199	5	117691302	117893539	+
10027	1438752_at	Region	A230058F20Rik	320270	X	36184309	36185041	+
10028	1443847_x_at	Region	None	None	X	64540436	64540649	+

10029	1425927_a_at	Region	Atf5	107503	7	38887185	38891499	-
10030	1457299_at	Region	Grm4	268934	17	25221819	25222622	-
10031	1460555_at	Region	6330500D04Rik	193385	13	24212440	24213658	+
10032	1445523_at	Region	Gje1	118446	5	136904409	136913559	-
10033	1458176_at	Region	Per3	18628	4	149497074	149536951	-
10034	1441233_at	Region	None	None	12	51914268	51914751	+
10035	1457424_at	Region	Eya1	14048	1	14348042	14481971	-
10036	1437522_x_at	Region	Gh	14599	11	106121357	106122899	-
10037	1417022_at	Region	Slc7a3	11989	X	95680582	95686713	-
10038	1436450_at	Region	None	None	11	118310853	118311221	-
10039	1451235_at	Region	1500001H12Rik	57754	7	135828437	135831408	-
10040	1415789_a_at	Region	BC002236	79560	11	44207410	44223282	-
10041	1420718_at	Region	Odz2	23964	11	35801678	36738275	-
10042	1417191_at	Region	Dnajb9	27362	12	121826595	121837207	-
10043	1450975_at	Region	Cacng4	54377	11	107555869	107615553	-
10044	1421568_at	Region	Kcna6	16494	6	127379948	127411808	-
10045	1442021_at	Region	Gnal	14680	18	67317669	67452049	+
10046	1419722_at	Region	Prss19	259277	7	37872977	37879728	+
10047	1431393_at	Region	4930447C04Rik	75801	12	69728240	69764946	-
10048	1421969_a_at	Region	Faah	14073	4	114955573	114976807	-
10049	1455056_at	Region	Lmo7	380928	14	96369275	96463310	+
10050	1424402_at	Region	D5Bwg0860e	52822	5	87850046	87908952	+
10051	1423619_at	Region	Rasd1	19416	11	59688823	59690584	-
10052	1425597_a_at	Region	Qk	19317	17	8850890	8959849	-
10053	1435712_a_at	Region	Rps18	20084	17	31655742	31659385	-
10054	1444028_s_at	Region	Dock9	105445	14	116113166	116206323	-
10055	1438671_at	Region	Ppp2r2c	269643	5	35375082	35461590	+
10056	1415907_at	Region	Ccnd3	12445	17	45112008	45118149	+
10057	1437992_x_at	Region	Gja1	14609	10	56627159	56640230	+
10058	1448910_at	Region	Pecr	111175	1	72555295	72582008	-
10059	1457867_at	Region	Sgpp2	433323	1	78598998	78708934	+
10060	1436182_at	Region	Satb1	20230	17	49274277	49367498	-
10061	1447551_x_at	Region	Lphn3	319387	5	80275281	81049432	+
10062	1433585_at	Region	Tnpo1	238799	13	95031172	95114281	-
10063	1429111_at	Region	Tln2	70549	9	67349709	67668766	-
10064	1424741_s_at	Region	Creb3	12913	4	43478591	43482789	+
10065	1424556_at	Region	Pycr1	209027	11	120460517	120464797	-
10066	1431826_a_at	Region	Brsk2	75770	7	136362546	136417039	+
10067	1426519_at	Region	P4ha1	18451	10	59286782	59335753	+
10068	1417131_at	Region	Cdc25a	12530	9	109902125	109918909	+
10069	1419979_s_at	Region	Creb3	12913	4	43478591	43482789	+
10070	1439412_at	Region	Arpp21	19050	NONE	NONE	NONE	NONE
10071	1420505_a_at	Region	Stxbp1	20910	2	32720124	32779288	-
10072	1417028_a_at	Region	Trim2	80890	3	83898180	83960638	-
10073	1424530_at	Region	Sec14l2	67815	11	3991826	4013516	-
10074	1438559_x_at	Region	1110028E10Rik	68682	9	21224117	21241293	+
10075	1438068_at	Region	None	None	5	107441765	107442579	-
10076	1415971_at	Region	Marcks	17118	10	37207003	37211096	-
10077	1422456_at	Region	Nsf	18195	11	103642871	103775145	-
10078	1435580_at	Region	C230081A13Rik	244895	9	56336855	56527185	-
10079	1434292_at	Region	E130013N09Rik	99358	NONE	NONE	NONE	NONE
10080	1437977_at	Region	2410002O22Rik	66975	13	100361503	100396609	-
10081	1448342_at	Region	Mapk10	26414	5	101946583	102247027	-
10082	1423544_at	Region	Ptpn5	19259	7	41163785	41219667	-
10083	1454796_at	Region	D5Ertd40e	231630	5	112846457	112851320	+
10084	1450696_at	Region	Psmb9	16912	17	31887730	31892961	-
10085	1455735_at	Region	Ap1s3	252903	1	79944670	80009757	-
10086	1435462_at	Region	LOC433022	433022	16	44845024	44847514	-
10087	1426606_at	Region	Crtac1	72832	19	41827441	41976053	-
10088	1433551_at	Region	AI427515	270097	8	113506193	113688506	+
10089	1417827_at	Region	Ngly1	59007	14	14745876	14808542	+
10090	1450164_at	Region	Ascl1	17172	10	87463685	87466044	-
10091	1416064_a_at	Region	Hspa5	14828	2	34704257	34708619	+
10092	1452065_at	Region	BC027127	211739	11	16152565	16178979	+
10093	1442150_at	Region	None	None	1	63409464	63410570	+

10094	1436660_at	Region	Rrbp1	81910	2	143442799	143468120	-
10095	1437868_at	Region	BC023892	212943	9	85652955	85678535	-
10096	1438684_at	Region	Nuak1	77976	10	84266116	84335276	-
10097	1435735_x_at	Region	H47	109815	7	59963883	59973627	+
10098	1431717_at	Region	3526401B18Rik	70774	12	73188475	73190231	-
10099	1416808_at	Region	Nid1	18073	13	12856586	12931160	+
10100	1427932_s_at	Region	1200003I10Rik /// 1200016E24Rik /// A130040M12Rik /// E430024C06Rik /// LOC433071 /// LOC546178 /// LOC547372	319202	NONE	NONE	NONE	NONE
10101	1454834_at	Region	Nfib	18028	4	81281923	81491315	-
10102	1460446_at	Region	Wrb	71446	16	95583096	95595535	+
10103	1417489_at	Region	Npy2r	18167	3	82267450	82277153	-
10104	1417653_at	Region	Pvalb	19293	15	78243110	78258344	-
10105	1439444_x_at	Region	1110014C03Rik	68581	12	82210494	82242351	-
10106	1442752_at	Region	Opcml	330908	9	28570800	28821443	+
10107	1451396_at	Region	Pomt2	217734	12	83988455	84027132	-
10108	1441429_at	Region	Irs4	16370	X	135151041	135165260	-
10109	1455066_s_at	Region	9130229H14Rik	236520	1	6211	14138	-
10110	1416885_at	Region	1110038F14Rik	117171	15	77000761	77002949	+
10111	1451627_a_at	Region	Slc1a2	20511	2	102411192	102482860	+
10112	1441259_s_at	Region	Wdr10	81896	6	116291063	116364225	+
10113	1426621_a_at	Region	Ppp2r2b	72930	18	42865902	42961824	-
10114	1439548_at	Region	Rap2b	74012	3	61043051	61046895	+
10115	1428792_at	Region	Bcas1	76960	2	169855881	169936561	-
10116	1427685_a_at	Region	Synj2	20975	17	5881998	5950653	+
10117	1460444_at	Region	Arrb1	109689	7	93624118	93692062	+
10118	1449450_at	Region	Ptges	64292	2	30821633	30835459	-
10119	1417301_at	Region	Fzd6	14368	15	38905352	38937760	+
10120	1428801_at	Region	Mgat3	17309	15	80263672	80268219	+
10121	1422629_s_at	Region	Shrm	27428	5	92019185	92300704	+
10122	1424086_at	Region	D9Ucla1	102644	9	43180556	43199094	-
10123	1416022_at	Region	Fabp5	16592	3	10011881	10015886	+
10124	1451595_a_at	Region	Kcnq2	16536	2	180792537	180852183	-
10125	1454656_at	Region	Spata13	219140	14	55169182	55299693	+
10126	1419249_at	Region	Pftk1	18647	5	4809738	5386208	-
10127	1434310_at	Region	Bmpr2	12168	1	60068175	60174399	+
10128	1436622_at	Region	None	None	X	145713414	145729005	+
10129	1449221_a_at	Region	Rrbp1	81910	2	143442799	143468120	-
10130	1434107_at	Region	Spata2	263876	2	166938128	166949596	-
10131	1428466_at	Region	Chd3	216848	11	69068931	69091238	-
10132	1441369_at	Region	None	None	18	40765877	40766773	+
10133	1417141_at	Region	Igtp	16145	11	57925185	57933220	+
10134	1416191_at	Region	Sec61a1	53421	6	88938985	88954178	-
10135	1429416_at	Region	2900074C18Rik	73032	7	23028864	23031149	+
10136	1433959_at	Region	Zmat4	320158	8	22424307	22817717	+
10137	1441899_x_at	Region	Bcan	12032	3	87731261	87744094	-
10138	1450287_at	Region	Npas3 /// LOC544865 /// LOC546536	27386	12	50702581	50824492	+
10139	1417636_at	Region	Slc6a9	14664	4	116794241	116828210	+
10140	1433591_at	Region	AI553587	103784	11	17106711	17128589	+
10141	1438796_at	Region	None	None	4	48001207	48002366	+
10142	1448237_x_at	Region	Ldh2	16832	6	143333518	143351212	-
10143	1451046_at	Region	Zfpm1	22761	8	121663726	121718834	+
10144	1455177_at	Region	Ahi1	52906	10	20882283	21010464	+
10145	1429246_a_at	Region	Anxa6	11749	11	54731952	54786255	-
10146	1453329_s_at	Region	6330571D19Rik	432450	10	31729508	31730785	-
10147	1428063_at	Region	Ankrd46	68839	15	36479034	36498329	-
10148	1420872_at	Region	Gucy1b3	54195	3	81761066	81803745	-
10149	1453008_at	Region	2300002D11Rik	69539	4	132452002	132459405	-
10150	1422508_at	Region	Atp6v1a1	11964	16	42970115	43022327	-

10151	1448739_x_at	Region	Rps18	20084	17	31655742	31659385	-
10152	1417943_at	Region	Gng4	14706	13	13229694	13273161	+
10153	1426167_a_at	Region	Camk4	12326	18	33179625	33409958	+
10154	1433802_at	Region	AW125688	381199	19	4868127	4874267	-
10155	1417380_at	Region	Iqgap1	29875	7	74515481	74605994	-
10156	1427905_at	Region	1810063B07Rik	67509	14	18438397	18446022	-
10157	1418494_at	Region	Ebf2	13592	14	61760384	61959861	+
10158	1450088_a_at	Region	Mobp	17433	9	120159695	120186044	+
10159	1423075_at	Region	Lman2	66890	13	53952542	53972056	-
10160	1423607_at	Region	Lum	17022	10	97546067	97553024	+
10161	1415822_at	Region	Scd2	20250	19	43838494	43851266	+
10162	1418505_at	Region	Nudt4	71207	10	95523184	95539880	-
10163	1426542_at	Region	2310067E08Rik	71946	9	14189509	14216547	-
10164	1426587_a_at	Region	Stat3	20848	11	100709309	100760491	-
10165	1423365_at	Region	Cacna1g	12291	11	94229603	94294812	-
10166	1441065_at	Region	None	None	13	15057247	15057834	+
10167	1447909_s_at	Region	MGI:1931466 /// LOC432812	432812	13	60225860	60254605	+
10168	1422018_at	Region	Hivep2	15273	10	14029087	14051962	+
10169	1459723_at	Region	Gm262	238331	12	83857606	83862901	-
10170	1416766_at	Region	Mosc2	67247	1	184311520	184344299	-
10171	1423545_a_at	Region	Zfp207	22680	11	80109005	80121796	+
10172	1426028_a_at	Region	Cit	12704	5	114963119	115127860	+
10173	1451796_s_at	Region	Hdc	15186	2	126107521	126132518	-
10174	1440323_at	Region	Syt2	20980	1	134496944	134599680	+
10175	1416445_at	Region	2810405J04Rik	72722	17	73347397	73361507	-
10176	1423679_at	Region	2810432L12Rik	67063	4	49500451	49513677	-
10177	1418320_at	Region	Prss8	76560	7	121975527	121979904	-
10178	1435308_at	Region	Fut9	14348	4	25753460	25935314	-
10179	1428250_at	Region	Gpr30	76854	5	138420671	138425280	+
10180	1449420_at	Region	Pde1b	18574	15	103566380	103593133	+
10181	1453273_at	Region	Kcnv1	67498	15	45052998	45061646	-
10182	1440201_at	Region	AV344025	106755	17	79205697	79206709	-
10183	1422452_at	Region	Bag3	29810	7	122573079	122596498	+
10184	1429891_at	Region	Capsl	75568	15	9248356	9279435	+
10185	1429315_at	Region	Syt11	229521	3	88489566	88516404	-
10186	1444693_at	Region	Cacnb2	12296	2	14530578	14913205	+
10187	1448380_at	Region	Lgals3bp	19039	11	118213845	118223024	-
10188	1436876_at	Region	D13Bwg1146e	52882	13	101166216	101269810	-
10189	1418135_at	Region	Aff1	17355	5	102817913	102885914	+
10190	1417241_at	Region	X83328	13929	11	109247065	109273461	+
10191	1419686_at	Region	Tsga14	83922	6	30701454	30741755	-
10192	1451219_at	Region	Ormdl1	227102	1	53604628	53617817	+
10193	1433687_at	Region	AI662250	106639	17	54402558	54406722	-
10194	1421546_a_at	Region	Racgap1	26934	15	99677712	99708837	-
10195	1424565_at	Region	Polr3d	67065	14	64754785	64759436	-
10196	1417039_a_at	Region	Cul7	66515	17	44160990	44175008	+
10197	1439612_at	Region	Cacna1b	12287	2	24538537	24695314	-
10198	1434657_at	Region	Gls	14660	1	52466745	52534174	-
10199	1448478_at	Region	Trfp	56771	17	45130056	45142878	+
10200	1459144_at	Region	Fndc3	319448	14	66891442	67062343	-
10201	1417577_at	Region	Trpc3	22065	3	36084163	36135201	-
10202	1448757_at	Region	Pml	18854	9	58332803	58364326	-
10203	1441801_at	Region	Gtf2f2	68705	14	70239863	70353356	-
10204	1423034_at	Region	Txnl5	52700	11	71933212	71936145	+
10205	1433708_at	Region	Srp68	217337	11	116066259	116095310	-
10206	1437390_x_at	Region	Stx1a	20907	5	134037064	134064591	+
10207	1448487_at	Region	Lrrfip1	16978	1	90875248	90952241	+
10208	1455672_s_at	Region	Cplx2	12890	13	52985707	52986681	+
10209	1455609_at	Region	C030025P15Rik	320895	5	115129574	115130430	+
10210	1437056_x_at	Region	Crispld2	78892	8	119359392	119408065	+
10211	1417027_at	Region	Trim2	80890	3	83898180	83960638	-
10212	1424949_at	Region	Huwe1	59026	X	145451533	145469813	+
10213	1416452_at	Region	Oat	18242	7	126923351	126936010	-
10214	1450380_at	Region	Epdr2	105298	13	19073208	19101305	-

10215	1435512_at	Region	AI836003	239650	15	98243222	98245696	+
10216	1433522_at	Region	Pskh1	244631	8	105196321	105227649	+
10217	1430820_a_at	Region	Bbx	70508	16	49039981	49275616	-
10218	1438784_at	Region	None	None	12	103385220	103385903	-
10219	1448325_at	Region	Myd116	17872	7	39605336	39608716	-
10220	1422773_at	Region	Myt1	17932	2	181483998	181544724	+
10221	1435120_at	Region	None	None	8	101921874	101922507	-
10222	1450017_at	Region	Ccng1	12450	11	40489722	40494170	-
10223	1436163_at	Region	Kcnj16	16517	11	110789161	110849057	+
10224	1435141_at	Region	SFT2 domain containing 2	108735	1	165097432	165117255	-
10225	1454666_at	Region	Klf3	16599	5	63601098	63627704	+
10226	1448018_at	Region	Rere	68703	4	148898702	149114159	+
10227	1423743_at	Region	Arcn1	213827	9	44730423	44756652	-
10228	1432164_a_at	Region	Gcsh	68133	8	116311176	116322181	-
10229	1453620_at	Region	3010022N24Rik	68069	6	18562938	18564218	-
10230	1428265_at	Region	Ppp2r1b	73699	9	50938901	50953949	+
10231	1439334_at	Region	None	None	X	135167546	135168172	+
10232	1452597_at	Region	2310061C15Rik	66531	8	116217815	116249733	-
10233	1451413_at	Region	Cast	12380	13	70751497	70865131	-
10234	1428976_at	Region	Tmpo	21917	10	91133084	91142861	-
10235	1459860_x_at	Region	Trim2	80890	3	83898180	83960638	-
10236	1438774_s_at	Region	Pgm2l1	70974	7	94334296	94382030	+
10237	1423262_a_at	Region	H3f3a	15078	1	180756388	180767423	-
10238	1429718_at	Region	Slitrk5	75409	14	106214046	106222066	+
10239	1425052_at	Region	2610034N03Rik	66307	18	58884044	58904170	+
10240	1437842_at	Region	A330045H12Rik	403178	5	109150217	109154339	+
10241	1435368_a_at	Region	Parp1	11545	1	180523221	180555500	+
10242	1451991_at	Region	Epha7	13841	4	28997442	29149372	+
10243	1435314_at	Region	Tph2	216343	10	114762957	114869788	-
10244	1422577_at	Region	Cs	12974	10	128074651	128099298	+
10245	1415801_at	Region	Gja1	14609	10	56627159	56640230	+
10246	1452267_at	Region	Flywch1	224613	17	21557603	21565956	-
10247	1418474_at	Region	1500005A01Rik	85308	14	50099490	50103265	-
10248	1428718_at	Region	Scrn1	69938	6	54653141	54710707	-
10249	1450047_at	Region	Hs6st2	50786	X	45909852	46201195	-
10250	1436961_at	Region	Hspa12a	73442	19	58396851	58460502	-
10251	1427426_at	Region	Kcnq5	226922	1	21628315	22188135	-
10252	1426013_s_at	Region	Plekha4	69217	7	39574953	39637592	+
10253	1425563_s_at	Region	Pcdh10	18526	3	44837486	44841910	+
10254	1433566_at	Region	Rasl10b	276952	11	83137304	83146823	+
10255	1457658_x_at	Region	Anxa4	11746	6	87171158	87227870	-
10256	1435117_a_at	Region	C330021A05Rik	217378	12	3234875	3262331	+
10257	1420123_at	Region	Tcta	102791	9	108372719	108375712	-
10258	1415978_at	Region	Tubb3	22152	8	122786461	122796907	+
10259	1460581_a_at	Region	Rpl13 /// LOC280047 /// LOC432861 /// LOC547376	270106	8	122483697	122486521	+
10260	1417040_a_at	Region	Bok	51800	1	93513185	93523253	+
10261	1439332_at	Region	Ddit4l	73284	3	136513537	136518197	+
10262	1422721_at	Region	Tnk1	83813	11	69576757	69584375	-
10263	1434301_at	Region	D330050I23Rik	399603	15	60824299	60827416	-
10264	1425053_at	Region	Isoc1	66307	18	58884044	58904170	+
10265	1423506_a_at	Region	Nnat	18111	2	157017092	157019473	+
10266	1424375_s_at	Region	Gimap4	107526	6	48816795	48824784	+
10267	1420473_at	Region	Mtpn	14489	6	35603109	35634015	-
10268	1453004_at	Region	3110004L20Rik	73102	13	33714148	33929039	-
10269	1421441_at	Region	Angpt1	11600	15	42335548	42587605	-
10270	1460360_at	Region	Asrgl1	66514	19	8308266	8332113	-
10271	1436508_at	Region	2410014A08Rik	109154	5	114254717	114267772	-
10272	1422578_at	Region	Cs	12974	10	128074651	128099298	+
10273	1430779_at	Region	A930010C08Rik	77943	3	109951781	109952402	-
10274	1437065_at	Region	7330412A13Rik	78143	16	42509171	42511979	+
10275	1455148_at	Region	C130036G08	243339	5	143778139	143803802	-

10276	1449245_at	Region	Grin2c	14813	11	115070262	115088336	-
10277	1455289_at	Region	B930093C12Rik	268445	11	77196147	77198731	-
10278	1417402_at	Region	1190017O12Rik	68936	16	91458563	91470749	+
10279	1417181_a_at	Region	Kifap3	16579	1	163697221	163834642	+
10280	1426306_a_at	Region	Maged2	80884	X	144340816	144348677	-
10281	1435788_at	Region	2900086B20Rik	73074	12	67798894	67799727	+
10282	1419706_a_at	Region	Akap12	83397	10	6145386	6238479	-
10283	1449441_a_at	Region	Wbp1	22377	6	83462734	83465112	-
10284	1455325_at	Region	A230057G18Rik	330166	5	111288191	111298937	-
10285	1418528_a_at	Region	Dad1	13135	14	48755589	48773800	-
10286	1434005_at	Region	Rbms1	56878	2	60608122	60818320	-
10287	1451028_at	Region	Baiap2	108100	11	119764194	119822580	+
10288	1444503_at	Region	Gbas	14467	5	128900966	128934118	+
10289	1432103_a_at	Region	Sh3gl3	20408	7	76116902	76163933	+
10290	1420172_at	Region	Myh9	17886	15	77812545	77894062	-
10291	1435750_at	Region	Gchfr	320415	2	118681637	118686238	+
10292	1448832_a_at	Region	Cplx1	12889	5	107594772	107626145	-
10293	1428945_at	Region	5730469D23Rik	231380	5	85372730	85434753	-
10294	1428928_at	Region	3110038O15Rik	73120	2	52360370	52361890	-
10295	1416276_a_at	Region	Rps4x	20102	X	96786304	96790667	-
10296	1456733_x_at	Region	Serpinh1	12406	7	93433937	93441711	-
10297	1424523_at	Region	Elmo1	140580	13	19666927	20088452	+
10298	1424707_at	Region	1110014C03Rik	68581	12	82210494	82242351	-
10299	1429503_at	Region	2900024C23Rik	67266	5	106978787	107057482	-
10300	1455375_at	Region	None	None	1	181855245	181855800	-
10301	1419546_at	Region	Atp6v1c1	66335	15	38667202	38697131	+
10302	1439740_s_at	Region	AI481316	98383	1	167156103	167156616	-
10303	1452654_at	Region	Zdhhc2	70546	8	39367458	39428485	+
10304	1424567_at	Region	Tspan2	70747	3	102162658	102198922	+
10305	1418476_at	Region	Crlf1	12931	8	1573	4254	-
10306	1455708_at	Region	Tmod3	50875	9	75635361	75697149	-
10307	1438572_at	Region	Csmd3	239420	15	47577326	48359844	-
10308	1453849_s_at	Region	Hnrpab	15384	11	51352941	51359688	-
10309	1451634_at	Region	2810051F02Rik	72704	NONE	NONE	NONE	NONE
10310	1428615_at	Region	P2ry5	67168	14	67589330	67591777	+
10311	1453065_at	Region	Aldh5a1	214579	13	24390909	24417538	-
10312	1417090_at	Region	Rcn1	19672	2	105091687	105103993	-
10313	1448534_at	Region	Ptpns1	19261	2	129107171	129144785	+
10314	1435255_at	Region	Plxnb1	235611	9	109142184	109166503	+
10315	1455558_at	Region	Gm114	228730	2	146291291	146427030	+
10316	1418774_a_at	Region	Atp7a	11977	X	100628682	100726282	+
10317	1428864_at	Region	5530400B01Rik	71434	7	136492281	136494259	-
10318	1449471_at	Region	Kcnmb4	58802	10	116107314	116162975	-
10319	1435450_at	Region	Cpne3	70568	4	19448948	19497068	-
10320	1424789_at	Region	Hkr2	232878	7	10891496	10901920	+
10321	1431280_at	Region	AI597468	103266	10	84997433	85015279	+
10322	1448949_at	Region	Car4	12351	11	84685835	84694036	+
10323	1423515_at	Region	Scn8a	20273	15	100993863	101098226	+
10324	1418851_at	Region	Trim39	79263	17	34008393	34021525	-
10325	1420667_at	Region	Doc2b	13447	11	75494748	75521715	-
10326	1460615_at	Region	Nt5c2l1	319638	10	34364736	34479712	-
10327	1435017_at	Region	Mel13	217149	11	97506688	97509712	+
10328	1423571_at	Region	Edg1	13609	3	114480955	114485563	-
10329	1417149_at	Region	P4ha2	18452	11	53854357	53884489	+
10330	1417674_s_at	Region	Golga4	54214	9	118500315	118578377	+
10331	1418868_at	Region	En2	13799	5	26616504	26622197	+
10332	1441983_at	Region	None	None	16	94153738	94154753	-
10333	1419601_at	Region	Kcnj10	16513	1	172270320	172303004	+
10334	1422586_at	Region	Ecel1	13599	1	86963814	86971186	-
10335	1425110_at	Region	Sorcs3	66673	19	47757239	48356719	+
10336	1452327_at	Region	Iqsec1	232227	6	91098830	91101520	-
10337	1418501_a_at	Region	Oxr1	170719	15	41691294	41762827	+
10338	1417304_at	Region	Chrd	12667	16	19504234	19513489	+
10339	1425248_a_at	Region	Tyro3	22174	2	119313419	119331330	+
10340	1418273_a_at	Region	Rpl30	19946	15	34440221	34442933	-

10341	1428316_a_at	Region	Fundc2	67391	X	70043077	70056771	+
10342	1438540_at	Region	None	None	3	129529323	129529976	+
10343	1452608_at	Region	Mycbp	56309	4	122932163	122939256	+
10344	1452081_a_at	Region	9130017N09Rik	78906	10	79943645	79953076	+
10345	1426550_at	Region	Sidt1	320007	16	43123510	43216168	-
10346	1444761_at	Region	None	None	7	13947701	13948427	+
10347	1450705_at	Region	Rdbp	27632	17	32553733	32558972	+
10348	1452225_at	Region	2010106G01Rik	66552	2	126407985	126447088	-
10349	1428709_a_at	Region	Mrpl24	67707	3	87663204	87667324	+
10350	1423472_at	Region	38597	18000	1	93316614	93337221	+
10351	1417289_at	Region	Plekha2	83436	8	23764086	23824498	-
10352	1437292_at	Region	A330019N05Rik	215890	10	33567437	33679703	-
10353	1444696_at	Region	None	None	14	54173610	54174656	-
10354	1424186_at	Region	2610001E17Rik	67896	16	43977206	44010706	+
10355	1442889_at	Region	None	None	13	18629651	18630452	+
10356	1455344_at	Region	AW494418	99908	NONE	NONE	NONE	NONE
10357	1427537_at	Region	Eppk1	223650	15	76152276	76168867	-
10358	1455618_x_at	Region	1300010A20Rik	232670	6	29741353	29765655	+
10359	1416807_at	Region	Rpl36a	19982	X	128130624	128132999	+
10360	1437706_x_at	Region	Rps14 /// LOC545121	20044	18	60999975	61003870	+
10361	1428741_at	Region	Elavl4	15572	4	109165070	109310814	-
10362	1450414_at	Region	Pdgfb	18591	15	80048194	80067128	-
10363	1451229_at	Region	Hdac11	232232	6	91599688	91617556	+
10364	1419823_s_at	Region	Ksr	16706	11	78740461	78872020	-
10365	1448401_at	Region	Smarcd2	83796	11	106084275	106088711	-
10366	1426752_at	Region	Phf17	269424	3	41010564	41046427	+
10367	1418510_s_at	Region	Fbxo8	50753	8	55605791	55648596	+
10368	1415881_at	Region	Ghitm	66092	14	35253316	35267764	-
10369	1421978_at	Region	Gad2	14417	2	22584120	22652794	+
10370	1456251_x_at	Region	Bzrp	12257	15	83612210	83636054	+
10371	1423765_at	Region	BC023151	212974	7	135343568	135349645	+
10372	1424373_at	Region	Armcx3	71703	X	128301536	128306395	+
10373	1425929_a_at	Region	Rnf14	56736	18	38520411	38541525	+
10374	1416329_at	Region	Cyfip1	20430	7	50141336	50199963	+
10375	1416026_a_at	Region	Rpl12	269261	2	32893888	32896203	+
10376	1451364_at	Region	Polr3gl	69870	3	96065710	96081979	-
10377	1419044_at	Region	Cntnap4	170571	8	111866933	112181180	+
10378	1435832_at	Region	Lrrc4	192198	6	28876498	28878711	-
10379	1435256_at	Region	1500005P14Rik	76686	7	25693378	25719722	+
10380	1437333_x_at	Region	Aldh18a1	56454	19	40094578	40132690	-
10381	1423817_s_at	Region	2010315L10Rik	67023	8	70517056	70519559	+
10382	1455165_at	Region	None	None	9	69518542	69520199	+
10383	1446622_at	Region	A330068G13Rik	414087	7	71965890	71966527	-
10384	1450970_at	Region	Got1	14718	19	43044161	43068915	-
10385	1426567_a_at	Region	Pqlc1	66943	18	80379828	80416235	+
10386	1457292_at	Region	4930553M18Rik	75316	9	15143402	15154138	+
10387	1460672_at	Region	2410002F23Rik	66976	7	38321775	38326651	+
10388	1429131_at	Region	Ube2v2	70620	16	14321751	14363568	-
10389	1443904_at	Region	Fads6	328035	11	115104459	115118607	-
10390	1424743_at	Region	2610003J06Rik	72106	17	23635208	23637927	+
10391	1431008_at	Region	0610037M15Rik	68395	NONE	NONE	NONE	NONE
10392	1453055_at	Region	Sema6d	214968	2	124124149	124181623	+
10393	1450027_at	Region	Sdc3	20970	4	129589463	129623202	+
10394	1452038_at	Region	Capza1 /// LOC546155	12340	3	104249759	104291308	-
10395	1439026_at	Region	Trpm3	226025	19	21686135	22227805	+
10396	1451339_at	Region	Suox	211389	10	128406984	128410951	-
10397	1426622_a_at	Region	Qpct	70536	17	76864189	76911773	+
10398	1423885_at	Region	Lamc1	226519	1	153103649	153217512	-
10399	1440270_at	Region	Fgf12	14167	16	26943562	27228998	-
10400	1454708_at	Region	Ablim1	226251	19	56622771	56801757	-
10401	1422866_at	Region	Col13a1	12817	10	61804824	61945626	-
10402	1427019_at	Region	Ptprz1	19283	6	22927206	23056382	+
10403	1417074_at	Region	Ceacam10	26366	7	19954879	19962377	+

10404	1419278_at	Region	Usp48	170707	4	136475016	136537437	+
10405	1427226_at	Region	Epn2	13855	11	61242891	61305293	-
10406	1438201_at	Region	None	None	4	11885151	11885960	-
10407	1436702_at	Region	BC034068	269642	5	32485166	32491323	+
10408	1426886_at	Region	Cln5	211286	14	97610921	97623064	+
10409	1452936_at	Region	Crtac1	72832	19	41827441	41976053	-
10410	1450114_at	Region	Ksr	16706	11	78740461	78872020	-
10411	1435460_at	Region	Prkg2	19092	5	97950831	98055222	-
10412	1440250_at	Region	E130010M05Rik	319847	1	82777551	82781741	-
10413	1418570_at	Region	Ncstn	59287	1	171995017	172011613	-
10414	1416123_at	Region	Ccnd2	12444	6	127802641	127824339	-
10415	1434635_at	Region	Rph3a	19894	5	120092306	120161325	-
10416	1453756_at	Region	2900075N08Rik	72991	X	95924146	95926159	+
10417	1422432_at	Region	Dbi	13167	1	119870842	119878615	-
10418	1415922_s_at	Region	Mlp	17357	4	128540757	128543088	+
10419	1424737_at	Region	Thrsp	21835	7	91459228	91463781	-
10420	1420405_at	Region	Slco1a4	28250	6	142613255	142664334	-
10421	1449423_at	Region	Mast1	56527	8	84179304	84205636	-
10422	1443375_at	Region	Gnas	14683	2	173709228	173771651	+
10423	1423477_at	Region	Zic1	22771	9	91251432	91256620	-
10424	1436688_x_at	Region	Rpl14	67115	9	120583908	120587044	+
10425	1425767_a_at	Region	Six4	20474	12	69946438	69959424	-
10426	1447521_x_at	Region	D15Wsu169e	223666	15	76775364	76869603	-
10427	1450697_at	Region	Slc30a7	66500	3	114716461	114777895	-
10428	1434761_at	Region	Lrrtm3	216028	10	63866884	64028642	-
10429	1416406_at	Region	Pea15	18611	1	172081687	172129498	-
10430	1425600_a_at	Region	Plcb1	18795	2	134734571	134986275	+
10431	1434849_at	Region	Tspyl2	52808	X	145871248	145876838	-
10432	1448356_at	Region	Ube2d2	56550	18	35995272	36030828	+
10433	1427281_at	Region	Scn2a1	110876	2	65619938	65621824	+
10434	1438072_at	Region	Nfib	18028	4	81281923	81491315	-
10435	1433991_x_at	Region	Dbi	13167	1	119870842	119878615	-
10436	1451842_a_at	Region	Chrnb3	108043	8	26137431	26169283	+
10437	1429138_at	Region	Npas3	27386	12	50702581	50824492	+
10438	1423453_at	Region	C78541	97961	15	78986217	78994920	+
10439	1428847_a_at	Region	Macf1	11426	4	122378001	122711301	-
10440	1448594_at	Region	Wisp1	22402	15	66915598	66947404	+
10441	1434102_at	Region	Nfib	18028	4	81281923	81491315	-
10442	1418540_a_at	Region	Ptpre	19267	7	129905416	130053886	+
10443	1439006_x_at	Region	6430550H21Rik	245386	X	32718102	32773193	-
10444	1434885_at	Region	5830435K17Rik	101685	7	41076681	41094192	-
10445	1437347_at	Region	Ednrb	13618	14	98359614	98388688	-
10446	1416368_at	Region	Gsta4	14860	9	78360919	78378190	+
10447	1455919_at	Region	None	None	11	66099894	66100557	-
10448	1435700_at	Region	Tln2	70549	9	67349709	67668766	-
10449	1452183_a_at	Region	Gtl2	17263	12	105023823	105039917	+
10450	1429115_at	Region	2010003O02Rik	66434	4	40408436	40409792	+
10451	1428427_at	Region	Fbxl2	72179	9	114028966	114079010	-
10452	1448063_at	Region	Iqsec2	245666	X	145713404	145759631	+
10453	1418010_a_at	Region	Sh3glb1	54673	3	143660519	143691998	-
10454	1415770_at	Region	Wdr6	83669	9	108621311	108627764	-
10455	1456214_at	Region	Pcdh7	54216	5	56521609	56532964	+
10456	1454424_at	Region	2610040L17Rik	72464	11	36470417	36472016	-
10457	1422481_at	Region	Krt2-1	16678	15	101903976	101909256	-
10458	1423511_at	Region	Asf1a	66403	10	53842489	53854684	+
10459	1455296_at	Region	Adcy5	224129	16	34082407	34088712	+
10460	1455631_at	Region	H13	14950	2	152126435	152163760	+
10461	1452716_at	Region	5730469M10Rik	70564	14	39139992	39160295	-
10462	1449405_at	Region	Tns1	21961	1	74217097	74291359	-
10463	1426978_at	Region	Klhl2	77113	8	63808844	63849038	-
10464	1427680_a_at	Region	Nfib	18028	4	81281923	81491315	-
10465	1439022_at	Region	Phactr1	218194	13	42242828	42700966	+
10466	1452344_at	Region	Synj2	20975	17	5881998	5950653	+
10467	1459894_at	Region	LOC544963	544963	13	91817361	91882649	-
10468	1426530_a_at	Region	Klhl5	71778	5	63933500	63955461	+

10469	1418104_at	Region	Nrip3	78593	7	103610927	103634416	-
10470	1449528_at	Region	Figf	14205	X	157972953	158002052	+
10471	1450974_at	Region	Timp4	110595	6	115681210	115687239	-
10472	1425529_s_at	Region	D19Wsu162e	226178	19	46146382	46202440	+
10473	1451227_a_at	Region	Slc10a3	214601	X	69029872	69033911	-
10474	1449059_a_at	Region	Oxct1	67041	15	3809284	3936636	+
10475	1450667_a_at	Region	Cs	12974	10	128074651	128099298	+
10476	1423136_at	Region	Fgf1	14164	18	39062902	39142421	-
10477	1433596_at	Region	Dnajc6	72685	4	100509499	100601704	+
10478	1419279_at	Region	Pip5k2a	18718	2	18884270	19040126	-
10479	1436921_at	Region	Atp7a	11977	X	100628682	100726282	+
10480	1455845_at	Region	BC030477	216881	11	71476361	71515305	+
10481	1423157_at	Region	Gnpnat1	54342	14	40454704	40467079	-
10482	1417373_a_at	Region	Tuba4	22145	1	75502339	75506618	-
10483	1435879_at	Region	Akt3	23797	1	176955410	177064396	-
10484	1460688_s_at	Region	AA407659	106840	5	114232192	114244601	-
10485	1423561_at	Region	Nell2	54003	15	95287908	95595549	-
10486	1423256_a_at	Region	Atp6v1g1	66290	4	62636028	62641892	+
10487	1449312_at	Region	Npy5r	18168	8	65789090	65797217	-
10488	1420583_a_at	Region	Rora	19883	9	69474948	69510983	+
10489	1440071_at	Region	Baiap1	14924	6	94131999	94737301	-
10490	1436866_at	Region	Efna5	13640	17	60297708	60574961	-
10491	1448460_at	Region	Acvr1	11477	2	58371977	58441698	-
10492	1416826_a_at	Region	Trfp	56771	17	45130056	45142878	+
10493	1456764_at	Region	Slc35f3	210027	8	125586128	125683011	+
10494	1460028_at	Region	None	None	6	92210633	92211004	-
10495	1423624_at	Region	Fancl	67030	11	26281874	26366666	+
10496	1434032_at	Region	None	None	5	22946572	22947083	+
10497	1460625_at	Region	Gm1568	380768	12	77617054	77621942	-
10498	1457243_at	Region	None	None	NONE	NONE	NONE	NONE
10499	1416029_at	Region	Klf10	21847	15	38299350	38305644	-
10500	1434784_s_at	Region	D15Ertd405e	380967	15	98040510	98045837	+
10501	1425885_a_at	Region	Kcnab2	16498	4	150883106	150969841	-
10502	1442214_at	Region	Nfib	18028	4	81281923	81491315	-
10503	1430675_at	Region	2900055J20Rik	73001	18	40480760	40481502	+
10504	1427888_a_at	Region	Spna2	20740	2	29927373	29963613	+
10505	1454675_at	Region	Thra	21833	11	98562960	98586198	+
10506	1452843_at	Region	Il6st	16195	13	108856634	108899366	+
10507	1437528_x_at	Region	A730017C20Rik	225583	18	59287736	59302184	+
10508	1455760_at	Region	None	None	8	104663942	104664976	+
10509	1429761_at	Region	Rtn1	104001	12	69054243	69252428	-
10510	1451215_at	Region	1190002C06Rik	73137	18	57571393	57606175	+
10511	1437614_x_at	Region	Zdhhc14	224454	17	5400177	5659646	+
10512	1452888_at	Region	1110034G24Rik	73747	2	132192859	132264907	+
10513	1439479_at	Region	Lct	226413	1	128127755	128156651	-
10514	1451075_s_at	Region	Ctdsp2	52468	10	126728331	126732688	+
10515	1437503_a_at	Region	MGI:1915044	66940	9	109085219	109104358	+
10516	1428848_a_at	Region	Macf1	11426	4	122378001	122711301	-
10517	1456134_x_at	Region	Yif1	68090	19	4877343	4881646	+
10518	1427123_s_at	Region	Copg2as2	54158	6	30846849	30849105	+
10519	1419630_a_at	Region	Trim11	94091	11	58703745	58717087	+
10520	1417251_at	Region	Palmd	114301	3	115687722	115739898	-
10521	1418829_a_at	Region	Eno2	13807	6	125416602	125426275	-
10522	1426799_at	Region	Rab8b	235442	9	66968816	67043633	-
10523	1427929_a_at	Region	Pdxk	216134	10	78549713	78575725	-
10524	1428991_at	Region	Hrasls	27281	16	27998437	28019272	+
10525	1427407_s_at	Region	Trip11	109181	12	97280506	97358629	-
10526	1438878_at	Region	6430537K16Rik	320480	17	60539632	60540356	-
10527	1415874_at	Region	Spry1	24063	3	37103574	37108195	+
10528	1459197_at	Region	None	None	15	92329361	92353854	+
10529	1439117_at	Region	Clmn	94040	12	100215893	100309212	-
10530	1459746_at	Region	R3hdm	226412	1	127946018	128080726	+
10531	1455321_at	Region	Ddhd1	114874	14	40672980	40701448	-
10532	1458534_at	Region	D13Bwg1146e	52882	13	101166216	101269810	-
10533	1449738_s_at	Region	Exosc8	69639	3	54362900	54369613	-

10534	1417673_at	Region	Grb14	50915	2	64767878	64878166	-
10535	1449142_a_at	Region	Yipf5	67180	18	40429229	40443156	-
10536	1433446_at	Region	Hmgcs1	208715	13	114526	140291	-
10537	1447927_at	Region	Mpa2l	100702	5	0	5299	-
10538	1435959_at	Region	Arhgap15	76117	2	43681013	44318825	+
10539	1440056_at	Region	None	None	19	41142196	41142866	-
10540	1422980_a_at	Region	Bet1l	54399	7	135255293	135258149	-
10541	1427934_at	Region	2610208E05Rik	108755	4	33079058	33081043	+
10542	1434887_at	Region	Freq	14299	2	31178085	31227634	+
10543	1425194_a_at	Region	6330577E15Rik	67788	19	47283059	47286799	+
10544	1448808_a_at	Region	Nme2	18103	11	93770901	93777094	-
10545	1417879_at	Region	Nenf	66208	1	191043062	191054383	-
10546	1437308_s_at	Region	F2r	14062	13	91791978	91808621	-
10547	1435706_at	Region	None	None	17	28025564	28026466	-
10548	1456436_x_at	Region	Rps20	67427	4	3761619	3762746	-
10549	1416042_s_at	Region	Nasp	50927	4	115560514	115586846	-
10550	1423150_at	Region	Sgne1	20394	2	113399605	113452364	-
10551	1417872_at	Region	Fhl1	14199	X	51509047	51548058	+
10552	1452616_s_at	Region	Ssbp1	381760	6	40612256	40622665	+
10553	1416092_a_at	Region	Mtap4	17758	9	109957034	110109905	+
10554	1415947_at	Region	Creg1	433375	1	165685557	165697081	+
10555	1449429_at	Region	Fkbp1b	14226	12	4005389	4013790	-
10556	1422596_at	Region	C030019F02Rik	58237	2	180651730	180671652	-
10557	1417355_at	Region	Peg3	18616	7	5909405	5933864	-
10558	1427674_a_at	Region	Sez6	20370	11	77656602	77704707	+
10559	1448153_at	Region	Cox5a	12858	9	57635235	57646353	+
10560	1451130_at	Region	2010315L10Rik	67023	8	70517056	70519559	+
10561	1421595_at	Region	9630031F12Rik	58227	5	44288471	44397958	-
10562	1426389_at	Region	Camk1d	227541	2	5214190	5631787	-
10563	1436350_at	Region	D430039N05Rik	241520	2	83510477	83579107	+
10564	1449084_s_at	Region	Sh3d19	27059	3	85819530	85872864	+
10565	1429159_at	Region	4631408O11Rik	66693	2	10169436	10172539	+
10566	1420362_a_at	Region	Biklk	12124	15	83575461	83593234	+
10567	1423392_at	Region	Clic4	29876	4	134095226	134154017	-
10568	1448157_s_at	Region	Rpl10	110954	X	68931512	68933781	+
10569	1417847_at	Region	Ulk2	29869	11	61501302	61580259	-
10570	1447034_at	Region	8030448K23Rik	320826	13	95096672	95097144	-
10571	1423135_at	Region	Thy1	21838	9	44034809	44037433	+
10572	1424319_at	Region	Oraov1	72284	7	139327211	139333154	+
10573	1417441_at	Region	Dnajc12	30045	10	63352440	63374606	+
10574	1430552_a_at	Region	Sbf1	77980	15	89339395	89368172	-
10575	1427405_s_at	Region	Rab11fip5	52055	6	85684683	85724625	-
10576	1446265_at	Region	Dnm3	103967	1	161896669	162387092	-
10577	1418738_at	Region	Scn1b	20266	7	26526157	26537211	-
10578	1416842_at	Region	Gstm5	14866	3	107691596	107694427	+
10579	1438494_at	Region	Hrh1	15465	6	114980341	115054903	+
10580	1429772_at	Region	Plxna2	18845	1	194358488	194552987	+
10581	1422505_at	Region	Chrac1	93696	15	73117409	73121072	+
10582	1436994_a_at	Region	Hist1h1c	50708	13	23218684	23220243	+
10583	1442080_at	Region	Creb3l2	208647	6	37465296	37576712	-
10584	1424572_a_at	Region	H2afy	26914	13	54691912	54753184	-
10585	1437230_at	Region	Kcna1	16485	6	127312007	127316405	-
10586	1424677_at	Region	Cyp2j9	74519	4	95543502	95566558	-
10587	1416727_a_at	Region	Cyb5	109672	18	85019156	85047605	+
10588	1433688_x_at	Region	Rpl14	67115	9	120583908	120587044	+
10589	1452893_s_at	Region	1110065P19Rik /// 2310040A07Rik	68919	4	41776994	41779094	-
10590	1426397_at	Region	Tgfbr2	21813	9	116074629	116162211	-
10591	1436468_at	Region	Zdhhc8	27801	16	16994248	17006052	-
10592	1453098_at	Region	Gria2	14800	3	80417443	80531582	-
10593	1437219_at	Region	A330103N21Rik	77773	7	61907803	61908932	+
10594	1418840_at	Region	Pdcd4	18569	19	53482344	53508727	+
10595	1454947_a_at	Region	BC002236	79560	11	44207410	44223282	-
10596	1440759_at	Region	None	None	3	106910406	106910834	+
10597	1416411_at	Region	Gstm2	14863	3	107777444	107782176	-

10598	1452184_at	Region	Ndufb9	66218	15	58944967	58950646	+
10599	1417031_at	Region	2310028N02Rik	66950	1	191062230	191089190	+
10600	1452227_at	Region	2310045A20Rik	231238	5	51917296	52023458	-
10601	1443612_at	Region	None	None	2	110280744	110281219	-
10602	1419077_at	Region	Mpp3	13384	11	101820749	101847992	-
10603	1458394_at	Region	Slc6a17	229706	3	107263377	107313847	-
10604	1433517_at	Region	None	None	1	92464637	92469415	-
10605	1420124_s_at	Region	Tcta	102791	9	108372719	108375712	-
10606	1451693_a_at	Region	Fgf12	14167	16	26943562	27228998	-
10607	1428259_at	Region	2310075M15Rik	69675	12	26573857	26588960	+
10608	1437947_x_at	Region	None	None	1	32009779	32010021	+
10609	1418492_at	Region	Grem2	23893	1	174766769	174854520	-
10610	1416303_at	Region	Litaf	56722	16	7206983	7214387	-
10611	1417255_at	Region	Wdtc2	106633	17	153401	174334	+
10612	1420875_at	Region	Ptk9	19230	15	94647782	94658539	-
10613	1417676_a_at	Region	Ptpro	19277	6	138044416	138255228	+
10614	1448851_a_at	Region	Dnajc5	13002	2	181237462	181269206	+
10615	1416546_a_at	Region	Rpl6	19988	5	120357217	120368957	+
10616	1420932_at	Region	Mapk8	26419	14	31516453	31580724	-
10617	1435338_at	Region	Cdk6	12571	5	3350317	3528230	+
10618	1434222_at	Region	Sipa1l1	217692	12	79172130	79311045	+
10619	1419021_at	Region	Mcf2	109904	X	54810674	54902358	-
10620	1416021_a_at	Region	Fabp5	16592	3	10011881	10015886	+
10621	1424410_at	Region	Ttc8	76260	12	94347654	94410409	+
10622	1421142_s_at	Region	Foxp1	108655	6	99395348	99630926	-
10623	1437298_at	Region	5730509C05Rik	68524	11	98684896	98724607	+
10624	1417062_at	Region	2810037C14Rik	67211	5	20098348	20114956	+
10625	1456098_a_at	Region	Elmo2	140579	2	164744764	164783212	-
10626	1453570_x_at	Region	Bet1l	54399	7	135255293	135258149	-
10627	1440970_at	Region	None	None	16	32777397	32778894	-
10628	1438060_at	Region	Npas3	27386	12	50702581	50824492	+
10629	1450878_at	Region	Sri	109552	5	8054494	8076270	+
10630	1428978_at	Region	2900009J20Rik	72876	12	69644051	69646703	+
10631	1439396_x_at	Region	Gpd1	14555	15	99774809	99782224	+
10632	1417558_at	Region	Fyn	14360	10	39440670	39635213	+
10633	1435731_x_at	Region	Stag1	20842	9	100545170	100860091	+
10634	1437246_x_at	Region	Rps6 /// LOC214738 /// LOC216036 /// LOC236932 /// LOC434404 /// LOC545640	20104	4	85840854	85843549	-
10635	1452444_at	Region	Napb	17957	2	148151593	148189356	-
10636	1423288_s_at	Region	Cbln1	12404	8	86752336	86755765	-
10637	1418643_at	Region	Tspan13	66109	12	32620734	32648498	-
10638	1436699_x_at	Region	Rpl18	19899	6	129003525	129004136	+
10639	1445804_at	Region	9630020C08Rik	327819	10	109788315	110103340	-
10640	1439725_at	Region	Ptprt	19281	2	160984592	162118116	-
10641	1436335_at	Region	A930027K05Rik	269615	4	153475739	153503344	-
10642	1429682_at	Region	4930431B09Rik	74645	3	99897447	99915097	-
10643	1455661_at	Region	None	None	5	89665368	89665916	+
10644	1422517_a_at	Region	Znrd1	66136	17	34713862	34717932	-
10645	1428545_at	Region	0610007L01Rik	71667	5	129392160	129415495	+
10646	1452351_at	Region	C030027K23Rik	77419	14	51170381	51181942	+
10647	1448392_at	Region	Sparc	20692	11	55147342	55172707	-
10648	1435013_at	Region	MGC60818	433956	5	138148281	138184566	+
10649	1420401_a_at	Region	Ramp3	56089	11	6553321	6572263	+
10650	1437450_x_at	Region	2700060E02Rik	68045	14	18174611	18186532	-
10651	1417174_at	Region	1810021J13Rik	66279	9	37149795	37164750	+
10652	1437350_at	Region	MGI:2179725	246257	11	74901601	74904466	-
10653	1424699_at	Region	4921511K06Rik	232664	6	29443258	29474046	+
10654	1422845_at	Region	Canx	12330	11	50047308	50078432	-
10655	1459151_x_at	Region	Ifi35	70110	11	101269568	101279549	+
10656	1448283_a_at	Region	Uble1b	50995	7	29294488	29322320	-
10657	1427427_at	Region	None	None	2	112254595	112259929	-

10658	1458428_at	Region	Kcnt1	227632	2	25848573	25849383	+
10659	1436849_x_at	Region	Gaa	14387	11	119089137	119106545	+
10660	1423592_at	Region	Rock2	19878	12	16262121	16345038	+
10661	1426964_at	Region	3110003A17Rik	73112	10	17935737	17974930	-
10662	1423803_s_at	Region	Gltscr2	68077	7	39	7752	-
10663	1420962_at	Region	Hapln2	73940	3	87765928	87771304	-
10664	1423893_x_at	Region	Apbb1	11785	7	99670922	99693881	-
10665	1458440_at	Region	Specc1	432572	11	61858013	61865361	+
10666	1454761_at	Region	BC005764	216152	10	79987416	79997269	-
10667	1424348_at	Region	1110007A13Rik	210711	7	122745901	122814730	-
10668	1431680_a_at	Region	Ptprk	19272	10	28105024	28627423	+
10669	1436046_x_at	Region	Rpl29 /// LOC433350 /// LOC433941	19944	9	106423093	106425121	+
10670	1438706_at	Region	6430517E21Rik	240843	1	158150731	158260041	-
10671	1421998_at	Region	Tor3a	30935	1	156560153	156579875	-
10672	1436231_at	Region	2900052N01Rik	73040	9	46937700	46938426	+
10673	1451140_s_at	Region	Prkag2	108099	5	23326651	23569979	-
10674	1424034_at	Region	Rora	19883	9	69474948	69510983	+
10675	1456202_at	Region	6.33E+19	207393	15	78721307	78725742	-
10676	1418097_a_at	Region	Tslpr	57914	5	108635082	108641057	-
10677	1434158_at	Region	Gmds	218138	13	31299208	31818166	-
10678	1449841_at	Region	Kif3a	16568	11	53320244	53354795	+
10679	1455531_at	Region	A930031D07Rik	213006	1	131888602	131919751	-
10680	1435237_at	Region	2310009A05Rik	66364	9	73172461	73175337	+
10681	1443964_at	Region	Tmie	20776	9	110907343	110921380	-
10682	1438667_at	Region	5730410E15Rik	319613	15	44706659	44733620	-
10683	1423399_a_at	Region	Yaf2	67057	15	93350109	93403125	-
10684	1416530_a_at	Region	Pnp	18950	14	46039165	46048052	+
10685	1418180_at	Region	Sp1	20683	15	102466747	102493098	+
10686	1428573_at	Region	Chn2	69993	6	54417146	54446135	+
10687	1420534_at	Region	Gucy1a3	60596	3	81821497	81874847	-
10688	1435367_at	Region	Mapk4	225724	18	74162718	74205506	-
10689	1451146_at	Region	Zfp386	56220	12	111816578	111828697	+
10690	1416551_at	Region	Atp2a2	11938	5	121605864	121654245	-
10691	1456953_at	Region	None	None	1	24502339	24503559	-
10692	1440999_at	Region	Zfp697	242109	3	97868692	97916581	+
10693	1436719_at	Region	Slc35f1	215085	10	52933079	53356820	+
10694	1448991_a_at	Region	Ina	226180	19	46566002	46575960	+
10695	1449506_a_at	Region	Eef1d	66656	15	75944869	75959409	-
10696	1419618_at	Region	Bbox1	170442	2	109888870	109929512	-
10697	1416301_a_at	Region	Ebf1	13591	11	44370971	44758012	+
10698	1420388_at	Region	Prss12	19142	3	122238559	122298307	+
10699	1449491_at	Region	Card10	105844	15	78826422	78854328	-
10700	1434461_at	Region	2610041B18Rik	69930	7	37370962	37388012	-
10701	1456527_at	Region	Hecw1	94253	13	13672338	13968180	-
10702	1440132_s_at	Region	Prkar1b	19085	5	138015364	138127749	-
10703	1425539_a_at	Region	Rtn3	20168	19	7139746	7197134	-
10704	1455346_at	Region	Masp1	17174	16	22233196	22304246	-
10705	1426328_a_at	Region	Scn3b	235281	9	40219687	40241396	+
10706	1426979_at	Region	AW228700	100951	5	122617914	122629974	+
10707	1454814_s_at	Region	AU021838	328099	12	53961902	53972469	+
10708	1424403_a_at	Region	D5Bwg0860e	52822	5	87850046	87908952	+
10709	1427271_at	Region	Btbd15	235132	9	30949477	30989044	+
10710	1454699_at	Region	Sesn1	140742	10	42001833	42014167	+
10711	1434628_a_at	Region	Rhpn2	52428	7	30518501	30557703	+
10712	1427035_at	Region	Slc39a14	213053	14	64619456	64667414	-
10713	1438705_at	Region	Cbfa2t3h	12398	8	122011527	122080570	-
10714	1436586_x_at	Region	Rps14	20044	18	60999975	61003870	+
10715	1424065_at	Region	Edem1	192193	6	109331841	109362540	+
10716	1449229_a_at	Region	Cdkl2	53886	5	91341903	91378846	-
10717	1454995_at	Region	Ddah1	69219	3	144733606	144866062	+
10718	1417626_at	Region	Usmg4	83679	3	97176102	97177020	-
10719	1441101_at	Region	Hecw1	94253	13	13672338	13968180	-
10720	1422313_a_at	Region	Igfbp5	16011	1	73159719	73176568	-

10721	1416023_at	Region	Fabp3	14077	4	129335891	129342576	+
10722	1418399_at	Region	Kctd9	105440	14	62244836	62270712	+
10723	1417919_at	Region	Ppp1r7	66385	1	93171136	93195109	+
10724	1429579_at	Region	6330407I18Rik	70710	9	3808267	3809504	+
10725	1448260_at	Region	Uchl1	22223	5	65441631	65452577	+
10726	1433720_s_at	Region	MGI:2143558	103172	10	76037313	76039474	+
10727	1456137_at	Region	Nrxn3	18191	12	84906378	85735809	+
10728	1434727_at	Region	2900042E01Rik	72927	9	37309145	37326635	+
10729	1422825_at	Region	MGI:1351330	27220	13	96090664	96092548	-
10730	1436990_s_at	Region	MGI:2143558	103172	10	76037313	76039474	+
10731	1425266_a_at	Region	Rap1gds1	229877	3	137815145	137964306	-
10732	1436656_at	Region	BC062109	231503	5	99096511	99178447	-
10733	1427946_s_at	Region	Dpyd	99586	3	117335295	118207632	+
10734	1433432_x_at	Region	Rps12	20042	10	58921849	58922343	+
10735	1451751_at	Region	Ddit4l	73284	3	136513537	136518197	+
10736	1460644_at	Region	Bckdk	12041	7	121953913	121958419	+
10737	1437455_a_at	Region	Btg1	12226	10	96594653	96597253	+
10738	1423824_at	Region	5031439A09Rik	68151	3	4597	6180	+
10739	1420596_at	Region	Cacng2	12300	15	78046913	78171273	-
10740	1420979_at	Region	Pak1	18479	7	91889708	91958070	+
10741	1439313_at	Region	Lphn3	319387	5	80275281	81049432	+
10742	1428669_at	Region	Bmyc	107771	2	25638994	25640056	+
10743	1426324_at	Region	H2-D1	14964	17	186801	249200	-
10744	1434315_at	Region	9130020G22Rik	74552	4	134332771	134375796	-
10745	1427762_x_at	Region	Hist1h2bp	319188	13	21267579	21267959	+
10746	1428793_at	Region	Slc36a1	215335	11	54957211	54986745	+
10747	1436691_x_at	Region	Prdx1	18477	4	115644504	115658905	+
10748	1423987_at	Region	Cdc26	66440	4	61485774	61495742	-
10749	1442379_at	Region	MGC99845	574403	11	34316333	34317435	+
10750	1448940_at	Region	Trim21	20821	7	96666869	96674091	-
10751	1435547_at	Region	None	None	16	12152379	12153526	+
10752	1433505_a_at	Region	Lrrc5	231549	5	104762811	104877194	+
10753	1428895_at	Region	3222401M22Rik	74013	1	55471318	55527941	-
10754	1415965_at	Region	Scd1	20249	19	43938861	43951959	-
10755	1416204_at	Region	Gpd1	14555	15	99774809	99782224	+
10756	1434741_at	Region	None	None	13	37473505	37474179	+
10757	1441636_at	Region	---	436334	16	4856428	4856935	+
10758	1427136_s_at	Region	Sfrs12	218543	13	99959715	99982622	-
10759	1417604_at	Region	Camk1	52163	6	113901957	113911755	-
10760	1451415_at	Region	1810011O10Rik	69068	8	23192443	23193773	-
10761	1421861_at	Region	Clstn1	65945	4	148079000	148140261	+
10762	1428745_a_at	Region	2310003L22Rik	69487	2	139684883	139717485	+
10763	1456603_at	Region	1500005K14Rik	76566	11	75744852	75746368	-
10764	1426615_s_at	Region	Ndrg4	234593	8	94984302	94996288	+
10765	1415800_at	Region	Gja1	14609	10	56627159	56640230	+
10766	1452587_at	Region	Actr2	66713	11	19957097	20007705	-
10767	1457670_s_at	Region	Lmna	16905	3	88225013	88247171	-
10768	1415790_at	Region	BC002236	79560	11	44207410	44223282	-
10769	1434755_at	Region	Coro2b	235431	9	62539533	62656961	-
10770	1426646_at	Region	9130011J15Rik	66818	8	71716686	71721857	-
10771	1428081_at	Region	Klhl21	242785	4	150500450	150510042	+
10772	1452213_at	Region	Tex2	21763	11	106323228	106434019	-
10773	1439913_at	Region	1810053B01Rik	75658	2	163506945	163507507	+
10774	1445297_at	Region	None	None	16	35929400	35930081	-
10775	1429290_at	Region	Cbx6	494448	15	79878420	79886557	-
10776	1425519_a_at	Region	Ii	16149	18	61029204	61037978	+
10777	1451363_a_at	Region	2010308M01Rik	72121	3	106281879	106296871	+
10778	1417398_at	Region	Rras2	66922	7	107899626	107913462	-
10779	1417569_at	Region	Ncald	52589	15	37371112	37797344	-
10780	1450693_at	Region	None	None	10	4588459	4678958	+
10781	1435016_at	Region	Als2cr3	70827	1	59205320	59251368	-
10782	1426929_at	Region	Brunol4	108013	18	25707613	25982655	-
10783	1427887_at	Region	2610304G08Rik	70470	2	157485763	157534289	+
10784	1450355_a_at	Region	Capg	12332	6	72878054	72896490	+
10785	1430136_at	Region	Grm3	108069	5	9495848	9738738	-

10786	1453129_a_at	Region	Rgs12	71729	5	33437125	33521273	+
10787	1418970_a_at	Region	Bcl10	12042	3	144898767	144908672	+
10788	1446421_at	Region	Schip1	30953	3	68244719	68298773	+
10789	1450052_at	Region	Kif2a	16563	13	103169548	103230223	-
10790	1452533_at	Region	None	None	2	112261367	112271614	-
10791	1453740_a_at	Region	Ccnl2	56036	4	154304851	154316905	+
10792	1416453_x_at	Region	Rps12 /// LOC546370 /// LOC546371 /// LOC546372 /// LOC546373	20042	10	58921849	58922343	+
10793	1439284_at	Region	E230025E14Rik	320932	1	119296466	119297552	-
10794	1436251_at	Region	Pde1c	18575	6	56226106	56506844	-
10795	1419922_s_at	Region	Atrnl1	226255	19	57196770	57719074	+
10796	1433826_at	Region	AW212607	241732	2	152679307	152682319	-
10797	1450244_a_at	Region	Map4k2	26412	19	6130040	6142317	+
10798	1458501_at	Region	Vapb	56491	2	173198044	173239833	+
10799	1450916_at	Region	Stau2	29819	1	16527085	16558282	-
10800	1455591_at	Region	None	None	4	62228874	62230900	+
10801	1458602_at	Region	Bbx	70508	16	49039981	49275616	-
10802	1421349_x_at	Region	1500001H12Rik	57754	7	135828437	135831408	-
10803	1457479_at	Region	None	None	1	72670519	72671275	+
10804	1428680_at	Region	Cds1	74596	5	100785901	100844694	+
10805	1426485_at	Region	Ubxd2	67812	1	128087153	128120583	+
10806	1425468_at	Region	Plp1	18823	X	130367715	130382986	+
10807	1422589_at	Region	Rab3a	19339	8	69909984	69913935	+
10808	1423455_at	Region	Ptma	19231	1	86331905	86336327	+
10809	1437707_at	Region	BC019561	225655	18	67695686	67710555	+
10810	1417071_s_at	Region	Cyp4v3	102294	8	44254051	44281445	-
10811	1430656_a_at	Region	2210409M21Rik	70396	1	53652158	53660213	-
10812	1451122_at	Region	Idi1	319554	13	8843118	8849517	+
10813	1440147_at	Region	Lgi2	246316	5	51348717	51377137	-
10814	1435754_at	Region	D6Bwg1452e	242610	6	107189346	107259993	-
10815	1450875_at	Region	Gpr37	14763	6	25712863	25735484	-
10816	1426501_a_at	Region	MGI:2182965	211550	3	126673949	126682478	+
10817	1433867_at	Region	1810030O07Rik	69155	X	10893824	10912486	-
10818	1456388_at	Region	Atp11a	50770	8	12135859	12243808	+
10819	1439466_s_at	Region	C77604	231889	5	144178833	144186510	+
10820	1429004_at	Region	Phip	83946	9	83198955	83305205	-
10821	1454653_at	Region	A730016F12Rik	211232	6	113845771	113872593	+
10822	1423250_a_at	Region	Tgfb2	21808	1	186121756	186210661	-
10823	1436274_at	Region	None	None	9	87528031	87528983	+
10824	1415740_at	Region	Psmc5	19184	11	106077274	106084201	+
10825	1427185_at	Region	Mef2a	17258	7	61121784	61125517	-
10826	1429126_at	Region	2600001M11Rik	76531	2	5786620	5788325	+
10827	1428816_a_at	Region	Gata2	14461	6	88634163	88642525	+
10828	1425180_at	Region	Sgip1	73094	4	101719268	101930082	+
10829	1435417_at	Region	AI464131	329828	4	41634456	41641908	-
10830	1454691_at	Region	Nrxn1	18189	17	87900413	88568779	-
10831	1416282_at	Region	Psmc3	19182	2	90758831	90764177	+
10832	1421841_at	Region	Fgfr3	14184	5	32210377	32225943	+
10833	1436265_at	Region	6330405H19	330153	5	107447072	107449021	+
10834	1421918_at	Region	Anp32a	11737	9	62461464	62498838	+
10835	1455698_at	Region	Tloc1	69276	3	30204876	30231459	+
10836	1425863_a_at	Region	Ptpro	19277	6	138044416	138255228	+
10837	1453837_at	Region	6330500D04Rik	193385	13	24201506	24211957	+
10838	1416754_at	Region	Prkar1b	19085	5	138015364	138127749	-
10839	1439808_at	Region	A130090K04Rik	320495	10	3616345	3617561	+
10840	1451349_at	Region	BC020077	230500	4	98868481	98871688	+
10841	1434866_x_at	Region	Cpt1a	12894	19	3137883	3174426	+
10842	1452621_at	Region	Pcbd2	72562	13	54343926	54393388	+
10843	1453003_at	Region	Sorl1	20660	9	41927330	42082982	-
10844	1440153_at	Region	---	433045	16	84023320	84061591	+
10845	1453576_at	Region	Nipbl	71175	15	8078809	8232047	-
10846	1417959_at	Region	Pdlim7	67399	13	54123331	54129992	-

10847	1450931_at	Region	Dock9	105445	14	116113166	116206323	-
10848	1436797_a_at	Region	Surf4	20932	2	26852201	26866090	-
10849	1443558_s_at	Region	C630002B14Rik	103466	10	86809415	86813050	+
10850	1423265_at	Region	Minpp1	17330	19	31811243	31840828	+
10851	1424396_a_at	Region	Asrgl1	66514	19	8308266	8332113	-
10852	1421845_at	Region	Golph3	66629	15	12132755	12162746	+
10853	1425620_at	Region	Tgfbr3	21814	5	106170128	106359608	-
10854	1454858_x_at	Region	3300001H21Rik	70152	15	100362355	100371886	+
10855	1436709_at	Region	C230096C10Rik	230866	4	138233859	138259973	+
10856	1423078_a_at	Region	Sc4mol	66234	8	63787318	63802751	-
10857	1445144_at	Region	None	None	NONE	NONE	NONE	NONE
10858	1443290_at	Region	None	None	9	35449110	35449758	-
10859	1418983_at	Region	Inadl	12695	4	97370899	97694676	+
10860	1419719_at	Region	Gabrb1	14400	5	70477380	70918453	+
10861	1449383_at	Region	Adssl1	11565	12	108099476	108120727	+
10862	1451012_a_at	Region	Csda	56449	6	132143594	132167186	-
10863	1448135_at	Region	Atf4	11911	15	80308201	80310204	+
10864	1425111_at	Region	Sorcs3	66673	19	47757239	48356719	+
10865	1430172_a_at	Region	Cyp4f16 /// LOC545195	545195	17	30453789	30500640	+
10866	1426322_a_at	Region	Kcnmb2	72413	3	31314154	31608812	+
10867	1438154_x_at	Region	2610002J02Rik	67513	4	153742328	153749046	+
10868	1457305_at	Region	Btrc	12234	19	44911191	45076729	+
10869	1424306_at	Region	Elovl4	83603	9	84108461	84135869	-
10870	1429830_a_at	Region	Cd59a	12509	2	103800607	103820103	+
10871	1456967_at	Region	Trim66	330627	7	103304494	103346871	-
10872	1437972_s_at	Region	Sf3b5	66125	10	12890149	12890854	+
10873	1417359_at	Region	Mfap2	17150	4	139891964	139897234	+
10874	1433542_at	Region	Inpp5f	101490	7	122660050	122745896	+
10875	1426540_at	Region	2310067E08Rik	71946	9	14189509	14216547	-
10876	1434487_at	Region	Mef2d	17261	3	87886291	87912587	+
10877	1442288_at	Region	Anxa6	11749	11	54731952	54786255	-
10878	1422550_a_at	Region	Mtap6	17760	7	93358278	93425764	+
10879	1416824_at	Region	B230118H07Rik	68170	2	101265464	101333669	-
10880	1421190_at	Region	Gabrb3	14402	7	51863803	52099137	+
10881	1428478_at	Region	2700029E10Rik	72549	14	64861612	64864921	+
10882	1451302_at	Region	1110012L19Rik	68618	X	65054404	65057907	+
10883	1427246_at	Region	Magi1	14924	6	94131999	94737301	-
10884	1418862_at	Region	Echdc3	67856	2	6105737	6130266	-
10885	1452894_at	Region	Elavl4	15572	4	109165070	109310814	-
10886	1460699_at	Region	Rps27	57294	3	90023087	90024065	-
10887	1422823_at	Region	Eps8	13860	6	138270172	138331399	-
10888	1446506_at	Region	B930041G04	245643	X	133926910	133939479	+
10889	1417356_at	Region	Peg3	18616	7	5909405	5933864	-
10890	1439336_at	Region	Tcf4	21413	18	69575005	69915006	+
10891	1416013_at	Region	Pld3	18807	7	22908314	22928711	-
10892	1415713_a_at	Region	Ddx24	27225	12	98853463	98871260	-
10893	1436674_at	Region	D11Ertd333e	68066	11	102224234	102228559	-
10894	1424710_a_at	Region	Gorasp2	70231	2	70359319	70389486	+
10895	1423664_at	Region	Qdpr	110391	5	44192821	44208924	-
10896	1436201_x_at	Region	Mbp	17196	18	82642914	82753380	+
10897	1424409_at	Region	Cldn23	71908	8	34627798	34629616	-
10898	1426851_a_at	Region	Nov	18133	15	54747888	54755722	+
10899	1439779_at	Region	None	None	10	4684375	4685279	+
10900	1417527_at	Region	Ap3m2	64933	8	21541934	21560210	-
10901	1429428_at	Region	Tcf7l2	21416	19	55323190	55515901	+
10902	1423406_at	Region	Sv2a	64051	3	95669059	95683015	+
10903	1418975_at	Region	Nckipsd	80987	9	108854911	108864897	+
10904	1434645_at	Region	C530008M17Rik	320827	5	76085039	76117985	+
10905	1456863_at	Region	Epha4	13838	1	77656420	77802175	-
10906	1438018_at	Region	Hook1	77963	4	94942452	94999350	+
10907	1429681_a_at	Region	Gpsn2	106529	8	82829225	82852112	-
10908	1448595_a_at	Region	Rex3	19716	X	129758911	129760452	-
10909	1448232_x_at	Region	Tuba6	22146	15	99088281	99096040	+
10910	1428394_at	Region	Phyhd1 /// Lrrc8	227696	2	30198365	30214309	+

10911	1422503_s_at	Region	Parp1	11545	1	180523221	180555500	+
10912	1451268_at	Region	Tram1l1	229801	3	123116273	123118424	+
10913	1420506_a_at	Region	Stxbp1	20910	2	32720124	32779288	-
10914	1416097_at	Region	Lrrc4	192198	6	28876498	28878711	-
10915	1424603_at	Region	Sumf1	58911	6	108608573	108687101	-
10916	1444052_at	Region	Flt4	14257	11	49362568	49405070	+
10917	1422484_at	Region	Cycs	13063	6	50708965	50711007	-
10918	1450890_a_at	Region	Abi1	11308	2	22882346	22926763	-
10919	1421046_a_at	Region	Pabpc4 /// LOC432881	230721	4	122310023	122325944	+
10920	1417102_a_at	Region	Ndufb5	66046	3	32145242	32159738	+
10921	1455927_x_at	Region	2510027N19Rik	67711	7	119517375	119541266	-
10922	1416410_at	Region	Pafah1b3	18476	7	20475289	20478195	-
10923	1432273_a_at	Region	Dfy	13349	1	173267162	173268795	-
10924	1436912_at	Region	3110038O15Rik	73120	2	52362896	52364144	-
10925	1423780_at	Region	Hibadh	58875	6	52690560	52784625	-
10926	1417539_at	Region	Slc35a1	24060	4	34802324	34826420	-
10927	1451308_at	Region	Elovl4	83603	9	84108461	84135869	-
10928	1435525_at	Region	Kctd17	72844	15	78479947	78489979	+
10929	1455450_at	Region	Ptpn3	19257	4	57182973	57243817	-
10930	1426074_at	Region	LOC434536	434536	NONE	NONE	NONE	NONE
10931	1433658_x_at	Region	Pcbp4	59092	9	106517872	106528323	+
10932	1457072_at	Region	Bcl11a	14025	11	23972907	24067984	+
10933	1425780_a_at	Region	0610041E09Rik	66074	13	86207541	86225129	+
10934	1435155_at	Region	Cgn	70737	3	94247892	94274753	-
10935	1430183_at	Region	4833414E09Rik	71633	1	168354070	168355687	-
10936	1458721_at	Region	Pcdhga12	93724	18	37929594	37930793	+
10937	1456798_at	Region	9330118A15Rik	319756	4	25147424	25148239	+
10938	1460343_at	Region	Neurl	18011	19	46730295	46810735	+
10939	1438729_at	Region	Sox1	20664	8	11774868	11776043	+
10940	1436393_a_at	Region	Trim37	68729	11	86855069	86948674	+
10941	1455548_at	Region	Dlgap4	228836	2	156157648	156219872	+
10942	1433772_at	Region	Stch	110920	16	74838985	74850259	-
10943	1438868_at	Region	D14Ertd668e	219132	14	53883795	53900507	-
10944	1423097_s_at	Region	Capn7	12339	14	29468562	29503108	+
10945	1428666_at	Region	Nars	70223	18	64732225	64749104	-
10946	1415914_at	Region	Hnrpab	15384	11	51352941	51359688	-
10947	1442393_at	Region	Zfhx1b	24136	2	44919283	45045178	-
10948	1416844_at	Region	Hrmt1l1	15468	10	76308969	76339515	-
10949	1450259_a_at	Region	Stat5a	20850	11	100680491	100706256	+
10950	1455796_x_at	Region	Olfm1	56177	2	28137838	28162624	+
10951	1423630_at	Region	Cygb	114886	11	116466688	116475406	-
10952	1448298_at	Region	Tnk2	51789	16	31477206	31491474	+
10953	1455266_at	Region	Kif5c	16574	2	49551476	49706940	+
10954	1455594_at	Region	Sec6l1	211446	13	70234117	70270772	-
10955	1435854_at	Region	Tmem10	226115	19	40607792	40621485	-
10956	1437985_a_at	Region	2310061I04Rik	69662	17	33611840	33616255	-
10957	1437188_at	Region	Gabbr1	54393	17	34805575	34833793	+
10958	1453373_at	Region	6330532G10Rik	76162	X	81401528	81403058	-
10959	1451121_a_at	Region	Gltscr2	68077	7	39	7752	-
10960	1448102_a_at	Region	Wdr61	66317	9	54834704	54852350	-
10961	1451176_at	Region	D430028G21Rik	228607	2	130747991	130761878	+
10962	1428431_at	Region	2310047A01Rik	71918	14	24086842	24133887	-
10963	1421192_a_at	Region	Itsn1	16443	16	90939437	91069253	+
10964	1443997_at	Region	C130040D06Rik	239031	14	32321209	32325379	-
10965	1448411_at	Region	Wfs1	22393	5	35472619	35495421	-
10966	1418298_s_at	Region	Dpysl4	26757	7	133487649	133503345	+
10967	1450012_x_at	Region	Ywhag	22628	5	134921901	134948016	-
10968	1437236_a_at	Region	Zfp110	65020	7	10828742	10844258	+
10969	1426658_x_at	Region	Phgdh	236539	3	97799380	97826178	-
10970	1424929_a_at	Region	Trim26	22670	17	34551189	34619192	+
10971	1435650_at	Region	Hapln4	330790	8	69237391	69243833	+
10972	1451053_a_at	Region	Mdm1	17245	10	117835760	117862925	+
10973	1435246_at	Region	Paqr8	74229	1	21115565	21163699	+
10974	1448867_at	Region	2310004K06Rik	56786	7	103588707	103605134	-

10975	1417472_at	Region	Myh9	17886	15	77812545	77894062	-
10976	1427915_s_at	Region	Tceb1	67923	1	16827250	16842139	-
10977	1455570_x_at	Region	Cnn3	71994	3	120205773	120237098	+
10978	1427900_at	Region	Pip5kl1	227733	2	32507981	32515941	+
10979	1435771_at	Region	Plcb4	18798	2	135319401	135526921	+
10980	1421018_at	Region	1110018J18Rik	66129	13	61676644	61701775	-
10981	1435509_x_at	Region	Cdk2ap1	13445	5	123516782	123525971	-
10982	1415679_at	Region	Psenen	66340	7	25974557	25975876	-
10983	1429204_at	Region	2900075A18Rik	73047	16	19391361	19393425	-
10984	1455152_at	Region	AI462493	107197	19	8076563	8077471	-
10985	1449286_at	Region	Ntng1	80883	3	109582822	109951623	-
10986	1419169_at	Region	Mapk6	50772	9	75524483	75535986	-
10987	1426509_s_at	Region	Gfap	14580	11	102709766	102718169	-
10988	1422772_at	Region	C1galt1	94192	6	7821663	7848379	+
10989	1423470_at	Region	Ptbp2	56195	3	118493626	118557890	-
10990	1416256_a_at	Region	Tubb5	22154	17	33552072	33555475	-
10991	1455908_a_at	Region	Scpep1	74617	11	88745107	88776519	-
10992	1448358_s_at	Region	Snrpg	68011	6	86805446	86812808	+
10993	1439083_at	Region	Ahi1	52906	10	20882283	21010464	+
10994	1440628_at	Region	None	None	NONE	NONE	NONE	NONE
10995	1437332_at	Region	None	None	7	54789671	54790247	-
10996	1429601_x_at	Region	1110019K23Rik	68621	5	90247014	90341747	+
10997	1448602_at	Region	Pygm	19309	19	6173218	6187146	+
10998	1451099_at	Region	Mbc2	23943	10	128247338	128262949	-
10999	1426233_at	Region	Map2k4	26398	11	65413894	65513947	-
11000	1430667_at	Region	Pcdh10	18526	3	44837486	44841910	+
11001	1417125_at	Region	Ahcy	269378	2	154516629	154531334	-
11002	1452097_a_at	Region	Dusp7	235584	9	106362584	106369022	+
11003	1460668_at	Region	Gal	14419	19	3198611	3203151	-
11004	1445549_at	Region	None	None	16	72127591	72128149	+
11005	1433715_at	Region	Cpne7	102278	8	122498738	122516463	+
11006	1428490_at	Region	C1galt1	94192	6	7821663	7848379	+
11007	1418275_a_at	Region	Elf2	69257	3	50887633	50957953	-
11008	1429475_at	Region	2810457I06Rik	72828	9	40966047	41111032	-
11009	1454941_at	Region	Nmt1	18107	11	102849651	102887194	+
11010	1421937_at	Region	Dapp1	26377	3	136820534	136870453	-
11011	1449158_at	Region	Kcnk2	16526	1	188922773	189058672	-
11012	1450871_a_at	Region	Bcat1	12035	6	145846706	145893811	-
11013	1434143_at	Region	BC060631	234776	8	116271705	116288863	+
11014	1420376_a_at	Region	H3f3b	15081	11	115843386	115845593	-
11015	1448216_at	Region	Syngr3	20974	17	22489447	22494793	-
11016	1438192_s_at	Region	Baz2a	116848	10	127845721	127863571	+
11017	1440954_at	Region	Pbx1	18514	1	168054563	168366387	-
11018	1427320_at	Region	LOC434002	434002	6	30844726	30846521	+
11019	1438781_at	Region	None	None	3	132369443	132369959	-
11020	1427344_s_at	Region	Rasd2	75141	8	74417802	74428505	+
11021	1448578_at	Region	Pafah1b1	18472	11	74399613	74450328	-
11022	1459211_at	Region	Gli2	14633	1	118586114	118804994	-
11023	1435305_at	Region	Ntrk2	18212	13	57448390	57572946	+
11024	1417493_at	Region	Bmi1	12151	2	18719028	18728639	+
11025	1427875_a_at	Region	1100001I22Rik	68436	3	129657460	129660947	-
11026	1421882_a_at	Region	Elavl2	15569	4	90225838	90375056	-
11027	1428762_at	Region	4921526G09Rik	66724	X	80291582	80295111	+
11028	1437920_at	Region	Epha5	13839	5	83317214	83675897	-
11029	1417540_at	Region	Elf1	13709	14	73835241	73936523	+
11030	1436155_at	Region	Nmnat2	226518	1	152958285	153003745	+
11031	1433545_s_at	Region	5730439E10Rik	102632	9	104041180	104105123	+
11032	1436123_at	Region	Bsn	12217	9	108169094	108256167	-
11033	1440880_at	Region	Mppe1	225651	18	67454864	67475164	-
11034	1437796_at	Region	None	None	7	55059532	55060213	-
11035	1438680_at	Region	Auts2	319974	5	130627794	130728707	-
11036	1445676_at	Region	Kcnn2	140492	18	45780779	45906508	+
11037	1448416_at	Region	Mgp	17313	6	137644232	137647601	-
11038	1452366_at	Region	4732435N03Rik	234356	8	67505844	67884445	-
11039	1435811_a_at	Region	Unc50	67387	1	37724660	37733461	+

11040	1421087_at	Region	Per3	18628	4	149497074	149536951	-
11041	1418710_at	Region	Cd59a	12509	2	103800607	103820103	+
11042	1416508_at	Region	Med28	66999	5	44279069	44284371	+
11043	1448319_at	Region	LOC432549	432549	11	40411477	40412835	-
11044	1418453_a_at	Region	Atp1b1	11931	1	164355219	164376238	-
11045	1455448_at	Region	MGI:2677061	269060	19	9444161	9468085	-
11046	1415778_at	Region	Morf4l2	56397	X	130277881	130286548	-
11047	1434921_at	Region	Nr2e1	21907	10	42668662	42690279	-
11048	1418691_at	Region	Rgs9	19739	11	109046444	109119218	-
11049	1416061_at	Region	Tbc1d15	66687	10	114883404	114937196	-
11050	1425554_a_at	Region	Cdc16	69957	8	13147970	13160545	+
11051	1435413_x_at	Region	2700060E02Rik /// LOC545536	545536	3	88320105	88321076	-
11052	1422728_at	Region	Inha	16322	1	75797800	75801077	+
11053	1428211_at	Region	4933406E20Rik	74443	9	108627920	108646375	-
11054	1433693_x_at	Region	Vamp3	22319	4	149539667	149550315	-
11055	1449319_at	Region	MGI:2183426	192199	4	124013543	124036212	+
11056	1436174_at	Region	Atad2	70472	15	58103062	58128331	-
11057	1416388_at	Region	Pip5k2c	117150	10	126932905	126947384	-
11058	1455544_at	Region	Zranb3	226409	1	127796898	127945759	-
11059	1423859_a_at	Region	Ptgds	19215	2	25398874	25401893	-
11060	1419295_at	Region	Creb3l1	26427	2	91687402	91728880	-
11061	1417606_a_at	Region	Calr	12317	8	84108911	84113928	-
11062	1418885_a_at	Region	Idh3b	170718	2	129793165	129798290	-
11063	1423103_at	Region	None	None	3	94441939	94449078	+
11064	1448383_at	Region	Mmp14	17387	14	48948545	48958231	+
11065	1450847_at	Region	Ncbp2	68092	16	30758010	30767552	+
11066	1454965_at	Region	D430039N05Rik	241520	2	83510477	83579107	+
11067	1448888_at	Region	Ppp1r7	66385	1	93171136	93195109	+
11068	1436150_at	Region	1700066J24Rik	76992	4	41656542	41657464	-
11069	1421203_at	Region	Chrna4	11438	2	180739269	180756135	-
11070	1421072_at	Region	Irx5	54352	8	91644914	91648574	+
11071	1448593_at	Region	Wisp1	22402	15	66915598	66947404	+
11072	1456785_at	Region	Crsp2	26896	X	10918689	11001013	-
11073	1428782_a_at	Region	Uqcrc1	22273	9	108982804	108996233	+
11074	1446264_at	Region	None	None	2	6999433	7000004	-
11075	1430514_a_at	Region	2410026K10Rik	66478	NONE	NONE	NONE	NONE
11076	1436992_x_at	Region	None	None	2	88278097	88278289	+
11077	1424268_at	Region	Smox	228608	2	131005796	131039025	+
11078	1456424_s_at	Region	Pltp	18830	2	164296251	164314349	-
11079	1454888_at	Region	Pfdn4	109054	2	170005161	170027799	+
11080	1454868_at	Region	D4Ertd429e	230917	4	148217245	148230336	-
11081	1449885_at	Region	Tmem47	192216	X	75731366	75757118	+
11082	1418083_at	Region	0610009B22Rik	66050	11	51438226	51441475	-
11083	1417753_at	Region	Pkd2	18764	5	103495425	103541936	+
11084	1443179_at	Region	None	None	8	17432035	17432702	-
11085	1457829_at	Region	Clgn	12745	8	82637493	82683940	+
11086	1419869_s_at	Region	Hdlbp	110611	1	93233431	93306299	-
11087	1434671_at	Region	B230337E12Rik	98262	1	119403893	119407079	-
11088	1448553_at	Region	Myh7	140781	14	49487183	49511559	-
11089	1443327_at	Region	D130043K22Rik	210108	13	24325012	24381147	+
11090	1415743_at	Region	Hdac5	15184	11	102016834	102045926	-
11091	1420620_a_at	Region	Rnf13	24017	3	57378718	57475204	+
11092	1428681_at	Region	Gm608	207806	16	43107623	43110794	+
11093	1428785_at	Region	Amotl1	75723	9	14380306	14523126	-
11094	1456595_x_at	Region	Gh	14599	11	106121357	106122899	-
11095	1436661_at	Region	Dpp10	269109	1	123096743	123812573	-
11096	1451908_a_at	Region	Sec14l1	74136	11	116936342	116980314	+
11097	1435867_at	Region	None	None	6	39272948	39274299	-
11098	1455162_at	Region	4922503N01Rik	230603	4	108366002	108403650	+
11099	1421815_at	Region	Epdr2	105298	13	19073208	19101305	-
11100	1448943_at	Region	Nrp1	18186	8	127666747	127814326	+
11101	1421979_at	Region	Phex	18675	X	150819810	151014683	-
11102	1455421_x_at	Region	Clcn1	12723	6	42430564	42458535	+
11103	1460543_x_at	Region	Rpl37a	19981	1	73012971	73015366	+

11104	1416431_at	Region	Tubb6	67951	18	67621399	67633418	+
11105	1460206_at	Region	Grasp	56149	15	101282359	101290906	+
11106	1448321_at	Region	Smoc1	64075	12	77888087	78047656	+
11107	1426599_a_at	Region	Slc2a1	20525	4	118067673	118096232	+
11108	1437290_at	Region	1110001C20Rik	242291	4	4691488	4720452	-
11109	1418384_at	Region	9430083G14Rik	68117	X	106733286	106771915	+
11110	1448960_at	Region	Cxxc5	67393	18	36053483	36085353	+
11111	1455484_at	Region	2600009P04Rik	66572	9	21904794	21906812	-
11112	1438112_at	Region	9430021M05Rik	77288	NONE	NONE	NONE	NONE
11113	1451155_at	Region	Cugbp2	14007	2	6459141	6638885	-
11114	1434228_at	Region	Ppm2c	381511	4	11886768	11892786	-
11115	1447683_x_at	Region	Mettl1	17299	10	126774651	126778180	+
11116	1437259_at	Region	Slc9a2	226999	1	41062726	41066386	+
11117	1436529_at	Region	Gpr123	52389	7	134230082	134274053	+
11118	1436432_at	Region	B230343J05Rik	320320	16	26942002	26943497	-
11119	1455029_at	Region	Kif21a	16564	15	90987323	91102467	-
11120	1441253_at	Region	Rfx3	19726	19	27008207	27251206	-
11121	1421963_a_at	Region	Cdc25b	12531	2	130700932	130712318	+
11122	1434593_at	Region	Eif5a2	208691	3	28198695	28216230	+
11123	1424873_at	Region	Rnf2	19821	1	151325156	151356573	-
11124	1448209_a_at	Region	Slc22a17	59049	14	49423568	49429642	-
11125	1450738_at	Region	Kif21a	16564	15	90987323	91102467	-
11126	1440084_at	Region	AI663975	103819	11	110343456	110344098	+
11127	1426826_at	Region	Rbm16	106583	17	3065810	3160145	+
11128	1447883_x_at	Region	Map1lc3a	66734	2	154733401	154735007	+
11129	1418575_at	Region	Shfdg1	20422	6	6531045	6551349	-
11130	1416669_s_at	Region	Naca	17938	10	127772283	127785343	+
11131	1428922_at	Region	1200009O22Rik	66873	6	53959793	53965019	-
11132	1428989_at	Region	0710001D07Rik	67679	17	28386340	28387472	+
11133	1435028_at	Region	Wdr7	104082	18	63940854	64221039	+
11134	1435659_a_at	Region	Tpi1	21991	6	125467483	125471180	-
11135	1424252_at	Region	Hnrpdl	50926	5	99053360	99057516	-
11136	1425195_a_at	Region	Acat2 /// Acat3	110460	17	11571688	11589509	+
11137	1449832_at	Region	1700091H14Rik	73553	14	3946	5292	+
11138	1455702_at	Region	None	None	12	77196560	77197213	-
11139	1419365_at	Region	Pex11a	18631	7	73535535	73541429	-
11140	1421960_at	Region	Adcy3	104111	12	3286276	3366296	+
11141	1455334_at	Region	C85843	97470	NONE	NONE	NONE	NONE
11142	1436047_at	Region	Gm672	269037	18	75666390	75932739	-
11143	1433672_at	Region	4732479N06Rik	215201	X	127767894	127821879	-
11144	1429218_at	Region	Det1	76375	7	72619581	72639318	-
11145	1420900_a_at	Region	Rab18	19330	18	60692	85718	-
11146	1456765_at	Region	6430511F03	331475	X	96686491	96687590	+
11147	1436743_at	Region	Igsf4d	239857	16	65672212	65964483	-
11148	1451064_a_at	Region	Psat1	107272	19	15129232	15149125	-
11149	1449942_a_at	Region	Ilk	16202	7	99851400	99857574	+
11150	1424680_at	Region	BB146404	103511	10	34152279	34157446	-
11151	1460341_at	Region	Plekhb2	226971	1	35119341	35148911	+
11152	1416706_at	Region	Rpe	66646	1	66993273	67012185	+
11153	1415866_at	Region	AW538196	101869	7	74125404	74140375	-
11154	1434731_x_at	Region	Prdx1	18477	4	115644504	115658905	+
11155	1422489_at	Region	Gcs1	57377	6	83459195	83462587	+
11156	1452456_at	Region	Nrip2	60345	6	129089953	129098438	+
11157	1431844_at	Region	Kcnmb2	72413	3	31314154	31608812	+
11158	1420537_at	Region	Kctd4	67516	14	70297498	70307712	+
11159	1452063_at	Region	2410081M15Rik	73680	4	128380745	128405141	-
11160	1434476_at	Region	Mect1	382056	8	69536831	69594238	-
11161	1423134_at	Region	BC003324	80291	5	123634608	123649578	-
11162	1455061_a_at	Region	Acaa2	52538	18	75014405	75041400	+
11163	1425660_at	Region	Btbd3	228662	2	137792375	137798774	+
11164	1422449_s_at	Region	Rcn2	26611	9	56150016	56166999	+
11165	1426539_at	Region	Usp11	236733	X	18942872	18959479	+
11166	1455482_at	Region	Ap2a2	11772	7	135965073	136035847	+
11167	1425733_a_at	Region	Eps8	13860	6	138270172	138331399	-
11168	1426258_at	Region	Sorl1	20660	9	41927330	42082982	-

11169	1429298_at	Region	Ddah1	69219	3	144733606	144866062	+
11170	1456899_at	Region	1500004F05Rik	69765	8	39251081	39251595	-
11171	1439291_at	Region	None	None	13	15451114	15451849	-
11172	1452721_a_at	Region	2900091E11Rik	67282	10	88172924	88217989	+
11173	1422155_at	Region	Hist2h3c2	97114	3	95725948	95726959	-
11174	1460223_a_at	Region	Epb4.9	13829	14	64919420	64946006	-
11175	1443471_at	Region	Zbtb20	56490	16	42442156	42491541	+
11176	1454646_at	Region	E430026E19Rik	216198	10	84471607	84509015	+
11177	1436803_a_at	Region	Ndufb9	66218	15	58944967	58950646	+
11178	1416036_at	Region	Fkbp1a	14225	2	150999557	151018627	+
11179	1452127_a_at	Region	Ptpn13	19249	5	102496160	102633144	+
11180	1437067_at	Region	Phtf2	68770	5	19211339	19334596	-
11181	1426877_a_at	Region	2610016F04Rik	66923	14	29151150	29207125	+
11182	1455787_x_at	Region	Minpp1	17330	19	31811243	31840828	+
11183	1457632_s_at	Region	Mrg1	17536	2	115376917	115578960	-
11184	1457270_at	Region	B230343A10Rik	320013	11	67412906	67414641	+
11185	1416236_a_at	Region	Eva1	14012	9	45031412	45042867	+
11186	1423251_at	Region	Luc7l2	192196	6	38687728	38745622	+
11187	1448787_at	Region	Moap1	64113	12	98187945	98204543	-
11188	1426275_a_at	Region	Uxs1	67883	1	44044005	44123567	-
11189	1437022_at	Region	D130059P03Rik	320538	6	38570159	38638809	+
11190	1434360_s_at	Region	Ptprg	19270	14	10014966	10698138	+
11191	1426873_s_at	Region	Jup	16480	11	100191706	100218826	-
11192	1418530_at	Region	Nup160	59015	2	90381964	90441077	+
11193	1420760_s_at	Region	Ndrl	17990	15	66953527	66993826	-
11194	1449615_s_at	Region	Hdlbp	110611	1	93233431	93306299	-
11195	1454828_at	Region	AI790205	277463	2	31084496	31148729	+
11196	1449520_at	Region	AI428795	209683	5	110273612	110336823	+
11197	1440413_at	Region	A830006F12Rik	320460	1	71020647	71027097	+
11198	1426899_at	Region	4930451A13Rik	67581	16	56073492	56138746	-
11199	1440565_at	Region	Zbtb20	56490	16	42442156	42491541	+
11200	1419361_at	Region	Ss18	268996	18	14813890	14871175	-
11201	1419646_a_at	Region	Mbp	17196	18	82642914	82753380	+
11202	1416753_at	Region	Prkar1b	19085	5	138015364	138127749	-
11203	1422660_at	Region	Rbm3	19652	X	6379007	6384637	-
11204	1421878_at	Region	Mapk9	26420	11	49599595	49638939	+
11205	1415897_a_at	Region	Mgst1	56615	6	138932495	138948710	+
11206	1450120_at	Region	Scn1a	20265	2	66128285	66206577	-
11207	1450202_at	Region	Grin1	14810	2	25224409	25251141	-
11208	1456532_at	Region	Pdgfd	71785	9	6119514	6328427	+
11209	1428584_a_at	Region	Haghl	68977	17	23588825	23591333	-
11210	1447275_at	Region	Gm1805	241950	3	36783406	36785048	+
11211	1417491_at	Region	Ctsb	13030	14	57653056	57676512	+
11212	1422429_at	Region	Rnf14	56736	18	38520411	38541525	+
11213	1435609_at	Region	None	None	2	120708685	120709340	-
11214	1456609_at	Region	1810006K23Rik	66259	4	137336512	137339406	+
11215	1440229_at	Region	2310034G01Rik	75579	19	45895538	45896325	-
11216	1450147_at	Region	Nptxr	73340	15	79841252	79857012	-
11217	1457559_at	Region	None	None	NONE	NONE	NONE	NONE
11218	1434925_at	Region	4833424O15Rik	75769	3	116349459	116463339	+
11219	1434389_at	Region	None	None	17	78220202	78221847	-
11220	1444235_at	Region	None	None	1	151769008	151770475	-
11221	1456783_at	Region	9330107J05Rik	319584	1	63606938	63607594	+
11222	1449012_s_at	Region	Fndc4	64339	5	29751186	29753072	-
11223	1431170_at	Region	None	None	3	89071108	89072915	-
11224	1423966_at	Region	Cd99l2	171486	X	66088561	66161136	-
11225	1417152_at	Region	Btbd14a	67991	2	25991155	26054942	-
11226	1417984_at	Region	Ube2v2	70620	16	14321751	14363568	-
11227	1423259_at	Region	Id4	15904	13	47859485	47862094	+
11228	1416711_at	Region	Tbr1	21375	2	61659853	61669514	+
11229	1449848_at	Region	Gna14	14675	19	15659862	15834920	+
11230	1434596_at	Region	AU023766	101257	13	50239213	50239730	-
11231	1421870_at	Region	Trim44	80985	2	102009026	102105530	-
11232	1427523_at	Region	Six3	20473	17	83450633	83453317	+
11233	1417115_at	Region	Map3k12	26404	15	102558068	102565989	-

11234	1436438_s_at	Region	None	None	12	77196560	77197213	-
11235	1433879_a_at	Region	C130032J12Rik	218975	14	42383095	42408162	+
11236	1437101_at	Region	Lats2	50523	14	52225857	52269062	-
11237	1427061_at	Region	Rbbp8	225182	18	11845010	11930602	+
11238	1447364_x_at	Region	Myo1b	17912	1	52050933	52208258	-
11239	1441127_at	Region	None	None	X	61329124	61329999	+
11240	1416150_a_at	Region	Sfrs3	20383	17	26839644	26846716	+
11241	1430024_at	Region	A430107J06Rik	77929	X	93539141	93548434	+
11242	1437099_x_at	Region	Hnrpf	98758	6	118349909	118358058	+
11243	1443954_at	Region	Rad18	58186	6	113184164	113259954	-
11244	1422946_a_at	Region	Dnmt1	13433	9	20784243	20829937	-
11245	1425161_a_at	Region	5730502D15Rik	67976	15	89127929	89138928	+
11246	1455418_at	Region	None	None	10	53518544	53519695	-
11247	1429586_at	Region	4930558N01Rik	75840	7	92747445	92749195	+
11248	1417637_a_at	Region	Hmg20b	15353	10	81481529	81485887	-
11249	1424676_s_at	Region	Sec14l4	103655	11	3926569	3942810	+
11250	1452728_at	Region	Kirrel3	67703	9	34948894	34950352	+
11251	1441722_at	Region	Rnpc2	170791	2	155604575	155637077	-
11252	1445473_at	Region	LOC544932	544932	13	51063134	51091231	-
11253	1417691_at	Region	Bin3	57784	14	64416073	64454195	+
11254	1425403_at	Region	Dnm3	103967	1	161896669	162387092	-
11255	1436159_at	Region	Usp32	237898	11	84712435	84833928	-
11256	1434813_x_at	Region	Wars	22375	12	104331027	104364190	-
11257	1449303_at	Region	Sesn3	75747	9	14111292	14161413	+
11258	1419606_a_at	Region	Tnnt1	21955	7	3735769	3759685	-
11259	1454867_at	Region	LOC433938	433938	5	110439114	110506716	+
11260	1451125_at	Region	BC017133	232164	6	84150399	84175677	-
11261	1445862_at	Region	BC031575	223989	16	12874640	12899363	-
11262	1417430_at	Region	Cdr2	12585	7	114832607	114857851	-
11263	1429097_at	Region	C030044C12Rik	77483	8	82331940	82333406	+
11264	1460580_at	Region	Pcnx	54604	12	78720867	78861705	+
11265	1448399_at	Region	Tax1bp1	52440	6	52858073	52910809	+
11266	1456981_at	Region	Tmc7	209760	7	112411701	112460543	-
11267	1420559_a_at	Region	Shox2	20429	3	66645577	66654082	-
11268	1418750_at	Region	LOC546318 /// LOC547400	546318	X	68417758	68433166	+
11269	1435382_at	Region	Ndn	17984	7	56122085	56123665	+
11270	1423160_at	Region	Spred1	114715	2	116635298	116693185	+
11271	1438376_s_at	Region	Trim27	19720	13	20660436	20672921	+
11272	1425011_x_at	Region	Stx18	71116	5	36545458	36643042	+
11273	1421255_a_at	Region	Cabp1	29867	5	114278572	114295747	-
11274	1437137_at	Region	6430550H21Rik	245386	X	32718102	32773193	-
11275	1458813_at	Region	Scn5a	20271	9	119479491	119556933	-
11276	1455723_at	Region	D1Ertd448e	52318	1	34717260	34718860	+
11277	1422868_s_at	Region	Gda	14544	19	20622929	20710514	-
11278	1455520_at	Region	Ppp2r5c	26931	12	105961123	106055786	+
11279	1417143_at	Region	Edg2	14745	4	58378433	58496968	-
11280	1448807_at	Region	Hrh3	99296	2	179816431	179821182	-
11281	1427306_at	Region	Ryr1	20190	7	24409764	24534347	-
11282	1442418_at	Region	B930096F20Rik	319332	19	7943380	7944071	+
11283	1448765_at	Region	Fyn	14360	10	39440670	39635213	+
11284	1449110_at	Region	Rhob	11852	12	7813873	7816100	-
11285	1458742_at	Region	Lphn3	319387	5	80275281	81049432	+
11286	1436984_at	Region	Abi2	329165	1	60713087	60784447	+
11287	1452353_at	Region	Gpr155	68526	2	73039256	73084321	-
11288	1438496_a_at	Region	6330505F04Rik	236790	X	51209591	51262553	+
11289	1460674_at	Region	Paqr7	71904	4	133457881	133469713	+
11290	1448501_at	Region	Tspan6	56496	X	127436007	127443302	-
11291	1426818_at	Region	Arrdc4	66412	7	62625680	62633272	-
11292	1446190_at	Region	Dcamkl1	13175	3	54876964	55171014	+
11293	1418332_a_at	Region	Agtpbp1	67269	13	58109493	58216884	-
11294	1420858_at	Region	Pkia	18767	3	7341242	7419938	+
11295	1452771_s_at	Region	Acsl3	74205	1	78992139	79040786	+
11296	1452653_at	Region	Slc25a22	68267	7	135831740	135839606	-
11297	1434088_at	Region	Zfp496	268417	11	59211191	59231540	-

11298	1421193_a_at	Region	Pbx3	18516	2	34104071	34304198	-
11299	1438670_at	Region	Ptpn1	19246	2	167396315	167485652	+
11300	1422431_at	Region	Magee1	107528	X	99721786	99725292	+
11301	1416780_at	Region	Pfkm	18642	15	98187678	98208013	+
11302	1423363_at	Region	Sort1	20661	3	108079874	108157253	+
11303	1415804_at	Region	Cx3cl1	20312	8	94056398	94066599	+
11304	1450990_at	Region	Gpc3	14734	X	46793222	47134717	-
11305	1438651_a_at	Region	Agtrl1	23796	2	84834149	84837672	+
11306	1431658_at	Region	Hist1h4d /// Hist1h4j /// Hist1h4k	319156	13	23061479	23061790	+
11307	1448402_at	Region	Tln1	21894	4	43447441	43472672	-
11308	1426032_at	Region	Nfatc2	18019	2	167988261	168098994	-
11309	1439443_x_at	Region	Tkt	21881	14	28683743	28709316	+
11310	1438641_x_at	Region	1500016O10Rik	68952	7	120873047	120879946	+
11311	1428400_at	Region	2200002K05Rik	69137	9	15076342	15096777	+
11312	1455037_at	Region	Plxna2	18845	1	194358488	194552987	+
11313	1436062_at	Region	Arcn1	213827	9	44730423	44756652	-
11314	1449412_at	Region	1810046J19Rik	103742	11	98258812	98260057	-
11315	1434333_a_at	Region	Prkd2	101540	7	13794089	13821505	+
11316	1434677_at	Region	Hps5	246694	7	40845814	40881026	-
11317	1445929_at	Region	Kcnk2	16526	1	188922773	189058672	-
11318	1438054_x_at	Region	Ppp2r2c	269643	5	35375082	35461590	+
11319	1428288_at	Region	2310051E17Rik	70273	19	22403452	22406756	+
11320	1433554_at	Region	AU022870	231874	5	143029522	143056466	-
11321	1455096_at	Region	None	None	12	91195053	91195851	+
11322	1436822_x_at	Region	Hk1 /// Rpl17 /// LOC432963 /// LOC545252 /// LOC545379	15275	10	62236465	62346218	-
11323	1439455_x_at	Region	Capza1	12340	3	104249759	104291308	-
11324	1438855_x_at	Region	Tnfaip2	21928	12	106918353	106928408	+
11325	1443546_at	Region	Hdlbp	110611	1	93233431	93306299	-
11326	1444025_at	Region	None	None	2	55522400	55523103	+
11327	1446532_at	Region	None	None	15	92957497	92958118	-
11328	1457726_at	Region	Rps15a	267019	7	111980231	111992004	-
11329	1434388_at	Region	Mobkl2a	208228	10	80817003	80833604	-
11330	1417612_at	Region	Ier5	15939	1	154988211	154990321	-
11331	1453372_at	Region	Dnajc1	13418	2	18259145	18434517	-
11332	1416584_at	Region	Man2b2	17160	5	35313433	35337139	-
11333	1417892_a_at	Region	Sirt3	64384	7	135265552	135284193	-
11334	1417627_a_at	Region	Limk1	16885	5	133669544	133702806	-
11335	1438040_a_at	Region	Tra1	22027	10	86665503	86680106	-
11336	1426678_at	Region	3110006P09Rik	68036	15	36999940	37010315	-
11337	1454964_at	Region	BC021395	225283	18	24716439	24758615	-
11338	1415876_a_at	Region	Rps26	27370	10	128361626	128363573	-
11339	1433605_at	Region	Inpp5a	212111	7	133784822	133975505	+
11340	1437772_s_at	Region	Fuca1	71665	4	134802014	134821570	+
11341	1456944_at	Region	C230009H10Rik	320399	7	40517772	40518700	+
11342	1439235_x_at	Region	Tm2d2	69742	8	23740385	23746364	+
11343	1429166_s_at	Region	Clmn	94040	12	100215893	100309212	-
11344	1417140_a_at	Region	Ptpn2	19255	18	67896299	67955363	-
11345	1446048_at	Region	Cdh11	12552	8	101922774	102074855	-
11346	1425753_a_at	Region	Ung	22256	5	113241844	113249965	+
11347	1434268_at	Region	Adar	56417	3	89536967	89557589	+
11348	1418062_at	Region	Eef1a2	13628	2	180864650	180873972	-
11349	1417688_at	Region	BC004044	80752	5	137753158	137808121	+
11350	1447922_at	Region	Stxbp5l	207227	16	35953363	36204390	-
11351	1418412_at	Region	Tpd52l1	21987	10	31372578	31486119	-
11352	1457944_at	Region	Etv6	14011	6	134792559	135023343	+
11353	1457736_at	Region	Wbscr24	194309	5	134086393	134091758	-
11354	1448221_at	Region	Bat1a	53817	17	32946813	32958600	+
11355	1418335_a_at	Region	Dph2l1 /// MGI:2179725	116905	11	74903301	74916132	-
11356	1438151_x_at	Region	Zdhhc14	224454	17	5400177	5659646	+

11357	1422453_at	Region	Prpf8	192159	11	75212748	75235041	+
11358	1434185_at	Region	Acaca	107476	11	84127739	84129644	+
11359	1440041_at	Region	Tgfbr3	21814	5	106170128	106359608	-
11360	1453113_at	Region	Wdsub1	72137	2	59707764	59737984	-
11361	1451366_at	Region	Cops6	26893	5	137133910	137136792	+
11362	1455128_x_at	Region	Tnrc6a	233833	7	117180427	117250357	+
11363	1426376_at	Region	Dp1	13476	18	34567907	34596351	-
11364	1419274_at	Region	C80913	19777	7	33124913	33183548	-
11365	1439889_at	Region	Scn8a	20273	15	100993863	101098226	+
11366	1426788_a_at	Region	Ssrp1	20833	2	84735021	84744858	+
11367	1451714_a_at	Region	Map2k3	26397	11	60657699	60678445	+
11368	1454914_at	Region	2610101N10Rik	67958	9	95369771	95420790	-
11369	1419358_at	Region	Sorcs2	81840	5	34504743	34885854	-
11370	1420906_at	Region	Cd2ap	12488	17	40292779	40376218	-
11371	1444790_at	Region	1810005K13Rik	66255	18	80353999	80360382	-
11372	1437738_at	Region	Atp2c1	235574	9	105393471	105477208	-
11373	1451036_at	Region	Spg21	27965	9	65583236	65609720	+
11374	1427138_at	Region	0610010D24Rik	68339	12	96358158	96474441	-
11375	1427044_a_at	Region	Amph	218038	13	18423821	18627334	+
11376	1434707_at	Region	Sbf1	77980	15	89339395	89368172	-
11377	1417365_a_at	Region	Calm1	12313	12	95642228	95652493	+
11378	1417793_at	Region	Iigp2	54396	11	57925267	57948404	+
11379	1451286_s_at	Region	Fus	233908	7	122017364	122031841	+
11380	1423695_at	Region	9530090G24Rik	108687	2	155158964	155186323	-
11381	1455976_x_at	Region	Dbi	13167	1	119870842	119878615	-
11382	1439293_at	Region	C130047D21Rik	320861	9	75161369	75161936	+
11383	1449981_a_at	Region	Nat2	17961	8	66602080	66609702	+
11384	1448992_at	Region	Ina	226180	19	46566002	46575960	+
11385	1455031_at	Region	None	None	10	40559347	40560722	+
11386	1448673_at	Region	Pvrl3	58998	16	45281022	45382981	-
11387	1421790_a_at	Region	Kcnab3	16499	11	69051916	69058699	+
11388	1435937_at	Region	Sptlc2	20773	12	84187587	84267928	-
11389	1441087_at	Region	2810011L19Rik	69952	12	100827808	100828069	+
11390	1418067_at	Region	Cfl2	12632	12	51564268	51568328	-
11391	1439586_at	Region	LOC548102	548102	16	42437719	42438534	+
11392	1448217_a_at	Region	Rpl27	19942	11	101263502	101266620	+
11393	1439042_at	Region	Adcyap1r1	11517	6	55596313	55643230	+
11394	1431812_a_at	Region	Slc6a9	14664	4	116794241	116828210	+
11395	1452055_at	Region	Ctdsp1	227292	1	74694064	74699752	+
11396	1425691_at	Region	B3gat1	76898	9	26645461	26655237	+
11397	1417743_at	Region	Clk2	12748	3	88921556	88933062	+
11398	1460566_at	Region	LOC545454	545454	2	120820915	120822007	+
11399	1423196_at	Region	Nedd1	17997	10	92653242	92690856	-
11400	1423532_at	Region	Rnf44	105239	13	53289019	53303558	-
11401	1455298_at	Region	Idb4	15904	13	47859485	47862094	+
11402	1433901_at	Region	Gpiap1	53872	2	103469773	103502351	-
11403	1434586_a_at	Region	Ptdss2	27388	7	135533273	135558141	+
11404	1422537_a_at	Region	Id2	15902	12	21646605	21648874	-
11405	1439090_at	Region	D030022P07Rik	320607	16	56084412	56085344	-
11406	1452157_at	Region	Eprs	107508	1	184861496	184926808	+
11407	1418391_at	Region	Phf21a	192285	2	91918732	92065593	+
11408	1426963_at	Region	Pacs1l	217893	12	108490808	108550569	+
11409	1438294_at	Region	Sca1	20238	13	45130048	45540117	-
11410	1452902_at	Region	2610209N15Rik	70451	11	77757998	77763521	+
11411	1438778_at	Region	A930025H08Rik	77807	13	27889889	27890934	-
11412	1451671_at	Region	Gorasp1	74498	9	119935626	119947511	-
11413	1458555_at	Region	AW121567	270028	8	8571891	9138527	-
11414	1417129_a_at	Region	Mrg1	17536	2	115376917	115578960	-
11415	1434744_at	Region	BC023823	230734	4	123877893	123882353	+
11416	1428108_x_at	Region	Tmcc2	68875	1	132213622	132248587	-
11417	1438430_at	Region	Hbp1	73389	12	28501152	28525243	-
11418	1439945_at	Region	Zfp449	78619	X	51117810	51120388	+
11419	1455397_at	Region	Caskin1	268932	17	22292368	22312494	+
11420	1418502_a_at	Region	Oxr1	170719	15	41691294	41762827	+
11421	1457366_at	Region	Cdc40	71713	10	40910293	40992658	-

11422	1422678_at	Region	Dgat2	67800	7	93245028	93274078	-
11423	1450170_x_at	Region	H2-D1	14964	17	186801	249200	-
11424	1440156_s_at	Region	AI851523	99101	2	162780917	162781136	+
11425	1444301_at	Region	Pcdh10	18526	3	44837486	44841910	+
11426	1448589_at	Region	Ndufb5	66046	3	32145242	32159738	+
11427	1450326_at	Region	Shc3	20418	13	49996373	50120768	-
11428	1449338_at	Region	D10Ertd641e	52717	10	59943553	59958756	-
11429	1429914_at	Region	Epc1	13831	18	6478658	6558794	-
11430	1460552_at	Region	Ascc3l1	320632	2	126722257	126754304	+
11431	1419714_at	Region	Cd274	60533	19	28620054	28640694	+
11432	1458470_at	Region	None	None	13	74295654	74296396	+
11433	1433819_s_at	Region	Agpat3	28169	10	78381798	78461806	-
11434	1416350_at	Region	Klf16	118445	10	80698354	80708753	-
11435	1450982_at	Region	Slc9a3r1	26941	11	114984496	115002271	+
11436	1417091_at	Region	Chuk	12675	19	43617742	43651851	-
11437	1421824_at	Region	Bace1	23821	9	45851835	45875693	+
11438	1427689_a_at	Region	Tnip1	57783	11	54663628	54715753	-
11439	1425183_a_at	Region	Rpl4	67891	9	64294180	64299471	+
11440	1443856_at	Region	Rabep1	54189	11	70570583	70666598	+
11441	1421569_at	Region	Grid1	14803	14	32951534	33710256	+
11442	1422659_at	Region	Camk2d	108058	3	125480582	125726370	+
11443	1425472_a_at	Region	Lmna	16905	3	88225013	88247171	-
11444	1442068_at	Region	Pinx1	72400	14	58390951	58450455	+
11445	1436293_x_at	Region	D1Ertd471e	27877	1	166238203	166238610	+
11446	1454705_at	Region	D15Ertd621e	210998	15	58436372	58467006	+
11447	1442810_x_at	Region	Scn9a	20274	2	66338406	66490352	-
11448	1428448_a_at	Region	Gtf3c2	71752	5	29615739	29638859	-
11449	1457690_at	Region	Kalrn	224126	16	32782324	32783165	-
11450	1434078_at	Region	D7Wsu128e	28018	7	115945280	115956990	+
11451	1418437_a_at	Region	Tcfl4	21428	11	100908397	100913294	+
11452	1436044_at	Region	Scn7a	20272	2	66530712	66640318	-
11453	1420402_at	Region	Atp2b2	11941	6	114316878	114489190	-
11454	1433493_at	Region	5730472N09Rik	108958	2	30296497	30317681	+
11455	1450350_a_at	Region	MGI:1932093	81703	12	82468979	82509441	+
11456	1425092_at	Region	Cdh10	320873	15	18747204	18942493	+
11457	1441904_x_at	Region	9130005N14Rik	68303	5	63771941	63843703	+
11458	1423725_at	Region	Pls3	102866	X	70446318	70535193	-
11459	1430634_a_at	Region	Pfkp	56421	13	6476970	6563786	-
11460	1419638_at	Region	Efnb2	13642	8	7983291	8026630	-
11461	1460600_at	Region	LOC245350	245350	X	11175895	11177481	+
11462	1437183_at	Region	Lrrc4b	272381	7	38517724	38540036	+
11463	1447277_s_at	Region	Pcyox1	66881	6	86820763	86870063	-
11464	1450756_s_at	Region	Cul3	26554	1	80599109	80655022	-
11465	1422414_a_at	Region	Calm2	12314	17	85290221	85303654	-
11466	1423165_a_at	Region	Mta2	23942	19	8138467	8148847	+
11467	1417845_at	Region	Cldn6	54419	17	21481545	21484620	+
11468	1450454_at	Region	Tor3a	30935	1	156560153	156579875	-
11469	1425859_a_at	Region	Psmd4	19185	3	94520536	94530376	-
11470	1431938_a_at	Region	Pmm2	54128	16	8311000	8330817	+
11471	1416187_s_at	Region	Pnrc2	52830	4	134752186	134755084	-
11472	1437196_x_at	Region	Rps16	20055	7	23752643	23754589	+
11473	1439283_at	Region	Osbpl9	100273	4	108020555	108061497	-
11474	1452594_at	Region	Dusp11	72102	6	86372013	86391226	-
11475	1440142_s_at	Region	Gfap	14580	11	102709766	102718169	-
11476	1435346_at	Region	2310043N13Rik	66396		48970084	48970693	+
11477	1433702_at	Region	D19Wsu12e	226090	19	28862793	28936642	-
11478	1429044_at	Region	Camsap1l1	67886	1	136115147	136192998	-
11479	1419102_at	Region	Sin3a	20466	9	57189858	57241642	+
11480	1443724_at	Region	Jph3	57340	8	121112150	121172408	+
11481	1450194_a_at	Region	Myb	17863	10	21054488	21090667	-
11482	1440051_at	Region	Ppp3ca	19055	3	135555655	135820664	+
11483	1451777_at	Region	BC013672	234311	8	60993208	61102544	+
11484	1448448_a_at	Region	Chkb	12651	15	89479240	89482772	-
11485	1427939_s_at	Region	Mycbp	56309	4	122932163	122939256	+
11486	1435370_a_at	Region	Ces3	104158	8	92449775	92481484	-

11487	1445377_at	Region	Als2cr19	72823	1	61929985	62930941	+
11488	1427844_a_at	Region	Cebpb	12608	2	167197648	167199151	+
11489	1423211_at	Region	Nola3	66181	2	111885207	111886138	+
11490	1448060_at	Region	Sema6d	214968	2	124124149	124181623	+
11491	1448691_at	Region	Ubqln4	94232	3	88297580	88313589	+
11492	1435000_at	Region	Gspt1	14852	16	10388595	10422192	+
11493	1456226_x_at	Region	Ddr1	12305	17	33397888	33421229	-
11494	1455476_a_at	Region	Gse1	382034	8	119846048	119938723	+
11495	1436485_s_at	Region	Whrn	73750	4	62506101	62587142	-
11496	1426340_at	Region	Slc1a3	20512	15	8424368	8500974	-
11497	1450972_at	Region	3110040N11Rik	67290	7	75590133	75597570	-
11498	1417242_at	Region	Ddx48	192170	11	119109456	119121136	-
11499	1417153_at	Region	Btbd14a	67991	2	25991155	26054942	-
11500	1433925_at	Region	Dnclic2	234663	8	103710711	103736151	-
11501	1430538_at	Region	2210013O21Rik	70123	X	147242260	147275811	+
11502	1415820_x_at	Region	Nono /// LOC434808	434808	X	75193538	75198479	-
11503	1417317_s_at	Region	Rpl35a /// 4632409L19Rik	57808	16	31865188	31868217	+
11504	1450389_s_at	Region	Pip5k1a	18719	19	23533932	23683715	-
11505	1444422_at	Region	Pcdh19	279653	X	127132549	127233781	-
11506	1451297_at	Region	Gulo	268756	14	60514207	60536630	-
11507	1455889_at	Region	Mlstd1	330450	6	149072613	149116582	+
11508	1449897_a_at	Region	Mtcp1	17763	X	70065501	70077198	-
11509	1450399_at	Region	Psen1	19164	12	80552589	80599225	+
11510	1434395_at	Region	Man1a2	17156	3	99992328	100111138	-
11511	1452973_at	Region	Ppm1k	243382	6	57667241	57693162	-
11512	1446147_at	Region	C79248	96982	2	155626395	155627019	-
11513	1460657_at	Region	Wnt10a	22409	1	75095603	75108385	+
11514	1422798_at	Region	Cntnap2	66797	6	45854973	47502306	+
11515	1434629_at	Region	None	None	2	37364330	37366015	-
11516	1450922_a_at	Region	Tgfb2	21808	1	186121756	186210661	-
11517	1456883_at	Region	Stox1	216021	10	62625575	62692453	-
11518	1426710_at	Region	Calm3	12315	7	13866351	13874927	-
11519	1417094_at	Region	MGI:1917275	70025	4	150670496	150764209	+
11520	1456334_s_at	Region	A230106D06Rik	232785	6	48067302	48076242	+
11521	1448424_at	Region	Frzb	20378	2	80109719	80145145	-
11522	1457183_at	Region	Slc6a1	232333	6	114853331	114888000	+
11523	1455069_x_at	Region	Slc25a4	11739	8	45151043	45155041	-
11524	1434404_at	Region	C030011O14Rik	215708	3	151257333	151321808	-
11525	1427899_at	Region	Rnf6	74132	5	145096046	145108279	-
11526	1451171_at	Region	2310008H04Rik	224008	16	14656989	14914568	-
11527	1421851_at	Region	Ddx26	18130	14	57209161	57294009	-
11528	1420734_at	Region	Ppp1r3f	54646	X	5797892	5813060	-
11529	1417165_at	Region	Mbd2	17191	18	70800857	70858783	+
11530	1417168_a_at	Region	Usp2	53376	9	44056022	44083332	+
11531	1460601_at	Region	Myrip	245049	9	120316489	120487292	+
11532	1416502_a_at	Region	Preb	50907	5	29413992	29419173	-
11533	1424495_a_at	Region	Cklf	75458	8	103545239	103557779	+
11534	1418822_a_at	Region	Arf6	11845	12	66203985	66205580	+
11535	1424824_at	Region	9630044O09Rik	105439	14	98195231	98249790	+
11536	1433614_at	Region	Snx27	76742	3	93985373	94070606	-
11537	1434660_at	Region	Alkbh	211064	12	84553626	84569673	+
11538	1438650_x_at	Region	Gja1	14609	10	56627159	56640230	+
11539	1460403_at	Region	Psip1	101739	4	82441872	82472582	-
11540	1439557_s_at	Region	Ldb2	16826	5	43229785	43558377	-
11541	1458163_at	Region	BC066028	407812	7	135211557	135223941	-
11542	1428398_at	Region	B3galt5	93961	16	95762296	95799852	+
11543	1457213_a_at	Region	Dgkh	380921	14	72922487	72981706	-
11544	1441277_s_at	Region	Ptprk	19272	10	28105024	28627423	+
11545	1432916_at	Region	5730407I07Rik	70515	15	33597307	33598609	+
11546	1443982_at	Region	Rgnef	110596	13	94087960	94394666	-
11547	1427371_at	Region	Abca8a	217258	11	109846725	109912709	-
11548	1418085_at	Region	Prkcz	18762	4	153754439	153853605	-
11549	1417777_at	Region	Ltb4dh	67103	4	58909039	58930527	-

11550	1448026_at	Region	Chd7	320790	4	8617703	8793952	+
11551	1426069_s_at	Region	Slc7a4	224022	16	16343928	16347174	-
11552	1418121_at	Region	Vrk3	101568	7	38823439	38852449	+
11553	1438116_x_at	Region	Slc9a3r1	26941	11	114984496	115002271	+
11554	1428500_at	Region	2210419D22Rik	73661	6	135203243	135206518	-
11555	1426323_x_at	Region	MGI:1353606	30954	12	108124289	108128532	+
11556	1446260_at	Region	4930447C04Rik	75801	12	69728240	69764946	-
11557	1435791_x_at	Region	Rpl17	319195	18	75235707	75238565	+
11558	1456888_at	Region	C230090D14	270198	9	109038494	109078817	+
11559	1426535_at	Region	9630046K23Rik	224143	16	37343910	37372402	-
11560	1438213_at	Region	A830018L16Rik	320492	1	11519751	12100440	+
11561	1428239_at	Region	Ankrd16	320816	2	11694586	11697273	+
11562	1433656_a_at	Region	Gnl3	30877	14	29144393	29151021	-
11563	1418947_at	Region	Nek3	23954	8	20880454	20918573	-
11564	1422147_a_at	Region	Pla2g6	53357	15	79337514	79379454	-
11565	1449972_s_at	Region	Zfp97 /// BC018101	22759	17	0	593	+
11566	1422467_at	Region	None	None	4	121863359	121886854	+
11567	1450016_at	Region	Ccng1	12450	11	40489722	40494170	-
11568	1436068_at	Region	Zbtb10	229055	3	9111529	9270577	+
11569	1450129_a_at	Region	Socs6	54607	18	89012087	89038416	-
11570	1443247_at	Region	None	None	13	42851494	42852002	-
11571	1435421_at	Region	Fsd1	240121	17	53625987	53635126	+
11572	1442111_at	Region	D430033H22Rik	320461	19	45731358	45732042	+
11573	1436724_a_at	Region	Lgtn	16865	1	131002708	131021706	+
11574	1448934_at	Region	Ndufa10	67273	1	92267199	92301249	-
11575	1437799_at	Region	4933407H18Rik	71101	5	31867548	31908144	+
11576	1433767_at	Region	1110018G07Rik	68497	12	81786755	81819896	-
11577	1459293_at	Region	C030038J10Rik	243385	6	59598164	59670385	-
11578	1441055_at	Region	R75368	97198	4	57659946	57660576	+
11579	1421958_at	Region	L1cam	16728	X	68515690	68530459	-
11580	1416418_at	Region	Gabarapl1	57436	6	130220677	130229816	+
11581	1434150_a_at	Region	3300001H21Rik /// MGI:3026615	393082	15	100410740	100418501	+
11582	1422733_at	Region	Fjx1	14221	2	102154049	102156475	-
11583	1416625_at	Region	Serping1	12258	2	84463134	84473153	-
11584	1424566_s_at	Region	Polr3d	67065	14	64754785	64759436	-
11585	1456204_at	Region	2010107H07Rik	66487	14	29256469	29262975	-
11586	1436589_x_at	Region	Prkd2	101540	7	13794089	13821505	+
11587	1426094_at	Region	Rhbdl1	214951	17	23640626	23643288	-
11588	1424557_at	Region	Zcchc9	69085	13	87933565	87943200	-
11589	1449050_at	Region	Recc1	19687	5	64063687	64137434	-
11590	1416432_at	Region	Pfkfb3	170768	2	11387835	11418034	-
11591	1425665_a_at	Region	Srp54	24067	12	51906616	51907500	+
11592	1426300_at	Region	Alcam	11658	16	51168811	51372049	-
11593	1430560_at	Region	Ppp1r10	52040	17	33636151	33651436	+
11594	1417029_a_at	Region	Trim2	80890	3	83898180	83960638	-
11595	1425408_a_at	Region	2610034M16Rik	69239	17	56562860	56568186	-
11596	1421369_a_at	Region	Mab21l1	17116	3	55415255	55417607	+
11597	1421982_a_at	Region	Unc50	67387	1	37724660	37733461	+
11598	1443390_at	Region	Dock1	330662	7	129038306	129541299	+
11599	1417411_at	Region	Nap1l5	58243	6	59067708	59069539	-
11600	1424468_s_at	Region	Phldb1	102693	9	44675163	44724049	-
11601	1438241_at	Region	Rgma	244058	7	67267239	67313292	+
11602	1434105_at	Region	Epm2aip1	77781	9	111313226	111320386	+
11603	1438483_at	Region	None	None	5	117083657	117084800	+
11604	1424604_s_at	Region	Sumf1	58911	6	108608573	108687101	-
11605	1441926_x_at	Region	Tmie	20776	9	110907343	110921380	-
11606	1444395_at	Region	Dixdc1	330938	9	50735078	50800301	-
11607	1453060_at	Region	Rgs8	67792	1	153537740	153582301	+
11608	1437774_at	Region	1700020I14Rik	66602	2	119108145	119117748	+
11609	1428300_at	Region	4932439K10Rik	74392	10	75332436	75414133	+
11610	1424847_at	Region	Nefh	380684	11	4833545	4842900	-
11611	1457490_at	Region	B230218L05Rik	330998	9	89615314	89625540	-
11612	1439234_a_at	Region	Tm2d2	69742	8	23740385	23746364	+

11613	1427120_at	Region	Zfp26	22688	9	20309411	20333047	-
11614	1450247_a_at	Region	Scamp5	56807	9	57554973	57581670	-
11615	1460565_at	Region	Slc41a1	98396	1	131678316	131697273	+
11616	1431619_a_at	Region	Dtnbp1	94245	13	44496235	44576050	-
11617	1424613_at	Region	Gprc5b	64297	7	112847904	112871012	-
11618	1423563_at	Region	ORF31	260297	17	32332934	32335508	+
11619	1435790_at	Region	Olfm2	244723	9	20538487	20598918	-
11620	1446258_at	Region	None	None	15	55843404	55844180	-
11621	1420892_at	Region	Wnt7b	22422	15	85584040	85630181	-
11622	1454792_s_at	Region	Sephs1	109079	2	4798835	4827828	+
11623	1448139_at	Region	Mlc1	170790	15	89007469	89030936	-
11624	1459483_at	Region	AW121567	270028	8	8571891	9138527	-
11625	1428644_at	Region	Mgat5	107895	1	127142098	127323193	+
11626	1426866_at	Region	D4st1	72136	2	118440373	118442434	+
11627	1433894_at	Region	AI591476	231986	6	52913122	52956624	-
11628	1417235_at	Region	Ehd3	57440	17	71586595	71613824	+
11629	1421433_at	Region	Zfhx4	80892	3	5216397	5389236	+
11630	1424390_at	Region	Nupl1	71844	14	54755759	54787669	-
11631	1416609_at	Region	BC004004	80748	17	27090378	27115403	+
11632	1452234_s_at	Region	D16Bwg1494e	224019	16	16047277	16049629	+
11633	1456390_at	Region	Ppp2ca	19052	11	51851665	51875590	+
11634	1422247_a_at	Region	Uty /// LOC546404	22290	Y	41346	184254	+
11635	1433156_at	Region	Kcnip1	70357	11	33524574	33738321	-
11636	1438673_at	Region	Slc4a7	218756	14	13169299	13264198	+
11637	1455693_x_at	Region	Rps6 /// LOC434404 /// LOC545640	20104	4	85840854	85843549	-
11638	1423474_at	Region	Top1	21969	2	160127068	160179680	+
11639	1437417_s_at	Region	Gpc6	23888	14	111487529	112531234	+
11640	1425131_at	Region	Ptpn5	19259	7	41163785	41219667	-
11641	1424030_at	Region	Tcfcp2l2	195733	12	21130474	21167143	+
11642	1439454_x_at	Region	Tm2d2	69742	8	23740385	23746364	+
11643	1443287_at	Region	---	383787	2	118216115	118217287	-
11644	1458307_at	Region	B230334C09Rik	319537	3	157082841	157083211	-
11645	1448364_at	Region	Ccng2	12452	5	92603007	92611974	+
11646	1427042_at	Region	Mal2	105853	15	54573445	54604798	+
11647	1436372_a_at	Region	AA415817	94184	16	12571455	12638826	-
11648	1438557_x_at	Region	Dnpep	13437	1	75598827	75608568	-
11649	1426598_at	Region	Uty /// LOC546404 /// LOC546411	22290	Y	41346	184254	+
11650	1422302_s_at	Region	Ftl1 /// Ftl2 /// LOC544951 /// LOC545679	14325	7	6038071	6038985	-
11651	1460070_at	Region	Chst2	54371	9	95313309	95315530	-
11652	1437312_at	Region	Bmpr1b	12167	3	140807013	140952143	-
11653	1457495_at	Region	2900052N01Rik	73040	NONE	NONE	NONE	NONE
11654	1454901_at	Region	6430570G24	327989	11	86664419	86665560	-
11655	1426824_at	Region	Psme4	103554	11	30666852	30774524	+
11656	1456433_at	Region	Rcbtb1	71330	14	53736681	53772708	+
11657	1460315_s_at	Region	Tbk1	56480	10	121202714	121243001	-
11658	1451741_a_at	Region	Cdk7	12572	13	96873946	96901672	-
11659	1456628_x_at	Region	Rps24	20088	14	22859652	22865117	+
11660	1438093_x_at	Region	Dbi	13167	1	119870842	119878615	-
11661	1418123_at	Region	Unc119	22248	11	78069181	78074815	+
11662	1423998_at	Region	Gtf3c5	70239	2	28498477	28515408	-
11663	1449043_at	Region	Naga	17939	15	82380266	82389523	-
11664	1451210_at	Region	Ppap2c	50784	10	79648047	79654486	-
11665	1424444_a_at	Region	1600014C10Rik	72244	7	33351336	33365081	+
11666	1433718_a_at	Region	Cbx1	12412	11	96610246	96629727	+
11667	1417960_at	Region	Cpeb1	12877	7	75155001	75262817	-
11668	1436918_at	Region	1810038L18Rik	69181	10	118438264	118440169	-
11669	1431530_a_at	Region	Tspan5	56224	3	137631430	137793659	+
11670	1418529_at	Region	Osgep	66246	14	46010322	46019468	-
11671	1439839_at	Region	D130051D11Rik	319737	15	85673593	85716318	+
11672	1416823_a_at	Region	Osbpl1a	64291	18	12943381	13129769	-

11673	1417683_at	Region	Diablo	66593	5	122682693	122695654	-
11674	1417213_a_at	Region	Rbm6	19654	9	107841550	107940685	-
11675	1424988_at	Region	Mylip	218203	13	44964531	44986418	+
11676	1437969_s_at	Region	0610007P22Rik	68327	17	23046050	23048646	+
11677	1417575_at	Region	Otub2	68149	12	98834218	98851823	+
11678	1421952_at	Region	Capn6	12338	X	137242282	137286776	-
11679	1416074_a_at	Region	Rpl28	19943	7	4039146	4044498	+
11680	1437746_at	Region	Lrrtm1	74342	6	77592843	77607877	+
11681	1421768_a_at	Region	Homer1	26556	13	89489518	89588558	+
11682	1449070_x_at	Region	Apcdd1	494504	18	63040206	63182348	+
11683	1428688_at	Region	Pdcd11	18572	19	46642066	46682446	+
11684	1448630_a_at	Region	Sdhc	66052	1	171058005	171079442	-
11685	1460189_at	Region	Wdr23	28199	14	50079168	50088008	+
11686	1436805_at	Region	2810457I06Rik	72828	9	40966047	41111032	-
11687	1455230_at	Region	Cacng4	54377	11	107555869	107615553	-
11688	1456326_at	Region	Gm784	333564	X	101021406	101086594	-
11689	1449422_at	Region	Cdh4	12561	2	179497210	179613363	+
11690	1450449_a_at	Region	2900002H16Rik	75695	5	123664423	123702734	-
11691	1435845_at	Region	2900006N09Rik	72898	5	111455434	111462202	-
11692	1424568_at	Region	Tspan2	70747	3	102162658	102198922	+
11693	1430037_at	Region	Snx27	76742	3	93985373	94070606	-
11694	1458802_at	Region	Hivep3	16656	4	119003584	119157855	+
11695	1456036_x_at	Region	Gsto1	14873	19	47406289	47416090	+
11696	1423220_at	Region	Eif4e	13684	3	137416519	137446834	+
11697	1426733_at	Region	Itpk1	217837	12	98013454	98150125	-
11698	1456393_at	Region	2310002J21Rik	66360	19	53175077	53508819	-
11699	1456739_x_at	Region	Armcx2	67416	X	128349086	128353933	-
11700	1457146_at	Region	Dock4	238130	12	37324777	37326167	+
11701	1428914_at	Region	2310014D11Rik	69633	19	46811476	46812774	-
11702	1460424_at	Region	1810008O21Rik	69094	7	13402158	13404869	+
11703	1455667_at	Region	None	None	5	29410615	29412616	-
11704	1440388_at	Region	None	None	6	28310038	28310758	+
11705	1424206_at	Region	Smarca5	93762	8	79911951	79951467	-
11706	1427604_a_at	Region	Atp9a	11981	2	168143171	168250533	-
11707	1435705_at	Region	LOC232875	232875	7	10761773	10775035	-
11708	1458409_at	Region	C86595	97935	14	49268738	49269867	-
11709	1435238_x_at	Region	2310009A05Rik	66364	9	73172461	73175337	+
11710	1434200_at	Region	BC010981	407830	14	54760287	54760969	-
11711	1455010_at	Region	1500012F01Rik	68949	2	166519800	166522596	+
11712	1430543_at	Region	1500005P14Rik	76686	7	25693378	25719722	+
11713	1438232_at	Region	Foxp2	114142	6	14888986	15429612	+
11714	1426084_a_at	Region	Tor1aip1	208263	1	155902618	155932908	-
11715	1430154_at	Region	4930543C13Rik	78932	19	15856656	15858955	-
11716	1419457_at	Region	Rgnef	110596	13	94087960	94394666	-
11717	1441780_at	Region	None	None	3	24837915	24838360	-
11718	1419462_s_at	Region	Gtl3	14894	8	94698722	94713270	-
11719	1431592_a_at	Region	Sh3kbp1	58194	X	153308006	153573572	+
11720	1455924_at	Region	Rab6b	270192	9	103083792	103160522	+
11721	1460739_at	Region	D11Bwg0280e	52915	11	6284151	6300949	+
11722	1440764_at	Region	Araf	11836	X	19087538	19099458	+
11723	1431597_a_at	Region	Nrip3	78593	7	103610927	103634416	-
11724	1435741_at	Region	Pde8b	218461	13	91208631	91434426	-
11725	1446610_at	Region	Elmo1	140580	13	19666927	20088452	+
11726	1418785_at	Region	Mapk8ip2	60597	15	89507005	89515320	+
11727	1420843_at	Region	Ptprf	19268	4	117168706	117236351	-
11728	1415986_at	Region	Clcn4-2	12727	7	241	13444	+
11729	1429564_at	Region	Pcgf5	76073	19	35721737	35798874	+
11730	1433973_at	Region	Sephs1	109079	2	4798835	4827828	+
11731	1443175_at	Region	A830010M09Rik	320317	3	25281887	25282477	-
11732	1424346_at	Region	Ppp6c	67857	2	39128960	39158501	-
11733	1443849_x_at	Region	Urod	22275	4	115949123	115953269	-
11734	1429696_at	Region	Gpr123	52389	7	134230082	134274053	+
11735	1428602_at	Region	Kcnj9	16524	1	172251006	172258201	-
11736	1428768_at	Region	2700050F09Rik	72587	5	146420994	146439998	+
11737	1440108_at	Region	Foxp2	114142	6	14888986	15429612	+

11738	1418560_at	Region	Pdha1	18597	X	153721624	153737741	-
11739	1431711_a_at	Region	9030409G11Rik	71529	4	140983652	141035828	-
11740	1455732_at	Region	1700025G04Rik	69399	1	151777069	151974583	-
11741	1419089_at	Region	Timp3	21859	10	86261055	86310024	+
11742	1449503_at	Region	Kpna1	16646	16	34764772	34817569	+
11743	1444679_at	Region	Bhc80	192285	2	91918732	92065593	+
11744	1425792_a_at	Region	Rorc	19885	3	93860609	93886411	+
11745	1420375_at	Region	Kif3a	16568	11	53320244	53354795	+
11746	1424024_at	Region	Mcfd2	193813	17	85111247	85122719	-
11747	1425338_at	Region	Plcb4	18798	2	135319401	135526921	+
11748	1428877_at	Region	Srp72	66661	5	76219172	76244365	+
11749	1422005_at	Region	Prkr	19106	17	76674799	76704807	-
11750	1426081_a_at	Region	Dio2	13371	12	86128063	86141948	-
11751	1422552_at	Region	Rprm	67874	2	54009632	54011091	-
11752	1442347_at	Region	Lrp8	16975	4	106761166	106833072	+
11753	1427384_at	Region	5430439G14Rik	71389	2	160403955	160419235	-
11754	1424529_s_at	Region	Cgref1	68567	5	29392087	29404535	-
11755	1439410_x_at	Region	D11Ertd333e	68066	11	102224234	102228559	-
11756	1456108_x_at	Region	Zfp179	22671	11	61174084	61179528	-
11757	1450249_s_at	Region	Kif5a /// Kif5c	16572	10	126965001	126999707	-
11758	1429348_at	Region	Sema3c	20348	5	15994279	16149731	+
11759	1420834_at	Region	Vamp2	22318	11	68814186	68818039	+
11760	1460440_at	Region	Lphn3	319387	5	80275281	81049432	+
11761	1435568_at	Region	AK129128	218343	13	72188389	72247425	+
11762	1451049_at	Region	Bcap31	27061	X	68346838	68376830	-
11763	1428662_a_at	Region	MGI:1916782	74318	5	76331480	76359676	-
11764	1428179_at	Region	Ndufv2	72900	17	63788360	63810997	-
11765	1420927_at	Region	St6gal1	20440	16	22008076	22143979	+
11766	1448295_at	Region	D13Wsu50e	28077	13	65842571	65848783	+
11767	1451280_at	Region	MGI:107562	74100	9	112222231	112229149	-
11768	1423972_at	Region	Etfa	110842	9	55567483	55625204	-
11769	1448905_at	Region	Nme3	79059	17	22702408	22703419	+
11770	1460680_a_at	Region	Rpl23	65019	11	97598630	97603460	-
11771	1418255_s_at	Region	Srf	20807	17	44056896	44066219	-
11772	1425326_at	Region	Acly	104112	11	100297443	100348988	-
11773	1455481_at	Region	Ids	15931	X	65014764	65033475	-
11774	1416484_at	Region	Ttc3	22129	16	93778128	93866771	+
11775	1423748_at	Region	Pdk1	228026	2	71570967	71599744	+
11776	1426256_at	Region	Timm17a	21854	1	135152008	135164210	-
11777	1442883_s_at	Region	D10Bwg1364e	216169	10	80715282	80721253	-
11778	1426775_s_at	Region	Scamp1	107767	13	90385498	90469347	-
11779	1423200_at	Region	Ncor1	20185	11	62043183	62164152	-
11780	1453369_a_at	Region	Fundc1	72018	X	15795509	15811237	-
11781	1436854_at	Region	Trpc2	22064	7	96181838	96202292	+
11782	1424240_at	Region	Arfip2	76932	7	99750533	99755261	-
11783	1430579_at	Region	Tnik	69014	3	27680596	28087969	+
11784	1444268_at	Region	BC004004	80748	17	27090378	27115403	+
11785	1431403_a_at	Region	2900002G04Rik	78283	X	153013977	153098157	+
11786	1416001_a_at	Region	Cotl1	72042	8	119165392	119197206	-
11787	1454721_at	Region	1110018G07Rik	68497	12	81786755	81819896	-
11788	1429360_at	Region	Klf3	16599	5	63601098	63627704	+
11789	1435278_at	Region	2610206C24Rik	67158	18	32150744	32151382	-
11790	1428212_x_at	Region	Rpl31	114641	1	39664836	39668824	+
11791	1433578_at	Region	E130304D01	231290	5	71790031	71796081	+
11792	1437751_at	Region	Ppargc1a	19017	5	50268292	50364500	-
11793	1453467_s_at	Region	Rps15a	267019	7	111980231	111992004	-
11794	1428705_at	Region	1700007K13Rik	69327	2	28394163	28398526	-
11795	1449172_a_at	Region	Lin7b	22342	7	39443916	39446815	-
11796	1450941_at	Region	None	None	4	6292828	6323429	+
11797	1420493_a_at	Region	Pcyt2	68671	11	120431180	120438983	-
11798	1440121_at	Region	A530083M17Rik	320505	3	152401753	152402203	-
11799	1425494_s_at	Region	Bmpr1a	12166	14	32547251	32638735	-
11800	1425811_a_at	Region	Csrp1	13007	1	135580251	135598910	+
11801	1429192_at	Region	Ski	20481	4	153649703	153714882	-
11802	1456620_at	Region	MGC79224	432486	10	88350966	88419158	+

11803	1448887_x_at	Region	Fxc1	14356	7	99755452	99756758	+
11804	1431510_s_at	Region	2010110K16Rik	67899	9	118060948	118143996	-
11805	1450735_at	Region	1810003N24Rik	66249	11	17097995	17106346	-
11806	1423622_a_at	Region	Ccnl1	56706	3	65619603	65631632	-
11807	1431191_a_at	Region	Syt1	20979	10	108385089	108577499	-
11808	1425803_a_at	Region	Mbd2	17191	18	70800857	70858783	+
11809	1437184_at	Region	AA407526	231279	5	68325793	68340721	+
11810	1433455_at	Region	None	None	5	120967826	120969276	-
11811	1434860_at	Region	Narg3	93698	4	11188265	11189818	-
11812	1449625_at	Region	None	None	NONE	NONE	NONE	NONE
11813	1416705_at	Region	Rpe	66646	1	66993273	67012185	+
11814	1425537_at	Region	Ppm1a	19042	12	69630561	69641441	+
11815	1429901_at	Region	6330571D19Rik	432450	10	31729508	31730785	-
11816	1429215_at	Region	2310058N22Rik	71921	12	112143610	112144601	+
11817	1435254_at	Region	Plxnb1	235611	9	109142184	109166503	+
11818	1431146_a_at	Region	Cpne8	66871	15	90540437	90732329	-
11819	1425686_at	Region	Cflar	12633	1	59018187	59060035	+
11820	1427694_at	Region	Gnrhr	14715	5	85444004	85459880	-
11821	1429692_s_at	Region	Gch1	14528	14	42234216	42269658	-
11822	1435383_x_at	Region	Ndn	17984	7	56122085	56123665	+
11823	1456072_at	Region	Ppp1r9a	243725	6	4856205	5118546	+
11824	1421876_at	Region	Mapk9	26420	11	49599595	49638939	+
11825	1425906_a_at	Region	Sema3e	20349	5	13478445	13707444	+
11826	1426122_a_at	Region	Coro6	216961	11	77189572	77195160	+
11827	1444596_at	Region	None	None	4	138618330	138618741	-
11828	1443070_at	Region	Auts2	319974	5	130627794	130728707	-
11829	1416578_at	Region	Rbx1	56438	15	81517173	81527164	+
11830	1437202_at	Region	C130067A03Rik	320713	4	93917113	93954172	-
11831	1429671_at	Region	2410018M08Rik	71970	5	129064817	129072750	+
11832	1435923_at	Region	Gm237	211488	10	67598925	67602736	-
11833	1424456_at	Region	Pvrl2	19294	7	16585905	16618713	-
11834	1438864_at	Region	C920006C10Rik	76740	15	65810937	65897708	+
11835	1450393_a_at	Region	Scamp4	56214	10	80733774	80746677	+
11836	1424368_s_at	Region	Ubqln1	56085	13	56804764	56844390	-
11837	1435183_at	Region	3110043L15Rik	73174	11	96957260	96970799	-
11838	1428667_at	Region	Maoa	17161	X	14858768	14924684	+
11839	1434179_at	Region	Mll3	231051	5	23741347	23769624	-
11840	1428397_at	Region	B3galt5	93961	16	95762296	95799852	+
11841	1460711_at	Region	4930461P20Rik	78244	15	10260892	10284727	-
11842	1455538_at	Region	6330403M23Rik	109169	18	36523201	36525181	+
11843	1434679_at	Region	Cspg3	13004	8	69246056	69273784	-
11844	1448428_at	Region	Nbl1	17965	4	137963553	137974126	-
11845	1455079_at	Region	AI836376	100737	5	72281021	72350725	+
11846	1416216_at	Region	Reps1	19707	10	18013924	18049606	+
11847	1428223_at	Region	Mfsd2	76574	4	121973962	121988282	-
11848	1438712_at	Region	2010308M01Rik	72121	3	106281879	106296871	+
11849	1432631_at	Region	1190002C06Rik	73137	18	57571393	57606175	+
11850	1449674_s_at	Region	Pdcd6ip	18571	9	113708127	113760882	-
11851	1457089_at	Region	None	None	11	3141987	3142641	-
11852	1455878_at	Region	2700023E23Rik	70036	5	72927647	72928860	+
11853	1430770_at	Region	3110080E11Rik	73219	8	16838586	16839641	-
11854	1452762_at	Region	8430436O14Rik	71506	9	116559671	116560865	-
11855	1457156_at	Region	Trhde	237553	10	114074464	114477689	-
11856	1430111_a_at	Region	Bcat1	12035	6	145846706	145893811	-
11857	1450258_a_at	Region	Elavl4	15572	4	109165070	109310814	-
11858	1436924_x_at	Region	Rpl31	114641	1	39664836	39668824	+
11859	1435358_at	Region	Cuedc1	103841	11	87827241	87920907	+
11860	1448510_at	Region	Efna1	13636	3	89028209	89036114	-
11861	1424178_at	Region	Tmem38a	74166	8	71722899	71738144	+
11862	1437621_x_at	Region	Phgdh /// LOC546010	236539	3	97799380	97826178	-
11863	1418594_a_at	Region	Ncoa1	17977	12	3409624	3575830	-
11864	1423449_a_at	Region	Actn4	60595	7	24299701	24368752	-
11865	1433643_at	Region	Cacna2d1	12293	5	14583526	14791513	+
11866	1456995_at	Region	2700045P11Rik	72555	16	10723051	11008542	+

11867	1452090_a_at	Region	Olfm3	229759	3	113850919	113895474	+
11868	1436916_at	Region	None	None	9	103459023	103459743	-
11869	1429310_at	Region	Flrt3	71436	2	140172056	140185326	-
11870	1415985_at	Region	Sf3b3	101943	8	110108214	110144495	-
11871	1419223_a_at	Region	Dtna	13527	18	23643946	23881842	+
11872	1438795_x_at	Region	Fkbp1a	14225	2	150999557	151018627	+
11873	1425560_a_at	Region	S100a16	67860	3	90349834	90350682	+
11874	1430303_at	Region	4921537D05Rik	77048	10	94662974	94764320	+
11875	1417660_s_at	Region	Vps29	56433	5	121506763	121515637	+
11876	1436503_at	Region	BC048546	232400	6	129230009	129271784	-
11877	1433549_x_at	Region	Rps21	66481	2	179974367	179975394	+
11878	1452094_at	Region	P4ha1	18451	10	59286782	59335753	+
11879	1418566_s_at	Region	Nudcd2	52653	11	40472609	40478985	+
11880	1451147_x_at	Region	AI481750	105859	15	81987653	82001658	+
11881	1416270_at	Region	Polr2g	67710	19	7989676	7995104	-
11882	1460380_at	Region	Dsg2	13511	18	20773051	20817758	+
11883	1437156_at	Region	Efcbp1	69352	4	14879213	15076079	-
11884	1418124_at	Region	2610318K02Rik	68032	2	111986257	111991265	-
11885	1456387_at	Region	Nol4	319211	18	22913931	23259189	-
11886	1457354_at	Region	6330509G02Rik	268481	11	99054185	99065154	-
11887	1423858_a_at	Region	Hmgcs2	15360	3	97766644	97796941	+
11888	1455042_at	Region	Tbl1x	21372	X	72294199	72320920	+
11889	1417468_at	Region	Nit1	27045	1	171271083	171274465	-
11890	1429735_at	Region	1110003F05Rik	66145	17	8844203	8846425	-
11891	1442348_at	Region	None	None	5	147768531	147769808	-
11892	1435245_at	Region	Gls2	216456	10	127943744	127946757	+
11893	1447676_x_at	Region	S100a16	67860	3	90349834	90350682	+
11894	1435583_at	Region	9330182L06Rik	231014	5	9273627	9491141	+
11895	1421328_at	Region	Mtap2 /// A730034C02	17756	1	66613900	66731968	+
11896	1433619_at	Region	AI894139	101197	6	48077721	48089796	+
11897	1424740_at	Region	Creb3	12913	4	43478591	43482789	+
11898	1452214_at	Region	Skil	20482	3	30508258	30530416	+
11899	1460430_at	Region	Rap2c	72065	X	45525090	45538760	-
11900	1457964_at	Region	1810044D09Rik	69798	6	91890127	91890879	+
11901	1434422_at	Region	AI428479	98341	1	57688292	57689151	+
11902	1451163_at	Region	Tinf2	28113	14	50197117	50199743	-
11903	1454045_a_at	Region	4933424M23Rik /// LOC260345	260345	11	117821888	117845019	+
11904	1444763_at	Region	Ptprk	19272	10	28105024	28627423	+
11905	1417967_at	Region	Mms19l	72199	19	41488079	41525508	-
11906	1416767_a_at	Region	1110003E01Rik	68552	5	64251633	64294659	-
11907	1426792_s_at	Region	Rusc2	100213	4	43322447	43343004	+
11908	1416640_at	Region	Kcne1l	66240	X	135744796	135746241	-
11909	1417765_a_at	Region	Amy1	11722	3	11288	30098	+
11910	1430756_at	Region	5430427G11Rik	71414	10	43326579	43327743	-
11911	1428967_at	Region	A330103N21Rik	77773	7	62113386	62117987	+
11912	1460003_at	Region	AI956758	99132	2	16683470	16684602	+
11913	1428333_at	Region	6530401D17Rik	76219	X	129538762	129579883	+
11914	1441376_at	Region	Gabarapl2	93739	8	111238426	111253086	+
11915	1436014_a_at	Region	Rusc1	72296	3	88827845	88837154	-
11916	1444508_s_at	Region	Arrdc1	215705	2	24857514	24867325	-
11917	1418057_at	Region	Tiam1	21844	16	88944544	89118259	-
11918	1437375_at	Region	None	None	19	27001787	27002323	-
11919	1440086_at	Region	Rnf182	328234	13	43178421	43231343	+
11920	1418684_at	Region	2310012P17Rik	69623	5	103619547	103620974	+
11921	1435672_at	Region	3830612M24	330635	7	111548725	111549404	+
11922	1418170_a_at	Region	Zcchc14	142682	8	120981449	121033085	-
11923	1426466_s_at	Region	Rps6kl1	238323	12	82004324	82019912	-
11924	1450248_at	Region	Adam11	11488	11	102582695	102600123	+
11925	1419752_at	Region	Nfx1	74164	4	41109832	41164029	+
11926	1423845_at	Region	AI481750	105859	15	81987653	82001658	+
11927	1417598_a_at	Region	Fxr1h	14359	3	33467491	33516721	+
11928	1422694_at	Region	Ttyh1	57776	7	3340443	3356202	+
11929	1417103_at	Region	Ddt	13202	10	75872973	75875114	-

11930	1453807_at	Region	6330563C09Rik	76186	13	114438290	114439758	+
11931	1418082_at	Region	Nmt1	18107	11	102849651	102887194	+
11932	1424768_at	Region	Cald1	109624	6	34803747	34868362	+
11933	1455955_s_at	Region	Snx17	266781	5	29652242	29657838	+
11934	1436101_at	Region	Rnf24	51902	2	130814673	130866701	-
11935	1419595_a_at	Region	Ggh	14590	4	20143507	20167394	+
11936	1435800_a_at	Region	Csda	56449	6	132143594	132167186	-
11937	1436420_a_at	Region	Ipo4	75751	14	50143923	50153613	-
11938	1440037_at	Region	Pbx1	18514	1	168054563	168366387	-
11939	1441197_at	Region	9530059O14Rik	319626	9	122695338	122696024	+
11940	1426884_at	Region	1110007A06Rik	68477	6	71723228	71772465	-
11941	1455369_at	Region	Apba1	319924	19	23142625	23185544	+
11942	1423115_at	Region	St6galnac6	50935	2	32539188	32551693	+
11943	1443401_at	Region	Trim32	69807	4	64696209	64707429	+
11944	1423828_at	Region	Fasn	14104	11	120628169	120645157	-
11945	1428940_at	Region	Gnaq	14682	19	15357188	15609698	+
11946	1436320_at	Region	None	None	NONE	NONE	NONE	NONE
11947	1425690_at	Region	B3gat1	76898	9	26645461	26655237	+
11948	1457988_at	Region	Sec63	140740	10	42868086	42935647	+
11949	1418322_at	Region	Crem	12916	18	3271060	3332223	-
11950	1446540_at	Region	Kirrel3	67703	9	34625377	34626028	+
11951	1435229_at	Region	A930008A22Rik	235283	9	40248196	40406053	-
11952	1419759_at	Region	Abcb1a	18671	5	8666835	8755262	+
11953	1435145_at	Region	Igsf4d	239857	16	65672212	65964483	-
11954	1432263_a_at	Region	Cox7a2l	20463	17	81335584	81347955	-
11955	1448142_x_at	Region	Rps13	68052	7	110187198	110189820	-
11956	1435115_at	Region	Fndc5	384061	4	128164173	128171706	+
11957	1418892_at	Region	Rhoj	80837	12	72164788	72257817	+
11958	1458285_at	Region	Gria1	14799	11	56764801	57055873	+
11959	1448433_a_at	Region	Pcolce	18542	5	136555709	136561968	-
11960	1456937_at	Region	Cdh26	381409	2	178147565	178204324	+
11961	1460194_at	Region	Phyh	16922	2	4836302	4855996	+
11962	1423897_at	Region	Rnf187	108660	11	58657917	58664545	-
11963	1423856_at	Region	Popdc3	78977	10	45407660	45436490	+
11964	1428392_at	Region	Rassf2	215653	2	131506703	131543663	-
11965	1434730_at	Region	AI854517	101694	7	73274371	73331599	+
11966	1436824_x_at	Region	LOC545856	545856	6	76842195	76844739	-
11967	1451149_at	Region	Pgm2	72157	4	98888410	98946194	+
11968	1426410_at	Region	Pdk3	236900	X	88425274	88492753	-
11969	1437109_s_at	Region	Lsm6	78651	8	21699568	21713205	-
11970	1438325_at	Region	Evi1	14013	3	29354762	29399710	-
11971	1448774_at	Region	Stoml2	66592	4	42943612	42947306	-
11972	1427245_at	Region	Arfgap1	228998	2	180684227	180699400	+
11973	1428930_at	Region	6330540D07Rik	76170	X	143942975	143993536	-
11974	1457177_at	Region	Rora	19883	9	69474948	69510983	+
11975	1434504_at	Region	Zfyve28	231125	5	32683213	32683988	-
11976	1416591_at	Region	Rab34	19376	11	77914493	77917847	+
11977	1429885_at	Region	4930431P19Rik	73886	7	103605116	103605970	+
11978	1433607_at	Region	Cbln4	228942	2	171545069	171552199	-
11979	1416005_at	Region	Psmc1	19179	12	95551741	95565014	+
11980	1424141_at	Region	Hectd1	207304	3	48495784	48579550	-
11981	1423056_at	Region	Nsg1	18196	5	36643418	36665681	-
11982	1449852_a_at	Region	Ehd4	98878	2	119603336	119668424	-
11983	1416641_at	Region	Lig1	16881	7	11271341	11305498	+
11984	1448536_at	Region	Lsm3	67678	6	91965158	91971745	+
11985	1423951_at	Region	Tm2d3	68634	7	59575395	59586131	+
11986	1444513_at	Region	Cnksr2	245684	X	151420978	151642515	-
11987	1426689_s_at	Region	Sdha	66945	13	70384460	70412084	-
11988	1416539_at	Region	Ysg2	22619	9	37555344	37589819	+
11989	1451245_at	Region	Lrrc3b	218763	14	13848834	13930422	-
11990	1420592_a_at	Region	Anp32e	66471	3	95417123	95434808	+
11991	1439123_at	Region	Bhc80	192285	2	91918732	92065593	+
11992	1416648_at	Region	Dnchc1	13424	12	106076688	106142158	+
11993	1436358_at	Region	Atpaf1	230649	4	114743890	114771219	+
11994	1456126_at	Region	Malt1	240354	18	65664223	65712018	+

11995	1448823_at	Region	Cxcl12	20315	6	117603898	117616686	+
11996	1424785_at	Region	Angptl6	70726	9	20751107	20756927	-
11997	1437043_a_at	Region	1110012M11Rik	73711	8	70692174	70697270	+
11998	1440891_at	Region	Gria4	14802	9	4328268	4706535	-
11999	1456279_a_at	Region	Bcap31	27061	X	68346838	68376830	-
12000	1423770_at	Region	Tmc6	217353	11	117587081	117601714	-
12001	1431811_a_at	Region	Fbxo34	78938	14	42557515	42616834	+
12002	1417707_at	Region	B230342M21Rik	100637	5	149512098	149517442	-
12003	1456212_x_at	Region	Socs3	12702	11	117787172	117790255	-
12004	1429344_at	Region	9.13E+15	74564	6	18479059	18480642	-
12005	1427227_at	Region	Gabrg1	14405	5	69531953	69623523	-
12006	1438880_at	Region	1700012D14Rik	75479	7	104974464	104975152	-
12007	1448913_at	Region	Smarcd1	83797	15	99759546	99771207	+
12008	1434828_at	Region	B430201A12Rik	329739	3	108768194	108823063	-
12009	1418611_at	Region	Gpr162	14788	6	125515212	125520685	-
12010	1433586_at	Region	Rgmb	68799	17	13809790	13830123	-
12011	1434034_at	Region	Cerk	223753	15	86189022	86235839	-
12012	1448777_at	Region	Mcm2	17216	6	89318530	89333755	-
12013	1428528_at	Region	1110007L15Rik	67604	5	138250433	138268108	+
12014	1450872_s_at	Region	Lip1	16889	19	33824007	33859684	-
12015	1450947_at	Region	2610528J11Rik	66451	4	117486180	117489122	+
12016	1439697_at	Region	Il1rap	16180	16	25368195	25502925	+
12017	1427017_at	Region	Satb2	212712	1	57097853	57272176	-
12018	1460375_at	Region	0610038D11Rik	67674	19	6623999	6624872	+
12019	1436111_at	Region	E030011K20Rik	208613	3	27283448	27313750	-
12020	1417515_at	Region	Lsm10	116748	4	125123865	125125693	+
12021	1450684_at	Region	Etv1	14009	12	35383685	35470632	+
12022	1455630_at	Region	1700010H15Rik	105000	12	80978687	81002773	+
12023	1426951_at	Region	Crim1	50766	17	76016353	76192698	+
12024	1443643_at	Region	T2	21331	17	1092	7498	+
12025	1438057_at	Region	None	None	3	80410446	80411354	-
12026	1428881_at	Region	Kns2	16593	12	107239749	107276237	+
12027	1417193_at	Region	Sod2	20656	17	11516794	11524420	-
12028	1421604_a_at	Region	Klf3	16599	5	63601098	63627704	+
12029	1456477_at	Region	Ccnt1	12455	15	98601104	98626096	-
12030	1436110_at	Region	None	None	7	75413613	75414289	-
12031	1433649_at	Region	Aof1	218214	13	46641719	46682749	+
12032	1435110_at	Region	Unc5b	107449	10	60725176	60793297	-
12033	1460704_at	Region	Rfng	19719	11	120601838	120605297	-
12034	1429447_at	Region	Evc2	68525	5	35845020	35931569	+
12035	1419164_at	Region	Zfp260	26466	7	25509675	25522233	+
12036	1441214_at	Region	MGI:2443248	320051	9	53366823	53442774	+
12037	1438017_at	Region	Rusc1	72296	3	88827845	88837154	-
12038	1435355_at	Region	Neb	17996	2	52065009	52266643	-
12039	1450746_at	Region	Keap1	50868	9	21116332	21125078	-
12040	1439515_at	Region	2900045N06Rik	72895	6	113640716	113716498	+
12041	1421090_at	Region	Epb4.1l1	13821	2	155877989	155997768	+
12042	1422803_at	Region	Fstl3	83554	10	79899919	79904946	+
12043	1455078_at	Region	None	None	11	77183548	77185877	+
12044	1426670_at	Region	Agrn	11603	4	154657652	154677497	-
12045	1441230_at	Region	1600019O04Rik	72007	3	26833958	27060578	-
12046	1434606_at	Region	Erbb3	13867	10	128304619	128305195	-
12047	1451611_at	Region	Hrasls3	225845	19	7271359	7299646	+
12048	1434674_at	Region	Lyst	17101	13	13033273	13222595	+
12049	1419022_a_at	Region	Eno1 /// LOC433182 /// LOC545568	13806	4	148729617	148741237	+
12050	1442025_a_at	Region	None	None	9	48679581	48680488	-
12051	1436229_at	Region	C130065N10Rik	319340	1	58831032	58831957	-
12052	1454487_at	Region	5830490A04Rik	76126	10	63982087	63983298	-
12053	1438941_x_at	Region	Ampd2	109674	3	107869804	107882369	-
12054	1415787_at	Region	Ganab	14376	19	8094658	8113213	+
12055	1424083_at	Region	Rod1	230257	4	59418515	59492735	-
12056	1451247_at	Region	Mfsd1	66868	3	67255079	67276542	+
12057	1450899_at	Region	Nedd1	17997	10	92653242	92690856	-

12058	1433829_a_at	Region	Hnrpa2b1	53379	6	51608402	51617123	-
12059	1441629_at	Region	B130020M22Rik	320217	10	119083490	119083894	+
12060	1426495_at	Region	2410042D21Rik	72425	2	112002449	112037475	+
12061	1448956_at	Region	Stard10	56018	7	95423673	95452600	+
12062	1452177_at	Region	Abcf3	27406	16	19320803	19332961	+
12063	1420880_a_at	Region	Ywhab	54401	2	163452166	163475556	+
12064	1434826_at	Region	AI256775	218341	13	72059883	72075496	-
12065	1436449_at	Region	None	None	X	115260331	115260889	+
12066	1419653_a_at	Region	Ddx5	13207	11	106602635	106609565	-
12067	1456116_at	Region	Catnd2	18163	15	30163743	31021141	+
12068	1460197_a_at	Region	Steap4	117167	5	7968888	7990629	+
12069	1456531_x_at	Region	Prp19	28000	19	10091789	10102029	+
12070	1454896_at	Region	None	None	5	52466436	52467501	+
12071	1422625_at	Region	Ly6h	23934	15	75615042	75616905	-
12072	1421154_at	Region	Hcn2	15166	10	79839258	79858732	+
12073	1424711_at	Region	Tmem2	83921	19	21045338	21094911	+
12074	1446803_at	Region	Dock9	105445	14	116113166	116206323	-
12075	1437843_s_at	Region	Nupl1	71844	14	54755759	54787669	-
12076	1429981_a_at	Region	4933426K21Rik	108653	6	123118089	123137380	-
12077	1428307_at	Region	Zdhhc13	243983	7	42903847	42942281	+
12078	1433645_at	Region	2210409B22Rik	70174	4	53482109	53483675	+
12079	1417124_at	Region	Dstn	56431	2	143372547	143400260	+
12080	1428510_at	Region	Lphn1	330814	8	83175087	83200065	+
12081	1436475_at	Region	Nr2f2	11819	7	64237865	64245019	-
12082	1424117_at	Region	BC056474	414077	8	84351649	84353025	+
12083	1436158_at	Region	Eif4ebp2	13688	10	61397930	61418102	-
12084	1452603_at	Region	5330431N19Rik	226162	19	45108051	45125723	+
12085	1426508_at	Region	Gfap	14580	11	102709766	102718169	-
12086	1416895_at	Region	Efna1	13636	3	89028209	89036114	-
12087	1423371_at	Region	Pole4	66979	6	82990518	82996671	-
12088	1426955_at	Region	Col18a1	12822	10	77153138	77270740	-
12089	1435752_s_at	Region	Abcc9	20928	6	143432402	143545542	-
12090	1426263_at	Region	Igsf4c	260299	7	19674773	19680043	+
12091	1444291_at	Region	4930506D23Rik	75099	7	61113618	61119077	+
12092	1452642_at	Region	None	None	15	95891608	95897370	-
12093	1443253_at	Region	None	None	NONE	NONE	NONE	NONE
12094	1435096_at	Region	BC051080	237422	10	84812276	84911107	+
12095	1455171_at	Region	Suv420h1	225888	19	3556211	3605181	+
12096	1445966_at	Region	Igf2r	16004	17	11321447	11408701	-
12097	1426620_at	Region	Chst10	98388	1	39160793	39194909	-
12098	1449944_a_at	Region	Sec61a2	57743	2	5788259	5812625	-
12099	1448766_at	Region	Gjb1	14618	X	95982922	95986986	+
12100	1433541_a_at	Region	Ubap2l	74383	3	89810481	89862883	-
12101	1439541_at	Region	4930414L22Rik	78108	6	72772008	72772590	+
12102	1450814_a_at	Region	Ipo4	75751	14	50143923	50153613	-
12103	1448293_at	Region	Ebf1	13591	11	44370971	44758012	+
12104	1454990_at	Region	Arid2	77044	15	96436802	96469595	+
12105	1416760_at	Region	Galntl1	108760	12	77379532	77465731	+
12106	1437031_at	Region	Acsl6	216739	11	54057075	54114371	+
12107	1433110_at	Region	5830474E16Rik	76094	1	22178810	22180166	-
12108	1452807_s_at	Region	1500016O10Rik	68952	7	120873047	120879946	+
12109	1415819_a_at	Region	Ppp2r1a	51792	17	18817271	18837722	+
12110	1434015_at	Region	Slc2a6	227659	2	26953530	26960152	-
12111	1429451_at	Region	2610301B20Rik	67157	4	10801573	10826390	+
12112	1417593_at	Region	Tusc2	80385	9	107629036	107631889	+
12113	1441161_at	Region	B230216G23Rik	319552	6	143257089	143262999	+
12114	1439549_at	Region	Gm368	208748	X	66623279	66633373	+
12115	1436754_at	Region	AI839735	104885	12	107979577	107990559	-
12116	1455539_at	Region	None	None	13	24467360	24470251	-
12117	1455190_at	Region	Gng7	14708	10	81080086	81084486	-
12118	1452844_at	Region	Pou6f1	19009	15	100632856	100643903	-
12119	1454775_at	Region	Hdac10	170787	15	89175231	89180553	-
12120	1428447_at	Region	Tmem14a	75712	1	21442755	21454142	+
12121	1419472_s_at	Region	Nudc /// Nudc-ps1	18221	4	132493443	132506848	-
12122	1436892_at	Region	Spred2	114716	11	19819235	19917390	+

12123	1436839_at	Region	None	None	12	28984449	28985321	+
12124	1451689_a_at	Region	Sox10	20665	15	79206217	79275996	-
12125	1435178_x_at	Region	Anapc5	59008	5	121940636	121974194	-
12126	1448982_at	Region	Prss18	19144	7	37900849	37907331	+
12127	1422506_a_at	Region	Cstb	13014	10	78536450	78538362	+
12128	1456333_a_at	Region	Arhgap6	11856	X	162395378	162903843	+
12129	1459850_x_at	Region	Glrb	14658	3	80572600	80642620	-
12130	1415782_at	Region	Sumo2	170930	11	115344202	115357323	-
12131	1425788_a_at	Region	Echdc2	52430	4	107124401	107138212	+
12132	1427985_at	Region	9630042H07Rik	270624	X	89623871	89628043	-
12133	1422530_at	Region	Prph1	19132	15	99113134	99116597	+
12134	1460387_a_at	Region	Ysg2	22619	9	37555344	37589819	+
12135	1430346_at	Region	5730507A09Rik	70638	7	58642774	58657690	-
12136	1418574_a_at	Region	Shfdg1	20422	6	6531045	6551349	-
12137	1448196_at	Region	Mat2b	108645	11	40418255	40431554	-
12138	1419745_at	Region	Arhgap23	58996	11	97311160	97323487	+
12139	1437991_x_at	Region	Rusc1	72296	3	88827845	88837154	-
12140	1433464_at	Region	Ipo13	230673	4	116853398	116873904	-
12141	1451105_at	Region	B130052G07Rik	226841	1	190684016	190715465	-
12142	1425277_at	Region	Slit1	20562	19	41144631	41288228	-
12143	1448625_at	Region	Golga2	99412	2	32220518	32240075	+
12144	1452980_at	Region	2810468N07Rik	72834	17	23376562	23380720	+
12145	1434714_at	Region	Ero1lb	67475	13	203	5259	-
12146	1447898_s_at	Region	Sfrs6	67996	2	162388514	162394090	+
12147	1434444_s_at	Region	None	None	2	128124162	128125728	-
12148	1426235_a_at	Region	Glul	14645	1	153783869	153793637	+
12149	1450005_x_at	Region	Egfl9	106565	17	43807132	43812952	+
12150	1435869_s_at	Region	Ap2a2	11772	7	135965073	136035847	+
12151	1435280_at	Region	AI452195	105178	13	106203504	106205191	+
12152	1434723_at	Region	None	None	10	39691327	39801885	-
12153	1439342_at	Region	Clpx	270166	9	65416574	65452907	+
12154	1449563_at	Region	Cntn1	12805	15	92196046	92407969	+
12155	1433765_at	Region	B230113M03Rik	217342	11	116358846	116402540	-
12156	1432270_a_at	Region	Chmp5	76959	4	41087404	41104153	+
12157	1451484_a_at	Region	Syn1	20964	X	19099459	19159931	-
12158	1424275_s_at	Region	Trim41	211007	11	48559241	48570190	-
12159	1434666_at	Region	Pcgf5	76073	19	35721737	35798874	+
12160	1451025_at	Region	Arl1	104303	10	88703486	88716220	+
12161	1418327_at	Region	1110058L19Rik	68002	1	24240602	24250296	-
12162	1451451_at	Region	Gca	227960	2	62519727	62549509	+
12163	1423462_at	Region	Map3k7ip2	68652	10	7771623	7790347	-
12164	1422473_at	Region	Pde4b	18578	4	101213908	101564744	+
12165	1444778_at	Region	38598	24050	15	82325610	82342778	+
12166	1422314_at	Region	Clcn6	26372	4	146498846	146531129	-
12167	1415727_at	Region	Apoa1bp	246703	3	87800387	87802359	-
12168	1458156_at	Region	E230012J19Rik	319664	4	148909732	148910382	+
12169	1452961_at	Region	1200009O22Rik	66873	6	53959793	53965019	-
12170	1434848_at	Region	Gpr27	14761	6	100160874	100162013	+
12171	1449514_at	Region	Gprk5	14773	19	6912	30724	-
12172	1448791_at	Region	Snx5	69178	2	143707062	143727467	-
12173	1423591_at	Region	Fgfr1op2	67529	6	147498625	147519880	+
12174	1421498_a_at	Region	2010204K13Rik	68355	NONE	NONE	NONE	NONE
12175	1455110_at	Region	None	None	3	94669597	94671651	-
12176	1428889_at	Region	1810020C19Rik	69113	2	93685235	93715406	-
12177	1438848_at	Region	Osbp	76303	19	11155242	11190607	+
12178	1416659_at	Region	Eif3s10	13669	19	60361493	60391011	-
12179	1415689_s_at	Region	Zfp306	72739	13	20867145	20884810	-
12180	1429418_at	Region	Cdc14b	218294	13	61579944	61659178	-
12181	1434840_at	Region	Hrb	15463	1	83168589	83225381	+
12182	1416208_at	Region	Usp14	59025	18	10040776	10075422	-
12183	1432408_a_at	Region	B230208H21	330549	7	54974364	55091137	-
12184	1452299_at	Region	Wwp1	107568	4	19537563	19635959	-
12185	1431372_at	Region	Srpk2	20817	5	21967253	22080468	-
12186	1438829_at	Region	Gm96	225743	18	77584095	77687047	-
12187	1448923_at	Region	Prkra	23992	2	76327686	76345743	-

12188	1416709_a_at	Region	AW552001	83485	7	74061151	74065438	+
12189	1417142_at	Region	4932442K08Rik	67544	17	13399931	13435680	+
12190	1420575_at	Region	Mt3	17751	8	93436916	93438303	+
12191	1448307_at	Region	Dscr2	56088	16	95417359	95428307	-
12192	1460677_at	Region	None	None	15	99184511	99271018	+
12193	1424441_at	Region	Slc27a4	26569	2	29734842	29749684	+
12194	1427831_s_at	Region	Zfp260	26466	7	25509675	25522233	+
12195	1426037_a_at	Region	Rgs16	19734	1	153624984	153630102	+
12196	1439817_at	Region	2900064A13Rik	73024	2	112078263	112090674	+
12197	1417780_at	Region	Lass4	67260	8	3848044	3880287	+
12198	1416321_s_at	Region	Prelp	116847	1	133761069	133772168	-
12199	1426971_at	Region	Ube1l	74153	9	108043554	108052043	+
12200	1451114_at	Region	Cklfsf6	67213	9	114704736	114722975	+
12201	1434523_x_at	Region	Eif3s6	16341	15	43195901	43228545	-
12202	1417846_at	Region	Ulk2	29869	11	61501302	61580259	-
12203	1439764_s_at	Region	C330012H03Rik	319765	16	20832300	20935863	-
12204	1434210_s_at	Region	Lrig1	16206	6	95066417	95161798	-
12205	1419673_at	Region	Spock1	20745	13	56048889	56210903	-
12206	1424093_x_at	Region	Cd151	12476	7	135869384	135873464	+
12207	1455231_s_at	Region	Apc2	23805	10	80426747	80441034	+
12208	1452874_at	Region	2510003E04Rik	72320	10	62524855	62544796	-
12209	1456413_at	Region	9430063L05Rik	229622	3	97225124	97254244	-
12210	1420666_at	Region	Doc2b	13447	11	75494748	75521715	-
12211	1450853_at	Region	Tle4	21888	19	13671934	13821845	-
12212	1426880_at	Region	BC026657	208618	2	20552057	20852534	+
12213	1417985_at	Region	Nrarp	67122	2	25113185	25115487	+
12214	1435359_at	Region	BC060632	244654	8	110019206	110039122	+
12215	1460276_a_at	Region	Gpr175	24100	6	89279841	89347284	+
12216	1435195_at	Region	D930046M13Rik	104880	12	83567774	83569620	+
12217	1450081_x_at	Region	Gpi1	14751	7	29355949	29384053	-
12218	1459358_at	Region	Pkp2	67451	16	14981080	15040447	+
12219	1456250_x_at	Region	Tgfbi	21810	13	55229952	55259688	+
12220	1444923_at	Region	None	None	12	71742721	71743618	-
12221	1415794_a_at	Region	Spin	20729	13	67875489	67920302	-
12222	1460008_x_at	Region	Rpl31	114641	1	39664836	39668824	+
12223	1448788_at	Region	Cd200	17470	16	44266133	44292916	-
12224	1436908_at	Region	Pcm1	18536	8	40177407	40269978	+
12225	1434002_at	Region	Ches1	71375	12	94622265	94816088	-
12226	1442704_at	Region	None	None	2	59877948	59878354	-
12227	1450428_at	Region	Lhx1	16869	11	84247371	84253526	-
12228	1457568_at	Region	C230004L04	330135	5	98975085	98975685	-
12229	1446805_at	Region	Gabra2	14395	5	69741965	69877243	-
12230	1438858_x_at	Region	H2-Aa	14960	17	31984134	31989153	-
12231	1421028_a_at	Region	Mef2c	17260	13	79604158	79763912	+
12232	1436828_a_at	Region	Tpd52l2	66314	2	181214210	181233714	+
12233	1424132_at	Region	Hras1	15461	7	135593335	135594927	-
12234	1442415_at	Region	5830454E08Rik	76100	9	120589590	120609801	+
12235	1451762_a_at	Region	Kif1b	16561	4	147668683	147799980	-
12236	1417503_at	Region	Rfc2	19718	5	93150	108790	-
12237	1423728_at	Region	Eif3s6ip	223691	15	79126509	79145686	+
12238	1422078_at	Region	Akt3	23797	1	176955410	177064396	-
12239	1458575_at	Region	Setbp1	240427	18	78876677	79209594	-
12240	1457589_at	Region	D430038H04Rik	270120	9	15750373	15766277	-
12241	1448005_at	Region	Sash1	70097	10	8593638	8757302	-
12242	1421141_a_at	Region	Foxp1	108655	6	99395348	99630926	-
12243	1437413_x_at	Region	Rps29	20090	12	65989514	65990859	-
12244	1439194_at	Region	C030048H21Rik	77481	2	26175614	26176728	-
12245	1424897_at	Region	Gpr85	64450	6	13822022	13826799	-
12246	1434537_at	Region	Slco3a1	108116	7	68181298	68451736	-
12247	1425628_a_at	Region	Gtf2i	14886	5	133529235	133606084	-
12248	1423729_a_at	Region	2500002L14Rik	66510	6	72691688	72694355	-
12249	1433834_at	Region	38417	223455	15	31457571	31531643	-
12250	1458341_x_at	Region	None	None	3	121650632	121650889	+
12251	1460650_at	Region	Atp6v0a1	11975	11	100830569	100884801	+
12252	1419238_at	Region	Abca7	27403	10	80120249	80138206	+

12253	1428636_at	Region	Steap2	74051	5	5676943	5699766	-
12254	1444523_s_at	Region	Ube2v1	66589	2	167064579	167088725	-
12255	1457401_at	Region	Dnahc9	237806	11	65556977	65846480	-
12256	1456759_at	Region	6430556C10Rik	241568	2	97172340	97336347	+
12257	1452207_at	Region	None	None	10	17647349	17649770	+
12258	1428201_at	Region	2310036O22Rik	68544	8	84297511	84300924	+
12259	1455142_at	Region	Socs4	67296	14	42361847	42376298	+
12260	1418752_at	Region	Aldh3a1	11670	11	60934387	60944056	+
12261	1426490_at	Region	Bfar	67118	16	12408146	12438648	+
12262	1428948_at	Region	5730414M22Rik	70528	14	21670471	21672182	-
12263	1427923_at	Region	Zmpste24	230709	4	120081682	120120687	-
12264	1427151_at	Region	4732486I23Rik	99003	2	104462387	104521514	-
12265	1424177_at	Region	Tmem38a	74166	8	71722899	71738144	+
12266	1425329_a_at	Region	Dia1	109754	15	83204176	83222883	-
12267	1426994_at	Region	Phlpp	98432	1	106112969	106335347	+
12268	1431007_at	Region	LOC381062	381062	17	13026322	13036818	+
12269	1417904_at	Region	Dclre1a	55947	19	56114717	56132771	-
12270	1448545_at	Region	Sdc2	15529	15	32919852	33033850	+
12271	1424976_at	Region	Rhov	228543	2	118783050	118785078	-
12272	1416381_a_at	Region	Prdx5	54683	19	6620667	6623493	-
12273	1460561_x_at	Region	Sepw1	20364	7	4226	7136	-
12274	1436760_a_at	Region	Rps8	20116	4	116112741	116115037	-
12275	1440350_at	Region	None	None	15	89121846	89122825	+
12276	1435094_at	Region	Kcnj16	16517	11	110789161	110849057	+
12277	1434701_at	Region	AW215868	104611	11	60283372	60283948	+
12278	1454922_at	Region	AI553587	103784	11	17106711	17128589	+
12279	1428538_s_at	Region	Rarres2	71660	6	48702577	48705549	-
12280	1418468_at	Region	Anxa11	11744	14	24217620	24262677	+
12281	1457266_at	Region	AW322671	105016	12	70199679	70200254	+
12282	1450440_at	Region	Gfra1	14585	19	57822423	58054639	-
12283	1428158_at	Region	Akt1s1	67605	7	38924155	38930338	+
12284	1426264_at	Region	Dlat	235339	9	50706961	50732106	-
12285	1454142_a_at	Region	Pwp1	103136	10	85832809	85850040	+
12286	1427773_a_at	Region	Rabac1	14470	7	20146731	20149637	-
12287	1447979_at	Region	Rutbc2	52850	5	112319917	112387408	-
12288	1423307_s_at	Region	Tgoln1 /// Tgoln2	22134	6	72943992	72950517	-
12289	1451295_a_at	Region	Chd4	107932	6	125752167	125786488	+
12290	1448379_at	Region	Pot1	101185	6	25788490	25853950	-
12291	1435668_at	Region	None	None	17	13436445	13437236	+
12292	1448111_at	Region	Ctps2	55936	X	156501010	156631724	+
12293	1436501_at	Region	Mtus1	102103	8	39926813	40021115	-
12294	1417004_at	Region	Tsg101	22088	7	40975338	41005870	-
12295	1429862_at	Region	2310026J01Rik	329502	2	119686022	119758931	-
12296	1430603_at	Region	4930579K19Rik	75881	9	98461587	98462950	-
12297	1435048_at	Region	AI854703	243373	6	48760814	48766567	+
12298	1431765_a_at	Region	Rps2 /// LOC193707 /// LOC232534 /// LOC243642 /// LOC380872 /// LOC381086 /// LOC432878 /// LOC433715 /// LOC434084 /// LOC436484 /// LOC544872 /// LOC545808 /// LOC546164 /// LOC546298 /// LOC546797	16898	17	22525236	22527051	+
12299	1416159_at	Region	Nr2f2	11819	7	64237865	64245019	-
12300	1429532_at	Region	Morc2a	74522	11	3553487	3585159	+
12301	1424702_a_at	Region	2410024A21Rik	76559	12	101063610	101094310	-
12302	1459986_a_at	Region	Rps17	20068	7	75150291	75152774	-
12303	1456606_a_at	Region	Chst11 /// Phactr1	218194	13	42242828	42700966	+

12304	1441325_at	Region	9430034D17Rik	77286	X	31508160	31510142	-
12305	1452834_at	Region	2600010E01Rik	72446	2	101420931	101502300	-
12306	1452780_at	Region	Gtf3c2	71752	5	29615739	29638859	-
12307	1416072_at	Region	Cd34	12490	1	194679286	194701657	+
12308	1441430_at	Region	None	None	3	129567099	129567575	+
12309	1423880_at	Region	D10Wsu52e	28088	10	85899581	85918735	-
12310	1436745_at	Region	Scyl1bp1	98376	1	163300126	163318858	-
12311	1434405_at	Region	A730024A03Rik	216742	11	54191030	54271077	+
12312	1435975_at	Region	AI115600	102442	9	65003770	65041605	+
12313	1451154_a_at	Region	Cugbp2	14007	2	6459141	6638885	-
12314	1420842_at	Region	Ptprf	19268	4	117168706	117236351	-
12315	1455141_at	Region	Tnrc6a	233833	7	117180427	117250357	+
12316	1448387_at	Region	Rbx1	56438	15	81517173	81527164	+
12317	1415688_at	Region	Ube2g1	67128	11	72333039	72409965	+
12318	1433997_at	Region	2410127E18Rik	76788	6	30449002	30501701	+
12319	1416237_at	Region	Eva1	14012	9	45031412	45042867	+
12320	1456602_at	Region	4932417I16Rik	234740	8	111209596	111211461	-
12321	1424365_at	Region	1810037I17Rik	67704	X	1870	3216	-
12322	1427476_a_at	Region	Trim32	69807	4	64696209	64707429	+
12323	1456213_x_at	Region	Qars	97541	9	108556974	108564815	+
12324	1415683_at	Region	Nmt1	18107	11	102849651	102887194	+
12325	1419509_a_at	Region	Nagk	56174	6	84139275	84146789	+
12326	1419210_at	Region	Hrh1	15465	6	114980341	115054903	+
12327	1426961_at	Region	6820402O20Rik	228829	2	155653612	155764373	+
12328	1426451_at	Region	6030465E24Rik	214585	2	121567379	121632227	-
12329	1416002_x_at	Region	Cotl1	72042	8	119165392	119197206	-
12330	1424442_a_at	Region	Pja2	224938	17	61986425	62035635	-
12331	1435784_at	Region	Atg9l1	245860	1	75468642	75479736	-
12332	1421095_a_at	Region	Trpc1	22063	9	95610171	95653902	-
12333	1415895_at	Region	Snrpn	20646	7	53759592	53781697	-
12334	1449388_at	Region	Thbs4	21828	13	88935778	88978946	-
12335	1424200_s_at	Region	Seh1l	72124	18	68005761	68023379	+
12336	1452330_a_at	Region	Mxra8	74761	4	154332120	154336450	+
12337	1439459_x_at	Region	Acly	104112	11	100297443	100348988	-
12338	1434515_at	Region	Ncoa1	17977	12	3409624	3575830	-
12339	1427445_a_at	Region	Ttn	22138	2	76401836	76406317	-
12340	1422869_at	Region	Mertk	17289	2	128212850	128316041	+
12341	1448596_at	Region	Slc6a8	102857	X	68333805	68343157	+
12342	1429767_at	Region	2810013P06Rik	67206	8	122423784	122425711	+
12343	1424547_at	Region	Car10	72605	11	92919497	93422360	+
12344	1428274_s_at	Region	1110065L07Rik	68904	8	9345221	9357725	+
12345	1451504_at	Region	Chchd3	66075	6	32872201	33140615	-
12346	1449465_at	Region	Reln	19699	5	20336833	20797054	-
12347	1423214_at	Region	Plxnc1	54712	10	94767461	94918749	-
12348	1420530_at	Region	Neud4	29861	7	24715394	24728970	+
12349	1454682_at	Region	A430005L14Rik	97159	4	152449599	152454276	+
12350	1427114_at	Region	Ttc19	72795	11	62007122	62040890	+
12351	1429288_x_at	Region	Stx18	71116	5	36545458	36643042	+
12352	1450728_at	Region	Fjx1	14221	2	102154049	102156475	-
12353	1426976_at	Region	Usp47	74996	7	105876643	105964517	+
12354	1428562_at	Region	2210403K04Rik	67098	11	75187288	75192350	+
12355	1455352_at	Region	AU023006	102683	9	95365380	95368889	-
12356	1439946_at	Region	Mef2c	17260	13	79604158	79763912	+
12357	1416049_at	Region	Gldc	104174	19	29351052	29427324	-
12358	1417013_at	Region	Hspb8	80888	5	115530010	115544379	-
12359	1421017_at	Region	Nrg3	18183	14	36505478	36542277	-
12360	1456520_at	Region	9530033F24Rik	268469	11	95584927	95586264	+
12361	1436321_at	Region	B3gnt7	227327	1	86109779	86113616	+
12362	1417113_at	Region	Gcl	23885	6	87126075	87167665	-
12363	1415797_at	Region	Ddr1	12305	17	33397888	33421229	-
12364	1434824_at	Region	Baz1b	22385	5	134200851	134258059	+
12365	1456380_x_at	Region	Cnn3	71994	3	120205773	120237098	+
12366	1423049_a_at	Region	Tpm1	22003	9	67151552	67178023	-
12367	1421124_at	Region	Cdk5r1	12569	11	80202705	80206840	+
12368	1436349_at	Region	2700094K13Rik	72657	2	84366970	84368431	-

12369	1436902_x_at	Region	Tmsb10	19240	6	20039912	20040386	+
12370	1418444_a_at	Region	MGI:1891827	56209	7	112564415	112581595	-
12371	1447771_at	Region	None	None	NONE	NONE	NONE	NONE
12372	1435720_at	Region	None	None	3	105468784	105469832	+
12373	1437975_a_at	Region	Rpl23a	268449	11	77906594	77909202	-
12374	1421890_at	Region	St3gal2	20444	8	110217644	110269279	+
12375	1448218_s_at	Region	Ywhaz	22631	15	36773662	36797651	-
12376	1423083_at	Region	Rab33b	19338	3	51115980	51128158	+
12377	1426554_a_at	Region	Pgam1	18648	19	41456332	41463037	+
12378	1419628_at	Region	Chx10	12677	12	81438199	81463804	+
12379	1441145_at	Region	D030065N23Rik	399586	2	92037458	92038145	+
12380	1442359_at	Region	AW045895	100891	5	5674532	5675294	-
12381	1434749_at	Region	BC067068	216292	10	105655737	105733844	-
12382	1428505_at	Region	2310015N07Rik	66365	7	2391	6258	-
12383	1448442_a_at	Region	Psma3	19167	12	67809701	67829929	+
12384	1442581_at	Region	Ksr	16706	11	78740461	78872020	-
12385	1423914_at	Region	C630004H02Rik	217310	11	115168802	115188812	-
12386	1434872_x_at	Region	None	None	6	34904862	34905086	-
12387	1426110_a_at	Region	Edg2	14745	4	58378433	58496968	-
12388	1449138_at	Region	Sf3b1	81898	1	55286605	55333156	-
12389	1415977_at	Region	MGI:1919030	71780	8	69750834	69753776	+
12390	1424736_at	Region	Eef2	13629	10	81312692	81318533	+
12391	1428217_at	Region	1600012H06Rik	67912	17	12995900	12998652	+
12392	1416217_a_at	Region	Rpl37a	19981	1	73012971	73015366	+
12393	1439113_at	Region	Hpcal1 /// 2410018L13Rik /// LOC245297 /// LOC544836 /// LOC544845 /// LOC544850	245297	12	17297145	17297932	+
12394	1416563_at	Region	Ctps	51797	4	119562310	119592708	-
12395	1435630_s_at	Region	Acat2	110460	17	11571688	11589509	+
12396	1456381_x_at	Region	Mcl1	17210	3	95146619	95151011	+
12397	1454650_at	Region	Trim35	66854	14	60824516	60836924	+
12398	1418817_at	Region	Chmp1b	67064	18	67434757	67436335	+
12399	1456216_at	Region	Csnk1a1	93687	18	61784394	61817111	+
12400	1418119_at	Region	Rbm8a	60365	3	96117769	96120746	+
12401	1424285_s_at	Region	Arl6ip4	65105	5	123287433	123289520	+
12402	1431771_a_at	Region	Irak1bp1	65099	9	83159766	83177453	+
12403	1428536_at	Region	Kcng4	66733	8	118977994	118989820	-
12404	1445827_at	Region	Prkcbp1	228880	2	165242034	165353683	-
12405	1447891_at	Region	None	None	7	124871214	124871393	+
12406	1451312_at	Region	Ndufs7	75406	10	80374361	80381701	+
12407	1422643_at	Region	Moxd1	59012	10	24198081	24277347	+
12408	1443353_at	Region	None	None	15	11942165	11942660	+
12409	1450033_a_at	Region	Stat1	20846	1	52420399	52461905	+
12410	1437172_x_at	Region	Hadhb	231086	5	28621184	28642098	+
12411	1455428_at	Region	A930008G19Rik	77938	7	127078161	127179580	-
12412	1456201_at	Region	4632427E13Rik	68186	7	86777643	86778378	-
12413	1434281_at	Region	1500034J01Rik	66498	8	70619056	70625966	+
12414	1424562_a_at	Region	Slc25a4	11739	8	45151043	45155041	-
12415	1447583_x_at	Region	Gpr30	76854	5	138420671	138425280	+
12416	1435612_at	Region	Opcml	330908	9	28570800	28821443	+
12417	1436971_x_at	Region	Ywhaz	22631	15	36773662	36797651	-
12418	1434087_at	Region	Mthfr	17769	4	146533584	146551911	+
12419	1441414_at	Region	2310047I15Rik	78887	11	3026676	3091300	-
12420	1434193_at	Region	Zfp258	100177	4	126104496	126151407	+
12421	1439368_a_at	Region	Slc9a3r2	65962	17	22442873	22453949	-
12422	1426234_s_at	Region	BC002199	211556	3	126691354	126692954	-
12423	1415738_at	Region	Txndc12	66073	4	107793583	107821025	+
12424	1435454_a_at	Region	BC006779	229003	2	180944576	180954007	-
12425	1439527_at	Region	BB114106	102824	9	8978959	8979854	+
12426	1425756_at	Region	Rab40b	217371	11	121177896	121209232	-
12427	1449938_at	Region	Pp11r	19011	15	97786664	97796795	-
12428	1434429_at	Region	Syt16	238266	12	70980664	71124581	+

12429	1421924_at	Region	Slc2a3	20527	6	123394798	123409491	-
12430	1426238_at	Region	Bmp1	12153	14	64790547	64836177	-
12431	1428325_at	Region	2610019P18Rik	66455	5	137160349	137166708	+
12432	1418716_at	Region	Mrps25	64658	6	92621555	92631448	-
12433	1434190_at	Region	Sms	20603	X	151043359	151091439	-
12434	1442037_at	Region	LOC434238	434238	7	116939048	116944447	+
12435	1447806_s_at	Region	Stk23	56504	X	68435077	68439580	+
12436	1426773_at	Region	Mfn1	67414	3	31937664	31985854	+
12437	1449271_a_at	Region	Hebp2	56016	10	18464596	18470549	-
12438	1438473_at	Region	Arfrp2	218639	13	110208602	110571472	+
12439	1425886_at	Region	Fev	260298	1	75185718	75189628	-
12440	1418464_at	Region	Matn4	17183	2	163846367	163861893	-
12441	1451062_a_at	Region	Pex2	58869	3	32358790	32491207	-
12442	1455348_x_at	Region	Rpl29 /// LOC433350 /// LOC433383 /// LOC433941	19944	9	106423093	106425121	+
12443	1429897_a_at	Region	D16Ertd472e	67102	16	77629000	77662117	-
12444	1417652_a_at	Region	Tbca	21371	13	90973009	91026965	+
12445	1442079_at	Region	Tmem23	208449	19	31448202	31573509	-
12446	1437174_at	Region	Tfdp2	211586	9	96095461	96217969	+
12447	1432750_at	Region	2810409C01Rik	69243	X	107033291	107034548	+
12448	1435484_at	Region	BF642829	503859	16	91243262	91244718	+
12449	1428821_at	Region	Agpat2	67512	2	26525749	26536372	-
12450	1427202_at	Region	4833442J19Rik	320204	6	150077480	150087054	+
12451	1428587_at	Region	Tmem41b	233724	7	103825350	103839101	-
12452	1436035_at	Region	3830431G21Rik	217682	12	77553846	77585467	+
12453	1448640_at	Region	Slc14a1	108052	18	78222739	78245970	-
12454	1417839_at	Region	Cldn5	12741	16	17547897	17549310	+
12455	1445767_at	Region	Ptprd	19266	NONE	NONE	NONE	NONE
12456	1422541_at	Region	Ptprm	19274	17	64435036	65122417	-
12457	1428289_at	Region	Klf9	16601	19	22379147	22404832	+
12458	1460607_at	Region	Igsf11	207683	16	37725194	37850220	+
12459	1424720_at	Region	Mgat4b	103534	11	49978474	49987491	+
12460	1435763_at	Region	Tbc1d16	207592	11	118968230	119049592	-
12461	1458592_at	Region	None	None	5	130812544	130812991	-
12462	1422538_at	Region	Extl2	58193	3	114777993	114799388	+
12463	1421181_at	Region	Nptxr	73340	15	79841252	79857012	-
12464	1434443_at	Region	None	None	2	128124162	128125728	-
12465	1421491_a_at	Region	Tmem49	75909	11	86311857	86411814	-
12466	1418053_at	Region	Sncb	104069	13	53366552	53374115	-
12467	1422500_at	Region	Idh3a	67834	9	54704020	54722171	+
12468	1416684_at	Region	Fbl /// LOC545184	14113	7	23565500	23575021	+
12469	1416175_a_at	Region	Vdac3	22335	8	21331680	21348337	-
12470	1439619_at	Region	Tcf12	21406	9	71980605	72246476	-
12471	1429649_at	Region	Slc35a3	229782	3	115439734	115481216	-
12472	1451264_at	Region	4930488L10Rik	319710	12	67659295	67737242	+
12473	1455170_at	Region	2810001G20Rik	66456	11	63805232	63808909	+
12474	1435297_at	Region	Gja9	14617	2	113523441	113527459	-
12475	1442502_at	Region	Lrch1	380916	14	69108806	69291857	-
12476	1445873_at	Region	Tfdp2	211586	9	96095461	96217969	+
12477	1437742_at	Region	Rab21	216344	10	114975874	115001563	-
12478	1450937_at	Region	Lin7c	22343	2	109514665	109524724	+
12479	1442101_at	Region	A930017N06Rik	243312	5	138904283	138971062	+
12480	1436177_at	Region	Plekha2	83436	8	23764086	23824498	-
12481	1415945_at	Region	Mcm5	17218	8	74273686	74291750	+
12482	1441023_at	Region	Eif2s2	67204	2	154328353	154349719	-
12483	1451743_at	Region	D19Wsu162e	226178	19	46146382	46202440	+
12484	1438404_at	Region	Rnf144	108089	12	22852685	22961373	-
12485	1455468_at	Region	9330164H19Rik	233115	7	30851673	30917234	-
12486	1456570_at	Region	6430704M03Rik	230235	4	56903585	56933806	-
12487	1451169_at	Region	Nomo1	211548	7	40119166	40169552	+
12488	1455727_at	Region	U2af1-rs2	22184	X	157534848	157558003	-
12489	1435870_at	Region	Sycp3	20962	10	88431421	88445069	+
12490	1422569_at	Region	Yy1	22632	12	104264095	104287625	+

12491	1459971_at	Region	None	None	1	140488179	140488433	+
12492	1437437_x_at	Region	Dnpep	13437	1	75598827	75608568	-
12493	1460260_s_at	Region	Kpna1	16646	16	34764772	34817569	+
12494	1455101_at	Region	None	None	10	13089390	13089847	-
12495	1420508_at	Region	Sema3f	20350	9	107747279	107776252	-
12496	1427012_at	Region	Lancl1	14768	1	67292813	67331161	-
12497	1420731_a_at	Region	Csrp2	13008	10	110543027	110562470	+
12498	1435456_at	Region	AI428795	209683	5	110273612	110336823	+
12499	1426187_a_at	Region	Hax1	23897	3	89805798	89809027	-
12500	1438455_at	Region	C330050A14Rik	78704	3	46037530	46038398	-
12501	1440866_at	Region	Prkr	19106	17	76674799	76704807	-
12502	1452309_at	Region	Cgnl1	68178	9	71761361	71906454	-
12503	1439009_at	Region	Gnl3l	237107	X	144517536	144551643	-
12504	1456114_at	Region	Cds1	74596	5	100785901	100844694	+
12505	1428561_at	Region	2610002J23Rik	69886	11	100278210	100279249	-
12506	1445600_at	Region	A330103N21Rik	77773	7	61919158	61919541	+
12507	1460506_s_at	Region	Ndufc2	68197	7	91446361	91454068	+
12508	1456193_x_at	Region	None	None	10	46037249	46037583	+
12509	1450522_a_at	Region	H1f0	14958	15	79079749	79081790	+
12510	1450561_a_at	Region	Surf1	20930	2	26845598	26848493	-
12511	1457966_at	Region	None	None	15	26643761	26644191	-
12512	1426657_s_at	Region	Phgdh	236539	3	97799380	97826178	-
12513	1458089_at	Region	Fkbp5	14229	17	26200322	26287578	-
12514	1450567_a_at	Region	Col2a1	12824	15	98051602	98080055	-
12515	1426289_at	Region	2610028H07Rik	69232	9	108566005	108594371	+
12516	1442243_at	Region	Per3	18628	4	149497074	149536951	-
12517	1440361_at	Region	None	None	4	37091817	37126884	+
12518	1437856_at	Region	Ipmk	69718	10	71401760	71439983	+
12519	1448870_at	Region	Ltbp1	268977	17	72812765	73202010	+
12520	1418767_at	Region	Cyp4f13	170716	17	30748639	30771938	-
12521	1456316_a_at	Region	Acbd3	170760	1	180679954	180707985	+
12522	1455115_a_at	Region	Crb3	224912	17	54752222	54755863	+
12523	1415775_at	Region	Rbbp7	245688	X	156359864	156378495	+
12524	1434648_a_at	Region	Ccm2	216527	11	6441744	6491532	+
12525	1451231_a_at	Region	Cul2	71745	18	3387735	3440960	+
12526	1449263_at	Region	Ufm1	67890	3	53489432	53497992	-
12527	1452738_at	Region	Stoml1	69106	9	58367732	58377239	+
12528	1451917_a_at	Region	Dcamkl1	13175	3	54876964	55171014	+
12529	1448694_at	Region	Jun	16476	4	94024127	94027259	-
12530	1415747_s_at	Region	Riok3	66878	18	12316360	12344298	+
12531	1433910_at	Region	Zcchc6	214290	13	58431477	58482699	-
12532	1437214_at	Region	Lrrtm4	243499	6	80352899	80359760	+
12533	1417804_at	Region	LOC381240	381240	19	6189191	6203905	+
12534	1456590_x_at	Region	Akr1b3 /// LOC432549	11677	6	34398202	34411739	-
12535	1439825_at	Region	Dtx3l	209200	16	34709546	34710081	-
12536	1456573_x_at	Region	Nnt	18115	13	10658	16286	-
12537	1455229_x_at	Region	4933424M23Rik /// LOC260345	260345	11	117826678	117845105	+
12538	1435595_at	Region	1810011O10Rik	69068	8	23192443	23193773	-
12539	1438627_x_at	Region	Pgd	110208	4	147642356	147659030	-
12540	1457374_at	Region	Nedd4l	83814	18	65256461	65446324	+
12541	1455655_a_at	Region	Tardbp	230908	4	147104744	147119358	-
12542	1438199_at	Region	AI316807	102032	8	21214785	21228840	-
12543	1443957_at	Region	Dixdc1	330938	9	50735078	50800301	-
12544	1455455_at	Region	4732486J07Rik	320302	3	85613266	85630813	-
12545	1416559_at	Region	1500003O22Rik	101867	7	99846861	99851152	-
12546	1442419_at	Region	None	None	18	40760490	40761167	+
12547	1438009_at	Region	Hist1h2ad	319165	13	23054539	23054931	+
12548	1442077_at	Region	2310076G05Rik	76963	9	81953359	81954073	-
12549	1421860_at	Region	Clstn1	65945	4	148079000	148140261	+
12550	1434547_at	Region	Cpd	12874	11	76507648	76572655	-
12551	1433611_s_at	Region	None	None	5	144178885	144186515	+
12552	1454015_a_at	Region	Cdh13	12554	8	118009574	118667271	+
12553	1427620_at	Region	Parc	78309	17	44009963	44037654	-

12554	1449302_at	Region	Abca2	11305	2	25360932	25380361	+
12555	1423507_a_at	Region	Sirt2	64383	7	24172893	24194867	+
12556	1451425_a_at	Region	Mkrn1	54484	6	39533206	39555816	-
12557	1456909_at	Region	Gpi1 /// Gm1840	14751	7	29355949	29384053	-
12558	1428675_at	Region	1110049F12Rik	66193	4	134856862	134868502	-
12559	1418086_at	Region	Ppp1r14a	68458	7	24700565	24704617	+
12560	1449281_at	Region	Nrtn	18188	17	54441310	54447662	-
12561	1456176_x_at	Region	D11Ertd333e	68066	11	102224234	102228559	-
12562	1438022_at	Region	Rab11fip3	215445	17	23782207	23862685	-
12563	1451097_at	Region	Vasp	22323	7	16126947	16134057	-
12564	1417573_at	Region	2010311D03Rik	109129	2	50212043	50228839	-
12565	1460671_at	Region	Gpx1	14775	9	108408939	108410079	+
12566	1432705_at	Region	5330428N10Rik	78277	10	108524259	108525835	-
12567	1421415_s_at	Region	Gcnt2	14538	13	40420765	40521482	+
12568	1437021_at	Region	Arl2l1	68146	16	61787624	61834525	-
12569	1438931_s_at	Region	Sesn1	140742	10	42013946	42014164	+
12570	1434377_x_at	Region	Rps6	20104	4	85840854	85843549	-
12571	1423484_at	Region	Bicc1	83675	10	70979053	71213601	-
12572	1423550_at	Region	Slc1a4	55963	11	20196998	20227464	-
12573	1428388_at	Region	Tnks2	74493	19	36161291	36235550	+
12574	1453283_at	Region	Pgm1	66681	5	62890880	62926108	+
12575	1448265_x_at	Region	Eva1	14012	9	45031412	45042867	+
12576	1424659_at	Region	Slit2	20563	5	46748038	47069334	+
12577	1455378_at	Region	BC057371	194237	4	118424188	118451484	-
12578	1438973_x_at	Region	Gja1	14609	10	56627159	56640230	+
12579	1451135_at	Region	Gtf2b	229906	3	141734221	141752580	+
12580	1446244_at	Region	D6Bwg1452e	242610	6	107189346	107259993	-
12581	1459214_at	Region	Odz2	23964	11	35801678	36738275	-
12582	1417685_at	Region	Ankfy1	11736	11	72437815	72496879	+
12583	1424528_at	Region	Cgref1	68567	5	29392087	29404535	-
12584	1456752_at	Region	None	None	2	173691335	173691965	-
12585	1435162_at	Region	Prkg2	19092	5	97950831	98055222	-
12586	1437597_at	Region	None	None	7	55299710	55667682	-
12587	1458830_at	Region	4931432E15Rik	70991	14	10630860	10631610	+
12588	1437331_a_at	Region	Arf3	11842	15	98795066	98820699	-
12589	1432757_at	Region	2900011L18Rik	77082	13	104041848	104042951	-
12590	1421075_s_at	Region	Cyp7b1	13123	3	17402749	17573964	-
12591	1456243_x_at	Region	Mcl1	17210	3	95146619	95151011	+
12592	1456862_at	Region	Six4	20474	12	69946438	69959424	-
12593	1433712_at	Region	AW555464	217882	12	108218013	108225980	+
12594	1424776_a_at	Region	Slc25a28	246696	19	43208207	43219287	-
12595	1450963_at	Region	Hnrpf	98758	6	118349909	118358058	+
12596	1434249_s_at	Region	None	None	12	67076694	67077367	-
12597	1417783_at	Region	Als2	74018	1	59468051	59542338	-
12598	1416867_at	Region	Bet1	12068	6	4029552	4039575	-
12599	1453181_x_at	Region	Plscr1	22038	9	92150623	92163255	+
12600	1417928_at	Region	Pdlim4	30794	11	53807773	53821809	-
12601	1431766_x_at	Region	Rps2	16898	17	22525236	22527051	+
12602	1450422_a_at	Region	Kdelc1	72050	1	44402365	44414592	-
12603	1428749_at	Region	Dmxl2	235380	9	54479317	54533979	-
12604	1426372_a_at	Region	Bet1l	54399	7	135255293	135258149	-
12605	1420908_at	Region	Cd2ap	12488	17	40292779	40376218	-
12606	1421567_at	Region	Npas3	27386	12	50702581	50824492	+
12607	1449228_at	Region	Sh3gl2	20404	4	84191648	84375572	+
12608	1415749_a_at	Region	Rragc	54170	4	122944623	122964102	+
12609	1417166_at	Region	Psip1	101739	4	82441872	82472582	-
12610	1439654_at	Region	LOC319225	319225	9	100448950	100449985	+
12611	1433928_a_at	Region	Rpl13a	22121	7	39202318	39205277	-
12612	1435206_at	Region	Slc24a4	238384	12	97574903	97712233	+
12613	1439802_at	Region	Stk35	67333	2	129314605	129341840	+
12614	1455994_x_at	Region	Elovl1	54325	4	117387050	117391522	+
12615	1431316_at	Region	Itch	16396	2	154590446	154683791	+
12616	1426406_at	Region	2410195B05Rik	67956	5	123611273	123633650	+
12617	1437100_x_at	Region	Pim3	223775	15	88914123	88917655	+
12618	1423318_at	Region	Rad18	58186	6	113184164	113259954	-

12619	1423822_a_at	Region	8430437G11Rik	101118	6	13567638	13595014	-
12620	1424071_s_at	Region	BC018507	218333	13	66640392	66677128	-
12621	1434232_a_at	Region	None	None	X	66483448	66485362	+
12622	1423871_at	Region	BC014795	208795	1	180896338	180928924	+
12623	1455778_at	Region	Zfp192	93681	13	20999835	21011161	-
12624	1435675_at	Region	Tbc1d12	209478	19	38180383	38263474	+
12625	1437094_x_at	Region	Dnaic1	68922	4	41708645	41777001	+
12626	1424276_at	Region	Snx16	74718	3	10419694	10441634	-
12627	1450729_at	Region	Hs2st1	23908	3	143402785	143541813	-
12628	1456227_x_at	Region	Rbbp7	245688	X	156359864	156378495	+
12629	1417574_at	Region	Cxcl12	20315	6	117603898	117616686	+
12630	1452416_at	Region	Il6ra	16194	3	89634609	89719225	-
12631	1456180_at	Region	LOC380843	380843	13	46016804	46028743	+
12632	1451270_at	Region	Dusp18	75219	11	3790068	3796083	+
12633	1424013_at	Region	Etf1	225363	18	35127577	35155611	-
12634	1418198_a_at	Region	Tm9sf1	74140	14	50153981	50161821	-
12635	1460081_at	Region	Syt7	54525	19	9585980	9644941	+
12636	1424250_a_at	Region	Arhgef3	71704	14	25376244	25542109	+
12637	1417045_at	Region	Bid	12122	6	121323904	121348810	-
12638	1415830_at	Region	Orc5l	26429	5	20939068	21004775	-
12639	1441462_at	Region	Dock4	238130	12	37409375	37410068	+
12640	1418996_a_at	Region	4930469P12Rik	67636	6	146057599	146063382	+
12641	1418651_at	Region	Spata6	67946	4	110678911	110787339	+
12642	1425700_at	Region	Grm1	14816	10	10563608	10961343	-
12643	1456763_at	Region	AA536749	26936	11	59388161	59501851	+
12644	1436084_at	Region	Scrt1	170729	15	76566591	76572559	-
12645	1423692_at	Region	Ndufa8	68375	2	35968496	35981454	-
12646	1427125_s_at	Region	Lrrc41	230654	4	115034370	115055948	+
12647	1416285_at	Region	Ndufc1	66377	3	51037443	51040919	-
12648	1429501_s_at	Region	Ppm1a	19042	12	69630561	69641441	+
12649	1455465_at	Region	None	None	NONE	NONE	NONE	NONE
12650	1448900_at	Region	D16H22S680E	27883	16	17071867	17095487	-
12651	1419545_a_at	Region	Atp6v1c1	66335	15	38667202	38697131	+
12652	1434178_at	Region	Mll3	231051	5	23741347	23769624	-
12653	1427050_at	Region	5730420B22Rik	70561	14	2827	4349	+
12654	1448559_at	Region	Flot1	14251	17	33540621	33550054	+
12655	1433864_at	Region	Lrp12	239393	15	39764001	39836637	-
12656	1425853_s_at	Region	Prlr	19116	15	9991399	10143512	+
12657	1448937_at	Region	Slc35b3	108652	13	38472884	38501276	-
12658	1433565_at	Region	Prpf38a	230596	4	107523775	107538232	-
12659	1452277_at	Region	6330406P08Rik	74008	11	109310969	109394419	+
12660	1423749_s_at	Region	Rangap1	19387	15	81754967	81780618	-
12661	1437123_at	Region	Mmrn2	105450	14	32512110	32539866	+
12662	1447745_at	Region	Aqp4	11829	18	15581082	15591914	-
12663	1437195_x_at	Region	Mapk10	26414	5	101946583	102247027	-
12664	1422795_at	Region	Cul3	26554	1	80599109	80655022	-
12665	1433490_s_at	Region	Epb4.1l2	13822	10	25420347	25491840	+
12666	1423675_at	Region	Usp1	230484	4	97898883	97910606	+
12667	1433769_at	Region	Als2cl	235633	9	110921471	110941821	+
12668	1426358_at	Region	Taok1	216965	11	77255718	77311108	-
12669	1451990_at	Region	Mapre2	212307	18	24032480	24122349	+
12670	1454514_at	Region	5730415C11Rik	70522	2	174275547	174277376	-
12671	1434944_at	Region	Dmpk	13400	7	15952756	15961863	+
12672	1452075_at	Region	4933435A13Rik	74467	11	23560487	23626315	+
12673	1450965_at	Region	Tex261	21766	6	84114647	84119990	-
12674	1438157_s_at	Region	Nfkbia	18035	12	52210017	52212762	-
12675	1416790_a_at	Region	Tdg /// LOC434200 /// LOC545124	21665	10	82517850	82538768	+
12676	1422645_at	Region	Hfe	15216	13	23183718	23190688	-
12677	1453010_at	Region	1700069O15Rik	73473	18	32307908	32337452	+
12678	1434318_a_at	Region	Tcfe3	209446	X	6001657	6014139	+
12679	1460182_at	Region	Snx4	69150	16	32059538	32107644	+
12680	1434011_a_at	Region	1110055N21Rik	109077	19	8089552	8094435	+
12681	1424836_a_at	Region	Clasp2	76499	9	113865413	113971281	+
12682	1416811_s_at	Region	Ctla2a /// Ctla2b	13024	13	59552384	59592485	-

12683	1433805_at	Region	Jak1	16451	4	100111937	100224138	-
12684	1417014_at	Region	Hspb8	80888	5	115530010	115544379	-
12685	1418726_a_at	Region	Tnnt2	21956	1	135683299	135699154	+
12686	1437491_at	Region	Bicd2	76895	13	48941109	48984662	+
12687	1422955_at	Region	Syt17	110058	7	112257713	112319409	-
12688	1426525_at	Region	Arid2	77044	15	96436802	96469595	+
12689	1439441_x_at	Region	Lats2	50523	14	52225857	52269062	-
12690	1448276_at	Region	Tspan4	64540	7	135877231	135896200	+
12691	1422601_at	Region	Serpinb9	20723	13	32484235	32496502	+
12692	1447923_at	Region	1810026B05Rik	69170	7	67437423	67511772	-
12693	1440005_at	Region	C730009D12	328974	18	64627679	64628366	+
12694	1423784_at	Region	Gars	353172	6	55182345	55223825	+
12695	1434271_at	Region	Gba2	230101	4	43482859	43494471	-
12696	1443739_at	Region	Lrba	80877	3	85969078	86527074	+
12697	1435892_at	Region	C230079D11Rik	211961	18	22565695	22746074	+
12698	1452965_at	Region	Ankrd13d	68423	19	4058970	4071838	-
12699	1442994_at	Region	None	None	17	11856039	11856765	+
12700	1417803_at	Region	1110032A04Rik	66183	3	69493969	69534304	-
12701	1435727_s_at	Region	D15Ertd366e	65970	15	99835724	99877288	-
12702	1451835_at	Region	Sox21	223227	14	112788599	112791264	-
12703	1434432_at	Region	1700051E09Rik	67338	11	82543481	82610403	-
12704	1449588_at	Region	Abca4	11304	3	120823356	120958398	+
12705	1429189_at	Region	1110007C02Rik	71784	13	90125585	90127079	+
12706	1450459_at	Region	2010106G01Rik	66552	2	126407985	126447088	-
12707	1424059_at	Region	Suv420h2	232811	7	3987805	3993512	+
12708	1416327_at	Region	Ufc1	66155	1	171217403	171223821	-
12709	1436117_at	Region	A830010M20Rik	231570	5	106566410	106581669	+
12710	1437253_at	Region	A630054L15Rik	211922	14	24675766	24724614	+
12711	1448785_at	Region	Cbfa2t1h	12395	4	13711747	13818082	+
12712	1415972_at	Region	Marcks	17118	10	37207003	37211096	-
12713	1423812_s_at	Region	AW146242	232023	6	57914676	57987537	-
12714	1417092_at	Region	Pthr1	19228	9	110763551	110783597	-
12715	1430769_s_at	Region	2900009I07Rik	68034	19	23715807	23717504	-
12716	1433953_at	Region	Zfp277	246196	12	36913519	37044268	-
12717	1416928_at	Region	Rbm12	75710	2	155551819	155568864	-
12718	1449575_a_at	Region	Gstp1	14870	19	3824201	3826702	-
12719	1438683_at	Region	Wasf2	242687	4	132091532	132159229	+
12720	1434954_at	Region	None	None	12	75695830	75697278	+
12721	1442198_at	Region	Nek7	59125	1	138334093	138469075	-
12722	1431109_at	Region	5430406M13Rik	71373	18	51332684	51520075	+
12723	1436512_at	Region	Arl7	320982	1	88517718	88521248	-
12724	1440234_at	Region	1810012P15Rik	66274	11	78552254	78563925	+
12725	1452054_at	Region	6130401J04Rik	66799	1	16725948	16804454	-
12726	1421898_a_at	Region	Mr1	15064	1	155018615	155037520	-
12727	1427955_a_at	Region	Deb1	26901	9	121724597	121727129	+
12728	1425422_a_at	Region	Parn	74108	16	12274917	12404322	-
12729	1450949_at	Region	Katna1	23924	10	7591699	7628626	+
12730	1420025_s_at	Region	LOC545863	545863	6	87429962	87436507	+
12731	1418371_at	Region	6720463E02Rik	68097	11	87707525	87715497	-
12732	1437494_at	Region	Mapkapk3	102626	9	107320708	107355658	-
12733	1420129_s_at	Region	D10Wsu52e	28088	10	85899581	85918735	-
12734	1424303_at	Region	AV216087	211896	2	104426538	104447555	-
12735	1421846_at	Region	Wsb2	59043	5	116486085	116507368	+
12736	1426939_at	Region	2310007F12Rik	69499	X	144621704	144630913	-
12737	1455030_at	Region	None	None	2	89887873	89889787	+
12738	1417533_a_at	Region	Itgb5	16419	16	32637841	32757227	+
12739	1418972_at	Region	Bcl10	12042	3	144898767	144908672	+
12740	1444444_at	Region	None	None	12	49908727	49909065	+
12741	1424393_s_at	Region	Adhfe1	76187	1	9652849	9682760	+
12742	1439607_at	Region	BC061928	241919	3	30617648	30721720	-
12743	1434450_s_at	Region	Adrbk2	320129	5	111987619	112085665	-
12744	1426693_x_at	Region	Cox15	226139	19	43279531	43297387	-
12745	1452398_at	Region	Plce1	74055	19	37868000	38127104	+
12746	1420995_at	Region	Plxna3	18846	X	68989754	69004418	+
12747	1428850_x_at	Region	2410026K10Rik	66478	NONE	NONE	NONE	NONE

12748	1433908_a_at	Region	Cttn	13043	7	138848729	138882584	-
12749	1439102_at	Region	Falz	207165	11	106856593	106978743	-
12750	1418021_at	Region	C4	12268	17	32432437	32448043	-
12751	1417387_at	Region	Med31	67279	11	71937382	71941250	-
12752	1419469_at	Region	Gnb4	14696	3	31991704	32024713	-
12753	1456162_x_at	Region	Add3	27360	19	52767533	52814957	+
12754	1460092_at	Region	Lsamp	268890	16	40710888	41011281	+
12755	1429299_at	Region	Ddah1	69219	3	144733606	144866062	+
12756	1419156_at	Region	Sox4	20677	13	28430338	28433304	-
12757	1444345_at	Region	None	None	4	141318858	141319798	-
12758	1434013_at	Region	Ablim3	319713	18	62028204	62140635	-
12759	1445029_at	Region	A330019N05Rik	215890	10	33567437	33679703	-
12760	1423730_at	Region	C130052I12Rik	218335	13	69663073	69679813	+
12761	1436045_at	Region	AI843383	98442	1	38126501	38127844	-
12762	1436511_at	Region	BC031781	208768	1	180804971	180821933	+
12763	1427357_at	Region	Cda	72269	4	137219789	137249216	-
12764	1417199_at	Region	1300007B12Rik	57439	1	134195640	134212218	-
12765	1433999_at	Region	Slk	20874	19	47131319	47193755	+
12766	1455040_s_at	Region	1110062M06Rik	68850	X	96692991	96693416	+
12767	1436175_at	Region	A430107N12Rik	78432	14	12568309	12569448	+
12768	1429176_at	Region	Lrsam1	227738	2	32857382	32893413	-
12769	1426671_a_at	Region	Rnpc2	170791	2	155604575	155637077	-
12770	1420411_a_at	Region	Pi4k2b	67073	5	51551747	51579476	+
12771	1460604_at	Region	Cybrd1	73649	2	70805450	70839291	+
12772	1456873_at	Region	Clic5	224796	17	41693345	41782570	+
12773	1415814_at	Region	Atp6v1b2	11966	8	68237311	68262233	+
12774	1438764_at	Region	Anxa7	11750	14	18816589	18841128	-
12775	1427420_at	Region	Nkx6-2	14912	7	133975237	133978657	-
12776	1428944_at	Region	5730469D23Rik	231380	5	85372730	85434753	-
12777	1434478_at	Region	Heca	380629	10	17823383	17874863	-
12778	1437364_at	Region	Coq3	230027	4	21949046	21981497	+
12779	1416468_at	Region	Aldh1a1	11668	19	19842337	19883555	+
12780	1441974_at	Region	None	None	18	33415337	33416619	+
12781	1433543_at	Region	Anln	68743	9	22222408	22279584	-
12782	1420497_a_at	Region	Cebpz	12607	17	76742080	76759276	-
12783	1451006_at	Region	Xdh	22436	17	71655241	71734545	-
12784	1456394_at	Region	1500019C06Rik	72772	5	22251612	22284194	+
12785	1450988_at	Region	Lgr5	14160	10	115138993	115274843	-
12786	1429253_at	Region	Zfp262	67785	4	125890741	125995291	-
12787	1454610_at	Region	38602	235072	9	25147069	25202879	+
12788	1460228_at	Region	Usf2	22282	7	26353591	26364359	-
12789	1421384_at	Region	Lyst	17101	13	13033273	13222595	+
12790	1428207_at	Region	Bcl7a	77045	5	122516563	122546119	+
12791	1451542_at	Region	Ssbp2	66970	13	87661341	87840293	+
12792	1425020_at	Region	Ubxd4 /// LOC432749	217379	12	4054763	4083224	-
12793	1455815_a_at	Region	Ywhab	54401	2	163452166	163475556	+
12794	1457124_at	Region	None	None	7	116945052	116945343	+
12795	1439610_at	Region	None	None	18	70209259	70209884	-
12796	1431528_at	Region	5830427D02Rik	76042	NONE	NONE	NONE	NONE
12797	1437469_at	Region	A030007D23Rik	319530	11	121332071	121340395	-
12798	1455064_at	Region	Rab36	76877	10	75138289	75155300	+
12799	1441054_at	Region	Apol2	239552	15	77799756	77807230	-
12800	1426677_at	Region	Flna	192176	X	68884445	68910522	-
12801	1440822_x_at	Region	Reps1	19707	10	18013924	18049606	+
12802	1425591_a_at	Region	Chmp2a	68953	7	11025700	11028425	-
12803	1427978_at	Region	4732418C07Rik	230648	4	114696978	114736232	+
12804	1434694_at	Region	Lrrc8	241296	2	30169884	30214310	+
12805	1430034_at	Region	Cct4	12464	11	22885383	22898126	+
12806	1448038_at	Region	None	None	15	89123488	89126156	-
12807	1450396_at	Region	Stag2	20843	X	36722649	36796970	+
12808	1426822_at	Region	Rhot2	214952	17	23644999	23651012	-
12809	1441258_at	Region	AF529169	209743	9	89477917	89510874	-
12810	1454046_x_at	Region	4933424M23Rik /// LOC260345	260345	11	117821888	117845019	+

12811	1416179_a_at	Region	Rdx	19684	9	52120601	52161012	+
12812	1427898_at	Region	Rnf6	74132	5	145096046	145108279	-
12813	1434970_a_at	Region	None	None	1	4736769	4738037	-
12814	1424490_at	Region	2410005H09Rik	232969	7	19682802	19691283	+
12815	1455861_at	Region	Epb4.1l5	226352	1	119299565	119400370	-
12816	1423432_at	Region	Phip	83946	9	83198955	83305205	-
12817	1437257_at	Region	Wdr47	99512	3	108387159	108441461	+
12818	1457040_at	Region	Lgi2	246316	5	51348717	51377137	-
12819	1454787_at	Region	Zdhhc9	208884	X	42692767	42729479	-
12820	1424431_at	Region	MGI:1926002	78752	6	118540028	118571686	-
12821	1435986_x_at	Region	Sdhc	66052	1	171058005	171079442	-
12822	1426434_at	Region	Tmem43	74122	6	91922874	91937581	+
12823	1418768_at	Region	Opa1	74143	16	28375142	28448290	+
12824	1439927_at	Region	None	None	10	79917657	79918235	+
12825	1448117_at	Region	Kitl	17311	10	99997658	100082002	+
12826	1417562_at	Region	Eif4ebp1	13685	8	25994476	26010747	+
12827	1424080_at	Region	Dcps	69305	9	35038458	35090031	-
12828	1431177_a_at	Region	Rpl10a	19896	17	26125374	26127806	+
12829	1453168_at	Region	1700029J07Rik	69479	8	44894553	44916210	-
12830	1445900_at	Region	None	None	16	32899003	32899863	-
12831	1416982_at	Region	Foxo1	56458	3	51900114	51981292	+
12832	1434235_at	Region	Slc20a2	20516	8	21228953	21324191	+
12833	1448341_a_at	Region	Stxbp2	20911	8	36313	47968	+
12834	1439327_at	Region	Ccbe1	320924	18	66290942	66524129	-
12835	1457729_at	Region	None	None	5	145471612	145472514	-
12836	1438156_x_at	Region	Cpt1a	12894	19	3137883	3174426	+
12837	1439015_at	Region	Gfra1	14585	19	57822423	58054639	-
12838	1443233_at	Region	Gtf2f2	68705	14	70239863	70353356	-
12839	1426043_a_at	Region	Capn3	12335	2	119977722	120018348	+
12840	1429240_at	Region	Stard4	170459	18	33424453	33436848	-
12841	1428805_at	Region	Slc35e3	215436	10	117424670	117437350	-
12842	1433473_x_at	Region	Tm2d2	69742	8	23740385	23746364	+
12843	1437107_at	Region	Rab6b	270192	9	103083792	103160522	+
12844	1436897_at	Region	Mfhas1	52065	8	34390870	34482521	+
12845	1450623_at	Region	Gnb2	14693	5	136478995	136484097	-
12846	1422607_at	Region	Etv1	14009	12	35383685	35470632	+
12847	1436401_at	Region	9330128J19Rik	271144	13	92607460	92662524	-
12848	1451517_at	Region	Rhobtb2	246710	14	64100920	64121398	-
12849	1416437_a_at	Region	Mapk8ip3	30957	17	22703470	22742877	-
12850	1449855_s_at	Region	Uchl3 /// Uchl4	50933	14	96180426	96270208	+
12851	1434472_at	Region	Dusp3	72349	11	101795246	101805857	-
12852	1419008_at	Region	Npy5r	18168	8	65789090	65797217	-
12853	1418231_at	Region	Lims1	110829	10	58281059	58380568	+
12854	1449987_at	Region	Alk	11682	17	69645483	70378834	-
12855	1420441_at	Region	Cenpc1	12617	5	85274034	85327481	-
12856	1439027_at	Region	C330023M02Rik	231713	5	120549789	120591920	+
12857	1438993_a_at	Region	Atp6v1d	73834	12	75699581	75718058	-
12858	1420807_a_at	Region	Egfl9	106565	17	43807132	43812952	+
12859	1425911_a_at	Region	Fgfr1	14182	8	24257087	24299020	+
12860	1448779_at	Region	Ciz1	68379	2	32295396	32310459	+
12861	1417175_at	Region	Csnk1e	27373	15	79469142	79493293	-
12862	1440202_at	Region	None	None	NONE	NONE	NONE	NONE
12863	1435589_at	Region	AI842788	240514	19	5245329	5246339	-
12864	1436489_x_at	Region	C85492	215494	9	122004262	122016175	-
12865	1422470_at	Region	Bnip3	12176	7	133277078	133295748	-
12866	1452367_at	Region	Coro2a	107684	4	46453481	46482362	-
12867	1417080_a_at	Region	MGI:1349469	26940	9	21963660	21976223	-
12868	1460401_at	Region	2310050N11Rik	66967	1	151613030	151679348	+
12869	1425968_s_at	Region	Apeg1	11790	1	75666020	75723037	+
12870	1447936_at	Region	2410006H16Rik	69221	11	62328546	62330460	+
12871	1436791_at	Region	Wnt5a	22418	14	26642805	26662537	+
12872	1422223_at	Region	Grin2b	14812	6	136493368	136805571	-
12873	1455869_at	Region	Camk2b	12323	11	5864459	5960535	-
12874	1451429_at	Region	5730466C23Rik	70575	8	105011889	105054467	-
12875	1444765_at	Region	Rbpms	19663	8	32589665	32735420	-

12876	1415980_at	Region	Atp5g2	67942	15	102723287	102731466	-
12877	1447585_s_at	Region	Mrps6	121022	16	91215580	91269471	+
12878	1426802_at	Region	38603	20362	11	53272641	53296937	+
12879	1443468_at	Region	4631427C17Rik	74340	6	29815630	29959406	+
12880	1423260_at	Region	Id4	15904	13	47859485	47862094	+
12881	1431896_at	Region	4930447C04Rik	75801	12	69728240	69764946	-
12882	1431126_a_at	Region	0610011F06Rik	68347	17	23682250	23683893	+
12883	1426609_at	Region	2810028N01Rik	72662	14	93601375	93623845	-
12884	1422677_at	Region	Dgat2	67800	7	93245028	93274078	-
12885	1457674_at	Region	Vps13a	271564	19	15839467	15841015	-
12886	1460652_at	Region	Esrra	26379	19	6624825	6635618	-
12887	1429005_at	Region	Mfhas1	52065	8	34390870	34482521	+
12888	1452209_at	Region	Pkp4	227937	2	59016420	59210603	+
12889	1450839_at	Region	D0H4S114	27528	18	33660051	33686464	-
12890	1435407_at	Region	None	None	NONE	NONE	NONE	NONE
12891	1421746_a_at	Region	Fbxo17	50760	7	24122746	24144393	+
12892	1449836_x_at	Region	Biklk	12124	15	83575461	83593234	+
12893	1418234_s_at	Region	Bcas2	68183	3	102599261	102606342	+
12894	1426865_a_at	Region	Ncam1	17967	9	49555319	49607941	-
12895	1450675_at	Region	Smap1l	69780	4	119990768	120039691	-
12896	1427610_at	Region	Dsp	109620	13	37690386	37736832	+
12897	1420911_a_at	Region	Mfge8	17304	7	72925876	72941120	-
12898	1436950_at	Region	Crkl	12929	16	16222955	16257223	+
12899	1424261_at	Region	Zfp672	319475	11	58040845	58048742	-
12900	1451973_at	Region	Ube1dc1	66663	9	104024992	104040598	-
12901	1434882_at	Region	Mtdh	67154	15	34082454	34141052	+
12902	1417009_at	Region	C1r	50909	6	125225539	125236397	+
12903	1428443_a_at	Region	Rap1ga1	110351	4	136546029	136611121	+
12904	1428834_at	Region	Dusp4	319520	8	33606678	33618962	+
12905	1448155_at	Region	Pdcd6ip	18571	9	113708127	113760882	-
12906	1439724_at	Region	BC051080	237422	10	84812276	84911107	+
12907	1428153_at	Region	Mrps10	64657	17	44887657	44897139	+
12908	1427080_at	Region	2610036D13Rik	75425	2	157438772	157466327	-
12909	1441254_at	Region	1810008K04Rik	69067	1	62934717	62935303	+
12910	1425118_at	Region	Spire2	234857	8	122707614	122744415	+
12911	1440287_at	Region	1110014D18Rik	68501	15	59384784	59612226	+
12912	1435852_at	Region	Spred3	101809	7	24570238	24580023	-
12913	1457470_at	Region	D3Ertd452e	51898	3	26922446	26923130	-
12914	1416828_at	Region	LOC545465	545465	2	136227307	136296207	+
12915	1455204_at	Region	Pitpnc1	71795	11	107033195	107291788	-
12916	1428931_a_at	Region	Parp6	67287	9	59734045	59767165	+
12917	1420634_a_at	Region	Smad2	17126	18	76478323	76539930	+
12918	1448463_at	Region	4933434E20Rik	99650	3	89863255	89872865	+
12919	1452946_a_at	Region	3222401M22Rik	74013	1	55471318	55527941	-
12920	1417907_at	Region	Ube2l3	22195	16	15923031	15972515	-
12921	1457256_x_at	Region	Ptch2	19207	4	116055261	116073736	+
12922	1433494_at	Region	Dos	216164	10	80253788	80255210	-
12923	1435085_at	Region	None	None	6	135615772	135616400	+
12924	1427072_at	Region	Stard8	236920	X	93644026	93676089	+
12925	1424114_s_at	Region	Lamb1-1	16777	12	27840552	27905646	+
12926	1416244_a_at	Region	Cnbp1	12785	6	88279741	88287657	-
12927	1428263_a_at	Region	Tceb2	67673	17	21626920	21631289	-
12928	1452179_at	Region	Phf17	269424	3	41010564	41046427	+
12929	1426319_at	Region	Pdgfd	71785	9	6119514	6328427	+
12930	1416872_at	Region	Tspan6	56496	X	127436007	127443302	-
12931	1457672_at	Region	Chd9	109151	8	90338072	90341034	+
12932	1426977_at	Region	Usp47	74996	7	105876643	105964517	+
12933	1421386_at	Region	Ankrd6	140577	4	33082828	33202298	-
12934	1437793_at	Region	5430432P15Rik	74493	19	36161291	36235550	+
12935	1460449_at	Region	C030032C09Rik	77531	10	90548071	90943317	+
12936	1439153_at	Region	Ibrdc2	218215	13	46720793	46844544	+
12937	1454809_at	Region	Ncoa7	211329	10	30689068	30845679	-
12938	1453253_a_at	Region	Rpusd1	106707	17	23533751	23537456	+
12939	1444357_at	Region	None	None	12	49826497	49894078	+
12940	1424331_at	Region	Rab40c	224624	17	23689151	23711170	-

12941	1428657_at	Region	B930013M22Rik	68750	13	37486050	37490651	+
12942	1417480_at	Region	Fbxo9	71538	9	78250521	78277651	-
12943	1448179_at	Region	Usmg5	66477	19	46634768	46641933	-
12944	1453688_at	Region	Cwf19l2	244672	9	3307769	3382920	+
12945	1419845_at	Region	Dlx1	13390	2	71227188	71231723	+
12946	1415869_a_at	Region	Trim28	21849	7	11017842	11024710	+
12947	1417465_at	Region	Fnta	14272	8	24723471	24740350	-
12948	1435184_at	Region	B430320C24Rik	319900	15	11651405	11653133	-
12949	1423451_at	Region	Pgrmc1	53328	X	31218779	31226633	+
12950	1429256_at	Region	Gtl2	17263	12	105023823	105039917	+
12951	1416424_at	Region	M6prbp1	66905	17	53916003	53927552	-
12952	1437457_a_at	Region	Mtpn	14489	6	35603109	35634015	-
12953	1433612_at	Region	Ap2s1	232910	7	13688769	13699919	+
12954	1458365_at	Region	Scyl1bp1	98376	1	163300126	163318858	-
12955	1436096_at	Region	None	None	6	31592775	31593795	+
12956	1427343_at	Region	Rasd2	75141	8	74417802	74428505	+
12957	1433989_at	Region	Gabt4	243616	6	114701558	114820031	+
12958	1436078_at	Region	Fcho1	74015	8	70858707	70875819	-
12959	1453212_at	Region	Zfp383	73729	7	25322940	25331524	+
12960	1426245_s_at	Region	Mapre2	212307	18	24032480	24122349	+
12961	1433787_at	Region	B230343H07Rik	338352	7	45020925	45021769	+
12962	1422990_at	Region	Met	17295	6	17474685	17555267	+
12963	1442075_at	Region	AI314604	102027	8	46042804	46043565	+
12964	1449304_at	Region	2310061J03Rik	66391	16	54869202	54870477	-
12965	1431069_at	Region	4930544G21Rik	77629	1	83594365	83738121	-
12966	1438606_a_at	Region	Clic4	29876	4	134095226	134154017	-
12967	1435167_at	Region	Ranbp6	240614	19	29060997	29065552	-
12968	1458371_at	Region	1110051M20Rik	228356	2	90982577	91149391	-
12969	1431302_a_at	Region	Nudt7	67528	8	113433067	113451764	+
12970	1429134_at	Region	Hivep3	16656	4	119003584	119157855	+
12971	1430622_at	Region	4833423F13Rik	73756	16	22077372	22078784	+
12972	1429979_a_at	Region	1810073N04Rik	72055	11	119925064	119972367	-
12973	1457212_at	Region	E530011G23Rik	319856	12	85714559	85715194	+
12974	1435126_at	Region	Dusp15	252864	2	152401869	152408341	-
12975	1450063_at	Region	Fmn2	54418	1	174435022	174755223	+
12976	1417285_a_at	Region	Ndufa5	68202	6	24568151	24577172	-
12977	1421828_at	Region	Kpna3	16648	14	55898702	55974523	-
12978	1439790_at	Region	None	None	13	32496551	32497249	+
12979	1425769_x_at	Region	Cklf	75458	8	103545239	103557779	+
12980	1423297_at	Region	Add3	27360	19	52767533	52814957	+
12981	1460545_at	Region	Thrap3	230753	4	125199956	125213701	-
12982	1434754_at	Region	Garnl4	380711	11	74109019	74113127	-
12983	1444001_at	Region	Strbp	20744	2	37415390	37579381	-
12984	1451336_at	Region	Lgals4	16855	7	24240082	24247836	+
12985	1424253_at	Region	1810073G14Rik	67726	11	57208629	57244244	-
12986	1416251_at	Region	Mcm6	17219	1	128174585	128202650	-
12987	1427235_at	Region	Utx	22289	X	16401607	16518298	+
12988	1433137_at	Region	5031415H12Rik	75984	17	68523143	68524423	+
12989	1419580_at	Region	None	None	11	69744499	69771997	+
12990	1434819_at	Region	St6gal2	240119	17	53083562	53137071	+
12991	1452813_a_at	Region	5033428A16Rik	382030	8	87404151	87419831	+
12992	1438324_at	Region	9330182L06Rik	231014	5	9273627	9491141	+
12993	1416920_at	Region	Rbm4	19653	19	4573943	4582649	-
12994	1427918_a_at	Region	Rhoq	104215	17	84819915	84856873	+
12995	1440322_at	Region	Cugbp2	14007	2	6459141	6638885	-
12996	1448611_at	Region	D8Ertd594e	52357	8	46797192	46960652	-
12997	1457704_at	Region	Zfp533	241494	2	77108849	77514565	-
12998	1419538_at	Region	Flt3	14255	5	146222241	146291790	-
12999	1449152_at	Region	Cdkn2b	12579	NONE	NONE	NONE	NONE
13000	1435917_at	Region	---	433904	5	72107568	72123547	-
13001	1450977_s_at	Region	Ndrg1 /// Ndrl	17988	15	66953527	66993844	-
13002	1455816_a_at	Region	Kctd3	226823	1	188502528	188539374	-
13003	1435982_at	Region	Stx12	100226	4	131814963	131845357	-
13004	1448492_a_at	Region	Psmd12	66997	11	107300617	107319125	+
13005	1456086_x_at	Region	Pqbp1	54633	X	6133463	6138168	-

13006	1455265_a_at	Region	Rgs16	19734	1	153624984	153630102	+
13007	1440946_at	Region	None	None	4	58904182	58904796	+
13008	1425631_at	Region	Ppp1r3c	53412	19	36074403	36079323	-
13009	1443036_at	Region	C630007C17Rik	241514	2	81932610	81957628	+
13010	1426690_a_at	Region	Srebf1	20787	11	59924731	59946246	-
13011	1435874_at	Region	Prkab2	108097	3	97153614	97159355	+
13012	1434566_a_at	Region	4732496O08Rik	242736	4	142327675	142336336	+
13013	1435015_at	Region	2210018M03Rik	67109	7	5324839	5325605	-
13014	1424170_at	Region	Phf5a	68479	15	81916034	81922620	-
13015	1419548_at	Region	Kpna1	16646	16	34764772	34817569	+
13016	1428970_at	Region	Mak3	72117	16	43023139	43046694	+
13017	1417514_at	Region	Ssx2ip	99167	3	145381238	145416690	+
13018	1435677_at	Region	C85492	215494	9	122004262	122016175	-
13019	1445531_at	Region	Csmd1	94109	8	15273983	16089202	-
13020	1426213_at	Region	Imp4	27993	1	34709403	34715250	+
13021	1443088_at	Region	9930031P18Rik	319589	13	104089563	104090247	-
13022	1436917_s_at	Region	Gpsm1	67839	2	26247702	26280397	+
13023	1433508_at	Region	Copeb	23849	13	5749069	5755318	+
13024	1454878_at	Region	2310047C04Rik	224170	16	47767664	47835780	-
13025	1424859_at	Region	Homer3	26558	8	69436115	69447552	+
13026	1422554_at	Region	Ndnl2	66647	7	58755688	58757078	-
13027	1452814_at	Region	Cpne3	70568	4	19448948	19497068	-
13028	1424455_at	Region	Gprasp1	67298	X	129287631	129348407	+
13029	1452222_at	Region	Utrn	22288	10	12263109	12646998	-
13030	1416742_at	Region	Cfdp1	23837	8	111066062	111151899	-
13031	1429653_at	Region	Gse1	382034	8	119846048	119938723	+
13032	1460682_s_at	Region	Ceacam2	26367	7	20680401	20704005	-
13033	1426680_at	Region	Sepn1	74777	4	133498798	133503259	-
13034	1419289_a_at	Region	Syngr1	20972	15	80144003	80172200	+
13035	1429887_at	Region	2310005C01Rik	76730	5	117086148	117087486	+
13036	1419593_at	Region	MGI:2149712	268527	12	16037980	16123596	-
13037	1425094_a_at	Region	Lhx6	16874	2	36015701	36037433	-
13038	1437735_at	Region	Ppp1r12a	17931	10	108049328	108167253	+
13039	1415837_at	Region	Klk6	16612	7	38299769	38303949	+
13040	1434551_at	Region	1110031M08Rik	68693	19	8016414	8030688	+
13041	1449130_at	Region	Cd1d1	12479	3	86740227	86743729	-
13042	1417306_at	Region	Tyk2	54721	9	20987596	21014720	-
13043	1449318_at	Region	Tubg2	103768	11	100977008	100982874	+
13044	1416633_a_at	Region	5730536A07Rik	68250	9	66249938	66262282	+
13045	1452244_at	Region	6330406I15Rik	70717	5	148350053	148369950	+
13046	1428353_at	Region	Foxk2	68837	11	121080635	121128991	+
13047	1428968_at	Region	3110002L15Rik	74360	9	13644844	13664101	-
13048	1422255_at	Region	Kcna4	16492	2	106914441	106922291	+
13049	1426249_at	Region	Adrbk1	110355	19	4074791	4094745	-
13050	1420669_at	Region	Arnt2	11864	7	78100192	78263873	-
13051	1434221_at	Region	BC030863	194404	6	121363681	121405625	-
13052	1428245_at	Region	G6pc3	68401	11	102010786	102015168	+
13053	1456641_at	Region	1190007F08Rik	68859	4	152512950	152518501	-
13054	1424039_at	Region	1810045K07Rik	67887	8	32955623	32971540	+
13055	1437160_at	Region	Nlgn1	192167	3	24847948	25748398	-
13056	1417757_at	Region	Unc13b	22249	4	42974891	43180793	+
13057	1436004_at	Region	Usp27x	54651	X	5610216	5614766	-
13058	1435244_at	Region	Vav2	22325	2	27195797	27358989	-
13059	1436885_a_at	Region	Cherp	27967	8	71610688	71625431	-
13060	1429355_at	Region	3300001K11Rik	70426	16	10033010	10067443	-
13061	1424723_s_at	Region	Cstf3	228410	2	104295277	104370179	+
13062	1423682_a_at	Region	Cdca4	71963	12	108296070	108305225	-
13063	1436753_at	Region	Adck5	268822	15	76627201	76646864	+
13064	1454807_a_at	Region	Snx12	55988	X	95813629	95823851	-
13065	1431802_a_at	Region	D5Wsu178e	28042	5	28691083	28726735	+
13066	1419218_at	Region	Ltap	93840	1	171933290	171956187	-
13067	1416158_at	Region	Nr2f2	11819	7	64237865	64245019	-
13068	1437431_at	Region	Cutl1	13047	5	135261627	135499343	-
13069	1434983_at	Region	LOC545328	545328	1	75206289	75226218	-
13070	1458038_at	Region	1700034P14Rik	73274	13	107815217	107879367	-

13071	1429909_at	Region	4833411O04Rik	74608	2	101418966	101420735	-
13072	1460241_a_at	Region	St3gal5	20454	6	72429820	72486545	+
13073	1424194_at	Region	Rcsd1	226594	1	165570866	165629791	-
13074	1455495_at	Region	AA536808	98214	1	156553004	156555188	+
13075	1441224_at	Region	None	None	X	128000851	128001513	+
13076	1436434_at	Region	9230110J10	329958	4	135076532	135077312	+
13077	1444669_at	Region	None	None	7	23872978	23874121	+
13078	1455425_at	Region	BB001228	103503	10	62771179	62772175	-
13079	1423295_at	Region	Tm9sf2	68059	14	116675627	116727419	+
13080	1418865_at	Region	Zfp385	29813	15	103376968	103403165	-
13081	1458144_at	Region	BC031353	235493	9	75115155	75171307	+
13082	1454890_at	Region	Amot	27494	X	138889991	138927092	-
13083	1428840_s_at	Region	Wdr53	68980	16	31056657	31066443	+
13084	1452000_s_at	Region	Sars1	20226	3	108222354	108240911	-
13085	1416068_at	Region	Kars	85305	8	111291147	111308972	-
13086	1456768_a_at	Region	Mmrn2	105450	14	32512110	32539866	+
13087	1417007_a_at	Region	Vps4b	20479	1	106711939	106737457	-
13088	1460305_at	Region	Itga3	16400	11	94865569	94897801	-
13089	1439184_s_at	Region	Txnl5	52700	11	71933212	71936145	+
13090	1423321_at	Region	Myadm	50918	7	4169	12984	-
13091	1449081_at	Region	Ces3	104158	8	92449775	92481484	-
13092	1438215_at	Region	Sfrs3	20383	17	26839644	26846716	+
13093	1452004_at	Region	Calca	12310	7	108492491	108495488	-
13094	1453418_at	Region	Col24a1	71355	3	144282520	144343093	+
13095	1455206_at	Region	C130006E23	331563	X	152964823	152967649	+
13096	1417608_a_at	Region	Rpl13a	22121	7	39202318	39205277	-
13097	1429164_at	Region	Prss36	77613	7	121982640	121985907	-
13098	1427941_at	Region	Dicer1	192119	12	100135572	100196088	-
13099	1451575_a_at	Region	Nudt3	56409	17	25375309	25419370	-
13100	1435846_x_at	Region	None	None	5	128897493	128898373	+
13101	1449248_at	Region	Clcn2	12724	16	19475107	19488386	-
13102	1417133_at	Region	Pmp22	18858	11	62857160	62885196	+
13103	1454962_at	Region	Spire1	68166	18	67718995	67783507	-
13104	1442325_at	Region	C530046L02Rik	224617	17	21982357	22009100	-
13105	1440295_at	Region	1110057K04Rik	68832	12	7453506	7531135	+
13106	1448685_at	Region	2900010M23Rik	67267	17	24918583	24929809	-
13107	1430516_at	Region	4930428B01Rik	73854	NONE	NONE	NONE	NONE
13108	1418372_at	Region	Adsl	11564	15	81001138	81021992	+
13109	1424822_at	Region	9630044O09Rik	105439	14	98195231	98249790	+
13110	1430418_at	Region	Tmem57	66146	4	133763662	133814033	-
13111	1435348_at	Region	D930009K15Rik	399585	X	145812361	145813493	+
13112	1425048_a_at	Region	Hmgb1 /// LOC213079 /// LOC545917	15289	5	147944154	147949960	-
13113	1431644_a_at	Region	Ica1	15893	6	8605506	8727274	-
13114	1423419_at	Region	Lig3	16882	11	82520876	82541867	+
13115	1427551_at	Region	Usp29	57775	7	5934028	6173502	+
13116	1427250_at	Region	Atp2a2	11938	5	121605864	121654245	-
13117	1424408_at	Region	Lims2	225341	18	32171651	32198763	+
13118	1422051_a_at	Region	Gabbr1	54393	17	34805575	34833793	+
13119	1455199_at	Region	AI429214	102069	8	35801976	35803100	+
13120	1441445_at	Region	Per3	18628	4	149497074	149536951	-
13121	1416992_at	Region	Mfng	17305	15	78807168	78824731	-
13122	1448664_a_at	Region	Apeg1	11790	1	75666020	75723037	+
13123	1438397_a_at	Region	Rnpc2	170791	2	155604575	155637077	-
13124	1436759_x_at	Region	Cnn3	71994	3	120205773	120237098	+
13125	1421184_a_at	Region	3300001H21Rik /// MGI:3026615 /// LOC554292	393082	15	100410740	100418501	+
13126	1422754_at	Region	Tmod1	21916	4	45976888	46030718	+
13127	1424380_at	Region	BC026744	330192	5	123175966	123203585	-
13128	1436319_at	Region	Sulf1	240725	1	12847254	12989080	+
13129	1438420_at	Region	Rnpc2	170791	2	155604575	155637077	-
13130	1420959_at	Region	Asph	65973	4	9376231	9595958	-
13131	1437728_at	Region	AW050020	268420	11	60263620	60281539	+

13132	1433850_at	Region	Ppp4r2	232314	6	69893516	69896400	+
13133	1439645_at	Region	Adra2b	11552	2	126877139	126881074	+
13134	1421096_at	Region	Trpc1	22063	9	95610171	95653902	-
13135	1436181_at	Region	Ddef2	211914	12	18182	20637	-
13136	1425173_s_at	Region	Golph3l	229593	3	95076790	95107073	+
13137	1421327_at	Region	Mtap2 /// A730034C02	17756	1	66613900	66731968	+
13138	1428219_at	Region	Rybp	56353	6	100696164	100754244	-
13139	1416479_a_at	Region	Tmem14c	66154	13	40576882	40583168	+
13140	1423594_a_at	Region	Ednrb	13618	14	98359614	98388688	-
13141	1416642_a_at	Region	Tpt1p	497210	6	70187826	70190793	+
13142	1434010_at	Region	Als2cr13	72750	1	60217249	60288662	+
13143	1436223_at	Region	None	None	12	114969500	114970422	-
13144	1424239_at	Region	2310066E14Rik	75687	8	104889579	104917967	+
13145	1449415_at	Region	Chd1l	68058	3	97047022	97096464	-
13146	1436577_at	Region	Arhgef9	236915	X	89650305	89797863	-
13147	1442307_at	Region	None	None	10	122795576	122796142	-
13148	1447248_at	Region	Slc39a12	277468	2	14314676	14421249	+
13149	1455045_at	Region	Srr	27364	11	74632454	74651361	-
13150	1416685_s_at	Region	Fbl /// LOC545184	14113	7	23565500	23575021	+
13151	1452846_at	Region	Ppfia4	68507	1	134149042	134182786	-
13152	1433921_s_at	Region	Zcsl2	105638	14	30212767	30217780	-
13153	1453024_at	Region	Wdr37	207615	13	8762169	8828183	-
13154	1427392_at	Region	Dscaml1	114873	9	45443056	45766529	+
13155	1433951_at	Region	Arl5	75423	2	52330113	52357036	-
13156	1419435_at	Region	Aox1	11761	1	58334809	58411218	+
13157	1443249_at	Region	A530081L18Rik	320253	18	56982085	57143467	-
13158	1434112_at	Region	Lphn2	99633	3	147794533	147874904	-
13159	1455549_at	Region	Sestd1	228071	2	76884304	76942838	-
13160	1426957_at	Region	Trp53bp1	27223	2	120712100	120784969	-
13161	1426700_a_at	Region	Usp52	103135	10	128040154	128058166	+
13162	1436139_at	Region	None	None	12	63245840	63246495	-
13163	1439046_at	Region	AI851076	237859	11	76769951	76804094	-
13164	1424905_a_at	Region	Slc39a11	69806	11	113065947	113386861	-
13165	1418002_at	Region	2010110M21Rik	67044	13	53200592	53201508	+
13166	1455602_x_at	Region	C430010P07Rik	227227	1	67011628	67093590	-
13167	1454084_a_at	Region	Senp8	71599	9	59852773	59867496	-
13168	1452746_at	Region	Atp13a2	74772	4	139868189	139888591	+
13169	1449590_a_at	Region	Mras	17532	9	99288432	99337984	-
13170	1433851_at	Region	None	None	6	101310462	101345533	+
13171	1433594_at	Region	Commd2	52245	3	57278796	57285107	-
13172	1429320_at	Region	4921511I16Rik	70925	8	46677113	46681564	-
13173	1424480_s_at	Region	Akt2	11652	7	22980718	23014488	+
13174	1438370_x_at	Region	Dos	216164	NONE	NONE	NONE	NONE
13175	1448382_at	Region	Ehhadh	74147	16	20532801	20559307	-
13176	1437044_a_at	Region	Gba	14466	3	88959413	88965154	+
13177	1428635_at	Region	Comtd1	69156	14	20210529	20213578	-
13178	1448247_at	Region	Bcl7b	12054	5	134181984	134195337	+
13179	1448970_at	Region	1200007B05Rik	67453	18	31819928	31849663	-
13180	1417173_at	Region	Crebl1	12915	17	32350416	32358414	+
13181	1423425_at	Region	1300012G16Rik	71772	5	119635089	119655425	-
13182	1424818_at	Region	Alg12	223774	15	88857174	88871213	-
13183	1452867_at	Region	Col4a3bp	68018	13	92734071	92828876	+
13184	1432344_a_at	Region	Aplp2	11804	9	31068390	31130767	-
13185	1438510_a_at	Region	Hars	15115	18	36990295	37006885	-
13186	1451527_at	Region	Pcolce2	76477	9	95547765	95599093	+
13187	1434033_at	Region	Tle1	21885	4	71208335	71292050	-
13188	1418934_at	Region	Mab21l2	23937	3	86289969	86292671	-
13189	1449001_at	Region	Ivd	56357	2	118375849	118395206	+
13190	1427938_at	Region	Mycbp	56309	4	122932163	122939256	+
13191	1441948_x_at	Region	Tex27	21769	17	27815127	28020060	+
13192	1433988_s_at	Region	C230098O21Rik	102153	8	68281185	68281915	-
13193	1430109_at	Region	4833412E19Rik	73905	6	120441783	120443595	-
13194	1447231_at	Region	Slc8a1	20541	17	79218202	79483338	-
13195	1434481_at	Region	4121402D02Rik	74026	11	98616603	98627514	+

13196	1427208_at	Region	Zfp451	98403	1	34030702	34083136	-
13197	1460637_s_at	Region	Pfdn5	56612	15	102386650	102391967	+
13198	1422743_at	Region	Phka1	18679	X	97117039	97245563	-
13199	1419584_at	Region	AI428795	209683	5	110273612	110336823	+
13200	1421043_s_at	Region	Arhgef2	16800	3	88365002	88390778	+
13201	1450154_at	Region	Folh1	53320	7	80588572	80645418	-
13202	1454614_at	Region	1810013D10Rik	66278	5	52073629	52088679	+
13203	1459873_x_at	Region	None	None	NONE	NONE	NONE	NONE
13204	1416044_at	Region	Fliih	14248	11	60439791	60452863	-
13205	1439844_at	Region	8430426J06Rik	78102	15	81293454	81298722	-
13206	1417200_at	Region	1300007B12Rik	57439	1	134195640	134212218	-
13207	1427449_a_at	Region	Adprhl2	100206	4	125343464	125348816	-
13208	1437164_x_at	Region	Atp5o /// LOC432676	28080	16	91082467	91088874	-
13209	1454823_at	Region	Wdr37	207615	13	8762169	8828183	-
13210	1419645_at	Region	Cstf2	108062	X	127604288	127631760	+
13211	1419217_at	Region	MGI:1351630	27414	7	40528521	40725138	-
13212	1429318_a_at	Region	Qk	19317	17	8850890	8959849	-
13213	1424461_at	Region	Dctn2	69654	10	127003036	127018444	+
13214	1438020_at	Region	Hapln1	12950	13	85655519	85726506	+
13215	1448144_at	Region	Hnrpab	15384	11	51352941	51359688	-
13216	1439789_at	Region	None	None	11	44760163	44760931	+
13217	1434999_at	Region	Suv420h1	225888	19	3556211	3605181	+
13218	1420642_a_at	Region	2010100O12Rik	67067	2	155601211	155602730	+
13219	1455759_a_at	Region	Ankhd1	108857	18	36872342	36882677	+
13220	1417822_at	Region	D17H6S56E-5	110956	17	32700504	32704526	-
13221	1437475_at	Region	Xrn1	24127	9	95858413	95956167	+
13222	1449346_s_at	Region	Riok1	71340	13	37576092	37600226	+
13223	1422531_at	Region	Syt5	53420	7	3785937	3792663	-
13224	1452656_at	Region	Zdhhc2	70546	8	39367458	39428485	+
13225	1423785_at	Region	Egln1	112405	8	124195909	124203103	-
13226	1460205_at	Region	6720485C15Rik	68087	11	102815145	102838236	-
13227	1425376_at	Region	Alox15b	11688	11	68909543	68923497	-
13228	1427300_at	Region	Lhx8	16875	3	153307269	153331050	-
13229	1456611_at	Region	D430015B01Rik	58909	6	59096390	59187371	-
13230	1416667_at	Region	Ebp	13595	X	6424271	6432452	-
13231	1454694_a_at	Region	Top2a	21973	11	98814490	98845202	-
13232	1451508_at	Region	1700108L22Rik	214048	3	40377212	40405216	+
13233	1433518_at	Region	Lcmt2	329504	2	120642156	120654227	-
13234	1434996_at	Region	Slc25a16	73132	10	62886863	62912733	+
13235	1424414_at	Region	Ogfrl1	70155	1	23503663	23655192	-
13236	1453429_at	Region	9530057J20Rik	78672	4	627	1537	-
13237	1436341_at	Region	F830020C16Rik	219150	14	59350496	59478125	-
13238	1419302_at	Region	Heyl	56198	4	122260965	122276982	+
13239	1445570_at	Region	2810405F18Rik	72181	4	114991747	115012781	-
13240	1434738_at	Region	Tarsl2	272396	7	59529138	59576315	+
13241	1451379_at	Region	Rab22a	19334	2	173120334	173165145	+
13242	1434868_at	Region	4.93E+26	329735	3	107687213	107691540	-
13243	1426078_a_at	Region	Gpr108	78308	17	54924244	54937394	-
13244	1437284_at	Region	Fzd1	14362	5	4759844	4763976	-
13245	1424435_a_at	Region	Gart	14450	16	90778573	90804117	-
13246	1435473_at	Region	Gm347	241289	2	28380103	28387670	+
13247	1438803_s_at	Region	Snx16	74718	3	10419694	10441634	-
13248	1450770_at	Region	3632451O06Rik	67419	14	44775306	44877074	-
13249	1422871_at	Region	Kcnj12	16515	11	60791446	60796771	+
13250	1420850_at	Region	Crnkl1	66877	2	145374418	145391636	-
13251	1438590_at	Region	Rapgef3	223864	15	97820598	97843317	-
13252	1416791_a_at	Region	Nxf1	53319	19	7953650	7967457	+
13253	1417622_at	Region	Slc12a2	20496	18	58093828	58161955	+
13254	1435323_a_at	Region	Oact1	218121	13	29616112	29726316	+
13255	1416972_at	Region	Nhp2l1	20826	15	82092712	82114989	-
13256	1453163_at	Region	Ppp1r12a	17931	10	108049328	108167253	+
13257	1433786_x_at	Region	Serf2	378702	2	120963266	120964906	+
13258	1458648_at	Region	AU042950	107306	19	27740819	27741278	-
13259	1421530_a_at	Region	Grm8	14823	6	27322607	28173190	-

13260	1460246_at	Region	Mecp2	17257	X	68687485	68746248	-
13261	1436692_at	Region	None	None	4	59704578	59704857	+
13262	1420984_at	Region	Pctp	18559	11	89805684	89823952	-
13263	1417561_at	Region	Apoc1	11812	7	16558729	16561817	-
13264	1451196_at	Region	Ypel5	383295	17	70612621	70627590	+
13265	1426909_at	Region	AI481316	98383	1	167156101	167157958	-
13266	1438089_a_at	Region	Bclaf1	72567	10	20239740	20267469	+
13267	1442971_at	Region	5830435C13Rik	407823	2	59777374	59778133	-
13268	1417349_at	Region	Pldn	18457	2	122252366	122261952	+
13269	1415873_a_at	Region	Actr1a	54130	19	45924156	45943077	-
13270	1456934_at	Region	None	None	4	15834477	15835032	+
13271	1454889_x_at	Region	Tmcc3	319880	10	94549417	94562436	+
13272	1417377_at	Region	None	None	9	47546173	47869344	+
13273	1459108_a_at	Region	Yeats2	208146	16	18922076	19003907	+
13274	1433852_at	Region	C330002I19Rik	77480	12	21526422	21612277	+
13275	1441208_at	Region	None	None	18	77095041	77095577	+
13276	1438500_at	Region	B230206N24Rik	210673	6	114064130	114069723	-
13277	1441465_at	Region	Cnot3	232791	7	95942392	95943786	-
13278	1457285_at	Region	Zfp187	432731	13	20922288	20923020	-
13279	1455189_at	Region	8030451N04Rik	320735	3	102785285	102786163	+
13280	1419154_at	Region	Tmprss2	50528	16	97049264	97095777	-
13281	1449297_at	Region	Casp12	12364	9	5255895	5283427	+
13282	1423685_at	Region	Aars	234734	8	110331687	110354590	+
13283	1418059_at	Region	Eltd1	170757	3	150417575	150524774	+
13284	1439657_at	Region	LOC319980	319980	1	189058764	189059732	+
13285	1423916_s_at	Region	Mlf2	30853	6	125588244	125592912	+
13286	1421991_a_at	Region	Igfbp4	16010	11	98862347	98873733	+
13287	1418656_at	Region	Lsm5	66373	6	56845388	56849008	-
13288	1416100_at	Region	Eif3s7	55944	15	78010958	78022717	-
13289	1434243_s_at	Region	None	None	16	56061063	56061574	+
13290	1423098_at	Region	Capn7	12339	14	29468562	29503108	+
13291	1444494_at	Region	None	None	2	69539010	69539630	+
13292	1448340_at	Region	Tmem30a	69981	9	79974649	79999130	-
13293	1428152_a_at	Region	Rpl18a	76808	8	70051092	70053111	-
13294	1438802_at	Region	Foxp1	108655	6	99395348	99630926	-
13295	1449494_at	Region	Rab3c	67295	13	106309703	106528782	-
13296	1423109_s_at	Region	Slc25a20	57279	9	108710938	108733881	+
13297	1454983_at	Region	AI465301	102534	9	70739539	70742254	-
13298	1435641_at	Region	9530018I07Rik	320137	1	37733814	37737383	-
13299	1450166_at	Region	Ids	15931	X	65014764	65033475	-
13300	1428674_at	Region	Prpf38b	66921	3	108698549	108707446	-
13301	1433531_at	Region	Acsl4	50790	X	135758036	135830547	-
13302	1431385_a_at	Region	Mbtps1	56453	8	118850260	118901165	-
13303	1417219_s_at	Region	Tmsb10	19240	6	20039912	20040386	+
13304	1438349_at	Region	BC043476	381067	17	19605202	19620447	+
13305	1437542_at	Region	A730095J18Rik	319462	1	69828384	69829626	-
13306	1442843_at	Region	4933411D12Rik	74086	8	12498847	12499967	+
13307	1427325_s_at	Region	AI597013	100182	4	62458296	62471256	-
13308	1458768_at	Region	Epb4.1l5	226352	1	119299565	119400370	-
13309	1447337_at	Region	Dapp1	26377	3	136820534	136870453	-
13310	1448425_at	Region	Eif3s10	13669	19	60361493	60391011	-
13311	1437296_at	Region	Pkn2	109333	3	141762433	141850964	-
13312	1448347_a_at	Region	Gpiap1	53872	2	103469773	103502351	-
13313	1418484_at	Region	Tekt2	24084	4	125349234	125352312	-
13314	1427248_at	Region	Whsc2	24116	5	32386442	32424521	-
13315	1424924_at	Region	Sec63	140740	10	42868086	42935647	+
13316	1449732_at	Region	Zipro1	22697	5	137090159	137106967	+
13317	1448315_a_at	Region	Pycr2	69051	1	180858129	180861908	+
13318	1434358_x_at	Region	Rps21	66481	2	179974367	179975394	+
13319	1435644_at	Region	G431001E03Rik	268396	11	32242634	32318819	+
13320	1428717_at	Region	Scrn1	69938	6	54653141	54710707	-
13321	1434448_at	Region	MGI:105968	109658	4	128653196	128667753	-
13322	1428696_at	Region	2310015N21Rik	76438	17	47525770	47722960	-
13323	1418106_at	Region	Hey2	15214	10	30875100	30885524	-
13324	1436556_at	Region	A930027H06Rik	109218	6	42405839	42408433	+

13325	1429202_at	Region	2610019N06Rik	66299	9	7175571	7176729	-
13326	1448109_a_at	Region	Rpl26	19941	11	68627241	68630192	+
13327	1423317_at	Region	3110001D03Rik	66928	4	74338573	74339502	-
13328	1427514_at	Region	None	None	NONE	NONE	NONE	NONE
13329	1424369_at	Region	Psmf1	228769	2	151172998	151198229	-
13330	1434703_at	Region	Extl3	54616	14	59578943	59607355	-
13331	1418016_at	Region	Pum2	80913	12	7990991	8066649	+
13332	1418079_at	Region	Psme3	19192	11	101137338	101144618	+
13333	1433764_at	Region	Clec2l	381758	6	38798928	38816417	+
13334	1422977_at	Region	Gp1bb	14724	16	17391369	17393453	-
13335	1454856_x_at	Region	Rpl35	66489	2	38933743	38937293	-
13336	1428382_at	Region	Smarcc2	68094	10	128198104	128227199	+
13337	1441056_at	Region	Usp3	235441	9	66641895	66716994	-
13338	1430164_a_at	Region	Grb10	14783	11	11828255	11865408	-
13339	1439687_at	Region	Rab14	68365	2	35113675	35132954	-
13340	1435439_at	Region	Dgcr8	94223	16	17025006	17060210	-
13341	1448864_at	Region	Snrk	20623	9	122152064	122189861	+
13342	1444338_at	Region	None	None	16	6726622	6727598	+
13343	1445693_at	Region	Araf	11836	X	19087538	19099458	+
13344	1422281_at	Region	Sstr4	20608	2	147852278	147857879	+
13345	1433521_at	Region	AI505652	433667	3	157015789	157017433	+
13346	1421535_a_at	Region	Pde4a	18577	9	21049525	21095183	+
13347	1452106_at	Region	Npnt	114249	3	131774826	131840439	-
13348	1419471_a_at	Region	Nudc	18221	4	132493443	132506848	-
13349	1428273_at	Region	1110065L07Rik	68904	8	9345221	9357725	+
13350	1417470_at	Region	Apobec3	80287	15	79944746	79960238	+
13351	1456087_at	Region	Nfia	18027	4	96752972	97087465	+
13352	1436549_a_at	Region	Hnrpa1	15382	15	103302595	103308263	+
13353	1451335_at	Region	Plac8	231507	5	99572372	99590844	-
13354	1425868_at	Region	---	229608	3	95757548	95765766	+
13355	1425668_a_at	Region	St3gal4	20443	9	34960623	35030854	-
13356	1445709_at	Region	Mdm1	17245	10	117835760	117862925	+
13357	1431593_a_at	Region	Leng5	66078	7	14664252	14671460	+
13358	1435296_at	Region	Adra2c	11553	5	33766347	33769514	+
13359	1417690_at	Region	Prkag1	19082	15	98869730	98888520	-
13360	1421564_at	Region	Serpina3c	16625	12	99591043	99598008	-
13361	1423374_at	Region	Ncoa6	56406	2	154847608	154895063	-
13362	1434252_at	Region	Tmcc3	319880	10	94549417	94562436	+
13363	1456699_s_at	Region	A730098D12Rik	231386	5	86098266	86098513	+
13364	1422443_at	Region	Xpnpep1	170750	19	52559059	52606418	-
13365	1434992_at	Region	9130206N08Rik	103406	10	81377132	81388250	+
13366	1434241_at	Region	Wdr67	210544	15	57921226	57979078	+
13367	1452795_at	Region	1110008B24Rik	73736	12	81839640	81851992	+
13368	1439025_at	Region	Zfp446	269870	7	10971529	10977990	+
13369	1455665_at	Region	None	None	8	35019239	35020080	-
13370	1417802_at	Region	1110032A04Rik	66183	3	69493969	69534304	-
13371	1426097_a_at	Region	BC018462	232821	7	4306107	4309695	+
13372	1449504_at	Region	Kpna1	16646	16	34764772	34817569	+
13373	1423538_at	Region	Ntan1	18203	16	12555143	12571313	+
13374	1449148_a_at	Region	Phtf1	18685	3	103395581	103434755	+
13375	1452328_s_at	Region	Pja2	224938	17	61986425	62035635	-
13376	1426364_at	Region	None	None	1	83643688	83645347	+
13377	1434339_at	Region	Fnbp1l	214459	3	121319281	121344496	-
13378	1454667_at	Region	Snx8	231834	5	139336643	139385591	-
13379	1444026_at	Region	AI593442	330941	9	52753199	52758226	-
13380	1424892_at	Region	Zfp95	22757	5	144243680	144260186	+
13381	1454116_a_at	Region	Mterfd1	66410	13	63514560	63535501	-
13382	1439503_at	Region	Zfp28	22690	7	5579089	5591525	+
13383	1437584_at	Region	None	None	17	45037685	45037880	+
13384	1451162_at	Region	Hsbp1	68196	8	118687933	118692324	+
13385	1435994_at	Region	None	None	1	192247678	192248924	+
13386	1424005_at	Region	B230219D22Rik	78521	13	54309682	54320058	+
13387	1458669_at	Region	None	None	8	18834847	18836188	+
13388	1421053_at	Region	Kif1a	16560	1	92845769	92929313	-
13389	1428829_at	Region	6820401H01Rik	75743	1	139020134	139023220	+

13390	1434106_at	Region	Epm2aip1	77781	9	111313226	111320386	+
13391	1451854_a_at	Region	Shrm	27428	5	92019185	92300704	+
13392	1425850_a_at	Region	Nek6	59126	2	38443862	38520203	+
13393	1426031_a_at	Region	Nfatc2	18019	2	167988261	168098994	-
13394	1460191_at	Region	0610042I15Rik	56418	11	5850619	5862567	+
13395	1427349_x_at	Region	2810021G02Rik	353208	NONE	NONE	NONE	NONE
13396	1437050_s_at	Region	D1Ertd396e	52477	1	190665121	190682759	+
13397	1423096_at	Region	None	None	14	29468476	29503944	+
13398	1459223_at	Region	B930095G15Rik	320268	14	115819440	115822231	+
13399	1425204_s_at	Region	Ddx19a /// Ddx19b	13680	8	110272730	110295521	-
13400	1428416_at	Region	3110050N22Rik	67306	3	7478034	7528335	+
13401	1432211_a_at	Region	Fbxo9	71538	9	78250521	78277651	-
13402	1430013_at	Region	2210008F06Rik	70057	11	77215513	77217069	+
13403	1423702_at	Region	H1f0	14958	15	79079749	79081790	+
13404	1451321_a_at	Region	0610033I05Rik	71684	2	51856619	51867325	-
13405	1438777_a_at	Region	AA881470	231841	5	139703855	139718166	+
13406	1432432_a_at	Region	Rab3c	67295	13	106309703	106528782	-
13407	1446592_at	Region	3526402H21Rik	66683	1	64841727	64842330	+
13408	1434805_at	Region	Mllt1	64144	17	54582533	54625310	-
13409	1429497_s_at	Region	Snx6	72183	12	0	675	-
13410	1450721_at	Region	Acp1	11431	12	27469324	27487670	-
13411	1437915_at	Region	Tom1l2	216810	11	59955659	60078526	-
13412	1436636_at	Region	None	None	NONE	NONE	NONE	NONE
13413	1439934_at	Region	E130106K10Rik	226781	1	184949416	184967214	+
13414	1427359_at	Region	A630082K20Rik	338523	6	39276016	39343119	-
13415	1447681_x_at	Region	None	None	NONE	NONE	NONE	NONE
13416	1432579_at	Region	Rshl2	66832	17	6405302	6448903	-
13417	1456808_at	Region	4933426M11Rik	217684	12	77651814	77742088	+
13418	1430359_a_at	Region	9130012B15Rik	78914	7	138209490	138236737	-
13419	1423018_at	Region	Kcna3	16491	3	106832252	106833838	+
13420	1417933_at	Region	Igfbp6	16012	15	102203722	102208872	+
13421	1459985_at	Region	Wdr61	66317	9	54834704	54852350	-
13422	1459420_at	Region	None	None		67480318	67480811	-
13423	1425140_at	Region	Lactb2	212442	1	13758941	13793555	-
13424	1430253_at	Region	2900006B11Rik	72897	11	90177649	90178918	-
13425	1434017_at	Region	Znrf2	387524	6	54961241	55034061	+
13426	1429708_at	Region	Ndufa11	69875	17	54407719	54415947	+
13427	1449096_at	Region	0610011N22Rik	67433	13	70412209	70421112	+
13428	1426444_at	Region	Rhbdl7	215160	5	134646202	134657638	+
13429	1426952_at	Region	Arhgap18	73910	10	26772308	26918428	+
13430	1415957_a_at	Region	Nnp1	18114	10	78510762	78523371	-
13431	1418971_x_at	Region	Bcl10	12042	3	144898767	144908672	+
13432	1439776_at	Region	4930415J21Rik	270086	8	93321463	93352094	+
13433	1426294_at	Region	Hapln1	12950	13	85655519	85726506	+
13434	1451401_a_at	Region	0610009K11Rik	68350	4	137315933	137323526	+
13435	1415917_at	Region	Mthfd1	108156	12	73111496	73175891	+
13436	1434764_at	Region	Akap11	219181	14	72844664	72875066	-
13437	1449213_at	Region	1110049G11Rik	66192	X	69012817	69014273	-
13438	1456648_at	Region	Man1a	17155	10	54152456	54322172	-
13439	1439743_at	Region	AW048948	106013	15	78718616	78719127	-
13440	1429551_at	Region	4930579G22Rik	69034	5	129077802	129080517	+
13441	1434196_at	Region	Dnaja4	58233	9	54817109	54833863	+
13442	1458279_at	Region	Foxo3	56484	10	42292466	42383422	-
13443	1439711_at	Region	None	None	16	71273621	71274227	+
13444	1454720_at	Region	Apba3 /// Hs3st5	319415	10	36577979	36905084	+
13445	1457265_at	Region	B230333C21Rik	338351	X	31228738	31232885	-
13446	1428846_at	Region	Ttc14	67120	3	33243957	33251783	+
13447	1422576_at	Region	Sca10	54138	15	85384867	85511564	+
13448	1455750_at	Region	BC053994	381383	2	145715500	145854821	-
13449	1440112_at	Region	Lrrtm3	216028	10	63866884	64028642	-
13450	1435906_x_at	Region	Gbp2	14469	3	141589637	141606982	+
13451	1424496_at	Region	5133401N09Rik	75731	13	56785992	56792260	+
13452	1417490_at	Region	Ctsb	13030	14	57653056	57676512	+
13453	1442039_at	Region	Tox	252838	4	6614604	6917869	-
13454	1423531_a_at	Region	Hnrpa1	15382	15	103302595	103308263	+

13455	1428068_at	Region	1110030L07Rik	68653	15	84240839	84264676	+
13456	1418874_a_at	Region	Psmd4	19185	3	94520536	94530376	-
13457	1422488_at	Region	Nxt1	56488	2	148129578	148132959	+
13458	1455139_at	Region	AI851716	98463	1	72936468	72937738	+
13459	1418162_at	Region	Tlr4	21898	4	65919004	65933990	+
13460	1450957_a_at	Region	Sqstm1	18412	11	49952993	49963631	-
13461	1456914_at	Region	AW209116	99896	3	107101334	107102010	-
13462	1452170_at	Region	2010209O12Rik	100910	5	23052073	23056385	+
13463	1427877_at	Region	2610312B22Rik	69082	2	83342327	83362366	+
13464	1431390_a_at	Region	Grinl1a	28015	9	71612808	71620280	-
13465	1460175_at	Region	Rps23	66475	13	87055520	87057074	+
13466	1423395_at	Region	Tsnax	53424	8	124300320	124321515	+
13467	1426483_at	Region	Prkrir	72981	7	92794813	92809512	+
13468	1436421_s_at	Region	Arpc5l	74192	2	38940301	38948034	+
13469	1448176_a_at	Region	Hnrpk	15387	13	57024107	57034672	-
13470	1416846_a_at	Region	Pdzrn3	55983	6	101625228	101853511	-
13471	1443127_at	Region	9630021D06Rik	319926	6	63877608	63878170	+
13472	1429545_at	Region	Ube2i /// F830028O17Rik /// LOC546265	22196	17	23066362	23080142	-
13473	1430542_a_at	Region	Slc25a5 /// LOC433923 /// LOC545025	11740	X	31416206	31419360	+
13474	1425706_a_at	Region	Ddb2	107986	2	90916605	90941731	-
13475	1435386_at	Region	Vwf	22371	6	126209758	126347067	+
13476	1440477_at	Region	None	None	2	164331594	164334746	-
13477	1421059_a_at	Region	Alg2	56737	4	47385756	47390247	-
13478	1418479_at	Region	Vps54	245944	11	21134074	21215926	+
13479	1425869_a_at	Region	Psen2	19165	1	180181252	180200147	-
13480	1452777_a_at	Region	6330412F12Rik	53312	5	23149873	23174275	+
13481	1439190_at	Region	2900090M10Rik	329977	4	140797550	140803687	-
13482	1423684_at	Region	Hnrpk	15387	13	57024107	57034672	-
13483	1434634_at	Region	Gm166 /// LOC546002	233899	7	121636263	121638050	-
13484	1448600_s_at	Region	Vav3	57257	3	109150078	109494759	+
13485	1417429_at	Region	Fmo1	14261	1	162739092	162775861	-
13486	1428766_at	Region	Rnmtl1	67390	11	75969428	75976280	+
13487	1446245_at	Region	None	None	2	52794997	52795679	+
13488	1456376_at	Region	None	None	2	155271934	155272202	+
13489	1422834_at	Region	Kcnd2	16508	6	21263145	21776841	+
13490	1460278_a_at	Region	D15Wsu75e	28075	15	82043240	82066857	-
13491	1434733_at	Region	Stk36	269209	1	74903970	74936564	+
13492	1421364_at	Region	Lrfn1	80749	7	23860311	23868010	+
13493	1416110_at	Region	Slc35a4	67843	18	36903357	36907622	+
13494	1435402_at	Region	A930008A22Rik	235283	9	40248196	40406053	-
13495	1427299_at	Region	Rps6ka3	110651	X	152855525	152962890	+
13496	1456421_at	Region	4930578M01Rik	75951	15	99043181	99043924	+
13497	1416951_a_at	Region	Atp6v1d	73834	12	75699581	75718058	-
13498	1439160_at	Region	4732496O08Rik	242736	4	142327675	142336336	+
13499	1434549_at	Region	Rab11a	53869	9	64838434	64859957	-
13500	1428499_at	Region	2810454L23Rik	72803	NONE	NONE	NONE	NONE
13501	1454811_a_at	Region	Tde2	56442	10	57778279	57794950	-
13502	1437918_at	Region	4930539E08Rik	207819	17	26697621	26717498	-
13503	1417810_a_at	Region	Kcnb1 /// Pacsin2	16500	2	166560111	166645532	-
13504	1433728_at	Region	BC038479	244757	9	26656943	26700320	-
13505	1424582_at	Region	Mulk	69923	6	40467075	40538342	+
13506	1437741_at	Region	Rab21	216344	10	114975874	115001563	-
13507	1440953_at	Region	1700096K18Rik	73571	5	24000313	24000831	+
13508	1450782_at	Region	Wnt4	22417	4	136158896	136178040	+
13509	1437150_at	Region	1700012H17Rik	242297	4	5571325	5727090	+
13510	1452761_a_at	Region	8430436O14Rik	71506	9	116559671	116560865	-
13511	1418587_at	Region	Traf3	22031	12	106693201	106740539	+
13512	1437540_at	Region	None	None	3	145117384	145118278	+
13513	1459826_at	Region	None	None	2	180795357	180795586	-
13514	1426413_at	Region	Neurod1	18012	2	79150390	79154385	-

13515	1446492_at	Region	C630028F04Rik	243274	5	126949997	127603737	-
13516	1455614_at	Region	Nr1i3	12355	1	171142875	171147683	+
13517	1435398_at	Region	Stxbp5	78808	10	9633720	9718785	-
13518	1433907_at	Region	None	None	9	36829898	36831652	-
13519	1416412_at	Region	Nsmaf	18201	4	6323372	6381407	-
13520	1439248_at	Region	4932432N11Rik	74386	13	57034424	57043384	+
13521	1437980_at	Region	9130230N09Rik	77671	11	22755630	22756054	+
13522	1433789_at	Region	Rnu17d	399101	4	131312832	131313595	-
13523	1435799_at	Region	Sfrs14	234373	8	69387208	69416080	+
13524	1447738_s_at	Region	Ankrd13d	68423	19	4058970	4071838	-
13525	1455676_x_at	Region	Tial1	21843	7	122491640	122510627	-
13526	1445415_at	Region	B230303A05	328191	13	15394036	15472574	-
13527	1438165_x_at	Region	Vat1	26949	11	101279835	101287286	-
13528	1435133_at	Region	Ugcg	22234	4	59132999	59166282	+
13529	1449977_at	Region	Egr4	13656	6	85861038	85863458	-
13530	1418260_at	Region	Hunk	26559	16	89543829	89656985	+
13531	1423927_at	Region	Slc35b2	73836	17	43074866	43078599	+
13532	1436540_at	Region	D10Bwg1379e	215821	10	18490148	18668026	-
13533	1428522_at	Region	Ttf2	74044	3	100364769	100395573	-
13534	1434820_s_at	Region	Pkig	18769	2	163150999	163183121	+
13535	1441082_at	Region	None	None	16	96074548	96074932	-
13536	1436104_a_at	Region	2310015A05Rik	72315	16	16417438	16421706	+
13537	1437875_at	Region	None	None	13	48984687	48986539	+
13538	1446326_at	Region	Col1a2	12843	6	4458646	4493615	+
13539	1430713_s_at	Region	Ndufa13	67184	8	69045459	69053837	-
13540	1416230_at	Region	Rfk	54391	19	16618183	16625435	+
13541	1442019_at	Region	B230343A10Rik	320013	11	67412753	67413382	-
13542	1420514_at	Region	Tmem47	192216	X	75731366	75757118	+
13543	1454862_at	Region	Phldb2	208177	16	44630808	44728527	-
13544	1458378_at	Region	Grin3a	242443	4	49579958	49588490	-
13545	1436339_at	Region	1810058I24Rik	67705	6	35346812	35356014	+
13546	1420891_at	Region	Wnt7b	22422	15	85584040	85630181	-
13547	1419108_at	Region	Ophn1	94190	X	93158876	93492346	-
13548	1426362_at	Region	5730537D05Rik	70652	3	79543274	79572782	-
13549	1422156_a_at	Region	Rps2 /// LOC432878 /// LOC434347 /// LOC544872 /// LOC545808 /// LOC546164 /// LOC546298	16898	17	22525236	22527051	+
13550	1423350_at	Region	Socs5	56468	17	84964483	84994389	+
13551	1458135_at	Region	None	None	X	65806702	65807363	+
13552	1451537_at	Region	Chi3l1	12654	1	134032745	134040294	+
13553	1417874_at	Region	2310004K06Rik	56786	7	103588707	103605134	-
13554	1424601_at	Region	Xrcc4	108138	13	85965021	86189500	-
13555	1454906_at	Region	Rarb	218772	14	14927459	15071124	-
13556	1446206_at	Region	B230209C24Rik	320705	1	34121165	34176734	-
13557	1440133_x_at	Region	Prkar1b	19085	5	138015364	138127749	-
13558	1422725_at	Region	Mak	17152	13	40586903	40631049	-
13559	1424790_at	Region	2900084M01Rik	73095	8	69337315	69347211	-
13560	1417399_at	Region	Gas6	14456	8	12844009	12873141	-
13561	1426725_s_at	Region	Ets1	23871	9	32617946	32676163	+
13562	1448543_at	Region	2310042G06Rik	66390	2	173889998	173897848	-
13563	1460394_a_at	Region	Inppl1	16332	7	95928562	95944145	-
13564	1435655_at	Region	Rpl12 /// LOC385403 /// LOC432518 /// LOC436225	269261	2	32893888	32896203	+
13565	1439093_at	Region	4921509J17Rik	70857	NONE	NONE	NONE	NONE
13566	1456440_s_at	Region	None	None	2	13567269	13568387	-
13567	1452923_at	Region	1810058I14Rik	75597	13	104279275	104385298	-
13568	1427293_a_at	Region	Auts2	319974	5	130627794	130728707	-
13569	1439321_at	Region	Arid1b	239985	17	4902975	5250462	+
13570	1426373_at	Region	Ski	20481	4	153649703	153714882	-

13571	1454778_x_at	Region	Rps28	54127	17	31521434	31522877	-
13572	1416796_at	Region	Nck2	17974	1	43740468	43865231	+
13573	1460213_at	Region	Golga4	54214	9	118500315	118578377	+
13574	1425332_at	Region	Zfp106	20402	2	120023462	120077639	-
13575	1428241_at	Region	2310035K24Rik	69596	2	130724293	130727367	+
13576	1439322_at	Region	None	None	14	88327571	88328263	-
13577	1417702_a_at	Region	Hnmt	140483	2	23935082	23981538	-
13578	1451344_at	Region	BC025600	231633	5	112904996	112911487	-
13579	1423760_at	Region	Cd44	12505	2	102518748	102606202	-
13580	1422346_at	Region	Titf1	21869	12	53257523	53262474	-
13581	1449089_at	Region	Nrip1	268903	16	75376752	75458939	-
13582	1449315_at	Region	Odz3	23965	8	47191599	47638956	-
13583	1455365_at	Region	Cdh8	12564	8	98317068	98691929	-
13584	1435044_at	Region	Ebf4	228598	2	129809792	129884319	+
13585	1433741_at	Region	Cd38	12494	5	42625902	42669430	+
13586	1451943_a_at	Region	Ppm1a	19042	12	69630561	69641441	+
13587	1427906_at	Region	1110037F02Rik	66185	4	11412923	11477649	+
13588	1451115_at	Region	Pias3	229615	3	96184305	96193798	+
13589	1416070_a_at	Region	Ddx18	66942	1	121317046	121331136	-
13590	1452801_at	Region	Pigk	329777	3	151707256	151782191	+
13591	1426348_at	Region	Col4a1	12826	8	10570852	10685155	-
13592	1453264_at	Region	Marveld3	73608	8	109245590	109259848	-
13593	1452039_a_at	Region	Bap1	104416	14	29383425	29391768	+
13594	1416141_a_at	Region	Rps6	20104	4	85840854	85843549	-
13595	1451672_at	Region	Gprk6	26385	13	54054856	54068930	+
13596	1433929_at	Region	Nhlrc2	66866	19	56133809	56184394	+
13597	1438039_at	Region	Hectd1	207304	3	48495784	48579550	-
13598	1418968_at	Region	Rb1cc1	12421	1	6180173	6241086	+
13599	1434863_at	Region	AU041129	107000	18	35894727	35895630	-
13600	1428346_at	Region	Trafd1	231712	5	120523537	120537378	-
13601	1428549_at	Region	Ccdc3	74186	2	5055048	5148143	+
13602	1452689_at	Region	Zfp512	269639	5	29911428	29940687	+
13603	1416755_at	Region	Dnajb1	81489	8	82866135	82869845	+
13604	1452854_at	Region	None	None	10	42936932	42938736	+
13605	1448784_at	Region	Taf10	24075	7	99857546	99858990	-
13606	1444046_at	Region	D430041B17	232813	7	4074328	4085024	-
13607	1425227_a_at	Region	Atp6v0a1	11975	11	100830569	100884801	+
13608	1428440_at	Region	Slc25a12	78830	2	70972038	71065297	-
13609	1422949_at	Region	Nos1	18125	5	116995765	117082459	+
13610	1455922_at	Region	4732493F09Rik	226407	1	127711504	127786580	+
13611	1447824_x_at	Region	Hspa5	14828	2	34704257	34708619	+
13612	1424831_at	Region	Cpne2	234577	8	93817200	93854701	+
13613	1453071_s_at	Region	Kdelc2	68304	9	53449283	53467127	+
13614	1444307_at	Region	None	None	4	122248532	122249344	+
13615	1422478_a_at	Region	Acas2	60525	2	154974980	155019680	+
13616	1434455_at	Region	Fbxo44	230903	4	146645163	146652242	-
13617	1460578_at	Region	Fgd5	232237	6	92435066	92523961	+
13618	1454813_at	Region	1110017O22Rik	66167	9	109124580	109128973	-
13619	1454728_s_at	Region	Atp8a1	11980	5	66388740	66614923	-
13620	1423559_at	Region	Kcnc1	16502	7	40482890	40513792	+
13621	1438720_at	Region	9330159F19Rik	212448	10	29242835	29259565	+
13622	1448280_at	Region	Syp	20977	X	5877651	5892191	+
13623	1435607_at	Region	Grm2	108068	9	106710294	106710874	-
13624	1440028_at	Region	4631423B10Rik	109292	6	115337300	115337941	-
13625	1455683_a_at	Region	Tbc1d8	54610	1	39668412	39699827	-
13626	1419174_at	Region	2410004B18Rik	66421	3	144912421	144918664	+
13627	1418485_at	Region	Slc4a3	20536	1	75837019	75849802	+
13628	1451525_at	Region	Arhgap12	75415	18	6067924	6178605	-
13629	1452877_at	Region	2700029M09Rik	72612	8	59937889	59955217	+
13630	1451435_at	Region	Cutl1	13047	5	135261627	135499343	-
13631	1449889_a_at	Region	Ociad1	68095	5	72077598	72098860	+
13632	1448772_at	Region	Ube2a	22209	X	31494920	31504776	+
13633	1426894_s_at	Region	C230093N12Rik	98952	2	32467521	32501912	+
13634	1434402_at	Region	Samd8	67630	14	20115199	20157956	+
13635	1434564_at	Region	E2f3	13557	13	29386198	29468641	-

13636	1416555_at	Region	Ei24	13663	9	36716320	36734233	-
13637	1436778_at	Region	Cybb	13058	X	7675646	7708207	-
13638	1416910_at	Region	Dnajd1	66148	14	72168649	72217349	-
13639	1433770_at	Region	Dpysl2	12934	14	61332083	61398082	-
13640	1433502_s_at	Region	AW550801	104662	11	74623765	74634998	+
13641	1416967_at	Region	Sox2	20674	3	34105755	34108159	+
13642	1443466_s_at	Region	Polr3b	70428	10	84517097	84621838	+
13643	1423783_at	Region	Tor2a	30933	2	32689433	32694406	+
13644	1437463_x_at	Region	Tgfbi	21810	13	55229952	55259688	+
13645	1425887_at	Region	4930511J11Rik	74720	16	8082569	8098429	-
13646	1451326_at	Region	1810013B01Rik	76491	9	106443674	106516765	+
13647	1425369_a_at	Region	Sox10	20665	15	79206217	79275996	-
13648	1418310_a_at	Region	Rlbp1	19771	7	73172072	73181986	-
13649	1421045_at	Region	Mrc2	17534	11	105113772	105171058	+
13650	1428827_at	Region	Whsc1	107823	5	32352922	32384263	+
13651	1416592_at	Region	Glrx1	93692	13	71896546	71906783	+
13652	1426644_at	Region	Tbc1d20	67231	2	151750808	151769526	+
13653	1451992_at	Region	Adrbk1	110355	19	4074791	4094745	-
13654	1452918_at	Region	D19Ertd737e	76539	19	59801013	59829124	-
13655	1424231_s_at	Region	Sec15l1	107371	19	36893738	37026525	+
13656	1448642_at	Region	Pcbp1	23983	6	86959261	86960934	-
13657	1452393_at	Region	AI597013	100182	4	62458296	62471256	-
13658	1455169_at	Region	Rab11fip2	74998	19	59505244	59508287	-
13659	1433847_at	Region	D330017J20Rik	320609	6	30005780	30006779	+
13660	1426991_at	Region	1810048J11Rik	67708	12	69380024	69386367	+
13661	1438291_x_at	Region	None	None	5	66219796	66219942	+
13662	1439634_at	Region	4930505D03Rik	74694	10	120920073	120967773	-
13663	1435052_at	Region	4930455F23Rik	74895	1	164194019	164206339	+
13664	1439569_at	Region	Gpr83	14608	9	14693694	14702939	+
13665	1435634_at	Region	A030012M09Rik	319263	1	7090301	7142844	+
13666	1448996_at	Region	Rom1	19881	19	8123939	8125735	-
13667	1456829_at	Region	Pnma3	245468	X	67725442	67728846	+
13668	1451199_at	Region	Qtrtd1	106248	16	42734124	42762404	-
13669	1425136_x_at	Region	Dnm2	13430	9	21313566	21396180	+
13670	1450642_at	Region	3110001I20Rik	70354	2	125250846	125296723	-
13671	1440083_at	Region	A430061O12Rik	319285	4	136217733	136218104	-
13672	1426861_at	Region	9130023F12Rik	216549	11	20579877	20636277	-
13673	1415717_at	Region	4931406I20Rik	66743	4	116230369	116455820	-
13674	1456946_at	Region	4831416G18Rik	237353	10	58942240	59096717	+
13675	1457310_x_at	Region	LOC545474	545474	2	151550295	151552735	+
13676	1439481_at	Region	Ipo9	226432	1	135234946	135287679	-
13677	1460676_at	Region	1300006C06Rik	74158	15	79726573	79740139	-
13678	1420670_at	Region	Arnt2	11864	7	78100192	78263873	-
13679	1438019_at	Region	1810043M15Rik	75678	13	49020824	49062503	+
13680	1441866_s_at	Region	Ptdss1	19210	13	63535264	63600822	+
13681	1418736_at	Region	B3galt3	26879	3	69244708	69269383	-
13682	1439643_at	Region	Orc4l	26428	2	48835014	48881396	-
13683	1424394_at	Region	MGI:2149786	114679	11	3409673	3412137	+
13684	1460393_a_at	Region	Dusp7	235584	9	106362584	106369022	+
13685	1426878_at	Region	2610016F04Rik	66923	14	29151150	29207125	+
13686	1416877_a_at	Region	Mrpl51	66493	6	125848878	125851070	+
13687	1415731_at	Region	D1Ertd396e	52477	1	190665121	190682759	+
13688	1424386_at	Region	BC020184	225362	18	35064316	35071113	+
13689	1456482_at	Region	Pik3r3	18710	4	115180819	115261961	+
13690	1426307_at	Region	Ncb5or	266690	9	87375157	87425198	+
13691	1416325_at	Region	Crisp1	11571	17	37800390	37825831	-
13692	1460010_a_at	Region	Ptdss2	27388	7	135533273	135558141	+
13693	1416612_at	Region	Cyp1b1	13078	17	77524347	77532435	-
13694	1423236_at	Region	Galnt1	14423	18	24433937	24514141	+
13695	1437073_x_at	Region	AV025504	105633	14	70102611	70102960	-
13696	1429783_at	Region	Pdlim5	56376	3	141211634	141364594	-
13697	1452757_s_at	Region	Hba-a1	15122	11	32178468	32179287	+
13698	1418953_at	Region	Fbxo16	50759	14	59793581	59848382	+
13699	1438401_at	Region	Ubn1	170644	16	4719862	4734397	+
13700	1416966_at	Region	Slc22a8	19879	19	7787836	7808381	+

13701	1454933_at	Region	2610027C15Rik	230752	4	125175116	125176987	+
13702	1453312_at	Region	Iqwd1	74106	1	165251609	165382346	-
13703	1416804_at	Region	LOC114601	114601	19	5496839	5515060	-
13704	1439980_at	Region	D14Ertd725e	52524	14	19966948	19968133	+
13705	1440895_at	Region	None	None	4	98095988	98096867	+
13706	1419411_at	Region	Tac2	21334	10	127461188	127468477	+
13707	1455195_at	Region	Rps24	20088	14	22859652	22865117	+
13708	1442983_at	Region	Slc23a2	54338	2	131566349	131658961	-
13709	1438318_x_at	Region	1500001L15Rik	68966	14	49532485	49541105	+
13710	1419070_at	Region	Cys1	12879	12	21215593	21231550	-
13711	1443167_at	Region	Rnf12	19820	X	98558532	98582649	-
13712	1426566_s_at	Region	Il17re	57890	6	114026314	114038581	+
13713	1456962_at	Region	Cntn2	21367	1	132366699	132400216	-
13714	1454980_at	Region	4930402E16Rik	319518	8	110392463	110432862	+
13715	1428998_at	Region	Phf3	213109	1	31075361	31146157	-
13716	1451283_at	Region	1810073G14Rik	67726	11	57208629	57244244	-
13717	1433806_x_at	Region	Calr	12317	8	84108911	84113928	-
13718	1425835_a_at	Region	Bbx	70508	16	49039981	49275616	-
13719	1429440_at	Region	1810041L15Rik	72301	15	84428627	84495696	-
13720	1439389_s_at	Region	Myadm	50918	7	4169	12984	-
13721	1434966_at	Region	Sfrs8	231769	5	128676882	128691989	+
13722	1423460_at	Region	Perq1	57330	5	136470004	136476570	+
13723	1421479_at	Region	Zfp318	57908	17	43894944	43929210	+
13724	1448873_at	Region	Ocln	18260	13	96649598	96704908	-
13725	1419399_at	Region	Mttp	17777	3	136979815	137020253	-
13726	1420840_at	Region	Plekha3	83435	2	76373044	76394322	+
13727	1457390_at	Region	Prpf3	70767	3	95318454	95343585	-
13728	1417833_at	Region	Zc3h10	103284	10	128280653	128284834	-
13729	1452141_a_at	Region	Sepp1	20363	15	3051735	3061247	+
13730	1415692_s_at	Region	Canx	12330	11	50047308	50078432	-
13731	1456401_at	Region	Cacnb2	12296	2	14530578	14913205	+
13732	1437938_x_at	Region	Dnm2	13430	9	21313566	21396180	+
13733	1434765_at	Region	Ep300	328572	15	81636928	81702791	+
13734	1458269_at	Region	Pcdh9	211712	14	88351670	88356571	-
13735	1460295_s_at	Region	Il6st	16195	13	108856634	108899366	+
13736	1421077_at	Region	Sertad3	170742	7	22849842	22853366	+
13737	1453956_a_at	Region	Pftk1	18647	5	4809738	5386208	-
13738	1455610_at	Region	Dmn	233335	7	61614971	61644496	-
13739	1428788_at	Region	1700012G19Rik	67078	17	22274058	22275187	+
13740	1451906_at	Region	Ubxd3	212190	4	137599799	137618428	-
13741	1423128_at	Region	Aip	11632	19	3903236	3914617	-
13742	1439220_at	Region	Ank3	11735	10	69589190	70079102	+
13743	1433504_at	Region	Pygb	110078	2	150243732	150288684	+
13744	1428781_at	Region	1110014F24Rik	73712	7	26172733	26205221	+
13745	1438739_at	Region	Cnbp1	12785	6	88279741	88287657	-
13746	1440372_at	Region	Arfrp2	218639	13	110208602	110571472	+
13747	1424850_at	Region	Map3k1	26401	13	108136840	108199233	-
13748	1455715_at	Region	None	None	17	92809459	92809967	+
13749	1433981_s_at	Region	Htatip	81601	19	5392208	5398819	-
13750	1425950_at	Region	BC019537	228993	2	180442337	180451134	+
13751	1426674_at	Region	Eif3s9	27979	5	139415645	139439698	+
13752	1435322_at	Region	1110011C06Rik	68494	11	94155722	94162935	+
13753	1419211_s_at	Region	4933424B01Rik	71177	6	147470322	147498449	-
13754	1437105_at	Region	Jarid1a	214899	6	120796139	120871561	+
13755	1416572_at	Region	Mmp14	17387	14	48948545	48958231	+
13756	1420915_at	Region	Stat1	20846	1	52420399	52461905	+
13757	1438882_at	Region	Arhgap18	73910	10	26772308	26918428	+
13758	1423076_at	Region	Snx9	66616	17	5750779	5837658	+
13759	1436398_at	Region	Lef1	16842	3	130040399	130153310	+
13760	1452988_at	Region	Aak1	269774	6	87282212	87283599	-
13761	1434510_at	Region	Papss2	23972	19	31944210	31991770	+
13762	1444112_at	Region	None	None	3	89148860	89154952	+
13763	1444578_at	Region	Spop	20747	11	95235201	95314100	+
13764	1436632_at	Region	D130059P03Rik	320538	6	38570159	38638809	+
13765	1456172_at	Region	Txndc5	105245	13	38041006	38069191	-

13766	1424043_at	Region	Ppil4	67418	10	7658399	7688620	+
13767	1416436_a_at	Region	2410003P15Rik	56046	2	155303838	155386985	-
13768	1423187_at	Region	Gabarapl2	93739	8	111238426	111253086	+
13769	1423818_a_at	Region	Arl6ip1	54208	7	111994757	112005420	-
13770	1423741_at	Region	Rbm10	236732	X	18858936	18889829	+
13771	1460428_at	Region	1100001D10Rik	68420	5	113884556	113915448	+
13772	1429077_x_at	Region	Rpl21	19933	5	145724838	145728723	+
13773	1452359_at	Region	AA536743	100532	5	62707529	62766865	-
13774	1457566_at	Region	A830058L05Rik	210503	17	19253175	19268702	+
13775	1427231_at	Region	Robo1	19876	16	71741946	72126752	+
13776	1418175_at	Region	Vdr	22337	15	97930914	97984749	-
13777	1429821_at	Region	2810046L04Rik	212127	3	53098037	53115975	+
13778	1448230_at	Region	Usp10	22224	8	119267518	119314223	+
13779	1435912_at	Region	AI196514	224111	16	31136065	31196127	+
13780	1423700_at	Region	Rfc3	69263	5	120	8093	+
13781	1438894_at	Region	None	None	13	28173624	28174273	-
13782	1418169_at	Region	Zcchc14	142682	8	120981449	121033085	-
13783	1418698_a_at	Region	Fech	14151	18	64689244	64721633	-
13784	1452515_a_at	Region	Xylt2	217119	11	94484938	94491627	-
13785	1417360_at	Region	Mlh1	17350	9	111269552	111312859	-
13786	1456336_at	Region	A330102K23Rik	77771	2	65701242	65878859	+
13787	1420554_a_at	Region	Rac3	170758	11	120542561	120545062	+
13788	1459639_at	Region	Brsk2	75770	7	136362546	136417039	+
13789	1448322_a_at	Region	Cox4i1	12857	8	120025652	120031553	+
13790	1428649_at	Region	Cand1	71902	10	118778384	118938138	-
13791	1436075_at	Region	Sfrp5	54612	19	41742303	41746463	-
13792	1431038_at	Region	Rassf4	213391	6	117070312	117109861	-
13793	1451692_at	Region	2410015B03Rik	71983	18	36958833	36966162	+
13794	1416703_at	Region	Mapk14	26416	17	26492551	26549631	+
13795	1452335_at	Region	2810423E13Rik	72175	3	40237869	40269375	-
13796	1456544_at	Region	Tmem38b	52076	4	53741941	53776470	+
13797	1423875_at	Region	AI450540	226252	19	56946405	56975311	+
13798	1424347_at	Region	Ppp6c	67857	2	39128960	39158501	-
13799	1416978_at	Region	Fcgrt	14132	7	39169606	39179634	-
13800	1430652_at	Region	9030612E09Rik	74530	10	43281064	43282922	+
13801	1434835_at	Region	BC037674	218914	14	32808636	32880366	+
13802	1417371_at	Region	Peli1	67245	11	21030262	21045116	+
13803	1434385_at	Region	Tom1l2	216810	11	59955659	60078526	-
13804	1456542_s_at	Region	Qrsl1	76563	10	43983931	44011492	-
13805	1417618_at	Region	Itih2	16425	2	10010705	10046778	-
13806	1452149_at	Region	Ube3b	117146	5	113501027	113531732	+
13807	1418253_a_at	Region	Hspa4l	18415	3	40165310	40210062	+
13808	1448130_at	Region	Fdft1	14137	14	57675741	57708384	-
13809	1449095_at	Region	Vps54	245944	11	21134074	21215926	+
13810	1429133_at	Region	4930519N16Rik	75124	13	15736	19903	-
13811	1415937_s_at	Region	Pdcd6ip	18571	9	113708127	113760882	-
13812	1455435_s_at	Region	None	None	14	28173232	28174329	+
13813	1433827_at	Region	Atp8a1	11980	5	66388740	66614923	-
13814	1451109_a_at	Region	Nedd4	17999	9	72794997	72882331	+
13815	1446844_at	Region	Chchd7	66433	4	3866060	3870669	+
13816	1448803_at	Region	Golga4	54214	9	118500315	118578377	+
13817	1442557_at	Region	Syt1	20979	10	108385089	108577499	-
13818	1429054_at	Region	Mrpl47	74600	3	32135718	32144954	-
13819	1443117_at	Region	Eya1	14048	1	14348042	14481971	-
13820	1415939_at	Region	Fmod	14264	1	133888996	133898985	+
13821	1417250_at	Region	Rnf12	19820	X	98558532	98582649	-
13822	1426893_at	Region	C230093N12Rik	98952	2	32467521	32501912	+
13823	1415781_a_at	Region	Sumo2 /// LOC236622	170930	11	115344202	115357323	-
13824	1430614_at	Region	4632415K11Rik	74347	8	119114536	119133850	-
13825	1437103_at	Region	C330012H03Rik	319765	16	20832300	20935863	-
13826	1437989_at	Region	Pde8b	218461	13	91208631	91434426	-
13827	1427152_at	Region	4732486I23Rik	99003	2	104462387	104521514	-
13828	1428795_at	Region	1110021L09Rik	76306	10	23801094	23802894	+
13829	1452747_at	Region	Atp13a2	74772	4	139868189	139888591	+

13830	1423956_at	Region	Smap1	98366	1	24090771	24166945	-
13831	1428369_s_at	Region	Arhgap21	71435	2	20889950	21009731	-
13832	1416797_at	Region	Nck2	17974	1	43740468	43865231	+
13833	1435011_x_at	Region	Akr1a4	58810	4	115595415	115610556	-
13834	1440317_at	Region	C130068B02Rik	402752	17	27974213	27974705	+
13835	1423171_at	Region	Gpr88	64378	3	115019299	115023427	-
13836	1430326_s_at	Region	1500040F11Rik	22272	11	53180763	53183655	-
13837	1455555_at	Region	AV071699	102723	9	121684531	121685430	+
13838	1429639_at	Region	2310032D16Rik	74182	2	132043251	132092031	-
13839	1428817_at	Region	2610528O22Rik	67074	10	122685914	122766593	-
13840	1436899_at	Region	2700019D07Rik	72580	10	33986876	34011152	-
13841	1420864_at	Region	Zfp161	22666	17	67159059	67165625	+
13842	1448440_x_at	Region	D17Wsu104e	28106	17	53813582	53820961	-
13843	1434387_at	Region	AI429612	106581	17	24005883	24037311	-
13844	1437339_s_at	Region	Pcsk5	18552	19	16656932	17062422	-
13845	1446583_at	Region	None	None	NONE	NONE	NONE	NONE
13846	1438371_x_at	Region	Ddx5	13207	11	106602635	106609565	-
13847	1421520_at	Region	Jph1	57339	1	17181923	17282257	-
13848	1426574_a_at	Region	Add3	27360	19	52767533	52814957	+
13849	1435406_at	Region	Cchcr1	240084	17	33234535	33248617	+
13850	1445534_at	Region	Flnb	286940	14	5564611	5619816	+
13851	1454967_at	Region	A930001N09Rik	77128	17	24510472	24568084	+
13852	1428449_at	Region	Gtf3c2	71752	5	29615739	29638859	-
13853	1441359_at	Region	9230115F04Rik	320841	4	107497810	107498665	+
13854	1417211_a_at	Region	1110032A03Rik	68721	9	50835154	50840478	-
13855	1416319_at	Region	Adk	11534	14	19417295	19813215	+
13856	1434796_at	Region	Vamp4	53330	1	162480609	162508875	+
13857	1431491_at	Region	9430087N24Rik	77440	15	43679380	43680585	-
13858	1418512_at	Region	Stk3	56274	15	34876656	35155734	-
13859	1443858_at	Region	None	None	7	98451112	98452137	-
13860	1416248_at	Region	Nadk	192185	4	154054766	154083359	+
13861	1417308_at	Region	Pkm2	18746	9	59773488	59796255	+
13862	1460296_a_at	Region	Fgf22	67112	10	79877743	79879585	+
13863	1415916_a_at	Region	Mthfd1	108156	12	73111496	73175891	+
13864	1423671_at	Region	Dner	227325	1	84703796	85030178	-
13865	1425622_at	Region	Edil3	13612	13	84938267	85437638	+
13866	1433891_at	Region	Gpr48	107515	2	109541897	109638012	+
13867	1436167_at	Region	LOC435684	435684	2	121862745	121882715	-
13868	1451989_a_at	Region	Mapre2	212307	18	24032480	24122349	+
13869	1417708_at	Region	Syt3	20981	7	38460249	38475359	+
13870	1444245_at	Region	B230118H07Rik	68170	2	101265464	101333669	-
13871	1424236_at	Region	Tbc1d10b	68449	7	121247196	121258199	-
13872	1450421_at	Region	Tgfa	21802	6	86626355	86707058	+
13873	1454822_x_at	Region	Apcdd1	494504	18	63040206	63182348	+
13874	1459133_at	Region	2310050N11Rik	66967	1	151613030	151679348	+
13875	1416500_at	Region	Sacm1l	83493	9	123553586	123616302	+
13876	1427903_at	Region	Phpt1	75454	2	25505593	25507033	-
13877	1425235_s_at	Region	Col20a1	73368	2	180703493	180734498	+
13878	1445393_at	Region	Slc24a2	76376	4	85975964	86216615	-
13879	1416620_at	Region	Smarcal1	54380	1	72884934	72934797	+
13880	1435068_at	Region	Pip5k2b	108083	11	97536244	97565791	-
13881	1437927_at	Region	Dlgh2	23859	7	85860491	86494465	+
13882	1452737_at	Region	2810008M24Rik	75616	13	104271134	104275859	+
13883	1416515_at	Region	Fscn1	14086	5	142024679	142037166	+
13884	1440207_at	Region	AI505034	99010	2	111863079	111870349	+
13885	1451603_at	Region	A330096I21Rik	234542	8	84215010	84224777	+
13886	1452112_a_at	Region	4921506I22Rik	66704	19	4545410	4554730	+
13887	1446371_at	Region	Rab18	19330	18	60692	85718	-
13888	1450718_at	Region	MGI:1345171	23921	5	135231641	135258395	-
13889	1455746_at	Region	Kif13a	16553	13	46347970	46527871	-
13890	1426836_s_at	Region	Metap1	75624	3	137348195	137378617	-
13891	1435603_at	Region	Sned1	208777	1	93063332	93128556	+
13892	1438966_x_at	Region	Fmod	14264	1	133888996	133898985	+
13893	1438700_at	Region	Fnbp4	55935	2	90450119	90485770	+
13894	1435729_at	Region	MGI:1932697	83409	3	88293683	88296791	-

13895	1417865_at	Region	Tnfaip1	21927	11	78248507	78261853	-
13896	1421339_at	Region	Extl3	54616	14	59578943	59607355	-
13897	1418911_s_at	Region	Acsl4	50790	X	135758036	135830547	-
13898	1416471_at	Region	Cept1	99712	3	106298089	106343590	-
13899	1425483_at	Region	Tox	252838	4	6614604	6917869	-
13900	1441823_at	Region	Rai17	328365	14	23834490	24039917	+
13901	1435539_at	Region	None	None	5	33399634	33400612	+
13902	1455089_at	Region	Gng12	14701	6	67148787	67270629	+
13903	1435918_at	Region	BC055107	268709	14	5963634	5976774	-
13904	1417520_at	Region	Nfe2l3	18025	6	51579895	51606046	+
13905	1435335_a_at	Region	MGC79224	432486	10	88350966	88419158	+
13906	1436164_at	Region	Slc30a1	22782	1	191646393	191649708	+
13907	1458443_at	Region	Mect1	382056	8	69536831	69594238	-
13908	1439999_at	Region	None	None	10	99007139	99008009	+
13909	1417696_at	Region	Soat1	20652	1	156336595	156379733	-
13910	1416999_at	Region	Smpd2	20598	10	41589000	41592168	-
13911	1422331_at	Region	Pou3f3	18993	1	42991862	42993355	+
13912	1424416_at	Region	Nkiras2	71966	11	100444042	100448689	+
13913	1425280_at	Region	Leng1	69757	7	14891000	14896729	-
13914	1433745_at	Region	Trio	223435	15	27358260	27724087	-
13915	1420905_at	Region	Il17r	16172	6	120895204	120915007	+
13916	1428619_at	Region	2310005N03Rik	66359	1	178273456	178277078	+
13917	1453015_at	Region	5830471E12Rik	77208	8	116794427	116795963	+
13918	1436594_at	Region	C630016O21Rik	210105	7	37654761	37668386	+
13919	1422710_a_at	Region	Cacna1h	58226	17	23180201	23239019	-
13920	1416128_at	Region	Tuba6	22146	15	99088281	99096040	+
13921	1448644_at	Region	Pef1	67898	4	129134669	129155247	+
13922	1438773_at	Region	Steap2	74051	5	5676943	5699766	-
13923	1458947_at	Region	Fancc	14088	13	60694745	60814715	-
13924	1438098_at	Region	Dlgap1	224997	17	68195196	68591524	+
13925	1448359_a_at	Region	MGI:1930666	56295	9	121862188	121871251	-
13926	1449853_at	Region	Sfxn2	94279	19	46120441	46142562	+
13927	1452626_a_at	Region	1810014F10Rik	69064	7	115559954	115560372	-
13928	1436596_at	Region	H2afv	77605	11	6323158	6339126	-
13929	1450759_at	Region	Bmp6	12161	13	37884616	38040468	+
13930	1422329_a_at	Region	Ntrk3	18213	7	71981888	72367304	-
13931	1426344_at	Region	Gle1l /// LOC383774	383774	2	166463726	166465455	-
13932	1458407_s_at	Region	AI429294	106580	17	44879273	44880181	+
13933	1434876_at	Region	None	None	15	93305950	93308554	-
13934	1434306_at	Region	Rab3ip	216363	10	116595236	116639832	-
13935	1439081_at	Region	Mgea5	76055	19	45297695	45330727	-
13936	1416449_x_at	Region	Stxbp2	20911	8	36313	47968	+
13937	1437852_x_at	Region	Cpsf3	54451	12	458	16231	-
13938	1441947_x_at	Region	BC033915	70661	9	46026130	46236394	+
13939	1431248_at	Region	5031426D15Rik	68144	2	6842085	6845806	-
13940	1426443_at	Region	Rhbdl7	215160	5	134646202	134657638	+
13941	1455047_at	Region	Fbxo3	57443	2	103732553	103767991	+
13942	1436187_at	Region	1110054M08Rik	68841	16	23178085	23179052	-
13943	1437902_s_at	Region	Rarres2	71660	6	48702577	48705549	-
13944	1449460_at	Region	Asb13	142688	13	3518456	3536159	+
13945	1427003_at	Region	Ppp2r5c	26931	12	105961123	106055786	+
13946	1457628_at	Region	2900091E11Rik	67282	10	88172924	88217989	+
13947	1452427_s_at	Region	AW742319	57874	9	65109184	65143902	-
13948	1416720_at	Region	Sfrs6	67996	2	162388514	162394090	+
13949	1442880_at	Region	None	None	16	33377005	33377830	-
13950	1436592_at	Region	None	None	17	30619011	30619737	-
13951	1430053_a_at	Region	2810409H07Rik	67059	2	72790552	72912196	-
13952	1450870_at	Region	Rala	56044	13	17345050	17361579	-
13953	1416690_at	Region	Gtpbp2	56055	17	43670992	43679289	+
13954	1435249_at	Region	Btaf1	107182	19	36299109	36355422	+
13955	1455075_at	Region	Pigv	230801	4	132621286	132633519	-
13956	1453058_at	Region	Wdr5b	69544	16	34822597	34824381	+
13957	1448835_at	Region	E2f6	50496	12	16177835	16193683	+
13958	1426659_a_at	Region	Rpl23a	268449	11	77906594	77909202	-

13959	1450669_at	Region	Map3k11	26403	19	5478560	5491655	+
13960	1426231_at	Region	Vit	74199	17	76325212	76449617	+
13961	1436557_at	Region	Kb36	223915	15	101851770	101860794	-
13962	1419883_s_at	Region	Atp6v1b2	11966	8	68237311	68262233	+
13963	1452662_a_at	Region	Eif2s1	13665	12	75718669	75742127	+
13964	1415849_s_at	Region	Stmn1	16765	4	133429314	133434742	+
13965	1449155_at	Region	Polr3g	67486	13	77747624	77784787	-
13966	1448161_a_at	Region	Clcn4-2	12727	7	241	13444	+
13967	1448548_at	Region	Tulp4	68842	17	6045120	6146999	+
13968	1453768_a_at	Region	5430432M24Rik	73847	2	151426334	151437065	-
13969	1443968_at	Region	Adarb1	110532	10	77405789	77470808	-
13970	1448615_at	Region	Ccs	12460	19	4614163	4628079	-
13971	1434445_at	Region	D15Wsu169e	223666	15	76775364	76869603	-
13972	1424586_at	Region	Ehbp1	216565	11	21900621	22180631	-
13973	1435449_at	Region	Bcl2l11	12125	2	127639891	127676025	+
13974	1436206_at	Region	Fbxo10	269529	4	44950173	45023955	-
13975	1428556_at	Region	2610022G08Rik	66459	6	57848988	57854482	-
13976	1439435_x_at	Region	Pgk1 /// LOC381164 /// LOC432633 /// LOC433594 /// LOC545273	18655	X	100788594	100805024	+
13977	1450666_s_at	Region	Sca10	54138	15	85384867	85511564	+
13978	1450103_a_at	Region	Pscd2	19158	7	39891518	39899167	-
13979	1439240_x_at	Region	Lin7b	22342	7	39443916	39446815	-
13980	1440842_at	Region	C230085N15Rik	320556	17	49294754	49295599	-
13981	1460350_at	Region	Osbp	76303	19	11155242	11190607	+
13982	1449351_s_at	Region	Pdgfc	54635	3	80765412	80943027	+
13983	1417381_at	Region	C1qa	12259	4	135777175	135780017	-
13984	1415887_at	Region	Tfg	21787	16	55593941	55620963	-
13985	1440348_at	Region	Zfyve9	230597	4	107596378	107682819	-
13986	1423369_at	Region	Fmr1	14265	X	63347151	63386559	+
13987	1453633_a_at	Region	Rnf41	67588	10	128148476	128178259	+
13988	1420870_at	Region	Mllt10	17354	2	18097281	18254400	+
13989	1421686_at	Region	Rfrp	60531	6	50798600	50802122	-
13990	1419332_at	Region	Egfl6	54156	X	160122412	160185121	-
13991	1449098_a_at	Region	Poli	26447	18	70741352	70762990	-
13992	1436913_at	Region	Cdc14a	229776	3	115042345	115193064	-
13993	1435223_at	Region	Spfh2	244373	8	25757135	25772700	+
13994	1421336_at	Region	Prox1	19130	1	189836615	189885751	-
13995	1417032_at	Region	Ube2g2	22213	10	77733757	77757426	+
13996	1420000_s_at	Region	Igbp1	18518	X	95095706	95117362	+
13997	1447421_at	Region	None	None	1	97830529	97830926	-
13998	1451284_at	Region	Yipf3	28064	17	43758020	43761938	+
13999	1426113_x_at	Region	Tcra /// A430107P09Rik	21473	14	48328382	48744068	+
14000	1442757_at	Region	Lrch1	380916	14	69108806	69291857	-
14001	1417483_at	Region	Nfkbiz	80859	16	54703494	54731495	-
14002	1420836_at	Region	Slc25a30	67554	14	70104494	70129532	-
14003	1452467_at	Region	Mmab	77697	5	113541603	113554596	-
14004	1415755_a_at	Region	Ube2v1	66589	2	167064579	167088725	-
14005	1415758_at	Region	2510002A14Rik	72313	5	71804336	71855006	-
14006	1433625_at	Region	5830434P21Rik	227723	2	32083319	32166687	+
14007	1429982_at	Region	4933426K21Rik	108653	6	123118089	123137380	-
14008	1432464_a_at	Region	2310057J16Rik	69697	8	23358073	23360766	+
14009	1428642_at	Region	Slc35d3	76157	10	19774080	19777622	-
14010	1439014_at	Region	A430031N04	239796	16	27616035	27624663	-
14011	1454872_at	Region	B230308N11Rik	320060	17	32236723	32238815	+
14012	1431391_at	Region	Ralgps1	241308	2	33069135	33303640	-
14013	1426685_a_at	Region	Cnot6	104625	11	49427539	49465551	-
14014	1416702_at	Region	Serpini1	20713	3	75287431	75372212	+
14015	1428608_at	Region	Mylc2b	67938	17	68745819	68762228	-
14016	1436771_x_at	Region	Pgd	110208	4	147642356	147659030	-
14017	1427266_at	Region	MGI:1923998	76748	14	29154679	29206935	+
14018	1451152_a_at	Region	Atp1b1	11931	1	164355219	164376238	-

14019	1452069_a_at	Region	Thap7	69009	16	16298950	16302020	-
14020	1418240_at	Region	Gbp2	14469	3	141589637	141606982	+
14021	1453323_at	Region	2900079G21Rik	331033	9	112275506	112277260	+
14022	1448627_s_at	Region	Pbk	52033	14	60333331	60345242	+
14023	1429618_at	Region	Cyld	74256	8	87990637	88033868	+
14024	1448895_a_at	Region	Ctnna2	12386	6	77231681	78264788	-
14025	1441380_at	Region	2810439F02Rik	72747	18	12831155	12924403	+
14026	1455393_at	Region	None	None	3	19329204	19334411	+
14027	1451083_s_at	Region	Aars	234734	8	110331687	110354590	+
14028	1447346_s_at	Region	5530400B01Rik	71434	7	136492284	136493974	-
14029	1453643_at	Region	1700001L19Rik	69315	13	64618222	64634272	+
14030	1429736_at	Region	1110003F05Rik	66145	17	8844203	8846425	-
14031	1446394_at	Region	Oprk1	18387	1	5551006	5565381	+
14032	1455082_at	Region	Cblb	208650	16	50949437	51125945	+
14033	1416081_at	Region	Smad1	17125	8	78552485	78613558	-
14034	1434218_at	Region	C330019G07Rik	215476	5	31277729	31316129	-
14035	1427987_at	Region	Safb2	224902	17	54251255	54263875	-
14036	1437821_at	Region	Diap1	13367	18	38068549	38159133	-
14037	1423825_at	Region	5031439A09Rik	68151	3	4597	6180	+
14038	1447602_x_at	Region	Sulf2	72043	2	165530645	165530946	-
14039	1437012_x_at	Region	Rapgef3	223864	15	97820598	97843317	-
14040	1453189_at	Region	Ube2i	22196	17	23066362	23080142	-
14041	1416039_x_at	Region	Cyr61	16007	3	144622756	144625761	-
14042	1438211_s_at	Region	Dbp	13170	7	39788555	39793474	+
14043	1434955_at	Region	38412	72925	8	65495563	65579550	+
14044	1439325_at	Region	Blmh	104184	11	76671417	76713035	+
14045	1456618_at	Region	Mark4	232944	7	16295319	16327738	-
14046	1432526_a_at	Region	D11Moh34	27681	11	95856004	95868492	+
14047	1436365_at	Region	Zbtb36	207259	18	76055363	76384962	+
14048	1421267_a_at	Region	Cited2	17684	10	17647360	17648901	+
14049	1436324_at	Region	Stard9	211824	2	120141779	120229931	+
14050	1422693_a_at	Region	Rpo2tc1	20024	15	11792969	11807128	-
14051	1427739_a_at	Region	Trp53	22059	11	69305639	69317529	+
14052	1447704_s_at	Region	D530033C11Rik	78581	15	51874725	51881202	+
14053	1443834_at	Region	None	None	5	70925059	70925299	+
14054	1436536_at	Region	C730015A04Rik	277978	8	104585552	104591726	-
14055	1444150_at	Region	Epb4.1	269587	4	130721061	130868982	-
14056	1458781_at	Region	Kcnk13	217826	12	95402386	95500239	+
14057	1428530_x_at	Region	Rps24	20088	14	22859652	22865117	+
14058	1445521_at	Region	Elavl1	15568	8	3643294	3667872	-
14059	1440311_at	Region	Sorbs1	20411	19	39839082	40058071	-
14060	1415925_a_at	Region	Nup62	18226	7	38891347	38905734	+
14061	1447513_at	Region	None	None	3	105467086	105467443	+
14062	1443508_at	Region	Dlgap1	224997	17	68195196	68591524	+
14063	1426835_at	Region	Metap1	75624	3	137348195	137378617	-
14064	1423917_a_at	Region	Cttn	13043	7	138848729	138882584	-
14065	1450381_a_at	Region	Bcl6	12053	16	22748871	22772551	-
14066	1416862_at	Region	Stam	20844	2	14000384	14074610	+
14067	1446681_at	Region	BB086117	105710	14	30541274	30541905	-
14068	1434421_at	Region	B930052A04Rik	320563	9	58310393	58316229	-
14069	1431179_at	Region	Entpd7	93685	19	43234237	43276021	+
14070	1455738_at	Region	AI851076	237859	11	76769951	76804094	-
14071	1449418_s_at	Region	Fbxo36	66153	1	33563905	33564395	-
14072	1455827_at	Region	Mbnl2	105559	14	114846670	115003029	+
14073	1451347_at	Region	AI225782	233875	7	120902164	120911194	-
14074	1433721_x_at	Region	Rps21	66481	2	179974367	179975394	+
14075	1434173_s_at	Region	D19Bwg1357e	52874	19	26629949	26669888	-
14076	1432189_a_at	Region	Sox5	20678	6	144679075	145055646	-
14077	1448146_at	Region	Wwp2	66894	8	106734091	106856288	+
14078	1437976_x_at	Region	Rpl23a	268449	11	77906594	77909202	-
14079	1448806_at	Region	None	None	2	155232340	155275328	+
14080	1434438_at	Region	Samhd1	56045	2	156554498	156592191	-
14081	1426004_a_at	Region	Tgm2	21817	2	157573374	157603361	-
14082	1419068_at	Region	Rabgef1	56715	5	129357005	129384162	+
14083	1434131_at	Region	Rufy1	216724	11	50142144	50183952	-

14084	1455497_at	Region	Leng9	243813	7	3369041	3370730	-
14085	1433980_at	Region	Htatip	81601	19	5392208	5398819	-
14086	1436011_at	Region	Elmo2	140579	2	164744764	164783212	-
14087	1450659_at	Region	Rgs7	24012	1	174991921	175425099	-
14088	1440053_at	Region	9430093N24Rik	320924	18	66290942	66524129	-
14089	1459078_at	Region	Rbpms	19663	8	32589665	32735420	-
14090	1424748_at	Region	Galnt11	231050	5	23692442	23735467	+
14091	1422686_s_at	Region	Sec8l1	20336	6	33334773	34062752	+
14092	1437849_x_at	Region	Armcx2	67416	X	128349086	128353933	-
14093	1457313_at	Region	Ocrl	320634	X	42433252	42486664	+
14094	1436627_at	Region	D17Ertd663e	52042	17	11702064	11702504	-
14095	1451930_at	Region	None	None	17	60378565	60379950	-
14096	1435267_at	Region	A430108E01Rik	384382		59299527	59300427	+
14097	1449510_at	Region	Zfp467	68910	6	48570465	48578486	-
14098	1424036_at	Region	2610031L17Rik	68879	2	181318349	181372617	+
14099	1426468_at	Region	0610037L13Rik	74098	4	106848804	106856707	+
14100	1449137_at	Region	Pdha1	18597	X	153721624	153737741	-
14101	1428309_s_at	Region	Pdrg1	68559	2	152465826	152472319	-
14102	1450852_s_at	Region	F2r	14062	13	91791978	91808621	-
14103	1424399_at	Region	Uck1	22245	2	32187164	32192224	-
14104	1434220_at	Region	Nup98	269966	7	96225342	96231267	-
14105	1432538_a_at	Region	Rfc3	69263	5	120	8093	+
14106	1452302_at	Region	Arhgef10	234094	8	14307382	14378920	+
14107	1456643_at	Region	9230114K14Rik	414108	5	51001477	51008589	+
14108	1416119_at	Region	Txn1	22166	4	57886822	57899740	-
14109	1450082_s_at	Region	Etv5	104156	16	21153380	21211575	-
14110	1416607_at	Region	4931406C07Rik	70984	9	15120247	15138698	-
14111	1418383_at	Region	Apcdd1	494504	18	63040206	63182348	+
14112	1418142_at	Region	Kcnj8	16523	6	143408208	143414626	-
14113	1442135_at	Region	Gm237	211488	10	67598925	67602736	-
14114	1440364_a_at	Region	A230062G08Rik	231326	5	76120378	76149959	-
14115	1428148_s_at	Region	Coro7	78885	16	4297661	4351229	-
14116	1434804_at	Region	Sec15l2	75914	6	84969806	85419115	-
14117	1456614_at	Region	Acn9	71238	6	6928768	7012424	+
14118	1460405_at	Region	Arhgef10l	72754	4	139395746	139530011	-
14119	1457782_at	Region	Tln1	21894	4	43447441	43472672	-
14120	1455098_a_at	Region	Vtn	22370	11	78224945	78227983	+
14121	1451143_at	Region	1110006G06Rik	73833	7	24563921	24567592	-
14122	1442087_at	Region	None	None	1	180760213	180760526	-
14123	1416838_at	Region	Mut	17850	17	38451138	38475743	+
14124	1454092_a_at	Region	Gtf2h3	209357	5	123750514	123768184	+
14125	1424746_at	Region	Kif1c	16562	11	70428458	70454537	+
14126	1455734_at	Region	Crbn	58799	6	107274263	107296083	-
14127	1445421_at	Region	None	None	2	16686767	16687400	+
14128	1437716_x_at	Region	Kif22	110033	7	121077462	121092151	-
14129	1427199_at	Region	2510002A14Rik	72313	5	71804336	71855006	-
14130	1436156_at	Region	Ccar1	67500	10	62710847	62758641	-
14131	1439185_x_at	Region	D430028G21Rik	228607	2	130747991	130761878	+
14132	1426681_at	Region	Zc3h5	217331	11	115851415	115882287	+
14133	1460448_s_at	Region	Ttc14	67120	3	33243957	33251783	+
14134	1436077_a_at	Region	Fcho1	74015	8	70858707	70875819	-
14135	1456805_a_at	Region	C030010B13Rik	77310	2	173046327	173120005	-
14136	1451932_a_at	Region	Tsrc1	229595	3	95164036	95175690	-
14137	1450034_at	Region	Stat1	20846	1	52420399	52461905	+
14138	1440052_at	Region	Efna5	13640	17	60297708	60574961	-
14139	1415863_at	Region	Eif4g2	13690	7	104925069	104936167	-
14140	1434769_at	Region	Btbd9	224671	17	28030141	28340405	-
14141	1452650_at	Region	Trim62	67525	4	127911253	127938439	+
14142	1441783_at	Region	None	None	14	51335750	51336002	+
14143	1416971_at	Region	Cox7a2	12866	9	79961062	79965429	-
14144	1423722_at	Region	Tmem49	75909	11	86311857	86411814	-
14145	1421708_a_at	Region	Stat6	20852	10	127343744	127397113	+
14146	1439915_at	Region	Mrg1	17536	2	115376917	115578960	-
14147	1421789_s_at	Region	Arf3	11842	15	98795066	98820699	-
14148	1423210_a_at	Region	Nola3	66181	2	111885207	111886138	+

14149	1426864_a_at	Region	Ncam1	17967	9	49555319	49607941	-
14150	1447278_at	Region	BC027092	214552	9	45780203	45790044	-
14151	1434296_at	Region	LOC234413	234413	8	71124838	71128362	+
14152	1417923_at	Region	Pak3	18481	X	137105791	137231857	+
14153	1438303_at	Region	Tgfb2	21808	1	186121756	186210661	-
14154	1433480_at	Region	2900010J23Rik	72931	2	32210979	32220175	-
14155	1443282_at	Region	2410002M20Rik	230596	4	107523775	107538232	-
14156	1423841_at	Region	Bxdc2	67832	15	10290307	10300033	-
14157	1453510_s_at	Region	4930589M24Rik	75906	10	53878840	53940180	-
14158	1436404_at	Region	Rpl23a	268449	11	77906594	77909202	-
14159	1455015_at	Region	4933431N12Rik	71310	8	82412909	82519979	+
14160	1439029_at	Region	Gpt2	108682	8	84765391	84800332	+
14161	1454785_at	Region	Dusp11	72102	6	86372013	86391226	-
14162	1452035_at	Region	Col4a1	12826	8	10570852	10685155	-
14163	1443851_at	Region	8430415E04Rik	74521	12	98976507	99057691	+
14164	1415674_a_at	Region	Trappc4	60409	9	44392614	44396403	-
14165	1430500_s_at	Region	Mtx2	53375	2	74523600	74576180	+
14166	1450116_at	Region	D3Ertd300e	56790	3	54327325	54351057	+
14167	1422102_a_at	Region	Stat5b	20851	11	100601818	100643615	-
14168	1430170_at	Region	1300007O09Rik	71769	10	110921530	110929949	+
14169	1438737_at	Region	Zic3	22773	X	52785357	52790617	+
14170	1423773_at	Region	Gpbp	73274	13	107815217	107879367	-
14171	1455913_x_at	Region	Ttr	22139	18	20880341	20889247	+
14172	1416292_at	Region	Prdx3	11757	19	60464492	60474920	-
14173	1438674_a_at	Region	Sfrs8	231769	5	128676882	128691989	+
14174	1456399_at	Region	None	None	9	45869953	45871399	-
14175	1423704_at	Region	Lypla3	192654	8	105446246	105460562	+
14176	1450799_at	Region	Adcyap1r1	11517	6	55596313	55643230	+
14177	1450066_at	Region	Ubr1	22222	2	120375495	120484564	-
14178	1421751_a_at	Region	Psmd14	59029	2	61567169	61655776	+
14179	1416192_at	Region	Napa	108124	7	3964	14735	+
14180	1420922_at	Region	Usp9x	22284	X	11310477	11410726	+
14181	1443952_at	Region	Nr1d1	217166	11	98589019	98595909	-
14182	1430417_s_at	Region	0610025P10Rik	216860	11	69637165	69639477	+
14183	1440018_at	Region	A330043J11Rik	320261	3	107236170	107236784	-
14184	1438431_at	Region	Abcd2	26874	15	91198527	91244463	-
14185	1437252_at	Region	Gats	80909	5	133391036	133432162	+
14186	1445724_at	Region	Iqgap1	29875	7	74515481	74605994	-
14187	1445898_at	Region	Ggcx	56316	6	72746307	72762681	+
14188	1439078_at	Region	Klhl4	237010	X	108874005	108960183	+
14189	1450149_a_at	Region	Ppp1cc /// LOC434233	19047	5	121310629	121327619	+
14190	1434037_s_at	Region	Pcaf	18519	17	51101486	51203822	+
14191	1439680_at	Region	Tnfsf10	22035	3	26734898	26753894	+
14192	1460116_s_at	Region	Spred1	114715	2	116635298	116693185	+
14193	1434414_at	Region	D15Bwg0759e	239554	15	77992479	77994068	-
14194	1416451_s_at	Region	Tbn	63856	17	45006510	45020747	-
14195	1424056_at	Region	Usp48	170707	4	136475016	136537437	+
14196	1448127_at	Region	Rrm1	20133	7	96546225	96573284	+
14197	1429741_at	Region	Kcnv1	67498	15	45052998	45061646	-
14198	1426531_at	Region	Zmynd11	66505	13	9669934	9749340	-
14199	1434084_at	Region	5730601F06Rik	77519	9	20365696	20393979	-
14200	1444670_at	Region	A130004G11Rik	319715	1	179191377	179192508	-
14201	1433537_at	Region	4833408C14Rik	67403	X	100398762	100399671	-
14202	1438335_at	Region	6030413G23Rik	210004	11	121437863	121494232	-
14203	1416257_at	Region	Capn2	12334	1	182426221	182476485	-
14204	1451864_at	Region	Cacng8	81905	7	0	20907	-
14205	1434253_s_at	Region	Tmcc3	319880	10	94549417	94562436	+
14206	1437182_at	Region	Dido1	23856	2	180385246	180426957	-
14207	1428339_at	Region	Cpsf5	68219	8	93303578	93321193	-
14208	1429270_a_at	Region	1700013H19Rik	71846	8	84139227	84154563	+
14209	1424733_at	Region	P2ry14	140795	3	58764614	58780613	-
14210	1456019_at	Region	Cwf19l2	244672	9	3307769	3382920	+
14211	1438974_x_at	Region	Pitpnm1	18739	19	3889412	3902751	+
14212	1448684_at	Region	Ppp1r2	66849	16	30062505	30084499	-

14213	1435300_at	Region	None	None	5	32228550	32229018	-
14214	1417740_at	Region	Cdc37l1	67072	19	28243091	28270166	+
14215	1418281_at	Region	Rad51	19361	2	118626666	118649919	+
14216	1439570_at	Region	Gm444	242915	5	28564942	28575812	+
14217	1423334_at	Region	1200007D18Rik	67458	17	24356346	24451768	+
14218	1426798_a_at	Region	None	None	1	132992804	133001249	+
14219	1423331_a_at	Region	Pvrl3	58998	16	45281022	45382981	-
14220	1454141_at	Region	5730416O20Rik	70557	10	69983928	69985175	+
14221	1454826_at	Region	Zbtb11	271377	16	54903180	54904843	+
14222	1420673_a_at	Region	Acox2	93732	14	5892491	5925790	-
14223	1433784_at	Region	AI265322	106543	NONE	NONE	NONE	NONE
14224	1451440_at	Region	Chodl	246048	16	78016595	78037203	+
14225	1431181_a_at	Region	Luc7l	66978	17	24046121	24075138	+
14226	1437235_x_at	Region	Lpp	210126	16	23179019	23766559	+
14227	1418137_at	Region	Mrp63	67840	14	52361111	52363617	+
14228	1454635_at	Region	Fbxl3	50789	14	97626484	97644968	-
14229	1431136_at	Region	Rab36	76877	10	75138289	75155300	+
14230	1424020_at	Region	Arl6ip6	65103	2	53124273	53151188	+
14231	1459042_at	Region	Rims3	242662	4	119900313	119914004	+
14232	1421189_at	Region	Gabrb3	14402	7	51863803	52099137	+
14233	1415719_s_at	Region	Armc1	74252	3	18469495	18500416	-
14234	1449200_at	Region	None	None	15	7896839	7947428	+
14235	1417542_at	Region	Rps6ka2	20112	17	6670716	6803866	+
14236	1421888_x_at	Region	Aplp2	11804	9	31068390	31130767	-
14237	1436301_at	Region	Ripk5	213452	1	132274936	132320783	+
14238	1453306_at	Region	6330531I01Rik	76173	14	72163103	72164855	-
14239	1434841_at	Region	None	None	1	63340819	63341444	-
14240	1438214_at	Region	Trps1	83925	15	50659276	50888038	-
14241	1426953_at	Region	Hmgb2l1	70823	8	74157765	74196034	+
14242	1419543_a_at	Region	Sfrs10	20462	16	21019108	21039299	-
14243	1458282_at	Region	Cdc27	217232	11	104326687	104371504	-
14244	1422737_at	Region	Ncoa3	17979	2	165449481	165527179	+
14245	1434492_at	Region	A130022J15Rik	101351	6	97577445	97615221	-
14246	1427720_a_at	Region	Nnp1	18114	10	78510762	78523371	-
14247	1417724_at	Region	Thoc4	21681	11	120415609	120419458	-
14248	1433589_at	Region	D6Wsu116e	28006	6	116645592	116700200	+
14249	1437635_at	Region	Dcbld2	73379	16	57413972	57455222	+
14250	1427143_at	Region	Jarid1b	75605	1	134410458	134483140	+
14251	1439821_at	Region	Lrp2bp	67620	8	44951547	44968583	+
14252	1435035_at	Region	Rg9mtd2	108943	3	137032375	137047205	+
14253	1428776_at	Region	Slc10a6	75750	5	102640503	102664195	-
14254	1426635_at	Region	Acbd3	170760	1	180679954	180707985	+
14255	1437250_at	Region	MGI:2151839	381269	1	72455562	72508424	-
14256	1436049_at	Region	Mettl3	56335	14	47394773	47405188	-
14257	1436317_at	Region	D230012E17Rik	241062	1	54781619	54783409	-
14258	1451090_a_at	Region	Eif2s3x	26905	X	88849367	88873309	-
14259	1447919_x_at	Region	Ndufab1	70316	7	115965902	115979706	-
14260	1457528_at	Region	Slc4a7	218756	14	13169299	13264198	+
14261	1424111_at	Region	Igf2r	16004	17	11321447	11408701	-
14262	1457550_at	Region	9530059O14Rik	319626	9	122597941	122598569	+
14263	1453169_a_at	Region	Gtf2h1	14884	7	40881451	40909149	+
14264	1439375_x_at	Region	Aldoa	11674	7	120844967	120848914	-
14265	1428284_at	Region	8430427H17Rik	329540	2	152864402	152940719	-
14266	1458113_at	Region	9530019H20Rik	320999	8	67685224	67685899	-
14267	1457800_at	Region	2900019G14Rik	72932	2	102490151	102490770	-
14268	1455877_a_at	Region	Nanos1	332397	19	60357055	60358437	+
14269	1416088_a_at	Region	Rps15	20054	10	80417366	80419021	+
14270	1418974_at	Region	Blzf1	66352	1	164208297	164225981	-
14271	1435445_at	Region	Ccnt2	72949	1	127616552	127647225	+
14272	1418424_at	Region	Tnfaip6	21930	2	51970275	51988843	+
14273	1417499_at	Region	Timm13a	30059	2	84524792	84527962	+
14274	1439239_at	Region	Lin7b	22342	7	39443916	39446815	-
14275	1428320_at	Region	Jmjd1b	277250	18	35000710	35062653	+
14276	1447481_at	Region	2900045N06Rik	72895	6	113640716	113716498	+
14277	1429148_at	Region	1110019L22Rik	68530	10	81531549	81532573	-

14278	1456731_x_at	Region	Polr3k	67005	2	181581407	181587121	+
14279	1416925_at	Region	Kpnb1	16211	11	96980801	97008707	-
14280	1440505_at	Region	A330045H12Rik	403178	5	109150217	109154339	+
14281	1455663_at	Region	Olfml1	244198	7	101517446	101541094	+
14282	1452201_at	Region	2310047B19Rik	66962	9	21845743	21848244	+
14283	1433593_at	Region	Ypel5	383295	17	70612621	70627590	+
14284	1448896_at	Region	Pigf	18701	17	84854063	84882205	-
14285	1450894_a_at	Region	Ap2m1	11773	16	19307645	19316194	+
14286	1449183_at	Region	Comt	12846	16	17178593	17197565	-
14287	1439807_at	Region	B230382K22Rik	239408	15	43812307	43815641	-
14288	1433778_at	Region	D130072O21Rik	102143	8	33625530	33628811	-
14289	1455181_at	Region	Rasa2	114713	9	96441803	96530851	-
14290	1441282_at	Region	1500011L16Rik	68991	4	154197486	154226144	+
14291	1451580_a_at	Region	Ttr	22139	18	20880341	20889247	+
14292	1460564_at	Region	Suhw2	64453	10	76134454	76144703	+
14293	1420557_at	Region	Epha5	13839	5	83317214	83675897	-
14294	1434483_at	Region	Usp12	22217	5	145626505	145686649	-
14295	1460200_s_at	Region	Lztfl1	93730	9	123721311	123741507	-
14296	1427270_a_at	Region	Bsdc1	100383	4	128488792	128515548	+
14297	1445326_at	Region	Sdk1	330222	5	140862361	141218991	+
14298	1440347_at	Region	Arhgap10	78514	8	76458897	76659183	-
14299	1456790_at	Region	AA407452	57867	6	28286926	28308906	-
14300	1449482_at	Region	Hist3h2ba	78303	11	58674540	58675161	+
14301	1457409_at	Region	Fut9	14348	4	25753460	25935314	-
14302	1416079_a_at	Region	Arpc1a	56443	5	144122295	144147182	+
14303	1450354_a_at	Region	Ptdss2	27388	7	135533273	135558141	+
14304	1456029_a_at	Region	Ttc7b	104718	12	95744448	95966178	-
14305	1443214_at	Region	Gpr155	68526	2	73039256	73084321	-
14306	1416269_at	Region	Atp5j2	57423	5	144222142	144230028	-
14307	1448327_at	Region	Actn2	11472	13	12307753	12379584	-
14308	1436937_at	Region	Rbms3	207181	9	116564518	117239065	-
14309	1454633_at	Region	Etnk1	75320	6	144013618	144053017	+
14310	1449411_at	Region	Dscam	13508	16	96075147	96653164	-
14311	1456259_at	Region	None	None	8	67977291	67978360	+
14312	1455882_x_at	Region	A930041G11Rik	319922	11	11009536	11162515	+
14313	1419260_a_at	Region	Snrpb	20638	2	129685499	129693214	-
14314	1442166_at	Region	Cpne5	240058	17	26965417	27049591	-
14315	1435332_at	Region	None	None	19	35301088	35302196	-
14316	1422685_at	Region	Sec8l1	20336	6	33334773	34062752	+
14317	1416094_at	Region	Adam9	11502	8	23672252	23739899	-
14318	1428087_at	Region	Dnm1l	74006	16	15081722	15126600	-
14319	1431319_at	Region	Ulk4	74372	9	121153720	121321311	-
14320	1434317_s_at	Region	Tex10	269536	4	48348434	48389381	-
14321	1431930_x_at	Region	0610009I22Rik	66586	2	132361587	132380004	+
14322	1430640_a_at	Region	Prkar2b	19088	12	28534800	28637055	-
14323	1416222_at	Region	Nsdhl	18194	X	67579176	67619183	+
14324	1417313_at	Region	Lsm7	66094	10	80984897	80987275	-
14325	1459958_at	Region	1200013F24Rik	66880	3	66658030	67030708	+
14326	1416560_at	Region	Slc13a3	114644	2	164862028	164929930	-
14327	1425457_a_at	Region	Grb10	14783	11	11828255	11865408	-
14328	1436538_at	Region	Lrp2bp	67620	8	44951547	44968583	+
14329	1440885_at	Region	Evl	14026	12	104025433	104159229	+
14330	1457956_at	Region	AW987475	100468	4	150272934	150273332	-
14331	1426514_at	Region	4631426J05Rik	77590	7	126599419	126683081	-
14332	1436487_x_at	Region	None	None	2	34739463	34739840	+
14333	1456858_at	Region	Gpr149	229357	3	62196472	62270897	-
14334	1455582_at	Region	C1qtnf1	56745	11	118254946	118271056	+
14335	1418780_at	Region	Cyp39a1	56050	17	41168941	41253050	+
14336	1426581_at	Region	2810004N20Rik	66461	2	90615475	90622778	-
14337	1436833_x_at	Region	Ttll1	319953	15	83532371	83559476	-
14338	1460133_at	Region	Efna5	13640	17	60297708	60574961	-
14339	1436296_x_at	Region	None	None	11	114578596	114578983	+
14340	1448403_at	Region	Lars	107045	18	42422980	42482702	-
14341	1422400_a_at	Region	Hemt1	15202	15	74845013	74846249	-
14342	1423804_a_at	Region	Idi1	319554	13	8843118	8849517	+

14343	1437718_x_at	Region	Fmod	14264	1	133888996	133898985	+
14344	1452898_at	Region	2210415M20Rik	70160	8	20945031	20970768	+
14345	1426927_at	Region	Ap3b2	11775	7	75268542	75301907	-
14346	1449407_at	Region	Cdv1	12589	5	121702852	121766929	-
14347	1434740_at	Region	Scarf2	224024	16	16568250	16579250	+
14348	1452758_s_at	Region	Eif4g2	13690	7	104925069	104936167	-
14349	1424724_a_at	Region	D16Ertd472e	67102	16	77629000	77662117	-
14350	1435003_at	Region	Pik4ca	224020	16	16051320	16177282	-
14351	1436025_at	Region	A430106J12Rik	108686	11	29375382	29404231	+
14352	1449893_a_at	Region	Lrig1	16206	6	95066417	95161798	-
14353	1418326_at	Region	Slc7a5	20539	8	121262737	121289261	-
14354	1435414_s_at	Region	Dctn1	13191	6	83526302	83543690	+
14355	1428422_at	Region	2210404D11Rik	76577	13	53231404	53273668	+
14356	1428917_at	Region	Stx17	67727	4	48040852	48099039	+
14357	1458891_at	Region	Garnl3	99326	2	32918528	33019366	-
14358	1419581_at	Region	Dlgh4	13385	11	69744522	69770426	+
14359	1448126_at	Region	MGI:1929091	56306	6	149856949	149947621	-
14360	1427539_a_at	Region	Zwint	52696	10	72709781	72729831	+
14361	1427822_a_at	Region	Copg2as2	54158	NONE	NONE	NONE	NONE
14362	1428082_at	Region	Acsl5	433256	19	54834176	54876865	+
14363	1438805_at	Region	9230106B05Rik	77709	17	45023700	45097756	+
14364	1450714_at	Region	Azin1	54375	15	38492445	38524417	-
14365	1424344_s_at	Region	Eif1a	13664	18	46819460	46831964	+
14366	1447480_at	Region	4931432E15Rik	70991	14	10487761	10488143	+
14367	1444673_at	Region	Dmtf1	23857	5	9125544	9168200	-
14368	1460701_a_at	Region	Mrpl52	68836	14	48943738	48946676	+
14369	1453049_at	Region	6620401M08Rik	75744	7	46166743	46175690	-
14370	1429938_at	Region	A930009E05Rik	77796	6	40395460	40396727	+
14371	1416168_at	Region	Serpinf1	20317	11	75135427	75148170	-
14372	1438385_s_at	Region	Gpt2	108682	8	84765391	84800332	+
14373	1433824_x_at	Region	Grsf1	231413	5	87925715	87942166	-
14374	1434960_at	Region	BC066223	407786	X	100808239	100820998	-
14375	1428000_at	Region	BC027828	212090	5	19338791	19339440	+
14376	1448753_at	Region	Srp9	27058	1	182083607	182091245	+
14377	1442945_at	Region	2010109K09Rik	70267	2	68822157	68823023	+
14378	1417292_at	Region	Ifi47	15953	11	48839896	48849812	+
14379	1427197_at	Region	Atr	245000	9	95844413	95855187	+
14380	1454992_at	Region	Slc7a1	11987	5	147223681	147291421	-
14381	1456225_x_at	Region	Trib3	228775	2	151794358	151800968	-
14382	1449668_s_at	Region	A730024A03Rik	216742	11	54191030	54271077	+
14383	1450054_at	Region	Add1	11518	5	33061487	33119289	+
14384	1459978_x_at	Region	Gm877	380694	11	43281564	43346257	+
14385	1428891_at	Region	9130213B05Rik	231440	5	90839183	90945494	+
14386	1449928_at	Region	Tcte1l	67117	X	7893210	7901923	-
14387	1433529_at	Region	E430002G05Rik	210622	2	102254804	102347791	+
14388	1427230_at	Region	B930041F14Rik	230991	4	154186704	154188843	+
14389	1424671_at	Region	Plekhf1	72287	7	33388336	33389823	-
14390	1430753_at	Region	8030491N06Rik	77505	7	99765659	99836451	+
14391	1438535_at	Region	Phip	83946	9	83198955	83305205	-
14392	1448350_at	Region	Asl	109900	5	129181328	129194156	-
14393	1423179_at	Region	Kcnb1	16500	2	166560111	166645532	-
14394	1434897_a_at	Region	Slc25a4	11739	8	45151043	45155041	-
14395	1431655_a_at	Region	Mulk	69923	6	40467075	40538342	+
14396	1418808_at	Region	Rdh5	19682	10	128650678	128656363	-
14397	1444946_at	Region	None	None	5	72473697	72474608	+
14398	1437002_at	Region	C030011O14Rik	215708	3	151257333	151321808	-
14399	1429771_at	Region	3110073H01Rik	73201	15	9001580	9004336	+
14400	1430774_at	Region	A430106A12Rik	68164	NONE	NONE	NONE	NONE
14401	1424377_at	Region	BC003885	225215	9	73246095	73255959	+
14402	1432808_at	Region	5830426K05Rik	76053	NONE	NONE	NONE	NONE
14403	1428430_at	Region	Rgmb	68799	17	13809790	13830123	-
14404	1418393_a_at	Region	Itga7	16404	10	128670908	128695372	+
14405	1455227_at	Region	Aadacl1	320024	3	26600437	26662708	+
14406	1434799_x_at	Region	Aldoa	11674	7	120844967	120848914	-
14407	1422216_at	Region	Mid2	23947	X	134209895	134312655	+

14408	1426742_at	Region	Atp5f1	11950	3	105738523	105755085	-
14409	1421297_a_at	Region	Cacna1c	12288	6	119025051	119540819	-
14410	1437782_at	Region	Cntnap2	66797	6	45854973	47502306	+
14411	1421318_at	Region	Ndst4	64580	3	124319686	124608030	+
14412	1428147_at	Region	Coro7	78885	16	4297661	4351229	-
14413	1435974_at	Region	Arhgef9	236915	X	89650305	89797863	-
14414	1423059_at	Region	Ptk2	14083	15	73233486	73423105	-
14415	1424552_at	Region	Casp8	12370	1	59100364	59151883	+
14416	1427935_at	Region	2610208E05Rik	108755	4	33079058	33081043	+
14417	1430394_a_at	Region	Abcb9	56325	5	123233182	123267116	-
14418	1446736_at	Region	Tbl1x	21372	X	72294199	72320920	+
14419	1433706_a_at	Region	AW742319	57874	9	65109184	65143902	-
14420	1428195_at	Region	4631427C17Rik	74340	6	29815630	29959406	+
14421	1422196_at	Region	Htr5b	15564	1	121272965	121291672	-
14422	1439553_s_at	Region	Nutf2	68051	8	105156479	105176249	+
14423	1456656_at	Region	Lin7a	108030	10	107159996	107307910	+
14424	1435657_at	Region	AI425999	104804	12	87048794	87049917	-
14425	1421914_s_at	Region	Mrpl19	56284	6	82245436	82301881	-
14426	1428902_at	Region	1110020P09Rik	68647	10	83082084	83083705	+
14427	1452062_at	Region	Prpsap2	212627	11	61455296	61487704	-
14428	1427990_at	Region	Usp45	77593	4	21845670	21907243	+
14429	1425933_a_at	Region	Nt5c2	76952	19	46438257	46513006	-
14430	1420161_at	Region	AA409749	103086	10	117479507	117479843	-
14431	1438409_at	Region	D9Mgc48e	28135	9	102536385	102575474	-
14432	1423517_at	Region	Cct6a	12466	5	128963793	128970908	+
14433	1422919_at	Region	Hrasls	27281	16	27998437	28019272	+
14434	1451357_at	Region	E130307M08Rik	68047	17	53647459	53654796	+
14435	1421468_at	Region	Kcnj3	16519	2	55362695	55521064	+
14436	1448677_at	Region	Noc4	18117	8	120015179	120025455	-
14437	1435171_at	Region	7530428D23Rik	319506	3	21417311	21418271	-
14438	1448546_at	Region	Rassf3	192678	10	121066604	121132504	-
14439	1452049_at	Region	Rpl7l1	66229	17	44284905	44294348	-
14440	1428631_a_at	Region	Uqcrc2	67003	7	114510774	114534837	+
14441	1447863_s_at	Region	Nr4a2	18227	2	57033695	57040794	-
14442	1460174_at	Region	Dexi	58239	16	10204712	10217539	-
14443	1418841_s_at	Region	Cdc2l1	12537	4	154117231	154142294	+
14444	1425421_at	Region	Rbbp6	19647	7	116848797	116880431	+
14445	1453742_at	Region	Vps33a	77573	5	122700612	122742738	-
14446	1421299_a_at	Region	Lef1	16842	3	130040399	130153310	+
14447	1420831_at	Region	Qscn6	104009	1	155674165	155708148	-
14448	1460568_at	Region	Trim46	360213	3	88990651	89002306	-
14449	1453581_at	Region	4933426L22Rik	71098	1	176666372	176710588	-
14450	1428629_at	Region	Zfp518	72672	19	40439085	40461071	+
14451	1421230_a_at	Region	Msi2h	76626	11	88067452	88208048	-
14452	1448868_at	Region	Scand1	19018	2	155768795	155769463	-
14453	1416668_at	Region	4921531G14Rik	66736	15	43423077	43473625	+
14454	1456956_at	Region	Zfpn1a2	22779	1	69831529	69979960	-
14455	1454197_a_at	Region	D19Ertd678e	108673	19	10139228	10145763	-
14456	1455923_at	Region	Kctd8	243043	5	67878286	68110392	-
14457	1437110_at	Region	2810474O19Rik	67246	6	24755596	24758726	+
14458	1437247_at	Region	Fosl2	14284	5	30595472	30616778	+
14459	1449268_at	Region	Gfpt1	14583	6	87476967	87522715	+
14460	1448378_at	Region	Fscn1	14086	5	142024679	142037166	+
14461	1416912_at	Region	6330407G11Rik	68050	4	122762469	122777457	-
14462	1444543_at	Region	2810405F15Rik	69974	2	115588468	115589010	+
14463	1425503_at	Region	Gcnt2	14538	13	40420765	40521482	+
14464	1429749_at	Region	9330180L21Rik	77268	14	28953089	28954172	+
14465	1417821_at	Region	D17H6S56E-5	110956	17	32700504	32704526	-
14466	1434026_at	Region	Atp8b2	54667	3	89745559	89769413	-
14467	1428462_at	Region	Ppp2r5e	26932	12	72307243	72452562	-
14468	1417144_at	Region	Tubg1	103733	11	100941218	100947506	+
14469	1417859_at	Region	Gas7	14457	11	67258648	67410609	+
14470	1437671_x_at	Region	Prss23	76453	7	83485290	83495057	-
14471	1439502_at	Region	4930573I19Rik	104859	12	106370708	106447662	+
14472	1440217_at	Region	LOC434197	434197	7	62246529	62247669	+

14473	1421771_a_at	Region	Ipp	16351	4	115488576	115497148	+
14474	1424936_a_at	Region	Dnahc8	13417	17	28434884	28686369	+
14475	1452045_at	Region	Zfp281	226442	1	136473723	136477067	+
14476	1455333_at	Region	Tns3	319939	11	8329286	8444531	-
14477	1435910_at	Region	Fads3	60527	19	9238187	9256168	+
14478	1436676_at	Region	Mapk8ip3	30957	17	22703470	22742877	-
14479	1428428_at	Region	Wbscr21	68758	5	134022791	134025662	+
14480	1442041_at	Region	LOC552876	552876	15	26696645	26697321	-
14481	1430886_at	Region	1700112E06Rik	76633	14	20942702	20964243	+
14482	1454023_a_at	Region	D1Bwg1363e	74241	1	75765292	75770124	-
14483	1452200_at	Region	D11Ertd497e	52626	11	51720504	51730177	+
14484	1460330_at	Region	Anxa3	11745	5	95811511	95864064	+
14485	1436888_at	Region	Nhlh2	18072	3	101435641	101439992	+
14486	1440647_at	Region	Sipa1l1	217692	12	79172130	79311045	+
14487	1416160_at	Region	Nr2f2	11819	7	64237865	64245019	-
14488	1434459_at	Region	BC057627	330474	7	13350629	13387074	+
14489	1433300_at	Region	2900005I04Rik	72861	X	82341373	82342486	-
14490	1418068_at	Region	Ndufa10	67273	1	92267199	92301249	-
14491	1447844_at	Region	None	None	NONE	NONE	NONE	NONE
14492	1428789_at	Region	Ralgps2	78255	1	156713690	156845148	-
14493	1416923_a_at	Region	Bnip3l	12177	14	61515922	61539638	-
14494	1416112_at	Region	Cox8a	12868	19	6929007	6931380	-
14495	1454812_at	Region	5730601F06Rik	77519	9	20365696	20393979	-
14496	1455279_at	Region	Gm1060	381738	5	28799630	28824879	+
14497	1450927_at	Region	Lztr1	66863	16	16279939	16297298	+
14498	1424706_at	Region	Zfp51	22709	17	19319829	19335088	+
14499	1434086_at	Region	AI790205	277463	2	31084496	31148729	+
14500	1436809_a_at	Region	Spin	20729	13	67875489	67920302	-
14501	1428342_at	Region	Rcor3	214742	1	191839449	191876966	-
14502	1425264_s_at	Region	Mbp	17196	18	82642914	82753380	+
14503	1443486_at	Region	Cog7	233824	NONE	NONE	NONE	NONE
14504	1457392_at	Region	Sfrs12	218543	13	99959715	99982622	-
14505	1442049_at	Region	Trf	22041	9	103184317	103205727	-
14506	1428953_at	Region	Za20d1	229603	3	95645016	95646747	+
14507	1426452_a_at	Region	Rsb30	75985	7	86781719	86878103	+
14508	1435362_at	Region	Foxj3	230700	4	118498593	118588024	+
14509	1419449_a_at	Region	Gnai2	14678	9	107680276	107701110	-
14510	1419090_x_at	Region	Klk26	16618	7	38087008	38091291	+
14511	1417726_at	Region	Sssca1	56390	19	5516878	5520503	-
14512	1426066_a_at	Region	Dtna	13527	18	23643946	23881842	+
14513	1426654_at	Region	Zc3hc1	232679	6	30413475	30438102	-
14514	1434407_at	Region	Srgap2	14270	1	131135970	131377678	-
14515	1437907_a_at	Region	Tbca	21371	13	90973009	91026965	+
14516	1419665_a_at	Region	Nupr1	56312	7	120672977	120675201	-
14517	1417240_at	Region	Zyx	22793	6	42494136	42502274	+
14518	1451232_at	Region	Cd151	12476	7	135869384	135873464	+
14519	1437135_at	Region	Fbxw10	213980	11	62602119	62603115	+
14520	1450379_at	Region	Msn	17698	X	90743247	90768662	+
14521	1421076_at	Region	Sertad3	170742	7	22849842	22853366	+
14522	1417934_at	Region	Dnajc4	57431	19	6701759	6706120	-
14523	1436069_at	Region	None	None	1	93650975	93652322	+
14524	1430536_a_at	Region	Erh	13877	12	77495292	77505045	-
14525	1458201_at	Region	Tcf4	21413	18	69575005	69915006	+
14526	1456448_at	Region	2610301B20Rik	67157	4	10801573	10826390	+
14527	1423955_a_at	Region	Lass2	76893	3	94803083	94811401	+
14528	1427289_at	Region	Ddhd1	114874	14	40672980	40701448	-
14529	1419341_at	Region	Epha8	13842	4	135810676	135838075	-
14530	1416864_at	Region	Surf6	20935	2	26822934	26834975	-
14531	1427482_a_at	Region	Car8	12319	4	8072625	8166127	-
14532	1432488_a_at	Region	Sf3a3	75062	4	123741889	123759573	+
14533	1433547_s_at	Region	Nudcd1	67429	15	44320855	44373512	-
14534	1443750_s_at	Region	Rpp40	208366	13	35428773	35441652	-
14535	1438171_x_at	Region	0610012D09Rik	59052	7	114910012	114952402	+
14536	1457361_at	Region	C630007C17Rik	241514	2	81932610	81957628	+
14537	1443926_at	Region	Mrg1	17536	2	115376917	115578960	-

14538	1448363_at	Region	Yap1	22601	9	7937967	8010552	-
14539	1426595_at	Region	Slc18a1	110877	8	68186560	68237686	-
14540	1418286_a_at	Region	Efnb1	13641	X	93737491	93750352	+
14541	1456604_a_at	Region	Pcmt1	18537	10	7494777	7527640	-
14542	1417161_at	Region	Cdk2ap2	52004	19	3886141	3887807	+
14543	1417715_a_at	Region	Got2	14719	8	95145470	95169698	-
14544	1448323_a_at	Region	Bgn	12111	X	68144299	68156586	+
14545	1448712_at	Region	Chm	12662	X	107440914	107585000	-
14546	1450900_at	Region	AW011752	104570	11	29067786	29113926	+
14547	1442006_at	Region	None	None	16	19304995	19305915	+
14548	1448914_a_at	Region	Csf1	12977	3	107536887	107556052	-
14549	1417983_a_at	Region	Ube2v2	70620	16	14321751	14363568	-
14550	1434140_at	Region	Mcf2l	17207	8	12294593	12399137	+
14551	1438801_at	Region	Dnm3	103967	1	161896669	162387092	-
14552	1431304_a_at	Region	1300007B12Rik	57439	1	134195640	134212218	-
14553	1428215_x_at	Region	Tomm7	66169	5	22303551	22308004	-
14554	1448164_at	Region	Klhdc3	71765	17	44185287	44191618	-
14555	1426910_at	Region	None	None	10	108219386	108301687	+
14556	1426359_at	Region	Zc3h11a	70579	1	133482838	133516350	-
14557	1424073_at	Region	5430437P03Rik	68251	8	70546548	70554224	+
14558	1434470_at	Region	Syt13	80976	2	92619784	92660734	+
14559	1457066_at	Region	Abcc8	20927	7	40193432	40201423	-
14560	1433811_at	Region	Mllt6	246198	11	97484501	97503617	+
14561	1439192_at	Region	Nova2	384569	7	15829586	15830872	+
14562	1425068_a_at	Region	Tex264	21767	9	106724534	106751448	-
14563	1435340_at	Region	Jmjd2a	230674	4	117100948	117138851	-
14564	1452769_at	Region	3732413I11Rik	74315	11	44271801	44318353	+
14565	1416200_at	Region	9230117N10Rik	77125	19	29202255	29213323	+
14566	1444274_at	Region	None	None	6	91225295	91226236	-
14567	1418504_at	Region	Hspa9a	15526	18	35161089	35177981	-
14568	1449734_s_at	Region	Bbs4	102774	9	59438725	59470219	-
14569	1420816_at	Region	Ywhag	22628	5	134921901	134948016	-
14570	1431482_at	Region	2900012M01Rik	72914	9	10812976	10814408	-
14571	1443671_x_at	Region	2010001J22Rik	70113	15	89430323	89430747	-
14572	1438037_at	Region	None	None	6	57820445	57827507	+
14573	1451463_at	Region	Arhgap8	109270	15	84729601	84752271	+
14574	1426357_at	Region	Taok1	216965	11	77255718	77311108	-
14575	1442508_at	Region	LOC192758 /// LOC216974	192758	11	77920367	77931422	+
14576	1427261_at	Region	BC037006	211652	11	35632430	35774550	-
14577	1417024_at	Region	Hars	15115	18	36990295	37006885	-
14578	1426063_a_at	Region	Gem	14579	4	11631422	11641717	+
14579	1449056_at	Region	E330009J07Rik	243780	6	40549078	40577713	-
14580	1437765_at	Region	None	None	19	36364552	36365194	-
14581	1451795_at	Region	Tom1l2	216810	11	59955659	60078526	-
14582	1435542_s_at	Region	BC003236	80281	3	104796431	104845993	-
14583	1416988_at	Region	Msh2	17685	17	85528737	85580669	+
14584	1453474_at	Region	1300007F04Rik	67477	11	77240780	77245704	+
14585	1455326_at	Region	4932416N17Rik	74374	16	10046792	10048449	-
14586	1437131_x_at	Region	Mrpl11	66419	19	4751105	4753733	+
14587	1456063_at	Region	None	None	X	145007406	145008534	+
14588	1444774_at	Region	Det1	76375	7	72619581	72639318	-
14589	1427006_at	Region	Rapgef1	107746	2	29551925	29670492	+
14590	1433993_at	Region	4931406P16Rik	233103	7	5289	54184	-
14591	1420543_at	Region	ORF28	246738	16	90772890	90776157	-
14592	1456867_x_at	Region	Sdbcag84	66366	2	155465054	155475216	+
14593	1436817_at	Region	None	None	14	44100274	44101615	-
14594	1430292_a_at	Region	1810030N24Rik	66291	4	34907739	34917404	-
14595	1448335_s_at	Region	Ccni	12453	5	92517687	92541778	-
14596	1448228_at	Region	Lox	16948	18	52731473	52745109	-
14597	1421023_at	Region	Pik3c2a	18704	7	110195247	110273738	-
14598	1442067_at	Region	Ror1	26563	4	99054696	99401450	+
14599	1448180_a_at	Region	Hn1	15374	11	115318446	115335463	-
14600	1424350_s_at	Region	Lpgat1	226856	1	191453775	191513698	+
14601	1425837_a_at	Region	Ccrn4l	12457	3	50820528	50883543	+

14602	1420745_a_at	Region	Ccndbp1	17151	2	120522360	120530740	+
14603	1460476_s_at	Region	1200015N20Rik	71721	10	70495074	70612698	+
14604	1429905_at	Region	3110009O07Rik	73077	1	138673401	138674811	-
14605	1447943_x_at	Region	Yeats2	208146	16	18922076	19003907	+
14606	1417693_a_at	Region	Gab1	14388	8	79976442	80092487	-
14607	1424407_s_at	Region	Cbx6	494448	15	79878420	79886557	-
14608	1430494_at	Region	9130001I21Rik	71608	11	5616292	5617664	-
14609	1428576_at	Region	Hif1an	319594	19	44107275	44174420	+
14610	1452715_at	Region	2310022K01Rik	71909	7	26066403	26077663	-
14611	1421633_a_at	Region	Hapln1	12950	13	85655519	85726506	+
14612	1417254_at	Region	Spata5	57815	3	36883896	37042689	+
14613	1417400_at	Region	Rai14	75646	15	10381371	10524496	-
14614	1455398_at	Region	Lrrc8c	100604	5	104581541	104671026	+
14615	1433876_at	Region	6430402H13Rik	378937	15	76766654	76773552	-
14616	1454875_a_at	Region	Rbbp4	19646	4	128334213	128362483	-
14617	1446510_at	Region	Phip	83946	9	83198955	83305205	-
14618	1421342_at	Region	Kcns2	16539	15	34837430	34842137	+
14619	1436093_at	Region	None	None	4	123944356	123944915	+
14620	1430232_at	Region	4933407E14Rik	71017	15	76085454	76086571	+
14621	1421368_at	Region	Scrt1	170729	15	76566591	76572559	-
14622	1444449_at	Region	None	None	6	121704215	121704688	-
14623	1418143_at	Region	Vps45	22365	3	95487692	95546287	-
14624	1426168_a_at	Region	Tcra /// A430107P09Rik /// LOC545051 /// LOC547333	21473	14	48285053	48742963	+
14625	1422732_at	Region	Poldip2	67811	11	78237955	78248395	+
14626	1418532_at	Region	Fzd2	57265	11	102425622	102427671	+
14627	1454622_at	Region	Slc38a5	209837	X	6510334	6519114	+
14628	1455226_at	Region	LOC383567	383567	12	73437073	73437895	-
14629	1451794_at	Region	Tmcc3	319880	10	94549417	94562436	+
14630	1452405_x_at	Region	Tcra /// A430107P09Rik /// LOC545051	21473	14	48197821	48744068	+
14631	1448224_at	Region	Tfam	21780	10	71279444	71292011	-
14632	1422902_s_at	Region	Mgea5	76055	19	45297695	45330727	-
14633	1452287_at	Region	Msi1h	17690	5	114542007	114567198	+
14634	1421139_a_at	Region	Zfp386	56220	12	111816578	111828697	+
14635	1448555_at	Region	D15Ertd682e	71919	15	97750750	97781467	-
14636	1445363_at	Region	2810055G20Rik	77994	16	12004013	12372207	+
14637	1442913_at	Region	None	None	NONE	NONE	NONE	NONE
14638	1425231_a_at	Region	Zfp46	22704	4	135167326	135173657	+
14639	1445275_at	Region	None	None		60273768	60274413	+
14640	1452276_at	Region	Smarcad1	13990	6	65294592	65368042	+
14641	1440757_at	Region	None	None	12	113111341	113111772	+
14642	1435061_at	Region	Nudt11	58242	X	4357660	4364650	+
14643	1422855_at	Region	Cpsf3	54451	12	458	16231	-
14644	1417361_at	Region	Asb3	65257	11	30849253	30996260	+
14645	1429122_a_at	Region	1700040I03Rik	73327	6	85796727	85801218	-
14646	1448360_s_at	Region	D1Ertd396e	52477	1	190665121	190682759	+
14647	1456139_at	Region	Igf2r	16004	17	11321447	11408701	-
14648	1418627_at	Region	Gclm	14630	3	121023929	121044985	+
14649	1446523_at	Region	None	None	14	58131731	58132379	-
14650	1437763_at	Region	BC030335	233805	7	113731380	113770970	-
14651	1454770_at	Region	Cckbr	12426	7	99538177	99548695	+
14652	1433539_at	Region	Commd3	12238	2	18714472	18718226	+
14653	1449999_a_at	Region	Cacna2d1	12293	5	14583526	14791513	+
14654	1451918_a_at	Region	Loh12cr1	67774	6	135398449	135468638	+
14655	1456028_x_at	Region	Marcks	17118	10	37207003	37211096	-
14656	1435656_at	Region	Gmps	229363	3	63642160	63687698	+
14657	1434176_x_at	Region	Poldip3	73826	15	83176653	83200011	-
14658	1454609_x_at	Region	6430527G18Rik	238330	12	83753740	83757850	-
14659	1454507_at	Region	8430432A02Rik	71524	1	43387305	43388553	+
14660	1418006_at	Region	LOC546337	546337	X	73515358	73518950	-
14661	1418214_at	Region	Klc2	16594	19	4896536	4907034	-

14662	1428698_at	Region	2310004I03Rik	76430	9	65203085	65206705	+
14663	1453313_at	Region	Sesn3	75747	9	14111292	14161413	+
14664	1419324_at	Region	Lhx9	16876	1	138672251	138686188	-
14665	1428080_at	Region	2610528A17Rik	72542	5	109309939	109320666	-
14666	1426151_a_at	Region	Stx3	20908	19	10973053	11015388	-
14667	1452397_at	Region	2810474O19Rik	67246	6	24756910	24759603	+
14668	1428851_at	Region	1300014I06Rik	66895	13	34160016	34184847	-
14669	1418860_a_at	Region	Letmd1	68614	15	100526632	100536702	+
14670	1455710_x_at	Region	Mtcp1	17763	X	70065501	70077198	-
14671	1417766_at	Region	1810044O22Rik	66427	8	106448348	106485157	+
14672	1423705_at	Region	2310057D15Rik	67870	5	99058653	99087401	+
14673	1423223_a_at	Region	Prdx6	11758	1	161144235	161155289	-
14674	1460135_at	Region	A930005H10Rik	68161	3	114652097	114658964	-
14675	1429642_at	Region	Anubl1	67492	6	116701745	116767837	+
14676	1442867_at	Region	None	None	9	64834053	64834915	-
14677	1418893_at	Region	Pbx2	18515	17	32293780	32299792	+
14678	1434117_at	Region	Tceb3	27224	4	134884627	134902906	-
14679	1441317_x_at	Region	MGI:1923321	76071	5	35557428	35631807	+
14680	1439273_at	Region	Ripk1	19766	13	33537383	33569679	+
14681	1437681_at	Region	Grik4	110637	9	42479281	42777274	-
14682	1425182_x_at	Region	Klk22 /// Klk9	13646	7	38187015	38191208	+
14683	1438026_at	Region	2310030G09Rik	68030	NONE	NONE	NONE	NONE
14684	1442917_at	Region	LOC432607	432607	11	102397577	102400730	-
14685	1440579_at	Region	Mib1	225164	18	10770969	10857561	+
14686	1459843_s_at	Region	Smad1	17125	8	78552485	78613558	-
14687	1416557_a_at	Region	MGI:1336880	20624	11	102659566	102702006	-
14688	1418324_at	Region	Fem1b	14155	9	62914316	62934099	-
14689	1449140_at	Region	Nudcd2	52653	11	40472609	40478985	+
14690	1428491_at	Region	Commd10	69456	18	47180632	47309751	+
14691	1460404_at	Region	Sr278	432766	13	61254171	61284103	+
14692	1427959_at	Region	Abhd10	213012	16	44615021	44627461	-
14693	1435226_at	Region	Ibrdc3	75234	4	128085580	128112996	+
14694	1435829_at	Region	B930008K04Rik	215693	X	128517716	128523567	-
14695	1416832_at	Region	Slc39a8	67547	3	134710465	134772768	+
14696	1450057_at	Region	2610042O14Rik	66460	2	163917729	163922245	+
14697	1416051_at	Region	C2	12263	17	32565421	32584866	-
14698	1457764_at	Region	A530058O07Rik	97881	13	15269258	15270095	-
14699	1432075_a_at	Region	Tekt1	21689	11	72070380	72087531	-
14700	1430699_at	Region	Gpr165	76206	X	91314802	91318847	+
14701	1438976_x_at	Region	Mat2a	232087	6	72764773	72771532	-
14702	1451208_at	Region	Etf1	225363	18	35127577	35155611	-
14703	1450725_s_at	Region	Car14	23831	3	95385632	95392471	-
14704	1438992_x_at	Region	Atf4	11911	15	80308201	80310204	+
14705	1457341_at	Region	None	None	2	119254672	119255259	-
14706	1451475_at	Region	Plxnd1	67784	6	116393275	116432499	-
14707	1419310_s_at	Region	Rfxank	19727	8	69285462	69292088	-
14708	1436520_at	Region	AI450948	382643	12	108251581	108278490	-
14709	1439460_a_at	Region	Zfp289 /// LOC434076	434076	6	113126598	113129317	+
14710	1423761_at	Region	5630401D24Rik	71449	1	162443384	162458080	-
14711	1451721_a_at	Region	H2-Ab1	14961	17	31964475	31970648	+
14712	1454532_at	Region	C030043A13Rik	77646	4	107809843	107810925	+
14713	1436199_at	Region	Trim14	74735	4	46422769	46452063	-
14714	1448692_at	Region	Ubqln4	94232	3	88297580	88313589	+
14715	1428303_at	Region	1500005I02Rik	72014	11	114611762	114616985	-
14716	1429224_at	Region	Pnma1	70481	12	81010448	81012742	-
14717	1421949_a_at	Region	2610507L03Rik	72140	7	30562511	30603407	+
14718	1435387_at	Region	AI505012	223804	15	91320346	91321979	-
14719	1434347_s_at	Region	None	None	10	62970027	62970706	+
14720	1419101_at	Region	Sin3a	20466	9	57189858	57241642	+
14721	1451975_at	Region	2810453I06Rik	67238	5	142607596	142623478	+
14722	1444062_at	Region	2900056L01Rik	73050	15	62273515	62274555	+
14723	1416631_at	Region	Ap4b1	67489	3	103237019	103249414	+
14724	1452750_at	Region	5530601H04Rik	71445	X	99638560	99671489	-
14725	1438289_a_at	Region	Sumo1	22218	1	59942975	59974347	-

14726	1451351_at	Region	Ttc13	234875	8	123958404	124009066	-
14727	1419798_at	Region	2610019E17Rik	75614	17	22331852	22332329	+
14728	1432472_a_at	Region	Mccc2	78038	13	96151384	96164931	-
14729	1459288_at	Region	Kcnd2	16508	6	21263145	21776841	+
14730	1441461_at	Region	Frmd4a	209630	2	4348468	4531213	+
14731	1437122_at	Region	Bcl2	12043	1	106479000	106655083	-
14732	1448662_at	Region	Fzd6	14368	15	38905352	38937760	+
14733	1433526_at	Region	Klhl8	246293	5	102896841	102946020	-
14734	1440803_x_at	Region	Tacr3	21338	3	133712014	133817494	+
14735	1424180_a_at	Region	Thrap4	23989	11	98525688	98550454	-
14736	1447432_s_at	Region	Zfp263	74120	16	3415695	3422348	+
14737	1420832_at	Region	Qscn6	104009	1	155674165	155708148	-
14738	1449569_at	Region	Thpo	21832	16	19496631	19505618	-
14739	1442494_at	Region	C79242	98068	17	44520980	44521682	-
14740	1459316_at	Region	Crybb3	12962	5	112147135	112151946	-
14741	1442562_at	Region	D630040G17Rik	244530	8	78407158	78407858	+
14742	1442216_at	Region	Atp6v0d1	11972	8	104820098	104861668	-
14743	1434570_at	Region	AK122525	331623	10	43585500	43621771	+
14744	1448187_at	Region	Pold1	18971	7	38608666	38624732	-
14745	1442054_at	Region	None	None	2	181006795	181007208	+
14746	1423908_at	Region	Ndufs8	225887	19	3697661	3701550	-
14747	1429259_a_at	Region	1810014B01Rik	544717	10	82507736	82511798	-
14748	1451398_at	Region	BC009118	234309	8	60552965	60568663	+
14749	1438429_at	Region	2610319H10Rik	72505	8	10994869	10998339	-
14750	1427565_a_at	Region	Abcc5	27416	16	19102921	19197967	-
14751	1423667_at	Region	Mat2a	232087	6	72764773	72771532	-
14752	1425073_at	Region	Plekhg6	213522	6	126019331	126037184	-
14753	1440930_a_at	Region	LOC545474	545474	2	151550295	151552735	+
14754	1441539_at	Region	None	None	6	145554705	145555373	-
14755	1455305_x_at	Region	None	None	14	55533909	55858469	-
14756	1442845_at	Region	C130075A20Rik	320395	3	52733872	52734993	+
14757	1452011_a_at	Region	Uxs1	67883	1	44044005	44123567	-
14758	1421941_at	Region	Camk4	12326	18	33179625	33409958	+
14759	1435203_at	Region	Man2a2	140481	7	74160715	74171123	-
14760	1426536_at	Region	Narg2	93697	9	69529686	69564588	+
14761	1427068_x_at	Region	4933439F18Rik	66771	11	60142945	60170919	+
14762	1424120_at	Region	Rnf8	58230	17	27424760	27451559	+
14763	1456930_at	Region	Camsap1	227634	2	25860417	25927345	-
14764	1430849_a_at	Region	Cgrrf1	68755	14	41912127	41934073	+
14765	1432271_a_at	Region	4833420K19Rik	76863	9	7176782	7200101	+
14766	1454641_at	Region	Cggbp1	106143	16	63876856	63884262	+
14767	1417605_s_at	Region	Camk1	52163	6	113901957	113911755	-
14768	1425572_a_at	Region	Ddef1	13196	15	64104369	64365898	-
14769	1421070_at	Region	D3Ertd300e	56790	3	54327325	54351057	+
14770	1434567_at	Region	4732496O08Rik	242736	4	142327675	142336336	+
14771	1423338_at	Region	Ccdc16	66983	11	82504010	82506449	+
14772	1455937_at	Region	Lrrtm4	243499	6	80352899	80359760	+
14773	1435553_at	Region	Pdzk3	68070	15	12395836	12406377	-
14774	1443665_at	Region	D030011O10Rik	320560	6	149949871	150037421	-
14775	1454295_at	Region	Nrip1	268903	16	75376752	75458939	-
14776	1439773_at	Region	Ly6e	17069	15	74988361	74992036	+
14777	1431469_a_at	Region	Cxxc5	67393	18	36053483	36085353	+
14778	1436107_at	Region	None	None	6	18903976	18905180	+
14779	1452963_at	Region	9530077C05Rik	68283	9	22301973	22335077	+
14780	1438094_x_at	Region	2810409H07Rik	67059	2	72790552	72912196	-
14781	1434201_at	Region	None	None	NONE	NONE	NONE	NONE
14782	1425678_a_at	Region	Snrk	20623	9	122152064	122189861	+
14783	1460240_a_at	Region	Hnrpc	15381	14	47175503	47204310	-
14784	1435608_at	Region	BC019575	407821	11	5174112	5343821	-
14785	1456682_at	Region	2900060P06Rik	381338	1	39094259	39094964	-
14786	1435849_at	Region	None	None	7	133484825	133485552	+
14787	1441964_at	Region	1110003F05Rik	66145	17	8843312	8844213	-
14788	1434632_at	Region	None	None	7	101542024	101542935	+
14789	1418092_s_at	Region	Trip10	106628	17	54939205	54953935	+
14790	1436015_s_at	Region	Stk4	58231	2	163531147	163612490	+

14791	1437613_s_at	Region	AW456874	218232	13	48175930	48223631	-
14792	1440209_at	Region	38412	72925	8	65495563	65579550	+
14793	1450690_at	Region	Ranbp2	19386	10	58404785	58452252	+
14794	1438133_a_at	Region	Cyr61	16007	3	144622756	144625761	-
14795	1443921_at	Region	C130037N17Rik	223332	15	8857529	8869181	+
14796	1421908_a_at	Region	Tcf12	21406	9	71980605	72246476	-
14797	1423547_at	Region	Lyzs	17105	10	116966784	116971715	-
14798	1451987_at	Region	Arrb2	216869	11	70158356	70166481	+
14799	1420709_s_at	Region	Dao1	13142	5	113120480	113135550	+
14800	1422791_at	Region	Pafah1b2	18475	9	45981441	45998071	-
14801	1442565_at	Region	Pcdh10	18526	3	44837486	44841910	+
14802	1415901_at	Region	Plod3	26433	5	136000144	136009661	+
14803	1435561_at	Region	Erf	13875	7	20426320	20430075	-
14804	1436820_at	Region	Kctd11	216858	11	69603335	69607064	-
14805	1444184_at	Region	1810020C19Rik	69113	2	93685235	93715406	-
14806	1444069_at	Region	None	None	NONE	NONE	NONE	NONE
14807	1432294_at	Region	9330177L23Rik	77246	5	130783832	130784932	+
14808	1441266_at	Region	5730526G10Rik	70656	12	48391185	48391586	-
14809	1435478_at	Region	Wdr26	226757	1	181127041	181127910	-
14810	1418631_at	Region	Ube2h	22214	6	30260446	30352143	-
14811	1434971_x_at	Region	None	None	1	4736769	4738037	-
14812	1456812_at	Region	Abcd2	26874	15	91198527	91244463	-
14813	1436706_at	Region	Tmem32	236792	X	51340226	51352633	-
14814	1426369_at	Region	Mlstd2	67420	7	107366377	107420898	+
14815	1451142_at	Region	Sec3l1	69940	5	75774556	75815526	+
14816	1434376_at	Region	Cd44	12505	2	102518748	102606202	-
14817	1437734_at	Region	Ppp1r12a	17931	10	108049328	108167253	+
14818	1460292_a_at	Region	Smarca1	93761	X	42330166	42413315	-
14819	1415899_at	Region	Junb	16477	8	84245110	84246926	-
14820	1450020_at	Region	Cx3cr1	13051	9	120059072	120078236	-
14821	1433683_at	Region	9530027K23Rik	77411	8	105427030	105432821	-
14822	1424438_a_at	Region	Leprot	230514	4	100606688	100618263	+
14823	1431293_a_at	Region	1110019C08Rik	224250	16	57715966	57722159	+
14824	1443002_at	Region	Zfr	22763	15	11948204	11996199	+
14825	1416349_at	Region	Mrpl34	94065	8	70614843	70615615	+
14826	1426747_at	Region	Abcf3	27406	16	19320803	19332961	+
14827	1443008_at	Region	Msi2h	76626	11	88067452	88208048	-
14828	1460249_at	Region	Lnx2	140887	5	145908796	145968222	-
14829	1457522_at	Region	1110034C04Rik	68734	12	96484976	96528858	-
14830	1416015_s_at	Region	Abce1	24015	8	78898390	78925732	-
14831	1460289_at	Region	Nrg3	18183	14	36505478	36542277	-
14832	1419866_s_at	Region	Sca2	20239	5	120898076	120967058	+
14833	1449931_at	Region	Cpeb4	67579	11	31767001	31826367	+
14834	1426748_s_at	Region	Abcf3	27406	16	19320803	19332961	+
14835	1456009_x_at	Region	Top3b	21976	16	15641732	15663698	+
14836	1450044_at	Region	Fzd7	14369	1	59785717	59790493	+
14837	1432499_a_at	Region	Ube4b	63958	4	147820778	147919111	-
14838	1426392_a_at	Region	Actr3	74117	1	125220868	125263588	-
14839	1433482_a_at	Region	Fubp1	51886	3	151191900	151214173	+
14840	1426655_a_at	Region	4930504E06Rik	75007	3	94770775	94783992	+
14841	1429479_at	Region	6430590I03Rik	76222	16	205454	207046	-
14842	1448693_at	Region	BC002216	79554	4	154357085	154361802	-
14843	1436613_at	Region	B930093C12Rik	268445	11	77196147	77198731	-
14844	1422901_at	Region	Mgea5	76055	19	45297695	45330727	-
14845	1457824_at	Region	None	None	9	92143523	92143854	+
14846	1455914_at	Region	AI987944	233168	7	35490664	35510152	-
14847	1439526_at	Region	None	None	18	73999184	74000845	-
14848	1436941_at	Region	Gm1752	385658	16	3376808	3417077	+
14849	1420879_a_at	Region	Ywhab	54401	2	163452166	163475556	+
14850	1437334_x_at	Region	Parn	74108	16	12274917	12404322	-
14851	1424150_at	Region	Gdpd5	233552	7	93470011	93549440	+
14852	1450312_at	Region	Cdh20	23836	1	104825391	104886917	+
14853	1418238_at	Region	Ivd	56357	2	118375849	118395206	+
14854	1451018_at	Region	Leprotl1	68192	8	32936611	32947770	-
14855	1438132_at	Region	C030005G22Rik	402739	14	116933406	116934077	-

14856	1448119_at	Region	Bpgm	12183	6	34570624	34599878	+
14857	1449121_at	Region	Fusip1	14105	4	134737416	134751165	+
14858	1450824_at	Region	Ptch1	19206	13	60894523	60948510	-
14859	1457532_at	Region	Garnl1	56784	12	52323288	52540884	-
14860	1429140_at	Region	9830002I17Rik	77577	11	72223814	72275904	-
14861	1446029_at	Region	None	None	2	59562139	59562875	+
14862	1429210_at	Region	Col23a1	237759	11	51042761	51333951	+
14863	1418779_at	Region	Rce1	19671	19	4411341	4414407	-
14864	1442094_at	Region	None	None	1	93650453	93651051	+
14865	1449124_at	Region	Rgl1	19731	1	152402014	152509595	-
14866	1436342_a_at	Region	D19Ertd721e	225896	19	8068106	8072203	+
14867	1435885_s_at	Region	Itsn1	16443	16	90939437	91069253	+
14868	1435051_at	Region	Wdr44	72404	X	21932006	22044942	+
14869	1441679_at	Region	Cacna1c	12288	6	119025051	119540819	-
14870	1425496_at	Region	Abca3	27410	17	22155713	22213193	+
14871	1434508_at	Region	Ube2q	70093	3	89579799	89590178	+
14872	1460726_at	Region	Adss	11566	1	177700835	177734173	-
14873	1426269_at	Region	Sybl1	20955	X	10737	39147	-
14874	1439415_x_at	Region	Rps21	66481	2	179974367	179975394	+
14875	1451753_at	Region	Plxna2	18845	1	194358488	194552987	+
14876	1458500_at	Region	AU021034	219170	14	72423456	72431439	-
14877	1431183_at	Region	1700066M21Rik	73467	1	57681353	57687348	+
14878	1421315_s_at	Region	Cttn	13043	7	138848729	138882584	-
14879	1435176_a_at	Region	Id2	15902	12	21646605	21648874	-
14880	1456261_at	Region	Sh3kbp1	58194	X	153308006	153573572	+
14881	1448248_at	Region	Crk	12928	11	75404968	75431752	+
14882	1427916_at	Region	St7l	229681	3	104291995	104353941	+
14883	1424335_at	Region	8430432M10Rik	66812	9	57526299	57551131	-
14884	1438707_at	Region	Atp13a4	224079	16	28184867	28337155	-
14885	1455979_at	Region	Arid1b	239985	17	4902975	5250462	+
14886	1438008_at	Region	Gga3	260302	11	115405804	115424978	-
14887	1430157_at	Region	1700095J03Rik	74293	7	103292083	103297768	+
14888	1436795_at	Region	9630058J23Rik	226744	1	179500775	179581719	+
14889	1424230_at	Region	Sec15l1	107371	19	36893738	37026525	+
14890	1445656_at	Region	None	None	9	120195740	120196163	+
14891	1446220_at	Region	Gm484	243967	7	39767950	39777898	+
14892	1420654_a_at	Region	Gbe1	74185	16	69381618	69637606	+
14893	1440227_at	Region	BF642829	503859	16	91241568	91242565	+
14894	1450500_at	Region	Uhmk1	16589	1	170133433	170149570	-
14895	1454064_a_at	Region	Rnf138	56515	18	21216269	21243151	+
14896	1457719_at	Region	None	None	8	116491224	116492693	-
14897	1456735_x_at	Region	Acpl2	235534	9	96722701	96788780	-
14898	1416939_at	Region	Pyp	67895	10	61614925	61640469	+
14899	1441593_at	Region	Pten	19211	19	32083643	32146174	+
14900	1426777_a_at	Region	Wasl	73178	6	24663290	24714456	-
14901	1420956_at	Region	Apc	11789	18	34484045	34541630	+
14902	1435381_at	Region	2610110G12Rik	73242	17	33619005	33629198	-
14903	1416815_s_at	Region	Bub3	12237	7	125876724	125888172	+
14904	1424731_at	Region	Nle1	217011	11	82640430	82648030	-
14905	1437291_at	Region	2700081O15Rik	108899	19	7134873	7139385	+
14906	1444667_at	Region	Brdt	114642	5	106401608	106456144	+
14907	1448738_at	Region	Calb1	12307	4	15808229	15833613	+
14908	1415718_at	Region	2310079P12Rik	50724	11	57527266	57538710	+
14909	1436780_at	Region	Ogt	108155	X	96241425	96285710	+
14910	1436737_a_at	Region	Sorbs1	20411	19	39839082	40058071	-
14911	1429768_at	Region	None	None	18	23644049	23888215	+
14912	1437717_x_at	Region	2610005L07Rik /// LOC546041	381598	15	1398	8167	+
14913	1437789_at	Region	Birc6	12211	17	72336066	72510856	+
14914	1440171_x_at	Region	BB026409	101901	7	23299621	23299846	-
14915	1425356_at	Region	Zfp142	77264	1	74868909	74890473	-
14916	1425311_at	Region	4930432F04Rik	78792	5	138366782	138369744	-
14917	1429112_at	Region	Tln2	70549	9	67349709	67668766	-
14918	1449239_at	Region	1700045I19Rik	74264	X	157359545	157360734	-
14919	1435942_at	Region	Kcnq2	16536	2	180792537	180852183	-

14920	1422792_at	Region	Pafah1b2	18475	9	45981441	45998071	-
14921	1451020_at	Region	Gsk3b	56637	16	36906890	37063968	+
14922	1436841_at	Region	B230380D07Rik	235461	9	70745120	70797683	-
14923	1448909_a_at	Region	Mrpl39	27393	16	83827402	83845155	-
14924	1426232_at	Region	BC024479	235184	9	37430864	37465703	+
14925	1436568_at	Region	Jam2	67374	16	83882574	83932843	+
14926	1426640_s_at	Region	Trib2	217410	12	15150515	15175525	-
14927	1455034_at	Region	Nr4a2	18227	2	57033695	57040794	-
14928	1439912_at	Region	9430098F02Rik	103793	11	51880107	51880621	+
14929	1457075_at	Region	A830031M15Rik	102902	NONE	NONE	NONE	NONE
14930	1435136_at	Region	Whsc1	107823	5	32363965	32365554	+
14931	1453767_a_at	Region	Nt5m	103850	11	59573702	59602164	+
14932	1453160_at	Region	Thrap1	327987	11	85997437	86085517	-
14933	1423926_at	Region	Slc39a13	68427	2	90766540	90774970	-
14934	1451179_a_at	Region	Qk	19317	17	8850890	8959849	-
14935	1437466_at	Region	Alcam	11658	16	51168811	51372049	-
14936	1437048_x_at	Region	Stxbp2	20911	8	36313	47968	+
14937	1446346_at	Region	Adamts9	101401	6	93379900	93410347	-
14938	1427064_a_at	Region	Scrib	105782	15	76095858	76118402	-
14939	1443649_at	Region	Ureb1	59026	X	145349955	145350400	+
14940	1444531_at	Region	None	None	NONE	NONE	NONE	NONE
14941	1451281_at	Region	Zfp96	22758	13	20792934	20923192	+
14942	1416586_at	Region	Zfp239	22685	6	118294871	118304561	+
14943	1438760_x_at	Region	Adam15	11490	3	89096372	89106699	-
14944	1450519_a_at	Region	Prkaca	18747	8	83233159	83256577	+
14945	1437187_at	Region	E2f7	52679	10	110368316	110410221	+
14946	1452720_a_at	Region	Fip1l1	66899	5	73370146	73431830	+
14947	1428872_at	Region	4121402D02Rik	74026	11	98616603	98627514	+
14948	1424286_at	Region	Prkx	19108	X	72422678	72456615	-
14949	1436728_s_at	Region	Rtel1	269400	2	181036742	181073573	+
14950	1425761_a_at	Region	Nfatc1	18018	18	80729557	80836410	-
14951	1424053_a_at	Region	D8Ertd325e	66855	8	122748919	122778703	+
14952	1423054_at	Region	Wdr1	22388	5	37274667	37309449	-
14953	1460255_at	Region	Tnfsf13b	24099	8	9374137	9403504	+
14954	1457204_at	Region	Zdhhc2	70546	8	39367458	39428485	+
14955	1456407_a_at	Region	Tlk1	228012	2	70410150	70523223	-
14956	1425242_at	Region	1810006K21Rik	69038	19	9400138	9404273	+
14957	1436874_x_at	Region	Slc25a5	11740	X	31416206	31419360	+
14958	1442026_at	Region	None	None	9	48679581	48680488	-
14959	1433956_at	Region	Cdh5	12562	8	103396382	103438830	+
14960	1437950_at	Region	BC035537	212326	8	44287329	44330422	-
14961	1449074_at	Region	Kcnk4	16528	19	6639538	6648363	-
14962	1427558_s_at	Region	Alg12	223774	15	88857174	88871213	-
14963	1456662_at	Region	None	None	8	46643401	46643598	+
14964	1419055_a_at	Region	Ptpn21	24000	12	94103584	94161513	-
14965	1449317_at	Region	Cflar	12633	1	59018187	59060035	+
14966	1436708_x_at	Region	Mcm4	17217	16	14393171	14406424	-
14967	1434697_at	Region	1110001P04Rik	76296	9	44628613	44629587	+
14968	1436252_at	Region	Dnb5	242773	4	149120934	149144527	-
14969	1419652_s_at	Region	2610200G18Rik	67149	NONE	NONE	NONE	NONE
14970	1451756_at	Region	Flt1	14254	5	146453691	146617483	-
14971	1419384_at	Region	Prkcabp	18693	15	79281099	79300746	+
14972	1457254_x_at	Region	6330442E10Rik /// LOC432460	268567	12	75818785	75863926	-
14973	1421239_at	Region	Il6st	16195	13	108856634	108899366	+
14974	1438081_at	Region	None	None	18	44644561	44645426	-
14975	1452699_at	Region	D14Ertd209e	52535	14	46979197	46986291	+
14976	1439504_s_at	Region	Zfp28	22690	7	5579089	5591525	+
14977	1446192_at	Region	Amph	218038	13	18423821	18627334	+
14978	1416774_at	Region	Wee1	22390	7	103974933	103996157	+
14979	1455578_x_at	Region	Rpl41	67945	10	128285207	128286206	-
14980	1433470_a_at	Region	Immt	76614	6	72203510	72209365	+
14981	1460395_at	Region	Nudcd3	209586	11	6000493	6094949	-
14982	1455033_at	Region	B430201A12Rik	329739	3	108768194	108823063	-
14983	1458861_at	Region	None	None	6	112065505	112070019	+

14984	1449003_a_at	Region	Vti1b	53612	12	76012660	76028779	-
14985	1424779_at	Region	D10Ucla1	28193	10	67048137	67134030	-
14986	1418125_at	Region	4632409L19Rik	68142	2	118886894	118947183	-
14987	1425276_at	Region	Fbs1	14123	7	121534337	121540615	+
14988	1423060_at	Region	Pa2g4	18813	10	128295733	128303038	-
14989	1440890_a_at	Region	None	None	9	22133399	22133751	+
14990	1441535_at	Region	Mllt3	70122	4	86758854	87019463	-
14991	1460170_at	Region	Ext2	14043	2	93400316	93527228	-
14992	1439246_x_at	Region	Tnrc6a	233833	7	117180427	117250357	+
14993	1420650_at	Region	Atbf1	11906	8	108012331	108254925	+
14994	1453763_at	Region	Txndc11	106200	16	10508059	10567680	+
14995	1423666_s_at	Region	Rpl5 /// LOC545265 /// LOC545304 /// LOC545478 /// LOC545858	19983	5	106971039	106978499	+
14996	1456140_at	Region	Zic5	65100	14	117028709	117035237	-
14997	1418150_at	Region	Mtmr4	170749	11	87320209	87344288	+
14998	1453827_at	Region	1110035H17Rik	68686	7	24567089	24568328	+
14999	1434760_at	Region	Lrrtm3	216028	10	63866884	64028642	-
15000	1439559_at	Region	1700040D17Rik	76602	3	93897218	93900934	-
15001	1438762_at	Region	None	None	9	72231020	72231565	-
15002	1451792_a_at	Region	Pja1	18744	X	94067507	94072041	-
15003	1428196_a_at	Region	1200015F23Rik	67809	2	118650852	118681688	-
15004	1434320_at	Region	Gtf3c4	269252	2	28757737	28772448	-
15005	1454956_at	Region	Rps6kb1	72508	11	86241975	86272766	-
15006	1432364_at	Region	4930556N13Rik	75285	11	88672691	88702483	+
15007	1442436_at	Region	Fn3k	63828	11	121256061	121271577	+
15008	1452582_at	Region	Galm	319625	17	77944865	78002426	+
15009	1440911_at	Region	Col23a1	237759	11	51042761	51333951	+
15010	1429178_at	Region	Odz3	23965	8	47191599	47638956	-
15011	1418981_at	Region	Casp12	12364	9	5255895	5283427	+
15012	1417640_at	Region	Cd79b	15985	11	106132430	106135759	-
15013	1426133_a_at	Region	1500032H18Rik	69028	1	38169275	38184881	-
15014	1429055_at	Region	4930506M07Rik	71653	19	58572915	58675626	-
15015	1423357_at	Region	2610209A20Rik	67164	7	94265949	94268037	+
15016	1436007_a_at	Region	Thumpd1	233802	7	113590660	113596332	-
15017	1418567_a_at	Region	Srp14	20813	2	117989699	117993510	-
15018	1420920_a_at	Region	Arf1	11840	11	58937042	58939152	-
15019	1439426_x_at	Region	Lzp-s	17110	10	116977248	116982319	-
15020	1453238_s_at	Region	E430024C06Rik	319443	NONE	NONE	NONE	NONE
15021	1460001_at	Region	Pgpep1	66522	8	69805459	69816029	-
15022	1418715_at	Region	Pank1	75735	19	34154273	34221296	-
15023	1452181_at	Region	Ckap4	216197	10	84420966	84428436	-
15024	1426477_at	Region	Rasa1	218397	13	81307760	81348198	-
15025	1428844_a_at	Region	Bclaf1	72567	10	20239740	20267469	+
15026	1445160_at	Region	Nav3	260315	10	109569106	109718861	-
15027	1426002_a_at	Region	Cdc7	12545	5	106027461	106047570	+
15028	1454771_at	Region	None	None	12	97413512	97421587	-
15029	1418259_a_at	Region	Entpd2	12496	2	25328120	25333480	+
15030	1447961_s_at	Region	Mrpl38	60441	11	115952914	115959871	-
15031	1423805_at	Region	Dab2	13132	15	6175647	6229535	+
15032	1439577_at	Region	None	None	NONE	NONE	NONE	NONE
15033	1438210_at	Region	Gpr149	229357	3	62196472	62270897	-
15034	1459807_x_at	Region	4933406E20Rik	74443	9	108627920	108646375	-
15035	1440116_at	Region	D630045J12Rik	330286	6	38261271	38390218	-
15036	1442920_at	Region	Klf3	16599	5	63601098	63627704	+
15037	1435053_s_at	Region	Plekhh1	211945	12	75867965	75938050	+
15038	1441137_at	Region	Bicc1	83675	10	70979053	71213601	-
15039	1445562_at	Region	Zbtb16	235320	9	48684510	48863820	-
15040	1439894_at	Region	A730056I06Rik	319521	NONE	NONE	NONE	NONE
15041	1415853_at	Region	Def8	23854	8	122817883	122838796	+
15042	1419325_at	Region	Nmu	56183	5	75578734	75609016	-
15043	1428618_at	Region	1700129L13Rik	67933	10	82611323	82629097	+
15044	1444042_at	Region	Trip11	109181	12	97280506	97358629	-

15045	1437357_at	Region	Ythdc2	240255	18	45048187	45109220	+
15046	1424763_at	Region	1700027N10Rik	75564	17	43639198	43654119	-
15047	1415880_a_at	Region	Lamp1	16783	8	12537914	12553973	+
15048	1416284_at	Region	Mrpl28	68611	17	23916702	23919783	+
15049	1427163_at	Region	Ubr2	224826	17	44441356	44523334	-
15050	1422564_at	Region	Actl6b	83766	5	136504421	136520439	+
15051	1453787_at	Region	D2Bwg1356e	52837	2	134108354	134157974	-
15052	1435963_at	Region	Sema5b	20357	16	34397801	34447067	+
15053	1457848_at	Region	A930016P21Rik	68073	15	31602722	31618141	+
15054	1435970_at	Region	Nlk	18099	11	78292827	78423032	-
15055	1426875_s_at	Region	Srxn1	76650	2	151562666	151568289	+
15056	1459909_at	Region	Nfix	18032	8	83974112	84039256	-
15057	1437879_at	Region	AU045404	380959	15	90277186	90329435	+
15058	1428315_at	Region	Ebna1bp2	69072	4	117579721	117586679	+
15059	1428842_a_at	Region	Ngfrap1	12070	X	129815282	129816905	+
15060	1428402_at	Region	Zcchc3	67917	2	151869891	151871963	-
15061	1456046_at	Region	C1qr1	17064	2	147893588	147900480	-
15062	1447159_at	Region	None	None	12	72716693	72717203	+
15063	1458381_at	Region	Clic5	224796	17	41693345	41782570	+
15064	1440543_at	Region	D930036F22Rik	320487	12	48628093	48722896	-
15065	1452306_at	Region	Zfyve26	211978	12	76088750	76155295	-
15066	1427418_a_at	Region	Hif1a	15251	12	70756396	70795257	+
15067	1417908_s_at	Region	Ube2l3	22195	16	15923031	15972515	-
15068	1422053_at	Region	Inhba	16323	13	15463482	15476441	+
15069	1452831_s_at	Region	Ppat	231327	5	76158008	76196037	-
15070	1435993_at	Region	AI841875	105219	13	20964875	20965568	+
15071	1429177_x_at	Region	Sox17	20671	1	4451317	4456802	-
15072	1426610_a_at	Region	Ttf1	22130	2	28992439	29019268	+
15073	1422969_s_at	Region	Ihpk1	27399	9	108070635	108116769	+
15074	1459349_at	Region	A930011G23Rik	319818	5	98315387	98751358	-
15075	1459632_at	Region	Crim1	50766	17	76085677	76086109	+
15076	1458623_at	Region	None	None	17	43951118	43951549	-
15077	1443012_at	Region	Tcf12	21406	9	71980605	72246476	-
15078	1437261_at	Region	None	None	13	77832696	77833441	+
15079	1438755_at	Region	C80068	97810	12	108529861	108530486	+
15080	1457148_at	Region	Csmd2	329942	4	127374143	127591281	+
15081	1446426_at	Region	Enah	13800	1	181864370	181978518	-
15082	1420643_at	Region	Lfng	16848	5	139606194	139613318	+
15083	1417130_s_at	Region	Angptl4	57875	17	31474204	31480810	-
15084	1436541_at	Region	2310008H09Rik	66356	7	112721617	112732114	-
15085	1451545_at	Region	Tdrd3	219249	14	81829185	81957574	+
15086	1460742_at	Region	Alg3	208624	16	19377613	19382885	-
15087	1433792_at	Region	Nrip2	60345	6	129089953	129098438	+
15088	1422123_s_at	Region	Ceacam1 /// Ceacam2	26365	7	20682164	20703996	-
15089	1435930_at	Region	Zfp291	244891	9	55662849	56048009	-
15090	1419416_a_at	Region	Rarg	19411	15	102294300	102316638	-
15091	1459307_at	Region	Sec24b	99683	3	128916056	128993778	-
15092	1454855_at	Region	Magi2	50791	5	18347411	19110332	+
15093	1453045_at	Region	4921537D05Rik	77048	10	94662974	94764320	+
15094	1454843_at	Region	Prps2	110639	X	160945738	160982104	-
15095	1436842_at	Region	B230380D07Rik	235461	9	70745120	70797683	-
15096	1428613_at	Region	Ldhd	52815	8	110923860	110927911	-
15097	1452681_at	Region	Dtymk	21915	1	93630666	93632046	-
15098	1422235_at	Region	Htr7	15566	19	35302942	35400012	-
15099	1435968_at	Region	None	None	12	113040573	113041796	+
15100	1449438_at	Region	Dpm1	13480	2	167717793	167739104	-
15101	1441946_at	Region	Itih5	209378	2	10069730	10167804	+
15102	1426118_a_at	Region	Tomm40	53333	7	16570562	16584500	-
15103	1420888_at	Region	Bcl2l1	12048	2	152237604	152288618	-
15104	1453498_x_at	Region	Steap3	68428	1	119985615	120002678	-
15105	1452870_at	Region	Apaf1	11783	10	90961928	91054028	-
15106	1454608_x_at	Region	Ttr	22139	18	20880341	20889247	+
15107	1433563_s_at	Region	Derl1	67819	15	57878521	57901434	-
15108	1416506_at	Region	Psma6	26443	12	52119691	52139243	+

15109	1423683_at	Region	Cdca4	71963	12	108296070	108305225	-
15110	1460455_at	Region	1110059H15Rik	68795	2	69802617	69879409	+
15111	1435770_at	Region	D2Bwg1356e	52837	2	134108354	134157974	-
15112	1451409_at	Region	2210021J22Rik	72355	15	85855615	85860302	-
15113	1453662_at	Region	B230205O20Rik	77413	1	172110653	172111993	+
15114	1444415_at	Region	Smarcd3	66993	5	23056519	23086151	-
15115	1435357_at	Region	D4Wsu53e	27981	4	133884543	133888276	+
15116	1428754_at	Region	3300001M20Rik	66926	2	132318068	132329908	-
15117	1427221_at	Region	MGI:2143217	102680	9	123659826	123702549	-
15118	1416289_at	Region	Plod1	18822	4	146402423	146429066	-
15119	1415915_at	Region	Ddx1	104721	12	12580692	12609750	-
15120	1444862_at	Region	None	None	14	20792584	20793577	+
15121	1434031_at	Region	Zfp692	103836	11	58032732	58040239	+
15122	1434937_at	Region	Phr1	105689	14	97658865	97891435	-
15123	1440384_at	Region	Tmcc1	330401	6	116456153	116631023	-
15124	1430408_at	Region	Cacna1a	12286	8	83678856	83903623	+
15125	1454831_at	Region	3230402J05Rik	108784	8	36569453	36572130	+
15126	1456312_x_at	Region	Gsn	227753	2	35214602	35240054	+
15127	1417517_at	Region	Plagl2	54711	2	152684705	152698294	-
15128	1422900_at	Region	Mgea5	76055	19	45297695	45330727	-
15129	1447220_at	Region	1700125H20Rik	73634	11	84899088	84909137	+
15130	1429573_at	Region	4921520P21Rik	70887	X	97504587	97510052	-
15131	1426351_at	Region	Hspd1	15510	1	55378992	55389004	-
15132	1420386_at	Region	Seh1l	72124	18	68005761	68023379	+
15133	1444753_at	Region	Nek7	59125	1	138334093	138469075	-
15134	1448273_at	Region	Gss	14854	2	155020120	155044513	-
15135	1417331_a_at	Region	Arl6	56297	16	58597575	58621129	-
15136	1439377_x_at	Region	Cdc20	107995	4	117391806	117396154	-
15137	1460724_at	Region	Ap2a1	11771	7	38976114	39005954	-
15138	1420986_s_at	Region	Kif3b	16569	2	152748365	152789150	+
15139	1436072_at	Region	None	None	NONE	NONE	NONE	NONE
15140	1416756_at	Region	Dnajb1	81489	8	82866135	82869845	+
15141	1450111_a_at	Region	Nudt8	66387	19	3789370	3790892	+
15142	1456210_at	Region	None	None	2	6015766	6016580	-
15143	1417239_at	Region	Cetn3	12626	13	77863539	77877404	+
15144	1434016_at	Region	Znrf2	387524	6	54961241	55034061	+
15145	1434587_x_at	Region	Ptdss2	27388	7	135533273	135558141	+
15146	1444144_at	Region	5530400K22Rik	74501	16	43060418	43061044	+
15147	1419111_at	Region	Ing1l	69260	8	46633720	46641700	-
15148	1436977_at	Region	None	None	6	138378338	138379100	-
15149	1437108_at	Region	Lsm6	78651	8	21699568	21713205	-
15150	1451568_at	Region	A630054L15Rik	211922	14	24675766	24724614	+
15151	1416331_a_at	Region	Nfe2l1	18023	11	96638518	96649042	-
15152	1457454_at	Region	Usp47	74996	7	105876643	105964517	+
15153	1434928_at	Region	Gas2l1	78926	11	4954212	4959638	-
15154	1417114_at	Region	Gcl	23885	6	87126075	87167665	-
15155	1459526_at	Region	None	None	NONE	NONE	NONE	NONE
15156	1425864_a_at	Region	Sorcs1	58178	19	49701709	50229374	-
15157	1429125_at	Region	Zbtb9	474156	17	24768242	24771266	+
15158	1447240_at	Region	None	None	NONE	NONE	NONE	NONE
15159	1448417_at	Region	Ninj1	18081	13	48787060	48795764	+
15160	1449731_s_at	Region	None	None	12	52209583	52210002	-
15161	1425705_a_at	Region	Ero1lb	67475	13	203	5259	-
15162	1422984_at	Region	None	None	5	13659986	13674277	-
15163	1419305_a_at	Region	Fbxo36	66153	1	33563905	33564395	-
15164	1423673_at	Region	Ldoc1l	223732	15	84601998	84606382	-
15165	1456042_s_at	Region	Tce4	57354	17	22767137	22821669	-
15166	1442032_at	Region	BC030500	234290	8	57944453	57946996	+
15167	1429096_at	Region	2810455D13Rik	72810	19	22967599	22969773	+
15168	1427085_at	Region	2810432D09Rik	69961	4	41850885	41852358	-
15169	1442700_at	Region	Pde4b	18578	4	101213908	101564744	+
15170	1429477_at	Region	D15Ertd785e	52683	15	89408645	89425699	+
15171	1431507_a_at	Region	Synj2bp	24071	12	78359200	78394057	-
15172	1426825_at	Region	Fmnl3	22379	15	99375027	99428271	-
15173	1436359_at	Region	None	None	6	118584313	118585075	-

15174	1452247_at	Region	Fxr1h	14359	3	33467491	33516721	+
15175	1450644_at	Region	Zfp36l1	12192	12	76968689	76973942	-
15176	1429458_at	Region	2410127L17Rik	67383	19	17917778	17951174	+
15177	1454730_at	Region	4932414K18Rik	231225	5	42932234	42949515	-
15178	1435711_at	Region	None	None	5	122286867	122287489	-
15179	1456898_at	Region	Pura	19290	18	36504827	36511914	+
15180	1452708_a_at	Region	Luc7l	66978	17	24046121	24075138	+
15181	1440882_at	Region	Lrp8	16975	4	106761166	106833072	+
15182	1448333_at	Region	Adprh	11544	16	37262216	37270966	-
15183	1425490_a_at	Region	Wdr13	73447	X	6362274	6371378	-
15184	1453607_at	Region	Mfap3l	71306	8	59692537	59709342	+
15185	1421844_at	Region	None	None	16	25368195	25504104	+
15186	1416583_at	Region	Bad	12015	19	6655703	6665741	+
15187	1453182_a_at	Region	4122402O22Rik	77626	16	16390322	16414351	+
15188	1420878_a_at	Region	Ywhab	54401	2	163452166	163475556	+
15189	1428125_at	Region	4921506J03Rik	382423	10	112606837	112607555	-
15190	1429415_at	Region	Prkcbp1	228880	2	165242034	165353683	-
15191	1448699_at	Region	Mrpl54	66047	10	81400852	81403056	-
15192	1419666_x_at	Region	Nupr1	56312	7	120672977	120675201	-
15193	1416915_at	Region	Msh6	17688	17	85834231	85850210	+
15194	1443751_at	Region	None	None	NONE	NONE	NONE	NONE
15195	1422008_a_at	Region	Aqp3	11828	4	41231657	41237029	-
15196	1456789_at	Region	Zfp462	242466	4	54936056	54997483	+
15197	1455499_at	Region	Nrxn2	18190	19	6319521	6322577	+
15198	1425932_a_at	Region	Cugbp1	13046	2	90645207	90721804	+
15199	1451318_a_at	Region	Lyn	17096	4	3605271	3718758	+
15200	1456378_s_at	Region	Fbxl20	72194	11	97909793	97970681	-
15201	1444584_at	Region	LOC238535 /// 4921509E05Rik	238535	13	20903256	20903692	-
15202	1429527_a_at	Region	Plscr1 /// LOC433328	22038	9	92150623	92163255	+
15203	1436294_at	Region	Ankrd29	225187	18	12441557	12493433	-
15204	1429139_at	Region	Za20d1	229603	3	95647489	95648962	+
15205	1421362_a_at	Region	Frk	14302	10	34545676	34672847	+
15206	1430989_a_at	Region	1700020I14Rik	66602	2	119108145	119117748	+
15207	1423007_a_at	Region	Gfra2	14586	14	65206370	65294008	+
15208	1417421_at	Region	S100a1	20193	3	90321818	90325979	-
15209	1454136_a_at	Region	4921524J17Rik	66714	8	84681533	84705607	-
15210	1416757_at	Region	Zmilch	68014	9	64258125	64293684	-
15211	1428511_at	Region	Phkg2	68961	7	121623135	121633036	+
15212	1442029_at	Region	Kcnq1	16535	7	137520467	137840061	+
15213	1454685_at	Region	None	None	5	138392661	138393907	+
15214	1439952_at	Region	3110082M05Rik	73226	15	98719129	98719698	-
15215	1439330_at	Region	D230040J21Rik	320216	5	149621768	149622304	+
15216	1440690_at	Region	None	None	15	8173777	8174403	-
15217	1454014_a_at	Region	Mkks	59030	2	136387917	136405031	-
15218	1416812_at	Region	Tia1	21841	6	86837265	86854216	+
15219	1440262_at	Region	AI854107	102623	9	27249246	27250532	+
15220	1435530_at	Region	Camsap1	227634	2	25860417	25927345	-
15221	1425321_a_at	Region	Clmn	94040	12	100215893	100309212	-
15222	1456308_x_at	Region	Trim28	21849	7	11017842	11024710	+
15223	1452776_a_at	Region	6330412F12Rik	53312	5	23149873	23174275	+
15224	1451988_s_at	Region	Chmp4b	75608	2	154114001	154151720	+
15225	1452922_at	Region	Ppp1r3d	228966	2	178130326	178131165	-
15226	1451562_at	Region	BC006662	223267	14	117455920	117465563	-
15227	1450376_at	Region	Mxi1	17859	19	52878345	52941221	+
15228	1417848_at	Region	MGI:2180715	170753	3	9425289	9606906	-
15229	1428826_at	Region	Nr6a1	14536	2	38655532	38859850	-
15230	1426985_s_at	Region	2810485I05Rik	72826	9	13664776	13683571	+
15231	1447264_at	Region	Rab11fip1	75767	8	25872507	25907865	-
15232	1421529_a_at	Region	Txnrd1	50493	10	82746224	82784757	+
15233	1435827_at	Region	4933404O12Rik	66752	5	135949114	135950263	+
15234	1453949_s_at	Region	Lypla1	18777	1	4768285	4807127	+
15235	1457995_at	Region	F730014I05Rik	228866	2	164336101	164348170	+
15236	1436247_at	Region	4632419I22Rik	74038	11	85938650	85939941	-

15237	1442811_at	Region	Rgmb	68799	17	13809790	13830123	-
15238	1440124_at	Region	B230334C09Rik	319537	3	157081963	157082492	-
15239	1425635_at	Region	Tnk1	83813	11	69576757	69584375	-
15240	1416654_at	Region	Slc31a2	20530	4	61377636	61389602	+
15241	1455381_at	Region	4921513D23Rik	320675	16	12845006	12845681	-
15242	1456159_at	Region	2900045N06Rik	72895	6	113640716	113716498	+
15243	1437984_x_at	Region	Bat1a	53817	17	32946813	32958600	+
15244	1455083_at	Region	AI315324	102887	X	54977735	54978667	-
15245	1449845_a_at	Region	Ephb4	13846	5	136301023	136325355	+
15246	1428843_at	Region	38416	69104	19	99847336	99848552	-
15247	1453048_at	Region	Nhlrc2	66866	19	56133809	56184394	+
15248	1442964_at	Region	AU020772	277939	7	94478918	94576823	+
15249	1431098_at	Region	Rsn	56430	5	122749321	122826732	-
15250	1419867_a_at	Region	Ankhd1	108857	18	36862126	36866764	+
15251	1432843_at	Region	5730405O12Rik	70488	NONE	NONE	NONE	NONE
15252	1421019_at	Region	1700021F05Rik	67851	10	43631481	43647355	-
15253	1440353_at	Region	Ntf5	78405	7	39490789	39494273	+
15254	1451095_at	Region	Asns	27053	6	7647928	7665939	-
15255	1451780_at	Region	Blnk	17060	19	40473299	40538907	-
15256	1418370_at	Region	Tnnc1	21924	14	29340150	29343549	+
15257	1437439_at	Region	2410089E03Rik	73692	15	7956639	8058734	+
15258	1438971_x_at	Region	Ube2h	22214	6	30260446	30352143	-
15259	1421897_at	Region	Elk1	13712	X	19173867	19189533	-
15260	1427269_at	Region	Sfrs11	69207	3	157020040	157032607	-
15261	1438365_x_at	Region	Laptm4b	114128	15	34237699	34283968	+
15262	1420882_a_at	Region	Acd	497652	13	53854390	53923720	+
15263	1433388_at	Region	2900022M07Rik	72951	NONE	NONE	NONE	NONE
15264	1445952_at	Region	Baiap1	14924	6	94131999	94737301	-
15265	1460684_at	Region	Tm7sf2	73166	19	5851612	5856632	-
15266	1448505_at	Region	MGI:1927354	57316	11	17152411	17163969	+
15267	1439464_s_at	Region	Tex10	269536	4	48348434	48389381	-
15268	1436355_at	Region	None	None	NONE	NONE	NONE	NONE
15269	1437604_x_at	Region	Apcdd1	494504	18	63040206	63182348	+
15270	1434076_at	Region	Wdr37	207615	13	8762169	8828183	-
15271	1437179_at	Region	Rif1	51869	2	52004994	52054222	+
15272	1418524_at	Region	Pcm1	18536	8	40177407	40269978	+
15273	1418457_at	Region	Cxcl14	57266	13	54906542	54914449	-
15274	1417719_at	Region	Sap30	60406	8	56521958	56527090	-
15275	1427087_at	Region	Luc7l2	192196	6	38687728	38745622	+
15276	1418776_at	Region	5830443L24Rik	76074	5	103928079	104182535	-
15277	1418174_at	Region	Dbp	13170	7	39788555	39793474	+
15278	1437570_at	Region	AI503301	103762	11	94109008	94110891	-
15279	1433576_at	Region	Mat2a	232087	6	72764773	72771532	-
15280	1438079_at	Region	BC050078	237775	11	59187430	59198103	-
15281	1418020_s_at	Region	Cpd	12874	11	76507648	76572655	-
15282	1426890_a_at	Region	Rpap1	68925	2	119277813	119301347	-
15283	1454989_at	Region	BC055915	211660	1	10178667	10241764	+
15284	1451133_s_at	Region	8430437G11Rik	101118	6	13567638	13595014	-
15285	1423833_a_at	Region	Brp44	70456	1	165383120	165403135	+
15286	1417214_at	Region	Rab27b	80718	18	70213787	70288489	-
15287	1419589_at	Region	C1qr1	17064	2	147893588	147900480	-
15288	1451845_a_at	Region	A230072I16Rik	217057	11	86411975	86420315	+
15289	1434580_at	Region	Enpp4	224794	17	41602556	41609249	-
15290	1420374_at	Region	Foxj2	60611	6	123487129	123512478	+
15291	1452176_at	Region	Nup153	218210	13	46278420	46325906	-
15292	1459729_at	Region	Slc13a5	237831	11	71969163	71992223	-
15293	1438458_a_at	Region	Sfpq	71514	4	126048448	126057909	+
15294	1449741_at	Region	Dsg2	13511	18	20773051	20817758	+
15295	1422300_at	Region	Nog	18121	11	89122409	89123107	-
15296	1426999_at	Region	2700069A02Rik	75553	12	94185830	94214695	+
15297	1452970_at	Region	Zfp198	76007	14	51415856	51488606	+
15298	1451185_at	Region	Sf3b5	66125	10	12890149	12890854	+
15299	1434806_at	Region	Mtx3	382793	13	89041743	89043160	+
15300	1429083_at	Region	Agl	77559	3	115512007	115576987	-
15301	1455872_at	Region	A030013D21	219148	14	57967046	57996089	+

15302	1450251_a_at	Region	Lnx1	16924	5	73427153	73512831	-
15303	1448314_at	Region	Cdc2a	12534	10	69391976	69408253	-
15304	1433582_at	Region	1190002N15Rik	68861	9	94379663	94444801	-
15305	1434705_at	Region	Ctbp2	13017	7	127353901	127489679	-
15306	1429883_at	Region	Actl6a	56456	3	32116905	32135138	+
15307	1420837_at	Region	Ntrk2	18212	13	57448390	57572946	+
15308	1427089_at	Region	Ccnt2	72949	1	127616552	127647225	+
15309	1418396_at	Region	Gpsm3	106512	17	32288936	32290990	+
15310	1440630_at	Region	None	None	3	24845240	24845907	-
15311	1447803_x_at	Region	Capg	12332	6	72878054	72896490	+
15312	1446567_at	Region	None	None	17	45068354	45069002	+
15313	1449078_at	Region	St3gal6	54613	16	57459955	57512884	-
15314	1439270_x_at	Region	Ran	19384	5	128195181	128199286	+
15315	1448192_s_at	Region	Prps1	19139	X	134001552	134021078	+
15316	1446158_at	Region	Sec15l2	75914	6	84969806	85419115	-
15317	1438629_x_at	Region	Grn	14824	11	102251594	102257892	+
15318	1427535_s_at	Region	AW822216	98733	1	75781262	75796364	-
15319	1428822_a_at	Region	Snx24	69226	18	53463791	53608966	+
15320	1424809_at	Region	Crb3	224912	17	54752222	54755863	+
15321	1446167_at	Region	Hipk2	15258	6	38834084	39012133	-
15322	1435010_at	Region	Asb7	117589	7	60532010	60573698	-
15323	1428723_at	Region	2310047M10Rik	71923	11	68785392	68787234	+
15324	1459548_at	Region	Spire1	68166	18	67718995	67783507	-
15325	1418293_at	Region	Ifit2	15958	19	33882125	33907702	+
15326	1435550_at	Region	C430014K11Rik	320003	15	98888709	98889520	-
15327	1428695_at	Region	9130227C08Rik	219094	14	50402985	50414797	+
15328	1419681_a_at	Region	Prok2	50501	6	100179494	100194587	-
15329	1420903_at	Region	St6galnac3	20447	3	152205547	152511989	-
15330	1435688_at	Region	None	None	8	90102632	90103531	-
15331	1424370_s_at	Region	Psmf1	228769	2	151172998	151198229	-
15332	1451589_at	Region	Gats	80909	5	133391036	133432162	+
15333	1422286_a_at	Region	Tgif	21815	17	68616183	68623166	-
15334	1435988_x_at	Region	Ik	24010	18	36968481	36981400	+
15335	1437872_at	Region	AB112350	242864	5	20115267	20153709	-
15336	1421820_a_at	Region	Nf2	18016	11	4662781	4744337	-
15337	1426734_at	Region	BC022623	224093	16	29408747	29411779	+
15338	1418345_at	Region	Tnfsf13	69583	11	69408706	69411183	-
15339	1438708_x_at	Region	Ywhab	54401	2	163452166	163475556	+
15340	1438309_at	Region	None	None	2	58192993	58193576	-
15341	1448172_at	Region	Mdh1	17449	11	21451580	21466622	-
15342	1449643_s_at	Region	Btf3	218490	13	94498396	94505466	-
15343	1418071_s_at	Region	Cdyl	12593	13	35194740	35408765	+
15344	1426874_at	Region	BC022641	234699	8	105176798	105189055	+
15345	1420899_at	Region	Rab18	19330	18	60692	85718	-
15346	1429129_at	Region	1200008A14Rik	71710	3	14530640	14569495	+
15347	1460294_at	Region	Atp8a2	50769	14	54182804	54623125	-
15348	1437524_x_at	Region	Coro7	78885	16	4297661	4351229	-
15349	1423122_at	Region	Avpi1	69534	19	41667647	41673365	-
15350	1456419_at	Region	5730455P16Rik	70591	11	80087114	80108980	-
15351	1429106_at	Region	4921509J17Rik	70857	3	40214140	40215805	+
15352	1422151_at	Region	Gats	80909	5	133391036	133432162	+
15353	1441300_at	Region	Kcnf1	382571	12	16538786	16543211	-
15354	1454731_at	Region	Myo10	17909	15	25611655	25800954	+
15355	1417070_at	Region	Cyp4v3	102294	8	44254051	44281445	-
15356	1428554_a_at	Region	1810035L17Rik	380773	12	84547466	84553473	-
15357	1435563_at	Region	Mrps5	77721	2	127101279	127117839	+
15358	1436277_at	Region	None	None	4	150799381	150801271	-
15359	1429190_at	Region	1110007C02Rik	71784	13	90125585	90127079	+
15360	1418676_at	Region	Isl2	104360	9	55654173	55659145	+
15361	1456379_x_at	Region	Dner	227325	1	84703796	85030178	-
15362	1454835_at	Region	Epm2aip1	77781	9	111313226	111320386	+
15363	1437704_at	Region	2900024O10Rik	72852	13	77830145	77830658	+
15364	1416083_at	Region	Za20d2	22682	19	20510205	20520216	+

15365	1448773_at	Region	Rpl11 /// LOC235424 /// LOC328825	235424	9	59260606	59267974	-
15366	1446868_at	Region	None	None	6	129133782	129134297	+
15367	1452652_at	Region	2310037P21Rik	72309	9	123277458	123278318	-
15368	1435268_at	Region	None	None	10	75117622	75118105	+
15369	1428648_at	Region	Cand1	71902	10	118778384	118938138	-
15370	1421973_at	Region	Gfra1	14585	19	57822423	58054639	-
15371	1430519_a_at	Region	Cnot7	18983	8	39436679	39454947	-
15372	1416199_at	Region	Kifc3	16582	8	94384005	94394596	-
15373	1434386_at	Region	Atp2c1	235574	9	105393471	105477208	-
15374	1435797_at	Region	None	None	5	28729806	28730574	+
15375	1430437_a_at	Region	1300018I17Rik	72325	8	122617239	122629245	-
15376	1430055_at	Region	1700080G18Rik	73533	6	30094506	30095087	-
15377	1451645_at	Region	Fbs1	14123	7	121534337	121540615	+
15378	1453485_s_at	Region	1110005A03Rik	74319	11	116664693	116670827	+
15379	1456161_at	Region	0610040B10Rik	67672	5	142387810	142391583	+
15380	1449888_at	Region	Epas1	13819	17	84588025	84665705	+
15381	1458302_at	Region	None	None	NONE	NONE	NONE	NONE
15382	1434381_at	Region	BC060631	234776	8	116271705	116288863	+
15383	1445741_at	Region	Ppm1e	320472	11	86954898	87086986	-
15384	1456617_a_at	Region	Eif2s2	67204	2	154328353	154349719	-
15385	1418003_at	Region	1190002H23Rik	66214	14	73642798	73655682	-
15386	1432177_a_at	Region	Mnat1	17420	12	69969909	70119026	+
15387	1430153_at	Region	Tmod2	50876	9	75710299	75748747	-
15388	1456530_x_at	Region	Elovl1	54325	4	117387050	117391522	+
15389	1452871_at	Region	Neil1	72774	9	57256745	57260523	-
15390	1435732_x_at	Region	Atp6v0c	11984	17	21967459	21973000	-
15391	1415826_at	Region	Atp6v1h	108664	1	5048448	5126164	+
15392	1429809_at	Region	8430438D04Rik	278279	10	105080153	105466941	-
15393	1447757_x_at	Region	Inpp5f	101490	7	122660050	122745896	+
15394	1435153_at	Region	Btbd6	399566	12	108452744	108455121	+
15395	1415946_at	Region	Pigq	14755	17	3046	4385	-
15396	1424281_at	Region	Ubap2	68926	4	41333166	41413986	-
15397	1438677_at	Region	Pkp4	227937	2	59016420	59210603	+
15398	1422844_a_at	Region	Wdr77	70465	3	105755327	105765589	+
15399	1447950_at	Region	A730011C13Rik	319916	3	94482696	94483792	-
15400	1433569_x_at	Region	Ran	19384	5	128195181	128199286	+
15401	1455339_at	Region	C430014M02Rik	338355	4	61391533	61451725	-
15402	1416855_at	Region	Gas1	14451	13	58831933	58834893	-
15403	1419914_s_at	Region	D10Ertd438e	52014	10	52670740	52691207	+
15404	1439168_at	Region	Camk2d	108058	3	125480582	125726370	+
15405	1430033_at	Region	5330431K02Rik	68189	13	95691241	95693153	-
15406	1430983_at	Region	Pdia6	71853	12	16627969	16646144	+
15407	1428919_at	Region	Fgfr1op	75296	17	778	15020	+
15408	1436788_at	Region	None	None	2	90917134	90917822	+
15409	1426755_at	Region	Ckap4	216197	10	84420966	84428436	-
15410	1452154_at	Region	Iars	105148	13	49279007	49331120	+
15411	1424062_at	Region	Ube2d1	216080	10	71308947	71339200	-
15412	1428237_at	Region	2700059D21Rik	433693	4	34689700	34705975	+
15413	1435375_at	Region	BC052328	223433	15	27644740	27670078	-
15414	1456952_at	Region	Arid1b	239985	17	4902975	5250462	+
15415	1422262_a_at	Region	Lhx6	16874	2	36015701	36037433	-
15416	1450130_at	Region	Xpr1	19775	1	155171522	155308066	-
15417	1431561_a_at	Region	Dhx34	71723	7	287	10829	-
15418	1455191_x_at	Region	Pip5k1b	18720	3	94547426	94594689	-
15419	1450739_at	Region	Tbl1xr1	81004	3	21419728	21554694	+
15420	1449154_at	Region	Col11a1	12814	3	26642	73590	-
15421	1448725_at	Region	Parg	26430	14	30335360	30430279	+
15422	1419458_at	Region	Rgnef	110596	13	94087960	94394666	-
15423	1430942_at	Region	8430437O03Rik	71536	1	172113346	172114653	+
15424	1419550_a_at	Region	Stk39	53416	2	68065847	68327381	-
15425	1424000_a_at	Region	Rps11	27207	7	39198928	39200922	-
15426	1423219_a_at	Region	Mrpl49	18120	19	5842420	5846541	-
15427	1430355_a_at	Region	Steap3	68428	1	119985615	120002678	-

15428	1448381_at	Region	Gfm1	28030	3	67102432	67147379	+
15429	1425476_at	Region	Col4a5	12830	X	134915604	135128563	+
15430	1419758_at	Region	Abcb1a	18671	5	8666835	8755262	+
15431	1418456_a_at	Region	Cxcl14	57266	13	54906542	54914449	-
15432	1451538_at	Region	Sox9	20682	11	112603317	112608842	+
15433	1451419_at	Region	MGI:2183445	211949	9	96842840	96917713	-
15434	1460702_at	Region	1810015M01Rik	69076	5	114451813	114454118	+
15435	1435236_at	Region	A630018P17Rik	245695	X	160099424	160109883	-
15436	1442139_at	Region	None	None	9	4194044	4195311	-
15437	1459630_at	Region	None	None	8	124551971	124552460	+
15438	1457806_at	Region	Dock1	330662	7	129038306	129541299	+
15439	1431308_at	Region	5730557B15Rik	67434	15	31296331	31368171	-
15440	1437929_at	Region	Dact2	240025	17	12245641	12254191	-
15441	1418254_at	Region	Apip	56369	2	102778429	102797375	+
15442	1456524_at	Region	Nrg1	211323	8	30634617	30806905	-
15443	1433653_at	Region	BC029169	208659	11	109494015	109543345	-
15444	1423693_at	Region	Ela1	109901	15	100731963	100745456	-
15445	1427167_at	Region	AI448196	102910	X	128238260	128241693	+
15446	1436234_at	Region	4732471D19Rik	319719	13	53135721	53161052	+
15447	1418521_a_at	Region	Mtx1	17827	3	88965562	88970378	-
15448	1454722_at	Region	Herc1	235439	9	66474369	66632783	+
15449	1443728_at	Region	None	None	NONE	NONE	NONE	NONE
15450	1432080_s_at	Region	Myst1	67773	7	121962327	121975572	+
15451	1435573_at	Region	DXImx46e	54644	X	6080771	6113800	+
15452	1439497_at	Region	None	None	12	29985088	29986593	+
15453	1451400_at	Region	BC023488	237221	X	159769900	159789915	+
15454	1436422_at	Region	BC026590	230234	4	56745799	56753050	+
15455	1437547_s_at	Region	Dnajc14	74330	10	128542766	128556536	+
15456	1428531_at	Region	5930412E23Rik	77065	1	191312045	191358940	+
15457	1418017_at	Region	Pum2	80913	12	7990991	8066649	+
15458	1416146_at	Region	Hspa4	15525	11	53014415	53053230	-
15459	1450783_at	Region	Ifit1	15957	19	33983921	33992786	+
15460	1448434_at	Region	Rnf103	22644	6	71825871	71842858	+
15461	1419663_at	Region	Ogn	18295	13	49204931	49221360	+
15462	1446603_at	Region	None	None	NONE	NONE	NONE	NONE
15463	1434888_a_at	Region	Matr3	17184	18	35795510	35815074	+
15464	1451249_at	Region	D8Ertd812e	212528	8	83954671	83965285	+
15465	1438452_at	Region	Nebl	74103	2	17385579	17386998	-
15466	1448696_at	Region	Heph	15203	X	91056822	91175846	+
15467	1441789_at	Region	Phtf1	18685	3	103395581	103434755	+
15468	1438317_a_at	Region	Endog	13804	2	30103686	30106231	+
15469	1421916_at	Region	Pdgfra	18595	5	73986573	74032425	+
15470	1440660_at	Region	Nfia	18027	4	96752972	97087465	+
15471	1433562_s_at	Region	Atp5f1	11950	3	105738523	105755085	-
15472	1441315_s_at	Region	Slc19a2	116914	1	164168307	164184621	+
15473	1421351_at	Region	Gria4	14802	9	4328268	4706535	-
15474	1429884_at	Region	Srgap2	14270	1	131135970	131377678	-
15475	1416938_at	Region	Chchd1	66121	14	19064381	19065729	+
15476	1452228_at	Region	4930451A13Rik	67581	16	56073492	56138746	-
15477	1426911_at	Region	Dsc2	13506	18	20248374	20277081	-
15478	1453343_s_at	Region	Vrk2	69922	11	26366194	26488401	-
15479	1459205_at	Region	Cpeb1	12877	7	75155001	75262817	-
15480	1436212_at	Region	AI661017	213068	15	66550198	66585032	-
15481	1419160_at	Region	Golga3	269682	5	109227485	109273939	+
15482	1460364_at	Region	Gtf2ird1	57080	5	87703	186724	-
15483	1444378_at	Region	Csnk1d	104318	11	120784515	120812412	-
15484	1426419_at	Region	1700009P03Rik	74213	14	99658921	99720050	-
15485	1421660_at	Region	Scn9a	20274	2	66338406	66490352	-
15486	1435186_s_at	Region	1200008A14Rik	71710	3	14530640	14569495	+
15487	1455287_at	Region	Cdk6	12571	5	3350317	3528230	+
15488	1439269_x_at	Region	Mcm7 /// LOC433027 /// LOC436369	17220	5	137137397	137144671	-
15489	1425930_a_at	Region	Tcfl4	21428	11	100908397	100913294	+
15490	1439397_at	Region	BB164513	99456	2	113339682	113340006	+

15491	1455885_at	Region	6530401C20Rik	231842	5	139722959	139752142	+
15492	1426051_a_at	Region	Cenpb	12616	2	130691142	130693865	-
15493	1423088_at	Region	Tmod3	50875	9	75635361	75697149	-
15494	1439851_at	Region	Rabl5	67286	5	135921272	135926366	+
15495	1459304_at	Region	A730054J21Rik	320862	1	178891343	178892013	+
15496	1430021_a_at	Region	Uble1a	56459	7	13308414	13337163	-
15497	1429291_at	Region	Psmd1	70247	1	85870986	85945610	+
15498	1419213_at	Region	Nat6	56441	9	107645950	107649829	+
15499	1448268_at	Region	Tmed9	67511	13	54209701	54214246	+
15500	1423814_at	Region	Ddx41	72935	13	54146976	54153070	-
15501	1437171_x_at	Region	Gsn	227753	2	35214602	35240054	+
15502	1449079_s_at	Region	St3gal6	54613	16	57459955	57512884	-
15503	1426400_a_at	Region	Capns1	12336	7	25600378	25608047	-
15504	1419988_at	Region	Map3k7	26409	4	32242911	32299581	+
15505	1455175_at	Region	Phf13	230936	4	150481995	150484924	-
15506	1418274_at	Region	Nutf2	68051	8	105156479	105176249	+
15507	1456117_at	Region	2600005C20Rik	72462	17	29844491	29868935	+
15508	1451747_a_at	Region	Atg12l	67526	18	46954183	46963313	-
15509	1438865_at	Region	H13	14950	2	152126435	152163760	+
15510	1442056_at	Region	None	None	18	55197430	55197921	-
15511	1448814_at	Region	Gab1	14388	8	79976442	80092487	-
15512	1416132_at	Region	C920006C10Rik	76740	15	65810937	65897708	+
15513	1423786_at	Region	8430410A17Rik	232210	6	88350638	88373276	+
15514	1426601_at	Region	Slc37a1	224674	17	29103349	29158548	+
15515	1426612_at	Region	MGI:1921571	66131	9	64403798	64426983	+
15516	1439174_at	Region	9430047L24Rik	320850	3	140498710	140499375	+
15517	1455293_at	Region	Gm185	235497	9	75579103	75604011	+
15518	1416710_at	Region	Tmem35	67564	X	127840164	127850908	+
15519	1423578_at	Region	Col11a2	12815	17	31743993	31771080	+
15520	1433500_at	Region	B930096L08Rik	209773	6	39597808	39693264	-
15521	1419817_s_at	Region	D1Ertd161e	52231	1	75480104	75487168	+
15522	1457055_at	Region	Nubp1	26425	16	10086475	10098031	+
15523	1440456_at	Region	LOC381255	381255	1	53842507	54014298	-
15524	1425162_at	Region	Rorb	225998	19	18184597	18248073	-
15525	1432436_a_at	Region	Ak3	56248	19	28273429	28300499	-
15526	1452596_at	Region	Polr2k	17749	15	36175320	36178275	+
15527	1436599_at	Region	AW493225	101862	7	117531763	117532746	-
15528	1431420_s_at	Region	2610524G07Rik	66494	13	53930924	53934141	+
15529	1443271_at	Region	AW121567	270028	8	8571891	9138527	-
15530	1460725_at	Region	Xpa	22590	4	46091161	46112233	-
15531	1418607_at	Region	Zfp99	67235	5	144191673	144240274	-
15532	1434144_s_at	Region	2410187C16Rik	76773	15	58150452	58167670	+
15533	1453371_at	Region	4930535B03Rik	75137	3	95249480	95306695	-
15534	1427031_s_at	Region	D16Ertd480e	212514	16	43230787	43271716	+
15535	1433898_at	Region	Slc25a30	67554	14	70104494	70129532	-
15536	1420975_at	Region	Baz1b	22385	5	134200851	134258059	+
15537	1444163_at	Region	Nek5	330721	8	20825787	20877224	-
15538	1446614_at	Region	Dgkz	104418	2	91637575	91668348	-
15539	1451587_a_at	Region	Tiprl	226591	1	165134760	165159417	-
15540	1451493_at	Region	Ndfip1	65113	18	38640514	38688909	+
15541	1424406_at	Region	Bcl2l13	94044	6	121268204	121320977	+
15542	1460338_a_at	Region	Crlf3	54394	11	79772158	79806616	-
15543	1452646_at	Region	Trp53inp2	68728	2	154838793	154846784	+
15544	1455508_at	Region	A530082C11Rik	320541	4	154093778	154115702	+
15545	1444232_at	Region	Prkg1	19091	19	29820457	30038883	-
15546	1421030_at	Region	Zfp64	22722	2	168434104	168463992	-
15547	1419072_at	Region	Gstm7	68312	3	107722076	107727569	-
15548	1437830_x_at	Region	Zbed3	72114	13	91509451	91522055	+
15549	1456081_a_at	Region	Aacs	78894	5	124642786	124684316	+
15550	1451393_at	Region	Pex26	74043	6	146495249	146507767	-
15551	1445260_at	Region	Tes3	114893	3	35346474	35374678	-
15552	1423271_at	Region	Gjb2	14619	14	51629902	51635949	-
15553	1436650_at	Region	Filip1	70598	9	80020763	80183546	-
15554	1425070_at	Region	Ntrk3	18213	7	71981888	72367304	-
15555	1437577_at	Region	Usp34	17847	11	23201764	23383978	+

15556	1437944_at	Region	Shc2	216148	10	79740620	79759030	-
15557	1446360_at	Region	Rora	19883	9	69474948	69510983	+
15558	1451491_at	Region	4930556P03Rik	75284	15	99527766	99532411	-
15559	1435259_s_at	Region	D2Ertd217e	51875	2	25552229	25554164	-
15560	1417201_at	Region	Nt5c2	76952	19	46438257	46513006	-
15561	1434269_at	Region	AA536717	105428	14	18739182	18744804	+
15562	1440338_at	Region	None	None	NONE	NONE	NONE	NONE
15563	1423452_at	Region	Stk17b	98267	1	54063026	54092666	-
15564	1426902_at	Region	Coq6	217707	12	81230394	81242137	+
15565	1442163_at	Region	Hace1	209462	10	45739404	45873759	+
15566	1437816_at	Region	None	None	2	105379944	105380641	-
15567	1418714_at	Region	Dusp8	18218	7	136494291	136503065	-
15568	1438118_x_at	Region	Vim	22352	2	13489961	13498816	+
15569	1451630_at	Region	Ttl	69737	2	128579837	128607602	+
15570	1455510_at	Region	Spop	20747	11	95235201	95314100	+
15571	1428646_at	Region	2310056B04Rik	70402	1	168088096	168092493	-
15572	1422442_at	Region	Smu1	74255	4	40875969	40896722	-
15573	1432360_a_at	Region	4930403J22Rik	70551	14	117484003	117547608	-
15574	1459245_s_at	Region	Grid2	14804	6	64146694	64904112	+
15575	1456786_at	Region	Ldb2	16826	5	43229785	43558377	-
15576	1435247_at	Region	Ube1dc1	66663	9	104024992	104040598	-
15577	1435193_at	Region	A230050P20Rik	319278	9	20745994	20751594	+
15578	1434669_at	Region	Ralgps1	241308	2	33069135	33303640	-
15579	1458997_at	Region	A230106M20Rik	210094	7	37547672	37564754	-
15580	1436755_at	Region	Itih5	209378	2	10069730	10167804	+
15581	1433088_at	Region	5330430C04Rik	78274	18	25819803	25821121	-
15582	1439660_at	Region	Hivep3	16656	4	119003584	119157855	+
15583	1434613_at	Region	1810013L24Rik	69053	16	8502119	8528217	+
15584	1443874_at	Region	2810002I04Rik	72350	12	82156575	82167280	+
15585	1442491_at	Region	Dpp10	269109	1	123096743	123812573	-
15586	1446516_at	Region	Bcl7c	12055	7	121754709	121758497	-
15587	1443147_at	Region	None	None	12	80924181	80924817	+
15588	1429242_at	Region	1110054O05Rik	66209	4	59713082	59727302	-
15589	1435755_at	Region	1110001A16Rik	68554	17	76738764	76742542	+
15590	1435266_at	Region	9330112M16	329395	2	38998379	38999136	-
15591	1423829_at	Region	0910001A06Rik	223601	15	63945267	64076491	-
15592	1416841_at	Region	1110059E24Rik	66206	19	20835240	20890718	-
15593	1456205_x_at	Region	Tbca	21371	13	90973009	91026965	+
15594	1451387_s_at	Region	0610039D01Rik	67675	17	24733035	24734531	-
15595	1444276_at	Region	Ict1	68572	11	115224859	115232006	+
15596	1438810_at	Region	D10Ertd755e	52038	10	45582774	45583446	-
15597	1457115_at	Region	None	None	8	83326787	83327440	-
15598	1418561_at	Region	Sf3b1	81898	1	55286605	55333156	-
15599	1434060_at	Region	Herc1	235439	9	66474369	66632783	+
15600	1450228_a_at	Region	Pip5k1c	18717	10	81429153	81451529	+
15601	1437175_at	Region	Pdik1l	230809	4	133235901	133248731	-
15602	1422471_at	Region	Pex13	72129	11	23541269	23560725	-
15603	1455323_at	Region	MGI:1927369	57782	5	142231068	142239657	-
15604	1452490_a_at	Region	Ap2a2	11772	7	135965073	136035847	+
15605	1440029_at	Region	St8sia3	20451	18	64496670	64507426	+
15606	1431471_at	Region	4833428M15Rik	77030	14	92558801	92560822	-
15607	1431663_a_at	Region	Cntfr	12804	4	41796349	41835894	-
15608	1457190_at	Region	None	None	NONE	NONE	NONE	NONE
15609	1418187_at	Region	Ramp2	54409	11	101067518	101069332	+
15610	1423641_s_at	Region	Cnot7	18983	8	39436679	39454947	-
15611	1428256_at	Region	2310047H23Rik	69672	13	54331208	54342785	+
15612	1421697_at	Region	Ush2a	22283	1	187770861	187963478	+
15613	1423044_at	Region	Prosc	114863	8	25775882	25789406	+
15614	1418091_at	Region	Tcfcp2l1	81879	1	118379384	118442320	+
15615	1422565_s_at	Region	Nfic	18029	10	81536258	81554530	-
15616	1416014_at	Region	Abce1	24015	8	78898390	78925732	-
15617	1440685_at	Region	None	None	NONE	NONE	NONE	NONE
15618	1429332_at	Region	4632427E13Rik	68186	7	86778897	86781465	-
15619	1457298_at	Region	None	None	13	17585800	17587390	-
15620	1428107_at	Region	Sh3bgrl	56726	X	103696772	103763832	+

15621	1436013_at	Region	C230098I05Rik	269994	7	119930434	120132142	-
15622	1427160_at	Region	2500001H09Rik	74737	7	86684260	86709889	-
15623	1438666_at	Region	AI194318	241576	2	101654886	101891043	-
15624	1415984_at	Region	Acadm	11364	3	152924851	152946924	-
15625	1423479_at	Region	1500002M01Rik	68979	11	106987749	107010349	-
15626	1446835_at	Region	None	None	17	6016032	6016627	+
15627	1438090_x_at	Region	C730048E16Rik	223690	15	79104380	79114145	-
15628	1460035_at	Region	Bcap37	12034	6	125367716	125372298	+
15629	1444158_at	Region	Jarid1c	20591	X	145767667	145808191	+
15630	1423534_at	Region	Pdcd2	18567	17	13525230	13530933	-
15631	1420088_at	Region	None	None	12	52209583	52210002	-
15632	1416730_at	Region	Rcl1	59028	19	28353974	28396442	+
15633	1437573_at	Region	1110013G13Rik	74776	3	132200512	132268465	+
15634	1442495_at	Region	Birc6	12211	17	72336066	72510856	+
15635	1424124_at	Region	Mospd2	76763	X	158535574	158579740	-
15636	1437591_a_at	Region	Wdr1	22388	5	37274667	37309449	-
15637	1460734_at	Region	Col9a3	12841	2	180315202	180339143	+
15638	1431744_a_at	Region	Smap1	98366	1	24090771	24166945	-
15639	1438719_at	Region	AI585793	106920	18	32471916	32473517	+
15640	1451680_at	Region	Srxn1	76650	2	151562666	151568289	+
15641	1435908_at	Region	Nrxn2	18190	19	6319521	6322577	+
15642	1422630_at	Region	Rad50	19360	11	53402411	53460138	-
15643	1454773_at	Region	None	None	2	27692280	27695120	+
15644	1424008_a_at	Region	Rbpms2	71973	9	65752847	65782783	+
15645	1434895_s_at	Region	Ppp1r13b	21981	12	107308924	107388462	-
15646	1417406_at	Region	Sertad1	55942	7	22862956	22866317	+
15647	1450701_a_at	Region	Gtf2h2	23894	13	96620603	96648043	-
15648	1435637_at	Region	D8Wsu49e	71927	8	84991315	85113385	-
15649	1422178_a_at	Region	Rab17	19329	1	90777744	90789229	-
15650	1420696_at	Region	Sema3c	20348	5	15994279	16149731	+
15651	1421673_s_at	Region	Stx1b2 /// Stx1b1	56216	7	121857026	121874109	-
15652	1418932_at	Region	Nfil3	18030	13	51530722	51544552	-
15653	1435228_at	Region	BC023829	236848	X	65128250	65145669	-
15654	1416065_a_at	Region	Ankrd10	102334	8	10984009	11008115	-
15655	1455571_x_at	Region	Calm1	12313	12	95642228	95652493	+
15656	1423983_at	Region	Sez6l2	233878	7	121000294	121020334	+
15657	1436431_at	Region	1700025G04Rik	69399	1	151777069	151974583	-
15658	1435757_a_at	Region	Uqcrc2	67003	7	114510774	114534837	+
15659	1459557_at	Region	Zbtb16	235320	9	48684510	48863820	-
15660	1417886_at	Region	1810009A15Rik	66276	19	8085450	8087287	+
15661	1452368_at	Region	Bcr	110279	10	75162849	75286438	+
15662	1441666_at	Region	None	None	NONE	NONE	NONE	NONE
15663	1426441_at	Region	Slc11a2	18174	15	100445436	100469800	-
15664	1421305_x_at	Region	Rabep1	54189	11	70570583	70666598	+
15665	1425996_a_at	Region	Smarca3	20585	3	19399088	19457883	+
15666	1436645_a_at	Region	Cnot4	53621	6	35139222	35227761	-
15667	1429364_at	Region	4930579G24Rik	75939	3	79357237	79360959	+
15668	1428170_at	Region	Zfp180	210135	7	19255708	19281482	+
15669	1450366_at	Region	Bid3	12123	5	117298410	117313603	+
15670	1422272_at	Region	Phxr4	18689	9	13260619	13262009	+
15671	1416066_at	Region	Cd9	12527	6	126114754	126150059	-
15672	1451221_at	Region	BC018507	218333	13	66640392	66677128	-
15673	1453034_at	Region	Zfp251	71591	15	76903660	76922974	-
15674	1455826_a_at	Region	Bace1	23821	9	45851835	45875693	+
15675	1455108_at	Region	Eif4e2	26987	1	87030076	87054566	+
15676	1458202_at	Region	6330500D04Rik	193385	13	24152816	24157897	+
15677	1430017_at	Region	1600016N20Rik	72000	7	135612060	135616438	-
15678	1434530_at	Region	Odz4	23966	7	90212889	90948341	+
15679	1423823_at	Region	2610012O22Rik	69902	4	138228706	138233559	-
15680	1427375_at	Region	Rg9mtd2	108943	3	137032375	137047205	+
15681	1441707_at	Region	Psma3	19167	12	67809701	67829929	+
15682	1417135_at	Region	Srpk2	20817	5	21967253	22080468	-
15683	1435960_at	Region	LOC545923	545923	7	13898722	13905169	+
15684	1431335_a_at	Region	Wfdc1	67866	8	119020384	119040470	+
15685	1437034_x_at	Region	Marcks	17118	10	37207003	37211096	-

15686	1421472_at	Region	2900083I11Rik	58212	5	134820382	134855460	+
15687	1416964_at	Region	Eefsec	65967	6	88692712	88881898	-
15688	1427310_at	Region	Falz	207165	11	106856593	106978743	-
15689	1446735_at	Region	Sh3d1B	20403	12	3775688	3876446	+
15690	1446088_at	Region	9430081I23Rik	320242	13	28249229	28249932	-
15691	1435288_at	Region	Coro1a	12721	7	120749505	120754485	-
15692	1432524_at	Region	4930550G17Rik	75287	2	69741128	69743555	+
15693	1427897_s_at	Region	2400003N08Rik	71954	5	116220464	116244713	-
15694	1452914_at	Region	2410024N18Rik	70229	5	149532332	149535114	-
15695	1436747_at	Region	1110014K08Rik /// LOC545968	319370	11	116255306	116259636	+
15696	1452172_at	Region	2810421I24Rik	75619	1	64023669	64046403	+
15697	1428187_at	Region	Cd47	16423	16	48697237	48753271	+
15698	1417372_a_at	Region	Peli1	67245	11	21030262	21045116	+
15699	1440222_at	Region	Sod1	20655	16	89378174	89383756	+
15700	1430108_at	Region	9030622M22Rik	74547	8	60010682	60012667	-
15701	1427027_a_at	Region	Gtf3a	66596	5	145840404	145847296	+
15702	1458528_at	Region	C330002I19Rik	77480	12	21526422	21612277	+
15703	1458296_at	Region	Ext1	14042	15	53068219	53345629	-
15704	1425498_at	Region	Prpf4b	19134	13	34407672	34435208	+
15705	1438859_x_at	Region	Rps29	20090	12	65989514	65990859	-
15706	1416764_at	Region	Ttc11	66437	5	135975208	135979354	+
15707	1436210_at	Region	C330018K18Rik	235533	9	96018675	96082256	+
15708	1436895_at	Region	Centd1	212285	5	61409120	61555599	-
15709	1444130_at	Region	1700081L11Rik	76719	11	104154591	104263380	-
15710	1428771_at	Region	2410127E18Rik	76788	6	30449002	30501701	+
15711	1424017_a_at	Region	Hint1	15254	11	54619279	54623337	+
15712	1434574_at	Region	9430008C03Rik	68108	2	157812752	157815433	-
15713	1445420_at	Region	Mef2c	17260	13	79604158	79763912	+
15714	1446069_at	Region	D930036B08Rik	319713	18	62028204	62140635	-
15715	1417570_at	Region	Anapc1	17222	2	128126557	128201233	-
15716	1456290_x_at	Region	Ccm2	216527	11	6441744	6491532	+
15717	1451127_at	Region	AW146242	232023	6	57914676	57987537	-
15718	1423414_at	Region	Ptgs1	19224	2	36162689	36184424	+
15719	1460195_at	Region	Mrps11	67994	7	72575418	72585096	+
15720	1451981_at	Region	Gtrgeo22	110012	10	79792045	79798647	+
15721	1440155_at	Region	Fstl4	320027	11	52517547	52940188	+
15722	1456823_at	Region	None	None	12	73294080	73295580	+
15723	1417494_a_at	Region	Cp	12870	3	19298453	19333864	+
15724	1429484_at	Region	1110002L01Rik	68571	12	21189697	21191824	-
15725	1444441_at	Region	None	None	11	98674110	98674746	+
15726	1424006_at	Region	2310044P18Rik	69684	11	101227926	101238520	-
15727	1441348_at	Region	Zfp422-rs1	77652	17	30988427	31006091	+
15728	1446809_at	Region	B830045N13Rik	215378	1	146348134	146735852	+
15729	1454617_at	Region	Arrdc3	105171	NONE	NONE	NONE	NONE
15730	1436951_x_at	Region	Txndc9	98258	1	38279664	38291679	-
15731	1452945_at	Region	2610020C11Rik	72154	5	137412978	137432168	+
15732	1440825_s_at	Region	1700009P13Rik	215814	10	18141400	18159451	-
15733	1443974_at	Region	Plcl1	227120	1	55707080	56052282	+
15734	1437091_at	Region	Accn4	241118	1	75741422	75765063	+
15735	1434014_at	Region	Atg4cl	242557	4	98169007	98234860	+
15736	1428150_at	Region	Coro7	78885	16	4297661	4351229	-
15737	1417344_at	Region	2900064A13Rik	73024	2	112078263	112090674	+
15738	1452255_at	Region	Fbxo38	107035	18	62732972	62777526	-
15739	1443520_at	Region	None	None	NONE	NONE	NONE	NONE
15740	1428677_at	Region	Wdr73	71968	7	74693423	74704117	-
15741	1423398_at	Region	Taf12	66464	4	131071298	131090212	+
15742	1424072_at	Region	2010107G23Rik	69894	10	62074258	62077615	-
15743	1423772_x_at	Region	Slc25a5	11740	X	31416206	31419360	+
15744	1438255_at	Region	Ches1	71375	12	94622265	94816088	-
15745	1419542_at	Region	Dazl	13164	17	47811864	47826062	-
15746	1419803_s_at	Region	Ccdc12	72654	9	110697800	110752851	+
15747	1450556_at	Region	Spnb1	20741	12	73452717	73566610	-
15748	1451172_at	Region	1200015A19Rik	67808	4	152649850	152653028	-
15749	1417615_a_at	Region	Rpl11	67025	4	134931205	134934628	-

15750	1455939_x_at	Region	Srp14	20813	2	117989699	117993510	-
15751	1425344_at	Region	4430402O11Rik	67608	11	121058346	121076948	+
15752	1436510_a_at	Region	Lrrfip2	71268	9	111159058	111266964	+
15753	1428157_at	Region	Gng2	14702	14	18235904	18323407	-
15754	1428496_at	Region	Secisbp2	75420	13	50205381	50237727	+
15755	1435063_at	Region	None	None	4	137275353	137275963	-
15756	1446606_at	Region	D730045B01Rik	77867	5	150306312	150335251	-
15757	1448515_at	Region	Tsn	22099	1	118048034	118060649	-
15758	1457821_at	Region	2610511M17Rik	74206	7	24734241	24734571	-
15759	1443009_at	Region	None	None	6	88427101	88427749	+
15760	1458271_at	Region	None	None	9	9680542	9681032	-
15761	1421064_at	Region	Mpp5	56217	12	75605620	75694686	+
15762	1440905_at	Region	Hs2st1	23908	3	143402785	143541813	-
15763	1429538_a_at	Region	5730406M06Rik	66625	4	21916954	21945846	+
15764	1455706_at	Region	Stxbp4	20913	11	90301004	90459167	-
15765	1431893_a_at	Region	Tprt	56075	2	22857432	22877326	+
15766	1457427_at	Region	Gm573	230959	4	151904613	151975173	-
15767	1429389_at	Region	Setmar	74729	6	108566600	108578667	+
15768	1427730_a_at	Region	Zfp148	22661	16	32188918	32308456	+
15769	1428364_at	Region	Scnm1	69269	3	94617550	94621819	-
15770	1440740_at	Region	D2Ertd485e	329506	2	121470306	121527439	+
15771	1453766_a_at	Region	4931407K02Rik	77627	15	83915311	84113948	-
15772	1460329_at	Region	B4galt6	56386	18	20900931	20961332	-
15773	1430356_at	Region	2210402A03Rik	72280	2	22923798	22925595	-
15774	1445031_at	Region	None	None	1	39762189	39762932	-
15775	1433746_at	Region	Wdr3	269470	3	99564091	99588300	-
15776	1426518_at	Region	Tubgcp5	233276	7	50063625	50100924	+
15777	1418568_x_at	Region	Srp14	20813	2	117989699	117993510	-
15778	1427058_at	Region	Eif4a1	13681	11	69392597	69397973	-
15779	1442435_at	Region	None	None	11	74810860	74811395	+
15780	1434553_at	Region	4930577M16Rik	99887	3	119983678	120044986	-
15781	1443044_at	Region	A830091E24	414271	11	46045436	46046132	-
15782	1453448_at	Region	2310067E19Rik	76455	4	81440974	81444291	-
15783	1455606_at	Region	BC004022	80750	8	86124928	86125904	-
15784	1453093_at	Region	Rasgef1c	74563	11	49654692	49733064	+
15785	1455769_at	Region	None	None	9	7172707	7174201	-
15786	1424392_at	Region	Adhfe1	76187	1	9652849	9682760	+
15787	1420730_a_at	Region	Tcp11	21463	17	25862669	25876483	-
15788	1424753_at	Region	Nudt14	66174	12	108410830	108418215	-
15789	1417903_at	Region	Dfna5h	54722	6	50351935	50406301	-
15790	1416637_at	Region	Slc4a2	20535	5	22889216	22904842	+
15791	1429313_at	Region	Ror1	26563	4	99054696	99401450	+
15792	1452068_at	Region	Asahl	67111	5	91594004	91613926	-
15793	1436412_at	Region	None	None	2	83364553	83365467	+
15794	1443589_at	Region	DXErtd242e	52175	X	159854060	159854549	+
15795	1424154_a_at	Region	Hbld1	74316	12	81641945	81643760	+
15796	1415672_at	Region	Golga7	57437	8	21995926	22011591	-
15797	1431035_at	Region	Daam1	208846	12	68668036	68829803	+
15798	1460187_at	Region	Sfrp1	20377	8	22166110	22202536	+
15799	1433677_at	Region	Sfrs8	231769	5	128676882	128691989	+
15800	1428390_at	Region	Wdr43	72515	17	69392451	69435728	+
15801	1456583_x_at	Region	5730537D05Rik	70652	3	79543274	79572782	-
15802	1432944_at	Region	2900046L07Rik	73027	15	44662072	44663876	-
15803	1460602_at	Region	Dlc1	50768	8	35376686	35421146	-
15804	1452085_at	Region	Gatad1	67210	5	3645974	3653922	-
15805	1458532_at	Region	Mtr	238505	13	12226977	12293945	-
15806	1455521_at	Region	Klf12	16597	14	94399045	94678209	-
15807	1446065_at	Region	Kcnd2	16508	6	21263145	21776841	+
15808	1456479_at	Region	4732481H14Rik	209131	4	59749298	59843221	+
15809	1451063_at	Region	Stxbp4	20913	11	90301004	90459167	-
15810	1419917_s_at	Region	Tmed7	66676	18	46809246	46819270	-
15811	1434341_x_at	Region	1110020P15Rik	66152	11	4596760	4599115	-
15812	1428839_at	Region	Wdr53	68980	16	31056657	31066443	+
15813	1443400_at	Region	4933425I22Rik	71148	4	102074543	102121674	+
15814	1429679_at	Region	Lrrc17	74511	5	19995827	20028265	+

15815	1445329_at	Region	Dtnb	13528	12	2476	11394	+
15816	1449683_x_at	Region	2410129E14Rik	73710	13	33661517	33664863	-
15817	1429136_at	Region	1700052K11Rik	73431	11	105000913	105002522	-
15818	1456277_at	Region	7530414M10Rik	320374	NONE	NONE	NONE	NONE
15819	1433849_at	Region	Cdc27	217232	11	104326687	104371504	-
15820	1434770_at	Region	Iqcb1	320299	16	35645833	35690138	+
15821	1434513_at	Region	Gm542	224087	16	29120345	29123528	-
15822	1428849_at	Region	Rps6kb1	72508	11	86241975	86272766	-
15823	1458183_at	Region	None	None	NONE	NONE	NONE	NONE
15824	1418194_at	Region	Galnt10	171212	11	57371071	57513130	+
15825	1422117_s_at	Region	Khdrbs2	170771	1	32445071	32930005	+
15826	1422557_s_at	Region	Mt1	17748	8	93463399	93464496	+
15827	1439536_at	Region	AI846133	103847	11	74553328	74554192	+
15828	1448141_at	Region	1110014J01Rik	74778	15	83164108	83173438	-
15829	1420095_s_at	Region	Zipro1	22697	5	137090159	137106967	+
15830	1460243_at	Region	Sptlc2	20773	12	84187587	84267928	-
15831	1447948_at	Region	A430107O13Rik	214642	6	22056746	22284808	+
15832	1418847_at	Region	Arg2	11847	12	75987226	76012698	+
15833	1428907_at	Region	2600011C06Rik	67039	12	80506670	80547102	+
15834	1418915_at	Region	1810037K07Rik	67096	4	115661340	115667226	-
15835	1449863_a_at	Region	Dlx5	13395	6	6850562	6854825	-
15836	1428033_at	Region	None	None	NONE	NONE	NONE	NONE
15837	1416921_x_at	Region	Aldoa	11674	7	120844967	120848914	-
15838	1448477_at	Region	Chst12	59031	5	139504038	139524070	+
15839	1450638_at	Region	Pdcd5	56330	7	30805587	30811064	-
15840	1435029_at	Region	B230120H23Rik	65964	2	71983520	72140353	+
15841	1449966_s_at	Region	Cab39l	69008	14	53976044	54083966	+
15842	1450997_at	Region	Stk17b	98267	1	54063026	54092666	-
15843	1440787_s_at	Region	1300007O09Rik	71769	10	110921530	110929949	+
15844	1439609_at	Region	None	None	1	78502311	78502943	-
15845	1459628_at	Region	9630048M01Rik	320158	8	22424307	22817717	+
15846	1437958_at	Region	Xpr1	19775	1	155171522	155308066	-
15847	1452808_at	Region	Rnut1	66069	9	57064413	57096688	+
15848	1446847_at	Region	0610010D24Rik	68339	12	96358158	96474441	-
15849	1427542_at	Region	5330439J01Rik	109205	10	43127348	43280569	-
15850	1424254_at	Region	Ifitm1	68713	7	135297162	135371805	+
15851	1457677_at	Region	BC057593	240067	17	30818041	30830148	+
15852	1449252_at	Region	9030611O19Rik	104943	12	27650830	27656802	+
15853	1451195_a_at	Region	Txndc1	72736	12	67285315	67299785	+
15854	1440261_at	Region	Ap4e1	108011	2	126522583	126581762	+
15855	1437869_at	Region	3222402P14Rik	235542	9	101055227	101161626	-
15856	1451631_at	Region	BC021395	225283	18	24716439	24758615	-
15857	1425026_at	Region	SFT2 domain containing 2	108735	1	165097432	165117255	-
15858	1417516_at	Region	Ddit3	13198	10	127027446	127032923	+
15859	1431751_a_at	Region	2700082O15Rik	77015	2	106432845	106487143	+
15860	1433700_at	Region	4933433P14Rik	66787	12	101145518	101150041	+
15861	1425834_a_at	Region	Gpam	14732	19	54649974	54679691	-
15862	1431320_a_at	Region	Myo5a	17918	9	75209875	75357058	+
15863	1425616_a_at	Region	2410005K17Rik	69216	4	118154314	118160203	+
15864	1458309_at	Region	2900024P20Rik	208440	13	9260573	9652519	+
15865	1424166_at	Region	Msh3	17686	13	88508019	88521294	-
15866	1421925_at	Region	Mapk11	19094	15	89194712	89201863	-
15867	1422574_at	Region	Mxd4	17122	5	32664918	32676048	-
15868	1423441_at	Region	Tfb2m /// Gm1818	15278	1	179482301	179500463	-
15869	1423539_at	Region	Pms2	18861	5	142951583	142973057	+
15870	1417646_a_at	Region	Snx5	69178	2	143707062	143727467	-
15871	1452057_at	Region	Actr1b	226977	1	36993793	37004516	-
15872	1431442_at	Region	2310047B19Rik	66962	9	21845743	21848244	+
15873	1427557_at	Region	Alg12	223774	15	88857174	88871213	-
15874	1435475_at	Region	Lman2l	214895	1	36717777	36719487	-
15875	1425436_x_at	Region	Klra3	16634	6	131077951	131092195	-
15876	1448579_at	Region	Glg1	20340	8	110455266	110556791	-
15877	1428442_at	Region	Mmp24	17391	2	155232322	155273266	+
15878	1426717_at	Region	Nipa2	93790	7	50201064	50231927	-

15879	1457607_at	Region	AW046457	103409	NONE	NONE	NONE	NONE
15880	1456521_at	Region	None	None	1	136691336	136692204	-
15881	1455894_at	Region	A230072I16Rik	217057	11	86411975	86420315	+
15882	1416917_at	Region	1700123O20Rik	58248	14	49203053	49207567	+
15883	1429026_at	Region	Hexim2	71059	11	102954403	102960965	+
15884	1428405_at	Region	Hcfc1r1	353502	17	21475788	21477704	+
15885	1438362_x_at	Region	2310035C23Rik	227446	1	105555177	105695178	+
15886	1437268_at	Region	Lancl3	236285	X	7438913	7507025	+
15887	1449645_s_at	Region	Cct3	12462	3	88040999	88065630	+
15888	1457215_at	Region	None	None	4	153136457	153137079	+
15889	1427713_x_at	Region	Pou2f2	18987	7	20270879	20309756	-
15890	1451462_a_at	Region	Ifnar2	15976	16	90530631	90563104	+
15891	1441726_at	Region	Clasp2	76499	9	113865413	113971281	+
15892	1415695_at	Region	Psma1	26440	7	108067002	108132938	-
15893	1441977_at	Region	9630023C09Rik	320378	11	44494449	44494985	+
15894	1428756_at	Region	Aasdhppt	67618	9	4203626	4217037	-
15895	1424755_at	Region	Hip1	215114	5	134421518	134558607	-
15896	1434462_at	Region	BC088983	382010	8	55586179	55605223	-
15897	1435486_at	Region	Pak3	18481	X	137105791	137231857	+
15898	1455642_a_at	Region	Tspan17	74257	13	53396633	53403940	+
15899	1425327_at	Region	BC008163	230789	4	131861517	131883410	-
15900	1425899_a_at	Region	Itsn1	16443	16	90939437	91069253	+
15901	1454067_a_at	Region	4931406C07Rik	70984	9	15120247	15138698	-
15902	1436009_at	Region	Usp30	100756	5	113210959	113234147	+
15903	1452534_a_at	Region	Hmgb2	97165	8	56551079	56553720	+
15904	1423206_s_at	Region	2310003F16Rik	67693	2	120970950	120972288	+
15905	1437446_at	Region	Rab5b	19344	10	128414967	128433354	-
15906	1416765_s_at	Region	MGI:1913699	66449	16	4287970	4296450	-
15907	1456286_at	Region	Cog3	338337	14	70044865	70097010	-
15908	1426817_at	Region	Mki67	17345	7	130056691	130084009	-
15909	1431202_at	Region	Herc3	73998	6	58996163	59083005	+
15910	1427055_at	Region	4921507I02Rik	75778	3	12891	32732	+
15911	1442560_at	Region	Mgll	23945	6	89159883	89263412	+
15912	1445081_at	Region	A930041I02Rik	320271	2	39006211	39122887	-
15913	1417190_at	Region	Pbef1	59027	12	29426677	29457809	+
15914	1424639_a_at	Region	Hmgcl	15356	4	134827702	134843870	+
15915	1435230_at	Region	Ankrd12	106585	17	63676340	63693124	-
15916	1439467_at	Region	Mtap4	17758	9	109957034	110109905	+
15917	1421631_at	Region	Pcdhb1	93872	18	37488720	37491176	+
15918	1417752_at	Region	Coro1c	23790	5	112953083	113019350	-
15919	1435192_at	Region	None	None	X	55559962	55560671	-
15920	1448754_at	Region	Rbp1	19659	9	98322319	98345909	+
15921	1456295_at	Region	B230114P17Rik	402735	10	106821116	106821943	-
15922	1456364_at	Region	C230057M02Rik	319463	8	122106516	122136298	-
15923	1434539_at	Region	Lrrn3	16981	12	38061114	38095055	-
15924	1429209_at	Region	Col23a1	237759	11	51042761	51333951	+
15925	1455607_at	Region	Thsd2	72780	10	29483782	29567151	-
15926	1427317_at	Region	Kin	16588	2	9996726	10008815	+
15927	1452706_a_at	Region	1700027J05Rik	69440	15	89237134	89248812	-
15928	1447448_s_at	Region	None	None	13	5757516	5757917	+
15929	1428106_at	Region	1300001I01Rik	74148	11	74375253	74396505	+
15930	1430205_a_at	Region	Cdc37l1	67072	19	28243091	28270166	+
15931	1433862_at	Region	Espl1	105988	15	102381118	102384834	+
15932	1456070_at	Region	5430405N12Rik	71324	14	10701821	10702250	+
15933	1458899_at	Region	Usp53	99526	3	121722848	121773789	-
15934	1457184_at	Region	4930488L10Rik	319710	12	67659295	67737242	+
15935	1434825_at	Region	LOC381742	381742	5	141738255	141754415	-
15936	1417510_at	Region	Vps4a	116733	8	106329013	106343443	+
15937	1450015_x_at	Region	Sgpp1	81535	12	72570627	72592022	-
15938	1423715_a_at	Region	Nedd8	18002	14	50180280	50189888	-
15939	1450450_at	Region	Dscr1l2	53902	4	134293565	134306678	-
15940	1419502_at	Region	D11Lgp1e	80860	11	100587419	100592044	-
15941	1428803_at	Region	4632408A20Rik	217700	12	80965095	80975660	+
15942	1426641_at	Region	Trib2	217410	12	15150515	15175525	-
15943	1427146_at	Region	AI790298	107239	19	3953116	3954804	-

15944	1455619_at	Region	BC062258	408063	4	98890802	98891227	+
15945	1450885_at	Region	Dffa	13347	4	147596613	147610300	+
15946	1430833_at	Region	Itk	16428	11	46077990	46142337	-
15947	1439302_at	Region	5730469D23Rik	231380	5	85372730	85434753	-
15948	1425516_at	Region	Ogt	108155	X	96241425	96285710	+
15949	1448601_s_at	Region	Msx1	17701	5	36327006	36330969	-
15950	1426813_at	Region	2610020N02Rik	353258	10	13059813	13074795	-
15951	1426665_at	Region	Katnb1	74187	8	94365461	94384047	+
15952	1451188_at	Region	Wdr26	226757	1	181127684	181165201	-
15953	1429403_x_at	Region	Glt8d2	74782	10	82538402	82578619	-
15954	1451214_at	Region	Kbtbd2	210973	6	56921849	56924634	-
15955	1444709_at	Region	Invs	16348	4	48195745	48347875	+
15956	1437773_x_at	Region	Ddx17	67040	15	79580294	79599072	-
15957	1425067_at	Region	Celsr3	107934	9	108872851	108898805	+
15958	1422433_s_at	Region	Idh1	15926	1	65452617	65473274	-
15959	1455597_at	Region	AI585793	106920	18	32473196	32476895	+
15960	1436092_at	Region	None	None	15	75795559	75796148	-
15961	1450332_s_at	Region	Fmo5	14263	3	97115149	97138825	+
15962	1416891_at	Region	Numb	18222	12	80659465	80706369	-
15963	1420851_at	Region	Pard6g	93737	18	80170776	80242901	+
15964	1458053_at	Region	Abi2	329165	1	60713087	60784447	+
15965	1422194_at	Region	Scn5a	20271	9	119479491	119556933	-
15966	1436382_at	Region	Zbtb12	193736	17	32597418	32599703	+
15967	1449548_at	Region	Efnb2	13642	8	7983291	8026630	-
15968	1434081_at	Region	Ap1g1	11765	8	109076386	109159034	+
15969	1436559_a_at	Region	Psmd10	53380	X	134493477	134501620	-
15970	1418406_at	Region	Pde8a	18584	7	75020032	75142088	+
15971	1447842_x_at	Region	Tcn2	21452	11	3811977	3826370	-
15972	1428025_s_at	Region	Pitpnc1	71795	11	107033195	107291788	-
15973	1428654_at	Region	1200016B10Rik	66875	1	151223449	151284131	-
15974	1428710_at	Region	Rit1	19769	3	88460728	88473591	+
15975	1425238_at	Region	None	None	9	22220525	22221040	-
15976	1437433_at	Region	B3galt2	26878	1	143472806	143481893	+
15977	1456832_at	Region	Atrx	22589	X	100401518	100530707	-
15978	1426686_s_at	Region	Map3k3	26406	11	105918079	105976535	+
15979	1426647_at	Region	9130011J15Rik	66818	8	71716686	71721857	-
15980	1419172_at	Region	Dhfr	13361	13	88522409	88553836	+
15981	1445201_at	Region	Zfp53	24132	17	19358540	19379787	+
15982	1439556_at	Region	Ncam1	17967	9	49555319	49607941	-
15983	1422859_a_at	Region	Rpl23	65019	11	97598630	97603460	-
15984	1440867_at	Region	Spry4	24066	18	38809987	38824990	-
15985	1429600_at	Region	1110019K23Rik	68621	5	90247014	90341747	+
15986	1446233_at	Region	Adarb2	94191	13	8160097	8716266	+
15987	1434316_at	Region	Chsy1	269941	7	59993739	60058012	+
15988	1420861_at	Region	Dctn4	67665	18	60751547	60784088	+
15989	1453005_at	Region	Prpf31	68988	7	10280102	10281569	+
15990	1423678_at	Region	BC017643	217370	11	121043681	121050358	-
15991	1442397_at	Region	Nfx1	74164	4	41109832	41164029	+
15992	1435250_at	Region	2810013E07Rik	72656	4	11126123	11181194	-
15993	1434337_at	Region	Cklfsf4	97487	8	103648852	103690032	-
15994	1455494_at	Region	None	None	11	94773711	94774134	+
15995	1415992_at	Region	Pigo	56703	4	42933560	42941678	-
15996	1438675_at	Region	Sfrs8	231769	5	128676882	128691989	+
15997	1427600_at	Region	Tnfrsf19	29820	14	55499179	55581612	-
15998	1435591_at	Region	AI426330	101563	7	23592212	23593020	+
15999	1427024_at	Region	5033405K12Rik	75991	5	71731074	71761395	+
16000	1448830_at	Region	Dusp1	19252	17	24300425	24303295	-
16001	1424453_at	Region	Pcyt1a	13026	16	31261076	31281386	+
16002	1428246_at	Region	2310075A12Rik	69091	9	26901688	26924026	-
16003	1441396_at	Region	B3galt1	26877	2	67973343	67974323	+
16004	1423403_at	Region	Mapkbp1	26390	2	119486752	119539188	+
16005	1435574_at	Region	None	None	9	18157144	18158439	+
16006	1423378_at	Region	Adam23	23792	1	63739150	63885988	+
16007	1435143_at	Region	Elk3	13713	10	93221365	93283561	-
16008	1430187_at	Region	6330516O17Rik	76165	7	23126638	23127862	+

16009	1424643_at	Region	Tcof1	21453	18	61039868	61074234	-
16010	1417311_at	Region	Crip2	68337	12	108616717	108621647	+
16011	1415825_s_at	Region	1810073N04Rik	72055	11	119925064	119972367	-
16012	1417915_at	Region	Rap2b	74012	3	61043051	61046895	+
16013	1455054_a_at	Region	Tes3-ps /// Tes3	114893	3	35346474	35374678	-
16014	1460412_at	Region	1600015H20Rik	70370	2	128377831	128409656	+
16015	1452880_at	Region	Trip3	448850	11	84638947	84644348	-
16016	1439119_a_at	Region	BC010304	218236	13	48508119	48513447	-
16017	1424666_at	Region	5430405G24Rik	237943	11	102298304	102329534	-
16018	1458551_at	Region	None	None	13	79729799	79730830	+
16019	1421013_at	Region	Pitpnb	56305	5	110380456	110438052	+
16020	1428751_at	Region	Pacrg	69310	17	9043067	9480290	-
16021	1427148_at	Region	Pja2	224938	17	61986425	62035635	-
16022	1449058_at	Region	Gli1	14632	10	127066522	127078216	-
16023	1453173_at	Region	2310005E10Rik	67861	6	34478555	34491217	+
16024	1420495_a_at	Region	Vps26	30930	10	62421607	62453087	-
16025	1434491_a_at	Region	Cox6c	12864	15	35933506	35939687	-
16026	1455820_x_at	Region	Scarb1	20778	5	124438866	124503358	-
16027	1433448_at	Region	B430110G05Rik	229517	3	88154362	88168982	-
16028	1423212_at	Region	Phc1	13619	6	122978698	122997967	-
16029	1425429_s_at	Region	Hif3a	53417	7	13986645	14007590	-
16030	1424981_at	Region	Nln	75805	13	100241399	100327761	-
16031	1457038_at	Region	None	None	3	53148159	53149746	-
16032	1418536_at	Region	LOC386462	386462	17	1644305	1648794	+
16033	1436099_at	Region	AI836003	239650	15	98243222	98245696	+
16034	1436675_at	Region	Wdr63	242253	3	145014992	145083811	-
16035	1425649_at	Region	Slc39a14	213053	14	64619456	64667414	-
16036	1416662_at	Region	Sardh	192166	2	27121544	27178974	-
16037	1444402_at	Region	C230027N18Rik	330940	9	52184293	52185019	-
16038	1450028_a_at	Region	Lancl2	71835	6	57865048	57900894	+
16039	1457749_at	Region	Cc2d1b	319965	4	107578861	107593027	+
16040	1445742_at	Region	None	None	11	74651781	74652998	+
16041	1449017_at	Region	Nutf2	68051	8	105156479	105176249	+
16042	1456343_at	Region	Slc35f1	215085	10	52933079	53356820	+
16043	1458126_at	Region	D030041H20Rik	320036	14	23928909	23929270	+
16044	1416714_at	Region	Icsbp1	15900	8	120093722	120114040	+
16045	1452819_at	Region	Lphn3	319387	5	80275281	81049432	+
16046	1416421_a_at	Region	Ssb	20823	2	69717028	69727204	+
16047	1455076_a_at	Region	4933424B01Rik	71177	6	147470322	147498449	-
16048	1426481_at	Region	Klhl22	224023	16	16530639	16564348	+
16049	1418316_a_at	Region	Mark3	17169	12	107049977	107134146	+
16050	1445922_at	Region	Xrcc4	108138	13	85965021	86189500	-
16051	1443162_at	Region	None	None	4	57156503	57157176	-
16052	1447696_x_at	Region	Adcy5	224129	16	34087039	34087315	+
16053	1423217_a_at	Region	2510049I19Rik	67922	8	71378153	71382197	+
16054	1456711_at	Region	4932425I24Rik	320214	16	37114992	37159933	-
16055	1426027_a_at	Region	Arhgap10	78514	8	76458897	76659183	-
16056	1424362_at	Region	D830019K17Rik	227721	2	32027813	32042982	+
16057	1445248_at	Region	None	None	NONE	NONE	NONE	NONE
16058	1456240_x_at	Region	Cdca4	71963	12	108296070	108305225	-
16059	1458979_at	Region	None	None	7	57533643	57534253	+
16060	1452949_at	Region	Polr3b	70428	10	84517097	84621838	+
16061	1435613_x_at	Region	Cox5b	12859	1	36986129	36987976	+
16062	1454882_at	Region	L3mbtl3	237339	10	26275100	26374931	-
16063	1444007_at	Region	A830082K12Rik	320174	13	74299710	74300170	+
16064	1428452_at	Region	2810025M15Rik	69953	1	157317920	157325803	+
16065	1459014_at	Region	2900019G14Rik	72932	2	102490690	102491215	-
16066	1418763_at	Region	Nit2	52633	16	56061530	56071962	-
16067	1419357_at	Region	5830446M03Rik	57905	6	88255108	88275421	-
16068	1452316_at	Region	Phf22	71793	3	131982349	132001384	+
16069	1428831_at	Region	6230429P13Rik	76134	16	30769041	30771424	-
16070	1438015_at	Region	BC068171	245474	X	69756509	69770234	+
16071	1439177_at	Region	None	None	11	97522883	97523511	+
16072	1426990_at	Region	Cubn	65969	2	13192347	13407933	-
16073	1436750_a_at	Region	Oxct1	67041	15	3809284	3936636	+

16074	1430546_at	Region	Cryzl1	66609	16	90846621	90885763	-
16075	1421225_a_at	Region	Slc4a4	54403	5	88232301	88532904	+
16076	1418022_at	Region	Narg1	74838	3	51048093	51105995	+
16077	1423272_at	Region	Polg	18975	7	73245122	73263390	-
16078	1422842_at	Region	Xrn2	24128	2	146470058	146532016	+
16079	1434511_at	Region	Phkb	102093	8	85113615	85333255	+
16080	1423651_at	Region	Hbld2	69046	13	58415013	58429387	-
16081	1436898_at	Region	None	None	4	126053961	126055205	+
16082	1416024_x_at	Region	Cct3	12462	3	88040999	88065630	+
16083	1433010_at	Region	5530400N10Rik	71420	15	55694375	55695934	-
16084	1439189_at	Region	D630023B12Rik	329679	3	79184389	79228661	-
16085	1418278_at	Region	Apoc3	11814	9	46246303	46248549	-
16086	1423894_a_at	Region	Dalrd3	67789	9	108618891	108621769	+
16087	1423566_a_at	Region	Hsp105	15505	5	148555092	148574465	-
16088	1428474_at	Region	Ppp3cb	19056	14	18861352	18907856	-
16089	1426538_a_at	Region	Trp53	22059	11	69305639	69317529	+
16090	1455341_at	Region	2010003J03Rik	69860	19	5155603	5160301	+
16091	1431415_a_at	Region	Tbpl1	237336	10	22693541	22721040	-
16092	1430649_at	Region	Baalc	118452	15	38833124	38850257	+
16093	1450919_at	Region	Mpp1	17524	X	69770388	69791604	-
16094	1433869_at	Region	Zxdc	80292	6	90805539	90839567	+
16095	1435173_at	Region	Ate1	11907	7	124514765	124642811	-
16096	1449766_at	Region	Syt6	54524	3	103002769	103058318	+
16097	1436768_x_at	Region	E130112L23Rik	268739	14	47085678	47106098	+
16098	1418402_at	Region	Adam19	11492	11	45808889	45900180	+
16099	1427967_at	Region	Srgap2	14270	1	131135970	131377678	-
16100	1437256_at	Region	4833420K19Rik	76863	9	7176782	7200101	+
16101	1440291_at	Region	Insm1	53626	2	145679042	145681349	+
16102	1438835_a_at	Region	MGI:1336880	20624	11	102659566	102702006	-
16103	1431772_a_at	Region	Sh3d1B	20403	12	3775688	3876446	+
16104	1425505_at	Region	Mylk	107589	16	33677883	33783686	+
16105	1415934_at	Region	Cops8	108679	1	90422604	90432727	+
16106	1423708_a_at	Region	Farslb	23874	1	78713481	78777515	-
16107	1453762_at	Region	2310075A12Rik	69091	9	26901688	26924026	-
16108	1438067_at	Region	Nf1	18015	11	79065551	79307268	+
16109	1419189_at	Region	Vti1a	53611	19	54896591	55207543	+
16110	1422082_a_at	Region	Nfya	18044	17	45916830	45939727	-
16111	1437483_at	Region	Zfp513	101023	5	29657919	29661171	-
16112	1449027_at	Region	Rhou	69581	8	122941001	122950952	+
16113	1429081_at	Region	2600014C01Rik	70297	10	58247922	58263193	+
16114	1419288_at	Region	Jam2	67374	16	83882574	83932843	+
16115	1456453_at	Region	Evl	14026	12	104025433	104159229	+
16116	1447061_at	Region	None	None	7	13984958	13985225	-
16117	1430318_at	Region	Sat2	69215	11	69347699	69349528	+
16118	1428708_x_at	Region	2610009E16Rik	69202	6	125570449	125574707	-
16119	1422697_s_at	Region	None	None	13	44305979	44495797	+
16120	1435765_at	Region	E130114P18Rik	319865	4	96543212	96753121	-
16121	1440224_at	Region	9330133O14Rik	319574	8	121825243	121827234	+
16122	1417155_at	Region	Nmyc1	18109	12	12294434	12300049	-
16123	1428264_at	Region	Wdr57	66585	NONE	NONE	NONE	NONE
16124	1426856_at	Region	Hsdl2	72479	4	59525139	59561516	+
16125	1454654_at	Region	Dirc2 /// LOC547255	224132	16	34476226	34550765	-
16126	1454250_at	Region	Sec15l2	75914	6	84969806	85419115	-
16127	1447241_at	Region	H2afy3	404634	10	61704953	61750170	-
16128	1438487_s_at	Region	Zzz3	108946	3	151378383	151441249	+
16129	1419376_at	Region	1110018M03Rik	67606	2	109984712	109986780	-
16130	1444677_at	Region	C77673	97696	11	94135186	94135967	-
16131	1442532_at	Region	None	None	19	27252273	27252945	+
16132	1460571_at	Region	Dicer1	192119	12	100135572	100196088	-
16133	1429663_at	Region	3110023G01Rik	71206	18	77114915	77169011	-
16134	1435861_at	Region	Ppfia1	233977	7	138888626	138965593	-
16135	1438422_at	Region	Lrrc20	216011	10	61441266	61548546	+
16136	1448174_at	Region	Cul1	26965	6	47656010	47727829	+
16137	1421835_at	Region	Mtap7	17761	10	20157697	20207739	+

16138	1460556_at	Region	D15Mit260	27008	15	79178305	79188187	+
16139	1424602_s_at	Region	Xrcc4	108138	13	85965021	86189500	-
16140	1457924_at	Region	Mbnl1	56758	3	60179725	60308221	+
16141	1416226_at	Region	Arpc1b	11867	5	144160088	144166617	+
16142	1455058_at	Region	Mtmr9	210376	14	58054197	58074540	-
16143	1459249_at	Region	Tdrd3	219249	14	81829185	81957574	+
16144	1420093_s_at	Region	Hnrpdl	50926	5	99053360	99057516	-
16145	1448286_at	Region	Hadh2	15108	X	145536292	145538834	+
16146	1429546_at	Region	Ecgf1	72962	15	89424804	89429909	-
16147	1423574_s_at	Region	Srd5a2l	57357	5	75385516	75400710	+
16148	1417164_at	Region	Dusp10	63953	1	183531691	183573242	+
16149	1438717_a_at	Region	Osbpl6	99031	2	76246089	76293794	+
16150	1427732_s_at	Region	Abcg4	192663	9	44262045	44277099	-
16151	1431598_a_at	Region	Lhx9	16876	1	138672251	138686188	-
16152	1448838_at	Region	Topors	106021	4	40394151	40408676	-
16153	1450622_at	Region	Bcar1	12927	8	111008063	111041398	-
16154	1445361_at	Region	Ankrd12	67107	12	101	1172	+
16155	1419366_at	Region	Zmat5	67178	11	4599465	4632453	+
16156	1438556_a_at	Region	Tmod3	50875	9	75635361	75697149	-
16157	1448159_at	Region	Rab7	19349	6	88435785	88481888	-
16158	1441992_at	Region	Rab14	68365	2	35113675	35132954	-
16159	1422561_at	Region	Adamts5	23794	16	84962621	85000488	-
16160	1442350_at	Region	None	None	13	111247362	111248669	-
16161	1442901_at	Region	Tax1bp1	52440	6	52858073	52910809	+
16162	1439311_at	Region	B830012L14Rik	320085	12	105178883	105179473	+
16163	1450471_at	Region	Smad3	17127	9	63767269	63878762	-
16164	1424440_at	Region	Mrps6	121022	16	91215580	91269471	+
16165	1421216_a_at	Region	Ids	15931	X	65014764	65033475	-
16166	1453771_at	Region	Gulp1	70676	1	44850896	45102275	+
16167	1455585_at	Region	Rnf168	70238	16	31086860	31110277	+
16168	1423475_at	Region	Cnnm2	94219	19	46312725	46429008	+
16169	1450650_at	Region	Myo10	17909	15	25611655	25800954	+
16170	1428321_at	Region	Eml1	68519	12	103881621	104010277	+
16171	1440331_at	Region	9430079B08Rik	320481	1	106664272	106664657	-
16172	1422688_a_at	Region	Nras	18176	3	102486135	102496241	+
16173	1457881_at	Region	Osbpl6	99031	2	76246089	76293794	+
16174	1436306_at	Region	B430201G11Rik	243819	7	3876696	3903491	-
16175	1417075_at	Region	2010309E21Rik	66488	6	4013569	4015176	+
16176	1433872_at	Region	2410042D21Rik	72425	2	112002449	112037475	+
16177	1436522_at	Region	0610025L06Rik	67803	11	105977345	105981231	-
16178	1429402_at	Region	Glt8d2	74782	10	82538402	82578619	-
16179	1455252_at	Region	Tsc1	64930	2	28573403	28619515	+
16180	1433749_at	Region	Gna13	14674	11	109184029	109219306	+
16181	1424950_at	Region	Sox9	20682	11	112603317	112608842	+
16182	1423750_a_at	Region	Zfp162	22668	19	6152878	6165003	+
16183	1424712_at	Region	Ahctf1	226747	1	179700337	179757911	-
16184	1419924_at	Region	None	None	11	54270443	54271077	-
16185	1451124_at	Region	Sod1	20655	16	89378174	89383756	+
16186	1430062_at	Region	1600002O04Rik	214305	12	103777190	103800577	+
16187	1457529_x_at	Region	2810021G02Rik	353208	NONE	NONE	NONE	NONE
16188	1441075_at	Region	LOC329416	329416	2	68991247	69044726	+
16189	1431646_a_at	Region	Stx6	58244	1	155049479	155094254	+
16190	1458539_at	Region	R3hdm	226412	1	127946018	128080726	+
16191	1425410_at	Region	1810034M08Rik	69786	6	86345388	86360039	+
16192	1451658_a_at	Region	Polr3c	74414	3	96199326	96215464	-
16193	1437001_at	Region	Gsk3b	56637	16	36906890	37063968	+
16194	1416660_at	Region	Eif3s10	13669	19	60361493	60391011	-
16195	1437617_x_at	Region	1110034G24Rik	73747	2	132192859	132264907	+
16196	1450295_s_at	Region	Pvr	52118	7	633	12546	-
16197	1420091_s_at	Region	Morc3	338467	16	93003378	93047429	+
16198	1439478_at	Region	MGI:2159605	171210	12	80852219	80858208	+
16199	1425061_at	Region	Wasf3	245880	5	145270584	145355994	+
16200	1425820_x_at	Region	Gpatc4	66614	3	87786988	87799852	+
16201	1448190_at	Region	Mrpl33	66845	5	30072910	30081579	+
16202	1456517_at	Region	Tmem44	224090	16	29326754	29359563	-

16203	1437995_x_at	Region	38602	235072	9	25147069	25202879	+
16204	1431430_s_at	Region	Trim59	66949	3	68705814	68715182	-
16205	1428753_a_at	Region	Dgcr6	13353	16	16823817	16842299	+
16206	1436570_at	Region	None	None	3	9684305	9685669	-
16207	1440913_at	Region	BC037704	269087	19	43219589	43220659	+
16208	1436010_at	Region	BC036313	268747	14	50024964	50025797	+
16209	1416340_a_at	Region	Man2b1	17159	8	84354016	84368320	+
16210	1448617_at	Region	Cd53	12508	3	106555761	106585879	-
16211	1437470_at	Region	Pknox1	18771	17	29391575	29415568	+
16212	1454286_at	Region	1110004M10Rik	68464	NONE	NONE	NONE	NONE
16213	1439319_at	Region	Elf1	13709	14	73835241	73936523	+
16214	1433626_at	Region	Plscr4	235527	9	92348147	92383290	+
16215	1445864_at	Region	1500032D16Rik	78330	17	29329211	29340379	+
16216	1422136_at	Region	Uhmk1	16589	1	170133433	170149570	-
16217	1426475_at	Region	Hmbs	15288	9	44325203	44333042	-
16218	1437456_x_at	Region	Ythdf1	228994	2	180621335	180637894	-
16219	1425825_at	Region	C230094A16Rik	237711	11	29638924	29659023	-
16220	1439678_at	Region	None	None	1	52527483	52528166	-
16221	1418999_at	Region	2310033P09Rik	67862	11	58933990	58936365	+
16222	1456052_at	Region	Cdkl3	213084	11	51757190	51780399	+
16223	1423709_s_at	Region	Farslb	23874	1	78713481	78777515	-
16224	1460253_at	Region	Cklfsf7	102545	9	114730469	114755551	-
16225	1447667_x_at	Region	Map3k4 /// LOC433718	26407	17	10867071	10957848	-
16226	1428488_at	Region	Pigk	329777	3	151707256	151782191	+
16227	1452591_a_at	Region	2410018G20Rik	72083	16	14616558	14631439	-
16228	1457246_at	Region	Rai16	239170	14	64899284	64918304	-
16229	1441112_at	Region	5730454B08Rik	70579	1	133482838	133516350	-
16230	1443049_at	Region	Tmem19	67226	10	115027802	115049241	-
16231	1426722_at	Region	Slc38a2	67760	15	96755014	96767320	-
16232	1423117_at	Region	Pum1	80912	4	129460274	129578157	+
16233	1416610_a_at	Region	Clcn3	12725	8	59960629	60030782	-
16234	1454978_at	Region	Ttyh3	78339	5	139619472	139647856	-
16235	1445830_at	Region	None	None	2	84309625	84310487	-
16236	1424932_at	Region	Egfr	13649	11	16646996	16808700	+
16237	1436445_at	Region	Gm996	381353	2	25507592	25512261	-
16238	1421270_at	Region	Sh3md2	59009	8	60271818	60461252	+
16239	1451252_at	Region	Irf2bp1	272359	7	15872826	15875524	+
16240	1423676_at	Region	Atp5h	71679	11	115236790	115241012	-
16241	1447408_at	Region	None	None	15	8211365	8211786	-
16242	1419316_s_at	Region	Gnb1l	13972	16	17269870	17335574	+
16243	1455220_at	Region	Frat2	212398	19	41390350	41392504	-
16244	1458314_at	Region	None	None	2	102483962	102484232	-
16245	1457345_at	Region	Polr3b	70428	10	84517097	84621838	+
16246	1456350_at	Region	Zfp451	98403	1	34030702	34083136	-
16247	1448941_at	Region	B4galt2	53418	4	116828165	116842060	-
16248	1437619_x_at	Region	Ddr1	12305	17	33397888	33421229	-
16249	1428190_at	Region	Slc25a1	13358	16	16696186	16699355	-
16250	1448202_x_at	Region	2610524G07Rik	66494	13	53930924	53934141	+
16251	1429214_at	Region	Adamtsl2	77794	2	27011543	27040775	+
16252	1457316_at	Region	Mtap6	17760	7	93358278	93425764	+
16253	1415721_a_at	Region	1200013P24Rik	74763	16	3556247	3576330	+
16254	1441191_at	Region	2810454H06Rik	72813	6	135657588	135658254	+
16255	1441274_at	Region	Casc3	192160	11	98625992	98654894	+
16256	1439970_at	Region	Prkwnk1	232341	6	120356763	120470204	-
16257	1446127_at	Region	Zfhx1a	21417	18	5709786	5817483	+
16258	1419243_at	Region	Rab14	68365	2	35113675	35132954	-
16259	1460717_at	Region	Tspyl1	22110	10	34343170	34345813	+
16260	1452745_at	Region	1810044A24Rik	76510	15	72616617	73082807	-
16261	1444439_at	Region	Bri3bp	76809	5	124608455	124621964	+
16262	1416279_at	Region	Ap1b1	11764	11	4881756	4937518	+
16263	1448222_x_at	Region	Cox8a	12868	19	6929007	6931380	-
16264	1434412_x_at	Region	Stub1	56424	17	23636797	23639522	-
16265	1448986_x_at	Region	Dnase2a	13423	8	84176120	84182800	+
16266	1454350_at	Region	Pdzk6	380614	3	40073399	40124437	+

16267	1436194_at	Region	C330008K14Rik	77619	18	42096231	42171850	-
16268	1427276_at	Region	Smc4l1	70099	3	68675494	68705145	+
16269	1428814_at	Region	None	None	17	30133243	30134192	+
16270	1416974_at	Region	Stam2	56324	2	52624368	52674311	-
16271	1416427_at	Region	Ccni	12453	5	92517687	92541778	-
16272	1460259_s_at	Region	Clca1 /// Clca2	12722	3	143701991	143732414	-
16273	1454642_a_at	Region	Commd3	12238	2	18714472	18718226	+
16274	1440941_at	Region	LOC552912	552912	11	49874351	49875017	+
16275	1452960_at	Region	Scyl3	240880	1	163847656	163872719	+
16276	1449431_at	Region	Trpc6	22068	9	8548619	8691692	+
16277	1440123_at	Region	None	None	X	145799131	145800126	+
16278	1443534_at	Region	None	None	3	60191372	60191815	+
16279	1454982_at	Region	Arfgef2	99371	2	166291195	166354676	+
16280	1448918_at	Region	Slco3a1	108116	7	68181298	68451736	-
16281	1451997_at	Region	Zfp426	235028	9	20341367	20365016	-
16282	1421721_a_at	Region	Arnt	11863	3	94936201	94983401	+
16283	1444109_at	Region	C130009A20Rik	399614	12	47365270	47365911	-
16284	1435409_at	Region	None	None	10	68837300	68837958	+
16285	1416182_at	Region	Apba3	57267	10	81404370	81409313	+
16286	1418872_at	Region	Abcb1b	18669	5	8804929	8873051	+
16287	1435101_at	Region	Derl2	116891	11	70733103	70744921	-
16288	1423386_at	Region	Psmd9	67151	5	122391343	122411976	+
16289	1423598_at	Region	Atp8a1	11980	5	66388740	66614923	-
16290	1425551_at	Region	Hip1r	29816	5	123144623	123174525	+
16291	1448178_a_at	Region	Cct3	12462	3	88040999	88065630	+
16292	1452313_at	Region	5930416I19Rik	72440	6	129047547	129053009	-
16293	1454611_a_at	Region	Calm1	12313	12	95642228	95652493	+
16294	1436083_at	Region	LOC435965	435965	7	30363538	30367683	-
16295	1423528_at	Region	Bcas3	192197	11	85081206	85554043	+
16296	1429980_x_at	Region	1810073N04Rik	72055	11	119925064	119972367	-
16297	1445267_at	Region	None	None	NONE	NONE	NONE	NONE
16298	1421809_at	Region	Dgcr2	13356	16	16612273	16662559	-
16299	1448410_at	Region	Ube4b	63958	4	147820778	147919111	-
16300	1417212_at	Region	9530058B02Rik	68241	17	23669857	23674885	-
16301	1428857_at	Region	2610304F09Rik	269003	18	31874527	31963205	+
16302	1418433_at	Region	Cab39	12283	1	85601719	85656721	+
16303	1423131_at	Region	5730427N09Rik /// LOC433230 /// LOC545264	433230	19	21678941	21680624	+
16304	1434973_at	Region	Car7	12354	8	103833942	103843478	+
16305	1441117_at	Region	None	None	3	88781825	88782901	+
16306	1460391_at	Region	2810409H07Rik	67059	2	72790552	72912196	-
16307	1431163_at	Region	2700046G09Rik	67188	19	31714681	31716657	+
16308	1449527_at	Region	Pcdhb7	93878	18	37565424	37568924	+
16309	1434191_at	Region	A530016O06Rik	319660	12	33844550	34007192	+
16310	1431784_a_at	Region	Bxdc5	70285	3	145482969	145497969	-
16311	1426333_a_at	Region	Ikbkb	16150	8	21414342	21461142	-
16312	1427595_at	Region	Acaca	107476	11	84006901	84040802	+
16313	1451547_at	Region	0610009A07Rik	70337	10	6951826	6966424	-
16314	1429595_at	Region	2700049A03Rik	76967	12	67971858	68080085	+
16315	1424993_at	Region	8430410K20Rik	78100	9	4293956	4297903	+
16316	1449173_at	Region	Mpp2	50997	11	101878106	101909589	-
16317	1460053_at	Region	Smyd4	319822	11	75074254	75131344	+
16318	1444298_at	Region	A130090K04Rik	320495	10	3613859	3614698	+
16319	1455507_s_at	Region	D8Ertd587e	52335	8	109024127	109027469	-
16320	1434945_at	Region	A330042H22	270084	8	92142468	92206397	+
16321	1441969_at	Region	Trim36	28105	18	46386470	46433150	-
16322	1438147_at	Region	Srcrb4d	109267	5	134973716	134981543	-
16323	1442153_at	Region	None	None	NONE	NONE	NONE	NONE
16324	1417203_at	Region	Ethe1	66071	7	19769996	19785025	+
16325	1444317_at	Region	LOC546479	546479	10	74737456	74741374	+
16326	1456681_at	Region	AI848149	102944	X	149195889	149196608	-
16327	1417559_at	Region	Sfxn1	14057	13	52636174	52672650	+
16328	1452047_at	Region	Cacybp	12301	1	160107568	160117424	-
16329	1428357_at	Region	2610019F03Rik	72148	8	13330624	13353305	-

16330	1439369_x_at	Region	Slc9a3r2	65962	17	22442873	22453949	-
16331	1448563_at	Region	Phb	18673	11	95488102	95501852	+
16332	1431107_at	Region	Stk35	67333	2	129314605	129341840	+
16333	1448279_at	Region	Arpc3	56378	5	121544278	121558528	+
16334	1460402_at	Region	Brpf1	78783	6	113874964	113892542	+
16335	1423533_a_at	Region	Rhot1	59040	11	79934743	79993145	+
16336	1421887_a_at	Region	Aplp2	11804	9	31068390	31130767	-
16337	1455243_at	Region	Brpf3	268936	17	26602697	26638455	+
16338	1422715_s_at	Region	Acp1	11431	12	27469324	27487670	-
16339	1435042_at	Region	9130004C02Rik	320594	9	25145503	25146631	-
16340	1434676_at	Region	Mtmr9	210376	14	58054197	58074540	-
16341	1418617_x_at	Region	Clgn	12745	8	82637493	82683940	+
16342	1442916_at	Region	None	None	8	67158135	67158773	-
16343	1421340_at	Region	Map3k5	26408	10	19860944	20067353	+
16344	1433015_at	Region	6330436F06Rik	76733	6	21322082	21323460	+
16345	1455580_at	Region	Usp6nl	98910	2	6270005	6360769	+
16346	1424293_s_at	Region	2610319K07Rik	72519	4	14791184	14842225	+
16347	1451038_at	Region	Apln	30878	X	42545942	42555630	-
16348	1418181_at	Region	Ptp4a3	19245	15	73776930	73787284	+
16349	1423346_at	Region	Degs1	13244	1	182232966	182239955	-
16350	1426217_at	Region	2810441K11Rik	68642	19	9746963	9752737	-
16351	1441459_at	Region	None	None	3	44728729	44729421	-
16352	1457318_at	Region	A330008L17Rik	234624	8	98707406	98715396	+
16353	1445370_at	Region	None	None	2	173246449	173247097	+
16354	1418461_at	Region	Sh3d19	27059	3	85819530	85872864	+
16355	1428764_at	Region	Gtl2	17263	12	105023823	105039917	+
16356	1435775_at	Region	Tparl	21982	5	75429189	75454483	+
16357	1459560_at	Region	4921517N04Rik	76857	2	23449966	23450521	-
16358	1430875_a_at	Region	Pak1ip1	68083	13	40561850	40573601	+
16359	1455563_at	Region	LOC234374	234374	8	69446573	69455569	-
16360	1440349_at	Region	Dmtf1	23857	5	9125544	9168200	-
16361	1431820_at	Region	4632404H12Rik	74034	3	89573694	89579423	-
16362	1418960_at	Region	Phf20l1	239510	15	66601624	66648123	+
16363	1418369_at	Region	Prim1	19075	10	127751925	127766740	+
16364	1425314_at	Region	Mass1	110789	13	77171389	77706926	-
16365	1444661_at	Region	Gpr26	233919	7	126330320	126349493	+
16366	1429724_at	Region	Prpf4	70052	4	61500023	61515662	+
16367	1455568_at	Region	2310015A05Rik	72315	16	16417438	16421706	+
16368	1415767_at	Region	Ythdf1	228994	2	180621335	180637894	-
16369	1430392_at	Region	9530086O07Rik	78741	6	101791069	101792336	-
16370	1446957_s_at	Region	BC004022	80750	8	86127817	86128370	-
16371	1425358_at	Region	Riok1	71340	13	37576092	37600226	+
16372	1434079_s_at	Region	Mcm2	17216	6	89318530	89333755	-
16373	1443860_at	Region	Ptprd	19266	4	75201576	75510952	-
16374	1445885_at	Region	Ube2d2	56550	18	35995272	36030828	+
16375	1418373_at	Region	Pgam2	56012	11	5696431	5698520	-
16376	1419662_at	Region	Ogn	18295	13	49204931	49221360	+
16377	1456189_x_at	Region	Ltbp3	16998	19	5530917	5547315	+
16378	1455533_at	Region	A930025D01Rik	319513	2	129932077	129938125	-
16379	1424144_at	Region	Ris2	67177	8	121949602	121954717	+
16380	1445375_at	Region	Sh3d1B	20403	12	3775688	3876446	+
16381	1429144_at	Region	2310032D16Rik	74182	2	132043251	132092031	-
16382	1447024_at	Region	BC055915	211660	1	10178667	10241764	+
16383	1428725_at	Region	Miz1	17344	18	77186923	77275518	+
16384	1434063_at	Region	AW060232	269704	5	124022903	124049431	+
16385	1444687_at	Region	C1ql2	226359	1	120098991	120101583	+
16386	1434004_at	Region	Dhps	330817	8	84342639	84345999	+
16387	1432582_at	Region	3110054G05Rik	73239	6	38668788	38670089	-
16388	1417026_at	Region	Pfdn1	67199	18	36627481	36678297	-
16389	1441213_at	Region	BC021891	234878	8	125197983	125232857	+
16390	1433535_x_at	Region	Cct2	12461	10	116740451	116753217	-
16391	1418795_at	Region	Cds2	110911	2	131777151	131821546	+
16392	1423633_at	Region	6530403A03Rik	67797	13	37743845	37765948	+
16393	1454231_a_at	Region	Rpgrip1	77945	14	47211021	47261035	+
16394	1448547_at	Region	Rassf3	192678	10	121066604	121132504	-

16395	1441480_at	Region	None	None	12	51916663	51917121	+
16396	1458039_at	Region	Ncoa3	17979	2	165449481	165527179	+
16397	1435050_at	Region	D10Bwg1379e	215821	10	18490148	18668026	-
16398	1452739_at	Region	Fbxo7	69754	10	85982941	86009273	+
16399	1452884_at	Region	Sfrs2ip	72193	15	96481149	96528137	-
16400	1429505_at	Region	2310076G13Rik	71938	1	60638785	60641619	+
16401	1449406_at	Region	Cyhr1	54151	15	76696649	76710731	-
16402	1434910_at	Region	A830080D01Rik	382252	X	153166512	153192473	+
16403	1453995_a_at	Region	Htf9c	15547	16	17019721	17024544	+
16404	1426809_at	Region	C430004E15Rik	97031	2	25194780	25202044	+
16405	1428652_at	Region	0610010F05Rik	71675	11	23468571	23528411	-
16406	1418015_at	Region	Pum2	80913	12	7990991	8066649	+
16407	1416984_at	Region	Mrps18a	68565	17	43620925	43638829	+
16408	1426394_at	Region	Eif3s1	78655	2	121561316	121567484	+
16409	1437307_at	Region	Senp8	71599	9	59852773	59867496	-
16410	1429488_at	Region	Zdhhc21	68268	4	81788755	81840307	-
16411	1449515_at	Region	Zfp292	30046	4	35020884	35026191	-
16412	1443933_at	Region	Mtac2d1	74413	12	97092021	97157363	-
16413	1417426_at	Region	Prg1	19073	10	62460763	62474090	-
16414	1429916_at	Region	9530077C05Rik	68283	9	22301973	22335077	+
16415	1421869_at	Region	Trim44	80985	2	102009026	102105530	-
16416	1415868_at	Region	Cct4	12464	11	22885383	22898126	+
16417	1433514_at	Region	Etnk1	75320	6	144013618	144053017	+
16418	1436672_at	Region	None	None	19	6722174	6722675	-
16419	1430317_at	Region	Ube2j2	140499	4	154436212	154451966	+
16420	1421550_a_at	Region	Trim34 /// LOC434218	434218	7	98356814	98374591	+
16421	1418133_at	Region	Bcl3	12051	7	16677706	16681757	-
16422	1442338_at	Region	None	None	4	153410697	153410928	-
16423	1442388_at	Region	2410137F16Rik	76798	5	115584252	115585180	-
16424	1427049_s_at	Region	Smo	319757	6	29782600	29808456	+
16425	1452053_a_at	Region	Tmem33	67878	5	66026818	66052665	+
16426	1458257_at	Region	None	None	5	30397662	30398180	+
16427	1419503_at	Region	Stc2	20856	11	31254231	31264851	-
16428	1423903_at	Region	Pvr	52118	7	633	12546	-
16429	1456022_at	Region	Hipk2	15258	6	38834084	39012133	-
16430	1433548_at	Region	Mare	17168	11	32127213	32162408	-
16431	1458943_at	Region	None	None	5	15042173	15042826	+
16432	1431310_s_at	Region	4833412E22Rik	330010	4	154536681	154551706	-
16433	1424662_at	Region	Pmpca	66865	2	26321510	26329283	+
16434	1439106_at	Region	Zfp462	242466	4	54936056	54997483	+
16435	1449209_a_at	Region	Rdh11	17252	12	76030739	76048695	-
16436	1438766_at	Region	Pnrc2	52830	4	134752186	134755084	-
16437	1425039_at	Region	Itgbl1	223272	14	118218981	118502984	+
16438	1429947_a_at	Region	Zbp1	58203	2	172667079	172679357	-
16439	1436373_at	Region	Map3k10	269881	7	23031316	23063618	-
16440	1438834_at	Region	Mospd2	76763	X	158535574	158579740	-
16441	1428413_at	Region	5730405I09Rik	67974	18	9356614	9490220	-
16442	1437288_at	Region	1110001C20Rik	242291	4	4691488	4720452	-
16443	1450159_at	Region	Rem1	19700	2	152083944	152092127	+
16444	1426584_a_at	Region	Sdh1	20322	2	121748692	121779190	+
16445	1417731_at	Region	Pqbp1	54633	X	6133463	6138168	-
16446	1435997_at	Region	Spag8	433700	4	43567651	43569474	-
16447	1445143_at	Region	G630009D10Rik	238328	12	83552631	83566022	+
16448	1418598_at	Region	Ubox5	140629	2	130103855	130143839	-
16449	1429074_at	Region	1700026D08Rik	75556	7	77632049	77651256	-
16450	1429395_at	Region	4933434L15Rik	67553	3	131872946	131982136	-
16451	1449574_a_at	Region	Cdc42	12540	4	136200964	136217395	-
16452	1418268_at	Region	Htr3a	15561	9	48927172	48938927	-
16453	1418448_at	Region	Rras	20130	7	39094549	39098186	+
16454	1436551_at	Region	Fgfr1	14182	8	24257087	24299020	+
16455	1445137_at	Region	Ptk2	14083	15	73233486	73423105	-
16456	1423697_at	Region	Psmd6	66413	14	12574333	12583052	-
16457	1423962_at	Region	Wdr26	226757	1	181127684	181165201	-
16458	1425197_at	Region	Ptpn2	19255	18	67896299	67955363	-

16459	1449219_at	Region	Fads3	60527	19	9238187	9256168	+
16460	1417924_at	Region	Pak3	18481	X	137105791	137231857	+
16461	1434239_at	Region	AA408556	107094	19	41407223	41440525	-
16462	1438659_x_at	Region	Chchd6	66098	6	89818799	90031255	-
16463	1456199_x_at	Region	Ranbp9	56705	13	42965819	43043413	-
16464	1451256_at	Region	Gpr172b	52710	15	76589887	76593074	+
16465	1441435_at	Region	Tbl1x	21372	X	72294199	72320920	+
16466	1446410_at	Region	2010308M01Rik	72121	3	106281879	106296871	+
16467	1448320_at	Region	Stim1	20866	7	96373782	96541218	+
16468	1449724_s_at	Region	D8Ertd738e	101966	8	83509311	83512777	-
16469	1427661_a_at	Region	Tssc4	56844	7	137482387	137484106	+
16470	1424880_at	Region	Trib1	211770	15	59657966	59666411	+
16471	1438050_x_at	Region	---	272713	NONE	NONE	NONE	NONE
16472	1452386_at	Region	Sall3	20689	18	81089711	81109930	-
16473	1445342_at	Region	None	None	X	55857372	55858036	+
16474	1441460_at	Region	Fgfr1op2	67529	6	147498625	147519880	+
16475	1427692_a_at	Region	Cask	12361	X	11759987	12085496	-
16476	1416965_at	Region	Pcsk1n	30052	X	6158762	6162175	+
16477	1423421_at	Region	MGI:1930842	56503	9	14613638	14616404	-
16478	1430300_at	Region	Sco1	52892	11	66778320	66792720	+
16479	1428974_s_at	Region	Lztfl1	93730	9	123721311	123741507	-
16480	1450085_at	Region	Ralgps1	241308	2	33069135	33303640	-
16481	1430671_a_at	Region	0610010K06Rik	71678	1	10115	28628	-
16482	1457656_s_at	Region	C230085N15Rik	320556	17	49292108	49292393	-
16483	1459657_s_at	Region	Rpo1-3	20018	5	145969234	146003052	+
16484	1436516_at	Region	1110065L07Rik	68904	8	9345221	9357725	+
16485	1424940_s_at	Region	BC022687	217887	12	108284811	108292081	+
16486	1450406_a_at	Region	St3gal3	20441	4	116891059	117093795	-
16487	1456640_at	Region	Sh3rf2	269016	18	42274341	42379082	+
16488	1459308_at	Region	Kcnn3	140493	3	89277142	89424152	+
16489	1427066_a_at	Region	4933439F18Rik	66771	11	60142945	60170919	+
16490	1452175_at	Region	1810026J23Rik	69773	9	21480123	21486981	+
16491	1419485_at	Region	Foxc1	17300	13	31286268	31290257	+
16492	1428169_at	Region	Atg16l	77040	1	87572642	87608993	+
16493	1435683_a_at	Region	Abcc5	27416	16	19102921	19197967	-
16494	1446041_at	Region	None	None	NONE	NONE	NONE	NONE
16495	1417061_at	Region	Slc40a1	53945	1	46211868	46229317	-
16496	1436515_at	Region	E030004N02Rik	319905	4	32863442	32864900	+
16497	1426882_at	Region	Ube3c	100763	5	28019724	28126560	+
16498	1416615_at	Region	Clpp	53895	17	54680231	54686109	+
16499	1449857_at	Region	1200011I18Rik	67467	14	70430926	70453271	-
16500	1428896_at	Region	Pdgfrl	68797	8	39862136	39926677	+
16501	1438361_at	Region	2310035C23Rik	227446	1	105555177	105695178	+
16502	1430631_at	Region	Ppm1f	68606	16	15674119	15698163	+
16503	1433692_at	Region	AI429152	98956	2	103426013	103466004	-
16504	1430940_at	Region	3110045A19Rik	73186	15	96018470	96019667	+
16505	1453686_x_at	Region	Nphp1	53885	2	127254585	127302707	-
16506	1452607_at	Region	None	None	X	66477436	66483670	+
16507	1442257_at	Region	B130021B11Rik	320860	15	12834885	12835556	-
16508	1443312_at	Region	None	None	10	84656000	84656376	+
16509	1444639_at	Region	Nphp4	260305	4	150970504	151055496	+
16510	1456357_at	Region	9330112M16	329395	2	39001301	39001944	-
16511	1426881_at	Region	Ube3c	100763	5	28019724	28126560	+
16512	1439795_at	Region	Gpr64	237175	X	154029755	154097475	+
16513	1426404_a_at	Region	Rnf11	29864	4	108411762	108435410	-
16514	1418564_s_at	Region	1200009K13Rik	66870	6	67519748	67538732	+
16515	1446303_at	Region	A330103N21Rik	77773	7	62080402	62080929	+
16516	1447258_at	Region	None	None	14	19903710	19904049	+
16517	1445532_at	Region	C230040D10Rik	244281	8	9521415	10002757	+
16518	1450163_a_at	Region	Wrn	22427	8	32039864	32191103	-
16519	1429494_at	Region	Trim35	66854	14	60824516	60836924	+
16520	1450845_a_at	Region	Bzw1	66882	1	58698537	58711303	+
16521	1415686_at	Region	Rab14	68365	2	35113675	35132954	-
16522	1456181_at	Region	9530020G05Rik	101240	6	34974689	34978615	-
16523	1427131_s_at	Region	1810012N18Rik	69110	4	111924563	111927246	+

16524	1448794_s_at	Region	Dnajc2	22791	5	20209641	20237529	-
16525	1440975_at	Region	1810057P16Rik	67622	11	116624497	116649139	-
16526	1434367_s_at	Region	Nutf2	68051	8	105156479	105176249	+
16527	1449849_a_at	Region	Fbxl6	30840	15	76586680	76589679	-
16528	1434700_at	Region	6030408C04Rik	217558	12	48125521	48128402	+
16529	1438117_x_at	Region	Tmem41b	233724	7	103825350	103839101	-
16530	1452995_at	Region	Nudt17	78373	3	96193904	96196398	-
16531	1429510_at	Region	2810410L24Rik	76377	11	120006436	120007769	+
16532	1442555_at	Region	Kcnd3	56543	3	105254643	105465036	+
16533	1426210_x_at	Region	Parp3	235587	9	106534690	106540942	-
16534	1418070_at	Region	Cdyl	12593	13	35194740	35408765	+
16535	1422622_at	Region	Nos3	18127	5	22828716	22848368	+
16536	1442453_at	Region	Fcho2	218503	13	94911844	95003580	-
16537	1457402_at	Region	Sulf1	240725	1	12847254	12989080	+
16538	1437623_x_at	Region	Xrcc3	74335	12	107284533	107295382	-
16539	1453387_at	Region	4833432E10Rik	78782	12	22162976	22165185	-
16540	1454802_x_at	Region	Arih2	23807	9	108651717	108698223	-
16541	1442224_at	Region	None	None	1	133062717	133063304	+
16542	1450418_a_at	Region	Yipf4	67864	17	72297116	72307880	+
16543	1451891_a_at	Region	Dysf	26903	6	84367321	84563049	+
16544	1441625_at	Region	A930033C01Rik	231760	5	127928057	128009592	-
16545	1443948_at	Region	2210019E14Rik	70387	19	8007843	8015835	-
16546	1455283_x_at	Region	Ndufs8	225887	19	3697661	3701550	-
16547	1442322_at	Region	Slit2	20563	5	46748038	47069334	+
16548	1448167_at	Region	Ifngr1	15979	10	19518117	19537501	+
16549	1426476_at	Region	Rasa1	218397	13	81307760	81348198	-
16550	1419377_at	Region	Med9	192191	11	59673892	59686765	+
16551	1456053_at	Region	None	None	3	17382979	17383446	-
16552	1439604_at	Region	Adamts16	271127	13	66812918	66894377	-
16553	1441630_at	Region	Ep400	75560	5	109714361	109820853	-
16554	1419655_at	Region	Tle3	21887	9	61493517	61536829	+
16555	1435773_at	Region	4930547N16Rik	75317	10	88063619	88119328	-
16556	1422172_x_at	Region	2810408B13Rik	76371	NONE	NONE	NONE	NONE
16557	1423928_at	Region	Phgdhl1	68889	14	116446863	116589587	+
16558	1425274_at	Region	Asph	65973	4	9376231	9595958	-
16559	1451503_at	Region	Nol3	78688	8	104572075	104577567	+
16560	1452270_s_at	Region	Cubn	65969	2	13192347	13407933	-
16561	1438328_at	Region	1700129L13Rik	67933	10	82611323	82629097	+
16562	1429243_at	Region	1110054O05Rik	66209	4	59713082	59727302	-
16563	1426810_at	Region	Jmjd1a	104263	6	71920949	71964715	-
16564	1422584_at	Region	Skiv2l	108077	17	32542108	32553012	-
16565	1440836_at	Region	BC035291	208043	5	122323659	122324827	+
16566	1439383_x_at	Region	Ppp2r4	110854	2	30348212	30379966	+
16567	1425109_at	Region	BC010552	213603	3	120238421	120313412	-
16568	1434807_s_at	Region	Mtx3	382793	13	89041743	89043160	+
16569	1437506_at	Region	None	None	13	100713757	100714447	+
16570	1418304_at	Region	Pcdh21	170677	14	35209848	35230461	-
16571	1449437_at	Region	D6Wsu163e	28040	6	127614300	127649698	+
16572	1417077_at	Region	Bcap29	12033	12	28170555	28209291	-
16573	1453356_at	Region	5730453I16Rik	269061	19	9721763	9744150	+
16574	1428070_at	Region	Syvn1	74126	19	5835452	5842499	+
16575	1456099_at	Region	D930017J03Rik	320391	14	47187248	47187838	-
16576	1434517_at	Region	Wdfy2	268752	14	57369182	57487094	+
16577	1437114_at	Region	A930001N09Rik	77128	17	24510472	24568084	+
16578	1458701_at	Region	2310032D16Rik	74182	2	132043251	132092031	-
16579	1417352_s_at	Region	Snrpa1	68981	7	59944793	59958808	+
16580	1448879_at	Region	Ube2l3	22195	16	15923031	15972515	-
16581	1452847_at	Region	2410008K03Rik	71962	11	4113038	4117196	+
16582	1434654_at	Region	Cog3	338337	14	70044865	70097010	-
16583	1434480_at	Region	4930402E16Rik	319518	8	110392463	110432862	+
16584	1447304_at	Region	None	None	8	69164816	69165213	-
16585	1436552_at	Region	6430702L21Rik	76217	18	43750692	43907422	-
16586	1454893_at	Region	1110013L07Rik	68521	3	88944407	88945763	+
16587	1417369_at	Region	Hsd17b4	15488	18	50343439	50411362	+
16588	1434423_at	Region	None	None	1	45102276	45102933	+

16589	1417591_at	Region	Ptges2	96979	2	32328420	32334901	+
16590	1436858_at	Region	Mbnl2	105559	14	114846670	115003029	+
16591	1438225_x_at	Region	Tram1	72265	1	13697903	13722901	-
16592	1458506_at	Region	Gm1582	380890	14	31703552	31707587	+
16593	1442695_at	Region	C030007I01Rik	77333	5	130019276	130019862	+
16594	1431753_x_at	Region	2900073H19Rik	68205	2	29759551	29777158	+
16595	1451521_x_at	Region	Wbscr1	22384	5	350218	369670	-
16596	1448645_at	Region	Msl31	17692	X	162253522	162273307	-
16597	1422245_a_at	Region	Mrvi1	17540	7	104721209	104835598	-
16598	1426017_a_at	Region	0610011L14Rik	68295	2	156004536	156025909	+
16599	1423568_at	Region	Psma7	26444	2	179753334	179759360	-
16600	1428139_at	Region	4930538D17Rik	75146	19	45904277	45922593	+
16601	1421122_at	Region	Cbll1	104836	12	28062305	28074949	-
16602	1448271_a_at	Region	Ddx21	56200	10	62546632	62565575	-
16603	1424734_at	Region	Rasgrf1	19417	9	89795814	89912965	+
16604	1418279_a_at	Region	Akap1	11640	11	88652757	88672590	-
16605	1423762_at	Region	Adck1	72113	12	4589	10832	-
16606	1434473_at	Region	Slc16a5	217316	11	115285663	115294699	+
16607	1444510_at	Region	None	None	2	180611591	180612082	+
16608	1429876_at	Region	Supt7l	72195	5	29973507	29985623	-
16609	1426528_at	Region	Nrp2	18187	1	62996720	63108906	+
16610	1459874_s_at	Region	Mtmr4	170749	11	87320209	87344288	+
16611	1415756_a_at	Region	Snapap	20615	3	90298452	90301491	-
16612	1419828_at	Region	Usp31	76179	7	115517588	115530585	-
16613	1449349_at	Region	Nudt1	17766	5	139328262	139334475	+
16614	1418836_at	Region	Qprt	67375	7	121157501	121171760	-
16615	1443986_at	Region	Hrpt2	214498	1	143439562	143534747	-
16616	1436848_x_at	Region	Impa1	55980	3	10315087	10332919	-
16617	1424675_at	Region	Slc39a6	106957	18	24808377	24830068	-
16618	1445273_at	Region	D10Ertd638e	52658	10	67136475	67136802	+
16619	1426498_at	Region	Jarid1c	20591	X	145767667	145808191	+
16620	1444912_at	Region	None	None	12	28493291	28494096	+
16621	1420098_s_at	Region	D13Ertd787e	52680	13	44331485	44331921	+
16622	1438293_at	Region	A930031F18Rik	77832	5	113817500	113831953	+
16623	1429193_at	Region	Ankib1	70797	5	3696004	3698199	-
16624	1434183_at	Region	LOC546078	546078	8	83257670	83259649	+
16625	1455801_x_at	Region	Tbcd	108903	11	121273095	121438261	+
16626	1434043_a_at	Region	None	None	6	48731274	48731962	+
16627	1456417_at	Region	Zic4	22774	9	91269057	91275258	+
16628	1448706_at	Region	Ttrap	56196	13	24311562	24322023	+
16629	1438883_at	Region	Fgf5	14176	5	97273813	97296659	+
16630	1454756_at	Region	None	None	16	31820778	31823841	+
16631	1456284_at	Region	None	None	13	94874779	94880829	-
16632	1425331_at	Region	Zfp106	20402	2	120023462	120077639	-
16633	1449047_at	Region	1600020H07Rik	56794	14	29739097	29772832	-
16634	1434607_at	Region	Ddx52	78394	11	83670170	83690182	+
16635	1433905_at	Region	Akap7	268287	10	25144311	25145838	-
16636	1433105_at	Region	5830438M01Rik	76032	16	31112565	31113866	-
16637	1424426_at	Region	Mtap	66902	4	88123618	88167275	+
16638	1440431_at	Region	Meis1	17268	11	18775221	18913473	-
16639	1437690_x_at	Region	Csnk1d	104318	11	120784515	120812412	-
16640	1421731_a_at	Region	Fen1	14156	19	9395636	9400440	-
16641	1457695_at	Region	Ap1gbp1	217030	11	83692463	83770081	+
16642	1449261_at	Region	Pbx2	18515	17	32293780	32299792	+
16643	1437766_at	Region	None	None	NONE	NONE	NONE	NONE
16644	1450223_at	Region	Apaf1	11783	10	90961928	91054028	-
16645	1440537_at	Region	Kcnv2	240595	19	26562685	26577245	+
16646	1417138_s_at	Region	Polr2e	66420	10	80158583	80162429	-
16647	1455441_at	Region	Map3k7	26409	4	32242911	32299581	+
16648	1446987_at	Region	3110037I16Rik	73172	4	119943646	119947449	-
16649	1419303_at	Region	Heyl	56198	4	122260965	122276982	+
16650	1450995_at	Region	Folr1	14275	7	95964436	95976787	-
16651	1454709_at	Region	9630015D15Rik	100201	4	15192785	15212331	+
16652	1434312_at	Region	AI788669	104877	12	66207139	66207778	+
16653	1433663_s_at	Region	LOC433702	433702	4	46054469	46088325	+

16654	1423348_at	Region	Fzd8	14370	18	9255490	9257547	+
16655	1456950_at	Region	Alms1	236266	6	85937447	86006447	+
16656	1453470_a_at	Region	Gna13	14674	11	109184029	109219306	+
16657	1451254_at	Region	Ikbkap	230233	4	56694223	56745708	-
16658	1435863_at	Region	Commd6	66200	14	96156180	96168796	-
16659	1430592_at	Region	2310036D22Rik	71900	6	13057102	13072310	+
16660	1450904_at	Region	0610041E09Rik	66074	13	86207541	86225129	+
16661	1449111_a_at	Region	Grb2	14784	11	115465223	115529690	-
16662	1420835_at	Region	Slc25a30	67554	14	70104494	70129532	-
16663	1420684_at	Region	Acox3	80911	5	34070572	34101313	+
16664	1429710_at	Region	Styx	56291	14	63481864	63493914	+
16665	1441053_at	Region	None	None	2	130081646	130082549	+
16666	1446566_at	Region	None	None	11	83065510	83066050	+
16667	1423703_at	Region	Ppan	235036	9	20765099	20769021	+
16668	1434130_at	Region	Lhfpl2	218454	13	90241860	90379474	+
16669	1438016_at	Region	BC068171	245474	X	69756509	69770234	+
16670	1429558_a_at	Region	C330027G06Rik	68280	4	91162379	91171661	-
16671	1417861_at	Region	Ccnc	51813	4	21797101	21819917	+
16672	1437166_at	Region	None	None	14	50194223	50195046	-
16673	1429690_at	Region	1300003B13Rik	74149	17	20344888	20377307	+
16674	1427036_a_at	Region	Eif4g1	208643	16	19444890	19465024	+
16675	1416394_at	Region	Bag1	12017	4	41075268	41087088	-
16676	1444241_at	Region	Wac	225131	18	7911809	7969644	+
16677	1457188_at	Region	Arhgef11	213498	3	87428572	87483137	+
16678	1423839_a_at	Region	Btf3	218490	13	94498396	94505466	-
16679	1437873_at	Region	6030490I01Rik	240064	17	30641170	30652881	-
16680	1443950_at	Region	A630042L21Rik	106894	18	61356597	61402376	-
16681	1441091_at	Region	None	None	13	104513355	104514581	+
16682	1440488_at	Region	Ptpn4	19258	1	119411179	119553909	-
16683	1451329_at	Region	Nudt22	68323	19	6706867	6709885	-
16684	1443771_x_at	Region	Smad7	17131	18	75602714	75631112	+
16685	1441152_at	Region	Rnpc2	170791	2	155604575	155637077	-
16686	1421062_s_at	Region	Clta	12757	4	43928606	43948768	+
16687	1429383_at	Region	Csnk1g3	70425	18	54125626	54174530	+
16688	1415684_at	Region	Atg5l	11793	10	44383463	44478754	+
16689	1433780_at	Region	Ubn1	170644	16	4746672	4756072	+
16690	1448415_a_at	Region	Sema3b	20347	9	107664136	107671183	-
16691	1437857_at	Region	9330164H19Rik	233115	7	30851673	30917234	-
16692	1427838_at	Region	Tubb2	22151	13	33608787	33612497	-
16693	1456630_x_at	Region	Son	20658	16	90805044	90836353	+
16694	1452838_at	Region	Ddx10	77591	9	53174536	53321395	-
16695	1453554_a_at	Region	Wdr33	74320	18	32067429	32147139	+
16696	1458635_at	Region	4832428D23Rik	403183	1	44499889	44514750	-
16697	1454841_at	Region	4921511H13Rik	207932	16	89908565	89987304	-
16698	1457885_at	Region	Prkag2	108099	5	23326651	23569979	-
16699	1435485_at	Region	C230096C10Rik	230866	4	138233859	138259973	+
16700	1459687_x_at	Region	None	None	NONE	NONE	NONE	NONE
16701	1434972_x_at	Region	Sfrs1	110809	11	87775771	87779641	+
16702	1439453_x_at	Region	1500026D16Rik	68209	19	5390675	5391722	+
16703	1418018_at	Region	Cpd	12874	11	76507648	76572655	-
16704	1438398_at	Region	Rnpc2	170791	2	155604575	155637077	-
16705	1419853_a_at	Region	None	None	5	121835802	121836241	+
16706	1452691_at	Region	Rbm17	76938	2	11501448	11519208	-
16707	1424774_s_at	Region	3230401I01Rik	67417	7	115916679	115944921	-
16708	1435671_at	Region	None	None	12	54063774	54229148	+
16709	1449686_s_at	Region	Scp2	20280	4	107002744	107077406	-
16710	1438995_at	Region	6330500D04Rik	193385	13	24134886	24135518	+
16711	1424693_at	Region	4933407N01Rik	66753	11	30826209	30848913	-
16712	1425920_at	Region	Cuedc1	103841	11	87827241	87920907	+
16713	1423130_a_at	Region	Sfrs5	20384	12	77807832	77811755	+
16714	1432848_a_at	Region	1200004M23Rik	67457	19	5639764	5663998	-
16715	1433704_s_at	Region	Tloc1	69276	3	30204876	30231459	+
16716	1419990_at	Region	A830082K12Rik	320174	NONE	NONE	NONE	NONE
16717	1440490_at	Region	Mpp6	56524	6	50254948	50343115	+
16718	1429160_at	Region	2810012L14Rik	76366	5	145846622	145855467	-

16719	1453456_at	Region	2900084O13Rik	73080	17	88742782	88743492	-
16720	1442849_at	Region	Lrp1	16971	10	127274824	127357767	-
16721	1434732_x_at	Region	Tomm7	66169	5	22303551	22308004	-
16722	1424838_at	Region	A330049M08Rik	230822	4	134251167	134279478	-
16723	1417216_at	Region	Pim2 /// LOC229005	18715	X	6117246	6122372	+
16724	1427414_at	Region	Prkar2a	19087	9	108741435	108795663	+
16725	1418248_at	Region	Gla	11605	X	128133295	128145894	-
16726	1433976_at	Region	AI265725	103168	4	76613810	76614393	+
16727	1431212_a_at	Region	3300001M20Rik	66926	2	132318068	132329908	-
16728	1443281_at	Region	C130076O07Rik	319504	12	40985533	41346376	+
16729	1424264_at	Region	Med6	69792	12	78433783	78455283	-
16730	1421943_at	Region	Tgfa	21802	6	86626355	86707058	+
16731	1420348_at	Region	Lhx5	16873	5	119583708	119593264	+
16732	1415754_at	Region	Polr2f	69833	15	79192653	79203053	+
16733	1424514_at	Region	Rnf126	70294	10	79881163	79889548	-
16734	1431804_a_at	Region	Sp3	20687	2	72634943	72710690	-
16735	1422101_at	Region	Tnfrsf23	79201	7	138080633	138098881	-
16736	1441727_s_at	Region	Zfp467	68910	6	48570465	48578486	-
16737	1416973_at	Region	LOC434401	434401	9	48460405	48473895	+
16738	1436896_at	Region	D14Ertd231e	210925	14	59478323	59566706	+
16739	1447994_at	Region	1700026B20Rik	69434	12	100474801	100476412	-
16740	1437267_x_at	Region	BC035291	208043	5	122320841	122321085	-
16741	1438360_x_at	Region	Slc25a5	11740	X	31416206	31419360	+
16742	1460354_a_at	Region	Mrpl13	68537	15	55539170	55562387	-
16743	1428395_at	Region	Smurf1	75788	5	143917448	144004286	-
16744	1415976_a_at	Region	Carhsp1	52502	16	8331880	8345446	-
16745	1440691_at	Region	Cyp2j6	13110	4	95492315	95528724	-
16746	1428097_at	Region	2510009E07Rik	72190	16	20420556	20466125	-
16747	1438885_at	Region	BB182387	103516	10	14670967	14671652	+
16748	1430547_s_at	Region	Cryzl1	66609	16	90846621	90885763	-
16749	1450706_a_at	Region	Arl3	56350	19	46078947	46106113	-
16750	1421204_a_at	Region	Nudt16	75686	9	105111048	105113181	-
16751	1417284_at	Region	Mapkap1	227743	2	34339345	34556075	+
16752	1439582_at	Region	Macf1	11426	4	122378001	122711301	-
16753	1460482_at	Region	3110047P20Rik	319807	5	62449370	62608204	+
16754	1440601_at	Region	None	None	NONE	NONE	NONE	NONE
16755	1424667_a_at	Region	Cutl1	13047	5	135261627	135499343	-
16756	1448464_at	Region	0610042I15Rik	56418	11	5850619	5862567	+
16757	1416302_at	Region	Ebf1	13591	11	44370971	44758012	+
16758	1438480_a_at	Region	MGI:1925112	77862	9	26893639	26901251	+
16759	1426945_at	Region	Ranbp5	70572	14	115481885	115518633	+
16760	1452512_a_at	Region	Ank1	11733	8	21812854	21905075	+
16761	1417501_at	Region	Fbxo6b	50762	4	146638149	146644186	-
16762	1421895_at	Region	Eif2s3x	26905	X	88849367	88873309	-
16763	1415714_a_at	Region	2610209M04Rik	66618	6	87109370	87118796	-
16764	1415761_at	Region	Mrpl52	68836	14	48943738	48946676	+
16765	1422770_at	Region	Rad51l3	19364	11	82614373	82630248	-
16766	1426268_at	Region	C130090K23Rik	231293	5	72195907	72243364	+
16767	1446391_at	Region	Snca	20617	6	60971674	61069793	-
16768	1423733_a_at	Region	Fiz1	23877	7	4254301	4261832	-
16769	1441620_at	Region	None	None	11	11868853	11869483	-
16770	1416426_at	Region	Rab5a	271457	17	51013405	51041849	+
16771	1423037_at	Region	Agtrl1	23796	2	84834149	84837672	+
16772	1452016_at	Region	Alox5ap	11690	5	148165072	148186592	+
16773	1442220_at	Region	Ube3a	22215	7	53507062	53583196	+
16774	1459066_at	Region	None	None	8	109390040	109390397	-
16775	1442995_at	Region	None	None	18	60961630	60962349	-
16776	1439788_at	Region	MGC86034	408022	8	45522371	45563977	-
16777	1453294_at	Region	1700012B15Rik	74173	12	273	3622	+
16778	1445881_at	Region	2310035P21Rik	75683	19	53483140	53483841	+
16779	1456905_at	Region	B4galt2	53418	4	116828165	116842060	-
16780	1435474_at	Region	Taf5	226182	19	46619048	46634768	+
16781	1449824_at	Region	Prg4	96875	1	150286578	150302927	-
16782	1440283_at	Region	1810059H22Rik	69820	NONE	NONE	NONE	NONE

16783	1446682_at	Region	Zswim6	67263	13	103950578	104158812	-
16784	1431188_a_at	Region	Tom1 /// LOC545878	21968	8	74197771	74234173	+
16785	1428835_at	Region	Myh14	71960	7	38680631	38740313	-
16786	1443633_at	Region	1700086O06Rik	73516	18	38462795	38473601	-
16787	1425969_a_at	Region	Hdh	15194	5	33249415	33397070	+
16788	1458625_at	Region	None	None	16	96073255	96073824	-
16789	1422776_at	Region	Serpinb8	20725	1	107578943	107597781	+
16790	1454624_at	Region	B930096L08Rik	209773	6	39597808	39693264	-
16791	1429634_at	Region	Zfp580	68992	7	4300418	4302602	+
16792	1416242_at	Region	Klhl13	67455	X	21458211	21603993	-
16793	1439794_at	Region	None	None	10	93719737	93720538	+
16794	1447147_at	Region	Apg7l	74244	6	115108368	115295327	+
16795	1459661_at	Region	AW492955	105362	13	24690134	24690584	+
16796	1424044_at	Region	Jmjd2b	193796	17	53963133	54039904	+
16797	1417342_at	Region	Ppp1r2 /// LOC433058 /// LOC433239 /// LOC546139 /// LOC546723	433058	17	6159954	6161505	+
16798	1430070_at	Region	1500035N22Rik	70258	5	23455391	23467712	+
16799	1452301_at	Region	Aldh3b1	67689	19	3702281	3712691	-
16800	1443026_at	Region	B130020M22Rik	320217	10	119050227	119050928	+
16801	1453121_at	Region	1700010L19Rik	71840	17	23277441	23282445	+
16802	1452484_at	Region	Car7	12354	8	103833942	103843478	+
16803	1438441_at	Region	Idb4	15904	13	47859485	47862094	+
16804	1417294_at	Region	Akr7a5	110198	4	138192005	138199686	+
16805	1455320_at	Region	AI480535	104840	12	29458299	29459571	+
16806	1424512_a_at	Region	Txndc9	98258	1	38279664	38291679	-
16807	1423662_at	Region	Atp6ap2	70495	X	10826811	10855905	+
16808	1415812_at	Region	Gsn	227753	2	35214602	35240054	+
16809	1424028_at	Region	5830457O10Rik	214987	8	106181580	106191288	-
16810	1423912_at	Region	Aspscr1	68938	11	120494132	120530540	+
16811	1447040_at	Region	Alcam	11658	16	51168811	51372049	-
16812	1435498_at	Region	Arfip2	76932	7	99750533	99755261	-
16813	1416544_at	Region	Ezh2	14056	6	47731971	47796710	-
16814	1458582_at	Region	AI461788	231506	5	99460839	99519278	-
16815	1417386_at	Region	Npepps	19155	11	97028020	97101650	-
16816	1428084_at	Region	Hrb2	52705	10	111675789	111690089	+
16817	1435209_at	Region	BC057079	230393	4	87080822	87397196	+
16818	1454668_at	Region	1810009A16Rik	69116	4	138281097	138370851	+
16819	1452194_at	Region	Tbcd	108903	11	121273095	121438261	+
16820	1427526_at	Region	Fgfr1op2	67529	6	147498625	147519880	+
16821	1446876_at	Region	Wdr22	320808	12	77197394	77297795	-
16822	1444722_at	Region	Psme4	103554	11	30666852	30774524	+
16823	1455057_at	Region	Gmps	229363	3	63642160	63687698	+
16824	1454732_at	Region	Bpy2ip1	270058	8	70061700	70073238	+
16825	1453614_a_at	Region	Nfe2l3	18025	6	51579895	51606046	+
16826	1430762_at	Region	4833427G06Rik	235345	9	51153639	51174404	-
16827	1441044_at	Region	C130002M15Rik	319741	16	42825408	42826081	+
16828	1423155_at	Region	Sri	109552	5	8054494	8076270	+
16829	1456622_at	Region	Camsap1	227634	2	25860417	25927345	-
16830	1446938_at	Region	AA408213	100498	5	145946467	145946881	-
16831	1420951_a_at	Region	Son	20658	16	90805044	90836353	+
16832	1433773_at	Region	Rrm2b	382985	15	37928889	37965992	-
16833	1459230_at	Region	Plod2	26432	9	92437061	92503040	+
16834	1430991_at	Region	1810014B01Rik	544717	10	82507736	82511798	-
16835	1434029_at	Region	LOC545794	545794	5	109470398	109498632	-
16836	1436385_at	Region	Ganc	76051	2	119918051	119974715	+
16837	1424657_at	Region	Taok1	216965	11	77255718	77311108	-
16838	1456215_at	Region	Tfam	21780	10	71279444	71292011	-
16839	1458056_at	Region	Sfrs12	218543	13	99959715	99982622	-
16840	1456626_a_at	Region	1110005A23Rik	66118	10	128559009	128614707	+
16841	1451591_a_at	Region	Efnb1	13641	X	93737491	93750352	+
16842	1417560_at	Region	Sfxn1	14057	13	52636174	52672650	+

16843	1422017_s_at	Region	4833439L19Rik	97820	13	53160979	53175095	-
16844	1430613_at	Region	1520402A15Rik	68075	4	115096204	115103521	-
16845	1439055_at	Region	None	None	11	88785745	88786362	-
16846	1440219_at	Region	BC055915	211660	1	10178667	10241764	+
16847	1436915_x_at	Region	Laptm4b	114128	15	34237699	34283968	+
16848	1424583_at	Region	Farp2	227377	1	93339616	93449273	+
16849	1436131_at	Region	6430529G09Rik	233274	7	50037705	50048312	+
16850	1448567_at	Region	C78915	56395	9	107599726	107604437	+
16851	1427376_a_at	Region	Map4k5	399510	12	66635229	66724562	-
16852	1417247_at	Region	AI597479	98404	1	43393427	43410663	+
16853	1434984_at	Region	6330514A18Rik	216166	10	80481671	80492695	+
16854	1434793_at	Region	BC028975	242584	4	101996974	102073129	-
16855	1423328_at	Region	Gdap1	14545	1	17329856	17348730	+
16856	1423650_at	Region	LOC545856	545856	6	76842195	76844739	-
16857	1423351_at	Region	Mrpl1	94061	5	95228007	95282266	+
16858	1444735_at	Region	D6Ertd365e	387524	6	54961241	55034061	+
16859	1415697_at	Region	E430034L04Rik	23881	5	91390328	91419486	-
16860	1429720_at	Region	Mak10	78689	13	58245009	58294396	+
16861	1438451_at	Region	MGI:2450166	330914	9	32129387	32182471	+
16862	1424265_at	Region	Npl	74091	1	153388014	153434345	-
16863	1434286_at	Region	Trps1	83925	15	50659276	50888038	-
16864	1446514_at	Region	Dpp10	269109	1	123096743	123812573	-
16865	1453744_a_at	Region	Ankrd40	71452	11	94149236	94161010	+
16866	1416769_s_at	Region	Atp6v0b	114143	4	116843235	116846234	-
16867	1430985_at	Region	1810027O10Rik	69186	11	69564182	69565267	+
16868	1450868_at	Region	D8Ertd354e	52120	8	24669208	24671231	-
16869	1451742_a_at	Region	Ugp2	216558	11	21215937	21265268	-
16870	1428913_at	Region	D2Bwg1335e	52838	2	26280280	26284262	-
16871	1456292_a_at	Region	Vim	22352	2	13489961	13498816	+
16872	1430702_at	Region	5830427D03Rik	76061	15	98817582	98819633	-
16873	1424247_at	Region	Rab6ip2	111173	6	120007511	120280559	-
16874	1455349_at	Region	Rap1b	215449	10	117505589	117536966	-
16875	1431018_at	Region	1810013L24Rik	69053	16	8502119	8528217	+
16876	1422982_at	Region	Ar	11835	X	92751111	92918508	+
16877	1423643_at	Region	Ddx39	68278	8	82975115	82983264	+
16878	1456304_at	Region	Gm996	381353	2	25507592	25512261	-
16879	1428232_at	Region	Cpsf6	432508	10	117034119	117066391	-
16880	1426450_at	Region	Plcl2	224860	17	48041884	48220970	+
16881	1426627_at	Region	None	None	4	32242902	32300043	+
16882	1443521_at	Region	Ggnbp2	217039	11	84560721	84598730	-
16883	1445224_at	Region	Ap3b2	11775	7	75268542	75301907	-
16884	1434187_at	Region	None	None	8	20822628	20823670	+
16885	1434919_at	Region	Agps	228061	2	75529941	75627399	+
16886	1450337_a_at	Region	Nek8	140859	11	77892179	77902325	-
16887	1420444_at	Region	Slc22a3	20519	17	11059072	11146804	-
16888	1417154_at	Region	Slc25a14	20523	X	43144374	43183090	+
16889	1417782_at	Region	Lass4	67260	8	3848044	3880287	+
16890	1440329_s_at	Region	Gpatc2	67769	1	186723158	186859064	+
16891	1425630_at	Region	Sin3b	20467	8	71873343	71907207	+
16892	1418452_at	Region	Gng2	14702	14	18235904	18323407	-
16893	1433982_at	Region	Usp28	235323	9	49014954	49072769	+
16894	1436374_x_at	Region	F11r	16456	1	171366400	171393432	+
16895	1449408_at	Region	Jam2	67374	16	83882574	83932843	+
16896	1433698_a_at	Region	D18Wsu98e	28062	18	80330416	80336262	+
16897	1455557_at	Region	LOC553095	553095	13	80118979	80157275	-
16898	1422727_at	Region	Nme5	75533	18	34785745	34801708	-
16899	1454309_at	Region	2810002N01Rik	68020	12	107191903	107212849	+
16900	1444736_at	Region	Cdh7	241201	1	109972834	110128098	+
16901	1425578_a_at	Region	Gfra2	14586	14	65206370	65294008	+
16902	1429617_at	Region	Cyld	74256	8	87990637	88033868	+
16903	1450174_at	Region	Ptprt	19281	2	160984592	162118116	-
16904	1442052_at	Region	C330019G07Rik	215476	5	31277729	31316129	-
16905	1423051_at	Region	Hnrpu	51810	1	178283700	178291584	-
16906	1425010_at	Region	Zfp119	104349	17	53503078	53517139	-
16907	1455487_at	Region	2600014M03Rik	69900	11	116675108	116696753	+

16908	1436098_at	Region	None	None	NONE	NONE	NONE	NONE
16909	1438886_at	Region	Heyl	56198	4	122260965	122276982	+
16910	1435108_at	Region	Arhgap22	239027	14	31353150	31504377	+
16911	1421334_x_at	Region	Mynn	80732	3	30012186	30028739	+
16912	1436346_at	Region	Cd109	235505	9	78815469	78916251	+
16913	1444973_at	Region	Kcnma1	16531	14	21672901	22164598	-
16914	1446613_at	Region	1190005F20Rik	98685	1	151302852	151303493	+
16915	1426778_at	Region	Dag1	13138	9	108271876	108333355	-
16916	1424038_a_at	Region	2310044H10Rik	69683	7	38565409	38571690	-
16917	1415926_at	Region	Nup62	18226	7	38891347	38905734	+
16918	1445178_at	Region	Sh3md2	59009	8	60271818	60461252	+
16919	1460718_s_at	Region	Mtch1	56462	17	27144760	27160995	-
16920	1455625_at	Region	None	None	7	99854045	99854808	-
16921	1418359_at	Region	Wbscr27	79565	5	133945865	133956129	+
16922	1429082_at	Region	D5Ertd585e	71782	5	109281825	109307432	+
16923	1436956_at	Region	Bace1	23821	9	45851835	45875693	+
16924	1450973_s_at	Region	Mapkbp1	26390	2	119486752	119539188	+
16925	1451717_s_at	Region	Senp2	75826	16	20782192	20820660	+
16926	1422047_at	Region	Cdh5	12562	8	103396382	103438830	+
16927	1434257_s_at	Region	Pitpnb	56305	5	110380456	110438052	+
16928	1430685_at	Region	6330503C03Rik	76156	6	42461663	42468459	-
16929	1424579_at	Region	Slc35a3	229782	3	115439734	115481216	-
16930	1436249_at	Region	Ttll4	67534	1	74991849	75001071	+
16931	1442535_at	Region	None	None	9	65927470	65928059	-
16932	1425749_at	Region	Stxbp6	217517	12	41600913	41820407	-
16933	1441867_x_at	Region	4930534B04Rik	75216	12	86694447	86789217	-
16934	1431092_at	Region	Ppp1r12c	232807	7	3721021	3732838	-
16935	1449523_at	Region	Bcl7c	12055	7	121754709	121758497	-
16936	1453054_at	Region	Scamp1	107767	13	90385498	90469347	-
16937	1429499_at	Region	Fbxo5	67141	10	4705451	4711373	+
16938	1451374_x_at	Region	Cklf	75458	8	103545239	103557779	+
16939	1457593_at	Region	2610202C22Rik	72493	10	58242224	58242771	+
16940	1422206_at	Region	B3galt1	26877	2	67973343	67974323	+
16941	1452282_at	Region	Crsp7	70625	8	71645137	71698918	-
16942	1415681_at	Region	Mrpl43	94067	19	44549420	44550848	-
16943	1441262_at	Region	None	None	19	47179184	47179656	+
16944	1426871_at	Region	Fbxo33	70611	12	55933702	55953036	-
16945	1438809_at	Region	Atp5c1	11949	2	9972148	9996543	-
16946	1427090_at	Region	Zbed4	223773	15	88806694	88836445	+
16947	1438396_at	Region	Ocrl	320634	X	42433252	42486664	+
16948	1427078_at	Region	Snx19	102607	9	30343376	30382899	+
16949	1443747_at	Region	D3Ertd300e	56790	3	54327325	54351057	+
16950	1443296_at	Region	Pctk1	18555	X	18927433	18938817	+
16951	1434625_at	Region	None	None	NONE	NONE	NONE	NONE
16952	1456853_at	Region	None	None	10	82258103	82258445	+
16953	1438617_at	Region	None	None	X	132624193	132624537	-
16954	1444848_at	Region	R3hdm	226412	1	127946018	128080726	+
16955	1439440_x_at	Region	Ptk9l	23999	9	106196703	106208982	+
16956	1421432_at	Region	Arl10a	56795	13	53185314	53191400	+
16957	1415950_a_at	Region	Pbp	23980	5	116411434	116416327	-
16958	1433664_at	Region	3010021M21Rik	109161	9	55261748	55318919	+
16959	1427957_at	Region	9530008L14Rik	109254	13	41322369	41397818	-
16960	1416357_a_at	Region	Mcam	84004	9	44123526	44131579	+
16961	1453430_at	Region	2010315L10Rik	67023	8	70517056	70519559	+
16962	1437513_a_at	Region	Tde2	56442	10	57778279	57794950	-
16963	1456219_at	Region	Zic5	65100	14	117028709	117035237	-
16964	1423617_at	Region	2610019N19Rik /// Cog8	68023	8	106343774	106346361	-
16965	1423552_at	Region	Leprotl1	68192	8	32936611	32947770	-
16966	1457170_at	Region	Lrrc36	270091	8	104709250	104751133	+
16967	1426801_at	Region	38603	20362	11	53272641	53296937	+
16968	1421940_at	Region	Stag1	20842	9	100545170	100860091	+
16969	1419554_at	Region	Cd47	16423	16	48697237	48753271	+
16970	1440612_at	Region	Rbbp8	225182	18	11845010	11930602	+
16971	1459595_at	Region	None	None	5	110079227	110079602	+

16972	1424620_at	Region	D13Wsu177e	28126	13	53194546	53200435	-
16973	1429151_at	Region	Wdr68	71833	11	105857961	105876453	+
16974	1416712_at	Region	Pep4	18624	7	30066190	30200069	+
16975	1416238_at	Region	Tie1	21846	4	117430096	117448966	-
16976	1434644_at	Region	Tbl1x	21372	X	72294199	72320920	+
16977	1458187_at	Region	Plcd4	18802	1	74846994	74868444	+
16978	1456886_at	Region	None	None	12	106344593	106345230	+
16979	1434985_a_at	Region	Eif4a1	13681	11	69392597	69397973	-
16980	1459415_at	Region	None	None	11	61866732	61867367	+
16981	1433856_at	Region	AW555814	227399	1	97486245	97541628	-
16982	1440390_at	Region	BC025575	217219	11	102258068	102268730	-
16983	1439937_at	Region	None	None	14	50163706	50164327	+
16984	1451202_at	Region	C330007P06Rik	77644	X	31444291	31484792	-
16985	1430308_at	Region	5730409N16Rik	70475	12	69886395	69888767	+
16986	1460210_at	Region	Pkd1	18763	17	22353535	22400068	+
16987	1424198_at	Region	Dlg5	71228	14	22497841	22611777	-
16988	1425315_at	Region	Dock7	67299	4	97911748	97959905	-
16989	1428957_at	Region	2300008B03Rik	66343	1	119661146	119664607	-
16990	1433094_at	Region	Slc1a2	20511	2	102411192	102482860	+
16991	1428194_at	Region	Usp9x	22284	X	11310477	11410726	+
16992	1432274_at	Region	4930543N07Rik	75187	7	132940484	132953189	+
16993	1436925_at	Region	Ches1	71375	12	94622265	94816088	-
16994	1435074_at	Region	2310036D22Rik	71900	6	13057102	13072310	+
16995	1454711_at	Region	Trio	223435	15	27358260	27724087	-
16996	1443436_at	Region	Map2k2	26396	10	81240906	81258829	+
16997	1418777_at	Region	Ccl25	20300	8	3706166	3716677	+
16998	1420747_at	Region	MGI:1349458	26930	19	55428551	55431406	+
16999	1417935_at	Region	Mkrn2	67027	6	116039545	116056254	+
17000	1449100_at	Region	Pard6a	56513	8	104997459	104999242	+
17001	1432513_a_at	Region	1700001C02Rik	75434	5	28925023	28943033	+
17002	1445888_x_at	Region	Parp3	235587	9	106534690	106540942	-
17003	1450144_at	Region	Pla2g1br	18779	2	60274439	60408661	-
17004	1459854_s_at	Region	Tcte1l	67117	X	7893210	7901923	-
17005	1438032_at	Region	Lrch1	380916	14	69108806	69291857	-
17006	1448369_at	Region	Pola2	18969	19	5729336	5752960	-
17007	1446571_at	Region	Mlr1	209707	5	44458346	44613918	-
17008	1424713_at	Region	Calml4	75600	9	62980601	62998416	+
17009	1451000_at	Region	1810020E01Rik	66271	7	84500510	84507121	-
17010	1439107_a_at	Region	Mll5	69188	5	21950220	21960092	+
17011	1421747_at	Region	Esrrg	26381	1	187506691	187723715	+
17012	1428073_a_at	Region	Nup88	19069	11	70668716	70695619	-
17013	1450748_at	Region	Smpd3	58994	8	105548395	105633835	-
17014	1422146_at	Region	Sema5b	20357	16	34397801	34447067	+
17015	1418525_at	Region	Pcm1	18536	8	40177407	40269978	+
17016	1447976_at	Region	AI596198	99029	2	149859678	149860364	-
17017	1456818_at	Region	Stk32a	269019	18	43427684	43537468	+
17018	1425302_at	Region	BC024997	215723	11	68281844	68283903	+
17019	1460459_at	Region	Paqr5	74090	9	62073688	62146820	-
17020	1420814_at	Region	Gdi3	14569	13	3422528	3450201	+
17021	1436883_at	Region	Mbtps2	270669	X	151149345	151198055	-
17022	1417854_at	Region	Map2k5	23938	9	63284916	63498276	-
17023	1436801_x_at	Region	Cdc42ep4	56699	11	113547943	113572411	-
17024	1455196_s_at	Region	AA987161	380856	13	64191293	64211253	-
17025	1449068_at	Region	Zfp148	22661	16	32188918	32308456	+
17026	1443350_at	Region	None	None	17	81656741	81657367	+
17027	1441475_at	Region	None	None	13	56813529	56814207	-
17028	1439508_at	Region	A730055L17Rik	320652	NONE	NONE	NONE	NONE
17029	1427412_s_at	Region	Rapgef6	192786	11	54410160	54451128	+
17030	1451832_at	Region	Cklf	75458	8	103545239	103557779	+
17031	1443779_s_at	Region	A630025C20Rik	212391	19	41094011	41104153	+
17032	1427009_at	Region	Lama5	16776	2	179893403	179942812	-
17033	1447878_s_at	Region	Fgfrl1	116701	5	107770717	107783113	+
17034	1436400_at	Region	None	None	3	33512020	33513142	+
17035	1428487_s_at	Region	1500041J02Rik	67876	1	55354394	55373108	+
17036	1429255_at	Region	2010007H12Rik	69871	5	136729518	136730707	+

17037	1441022_at	Region	Arih1	23806	9	59509355	59603044	-
17038	1435822_at	Region	D830012I24Rik	320070	X	99111808	99112922	+
17039	1445207_at	Region	D230050A05	208426	3	67713505	67728904	+
17040	1423879_at	Region	D030056L22	225995	19	17960188	17965380	+
17041	1450019_at	Region	Cx3cr1	13051	9	120059072	120078236	-
17042	1434103_at	Region	AA408278	101934	8	71630980	71633661	-
17043	1435838_at	Region	LOC268782	268782	15	10223728	10224163	+
17044	1418473_at	Region	Cutc	66388	19	43297402	43313037	+
17045	1419031_at	Region	Fads2	56473	19	9260661	9298000	-
17046	1426629_at	Region	Dhx8	217207	11	101554043	101588445	+
17047	1450309_at	Region	Astn2	56079	4	64471994	65495676	-
17048	1417769_at	Region	None	None	14	11426901	11490818	-
17049	1457058_at	Region	Adamts2	216725	11	50354926	50556890	+
17050	1436157_at	Region	Ccar1	67500	10	62710847	62758641	-
17051	1419042_at	Region	Iigp1	60440	18	60615121	60617960	+
17052	1448620_at	Region	Fcgr3	14131	1	170986418	170994875	-
17053	1441059_at	Region	1700049G17Rik	73430	7	23299727	23300353	+
17054	1420395_a_at	Region	Kif9	16578	9	110518322	110566471	+
17055	1450059_at	Region	None	None	4	42918265	42926429	-
17056	1426687_at	Region	Map3k3	26406	11	105918079	105976535	+
17057	1454839_a_at	Region	D630044F24Rik	382073	9	44399433	44406876	-
17058	1448446_at	Region	Deaf1	54006	7	135699172	135729565	-
17059	1460376_a_at	Region	Cox15	226139	19	43279531	43297387	-
17060	1416080_at	Region	Adam15	11490	3	89096372	89106699	-
17061	1448194_a_at	Region	H19	14955	7	136988551	136991165	-
17062	1442715_at	Region	Tsga8	60600	X	78147334	78148580	-
17063	1458108_at	Region	Ptcd2	68927	13	95508567	95533596	-
17064	1455823_at	Region	Bbs4	102774	9	59438725	59470219	-
17065	1421797_a_at	Region	Snx12	55988	X	95813629	95823851	-
17066	1416919_a_at	Region	Nphp1	53885	2	127254585	127302707	-
17067	1436931_at	Region	Rfx4	71137	10	84732808	84767164	+
17068	1416233_at	Region	Eif3s2	54709	4	128619087	128627726	-
17069	1427590_at	Region	Zfp39	22698	11	58613782	58629854	-
17070	1445941_at	Region	Atbf1	11906	8	108012331	108254925	+
17071	1441490_at	Region	Tmem39a	67846	16	37385037	37413429	+
17072	1439161_at	Region	D19Ertd703e	52036	19	3243622	3364414	-
17073	1421539_at	Region	Zic4	22774	9	91269057	91275258	+
17074	1420276_x_at	Region	None	None	14	32540188	32540403	+
17075	1459851_x_at	Region	Riok1	71340	13	37576092	37600226	+
17076	1433724_at	Region	D15Ertd621e	210998	15	58436372	58467006	+
17077	1419114_at	Region	5430428G01Rik	66789	3	120073487	120141148	+
17078	1442013_at	Region	4930532D21Rik	320022	8	103739854	103803022	-
17079	1424021_at	Region	Arl6ip6	65103	2	53124273	53151188	+
17080	1455799_at	Region	Rorb	225998	19	18184597	18248073	-
17081	1440992_at	Region	3110052M02Rik	73229	17	19528262	19534084	+
17082	1425640_at	Region	Aff1	17355	5	102817913	102885914	+
17083	1437705_at	Region	None	None	7	10459348	10459633	-
17084	1455299_at	Region	1700110N18Rik	73569	16	64821348	64869062	+
17085	1459000_at	Region	Sympk	68188	7	15893026	15923378	+
17086	1455733_at	Region	A130052D22	330177	5	116369746	116402638	+
17087	1431638_at	Region	4930592A05Rik	75880	15	33592907	33651627	-
17088	1426374_at	Region	2410166I05Rik	76824	4	133486465	133493655	-
17089	1457834_at	Region	Yy1	22632	12	104264095	104287625	+
17090	1418515_at	Region	Mtf2	17765	5	107157468	107177436	+
17091	1456806_at	Region	A130010C12Rik	320211	8	116595124	116595813	+
17092	1456717_at	Region	Tead1	21676	7	106533225	106753684	+
17093	1423611_at	Region	Akp2	11647	4	136623000	136677584	-
17094	1449996_a_at	Region	Tpm3	59069	3	89883112	89911022	+
17095	1416678_at	Region	Cops3	26572	11	59543433	59565396	-
17096	1421014_a_at	Region	Clybl	69634	14	116879361	116970824	+
17097	1435836_at	Region	Pdk1	228026	2	71570967	71599744	+
17098	1431029_at	Region	1700016F23Rik	74711	2	152419442	152460140	+
17099	1436869_at	Region	Shh	20423	5	26906864	26917112	-
17100	1417534_at	Region	Itgb5	16419	16	32637841	32757227	+
17101	1448008_at	Region	Ankhd1	108857	18	36858496	36869011	+

17102	1446999_at	Region	None	None	2	169504669	169505227	+
17103	1457373_at	Region	4921525D07Rik	70880	1	110880851	110881643	-
17104	1431082_a_at	Region	Rabl3	67657	16	36359367	36391849	+
17105	1438545_at	Region	Slc25a5 /// LOC383528 /// LOC433326 /// LOC433923 /// LOC545025	11740	X	31416206	31419360	+
17106	1426218_at	Region	Glcci1	170772	6	8234651	8573400	+
17107	1417583_a_at	Region	Crlz1	65961	5	87822320	87822934	+
17108	1427457_a_at	Region	Bmp1	12153	14	64790547	64836177	-
17109	1418739_at	Region	Sgk2	27219	2	162444515	162471096	+
17110	1415730_at	Region	5730453I16Rik	269061	19	9721763	9744150	+
17111	1434344_at	Region	Gpkow	209416	X	5936153	5949197	+
17112	1426098_a_at	Region	Cast	12380	13	70751497	70865131	-
17113	1426086_a_at	Region	Fmr1	14265	X	63347151	63386559	+
17114	1451098_at	Region	Pcoln3	234852	8	122579147	122587621	-
17115	1448122_at	Region	Tcp1	21454	17	11607834	11615885	-
17116	1416948_at	Region	Mrpl23	19935	7	136946136	136953761	+
17117	1425732_a_at	Region	Mxi1	17859	19	52878345	52941221	+
17118	1459895_at	Region	C030010B13Rik	77310	2	173046327	173120005	-
17119	1424691_at	Region	5930434B04Rik	381356	2	26942112	26953251	+
17120	1451760_s_at	Region	2010001E11Rik /// AI317395	215929	10	40071458	40102306	-
17121	1446392_at	Region	Slit2	20563	5	46748038	47069334	+
17122	1442168_at	Region	A230071A22Rik	320380	6	35215568	35216390	-
17123	1451887_at	Region	Lrba	80877	3	85969078	86527074	+
17124	1419447_s_at	Region	None	None	5	62958072	63148140	+
17125	1443500_at	Region	Mllt10	17354	2	18097281	18254400	+
17126	1460720_at	Region	Trpc4ap	56407	2	155091245	155149269	-
17127	1425272_at	Region	Emp2	13731	16	9952848	9985199	-
17128	1439673_at	Region	E230008O15Rik	319862	5	30931786	30932506	+
17129	1418443_at	Region	Xpo1	103573	11	23150860	23192321	+
17130	1443783_x_at	Region	H2-Aa	14960	17	31984134	31989153	-
17131	1451911_a_at	Region	Ace	11421	11	105789071	105810342	+
17132	1431707_a_at	Region	Pscd3	19159	5	142683118	142770834	+
17133	1425581_s_at	Region	Galnt7	108150	8	56563802	56692236	-
17134	1423635_at	Region	Bmp2	12156	2	133066861	133075568	+
17135	1433440_x_at	Region	Uble1b	50995	7	29294488	29322320	-
17136	1439403_x_at	Region	Rnf12	19820	X	98558532	98582649	-
17137	1421155_at	Region	B3galt6	117592	4	154482891	154485011	-
17138	1428482_at	Region	Akap10	56697	11	61596949	61655868	-
17139	1451914_a_at	Region	Add2	11519	6	86507734	86549220	+
17140	1415843_at	Region	MGI:1929514	56716	17	22277138	22282663	-
17141	1423370_a_at	Region	Csnk1g2	103236	10	80753732	80771635	+
17142	1425019_at	Region	Ubxd4	217379	12	4054763	4083224	-
17143	1442504_at	Region	D18Ertd653e	52662	18	68295003	68486335	+
17144	1426480_at	Region	Sbds	66711	5	129417562	129427294	-
17145	1428534_at	Region	2310073E15Rik	75692	8	69284310	69286724	+
17146	1431292_a_at	Region	Ptk9l	23999	9	106196703	106208982	+
17147	1434204_x_at	Region	Shmt2	108037	10	127253776	127259072	-
17148	1447649_x_at	Region	Dnajc1	13418	2	18259145	18434517	-
17149	1421009_at	Region	Rsad2	58185	12	22988909	23002166	-
17150	1460610_at	Region	9430057O19Rik	231093	5	29347796	29355631	+
17151	1419805_s_at	Region	Ggps1	14593	13	13494995	13505951	-
17152	1460049_s_at	Region	1500015O10Rik	78896	1	44025389	44037264	+
17153	1418341_at	Region	Rab4a	19341	8	123093097	123122359	+
17154	1428193_at	Region	Usp9x	22284	X	11310477	11410726	+
17155	1438084_at	Region	Adam23	23792	1	63739150	63885988	+
17156	1420987_at	Region	Kif3b	16569	2	152748365	152789150	+
17157	1427471_at	Region	Fbxl3	50789	14	97626484	97644968	-
17158	1455178_at	Region	Rutbc1	97761	11	74574922	74622729	-
17159	1435789_x_at	Region	Rims2	116838	15	39098033	39582362	+
17160	1418681_at	Region	Glt28d1	67574	X	137758067	137765234	+
17161	1438012_at	Region	Ppm1l	242083	3	68987383	69225607	+

17162	1431755_a_at	Region	1300013D05Rik	67480	11	97566702	97587643	-
17163	1443593_at	Region	C330012H03Rik	319765	16	20832300	20935863	-
17164	1447576_at	Region	2010001K21Rik	69829	13	46608975	46609341	+
17165	1456787_at	Region	D130020G16Rik	320972	16	76627648	76628158	+
17166	1416371_at	Region	Apod	11815	16	30106394	30123724	-
17167	1422684_a_at	Region	None	None	6	33334773	34064398	+
17168	1449374_at	Region	Pipox	19193	11	77606274	77619443	-
17169	1443491_at	Region	Ptprk	19272	10	28105024	28627423	+
17170	1427913_at	Region	Rwdd1	66521	10	34056530	34079596	-
17171	1421157_at	Region	Fzd3	14365	14	59729279	59780310	-
17172	1458588_at	Region	None	None	3	68221154	68221467	+
17173	1431614_at	Region	2810049E08Rik	72677	NONE	NONE	NONE	NONE
17174	1418924_at	Region	2400009B11Rik	66985	7	135617908	135620645	+
17175	1421954_at	Region	Crkl	12929	16	16222955	16257223	+
17176	1432547_at	Region	C030005H24Rik	78537	13	43266875	43267412	+
17177	1452375_at	Region	Aldh4a1	212647	4	138504271	138530948	+
17178	1429292_a_at	Region	2310046K01Rik	69698	2	151460988	151466192	+
17179	1416748_a_at	Region	Mre11a	17535	9	14618147	14667859	+
17180	1448667_x_at	Region	Tob2	57259	15	81899631	81902862	-
17181	1452148_at	Region	Lrpap1	16976	5	33580650	33593377	-
17182	1449237_at	Region	Aloxe3	23801	11	68852035	68874772	+
17183	1427150_at	Region	Mll3	231051	5	23741347	23769624	-
17184	1443744_at	Region	C030036D22Rik	77607	18	72562172	72562505	-
17185	1425528_at	Region	Prrx1	18933	1	163163407	163227993	-
17186	1442620_at	Region	D430018P08	234353	8	66818806	66920716	-
17187	1438660_at	Region	Gcnt2	14538	13	40420765	40521482	+
17188	1448695_at	Region	Prkci	18759	3	30407337	30464163	+
17189	1427896_at	Region	2400003N08Rik	71954	5	116220464	116244713	-
17190	1451182_s_at	Region	C730048E16Rik	223690	15	79104380	79114145	-
17191	1441479_at	Region	Ppm1b	19043	17	82787863	82846969	+
17192	1455246_at	Region	None	None	9	110278696	110279940	+
17193	1426694_at	Region	9030624J02Rik	71517	7	112616598	112717342	+
17194	1460396_at	Region	Ddx54	71990	5	119764937	119780399	+
17195	1444445_at	Region	C77648	97695	NONE	NONE	NONE	NONE
17196	1426578_s_at	Region	Snapap	20615	3	90298452	90301491	-
17197	1436847_s_at	Region	Cdca8	52276	4	123945578	123964016	-
17198	1435684_at	Region	Abcc5	27416	16	19102921	19197967	-
17199	1434646_s_at	Region	Sap18	20220	14	52333126	52339849	+
17200	1452868_at	Region	Usp24	329908	4	105320122	105400231	+
17201	1441787_at	Region	C230027N18Rik	330940	9	52183805	52184071	-
17202	1444108_at	Region	None	None	4	58968543	58969021	+
17203	1435369_at	Region	C78212	380601	2	130127699	130175724	-
17204	1450341_at	Region	Pcdhb8	93879	18	37578993	37581332	+
17205	1428704_at	Region	Zfp661	72180	2	127089379	127098521	-
17206	1424820_a_at	Region	Ndfip1	65113	18	38640514	38688909	+
17207	1417543_at	Region	Rps6ka2	20112	17	6670716	6803866	+
17208	1459229_at	Region	None	None	10	32625765	32626466	-
17209	1436058_at	Region	Rsad2	58185	12	22988909	23002166	-
17210	1422778_at	Region	Taf9	108143	13	96808152	96810020	+
17211	1459443_at	Region	Kcnq1	16535	7	137520467	137840061	+
17212	1441114_at	Region	9330156P08Rik	320141	1	180081784	180082898	+
17213	1439280_at	Region	B230396O12Rik	242800	4	153348589	153359487	+
17214	1423284_at	Region	Mansc1	67729	6	135366678	135389956	-
17215	1416931_at	Region	Nif3l1	65102	1	58752501	58767146	+
17216	1435669_at	Region	Zfp532	328977	18	65813456	65922159	+
17217	1451352_s_at	Region	Mta3	116871	17	81539641	81648553	+
17218	1458119_at	Region	Slc25a27	74011	17	41143342	41168531	-
17219	1434658_at	Region	3110056O03Rik	73218	10	80987323	81000771	+
17220	1452978_at	Region	2900055D14Rik	72982	19	9767378	9773594	-
17221	1416194_at	Region	Cyp4b1	13120	4	114583633	114606610	-
17222	1443352_at	Region	C77614	30873	14	37126594	37127165	-
17223	1421224_a_at	Region	Tcf2	21410	11	83579041	83633339	+
17224	1449653_at	Region	None	None	2	52067092	52067578	+
17225	1440379_at	Region	Slc1a5	20514	7	13733002	13749749	+
17226	1429087_at	Region	1110054O05Rik	66209	4	59717529	59727289	-

17227	1436394_at	Region	Trim37	68729	11	86855069	86948674	+
17228	1456003_a_at	Region	Slc1a4	55963	11	20196998	20227464	-
17229	1418247_s_at	Region	Rbm9	93686	15	77134294	77358885	-
17230	1436666_at	Region	Flot2	14252	11	77765963	77786091	+
17231	1430075_at	Region	Sf3b3	101943	8	110108214	110144495	-
17232	1418088_a_at	Region	Stx8	55943	11	67692133	67932806	+
17233	1453936_at	Region	None	None	17	31916915	31920078	-
17234	1452379_at	Region	Auts2	319974	5	130627794	130728707	-
17235	1426720_at	Region	Apbb2	11787	5	65067237	65384014	-
17236	1439925_at	Region	None	None	3	56918591	56918943	-
17237	1438247_at	Region	Klhl15	236904	X	88895662	88914725	+
17238	1456916_at	Region	Nsd1	18193	13	53879224	53879921	+
17239	1422762_at	Region	Kif17	16559	4	137143512	137182753	+
17240	1451646_at	Region	Pdik1l	230809	4	133235901	133248731	-
17241	1424213_at	Region	1200002M06Rik	71707	4	146926859	146937113	-
17242	1429025_a_at	Region	Hexim2	71059	11	102954403	102960965	+
17243	1457486_at	Region	None	None	9	43187994	43188540	-
17244	1429523_a_at	Region	Slc39a5	72002	10	128132753	128137336	-
17245	1451004_at	Region	Acvr2a	11480	2	48746319	48834325	+
17246	1434392_at	Region	Usp34	17847	11	23201764	23383978	+
17247	1426933_at	Region	Oxsr1	108737	9	119232699	119316671	-
17248	1443237_at	Region	None	None	4	76915066	76915728	-
17249	1421324_a_at	Region	Akt2	11652	7	22980718	23014488	+
17250	1436763_a_at	Region	Klf9	16601	19	22379147	22404832	+
17251	1448455_at	Region	Cln8	26889	8	14267371	14278844	+
17252	1425937_a_at	Region	Hexim1	192231	11	102937414	102940814	+
17253	1417599_at	Region	Cd276	102657	9	58638375	58656877	-
17254	1429114_at	Region	Sestd1	228071	2	76884304	76942838	-
17255	1448310_at	Region	Ick	56542	9	78282435	78336986	+
17256	1450758_at	Region	Cntnap2	66797	6	45854973	47502306	+
17257	1457723_at	Region	Pspc1	66645	14	51244701	51300567	-
17258	1429674_at	Region	1700113H08Rik	76640	10	87194215	87205104	+
17259	1448374_at	Region	Med28	66999	5	44279069	44284371	+
17260	1437729_at	Region	None	None	3	15039734	15040196	-
17261	1438263_at	Region	9430020K01Rik	240185	18	4653875	4685495	+
17262	1446983_at	Region	Hrpt2	214498	1	143439562	143534747	-
17263	1452430_s_at	Region	Sfrs1	110809	11	87775771	87779641	+
17264	1428376_at	Region	4932415G12Rik	67723	10	94647892	94662897	-
17265	1433616_a_at	Region	2310028O11Rik	433771	4	137984815	138012312	-
17266	1422978_at	Region	Cybb	13058	X	7675646	7708207	-
17267	1435953_at	Region	Btaf1	107182	19	36299109	36355422	+
17268	1450425_a_at	Region	2700062C07Rik	68046	18	24699366	24706263	+
17269	1450586_at	Region	Bdkrb1	12061	12	101051651	101052655	+
17270	1441397_at	Region	Pard3	93742	8	126354044	126919239	+
17271	1435030_at	Region	Upf2	326622	2	5868739	5973970	+
17272	1451300_a_at	Region	Chmp7	105513	14	64032927	64048462	-
17273	1446484_at	Region	Mef2c	17260	13	79604158	79763912	+
17274	1428317_at	Region	None	None	X	70047755	70057514	+
17275	1426572_at	Region	Me2	107029	18	74004269	74049621	-
17276	1423168_at	Region	Prei3	19070	1	55432452	55455260	+
17277	1433439_at	Region	Cpne1	266692	2	155528782	155568901	-
17278	1428755_at	Region	3526402H21Rik	66683	1	64893251	64897055	+
17279	1426521_at	Region	Lin10	234678	8	104520933	104548681	+
17280	1444258_at	Region	Actn4	60595	7	24299701	24368752	-
17281	1429241_at	Region	9130020G22Rik	74552	4	134332771	134375796	-
17282	1458932_at	Region	Pex2	58869	3	32358790	32491207	-
17283	1448494_at	Region	Gas1	14451	13	58831933	58834893	-
17284	1424357_at	Region	Tmem45b	235135	9	31348544	31386599	-
17285	1438736_at	Region	6330441O12Rik	331401	X	36316177	36444623	-
17286	1458099_at	Region	None	None	12	37769208	37769889	+
17287	1432676_at	Region	None	None	19	45699731	45701598	+
17288	1441865_at	Region	None	None	7	85102845	85103077	+
17289	1431132_x_at	Region	0610007P22Rik /// LOC546103	546103	8	111533219	111551012	-
17290	1437120_at	Region	4732481H14Rik	209131	4	59749298	59843221	+

17291	1426023_a_at	Region	Rabep1	54189	11	70570583	70666598	+
17292	1439438_a_at	Region	1110005A23Rik	66118	10	128559009	128614707	+
17293	1443741_x_at	Region	Whsc1	107823	5	32383490	32383756	+
17294	1426315_a_at	Region	6330416G13Rik	230279	4	62651547	62677546	+
17295	1427316_s_at	Region	AA881470	231841	5	139703855	139718166	+
17296	1430378_at	Region	2900011G08Rik	72933	14	72166170	72167530	-
17297	1457926_at	Region	None	None	NONE	NONE	NONE	NONE
17298	1430057_s_at	Region	2810002D13Rik	66606	2	120118087	120123123	-
17299	1457339_at	Region	Ccm2	216527	11	6441744	6491532	+
17300	1428457_at	Region	5830472M02Rik	76080	2	163059320	163074164	+
17301	1454707_at	Region	2310035C23Rik	227446	1	105555177	105695178	+
17302	1449997_at	Region	Tpm3	59069	3	89883112	89911022	+
17303	1427200_at	Region	Zranb1	360216	7	127315953	127350282	+
17304	1459196_at	Region	Dnmbp	71972	19	43392373	43456795	-
17305	1417551_at	Region	Cln3	12752	7	120621131	120632951	-
17306	1455291_s_at	Region	Znrf2	387524	6	54961241	55034061	+
17307	1443669_at	Region	Zfyve28	231125	5	32700148	32700580	-
17308	1455104_at	Region	Mad	17119	6	87085070	87103255	-
17309	1416795_at	Region	Cryl1	68631	14	51806438	51929961	-
17310	1455545_at	Region	1110065P20Rik	68920	4	123876604	123877771	-
17311	1443979_at	Region	A830021K08Rik	320427	14	113261116	113262926	+
17312	1420464_s_at	Region	Pira1 /// Pira2 /// Pira3 /// Pira4 /// Pira6 /// Pirb /// LOC546027	18722	7	5565	13810	-
17313	1445336_at	Region	None	None	NONE	NONE	NONE	NONE
17314	1436969_at	Region	1700013E18Rik	71845	3	153718138	153756017	+
17315	1432065_at	Region	3100003L13Rik	73099	8	114290214	114291684	+
17316	1442165_at	Region	4930438O05Rik	78795	1	85961103	86084595	+
17317	1433516_a_at	Region	1110002M09Rik	66915	1	92464637	92469415	-
17318	1418875_at	Region	Syngr4	58867	7	39974135	39981975	-
17319	1447544_at	Region	1700020L24Rik	66330	11	83165685	83169205	+
17320	1455091_at	Region	3222402P14Rik	235542	9	101055227	101161626	-
17321	1430705_at	Region	2810454F19Rik	72802	15	76128535	76129555	-
17322	1436572_at	Region	4732496G21Rik	320162	11	106610364	106639918	+
17323	1442005_at	Region	AW987390	100467	4	148467320	148468165	-
17324	1456027_at	Region	BB131279	103061	X	133486477	133488005	-
17325	1434488_at	Region	Arfrp1	76688	2	181074790	181082318	-
17326	1423139_at	Region	Wdr4	57773	17	29304618	29321311	-
17327	1451938_a_at	Region	Sntb1	20649	15	55643660	55798909	-
17328	1438709_at	Region	D11Ertd498e	52639	11	109394610	109432478	-
17329	1427795_s_at	Region	None	None	NONE	NONE	NONE	NONE
17330	1460017_at	Region	LOC434179	434179	7	36842546	36875156	-
17331	1459991_at	Region	Myo9a	270163	9	59868073	60046149	+
17332	1452974_at	Region	Nol8	70930	13	49249941	49275842	+
17333	1435679_at	Region	Optn	71648	2	4937914	4981210	-
17334	1454588_at	Region	9430006E15Rik	77247	9	20558366	20559139	-
17335	1429099_at	Region	1110051B16Rik	278672	14	24358464	24365306	+
17336	1433272_at	Region	A430110A21Rik	77788	NONE	NONE	NONE	NONE
17337	1455322_at	Region	Ttc21b	73668	2	66040107	66112017	-
17338	1423659_a_at	Region	Tbc1d17	233204	7	38915960	38924007	-
17339	1421957_a_at	Region	Pcyt1a	13026	16	31261076	31281386	+
17340	1439256_x_at	Region	Tm7sf1	83924	13	12777506	12812482	-
17341	1425241_a_at	Region	Wsb1	78889	11	78965045	78980093	-
17342	1425711_a_at	Region	Akt1	11651	12	108133208	108153577	-
17343	1416143_at	Region	Atp5j	11957	16	83935942	83943643	-
17344	1435006_s_at	Region	Abcb7	11306	X	98882022	99015261	-
17345	1446163_at	Region	None	None	19	60063579	60064272	-
17346	1452764_at	Region	Socs6	54607	18	89012087	89038416	-
17347	1425393_a_at	Region	Map2k7	26400	8	3593945	3601130	+
17348	1428326_s_at	Region	Hrsp12	15473	15	34483848	34494901	-
17349	1419675_at	Region	Ngfb	18049	3	101897619	101948696	+
17350	1429345_at	Region	D2Ertd435e	51885	2	120685055	120710967	+
17351	1459818_x_at	Region	Zfp261	56364	X	96005746	96022210	-
17352	1416548_at	Region	Slc35b4	58246	6	34249834	34271009	-

17353	1423073_at	Region	Cmpk	66588	4	113919543	113948234	-
17354	1435213_at	Region	Nhlrc1	105193	13	46610707	46612969	-
17355	1418102_at	Region	Hes1	15205	16	28870268	28872833	+
17356	1425985_s_at	Region	Masp1	17174	16	22233196	22304246	-
17357	1428485_at	Region	Car12	76459	9	66838894	66891997	+
17358	1460589_at	Region	Zfp597	71063	16	3533092	3535419	-
17359	1452372_at	Region	Bsdc1	100383	4	128488792	128515548	+
17360	1427553_at	Region	None	None	17	49385144	49387422	+
17361	1459879_at	Region	BC031575	223989	16	12874640	12899363	-
17362	1428214_at	Region	Tomm7	66169	5	22303551	22308004	-
17363	1455000_at	Region	Gpr68	238377	12	96322143	96333339	-
17364	1457173_at	Region	Ywhae	22627	11	75458597	75491496	+
17365	1442914_at	Region	None	None	6	97618288	97618934	-
17366	1442858_at	Region	Phr1	105689	14	97658865	97891435	-
17367	1443679_at	Region	2510003B16Rik	76786	4	115082881	115096735	+
17368	1441228_at	Region	---	381823	6	135739376	135741778	+
17369	1456402_at	Region	A330076H08Rik	320026	7	55716635	55717327	-
17370	1428312_at	Region	2810002D13Rik	66606	2	120118087	120123123	-
17371	1425907_s_at	Region	Amot	27494	X	138889991	138927092	-
17372	1448365_at	Region	Exosc7	66446	9	123131019	123153902	+
17373	1457779_at	Region	1110046J04Rik	68808	13	33484935	33491803	+
17374	1454638_a_at	Region	Pah	18478	10	87495513	87557284	+
17375	1421486_at	Region	Egr3	13655	14	64393373	64396085	+
17376	1459800_s_at	Region	Map3k4	26407	17	10867071	10957848	-
17377	1444140_at	Region	Pum1	80912	4	129460274	129578157	+
17378	1456358_at	Region	Etv3	27049	3	87270684	87284681	+
17379	1422698_s_at	Region	Jarid2	16468	13	44305547	44495137	+
17380	1427342_at	Region	5330408N05Rik	320720	2	69542220	69568006	-
17381	1459030_at	Region	None	None	NONE	NONE	NONE	NONE
17382	1442663_at	Region	None	None	4	12031968	12032589	+
17383	1418879_at	Region	9030611O19Rik	104943	12	27650830	27656802	+
17384	1422664_at	Region	Rab10	19325	12	3108	12388	-
17385	1451173_at	Region	Lrrc49	102747	9	60704784	60805455	-
17386	1427032_at	Region	Herc4	67345	10	63208216	63282585	+
17387	1458096_at	Region	None	None	X	155564714	155565242	-
17388	1443062_at	Region	Auts2	319974	5	130627794	130728707	-
17389	1431769_at	Region	2210406O10Rik	76710	5	34943818	34952559	-
17390	1455728_at	Region	Pten	19211	19	32083643	32146174	+
17391	1438444_at	Region	A230091H23	328971	18	62777723	62889947	+
17392	1459240_at	Region	D8Ertd325e	66855	8	122748919	122778703	+
17393	1459948_at	Region	Ghr	14600	15	3098508	3365766	-
17394	1443422_at	Region	2410089E03Rik	73692	15	7956639	8058734	+
17395	1452118_at	Region	2600005C20Rik	72462	17	29844491	29868935	+
17396	1440197_at	Region	BC032203	210982	17	44309813	44343803	-
17397	1432255_at	Region	Mast1	56527	8	84179304	84205636	-
17398	1438010_at	Region	D4Ertd429e	230917	4	148217245	148230336	-
17399	1453156_s_at	Region	Zadh1	77219	12	81153770	81182471	+
17400	1443434_s_at	Region	Plxnc1	54712	10	94767461	94918749	-
17401	1435181_at	Region	AI461788	231506	5	99460839	99519278	-
17402	1458377_at	Region	Crsp7	70625	8	71645137	71698918	-
17403	1431892_a_at	Region	Plcd3	72469	11	102891399	102922691	-
17404	1423711_at	Region	Ndufaf1	69702	2	119169300	119176647	-
17405	1423245_at	Region	Cops7a	26894	6	125614820	125621370	-
17406	1456415_at	Region	Zfp451	98403	1	34030702	34083136	-
17407	1460348_at	Region	Mad2l2	71890	4	146632852	146638058	+
17408	1444196_at	Region	AI662501	103817	11	59570266	59570909	-
17409	1425993_a_at	Region	Hsp105	15505	5	148555092	148574465	-
17410	1443533_at	Region	Ppp2r5e	26932	12	72307243	72452562	-
17411	1445701_at	Region	None	None	1	133563717	133563946	-
17412	1443670_at	Region	2010001J22Rik	70113	15	89430323	89430747	-
17413	1427992_a_at	Region	Rab12	19328	17	64262451	64265612	-
17414	1430028_at	Region	2210018M11Rik	233545	7	92710530	92747815	-
17415	1434024_at	Region	Nphp4	260305	4	150970504	151055496	+
17416	1434460_at	Region	Bbs4	102774	9	59438725	59470219	-
17417	1440934_at	Region	6230409E13Rik	76132	4	22000704	22020260	+

17418	1446906_at	Region	C81615	97548	NONE	NONE	NONE	NONE
17419	1426377_at	Region	Zfp281	226442	1	136473723	136477067	+
17420	1456557_at	Region	1700041C02Rik	73332	4	118281798	118374408	-
17421	1449628_s_at	Region	Stard7	99138	2	126784092	126812786	+
17422	1416016_at	Region	Tap1	21354	17	31893511	31902354	+
17423	1437462_x_at	Region	Mmp15	17388	8	94636817	94655679	+
17424	1426833_at	Region	Eif4g3	230861	4	136874731	137087744	+
17425	1451596_a_at	Region	Sphk1	20698	11	116354440	116357763	+
17426	1438952_x_at	Region	None	None	6	47633471	47633699	-
17427	1435416_x_at	Region	Pigq	14755	17	3046	4385	-
17428	1425845_a_at	Region	Shoc2	56392	19	53523183	53611744	+
17429	1428026_at	Region	None	None	2	169394568	169421827	+
17430	1460429_at	Region	Cdc5l	71702	17	42903188	42944108	-
17431	1425305_at	Region	Zfp295	114565	16	140871	154115	+
17432	1446649_at	Region	Centd1	212285	5	61409120	61555599	-
17433	1415999_at	Region	Hey1	15213	3	8650290	8653917	-
17434	1443539_at	Region	None	None	3	87363320	87363857	-
17435	1429420_at	Region	4931414P19Rik	74359	14	49099522	49167302	-
17436	1426253_at	Region	4933428G09Rik	66768	5	47136215	47152579	+
17437	1425495_at	Region	Zfp62	22720	11	48957160	48970604	+
17438	1450095_a_at	Region	Acyp1	66204	12	82141604	82149527	-
17439	1443183_at	Region	Huwe1	59026	NONE	NONE	NONE	NONE
17440	1419664_at	Region	Srr	27364	11	74632454	74651361	-
17441	1418421_at	Region	Bcl6b	12029	11	69949786	69955379	-
17442	1429536_at	Region	2310040C09Rik	69640	11	61410106	61433797	+
17443	1424016_at	Region	2310007F21Rik	66939	9	63723018	63762391	+
17444	1426523_a_at	Region	Gnpda2	67980	5	68341941	68359622	-
17445	1420812_at	Region	Hdac7a	56233	15	97868326	97887406	-
17446	1416751_a_at	Region	Ddx20	53975	3	105474108	105483403	-
17447	1429119_at	Region	4833421E05Rik	67732	12	18821943	18829156	-
17448	1420413_at	Region	Slc7a11	26570	3	49959006	50031625	-
17449	1438636_s_at	Region	None	None	9	114157277	114157569	+
17450	1459791_at	Region	Dnajc1	13418	2	18259145	18434517	-
17451	1444230_at	Region	2900046D03Rik	402731	7	120849466	120849638	+
17452	1426533_at	Region	Nol5a	67134	2	129788312	129793166	+
17453	1447165_at	Region	None	None	17	63707361	63707841	-
17454	1440530_at	Region	AU019752	101756	7	4267037	4267674	+
17455	1444072_at	Region	2610528H13Rik	67179	14	60364796	60394022	+
17456	1445664_at	Region	A330103N21Rik	77773	7	62004449	62004857	+
17457	1448587_at	Region	Tbc1d10a	103724	11	4081620	4110292	+
17458	1417206_at	Region	Urod	22275	4	115949123	115953269	-
17459	1453319_at	Region	Ccar1	67500	10	62710847	62758641	-
17460	1417033_at	Region	Ube2g2	22213	10	77733757	77757426	+
17461	1438750_at	Region	Atrx	22589	X	100401518	100530707	-
17462	1427677_a_at	Region	Sox6	20679	7	109336962	109662307	-
17463	1439223_at	Region	Tmod2	50876	9	75710299	75748747	-
17464	1448771_a_at	Region	Fth1	14319	19	9179200	9181589	+
17465	1435442_at	Region	Wdsof1	223499	15	39012633	39046557	+
17466	1451501_a_at	Region	Ghr	14600	15	3098508	3365766	-
17467	1439094_at	Region	Cltc	67300	11	86422645	86485484	-
17468	1460500_at	Region	5033421C21Rik	75963	14	3569655	4235927	+
17469	1447982_at	Region	1110008P14Rik	73737	2	32311270	32314046	-
17470	1417998_at	Region	Ptges3	56351	10	127795692	127813964	+
17471	1426367_at	Region	Cab39l	69008	14	53976044	54083966	+
17472	1419968_at	Region	C77370	245555	X	98684555	98802550	-
17473	1418380_at	Region	Terf1	21749	1	15983322	16021093	+
17474	1429434_at	Region	Pik3ca	18706	3	31844384	31871009	+
17475	1445213_at	Region	Cyld	74256	8	87990637	88033868	+
17476	1454997_at	Region	Msrb3	320183	10	120367469	120482385	-
17477	1447915_x_at	Region	BC054438	407831	17	22863553	22886965	-
17478	1448105_at	Region	Prm2	19119	16	7040255	7040971	-
17479	1455411_at	Region	None	None	7	91771241	91772267	-
17480	1435517_x_at	Region	Ralb	64143	1	119221743	119256062	-
17481	1435951_at	Region	Grip1	74053	10	119395694	119653294	+
17482	1454156_at	Region	A230104H11Rik	77759	1	58079224	58105244	+

17483	1430036_at	Region	2310015B20Rik	69563	10	70267391	70273858	+
17484	1436428_at	Region	Chrnb2	11444	3	89559631	89570768	-
17485	1423605_a_at	Region	Mdm2	17246	10	117379897	117401708	-
17486	1454695_at	Region	Wdr18	216156	10	80091007	80092606	+
17487	1452133_at	Region	Uqcrh	66576	4	115025870	115033953	-
17488	1426723_at	Region	Wdr48	67561	9	119904848	119936532	+
17489	1447708_x_at	Region	Pde2a	207728	7	95527997	95619103	+
17490	1445437_at	Region	2310015A05Rik	72315	16	16417438	16421706	+
17491	1441033_at	Region	8430438D04Rik	278279	10	105080153	105466941	-
17492	1438869_at	Region	4930515G01Rik	67642	5	113883363	113884609	-
17493	1456302_at	Region	Pex6	224824	17	44222425	44236648	+
17494	1422777_at	Region	C1ql1	23829	11	102760822	102767550	-
17495	1429084_at	Region	Vezf1	22344	11	87796271	87812715	+
17496	1434802_s_at	Region	Ntf3	18205	6	126767210	126830735	-
17497	1450662_at	Region	Tesk1	21754	4	43357861	43363983	+
17498	1440271_at	Region	None	None	3	96286466	96287414	+
17499	1428407_at	Region	Hnrpa0 /// LOC544935	544935	13	56751138	56753586	-
17500	1452292_at	Region	Ap2b1	71770	11	83030763	83131001	+
17501	1422265_at	Region	Brs3	12209	X	51797788	51803472	+
17502	1456958_at	Region	C230072F16Rik	320784	17	78730407	78731047	+
17503	1416681_at	Region	Ube3a	22215	7	53507062	53583196	+
17504	1445443_at	Region	None	None	7	39515619	39516240	+
17505	1423410_at	Region	Meig1	104362	2	3326314	3339919	-
17506	1450727_a_at	Region	Poldip2	67811	11	78237955	78248395	+
17507	1453320_at	Region	1700027A23Rik	76420	3	105792499	105810475	-
17508	1427986_a_at	Region	Col16a1	107581	4	129075001	129126388	+
17509	1439011_at	Region	None	None	12	28978759	28979471	-
17510	1433188_at	Region	B230112I24Rik	77984	6	45275126	45276301	+
17511	1458664_at	Region	A430103D13Rik	77775	12	73200611	73200870	-
17512	1424447_at	Region	1700030K09Rik	72254	8	71592026	71610746	+
17513	1432583_at	Region	2900042E19Rik	72941	17	79271105	79272456	-
17514	1444239_at	Region	BC023488	237221	X	159769900	159789915	+
17515	1428626_at	Region	Lysmd2	70082	9	75763320	75775350	+
17516	1445079_at	Region	None	None	15	86404486	86405109	+
17517	1416392_a_at	Region	Atp6v0c	11984	17	21967459	21973000	-
17518	1457887_at	Region	Scospondin	243369	6	48581108	48634114	+
17519	1448499_a_at	Region	Ephx2	13850	14	60611797	60651920	-
17520	1436132_at	Region	D430036N24Rik	98949	2	156467410	156468077	-
17521	1452555_at	Region	None	None	6	65580099	65581148	+
17522	1417582_s_at	Region	Dhodh	56749	8	108891153	108906299	-
17523	1420619_a_at	Region	Aes	14797	10	81695504	81702143	+
17524	1437801_at	Region	Lpp	210126	16	23179019	23766559	+
17525	1438254_at	Region	1110007A13Rik	210711	7	122745901	122814730	-
17526	1433709_at	Region	Cant1	76025	11	118231701	118240124	-
17527	1459996_at	Region	Cacna1a	12286	8	83678856	83903623	+
17528	1452688_at	Region	Prpf39	328110	12	61831742	61841076	+
17529	1415762_x_at	Region	Mrpl52	68836	14	48943738	48946676	+
17530	1456577_x_at	Region	Pitrm1	69617	13	6445093	6477254	+

			BC003993 /// LOC432468 /// LOC432512 /// LOC432823 /// LOC432857 /// LOC432890 /// LOC432924 /// LOC432932 /// LOC433043 /// LOC433244 /// LOC433373 /// LOC433596 /// LOC433657 /// LOC433893 /// LOC433915 /// LOC434014 /// LOC434071 /// LOC434166 ///					
17531	1424607_a_at	Region	LOC4	432468	10	66312437	66314332	+
17532	1439152_at	Region	BC052066	234814	8	120453461	120463839	-
17533	1451480_at	Region	E2f4	104394	8	104593291	104600998	+
17534	1452224_at	Region	Morc3	338467	16	93003378	93047429	+
17535	1429629_at	Region	Ezh1	14055	11	101012203	101049035	-
17536	1459911_at	Region	D030068L24Rik	237988	11	115203023	115215330	+
17537	1420174_s_at	Region	Tax1bp1	52440	6	52858073	52910809	+
17538	1427928_s_at	Region	Cdc91l1	228812	2	154735189	154814361	-
17539	1451244_a_at	Region	Zfp422	67255	6	117061320	117066281	-
17540	1417918_at	Region	Mrpl11	66419	19	4751105	4753733	+
17541	1427056_at	Region	Adamts15	235130	9	30819450	30825116	-
17542	1437532_at	Region	2810055G22Rik	108086	5	38	2110	-
17543	1434035_at	Region	Dnajb6	23950	5	28186480	28217572	+
17544	1454236_a_at	Region	C030004A17Rik	109229	9	35131009	35181307	-
17545	1451052_at	Region	Cog8	97484	8	106338850	106354346	-
17546	1448643_at	Region	Ssna1	68475	2	25197696	25204574	-
17547	1437102_at	Region	Ythdf1	228994	2	180621335	180637894	-
17548	1441360_at	Region	Rps6ka3	110651	X	152855525	152962890	+
17549	1416457_at	Region	Ddah2	51793	17	32762881	32765875	+
17550	1439635_at	Region	Rgs9	19739	11	109046444	109119218	-
17551	1458876_at	Region	Enah	13800	1	181864370	181978518	-
17552	1424835_at	Region	Gstm4	14865	3	107836150	107840530	-
17553	1443399_at	Region	AU021001	99801	3	85975567	85976162	+
17554	1458535_at	Region	None	None	17	34839479	34840343	+
17555	1453137_at	Region	Fbxo30	71865	10	11158930	11174522	+
17556	1424033_at	Region	Sfrs7	225027	17	78017474	78024683	-
17557	1428799_at	Region	4930431B11Rik	75782	9	83721124	83770867	-
17558	1436877_at	Region	Lrch2	210297	X	140910434	140993991	-
17559	1441526_at	Region	Mbtd1	103537	11	93733496	93766406	+
17560	1418546_a_at	Region	1700095N21Rik	76630	19	33523377	33571435	+
17561	1454679_at	Region	D8Ertd457e	101994	8	13248344	13259184	+
17562	1433975_at	Region	Cdk10	234854	8	122599724	122607137	+
17563	1441980_at	Region	C030007I09Rik	109250	3	131729850	131732085	+
17564	1438819_at	Region	None	None	1	52755942	52756828	-
17565	1438519_at	Region	4930429H24Rik	75785	16	18869169	18899359	+
17566	1452818_at	Region	Ttf2	74044	3	100364769	100395573	-
17567	1448024_at	Region	B430320C24Rik	319900	15	11653235	11653847	-
17568	1440200_at	Region	9630031F12Rik	58227	5	44288471	44397958	-
17569	1417052_at	Region	Psmb3	26446	11	97524521	97534587	+
17570	1460144_at	Region	BC052040	399568	2	115095624	115292616	+
17571	1459701_x_at	Region	None	None	4	97679915	97680167	+
17572	1441338_at	Region	5930412G12Rik	319616	5	127749366	127770917	-
17573	1450022_at	Region	Gtpbp1	14904	15	79743329	79772459	+
17574	1417794_at	Region	Zfp261	56364	X	96005746	96022210	-
17575	1434275_at	Region	Nkd2	72293	13	69880109	69907684	-
17576	1441456_at	Region	Mmp24	17391	2	155232322	155273266	+
17577	1423303_at	Region	Paxip1	55982	5	26181592	26232266	-

17578	1457279_at	Region	Sirt7	209011	11	120439464	120446060	-
17579	1444223_at	Region	Hcrtr2	387285	9	76364006	76461704	-
17580	1434071_a_at	Region	Pelo	105083	13	111502233	111504767	-
17581	1451822_a_at	Region	Scrn2	217140	11	96851081	96855047	+
17582	1440896_at	Region	Sfrs15	224432	16	89386383	89441935	-
17583	1428495_at	Region	2410003K15Rik	75593	6	49205067	49220188	+
17584	1430439_at	Region	2810465F10Rik	78771	13	72442758	73090988	+
17585	1428786_at	Region	Nckap1l	105855	15	103516906	103561881	+
17586	1456553_at	Region	None	None	4	107510627	107511000	+
17587	1450842_a_at	Region	Cenpa	12615	5	29125844	29133768	+
17588	1423456_at	Region	Bzw2	66912	12	32698936	32763848	-
17589	1458340_at	Region	None	None	16	43602143	43602419	-
17590	1455254_at	Region	4833420G11Rik	108863	2	180436492	180438558	+
17591	1454139_at	Region	4930405N21Rik	73797	NONE	NONE	NONE	NONE
17592	1437322_at	Region	Rbm4	19653	19	4573943	4582649	-
17593	1430132_at	Region	Krt25d	70843	11	99185959	99196076	-
17594	1443051_at	Region	None	None	4	148563720	148564156	+
17595	1440430_at	Region	A130004G07Rik	320687	2	105442568	105443237	-
17596	1426496_at	Region	Wdr55	67936	18	36984004	36987473	+
17597	1459951_at	Region	4732464A07Rik	320232	11	86232678	86232978	-
17598	1441690_at	Region	Cdh8	12564	8	98317068	98691929	-
17599	1430749_at	Region	2810040C05Rik	72719	4	34994564	34995919	-
17600	1453247_at	Region	2810031P15Rik	72701	4	62056769	62225062	+
17601	1419076_a_at	Region	Brca2	12190	5	149463480	149510198	+
17602	1431297_a_at	Region	4933436C20Rik	71296	8	91612610	91643238	-
17603	1430224_at	Region	Wfdc3	71856	2	164187919	164200045	-
17604	1423716_s_at	Region	Atp5d	66043	10	80265814	80269260	+
17605	1429436_at	Region	Fnbp3	56194	2	53070637	53123349	-
17606	1424429_s_at	Region	AI225782	233875	7	120902164	120911194	-
17607	1442095_at	Region	C230079D11Rik	211961	18	22565695	22746074	+
17608	1439538_at	Region	0610011N22Rik	67433	13	70412209	70421112	+
17609	1460499_at	Region	9230110I02Rik	77698	3	96758002	96759021	-
17610	1451643_a_at	Region	Rab4b	19342	7	22541255	22552404	-
17611	1418544_at	Region	Csen	56461	2	126970351	127035223	-
17612	1427065_at	Region	4933439F18Rik	66771	11	60142945	60170919	+
17613	1424337_at	Region	Snx15	69024	19	5908194	5917005	-
17614	1415981_at	Region	5031400M07Rik	80517	9	25003408	25046786	-
17615	1425804_at	Region	Hmx2	15372	7	125864584	125872325	+
17616	1421268_at	Region	Ugcg	22234	4	59132999	59166282	+
17617	1447926_at	Region	Arl5	75423	2	52330113	52357036	-
17618	1422641_at	Region	Dok5	76829	2	170240673	170388501	+
17619	1429007_at	Region	Slc35b2	73836	17	43074866	43078599	+
17620	1429041_at	Region	LOC436177	436177	8	19627469	19643172	+
17621	1423499_at	Region	Sncaip	67847	18	52984667	53125606	+
17622	1446318_at	Region	Cdw92	100434	4	53356536	53538357	+
17623	1422906_at	Region	Abcg2	26357	6	58818112	58854896	+
17624	1445727_at	Region	Ube3a	22215	7	53507062	53583196	+
17625	1458916_at	Region	Slc12a6	107723	2	111890057	111986394	+
17626	1418430_at	Region	Kif5b	16573	18	6245790	6284051	-
17627	1435189_at	Region	Frmpd1	242417	4	45119857	45201858	+
17628	1429153_at	Region	6530406A20Rik	76213	12	108431856	108433798	-
17629	1444086_at	Region	E030049G20Rik	210356	1	125748845	126327642	-
17630	1445239_at	Region	Gatad2a	234366	8	69059749	69147743	-
17631	1417621_at	Region	Nfatc1	18018	18	80729557	80836410	-
17632	1423774_a_at	Region	Prc1	233406	7	74094603	74116367	+
17633	1430662_at	Region	9430091E24Rik	434350	8	110441032	110450927	-
17634	1436762_x_at	Region	Elp3	74195	14	60057876	60120430	-
17635	1460731_at	Region	Slc35c2	228875	2	164733289	164744560	-
17636	1420518_a_at	Region	Igsf9	93842	1	172411236	172427797	+
17637	1417775_at	Region	Rpo1-4	20019	6	72241050	72311336	+
17638	1441092_at	Region	9330159M07Rik	319673		60831014	60844838	-
17639	1446539_at	Region	Inpp4b	234515	8	81240635	81241286	+
17640	1422880_at	Region	Sypl	19027	12	29560169	29583105	+
17641	1439997_at	Region	A930006D20Rik	77939	16	90166348	90168084	-
17642	1445161_at	Region	Usp6nl	98910	2	6270005	6360769	+

17643	1439089_at	Region	Zbtb41	226470	1	139269557	139296474	+
17644	1438689_at	Region	4632433K11Rik	77043	7	4282563	4285253	+
17645	1458018_at	Region	Ppm1h	319468	10	122369636	122614468	+
17646	1442626_at	Region	5730405I09Rik	67974	18	9356614	9490220	-
17647	1454937_at	Region	B630005N14Rik	101148	6	13612888	13664917	-
17648	1432462_a_at	Region	Crsp8	68975	2	29278998	29456950	+
17649	1455466_at	Region	Gpr133	243277	5	128303253	128379025	+
17650	1459823_at	Region	Ehd2	259300	7	86	1894	+
17651	1442454_at	Region	Top2a	21973	11	98814490	98845202	-
17652	1426223_at	Region	2810439F02Rik	72747	18	12831155	12924403	+
17653	1440500_at	Region	Map3k10	269881	7	23031316	23063618	-
17654	1445340_at	Region	Phr1	105689	14	97658865	97891435	-
17655	1444244_at	Region	None	None	18	21339796	21341335	-
17656	1451160_s_at	Region	Pvr	52118	7	633	12546	-
17657	1423657_at	Region	Cdipt	52858	7	121026080	121030232	+
17658	1419446_at	Region	Tbc1d1	57915	5	62958072	63146849	+
17659	1458363_at	Region	Zdhhc17	320150	10	110565885	110632856	-
17660	1428904_at	Region	E230022H04Rik	225339	18	32000024	32022887	+
17661	1423159_at	Region	Dld	13382	12	27907566	27927401	-
17662	1424489_a_at	Region	Trit1	66966	4	122043709	122082046	+
17663	1429667_at	Region	E130119J07Rik	78006	14	99005077	99007471	-
17664	1456145_at	Region	Dleu2	328425	14	56161792	56162846	-
17665	1460487_at	Region	1110014L15Rik	66127	7	5874719	5876524	-
17666	1451251_at	Region	Appbp2	66884	11	84919300	84963112	-
17667	1451037_at	Region	Ptpn9	56294	9	57108988	57176293	+
17668	1422887_a_at	Region	Ctbp2	13017	7	127353901	127489679	-
17669	1438679_at	Region	Trim8	93679	19	46050008	46064111	+
17670	1429449_at	Region	Samd4	74480	14	41964485	42183012	+
17671	1458347_s_at	Region	Tmprss2	50528	16	97049264	97095777	-
17672	1439012_a_at	Region	Dck	13178	5	88030861	88049111	+
17673	1459532_at	Region	Grm7	108073	6	112054724	112055296	+
17674	1421379_at	Region	Zfp354b	27274	11	50674664	50683826	-
17675	1430032_at	Region	4921506I22Rik	66704	19	4545410	4554730	+
17676	1422806_x_at	Region	Ing3	71777	6	21996682	22023073	+
17677	1421115_a_at	Region	Zdhhc16	74168	19	41477885	41488472	+
17678	1450881_s_at	Region	Tm7sf1	83924	13	12777506	12812482	-
17679	1428694_at	Region	5033413D16Rik	75957	14	109602514	109605268	+
17680	1433882_at	Region	Cnot10	78893	9	114550674	114606723	-
17681	1427075_s_at	Region	5330414D10Rik	245867	2	181554859	181574406	+
17682	1445545_at	Region	2610318G18Rik	67171	3	106343709	106369981	+
17683	1426431_at	Region	Jag2	16450	12	108384687	108405592	-
17684	1454931_at	Region	Cri2	386655	7	23665390	23666100	+
17685	1442264_at	Region	LOC381240	381240	19	6197625	6198089	+
17686	1417104_at	Region	Emp3	13732	7	40003815	40007155	-
17687	1437580_s_at	Region	Nek2	18005	1	191557109	191568589	+
17688	1433210_at	Region	Rptn	20129	3	93110842	93116585	+
17689	1427335_at	Region	6720456H20Rik	218989	14	43535510	43604020	+
17690	1417586_at	Region	Timeless	21853	10	127972037	127989758	+
17691	1436531_at	Region	Metap2 /// A930035J23Rik	109310	10	93824804	93833552	+
17692	1459210_at	Region	B130017P16Rik	81907	9	103460526	103476281	-
17693	1459609_at	Region	Arhgap10	78514	8	76458897	76659183	-
17694	1429212_a_at	Region	Lrrc51	69358	7	96019084	96027318	-
17695	1458633_at	Region	Snx8	231834	5	139336643	139385591	-
17696	1443652_x_at	Region	Spred1	114715	2	116635298	116693185	+
17697	1450883_a_at	Region	Cd36	12491	5	16201482	16256089	-
17698	1438541_at	Region	4933439F18Rik	66771	11	60142945	60170919	+
17699	1415792_at	Region	AL033326	24105	2	151773272	151789571	-
17700	1458897_at	Region	Ust	338362	10	8072157	8390183	-
17701	1446947_at	Region	None	None	2	135431833	135432403	+
17702	1446293_at	Region	Bcl11a	14025	11	23972907	24067984	+
17703	1419679_at	Region	Lats2	50523	14	52225857	52269062	-
17704	1425088_at	Region	Scnn1a	20276	6	125978533	126001623	+
17705	1423194_at	Region	Arhgap5	11855	12	49267090	49318371	+
17706	1415886_at	Region	Sh2d3c	27387	2	32653234	32687156	+

17707	1430982_at	Region	Sfrs1	110809	11	87775771	87779641	+
17708	1418621_at	Region	Rab2	59021	4	8462790	8534848	+
17709	1440986_at	Region	Rpap1	68925	2	119277813	119301347	-
17710	1435928_at	Region	None	None	8	119519010	119520814	+
17711	1452120_at	Region	4931433E08Rik	70999	19	6941795	6955246	-
17712	1448836_s_at	Region	AI838661	100210	4	132545279	132552633	+
17713	1417064_at	Region	Jagn1	67767	6	114010464	114016057	+
17714	1459733_at	Region	None	None	1	172130713	172131101	-
17715	1422397_a_at	Region	Il15ra	16169	2	11621545	11649971	+
17716	1417911_at	Region	Ccna2	12428	3	36028468	36035544	-
17717	1436429_at	Region	Zfp606	67370	7	10522586	10539938	+
17718	1454384_at	Region	2900057C01Rik	72987	8	106272520	106273640	+
17719	1442212_at	Region	Cpd	12874	11	76507648	76572655	-
17720	1448981_x_at	Region	Tcl1b1	27379	12	100603840	100610761	+
17721	1426096_at	Region	BC006705	214572	8	105506901	105547541	+
17722	1447846_x_at	Region	5730589L02Rik	77582	7	1165	3428	-
17723	1426618_a_at	Region	Pomgnt1	68273	4	115109430	115118749	+
17724	1455535_at	Region	A730017D01Rik	319649	6	144674668	144675623	-
17725	1426515_a_at	Region	Tor1a	30931	2	30892789	30900053	-
17726	1447146_s_at	Region	D11Ertd730e	193116	11	43186568	43200818	+
17727	1423562_at	Region	ORF31	260297	17	32332934	32335508	+
17728	1439367_x_at	Region	Arf4	11843	14	24734402	24753463	+
17729	1440259_at	Region	Man1b	17156	3	99992328	100111138	-
17730	1454929_s_at	Region	Safb	224903	17	54273431	54294731	+
17731	1456736_x_at	Region	5230400G24Rik	75734	1	83054024	83081496	+
17732	1434322_at	Region	A930021H16Rik	231830	5	138703020	138729158	-
17733	1425347_a_at	Region	Zfp318	57908	17	43894944	43929210	+
17734	1421740_at	Region	Gnas	14683	2	173709228	173771651	+
17735	1418800_at	Region	Bhlhb8	17341	5	143231899	143236054	+
17736	1459963_at	Region	None	None	X	88224301	88224647	-
17737	1445934_at	Region	Arih2 /// LOC432565	23807	9	108651717	108698223	-
17738	1421892_at	Region	None	None	8	110254745	110270197	+
17739	1451765_a_at	Region	Entpd5	12499	12	81245023	81273816	-
17740	1454661_at	Region	Atp5g3	228033	2	73606199	73609043	-
17741	1422423_at	Region	Magea7	17143	X	71008049	71008291	+
17742	1450873_at	Region	Gtpbp4	69237	13	8956055	8979603	-
17743	1425313_at	Region	Carf	241066	1	60401596	60454354	+
17744	1441321_at	Region	LOC552911	552911	3	51633932	51635160	-
17745	1453726_s_at	Region	2810407C02Rik	69227	3	58220979	58236191	+
17746	1433442_at	Region	Klhl9	242521	4	87705774	87708653	-
17747	1417055_at	Region	0610009D07Rik	66055	12	3985835	3999611	+
17748	1424297_at	Region	Zfp282	101095	6	48002230	48031647	+
17749	1453300_at	Region	Slc35d2	70484	13	61482262	61514080	-
17750	1426138_a_at	Region	Ube2j2	140499	4	154436212	154451966	+
17751	1439726_at	Region	4432406C05Rik	66690	16	8307024	8310994	-
17752	1453747_at	Region	2810021J22Rik	69944	11	58592869	58609967	+
17753	1437689_x_at	Region	Clu	12759	14	60496045	60508968	+
17754	1442806_at	Region	9430030N17Rik	77336	19	44574409	44575550	+
17755	1444704_at	Region	D5Ertd606e	52398	5	92429207	92510708	+
17756	1442171_at	Region	Fnbp3	56194	2	53070637	53123349	-
17757	1429670_a_at	Region	Lrriq2	74201	16	54796937	54831308	-
17758	1433258_at	Region	A330102K18Rik	77919	6	96497563	96498566	-
17759	1438045_at	Region	Eea1	216238	10	95914281	96016681	+
17760	1451035_a_at	Region	Akr1a4	58810	4	115595415	115610556	-
17761	1418120_at	Region	Rbm8a	60365	3	96117769	96120746	+
17762	1448171_at	Region	Siah2	20439	3	58325606	58343045	-
17763	1434542_at	Region	Gpt2	108682	8	84765391	84800332	+
17764	1425621_at	Region	Trim35	66854	14	60824516	60836924	+
17765	1435080_x_at	Region	5730406M06Rik	66625	4	21916954	21945846	+
17766	1456846_at	Region	LOC382639	382639	12	108158656	108162134	+
17767	1454945_at	Region	Lrriq2	74201	16	54796937	54831308	-
17768	1451207_at	Region	Cbara1	216001	10	59664687	59819557	+
17769	1425595_at	Region	Gabbr1	54393	17	34805575	34833793	+
17770	1454551_at	Region	9530034D02Rik	78417	6	31407610	31408715	+

17771	1416433_at	Region	Rpa2	19891	4	131729260	131739382	+
17772	1442093_at	Region	None	None	4	127958354	127959178	-
17773	1415733_a_at	Region	1110019J04Rik	68512	4	45021131	45024027	-
17774	1423964_at	Region	Cpsf3l	71957	4	154361929	154381465	+
17775	1449234_at	Region	Car15	80733	16	16606239	16609149	-
17776	1452099_at	Region	AA408296	215193	1	192843167	192869015	-
17777	1457350_at	Region	Per2	18627	1	91239024	91282480	-
17778	1442184_at	Region	Slc24a3	94249	2	144905442	145098889	+
17779	1458420_at	Region	9430057O19Rik	231093	5	29347796	29355631	+
17780	1451085_at	Region	C030006K11Rik	223665	15	76772844	76775219	-
17781	1446455_at	Region	None	None	NONE	NONE	NONE	NONE
17782	1448019_at	Region	2900006A08Rik	72857	5	116403447	116403882	+
17783	1456260_at	Region	Rbbp4	19646	4	128334213	128362483	-
17784	1455906_at	Region	6030446N20Rik	338363	18	12168904	12309081	-
17785	1415940_at	Region	AA407930	100494	5	138468708	138481909	-
17786	1445212_at	Region	C330023M02Rik	231713	5	120549789	120591920	+
17787	1422603_at	Region	Rnase4	58809	14	46185837	46200781	+
17788	1449477_s_at	Region	Slc2a10	170441	2	164960708	164976644	+
17789	1429584_at	Region	Mynn	80732	3	30012186	30028739	+
17790	1457131_at	Region	9330186A19Rik	320365	5	149134722	149437633	+
17791	1439870_at	Region	A330008L17Rik	234624	8	98707406	98715396	+
17792	1415682_at	Region	Xpo7	65246	14	64979347	65026150	-
17793	1445867_at	Region	AL023008	101715	7	91651148	91651930	+
17794	1439126_at	Region	1110007A13Rik	210711	7	122745901	122814730	-
17795	1424895_at	Region	Gpsm2	76123	3	108474380	108517885	-
17796	1428954_at	Region	Slc9a3r2	65962	17	22442873	22453949	-
17797	1425224_at	Region	BC019561	225655	18	67695686	67710555	+
17798	1418729_at	Region	Star	20845	8	24533262	24540731	+
17799	1429363_at	Region	D8Ertd531e	52348	8	39455470	39493394	+
17800	1446641_at	Region	Syt7	54525	19	9585980	9644941	+
17801	1452143_at	Region	Spnb2	20742	11	29995224	30114564	-
17802	1438610_a_at	Region	Cryz	12972	3	153604204	153630187	+
17803	1458006_at	Region	Cpm	70574	10	117346301	117376298	+
17804	1424781_at	Region	D10Ucla1	28193	10	67048137	67134030	-
17805	1423755_at	Region	Zcchc8	70650	5	122870982	122892129	-
17806	1456653_a_at	Region	Mthfd1l	270685	10	6349713	6535179	-
17807	1442164_at	Region	Blzf1	66352	1	164208297	164225981	-
17808	1424966_at	Region	Tmem40	94346	6	116166666	116179922	-
17809	1452087_at	Region	Epsti1	108670	14	72246823	72345085	+
17810	1442427_at	Region	9630026M06Rik	320524	7	17493226	17493795	-
17811	1453446_at	Region	6430411K18Rik	76880	12	105068540	105069857	+
17812	1439868_at	Region	Sidt2	214597	9	45951127	45968501	-
17813	1454109_a_at	Region	Ptdsr	107817	11	116658525	116664468	-
17814	1430386_at	Region	9030417F11Rik	74537	19	15392526	15394428	+
17815	1459516_at	Region	Cul1	26965	6	47656010	47727829	+
17816	1446086_s_at	Region	Gli2	14633	1	118586114	118804994	-
17817	1457059_at	Region	B830013J05Rik	240186	18	5214775	5339178	-
17818	1455166_at	Region	None	None	2	15007173	15008729	+
17819	1437220_x_at	Region	Psmd13	23997	7	135284278	135300400	+
17820	1434496_at	Region	Plk3	12795	4	116087560	116092849	-
17821	1424819_a_at	Region	AI114950	101489	7	135259333	135265613	+
17822	1457510_at	Region	None	None	NONE	NONE	NONE	NONE
17823	1425506_at	Region	Mylk	107589	16	33677883	33783686	+
17824	1437149_at	Region	Slc6a6	21366	6	92133220	92208188	+
17825	1432287_a_at	Region	Sntg1	71096	1	8450893	9404007	-
17826	1458566_at	Region	Gpatc2	67769	1	186723158	186859064	+
17827	1451522_s_at	Region	Lrch4	231798	5	136579783	136591102	+
17828	1458015_at	Region	2410080H04Rik	214058	9	64507881	64823155	+
17829	1440961_at	Region	9130604C24Rik	77707	5	136453502	136454495	+
17830	1448484_at	Region	Amd1	11702	10	40363437	40378151	-
17831	1447895_x_at	Region	2700050F09Rik	72587	5	146439784	146439986	+
17832	1425629_a_at	Region	Nol6	230082	4	41253278	41263139	-
17833	1416358_at	Region	0610009O03Rik	68294	5	33121321	33124788	-
17834	1449534_at	Region	Sycp3	20962	10	88431421	88445069	+
17835	1443276_at	Region	None	None	5	26294686	26295340	+

17836	1448472_at	Region	Vars2	22321	17	32704913	32720106	+
17837	1418858_at	Region	Aox3	71724	1	58417941	58505250	+
17838	1417513_at	Region	Evi5	14020	5	106815244	106945556	-
17839	1415783_at	Region	Vps35	65114	8	84533166	84572272	-
17840	1438257_at	Region	Zfp313	81018	2	166949378	166972899	+
17841	1429974_at	Region	Tbx18	76365	9	88049059	88076120	-
17842	1426931_s_at	Region	D19Bwg1357e	52874	19	26629949	26669888	-
17843	1438788_at	Region	D5Wsu152e	28022	5	89612092	89612660	-
17844	1418888_a_at	Region	Sepx1	27361	17	22541758	22547868	+
17845	1423224_at	Region	4432405B04Rik	67978	5	123770127	123798992	+
17846	1436286_at	Region	B830017H08Rik	433004	16	16604082	16605982	+
17847	1454804_at	Region	AK129375	217365	11	120201464	120258761	-
17848	1453427_at	Region	Csnk2a1	12995	2	151683883	151738783	+
17849	1454692_x_at	Region	Hnrpk	15387	13	57024107	57034672	-
17850	1443455_at	Region	None	None	NONE	NONE	NONE	NONE
17851	1425350_a_at	Region	Myef2	17876	2	124602727	124630728	-
17852	1436008_at	Region	None	None	3	8915557	8916202	-
17853	1446372_at	Region	None	None	5	114258946	114259303	-
17854	1439547_at	Region	A930007A09Rik /// LOC436338	432999	16	12491310	12495281	+
17855	1434577_at	Region	BC052040	399568	2	115095624	115292616	+
17856	1435882_at	Region	Ubap2l	74383	3	89810481	89862883	-
17857	1452235_at	Region	Man1b1	227619	2	25264929	25284370	+
17858	1420486_at	Region	Nol7	70078	13	42960833	42965301	+
17859	1416231_at	Region	Vac14	234729	8	109916360	110018120	+
17860	1424584_a_at	Region	Ranbp10	74334	8	105064168	105123194	-
17861	1437242_at	Region	BC055368	223723	15	83624409	83643711	-
17862	1457850_at	Region	None	None	3	30727288	30728156	+
17863	1424001_at	Region	Mki67ip	67949	1	118071365	118083106	+
17864	1427282_a_at	Region	Fxn	14297	19	23500411	23519551	-
17865	1427016_at	Region	4932438A13Rik	229227	3	36326717	36516613	+
17866	1439434_x_at	Region	BC036961	230863	4	137131739	137142223	+
17867	1457073_at	Region	None	None	NONE	NONE	NONE	NONE
17868	1437761_at	Region	Luc7l2	192196	6	38687728	38745622	+
17869	1420966_at	Region	Slc25a15	18408	8	21128178	21150724	-
17870	1424900_at	Region	Slc29a4	243328	5	141710941	141734430	+
17871	1438911_at	Region	38602	235072	9	25147069	25202879	+
17872	1436870_s_at	Region	AU041783	226250	19	56497989	56593808	-
17873	1421683_at	Region	Tcte3	21647	17	13011845	13026205	-
17874	1451376_at	Region	5730596K20Rik	109168	19	7208422	7249028	+
17875	1459150_at	Region	Lrch1	380916	14	69108806	69291857	-
17876	1424653_at	Region	Tspan15	70423	10	62153969	62197769	-
17877	1455060_at	Region	MGI:1351465	27041	11	55238464	55253728	+
17878	1421947_at	Region	Gng12	14701	6	67148787	67270629	+
17879	1429376_s_at	Region	Anapc10	68999	8	78925931	78991431	+
17880	1429174_at	Region	Wdr34	71820	2	29963713	29980992	-
17881	1452354_at	Region	2810459M11Rik	72792	1	85852319	85861811	+
17882	1418844_at	Region	Dibd1	102580	9	50847656	50915854	+
17883	1446722_at	Region	Gna13	14674	11	109184029	109219306	+
17884	1458222_at	Region	None	None	5	149601867	149602390	-
17885	1435804_at	Region	Eif4e2	26987	1	87030076	87054566	+
17886	1431377_at	Region	Sca10	54138	15	85384867	85511564	+
17887	1434175_s_at	Region	2210010N04Rik	70381	5	143236935	143265219	-
17888	1418907_at	Region	F5	14067	1	164069551	164140290	+
17889	1434536_at	Region	Gpr75	237716	11	30785887	30787509	+
17890	1447122_at	Region	4930438O05Rik	78795	1	85961103	86084595	+
17891	1431455_at	Region	4933401B01Rik	71027	16	56176496	56201633	-
17892	1453512_at	Region	5830407P18Rik	78818	14	114968680	114970273	+
17893	1442223_at	Region	Enah	13800	1	181864370	181978518	-
17894	1442126_at	Region	5830417C01Rik	78825	1	178140297	178205198	+
17895	1455608_at	Region	2610207F23Rik	67161	3	41056512	41247364	-
17896	1441753_at	Region	4921505C17Rik	78757	15	6496946	6588954	+
17897	1429863_at	Region	A830039N02Rik	102994	X	30948963	30982898	+
17898	1459250_at	Region	MGI:2153084	228911	2	169579870	169580552	+
17899	1438887_a_at	Region	Gcl	23885	6	87126075	87167665	-

17900	1416486_at	Region	Scye1	13722	3	131550899	131574226	-
17901	1446007_at	Region	Bmp1	12153	14	64790547	64836177	-
17902	1433859_at	Region	D030070L09Rik	225280	18	24333257	24350315	-
17903	1455290_at	Region	Znrf2	387524	6	54961241	55034061	+
17904	1437300_at	Region	Mef2d	17261	3	87886291	87912587	+
17905	1457349_at	Region	Gm69	217602	12	54442560	54518225	+
17906	1457508_at	Region	C430003N24Rik	320358	10	108071515	108072185	+
17907	1436909_at	Region	B430110G05Rik	229517	3	88154362	88168982	-
17908	1454168_a_at	Region	Skd3	20480	7	95770056	95896228	+
17909	1435562_at	Region	Pdzk8	107368	19	58895293	58896325	-
17910	1439289_s_at	Region	0710005I19Rik	71691	7	13910706	13913231	+
17911	1452254_at	Region	Mtmr9	210376	14	58054197	58074540	-
17912	1435810_at	Region	5730455O13Rik	70567	19	37532284	37607394	-
17913	1439178_at	Region	Adrbk2	320129	5	111987619	112085665	-
17914	1458520_at	Region	Hipk2	15258	6	38834084	39012133	-
17915	1436473_at	Region	Zfp248	72720	6	118859906	118888093	-
17916	1454238_a_at	Region	1700010H22Rik	75500	5	97573258	97586555	-
17917	1446886_at	Region	Usp3	235441	9	66641895	66716994	-
17918	1434578_x_at	Region	Ran	19384	5	128195181	128199286	+
17919	1418256_at	Region	Srf	20807	17	44056896	44066219	-
17920	1453366_at	Region	Tdrkh	72634	3	93901088	93920457	+
17921	1437260_at	Region	Mmrn1	70945	6	61184420	61229308	+
17922	1450896_at	Region	Arhgap5	11855	12	49267090	49318371	+
17923	1455581_x_at	Region	9530028C05	330256	6	3285716	3298593	-
17924	1443155_at	Region	None	None	5	4872941	4873474	-
17925	1437124_at	Region	A630052C17Rik	320757	11	29066121	29068348	-
17926	1451764_at	Region	Marveld3	73608	8	109245590	109259848	-
17927	1452612_at	Region	Zfp294	78913	16	86525259	86582064	-
17928	1425193_at	Region	2010106G01Rik	66552	2	126407985	126447088	-
17929	1435670_at	Region	Tcfap2b	21419	1	19426696	19452210	+
17930	1435995_at	Region	Mrpl22	216767	11	57897290	57905194	+
17931	1418826_at	Region	Ms4a6b	69774	19	10715058	10726902	+
17932	1428962_at	Region	1700013F07Rik	75504	3	108333288	108340461	+
17933	1442332_at	Region	Tgfbr3	21814	5	106170128	106359608	-
17934	1416299_at	Region	Shcbp1	20419	8	4097851	4141461	-
17935	1443830_x_at	Region	Rnf103	22644	6	71825871	71842858	+
17936	1437428_x_at	Region	Eif2b2	217715	12	82088696	82095811	+
17937	1433783_at	Region	Ldb3	24131	14	32665465	32724864	-
17938	1439096_at	Region	5330420D20Rik	70503	10	40708813	40729005	+
17939	1458229_at	Region	Robo2	268902	16	73001837	73492850	-
17940	1429622_at	Region	Cand2	67088	6	116212110	116242404	+
17941	1417099_at	Region	Ftsj1	54632	X	6477608	6491346	-
17942	1421426_at	Region	Hhip	15245	8	79185726	79272113	-
17943	1435304_at	Region	Sod1	20655	16	89378174	89383756	+
17944	1448654_at	Region	Mtch2	56428	2	90551904	90571383	+
17945	1457117_at	Region	Nfe2l2	18024	2	75373265	75402331	-
17946	1452638_s_at	Region	Dnm1l	74006	16	15081722	15126600	-
17947	1429441_at	Region	Fbxo30	71865	10	11158930	11174522	+
17948	1448514_at	Region	Cox5b	12859	1	36986129	36987976	+
17949	1446164_at	Region	Ncoa6ip	116940	4	3502044	3543770	+
17950	1441913_at	Region	Luzp2	233271	7	49005909	49477620	+
17951	1442652_at	Region	---	242037	3	57285949	57326058	-
17952	1425624_at	Region	Epm2aip1	77781	9	111313226	111320386	+
17953	1453529_at	Region	6330418B08Rik	70745	1	174994661	174997616	-
17954	1423869_s_at	Region	Txnrd3	232223	6	90079638	90111154	+
17955	1418985_at	Region	BC003236	80281	3	104796431	104845993	-
17956	1441241_at	Region	9630013D21Rik	319743	4	108611339	108616732	-
17957	1438502_x_at	Region	Rps17 /// LOC383032	20068	7	75150291	75152774	-
17958	1456060_at	Region	Maf	17132	8	115016686	115019625	-
17959	1452219_at	Region	BC026370	224807	17	43170690	43196864	-
17960	1449821_a_at	Region	0610016J10Rik	76890	11	72006473	72064344	-
17961	1460177_at	Region	Cndp2	66054	18	84835201	84853374	-
17962	1416356_at	Region	Gmpr2	105446	14	50190251	50197215	+
17963	1415941_s_at	Region	AA407930	100494	5	138468708	138481909	-

17964	1425801_x_at	Region	Cotl1	72042	8	119165392	119197206	-
17965	1457230_at	Region	Gnpda2	67980	5	68341941	68359622	-
17966	1442760_x_at	Region	Rtn4	68585	11	29587737	29637706	+
17967	1454877_at	Region	Sertad4	214791	1	192583252	192594485	-
17968	1421298_a_at	Region	Hipk1	15257	3	103171062	103218570	-
17969	1418235_at	Region	Atg5l	11793	10	44383463	44478754	+
17970	1434180_at	Region	Plekhc1	218952	14	40537053	40545664	-
17971	1436391_s_at	Region	MGI:2385186	229725	3	108449989	108473754	+
17972	1456874_at	Region	Flrt2	399558	12	91102888	91191660	+
17973	1436730_at	Region	MGI:2385186	229725	3	108449989	108473754	+
17974	1418330_at	Region	Ctcf	13018	8	104932169	104978643	+
17975	1426381_at	Region	Pprc1	226169	19	45603729	45620082	+
17976	1428659_at	Region	Phf7	71838	14	29369534	29383056	-
17977	1452904_at	Region	1700026L06Rik	69987	2	28624242	28631813	-
17978	1423502_at	Region	Brd2	14312	17	31817639	31824919	-
17979	1450183_a_at	Region	Lnk	16923	5	120969565	120989151	-
17980	1446006_at	Region	1500034J20Rik	66541	2	105609439	105671940	+
17981	1418023_at	Region	Narg1	74838	3	51048093	51105995	+
17982	1423486_at	Region	1200020A08Rik	56724	17	84882400	84892614	+
17983	1460519_a_at	Region	Mettl5	75422	2	69733938	69741002	-
17984	1425166_at	Region	Rbl1	19650	2	156602867	156661488	-
17985	1449507_a_at	Region	Cd47	16423	16	48697237	48753271	+
17986	1460063_at	Region	D5Ertd798e	52409	5	44528855	44530831	-
17987	1453541_at	Region	Pfkl	18641	10	78096466	78119117	-
17988	1452323_at	Region	BC008150	223918	15	102175888	102196929	-
17989	1455809_x_at	Region	AI114950	101489	7	135259333	135265613	+
17990	1439734_at	Region	Mmp15	17388	8	94636817	94655679	+
17991	1419402_at	Region	Mns1	17427	9	72562062	72591224	+
17992	1428546_at	Region	Syncrip	56403	9	88814754	88842894	-
17993	1456317_at	Region	1700055N04Rik	73458	19	3759421	3769982	+
17994	1448994_at	Region	Sp1	20683	15	102466747	102493098	+
17995	1459783_s_at	Region	Cno	117197	5	35253726	35255215	-
17996	1428951_at	Region	Nol8	70930	13	49249941	49275842	+
17997	1460736_at	Region	AF013969	100563	5	40543493	40600266	-
17998	1450647_at	Region	Hps3	12807	3	19337225	19376554	-
17999	1439425_x_at	Region	BC024814	239706	16	8144106	8163490	+
18000	1420132_s_at	Region	Pttg1ip	108705	10	77693207	77710168	+
18001	1431028_a_at	Region	Pank1	75735	19	34154273	34221296	-
18002	1451436_at	Region	Sbno1	243272	5	123544504	123597297	-
18003	1428564_at	Region	Zfp579	68490	7	4239821	4241149	-
18004	1448634_at	Region	Ralbp1	19765	17	63557880	63594264	-
18005	1449915_at	Region	Zfp202	80902	9	40142614	40163478	+
18006	1424574_at	Region	Tmed5	73130	5	107193657	107204601	-
18007	1452434_s_at	Region	Dgcr6	13353	16	16823817	16842299	+
18008	1423461_a_at	Region	Ubl3	24109	5	147397249	147446449	-
18009	1419182_at	Region	Svep1	64817	4	57986245	58150045	-
18010	1439205_at	Region	AI607462	99036	2	167985145	167985705	-
18011	1455362_at	Region	D1Ertd396e	52477	1	190665121	190682759	+
18012	1453461_at	Region	Fxc1	14356	7	99755452	99756758	+
18013	1447187_at	Region	Ripk5	213452	1	132274936	132320783	+
18014	1425921_a_at	Region	1810055G02Rik	72056	19	3497123	3506671	+
18015	1435158_at	Region	3000004N20Rik	72397	4	12067282	12073696	+
18016	1437329_at	Region	Ptplb	70757	16	33805502	33889652	+
18017	1432372_a_at	Region	Spr	20751	6	85483272	85487405	-
18018	1415734_at	Region	Rab7	19349	6	88435785	88481888	-
18019	1445225_at	Region	5730427M17Rik	98814	2	171960155	171960750	+
18020	1431809_at	Region	4932442L08Rik	74432	X	89015709	89027435	-
18021	1420489_at	Region	Mrps14	64659	1	160099907	160105833	+
18022	1424509_at	Region	1190003K14Rik	68891	7	19898555	19937469	-
18023	1449065_at	Region	Cte1	26897	12	80873838	80882079	+
18024	1458043_at	Region	Btbd9	224671	17	28030141	28340405	-
18025	1438591_at	Region	2610019A05Rik	72149	11	105984425	106014637	-
18026	1440343_at	Region	Rps6ka5	73086	12	95995143	96170417	-
18027	1456986_at	Region	Zbtb16	235320	9	48684510	48863820	-
18028	1417197_at	Region	D8Ertd594e	52357	8	46797192	46960652	-

18029	1442027_at	Region	LOC545323	545323	1	60484054	60635087	+
18030	1419190_at	Region	Vti1a	53611	19	54896591	55207543	+
18031	1424430_at	Region	Mterfd2	69821	1	93126702	93133361	-
18032	1459149_at	Region	None	None	9	22136646	22137072	+
18033	1426870_at	Region	Fbxo33	70611	12	55933702	55953036	-
18034	1417966_at	Region	Mrpl39	27393	16	83827402	83845155	-
18035	1446474_at	Region	---	433759	4	128543217	128569759	-
18036	1460450_at	Region	E230022H04Rik	225339	18	32000024	32022887	+
18037	1449895_at	Region	Acr	11434	15	89621235	89627301	+
18038	1419466_at	Region	Nkd2	72293	13	69880109	69907684	-
18039	1436005_at	Region	Sfrs14	234373	8	69387208	69416080	+
18040	1424243_at	Region	BC016198	192174	8	46500251	46519367	+
18041	1426847_at	Region	Sirt4	75387	5	114591018	114605341	-
18042	1456586_x_at	Region	Mvp	78388	7	121036597	121064325	-
18043	1429727_at	Region	Slc16a9	66859	10	70299424	70340099	+
18044	1416922_a_at	Region	Bnip3l	12177	14	61515922	61539638	-
18045	1427156_s_at	Region	Ascc2	75452	11	4532580	4578172	+
18046	1431929_a_at	Region	Stx17	67727	4	48040852	48099039	+
18047	1449894_at	Region	4930442L21Rik	67580	14	31128052	31151879	+
18048	1436427_at	Region	Prpf4b	19134	13	34407672	34435208	+
18049	1416347_at	Region	Men1	17283	19	6123829	6129679	+
18050	1460324_at	Region	Dnmt3a	13435	12	3001834	3066656	+
18051	1433590_at	Region	Herc3	73998	6	58996163	59083005	+
18052	1458877_at	Region	None	None	3	80404755	80405419	-
18053	1419765_at	Region	Cul2	71745	18	3387735	3440960	+
18054	1449779_at	Region	None	None	NONE	NONE	NONE	NONE
18055	1452710_at	Region	Rpusd4	71989	9	35181408	35189406	+
18056	1449641_at	Region	None	None	14	19682793	19691460	+
18057	1420772_a_at	Region	Tsc22d3	14605	X	134084467	134088106	-
18058	1441131_at	Region	None	None	5	146390002	146390634	+
18059	1420930_s_at	Region	Ctnnal1	54366	4	56754384	56808637	-
18060	1428855_at	Region	H13	14950	2	152126435	152163760	+
18061	1436906_at	Region	Rnf166	68718	8	121847734	121858060	-
18062	1427018_at	Region	Tsnaxip1	72236	8	105123588	105140520	+
18063	1455901_at	Region	Chpt1	212862	10	88445367	88476119	-
18064	1425961_at	Region	BC016548	211039	2	103088733	103093796	-
18065	1419271_at	Region	Pax6	18508	2	105381199	105401823	+
18066	1442221_at	Region	Gnefr	27414	7	40528521	40725138	-
18067	1437737_at	Region	AV340375	213550	9	64429200	64463381	-
18068	1418569_at	Region	Fblim1	74202	4	140458190	140477211	-
18069	1441869_x_at	Region	Auts2	319974	5	130627794	130728707	-
18070	1445877_at	Region	None	None	13	72058644	72059366	-
18071	1420796_at	Region	Ahrr	11624	13	70273194	70354514	-
18072	1431334_a_at	Region	4933433P14Rik	66787	12	101145518	101150041	+
18073	1458747_at	Region	Fbxo45	268882	16	31039483	31056396	-
18074	1452694_at	Region	Ihpk1	27399	9	108070635	108116769	+
18075	1459321_at	Region	None	None	1	130319002	130319663	+
18076	1425465_a_at	Region	Senp2	75826	16	20782192	20820660	+
18077	1433041_at	Region	Rabepk	227746	2	34710828	34731953	-
18078	1429930_at	Region	Ccdc19	71870	1	172450058	172474789	+
18079	1425713_a_at	Region	Rnf146	68031	10	29378124	29394428	-
18080	1443819_x_at	Region	Ttc11	66437	5	135975208	135979354	+
18081	1420804_s_at	Region	Clec4d	17474	6	123929332	123942075	+
18082	1422912_at	Region	Bmp4	12159	14	41463739	41467068	-
18083	1437756_at	Region	Gimap9	317758	6	48809017	48811587	+
18084	1422461_at	Region	Atad3a	108888	4	154233003	154253455	-
18085	1453322_at	Region	Wdr33	74320	18	32067429	32147139	+
18086	1424804_at	Region	BC020002	252875	6	8183603	8211482	+
18087	1453358_s_at	Region	5830467E07Rik /// C730024G19Rik	232566	6	150093461	150124565	-
18088	1458548_at	Region	None	None	4	3801782	3802329	-
18089	1434977_at	Region	4933403F05Rik	108654	18	68497464	68531006	-
18090	1426840_at	Region	Ythdf3	229096	3	15522868	15556473	+
18091	1442643_at	Region	Jmjd3	216850	11	69128612	69129226	-
18092	1423953_at	Region	Cdkal1	68916	13	28804928	29335210	-

18093	1446321_at	Region	B230208B08Rik	319499	4	77275097	77275770	+
18094	1423299_at	Region	Txnl1	53382	18	63894960	63924518	-
18095	1422012_at	Region	Crhr2	12922	6	55234412	55277297	-
18096	1435001_at	Region	Plaa	18786	4	93544242	93578250	-
18097	1421854_at	Region	Fgl2	14190	5	19824807	19830476	+
18098	1454290_at	Region	4930573O21Rik	114670	9	3072735	3074305	+
18099	1426846_at	Region	G630055P03Rik	320394	8	105140522	105147852	-
18100	1448795_a_at	Region	Tbrg4	21379	11	6511211	6520811	-
18101	1456112_at	Region	Tpr	108989	1	150229615	150286691	+
18102	1436550_at	Region	Fbxo30	71865	10	11158930	11174522	+
18103	1451136_a_at	Region	Eif2b2	217715	12	82088696	82095811	+
18104	1437543_at	Region	Fubp1	51886	3	151191900	151214173	+
18105	1418095_at	Region	Smpx	66106	X	151298523	151351994	+
18106	1425319_s_at	Region	6530403A03Rik	67797	13	37743845	37765948	+
18107	1424267_at	Region	1810043G02Rik	67884	10	78087790	78093995	+
18108	1434989_at	Region	A030001O10Rik	503691	1	66990410	66991529	+
18109	1447931_at	Region	Whsc1l1	234135	8	24322608	24439465	+
18110	1424998_at	Region	Emr4	52614	17	53388173	53491657	+
18111	1450510_a_at	Region	None	None	8	83678710	83904625	+
18112	1440082_at	Region	Ptk2	14083	15	73233486	73423105	-
18113	1438065_at	Region	BC021395	225283	18	24716439	24758615	-
18114	1417767_at	Region	1810044O22Rik	66427	8	106448348	106485157	+
18115	1429213_at	Region	2310030N02Rik	76947	4	10978015	11025490	-
18116	1446590_at	Region	None	None	7	101351774	101352419	+
18117	1453863_at	Region	3300002P09Rik	70246	10	80531466	80532223	+
18118	1423632_at	Region	Gpr146	80290	5	138378137	138391571	+
18119	1433937_at	Region	Trp53bp2	209456	1	182387965	182421393	+
18120	1419004_s_at	Region	Bcl2a1a /// Bcl2a1b /// Bcl2a1d	12044	9	89087713	89096373	+
18121	1458640_at	Region	None	None	13	74232685	74233184	+
18122	1438058_s_at	Region	Ptov1	84113	7	38938217	38944997	-
18123	1453776_at	Region	5730407K14Rik	101113	2	164242750	164244365	+
18124	1418680_at	Region	Serpind1	15160	16	16102383	16114540	+
18125	1428037_at	Region	Otoa	246190	7	114959005	115038655	+
18126	1428235_at	Region	Sdhd	66925	9	50668672	50676143	-
18127	1455765_a_at	Region	Abcc8	20927	7	40189876	40191508	-
18128	1456274_at	Region	C230071H18Rik	399569	2	92895734	92898091	+
18129	1435440_at	Region	Pdzk8	107368	19	58896247	58899785	-
18130	1417980_a_at	Region	Insig2	72999	1	121067561	121095788	-
18131	1459910_at	Region	Tnks	21951	8	33630639	33689219	-
18132	1445861_at	Region	Usp25	30940	16	76100019	76202730	+
18133	1456303_at	Region	None	None	6	12005918	12006184	+
18134	1450484_a_at	Region	Tyki	22169	12	23014305	23024800	+
18135	1440277_at	Region	AU041474	99229	2	181250091	181250809	+
18136	1445108_at	Region	Lphn3	319387	5	80275281	81049432	+
18137	1451604_a_at	Region	Acvrl1	11482	15	101186060	101201176	+
18138	1424571_at	Region	Ddx46	212880	13	54268652	54296831	+
18139	1422938_at	Region	Bcl2	12043	1	106479000	106655083	-
18140	1428360_x_at	Region	Ndufa7	66416	17	31523009	31536811	+
18141	1432852_at	Region	Phactr1	218194	13	42242828	42700966	+
18142	1454842_a_at	Region	B3galnt2	97884	13	13397005	13441487	+
18143	1450509_at	Region	Chst11	58250	10	82872541	83080006	+
18144	1458028_at	Region	None	None	11	78571764	78572530	+
18145	1437715_x_at	Region	Apex1	11792	14	46019661	46021778	+
18146	1445200_at	Region	None	None	14	115621774	115622195	+
18147	1452466_a_at	Region	Rbm6	19654	9	107841550	107940685	-
18148	1439813_at	Region	Sppl3	74585	5	114199863	114200682	+
18149	1438890_at	Region	Fbxl11	225876	19	4106596	4187056	-
18150	1450580_at	Region	Gpr45	93690	1	43247681	43330167	+
18151	1420774_a_at	Region	4930583H14Rik	67749	3	51020380	51028473	-
18152	1441306_at	Region	6820408C15Rik	228778	2	151872523	151901264	+
18153	1457745_at	Region	Gpr4	319197	7	16090199	16092985	+
18154	1426316_at	Region	6330416G13Rik	230279	4	62651547	62677546	+
18155	1448619_at	Region	Dhcr7	13360	7	138237063	138262306	+

18156	1440404_at	Region	None	None	17	6066727	6067437	+
18157	1444107_at	Region	C130039O16Rik	238317	12	81016718	81058346	-
18158	1418347_at	Region	DXImx40e	54638	X	5832751	5844332	-
18159	1439350_s_at	Region	Cdc91l1	228812	2	154735189	154814361	-
18160	1436347_a_at	Region	5530601H04Rik	71445	X	99638560	99671489	-
18161	1437208_at	Region	38605	103080	10	59120943	59121745	-
18162	1441444_at	Region	None	None	1	60532662	60533359	+
18163	1429728_at	Region	4930429M06Rik	252876	1	97550286	97573055	+
18164	1442570_at	Region	Zmym1	68310	4	126074207	126088245	-
18165	1426908_at	Region	Galnt7	108150	8	56563802	56692236	-
18166	1452809_at	Region	9030607L17Rik	71564	10	80834056	80837087	+
18167	1443535_at	Region	Gpr21	338346	2	37448788	37451445	+
18168	1418222_at	Region	2610024G14Rik	56412	5	76538496	76554303	-
18169	1455598_at	Region	Usp30	100756	5	113210959	113234147	+
18170	1418863_at	Region	Gata4	14463	14	57729513	57775851	-
18171	1443598_at	Region	None	None	NONE	NONE	NONE	NONE
18172	1449371_at	Region	Harsl	70791	18	37007045	37016327	+
18173	1430902_at	Region	Ankrd24	70615	10	81777736	81784518	+
18174	1452855_at	Region	Ly6k	76486	15	74828518	74831524	-
18175	1449144_at	Region	Gna11	14672	10	81666416	81680827	-
18176	1449180_at	Region	Kcmf1	74287	6	73177405	73198187	-
18177	1419945_s_at	Region	Rab2	59021	4	8462790	8534848	+
18178	1441632_at	Region	C130079B09Rik	399637	15	73429144	73429771	-
18179	1451532_s_at	Region	Steap	70358	5	5742327	5755316	-
18180	1457301_at	Region	AI931714	102182	8	76707830	76793401	+
18181	1422277_at	Region	Glra1	14654	11	55267654	55360631	-
18182	1431032_at	Region	Agl	77559	3	115512007	115576987	-
18183	1433558_at	Region	Dab2ip	69601	2	35624156	35663154	+
18184	1447016_at	Region	Tbc1d1	57915	5	62958072	63146849	+
18185	1435833_at	Region	None	None	6	115679812	115680524	-
18186	1451279_at	Region	Rab6ip2	111173	6	120007511	120280559	-
18187	1450710_at	Region	Jarid2	16468	13	44305547	44495137	+
18188	1423137_at	Region	Rala	56044	13	17345050	17361579	-
18189	1455405_at	Region	Pstpip2	19201	18	77916484	78001401	+
18190	1454581_at	Region	5330425B07Rik	77063	3	114918688	114919717	+
18191	1441842_s_at	Region	1500031N24Rik	69020	15	76010507	76025834	+
18192	1447503_at	Region	BC016495	225994	19	17878968	17899143	+
18193	1418241_at	Region	Usf2	22282	7	26353591	26364359	-
18194	1452310_at	Region	Tada2l	217031	11	83806912	83857560	-
18195	1440826_s_at	Region	2610002I17Rik	72341	9	110345158	110362186	+
18196	1420637_at	Region	Prps2	110639	X	160945738	160982104	-
18197	1439099_at	Region	---	384569	7	15811241	15826525	+
18198	1423375_at	Region	1700023B02Rik	66935	2	72981621	73010252	-
18199	1453198_at	Region	MGI:3028594	77652	17	30988427	31006091	+
18200	1432489_a_at	Region	2410187C16Rik	76773	15	58150452	58167670	+
18201	1429811_at	Region	4933424B01Rik	71177	6	147470322	147498449	-
18202	1440659_at	Region	2610027L16Rik	67842	14	50263729	50272080	+
18203	1437241_at	Region	Tieg3	194655	12	21201029	21212430	+
18204	1424956_at	Region	D030015G18Rik	230793	4	131972405	132038141	+
18205	1417512_at	Region	Evi5 /// LOC544746	14020	5	106815244	106945556	-
18206	1454870_x_at	Region	Gpr172b	52710	15	76589887	76593074	+
18207	1420488_at	Region	Mrps14	64659	1	160099907	160105833	+
18208	1419547_at	Region	Fahd1	68636	17	22654316	22655550	-
18209	1443592_at	Region	AA617406	98819	2	164337673	164338034	+
18210	1448843_at	Region	Ssr1	107513	13	37516882	37533029	-
18211	1440595_at	Region	Epb4.1l3	13823	17	66934864	67065569	+
18212	1458150_at	Region	D030051N19Rik	228361	2	91434974	91623598	+
18213	1425944_a_at	Region	Rad51l3	19364	11	82614373	82630248	-
18214	1416945_at	Region	Ptov1	84113	7	38938217	38944997	-
18215	1425511_at	Region	Mark1	226778	1	184396055	184497931	-
18216	1425016_at	Region	Ephb2	13844	4	135534865	135717192	-
18217	1458299_s_at	Region	Nfkbie	18037	17	43066249	43074099	+
18218	1440608_at	Region	None	None	13	22921886	22922393	-
18219	1416032_at	Region	1110006I15Rik	68539	19	10067157	10078240	-

18220	1422959_s_at	Region	Zfp313	81018	2	166949378	166972899	+
18221	1438336_at	Region	Fbxw11	103583	11	32537669	32641610	+
18222	1417417_a_at	Region	Cox6a1	12861	5	114456220	114459517	-
18223	1455679_at	Region	5830411E10Rik	109019	1	51769634	51779343	-
18224	1454897_at	Region	6330509M05Rik	102913	X	18337839	18339212	-
18225	1459411_at	Region	Sdccag10	67285	13	100849082	101034691	-
18226	1428856_at	Region	H13	14950	2	152126435	152163760	+
18227	1429435_x_at	Region	Pik3ca	18706	3	31844384	31871009	+
18228	1421387_at	Region	Kremen1	84035	11	5086339	5156397	-
18229	1453009_at	Region	Cpm	70574	10	117346301	117376298	+
18230	1418827_at	Region	Thex1	67276	8	34268325	34298553	-
18231	1448671_at	Region	Ube2e3	22193	2	78566796	78618332	+
18232	1459396_at	Region	6030446N20Rik	338363	18	12168904	12309081	-
18233	1443263_at	Region	E030004N02Rik	319905	4	32819009	32819665	+
18234	1444412_at	Region	Aim1	11630	10	44071674	44072380	-
18235	1428168_at	Region	Mpzl1	68481	1	165514161	165556442	-
18236	1440872_at	Region	None	None	13	48509128	48509859	-
18237	1429959_at	Region	None	None	16	90570636	90572050	+
18238	1439900_at	Region	None	None	14	49577473	49578502	-
18239	1437909_at	Region	D030022P06Rik	338353	7	121609117	121614997	+
18240	1455980_a_at	Region	Gas2l3	237436	10	89385653	89416830	-
18241	1457678_at	Region	2310035C23Rik	227446	1	105555177	105695178	+
18242	1418707_at	Region	Bag4	67384	8	24492266	24510265	-
18243	1425087_at	Region	2310003F16Rik	67693	2	120970950	120972288	+
18244	1415763_a_at	Region	None	None	4	128627781	128635642	+
18245	1419975_at	Region	None	None	4	107002747	107003340	+
18246	1417016_at	Region	Mapkapk5	17165	5	120676857	120697697	-
18247	1420372_at	Region	Sntb2	20650	8	106233437	106311879	+
18248	1438907_at	Region	None	None	NONE	NONE	NONE	NONE
18249	1434524_at	Region	Eif2b3	108067	4	116000045	116047677	+
18250	1432746_at	Region	6030442H21Rik	77070	2	64872641	64873910	-
18251	1424208_at	Region	Ptger4	19219	15	5019431	5029397	-
18252	1452123_s_at	Region	Frmd4b	232288	6	97752751	97993840	-
18253	1453245_at	Region	9130024F11Rik	78900	1	57272992	57276039	+
18254	1439565_at	Region	AW492303	103035	X	90021677	90022540	-
18255	1457369_at	Region	Phactr4	100169	4	131116497	131182926	-
18256	1431204_at	Region	4930578N16Rik	75051	2	69613434	69644977	-
18257	1439879_at	Region	LOC243905	243905	7	25398672	25442460	+
18258	1418717_at	Region	Mrps25	64658	6	92621555	92631448	-
18259	1446160_x_at	Region	A630048M13Rik	230657	4	115510433	115514832	-
18260	1444952_a_at	Region	Nucks1	98415	1	131762351	131784790	+
18261	1428176_at	Region	Edg5	14739	9	20842557	20853293	-
18262	1454655_at	Region	Dgkd	227333	1	87696734	87764305	+
18263	1455087_at	Region	D7Ertd715e	52480	7	53714049	53752237	-
18264	1427936_at	Region	Thnsl1	208967	2	21247734	21256386	+
18265	1457861_at	Region	A230009B12Rik	319750	17	9096278	9113813	+
18266	1423835_at	Region	Zfp503	218820	14	20348630	20354269	-
18267	1418191_at	Region	Usp18	24110	6	121677931	121702891	+
18268	1427945_at	Region	Dpyd	99586	3	117335295	118207632	+
18269	1454847_at	Region	Lhfpl2	218454	13	90241860	90379474	+
18270	1448488_at	Region	Mrps5	77721	2	127101279	127117839	+
18271	1418454_at	Region	Mfap5	50530	6	123177943	123193877	+
18272	1446877_at	Region	None	None	NONE	NONE	NONE	NONE
18273	1419809_s_at	Region	Cog4	102339	8	110144769	110179947	+
18274	1423654_a_at	Region	Rnf4	19822	5	32824728	32841768	+
18275	1443438_at	Region	Ncoa1	17977	12	3409624	3575830	-
18276	1443382_s_at	Region	None	None	8	83945298	83945730	-
18277	1418291_at	Region	Zfp87	170763	13	64115135	64125488	-
18278	1454631_at	Region	6330549H03Rik	108667	12	86970768	86973492	-
18279	1416277_a_at	Region	Rplp1	56040	9	62033165	62034420	-
18280	1421819_a_at	Region	Set	56086	2	29994205	30003957	+
18281	1434704_at	Region	None	None	5	21966120	21968134	+
18282	1434238_at	Region	None	None	15	55017077	55018450	-
18283	1450908_at	Region	Eif4e	13684	3	137416519	137446834	+
18284	1456723_at	Region	BC006909	233895	7	121521345	121526489	+

18285	1440158_x_at	Region	None	None	4	3878195	3878529	+
18286	1449932_at	Region	Csnk1d	104318	11	120784515	120812412	-
18287	1446197_at	Region	Lphn3	319387	5	80275281	81049432	+
18288	1437982_x_at	Region	Cox15	226139	19	43279531	43297387	-
18289	1416474_at	Region	Nope	56741	9	65223736	65260207	+
18290	1439967_at	Region	1700071A11Rik	76611	9	55260185	55260501	-
18291	1434168_at	Region	Peo1	226153	19	44550964	44557168	+
18292	1460620_at	Region	Zfp592	233410	7	74796384	74845397	+
18293	1416221_at	Region	Fstl1	14314	16	36596522	36655983	+
18294	1452707_at	Region	4631423F02Rik	70788	1	91174115	91185446	+
18295	1420054_s_at	Region	Slc35c2	228875	2	164733289	164744560	-
18296	1454935_at	Region	D930001I22Rik	228859	2	162925290	162929568	-
18297	1425059_at	Region	Hrmt1l6	99890	3	110054499	110059366	-
18298	1419263_a_at	Region	Adrm1	56436	2	179888546	179893241	+
18299	1433125_at	Region	5330422M15Rik	77062	4	149471005	149472085	+
18300	1455212_at	Region	Wdr24	268933	17	23629877	23634885	+
18301	1452786_at	Region	Trmt1	72026	15	85927970	85946036	+
18302	1434797_at	Region	6720469N11Rik	320339	3	86823037	86823854	-
18303	1435277_x_at	Region	Nme1	18102	11	93780012	93789608	-
18304	1438183_x_at	Region	Sdh1	20322	2	121748692	121779190	+
18305	1452592_at	Region	Mgst2	211666	3	51293201	51314729	+
18306	1419984_s_at	Region	Zfp644	52397	5	105678595	105758844	-
18307	1443757_x_at	Region	D1Ertd161e	52231	1	75480104	75487168	+
18308	1453823_a_at	Region	A930028L21Rik	229780	3	115354021	115363721	-
18309	1417320_at	Region	Grpel1	17713	5	34952931	34961823	+
18310	1417237_at	Region	Pld2	18806	11	70265822	70283768	+
18311	1460355_at	Region	D030060M11Rik	215051	9	46296268	46312116	+
18312	1447475_at	Region	Gm967	381217	19	23211527	23268980	-
18313	1426497_at	Region	Jarid1c	20591	X	145767667	145808191	+
18314	1422648_at	Region	Slc7a2	11988	8	39830084	39853621	+
18315	1449913_at	Region	Zfp2	22678	11	50651553	50669006	-
18316	1418066_at	Region	Cfl2	12632	12	51564268	51568328	-
18317	1460477_at	Region	1700010C24Rik	70363	15	71476555	71493104	-
18318	1439386_x_at	Region	Mat2a	232087	6	72764773	72771532	-
18319	1438463_x_at	Region	Zdhhc6	66980	19	54859814	54896233	-
18320	1426526_s_at	Region	Ovgp1	12659	3	105769631	105783252	+
18321	1441624_at	Region	9430041O17Rik	234214	8	44653046	44768456	+
18322	1431207_at	Region	2900024O10Rik	72852	13	77784829	77786286	+
18323	1441572_at	Region	C030036D22Rik	77607	18	71487787	71488446	-
18324	1444828_at	Region	Ppp2r5c	26931	12	105961123	106055786	+
18325	1451530_at	Region	Egfr	13649	11	16646996	16808700	+
18326	1441574_at	Region	Pitpnc1	71795	11	107033195	107291788	-
18327	1415728_at	Region	Pabpn1	54196	14	49410993	49414893	+
18328	1447672_x_at	Region	Pskh1	244631	8	105196321	105227649	+
18329	1425865_a_at	Region	Lig3	16882	11	82520876	82541867	+
18330	1425484_at	Region	Tox	252838	4	6614604	6917869	-
18331	1444082_at	Region	A730017C20Rik	225583	18	59287736	59302184	+
18332	1434495_at	Region	Zfp278	56218	11	3185244	3203304	+
18333	1427894_at	Region	Slitl2	246154	16	4311498	4322290	+
18334	1448734_at	Region	Cp	12870	3	19298453	19333864	+
18335	1417974_at	Region	Kpna4	16649	3	68742743	68797614	-
18336	1456337_at	Region	Centd1	212285	5	61409120	61555599	-
18337	1422687_at	Region	None	None	3	102486135	102494605	+
18338	1433283_s_at	Region	4930529H12Rik	75204	Y	25782383	25792672	+
18339	1455013_at	Region	Arih2	23807	9	108651717	108698223	-
18340	1428104_at	Region	Tpx2	72119	2	152304999	152350954	+
18341	1453720_at	Region	Rnf157	217340	11	116157470	116234559	-
18342	1456363_at	Region	St7l	229681	3	104291995	104353941	+
18343	1427782_a_at	Region	Crhr1	12921	11	103954131	103996611	+
18344	1420937_at	Region	Cpsf2	51786	12	97421457	97450986	+
18345	1420657_at	Region	Ucp3	22229	7	94579662	94593103	+
18346	1428797_at	Region	0610039J04Rik	66523	8	124126680	124150314	-
18347	1429459_at	Region	Sema3d	108151	5	11823702	12027204	+
18348	1454628_at	Region	A930037G23Rik	320678	6	125801418	125818067	+
18349	1431825_at	Region	Stk23	56504	X	68435077	68439580	+

18350	1448613_at	Region	Ecm1	13601	3	95221981	95227394	-
18351	1438520_at	Region	Slc25a36	192287	9	96976369	97010399	-
18352	1435157_at	Region	5830454D03Rik	109029	8	24746173	24747112	-
18353	1446276_at	Region	None	None	16	14778215	14778894	-
18354	1437953_at	Region	2310032D16Rik	74182	2	132043251	132092031	-
18355	1438146_x_at	Region	None	None	7	41076380	41076877	-
18356	1457357_at	Region	Tlk2	24086	11	104998272	105103265	+
18357	1444211_at	Region	Endogl1	208194	9	119439221	119458491	+
18358	1452438_s_at	Region	Taf4a	228980	2	179629110	179693604	-
18359	1429506_at	Region	Nkd1	93960	8	87806676	87877632	+
18360	1416463_at	Region	Gpiap1	53872	2	103469773	103502351	-
18361	1426969_at	Region	Trim23	81003	13	100397284	100420234	+
18362	1425273_s_at	Region	Emp2	13731	16	9952848	9985199	-
18363	1450967_at	Region	4933428I03Rik	66775	4	87399122	87425111	-
18364	1443824_s_at	Region	Car7	12354	8	103833942	103843478	+
18365	1457042_at	Region	None	None	6	101286708	101287488	+
18366	1441204_at	Region	A130019P10Rik	399617	10	61406376	61407057	-
18367	1454393_at	Region	2310047C04Rik	224170	16	47767664	47835780	-
18368	1438169_a_at	Region	Frmd4b	232288	6	97752751	97993840	-
18369	1454923_at	Region	AW214353	107036	NONE	NONE	NONE	NONE
18370	1435911_s_at	Region	Slc2a12	353169	10	22634051	22693947	+
18371	1424051_at	Region	Col4a2	12827	8	10685449	10825114	+
18372	1444762_at	Region	Ppp1r12a	17931	10	108049328	108167253	+
18373	1439304_at	Region	B230216N24Rik	78603	1	97825770	97826481	-
18374	1441767_at	Region	Zfp142	77264	1	74868909	74890473	-
18375	1440470_at	Region	C030006K11Rik	223665	15	76772844	76775219	-
18376	1459658_at	Region	None	None	8	74292042	74292364	+
18377	1453170_at	Region	2610206C24Rik	67158	18	32150744	32151382	-
18378	1416975_at	Region	Stam2	56324	2	52624368	52674311	-
18379	1440563_at	Region	Fancc	14088	13	60694745	60814715	-
18380	1439705_at	Region	Ptpn12	19248	5	19439215	19508367	-
18381	1433496_at	Region	Glt25d1	234407	8	70760670	70774886	+
18382	1458216_at	Region	None	None	1	38160302	38160649	+
18383	1455478_at	Region	Ppfia1	233977	7	138888626	138965593	-
18384	1424296_at	Region	Gclc	14629	9	77899181	77938127	+
18385	1416722_at	Region	Hmg20a	66867	9	56527993	56606058	+
18386	1417207_at	Region	Dvl2	13543	11	69726284	69735769	+
18387	1451333_a_at	Region	Acrbp	54137	6	125705979	125719247	+
18388	1453534_at	Region	2810004I08Rik	69932	10	105730775	105733702	-
18389	1458436_at	Region	None	None	13	51470568	51470781	-
18390	1437771_at	Region	1110067I12Rik	68852	19	8049785	8050457	+
18391	1434133_s_at	Region	None	None	1	172108858	172125304	+
18392	1421836_at	Region	None	None	10	20074928	20208696	+
18393	1418669_at	Region	None	None	4	136350082	136451892	+
18394	1434437_x_at	Region	Rrm2	20135	12	21258038	21263862	+
18395	1426421_s_at	Region	1700009P03Rik	74213	14	99658921	99720050	-
18396	1422113_at	Region	Slc5a5	114479	8	70039215	70048437	-
18397	1429842_at	Region	Mdh1b	76668	1	63991845	64023336	-
18398	1457778_at	Region	Stox2	71069	8	46138301	46167033	-
18399	1434122_at	Region	Fbxo27	233040	7	24098722	24105207	+
18400	1454825_at	Region	1110014N23Rik	68505	19	5858871	5865993	-
18401	1456859_at	Region	None	None	2	74212586	74213047	-
18402	1426669_at	Region	C530044N13Rik	223978	16	8039700	8138558	-
18403	1423099_a_at	Region	Mettl3	56335	14	47394773	47405188	-
18404	1427678_at	Region	Zim3	116811	7	6167455	6182644	-
18405	1427339_at	Region	Slc30a2	230810	4	133303948	133315380	+
18406	1448046_at	Region	Rabepk	227746	2	34710828	34731953	-
18407	1459801_at	Region	B3galt5	93961	16	95762296	95799852	+
18408	1430391_a_at	Region	St8sia4	20452	1	95359334	95439253	-
18409	1428475_at	Region	1190005P17Rik	66225	10	119804757	119809767	+
18410	1458746_at	Region	A430107O13Rik	214642	6	22056746	22284808	+
18411	1427703_at	Region	Pafah1b1	18472	11	74399613	74450328	-
18412	1419097_a_at	Region	Stom	13830	2	35246152	35269171	-
18413	1416337_at	Region	Uqcrb	67530	13	63503055	63507784	-
18414	1460234_at	Region	BC054822	192652	11	75166610	75180353	-

18415	1422764_at	Region	Mapre1	13589	2	153198313	153225162	+
18416	1437362_at	Region	A930028L21Rik	229780	3	115354021	115363721	-
18417	1436300_at	Region	Ripk5	213452	1	132274936	132320783	+
18418	1452134_at	Region	3010001K23Rik	72392	5	107706217	107723478	+
18419	1459626_at	Region	None	None	11	97671401	97671893	-
18420	1425842_at	Region	Edil3	13612	13	84938267	85437638	+
18421	1430107_at	Region	9230116B18Rik	78245	2	3250081	3258264	+
18422	1436802_at	Region	Ilf3	16201	9	21254848	21291586	+
18423	1425203_at	Region	Ddx19b	234733	8	110304375	110325446	-
18424	1460111_at	Region	Myt1l	17933	12	26094752	26491102	+
18425	1456082_x_at	Region	Cct4	12464	11	22885383	22898126	+
18426	1436952_at	Region	Klf9	16601	19	22379147	22404832	+
18427	1437957_at	Region	7030407O06Rik	320392	12	78436467	78437126	-
18428	1440668_at	Region	Adamtsl3	269959	7	76340040	76470969	+
18429	1434284_at	Region	G630013P12Rik	218513	13	96212928	96218702	-
18430	1417440_at	Region	Arid1a	93760	4	132639909	132683944	-
18431	1435172_at	Region	Eomes	13813	9	118472453	118479608	+
18432	1421948_a_at	Region	2610507L03Rik	72140	7	30562511	30603407	+
18433	1434692_at	Region	1110034B05Rik	68736	1	57691976	57710415	-
18434	1415776_at	Region	Aldh3a2	11671	11	60970498	60992756	-
18435	1425268_a_at	Region	3110045G13Rik	73182	3	87494202	87514047	-
18436	1427702_at	Region	Zfp1	22640	8	110941032	110968597	+
18437	1426129_at	Region	Brms1	107392	19	4830194	4838707	+
18438	1448701_a_at	Region	Ccm1	79264	5	3809194	3847364	+
18439	1459690_at	Region	Zfp426	235028	9	20341367	20365016	-
18440	1451306_at	Region	Cdca7l	217946	12	113608220	113642229	+
18441	1434689_at	Region	Zfp637	232337	6	118272695	118277409	+
18442	1445425_at	Region	LOC432958	432958	15	63427667	63498744	-
18443	1438643_at	Region	Camk1d	227541	2	5214190	5631787	-
18444	1430642_at	Region	2900001G08Rik	76950	17	28751040	28753533	+
18445	1424151_at	Region	MGI:2385237	231872	5	142944317	142951426	-
18446	1452204_at	Region	Anks1	224650	17	25705258	25858555	+
18447	1433570_s_at	Region	Mak10	78689	13	58245009	58294396	+
18448	1422046_at	Region	Itgam	16409	7	122085995	122167434	+
18449	1457384_at	Region	None	None	8	21163057	21163480	-
18450	1422796_at	Region	Prep	19072	10	45184189	45275958	+
18451	1449969_at	Region	Tmod4	50874	3	94613362	94617039	+
18452	1445500_at	Region	None	None	12	18791693	18792112	+
18453	1438688_at	Region	Srrm2	75956	17	21605406	21626916	+
18454	1440627_at	Region	Atp8a2	50769	14	54182804	54623125	-
18455	1424596_s_at	Region	Lmcd1	30937	6	112835874	112893523	+
18456	1440048_at	Region	Smarca5	93762	8	79911951	79951467	-
18457	1446194_at	Region	Nr2c1	22025	10	94129628	94168693	+
18458	1424459_at	Region	BC005662	210992	13	69524486	69573696	+
18459	1429957_at	Region	2310002B14Rik	69533	16	87804256	87805228	-
18460	1419290_at	Region	1600013P15Rik	67011	14	29610661	29626848	-
18461	1434505_a_at	Region	None	None	8	119519703	119520816	+
18462	1457880_at	Region	None	None	5	98226104	98226705	-
18463	1459357_at	Region	Nfasc	269116	1	132427503	132603217	-
18464	1459237_at	Region	Atf2	11909	2	73516345	73590377	-
18465	1449215_at	Region	Slc22a21	56517	11	53703665	53732868	-
18466	1438917_x_at	Region	Nup62	18226	7	38891347	38905734	+
18467	1427907_at	Region	1110037F02Rik	66185	4	11412923	11477649	+
18468	1427309_at	Region	BC027073	230577	4	105610011	105614187	+
18469	1428330_at	Region	2610510B01Rik	70028	16	92972121	92980341	+
18470	1441037_at	Region	None	None	18	53128861	53129549	+
18471	1459716_at	Region	AI835735	102589	9	50764983	50765345	-
18472	1425648_at	Region	Trim60 /// Trim61	234329	8	64114328	64132822	-
18473	1443134_at	Region	---	503690	5	114532913	114533579	-
18474	1428689_at	Region	Tysnd1	71767	10	61661818	61669070	+
18475	1428243_at	Region	1700021K19Rik	224118	16	31629788	31676391	-
18476	1458452_at	Region	3010027A04Rik	77087	8	122264618	122423554	-
18477	1427258_at	Region	Trim24	21848	6	38005830	38101311	+
18478	1453404_at	Region	2900002H16Rik	75695	5	123664423	123702734	-
18479	1428845_at	Region	Bclaf1	72567	10	20239740	20267469	+

18480	1420519_a_at	Region	Eral1	57837	11	77799035	77806004	-
18481	1428746_a_at	Region	1110007F05Rik	71787	4	131272654	131290416	-
18482	1445713_at	Region	9630041G16Rik	100223	4	56901589	56901783	+
18483	1444073_at	Region	Maf	17132	8	115014984	115016041	-
18484	1453174_at	Region	2310076G13Rik	71938	1	60638785	60641619	+
18485	1447728_x_at	Region	Hspa9a	15526	18	35161089	35177981	-
18486	1440791_x_at	Region	Tcea2	21400	2	181397266	181405007	+
18487	1449090_a_at	Region	Yes1	22612	5	31069457	31146984	+
18488	1428961_a_at	Region	Sfrs16	53609	7	16450287	16473605	-
18489	1418033_s_at	Region	Zfp535	52712	11	65532894	65554889	+
18490	1457642_at	Region	5730507N06Rik	70622	2	18090623	18090916	-
18491	1454428_at	Region	4930467D19Rik	75004	11	103326371	103328046	+
18492	1431352_s_at	Region	Pvt1	19296	15	62055194	62257049	+
18493	1447591_x_at	Region	A830082N09Rik	414093	10	34047187	34047372	-
18494	1423900_at	Region	Trip12	14897	1	85055153	85095994	-
18495	1440480_at	Region	LOC192950	192950	11	6492611	6500842	-
18496	1417882_at	Region	Slc39a3	106947	10	81162385	81167757	-
18497	1425839_at	Region	Fkbp11	66120	15	98781810	98785637	-
18498	1416438_at	Region	2410104I19Rik	67959	15	76118862	76129542	-
18499	1439505_at	Region	Clic5	224796	17	41693345	41782570	+
18500	1416848_at	Region	Ubl5	66177	9	20514222	20517693	+
18501	1418112_at	Region	Mrpl10	107732	11	96862673	96870300	+
18502	1459573_at	Region	2310066N05Rik	70285	3	145482969	145497969	-
18503	1443513_at	Region	Cox11	69802	11	90459260	90466023	+
18504	1440408_at	Region	B830008J18Rik	319947	NONE	NONE	NONE	NONE
18505	1441330_at	Region	Crb1	170788	1	139045517	139224215	-
18506	1443764_x_at	Region	Rab27b	80718	18	70213787	70288489	-
18507	1453053_at	Region	2610036L11Rik	66311	10	30232476	30408425	-
18508	1455394_at	Region	None	None	10	81288081	81288804	-
18509	1428311_at	Region	2810012H18Rik	76551	10	70243818	70247349	+
18510	1424786_s_at	Region	Wdr45	54636	X	5961189	5967141	+
18511	1424356_a_at	Region	Metrnl	210029	11	121523520	121538482	+
18512	1433068_at	Region	6330582A15Rik	76878	NONE	NONE	NONE	NONE
18513	1448116_at	Region	Ube1x	22201	X	18897361	18922119	+
18514	1449688_at	Region	D10Ertd610e	52666	10	126915321	126923060	-
18515	1447531_x_at	Region	Cugbp2	14007	2	6459141	6638885	-
18516	1416450_at	Region	Tbn	63856	17	45006510	45020747	-
18517	1451779_at	Region	BC027061	232491	6	143188941	143205305	+
18518	1440373_at	Region	Elavl1	15568	8	3643294	3667872	-
18519	1420070_a_at	Region	Gm696	277973	8	104644962	104658911	+
18520	1455995_at	Region	D10Bwg1379e	215821	10	18490148	18668026	-
18521	1460356_at	Region	Esam1	69524	9	37469661	37479855	+
18522	1457653_at	Region	Csf1r	12978	18	61331201	61356458	+
18523	1435889_at	Region	Mark2	13728	19	6990598	7055161	-
18524	1453834_at	Region	1500002C15Rik	68946	4	154226027	154227364	-
18525	1444131_at	Region	2610101N10Rik	67958	9	95369771	95420790	-
18526	1431962_a_at	Region	Stambp	70527	6	83887437	83916666	-
18527	1423141_at	Region	Lip1	16889	19	33824007	33859684	-
18528	1452220_at	Region	Dock1	330662	7	129038306	129541299	+
18529	1428479_at	Region	Nfatc1	18018	18	80729557	80836410	-
18530	1449935_a_at	Region	Dnaja3	83945	16	4355627	4379131	+
18531	1418404_at	Region	Rad9	19367	19	3983988	3990393	-
18532	1448504_a_at	Region	None	None	1	58951467	58953202	+
18533	1436712_at	Region	Pla2g4c	232889	7	11323270	11353889	+
18534	1454222_a_at	Region	Ccdc13	72894	9	121810629	121823689	-
18535	1455506_at	Region	None	None	4	140500001	140500487	-
18536	1428277_at	Region	2600013N14Rik	72201	4	14736470	14753349	-
18537	1441901_x_at	Region	Fahd2a	68126	2	126950068	126958394	-
18538	1453040_at	Region	2810402A17Rik	67062	X	130545957	130586403	-
18539	1419204_at	Region	Dll1	13388	17	13371006	13379239	-
18540	1434953_at	Region	4633402D15Rik	70829	1	121194302	121212958	+
18541	1419513_a_at	Region	Ect2	13605	3	26515043	26571653	-
18542	1448056_at	Region	Huwe1	59026	X	145444189	145445007	+
18543	1436597_at	Region	Ankhd1	108857	18	36872342	36882677	+
18544	1449827_at	Region	Agc1	11595	7	72845590	72907206	+

18545	1436059_at	Region	Rfx1	19724	8	83330088	83360431	+
18546	1448482_at	Region	Slc39a8	67547	3	134710465	134772768	+
18547	1426426_at	Region	Rbm13	67920	8	29942286	29951542	-
18548	1449608_a_at	Region	None	None	13	48081110	48081498	-
18549	1440709_at	Region	C130081A10Rik	97550	9	50921489	50922068	+
18550	1427467_a_at	Region	Rpgr	19893	X	8397164	8455861	-
18551	1430275_a_at	Region	Aqr	11834	2	113615012	113689159	-
18552	1427171_at	Region	Rlf	109263	4	120167817	120237523	-
18553	1442295_at	Region	Arpc2	76709	1	74538971	74570677	+
18554	1443294_at	Region	Crk7	69131	11	98024401	98067791	+
18555	1430488_at	Region	1700066B19Rik	73449	18	35950670	35952866	+
18556	1443729_at	Region	Mtss1	211401	15	58954404	59092928	-
18557	1428779_at	Region	Zbtb41	226470	1	139269557	139296474	+
18558	1442018_at	Region	AI426953	103207	10	96599811	96600436	+
18559	1436189_at	Region	Serpinb6a	20719	13	33449537	33467606	-
18560	1460559_at	Region	Ankrd25	235041	9	21661250	21691677	-
18561	1418089_at	Region	Stx8	55943	11	67692133	67932806	+
18562	1424255_at	Region	Supt5h	20924	7	23712498	23737897	-
18563	1435737_a_at	Region	Nde1	67203	16	12905487	12928489	+
18564	1417248_at	Region	Ralbp1	19765	17	63557880	63594264	-
18565	1418188_a_at	Region	Ramp2	54409	11	101067518	101069332	+
18566	1448266_at	Region	Edf1	59022	2	25490062	25494246	+
18567	1451746_a_at	Region	Atg12l	67526	18	46954183	46963313	-
18568	1428543_at	Region	Ppat	231327	5	76158008	76196037	-
18569	1435664_at	Region	Zfp397	69256	18	24183182	24191362	+
18570	1460692_at	Region	Ehmt2	110147	17	32601358	32617827	+
18571	1433001_at	Region	1700019L13Rik	72226	9	119993099	119994745	+
18572	1432047_at	Region	Depdc2	76135	1	11099255	11255533	+
18573	1456623_at	Region	Tpm1	22003	9	67151552	67178023	-
18574	1440790_x_at	Region	AL024069	98496	1	84370257	84618548	-
18575	1434516_at	Region	Pstk	214580	7	125686223	125703402	+
18576	1451334_at	Region	1810009O10Rik	69109	11	78476165	78477388	-
18577	1421875_a_at	Region	Mrps23	64656	11	87932410	87939499	+
18578	1426561_a_at	Region	Npnt	114249	3	131774826	131840439	-
18579	1434167_at	Region	Slc35e4	103710	11	3801809	3809451	-
18580	1455312_at	Region	Phc3	241915	3	30318234	30380826	-
18581	1419209_at	Region	Cxcl1	14825	5	90207981	90209786	+
18582	1443909_at	Region	Cstf3	228410	2	104295277	104370179	+
18583	1434544_at	Region	1110025L05Rik	66162	7	120745731	120746424	+
18584	1425489_at	Region	D11Ertd730e	193116	11	43186568	43200818	+
18585	1428249_at	Region	Pygo2	68911	3	89186990	89191857	+
18586	1418674_at	Region	Osmr	18414	15	6602157	6662956	-
18587	1437239_x_at	Region	Phc2	54383	4	127732109	127779987	+
18588	1428915_at	Region	Sirt5	68346	13	42933150	42957643	+
18589	1424427_at	Region	Tada1l	27878	1	166301077	166315398	+
18590	1437850_a_at	Region	Cnbp1	12785	6	88279741	88287657	-
18591	1418906_at	Region	Nubp1	26425	16	10086475	10098031	+
18592	1430510_at	Region	B130050I23Rik	213027	8	3521545	3563004	+
18593	1426883_at	Region	AW491445	107375	19	5667256	5674558	+
18594	1427013_at	Region	Car9	230099	4	43422929	43429651	+
18595	1451469_at	Region	B430108F07Rik	78519	4	84084296	84117940	+
18596	1437007_x_at	Region	Usp39	28035	6	72650650	72677149	-
18597	1441844_at	Region	Eps15-rs	13859	8	71489790	71569758	-
18598	1423982_at	Region	Fusip1	14105	4	134737416	134751165	+
18599	1444477_at	Region	Lrrtm3	216028	10	63866884	64028642	-
18600	1449577_x_at	Region	Tpm2	22004	4	43430634	43439310	-
18601	1439198_at	Region	Ptk2	14083	15	73233486	73423105	-
18602	1430185_at	Region	5830460E08Rik	76109	7	69515421	69516877	+
18603	1428022_at	Region	Lcn13	227627	2	25632236	25635488	+
18604	1450438_at	Region	Ncam1	17967	9	49555319	49607941	-
18605	1435072_at	Region	Zfyve1	217695	12	80410951	80458920	-
18606	1455017_a_at	Region	Zfp261	56364	X	96005746	96022210	-
18607	1446562_at	Region	Sh3kbp1	58194	X	153308006	153573572	+
18608	1459602_at	Region	Ccni	12453	5	92517687	92541778	-
18609	1457792_at	Region	None	None	15	98968123	98968651	-

18610	1442103_at	Region	Nipbl	71175	15	8078809	8232047	-
18611	1423738_at	Region	Oxa1l	69089	14	48877816	48886644	+
18612	1436834_x_at	Region	Mdh1	17449	11	21451580	21466622	-
18613	1442437_at	Region	Ptk2b	19229	14	60680682	60808472	-
18614	1457360_at	Region	Cfdp1	23837	8	111066062	111151899	-
18615	1417758_at	Region	Itga2b	16399	11	102274384	102290831	-
18616	1433441_at	Region	Fbxl5	242960	5	42512957	42539147	-
18617	1425819_at	Region	Zbtb36	207259	18	76055363	76384962	+
18618	1453622_s_at	Region	Mllt3	70122	4	86758854	87019463	-
18619	1422626_at	Region	Mmp16	17389	4	17780448	18043598	+
18620	1440260_at	Region	A930007B11Rik	77944	16	34858068	34859199	+
18621	1458289_at	Region	Galntl4	233733	7	105324798	105633114	-
18622	1425006_a_at	Region	Vrk1	22367	12	101457929	101523008	+
18623	1448839_at	Region	D17Ertd288e	80880	17	31509056	31521298	+
18624	1417448_at	Region	1810008A18Rik	108707	10	77598097	77627221	-
18625	1455704_at	Region	4931440N07Rik	381560	4	131685803	131693445	-
18626	1419840_at	Region	AI507495	105866	15	101835022	101844920	-
18627	1440994_at	Region	None	None	10	11094929	11095592	+
18628	1427593_at	Region	Trim8	93679	19	46050008	46064111	+
18629	1450865_s_at	Region	Mrps24	64660	11	5598770	5602472	-
18630	1454449_at	Region	Rabepk	227746	2	34710828	34731953	-
18631	1436354_at	Region	Dzip1l	72507	9	99531123	99570401	+
18632	1438172_x_at	Region	4933424N09Rik	71151	7	113660463	113669549	-
18633	1459453_at	Region	Robo1	19876	16	71741946	72126752	+
18634	1453416_at	Region	Gas2l3	237436	10	89385653	89416830	-
18635	1437848_x_at	Region	Adpgk	72141	9	59408367	59432955	+
18636	1453724_a_at	Region	Serpinf1	20317	11	75135427	75148170	-
18637	1417824_at	Region	Gcat	26912	15	79082206	79089640	+
18638	1456191_x_at	Region	Pacsin3	80708	2	90957350	90969427	+
18639	1431353_at	Region	C330050A14Rik	78704	3	46037953	46038960	+
18640	1447595_x_at	Region	1810012K16Rik	69108	8	21150892	21151766	+
18641	1443261_at	Region	E130307A14Rik	327744	10	39748918	39801774	-
18642	1440130_at	Region	BB116930	98740	NONE	NONE	NONE	NONE
18643	1417238_at	Region	Ewsr1	14030	11	4964688	4993864	-
18644	1424935_at	Region	Gdap1l1	228858	2	162895447	162910921	+
18645	1433475_a_at	Region	C78339	97863	13	46253320	46273975	+
18646	1455756_at	Region	F730014I05Rik	228866	2	164336101	164348170	+
18647	1422309_a_at	Region	Lenep /// A930017E24Rik	319945	3	89157732	89168601	-
18648	1451226_at	Region	Pex6	224824	17	44222425	44236648	+
18649	1428798_s_at	Region	0610039J04Rik	66523	8	124126680	124150314	-
18650	1439400_x_at	Region	5430433E21Rik	74492	9	65510949	65513917	-
18651	1433618_at	Region	C330006A16Rik	109299	2	26068973	26070848	-
18652	1458395_at	Region	B930054O08	327837	10	122889763	122890511	+
18653	1426782_at	Region	Gpr125	70693	5	48771796	48873049	-
18654	1420881_at	Region	Nsd1	18193	13	53854390	53923720	+
18655	1423385_at	Region	Actr8	56249	14	28112122	28126949	+
18656	1443005_at	Region	Zfhx1a	21417	18	5709786	5817483	+
18657	1455633_at	Region	Zfp647	239546	15	76961299	76976539	-
18658	1436219_at	Region	4933403F05Rik	108654	18	68497464	68531006	-
18659	1442319_at	Region	Usp4	22258	9	108417796	108462269	+
18660	1452629_at	Region	Safb2	224902	17	54251255	54263875	-
18661	1422482_at	Region	Ruvbl2	20174	7	39499052	39511390	-
18662	1453211_at	Region	1700019B01Rik	70054	7	84476433	84478520	+
18663	1437040_at	Region	Etnk2	214253	1	133225130	133236393	+
18664	1429831_at	Region	Pik3ap1	83490	19	40818590	40929442	-
18665	1448451_at	Region	Ak2	11637	4	128020427	128038642	+
18666	1421137_a_at	Region	Pkib	18768	10	57894545	58001405	+
18667	1446969_at	Region	9630037P07Rik	227210	1	64989291	65019720	+
18668	1460138_at	Region	None	None	19	20911861	20912164	+
18669	1456236_s_at	Region	Commd10	69456	18	47180632	47309751	+
18670	1439388_s_at	Region	Bcar1	12927	8	111008063	111041398	-
18671	1450025_at	Region	Pard6g	93737	18	80170776	80242901	+
18672	1457544_at	Region	6130401J04Rik	66799	1	16725948	16804454	-
18673	1435180_at	Region	Podn	242608	4	106973698	106990995	-

18674	1433952_at	Region	Tufm	233870	7	120537163	120540461	+
18675	1431940_at	Region	4933424M13Rik	74457	5	134724380	134725692	+
18676	1444550_at	Region	1300018I05Rik	74157	17	27470422	27513276	+
18677	1430505_at	Region	1110032O16Rik	68742	7	120935951	120972691	-
18678	1460646_at	Region	Csnk2a2	13000	8	94726673	94766378	-
18679	1442590_at	Region	Tnfrsf22	79202	7	138049744	138062657	-
18680	1419207_at	Region	Zfp37	22696	4	61280734	61299618	-
18681	1440279_at	Region	None	None	18	14170053	14170865	-
18682	1459056_at	Region	Rasgrf1	19417	9	89795814	89912965	+
18683	1428690_at	Region	Tysnd1	71767	10	61661818	61669070	+
18684	1420608_at	Region	Rbm18	67889	2	36048243	36068773	-
18685	1443254_at	Region	D030011O10Rik	320560	6	149949871	150037421	-
18686	1430128_a_at	Region	Dp1l1	70335	10	80455181	80461452	+
18687	1443792_at	Region	Tsga14	83922	6	30701454	30741755	-
18688	1440832_at	Region	Ang4	219033	14	46858315	46868013	-
18689	1423901_at	Region	Trip12	14897	1	85055153	85095994	-
18690	1426593_a_at	Region	Fbxo22	71999	9	55320406	55335959	+
18691	1420815_at	Region	Gdi3	14569	13	3422528	3450201	+
18692	1448137_at	Region	Aldh7a1	110695	18	56744086	56781108	-
18693	1423781_at	Region	Appbp1	234664	8	103804159	103830138	-
18694	1455221_at	Region	Abcg1	11307	17	28870974	28929775	+
18695	1438614_x_at	Region	Osbpl9	100273	4	108020555	108061497	-
18696	1429770_at	Region	Pggt1b	225467	18	46460574	46501475	-
18697	1451556_a_at	Region	2700078E11Rik	78832	19	60127914	60181028	-
18698	1439787_at	Region	None	None	5	121836078	121837200	+
18699	1426356_at	Region	6330578E17Rik	76178	1	37711567	37713525	-
18700	1459303_at	Region	Gpm6a	234267	8	53936872	54043110	+
18701	1456059_at	Region	Psmd11	69077	11	80154287	80197624	+
18702	1454256_s_at	Region	1700113I22Rik	73635	11	101239901	101246420	-
18703	1447706_at	Region	None	None	NONE	NONE	NONE	NONE
18704	1453175_at	Region	Zfp50	109929	12	73204978	73206174	-
18705	1440451_at	Region	D730048J04Rik	77856	17	41035584	41036004	-
18706	1438170_x_at	Region	Adrm1	56436	2	179888546	179893241	+
18707	1417659_at	Region	Vps29	56433	5	121506763	121515637	+
18708	1434710_at	Region	Dhx29	218629	13	109322761	109364225	+
18709	1451770_s_at	Region	Dhx9	13211	1	153340539	153372335	-
18710	1439638_at	Region	Erbb2ip	59079	13	100037161	100140118	-
18711	1457045_at	Region	Galnt13	271786	2	54361926	55038861	+
18712	1432108_at	Region	Pcgf6	71041	19	46584919	46602145	-
18713	1442863_at	Region	5730412N02Rik	319734	6	119700072	119709391	+
18714	1459593_x_at	Region	LOC547217	547217	7	265	9009	+
18715	1455774_at	Region	None	None	8	69851008	69851565	-
18716	1454963_at	Region	E430028B21Rik	211948	14	24760322	24766051	-
18717	1447907_x_at	Region	Apba2bp	56846	2	154001342	154012591	-
18718	1450911_at	Region	Ppib	19035	9	66145681	66189949	+
18719	1442775_at	Region	St13	70356	15	81415844	81450494	-
18720	1440119_at	Region	Sipa1l1	217692	12	79172130	79311045	+
18721	1443656_at	Region	Fut8	53618	12	74095608	74332578	+
18722	1439896_at	Region	Limk2	16886	11	3239044	3303961	-
18723	1415851_a_at	Region	Impdh2	23918	9	108609499	108614571	+
18724	1427132_at	Region	Mtmr13	319934	7	104160882	104472741	-
18725	1429163_at	Region	3110041P15Rik	73159	7	99867642	99878087	-
18726	1458666_at	Region	D2Ertd391e	51897	2	91379374	91415325	-
18727	1431374_at	Region	6330407A03Rik	70720	4	3642110	3643949	+
18728	1434050_at	Region	AI315068	209018	16	20205751	20416183	+
18729	1416485_at	Region	Timm23	53600	14	30313306	30335011	-
18730	1451599_at	Region	Sesn2	230784	4	131453703	131471355	-
18731	1459861_s_at	Region	Fbxl10	30841	5	122023577	122142001	-
18732	1424967_x_at	Region	Tnnt2	21956	1	135683299	135699154	+
18733	1429583_at	Region	LOC554362	554362	8	23373351	23375971	+
18734	1454070_a_at	Region	Ddhd1	114874	14	40672980	40701448	-
18735	1434506_at	Region	Arid2	77044	15	96436802	96469595	+
18736	1441470_at	Region	AV249152	216560	11	21467074	21793479	+
18737	1457044_at	Region	4732474O15Rik	238455	12	115250643	115278405	+
18738	1439008_at	Region	Zfp319	79233	8	94610307	94616121	-

18739	1419087_s_at	Region	Sf3a1	67465	11	4055155	4075416	+
18740	1441162_at	Region	None	None	18	10114639	10115304	-
18741	1453443_at	Region	1110015O18Rik	68516	3	4773012	4788431	+
18742	1445914_at	Region	Nrf1	18181	6	30095085	30200555	+
18743	1441856_x_at	Region	Tysnd1	71767	10	61661818	61669070	+
18744	1444174_at	Region	Adamts8	30806	9	30861390	30881684	+
18745	1444686_at	Region	A930016P21Rik	68073	15	31602722	31618141	+
18746	1442816_at	Region	AU041882	104525	11	63609804	63610896	-
18747	1441301_at	Region	Gababrbp	76071	5	35557428	35631807	+
18748	1456422_at	Region	None	None	6	149928454	149929684	-
18749	1444570_at	Region	Cfdp1	23837	8	111066062	111151899	-
18750	1450349_at	Region	Stx1b1	79361	7	121856654	121874341	-
18751	1441334_at	Region	None	None	X	11432477	11433376	+
18752	1442869_at	Region	A930013K19	231134	5	33544762	33575509	+
18753	1417557_at	Region	Ubxd1	66530	17	53706451	53713187	-
18754	1453030_at	Region	Rnf184	77853	9	101048875	101053251	+
18755	1447213_at	Region	Sp100	20684	1	85456739	85507923	+
18756	1437045_at	Region	Mapk8	26419	14	31516453	31580724	-
18757	1431677_at	Region	None	None	11	43462297	43463723	+
18758	1420933_a_at	Region	Eya3	14050	4	131599945	131685662	+
18759	1441879_x_at	Region	Mkrn1	54484	6	39533206	39555816	-
18760	1454416_at	Region	5730575I04Rik	70649	2	115282017	115283861	+
18761	1459414_at	Region	Wdfy3	72145	5	100857137	101091457	-
18762	1458933_at	Region	A530052I06Rik	99953	3	101282357	101282915	-
18763	1436027_at	Region	Osbpl11	106326	16	31993123	32051348	+
18764	1441718_at	Region	4432411E13Rik	70790	15	37972806	38204070	-
18765	1437244_at	Region	Gas2l3	237436	10	89385653	89416830	-
18766	1439401_x_at	Region	Ppp2r5e	26932	12	72307243	72452562	-
18767	1452440_at	Region	Tnfsf12 /// Tnfsf13	21944	11	69412006	69421412	-
18768	1438653_x_at	Region	Sca10	54138	15	85384867	85511564	+
18769	1456054_a_at	Region	Pum1	80912	4	129460274	129578157	+
18770	1432853_at	Region	2900057C01Rik	72987	8	106272520	106273640	-
18771	1433021_at	Region	5830413G11Rik	74730	5	64765682	64767115	+
18772	1431011_at	Region	Dlst	78920	12	81979590	82002780	+
18773	1456195_x_at	Region	Itgb5	16419	16	32637841	32757227	+
18774	1428898_at	Region	Mon1a	72825	9	107956169	107971112	+
18775	1440858_at	Region	Crk7	69131	11	98024401	98067791	+
18776	1439063_at	Region	Asxl2	75302	12	1933	29919	+
18777	1443285_at	Region	Gria4	14802	9	4328268	4706535	-
18778	1418730_at	Region	Rnf12	19820	X	98558532	98582649	-
18779	1448509_at	Region	3110001A13Rik	66540	2	3630729	3699405	+
18780	1453349_at	Region	Casz1	69743	4	147393514	147437492	+
18781	1415913_at	Region	Rps13	68052	7	110187198	110189820	-
18782	1423111_at	Region	Atp5a1	11946	18	77895834	77904683	+
18783	1421664_a_at	Region	Styx	56291	14	63481864	63493914	+
18784	1429462_at	Region	MGI:1917156	69906	15	38993909	39012345	-
18785	1448652_at	Region	Ttc10	21821	14	51955604	52049413	+
18786	1437935_at	Region	4930486G11Rik	75033	9	82188987	82352734	+
18787	1449516_a_at	Region	Rgs3	50780	4	61651038	61793526	+
18788	1457545_at	Region	None	None	NONE	NONE	NONE	NONE
18789	1416979_at	Region	2510048O06Rik	66537	5	146752123	146767273	+
18790	1418798_s_at	Region	Stk23	56504	X	68435077	68439580	+
18791	1439804_at	Region	2310015A10Rik	69548	12	76981780	76982965	-
18792	1420909_at	Region	Vegfa	22339	17	43526914	43541671	-
18793	1456638_at	Region	Wdr59	319481	8	110747797	110819576	-
18794	1416458_at	Region	Arf2	11841	11	103787960	103806425	+
18795	1442744_at	Region	C79248	96982	2	155626396	155626710	-
18796	1416466_at	Region	Vapa	30960	17	63289439	63322939	-
18797	1423292_a_at	Region	Prx	19153	7	22875327	22896473	+
18798	1419434_at	Region	Slc2a10	170441	2	164960708	164976644	+
18799	1454206_a_at	Region	Adam15	11490	3	89096372	89106699	-
18800	1454975_at	Region	BC033596	216345	10	115072590	115119560	+
18801	1437391_x_at	Region	Mrpl44	69163	1	80106651	80112515	+
18802	1429756_at	Region	4931428F04Rik	74356	8	104576037	104584678	-
18803	1434417_at	Region	None	None	17	23751729	23752579	-

18804	1452909_at	Region	2410127E18Rik	76788	6	30449002	30501701	+
18805	1445718_at	Region	Wdfy3	72145	5	100857137	101091457	-
18806	1438229_at	Region	Pggt1b	225467	18	46460574	46501475	-
18807	1431424_at	Region	2810055G20Rik	77994	16	12004013	12372207	+
18808	1429235_at	Region	Galntl2	78754	14	30160461	30194395	+
18809	1418490_at	Region	Sdsl	257635	5	119610009	119624570	-
18810	1445280_at	Region	Ap3d1	11776	10	80838692	80873927	-
18811	1452340_at	Region	6820424L24Rik	100515	5	37416340	37432643	-
18812	1427326_at	Region	4732471D19Rik	319719	13	53135721	53161052	+
18813	1445558_at	Region	None	None	NONE	NONE	NONE	NONE
18814	1441458_at	Region	Rasa4	54153	5	135097409	135125352	+
18815	1423963_at	Region	Wdr26	226757	1	181127684	181165201	-
18816	1455207_at	Region	LOC544825	544825	12	3393894	3397922	+
18817	1429719_at	Region	Foxp4	74123	17	45386201	45444354	-
18818	1421451_at	Region	Crb1	170788	1	139045517	139224215	-
18819	1437539_at	Region	Prkaa1	105787	15	4964811	4967465	+
18820	1427966_at	Region	BC087945	432940	15	27595458	27609229	-
18821	1427466_at	Region	Cdc91l1	228812	2	154735189	154814361	-
18822	1427970_at	Region	Zfp689	71131	7	121491869	121498852	-
18823	1447294_at	Region	Fbxw11	103583	11	32537669	32641610	+
18824	1415865_s_at	Region	Bpgm	12183	6	34570624	34599878	+
18825	1436554_at	Region	Zfpn1a5	67143	7	125704215	125726182	-
18826	1433723_s_at	Region	Serf2	378702	2	120963266	120964906	+
18827	1440893_at	Region	None	None	13	37596012	37597959	+
18828	1440971_x_at	Region	G630024C07Rik	244216	7	121294257	121304532	+
18829	1459575_at	Region	D1Ertd704e	51929	1	179355078	179355589	-
18830	1431508_at	Region	4930423M02Rik	73872	4	5560260	5569431	+
18831	1438166_x_at	Region	Ndufs4	17993	13	110702711	110802452	-
18832	1426055_a_at	Region	Pigq	14755	17	3046	4385	-
18833	1426382_at	Region	Ppm1b	19043	17	82787863	82846969	+
18834	1417856_at	Region	Relb	19698	7	16475466	16498682	-
18835	1446079_at	Region	Zmat2	66492	18	37017688	37023425	+
18836	1424982_a_at	Region	2700078E11Rik	78832	19	60127914	60181028	-
18837	1451246_s_at	Region	Aurkb	20877	11	68771301	68777320	+
18838	1460743_at	Region	Tigd5	105734	15	75959829	75964603	+
18839	1432394_a_at	Region	Aatf	56321	11	84150848	84241493	-
18840	1451773_s_at	Region	Polr3f	70408	2	143984692	143996204	+
18841	1457664_x_at	Region	C2	12263	17	32565421	32584866	-
18842	1433429_at	Region	Pigs	276846	11	78054081	78068435	+
18843	1430745_at	Region	5930409G06Rik	77066	3	44732166	44733710	-
18844	1416151_at	Region	Sfrs3	20383	17	26839644	26846716	+
18845	1422705_at	Region	Tmepai	65112	2	172688256	172737000	-
18846	1439150_x_at	Region	Grtp1	66790	8	12555505	12579170	-
18847	1428271_at	Region	Acbd4	67131	11	102922863	102933289	+
18848	1451024_at	Region	Edg6	13611	10	81633943	81635602	-
18849	1452810_at	Region	4921521J11Rik	70885	8	67943264	67976954	+
18850	1425267_a_at	Region	3110045G13Rik	73182	3	87494202	87514047	-
18851	1424499_s_at	Region	5730596K20Rik	109168	19	7208422	7249028	+
18852	1430619_a_at	Region	Mvk	17855	5	113554875	113571160	+
18853	1460370_at	Region	Top1mt	72960	15	75707102	75728859	-
18854	1424716_at	Region	0610039N19Rik	67442	6	72932145	72940919	+
18855	1440881_at	Region	Brwd3	382236	X	103336563	103435714	-
18856	1452432_at	Region	Tfpi	21788	2	84130609	84171923	-
18857	1443201_at	Region	Gpc6	23888	14	111487529	112531234	+
18858	1459208_at	Region	AU021889	105972	15	39577818	39578181	-
18859	1431002_x_at	Region	Fahd2a	68126	2	126950068	126958394	-
18860	1440814_x_at	Region	Hs3st2	195646	7	115360837	115377332	+
18861	1452443_s_at	Region	Helz	78455	11	107369049	107508032	+
18862	1425398_at	Region	Hist1h2bc	68024	13	23164082	23172357	+
18863	1420119_s_at	Region	AU020177	98560	1	31101687	31102046	-
18864	1416334_at	Region	Wwox	80707	8	113753199	114666211	+
18865	1425903_at	Region	Sema6a	20358	18	47470645	47528251	-
18866	1452615_s_at	Region	Tpt1h	107328	19	6710181	6712894	+
18867	1454075_s_at	Region	Nudt13	67725	14	18656034	18678071	+
18868	1417922_at	Region	Kbtbd4	67136	2	90609535	90615309	+

18869	1447000_at	Region	None	None	11	119750353	119750873	-
18870	1447847_x_at	Region	None	None	3	53142481	53142715	+
18871	1420340_at	Region	4930413O22Rik	67986	1	10144887	10173449	+
18872	1450569_a_at	Region	Rbm14	56275	19	4589715	4600375	-
18873	1450898_at	Region	Hiat1	15247	3	115400100	115431486	-
18874	1416053_at	Region	Lrrn1	16979	6	108031527	108072471	+
18875	1440816_x_at	Region	Ddx1	104721	12	12580692	12609750	-
18876	1440417_at	Region	D19Ertd409e	52016	19	45318129	45318715	-
18877	1434477_at	Region	Heca	380629	10	17823383	17874863	-
18878	1434416_a_at	Region	None	None	17	23751729	23752579	-
18879	1444303_at	Region	None	None	19	7011503	7018205	+
18880	1445509_at	Region	Atf7	223922	15	102597062	102685840	-
18881	1435073_a_at	Region	4930438O05Rik	78795	1	85961103	86084595	+
18882	1423043_s_at	Region	Ddx3x	13205	X	11519978	11531554	+
18883	1450719_at	Region	Mep1a	17287	17	40976564	41005347	-
18884	1443113_at	Region	Jmjd2b	193796	17	53963133	54039904	+
18885	1416476_a_at	Region	Ube2d2	56550	18	35995272	36030828	+
18886	1446929_at	Region	C76824	97128	4	32524734	32525270	+
18887	1439950_at	Region	Dnchc1	13424	12	106076688	106142158	+
18888	1452320_at	Region	LOC545422	545422	2	69279737	69361316	-
18889	1455884_at	Region	Dpp9	224897	17	53823723	53855877	-
18890	1425147_at	Region	2410075B13Rik	223648	15	76030756	76031978	-
18891	1455552_at	Region	Snapc4	227644	2	26294936	26312781	-
18892	1431792_a_at	Region	Stk11ip	71728	1	75812269	75828058	+
18893	1436460_at	Region	BC030440	210035	10	127413775	127431878	+
18894	1443148_at	Region	Hip2	53323	5	64339100	64400757	+
18895	1416465_a_at	Region	Vapa	30960	17	63289439	63322939	-
18896	1431453_at	Region	4933406K04Rik	71033	12	102156850	102167077	-
18897	1428540_at	Region	3321401G04Rik	77574	6	42816018	42836530	-
18898	1429466_s_at	Region	Aph1c /// 2310057K14Rik /// Aph1b	208117	9	66900639	66920575	-
18899	1456868_at	Region	2900073G15Rik	67268	17	68765368	68773934	-
18900	1419492_s_at	Region	Defb1	13214	8	20528771	20547357	+
18901	1448497_at	Region	Ercc3	13872	18	32480475	32510291	+
18902	1440040_at	Region	Cdk6	12571	5	3350317	3528230	+
18903	1448145_at	Region	Wwp2	66894	8	106734091	106856288	+
18904	1441764_at	Region	Prdm10	382066	9	31203629	31300995	+
18905	1424783_a_at	Region	Ugt1a2 /// Ugt1a6a /// Ugt1a10 /// Ugt1a7c /// Ugt1a5 /// Ugt1a9 /// Ugt1a6b /// Ugt1a1	22236	1	87889884	88038101	+
18906	1420612_s_at	Region	Ptp4a2	19244	4	128866586	128877088	+
18907	1430460_at	Region	5830410F13Rik	74745	2	12280342	12282388	-
18908	1458406_at	Region	AI429294	106580	17	44879273	44880181	+
18909	1448761_a_at	Region	Copg2	54160	6	30849464	30936789	-
18910	1443935_at	Region	BC032203	210982	17	44309813	44343803	-
18911	1450767_at	Region	Nedd9	18003	13	40868522	41047402	-
18912	1442298_at	Region	Bicc1	83675	10	70979053	71213601	-
18913	1442471_at	Region	Ephb2	13844	4	135534865	135717192	-
18914	1446855_at	Region	---	433781	4	144091609	144110244	-
18915	1436904_at	Region	Thrap1	327987	11	85997437	86085517	-
18916	1457276_at	Region	Snf1lk2	235344	9	50965127	51081399	-
18917	1448709_at	Region	Arid1a	93760	4	132639909	132683944	-
18918	1438129_at	Region	Wsb2	59043	5	116486085	116507368	+
18919	1439897_at	Region	None	None	2	17417029	17432993	-
18920	1439224_at	Region	Ankib1	70797	5	3785355	3786027	-
18921	1457489_at	Region	Eif4e	13684	3	137416519	137446834	+
18922	1439993_at	Region	1200013F24Rik	66880	3	66658030	67030708	+
18923	1441993_at	Region	Ap3s2	11778	7	73672386	73717617	-
18924	1444056_at	Region	2900006B13Rik	72947	11	51337644	51355878	+
18925	1437986_x_at	Region	Fuk	234730	8	110180185	110200181	-
18926	1424115_at	Region	Ppp5c	19060	7	13955563	13978781	-
18927	1447272_s_at	Region	Atp10a	11982	7	52936576	53107035	+

18928	1446228_at	Region	D19Wsu12e	226090	19	28862793	28936642	-
18929	1436367_at	Region	None	None	10	116077690	116078996	+
18930	1426455_at	Region	Sdccag10	67285	13	100849082	101034691	-
18931	1415983_at	Region	Lcp1	18826	14	69480274	69574658	+
18932	1416295_a_at	Region	Il2rg	16186	X	95865748	95869567	-
18933	1448456_at	Region	Cln8	26889	8	14267371	14278844	+
18934	1456670_at	Region	A930007A09Rik /// LOC436338	432999	16	12491310	12495281	+
18935	1440535_at	Region	AI415298	101555	7	128059787	128060309	-
18936	1435535_at	Region	Depdc5	277854	5	31352231	31444754	+
18937	1442703_at	Region	AK220484	381157	18	10495274	10605827	+
18938	1427633_a_at	Region	Pappa	18491	4	64215365	64448700	+
18939	1450543_at	Region	Myo1h	231646	5	113467384	113475145	+
18940	1456460_at	Region	BC068281	238037	12	4015662	4024850	+
18941	1445206_at	Region	Zw10	26951	9	49086054	49109318	+
18942	1450934_at	Region	Eif4a2	13682	16	21890925	21897116	+
18943	1433527_at	Region	LOC546132	546132	9	54982178	55030883	+
18944	1443187_at	Region	Thsd2	72780	10	29483782	29567151	-
18945	1442528_at	Region	Lats2 /// Xpo4	50523	14	52225857	52269062	-
18946	1439488_at	Region	None	None	10	80926387	80927401	+
18947	1431630_a_at	Region	Klf3	16599	5	63601098	63627704	+
18948	1451110_at	Region	Egln1	112405	8	124195909	124203103	-
18949	1434650_at	Region	Pogz	229584	3	94341777	94368636	+
18950	1454725_at	Region	G430041M01Rik	101214	6	49378306	49398393	-
18951	1443340_at	Region	Cbara1	216001	10	59664687	59819557	+
18952	1456646_at	Region	Gm142	230983	4	153535203	153564795	+
18953	1430859_at	Region	5330427O13Rik	78271	14	58677127	58679379	-
18954	1456816_at	Region	Rai17	328365	14	23834490	24039917	+
18955	1450178_at	Region	Brdt	114642	5	106401608	106456144	+
18956	1456996_at	Region	1190005F20Rik	98685	1	151285836	151286402	+
18957	1443123_at	Region	None	None	NONE	NONE	NONE	NONE
18958	1442465_s_at	Region	Strbp	20744	2	37415390	37579381	-
18959	1417202_s_at	Region	Ube1c	22200	6	97650448	97671630	-
18960	1443428_at	Region	None	None	NONE	NONE	NONE	NONE
18961	1415832_at	Region	Agtr2	11609	X	19723566	19728104	+
18962	1426041_a_at	Region	Fgd4	224014	16	15192773	15330937	-
18963	1436020_at	Region	D8Ertd457e	101994	8	13248344	13259184	+
18964	1424910_at	Region	Kif12	16552	4	62256828	62263322	-
18965	1440575_at	Region	Hspa4	15525	11	53014415	53053230	-
18966	1443557_at	Region	None	None	18	42797108	42797640	+
18967	1419037_at	Region	Csnk2a1	12995	2	151683883	151738783	+
18968	1447550_at	Region	None	None	5	105619367	105619692	-
18969	1430716_at	Region	5330421F07Rik	78286	7	43479111	43568752	+
18970	1437656_at	Region	9330140K16Rik	226970	1	35071064	35082096	+
18971	1432352_at	Region	5730405I09Rik	67974	18	9356614	9490220	-
18972	1418733_at	Region	Twist1	22160	12	30570330	30572490	+
18973	1447734_x_at	Region	Aldoa	11674	7	120844967	120848914	-
18974	1454120_a_at	Region	Pcgf6	71041	19	46584919	46602145	-
18975	1451418_a_at	Region	MGI:2183445	211949	9	96842840	96917713	-
18976	1442268_a_at	Region	None	None	18	68004512	68005173	-
18977	1435636_at	Region	2310051F07Rik	108745	15	1069902	1071573	+
18978	1417995_at	Region	Ptpn22	19260	3	103287683	103339638	+
18979	1445438_at	Region	Ddhd1	114874	14	40672980	40701448	-
18980	1436634_at	Region	Robo3	19649	9	37357703	37371673	-
18981	1448339_at	Region	Tmem30a	69981	9	79974649	79999130	-
18982	1442578_at	Region	None	None	17	54490134	54490519	-
18983	1448205_at	Region	Ccnb1-rs1 /// Ccnb1	12429	4	117900860	117902355	-
18984	1416255_at	Region	Gja4	14612	4	126338534	126341091	-
18985	1450024_at	Region	Sufu	24069	19	45944301	46033419	+
18986	1422968_at	Region	Ihpk1	27399	9	108070635	108116769	+
18987	1446090_at	Region	6530409C15Rik	76224	6	28262247	28265897	+
18988	1438497_at	Region	2810423E13Rik	72175	3	40237869	40269375	-
18989	1450448_at	Region	Stc1	20855	14	63559854	63572230	+
18990	1418394_a_at	Region	Cd97	26364	8	82983280	83001085	-

18991	1425891_a_at	Region	Grtp1	66790	8	12555505	12579170	-
18992	1450637_a_at	Region	Aebp1	11568	11	5756734	5766837	+
18993	1441748_at	Region	Zzz3	108946	3	151378383	151441249	+
18994	1451967_x_at	Region	Kpnb1	16211	11	96980801	97008707	-
18995	1415771_at	Region	Ncl	17975	1	86155962	86165711	-
18996	1454031_at	Region	2310029O18Rik	76946	4	41316504	41318485	+
18997	1429927_at	Region	5830409B07Rik	76020	4	131599980	131601170	+
18998	1432593_at	Region	4933428P19Rik	71229	10	62544079	62545398	-
18999	1449265_at	Region	Casp1	12362	9	5209682	5217161	+
19000	1460371_at	Region	Hspa12b	72630	2	130641265	130659838	+
19001	1456158_at	Region	None	None	4	146590799	146591631	-
19002	1418435_at	Region	Mkrn1	54484	6	39533206	39555816	-
19003	1436711_at	Region	Actr5	109275	2	158081901	158096167	+
19004	1416739_a_at	Region	Brap	72399	5	120815393	120837810	+
19005	1437410_at	Region	Aldh2	11669	5	120719784	120745669	-
19006	1431180_at	Region	1700123A16Rik	73610	NONE	NONE	NONE	NONE
19007	1426109_a_at	Region	Slc14a2	27411	18	78268796	78719616	-
19008	1451174_at	Region	Lrrc33	224109	16	30952196	30974847	-
19009	1441855_x_at	Region	Cxcl1	14825	5	90207981	90209786	+
19010	1451303_at	Region	BC002230	217827	12	95567102	95601742	-
19011	1459655_at	Region	None	None	16	90776954	90777241	-
19012	1452784_at	Region	Itgav	16410	2	83422264	83501556	+
19013	1443811_at	Region	Cstf2t	83410	19	30334913	30338502	+
19014	1435900_at	Region	Zfp297b	71834	2	33385684	33400667	-
19015	1429413_at	Region	Cpm	70574	10	117346301	117376298	+
19016	1440066_at	Region	Smarcad1	13990	6	65340694	65341270	+
19017	1425478_x_at	Region	Ube2i	22196	17	23066362	23080142	-
19018	1430438_at	Region	8430439J12Rik	71553	5	40545923	40547797	-
19019	1433462_a_at	Region	Pi4k2a	84095	19	41634859	41666403	+
19020	1460707_at	Region	Ptp4a2	19244	4	128866586	128877088	+
19021	1457268_at	Region	Dot1l	208266	10	80886923	80926284	+
19022	1457891_at	Region	C230011H18Rik	399590	2	6483408	6484140	-
19023	1434780_at	Region	C130038G02Rik	77521	5	147156816	147205340	+
19024	1434475_at	Region	Ppig	228005	2	69578493	69605782	+
19025	1416990_at	Region	Rxrb	20182	17	31736002	31742955	+
19026	1444884_at	Region	9530043G02Rik	109338	4	121883050	121883998	+
19027	1421301_at	Region	Zic2	22772	14	117045015	117049406	+
19028	1444143_at	Region	None	None	5	149520562	149521297	-
19029	1423335_at	Region	1110004F10Rik	56372	7	109948983	109960813	+
19030	1424417_at	Region	2210415M20Rik	70160	8	20945031	20970768	+
19031	1439449_at	Region	Satb1	20230	17	49274277	49367498	-
19032	1451494_at	Region	Wac	225131	18	7911809	7969644	+
19033	1457271_at	Region	Gm131	229697	3	107007124	107017561	-
19034	1458496_at	Region	Cyb561	13056	11	105754793	105765236	-
19035	1444563_at	Region	B4galt5	56336	2	166757794	166805916	-
19036	1420934_a_at	Region	Srrm1	51796	4	134203290	134234393	-
19037	1425225_at	Region	Fcgr3a	246256	1	170954190	170964923	+
19038	1439852_at	Region	None	None	4	83256428	83257125	-
19039	1430951_at	Region	2810011L19Rik	69952	12	100781129	100826247	+
19040	1423336_at	Region	Orc4l	26428	2	48835014	48881396	-
19041	1442122_at	Region	AI451458	102916	X	31537925	31538503	-
19042	1421800_at	Region	Phxr1	18686	2	3349136	3349656	+
19043	1447372_at	Region	Aldh16a1	69748	7	39218382	39231080	-
19044	1446526_at	Region	9330199F22Rik	105398	13	93701091	93701537	+
19045	1434313_at	Region	6330407D12Rik	57895	6	49455597	49479041	+
19046	1441400_at	Region	1810029B16Rik	66282	8	65585469	65595630	-
19047	1436193_at	Region	Man1c1	230815	4	133522593	133665193	-
19048	1418745_at	Region	Omd	27047	13	49182266	49192132	+
19049	1419816_s_at	Region	1300002F13Rik	74155	4	149347453	149361242	+
19050	1438863_at	Region	A530079E22Rik	319977	1	87321824	87323030	-
19051	1453524_at	Region	5530401D11Rik	71360	18	6276895	6278468	-
19052	1432356_at	Region	1700056N10Rik	73392	16	15818873	15820465	+
19053	1440886_at	Region	Cdc37l1	67072	19	28243091	28270166	+
19054	1434563_at	Region	Rps6kc1	320119	1	190532334	190648036	-
19055	1428189_at	Region	5730494M16Rik	66648	18	25367107	25397117	-

19056	1444764_at	Region	A130022J21Rik	320617	17	30924776	30925455	+
19057	1425983_x_at	Region	Hipk2	15258	6	38834084	39012133	-
19058	1437601_at	Region	Otx1	18423	11	21889558	21896408	-
19059	1423036_at	Region	Txnl5	52700	11	71933212	71936145	+
19060	1417314_at	Region	H2-Bf	14962	17	32559195	32565031	-
19061	1454562_at	Region	4930488N24Rik	75011	17	12152002	12195662	-
19062	1418334_at	Region	AA545217	27214	5	8403658	8429252	-
19063	1424927_at	Region	Glipr1	73690	10	111688463	111700201	-
19064	1427073_at	Region	Lace1	215951	10	42419265	42585094	-
19065	1432892_at	Region	5730564L20Rik	70651	12	69507237	69509467	-
19066	1439447_x_at	Region	Rpl37a	19981	1	73012971	73015366	+
19067	1437677_at	Region	AI449595	58901	6	115465848	115466746	-
19068	1446550_at	Region	Gspt1	14852	16	10388595	10422192	+
19069	1441029_at	Region	None	None	17	23980037	23980641	+
19070	1437032_x_at	Region	Rbm14	56275	19	4589715	4600375	-
19071	1458410_at	Region	Garnl1	56784	12	52323288	52540884	-
19072	1442059_at	Region	Fxr1h	14359	3	33467491	33516721	+
19073	1458529_at	Region	LOC215714	215714	1	135435245	135450422	+
19074	1420419_a_at	Region	Otof	83762	5	28825257	28920848	-
19075	1444743_at	Region	Oact2	67216	12	21381378	21511358	+
19076	1442341_at	Region	LOC545157	545157	16	33041252	33041602	-
19077	1429399_at	Region	Rnf125	67664	18	21159553	21198776	+
19078	1447744_s_at	Region	None	None	9	40293613	40293854	+
19079	1427116_at	Region	BC010250	233904	7	121846214	121849933	+
19080	1442618_at	Region	Ldh2	16832	6	143333518	143351212	-
19081	1440013_at	Region	Trim44	80985	2	102009026	102105530	-
19082	1460297_at	Region	Nphs2	170484	1	156209762	156226998	+
19083	1449115_at	Region	Mtf2	17765	5	107157468	107177436	+
19084	1443316_at	Region	None	None	9	61064691	61065335	+
19085	1438063_at	Region	Mphosph9	269702	5	123422304	123499213	-
19086	1449444_a_at	Region	Mfap1	67532	2	121007192	121020508	-
19087	1442100_at	Region	Inpp5f	101490	7	122660050	122745896	+
19088	1418200_at	Region	Hkr3	100090	4	150512136	150520020	-
19089	1415746_at	Region	Cic	71722	7	20463051	20474387	+
19090	1420910_at	Region	Ppap2c	50784	10	79648047	79654486	-
19091	1428197_at	Region	Tspan9	109246	6	128652920	128772541	-
19092	1458615_at	Region	Depdc5	277854	5	31352231	31444754	+
19093	1457176_at	Region	Garnl1	56784	12	52323288	52540884	-
19094	1437747_at	Region	Ube4a	140630	9	44921926	44954380	-
19095	1455193_at	Region	Zbtb8	215627	4	128453383	128460424	-
19096	1441399_at	Region	4933400E14Rik	71007	7	128633927	128709638	-
19097	1454903_at	Region	None	None	11	95389915	95390859	-
19098	1431000_at	Region	2310002B06Rik	53951	17	76657865	76667596	+
19099	1438884_at	Region	D830007B15Rik	330096	5	66374549	66380118	+
19100	1416680_at	Region	Ube3a	22215	7	53507062	53583196	+
19101	1428800_a_at	Region	3000003F02Rik	78895	15	94591343	94612193	-
19102	1428100_at	Region	Sfrs1	110809	11	87775771	87779641	+
19103	1460467_at	Region	Iqcg	69707	16	31823437	31864222	-
19104	1444305_at	Region	LOC381400	381400	2	155085997	155091244	+
19105	1430092_at	Region	Serac1	321007	17	5950429	5986100	-
19106	1415726_at	Region	Ankrd17	81702	5	89524594	89663613	-
19107	1430217_at	Region	4921528H16Rik	74354	6	34120464	34227989	+
19108	1453400_at	Region	4933428D01Rik	71200	14	39195997	39216086	-
19109	1443398_at	Region	Snap25	20614	2	136289818	136290331	+
19110	1442905_at	Region	None	None	14	24754304	24754736	+
19111	1431043_at	Region	Kbtbd5	72330	9	121792117	121793371	+
19112	1445186_at	Region	Stc2	20856	11	31254231	31264851	-
19113	1444478_at	Region	2900057D21Rik	72993	14	25057193	25108756	-
19114	1420727_a_at	Region	Tmlhe	192289	X	1320	3062	+
19115	1433487_at	Region	Clcn3	12725	8	59960629	60030782	-
19116	1443248_at	Region	B230339H12Rik	234076	8	12666914	12700030	+
19117	1429245_at	Region	2510022D24Rik	66567	NONE	NONE	NONE	NONE
19118	1443626_at	Region	None	None	6	54408285	54408751	+
19119	1430042_at	Region	A930012M21Rik	77954	7	119761889	119763594	+
19120	1438424_at	Region	C530046L02Rik	224617	17	21982357	22009100	-

19121	1430571_s_at	Region	Armc6	76813	8	69373170	69384382	-
19122	1445768_at	Region	LOC544965	544965	13	92978853	93024229	+
19123	1440977_at	Region	Akap13	233400	7	69488734	69518761	+
19124	1424532_at	Region	Ylpm1	56531	12	81865083	81939186	+
19125	1449097_at	Region	Txnrd2	26462	16	17197467	17250120	+
19126	1440870_at	Region	Prdm16	70673	4	152812654	153021293	-
19127	1458509_at	Region	Mre11a	17535	9	14618147	14667859	+
19128	1440141_at	Region	Kcnq2	16536	2	180792537	180852183	-
19129	1441232_at	Region	None	None	6	29069779	29070415	+
19130	1417003_at	Region	0610012G03Rik	106264	16	30756422	30757865	-
19131	1426779_x_at	Region	Dag1	13138	9	108271876	108333355	-
19132	1443603_at	Region	A030012M09Rik	319263	1	7090301	7142844	+
19133	1421135_a_at	Region	Cnot8	69125	11	57829782	57844223	+
19134	1441775_at	Region	Wdr7	104082	18	63940854	64221039	+
19135	1424217_at	Region	Papola	18789	12	101247264	101284473	+
19136	1457580_at	Region	Chd8	67772	14	47298256	47322726	-
19137	1445559_at	Region	None	None	NONE	NONE	NONE	NONE
19138	1419963_at	Region	Depdc6	97998	15	55114270	55254734	+
19139	1431520_at	Region	4933406J09Rik	74064	6	135065548	135160020	-
19140	1455099_at	Region	Mogat2	233549	7	93310449	93329968	-
19141	1459274_at	Region	None	None	12	68907075	68907754	-
19142	1446221_at	Region	None	None	NONE	NONE	NONE	NONE
19143	1418722_at	Region	Ngp	18054	9	110461384	110464545	+
19144	1437473_at	Region	Maf	17132	8	115016686	115019625	-
19145	1451818_at	Region	Mib1	225164	18	10770969	10857561	+
19146	1452044_at	Region	Arpc5l	74192	2	38940301	38948034	+
19147	1456370_s_at	Region	0610037L13Rik	74098	4	106848804	106856707	+
19148	1452617_at	Region	Ssbp1	381760	6	40612256	40622665	+
19149	1445662_x_at	Region	---	385300	NONE	NONE	NONE	NONE
19150	1417881_at	Region	Slc39a3	106947	10	81162385	81167757	-
19151	1458383_at	Region	Idh2	269951	7	73893703	73914172	-
19152	1444344_at	Region	None	None	13	9683953	9684900	+
19153	1417450_a_at	Region	Tacc3	21335	5	32147518	32160849	+
19154	1419624_a_at	Region	1700010I14Rik	66931	17	7625204	7645159	+
19155	1429304_at	Region	Ankrd10	102334	8	10984009	11008115	-
19156	1454601_at	Region	8030497O21Rik	77506	NONE	NONE	NONE	NONE
19157	1444465_at	Region	Ube4b	63958	4	147820778	147919111	-
19158	1456313_x_at	Region	Mrpl28	68611	17	23916702	23919783	+
19159	1423838_s_at	Region	2400003C14Rik	71955	8	108969005	108990940	-
19160	1421389_a_at	Region	Eif2ak4	27103	2	117902466	117989082	+
19161	1432554_at	Region	2310040G07Rik	70284	5	76172827	76173305	+
19162	1458809_at	Region	None	None	NONE	NONE	NONE	NONE
19163	1436211_at	Region	Thoc4	21681	11	120415609	120419458	-
19164	1436704_x_at	Region	Mthfd1	108156	12	73111496	73175891	+
19165	1442372_at	Region	None	None	17	73342445	73343167	-
19166	1460523_at	Region	None	None	13	24151993	24152957	+
19167	1430264_at	Region	2610030P05Rik	69258	2	38150530	38152000	-
19168	1444259_at	Region	AW495222	105364	13	89890163	89903238	-
19169	1434391_at	Region	Hnrpu	51810	1	178283700	178291584	-
19170	1447608_x_at	Region	Btbd14a	67991	2	25991155	26054942	-
19171	1452422_a_at	Region	Snrpb2	20639	2	142525198	142528988	+
19172	1447940_a_at	Region	Braf	109880	6	39749542	39860924	-
19173	1441206_at	Region	Synpo2	118449	3	121904047	121951604	-
19174	1438408_at	Region	5730467H21Rik	78088	5	92376921	92380701	-
19175	1437818_at	Region	9430016H08Rik	68115	1	57710278	57721683	+
19176	1439212_at	Region	6820429M01	233905	7	121927511	121935806	+
19177	1424705_at	Region	Rbmx2	209003	X	43215800	43231515	+
19178	1455853_x_at	Region	Sas	67125	10	126800009	126802980	-
19179	1434479_at	Region	AI413331	98940	2	27970650	27971677	+
19180	1441411_at	Region	Lims1	110829	10	58281059	58380568	+
19181	1429808_at	Region	1110020C03Rik	68625	4	117513456	117528352	-
19182	1442774_x_at	Region	None	None	NONE	NONE	NONE	NONE
19183	1436490_x_at	Region	Ran	19384	5	128195181	128199286	+
19184	1442558_at	Region	None	None	12	112907529	112914136	-
19185	1428950_s_at	Region	Nol8	70930	13	49249941	49275842	+

19186	1440634_at	Region	Gpc6	23888	14	111487529	112531234	+
19187	1418747_at	Region	Sfpi1	20375	2	90801546	90820505	+
19188	1452319_at	Region	Zfp82	330502	7	25473958	25487536	-
19189	1424868_at	Region	Glyat	107146	19	11829393	11847822	+
19190	1456976_at	Region	Wnt5a	22418	14	26642805	26662537	+
19191	1430604_a_at	Region	Dab2	13132	15	6175647	6229535	+
19192	1447280_at	Region	Dtna	13527	18	23643946	23881842	+
19193	1420333_at	Region	Txndc8	67402	4	57927478	57952573	-
19194	1458293_at	Region	Hmox2	15369	16	4413295	4438825	+
19195	1443411_at	Region	1700030K01Rik	75571	13	72024848	72056077	+
19196	1436284_s_at	Region	None	None	8	94607184	94607824	-
19197	1422595_s_at	Region	5730470L24Rik	66641	3	102423707	102431881	+
19198	1430063_at	Region	4930571K23Rik	75861	7	119419569	119420745	+
19199	1417264_at	Region	D5Ertd33e	52064	5	114389328	114406598	+
19200	1457770_at	Region	Slc39a14	213053	14	64619456	64667414	-
19201	1443877_a_at	Region	Rapgef6	192786	11	54384103	54395691	+
19202	1430404_at	Region	4833416J08Rik	75771	7	17482750	17484811	-
19203	1436091_at	Region	2810022L02Rik	67198	1	58079733	58253199	+
19204	1427368_x_at	Region	Fes	14159	7	74178215	74187851	-
19205	1443408_at	Region	Plk1	18817	7	116037351	116047721	+
19206	1458900_at	Region	None	None	2	144669552	144670182	+
19207	1447555_at	Region	Utrn	22288	10	12263109	12646998	-
19208	1421706_at	Region	Mmp20	30800	9	7622916	7669655	+
19209	1417350_at	Region	Pldn	18457	2	122252366	122261952	+
19210	1430965_at	Region	9430064K01Rik	77294	10	69744425	69745520	+
19211	1445398_at	Region	3732412D22Rik	77569	5	65624008	65822238	+
19212	1440324_at	Region	Mrpl19	56284	6	82245436	82301881	-
19213	1432086_a_at	Region	Ribc2	67747	15	85180677	85193168	+
19214	1439954_at	Region	6430514M23Rik	399595	1	132063053	132063579	+
19215	1430415_at	Region	Phf6	70998	X	47433062	47477739	+
19216	1437847_x_at	Region	Rbmx	19655	X	52141072	52147740	-
19217	1446278_at	Region	Utx	22289	X	16401607	16518298	+
19218	1441617_at	Region	Pcqap	94112	16	16422190	16493791	-
19219	1456169_at	Region	LOC226654	226654	1	171347872	171349193	+
19220	1440288_at	Region	LOC545719	545719	4	146732892	146780309	-
19221	1419086_at	Region	Fgfbp1	14181	5	42735916	42738818	-
19222	1459659_at	Region	Zfp131	72465	13	10118	57012	+
19223	1438474_at	Region	Ankrd35	213121	3	96157967	96178304	+
19224	1453336_at	Region	3830405G04Rik	70681	5	99825455	99841588	-
19225	1428999_at	Region	Phf3	213109	1	31075361	31146157	-
19226	1429797_at	Region	5730596K20Rik	109168	19	7208422	7249028	+
19227	1444005_at	Region	None	None	2	33704058	33704844	-
19228	1440495_at	Region	Dmt2	110333	10	92050243	92051272	-
19229	1448539_a_at	Region	Acy3	71670	19	3775451	3778797	+
19230	1440178_x_at	Region	Zap70	22637	1	37056452	37077407	+
19231	1437157_at	Region	None	None	1	164371337	164376031	+
19232	1455155_at	Region	BC040823	241846	2	179743356	179751614	+
19233	1455777_x_at	Region	Hsd17b4	15488	18	50343439	50411362	+
19234	1439600_at	Region	BC004012	192185	4	154054766	154083359	+
19235	1435169_at	Region	A930001N09Rik	77128	17	24510472	24568084	+
19236	1448969_at	Region	Ftsj2	68017	5	139324014	139328238	-
19237	1428861_at	Region	4631422O05Rik	78749	16	56262180	56481521	+
19238	1422706_at	Region	Tmepai	65112	2	172688256	172737000	-
19239	1445868_at	Region	Cpeb3	208922	19	36366605	36550135	-
19240	1421893_a_at	Region	Tpp2	22019	1	44229826	44298790	+
19241	1441159_at	Region	Med12l	329650	3	58907051	58912332	+
19242	1420737_at	Region	Pmfbp1	56523	8	108791703	108840316	+
19243	1449282_at	Region	Cysltr1	58861	X	101177882	101204827	-
19244	1427516_a_at	Region	Boc	117606	16	43368375	43441912	-
19245	1453951_a_at	Region	4930463G05Rik	70806	19	47337880	47388682	-
19246	1443250_at	Region	None	None	1	143831400	143832062	-
19247	1422976_x_at	Region	Ndufa7	66416	17	31523009	31536811	+
19248	1423616_at	Region	Tarbp2	21357	15	102578878	102584093	+
19249	1436851_at	Region	Pkn1	320795	8	82930012	82959042	-
19250	1450720_at	Region	Acp1	11431	12	27469324	27487670	-

19251	1418061_at	Region	Ltbp2	16997	12	81651887	81745207	-
19252	1424339_at	Region	Oasl1	231655	5	114032866	114047537	+
19253	1438731_at	Region	None	None	11	119164523	119165402	-
19254	1428492_at	Region	Glipr2	384009	4	43873624	43895040	+
19255	1430665_at	Region	5730480H06Rik	70592	5	47159396	47178118	+
19256	1446625_at	Region	1810057C19Rik	67888	11	89851435	89857592	+
19257	1455359_at	Region	C130080N23Rik	226829	1	189588471	189591536	+
19258	1456319_at	Region	X83313	13957	NONE	NONE	NONE	NONE
19259	1427462_at	Region	E2f3	13557	13	29386198	29468641	-
19260	1453780_at	Region	None	None	13	113671004	113673398	-
19261	1437399_at	Region	1110019C08Rik	224250	16	57715966	57722159	+
19262	1456539_at	Region	None	None	NONE	NONE	NONE	NONE
19263	1447874_x_at	Region	Smpd1	20597	7	99666853	99670820	+
19264	1436236_x_at	Region	Cotl1	72042	8	119165392	119197206	-
19265	1418620_at	Region	Phox2a	11859	7	95924390	95928627	+
19266	1431596_at	Region	None	None	9	83475771	83477134	+
19267	1421119_at	Region	Kif21b	16565	1	135978315	136024892	+
19268	1421710_at	Region	Zfp92	22754	X	68071751	68084810	+
19269	1441164_at	Region	None	None	18	39289221	39289982	+
19270	1419029_at	Region	Ero1l	50527	14	950	11169	+
19271	1442057_at	Region	AA986695	98247	1	86041449	86041741	+
19272	1451211_a_at	Region	Lgtn	16865	1	131002708	131021706	+
19273	1434942_at	Region	2610101J03Rik	66580	2	139633736	139684417	-
19274	1422988_at	Region	Sgsh	27029	11	119164585	119176595	-
19275	1438697_at	Region	4632425D07Rik	208213	5	126411362	126733552	+
19276	1419567_at	Region	Fank1	66930	7	128144543	128249184	+
19277	1429236_at	Region	Galntl2	78754	14	30160461	30194395	+
19278	1423656_x_at	Region	1500010J02Rik	68964	11	68751840	68762132	+
19279	1430247_at	Region	Daam2	76441	17	46989103	47096816	-
19280	1425725_s_at	Region	Ppp2r5c	26931	12	105961123	106055786	+
19281	1440267_at	Region	E330005K07Rik	320623	5	3623342	3624129	+
19282	1437425_at	Region	Gdap1	14545	1	17329856	17348730	+
19283	1433942_at	Region	Myo6	17920	9	80370873	80516811	+
19284	1459171_at	Region	None	None	6	136125659	136126364	+
19285	1452586_at	Region	Anapc13	69010	9	102575670	102583591	+
19286	1450086_at	Region	Gmeb1	56809	4	131021904	131048737	-
19287	1454383_at	Region	5430434G16Rik	71385	15	36252623	36253976	+
19288	1418658_at	Region	2410005O16Rik	66302	4	19502173	19533898	+
19289	1439535_at	Region	Dstn	56431	2	143372547	143400260	+
19290	1433661_at	Region	BC034204	270151	9	44241572	44257253	-
19291	1455817_x_at	Region	None	None	NONE	NONE	NONE	NONE
19292	1440931_at	Region	LOC545474	545474	2	151550295	151552735	+
19293	1449633_s_at	Region	C330027I04Rik	68106	11	100243408	100262161	-
19294	1457376_at	Region	Itga4	16401	2	78953394	79028523	+
19295	1441701_at	Region	Zfp148	22661	16	32188918	32308456	+
19296	1424219_at	Region	A530065E19Rik	217038	11	84541053	84547507	-
19297	1450308_a_at	Region	Xrn1	24127	9	95858413	95956167	+
19298	1453778_at	Region	2810407C02Rik	69227	3	58220979	58236191	+
19299	1428049_a_at	Region	Nudt16l1	66911	16	4609000	4610909	+
19300	1425100_a_at	Region	Pde6g	18588	11	120268703	120274576	-
19301	1454397_at	Region	4632418H02Rik	78265	9	122147976	122150177	+
19302	1423769_at	Region	Ptcd2	68927	13	95508567	95533596	-
19303	1427081_at	Region	A630072M18Rik	320770	5	19403559	19405868	+
19304	1431601_at	Region	LOC547315	547315	10	51562	55838	-
19305	1422088_at	Region	Lmyc1	16918	4	122023211	122029597	+
19306	1458729_at	Region	Fkbp4	14228	6	129120294	129128818	-
19307	1453239_a_at	Region	Ankrd22	52024	19	33450964	33497153	-
19308	1440810_x_at	Region	LOC433524	433524	2	175111912	175117397	-
19309	1441482_at	Region	None	None	11	110220839	110221524	+
19310	1459705_at	Region	Olfm3	229759	3	113850919	113895474	+
19311	1445918_at	Region	Tmem2	83921	19	21045338	21094911	+
19312	1444554_at	Region	None	None	15	80964789	80965321	+
19313	1424684_at	Region	Rab5c	19345	11	100536098	100559277	-
19314	1457214_at	Region	None	None	2	165459192	165459675	+
19315	1416045_a_at	Region	Smarcb1	20587	10	75998515	76023328	-

19316	1416364_at	Region	Hspcb	15516	17	43078708	43084593	-
19317	1438122_at	Region	None	None	12	81296762	81302137	+
19318	1420690_at	Region	Fgf10	14165	13	7290	84381	+
19319	1434950_a_at	Region	Armc8	74125	9	99381642	99469875	-
19320	1443457_at	Region	A230055J12Rik	320314	5	80771250	80771843	+
19321	1460096_at	Region	Lamp1	16783	8	12537914	12553973	+
19322	1457627_x_at	Region	Ropn1l	252967	15	31441819	31454253	-
19323	1441203_at	Region	None	None	2	158055142	158055821	-
19324	1459301_at	Region	Mrg1	17536	2	115376917	115578960	-
19325	1416288_at	Region	Dnaja1	15502	4	40861384	40873815	+
19326	1445892_at	Region	Per2	18627	1	91239024	91282480	-
19327	1436843_at	Region	C430048L16Rik	77604	4	12022506	12023317	+
19328	1435955_at	Region	Siglec10	243958	7	37483033	37493112	+
19329	1436687_x_at	Region	3110006P09Rik	68036	15	36999940	37010315	-
19330	1441534_at	Region	C86753	97833	12	95398236	95398854	-
19331	1419340_at	Region	Mov10l1	83456	15	89037487	89107077	+
19332	1442477_at	Region	None	None	2	91615049	91615930	+
19333	1420160_s_at	Region	Myo1e	71602	9	70344868	70497375	+
19334	1444038_at	Region	AU015836	385493	X	88629353	88636128	+
19335	1425205_at	Region	Ddx19b	234733	8	110304375	110325446	-
19336	1447486_at	Region	Ppp1r9b	217124	11	94812299	94827971	+
19337	1440529_at	Region	None	None	2	178709014	178727397	+
19338	1440745_at	Region	C130091E20	330006	4	152810842	152811305	-
19339	1456963_at	Region	1110039B18Rik	68796	5	29328591	29336411	+
19340	1445090_at	Region	None	None	10	49330125	49330696	-
19341	1458292_at	Region	Psma1	26440	7	108067002	108132938	-
19342	1421980_at	Region	Kcnc3	16504	7	38665933	38671377	+
19343	1447631_at	Region	Myst2	217127	11	95095346	95131288	-
19344	1423019_at	Region	Gja9	14617	2	113523441	113527459	-
19345	1436130_s_at	Region	4930523C11Rik /// Adam6	238405	12	108966267	108968537	+
19346	1437633_at	Region	Ankrd11	77087	8	122264618	122423554	-
19347	1428023_at	Region	3110009E18Rik	73103	1	119908371	119945964	+
19348	1439989_at	Region	Tsc1	64930	2	28573403	28619515	+
19349	1457105_at	Region	Pkd2l1	329064	19	43692043	43736814	-
19350	1427214_at	Region	Agmat	75986	4	140628016	140640523	+
19351	1432882_at	Region	4932431P20Rik	114675	7	24939867	24952715	+
19352	1424997_at	Region	Sfrs8	231769	5	128676882	128691989	+
19353	1418802_at	Region	R74862	97423	7	137446608	137466115	-
19354	1442129_at	Region	1810058I24Rik	67705	6	35357033	35357540	+
19355	1445789_at	Region	None	None	NONE	NONE	NONE	NONE
19356	1444642_at	Region	None	None	1	44041266	44042120	-
19357	1423981_x_at	Region	Slc25a29	214663	12	104296871	104306869	-
19358	1431138_at	Region	4930438O05Rik	78795	1	85961103	86084595	+
19359	1419293_at	Region	Dscam	13508	16	96075147	96653164	-
19360	1433170_at	Region	2900034C19Rik	72854	4	102670564	102671805	-
19361	1445414_at	Region	None	None	8	17786777	17787407	+
19362	1439281_at	Region	None	None	17	26439269	26439979	-
19363	1418305_s_at	Region	Nola1	68147	3	128757791	128764027	-
19364	1441128_at	Region	2310040A13Rik	69641	12	106273432	106274298	+
19365	1423681_at	Region	1300018I05Rik /// LOC445272	445272	17	27470394	27513279	+
19366	1430561_at	Region	Dnajb14	70604	3	136756714	136798772	+
19367	1432322_at	Region	2310016E02Rik	67695	5	29365462	29366669	-
19368	1431061_s_at	Region	Peli1	67245	11	21030262	21045116	+
19369	1443108_at	Region	6530409C15Rik	76224	6	28262247	28265897	+
19370	1459283_at	Region	6430510B20Rik	320025	5	113869150	113869825	-
19371	1455053_a_at	Region	Tes3	114893	3	35346474	35374678	-
19372	1439340_at	Region	D630036G22Rik	442807	1	34507293	34507933	+
19373	1453665_at	Region	4930529M08Rik	78774	2	145391741	145423157	+
19374	1444939_at	Region	None	None	19	10102765	10103686	+
19375	1455567_at	Region	Crk7	69131	11	98024401	98067791	+
19376	1456489_at	Region	2500001H09Rik	74737	7	86684260	86709889	-
19377	1460273_a_at	Region	Birc1b	17948	13	96337711	96397838	-
19378	1441579_at	Region	Dmrta1	242523	4	88663270	88669838	+

19379	1419394_s_at	Region	S100a8	20201	3	90477555	90478504	+
19380	1430181_at	Region	1700026P10Rik	70069	15	98310954	98312251	-
19381	1438873_at	Region	None	None	13	20984377	20984994	-
19382	1438106_at	Region	Pcdhb22	93893	18	37742075	37745141	+
19383	1443069_at	Region	None	None	11	108000689	108001382	-
19384	1436133_at	Region	A430066A18	328290	13	70426535	70427235	+
19385	1452329_at	Region	Plekhn1	231002	NONE	NONE	NONE	NONE
19386	1421431_at	Region	Ptrf	19285	11	100779597	100791659	-
19387	1452693_at	Region	Dhx35	71715	2	158251789	158315183	+
19388	1451203_at	Region	Mb	17189	15	77067442	77102623	-
19389	1451350_a_at	Region	Leprot	230514	4	100606688	100618263	+
19390	1455459_at	Region	Prdm15	114604	16	97276177	97324273	-
19391	1449391_at	Region	Zfp37	22696	4	61280734	61299618	-
19392	1442882_at	Region	None	None	X	70059708	70060536	-
19393	1419755_at	Region	Mfi2 /// LOC547262	30060	16	30688181	30708391	+
19394	1445795_at	Region	AU022848	107294	NONE	NONE	NONE	NONE
19395	1442251_at	Region	Vcpip1	70675	1	9829046	9852904	-
19396	1431087_at	Region	Spbc24	67629	9	21648325	21653187	-
19397	1423312_at	Region	Tpbg	21983	9	86173432	86176811	+
19398	1440888_at	Region	None	None	6	113036652	113037795	-
19399	1453603_at	Region	2700022O18Rik	72591	14	51189231	51190263	-
19400	1456796_at	Region	Snai3	30927	8	121835793	121842279	-
19401	1442813_at	Region	C130010K08Rik	320127	6	36981421	37434251	-
19402	1450032_at	Region	Slco2a1	24059	9	102980160	103058431	+
19403	1445097_at	Region	None	None	4	115246360	115247142	+
19404	1435888_at	Region	Egfr	13649	11	16646996	16808700	+
19405	1416454_s_at	Region	Acta2	11475	19	33572198	33586443	-
19406	1435597_at	Region	---	237877	11	79860788	79861438	+
19407	1436623_at	Region	Entpd7	93685	19	43234237	43276021	+
19408	1436417_at	Region	None	None	1	83341743	83342445	-
19409	1422571_at	Region	Thbs2	21826	17	12721384	12748324	-
19410	1441386_at	Region	Rapgef1	107746	2	29551925	29670492	+
19411	1453560_at	Region	Fem1a	14154	17	53893863	53898116	+
19412	1460034_at	Region	BC042901	233033	7	23806290	23841166	-
19413	1442470_at	Region	None	None	NONE	NONE	NONE	NONE
19414	1441099_at	Region	None	None	12	73403758	73404726	-
19415	1426625_at	Region	Zfp623	78834	15	75991021	75999469	+
19416	1449126_at	Region	Zfp90	22751	8	105711228	105721681	+
19417	1429541_at	Region	2500002G23Rik	72306	6	163176	187592	+
19418	1450077_at	Region	Chd1	12648	17	13708827	13773901	+
19419	1449409_at	Region	Sult1c2	69083	17	51371775	51387696	-
19420	1435964_a_at	Region	None	None	NONE	NONE	NONE	NONE
19421	1431345_a_at	Region	Taf1b	21340	12	21048387	21108119	+
19422	1452119_at	Region	2600005C20Rik	72462	17	29844491	29868935	+
19423	1447933_at	Region	Kif26a	238403	12	107630689	107661092	+
19424	1438135_at	Region	E130114P18Rik	319865	4	96543212	96753121	-
19425	1452008_at	Region	9130422G05Rik	66819	4	82208143	82211319	-
19426	1436945_x_at	Region	Stim1	20866	7	96373782	96541218	+
19427	1450678_at	Region	Itgb2	16414	10	77653405	77676819	+
19428	1434006_at	Region	BC051227	234384	8	69913907	69916179	-
19429	1450006_at	Region	Ncoa4	27057	14	30303888	30312393	+
19430	1424984_at	Region	2700078E11Rik	78832	19	60127914	60181028	-
19431	1437027_x_at	Region	Rnps1	19826	17	22218678	22229480	+
19432	1416492_at	Region	Ccne1	12447	7	33263859	33273205	-
19433	1416892_s_at	Region	3110001A13Rik	66540	2	3630729	3699405	+
19434	1436384_at	Region	Rps10	67097	17	25426347	25431160	-
19435	1427997_at	Region	1110007M04Rik	68493	4	25033642	25037728	+
19436	1440107_at	Region	None	None	1	152764334	152764954	-
19437	1456360_at	Region	1700022C02Rik	66336	13	49063572	49249594	-
19438	1435099_at	Region	Utp14a	72554	X	42777730	42803245	+
19439	1443923_at	Region	Akap13	233400	7	69488734	69518761	+
19440	1422735_at	Region	Foxq1	15220	13	31038059	31040092	+
19441	1443477_at	Region	Psmd2	21762	16	19423847	19435555	+
19442	1421065_at	Region	Jak2	16452	19	28505054	28564581	+

19443	1451212_at	Region	Ccdc21	70012	4	133090758	133147949	-
19444	1428597_at	Region	2700008N14Rik	76795	11	49884214	49925626	+
19445	1433147_at	Region	Cald1	109624	6	34803747	34868362	+
19446	1424777_at	Region	Wdr21	73828	12	80390109	80405943	+
19447	1429109_at	Region	Rnf184	77853	9	101048875	101053251	+
19448	1443238_at	Region	None	None	NONE	NONE	NONE	NONE
19449	1433525_at	Region	Ednra	13617	8	76876810	76938556	-
19450	1438956_x_at	Region	Pim3	223775	15	88914123	88917655	+
19451	1444992_at	Region	AI120166	103141	10	82927798	82928617	+
19452	1457348_at	Region	None	None	NONE	NONE	NONE	NONE
19453	1422290_at	Region	Htr1d	15552	4	135304781	135325655	+
19454	1442108_at	Region	Rnf25	57751	1	74896219	74903844	-
19455	1434609_at	Region	B930007L02Rik	321006	9	106945364	106946769	+
19456	1449920_at	Region	Cyp19a1	13075	9	54269958	54297857	-
19457	1445173_at	Region	AK129341	234915	9	8082425	8140437	-
19458	1445721_at	Region	A830021M18	223483	15	36724999	36730441	+
19459	1459118_at	Region	D230038C21	328872	17	83898126	83899103	-
19460	1441660_at	Region	None	None	NONE	NONE	NONE	NONE
19461	1421071_at	Region	Vhlh	22346	6	114191852	114199465	+
19462	1453033_at	Region	2010003O02Rik	66434	4	40408436	40409792	+
19463	1442679_at	Region	Map2k4	26398	11	65413894	65513947	-
19464	1432858_at	Region	D730048M19Rik	77991	6	120828832	120829806	-
19465	1447567_at	Region	Odz3	23965	8	47191599	47638956	-
19466	1430651_s_at	Region	Zfp191	59057	18	24240763	24249267	-
19467	1417000_at	Region	Abtb1	80283	6	89270972	89277509	-
19468	1442588_at	Region	9530060I07	329217	1	96655825	96656270	+
19469	1439166_at	Region	1500002M01Rik	68979	11	106987749	107010349	-
19470	1441039_at	Region	4930532J02Rik	75195	3	94441141	94441819	-
19471	1446757_at	Region	Sugt1	67955	14	73941738	73983802	+
19472	1451995_at	Region	Taf11	68776	17	25697046	25703642	-
19473	1453190_at	Region	Usp19	71472	9	108541037	108550266	+
19474	1457686_at	Region	B230333C21Rik /// LOC547167	338351	X	31228738	31232885	-
19475	1419985_s_at	Region	D11Ertd461e	52570	11	54803571	54830972	-
19476	1433380_at	Region	4921509A06Rik	70883	9	78829212	78830238	+
19477	1425146_at	Region	2410075B13Rik	223648	15	76030756	76031978	-
19478	1449561_at	Region	4921504I05Rik	66707	13	20947163	20948551	-
19479	1435329_at	Region	Fbxl11	225876	19	4106596	4187056	-
19480	1447540_at	Region	Tigd3	332359	19	5679928	5682897	-
19481	1456367_at	Region	Fut8	53618	12	74095608	74332578	+
19482	1427541_x_at	Region	Hmmr	15366	11	40440695	40472363	-
19483	1459138_at	Region	None	None	4	133777531	133778024	-
19484	1423257_at	Region	Cyp4a14	13119	4	114445050	114455042	-
19485	1425815_a_at	Region	Hmmr	15366	11	40440695	40472363	-
19486	1423079_a_at	Region	Tomm20 /// LOC546321	546321	X	69368395	69375252	-
19487	1436256_at	Region	Grpel2	17714	18	61939158	61955132	-
19488	1453698_at	Region	6030451C04Rik	77115	4	104159180	104160819	+
19489	1458801_at	Region	AU022320	99812	3	88683581	88684110	-
19490	1441119_at	Region	Garnl1	56784	12	52323288	52540884	-
19491	1417678_at	Region	Mmp24	17391	2	155232322	155273266	+
19492	1455451_at	Region	AI449310	101584	7	91504850	91505827	+
19493	1427682_a_at	Region	Egr2	13654	10	67591672	67595982	+
19494	1417137_at	Region	Uck2	80914	1	167159294	167218315	-
19495	1441796_at	Region	2010309E21Rik	66488	6	4013569	4015176	+
19496	1440363_at	Region	None	None	9	14836388	14837430	-
19497	1435271_at	Region	Irf3	54131	7	39074202	39079390	+
19498	1434817_s_at	Region	4930535B03Rik	75137	3	95249480	95306695	-
19499	1449752_at	Region	4930422J18Rik	74646	4	148388645	148447368	-
19500	1448495_at	Region	Tsta3	22122	15	75974752	75979799	-
19501	1447219_at	Region	None	None	7	23436666	23437058	-
19502	1455067_at	Region	Psd4	215632	2	24317559	24340891	+
19503	1443160_at	Region	Mtmr13	319934	7	104160882	104472741	-
19504	1447778_x_at	Region	MGI:2389572	210766	X	70077355	70114652	+
19505	1440063_at	Region	Farsla	66590	8	84124139	84134142	+

19506	1455185_s_at	Region	None	None	X	18756973	18758878	+
19507	1421825_at	Region	Bace1	23821	9	45851835	45875693	+
19508	1459927_at	Region	4833445I07Rik	102731	9	108616531	108618263	+
19509	1449725_at	Region	Gfpt1	14583	6	87476967	87522715	+
19510	1422134_at	Region	Fosb	14282	7	16172156	16178997	-
19511	1428727_at	Region	4631422C13Rik	70799	18	68028805	68115956	+
19512	1419566_at	Region	Fank1	66930	7	128144543	128249184	+
19513	1439465_x_at	Region	9430057O19Rik	231093	5	29347796	29355631	+
19514	1430137_at	Region	Map3k13	71751	16	20663480	20702856	+
19515	1458045_at	Region	Odz4	23966	7	90212889	90948341	+
19516	1426928_at	Region	4633402D15Rik	70829	1	121194302	121212958	+
19517	1456332_at	Region	Tmem17	103765	11	22407076	22414024	+
19518	1431265_at	Region	Zfp28	22690	7	5579089	5591525	+
19519	1445665_at	Region	38601	56526	X	31532799	31610011	-
19520	1447109_at	Region	None	None	8	3647034	3647305	-
19521	1431099_at	Region	Hoxd8	15437	2	74403297	74405003	+
19522	1451138_x_at	Region	2700087H15Rik	70427	10	79660963	79675809	-
19523	1445460_at	Region	None	None	4	32862076	32862614	-
19524	1432221_at	Region	5330417H12Rik	76679	7	101573971	101575148	-
19525	1421151_a_at	Region	Epha2	13836	4	140182501	140210639	+
19526	1451056_at	Region	Psmd7	17463	8	106878074	106886175	-
19527	1425154_a_at	Region	Csf1	12977	3	107536887	107556052	-
19528	1429375_at	Region	Anapc10	68999	8	78925931	78991431	+
19529	1441540_at	Region	9030406N13Rik	211329	10	30689068	30845679	-
19530	1445198_at	Region	Utx	22289	X	16401607	16518298	+
19531	1439210_at	Region	Mrps9	69527	1	43145999	43200398	+
19532	1443458_at	Region	D630033O11Rik	235302	9	43219236	43239030	+
19533	1434212_at	Region	Ndufs8	225887	19	3697661	3701550	-
19534	1445640_at	Region	Hlcs	110948	16	93524578	93682724	-
19535	1457914_at	Region	None	None	17	71992621	71993287	-
19536	1450493_at	Region	Gpr54	114229	10	80039605	80044817	+
19537	1422297_at	Region	Pfdn5	56612	15	102386650	102391967	+
19538	1445151_at	Region	Mtf2	17765	5	107157468	107177436	+
19539	1435024_at	Region	4931428F04Rik	74356	8	104576037	104584678	-
19540	1446798_at	Region	Map4k3	225028	17	78411242	78558951	-
19541	1444373_at	Region	Tiam1	21844	16	88944544	89118259	-
19542	1439528_at	Region	4833423E24Rik	228151	2	85181343	85216773	-
19543	1417818_at	Region	Wwtr1	97064	3	57091063	57208422	-
19544	1445193_x_at	Region	D8Ertd575e	52367	8	93315963	93316467	-
19545	1452343_at	Region	D18Ertd653e	52662	18	68295003	68486335	+
19546	1456566_x_at	Region	Rbm14	56275	19	4589715	4600375	-
19547	1451405_at	Region	Pcca	110821	14	117100264	117454874	+
19548	1432770_at	Region	0610040A22Rik	68376	13	70182951	70184122	-
19549	1422295_at	Region	Mds1	17251	3	29648096	29649848	-
19550	1452668_x_at	Region	Rab2b	76338	14	47361876	47379434	-
19551	1437075_at	Region	Frmd3	242506	4	73074880	73251147	+
19552	1452429_s_at	Region	Abcf1	224742	17	33675840	33688771	-
19553	1442263_at	Region	Rgs13	246709	1	143970730	144009435	-
19554	1453552_at	Region	2310014F07Rik	69631	9	50561726	50577132	-
19555	1429504_at	Region	Rnpc3	67225	3	14547	39217	+
19556	1449706_s_at	Region	Nr5a2	26424	1	136693054	136812271	-
19557	1417979_at	Region	Tnmd	64103	X	127395947	127410516	+
19558	1434713_at	Region	AI452372	239555	15	80286820	80305424	+
19559	1422336_at	Region	Hoxa13	15398	6	52403288	52404624	-
19560	1458069_at	Region	Tbc1d5	72238	17	48265628	48712039	-
19561	1442048_at	Region	Rnf11	29864	4	108411762	108435410	-
19562	1444582_at	Region	4833420K19Rik	76863	9	7176782	7200101	+
19563	1445105_at	Region	Pcaf	18519	17	51101486	51203822	+
19564	1447201_at	Region	None	None	7	29712564	29713054	+
19565	1422813_at	Region	Cacng1	12299	11	107524753	107537611	-
19566	1459468_at	Region	C79743	97075	NONE	NONE	NONE	NONE
19567	1420104_at	Region	Sertad2	58172	11	20438127	20543916	+
19568	1428992_at	Region	Unc13d	70450	11	115883182	115889569	-
19569	1458121_at	Region	A430107O13Rik	214642	6	22056746	22284808	+
19570	1447996_at	Region	AI848149	102944	X	149196622	149197101	+

19571	1449646_s_at	Region	Tigd5	105734	15	75959829	75964603	+
19572	1430188_at	Region	1700037C18Rik	73261	16	3577365	3580224	-
19573	1441431_at	Region	1700041C02Rik	73332	4	118281798	118374408	-
19574	1431904_at	Region	4933427G17Rik	74466	7	114858076	114890354	+
19575	1428744_s_at	Region	Bri3bp	76809	5	124608455	124621964	+
19576	1455281_at	Region	Wdr33	74320	18	32067429	32147139	+
19577	1449125_at	Region	Tnfaip8l1	66443	17	53799615	53810994	+
19578	1441708_at	Region	Spag16	66722	1	70121849	70562157	+
19579	1428230_at	Region	Prkcn	75292	17	76772784	76835125	-
19580	1443437_at	Region	None	None	3	82566899	82567494	-
19581	1446217_at	Region	LOC433202	433202	18	74294711	74302741	+
19582	1446482_at	Region	D9Mgc48e	28135	9	102536385	102575474	-
19583	1432731_at	Region	5830437K03Rik	76068	3	129832319	129833698	+
19584	1440162_x_at	Region	A630043P06	328187	13	12510581	12512925	-
19585	1428276_at	Region	1110065L07Rik	68904	8	9345221	9357725	+
19586	1445374_at	Region	Vti1a	53611	19	54896591	55207543	+
19587	1446234_at	Region	Utx	22289	X	16401607	16518298	+
19588	1435545_at	Region	BC032203	210982	17	44309813	44343803	-
19589	1442731_at	Region	B230308N11Rik	320060	5	64425311	64425695	-
19590	1415722_a_at	Region	1110059P08Rik	66201	10	14555698	14606206	-
19591	1433530_at	Region	2210411K19Rik	70164	10	128285204	128286227	-
19592	1429939_at	Region	D19Ertd703e	52036	19	3243622	3364414	-
19593	1445669_at	Region	Spry4	24066	18	38809987	38824990	-
19594	1421153_at	Region	Loxl4	67573	19	42138388	42157154	-
19595	1425771_at	Region	Akr1d1	208665	6	37665377	37703622	+
19596	1429877_at	Region	Lrrc44	74435	3	154100544	154201154	+
19597	1428350_at	Region	2310061F22Rik /// LOC547026	234839	8	121857730	121863335	+
19598	1458017_at	Region	6820402O20Rik	228829	2	155653612	155764373	+
19599	1430855_at	Region	Col20a1	73368	2	180703493	180734498	+
19600	1454110_at	Region	4930408K08Rik	76865	3	108874446	108876157	-
19601	1427729_at	Region	Ehd2	259300	7	86	1894	+
19602	1421986_at	Region	Eif4e2	26987	1	87030076	87054566	+
19603	1424181_at	Region	38601	56526	X	31532799	31610011	-
19604	1455770_at	Region	Tdo2	56720	3	81687472	81704788	-
19605	1453690_at	Region	Mpp7	75739	18	7422318	7651672	-
19606	1450348_at	Region	Slc19a3	80721	1	83343035	83367686	-
19607	1430226_at	Region	2900036K24Rik	72891	X	67894341	67903682	-
19608	1417603_at	Region	Per2	18627	1	91239024	91282480	-
19609	1432431_s_at	Region	1110033L15Rik	73752	2	139909313	139934227	+
19610	1437059_at	Region	Sox21	223227	14	112788599	112791264	-
19611	1440009_at	Region	Olfr78	170639	7	96847781	96866447	-
19612	1454727_at	Region	AI173486	106877	18	61959074	62015474	-
19613	1431435_at	Region	None	None	10	7138703	7142174	+
19614	1445958_at	Region	None	None	3	105238596	105239271	-
19615	1429417_at	Region	4833446K15Rik	78923	18	59401367	59636538	+
19616	1419468_at	Region	Clec14a	66864	12	54996197	55000365	-
19617	1446172_at	Region	BC026657	208618	2	20552057	20852534	+
19618	1428743_at	Region	Bri3bp	76809	5	124608455	124621964	+
19619	1435243_at	Region	2810407L07Rik	69228	6	48187302	48189160	-
19620	1446725_at	Region	A130094D17Rik	319460	2	150497292	150497911	+
19621	1450400_at	Region	Ncoa6ip	116940	4	3502044	3543770	+
19622	1438910_a_at	Region	Stom	13830	2	35246152	35269171	-
19623	1442186_at	Region	Sca7	246103	14	12474639	12566208	+
19624	1450951_at	Region	Cspg6	13006	19	53166173	53210193	+
19625	1457408_at	Region	Pde4a	18577	9	21049525	21095183	+
19626	1434675_at	Region	1700065O13Rik	73451	17	30841770	30858356	-
19627	1445598_at	Region	None	None	8	56653428	56653779	-
19628	1419261_at	Region	Acad8	66948	9	26868034	26893463	-
19629	1419235_s_at	Region	Helb	117599	10	119661305	119690587	-
19630	1429943_at	Region	Ctbs	74245	3	145427044	145442322	+
19631	1442483_at	Region	Fut8	53618	12	74095608	74332578	+
19632	1427691_a_at	Region	Ifnar2	15976	16	90530631	90563104	+
19633	1417444_at	Region	E2f5	13559	3	14575523	14603161	+
19634	1454378_at	Region	Ywhaq	22630	12	18728420	18754333	+

19635	1438845_at	Region	Ihpk1	27399	9	108070635	108116769	+
19636	1426363_x_at	Region	H2afy3	404634	10	61704953	61750170	-
19637	1423530_at	Region	Stk32c	57740	7	133505216	133584149	-
19638	1427725_a_at	Region	Pou2f2	18987	7	20270879	20309756	-
19639	1452470_at	Region	4933409L06Rik	74081	1	155740307	155868596	-
19640	1460067_at	Region	Ccr2	12772	9	124128581	124134948	+
19641	1443334_at	Region	D430042O09Rik	233865	7	119802703	119911727	+
19642	1442476_at	Region	Cdv1	12589	5	121702852	121766929	-
19643	1422513_at	Region	Ccnf	12449	17	22026134	22054932	-
19644	1456353_at	Region	None	None	11	87471614	87472255	+
19645	1440403_at	Region	Tmsb10	19240	6	20039912	20040386	+
19646	1428825_at	Region	Nr6a1	14536	2	38655532	38859850	-
19647	1423782_at	Region	Mobk1b	232157	6	83670277	83687043	+
19648	1457755_at	Region	Gng8	14709	7	13845956	13846375	+
19649	1452025_a_at	Region	Zfp2	22678	11	50651553	50669006	-
19650	1432807_at	Region	5830426K05Rik	76053	NONE	NONE	NONE	NONE
19651	1423595_at	Region	Mina	67014	16	58457856	58478079	+
19652	1435315_s_at	Region	2900034E22Rik	72904	7	107423520	107424027	+
19653	1419967_at	Region	Seh1l	72124	18	68005761	68023379	+
19654	1439125_at	Region	Add1	11518	5	33061487	33119289	+
19655	1450285_at	Region	Ube1y1	22202	Y	155155	180666	+
19656	1439668_at	Region	None	None	11	112613874	112619995	-
19657	1424448_at	Region	Trim6	94088	7	98344459	98347510	+
19658	1433177_at	Region	5830411G16Rik	78937	6	56859230	56906236	+
19659	1456276_at	Region	None	None	9	63516160	63516590	-
19660	1430596_s_at	Region	1700110N18Rik	73569	16	64821348	64869062	+
19661	1429610_a_at	Region	Zfp511	69752	7	134435402	134439603	+
19662	1419368_a_at	Region	Rnf138	56515	18	21216269	21243151	+
19663	1459734_at	Region	Psmd14	59029	2	61567169	61655776	+
19664	1447527_at	Region	Irf2	16363	8	45686560	45794326	+
19665	1442862_at	Region	None	None	12	96416382	96417245	-
19666	1424962_at	Region	Tm4sf4	229302	3	57058070	57074280	+
19667	1443625_at	Region	4931433E08Rik	70999	19	6941795	6955246	-
19668	1418297_at	Region	Dpysl4	26757	7	133487649	133503345	+
19669	1453164_a_at	Region	Ptdss2	27388	7	135533273	135558141	+
19670	1437055_x_at	Region	1200003O06Rik	66868	3	67255079	67276542	+
19671	1441345_at	Region	None	None		24679117	24680249	-
19672	1418064_at	Region	Tfpt	69714	7	9788940	10280008	-
19673	1422814_at	Region	Calmbp1	12316	1	139301969	139341264	+
19674	1446363_at	Region	None	None	10	97705646	97706194	-
19675	1433103_at	Region	3010015K02Rik	76832	9	35471945	35473316	-
19676	1453110_at	Region	Wdr51b	382406	10	99087833	99178075	+
19677	1447862_x_at	Region	Thbs2	21826	17	12721384	12748324	-
19678	1421050_at	Region	D11Wsu68e	28084	11	101074830	101080633	+
19679	1417906_at	Region	1700001F09Rik	71826	14	40267851	40273273	-
19680	1444971_at	Region	Rbm5	83486	9	107808484	107838963	-
19681	1444819_at	Region	C86090	97662	NONE	NONE	NONE	NONE
19682	1442125_at	Region	Ccm1	79264	5	3809194	3847364	+
19683	1447892_at	Region	None	None	18	23435293	23435498	+
19684	1450038_s_at	Region	Usp9x	22284	X	11310477	11410726	+
19685	1457474_at	Region	None	None	19	41933035	41933940	-
19686	1449751_at	Region	Slc6a6	21366	6	92133220	92208188	+
19687	1442375_at	Region	Ubap2	68926	4	41333166	41413986	-
19688	1437699_at	Region	E430014B02Rik	320908	17	49280333	49281014	-
19689	1449436_s_at	Region	Ubb	22187	11	62277156	62278863	+
19690	1431704_a_at	Region	Ralgps2	78255	1	156713690	156845148	-
19691	1430530_s_at	Region	1110025F24Rik	67824	16	4382757	4390413	-
19692	1452749_at	Region	Papd1	67440	18	4380336	4401528	+
19693	1456893_at	Region	None	None	NONE	NONE	NONE	NONE
19694	1441019_at	Region	Fbxo3	57443	2	103732553	103767991	+
19695	1458037_at	Region	Ncald	52589	15	37371112	37797344	-
19696	1428863_at	Region	Ankrd39	109346	1	36832764	36841792	-
19697	1460339_at	Region	Psma4	26441	9	55070048	55077219	+
19698	1444512_at	Region	Arhgap29	214137	3	120732219	120795050	+
19699	1429319_at	Region	Rhoh	74734	5	64661623	64698386	+

19700	1444595_at	Region	None	None	NONE	NONE	NONE	NONE
19701	1432155_at	Region	Wasl	73178	6	24663290	24714456	-
19702	1430304_at	Region	Wdr76	241627	2	121020636	121058710	+
19703	1428985_at	Region	Phf22	71793	3	131982349	132001384	+
19704	1453539_at	Region	4930562C03Rik	67667	9	3239798	3247879	+
19705	1458874_at	Region	---	382129	9	16052787	16053507	-
19706	1450772_at	Region	Wnt11	22411	7	92930295	92945198	+
19707	1424451_at	Region	MGC29978	235674	9	119142304	119151354	-
19708	1453719_at	Region	4930506C21Rik	75060	17	8864993	8870266	-
19709	1441763_at	Region	Zdhhc6	66980	19	54859814	54896233	-
19710	1440237_at	Region	Ercc4	50505	16	11850826	11883488	+
19711	1437956_at	Region	BB220380	104709	11	68228749	68278356	+
19712	1420985_at	Region	Ash1l	192195	3	88709675	88822741	+
19713	1442395_at	Region	Dst	13518	1	34280938	34577763	+
19714	1415774_at	Region	Statip1	58523	18	24832491	24867306	+
19715	1417496_at	Region	Cp	12870	3	19298453	19333864	+
19716	1458422_at	Region	D730002M21Rik	269094	19	55617518	55622419	+
19717	1427083_a_at	Region	Map4k5	399510	12	66635229	66724562	-
19718	1432820_at	Region	Psmd7	17463	8	106878074	106886175	-
19719	1439521_at	Region	None	None	NONE	NONE	NONE	NONE
19720	1456102_a_at	Region	Cul5	75717	9	53680190	53730439	-
19721	1417811_at	Region	Slc24a6	170756	5	119662999	119685826	+
19722	1450816_at	Region	Polg2	50776	11	106589395	106600531	-
19723	1451621_at	Region	5830417C01Rik	78825	1	178140297	178205198	+
19724	1424060_at	Region	Neil3	234258	8	52551737	52603865	-
19725	1457297_at	Region	Mef2a	17258	7	61136273	61137731	-
19726	1456294_at	Region	0610013E23Rik	76892	11	86212649	86226999	+
19727	1442833_at	Region	D15Ertd30e	52238	15	27497770	27499035	+
19728	1426749_at	Region	Hrmt1l3	71974	7	43892468	43972375	+
19729	1420175_at	Region	Tax1bp1	52440	6	52858073	52910809	+
19730	1417415_at	Region	Slc6a3	13162	13	69595107	69637297	+
19731	1447882_x_at	Region	Ddx54	71990	5	119764937	119780399	+
19732	1453084_s_at	Region	2310067L16Rik	69700	15	71858269	71984039	-
19733	1427529_at	Region	Fzd9	14371	5	134262447	134264521	-
19734	1453183_at	Region	1110034A24Rik	109065	12	66020879	66028871	-
19735	1449755_at	Region	D7Ertd183e	52234	7	112068457	112068680	-
19736	1458540_at	Region	None	None	5	76042866	76045459	-
19737	1440423_at	Region	Zbtb37	240869	1	160921704	160938390	-
19738	1442317_at	Region	G3bp	27041	11	55238464	55253728	+
19739	1458191_at	Region	Foxp2	114142	6	14888986	15429612	+
19740	1437505_at	Region	None	None	15	27892215	27893417	-
19741	1442386_at	Region	C030010B13Rik	77310	2	173046327	173120005	-
19742	1442107_at	Region	Flnb	286940	14	5564611	5619816	+
19743	1450464_at	Region	E4f1	13560	17	22247378	22255104	-
19744	1432918_at	Region	4921511E18Rik	70879	X	148926900	148928840	+
19745	1421147_at	Region	Terf2	21750	8	106367591	106394116	-
19746	1417456_at	Region	Gnpat	14712	8	124150410	124177380	+
19747	1447735_x_at	Region	MGI:1926224	268859	16	6740061	7081334	+
19748	1424887_at	Region	Klhdc4	234825	8	121177917	121211156	-
19749	1438797_at	Region	6330509M05Rik	102913	X	18337839	18339194	+
19750	1434772_at	Region	Adora2b	11541	11	61974626	61992094	+
19751	1440144_x_at	Region	C330046E03	328468	NONE	NONE	NONE	NONE
19752	1435660_at	Region	LOC545340	545340	1	85323446	85335993	-
19753	1434486_x_at	Region	Ugp2	216558	11	21215937	21265268	-
19754	1417270_at	Region	Wdr12	57750	1	60380183	60401193	-
19755	1432868_at	Region	2310047C04Rik	224170	16	47767664	47835780	-
19756	1430314_at	Region	4933437F05Rik	71275	12	84100666	84118416	-
19757	1439867_at	Region	Ubox5	140629	2	130103855	130143839	-
19758	1424422_s_at	Region	Lenep /// A930017E24Rik	319945	3	89157732	89168601	-
19759	1436505_at	Region	Ppig	228005	2	69578493	69605782	+
19760	1420249_s_at	Region	Ccl6	20305	11	83315881	83320979	-
19761	1420217_x_at	Region	None	None	NONE	NONE	NONE	NONE
19762	1454634_at	Region	Fuk	234730	8	110180185	110200181	-
19763	1418511_at	Region	Dpt	56429	1	164716777	164744311	+

19764	1428175_at	Region	2810446P07Rik	72745	13	80317260	80390898	+
19765	1446834_at	Region	Ctsc	13032	7	82254582	82287335	+
19766	1449746_s_at	Region	Glipr1	73690	10	111688463	111700201	-
19767	1443076_at	Region	D030041N04Rik	270035	8	24316834	24322553	-
19768	1456585_x_at	Region	E130309D02Rik	231868	5	142360090	142374242	-
19769	1442452_at	Region	Als2cr2	227154	1	59278531	59300058	+
19770	1432765_at	Region	C030007D22Rik	77319	4	109394328	109394897	+
19771	1448540_a_at	Region	0610012G03Rik	106264	16	30756422	30757865	-
19772	1447888_x_at	Region	Msh2	17685	17	85528737	85580669	+
19773	1442908_at	Region	Tm9sf1	74140	14	50153981	50161821	-
19774	1455224_at	Region	Angptl1	72713	1	156744510	156751183	+
19775	1443081_at	Region	Gata6	14465	18	11097852	11130974	+
19776	1447322_at	Region	None	None	18	32178791	32179243	+
19777	1422410_at	Region	Ferd3l	114712	12	30541084	30541969	+
19778	1457669_x_at	Region	None	None	5	1324536	1324662	+
19779	1430515_s_at	Region	Aasdhppt	67618	9	4203626	4217037	-
19780	1438033_at	Region	Tef	21685	15	81853534	81877580	+
19781	1442259_at	Region	None	None	NONE	NONE	NONE	NONE
19782	1430474_a_at	Region	Mtch2	56428	2	90551904	90571383	+
19783	1430430_at	Region	6130401L20Rik	75740	3	28484617	29093093	+
19784	1458931_at	Region	6030427F01Rik	97411	7	133271567	133272160	+
19785	1455576_at	Region	5830482F20Rik	320435	7	24195184	24204826	+
19786	1458610_at	Region	None	None	2	25157727	25158335	-
19787	1439672_at	Region	Synj1	104015	16	90104242	90168538	-
19788	1431016_at	Region	Cwf19l1	72502	19	43654425	43680279	-
19789	1430846_at	Region	1700061G19Rik	78625	17	54565405	54578826	+
19790	1424837_at	Region	2810428C21Rik	69942	X	31811897	31813021	+
19791	1456928_at	Region	None	None	2	151571075	151571717	+
19792	1457138_x_at	Region	None	None	NONE	NONE	NONE	NONE
19793	1430994_at	Region	LOC434321	434321	8	68387408	68388621	-
19794	1446686_at	Region	Dnajc11	230935	4	150426093	150469046	+
19795	1431692_a_at	Region	Cblc	80794	7	16648964	16666053	-
19796	1446298_at	Region	C630016I17Rik	320000	12	78379513	78380190	-
19797	1451365_at	Region	Rbm19	74111	5	119268320	119350778	+
19798	1426651_at	Region	Mrpl44	69163	1	80106651	80112515	+
19799	1459378_at	Region	Ppp3cb	19056	14	18861352	18907856	-
19800	1442053_at	Region	Phkb	102093	8	85113615	85333255	+
19801	1459525_at	Region	Cbara1	216001	10	59664687	59819557	+
19802	1460521_a_at	Region	5830411E10Rik	109019	1	51769634	51779343	-
19803	1453201_at	Region	4632411J06Rik	78748	7	106807656	106811140	+
19804	1421519_a_at	Region	Zfp120	104348	2	149573143	149593614	-
19805	1448519_at	Region	Tead2	21677	7	39292295	39310161	+
19806	1453258_at	Region	Cldn3	12739	5	133999706	134000964	+
19807	1441105_at	Region	None	None	17	54782081	54793732	-
19808	1439688_at	Region	Fbln1	14114	15	85254610	85334255	+
19809	1426220_at	Region	4930471M23Rik	74919	5	29106904	29118671	+
19810	1419281_a_at	Region	Zfp259	22687	9	46228559	46294858	+
19811	1451218_at	Region	Edem1	192193	6	109331841	109362540	+
19812	1426718_at	Region	Skiv2l2	72198	13	109262266	109322348	-
19813	1420780_at	Region	Ascl3	56787	7	103580336	103584603	-
19814	1421144_at	Strain	Rpgrip1	77945	14	47211021	47261035	+
19815	1432198_at	Strain	None	None	19	16446831	16448032	-
19816	1433438_x_at	Strain	Mela	17276	8	122800775	122803541	-
19817	1455904_at	Strain	Gas5	14455	1	160941991	160942669	+
19818	1452544_x_at	Strain	H2-D1	14964	17	186801	249200	-
19819	1449526_a_at	Strain	Gdpd3	68616	7	120816902	120825376	+
19820	1434278_at	Strain	Mtm1	17772	X	65883573	65983756	+
19821	1444198_at	Strain	None	None	10	92118279	92118907	-
19822	1442632_at	Strain	Centg2	347722	1	89273393	89708522	+
19823	1433685_a_at	Strain	6430706D22Rik	381280	1	88082214	88096625	-
19824	1436240_at	Strain	G430041M01Rik	101214	6	49378306	49398393	-
19825	1438390_s_at	Strain	Pttg1	30939	11	43173101	43178818	-
19826	1460670_at	Strain	Riok3	66878	18	12316360	12344298	+
19827	1418217_at	Strain	Nme7	171567	1	164226222	164355246	+
19828	1427651_x_at	Strain	H2-D1	14964	17	186801	249200	-

19829	1453145_at	Strain	4933439C20Rik	236604	11	3020573	3026734	+
19830	1425614_x_at	Strain	H2-D1	14964	17	186801	249200	-
19831	1431214_at	Strain	1300007C21Rik /// LOC433762 /// LOC546083	433762	4	132057341	132062403	+
19832	1456182_x_at	Strain	Mela	17276	8	122800775	122803541	-
19833	1418350_at	Strain	Hbegf	15200	18	36728734	36739576	-
19834	1426387_x_at	Strain	4933439C20Rik /// Pisd	236604	11	3020573	3026734	+
19835	1424105_a_at	Strain	Pttg1	30939	11	43173101	43178818	-
19836	1436533_at	Strain	Ssa2	20822	1	143587635	143609041	-
19837	1441937_s_at	Strain	Pink1	68943	4	137194673	137207532	-
19838	1447831_s_at	Strain	Mtmr7	54384	8	39494737	39579044	-
19839	1453172_at	Strain	Stch	110920	16	74838985	74850259	-
19840	1447901_x_at	Strain	Sfi1 /// LOC545744	545744	5	31212784	31224812	+
19841	1419100_at	Strain	Serpina3n	20716	12	99850891	99858461	+
19842	1439070_x_at	Strain	4933439C20Rik /// Pisd	236604	11	3020573	3026734	+
19843	1440305_at	Strain	None	None	17	33718187	33718773	+
19844	1455892_x_at	Strain	None	None	NONE	NONE	NONE	NONE
19845	1419635_at	Strain	4833420G17Rik	67392	13	16951468	16974828	+
19846	1436850_at	Strain	Creg2	263764	1	39917918	39948099	-
19847	1439483_at	Strain	AI506816	433855	NONE	NONE	NONE	NONE
19848	1458719_at	Strain	Glp1r	14652	17	28714662	28749274	+
19849	1427278_at	Strain	Rsnl2	78785	17	69558453	69633262	+
19850	1434975_x_at	Strain	4933439C20Rik	236604	11	3020573	3026734	+
19851	1416366_at	Strain	Ndufc2	68197	7	91446361	91454068	+
19852	1419327_at	Strain	AA415817	94184	16	12571455	12638826	-
19853	1453208_at	Strain	2700089E24Rik	381820	6	133875346	133882217	+
19854	1441404_at	Strain	Pafah1b1	18472	11	74399613	74450328	-
19855	1454904_at	Strain	Mtm1	17772	X	65883573	65983756	+
19856	1454714_x_at	Strain	Phgdh /// LOC546010	236539	3	97799380	97826178	-
19857	1434340_at	Strain	None	None	11	4596762	4599122	-
19858	1441797_at	Strain	None	None	17	33715526	33715796	+
19859	1435579_at	Strain	None	None	4	42627267	42628153	+
19860	1437478_s_at	Strain	Efhd2	27984	4	140739403	140756181	-
19861	1417644_at	Strain	Sspn	16651	6	146867107	146898185	+
19862	1419188_s_at	Strain	Ccl27	20301	4	41908171	41908905	-
19863	1447937_a_at	Strain	4933409K07Rik /// LOC545604 /// LOC545605 /// LOC545606 /// LOC545608 /// LOC545609 /// LOC545612 /// LOC545614	108816	4	42704619	42707003	+
19864	1436070_at	Strain	None	None	17	28401483	28402217	-
19865	1457973_at	Strain	None	None	14	88293748	88294170	-
19866	1424109_a_at	Strain	Glo1	109801	17	28402993	28422646	-
19867	1419637_s_at	Strain	4833420G17Rik	67392	13	16951468	16974828	+
19868	1435353_a_at	Strain	4933439C20Rik	236604	11	3020573	3026734	+
19869	1424108_at	Strain	Glo1	109801	17	28402993	28422646	-
19870	1444489_at	Strain	Slc25a12	78830	2	70972038	71065297	-
19871	1439241_x_at	Strain	Srd5a2l	57357	5	75385516	75400710	+
19872	1452907_at	Strain	Galc	14420	12	93629142	93686179	-
19873	1459253_at	Strain	Arrdc3	105171	NONE	NONE	NONE	NONE
19874	1415694_at	Strain	Wars	22375	12	104331027	104364190	-
19875	1447360_at	Strain	Tgfb1i4	21807	14	70758316	70850256	+
19876	1420940_x_at	Strain	Rgs5	19737	1	169589678	169627703	+
19877	1445111_at	Strain	Sec8l1	20336	6	33334773	34062752	+
19878	1431213_a_at	Strain	1300007C21Rik /// LOC433762 /// LOC546083	433762	4	132057341	132062403	+

19879	1429951_at	Strain	Ssbp2	66970	13	87661341	87840293	+
19880	1419484_a_at	Strain	Gbas	14467	5	128900966	128934118	+
19881	1436894_at	Strain	D1Ertd471e	27877	1	166232896	166234355	+
19882	1437303_at	Strain	Il6st	16195	13	108856634	108899366	+
19883	1454686_at	Strain	6430706D22Rik	381280	1	88082214	88096625	-
19884	1445307_at	Strain	Auts2	319974	5	130627794	130728707	-
19885	1434216_a_at	Strain	Nudt19	110959	7	30711030	30719686	-
19886	1436462_at	Strain	3100002L24Rik	66376	2	175129264	175144107	-
19887	1438663_at	Strain	Bat2d	226562	1	162581319	162650087	-
19888	1447096_at	Strain	Pbx1	18514	1	168054563	168366387	-
19889	1420354_at	Strain	Cnnm1	83674	19	42985617	43040169	+
19890	1447808_s_at	Strain	Slc15a2	57738	16	35567606	35602387	-
19891	1457323_at	Strain	C030030A07Rik	320135	6	137600237	137612458	+
19892	1438130_at	Strain	Taf15	70439	11	83201097	83234729	+
19893	1424454_at	Strain	A930025J12Rik	211499	2	119869161	119917938	-
19894	1458491_at	Strain	4930422I07Rik	71640	9	88910067	88931850	+
19895	1455773_at	Strain	None	None	NONE	NONE	NONE	NONE
19896	1419130_at	Strain	Deadc1	66757	10	13435001	13444624	+
19897	1417462_at	Strain	Cap1	12331	4	121886167	121899570	-
19898	1459957_at	Strain	Tnrc6	233833	7	117180427	117250357	+
19899	1452705_at	Strain	AA415817	94184	16	12571455	12638826	-
19900	1433831_at	Strain	4833418A01Rik	75763	2	70753595	70786387	+
19901	1446144_at	Strain	Pex2	58869	3	32358790	32491207	-
19902	1443153_at	Strain	6030460N08Rik	109181	12	97280506	97358629	-
19903	1457847_at	Strain	9930116O05Rik	319758	9	72689193	72690196	+
19904	1440274_at	Strain	Rapgef4	56508	2	71679030	71954119	+
19905	1454696_at	Strain	Gnb1	14688	4	153983732	154050180	+
19906	1441955_s_at	Strain	Paip1	218693	13	232	27441	+
19907	1421360_at	Strain	Inpp4a	269180	1	37652393	37702733	+
19908	1460709_a_at	Strain	Bat5	193742	17	32793071	32806767	+
19909	1425521_at	Strain	Paip1	218693	13	232	27441	+
19910	1423322_at	Strain	Lin7c	22343	2	109514665	109524724	+
19911	1422640_at	Strain	Pcdhb9	93880	18	37624577	37627631	+
19912	1425349_a_at	Strain	Myef2	17876	2	124602727	124630728	-
19913	1419636_at	Strain	4833420G17Rik	67392	13	16951468	16974828	+
19914	1429244_at	Strain	2610524H06Rik	330173	NONE	NONE	NONE	NONE
19915	1428778_at	Strain	Sfi1	78887	11	3026676	3091300	-
19916	1433293_at	Strain	1500032O14Rik	69027	13	70411011	70412067	-
19917	1434962_x_at	Strain	Ccl27	20301	4	41908171	41908905	-
19918	1436944_x_at	Strain	4933439C20Rik /// Pisd	236604	11	3020573	3026734	+
19919	1422128_at	Strain	Rpl14	67115	9	120583908	120587044	+
19920	1452195_s_at	Strain	Sfi1	78887	11	3026676	3091300	-
19921	1418701_at	Strain	Comt	12846	16	17178593	17197565	-
19922	1460662_at	Strain	Per3	18628	4	149497074	149536951	-
19923	1451240_a_at	Strain	Glo1	109801	17	28402993	28422646	-
19924	1456257_at	Strain	C130065N10Rik	319340	1	58829562	58831150	-
19925	1448793_a_at	Strain	Sdc4	20971	2	163880980	163899921	-
19926	1416494_at	Strain	Ndufs5	170658	4	122739822	122743439	-
19927	1426607_at	Strain	None	None	NONE	NONE	NONE	NONE
19928	1451242_a_at	Strain	Ppp5c	19060	7	13955563	13978781	-
19929	1428381_a_at	Strain	2700038C09Rik	66496	2	180904300	180905461	+
19930	1438426_at	Strain	9330132O05Rik	77552	1	135221929	135225536	-
19931	1431225_at	Strain	Sox11	20666	12	23889533	23891005	+
19932	1442893_at	Strain	Lrrtm1	74342	6	77592843	77607877	+
19933	1430648_at	Strain	Scn2b	72821	9	45117694	45118924	-
19934	1429870_at	Strain	C630040K21Rik	78708	3	27777068	27777886	+
19935	1429691_at	Strain	5430405N12Rik	71324	14	10699156	10701295	+
19936	1456471_x_at	Strain	LOC224870 /// Phgdh /// LOC269242 /// LOC383450 /// LOC434173 /// LOC546010	224870	17	50730422	50732759	+
19937	1433913_at	Strain	C80913	19777	7	33124913	33183548	-

19938	1441373_at	Strain	None	None	11	88248088	88248903	-
19939	1438758_at	Strain	None	None	NONE	NONE	NONE	NONE
19940	1440557_at	Strain	Ipw	16353		26442375	26443088	+
19941	1454592_at	Strain	9430012M22Rik	77244	3	54925280	54926282	+
19942	1456518_at	Strain	4930422I07Rik	71640	9	88910067	88931850	+
19943	1425530_a_at	Strain	Stx3	20908	19	10973053	11015388	-
19944	1456781_at	Strain	Sox2	20674	3	34105755	34108159	+
19945	1428368_at	Strain	Arhgap21	71435	2	20889950	21009731	-
19946	1438437_a_at	Strain	4933439C10Rik	74476	11	59235162	59235785	+
19947	1438754_at	Strain	None	None	NONE	NONE	NONE	NONE
19948	1445204_at	Strain	E030025D05Rik	216613	11	28283605	28479086	-
19949	1423216_a_at	Strain	2510049I19Rik	67922	8	71378153	71382197	+
19950	1452660_s_at	Strain	Klhl7	52323	5	22564507	22624682	+
19951	1443621_at	Strain	---	327959	11	72027329	72045073	+
19952	1430375_a_at	Strain	Ccl27	20301	4	41908171	41908905	-
19953	1424168_a_at	Strain	None	None	4	138074245	138173019	+
19954	1438644_x_at	Strain	Commd9	76501	2	101590945	101606322	+
19955	1439845_at	Strain	None	None	4	137124969	137125945	+
19956	1458585_at	Strain	None	None	NONE	NONE	NONE	NONE
19957	1460587_at	Strain	B230215L15Rik	320478	3	34133052	34133698	+
19958	1417432_a_at	Strain	Gnb1	14688	4	153983732	154050180	+
19959	1437892_at	Strain	Zfp306	72739	13	20867145	20884810	-
19960	1456655_at	Strain	Ext1	14042	15	53068219	53345629	-
19961	1439300_at	Strain	None	None	X	97992310	97993041	+
19962	1417259_a_at	Strain	Capzb	12345	4	138120169	138172527	+
19963	1417461_at	Strain	Cap1	12331	4	121886167	121899570	-
19964	1434328_at	Strain	Rpl15 /// LOC384179	384179	5	42671761	42701560	-
19965	1426787_at	Strain	Sfi1	78887	11	3026676	3091300	-
19966	1429509_at	Strain	None	None	11	101983584	101984643	-
19967	1456720_at	Strain	---	380686	11	16961308	16961698	+
19968	1455682_at	Strain	AA536808	98214	1	156550685	156552389	+
19969	1437608_x_at	Strain	Ywhaq	22630	12	18728420	18754333	+
19970	1444128_at	Strain	4933432P15Rik	71302	18	39216929	39600082	+
19971	1447517_at	Strain	Skiv2l2	72198	13	109262266	109322348	-
19972	1429628_at	Strain	6330407J23Rik	67412	10	29174943	29230605	+
19973	1417849_at	Strain	MGI:2180715	170753	3	9425289	9606906	-
19974	1455806_x_at	Strain	Ndufa12	66414	10	94172393	94194766	+
19975	1453297_at	Strain	4933417O08Rik	71146	19	41800115	41814754	+
19976	1449603_at	Strain	AI594671	103795	11	39775166	39775334	-
19977	1447938_at	Strain	LOC545608	545608	4	42087810	42117024	-
19978	1444260_at	Strain	None	None	1	67773063	67773539	+
19979	1441388_at	Strain	None	None	18	70861108	70861785	+
19980	1443100_at	Strain	---	380887	14	16345175	16345719	+
19981	1447894_x_at	Strain	Vps52	224705	17	31670801	31670899	+
19982	1434375_at	Strain	B930006L02Rik	319604	7	94813378	94948322	+
19983	1416524_at	Strain	Spop	20747	11	95235201	95314100	+
19984	1424877_a_at	Strain	Alad	17025	4	61600361	61611254	-
19985	1436846_x_at	Strain	LOC546165	546165	9	119152266	119158348	+
19986	1441573_at	Strain	Scmh1	29871	4	119469474	119552399	+
19987	1444890_at	Strain	AA536749	26936	11	59388161	59501851	+
19988	1449964_a_at	Strain	Mlycd	56690	NONE	NONE	NONE	NONE
19989	1416947_s_at	Strain	Acaa1 /// MGC29978	113868	9	119335555	119344556	+
19990	1451626_x_at	Strain	None	None	NONE	NONE	NONE	NONE
19991	1439630_x_at	Strain	MGI:2446326	282619	7	26160849	26165448	+
19992	1418349_at	Strain	Hbegf	15200	18	36728734	36739576	-
19993	1430485_at	Strain	3010009O07Rik	76847	7	96171709	96173586	+
19994	1454751_at	Strain	G430022H21Rik	210529	3	122159943	122177594	-
19995	1443239_at	Strain	Mtap2	17756	1	66623478	66624138	+
19996	1417648_s_at	Strain	Snx5	69178	2	143707062	143727467	-
19997	1428418_s_at	Strain	3110050N22Rik	67306	3	7478034	7528335	+

19998	1428301_at	Strain	LOC544986 /// LOC544988 /// LOC544991 /// LOC545001 /// LOC545005 /// LOC545007 /// 2610042L04Rik /// LOC545017	544986	14	3010193	3030911	+
19999	1438691_at	Strain	Zzef1	195018	11	72599528	72652778	+
20000	1436040_at	Strain	2310005L22Rik	69471	4	131105578	131107910	+
20001	1424336_at	Strain	8430432M10Rik	66812	9	57526299	57551131	-
20002	1417087_at	Strain	Glg1	20340	8	110455266	110556791	-
20003	1459897_a_at	Strain	MGI:2446326	282619	7	26160849	26165448	+
20004	1452665_at	Strain	2610511O17Rik	74196	17	72525543	72670806	+
20005	1419040_at	Strain	Cyp2d22	56448	15	82422189	82430879	-
20006	1422998_a_at	Strain	Glrx2	69367	1	143571711	143578676	+
20007	1449083_at	Strain	1810060J02Rik	67015	6	148405067	148561800	+
20008	1441684_at	Strain	Ttc3	22129	16	93778128	93866771	+
20009	1458886_at	Strain	Spop	20747	11	95235201	95314100	+
20010	1425320_at	Strain	A830023I12Rik	320875	5	109178408	109186230	+
20011	1420889_at	Strain	Hccs	15159	X	162910940	162919685	-
20012	1423205_at	Strain	Tm9sf4	99237	2	152618237	152667399	+
20013	1430026_at	Strain	Stch	110920	16	74838985	74850259	-
20014	1445427_at	Strain	Nlk	18099	11	78292827	78423032	-
20015	1416103_at	Strain	Ywhaz	22631	15	36773662	36797651	-
20016	1437070_at	Strain	Cdc14b	218294	13	61579944	61659178	-
20017	1425029_a_at	Strain	Oact2	67216	12	21381378	21511358	+
20018	1442466_a_at	Strain	B430315C20Rik	327655	2	120824412	120864866	-
20019	1449018_at	Strain	Pfn1	18643	11	70377507	70380293	-
20020	1443772_at	Strain	Cldn10	58187	14	113343391	113430546	+
20021	1451457_at	Strain	Sc5d	235293	9	42213010	42223066	-
20022	1438435_at	Strain	None	None	7	92260395	92261273	-
20023	1442241_at	Strain	Srpk2	20817	5	21967253	22080468	-
20024	1419039_at	Strain	Cyp2d22	56448	15	82422189	82430879	-
20025	1426164_a_at	Strain	Usf1	22278	1	171340520	171347582	+
20026	1448799_s_at	Strain	Mrps12	24030	7	24145890	24147995	-
20027	1415966_a_at	Strain	Ndufv1	17995	19	3796295	3801515	-
20028	1453749_at	Strain	2610507I01Rik	72203	11	58923888	58925105	-
20029	1435948_at	Strain	5930418K15Rik	77106	13	45825942	45833503	-
20030	1453752_at	Strain	Rpl17	319195	18	75235707	75238565	+
20031	1457324_at	Strain	None	None	4	42079370	42080219	+
20032	1417714_x_at	Strain	Hba-a1	15122	11	32178468	32179287	+
20033	1426904_s_at	Strain	None	None	2	80013449	80051797	+
20034	1441100_at	Strain	Mbtd1	103537	11	93733496	93766406	+
20035	1436713_s_at	Strain	None	None	12	105022871	105023459	+
20036	1428655_at	Strain	1110018J12Rik	73825	17	86391811	86450187	+
20037	1457260_at	Strain	7530403E16Rik	100224	4	125640721	125641485	+
20038	1440573_at	Strain	None	None	13	100116358	100116927	-
20039	1423597_at	Strain	Atp8a1	11980	5	66388740	66614923	-
20040	1452593_a_at	Strain	Tceb1	67923	1	16827250	16842139	-
20041	1456746_a_at	Strain	Cd99l2	171486	X	66088561	66161136	-
20042	1436967_at	Strain	Ankrd11	77087	8	122264618	122423554	-
20043	1426544_a_at	Strain	Ttc14	67120	3	33243957	33251783	+
20044	1456947_at	Strain	Pafah1b1	18472	11	74399613	74450328	-
20045	1437536_at	Strain	Fkrp	243853	7	13760291	13767676	-
20046	1439578_at	Strain	Lsm11	72290	11	45683917	45697772	-
20047	1439170_at	Strain	None	None	8	32209525	32210204	+
20048	1436506_a_at	Strain	1110008H02Rik	73824	1	10047073	10049162	-
20049	1415690_at	Strain	Mrpl27	94064	11	94474878	94481174	+
20050	1428083_at	Strain	2310043N10Rik	66961	19	5631092	5633434	-
20051	1418908_at	Strain	Pam	18484	1	97601180	97757218	-
20052	1432646_a_at	Strain	2900097C17Rik	347740	6	118152376	118153424	+
20053	1437128_a_at	Strain	A630033E08Rik /// C730040L01Rik	240041	17	20768707	20772918	-
20054	1433497_at	Strain	Aqr	11834	2	113615012	113689159	-

20055	1439650_at	Strain	Rtn4	68585	11	29587737	29637706	+
20056	1437537_at	Strain	Casp9	12371	4	140674873	140697236	+
20057	1415844_at	Strain	Syt4	20983	18	31677574	31687445	-
20058	1443905_at	Strain	None	None	5	128723621	128724771	+
20059	1453960_a_at	Strain	Capzb	12345	4	138120169	138172527	+
20060	1435163_at	Strain	9030612M13Rik	208292	17	30596599	30610894	-
20061	1418462_at	Strain	Exosc9	50911	3	36016246	36029330	+
20062	1439069_a_at	Strain	4933439C20Rik	236604	11	3020573	3026734	+
20063	1418427_at	Strain	Kif5b	16573	18	6245790	6284051	-
20064	1438462_x_at	Strain	None	None	4	128741972	128742465	-
20065	1434362_at	Strain	AW550831	98715	1	156331330	156332635	-
20066	1423204_at	Strain	Tm9sf4	99237	2	152618237	152667399	+
20067	1444057_at	Strain	Ubxd2	67812	1	128087153	128120583	+
20068	1442886_at	Strain	1500010G04Rik	68965	6	49394626	49398413	-
20069	1436617_at	Strain	Cetn4	207175	3	36772225	36776044	-
20070	1451447_at	Strain	Cuedc1	103841	11	87827241	87920907	+
20071	1449262_s_at	Strain	Lin7c	22343	2	109514665	109524724	+
20072	1428738_a_at	Strain	D14Ertd449e	66039	14	24299743	24302783	-
20073	1455217_at	Strain	Lrig2	269473	3	103881767	103882395	-
20074	1452426_x_at	Strain	None	None	NONE	NONE	NONE	NONE
20075	1456685_at	Strain	Nsg2	18197	11	31895253	31953996	+
20076	1429729_at	Strain	Syt11	229521	3	88489566	88516404	-
20077	1455550_x_at	Strain	None	None	2	163081580	163082102	-
20078	1448959_at	Strain	Ndufs4	17993	13	110702711	110802452	-
20079	1435514_at	Strain	Lztfl1	93730	9	123721311	123741507	-
20080	1452825_at	Strain	5330410G16Rik	67937	8	69638900	69642391	-
20081	1418887_a_at	Strain	D11Wsu99e	28081	11	113482413	113505125	-
20082	1440682_at	Strain	Odz4	23966	7	90212889	90948341	+
20083	1445561_at	Strain	B130020M22Rik	320217	10	119196017	119196541	+
20084	1451993_at	Strain	9130404D08Rik	74549	8	69170702	69194854	-
20085	1448368_at	Strain	Dctn6	22428	8	32891565	32909677	-
20086	1443881_at	Strain	Pofut1	140484	2	152698491	152724222	+
20087	1435426_s_at	Strain	4933439C20Rik /// Pisd	236604	11	3020573	3026734	+
20088	1453206_at	Strain	Acad9	229211	3	35519660	35569683	+
20089	1439195_at	Strain	None	None	13	87636579	87637361	+
20090	1426631_at	Strain	C330017I15Rik	78697	5	22205737	22247905	-
20091	1459726_at	Strain	Ptprn2	19276	12	112490374	113040421	+
20092	1453278_a_at	Strain	Rsnl2	78785	17	69558453	69633262	+
20093	1419612_at	Strain	4632415L05Rik	70808	3	19236160	19237248	+
20094	1429093_at	Strain	Ddi2	68817	4	140561546	140564869	-
20095	1448715_x_at	Strain	Ccrn4l /// Iap /// LOC280487 /// LOC432899	12457	3	50820528	50883543	+
20096	1417185_at	Strain	Ly6a	110454	15	75027582	75030593	-
20097	1428692_at	Strain	1110033O09Rik	68695	7	74143304	74146265	+
20098	1428437_at	Strain	2700023B17Rik	67070	7	29498435	29543405	-
20099	1428417_at	Strain	3110050N22Rik	67306	3	7478034	7528335	+
20100	1439882_at	Strain	None	None	7	122858919	122859373	+
20101	1452726_a_at	Strain	1110061L23Rik	68845	7	39230940	39236606	+
20102	1452696_a_at	Strain	4933439C10Rik	74476	11	59231291	59236892	+
20103	1455316_x_at	Strain	Ccrn4l	12457	3	50820528	50883543	+
20104	1428568_at	Strain	B230217C12Rik	68127	11	97661980	97664130	+
20105	1429502_at	Strain	Stch	110920	16	74838985	74850259	-
20106	1437142_a_at	Strain	Pigo	56703	4	42933560	42941678	-
20107	1438969_x_at	Strain	Dhx30	72831	9	110110278	110141557	-
20108	1423000_a_at	Strain	Dgke	56077	11	88861819	88881472	-
20109	1437538_at	Strain	2610002F03Rik	72091	2	26569338	26570141	-
20110	1440296_at	Strain	Gm994	381345	1	178099620	178124237	+
20111	1439059_at	Strain	BC031748	245622	X	130594568	130622430	+
20112	1421163_a_at	Strain	Nfia	18027	4	96752972	97087465	+
20113	1444164_at	Strain	Fkbp3	30795	12	61840122	61851631	-
20114	1458065_at	Strain	Rere	68703	4	148898702	149114159	+
20115	1440498_at	Strain	Rabif	98710	1	134344923	134358148	+
20116	1437558_at	Strain	B130021B11Rik	320860	15	12838709	12840317	-

20117	1452799_at	Strain	2310009E04Rik	75578	4	94554575	94796641	+
20118	1432589_at	Strain	None	None	2	160225070	160226730	+
20119	1431020_a_at	Strain	Fgfr1op2	67529	6	147498625	147519880	+
20120	1420524_a_at	Strain	Masp2 /// LOC547378	17175	4	147094908	147107844	+
20121	1448419_at	Strain	Pop4	66161	7	33430376	33438904	-
20122	1453246_at	Strain	Rab39b	67790	X	70232713	70238859	-
20123	1428370_at	Strain	1500011B03Rik	66236	5	113917783	113923550	-
20124	1440248_at	Strain	Casc4	319996	2	121380983	121449712	+
20125	1432391_at	Strain	Ccdc21	70012	4	133090758	133147949	-
20126	1422591_at	Strain	Tceb3	27224	4	134884627	134902906	-
20127	1433665_at	Strain	Vps41	218035	13	18192301	18341778	+
20128	1441493_at	Strain	Rab6ip2	111173	6	120007511	120280559	-
20129	1434949_at	Strain	Armc8	74125	9	99381642	99469875	-
20130	1448891_at	Strain	Msr2	80891	3	86995152	87007982	-
20131	1458188_at	Strain	Dpysl2	12934	14	61332083	61398082	-
20132	1417210_at	Strain	Eif2s3y	26908	Y	347054	365036	+
20133	1442364_at	Strain	Mapk14	26416	17	26492551	26549631	+
20134	1429335_at	Strain	Snapc1	75627	12	70812924	70832758	+
20135	1459747_at	Strain	None	None	4	35091185	35091393	+
20136	1416245_at	Strain	Aurkaip1	66077	4	154324008	154325421	+
20137	1430660_at	Strain	5830417I10Rik	76022	3	420	48501	+
20138	1444147_at	Strain	Pcsk2	18549	2	143003115	143273220	+
20139	1452848_at	Strain	5930418K15Rik	77106		81196364	81197494	+
20140	1458376_at	Strain	B930025B16Rik	503851	14	114911692	114912335	+
20141	1439928_at	Strain	None	None	11	30119337	30119976	-
20142	1436239_at	Strain	Slc5a5	114479	8	70039215	70048437	-
20143	1423696_a_at	Strain	Psmd6	66413	14	12574333	12583052	-
20144	1452077_at	Strain	Ddx3y	26900	Y	598006	622997	-
20145	1446972_at	Strain	D15Wsu126e	28112	15	53308367	53308935	-
20146	1448822_at	Strain	Psmb6	19175	11	70251058	70253307	+
20147	1437358_at	Strain	Wdfy1	69368	1	80056762	80093054	-
20148	1445411_at	Strain	None	None	NONE	NONE	NONE	NONE
20149	1442255_at	Strain	5033414D02Rik	67759	19	28601276	28614470	-
20150	1419839_x_at	Strain	Prp19	28000	19	10091789	10102029	+
20151	1435523_s_at	Strain	2700089E24Rik	381820	6	133875346	133882217	+
20152	1442058_s_at	Strain	Psmc3ip	19183	11	100913244	100916490	-
20153	1457637_at	Strain	---	432977	15	93659677	93659981	-
20154	1455719_at	Strain	Tubb5	22154	17	33552072	33555475	-
20155	1444806_at	Strain	6720456B07Rik	101314	6	114172603	114184782	+
20156	1439299_at	Strain	None	None	11	30134727	30135502	-
20157	1453144_at	Strain	4933439C20Rik	236604	11	3020573	3026734	+
20158	1417063_at	Strain	C1qb	12260	4	135761402	135767434	-
20159	1458426_at	Strain	Kif1b	16561	4	147668683	147799980	-
20160	1420890_at	Strain	Hccs	15159	X	162910940	162919685	-
20161	1441172_at	Strain	None	None	1	38887870	38888631	-
20162	1422807_at	Strain	Arf5	11844	6	28470635	28473494	+
20163	1420862_at	Strain	Dctn4	67665	18	60751547	60784088	+
20164	1429822_at	Strain	4633401B06Rik	70828	3	120566391	120567466	-
20165	1416745_x_at	Strain	Uap1	107652	1	170076180	170109123	-
20166	1426905_a_at	Strain	Dnajc10	66861	2	80013538	80050631	+
20167	1425966_x_at	Strain	Ubc	22190	11	124553698	124555581	-
20168	1442148_at	Strain	Psip1	101739	4	82441872	82472582	-
20169	1450740_a_at	Strain	Mapre1	13589	2	153198313	153225162	+
20170	1435129_at	Strain	Ptp4a2	19244	4	128866586	128877088	+
20171	1428973_s_at	Strain	0610012D17Rik	66061	16	31229115	31238469	+
20172	1417705_at	Strain	Otub1	107260	19	6912051	6920129	-
20173	1423747_a_at	Strain	Pdk1	228026	2	71570967	71599744	+
20174	1427797_s_at	Strain	Ctse	13034	1	131488640	131525829	+
20175	1429452_x_at	Strain	4933439C20Rik	236604	11	3020573	3026734	+
20176	1451045_at	Strain	Syt13	80976	2	92619784	92660734	+
20177	1455342_at	Strain	A230083H22Rik	353211	19	16447395	16448035	+
20178	1435925_at	Strain	Git2	26431	5	113837037	113883117	-
20179	1420329_at	Strain	4930455C21Rik	76916	16	37316787	37341355	-

20180	1452731_x_at	Strain	LOC544986 /// LOC544991 /// LOC544997 /// B930046C15Rik /// LOC545001 /// LOC545005 /// LOC545007 /// LOC545008 /// 2610042L04Rik /// LOC545017	544986	14	3010193	3030911	+
20181	1458235_at	Strain	Fbxl17	50758	17	60753894	60800293	-
20182	1438989_s_at	Strain	B130021B11Rik	320860	15	12839586	12839830	-
20183	1428823_at	Strain	2310057G13Rik	69692	10	31353580	31368402	+
20184	1418148_at	Strain	Abhd1	57742	5	29409016	29414035	+
20185	1444377_at	Strain	Psmb2	26445	4	125704779	125736819	+
20186	1429337_at	Strain	2610301K12Rik	72477	2	128332164	128366477	+
20187	1456430_at	Strain	D3Ertd789e	51938	3	33256108	33288047	-
20188	1443158_at	Strain	Scmh1	29871	4	119469474	119552399	+
20189	1440278_at	Strain	None	None	5	114406747	114407677	-
20190	1426573_at	Strain	Me2	107029	18	74004269	74049621	-
20191	1447458_at	Strain	Siat4c	20443	9	34960623	35030854	-
20192	1427328_a_at	Strain	Clasp2	76499	9	113865413	113971281	+
20193	1426270_at	Strain	Smc5l1	226026	19	22446187	22511419	-
20194	1424784_at	Strain	1700029I01Rik /// LOC433791 /// LOC545705	433791	4	145055149	145074355	-
20195	1430332_a_at	Strain	Gusb	110006	5	129158844	129172652	-
20196	1460466_at	Strain	1700047I17Rik	73385	12	51918988	51936568	+
20197	1429073_at	Strain	2210015D19Rik	76508	11	5656916	5679497	+
20198	1425113_x_at	Strain	None	None	NONE	NONE	NONE	NONE
20199	1419423_at	Strain	Stab2	192188	10	86815870	86982604	-
20200	1446799_at	Strain	None	None	2	67473789	67474327	+
20201	1457458_at	Strain	None	None	7	13351413	13352345	+
20202	1457797_at	Strain	AI605517	106622	17	79337776	79338412	-
20203	1449076_x_at	Strain	AL024210	104923	12	25240447	25247415	+
20204	1428336_at	Strain	Agpat4	68262	17	10758734	10859090	+
20205	1438678_at	Strain	1500011K16Rik	67885	2	127305241	127306341	-
20206	1440695_at	Strain	None	None	17	21087116	21087528	-
20207	1446332_at	Strain	Pcdhga12	93724	18	37969124	37969796	+
20208	1454112_a_at	Strain	2410007P03Rik	66296	2	120123285	120133662	+
20209	1419356_at	Strain	Klf7	93691	1	64328681	64414407	-
20210	1447750_x_at	Strain	1110061L23Rik	68845	7	39230940	39236606	+
20211	1436670_x_at	Strain	1700019G17Rik	75541	6	86328677	86334511	-
20212	1459942_at	Strain	ORF34	207375	X	144878618	145004200	+
20213	1452730_at	Strain	1110033J19Rik	66184	6	149288478	149289420	+
20214	1424903_at	Strain	Jarid1d	20592	Y	234392	280253	+
20215	1455219_at	Strain	1110030E23Rik	68681	7	120739199	120740167	-
20216	1458920_at	Strain	Stx6	58244	1	155049479	155094254	+
20217	1417600_at	Strain	Slc15a2	57738	16	35567606	35602387	-
20218	1431380_at	Strain	5730409L17Rik	70499	6	8695789	8697330	-
20219	1422903_at	Strain	Ly86	17084	13	36887185	36960718	+
20220	1427437_at	Strain	2610203C20Rik	70455	9	41538340	41539795	+
20221	1418245_a_at	Strain	Rbm9	93686	15	77134294	77358885	-
20222	1433815_at	Strain	MGI:1923321	76071	5	35557428	35631807	+
20223	1443541_at	Strain	None	None	3	37177674	37178229	-
20224	1446750_at	Strain	Impact	16210	18	13160301	13180997	+
20225	1457551_at	Strain	Acvr1	11477	2	58371977	58441698	-
20226	1416946_a_at	Strain	Acaa1 /// MGC29978	113868	9	119335555	119344556	+
20227	1447206_at	Strain	Arhgap21	71435	2	20889950	21009731	-
20228	1441643_at	Strain	38414	320253	18	56982085	57143467	-
20229	1418141_at	Strain	Dcx	13193	X	137295888	137373265	-
20230	1436644_x_at	Strain	Tmem25	71687	9	44782634	44788071	-
20231	1426988_at	Strain	Klhdc5	232539	6	148018350	148040641	+
20232	1434585_at	Strain	Tulp4	68842	17	6045120	6146999	+

20233	1452677_at	Strain	Pnpt1	71701	11	29025541	29056618	+
20234	1433816_at	Strain	Mcart1	230125	4	45311988	45320724	-
20235	1454772_at	Strain	Ascc3l1	320632	2	126722257	126754304	+
20236	1425263_a_at	Strain	Mbp	17196	18	82642914	82753380	+
20237	1426438_at	Strain	Ddx3y	26900	Y	598006	622997	-
20238	1416402_at	Strain	Abcb10	56199	8	123239531	123269924	-
20239	1438249_at	Strain	Usp7	252870	16	8364073	8389916	-
20240	1442279_at	Strain	Epc1	13831	18	6478658	6558794	-
20241	1427338_at	Strain	Crocc	230872	4	139897898	139934922	-
20242	1421036_at	Strain	Npas2	18143	1	39491116	39659103	+
20243	1435284_at	Strain	Rtn4	68585	11	29587737	29637706	+
20244	1455208_at	Strain	Pex19	19298	1	172055691	172064699	+
20245	1416743_at	Strain	Uap1	107652	1	170076180	170109123	-
20246	1424324_at	Strain	Esco1	77805	18	10611852	10655599	-
20247	1445214_at	Strain	None	None	4	146008858	146078637	+
20248	1439058_at	Strain	Sfpq	71514	4	126048448	126057909	+
20249	1429410_at	Strain	Eny2	223527	15	44373735	44383309	+
20250	1458203_at	Strain	Spire1	68166	18	67718995	67783507	-
20251	1431328_at	Strain	Ppp1cb	19046	5	30917485	30950978	+
20252	1424778_at	Strain	D10Ucla1	28193	10	67048137	67134030	-
20253	1443638_at	Strain	None	None	15	30186958	30187416	+
20254	1427998_at	Strain	2600001B17Rik	268490	11	101984576	102006343	-
20255	1438832_x_at	Strain	Dhx30	72831	9	110110278	110141557	-
20256	1428302_at	Strain	Mrpl48	52443	7	94655385	94714672	-
20257	1457259_at	Strain	None	None		61687581	61687804	+
20258	1431880_at	Strain	3100003M19Rik	73082	18	16792263	16794254	-
20259	1448580_at	Strain	Glg1	20340	8	110455266	110556791	-
20260	1445670_at	Strain	None	None	4	115498181	115498443	+
20261	1441362_at	Strain	None	None	14	10427614	10428551	+
20262	1435119_at	Strain	None	None	3	87776444	87776731	+
20263	1420535_a_at	Strain	6330412F12Rik	53312	5	23149873	23174275	+
20264	1443529_at	Strain	Rutbc2	52850	5	112319917	112387408	-
20265	1434353_at	Strain	Sfmbt2	353282	2	10510061	10511262	+
20266	1457557_at	Strain	A330076H08Rik	320026	NONE	NONE	NONE	NONE
20267	1440571_at	Strain	Eif4g3	230861	4	136874731	137087744	+
20268	1426277_at	Strain	C730025P13Rik	227615	2	25187616	25188453	+
20269	1455657_at	Strain	2610207I05Rik	233789	7	112010248	112015656	-
20270	1442654_at	Strain	None	None	NONE	NONE	NONE	NONE
20271	1438759_x_at	Strain	None	None	NONE	NONE	NONE	NONE
20272	1441263_a_at	Strain	A930005H10Rik	68161	3	114652097	114658964	-
20273	1432827_x_at	Strain	Ubc	22190	11	124553698	124555581	-
20274	1455744_at	Strain	LOC547127	547127		81146073	81147601	+
20275	1456837_at	Strain	A830007L07Rik	233879	7	120995718	120999313	-
20276	1421179_at	Strain	Ttyh2	117160	11	114496618	114540296	+
20277	1420941_at	Strain	Rgs5	19737	1	169589678	169627703	+
20278	1436362_x_at	Strain	Ccrn4l /// LOC433439 /// LOC436521 /// LOC545596 /// LOC546093 /// LOC547395	12457	3	50820528	50883543	+
20279	1456080_a_at	Strain	Tde1	26943	2	163080242	163102100	-
20280	1441339_at	Strain	Chd9	109151	8	90115372	90161290	+
20281	1445226_at	Strain	BC023969	407828	15	33221551	33222192	+
20282	1422510_at	Strain	Ctdspl	69274	9	118920803	119038259	+
20283	1442406_at	Strain	9230104K21Rik	77688	4	45322134	45322841	-
20284	1440078_at	Strain	Brd4	57261	17	30005231	30094101	-
20285	1440989_at	Strain	None	None	6	72144980	72145935	-
20286	1417826_at	Strain	Akr1e1	56043	13	4477155	4496595	-
20287	1433899_x_at	Strain	Tgfb1i4	21807	14	70758316	70850256	+
20288	1439631_at	Strain	Zcchc11	230594	4	107418943	107518318	+
20289	1456849_at	Strain	Usp48	170707	4	136475016	136537437	+
20290	1431300_at	Strain	Sgip1	73094	4	101719268	101930082	+
20291	1442824_at	Strain	8030497I03Rik	399593	2	154269180	154269985	+
20292	1435658_at	Strain	Slc27a1	26457	8	70718241	70736379	+

20293	1443053_at	Strain	None	None	4	75496559	75497252	-
20294	1447047_at	Strain	2610203C20Rik	70455	9	41557881	41558415	+
20295	1444195_at	Strain	1110007A06Rik	68477	6	71723228	71772465	-
20296	1449190_a_at	Strain	Entpd4	67464	14	63870758	63900260	+
20297	1460045_at	Strain	Cdh7	241201	1	109972834	110128098	+
20298	1419115_at	Strain	5430428G01Rik	66789	3	120073487	120141148	+
20299	1458003_at	Strain	Zfp398	272347	6	47960121	47992931	+
20300	1416267_at	Strain	Scoc	56367	8	82691600	82699587	-
20301	1437297_at	Strain	Chd8	67772	14	47298256	47322726	-
20302	1422279_at	Strain	Fv1	14349	4	146361272	146364396	+
20303	1451799_at	Strain	2610528H13Rik	67179	14	60364796	60394022	+
20304	1443050_at	Strain	BC032265	238024	11	121242520	121251857	+
20305	1430593_at	Strain	1700041C02Rik	73332	4	118281798	118374408	-
20306	1415710_at	Strain	BC038311	231430	5	89512113	89521416	-
20307	1442124_at	Strain	AI450326	100132	4	118178807	118179243	-
20308	1457945_at	Strain	Eif2s3y	26908	Y	347054	365036	+
20309	1449048_s_at	Strain	Rab4a	19341	8	123093097	123122359	+
20310	1426439_at	Strain	Ddx3y	26900	Y	598006	622997	-
20311	1428293_at	Strain	2310022M17Rik	69556	11	31559934	31566451	-
20312	1441238_at	Strain	LOC545754	545754	5	64452218	64499685	-
20313	1429763_at	Strain	Cnih4	98417	1	181104746	181120255	+
20314	1417227_at	Strain	Mccc1	72039	3	35413327	35454130	-
20315	1429220_at	Strain	2810443J12Rik	67228	2	24894607	24905633	+
20316	1445341_at	Strain	None	None	12	18741687	18742299	+
20317	1430877_at	Strain	8030425K09Rik	77207	7	90627151	90627890	+
20318	1428588_a_at	Strain	2810443J12Rik	67228	2	24894607	24905633	+
20319	1437901_a_at	Strain	Vps41	218035	13	18192301	18341778	+
20320	1440830_at	Strain	Gpr116	224792	17	40866790	40962389	+
20321	1416495_s_at	Strain	Ndufs5	170658	4	122739822	122743439	-
20322	1444738_at	Strain	None	None	6	143917748	143918451	-
20323	1447754_x_at	Strain	Thap4	67026	1	93532881	93578284	-
20324	1427229_at	Strain	Hmgcr	15357	13	92840662	92862190	-
20325	1443346_at	Strain	2700007P21Rik	212772	2	106588216	106594178	-
20326	1430047_at	Strain	Brctd1	72573	13	73164743	73194647	-
20327	1428760_at	Strain	Snapc3	77634	4	82403941	82453789	+
20328	1437923_at	Strain	AI314760	106885	18	61812292	61815574	+
20329	1441181_at	Strain	Rora	19883	9	69474948	69510983	+
20330	1443511_at	Strain	Rora	19883	9	69474948	69510983	+
20331	1426471_at	Strain	Zfp52	22710	17	19404937	19434146	+
20332	1428174_x_at	Strain	Khsrp	16549	17	54710978	54721434	-
20333	1457231_at	Strain	Hif1a	15251	12	70756396	70795257	+
20334	1441050_at	Strain	5730526G10Rik	70656	12	48407202	48407846	-
20335	1438301_at	Strain	Ddef1	13196	15	64104369	64365898	-
20336	1442447_at	Strain	BC025031	208987	11	5311578	5312772	-
20337	1443212_at	Strain	Large	16795	8	71965827	72364115	-
20338	1426484_at	Strain	Ubxd2	67812	1	128087153	128120583	+
20339	1428820_at	Strain	Mapre1	13589	2	153198313	153225162	+
20340	1456500_at	Strain	Aph1b	208117	9	66900639	66920575	-
20341	1437018_at	Strain	Pnma2	239157	14	61446017	61450669	+
20342	1417697_at	Strain	Soat1	20652	1	156336595	156379733	-
20343	1440455_at	Strain	AI848599	100222	4	137340821	137341388	+
20344	1460151_at	Strain	None	None	NONE	NONE	NONE	NONE
20345	1425191_at	Strain	9430098E02Rik	77090	8	70521184	70522988	+
20346	1439363_at	Strain	1200014J11Rik	66874	11	72773538	72805874	+
20347	1450288_at	Strain	Cdh6	12563	15	12840386	12897433	-
20348	1458186_at	Strain	None	None	4	107714022	107714454	-
20349	1440464_at	Strain	Elavl1	15568	8	3643294	3667872	-
20350	1457762_at	Strain	Ttc15	217449	12	25256255	25315622	-
20351	1415903_at	Strain	Slc38a1	105727	15	96641973	96691719	-
20352	1454607_s_at	Strain	Psat1	107272	19	15129232	15149125	-
20353	1416914_s_at	Strain	Mtvr2	17826	19	5516877	5518438	-
20354	1437710_x_at	Strain	1700021P22Rik	75538	7	38571874	38576768	+
20355	1441006_at	Strain	Dus4l	71916	12	28214747	28229518	-
20356	1416247_at	Strain	Dctn3	53598	4	41853650	41862010	-
20357	1452230_at	Strain	None	None	2	80013449	80051797	+

20358	1437579_at	Strain	Nek2	18005	1	191557109	191568589	+
20359	1415685_at	Strain	Mtif2	76784	11	29421255	29440069	+
20360	1456262_at	Strain	Rbm5	83486	9	107808484	107838963	-
20361	1437878_s_at	Strain	D3Ertd789e	51938	3	33256108	33288047	-
20362	1416403_at	Strain	Abcb10	56199	8	123239531	123269924	-
20363	1457936_at	Strain	Mapk8	26419	14	31516453	31580724	-
20364	1459698_at	Strain	None	None	7	91379614	91380035	+
20365	1458240_at	Strain	Baiap1	14924	6	94131999	94737301	-
20366	1434548_at	Strain	Tde1	26943	2	163080242	163102100	-
20367	1444772_at	Strain	Smyd3	69726	1	178910022	179472249	-
20368	1453018_at	Strain	Nvl	67459	1	181047238	181097972	-
20369	1438177_x_at	Strain	Entpd4	67464	14	63870758	63900260	+
20370	1433895_at	Strain	2310003P10Rik	69470	2	126761828	126774617	+
20371	1442548_at	Strain	Crim1	50766	17	76155816	76156378	+
20372	1420342_at	Strain	Gdap10	14546	12	29428074	29433121	+
20373	1416090_at	Strain	Pdhb	68263	14	5833813	5841767	-
20374	1455389_s_at	Strain	2310051F07Rik	108745	15	1069902	1071573	+
20375	1442384_at	Strain	None	None		70261410	70262475	-
20376	1438363_at	Strain	LOC434128	434128	7	13895593	13898209	+
20377	1432332_a_at	Strain	Nudt19	110959	7	30711030	30719686	-
20378	1420287_at	Strain	None	None	8	83678088	83678241	+
20379	1456056_a_at	Strain	D6Wsu116e	28006	6	116645592	116700200	+
20380	1439886_at	Strain	None	None	NONE	NONE	NONE	NONE
20381	1425764_a_at	Strain	Bcat2	12036	7	39652923	39672489	+
20382	1421086_at	Strain	Per3	18628	4	149497074	149536951	-
20383	1426229_s_at	Strain	Kras	16653	6	146066254	146096662	-
20384	1445725_at	Strain	4931417A20	211936	2	104656658	104704491	+
20385	1430196_at	Strain	8430408J09Rik	66804	5	110159095	110160762	+
20386	1448938_at	Strain	Rpa3	68240	6	8231143	8234319	-
20387	1445032_at	Strain	Dapk1	69635	13	59260847	59420940	+
20388	1418693_at	Strain	Hnrpc /// LOC329893	15381	14	47175503	47204310	-
20389	1438092_x_at	Strain	H2afz /// LOC544879	51788	3	136753528	136755851	+
20390	1424893_at	Strain	Ndel1	83431	11	68547092	68578720	-
20391	1452295_at	Strain	Tmepai	65112	2	172688256	172737000	-
20392	1441716_at	Strain	None	None	8	32199713	32200245	+
20393	1429902_at	Strain	5830443J22Rik	76056	9	72045348	72046508	-
20394	1442680_at	Strain	Ncam1	17967	9	49555319	49607941	-
20395	1429453_a_at	Strain	Mrpl55	67212	11	58928146	58931631	+
20396	1442408_at	Strain	2010305B15Rik	72110	2	165529263	165530067	-
20397	1434833_at	Strain	Map4k2	26412	19	6130040	6142317	+
20398	1455600_at	Strain	Rps3	27050	7	93560951	93572082	-
20399	1445263_at	Strain	None	None	NONE	NONE	NONE	NONE
20400	1438575_a_at	Strain	2900056M20Rik	72997	NONE	NONE	NONE	NONE
20401	1430556_at	Strain	Spag9	70834	11	93817190	93944505	+
20402	1431506_s_at	Strain	Ppih	66101	4	118258916	118279389	-
20403	1442624_at	Strain	C920008N22Rik	319366	7	91698072	91698421	+
20404	1446730_at	Strain	None	None	4	138458968	138459428	+
20405	1416234_at	Strain	AA959742	98238	11	94450911	94466303	+
20406	1421402_at	Strain	Mta3	116871	17	81539641	81648553	+
20407	1425343_at	Strain	Hdhd3	72748	4	61590245	61593391	-
20408	1442513_at	Strain	None	None	13	3478100	3478978	-
20409	1419506_at	Strain	Ggps1	14593	13	13494995	13505951	-
20410	1426417_at	Strain	Yipf4	67864	17	72297116	72307880	+
20411	1452686_s_at	Strain	D4Ertd196e	52174	4	132226946	132238686	-
20412	1459827_x_at	Strain	Hps1	192236	19	42299619	42323051	-
20413	1451490_at	Strain	Lyplal1	226791	1	185586332	185615762	-
20414	1426896_at	Strain	Zfp191	59057	18	24240763	24249267	-
20415	1438238_at	Strain	2010315B03Rik	72071		43771107	43771677	-
20416	1438155_x_at	Strain	Pigo	56703	4	42933560	42941678	-
20417	1429331_at	Strain	4632427E13Rik	68186	7	86778897	86781465	-
20418	1429846_at	Strain	9030411K21Rik	71557	9	70690029	70691237	+
20419	1423495_at	Strain	Decr2	26378	17	23874381	23883334	-
20420	1452318_a_at	Strain	Hspa1b	15511	17	32660211	32661550	-

20421	1423180_at	Strain	Kcnb1	16500	2	166560111	166645532	-
20422	1446207_at	Strain	None	None	18	64510257	64510890	+
20423	1440862_at	Strain	5930418K15Rik	77106		81150956	81151474	-
20424	1415748_a_at	Strain	Dctn5	59288	7	116010930	116026902	+
20425	1431406_at	Strain	Agxt2l1	71760	3	129547666	129565839	+
20426	1416665_at	Strain	Coq7	12850	7	112400919	112409170	-
20427	1452265_at	Strain	Clasp1	76707	1	118312452	118360897	+
20428	1418386_at	Strain	2510005D08Rik	68043	14	52081084	52106667	-
20429	1452187_at	Strain	Rbm5	83486	9	107808484	107838963	-
20430	1444535_at	Strain	Brd4	57261	17	30005231	30094101	-
20431	1423626_at	Strain	Dst	13518	1	34280938	34577763	+
20432	1452690_at	Strain	Khsrp	16549	17	54710978	54721434	-
20433	1427820_at	Strain	None	None		23266426	23267697	+
20434	1438862_at	Strain	A630005I04Rik	320743	7	107093589	107097051	+
20435	1434541_x_at	Strain	None	None	4	128741974	128742810	-
20436	1440139_at	Strain	None	None	18	65170467	65170733	+
20437	1434721_at	Strain	D5Ertd585e	71782	5	109281825	109307432	+
20438	1419179_at	Strain	Txnl4	27366	18	80330360	80347046	+
20439	1456955_at	Strain	5830417C01Rik	78825	1	178140297	178205198	+
20440	1416499_a_at	Strain	Dctn6	22428	8	32891565	32909677	-
20441	1440416_at	Strain	Usp46	69727	5	72834085	72900782	-
20442	1448406_at	Strain	Cri1	58521	2	125186948	125188636	+
20443	1455452_x_at	Strain	AI449310	101584	7	91504850	91505827	+
20444	1439272_at	Strain	A830039H10Rik	209707	5	44458346	44613918	-
20445	1446772_at	Strain	None	None	15	32252017	32252626	+
20446	1438891_at	Strain	Catnd2	18163	15	30163743	31021141	+
20447	1432003_a_at	Strain	Rnf41	67588	10	128148476	128178259	+
20448	1459376_at	Strain	None	None	10	14005933	14006582	+
20449	1448367_at	Strain	Sdf4	20318	4	154485276	154505972	+
20450	1446104_at	Strain	None	None	6	113473785	113474399	-
20451	1454684_at	Strain	Bbs7	71492	3	36036749	36076979	-
20452	1460025_at	Strain	Lrig2	269473	3	103884911	103892007	-
20453	1442837_at	Strain	None	None	4	75448689	75449905	-
20454	1428343_at	Strain	Rcor3	214742	1	191839449	191876966	-
20455	1431382_a_at	Strain	Rsnl2	78785	17	69558453	69633262	+
20456	1449401_at	Strain	C1qg	12262	4	135771067	135774130	-
20457	1444517_at	Strain	Gm872	380653	10	92744601	92861446	-
20458	1437481_at	Strain	Spdy1	70891	17	69327615	69364244	+
20459	1436222_at	Strain	Gas5	14455	1	160940158	160942671	+
20460	1449635_at	Strain	Prp19	28000	19	10091789	10102029	+
20461	1442400_at	Strain	---	432977	15	93660269	93661107	-
20462	1439929_at	Strain	Mfhas1	52065	8	34390870	34482521	+
20463	1443109_at	Strain	5830417C01Rik	78825	1	178140297	178205198	+
20464	1427580_a_at	Strain	Rian	75745	12	105123125	105129266	+
20465	1434493_at	Strain	1810022K09Rik	69126	3	14603141	14606511	-
20466	1441375_at	Strain	Lrig1	16206	6	95066417	95161798	-
20467	1439197_at	Strain	Pik4cb	107650	3	94462567	94494420	+
20468	1442365_at	Strain	Rtn3	20168	19	7139746	7197134	-
20469	1457970_at	Strain	Actr1a	54130	19	45924156	45943077	-
20470	1448801_a_at	Strain	Timm44	21856	8	3614696	3630853	-
20471	1451187_at	Strain	0610037P05Rik	66086	16	13035040	13053128	-
20472	1417466_at	Strain	None	None	1	169628085	169629980	+
20473	1453755_at	Strain	Lsm11	72290	11	45683917	45697772	-
20474	1439546_at	Strain	4933417O08Rik	71146	19	41800115	41814754	+
20475	1423170_at	Strain	Taf7	24074	18	37864624	37867888	-
20476	1438526_at	Strain	Gpr158	241263	2	21409577	21872554	+
20477	1422126_a_at	Strain	Nudt13	67725	14	18656034	18678071	+
20478	1444037_at	Strain	Lman1	70361	18	66213875	66235740	-
20479	1423457_at	Strain	Slc35a5	74102	16	44022615	44041681	-
20480	1424717_at	Strain	Mis12	67139	11	70745269	70752792	+
20481	1455862_at	Strain	9630054F20Rik	327900	11	32350166	32413503	+
20482	1434816_at	Strain	Vps33a	77573	5	122700612	122742738	-
20483	1418203_at	Strain	Pmaip1	58801	18	66687938	66694892	+
20484	1424628_a_at	Strain	1500032D16Rik	78330	17	29329211	29340379	+
20485	1424730_a_at	Strain	Slc15a2	57738	16	35567606	35602387	-

20486	1431725_at	Strain	Fmn2	54418	1	174435022	174755223	+
20487	1429963_at	Strain	2610021I23Rik	70413	9	75545906	75547570	-
20488	1457465_at	Strain	D430043L16Rik	208431	X	4887205	4947347	+
20489	1440046_at	Strain	BC031748	245622	X	130594568	130622430	+
20490	1423487_at	Strain	1200020A08Rik	56724	17	84882400	84892614	+
20491	1440871_at	Strain	Magi1	14924	6	94131999	94737301	-
20492	1452508_x_at	Strain	2610009E16Rik	69202	NONE	NONE	NONE	NONE
20493	1418667_at	Strain	2410002O22Rik	66975	13	100361503	100396609	-
20494	1440475_at	Strain	AW011738	100382	NONE	NONE	NONE	NONE
20495	1416632_at	Strain	Mod1	17436	9	86923622	87038833	-
20496	1455965_at	Strain	None	None	1	171188791	171189477	+
20497	1427126_at	Strain	Hspa1b	15511	17	32660211	32661550	-
20498	1449716_s_at	Strain	Nrd1	230598	4	107959710	108020676	+
20499	1431241_at	Strain	Chchd3	66075	6	32872201	33140615	-
20500	1416865_at	Strain	Fgd1	14163	X	144581628	144623494	+
20501	1442956_at	Strain	Ppp1r13b	21981	12	107308924	107388462	-
20502	1459224_at	Strain	None	None	12	107367073	107367440	-
20503	1456923_at	Strain	Trpm3	226025	19	21686135	22227805	+
20504	1429968_at	Strain	2310047L11Rik	70280	12	68925069	68926413	+
20505	1424508_at	Strain	Ttc5	219022	14	45870095	45880135	-
20506	1450848_at	Strain	Dap3	65111	3	88667305	88694146	-
20507	1423842_a_at	Strain	Rnf41	67588	10	128148476	128178259	+
20508	1429257_at	Strain	Gtl2	17263	12	105023823	105039917	+
20509	1457969_at	Strain	Rabif	98710	1	134344923	134358148	+
20510	1442402_at	Strain	Sh3md2	59009	8	60271818	60461252	+
20511	1424749_at	Strain	Wdfy1	69368	1	80056762	80093054	-
20512	1439312_at	Strain	None	None	6	106160316	106160743	-
20513	1451301_at	Strain	Tmod2	50876	9	75710299	75748747	-
20514	1458989_at	Strain	Limk1	16885	5	133669544	133702806	-
20515	1446140_at	Strain	Pcm1	18536	8	40177407	40269978	+
20516	1458994_at	Strain	Csnk1g3	70425	18	54125626	54174530	+
20517	1449113_at	Strain	5330440M15Rik	77110	4	115516632	115552787	+
20518	1434643_at	Strain	Tbl1x	21372	X	72294199	72320920	+
20519	1454712_at	Strain	Mcart1	230125	4	45311988	45320724	-
20520	1440444_at	Strain	Fads1	76267	19	9379470	9393371	+
20521	1457262_at	Strain	2610207I05Rik	233789	15	9030885	9032009	+
20522	1428004_at	Strain	3300001G02Rik	78372	11	32100209	32103778	+
20523	1450924_at	Strain	Hdgfrp3	29877	7	75691975	75742185	-
20524	1431233_at	Strain	Cnnm4	94220	1	36766575	36801553	+
20525	1458903_at	Strain	A230057G18Rik	330166	5	111288191	111298937	-
20526	1417327_at	Strain	Cav2	12390	6	17264785	17272455	+
20527	1449490_at	Strain	Mbd4	17193	6	116279963	116291256	-
20528	1455574_at	Strain	9430023P16Rik	226517	1	152721479	152787106	-
20529	1451022_at	Strain	Lrp6	16974	6	135206976	135323616	-
20530	1424668_a_at	Strain	Cutl1	13047	5	135261627	135499343	-
20531	1432052_at	Strain	Exosc1	66583	19	41468091	41477666	-
20532	1442725_at	Strain	AI663975	103819	11	110336741	110337194	+
20533	1440125_at	Strain	None	None	13	64219825	64220090	-
20534	1438041_at	Strain	Pde7a	18583	3	18563269	18602308	-
20535	1452192_at	Strain	BC053440	234344	8	65955121	65999681	+
20536	1445387_at	Strain	Senp6	215351	9	80272603	80350480	+
20537	1445540_at	Strain	Dnm3	103967	1	161896669	162387092	-
20538	1435491_at	Strain	AI875089	102177	NONE	NONE	NONE	NONE
20539	1459464_at	Strain	Macf1	11426	4	122378001	122711301	-
20540	1441502_at	Strain	Lrfn5	238205	12	58254745	58585291	+
20541	1453068_at	Strain	Prdm2	110593	4	142024189	142128244	-
20542	1426293_at	Strain	6330581L23Rik	233056	7	25230836	25245657	+
20543	1424101_at	Strain	Hnrpl	15388	7	24216779	24228110	+
20544	1456634_at	Strain	9830001H06Rik	320706	2	156472766	156544977	-
20545	1426125_a_at	Strain	Casp9	12371	4	140674873	140697236	+
20546	1443262_at	Strain	Mrps14	64659	1	160099907	160105833	+
20547	1457180_at	Strain	2900024P20Rik	208440	13	9260573	9652519	+
20548	1428137_at	Strain	Arl10c	67166	6	109286299	109326908	+
20549	1455237_at	Strain	Usp36	72344	11	118078029	118111324	-
20550	1441858_at	Strain	None	None	NONE	NONE	NONE	NONE

20551	1438043_at	Strain	Hmg20a	66867	9	56527993	56606058	+
20552	1444218_at	Strain	D19Ertd737e	76539	19	59801013	59829124	-
20553	1446284_at	Strain	Mtss1	211401	15	58954404	59092928	-
20554	1418898_at	Strain	Lin7c	22343	2	109514665	109524724	+
20555	1452012_a_at	Strain	Exosc1	66583	19	41468091	41477666	-
20556	1456351_at	Strain	Brd8	78656	18	34822848	34847583	-
20557	1445020_at	Strain	None	None	11	29890094	29890949	-
20558	1429627_at	Strain	Ppil3	70225	1	58735867	58750356	-
20559	1455213_at	Strain	4930488E11Rik	399591	X	130500205	130502929	-
20560	1457754_at	Strain	4930430F08Rik	68281	10	100478624	100495565	-
20561	1454659_at	Strain	Dctd	320685	8	47080095	47111758	+
20562	1418535_at	Strain	Rgl1	19731	1	152402014	152509595	-
20563	1452664_a_at	Strain	Tm7sf3	67623	6	147522941	147555233	-
20564	1436790_a_at	Strain	Sox11	20666	12	23892134	23893641	-
20565	1419741_at	Strain	Supt16h	114741	14	47260522	47297306	-
20566	1426906_at	Strain	LOC545386	545386	1	173789631	173812568	-
20567	1439200_x_at	Strain	Erdr1	170942	NONE	NONE	NONE	NONE
20568	1429116_at	Strain	Slc17a5	235504	9	78736237	78788257	-
20569	1417208_at	Strain	Amacr	17117	15	10790819	10805687	+
20570	1454402_at	Strain	3110048L19Rik	73233	17	19799824	19802130	-
20571	1420942_s_at	Strain	None	None	1	169589681	169628824	+
20572	1452791_at	Strain	Coq2	71883	6	137247204	137248859	-
20573	1435902_at	Strain	Nudt18	213484	14	64894081	64896270	+
20574	1433713_at	Strain	Gcn1l1 /// LOC545697	231659	5	114676923	114736096	+
20575	1453994_at	Strain	C230094A16Rik	237711	11	29638924	29659023	-
20576	1435935_at	Strain	None	None	5	117394280	117395037	+
20577	1420494_x_at	Strain	Ubc	22190	11	124553698	124555581	-
20578	1418429_at	Strain	Kif5b	16573	18	6245790	6284051	-
20579	1438824_at	Strain	Slc20a1	20515	2	128712653	128725462	+
20580	1436535_at	Strain	Ssa2	20822	1	143587635	143609041	-
20581	1423847_at	Strain	2810406C15Rik	68298	6	125824301	125848264	-
20582	1445902_at	Strain	None	None	11	105709036	105709927	+
20583	1418431_at	Strain	Kif5b	16573	18	6245790	6284051	-
20584	1441331_at	Strain	A230061C15Rik	320855	10	13880001	13881139	+
20585	1439517_at	Strain	C130067A03Rik	320713	4	93917113	93954172	-
20586	1433998_at	Strain	4933427D14Rik	74477	11	71879755	71929029	-
20587	1449813_at	Strain	Zfp30	22693	7	25199507	25209257	+
20588	1452110_at	Strain	Mtrr	210009	13	64582734	64602904	-
20589	1437680_x_at	Strain	Glrx2	69367	1	143571711	143578676	+
20590	1451731_at	Strain	Abca3	27410	17	22155713	22213193	+
20591	1455496_at	Strain	Pfas	237823	11	68711358	68729532	-
20592	1431473_at	Strain	5330423I11Rik	78268	9	34665971	34667890	+
20593	1446529_at	Strain	AI449175	234362	8	68761621	68776282	-
20594	1417105_at	Strain	1810017G16Rik	59005	8	121993095	121997038	+
20595	1424860_at	Strain	D930016D06Rik	100662	5	103588142	103590334	+
20596	1429247_at	Strain	Anxa6	11749	11	54731952	54786255	-
20597	1449072_a_at	Strain	2510005D08Rik	68043	14	52081084	52106667	-
20598	1448819_at	Strain	Eif2s2	67204	2	154328353	154349719	-
20599	1428013_at	Strain	6030458C11Rik	77877	15	12615740	12631966	-
20600	1417266_at	Strain	Ccl6	20305	11	83315881	83320979	-
20601	1453321_at	Strain	Fndc1	68655	17	333	29629	+
20602	1453123_at	Strain	Sf3b2	319322	19	5062722	5084238	-
20603	1456238_at	Strain	Zfp133	171588	2	143924439	143924731	+
20604	1416443_a_at	Strain	Uble1a	56459	7	13308414	13337163	-
20605	1439279_at	Strain	3110007F17Rik /// LOC382265 /// LOC546367	382265	X	118148022	118182394	+
20606	1432601_at	Strain	Mll5	69188	5	21957157	21958405	+
20607	1420613_at	Strain	Ptp4a2	19244	4	128866586	128877088	+
20608	1427127_x_at	Strain	Hspa1b	15511	17	32660211	32661550	-
20609	1429847_a_at	Strain	4833418A01Rik	75763	2	70753595	70786387	+
20610	1445928_at	Strain	38417	223455	15	31457571	31531643	-
20611	1431423_a_at	Strain	Med8	80509	4	117368260	117374016	+
20612	1426611_at	Strain	Psmc2	19181	5	20237647	20256148	+

20613	1428858_at	Strain	Wdr70	73770	8	108586498	108639211	-
20614	1435588_at	Strain	Wdfy1	69368	1	80056762	80093054	-
20615	1430000_at	Strain	B230117O15Rik	78871	4	3482316	3483437	-
20616	1423314_s_at	Strain	Pde7a	18583	3	18563269	18602308	-
20617	1460705_at	Strain	Rps6kb1	72508	11	86241975	86272766	-
20618	1444401_at	Strain	C80913	19777	7	33124913	33183548	-
20619	1433860_at	Strain	6030458C11Rik	77877	15	12615740	12631966	-
20620	1452994_at	Strain	Pip5k3	18711	1	65480863	65568017	+
20621	1432443_at	Strain	1700021P22Rik	75538	7	38571874	38576768	+
20622	1438718_at	Strain	Fgf9	14180	14	52607790	52644523	+
20623	1417825_at	Strain	Esd	13885	14	69077924	69096025	+
20624	1452231_x_at	Strain	LOC545386	545386	1	173789631	173812568	-
20625	1446904_at	Strain	Arhgef11	213498	3	87428572	87483137	+
20626	1437858_at	Strain	9330164H19Rik	233115	7	30851673	30917234	-
20627	1437404_at	Strain	Mast4	328329	13	98930827	99546711	-
20628	1416174_at	Strain	Rbbp9	26450	2	144000757	144007714	-
20629	1438353_at	Strain	None	None	9	70686640	70687047	+
20630	1457257_x_at	Strain	None	None	16	45273705	45273960	-
20631	1447341_at	Strain	D12Ertd551e	52635	12	112138486	112138920	+
20632	1433050_at	Strain	5730478J17Rik	70580	18	72390116	72390848	-
20633	1435865_at	Strain	Trim17 /// Hist3h2a	319162	11	58680314	58682303	+
20634	1422810_at	Strain	Zfp191	59057	18	24240763	24249267	-
20635	1442182_at	Strain	1810055D05Rik	67713	3	33524817	33528645	-
20636	1455959_s_at	Strain	Gclc	14629	9	77899181	77938127	+
20637	1430096_at	Strain	2900017F05Rik	72915	19	22014742	22016249	+
20638	1459710_at	Strain	1700001K19Rik	66323	12	106142923	106157841	-
20639	1418764_a_at	Strain	Bpnt1	23827	1	184830611	184856221	+
20640	1438466_at	Strain	Gm107	227058	1	46635424	46672653	+
20641	1454040_at	Strain	5730591J02Rik	77581	4	9697553	9707582	+
20642	1443564_at	Strain	None	None	13	63826369	63826737	-
20643	1435559_at	Strain	Myo6	17920	9	80370873	80516811	+
20644	1448437_a_at	Strain	Gtpbp2	56055	17	43670992	43679289	+
20645	1452709_at	Strain	Poldip3	73826	15	83176653	83200011	-
20646	1419660_at	Strain	1600012F09Rik	67008	13	17452501	17457637	-
20647	1428539_at	Strain	2610207I05Rik	233789	15	9031897	9034573	+
20648	1421000_at	Strain	Cnot4	53621	6	35139222	35227761	-
20649	1428297_at	Strain	Map4k2	26412	19	6130040	6142317	+
20650	1439917_at	Strain	Pdzk8	107368	19	58892773	58893596	-
20651	1419668_at	Strain	Sgcb	24051	5	72423553	72437683	-
20652	1454902_at	Strain	Prkcz	18762	4	153754439	153853605	-
20653	1427297_at	Strain	Mrpl9	78523	3	93931159	93936531	+
20654	1446324_at	Strain	None	None	5	13291160	13291820	-
20655	1435984_at	Strain	1110033F14Rik	68699	17	21083663	21084304	-
20656	1459105_at	Strain	None	None	11	69772201	69773156	+
20657	1438703_at	Strain	Ankrd26	232339	6	118934772	118994843	-
20658	1429258_at	Strain	1110025D03Rik	68620	7	39718715	39721902	-
20659	1460102_at	Strain	Clasp1	76707	1	118258317	118258939	+
20660	1443057_at	Strain	---	432669	12	66451383	66452077	-
20661	1456707_at	Strain	None	None	7	4295947	4306022	-
20662	1445824_at	Strain	Zfp458	238690	13	63854473	63868626	-
20663	1419363_a_at	Strain	Mrpl35	66223	6	72147085	72155754	-
20664	1420549_at	Strain	Gbp1	14468	3	141567114	141588140	+
20665	1438511_a_at	Strain	1190002H23Rik	66214	14	73642798	73655682	-
20666	1459617_at	Strain	Mapk14	26416	17	26492551	26549631	+
20667	1440637_at	Strain	Itsn1	16443	16	90939437	91069253	+
20668	1447864_s_at	Strain	Pogk	71592	1	166319364	166331981	-
20669	1452406_x_at	Strain	MGI:2384747	170942	NONE	NONE	NONE	NONE
20670	1437666_x_at	Strain	Ubc	22190	11	124553698	124555581	-
20671	1426704_at	Strain	Gak	231580	5	107645627	107705954	-
20672	1435270_x_at	Strain	2510005D08Rik	68043	14	52081084	52106667	-
20673	1451343_at	Strain	2210415M20Rik	70160	8	20945031	20970768	+
20674	1431993_a_at	Strain	Rnf38	73469	4	44044581	44074976	-
20675	1459917_at	Strain	AI451896	103736	11	84596860	84597291	-
20676	1457503_at	Strain	2310035C23Rik	227446	1	105555177	105695178	+

20677	1420052_x_at	Strain	Psmb1	19170	17	13479611	13501932	-
20678	1446558_at	Strain	F730015K02Rik	319526	9	64933661	64966353	+
20679	1427076_at	Strain	Mpeg1	17476	19	11657132	11661361	+
20680	1438285_at	Strain	2210015D19Rik	76508	11	5656916	5679497	+
20681	1444575_at	Strain	1500002O20Rik	71997	7	19575337	19598486	+
20682	1424415_s_at	Strain	Spon1	233744	7	107618725	107894210	+
20683	1451050_at	Strain	Nt5c3	107569	6	57026464	57068191	-
20684	1424554_at	Strain	Ppp1r8	100336	4	131787828	131804018	-
20685	1452805_at	Strain	D11Wsu47e	276852	11	113505503	113515740	+
20686	1422892_s_at	Strain	H2-Ea	14968	17	32042836	32045269	-
20687	1454905_at	Strain	Ibtk	108837	9	86018193	86080240	-
20688	1435682_at	Strain	Lars2	102436	9	123390644	123486368	+
20689	1420935_a_at	Strain	Srrm1	51796	4	134203290	134234393	-
20690	1455164_at	Strain	Cdgap	12549	16	37420388	37534097	-
20691	1444878_at	Strain	Dock10	210293	1	80833085	80997044	-
20692	1460084_at	Strain	None	None	NONE	NONE	NONE	NONE
20693	1422882_at	Strain	Sypl	19027	12	29560169	29583105	+
20694	1434659_at	Strain	5830411G16Rik	78937	6	56859230	56906236	+
20695	1454836_at	Strain	Tmem18	211986	12	27161018	27171249	+
20696	1456093_at	Strain	Zfp536	243937	7	32643367	32934010	-
20697	1448885_at	Strain	Rap2b	74012	3	61043051	61046895	+
20698	1419716_a_at	Strain	Pou2f1	18986	1	165797249	165856725	-
20699	1456408_x_at	Strain	4933439C10Rik	74476	11	59235162	59235785	+
20700	1441669_at	Strain	Centb2	78618	NONE	NONE	NONE	NONE
20701	1441108_at	Strain	None	None	NONE	NONE	NONE	NONE
20702	1446595_at	Strain	Sh3d1B	20403	12	3775688	3876446	+
20703	1451791_at	Strain	Tfpi	21788	2	84130609	84171923	-
20704	1442071_at	Strain	Abce1	24015	8	78898390	78925732	-
20705	1415795_at	Strain	Spin	20729	13	67875489	67920302	-
20706	1435070_at	Strain	Aebp2	11569	6	141421299	141478643	+
20707	1446536_at	Strain	Sema6d	214968	2	124124149	124181623	+
20708	1449552_at	Strain	Zfr	22763	15	11948204	11996199	+
20709	1428425_at	Strain	Tgfbrap1	73122	1	43341936	43348292	-
20710	1418629_a_at	Strain	None	None	4	128740932	128769419	-
20711	1437162_at	Strain	Gpiap1	53872	2	103469773	103502351	-
20712	1443913_at	Strain	None	None	NONE	NONE	NONE	NONE
20713	1444092_at	Strain	9430025M13Rik	233147	7	556	2501	+
20714	1459720_x_at	Strain	Tipin	66131	9	64403798	64426983	+
20715	1458985_at	Strain	9330186A19Rik	320365	5	149134722	149437633	+
20716	1450073_at	Strain	Kif3b	16569	2	152748365	152789150	+
20717	1416839_at	Strain	Mut	17850	17	38451138	38475743	+
20718	1421116_a_at	Strain	Rtn4	68585	11	29587737	29637706	+
20719	1421025_at	Strain	Agpat1	55979	17	32312217	32316761	+
20720	1456775_at	Strain	Ccne2	12448	4	11119669	11130063	+
20721	1430190_at	Strain	1700041C02Rik	73332	4	118281798	118374408	-
20722	1454994_at	Strain	Klhl20	226541	1	160992509	161035559	-
20723	1456341_a_at	Strain	Klf9	16601	19	22379147	22404832	+
20724	1436272_at	Strain	1110059F07Rik	98732	1	184746296	184782637	+
20725	1436669_at	Strain	1700019G17Rik	75541	6	86328677	86334511	-
20726	1438746_at	Strain	A530058N18Rik	320846	2	113545163	113546130	+
20727	1432947_at	Strain	4921519G19Rik	70872	10	42961228	42963044	+
20728	1434933_at	Strain	5730557L09Rik	108770	1	160875856	160878819	+
20729	1444113_at	Strain	None	None	4	147995249	147995848	+
20730	1442785_at	Strain	Smyd3	69726	1	178910022	179472249	-
20731	1452439_s_at	Strain	Sfrs2	20382	11	116671000	116674175	-
20732	1431314_a_at	Strain	5830417I10Rik /// LOC545537	545537	3	88519001	88569840	+
20733	1417891_at	Strain	Tce1	79043	17	22693397	22698063	+
20734	1457886_at	Strain	LOC552873	552873	18	72339648	72340404	-
20735	1436023_at	Strain	Bclaf1	72567	10	20239740	20267469	+
20736	1439987_at	Strain	Grik1	14805	16	87069420	87213311	-
20737	1448805_at	Strain	Usf1	22278	1	171340520	171347582	+
20738	1451629_at	Strain	MGI:1925139	77889	17	70694580	70718332	+
20739	1430599_at	Strain	Myt1l	17933	12	26094752	26491102	+
20740	1426352_s_at	Strain	Tial1	21843	7	122491640	122510627	-

20741	1440582_at	Strain	8030492O04Rik	319234	2	154659120	154659815	+
20742	1444622_at	Strain	None	None	4	75459434	75460720	-
20743	1422167_at	Strain	Sema5a	20356	15	32419980	32698954	+
20744	1433671_at	Strain	A130022J15Rik	101351	6	97577445	97615221	-
20745	1418189_s_at	Strain	Ramp2	54409	11	101067518	101069332	+
20746	1457233_at	Strain	Dnaja2	56445	8	84810414	84828045	-
20747	1417120_at	Strain	D4Wsu114e	28010	4	146353153	146360824	-
20748	1419257_at	Strain	Tcea1	21399	1	4818207	4858297	+
20749	1450980_at	Strain	Gtpbp3	70359	8	70637908	70643206	+
20750	1441212_at	Strain	6720467C03Rik	68099	4	12080689	12098962	-
20751	1453754_at	Strain	4930429A08Rik	74648	4	128177291	128216596	-
20752	1456143_at	Strain	Prkcbp1	228880	2	165242034	165353683	-
20753	1424861_at	Strain	D930016D06Rik	100662	5	103588142	103590334	+
20754	1415759_a_at	Strain	Hbxip	68576	3	107074746	107083731	+
20755	1439405_x_at	Strain	1700051C09Rik	68107	11	101100290	101109788	+
20756	1439686_at	Strain	Mbnl1	56758	3	60179725	60308221	+
20757	1459332_at	Strain	None	None	2	154633066	154633549	+
20758	1444406_at	Strain	Map4k3	225028	17	78411242	78558951	-
20759	1448760_at	Strain	Zfp68	24135	5	137577594	137591201	-
20760	1460442_at	Strain	Rps19	20085	7	20062265	20067353	+
20761	1429836_at	Strain	1810064L21Rik	66435	14	113542917	113545445	-
20762	1417571_at	Strain	Mpg	268395	11	32121305	32127494	+
20763	1424721_at	Strain	Mfap3	216760	11	57244336	57256528	+
20764	1415859_at	Strain	Eif3s8	56347	7	120596642	120616097	-
20765	1459751_s_at	Strain	Ppp1r16a	73062	15	76741364	76745811	+
20766	1440771_at	Strain	Zkscan1	74570	5	137058118	137075887	+
20767	1459048_s_at	Strain	Zfp142	77264	1	74868909	74890473	-
20768	1430811_a_at	Strain	Cdca1	66977	1	169432488	169465605	-
20769	1457904_at	Strain	None	None	NONE	NONE	NONE	NONE
20770	1438187_at	Strain	Slc25a29	214663	12	104296871	104306869	-
20771	1456293_s_at	Strain	Ccnh	66671	13	81281211	81305457	+
20772	1439815_at	Strain	A230048G03Rik	320473	17	76575104	76657599	-
20773	1453668_at	Strain	3110052M02Rik	73229	17	19528262	19534084	+
20774	1415919_at	Strain	Npdc1	18146	2	25335245	25341656	+
20775	1421251_at	Strain	Zfp40	22700	17	21075485	21079148	-
20776	1445555_at	Strain	Trpm3	226025	19	21686135	22227805	+
20777	1428282_at	Strain	Tbce	70430	13	13440370	13482145	-
20778	1455650_at	Strain	Srpk2	20817	5	21967253	22080468	-
20779	1440662_at	Strain	Rgl1	19731	1	152402014	152509595	-
20780	1446934_at	Strain	None	None		18060337	18060883	+
20781	1441576_at	Strain	2410002O22Rik	66975	13	100361503	100396609	-
20782	1445618_at	Strain	None	None	NONE	NONE	NONE	NONE
20783	1441437_at	Strain	Rbms1	56878	2	60608122	60818320	-
20784	1453044_at	Strain	C030014I23Rik	77381	9	44467633	44470847	+
20785	1435814_at	Strain	Xpo7	65246	14	64979347	65026150	-
20786	1456255_at	Strain	AI314180	230249	4	58743479	58856174	-
20787	1456187_at	Strain	Slc7a14	241919	3	30617648	30721720	-
20788	1453634_a_at	Strain	Erp29	67397	5	120596560	120604281	-
20789	1448465_at	Strain	Nipsnap1	18082	11	4768790	4788987	+
20790	1445235_at	Strain	Ythdf3	229096	3	15522868	15556473	+
20791	1460567_at	Strain	9930116O05Rik	319758	9	72752141	72755606	+
20792	1432393_a_at	Strain	5730409G07Rik	66628	11	45700742	45708331	-
20793	1425557_x_at	Strain	Tsc22d3	14605	X	134084467	134088106	-
20794	1440555_at	Strain	Rragd	52187	4	33274625	33297732	+
20795	1416180_a_at	Strain	Rdx	19684	9	52120601	52161012	+
20796	1452625_at	Strain	Kctd2	70382	11	115241243	115252365	+
20797	1443087_at	Strain	Cdc23	52563	18	34854705	34874773	-
20798	1460159_at	Strain	C130067A03Rik	320713	4	93917113	93954172	-
20799	1446648_at	Strain	Stxbp4	20913	11	90301004	90459167	-
20800	1453125_at	Strain	Sox11	20666	12	23888762	23891665	-
20801	1417261_at	Strain	Mbtd1	103537	11	93733496	93766406	+
20802	1435661_at	Strain	Gm972	381259	1	59405529	59424743	-
20803	1453077_a_at	Strain	Snapc3	77634	4	82403941	82453789	+
20804	1441498_at	Strain	A230006I23Rik	319919	4	75378935	75379616	-
20805	1441580_at	Strain	Sgpp2	433323	1	78598998	78708934	+

20806	1425288_at	Strain	Samd11	231004	NONE	NONE	NONE	NONE
20807	1458010_at	Strain	None	None	4	76880139	76880987	-
20808	1443210_at	Strain	None	None	5	103491009	103491676	-
20809	1436386_x_at	Strain	2610036A22Rik	70190	4	144744997	144745372	+
20810	1452160_at	Strain	Tiparp	99929	3	65201879	65228950	+
20811	1438963_s_at	Strain	Tfpt /// LOC546028	546028	7	4443	5254	-
20812	1442992_at	Strain	130004C03	403343	9	46088068	46088679	+
20813	1423240_at	Strain	None	None	2	156914044	156928808	+
20814	1436740_at	Strain	LOC546041	546041	8	19729849	19734189	+
20815	1420982_at	Strain	Rnpc2	170791	2	155604575	155637077	-
20816	1453179_at	Strain	Phca	66190	7	92263182	92356266	-
20817	1437667_a_at	Strain	Bach2	12014	4	32517743	32859992	+
20818	1423810_at	Strain	2700017M01Rik	72590	7	94433409	94478568	-
20819	1434206_s_at	Strain	Ppp2r5c	26931	12	105961123	106055786	+
20820	1436860_at	Strain	Senp7	66315	16	54970744	55086065	+
20821	1426355_a_at	Strain	6330578E17Rik	76178	1	37711567	37713525	-
20822	1452674_a_at	Strain	Eif3s12	73830	7	24377989	24388482	-
20823	1441995_at	Strain	Ncam1	17967	9	49555319	49607941	-
20824	1448516_at	Strain	Tsn	22099	1	118048034	118060649	-
20825	1438299_at	Strain	9230108I15Rik	78078	2	176507430	176508342	+
20826	1448993_at	Strain	Atg3l	67841	16	44041871	44071580	+
20827	1427198_at	Strain	BC022960	237246	X	162967594	162969545	+
20828	1454283_at	Strain	9130023D20Rik	268706	13	109055082	109126917	+
20829	1455131_at	Strain	Opa3	403187	7	16097225	16115805	+
20830	1420999_at	Strain	Cnot4	53621	6	35139222	35227761	-
20831	1456265_at	Strain	---	433605	3	65339678	65400714	+
20832	1453507_at	Strain	C030040A15Rik	77539	2	49251015	49251960	+
20833	1435252_at	Strain	B3galt6	117592	4	154482891	154485011	-
20834	1439111_at	Strain	None	None	14	70761378	70783322	+
20835	1444034_at	Strain	Rapgef4	56508	2	71679030	71954119	+
20836	1437749_s_at	Strain	Mrpl9	78523	3	93931159	93936531	+
20837	1439663_at	Strain	Ptch1	19206	13	60894523	60948510	-
20838	1425548_a_at	Strain	Lst1	16988	17	32888815	32889030	-
20839	1418514_at	Strain	Mtf2	17765	5	107157468	107177436	+
20840	1456748_a_at	Strain	Nipsnap1	18082	11	4768790	4788987	+
20841	1442305_at	Strain	Gtpbp2	56055	17	43670992	43679289	+
20842	1445299_at	Strain	Ppm1h	319468	10	122369636	122614468	+
20843	1438407_at	Strain	9330132E09Rik	319901	1	111860088	111864183	-
20844	1435135_at	Strain	Aadacl1	320024	3	26600437	26662708	+
20845	1429463_at	Strain	Prkaa2	108079	4	103993895	104068774	-
20846	1441381_at	Strain	9430029E18Rik	320780	NONE	NONE	NONE	NONE
20847	1435860_at	Strain	Slc5a6 /// LOC434043	330064	5	29494974	29507187	-
20848	1443931_at	Strain	Zfp617	170938	8	71079283	71092128	+
20849	1454510_at	Strain	2900034C19Rik	72854	4	102670564	102671805	+
20850	1440465_at	Strain	Cutl1	13047	5	135261627	135499343	-
20851	1426803_at	Strain	1700009P03Rik	74213	14	99658921	99720050	-
20852	1419685_at	Strain	Rent1	19704	8	69485033	69506716	-
20853	1452735_at	Strain	Pcnp	328694	16	54911436	54920579	-
20854	1419041_at	Strain	D8Wsu49e	71927	8	84991315	85113385	-
20855	1420863_at	Strain	Dctn4	67665	18	60751547	60784088	+
20856	1423734_at	Strain	Rac1	19353	5	142563708	142586223	-
20857	1422805_a_at	Strain	Ing3	71777	6	21996682	22023073	+
20858	1417784_at	Strain	Als2	74018	1	59468051	59542338	-
20859	1424317_at	Strain	Slc25a19	67283	11	115436076	115449232	-
20860	1460585_x_at	Strain	Pisd	320951	5	31224767	31274080	-
20861	1431337_a_at	Strain	1810055E12Rik	67894	19	60412054	60436632	+
20862	1457756_at	Strain	Zfp192	93681	13	20999835	21011161	-
20863	1447628_x_at	Strain	None	None	2	127118377	127118517	+
20864	1435321_at	Strain	3732412D22Rik	77569	5	65624008	65822238	+
20865	1431053_at	Strain	Mphosph9	269702	5	123422304	123499213	-
20866	1445290_at	Strain	Hunk	26559	16	89543829	89656985	+
20867	1440345_at	Strain	None	None	11	83021588	83022623	+
20868	1446445_at	Strain	None	None	12	33216600	33217261	+
20869	1437301_a_at	Strain	Dvl1	13542	4	154339774	154351665	+

20870	1436388_a_at	Strain	3830406C13Rik	218734	14	10742091	10761089	+
20871	1428589_at	Strain	2810443J12Rik	67228	2	24894607	24905633	+
20872	1418225_at	Strain	Orc2l	18393	1	58767641	58809789	-
20873	1455905_at	Strain	2610507B11Rik	72503	11	77987415	78016282	+
20874	1438936_s_at	Strain	Ang1	11727	14	46191054	46196646	+
20875	1456767_at	Strain	Lrfn3	233067	7	25769598	25776923	-
20876	1429535_at	Strain	Armc8	74125	9	99381642	99469875	-
20877	1422953_at	Strain	Fpr-rs2	14289	17	15751691	15757819	+
20878	1455933_at	Strain	1500010G04Rik	68965	6	49394626	49398413	-
20879	1439847_s_at	Strain	Klf12	16597	14	94399045	94678209	-
20880	1437883_s_at	Strain	None	None	5	146421353	146424825	+
20881	1437904_at	Strain	MGI:2387367	241490	2	76067817	76081465	+
20882	1439647_at	Strain	LOC433698 /// LOC545611	433698	4	42763995	42769810	-
20883	1418962_at	Strain	1110005F07Rik	66147	4	139947778	139959595	-
20884	1458026_at	Strain	None	None	4	54929247	54930034	+
20885	1426559_at	Strain	Sbno1	243272	5	123544504	123597297	-
20886	1427406_at	Strain	Trip11	109181	12	97280506	97358629	-
20887	1452590_a_at	Strain	Plac9	211623	14	24264282	24278771	-
20888	1418794_at	Strain	Cds2	110911	2	131777151	131821546	+
20889	1453550_a_at	Strain	Mlstd2	67420	7	107366377	107420898	+
20890	1424159_at	Strain	1300010M03Rik	67998	11	100917409	100940930	-
20891	1457338_at	Strain	AI132431	329251	1	134609330	134806191	-
20892	1443323_at	Strain	A930028L21Rik	229780	3	115354021	115363721	-
20893	1444589_at	Strain	None	None	17	20092968	20093341	-
20894	1416035_at	Strain	None	None	12	70756312	70795958	+
20895	1433799_at	Strain	Rdh13	108841	7	3656644	3676589	-
20896	1439712_at	Strain	4921521J11Rik	70885	8	67943264	67976954	+
20897	1456398_at	Strain	AI316828	544752	11	3541653	3542296	+
20898	1425201_a_at	Strain	Hyi	68180	4	117318916	117321649	+
20899	1416744_at	Strain	Uap1	107652	1	170076180	170109123	-
20900	1443949_at	Strain	Ppp2r5e	26932	12	72307243	72452562	-
20901	1452787_a_at	Strain	Hrmt1l2	15469	7	39053300	39062874	-
20902	1448214_at	Strain	Pdhb	68263	14	5833813	5841767	-
20903	1438576_x_at	Strain	2900056M20Rik	72997	NONE	NONE	NONE	NONE
20904	1420547_at	Strain	Galc	14420	12	93629142	93686179	-
20905	1437629_at	Strain	Arhgef19	213649	4	140124145	140138823	+
20906	1441945_s_at	Strain	Abhd14a	68644	9	106433630	106441207	-
20907	1427329_a_at	Strain	Igh-6	16019	12	110057802	110059089	-
20908	1452827_at	Strain	1500009C09Rik	76505	15	82306812	82311469	+
20909	1419453_at	Strain	Uchl5	56207	1	143609398	143638878	+
20910	1440807_at	Strain	Acvrinp1	50791	5	18347411	19110332	+
20911	1429239_a_at	Strain	Stard4	170459	18	33424453	33436848	-
20912	1448491_at	Strain	Ech1	51798	7	24231204	24238206	+
20913	1416409_at	Strain	Acox1	11430	11	115992984	116019869	-
20914	1456933_at	Strain	None	None	1	152772718	152773104	-
20915	1424385_at	Strain	5830417I10Rik	76022	3	420	48501	+
20916	1439493_at	Strain	D630040G17Rik	244530	8	78406599	78407363	+
20917	1429371_at	Strain	2810426N06Rik	67607	7	35800477	35817826	+
20918	1438419_at	Strain	Rbm16	106583	17	3065810	3160145	+
20919	1423694_at	Strain	Kctd10	330171	5	113474143	113491040	-
20920	1433682_at	Strain	Arhgef17	207212	7	94978006	95038415	-
20921	1457822_at	Strain	None	None	1	37209777	37210188	-
20922	1441058_at	Strain	Itpkb	320404	1	180285325	180377369	+
20923	1423417_at	Strain	Smarcc1	20588	9	110159869	110278595	+
20924	1440375_at	Strain	None	None	6	143921132	143921901	-
20925	1418008_at	Strain	1810007M14Rik	67367	16	90171076	90201546	-
20926	1439463_x_at	Strain	Hmgb1 /// LOC433238 /// LOC434174 /// LOC545555 /// LOC545917	15289	5	147944154	147949960	-
20927	1453559_a_at	Strain	Sel1h	20338	12	87225863	87265702	-
20928	1453739_at	Strain	1110001A23Rik	68472	7	84518566	84525733	-
20929	1438123_at	Strain	None	None	8	68532721	68533275	-

20930	1451602_at	Strain	Snx6	72183	12	0	675	-
20931	1439554_at	Strain	Scmh1	29871	4	119469474	119552399	+
20932	1429490_at	Strain	Rif1	51869	2	52004994	52054222	+
20933	1453067_at	Strain	2610040C18Rik	69928	4	147620711	147629912	-
20934	1430992_s_at	Strain	1500009M05Rik	67006	3	134289421	134306505	-
20935	1418409_at	Strain	Jrk	16469	15	74734074	74740959	-
20936	1456066_a_at	Strain	Rpo1-4	20019	6	72241050	72311336	+
20937	1458408_at	Strain	Samd8	67630	14	20115199	20157956	+
20938	1456683_at	Strain	5730555F13Rik	66660	9	70720790	70732757	+
20939	1431068_at	Strain	1110007A06Rik	68477	6	71723228	71772465	-
20940	1440651_at	Strain	Dusp16	70686	6	135472936	135549943	-
20941	1443235_at	Strain	Eif2ak4	27103	2	117902466	117989082	+
20942	1424082_at	Strain	Tbc1d13	70296	2	30066028	30084175	+
20943	1424598_at	Strain	Ddx6	13209	9	44593753	44625961	+
20944	1435777_at	Strain	E030018N11Rik	319622	7	112360968	112362974	-
20945	1430997_at	Strain	Cd47	16423	16	48697237	48753271	+
20946	1451238_at	Strain	1200003C05Rik	104771	12	68925221	68939959	+
20947	1443884_at	Strain	Thada	240174	17	82025019	82299790	-
20948	1443991_at	Strain	Dock1	330662	7	129038306	129541299	+
20949	1428592_s_at	Strain	Usp38	74841	8	80192741	80226914	-
20950	1440614_at	Strain	A330041B18Rik	104707	11	97311166	97311837	+
20951	1450007_at	Strain	1500003O03Rik	56398	2	119061563	119100869	+
20952	1460392_a_at	Strain	Eny2	223527	15	44373735	44383309	+
20953	1437743_at	Strain	Aebp2	11569	6	141421299	141478643	+
20954	1453133_at	Strain	Slc25a31	73333	3	40128629	40145290	+
20955	1456706_at	Strain	4833441D16Rik	108962	5	3578638	3579464	+
20956	1442537_at	Strain	None	None	3	17854831	17855401	+
20957	1416525_at	Strain	Spop	20747	11	95235201	95314100	+
20958	1418506_a_at	Strain	Prdx2	21672	8	84237800	84242986	+
20959	1434753_at	Strain	Nfrkb	235134	9	31308665	31343681	+
20960	1438228_at	Strain	4930452B06Rik	74430	14	6100611	6335659	-
20961	1438069_a_at	Strain	Rbm5	83486	9	107808484	107838963	-
20962	1434020_at	Strain	Pdap1	231887	5	144168088	144178675	-
20963	1458091_at	Strain	Dpp10	269109	1	123096743	123812573	-
20964	1449960_at	Strain	Nptx2	53324	5	143587516	143599211	+
20965	1446700_at	Strain	AA536808	98214	NONE	NONE	NONE	NONE
20966	1435998_at	Strain	---	239083	14	45884453	45890368	-
20967	1427353_at	Strain	Clasp1	76707	1	118140712	118256976	+
20968	1439075_at	Strain	None	None	2	143996949	143998929	+
20969	1430436_at	Strain	3321401G04Rik	77574	6	42816018	42836530	-
20970	1439477_at	Strain	5430406J06Rik	73848	11	51746391	51747335	-
20971	1444855_at	Strain	None	None	9	44479760	44480621	+
20972	1435082_at	Strain	Sypl	19027	12	29560169	29583105	+
20973	1459902_at	Strain	2700007P21Rik	212772	2	106588216	106594178	-
20974	1446353_at	Strain	Tubb6	67951	18	67621399	67633418	+
20975	1444530_at	Strain	None	None	2	52067889	52068457	+
20976	1457817_at	Strain	Bcas3	192197	11	85081206	85554043	+
20977	1436678_at	Strain	---	433905	5	72422689	72423167	-
20978	1424654_at	Strain	Acp2	11432	2	90907679	90916892	+
20979	1459270_at	Strain	Abcc1	17250	16	13097054	13210369	+
20980	1448939_at	Strain	Usp25	30940	16	76100019	76202730	+
20981	1455380_at	Strain	C630049M13	240334	18	61925649	61936447	-
20982	1441683_at	Strain	1110033M05Rik	68675	13	73773385	74230386	+
20983	1427695_a_at	Strain	Pou2f1	18986	1	165797249	165856725	-
20984	1455849_at	Strain	Nav1	215690	1	135289346	135436181	-
20985	1430696_at	Strain	Eif2ak1	15467	5	142939806	142944198	+
20986	1447058_at	Strain	A530058N18Rik	320846	2	113529139	113529687	+
20987	1460020_at	Strain	None	None	8	122312780	122423305	-
20988	1444701_at	Strain	A130001D14Rik	399620	18	73811546	73812561	+
20989	1415712_at	Strain	Zranb1	360216	7	127315953	127350282	+
20990	1452151_at	Strain	BC021523	223752	15	86107573	86187401	+
20991	1451735_at	Strain	Arfrp1	76688	2	181074790	181082318	-
20992	1441423_at	Strain	Ece1	230857	4	136743558	136846490	+
20993	1423169_at	Strain	Taf7	24074	18	37864624	37867888	-
20994	1451839_a_at	Strain	Pde7a	18583	3	18563269	18602308	-

20995	1436380_at	Strain	Cdc42bpa	226751	1	180019272	180119848	+
20996	1417034_at	Strain	Trappc6a	67091	7	16377969	16385389	+
20997	1434562_at	Strain	Mfap3	216760	11	57244336	57256528	+
20998	1455712_at	Strain	Hist3h2a	319162	11	58680314	58682303	+
20999	1459194_at	Strain	2210408I21Rik	72371	13	73228402	73229101	+
21000	1434280_at	Strain	None	None	NONE	NONE	NONE	NONE
21001	1459101_at	Strain	C78760	96972	NONE	NONE	NONE	NONE
21002	1417066_at	Strain	Cabc1	67426	1	180120861	180136493	-
21003	1458247_s_at	Strain	Dctn5	59288	7	116010930	116026902	+
21004	1440513_at	Strain	C80258	97540	9	46093722	46094403	+
21005	1438682_at	Strain	Pik3r1	18708	13	97857040	97865195	-
21006	1447526_at	Strain	D5Ertd255e	52183	5	117735349	117735680	+
21007	1419564_at	Strain	Zfp467	68910	6	48570465	48578486	-
21008	1426046_a_at	Strain	Rabggta	56187	14	50233893	50240192	-
21009	1446407_at	Strain	C130067A03Rik	320713	4	93917113	93954172	-
21010	1428769_at	Strain	Tatdn3	68972	1	190769675	190799261	-
21011	1428693_at	Strain	2610044O15Rik	72139	8	624	17937	-
21012	1439655_at	Strain	Ube2d2	56550	18	35995272	36030828	+
21013	1459953_at	Strain	AW011752	104570	11	29067786	29113926	+
21014	1418801_at	Strain	Zkscan1	74570	5	137058118	137075887	+
21015	1437521_s_at	Strain	E230022H04Rik	225339	18	32000024	32022887	+
21016	1455757_at	Strain	LOC241944	241944	3	35611228	35628140	+
21017	1444585_at	Strain	Adc	242669	4	127959259	127989530	-
21018	1430585_at	Strain	5930436O19Rik	77068	17	85916777	85918308	-
21019	1447977_x_at	Strain	None	None	NONE	NONE	NONE	NONE
21020	1431062_a_at	Strain	Sec8l1	20336	6	33334773	34062752	+
21021	1435520_at	Strain	Msi2h	76626	11	88067452	88208048	-
21022	1458074_at	Strain	---	328280	13	64446036	64455687	+
21023	1436781_at	Strain	Man2b1	17159	8	84354016	84368320	+
21024	1433838_at	Strain	Dars2	226539	1	160945706	160974780	-
21025	1429040_at	Strain	2610005L07Rik	381598	15	1398	8167	+
21026	1418664_at	Strain	Mpdz	17475	4	80278786	80441865	-
21027	1460621_x_at	Strain	Ywhaq	22630	12	18728420	18754333	+
21028	1446434_at	Strain	Galnact2	78752	6	118540028	118571686	-
21029	1441434_at	Strain	2810426N06Rik	67607	7	35800477	35817826	+
21030	1458617_at	Strain	Prkcbp1	228880	2	165242034	165353683	-
21031	1424171_a_at	Strain	Hagh	14651	17	22655960	22670054	+
21032	1450856_at	Strain	Arvcf	11877	16	17119316	17178126	+
21033	1452452_at	Strain	None	None	NONE	NONE	NONE	NONE
21034	1443573_at	Strain	Parp1	11545	1	180523221	180555500	+
21035	1423787_at	Strain	Nup133	234865	8	123186372	123236337	-
21036	1456264_at	Strain	Commd7	99311	2	153075770	153089667	-
21037	1429372_at	Strain	Sox11	20666	12	23888762	23891665	-
21038	1420936_s_at	Strain	Cpsf2	51786	12	97421457	97450986	+
21039	1439020_at	Strain	AW146020	330361	6	82259659	82294463	+
21040	1438686_at	Strain	Eif4g1 /// LOC547244	208643	16	19444890	19465024	+
21041	1424136_a_at	Strain	Ppih /// LOC433064 /// LOC434090	433064	17	8062709	8069598	-
21042	1426743_at	Strain	MGI:2384914	216190	10	83487957	83536588	-
21043	1438053_at	Strain	Tfg	21787	16	55593941	55620963	-
21044	1425076_at	Strain	Dnajc18	76594	18	35897492	35926786	-
21045	1452727_at	Strain	1300003K24Rik	71750	10	127157504	127235986	+
21046	1441704_at	Strain	Plekha5	109135	6	141327040	141388972	+
21047	1441959_s_at	Strain	1200003C05Rik	104771	12	68925221	68939959	+
21048	1432471_at	Strain	5730419I09Rik	74741	6	143857967	143947128	-
21049	1449042_at	Strain	Ctcf	13018	8	104932169	104978643	+
21050	1451243_at	Strain	Rnpep	215615	1	135113205	135134507	-
21051	1443837_x_at	Strain	Bcl2	12043	1	106479000	106655083	-
21052	1453311_at	Strain	2310008B10Rik	70094	2	155336822	155338298	-
21053	1458318_at	Strain	3110047M12Rik	73184	15	41732796	41733127	+
21054	1453582_at	Strain	Chka	12660	19	3640704	3683157	+
21055	1443006_at	Strain	Timp3	21859	10	86261055	86310024	+
21056	1452433_at	Strain	None	None	NONE	NONE	NONE	NONE

21057	1427207_s_at	Strain	Afg3l2	69597	18	67635433	67694777	-
21058	1452667_at	Strain	Rab2b	76338	14	47361876	47379434	-
21059	1460590_s_at	Strain	Ywhaq	22630	12	18728420	18754333	+
21060	1431102_at	Strain	4933409L06Rik	74081	1	155740307	155868596	-
21061	1450527_at	Strain	Sstr1	20605	12	54943281	54945921	+
21062	1457137_at	Strain	None	None	4	77276562	77276830	+
21063	1418846_at	Strain	Ap4m1	11781	5	137144830	137151495	+
21064	1421500_at	Strain	Sts	20905	NONE	NONE	NONE	NONE
21065	1436766_at	Strain	None	None	6	38703619	38727602	+
21066	1424325_at	Strain	Esco1	77805	18	10611852	10655599	-
21067	1429158_at	Strain	Fbxo28	67948	1	182273786	182300573	-
21068	1438491_x_at	Strain	A530054K11Rik /// LOC432771	212281	13	64221810	64243402	-
21069	1442109_at	Strain	None	None	NONE	NONE	NONE	NONE
21070	1455153_at	Strain	Zfp236	329002	18	82763227	82861085	-
21071	1444120_at	Strain	Bin1	30948	18	32617401	32675875	+
21072	1452563_a_at	Strain	Jarid1d	20592	Y	234392	280253	+
21073	1425485_at	Strain	Mtmr6	219135	14	54801634	54838504	+
21074	1453083_at	Strain	6430701C03Rik	76229	7	6418978	6446169	-
21075	1429232_at	Strain	2610528B01Rik	70042	4	135225508	135227205	+
21076	1453795_at	Strain	Fahd2a	68126	2	126950068	126958394	-
21077	1444810_at	Strain	Acac	107476	11	84051605	84052700	+
21078	1449839_at	Strain	Casp3	12367	8	45564226	45585494	+
21079	1435868_at	Strain	AI505652	433667	3	156956709	157015617	+
21080	1431266_at	Strain	5830433G22Rik	76035	1	186744325	186745845	+
21081	1456492_at	Strain	9130404D08Rik	74549	8	69170702	69194854	-
21082	1433374_at	Strain	6030455L14Rik	77725	5	29980760	29981515	-
21083	1457908_at	Strain	6430585N13Rik	240476	18	84375240	84757026	-
21084	1421816_at	Strain	Gsr	14782	8	32458839	32503763	+
21085	1417913_at	Strain	2810037C03Rik	109145	8	21981204	21992238	-
21086	1429283_at	Strain	1500009M05Rik	67006	3	134289421	134306505	-
21087	1436494_x_at	Strain	D8Ertd812e	212528	8	83954671	83965285	+
21088	1415807_s_at	Strain	Sfrs2	20382	11	116671000	116674175	-
21089	1438652_x_at	Strain	Pigq	14755	17	3046	4385	-
21090	1428306_at	Strain	Ddit4	74747	10	59905319	59907414	-
21091	1428909_at	Strain	A130040M12Rik	319269	NONE	NONE	NONE	NONE
21092	1437840_s_at	Strain	AI325464	98314	1	3039	7113	+
21093	1444564_at	Strain	Apod	11815	16	30106394	30123724	-
21094	1419426_s_at	Strain	Ccl21b /// Ccl21a /// Ccl21c	18829	4	42689442	42690573	-
21095	1443697_at	Strain	None	None	7	55605058	55605306	-
21096	1431926_a_at	Strain	1300010M03Rik	67998	11	100917409	100940930	-
21097	1424573_at	Strain	Tmed5	73130	5	107193657	107204601	-
21098	1451181_at	Strain	2410008J05Rik	69195	12	108662283	108665876	+
21099	1433241_at	Strain	9430013L17Rik	77266	10	6079229	6080201	+
21100	1442188_at	Strain	4921504N20Rik	78251	13	63642005	63664985	-
21101	1436812_at	Strain	Fkrp	243853	7	13760291	13767676	-
21102	1435309_at	Strain	BC019943	234138	8	29932839	29943872	+
21103	1440776_at	Strain	3732412D22Rik	77569	5	65624008	65822238	+
21104	1459635_at	Strain	Dlgh1	13383	16	30473410	30682717	+
21105	1446362_at	Strain	None	None	NONE	NONE	NONE	NONE
21106	1442524_at	Strain	None	None	NONE	NONE	NONE	NONE
21107	1453654_at	Strain	Cnot10	78893	9	114550674	114606723	-
21108	1416523_at	Strain	Rnase1	19752	14	46239425	46241190	-
21109	1442102_at	Strain	Aco2	11429	15	81923222	81965850	+
21110	1450660_at	Strain	Pts	19286	9	50593943	50600967	-
21111	1430566_at	Strain	4733401A01Rik	70833	4	130684788	130686185	+
21112	1418282_x_at	Strain	Serpina1b /// Serpina1d /// Serpina1e	20701	12	99172098	99219702	-
21113	1459187_at	Strain	Siat4c	20443	9	34960623	35030854	-
21114	1428200_a_at	Strain	Ntan1	18203	16	12555143	12571313	+
21115	1452258_at	Strain	6820402O20Rik	228829	2	155653612	155764373	+
21116	1427077_a_at	Strain	Ap2b1	71770	11	83030763	83131001	+
21117	1415784_at	Strain	Vps35	65114	8	84533166	84572272	-

21118	1452030_a_at	Strain	Hnrpr	74326	4	135192229	135221935	+
21119	1434118_at	Strain	0610009K11Rik	68350	4	137315933	137323526	+
21120	1448674_at	Strain	Rnf25	57751	1	74896219	74903844	-
21121	1448933_at	Strain	Pcdhb17	93888	18	37708741	37712012	+
21122	1419920_s_at	Strain	Usp7	252870	16	8364073	8389916	-
21123	1447693_s_at	Strain	Neo1	18007	9	58995038	59154898	-
21124	1439138_at	Strain	2310035C23Rik	227446	1	105555177	105695178	+
21125	1453109_at	Strain	2810429K17Rik	77041	13	72117991	72155720	-
21126	1450083_at	Strain	Cnot4	53621	6	35139222	35227761	-
21127	1452064_at	Strain	Crsp3	70208	10	24869993	24889699	+
21128	1428949_at	Strain	Xpot	73192	10	121243639	121282407	-
21129	1449211_at	Strain	Bpnt1	23827	1	184830611	184856221	+
21130	1428161_a_at	Strain	Chchd2	14004	5	34624797	34625568	+
21131	1437118_at	Strain	Usp7	252870	16	8364073	8389916	-
21132	1443793_x_at	Strain	1110015K06Rik	68510	5	138747607	138771977	-
21133	1440699_at	Strain	None	None	1	66699737	66700337	+
21134	1456922_at	Strain	4933437K13Rik	74478	16	7646332	7992303	+
21135	1448999_at	Strain	Trappc5	66682	8	3024416	3029159	+
21136	1453402_at	Strain	6430500C12Rik	76883	8	38068592	38070360	+
21137	1431097_at	Strain	Garnl1	56784	12	52323288	52540884	-
21138	1438062_at	Strain	4832420A03Rik	320612	7	91736186	91737470	+
21139	1423152_at	Strain	Vapb	56491	2	173198044	173239833	+
21140	1422994_at	Strain	Pip5k3	18711	1	65480863	65568017	+
21141	1435262_at	Strain	Pign	27392	1	105412403	105554926	-
21142	1452774_at	Strain	Hnrpa3 /// LOC545592	229279	2	75357026	75367156	+
21143	1458917_at	Strain	None	None	5	31474465	31475030	+
21144	1455648_at	Strain	6.72E+21	210853	17	20033048	20055559	-
21145	1424692_at	Strain	2810055F11Rik	67217	12	68912526	68924411	-
21146	1453399_at	Strain	Ccnt2	72949	1	127616552	127647225	+
21147	1449934_at	Strain	Pura	19290	18	36504827	36511914	+
21148	1452126_at	Strain	Zfp160	224585	17	18880791	18900678	+
21149	1435034_at	Strain	AW060207	231571	5	106668184	106732285	+
21150	1421934_at	Strain	Cbx5	12419	15	103258024	103277308	-
21151	1428286_at	Strain	2900097C17Rik	347740	2	155845002	155848087	-
21152	1435425_at	Strain	4933439C20Rik /// Pisd	236604	11	3020573	3026734	+
21153	1418786_at	Strain	Mapk8ip2	60597	15	89507005	89515320	+
21154	1456359_at	Strain	Ppwd1	238831	13	100423310	100447008	-
21155	1424610_at	Strain	Trub2	227682	2	29708273	29720664	-
21156	1424064_at	Strain	Rab1b	76308	19	4887997	4895786	-
21157	1416446_at	Strain	Tmem30a	69981	9	79974649	79999130	-
21158	1440339_at	Strain	4833416E15Rik	73762	10	24612408	24612830	-
21159	1450909_at	Strain	Eif4e	13684	3	137416519	137446834	+
21160	1425072_at	Strain	Skp2	27401	15	8914251	8942170	-
21161	1427142_s_at	Strain	Jarid1b	75605	1	134410458	134483140	+
21162	1459167_at	Strain	Cdc42bpb	217866	12	106766370	106850750	-
21163	1418635_at	Strain	Etv3	27049	3	87270684	87284681	+
21164	1424538_at	Strain	Ubl4	27643	X	69028056	69029182	-
21165	1442830_at	Strain	Nusap1	108907	2	119132566	119163626	+
21166	1437557_at	Strain	D3Ertd254e	52237	3	35628679	35630961	+
21167	1454636_at	Strain	Cbx5	12419	15	103258024	103277308	-
21168	1429362_a_at	Strain	Sf3b2	319322	19	5062722	5084238	-
21169	1459318_at	Strain	Sema6d	214968	2	124124149	124181623	+
21170	1447359_at	Strain	LOC381955	381955	7	19761532	19763742	-
21171	1422884_at	Strain	Snrpd3	67332	10	75619782	75637180	+
21172	1451285_at	Strain	Fus	233908	7	122017364	122031841	+
21173	1441597_at	Strain	None	None	18	36811993	36812639	+
21174	1440318_at	Strain	Wdr35	74682	12	8290207	8345162	+
21175	1442951_at	Strain	Atxn7l4	72174	12	29753981	29857347	+
21176	1429048_at	Strain	Bloc1s2	73689	19	52550788	52552535	+
21177	1460113_at	Strain	B930093H17Rik	319770	NONE	NONE	NONE	NONE
21178	1418537_at	Strain	0610042E07Rik	67441	7	4090781	4112002	-
21179	1436890_at	Strain	Uap1l1	227620	2	25292055	25297811	-
21180	1456869_at	Strain	2210018M03Rik	67109	7	5326129	5349319	-

21181	1449518_at	Strain	Qpctl	67369	7	16008978	16017957	-
21182	1429894_a_at	Strain	Mtap7	17761	10	20157697	20207739	+
21183	1445512_at	Strain	None	None	17	30051143	30052024	-
21184	1443998_at	Strain	None	None	2	131503322	131504105	-
21185	1453230_at	Strain	Zfp74	72723	7	25347508	25366610	-
21186	1431182_at	Strain	Hspa8 /// LOC432432 /// LOC432883 /// LOC434047	15481	9	40751958	40756793	+
21187	1451888_a_at	Strain	Odz4	23966	7	90212889	90948341	+
21188	1460648_at	Strain	Nr2f6	13864	8	70523955	70531214	-
21189	1428352_at	Strain	Arrdc2	70807	8	69990851	69995433	-
21190	1429336_at	Strain	2610301K12Rik	72477	2	128332164	128366477	+
21191	1417654_at	Strain	Sdc4	20971	2	163880980	163899921	-
21192	1439411_a_at	Strain	Xpo7	65246	14	64979347	65026150	-
21193	1437203_at	Strain	None	None	12	28060233	28061507	-
21194	1415888_at	Strain	Hdgf	15191	3	87650025	87659786	+
21195	1439774_at	Strain	Prrx1	18933	1	163163407	163227993	-
21196	1424869_at	Strain	BC003479	216820	11	60556330	60583788	+
21197	1458692_at	Strain	Supt3h	109115	17	42281336	42624366	+
21198	1426205_at	Strain	Ppp1cb	19046	5	30917485	30950978	+
21199	1458685_at	Strain	Garnl1	56784	12	52323288	52540884	-
21200	1441604_at	Strain	Esd	13885	14	69077924	69096025	+
21201	1422675_at	Strain	Smarce1	57376	11	99030134	99052104	-
21202	1448324_at	Strain	Rnps1	19826	17	22218678	22229480	+
21203	1441142_at	Strain	2700081L22Rik	72648	4	52346174	52355876	-
21204	1424500_at	Strain	4732497O03Rik	216987	11	79659615	79688046	-
21205	1447223_at	Strain	None	None	11	30794442	30794922	-
21206	1439846_at	Strain	Klf12	16597	14	94399045	94678209	-
21207	1444483_at	Strain	4931428D14Rik	216618	11	29116326	29142062	-
21208	1421133_at	Strain	Pvrl3	58998	16	45281022	45382981	-
21209	1448050_s_at	Strain	Map4k4	26921	1	40195342	40319092	+
21210	1434706_at	Strain	Vcpip1	70675	1	9829046	9852904	-
21211	1434208_at	Strain	2900057K09Rik	108937	7	94009760	94013127	-
21212	1445334_at	Strain	1700047I17Rik	73385	12	51918988	51936568	+
21213	1449014_at	Strain	Lactb	80907	9	67082765	67102856	-
21214	1419362_at	Strain	Mrpl35	66223	6	72147085	72155754	-
21215	1422669_at	Strain	Ebag9	55960	15	44565229	44586536	+
21216	1457582_at	Strain	Uty	22290	Y	41346	184254	+
21217	1423523_at	Strain	Aass	30956	6	23124207	23184664	-
21218	1434474_at	Strain	Abca5	217265	11	110093339	110158764	-
21219	1425515_at	Strain	Pik3r1	18708	13	97857040	97865195	-
21220	1441848_at	Strain	None	None	16	8181258	8321087	+
21221	1415765_at	Strain	1110031M08Rik	68693	19	8016414	8030688	+
21222	1428134_at	Strain	2310005O14Rik	67914	8	94122589	94139067	+
21223	1422799_at	Strain	Bat2	53761	17	32853100	32866823	-
21224	1457884_at	Strain	4732496C06Rik	319685	7	110414567	110415253	+
21225	1425544_at	Strain	Plekha5	109135	6	141327040	141388972	+
21226	1420849_at	Strain	Crnkl1	66877	2	145374418	145391636	-
21227	1455525_at	Strain	Endogl1	208194	9	119439221	119458491	+
21228	1428921_at	Strain	2810021B07Rik	66308	13	17159108	17162800	+
21229	1459068_at	Strain	1110006I15Rik	68539	19	10067157	10078240	-
21230	1425562_s_at	Strain	Trnt1	70047	6	107265186	107278483	+
21231	1435721_at	Strain	Kcnq4	60613	4	119720071	119772048	-
21232	1443387_at	Strain	BB045044	99440	NONE	NONE	NONE	NONE
21233	1455182_at	Strain	Kif1b	16561	4	147668683	147799980	-
21234	1457484_at	Strain	D930050J11	414326	11	115516215	115516948	-
21235	1452584_at	Strain	1500032L24Rik	69029	15	82396763	82399779	+
21236	1429430_at	Strain	A030012M09Rik	319263	1	7090301	7142844	+
21237	1455107_at	Strain	Srpk1	20815	17	26391082	26423944	-
21238	1452906_at	Strain	Gtl2	17263	12	105023823	105039917	+
21239	1445431_at	Strain	Stk39	53416	2	68065847	68327381	-
21240	1436176_at	Strain	Syt11	229521	3	88489566	88516404	-
21241	1460384_a_at	Strain	Arid4b	94246	13	13506342	13643882	+
21242	1457991_at	Strain	Abcd3	19299	3	120537803	120594108	-

21243	1422874_at	Strain	Acrbp	54137	6	125705979	125719247	+
21244	1428234_at	Strain	Cpsf6	432508	10	117034119	117066391	-
21245	1416156_at	Strain	Vcl	22330	14	19290931	19398336	+
21246	1445564_at	Strain	None	None	5	90246138	90246389	-
21247	1428128_at	Strain	4921506J03Rik	382423	10	112606837	112607555	-
21248	1448626_at	Strain	Cdk5rap1	66971	2	153792323	153829656	-
21249	1449546_a_at	Strain	Zfp617	170938	8	71079283	71092128	+
21250	1439371_x_at	Strain	Timm44	21856	8	3614696	3630853	-
21251	1431948_a_at	Strain	Pank2	74450	2	130776353	130810544	+
21252	1429832_at	Strain	Ppih	66101	4	118258916	118279389	-
21253	1424738_at	Strain	4932432K03Rik	74385	14	44155313	44176487	+
21254	1444188_at	Strain	None	None	4	128877553	128878090	+
21255	1459001_at	Strain	Vps33a	77573	5	122700612	122742738	-
21256	1430763_at	Strain	4930563E22Rik	75304	11	71940830	71944102	+
21257	1452611_at	Strain	Zfp294	78913	16	86525259	86582064	-
21258	1451408_at	Strain	Trub2	227682	2	29708273	29720664	-
21259	1422040_at	Strain	Sema7a	20361	9	58054059	58076850	+
21260	1437649_x_at	Strain	Ppib	19035	9	66145681	66189949	+
21261	1433205_at	Strain	2810436B12Rik	72762	14	99801699	99802907	+
21262	1432304_a_at	Strain	9030624J02Rik	71517	7	112616598	112717342	+
21263	1457426_at	Strain	1700048O20Rik	69430	NONE	NONE	NONE	NONE
21264	1423320_at	Strain	Dnase1l2	66705	17	22244150	22246669	-
21265	1428198_at	Strain	4930578F03Rik	75894	2	120654277	120670529	+
21266	1460486_at	Strain	Rabgap1	227800	2	37375531	37441301	+
21267	1426370_at	Strain	Mlstd2	67420	7	107366377	107420898	+
21268	1452614_at	Strain	Gm566	229672	3	103259992	103266043	+
21269	1456047_at	Strain	Pla2g4b	211429	2	119547266	119555814	+
21270	1431676_x_at	Strain	Gtf2i	14886	5	133529235	133606084	-
21271	1457498_at	Strain	Man2a2	140481	7	74160715	74171123	-
21272	1419950_s_at	Strain	Tnpo3	320938	6	29587924	29656704	-
21273	1427583_at	Strain	4921505C17Rik	78757	15	6496946	6588954	+
21274	1446443_at	Strain	B230213L16Rik	320943	1	38310325	38310986	+
21275	1429296_at	Strain	Rab10	19325	12	3108	12388	-
21276	1443314_at	Strain	2410042D21Rik	72425	2	112002449	112037475	+
21277	1447263_at	Strain	Metap2	56307	10	93834268	93863130	-
21278	1416708_a_at	Strain	D7Bwg0611e	52857	7	26540708	26555116	-
21279	1428860_at	Strain	4930572J05Rik	223626	15	74752896	74756035	+
21280	1434612_s_at	Strain	Sbno1	243272	5	123544504	123597297	-
21281	1458798_at	Strain	None	None	17	44384370	44384855	-
21282	1436022_at	Strain	Endogl1	208194	9	119439221	119458491	+
21283	1448597_at	Strain	Cstf1	67337	2	171879736	171889819	+
21284	1458704_at	Strain	None	None	NONE	NONE	NONE	NONE
21285	1442675_at	Strain	Fancc	14088	13	60694745	60814715	-
21286	1434884_at	Strain	Mtdh	67154	15	34082454	34141052	+
21287	1444749_at	Strain	Apod	11815	16	30106394	30123724	-
21288	1454924_at	Strain	Fut10	171167	8	29970150	30020391	+
21289	1430984_at	Strain	Azin1	54375	15	38492445	38524417	-
21290	1428052_a_at	Strain	Zmym1	68310	4	126074207	126088245	-
21291	1460099_at	Strain	Atp9b	50771	18	80858429	81057410	-
21292	1458370_at	Strain	Bmp2k	140780	5	96015785	96109144	+
21293	1424277_at	Strain	1110020L19Rik	73738	X	68097970	68119684	-
21294	1449250_at	Strain	Prcc	94315	3	87604018	87630364	-
21295	1426575_at	Strain	Tmem23	208449	19	31448202	31573509	-
21296	1457005_at	Strain	None	None	NONE	NONE	NONE	NONE
21297	1430520_at	Strain	Cpne8	66871	15	90540437	90732329	-
21298	1418894_s_at	Strain	Pbx2	18515	17	32293780	32299792	+
21299	1455006_at	Strain	2310016M24Rik	66379	15	99782863	99785330	+
21300	1448622_at	Strain	Lsm4	50783	8	69829740	69835099	+
21301	1421533_at	Strain	Slc7a1	11987	5	147223681	147291421	-
21302	1448782_at	Strain	Txndc11	106200	16	10508059	10567680	+
21303	1455474_at	Strain	D6Wsu116e	28006	6	116645592	116700200	+
21304	1459950_at	Strain	6330415M09Rik	70762	3	86530542	86665241	-
21305	1443379_at	Strain	A430033K04	243308	5	137594368	137620231	+
21306	1428697_at	Strain	Dpp8	74388	9	65154659	65204852	+
21307	1446982_at	Strain	None	None	8	126821262	126821828	+

21308	1430918_at	Strain	38414	320253	18	56982085	57143467	-
21309	1450903_at	Strain	Rad23b	19359	4	55265936	55307091	+
21310	1453207_at	Strain	LOC554362	554362	8	23373351	23375971	+
21311	1445717_at	Strain	E130108L08Rik	78000	6	38734229	38735401	+
21312	1424863_a_at	Strain	Hipk2	15258	6	38834084	39012133	-
21313	1440925_at	Strain	Rhoq	104215	17	84819915	84856873	+
21314	1429684_at	Strain	5830472M02Rik	76080	2	163059320	163074164	+
21315	1448163_at	Strain	Gnpda1 /// LOC231914	231914	5	148052836	148073109	-
21316	1433748_at	Strain	Zdhhc18	503610	4	132567633	132594023	-
21317	1450102_a_at	Strain	Amfr	23802	8	93256098	93297014	-
21318	1419133_at	Strain	Evpl	14027	11	116041848	116059090	-
21319	1451513_x_at	Strain	Serpina1b	20701	12	99172098	99219702	-
21320	1446490_at	Strain	Ptbp2	56195	3	118493626	118557890	-
21321	1429680_at	Strain	1500010G04Rik	68965	6	49394626	49398413	-
21322	1451399_at	Strain	MGI:1930773	56695	1	74587397	74662104	+
21323	1424541_at	Strain	1110020A09Rik	70397	1	16850492	16863658	+
21324	1456386_at	Strain	Rnpc2	170791	2	155604575	155637077	-
21325	1455763_at	Strain	Rnf41	67588	10	128148476	128178259	+
21326	1421346_a_at	Strain	Slc6a6	21366	6	92133220	92208188	+
21327	1434207_at	Strain	2900057K09Rik	108937	7	94009760	94013127	-
21328	1451477_at	Strain	LOC545705 /// LOC545706	545705	4	144696792	144745191	+
21329	1437544_at	Strain	Fubp1	51886	3	151191900	151214173	+
21330	1437767_s_at	Strain	Fts	14339	8	90409865	90421862	-
21331	1426579_at	Strain	Gnl2	230737	4	124057151	124082485	+
21332	1419972_at	Strain	None	None	16	44024318	44024890	+
21333	1456896_at	Strain	6720462K09Rik	399579	4	147747513	147748160	-
21334	1426011_a_at	Strain	Ggnbp2	217039	11	84560721	84598730	-
21335	1422849_a_at	Strain	Pabpn1	54196	14	49410993	49414893	+
21336	1428426_s_at	Strain	Tgfbrap1	73122	1	43341936	43348292	-
21337	1423971_at	Strain	Thoc3	73666	13	53069551	53078853	-
21338	1438326_at	Strain	3300001M20Rik	66926	2	132318068	132329908	-
21339	1427037_at	Strain	Eif4g1	208643	16	19444890	19465024	+
21340	1419138_at	Strain	B3galt4	54218	17	31653655	31655222	-
21341	1442681_at	Strain	AU067695	381802	6	115981750	116015764	+
21342	1418012_at	Strain	Sh3glb1	54673	3	143660519	143691998	-
21343	1439216_at	Strain	1500004F05Rik	69765	8	39304569	39305148	+
21344	1430718_s_at	Strain	Zcsl3	99349	2	105671471	105708303	-
21345	1429528_at	Strain	Rae1	66679	2	172460604	172476199	+
21346	1457447_at	Strain	Rb1	19645	14	67546936	67677175	-
21347	1416779_at	Strain	Sdpr	20324	1	51590070	51603904	+
21348	1421376_at	Strain	Traf6	22034	2	101383123	101405659	+
21349	1427279_at	Strain	Rsnl2	78785	17	69558453	69633262	+
21350	1425014_at	Strain	Nr2c2	22026	6	92539374	92616647	+
21351	1426371_at	Strain	Mlstd2	67420	7	107366377	107420898	+
21352	1454985_at	Strain	D030051N19Rik	228361	2	91434974	91623598	+
21353	1451727_at	Strain	D11Ertd730e	193116	11	43186568	43200818	+
21354	1446968_at	Strain	Picalm	233489	7	84121344	84258493	+
21355	1434230_at	Strain	Polb /// A430088C08Rik	18970	8	21382844	21407993	-
21356	1442543_at	Strain	Tex264	21767	9	106724534	106751448	-
21357	1426191_a_at	Strain	Bcl2l1	12048	2	152237604	152288618	-
21358	1417427_at	Strain	1500026D16Rik	68209	19	5390675	5391722	+
21359	1450434_s_at	Strain	Pcyt1a	13026	16	31261076	31281386	+
21360	1456420_at	Strain	Arid4a	238247	12	67850998	67933600	+
21361	1451809_s_at	Strain	Rwdd3	73170	3	119937535	119953364	-
21362	1443969_at	Strain	Irs2	384783	8	10358726	10476803	-
21363	1440835_at	Strain	Zfp27	22689	7	25308433	25321249	-
21364	1454913_at	Strain	None	None	4	123971225	123971775	+
21365	1428612_at	Strain	Atg7l	74244	6	115108368	115295327	+
21366	1457096_at	Strain	6430520M22Rik	319716	6	146791203	146792072	-
21367	1429688_at	Strain	Arntl2	272322	6	147726463	147759658	+
21368	1426839_at	Strain	Pold3	67967	7	94188784	94228172	-
21369	1416549_at	Strain	Slc35b4	58246	6	34249834	34271009	-

21370	1455222_a_at	Strain	Ubp1	22221	9	113984479	114029010	+
21371	1452837_at	Strain	Lpin2	64898	17	68977342	69020903	+
21372	1442834_at	Strain	None	None	6	101329801	101331429	+
21373	1444430_at	Strain	Armc8	74125	9	99381642	99469875	-
21374	1457709_a_at	Strain	A930005H10Rik	68161	3	114652097	114658964	-
21375	1437353_at	Strain	BC035291	208043	5	122317613	122323666	+
21376	1431259_at	Strain	None	None	2	120671393	120673288	-
21377	1459226_at	Strain	None	None	15	34144654	34145103	+
21378	1443378_s_at	Strain	Adam1a	280668	5	120670410	120673501	-
21379	1451330_a_at	Strain	Inpp5b	16330	4	123768991	123828624	+
21380	1424715_at	Strain	0610039N19Rik	67442	6	72932145	72940919	+
21381	1420845_at	Strain	Mrps2	118451	2	28400228	28403339	+
21382	1448207_at	Strain	Lasp1	16796	11	97620759	97659851	+
21383	1457639_at	Strain	Atp6v1h	108664	1	5048448	5126164	+
21384	1421284_at	Strain	Pign	27392	1	105412403	105554926	-
21385	1433574_at	Strain	Cdc37l1	67072	19	28243091	28270166	+
21386	1440014_at	Strain	Pacs1	107975	19	4922475	5061909	-
21387	1453195_at	Strain	Sdccag3	68112	2	26315523	26321468	-
21388	1425097_a_at	Strain	Zfp106	20402	2	120023462	120077639	-
21389	1433804_at	Strain	Jak1	16451	4	100111937	100224138	-
21390	1427283_at	Strain	Mll	214162	9	44793512	44870129	-
21391	1453108_at	Strain	2810429K17Rik	77041	13	72117991	72155720	-
21392	1421495_a_at	Strain	1700052N19Rik	73419	10	6064884	6072237	-
21393	1451386_at	Strain	Blvrb	233016	7	22823535	22841923	+
21394	1418365_at	Strain	Ctsh	13036	9	89942041	89963703	+
21395	1431067_at	Strain	6330404A07Rik	70754	4	106093067	106094126	-
21396	1415806_at	Strain	Plat	18791	8	21512347	21537423	+
21397	1435295_at	Strain	D9Ertd809e	320615	9	86810919	86864199	+
21398	1420703_at	Strain	Csf2ra	12982	11	15	635	+
21399	1459956_at	Strain	Dock10	210293	1	80833085	80997044	-
21400	1458298_at	Strain	Cadps	27062	14	10830082	11279704	-
21401	1418185_at	Strain	4733401H18Rik	66706	9	108614863	108616340	-
21402	1453380_a_at	Strain	2410012H02Rik	68876	10	126601326	126634251	-
21403	1419382_a_at	Strain	Dhrs4	28200	14	49995929	50005102	+
21404	1453265_at	Strain	4930579C15Rik	67753	4	93882365	93903882	-
21405	1459583_at	Strain	Grip1	74053	10	119395694	119653294	+
21406	1438425_at	Strain	Gtf3c1	233863	7	119690685	119757419	-
21407	1435802_at	Strain	Gm157	232879	7	10999345	11013464	-
21408	1435904_at	Strain	Eif2c3	214150	4	125372498	125456507	-
21409	1428046_a_at	Strain	Zfx	22764	X	88735290	88783558	-
21410	1440292_at	Strain	D13Ertd666e	52695	13	61643018	61643849	-
21411	1425044_at	Strain	Kcnj6	16522	16	94170119	94405052	-
21412	1450537_at	Strain	Mid2	23947	X	134209895	134312655	+
21413	1453988_a_at	Strain	Ide	15925	19	36613620	36677821	-
21414	1452719_at	Strain	Zdhhc24	70605	19	4667494	4673298	+
21415	1458002_at	Strain	Mapk10	26414	5	101946583	102247027	-
21416	1425461_at	Strain	Fbxw11	103583	11	32537669	32641610	+
21417	1452111_at	Strain	Mrps35	232536	6	147969392	147997627	+
21418	1436547_at	Strain	None	None	11	88860304	88861610	-
21419	1457934_at	Strain	Rbm12	75710	2	155551819	155568864	-
21420	1433736_at	Strain	Hcfc1	15161	X	68603450	68626970	-
21421	1421903_at	Strain	Ixl	67224	7	23791956	23798275	-
21422	1460198_a_at	Strain	Psmb3 /// LOC240289	240289	18	52080386	52081047	-
21423	1423163_at	Strain	Bat4	81845	17	32826733	32828587	+
21424	1416991_at	Strain	Mto1	68291	9	78648099	78674470	+
21425	1438110_at	Strain	Zbtb1	268564	12	73226354	73244538	+
21426	1452185_at	Strain	Ipo8	320727	6	149706566	149767322	-
21427	1458405_at	Strain	B430201F14 /// LOC546946	330503	7	25592926	25593902	+
21428	1443241_at	Strain	C330017I15Rik	78697	5	22205737	22247905	-
21429	1450100_a_at	Strain	Tcerg1	56070	18	42732154	42795475	+
21430	1418250_at	Strain	Arfl4	66182	11	101486648	101488916	+
21431	1457562_at	Strain	Rps6kb1	72508	11	86241975	86272766	-
21432	1447960_at	Strain	9630017O17	331021	9	105390892	105392738	+

21433	1458057_at	Strain	Cald1	109624	6	34803747	34868362	+
21434	1424545_at	Strain	BC003965	214489	17	22990107	22993259	+
21435	1455681_at	Strain	Zfp369	170936	13	62709247	62728187	+
21436	1439196_at	Strain	Hook3	320191	8	24756639	24843763	-
21437	1431050_at	Strain	Rps6ka5	73086	12	95995143	96170417	-
21438	1435915_at	Strain	Jmjd4	194952	11	59175717	59183697	+
21439	1441543_at	Strain	Eya3	14050	4	131599945	131685662	+
21440	1456827_at	Strain	AA987161	380856	13	64191293	64211253	-
21441	1443857_at	Strain	Hook3	320191	8	24756639	24843763	-
21442	1430167_a_at	Strain	Rwdd3	73170	3	119937535	119953364	-
21443	1450747_at	Strain	Keap1	50868	9	21116332	21125078	-
21444	1444012_at	Strain	Yipf3	28064	17	43758020	43761938	+
21445	1433550_at	Strain	Chfr	231600	5	109186644	109222753	+
21446	1456855_at	Strain	None	None	7	94045303	94045640	-
21447	1452793_at	Strain	Dzip1	66573	14	113431542	113479731	-
21448	1436331_at	Strain	Vps13d	230895	4	143890039	144036650	-
21449	1430578_at	Strain	Eif4g3	230861	4	136874731	137087744	+
21450	1417831_at	Strain	Smc1l1	24061	X	145550823	145596362	+
21451	1416272_at	Strain	Map2k1ip1	56692	3	136807484	136817691	+
21452	1436943_at	Strain	9330151E16Rik	192986	11	72502890	72504168	-
21453	1436519_a_at	Strain	1110057K04Rik	68832	12	7453506	7531135	+
21454	1427490_at	Strain	Abcb7	11306	X	98882022	99015261	-
21455	1444414_at	Strain	Apod	11815	16	30106394	30123724	-
21456	1417243_at	Strain	2310065K24Rik	102122	8	93858310	93885851	-
21457	1424540_at	Strain	Hipk1	15257	3	103171062	103218570	-
21458	1456839_at	Strain	A930008A22Rik	235283	9	40248196	40406053	-
21459	1454201_a_at	Strain	5730405I09Rik	67974	18	9356614	9490220	-
21460	1416819_at	Strain	Cdc37	12539	9	21022051	21033416	-
21461	1416475_at	Strain	Ube2d2	56550	18	35995272	36030828	+
21462	1424381_at	Strain	Sf4	70616	8	69194933	69224824	+
21463	1426583_at	Strain	Atf2	11909	2	73516345	73590377	-
21464	1419398_a_at	Strain	Dp1	13476	18	34567907	34596351	-
21465	1437676_at	Strain	Spag9	70834	11	93817190	93944505	+
21466	1445123_at	Strain	Cbx1	12412	11	96610246	96629727	+
21467	1459674_at	Strain	Prkcb1	18751	7	116167155	116506625	+
21468	1455215_at	Strain	C530028O21Rik	319352	6	125652724	125659098	+
21469	1423713_at	Strain	Abcb8	74610	5	22857593	22873844	+
21470	1429775_a_at	Strain	Tm7sf1	83924	13	12777506	12812482	-
21471	1443222_at	Strain	Akt3	23797	1	176955410	177064396	-
21472	1458567_at	Strain	D130017N08Rik	320064	5	142826713	142827569	+
21473	1436006_at	Strain	Eif2ak1	15467	5	142939806	142944198	+
21474	1453830_at	Strain	None	None	7	83971856	83972840	+
21475	1457483_at	Strain	None	None	10	68276214	68276914	-
21476	1439035_at	Strain	Zfp322a	218100	13	22835406	22849233	-
21477	1434349_at	Strain	Vars2l	68915	17	33372705	33383911	-
21478	1428011_a_at	Strain	Erbb2ip	59079	13	100037161	100140118	-
21479	1457293_at	Strain	Zbtb4	75580	11	69492223	69505059	+
21480	1439770_at	Strain	6430598A04Rik	243300	5	136681548	136690598	-
21481	1428906_at	Strain	D17Wsu155e	66467	17	5986238	5991852	+
21482	1444705_at	Strain	App	11820	16	84062547	84281536	-
21483	1431985_at	Strain	2210010B09Rik	244721	9	20454212	20467883	+
21484	1443236_at	Strain	B230118H07Rik	68170	2	101265464	101333669	-
21485	1420651_at	Strain	Ate1	11907	7	124514765	124642811	-
21486	1427743_at	Strain	Gm672	269037	18	75666390	75932739	-
21487	1430271_x_at	Strain	4930553M18Rik	75316	9	15143402	15154138	+
21488	1451684_a_at	Strain	Bicd1	12121	6	4102	11525	+
21489	1415796_at	Strain	Dazap2	23994	15	100673200	100678299	+
21490	1420989_at	Strain	4933411K20Rik	66756	8	45112900	45139459	+
21491	1442142_at	Strain	2700050L05Rik	214764	7	128005358	128040623	+
21492	1455911_x_at	Strain	Ndufb11	104130	X	18854266	18856494	-
21493	1426368_at	Strain	LOC547368	547368	2	14042	56936	+
21494	1457805_at	Strain	None	None	13	8789786	8790211	+
21495	1443762_s_at	Strain	Mtmr13	319934	7	104160882	104472741	-
21496	1457492_at	Strain	None	None	15	27826114	27826821	-
21497	1422769_at	Strain	Syncrip	56403	9	88814754	88842894	-

21498	1420227_at	Strain	Hoxa4	15401	6	52333394	52335114	-
21499	1434818_at	Strain	9330159K06	327812	NONE	NONE	NONE	NONE
21500	1438345_at	Strain	None	None	17	77679557	77680194	-
21501	1417955_at	Strain	2600016J21Rik	72454	9	108530195	108535186	+
21502	1416661_at	Strain	Eif3s10	13669	19	60361493	60391011	-
21503	1449910_at	Strain	2210418O10Rik	76958	2	176402416	176414564	-
21504	1444724_at	Strain	None	None	14	88189357	88190260	-
21505	1420829_a_at	Strain	Ywhaq	22630	12	18728420	18754333	+
21506	1426633_s_at	Strain	Kctd14	233529	7	91499475	91504764	+
21507	1443755_at	Strain	None	None	NONE	NONE	NONE	NONE
21508	1435540_at	Strain	Irgq1	210146	7	19706649	19714490	+
21509	1457122_at	Strain	None	None	2	48903598	48904235	+
21510	1424681_a_at	Strain	Psma5	26442	3	108052953	108075715	+
21511	1422718_at	Strain	Ap3s2	11778	7	73672386	73717617	-
21512	1427559_a_at	Strain	Atf2	11909	2	73516345	73590377	-
21513	1446312_at	Strain	None	None	5	116901974	116902649	+
21514	1426932_at	Strain	D19Bwg1357e	52874	19	26629949	26669888	-
21515	1459354_at	Strain	Siat10	54613	16	57459955	57512884	-
21516	1420972_at	Strain	Arid5b	71371	10	68150685	68334013	-
21517	1447449_at	Strain	None	None	5	41989237	41989637	+
21518	1458700_at	Strain	E430036I04Rik	100604	5	104581541	104671026	+
21519	1441721_at	Strain	None	None	5	127619155	127619848	+
21520	1460285_at	Strain	Itga9	104099	9	118602968	118893288	+
21521	1452171_at	Strain	Grwd1	101612	7	39909985	39915506	-
21522	1444030_at	Strain	Dcp1b	319618	6	119607529	119653880	+
21523	1448047_at	Strain	None	None	1	181042998	181044100	-
21524	1452887_at	Strain	Traf3ip1	74019	1	91320407	91356416	+
21525	1446155_at	Strain	2700089E24Rik	381820	6	133875346	133882217	+
21526	1429385_at	Strain	Wdr68	71833	11	105857961	105876453	+
21527	1454314_at	Strain	1700031K17Rik	73343	4	42497298	42498204	+
21528	1458522_at	Strain	1810011K17Rik	226591	1	165134760	165159417	-
21529	1437997_x_at	Strain	Mrpl48	52443	7	94655385	94714672	-
21530	1435459_at	Strain	Fmo2	55990	1	162784360	162808025	-
21531	1446081_at	Strain	Zzef1	195018	11	72599528	72652778	+
21532	1428365_a_at	Strain	Prss15	74142	17	54302752	54315339	-
21533	1426753_at	Strain	Phf17	269424	3	41010564	41046427	+
21534	1428656_at	Strain	Rnasen	14000	15	12641215	12742571	+
21535	1417549_at	Strain	Zfp68	24135	5	137577594	137591201	-
21536	1456157_at	Strain	None	None	NONE	NONE	NONE	NONE
21537	1422877_at	Strain	Pcdhb12	93883	18	37659525	37661894	+
21538	1455658_at	Strain	Cggbp1	106143	16	63876856	63884262	+
21539	1442409_at	Strain	D9Wsu90e	27962	9	78113701	78114479	+
21540	1449039_a_at	Strain	Hnrpdl	50926	5	99053360	99057516	-
21541	1428177_at	Strain	Trappc6b /// LOC433458	433458	2	103523252	103528952	-
21542	1456374_x_at	Strain	Eif3s8	56347	7	120596642	120616097	-
21543	1447171_at	Strain	None	None	18	65146079	65146574	+
21544	1427933_at	Strain	Vps33b	233405	7	74069720	74091665	+
21545	1441057_at	Strain	Myh10	77579	11	68417625	68542200	+
21546	1445362_at	Strain	AW456874	218232	13	48175930	48223631	-
21547	1450777_at	Strain	Xrn2	24128	2	146470058	146532016	+
21548	1440761_at	Strain	None	None	13	87844762	87845158	-
21549	1431935_at	Strain	Lias	79464	5	64193326	64211036	+
21550	1417912_at	Strain	0610009E20Rik	66048	11	72901191	72902695	-
21551	1429781_s_at	Strain	D3Ertd789e	51938	3	33256108	33288047	-
21552	1421873_s_at	Strain	Rab24	19336	13	53928673	53930803	-
21553	1433848_at	Strain	Cdc27	217232	11	104326687	104371504	-
21554	1442404_at	Strain	Ncl	17975	1	86155962	86165711	-
21555	1434956_at	Strain	None	None	7	21409170	21410373	+
21556	1431608_at	Strain	Als2	74018	1	59468051	59542338	-
21557	1453471_at	Strain	4833421E05Rik	67732	12	18821943	18829156	-
21558	1456121_at	Strain	Lrriq2	74201	16	54796937	54831308	-
21559	1454460_at	Strain	5730433N10Rik	70545	1	80170965	80172421	-
21560	1430837_a_at	Strain	Mbd1	17190	18	74502520	74516914	+
21561	1424334_at	Strain	Tspan17	74257	13	53396633	53403940	+

21562	1452385_at	Strain	Usp53	99526	3	121722848	121773789	-
21563	1421872_at	Strain	Rab24	19336	13	53928673	53930803	-
21564	1416746_at	Strain	H2afx	15270	9	44323570	44324932	+
21565	1440205_at	Strain	None	None	2	24891628	24894057	+
21566	1451072_a_at	Strain	Rnf4	19822	5	32824728	32841768	+
21567	1443869_at	Strain	E430028B21Rik	211948	14	24760322	24766051	-
21568	1439722_at	Strain	BC020002	252875	6	8183603	8211482	+
21569	1417932_at	Strain	Il18	16173	9	50637694	50654163	+
21570	1447143_at	Strain	None	None	6	12046742	12047259	+
21571	1455577_at	Strain	Ccl28	56838	13	7664	8154	-
21572	1434419_s_at	Strain	Tardbp	230908	4	147104744	147119358	-
21573	1432001_at	Strain	Zmynd17	74843	14	18844181	18858213	-
21574	1458123_at	Strain	9630002A11Rik	319588	11	93150673	93151337	+
21575	1422681_at	Strain	Sh2bp1	22083	7	104882158	104909760	+
21576	1457949_at	Strain	None	None	19	46435816	46436365	-
21577	1429168_at	Strain	Btbd4	72147	2	181127507	181176383	-
21578	1449053_s_at	Strain	Cog1	16834	11	113470639	113483494	+
21579	1452404_at	Strain	Phactr2	215789	10	13095457	13358121	-
21580	1427461_at	Strain	BC005561	243171	5	103544472	103558503	+
21581	1443495_at	Strain	Atp5j2	57423	5	144222142	144230028	-
21582	1444411_at	Strain	Myo5a	17918	9	75209875	75357058	+
21583	1460435_at	Strain	1500002O20Rik	71997	7	19575337	19598486	+
21584	1430987_s_at	Strain	Wbp11	60321	6	137585452	137599924	-
21585	1415702_a_at	Strain	Ctbp1	13016	5	31736177	31763423	-
21586	1458584_at	Strain	4832406H04Rik	320971	10	29241282	29242029	+
21587	1436864_at	Strain	Arfrp2	218639	13	110208602	110571472	+
21588	1453522_at	Strain	6530401N04Rik	328092	12	48749076	48758074	-
21589	1415974_at	Strain	Map2k2	26396	10	81240906	81258829	+
21590	1439353_x_at	Strain	4930577M16Rik	99887	3	119983678	120044986	-
21591	1447403_a_at	Strain	Zmynd19	67187	2	24881964	24891575	+
21592	1446138_at	Strain	Kifap3	16579	1	163697221	163834642	+
21593	1459450_at	Strain	Chd9	109151	8	90136946	90137565	+
21594	1430971_a_at	Strain	Aqr	11834	2	113615012	113689159	-
21595	1435938_at	Strain	2610318C08Rik	70466	2	128782734	128811017	-
21596	1457241_at	Strain	4930449E07Rik	74665	11	60079022	60119975	+
21597	1416736_at	Strain	Casc3	192160	11	98625992	98654894	+
21598	1424142_at	Strain	Ikbkap	230233	4	56694223	56745708	-
21599	1437817_at	Strain	Snap91	20616	9	87108242	87224260	-
21600	1449659_s_at	Strain	Mphosph9	269702	5	123422304	123499213	-
21601	1422522_at	Strain	Fxr2h	23879	11	69358976	69378357	+
21602	1446183_at	Strain	Als2	74018	1	59468051	59542338	-
21603	1459339_at	Strain	None	None	NONE	NONE	NONE	NONE
21604	1423072_at	Strain	6720475J19Rik	68157	NONE	NONE	NONE	NONE
21605	1450357_a_at	Strain	Ccr6	12458	17	840	2215	+
21606	1460258_at	Strain	Lect1	16840	14	73991740	74016217	-
21607	1419352_at	Strain	0610007P06Rik	67669	7	83909488	83932405	-
21608	1453019_at	Strain	Nvl	67459	1	181047238	181097972	-
21609	1441994_at	Strain	Pcdhb16	93887	18	37701562	37706506	+
21610	1421260_a_at	Strain	Srm	20810	4	147083875	147086981	+
21611	1453038_at	Strain	4930422G04Rik	71643	3	126444767	126499719	+
21612	1423313_at	Strain	Pde7a	18583	3	18563269	18602308	-
21613	1434879_at	Strain	Cdc34	216150	10	79805178	79811022	+
21614	1458856_at	Strain	Pfas	237823	11	68711358	68729532	-
21615	1421047_at	Strain	Smad5	17129	13	55343778	55362726	+
21616	1419605_at	Strain	Mgl1	17312	11	69892432	69896492	+
21617	1441515_at	Strain	None	None	12	3902173	3902775	-
21618	1436415_at	Strain	None	None	7	23790280	23790791	-
21619	1446875_at	Strain	1700025D03Rik	67944	X	131753104	131753700	-
21620	1418354_at	Strain	Cog1	16834	11	113470639	113483494	+
21621	1451485_at	Strain	3300001P08Rik	67684	11	94112226	94142998	-
21622	1460426_at	Strain	9430063L05Rik	229622	3	97225124	97254244	-
21623	1417645_at	Strain	Sspn	16651	6	146867107	146898185	+
21624	1444775_at	Strain	9930033D15Rik	320147	2	31085935	31086588	+
21625	1420830_x_at	Strain	Ywhaq	22630	12	18728420	18754333	+
21626	1424360_at	Strain	BC019943	234138	8	29932839	29943872	+

21627	1454003_at	Strain	Afg3l2	69597	18	67635433	67694777	-
21628	1457417_at	Strain	D2Ertd485e	329506	2	121470306	121527439	+
21629	1451222_at	Strain	4632412E09Rik	70533	4	107773200	107792489	-
21630	1460147_at	Strain	A730009E18Rik	319603	13	105886918	105887540	+
21631	1437377_a_at	Strain	Polrmt	216151	10	79858754	79869197	-
21632	1446807_at	Strain	Usp8	84092	2	126221216	126273150	+
21633	1430158_at	Strain	3110021A11Rik	67289	6	120280712	120281445	+
21634	1415725_at	Strain	Rrn3	106298	16	12509662	12550696	+
21635	1447290_at	Strain	2610042O14Rik	66460	2	163917729	163922245	+
21636	1416365_at	Strain	Hspcb	15516	17	43078708	43084593	-
21637	1430078_a_at	Strain	Ogg1	18294	6	113894844	113902017	+
21638	1421906_at	Strain	Pparbp	19014	11	97973241	98014347	-
21639	1446012_at	Strain	Dtnb	13528	12	2476	11394	+
21640	1431404_at	Strain	4930577M16Rik	99887	3	119983678	120044986	-
21641	1435152_at	Strain	Leng8	232798	7	3357908	3367446	+
21642	1447454_at	Strain	Kif5c	16574	2	49551476	49706940	+
21643	1437013_x_at	Strain	Atp6v0b	114143	4	116843235	116846234	-
21644	1428841_at	Strain	Vmd2	24115	19	9181671	9193711	-
21645	1428942_at	Strain	Mt2	17750	8	93456956	93457734	+
21646	1448564_at	Strain	Cib1	23991	7	74026419	74031875	-
21647	1459625_at	Strain	Frap1	56717	4	146940969	147050045	+
21648	1450076_at	Strain	4933411K20Rik	66756	8	45112900	45139459	+
21649	1422560_at	Strain	Ddi2	68817	4	140564823	140567153	-
21650	1450861_at	Strain	Fancc	14088	13	60694745	60814715	-
21651	1419916_at	Strain	Rnf20	109331	4	49547995	49572760	+
21652	1457287_at	Strain	None	None	6	109171346	109365184	+
21653	1443540_at	Strain	Map3k1	26401	13	108136840	108199233	-
21654	1437401_at	Strain	Igf1	16000	10	87833042	87904980	+
21655	1436115_at	Strain	Gm266	212539	12	106958000	106958491	-
21656	1416794_at	Strain	Arl6ip2	56298	17	77665786	77673524	-
21657	1455433_at	Strain	3110048L19Rik	73233	17	19796748	19797257	-
21658	1438979_s_at	Strain	1700029I15Rik	75641	2	92087601	92088284	+
21659	1441457_at	Strain	None	None	9	58771161	58771846	+
21660	1448009_at	Strain	Ugcgl1	320011	1	36434885	36535646	-
21661	1454477_at	Strain	5530401A10Rik	71431	19	4183614	4184990	-
21662	1434080_at	Strain	Aebp2	11569	6	141421299	141478643	+
21663	1416056_a_at	Strain	Ndufb11	104130	X	18854266	18856494	-
21664	1415858_at	Strain	Eif3s8	56347	7	120596642	120616097	-
21665	1447924_at	Strain	Nucks1	98415	1	131762351	131784790	+
21666	1447944_at	Strain	Zkscan1	74570	5	137058118	137075887	+
21667	1439621_at	Strain	None	None	NONE	NONE	NONE	NONE
21668	1450778_a_at	Strain	Rnuxa	56698	18	56791046	56805535	+
21669	1424687_at	Strain	2700008B19Rik	217026	11	83481700	83510195	+
21670	1448032_at	Strain	Azi2	27215	9	118036598	118059690	+
21671	1455407_at	Strain	Zfp236	329002	18	82763227	82861085	-
21672	1449446_at	Strain	D10Ertd718e	52713	10	105733969	105740000	+
21673	1433479_at	Strain	5730410I19Rik	66622	12	98203846	98224080	+
21674	1448585_at	Strain	Gtf2h4	14885	17	33384058	33390001	-
21675	1444433_at	Strain	Auh	11992	13	51398632	51493178	-
21676	1431413_at	Strain	9130218E19Rik	77677	1	91047054	91048226	+
21677	1418074_at	Strain	St6galnac4	20448	2	32519652	32532644	+
21678	1453302_at	Strain	4930429O20Rik	74626	1	132363502	132365909	+
21679	1434396_a_at	Strain	Myl6 /// LOC432856 /// LOC433297 /// LOC546126	17904	10	128227888	128230852	-
21680	1418702_a_at	Strain	2810428I15Rik	66462	8	69659864	69662106	-
21681	1431669_at	Strain	4930583I09Rik	78057	17	62534920	62538468	-
21682	1457515_at	Strain	Hipk1	15257	3	103171062	103218570	-
21683	1460237_at	Strain	Trim8	93679	19	46050008	46064111	+
21684	1416670_at	Strain	Setdb1	84505	3	94811356	94845004	-
21685	1456209_x_at	Strain	Klf13	50794	7	57665144	57712634	-
21686	1434691_at	Strain	None	None	15	96479068	96480977	-
21687	1445847_at	Strain	None	None	13	57521233	57522123	+
21688	1432719_at	Strain	4833412K13Rik	74607	1	78754094	78755563	-

21689	1443219_at	Strain	None	None	11	46726354	46727019	-
21690	1438034_at	Strain	2410005O16Rik	66302	4	19502173	19533898	+
21691	1444996_at	Strain	Depdc5	277854	5	31352231	31444754	+
21692	1428988_at	Strain	Abcc3	76408	11	94164382	94182408	-
21693	1445612_at	Strain	Tox	252838	4	6614604	6917869	-
21694	1456488_at	Strain	Wdr33	74320	18	32067429	32147139	+
21695	1451797_at	Strain	BC024683	229512	3	88079530	88106201	+
21696	1421109_at	Strain	Cml2	93673	6	86300991	86304727	-
21697	1441170_a_at	Strain	Dab2ip	69601	2	35624156	35663154	+
21698	1438295_at	Strain	Glcci1	170772	6	8234651	8573400	+
21699	1447140_at	Strain	C130078N14	329478	NONE	NONE	NONE	NONE
21700	1440915_at	Strain	Mphosph9	269702	5	123422304	123499213	-
21701	1437916_at	Strain	Klf13	50794	7	57665144	57712634	-
21702	1438070_at	Strain	Phf3	213109	1	31075361	31146157	-
21703	1452479_at	Strain	Copg2as2	54158	NONE	NONE	NONE	NONE
21704	1447204_at	Strain	Flrt2	399558	12	91102888	91191660	+
21705	1417180_at	Strain	Pcsk7	18554	9	45920249	45942975	+
21706	1428178_s_at	Strain	Trappc6b	78232	12	55776176	55794473	-
21707	1451316_a_at	Strain	Picalm	233489	7	84121344	84258493	+
21708	1446925_at	Strain	Btrc	12234	19	44911191	45076729	+
21709	1443104_at	Strain	4933431N12Rik	71310	8	82412909	82519979	+
21710	1425054_a_at	Strain	2510006D16Rik	76799	4	128627820	128635003	+
21711	1457245_at	Strain	None	None	16	34475375	34475780	-
21712	1437078_at	Strain	Vps52	224705	17	31665443	31670899	+
21713	1437891_at	Strain	Frs2	327826	10	116759579	116837926	-
21714	1437441_at	Strain	None	None	NONE	NONE	NONE	NONE
21715	1455887_at	Strain	Alg8	381903	7	91417839	91438435	+
21716	1427350_a_at	Strain	Gtpbp6	107999	5	109154503	109158389	-
21717	1429034_at	Strain	Eme2	193838	17	22698429	22700965	-
21718	1456024_at	Strain	Gtf3c1	233863	7	119690685	119757419	-
21719	1442944_at	Strain	C76555	97125	NONE	NONE	NONE	NONE
21720	1452274_at	Strain	Tex27	21769	17	27815127	28020060	+
21721	1439691_at	Strain	D5Ertd579e	320661	5	35088907	35109670	-
21722	1458172_at	Strain	Me2	107029	18	74004269	74049621	-
21723	1428248_at	Strain	Nfx1	74164	4	41109832	41164029	+
21724	1458605_at	Strain	Arfrp2	218639	13	110208602	110571472	+
21725	1424958_at	Strain	Car8	12319	4	8072625	8166127	-
21726	1422268_a_at	Strain	Rps6kb2	58988	19	3945767	3952035	-
21727	1430798_x_at	Strain	Mrpl15	27395	1	4733601	4746102	-
21728	1430003_at	Strain	6330412A17Rik	74005	18	12118176	12119978	+
21729	1433958_at	Strain	9830165K03Rik	216846	11	69025154	69049433	-
21730	1457351_at	Strain	Taf2	319944	15	55050189	55051519	-
21731	1455420_at	Strain	Rad23b	19359	4	55265936	55307091	+
21732	1458082_at	Strain	Cd200	17470	16	44266133	44292916	-
21733	1418408_at	Strain	Zfand1	66361	3	10341503	10352848	-
21734	1457591_at	Strain	Gm67	217431	12	16711160	16792762	+
21735	1442442_at	Strain	None	None	14	12422037	12422505	-
21736	1420422_at	Strain	Pcdhb21	93892	18	37737542	37740085	+
21737	1454750_a_at	Strain	BC057552	212123	8	83360504	83368261	-
21738	1456256_at	Strain	Eif5	217869	12	107012763	107017765	+
21739	1443354_at	Strain	Trim59	66949	3	68705814	68715182	-
21740	1446538_at	Strain	Kpna3	16648	14	55898702	55974523	-
21741	1420732_at	Strain	2510027J23Rik	66568	3	119952653	119953364	-
21742	1453520_at	Strain	Ttc21b	73668	2	66040107	66112017	-
21743	1430712_at	Strain	Arhgap24	231532	5	101508673	101932043	+
21744	1439358_a_at	Strain	Nrxn1	18189	17	87900413	88568779	-
21745	1436765_at	Strain	Pard3	93742	8	126354044	126919239	+
21746	1441370_at	Strain	3632431M01Rik	330401	6	116456153	116631023	-
21747	1443188_at	Strain	6130401J04Rik	66799	1	16725948	16804454	-
21748	1456091_at	Strain	Sec22l3	215474	9	121695485	121719087	-
21749	1458527_at	Strain	None	None	NONE	NONE	NONE	NONE
21750	1447964_at	Strain	Ttl	69737	2	128579837	128607602	+
21751	1442517_a_at	Strain	9630013A20Rik	319903	14	78810131	78835054	-
21752	1446010_at	Strain	C85067	97889	13	101785583	101786305	-
21753	1444076_at	Strain	Zfp81	224694	17	31036209	31061836	-

21754	1428870_at	Strain	Nolc1	70769	19	45623180	45630496	+
21755	1445858_at	Strain	4933439G19Rik	414080		71855359	71855772	-
21756	1435914_at	Strain	Ncor1	20185	11	62043183	62164152	-
21757	1452442_at	Strain	Usp13	72607	3	32323945	32343734	+
21758	1447426_at	Strain	Dip3b	216190	10	83487957	83536588	-
21759	1433813_at	Strain	Tmem48	72787	4	106326943	106373244	+
21760	1416512_at	Strain	Nubp2	26426	17	22688533	22692246	-
21761	1446057_at	Strain	Rlf	109263	4	120167817	120237523	-
21762	1444757_at	Strain	Eef1e1	66143	13	38186438	38199754	-
21763	1455776_x_at	Strain	1110025L05Rik	66162	7	120745731	120746424	+
21764	1434355_at	Strain	Zfp617	170938	8	71079283	71092128	+
21765	1432262_at	Strain	4930504E06Rik	75007	3	94770775	94783992	+
21766	1438432_at	Strain	2010309L07Rik	66485	17	15387098	15388425	-
21767	1417315_at	Strain	Gripap1	54645	X	6028935	6059503	+
21768	1450711_at	Strain	Brd4	57261	17	30005231	30094101	-
21769	1439875_at	Strain	Zfp128	243833	7	10874888	10887103	+
21770	1451079_at	Strain	Adpgk	72141	9	59408367	59432955	+
21771	1435422_at	Strain	4933433P14Rik	66787	12	101145518	101150041	+
21772	1430834_at	Strain	C030038J10Rik	243385	6	59598164	59670385	-
21773	1433091_at	Strain	6430514K02Rik	76885	8	48702749	48704166	-
21774	1418663_at	Strain	Mpdz	17475	4	80278786	80441865	-
21775	1459467_at	Strain	None	None	14	22602017	22602617	-
21776	1420165_s_at	Strain	Dnajc17	69408	2	118686349	118722623	-
21777	1424457_at	Strain	Apbb3	225372	18	36894927	36903131	-
21778	1434844_at	Strain	MGC78289	238023	11	121028780	121043740	+
21779	1422469_at	Strain	Tbk1	56480	10	121202714	121243001	-
21780	1458401_at	Strain	4932438A13Rik	229227	3	36326717	36516613	+
21781	1431405_a_at	Strain	4930413O22Rik	67986	1	10144887	10173449	+
21782	1458148_at	Strain	D230007K08Rik	268857	16	3617932	3648198	-
21783	1451328_at	Strain	Pcnxl3	104401	19	5453430	5476939	-
21784	1427319_at	Strain	A230046K03Rik	319277	10	83431696	83484395	+
21785	1417671_at	Strain	Scly	50880	1	91121380	91144116	+
21786	1435144_at	Strain	None	None	15	6453936	6454430	+
21787	1453589_a_at	Strain	2610005L07Rik	381598	15	1398	8167	+
21788	1423920_at	Strain	Brrn1	215387	2	126617682	126647758	-
21789	1424816_at	Strain	Cecr5	214932	6	120941468	120963273	-
21790	1425404_a_at	Strain	2310014H19Rik	69179	14	28957085	29008988	+
21791	1443286_at	Strain	AU019754	105964	15	100312989	100313641	+
21792	1418306_at	Strain	Crybb1	12960	5	111325810	111339571	+
21793	1425599_a_at	Strain	Gatad1	67210	5	3645974	3653922	-
21794	1446102_at	Strain	D9Ertd292e	52208	9	107909481	107910183	+
21795	1426805_at	Strain	Smarca4	20586	9	21522528	21593154	+
21796	1420540_a_at	Strain	Rit1	19769	3	88460728	88473591	+
21797	1454701_at	Strain	4930503L19Rik	269033	18	70685809	70705149	-
21798	1432059_x_at	Strain	5031425E22Rik	75977	5	21848106	21897224	-
21799	1427322_at	Strain	Brwd1	93871	16	95429851	95519830	-
21800	1424611_x_at	Strain	Trub2	227682	2	29708273	29720664	-
21801	1458181_at	Strain	9030624J02Rik	71517	7	112616598	112717342	+
21802	1425997_a_at	Strain	Pign	27392	1	105412403	105554926	-
21803	1421557_x_at	Strain	Txn2	56551	15	77967008	77980951	-
21804	1444119_at	Strain	B930006L02Rik	319604	7	94813378	94948322	+
21805	1426849_at	Strain	Sec24b	99683	3	128916056	128993778	-
21806	1423788_at	Strain	Nup133	234865	8	123186372	123236337	-
21807	1421817_at	Strain	Gsr	14782	8	32458839	32503763	+
21808	1440483_at	Strain	Plekhm2	69582	4	140506995	140546160	-
21809	1446521_at	Strain	Psmd14	59029	2	61567169	61655776	+
21810	1416423_x_at	Strain	Ssb	20823	2	69717028	69727204	+
21811	1433651_at	Strain	Wtip	101543	7	29263338	29287059	-
21812	1418769_at	Strain	Myoz2	59006	3	121795553	121824585	-
21813	1443110_at	Strain	Gtf2e1	74197	16	36329263	36359236	-
21814	1421476_a_at	Strain	Cant1	76025	11	118231701	118240124	-
21815	1458525_at	Strain	App	11820	16	84062547	84281536	-
21816	1460582_x_at	Strain	a	50518	2	154470514	154507920	+
21817	1457796_at	Strain	Ubr1	22222	2	120375495	120484564	-
21818	1447615_at	Strain	Fmn	14260	2	112951020	113333093	+

21819	1436640_x_at	Strain	Agpat4	68262	17	10758734	10859090	+
21820	1453146_at	Strain	Gapvd1	66691	2	34609129	34687394	-
21821	1455211_a_at	Strain	Timm9	30055	10	81031392	81032707	-
21822	1453296_at	Strain	2610002I17Rik	72341	9	110345158	110362186	+
21823	1445649_x_at	Strain	Zfp142	77264	1	74868909	74890473	-
21824	1435768_at	Strain	Arid4b	94246	13	13506342	13643882	+
21825	1436923_at	Strain	Rab2b	76338	14	47361876	47379434	-
21826	1449903_at	Strain	Crtam	54698	9	40925014	40956938	-
21827	1441136_at	Strain	None	None		46546772	46547481	+
21828	1452167_at	Strain	2810407C02Rik	69227	3	58220979	58236191	+
21829	1453775_at	Strain	4921505C17Rik	78757	15	6496946	6588954	+
21830	1428414_at	Strain	5730405I09Rik	67974	18	9356614	9490220	-
21831	1417268_at	Strain	Cd14	12475	18	36948832	36950419	-
21832	1419658_at	Strain	None	None	17	33748708	33751704	-
21833	1458218_s_at	Strain	Pde7a	18583	3	18563269	18602308	-
21834	1416322_at	Strain	Prelp	116847	1	133761069	133772168	-
21835	1422524_at	Strain	Abcb6	74104	1	75459418	75468059	-
21836	1438313_at	Strain	None	None	7	86879042	86879312	+
21837	1419909_at	Strain	Mphosph9	269702	5	123422304	123499213	-
21838	1460041_at	Strain	D630040I23Rik	107398	19	6805842	6807622	-
21839	1423992_at	Strain	Gatad2a	234366	8	69059749	69147743	-
21840	1443517_at	Strain	6030443O07Rik	226151	19	44475557	44527665	+
21841	1428192_at	Strain	Gm295	211255	14	73782842	73785087	+
21842	1438028_at	Strain	4930535B03Rik	75137	3	95249480	95306695	-
21843	1441226_at	Strain	Spon1	233744	7	107618725	107894210	+
21844	1432428_at	Strain	C030011J08Rik	77318	13	108676996	108772712	+
21845	1439988_at	Strain	C81203	96909	1	4740358	4741427	-
21846	1444327_at	Strain	Edd1	70790	15	37972806	38204070	-
21847	1440604_at	Strain	Eif4g3	230861	4	136874731	137087744	+
21848	1456732_at	Strain	Gpd1	14555	15	99774809	99782224	+
21849	1418516_at	Strain	Mtf2	17765	5	107157468	107177436	+
21850	1424172_at	Strain	Hagh	14651	17	22655960	22670054	+
21851	1419092_a_at	Strain	Slk	20874	19	47131319	47193755	+
21852	1422676_at	Strain	Smarce1	57376	11	99030134	99052104	-
21853	1455059_at	Strain	9430093I07Rik	109278	11	107513385	107514913	+
21854	1435347_at	Strain	Stau1	20853	2	166405006	166453032	-
21855	1427069_at	Strain	Fbxo28	67948	1	182273786	182300573	-
21856	1445316_at	Strain	4930438O05Rik	78795	1	85961103	86084595	+
21857	1423199_at	Strain	Brd3	67382	2	27379952	27419365	-
21858	1454303_at	Strain	Epn2	13855	11	61242891	61305293	-
21859	1423970_at	Strain	Thoc3	73666	13	53069551	53078853	-
21860	1430819_at	Strain	Asahl	67111	5	91594004	91613926	-
21861	1445773_at	Strain	Meis1	17268	11	18775221	18913473	-
21862	1416202_at	Strain	Bcap37	12034	6	125367716	125372298	+
21863	1425040_at	Strain	Cybrd1	73649	2	70805450	70839291	+
21864	1438207_at	Strain	Gbf1	107338	19	45699725	45833852	+
21865	1438692_at	Strain	Gtf3c4	269252	2	28757737	28772448	-
21866	1458515_at	Strain	Zfp128	243833	7	10874888	10887103	+
21867	1437263_at	Strain	A730089K16Rik	320411	5	75901168	75903524	-
21868	1423808_at	Strain	BC021790	216853	11	69287412	69304982	-
21869	1420810_at	Strain	1500003O03Rik	56398	2	119061563	119100869	+
21870	1439587_at	Strain	---	380664	10	120506866	120563150	-
21871	1426843_at	Strain	BC023754	329015	19	6045057	6051092	+
21872	1430298_at	Strain	A330015D16Rik	320226	4	82511740	82513118	+
21873	1447063_at	Strain	1700017B05Rik	74211	9	57364789	57376245	-
21874	1438740_at	Strain	Nmt2	18108	2	3201559	3243640	+
21875	1443919_at	Strain	B230206N24Rik	210673	6	114064130	114069723	-
21876	1451642_at	Strain	Kif1b	16561	4	147668683	147799980	-
21877	1416851_at	Strain	St13	70356	15	81415844	81450494	-
21878	1444066_at	Strain	Gapvd1	66691	2	34609129	34687394	-
21879	1444766_at	Strain	None	None	12	29978367	29979055	+
21880	1456848_at	Strain	None	None	7	30594636	30595183	+
21881	1440620_at	Strain	Rab8a	17274	8	71319426	71338872	+
21882	1456913_at	Strain	Tmod3	50875	9	75635361	75697149	-
21883	1443357_at	Strain	None	None	15	11940774	11941273	+

21884	1457564_at	Strain	Dffa	13347	4	147596613	147610300	+
21885	1442310_at	Strain	Pip5k2a	18718	2	18884270	19040126	-
21886	1445428_at	Strain	F830020C16Rik	219150	14	59350496	59478125	-
21887	1446694_at	Strain	Dnm3	103967	1	161896669	162387092	-
21888	1453128_at	Strain	Xlkd1	114332	7	104703544	104715890	-
21889	1439326_at	Strain	2010309L07Rik	66485	17	15433659	15434345	-
21890	1441151_at	Strain	AU044157	100334	4	145686824	145836683	+
21891	1458954_at	Strain	None	None	4	42018007	42018646	+
21892	1457753_at	Strain	Tlr13	279572	X	100744640	100761858	+
21893	1451111_at	Strain	Nup133	234865	8	123186372	123236337	-
21894	1425843_at	Strain	Mrpl33	66845	5	30072910	30081579	+
21895	1434330_at	Strain	Lrrc35	272589	9	42371164	42431073	-
21896	1445563_at	Strain	None	None	NONE	NONE	NONE	NONE
21897	1444500_at	Strain	Ahsa1	217737	12	84146427	84153650	+
21898	1433797_at	Strain	E130309D02Rik	231868	5	142360090	142374242	-
21899	1427904_s_at	Strain	2410091C18Rik	73694	17	76759371	76769734	+
21900	1427518_at	Strain	D10627	234358	NONE	NONE	NONE	NONE
21901	1435698_at	Strain	4921505C17Rik	78757	15	6496946	6588954	+
21902	1445779_at	Strain	Grid1	14803	14	32951534	33710256	+
21903	1420077_at	Strain	AI452102	330594	7	84020274	84040181	+
21904	1435379_at	Strain	None	None	8	123334253	123335579	+
21905	1447557_at	Strain	Rabif	98710	1	134344923	134358148	+
21906	1433126_at	Strain	5330422M15Rik	77062	4	149471005	149472085	-
21907	1439073_at	Strain	Zfp160	224585	17	18880791	18900678	+
21908	1451161_a_at	Strain	Emr1	13733	17	55046956	55165548	+
21909	1439217_at	Strain	Nalp9c	330490	7	21681793	21753568	-
21910	1428684_at	Strain	1500001M20Rik	68971	6	115439712	115473268	-
21911	1436048_at	Strain	Exoc8	102058	8	124180431	124185028	-
21912	1442931_at	Strain	2600003E23Rik	70292	5	34380831	34487415	+
21913	1439337_at	Strain	Tom1l1	71943	11	90464544	90465194	-
21914	1445848_at	Strain	9630009C16	330433	6	133897437	133897989	+
21915	1434597_at	Strain	Larp5	217980	13	9077472	9154695	+
21916	1442343_at	Strain	None	None	16	10549414	10549723	-
21917	1439037_at	Strain	Ddx17	67040	15	79580294	79599072	-
21918	1439948_at	Strain	BC046401	394191	2	165315250	165315749	-
21919	1433595_at	Strain	None	None	4	102129558	102130187	-
21920	1455640_a_at	Strain	Txn2	56551	15	77967008	77980951	-
21921	1442354_at	Strain	B230325K18Rik	319527	7	122032889	122035511	+
21922	1458497_at	Strain	Crim1	50766	17	76086689	76087042	+
21923	1424102_at	Strain	Atg4bl	66615	1	93597866	93619652	+
21924	1443018_at	Strain	Ncam1	17967	9	49555319	49607941	-
21925	1430792_at	Strain	3230402G14Rik	70770	NONE	NONE	NONE	NONE
21926	1425031_at	Strain	Fcmd	246179	4	53635886	53679150	+
21927	1439545_at	Strain	Nrf1	18181	6	30095085	30200555	+
21928	1434529_x_at	Strain	Chfr	231600	5	109186644	109222753	+
21929	1436330_x_at	Strain	6.72E+21	210853	17	20033048	20055559	-
21930	1417964_at	Strain	Ap3d1	11776	10	80838692	80873927	-
21931	1429614_at	Strain	Prpf18	67229	2	4539438	4569357	-
21932	1442017_at	Strain	Nfs1	18041	2	155580586	155601122	-
21933	1443077_at	Strain	9430041J06Rik	319779	11	104161593	104162284	-
21934	1451870_a_at	Strain	Brd4	57261	17	30005231	30094101	-
21935	1450781_at	Strain	Hmga2	15364	10	119939358	120054667	-
21936	1426557_at	Strain	Mesp1	17292	7	73590348	73591713	-
21937	1443867_at	Strain	AI447928	106585	17	63695390	63696403	-
21938	1441042_at	Strain	Fgf1	14164	18	39062902	39142421	-
21939	1431047_at	Strain	5430439G14Rik	71389	2	160440153	160566045	-
21940	1459292_at	Strain	Map2k6	26399	11	110220211	110334726	+
21941	1453426_a_at	Strain	Wdfy1	69368	1	80056762	80093054	-
21942	1460333_at	Strain	Ddx59	67997	1	136261992	136289085	+
21943	1446327_at	Strain	None	None	1	166224527	166225201	+
21944	1445601_at	Strain	None	None	NONE	NONE	NONE	NONE
21945	1429707_at	Strain	Plaa	18786	4	93544242	93578250	-
21946	1439601_at	Strain	2700008B19Rik	217026	11	83481700	83510195	+
21947	1451977_at	Strain	Dyrk1a	13548	16	94067810	94100573	+
21948	1438168_x_at	Strain	Ddx39	68278	8	82975115	82983264	+

21949	1435007_s_at	Strain	AI132487	104910	12	104322122	104327808	+
21950	1433839_at	Strain	AI875199	244141	7	90992627	91105853	+
21951	1448961_at	Strain	Plscr2	18828	9	92167000	92189011	+
21952	1433413_at	Strain	Nrxn1	18189	17	87900413	88568779	-
21953	1436770_x_at	Strain	Psma1	26440	7	108067002	108132938	-
21954	1458425_at	Strain	None	None	NONE	NONE	NONE	NONE
21955	1439738_at	Strain	5630401D24Rik	71449	1	162443384	162458080	-
21956	1422636_at	Strain	Dmtf1	23857	5	9125544	9168200	-
21957	1444366_at	Strain	A130052D22	330177	5	116273764	116274460	+
21958	1459885_s_at	Strain	Cox7c	12867	13	82151364	82153299	-
21959	1416944_a_at	Strain	Tlk2	24086	11	104998272	105103265	+
21960	1424522_at	Strain	B130016L12Rik	217995	13	12434470	12477878	+
21961	1417037_at	Strain	Orc6l	56452	8	84572565	84580844	+
21962	1446243_at	Strain	None	None	16	97394579	97395262	+
21963	1433684_at	Strain	---	208092	11	119726141	119740170	+
21964	1424446_at	Strain	Armc7	276905	11	115296770	115311559	+
21965	1420230_at	Strain	AA414993	103569	11	59561235	59561500	+
21966	1450321_at	Strain	Zfp354c	30944	11	50564207	50580572	-
21967	1448586_at	Strain	Hspa14	50497	2	3406125	3430020	-
21968	1453435_a_at	Strain	Fmo2	55990	1	162784360	162808025	-
21969	1447465_at	Strain	None	None	7	92750417	92750809	+
21970	1421813_a_at	Strain	Psap	19156	10	60236420	60261282	+
21971	1451468_s_at	Strain	Xpo5	72322	17	43712776	43752220	+
21972	1450892_a_at	Strain	Usp4	22258	9	108417796	108462269	+
21973	1457185_at	Strain	Zfp114	232966	NONE	NONE	NONE	NONE
21974	1435451_at	Strain	MGI:2176740	191578	5	99782804	99819230	-
21975	1451423_at	Strain	None	None	17	44403085	44404872	+
21976	1420755_a_at	Strain	Park2	50873	17	9877473	10702789	+
21977	1450628_at	Strain	Slc2a8	56017	2	32905150	32914234	-
21978	1444650_at	Strain	Arhgef18	102098	8	4103	13701	-
21979	1424484_at	Strain	Mobk1b	232157	6	83670277	83687043	+
21980	1433461_at	Strain	Sf3b2	319322	19	5062722	5084238	-
21981	1428018_a_at	Strain	Cd300d	140497	11	114817862	114822973	-
21982	1443958_at	Strain	9030203C11Rik	320854	10	14558036	14558896	-
21983	1456441_at	Strain	4931419P11Rik	399599	19	4628205	4630953	+
21984	1436288_at	Strain	1700049M11Rik	68221	2	163655841	163657086	-
21985	1454063_at	Strain	4933412E24Rik	71088	15	60024704	60026451	-
21986	1451009_at	Strain	Rnf151	67504	17	22520924	22523184	-
21987	1446512_at	Strain	2610312B22Rik	69082	2	83342327	83362366	+
21988	1446100_at	Strain	Trp53bp1	27223	2	120712100	120784969	-
21989	1445708_x_at	Strain	3110021A11Rik	67289	6	120281251	120281436	+
21990	1446209_at	Strain	Usp40	227334	1	87765405	87827652	-
21991	1454304_at	Strain	Epn2	13855	11	61242891	61305293	-
21992	1457789_at	Strain	Cln3	12752	7	120621131	120632951	-
21993	1425522_at	Strain	2600011C06Rik	67039	12	80506670	80547102	+
21994	1442098_at	Strain	AU022434	106337	16	8047871	8048623	-
21995	1432218_a_at	Strain	4632412I24Rik	74035	4	150531696	150553856	+
21996	1442840_at	Strain	D4Ertd669e	52379	4	118419653	118420673	-
21997	1437887_at	Strain	E130306M17Rik	320825	10	9499237	9548513	-
21998	1438332_at	Strain	Slc22a6	18399	19	7814586	7823070	+
21999	1459760_at	Strain	Ndufs4	17993	13	110702711	110802452	-
22000	1449675_at	Strain	Ccnb1-rs1 /// LOC231869 /// Ccnb1 /// LOC434175 /// LOC545021 /// LOC545676	12429	5	142497143	142497719	+
22001	1452115_a_at	Strain	Plk4	20873	3	40219747	40236580	+
22002	1442596_at	Strain	Klhdc2	69554	12	66128557	66142253	+
22003	1428533_at	Strain	D1Bwg0212e	52846	1	39832719	39843798	+
22004	1444229_at	Strain	Nr2f2	11819	7	64237865	64245019	-
22005	1422050_at	Strain	Nkx1-2	20231	7	126962111	126965509	-
22006	1422243_at	Strain	Fgf7	14178	2	125548637	125604378	+
22007	1454338_at	Strain	E130112L15Rik	77858	1	162622333	162623548	-
22008	1440609_at	Strain	Map4k4	26921	1	40195342	40319092	+

22009	1423943_at	Strain	Dus1l	68730	11	120610295	120617488	-
22010	1437255_at	Strain	Zbtb7	16969	10	81272657	81283255	+
22011	1441529_at	Strain	B3gnt1	53625	11	22729537	22754478	-
22012	1455541_a_at	Strain	4430402I18Rik	381218	19	28175661	28216751	-
22013	1444419_at	Strain	Prcp	72461	7	86915848	86974762	+
22014	1442540_at	Strain	C77609	96961	2	96165620	96166148	+
22015	1442996_x_at	Strain	None	None	NONE	NONE	NONE	NONE
22016	1419177_at	Strain	D8Ertd531e	52348	8	39455470	39493394	+
22017	1459612_at	Strain	Plekhe1	98432	1	106112969	106335347	+
22018	1437748_at	Strain	Fut11	73068	14	19056280	19061509	+
22019	1427727_x_at	Strain	Psg19	26439	7	15659846	15668568	-
22020	1425918_at	Strain	Egln3	112407	12	50934400	50959266	-
22021	1435071_at	Strain	Zfyve1	217695	12	80410951	80458920	-
22022	1424304_at	Strain	Tpcn2	233979	7	139669045	139699049	-
22023	1448714_at	Strain	Rngtt	24018	4	33589223	33781403	+
22024	1421308_at	Strain	Car13	71934	3	14638758	14659380	+
22025	1449847_a_at	Strain	Col4a3bp	68018	13	92734071	92828876	+
22026	1460162_at	Strain	D8Ertd124e	52176	8	60137958	60138855	+
22027	1438330_at	Strain	LOC436230	436230	X	130339413	130348302	+
22028	1451682_at	Strain	Zfp142	77264	1	74868909	74890473	-
22029	1448876_at	Strain	Evc	59056	5	35805685	35843373	-
22030	1442205_at	Strain	None	None	13	99957448	99957928	+
22031	1451725_a_at	Strain	Psmd4	19185	3	94520536	94530376	-
22032	1444387_at	Strain	Nmt2	18108	2	3201559	3243640	+
22033	1457206_at	Strain	Zfp521	225207	18	13876625	14161173	-
22034	1433151_at	Strain	4933415B22Rik	71178	5	111437201	111438462	+
22035	1441402_at	Strain	Csrp2bp	228714	2	143825971	143864569	+
22036	1429890_at	Strain	A930007D18Rik	77789	4	82457935	82459213	+
22037	1430175_at	Strain	4930588G05Rik	78817	10	105416530	105419021	-
22038	1439193_at	Strain	Prrxl1	107751	14	30736662	30786328	+
22039	1437994_x_at	Strain	2700087H15Rik	70427	10	79660963	79675809	-
22040	1427843_at	Strain	AI840826	407243	2	167099958	167170277	-
22041	1446165_at	Strain	4930420O11Rik	328329	13	98930827	99546711	-
22042	1447580_at	Strain	None	None	11	97668168	97668311	-
22043	1439344_at	Strain	Stag1	20842	9	100545170	100860091	+
22044	1421578_at	Strain	Ccl4	20303	11	83390576	83392669	+
22045	1458339_at	Strain	Cdadc1	71891	14	54094451	54133002	-
22046	1458103_at	Strain	Ncor1	20185	11	62043183	62164152	-
22047	1427493_at	Strain	2610207F23Rik	67161	3	41056512	41247364	-
22048	1431768_a_at	Strain	Hrmt1l3	71974	7	43892468	43972375	+
